# The health system costs of post abortion care in Tanzania

**DOI:** 10.1186/s12913-021-06688-7

**Published:** 2021-07-22

**Authors:** Naomi Lince-Deroche, George Ruhago, Philicia W. Castillo, Patrice Williams, Projestine Muganyizi, Akinrinola Bankole

**Affiliations:** 1 Guttmacher Institute, New York, NY USA; 2 Muhimbili University of Health and Allied Sciences (MUHAS), Dar es Salaam, Tanzania

**Keywords:** Post abortion care, Cost, Tanzania, Health system

## Abstract

**Background:**

Unsafe abortion is common in Tanzania. Currently, postabortion care (PAC) is legally provided, but there is little information on the national cost. We estimated the health system costs of offering PAC in Tanzania in 2018, at existing levels of care and when hypothetically expanded to meet all need.

**Methods:**

We employed a bottom-up costing methodology. Between October 2018 and February 2019, face-to-face interviews were conducted with facility administrators and PAC providers in a sample of 40 health facilities located across seven mainland regions and Zanzibar. We collected data on the direct and indirect cost of care, fees charged to patients, and costs incurred by patients for PAC supplies. Sensitivity analysis was used to explore the impact of uncertainty in the analysis.

**Results:**

Overall, 3850 women received PAC at the study facilities in 2018. At the national level, 77,814 women received PAC, and the cost per patient was $58. The national health system cost for PAC provision at current levels totaled nearly $4.5 million. Meeting all need for PAC would increase costs to over $11 million. Public facilities bore the majority of PAC costs, and facilities recovered just 1% of costs through charges to patients. On average PAC patients incurred $7 in costs ($6.17 for fees plus $1.35 in supplies).

**Conclusions:**

Resources for health care are limited. While working to scale up access to PAC services to meet women’s needs, Tanzanian policymakers should consider increasing access to contraception to prevent unintended pregnancies.

**Supplementary Information:**

The online version contains supplementary material available at 10.1186/s12913-021-06688-7.

## Background

A number of ongoing initiatives globally highlight and support country-level commitments to expanding access to health care and acknowledge the synergistic benefits of working towards improved health, development, and equality [1, 2]. Tanzania, like many countries in Sub-Saharan Africa, has committed to achieving the Sustainable Development Goals (SDGs) and Universal Health Coverage (UHC) [3, 4]. In doing so, the country has committed to working to reduce maternal mortality, one focus of SDG 3 [5], and to taking steps to ensure that access to health care is equitable and affordable for all who need it [6].

In Tanzania, maternal mortality is estimated at 524 deaths per 100,000 live births [7]. Although slightly lower than the average rate in Sub-Saharan Africa (542 per 100,000) [7], significant effort will be required to meet the SDG goal of reducing maternal mortality to under 70 per 100,000 live births by 2030 [5]. Reducing unintended pregnancy is an important intervention for reducing maternal mortality. In 2013, the unintended pregnancy rate in Tanzania was estimated to be 93 per 1000 women of reproductive age [8].

Addressing the level of unsafe abortion occurring in the country will also be important for reducing maternal mortality [9, 10]. A recent estimate suggests that unsafe abortion accounts for 11% of national maternal mortality figures [11]. The country’s Penal Code indicates that abortion is allowed only to save a woman’s life [12, 13]. Yet, abortion is not uncommon. In 2013, 405,000 induced abortions occurred nationwide [8]. The vast majority of these were unsafe. Also in 2013, 66,640 women received postabortion care (PAC), or treatment for complications from induced abortion, nationwide [8]. An additional nearly 100,000 women had complications requiring treatment in a health facility but did not receive this care [14]. The consequences of this missed care include short- and long-term injury and possibly death, [15] as well as household consequences, including loss of productivity, negative consequences for children and deterioration in economic circumstances [16].

The Tanzanian government is committed to improving access to life-saving PAC services, as a means of reducing maternal mortality. Adequate planning for service expansion requires information on the costs of providing such care, which can be expensive. In 2012, it was estimated that total expenditure for PAC in Rwanda was $1.7 million [17]. In Uganda in 2010, PAC cost the health system an estimated $13.9 million [18]. Given that PAC costs are largely avoidable, these are resources that could be spent elsewhere.

Finally, as countries like Tanzania strive to meet their UHC goals, considerations of affordability are paramount. A 2018 study found that less than 10% of women in Tanzania had a form of health insurance [19], and financing for health is reportedly fragmented [20]. Tanzania’s National Health Insurance Fund (NHIF) is one option [21]. Contributions vary: civil servants pay 3% of their monthly wages and the Government provides a matching 3% [19]. According to the NHIF website, other individuals can pay roughly $830 (1,908,000 Shillings) per year for full coverage [22]. Other options for private coverage and care also exist, and their prices vary [19, 23].

In this study, to support national efforts to provide provision of PAC to all women who need it, we estimated the total annual and per client cost to the Tanzanian health system of providing PAC services—currently and for all in need of care. To further local understanding of the affordability of PAC, we also estimated contributions made by women and their families towards the costs of their care. Finally, we contextualize the opportunity cost of spending on PAC and highlight the potential for mitigating PAC costs to the health system by increasing access to contraceptive services.

## Methods

We estimated PAC service provision costs from the health provider perspective using a cross-sectional study design and a pre-tested, bottom-up costing methodology: the Post-Abortion Care Costing Methodology (PACCM), which has been previously used in several settings [17, 18, 24–26].

### Sample selection

We established our sampling frame with input from government sources. We compiled a list of all public and private health facilities (including faith-based and NGO providers) offering PAC services in Tanzania’s mainland (*n* = 1187) and in Zanzibar (*n* = 38) in 2017. To reduce recall bias, we used facilities’ reported manual vacuum aspiration (MVA) caseload as a proxy for PAC service provision and excluded 450 mainland facilities that offered MVA to fewer than five patients between January 1 and October 31, 2017. (MVA caseload information was not available for facilities in Zanzibar.) We also excluded eight maternity homes and nine military health facilities due to expectations about low PAC caseloads and restrictions with obtaining approval for research onsite. Finally, we removed one site known to have closed after the list was generated. After these exclusions, there were 757 mainland and Zanzibar health facilities eligible for selection. These facilities were grouped based on ownership (public/private) and facility level (e.g. national/regional hospital, health center, dispensary, etc.) to establish our proportional sampling targets in these categories.

For sampling, we selected geographical regions and then facilities within selected regions. There are eight geographical zones in Tanzania, including Zanzibar, which are further divided into 31 regions. Due to their political importance locally, we purposively selected Dar es Salaam region and Zanzibar. Dar es Salaam region in the Eastern Zone is the country’s former capital and the most populated area in the country. It also has the largest number of health facilities per region (i.e. three regional hospitals compared to one hospital in other regions). Zanzibar is a semi-autonomous region of Tanzania, which united with the Tanzania mainland to form the United Republic of Tanzania in 1964. We then randomly selected six additional regions. There were 237 eligible health facilities in the selected regions, and we randomly selected 40 facilities using the pre-established ownership/facility-level targets noted above.

After selection, a few facility replacements were necessary. Five sites were replaced due to concerns about the feasibility of accessing the location (*n* = 1) or reports of sites no longer offering PAC when the interviewer arrived (*n* = 4). The interviewing team in the field replaced the facilities by going to a neighboring facility within the same region, district and ownership level. One of these facilities reported an MVA caseload of fewer than five and thus was not in the original sampling universe.

### Costing fieldwork and data management

From October 2018 to February 2019, at each facility, trained interviewers used purposive methods to recruit staff members knowledgeable regarding PAC service provision and the costs of the resources used. The interviewers conducted face-to-face interviews using two electronic questionnaires, referred to as A and B (See supplementary files provided in five parts: A, B-1, B-2, B-3, and B-4). The questionnaires were designed in Survey CTO [27]. They were piloted and updated based on expert advice from individuals familiar with PAC services in Tanzania.

Questionnaire A included questions about patient volumes, staff types employed and average salaries, the costs of constructing and equipping the facility, annual overhead costs, and patient fees charged. This questionnaire also asked for information on the family planning methods offered at the site and detailed information on PAC for management of abortion-related complications. This included the proportion of PAC patients managed as inpatients or outpatients, and within each group, the proportion of patients who were treated for five main complication types: incomplete abortion, shock, sepsis, lacerations, and perforations. Finally, for each of the five complication types, Questionnaire A also captured the proportion of PAC patients seen by each clinical personnel type employed at the facility, and the average amount of time spent per PAC patient during their entire stay at the facility.

Questionnaire B asked about the resource requirements when treating PAC patients in the five complication categories noted above. The questionnaire was split into four components, each of which listed items commonly used for PAC services and prompted listing of other items if used. The four questionnaire components covered use of consumables, small equipment, laboratory tests, and medications. For each component, the respondents were requested to provide, for each of the five complication types separately, whether the item was used at the facility, the proportion of PAC patients who were treated with the item, and the volume used. Finally, if the item was used at the site for any PAC patient, we asked for the unit cost per item. Costs were collected with the year of purchase and the currency. Some sites were unable to report prices, and we used the Tanzania’s Medical Stores Department Price Catalog or in a few cases, prices from UNICEF’s Supply Catalog [28, 29].

After data collection, all data were exported to Stata (v 16) for cleaning and analysis. Cost results are presented in 2018 US dollars ($) (in the text and tables) and Tanzanian Shillings (TzSh) (in the text only). If required, we inflated costs to 2018 values using currency-specific consumer price indices [30], and then converted non-US dollar currencies to US dollars using the average annual exchange rate for 2018 (TzSh 2322: $ 1.00) [31]. Capital and equipment costs were annualized using the government’s discount rate of 8.17% [32]. We used 60 years of useful life for buildings as reported in local accounting guidelines [33] and replacement rates reported by the respondents for equipment.

### Costs at study facilities

We generated descriptive service-level statistics by facility level, ownership category, and region. These include a summary of the components included in the PAC services and the family planning methods offered at the study facilities. These also include the average time spent per clinical staff type with each PAC patient (if seen by the staff type). The average time spent per patient is weighted by the proportion of complications within the outpatient and inpatient groups and the proportion in- versus outpatient. We also present patient volume information that includes all patients (PAC or not), all maternal and neonatal health (MNH) patients, all PAC patients, and the number of each of the five complications treated in the last year. Note that the number of PAC patients and complications treated are not the same at all sites because women could be treated for more than one complication.

We then estimated the direct, indirect, and total costs for PAC provision at the study facilities, as well as fees paid by patients, for management of each of the five main PAC complication types. We defined direct costs as the sum of costs for clinical personnel time and medical supplies (i.e. consumables, small equipment, laboratory tests, and medications). Indirect costs included capital (buildings and large equipment), utilities, and administrative/support staff salaries plus administrative time spent by clinical staff administrative activities. Detail on how these components were calculated is provided below.

#### Direct costs: clinical personnel

To estimate clinical staff costs for PAC provision, we calculated the cost per minute for each staff type employed at each facility using reported annual salaries and monthly working hours. Then we created a set of average costs per minute per staff type at public or private facilities in the sample. Subsequently, for each facility and each PAC inpatient or outpatient complication type, we multiplied the site’s reported proportion of patients seen per staff type by the number of minutes spent when seen to obtain the average time spent per PAC complication type by each staff type. Then we multiplied that by the appropriate (i.e. public or private sector) average cost per minute per staff type to arrive at the average clinical cost per staff type per PAC complication type. These staff costs were then summed across the staff types to obtain the total direct personnel cost per PAC complication type at each facility.

#### Direct costs: medical supplies

For medical supplies (i.e. consumables, small equipment, laboratory tests, and medications), we followed a similar approach as described for direct personnel costs. Each site was asked to report unit costs for all items used for PAC patients. For each facility and each PAC inpatient or outpatient complication type, we multiplied the proportion of PAC patients with whom the item was used by the volume used to obtain the average volume used per PAC complication type. That average volume was then multiplied by the unit cost to produce the average cost per item per PAC complication type, and costs were summed across all items to produce total costs for medical supplies per PAC complication type.

#### Direct costs: total

Total direct costs are the sum of clinical personnel costs and medical supply costs. We present average direct costs per complication type and per patient. The latter are offered by facility type and region and reflect the reported frequency of patients having more than one complication. Total direct costs for all PAC patients seen at each site were calculated by multiplying the direct cost for management of each type of PAC complication by the number of women treated for the complication and then summing across complication types.

#### Indirect costs: capital and overhead

Reported costs for building and equipping each facility were first annualized as noted above. Then we created an average capital cost per facility level (e.g. hospital, health center, etc.) for public and private facilities separately. For each facility, the appropriate average annualized capital cost was divided by the number of patients (PAC or not) seen in the last year to produce an average capital cost per patient. Annual overhead costs were also divided by the number of patients seen in the last year, and this was added to the capital costs to produce an average capital and overhead cost per patient at each site.

#### Indirect: personnel

Indirect personnel costs were handled in two ways. First, we summed the annual wage bill for all administrative and support staff at each facility. These included security guards, cleaners, clerks, receptionists, etc. As with the capital and overhead costs, this annual wage bill was divided by the total number of patients seen at the facility in the last year to produce a cost per patient. Second, we estimated the cost of administrative time spent by clinical staff. This involved multiplying the average cost per minute for each clinical staff type by the number of minutes reportedly spent by these staff in administrative tasks (e.g. department meetings, trainings, inventory, etc.) per year, and summing across clinical staff types. That was divided by the reported number of MNH patients (which included PAC patients) seen by the site per year to produce an average administrative time cost per patient. All per patient indirect personnel costs were then summed.

#### Indirect costs: total

Total indirect costs represent the sum of capital, overhead, and administrative personnel costs. We assumed that the average indirect cost would be the same for any PAC patient seen at a given site. We present these average costs per facility type and region. Note that these costs are constant per patient regardless of whether the patient had more than one complication type. Total indirect costs for all PAC patients seen at the site represent the indirect cost per patient multiplied by the number of PAC patients seen in the last year.

#### Total costs at study facilities

Finally, we summed direct and indirect costs to produce estimates of the total costs for management of complications of induced abortion at all study facilities.

### Patient expenses

Although delivery services should technically be free in public facilities in Tanzania [34], women must pay for some aspects of PAC services. Women who access care in private facilities also contribute to the costs of care—either out-of-pocket or through insurance premiums. To estimate the fees paid by PAC patients to the health facilities offering care, we used the study facilities’ reports of fees charged for PAC. Facilities were asked what proportion of PAC patients would be expected to pay for any portion of their care, and if expected to pay, how much they would pay in total. Not all facilities charged fees, so we present the fees per woman charged and per PAC patient in the sample in addition to the total fees charged across all the study facilities for a one-year period. We also asked the facilities what proportion of PAC patients would be expected to contribute supplies (e.g. food, medications, etc.) while receiving their PAC care, and if expected to contribute, what the total cost to the patient might be. Again, we present the results per women incurring a cost and per PAC patient.

### National costs

Keogh and colleagues estimated the number of women who received PAC for abortion complications nationally by facility type, and the number of women who needed but did not receive PAC in Tanzania in 2013 [8]. We distributed the group of women who needed but did not receive PAC across facility types using the reported distribution for women who did receive care. Then, we inflated the 2013 numbers to obtain a 2018 estimate, using an average annual population growth rate of 3.1% [35], assuming a constant abortion rate and similar patterns of service delivery over time. Finally, we multiplied the average facility-level cost per PAC patient as calculated in the study sample by the number of women estimated to receive PAC across all PAC facilities nationally, and repeated this for the group of women who needed but did not receive PAC. For both groups of women, the direct and indirect costs were calculated separately to allow for discussion of these components within the total cost. We extrapolated patient costs to the national level following the same steps.

### Outliers, missing data, and sensitivity analysis

Estimating the resources and volumes required for bottom-up costing can be challenging for respondents. When necessary, we imputed values for missing responses in our data. Wherever possible, we imputed the mean of non-missing responses within a site’s facility level (for public facilities) or ownership category (for private facilities). However, there were some cases where there were no responses given within a facility level/category. For those cases, we used the next most similar facility level/category, or when required, the mean of all facilities in the sample.

In addition, although some facilities reported a proportion of PAC patients being treated for a particular complication in the last year, when asked about the resources required for treatment, they noted that either no staff members saw the patient type or that no medical supplies were used (inferring that they thought the patient type was not seen at the site). This was the result of different staff being interviewed for the different parts of the costing interviews. For sites that lacked reported personnel costs or medical supply costs but did report seeing a particular patient type in the last year, we imputed the mean personnel or supply cost per patient using information provided by facilities in the same category.

Finally, we conducted univariate and multivariate sensitivity analyses to explore the impact of uncertainty in the analysis inputs on the cost outcomes. Specifically, we varied our estimates of the number of PAC cases nationally in 2018, the average personnel cost per PAC patient, and certain indirect cost components. Each variable was adjusted over a pre-set range independently, and then all variables were adjusted at the same time. The results are presented in terms of the proportional impact on patient-level and national-level costs.

## Results

### Study facilities

We included a total of 40 health facilities (7 hospitals (17.5%) and 33 lower-level facilities (82.5%)) in the final study sample (Table 1). These facilities were located in ten regions spread across all eight geographic zones in the country. They represented 5.3% of the 757 sample-eligible facilities offering PAC nationwide at the time of sampling. Nearly 90% of the selected facilities were publicly owned. Among the five private facilities, four were faith-based, and one was an NGO. Also, three of the five private facilities were dispensaries; one was a health center; and one was a district/cottage hospital (data not shown).

**Table 1:** Facilities selected for costing with level and ownership, by region (*N* = 40)

	Dar es Salaam		Other mainland regions^**a**^		Zanzibar		All facilities	
	n	%	n	%	N	%	n	%
**Facility Level**								
National Hospital	1	8.3	0	0.0	0	0.0	1	2.5
Regional Hospital	1	8.3	1	4.3	0	0.0	2	5.0
District/Cottage Hospital	1	8.3	2	8.7	1	20.0	4	10.0
Health Center/PHCU+ ^b^	3	25.0	9	39.1	4	80.0	16	40.0
Dispensary/PHCU^b^	6	50.0	11	47.8	0	0.0	17	42.5
**Ownership**								
Public	8	66.7	22	95.7	5	100.0	35	87.5
Private	4	33.3	1	4.3	0	0.0	5	12.5
**All facilities**	12	100.0	23	100.0	5	100.0	40	100.0

*PHCU* Primary Health Care Unit, *PHCU+* PHCU offering additional services

^a^Other regions include Kagera, Kigoma, Kilimanjaro, Ruvuma, Singida, and Songwe

^b^PHCU and PHCU+ are designations used in Zanzibar. Health center and dispensary are used on the mainland

### Service delivery

In total, 3850 women received PAC services in the study facilities in 2018. Regional hospitals had the highest annual PAC caseloads, averaging 553 cases per year per facility (Table 2). PAC cases represented 5.0% of maternal and neonatal health patients across all facilities. Most women receiving PAC received care for incomplete abortion, though many women also required treatment for sepsis and shock.

**Table 2:** Annual service volume, including PAC complications seen, by facility level and ownership (2018)

	Facility Level					Ownership		All facilities
Study facilities	National Hospital	Regional Hospital	District/ Cottage Hospital	Health Center/ PHCU+	Dispensary/ PHCU	Public	Private	
	(***n*** = 1)	(***n*** = 2)	(***n*** = 4)	(***n*** = 16)	(***n*** = 17)	(***n*** = 35)	(***n*** = 5)	(***n*** = 40)
Average number of patients seen per year for any condition	459,627	246,388	44,091	20,293	10,239	44,928	11,006	40,688
Average number of PAC patients per year	211	553	304	51	29	105	36	96
Average proportion of MNH patients that are PAC patients	1.1%	2.9%	6.4%	5.7%	4.5%	5.3%	3.2%	5.0%
Average proportion of PAC patients managed as inpatients	100%	45%	16%	14%	na	13%	1%	12%
If admitted, average number of nights in hospital	3.3	4.1	2.0	1.8	na	2.4	1.6	2.3
Average proportion of PAC patients with complication type^a^								
Incomplete abortion	56.0%	91.1%	85.3%	95.7%	90.4%	90.3%	97.3%	91.2%
Sepsis	17.0%	37.9%	11.9%	3.7%	5.1%	8.0%	1.4%	7.2%
Shock	11.0%	17.7%	11.5%	1.3%	4.7%	5.3%	1.4%	4.8%
Lacerations	11.0%	5.9%	1.0%	0.3%	0.0%	0.9%	0.0%	0.8%
Perforations	5.0%	1.8%	0.3%	0.1%	0.0%	0.3%	0.0%	0.3%
Number out of 1000 PAC patients with rare complications^c^	0.00	7.00	0.00	0.60	0.00	0.62	0.00	0.59
Total PAC complications treated in last year^b^								
Incomplete abortion	118.2	1025.6	1080.3	787.6	485.3	3321.9	175.1	3497.0
Sepsis	35.9	446.2	204.0	40.5	10.7	734.8	2.5	737.3
Shock	23.2	211.0	179.4	7.5	5.9	424.6	2.5	427.1
Lacerations	23.2	69.3	21.3	4.4	0.0	118.3	0.0	118.3
Perforations	10.6	20.3	6.3	1.7	0.0	38.9	0.0	38.9
Total number of women treated for any PAC complication	211	1105	1215	821	498	3670	180	3850
Dar es Salaam	211	658	709	206	317	1973	128	2101
Other mainland regions	na	447	326	560	181	1462	52	1514
Zanzibar	na	na	180	55	na	235	na	235
**National level**								
Total number of women treated for any PAC complication annually	6319	15,112	37,550	16,666	2167	50,109	27,705	77,814
Total number of women requiring but not accessing PAC	9280	22,192	55,144	24,474	3182	73,586	40,686	114,272

*na* Not applicable, *MNH* Maternal and newborn health, *PAC* postabortion care, *PHCU* Primary Health Care Unit, *PHCU+* PHCU that offers additional services

^a^All facilities values are weighted based on representation of facility level categories in total sample

^b^Total can exceed 100% because women can have more than one complication type

^c^ Renal failure, peritonitis, heart failure, psychosis, etc.

Asked about the components of the care provided, the respondents noted that, in addition to clinical PAC, the services included community outreach/awareness raising regarding service availability (20%, 8/40), counseling prior to service delivery (95%, 38/40), offering referral letters in case of ongoing complications (75%, 30/40), and post-service family planning (92.5%, 37/40) (data not shown).

A number of different staff types contributed to PAC services at the study facilities. Table 3 provides the average number of minutes spent per clinical staff type, if that staff type saw patients. Nurses and midwives were critical for PAC service delivery. Below the national level, they spent over 45 min, and sometimes over 1 h, per PAC patient seen. Below the regional level, counselors also played an important role in PAC service provision, spending between 20 and 60 min per PAC patient. Time spent per patient at private facilities was similar to, but often just slightly less than, the time spent per patient in public facilities.

**Table 3:** If employed and seeing PAC patients, minutes spent by health care worker per patient^a^

	Facility Level					Ownership		All facilities^**b**^
	National Hospital	Regional Hospital	District/ Cottage Hospital	Health Center/ PHCU+	Dispensary/ PHCU	Public	Private	
	(***n*** = 1)	(***n*** = 2)	(***n*** = 4)	(***n*** = 16)	(***n*** = 17)	(***n*** = 35)	(***n*** = 5)	(***n*** = 40)
Ob/gyne	11.5	12.0	na	na	na	11.8	na	11.8
Anesthetist	8.0	27.6	38.2	39.2	na	33.4	38.2	34.0
Doctor/medical officer	34.8	62.5	45.3	31.0	na	41.0	38.1	40.5
Asst. medical officer	na	58.8	43.8	35.7	50.0	41.0	39.7	40.9
Clinical officer	na	21.5	41.1	31.1	34.8	33.0	35.3	33.4
Asst. clinical officer	na	23.3	na	40.2	44.0	40.7	na	40.7
Nurse/nursing officer	34.8	79.5	63.1	47.9	41.5	50.3	55.5	50.7
Midwife	34.8	79.5	67.7	47.1	45.5	50.0	50.3	50.0
Nursing asst.	na	125.1	56.3	52.1	54.7	56.6	54.5	56.2
Lab technician	15.6	17.4	15.2	17.7	11.7	16.0	13.6	15.6
Sonographer	24.5	37.3	17.0	21.4	na	22.2	19.2	21.6
Pharmacist	na	16.2	5.5	7.0	na	8.7	6.3	8.3
Pharmacy asst.	10.0	29.8	9.8	9.1	5.0	11.7	8.0	10.8
Dispensing pharmacist	na	na	5.0	3.6	9.8	4.1	6.1	5.1
Counselor	na	na	45.3	60.0	20.0	55.1	32.6	46.1

*Ob./gyne* Obstetrician/gynecologist, *Asst.* Assistant, *na* patient is not seen by provider at any site in category

^a^ Time is average from sites where patient was seen by provider type. Average time is weighted by proportion of inpatients and ouptatients and the representation of the five main complication types in each group

^b^ Average values here are weighted by representation of facility levels in sample

### Average costs per PAC patient

Table 4 presents the average direct, indirect and total cost per PAC patient seen. The total average direct cost was $36.64 (TzSh 85,078) per patient seen. Medical supplies comprised the largest component accounting for 80% of the total costs per patient. Medical supply per patient costs were lower in lower-level facilities. Personnel costs were higher at higher-level facilities due to higher-level staff.

**Table 4:** Average direct, indirect and total costs per PAC patient by facility type, region, and complication type (USD 2018)

	**Facility type**										**Ownership**					
	**National Hospital**		**Regional Hospital**		**District/ Cottage Hospital**		**Health Center/ PHCU+**		**Dispensary/ PHCU**		**Public**		**Private**		**All Facilities**	
*Any complication*^*a*^	**$**	**%**	**$**	**%**	**$**	**%**	**$**	**%**	**$**	**%**	**$**	**%**	**$**	**%**	**$**	**%**
**Direct costs**																
Personnel	8.03	16%	13.19	19%	9.44	15%	2.51	6%	1.16	3%	3.62	9%	1.03	3%	3.30	8%
Medical supplies	39.73	79%	53.80	78%	44.75	71%	33.01	79%	28.19	85%	33.64	80%	31.26	79%	33.35	80%
Consumables	17.12		19.53		24.42		16.23		12.03		15.41		15.74		15.45	
Equipment	11.45		19.18		11.56		11.15		12.52		12.71		8.47		12.18	
Laboratory	10.90		13.45		6.40		5.05		2.72		4.65		5.52		4.76	
Medications	0.26		1.64		2.38		0.59		0.93		0.88		1.52		0.96	
Total direct	47.76	95%	66.99	97%	54.19	86%	35.52	85%	29.35	88%	37.27	88%	32.28	82%	36.64	88%
**Indirect costs**																
Capital	0.39	1%	0.09	0%	0.80	1%	1.34	3%	0.44	1%	0.77	2%	1.13	3%	0.82	2%
Overhead	0.39	1%	0.80	1%	2.73	4%	1.53	4%	1.10	3%	1.31	3%	2.08	5%	1.40	3%
Adm. staff	1.91	4%	1.50	2%	5.15	8%	3.15	8%	2.43	7%	2.80	7%	3.83	10%	2.93	7%
Total indirect	2.69	5%	2.39	3%	8.68	14%	6.03	15%	3.97	12%	4.88	12%	7.04	18%	5.15	12%
**Total costs**	**50.45**	**100%**	**69.38**	**100%**	**62.87**	**100%**	**41.54**	**100%**	**33.32**	**100%**	**42.15**	**100%**	**39.32**	**100%**	**41.80**	**100%**
	**Complication type**										**Region**					
	**Incomplete abortion**		**Sepsis**		**Shock**		**Lacerations**		**Perforations**		**Dar es Salaam**		**Other regions**		**Zanzibar**	
*Any facility/ region*	**$**	**%**	**$**	**%**	**$**	**%**	**$**	**%**	**$**	**%**	**$**	**%**	**$**	**%**	**$**	**%**
**Direct costs**																
Personnel	2.52	6%	5.05	13%	5.38	16%	3.29	15%	10.29	32%	4.85	11%	2.45	6%	3.47	7%
Medical supplies	31.88	81%	27.52	73%	23.49	69%	13.45	61%	16.38	51%	33.80	79%	32.62	81%	35.58	77%
Consumables	14.26		14.31		12.79		8.30		9.37		15.28		15.48		15.73	
Equipment	12.50		7.35		4.80		1.96		2.34		11.36		11.70		16.37	
Laboratory	4.25		4.76		4.86		2.69		2.37		6.67		4.23		2.60	
Medications	0.88		1.11		1.04		0.49		2.30		0.49		1.22		0.88	
Total direct	34.40	87%	32.58	86%	28.87	85%	16.74	76%	26.67	84%	38.65	91%	35.08	87%	39.05	84%
**Indirect costs**^**b**^																
Capital	0.82	2%	0.82	2%	0.82	2%	0.82	4%	0.82	3%	0.58	1%	0.74	2%	1.74	4%
Overhead	1.40	4%	1.40	4%	1.40	4%	1.40	6%	1.40	4%	1.09	3%	1.44	4%	1.97	4%
Adm. staff	2.93	7%	2.93	8%	2.93	9%	2.93	13%	2.93	9%	2.24	5%	3.12	8%	3.72	8%
Total indirect	5.15	13%	5.15	14%	5.15	15%	5.15	24%	5.15	16%	3.91	9%	5.31	13%	7.43	16%
**Total costs**	**39.55**	**100%**	**37.73**	**100%**	**34.03**	**100%**	**21.89**	**100%**	**31.83**	**100%**	**42.56**	**100%**	**40.38**	**100%**	**46.47**	**100%**

*Admin.* administrative

^a^ Includes the five major complication types. Within facility type and ownership categories, the costs are weighted by the proportion of inpatients and outpatients as well as the prevalence of complications. Women can have more than one complication type

^b^ Indirect costs are considered constant across all patients in a facility

The total indirect cost per PAC patient seen at the study facilities was $5.15 (TzSh 11,963). Across facility levels, administrative staff wages constituted more than half of the total indirect cost. Costs per patient were lower at facilities with higher PAC caseloads, due to the total annual indirect costs being spread across more PAC patients. Indirect costs were $6.03 (TzSh 13,994) per patient at health centers which saw on average 51 PAC patients per year (Table 2) and just $2.39 (TzSh 5539) per patient at regional hospitals which reported caseloads of 553 PAC patients per year.

The average total health system cost per PAC patient at any site was $41.80 (TzSh 97,041). This includes care for all complications experienced by the patient. Total costs were highest per patient at regional hospitals at $69.38 (TzSh 161,083) per patient, and lowest at dispensaries at $33.32 (TzSh 77,369).

Finally, Table 4 provides the average cost of treating each complication type separately. The average total cost per PAC patient with a laceration was $21.89 (TzSh 50,821), compared with $39.55 (TzSh 91,836) for a patient with an uncomplicated incomplete abortion. These differences were largely driven by variation in the kinds and quantities of medical supplies required. It is also important to remember here that patients could have more than one complication, and the cost per average patient ($41.80 (TzSh 97,041)) reflects reported combinations of complications as well as the variation in costs at the different facility types.

### Total annual costs

Across all 40 health facilities in the study sample, the total cost of providing PAC to the 3850 women who received care in 2018 was $213,639 (TzSh 496,017,321) (Table 5). Clinical personnel and supplies contributed 92% of this total, or $195,512 (TzSh 453,932,264). Medical supplies accounted for the largest share at 75% of the total costs.

**Table 5:** Total annual direct, indirect and overall costs in study facilities, by facility type and region (*N* = 40) (USD 2018)

	**Facility type**										**All facilities**	
	**National Hospital**		**Regional Hospital**		**District/ Cottage Hospital**		**Health Center/ PHCU+**		**Dispensary/ PHCU**			
*All complications*	**$**	**%**	**$**	**%**	**$**	**%**	**$**	**%**	**$**	**%**	**$**	**%**
**Direct costs**												
Personnel	1694	16%	15,459	19%	15,000	20%	1662	5%	982	7%	34,797	16%
Medical supplies	8384	79%	62,475	78%	50,844	68%	28,174	83%	10,838	81%	160,715	75%
Consumables	3613		23,189		27,625		14,857		4606		73,889	
Equipment	2415		21,788		12,970		8094		4497		49,763	
Laboratory	2301		15,684		8900		4788		1155		32,828	
Medications	55		1815		1349		436		580		4235	
Total direct	10,077	95%	77,934	97%	65,844	88%	29,836	87%	11,820	88%	195,512	92%
**Indirect costs**												
Capital	82	1%	89	0%	953	1%	923	3%	156	1%	2204	1%
Overhead	82	1%	847	1%	3185	4%	1270	4%	454	3%	5838	3%
Adm. Staff	403	4%	1513	2%	5095	7%	2103	6%	971	7%	10,085	5%
Total indirect	567	5%	2449	3%	9233	12%	4296	13%	1581	12%	18,127	8%
**Total costs**	**10,644**	**100%**	**80,383**	**100%**	**75,077**	**100%**	**34,132**	**100%**	**13,401**	**100%**	**213,639**	**100%**
	**Ownership**				**Region**							
	**Public**		**Private**		**Dar es Salaam**		**Other regions**		**Zanzibar**			
*All complications*	**$**	**%**	**$**	**%**	**$**	**%**	**$**	**%**	**$**	**%**		
**Direct costs**												
Personnel	34,598	17%	199	3%	23,910	19%	9090	12%	1798	14%		
Medical supplies	154,714	75%	6001	81%	97,163	76%	55,095	75%	8458	68%		
Consumables	70,886		3003		44,511		24,747		4631			
Equipment	48,098		1665		28,800		18,139		2824			
Laboratory	31,853		975		22,148		9992		688			
Medications	3877		358		1704		2216		314			
Total direct	189,311	92%	6201	84%	121,073	95%	64,184	87%	10,255	82%		
**Indirect costs**												
Capital	2060	1%	144	2%	646	1%	1257	2%	302	2%		
Overhead	5495	3%	343	5%	2509	2%	2723	4%	605	5%		
Adm. Staff	9386	5%	699	9%	3572	3%	5237	7%	1275	10%		
Total indirect	16,941	8%	1186	16%	6727	5%	9217	13%	2182	18%		
**Total costs**	**206,252**	**100%**	**7387**	**100%**	**127,800**	**100%**	**73,401**	**100%**	**12,437**	**100%**		

At the national level, we estimated that in 2018, the total cost to the Tanzanian health system of offering PAC services to the 77,814 women who received care was nearly $4.5 million (TzSh 10.4 billion) (Table 6). Over half of this cost was incurred at mid-level facilities: district hospitals and health centers combined incurred costs of over $3 million (TzSh 7.1 billion). Assuming the proportion of public (versus private) health facilities providing PAC nationally in 2013 (74%) [8] has not changed considerably since then, the public health system incurred the vast majority of these costs. Given the distribution of facilities at the national level and their facility-specific costs, we estimate that the average cost per woman seen at any facility nationally was $57.73 (TzSh 134,043).

**Table 6:** Total annual direct, indirect and overall costs for all women receiving PAC nationally and for women needing but not currently receiving care, by facility type (USD 2018)

	Facility type											
	National Hospital		Regional Hospital		District/ Cottage Hospital		Health Center/ PHCU+		Dispensary/ PHCU		All facilities	
**National, provision of PAC for 77,814 women**												
**Direct costs**												
Personnel	50,722	16%	199,383	19%	354,295	15%	41,754	6%	2515	3%	648,669	14%
Medical supplies	251,084	79%	813,016	78%	1,680,432	71%	550,135	79%	61,094	85%	3,355,760	75%
Total direct	301,806	95%	1,012,399	97%	2,034,726	86%	591,888	85%	63,609	88%	4,004,428	89%
**Indirect costs**												
Capital	2461	1%	1296	0%	29,909	1%	22,350	3%	962	1%	56,977	1%
Overhead	2466	1%	12,023	1%	102,627	4%	25,556	4%	2375	3%	145,048	3%
Adm. Staff	12,060	4%	22,736	2%	193,454	8%	52,543	8%	5264	7%	286,056	6%
Total indirect	16,986	5%	36,055	3%	325,990	14%	100,448	15%	8601	12%	488,081	11%
**Total costs**	318,792	100%	1,048,453	100%	2,360,717	100%	692,337	100%	72,210	100%	4,492,509	100%
**National, required additional care for 114,272 women**												
**Direct costs**												
Personnel	74,487	16%	292,800	19%	520,293	15%	61,316	6%	3694	3%	952,590	14%
Medical supplies	368,724	79%	1,193,939	78%	2,467,766	71%	807,890	79%	89,718	85%	4,928,036	75%
Total direct	443,211	95%	1,486,739	97%	2,988,058	86%	869,206	85%	93,412	88%	5,880,626	89%
**Indirect costs**												
Capital	3613	1%	1903	0%	43,923	1%	32,821	3%	1413	1%	83,672	1%
Overhead	3621	1%	17,656	1%	150,711	4%	37,530	4%	3488	3%	213,007	3%
Adm. Staff	17,710	4%	33,388	2%	284,093	8%	77,160	8%	7730	7%	420,082	6%
Total indirect	24,945	5%	52,947	3%	478,727	14%	147,512	15%	12,631	12%	716,761	11%
**Total costs**	468,156	100%	1,539,686	100%	3,466,785	100%	1,016,718	100%	106,043	100%	6,597,388	100%
**National, expenses incurred by patients currently**												
PAC fees	680,452		227,807		88,954		33,199		8620		1,039,031	
PAC supplies	8986		10,744		29,522		13,514		850		63,615	
Fees+supplies	689,437		238,551		118,475		46,713		9469		1,102,646	

Medical supplies: = Consumables, small equipment, medications, and laboratory tests

Finally, if the Tanzanian health system had provided PAC to the estimated 114,272 women who required PAC but did not receive it in 2018, the additional cost of providing care would have been over $6.5 million (TzSh 15.3 billion). The cost of meeting all need for PAC services nationally—including current services and these additional women in need of care—in 2018 would have been over $11 million (TzSh 25.7 billion) (data not shown).

### Patient expenses

According to the study respondents, obtaining PAC services at their facilities sometimes required payments from patients. Nearly half (48%) of the facilities reported charging for PAC services, whether inpatient or outpatient (Table 7). This could have included pre-PAC counseling, consultation with a nurse or doctor, ultrasound, evacuation of the uterus, medication, hospitalization (including food costs), other supplies or another component of care. No facility reported charging for family planning services.

**Table 7:** Facilities charging patients for PAC, fees per patient, and additional PAC supply costs (USD 2018)

	Facility Level					Ownership		
	National Hospital	Regional Hospital	District/ Cottage Hospital	Health Center/ PHCU+	Dispensary/ PHCU	Public	Private	All facilities
	(***n*** = 1)	(***n*** = 2)	(***n*** = 4)	(***n*** = 16)	(***n*** = 17)	(***n*** = 35)	(***n*** = 5)	(***n*** = 40)
**Proportion of facilities that charge any patient for …**								
Inpatient services	100%	100%	50%	56%	na	34%	40%	35%
Outpatient services	na	100%	75%	75%	88%	77%	100%	80%
PAC services	100%	100%	50%	38%	47%	40%	100%	48%
Family planning services	0%	0%	0%	0%	0%	0%	0%	0%
**PAC service fees**								
Proportion of PAC patients expected to pay fees for any part of care^a^	100%	100%	38%	34%	47%	38%	90%	45%
If expected to pay, average total fee per patient	107.68	15.07	4.74	5.31	11.27	13.39	17.23	14.52
Average fee per any PAC patient	107.68	15.07	2.37	1.99	3.98	4.59	17.23	6.16
Total annual fees charged to all PAC patients	22,720	14,840	1059	1949	2505	40,249	2823	43,073
**PAC supplies**								
Proportion of facilities where PAC patients need to bring own supplies^b^	100%	50%	50%	56%	35%	43%	80%	48%
Proportion of PAC patients expected to contribute supplies	78%	39%	40%	45%	22%	30%	76%	35%
If expected to contribute, average estimated amount spent	1.42	1.42	1.57	1.44	1.11	1.19	1.94	1.35
Average estimated spending on PAC supplies by any patient	1.42	0.71	0.79	0.81	0.39	0.51	1.55	0.64
Total annual spending on PAC supplies by all patients	300	636	479	610	140	1858	307	2165
**PAC fees + supplies**								
Average expenses (fees + supplies) incurred per patient	109.10	15.79	3.16	2.80	4.37	5.10	18.78	6.81
Annual total expenses (fees + supplies) incurred by patients	23,020	11,225	538	1694	1503	35,215	2764	37,979

^a^ This could include pre-PAC counseling, consultation with a nurse or doctor, ultrasound, evacuation of the uterus, medication, hospitalization (including food costs), other supplies and any other component of care

^b^ This most often included food, but may have also included sonography results, medications, and other item

Where facilities charged for PAC, the average fee was $14.52 (TzSh 33,706). At the national hospital, the fee was over $100 (TzSh 250,000) per patient seen. However, because not all facilities charged for PAC services, the average fee for any PAC patient seen at the study facilities was $6.17 (TzSh 14,325).

The study respondents were also asked whether they expected PAC patients to bring their own supplies, such as medications, food, etc. According to the respondents, across all facilities, over a third (35%) of PAC patients could be expected to bring some supplies. Food was the most commonly noted item. The respondents thought that women bringing their own supplies might incur costs of approximately $1.35 (TzSh 3132) on average. Combining fees charged by the facilities and out-of-pocket supply costs, we estimated that an average PAC patient obtaining care at the study facilities could be expected to contribute $6.81 (TzSh 15,813).

### Sensitivity analysis

Finally, when examining the impact of parameter uncertainty on total national costs, the most influential variable was the estimated number of women receiving PAC. Using the uncertainty range noted in Table 8, the estimated total national health system cost could be lower by 621.6% or higher by 35.6%. Varying the allowable period for depreciation of buildings and the discount factor had negligible effects on the total national cost. When combined in the multivariate analysis, adjusting all three parameters simultaneously up or down, the results were driven by the impact of the estimated number of women needing PAC.

**Table 8:** Sensitivity analysis: Items varied and impact on per patient and national cost outcomes

Item varied	Estimate in base case	Range varied		Impact on total national cost	
		Low	High	Low	High
Number of PAC patients receiving care nationally^a^	77,814	28,040	105,242	− 64.0%	35.2%
Lifetime of buildings (years)	60	45	75	0.0%	0.0%
Discount rate	8.17%	5%	10%	−0.5%	0.3%
All variables^**b**^	See above	See above	See above	−61.6%	35.6%

*NC* No change to outcome when input was varied

^a^ Currently receiving PAC. The range for current PAC patients is based on the range estimated by Keogh et al. 2015. The range for women requiring but not obtaining PAC is estimated using the size of the range for current PAC patients relative to the base case estimate

^b^ For this option, low = lowest number of patients, highest number of years for depreciation, and the lowest discount rate. High = highest number of patients, lowest number of years for depreciation, and the highest discount rate

## Discussion

Unsafe abortion is not uncommon in Tanzania and results in significant costs to the health system. Our results provide an estimated average cost per woman treated for complications from unsafe abortion as well as the national costs in 2018. We estimate that at an average cost of roughly $58 (TzSh 134,043) per woman provided with PAC nationally, the Tanzanian health system incurs costs of nearly $4.5 million (TzSh 10.4 billion) per year. If all need for PAC were met, the annual cost would rise to over $11 million (TzSh 25.7 billion). Nurses and midwives are critical for PAC provision nationally, but clinical and medical officers also contribute at facilities above the dispensary level.

Average costs per PAC patient varied for a number of reasons. In our study sample, regional and district hospitals had the highest annual caseloads and the highest per patient costs, likely due to use of higher level staff and more serious, or multiple, complications among PAC patients. Interestingly, average costs at private facilities in the study sample were slightly less than at public facilities: roughly $39 (TzSh 91,299) at private facilities and $42 (TzSh 97,861) at public facilities per PAC patient seen. This was due to private facilities seeing less complicated patients. However, due to there being far more public facilities at the national level—74% of all facilities providing PAC [8], the public sector bore the brunt of PAC costs in the country.

The PACCM approach for estimating PAC costs has been used in other African countries, including Senegal, Rwanda and Uganda. The total health system costs of treating PAC patients in Uganda in 2010 was roughly $13.9 million [18]. In Rwanda in 2012, PAC services totaled $1.7 million [17], and in Senegal in 2016, the national cost was nearly $500,000 [24]. The differences in costs between countries are almost entirely driven by differences in the number of women receiving PAC. In Uganda, where the costs were most similar to the total in Tanzania, 105,900 women received PAC in 2010. In contrast, in Senegal in 2016, just under 19,000 women received PAC.

In 2017, Baynes et al. used similar methodology to that used in this study to estimate the costs of introducing and scaling up PAC services at 31 public and private facilities spread across two Tanzanian mainland regions and Zanzibar [36]. They estimated the cost of training a health care provider on PAC provision was $163.43. Once trained, the average cost of offering PAC, including personnel, medicines/supplies and hospitalization was $72.91, including 59% in direct costs. In our study, the national average cost per patient was lower—at roughly $58 per patient. Comparing the cost components, medical supply costs were lower, and personnel costs were higher on average in their study. These differences could be explained by variations in methodology or the sources of price information. We used average salaries and prices as reported by the sites and supplemented them with other sources only in a minority of cases. It is unclear whether Baynes et al. relied on respondent reports or other sources to obtain salaries and purchase prices for medicines/supplies. Despite differences between the findings of the two studies, it is clear that the national costs to the health system of offering PAC services each year in Tanzania are substantial.

Spending on health services in a given country is influenced by political decisions and budgetary constraints, but it also reflects need for care and the health system’s ability to meet that need. In 2017, Tanzania spent 3.6% of its national gross domestic product (GDP) [37] (estimated at roughly $53.3 billion [38])—on health care, translating to an annual health expenditure of roughly $1.9 billion, or $35 per capita. Our national estimates of spending on PAC constitute 0.31% of this total annual spending on health, or $0.11 of the $35 per capita spending envelope. However, just 41% of women in need of PAC are able to obtain it. In contrast, in 2016, Senegal spent 5.5% of GDP, or $53 per capita on health, and 58% of women in need of PAC were able to obtain it [24].

Although the per capita cost of offering PAC currently in Tanzania may seem small, it is largely an avoidable cost, and if reduced, could impact on the country’s ability to pay for other types of needed health care. According to 2019 data from FP2020, just 33.3% of all women ages 15–49 in Tanzania use modern contraception, and more than a quarter (26.3%) of married women have an unmet need [39]. Scaling up provision of modern family planning could reduce the unintended pregnancies that often result in unsafe abortion, and doing so could be a cost-effective strategy. Lince-Deroche et al. estimated that in Tanzania in 2019, the annual health system costs of providing modern contraception per woman varied from just $0.62 for intrauterine contraceptive devices (IUDs) to $6.54 for injectable methods [40]. Further, in 2019 Sully et al. estimated that meeting the contraceptive needs of all women in low- and middle-income countries would cost an average of $1.94 per person per year [41]. They also concluded that for each $1 spent on contraception beyond current levels, countries can reap $3 in savings on pregnancy-related and newborn care.

Finally, as countries such as Tanzania strive to achieve UHC, it is important to consider who pays for health care. In our study, patients were charged roughly $6 (TzSh 15,000) on average for PAC services. At the national level, these fees resulted in recovery of just under 1% of all costs incurred by the health system. We are unable to comment on costs to patients for purchase of health insurance or out-of-pocket costs for other aspects of PAC care. However, Baynes et al. collected patients’ out-of-pocket costs from a sample of 25 PAC patients across 16 health facilities [36]. They were able to explore women’s costs for travel, hospitalization, food, medicines, laboratory tests and other related-items. They estimated that women spent nearly $23 dollars for their care. To set this in context, the minimum monthly wage in Tanzania, which varies by sector, was as low as $50 per month for individuals employed as domestic workers in 2013 [42, 43].

There are a number of limitations to our study, some of which apply to costing studies in general. In all costing studies, tradeoffs must be made between precision and efficiency when collecting the study inputs. Interviewing experts in the provision of care can be faster than reviewing site records. Of course, individuals can fail to recall specific details, but missing information is also a problem when relying on site records. Costing interviews can also be long and taxing, potentially leading to respondent fatigue and less accurate responses. There is a limit to the number of questions that can be included. We did not cover the costs of training providers in PAC service provision, costs associated with stabilizing PAC patients prior to referring to higher-level facilities, or the costs of offering post-PAC family planning services. We were also unable to obtain information on the costs of providing PAC for very rare complications. However, because rare complications occur so infrequently, their contributions to overall PAC costs are extremely limited and any underestimates due to this limitation are likely minimal.

We attempted to explore the impact of uncertainty in our study inputs on our estimates of national PAC costs through sensitivity analysis. The most important driver of national costs was the number of women receiving care. When extrapolating the costs estimated in the study sample to the national level, we relied on abortion and PAC service provision data collected in 2015 and assumed a constant abortion rate and service provision patterns over time. Modern contraceptive use among married women has increased in Tanzania—from 27.4% in 2015 to 33.3% in 2019 [39]. This may have led to an overestimate of need for PAC in our study. However, potential simultaneous increases in PAC availability, through efforts similar to the work described by Baynes et al. [36], may have increased the volume of PAC services provided annually.

## Conclusions

Tanzania’s commitments to UHC and recent progress in terms of increasing access to health insurance and vital health services, such as modern contraception, are commendable. But much work remains if the country is going to meet the SDG of universal access to reproductive health by 2030. In an environment of constrained resources, spending should be prioritized and efficient. While working to scale up access to PAC services to meet women’s needs, local policymakers should consider the longer term costs savings that could come from policy shifts that reduce unsafe abortion. Preventing unintended pregnancies through scale up of contraceptive access is one critical strategy for mitigating PAC costs. Finally, any effort to scale up access to reproductive health services, including PAC, must address affordability. Fees paid to health facilities are just one part of patient costs. Other out-of-pocket costs are important to measure and guard against if the aim is to avoid cost being a barrier to accessing care.

## Supplementary Information

**Additional file 1.**

## Data Availability

All data generated as part of this study are protected by the Guttmacher IRB and the Tanzanian authorities noted above. The data may be shared on request to the corresponding author with permission of those authorities*.*

## Abbreviations

GDP: Gross domestic product
NGO: Non-governmental organization
NHIF: National Health Insurance Fund
MVA: Manual vacuum aspiration
PAC: Postabortion care
SDGs: Sustainable Development Goals
TzSh: Tanzanian Shillings
UHC: Universal Health Coverage
