# Supplementary material for: The health system costs of post abortion care in Tanzania
Source: BMC Health Serv Res. 2021 Jul 22;21:720. doi: 10.1186/s12913-021-06688-7 (PMC8296742; doi:10.1186/s12913-021-06688-7)
Supplement: Supplementary file 1 — Additional file 1. [file 12913_2021_6688_MOESM1_ESM.zip › QA v2.0 2018.01.28 FINALR3.pdf]

Tanzania PAC cost study - Quest. A

Field	Question	Answer
about_survey	**Tanzania Cost Evaluation Questionnaire A**<br><br>INTERVIEWER INSTRUCTIONS<br>• DO NOT READ TEXT IN ALL CAPS OR HINTS IN *ITALICS* ALOUD TO THE PARTICIPANT.<br>• BEFORE STARTING THE INTERVIEW, CHECK THE FACILITY NUMBER AND THE NUMBER OF INTERVIEWS ALREADY DONE AT THE SITE. ALSO CHECK WHICH SECTIONS OF QUESTIONNAIRE A HAVE NOT BEEN COMPLETED.<br>• MAKE SURE THE PERSON YOU ARE ABOUT TO INTERVIEW HAS PROVIDED CONSENT.	
COVER PAGE		
q1_interviewer_name *(required)*	1. NAME OF INTERVIEWER	
q2_facility_name *(required)*	2. SELECT: NAME OF FACILITY	1 Amana Hospital - Regional Referral Hospital<br>2 Bugara Dispensary<br>3 Bukiriro Health Center<br>4 Matemwe PHCU+<br>5 Ilagala Health Center<br>6 Iporoto Dispensary<br>7 Junguni PHCU<br>8 Kasulu District Hospital<br>9 Kifaru Dispensary<br>10 Kimara Health Center<br>11 Kimeya Health Center<br>12 Kintinku Health Center<br>13 Kishogo Dispensary<br>14 Kitahana Dispensary<br>15 Mahonda PHCU+<br>16 Majohe Dispensary<br>17 Marie Stopes Dispensary<br>18 Mbagala Kuu Dispensary<br>19 Mbagala Roundtable Health Center<br>20 Micheweni PHCC<br>21 Mission Mbagala Dispensary<br>22 Mnazi Mmoja Hospital<br>23 Mteke Dispensary<br>24 Mugana DD Hospital<br>25 Muhimbili National Hospital<br>26 Muyenje Dispensary<br>27 Muzdalfa Kiwalani Dispensary<br>28 Mwandiga Dispensary<br>29 Narumu Govt Dispensary<br>30 Nkwenda Health Center<br>31 Ntungwa Dispensary<br>32 Pamila Dispensary<br>33 Rusesa Health Center<br>34 Rusohoko Dispensary<br>35 Ruvuma Regional Hospital<br>36 Semeni Dispensary<br>37 Siha Health Center<br>38 Tabata A Dispensary<br>39 Tumaini Mission Health Center<br>40 Chukwani PHCU+
q3_GPS_QA *(required)*	3. CAPTURE THE GPS LOCATION<br>*Press the button to capture the GPS location at this point in the survey.*	
COVER PAGE		

Field	Question	Answer
note_confirm_intro	INTERVIEWER: PLEASE CONFIRM THE FOLLOWING INFORMATION. IF THERE ARE MISTAKES, MAKE A NOTE AND ENTER AT THE END OF THIS PAGE.	
q4i_facility_id2	q4i. FACILITY ID = '[q4i_facility_id]"	
q4ii_facility_zone2	q4ii. FACILITY ZONE = '[q4ii_facility_zone]"	
q4iii_facility_region2	q4iii. FACILITY REGION = '[q4iii_facility_region]"	
q4iv_facility_location_district2	q4iv. FACILITY DISTRICT = '[q4iv_facility_district]"	
q4v_facility_type2	q4v. FACILITY TYPE = '[q4v_facility_type]"	
q4vi_facility_ownership2	q4vi. FACILITY OWNERSHIP = '[q4vi_facility_ownership]"	
q5_interviewer_comments	q5. INTERVIEWER COMMENTS<br>*Indicate any errors with the information above. Include the correct information.*	
RESPONDENTS (1-5)		
cover_page_note_1	INTERVIEWER INSTRUCTIONS: INDICATE NAMES, JOB TITLES AND CONTACT INFORMATION FOR UP TO 5 RESPONDENTS OF QUESTIONNAIRE A.<br>*NB: Information for only the first respondent is required in order to proceed to the next page. Ask for the information as a question if not already known prior to the interview.*	
group_respondent1	RESPONDENT 1	
q6a_name *(required)*	6a. Name of 1st respondent<br>*First and last name*	
q6b_date *(required)*	6b. Date of interview with 1st respondent<br>*Default is today's date.*	
q6c_title *(required)*	6c. Designation/title of 1st respondent<br>*Job title and designation (e.g. Medical officer in charge)*	
q6d_phone	6d. Phone number of 1st respondent<br>*NB: Not required, but you might need this to follow up with questions.*	
group_respondent2	RESPONDENT 2	
q7a_name	7a. Name of 2nd respondent<br>*First and last name*	
q7b_date	7b. Date of interview with 2nd respondent<br>*Default is today's date.*	
q7c_title	7c. Designation/title of 2nd respondent<br>*Job title and designation (e.g. Medical officer in charge)*	
q7d_phone	7d. Phone number of 2nd respondent<br>*NB: Not required, but you might need this to follow up with questions.*	
group_respondent3	RESPONDENT 3	
q8a_name	8a. Name of 3rd respondent<br>*First and last name*	
q8b_date	8b. Date of interview with 3rd respondent<br>*Default is today's date.*	
q8c_title	8c. Designation/title of 3rd respondent<br>*Job title and designation (e.g. Medical officer in charge)*	
q8d_phone	8d. Phone number of 3rd respondent<br>*NB: Not required, but you might need this to follow up with questions.*	
group_respondent4	RESPONDENT 4	
q9a_name	9a. Name of 4th respondent<br>*First and last name*	
q9b_date	9b. Date of interview with 4th respondent<br>*Default is today's date.*	
q9c_title	9c. Designation/title of 4th respondent<br>*Job title and designation (e.g. Medical officer in charge)*	
q9d_phone	9d. Phone number of 4th respondent<br>*NB: Not required, but you might need this to follow up with questions.*	
group_respondent5	RESPONDENT 5	
q10a_name	10a. Name of 5th respondent<br>*First and last name*	
q10b_date	10b. Date of interview with 5th respondent<br>*Default is today's date.*	
q10c_title	10c. Designation/title of 5th respondent<br>*Job title and designation (e.g. Medical officer in charge)*	
q10d_phone	10d. Phone number of 5th respondent<br>*NB: Not required, but you might need this to follow up with questions.*	
q11_interviewer_comments	11. INTERVIEWER COMMENTS: ENTER ANY RELEVANT NOTES BELOW (E.G. DONE USING PAPER, TRANSCRIBED INTERVIEW, ETC.)	
q12_time_start_qa *(required)*	12. ENTER START TIME OF INTERVIEW<br>*NB: The default is the current time.*	
group_section_one_intro		
section1_start	**SECTION I. FACILITY OVERVIEW, INCLUDING PAYMENTS FOR SERVICES**	

Field	Question	Answer
section_one_skip	INTERVIEWER: WOULD YOU LIKE TO COMPLETE THIS SECTION NOW OR SKIP THIS SECTION AND RETURN TO IT LATER?<br>*You may need to skip if the participant has indicated that s/he cannot answer the questions in this section.*	1 Skip and come back to this section later.<br>2 Do not skip, complete this section now.
FACILITY OWNERSHIP/ORIENTATION<br>*Group relevant when: (selected(${section_one_skip} , '2'))*		
q101_ownership *(required)*	101. Can you tell me whether this facility is publicly or privately owned and operated, or operated as faith-based organization?<br>*NB: An NGO facility is a private facility.*	1 Public<br>2 Private<br>3 Faith-based<br>4 Other
q101a_other	If 101=other, please specify:<br>*Question relevant when: (selected(${q101_ownership} , '4'))*	
q102_profit *(required)*	102. Can you tell me whether this facility offers services as a not-for-profit or for-profit institution?	1 Not-for-profit<br>2 For-profit<br>3 Other
q102a_other	If 102=other, please specify:<br>*Question relevant when: (selected(${q102_profit} , '3'))*	
q103a *(required)*	103a. Are inpatients managed at this facility?<br>*Question relevant when: (selected(${section_one_skip} , '2'))*	1 Yes<br>0 No<br>99 Don't know
q103b *(required)*	103b. Do inpatients pay for any part of the goods or services they receive at this facility? That could include hospitalization, including food while hospitalized; medications; consultations; other procedures; family planning; etc.<br>*If they pay even a small amount, that = YES.*<br>*Question relevant when: (selected(${section_one_skip} , '2')) and (selected(${q103a} , '1'))*	1 Yes, they must pay for everything<br>2 Yes, they pay for some things<br>3 No, everything is free<br>4 Don't know
q103c *(required)*	103c. Do outpatients pay for any part of the goods or services they receive at this facility? That could include hospitalization, including food while hospitalized; medications; consultations; other procedures; family planning; etc.<br>*If they pay even a small amount, that = YES.*<br>*Question relevant when: (selected(${section_one_skip} , '2'))*	1 Yes, they must pay for everything<br>2 Yes, they pay for some things<br>3 No, everything is free<br>4 Don't know
Patient costs<br>*Group relevant when: (selected(${section_one_skip} , '2')) and ((selected(${q103b} , '1')) or (selected(${q103b} , '2')) or (selected(${q103c} , '1')) or (selected(${q103c} , '2')))*		
q104 *(required)*	104. Do inpatients pay part or all of the costs for hospitalization, including food costs?<br>*If they pay for any part of their hospitalization, including food, that = YES.*<br>*Question relevant when: (selected(${q103a} , '1'))*	1 Yes<br>0 No<br>99 Don't know
q105a	105a. If yes, do they pay per day or per stay?<br>*If amount unknown, indicate 999 for missing.*<br>*Question relevant when: (selected(${q103a} , '1'))*	1 Per day<br>2 Per stay<br>99 Don't know
q105b	105b. If yes, how much do they pay for hospitalization (in Shillings)?<br>*If amount unknown, indicate 999 for missing.*<br>*Question relevant when: (selected(${q103a} , '1'))*<br>*Response constrained to: .>=0*	
q106 *(required)*	106. Do out-patients pay part or all of the costs for hospitalization, including food costs?	1 Yes<br>0 No<br>99 Don't know
q107	107. If yes, how much do they pay per visit (in Shillings)?<br>*If amount unknown, indicate 999 for missing.*<br>*Question relevant when: (selected(${q106} , '1'))*<br>*Response constrained to: .>=0*	
q108 *(required)*	108. Do patients pay to receive family planning at your facility? This could include paying for the commodities or the services required to offer the commodities (e.g. consultations, pregnancy tests, etc.).<br>*If they pay even a part of the costs, that = YES.*	1 Yes<br>0 No<br>99 Don't know
q108.1 *(required)*	108.1 You previously noted that inpatients or outpatients pay for a part of the goods and services that they receive at this facility. But in the last few questions you said that they do NOT pay for hospitalizaion, food costs, outpatient fees, or family planning. What other goods or services do they pay for and how much do they pay?<br>*Question relevant when: (selected(${section_one_skip} , '2')) and ((selected(${q103b} , '1')) or (selected(${q103b} , '2'))) and ((selected(${q103c} , '1')) or (selected(${q103c} , '2'))) and (selected(${q104} , '0')) and (selected(${q106} , '0')) and (selected(${q108} , '0'))*	
FAMILY PLANNING METHODS THAT WOMEN PAY FOR:<br>*Group relevant when: not(selected(${section_one_skip} , '1')) and (selected(${q108} , '1'))*		

Field	Question	Answer
q109 *(required)*	109. You noted that women pay for some or all of the family planning services here, including the extra services required to offer the methods (e.g. consultations, pregnancy tests, etc.). Can you tell me for which methods women might need to pay (for the method or related services)? [READ RESPONSE OPTIONS]<br>*Question relevant when: selected(${q108} , '1')*	1 Male or female sterilization<br>2 IUDs - hormonal/LNG system (e.g. Mirena)<br>3 IUDs - copper (e.g. Copper T)<br>4 Implants<br>5 Contraceptive injections<br>6 Oral contraceptive pills<br>7 Emergency contraceptive pills<br>8 Female condoms<br>9 Male condoms<br>10 Other method<br>11 Not sure which methods
q109_other	109 Other. List other method(s):<br>*Question relevant when: selected(${q109} , '10')*	
WOMEN'S FAMILY PLANNING COSTS (IN SHILLINGS) PER METHOD DISPENSED<br>*Group relevant when: (selected(${section_one_skip} , '2'))*		
q110a_pay_sterilization *(required)*	110a How much do they pay for sterilization?<br>*Enter 999 if amount unknown.*<br>*Question relevant when: selected(${q109} , '1')*<br>*Response constrained to: .>=0*	
q110b_pay_LNGsystem *(required)*	110b How much do they pay per IUD (hormonal/LNG system) placement (including the device)?<br>*Enter 999 if amount unknown.*<br>*Question relevant when: selected(${q109} , '2')*<br>*Response constrained to: .>=0*	
q110c_pay_IUDcopper *(required)*	110c How much do they pay per IUD (copper) placement (including the device)?<br>*Enter 999 if amount unknown.*<br>*Question relevant when: selected(${q109} , '3')*<br>*Response constrained to: .>=0*	
q110d_pay_implant *(required)*	110d How much do they pay per implant (including placement, device and removal)?<br>*Enter 999 if amount unknown.*<br>*Question relevant when: selected(${q109} , '4')*<br>*Response constrained to: .>=0*	
q110e_pay_injectable *(required)*	110e How much do they pay per injection?<br>*Enter 999 if amount unknown.*<br>*Question relevant when: selected(${q109} , '5')*<br>*Response constrained to: .>=0*	
q110f_pay_OCP *(required)*	110f How much do they pay per oral contraceptive pill pack (on average across pill types)?<br>*Enter 999 if amount unknown.*<br>*Question relevant when: selected(${q109} , '6')*<br>*Response constrained to: .>=0*	
q110g_pay_EC *(required)*	110g How much do they pay per pack of emergency contraceptive pills?<br>*Enter 999 if amount unknown.*<br>*Question relevant when: selected(${q109} , '7')*<br>*Response constrained to: .>=0*	
q110h_pay_fem_condom *(required)*	110h How much do they pay per female condom?<br>*Enter 999 if amount unknown.*<br>*Question relevant when: selected(${q109} , '8')*<br>*Response constrained to: .>=0*	
q110i_pay_male_condom *(required)*	110i How much do they pay per male condom?<br>*Enter 999 if amount unknown.*<br>*Question relevant when: selected(${q109} , '9')*<br>*Response constrained to: .>=0*	
q110j_pay_other_FP *(required)*	110j How much do they pay for the other method(s)? (You said ([q109_other]).)<br>*Enter 999 if amount unknown.*<br>*Question relevant when: selected(${q109} , '10')*<br>*Response constrained to: .>=0*	
WOMEN'S PAC COSTS<br>*Group relevant when: (selected(${section_one_skip} , '2'))*		

Field	Question	Answer
q111 *(required)*	111. Considering post-abortion care, or PAC, services, would a woman be expected to pay your facility for [READ RESPONSE OPTIONS]?<br>*Select if yes. More than one option allowed.*<br>*Response constrained to: if(selected(., 9), count-selected(.) = 1, count-selected(.) >= 1)*	1 Pre-PAC counselling<br>2 Consultation with a nurse or doctor<br>3 Ultrasound<br>4 Evacuation of the uterus using MVA/metal curettage<br>5 Medication<br>6 Other supplies<br>7 Hospitalization, if hospitalized (including linens, food, etc.)<br>8 Other<br>9 None of the above
q111_other	111 Other. If other supplies or other, please specify:<br>*Question relevant when: (selected(${q111} , '6')) or (selected(${q111} , '8'))*	
WOMEN'S PAC COSTS CONTINUED<br>*Group relevant when: (selected(${section_one_skip} , '2'))*		
q112a *(required)*	112a. Can you estimate out of 100 women who come for PAC services, how many would be expected to pay for any part of their PAC care?<br>*Enter 999 if proportion is unknown.*<br>*Response constrained to: (.>=0 and .<=100) or .=999*	
q112 *(required)*	112. If women pay for any part of their PAC service, how much on average would a woman pay for her PAC care in total (in Shillings)?<br>*Enter 999 if amount unknown.*<br>*Question relevant when: not(selected(${q111} , '9'))*<br>*Response constrained to: .>=0*	
q113 *(required)*	113. Considering post-abortion care, or PAC, services, are women or women's families ever expected to bring their own supplies (i.e. that they would purchase before coming to the facility)? That might include food for overnight hospitalization.	1 Yes<br>0 No<br>99 Don't know
WOMEN'S PAC COSTS CONTINUED<br>*Group relevant when: (selected(${section_one_skip} , '2')) and selected(${q113} , '1')*		
q114 *(required)*	114. Can you tell me what items women or their families might be expected to bring for their PAC services? I'll list some possible options [READ RESPONSE OPTIONS]:	1 Ultrasound results<br>2 MVA kit supplies<br>3 Medication<br>4 Food<br>5 Other
q114_other	114 Other. If other supplies or other, please list the items.<br>*Question relevant when: selected(${q114} , '5')*	
q115 *(required)*	115. Can you estimate out of 100 women who come for PAC services, how many would need to bring any of their own PAC-related supplies to your facility?<br>*Enter 999 if proportion is unknown.*<br>*Response constrained to: (.>=0 and .<=100) or .=999*	
q116 *(required)*	116. For those women who need to bring any PAC supplies to your facility, can you estimate how much they might spend on average?<br>*Enter 999 if amount unknown.*<br>*Response constrained to: .>=0*	
group_section_two_intro		
section2_start	**SECTION II. SERVICE STATISTICS, INCLUDING PAC STATISTICS, PAC DESCRIPTION, HOSPITALIZATION RATES**	
section_two_skip	INTERVIEWER: WOULD YOU LIKE TO COMPLETE THIS SECTION NOW OR SKIP THIS SECTION AND RETURN TO IT LATER?<br>*You may need to skip if the participant has indicated that s/he cannot answer the questions in this section.*	1 Skip and come back to this section later.<br>2 Do not skip, complete this section now.
SERVICE STATISTICS - GENERAL<br>*Group relevant when: (selected(${section_two_skip} , '2'))*		
q201 *(required)*	201. Can you please estimate the total number of patients seen in the last 12 months in your facility (for any condition)?<br>*If the annual total isn't known, take the monthly average and multiply by 12 (or enter 9999 for missing).*	
q202 *(required)*	202. And can you estimate what proportion of all cases were managed as inpatients?<br>*Calculate: Inpatients/Total patients. Please enter a number between 0-100 (or 999 for missing).*<br>*Question relevant when: (selected(${q103a} , '1'))*<br>*Response constrained to: (.>=0 and .<=100) or .=999*	
q203 *(required)*	203. Can you please estimate the number of cases in this facility in the last 12 months that were related to maternal and newborn health, including abortions/miscarriages, in your facility?<br>*If the annual total isn't known, take the monthly average and multiply by 12 (or enter 9999 for missing).*	

If the annual total isn't known, take the monthly average and multiply by 12 (or enter 9999 for missing).

Field	Question	Answer
q204 *(required)*	204. And can you estimate what proportion of all maternal and newborn health patients, including abortions/miscarriages, were managed as inpatients in the last 12 months (Maternal and newborn inpatients/All maternal and newborn cases at the facility)?<br>*Calculate: Maternal and newborn admissions/All maternal and newborn cases at the facility. Please enter a number between 0-100 (or 999 for missing).*<br>*Question relevant when: (selected(${q103a} , '1'))*<br>*Response constrained to: (.>=0 and .<=100) or .=999*	
q205 *(required)*	205. Does this facility maintain statistics on the number of women who come to the facility with post-abortion complications, or PAC?<br>*Question relevant when: (selected(${section_two_skip} , '2'))*	1 Yes<br>0 No<br>99 Don't know
PAC STATS - FROM RECORDS<br>*Group relevant when: (selected(${section_two_skip} , '2')) and selected(${q205} , '1')*		
q206 *(required)*	206. What was the number of women who came to this facility with post-abortion complications in the last year for which statistics are available?<br>*Enter 999 if unknown.*	
q207 *(required)*	207. What is the year referred to in the previous question?<br>*Enter full year (e.g. 2017). If year unknown, enter 2999.*<br>*Response constrained to: regex(., '[2][0][0-1][0-9]')*	
PAC STATS - ESTIMATED<br>*Group relevant when: (selected(${section_two_skip} , '2')) and selected(${q205} , '0') or selected(${q205} , '99')*		
q208 *(required)*	208. Can you estimate the number of women who came to this facility during all of last year with post-abortion complications?<br>*Enter 999 if unknown.*	
PAC REFERRALS FROM OTHER FACILITIES<br>*Group relevant when: (selected(${section_two_skip} , '2'))*		
q209	209. Thinking about all of the women who come to your facility with post-abortion complications, can you please estimate how many out of 100 PAC cases are referred to your facility from the following facilities:<br>*The total should not exceed 100% (but can be less than 100% if the facility has walk-ins).*	
q209a *(required)*	q209a. Clinics<br>*Enter 999 if unknown.*<br>*Response constrained to: (.>=0 and .<=100) or .=999*	
q209b *(required)*	q209b. Health centers/PHCUs<br>*Enter 999 if unknown.*<br>*Response constrained to: (.>=0 and .<=100) or .=999*	
q209c *(required)*	q209c. Dispensaries/PHCU+s<br>*Enter 999 if unknown.*<br>*Response constrained to: (.>=0 and .<=100) or .=999*	
q209d *(required)*	q209d. Cottage hospitals/PHCCs<br>*Enter 999 if unknown.*<br>*Response constrained to: (.>=0 and .<=100) or .=999*	
q209e *(required)*	q209e. District hospitals<br>*Enter 999 if unknown.*<br>*Response constrained to: (.>=0 and .<=100) or .=999*	
q209f *(required)*	q209f. Regional hospitals<br>*Enter 999 if unknown.*<br>*Response constrained to: (.>=0 and .<=100) or .=999*	
q209g *(required)*	q209g. Non-regional (other) hospitals<br>*Enter 999 if unknown.*<br>*Response constrained to: (.>=0 and .<=100) or .=999*	
referral_note	INTERVIEWER: THE TOTAL OF ALL RESPONSES ON THE PREVIOUS SCREEN EXCEEDS 100%. THE TOTAL WAS '[q209_calculate]"%. PLEASE REVIEW THE RESPONSES WITH THE RESPONDENT AND ENSURE THAT THE TOTAL DOES NOT EXCEED 100%.<br>*Question relevant when: (selected(${section_two_skip} , '2')) and ${q209_calculate} >100*	
PAC COMPONENTS AT FACILITY<br>*Group relevant when: (selected(${section_two_skip} , '2'))*		

Field	Question	Answer
q210 *(required)*	210. Considering post-abortion care, or PAC, services, can you please tell me which components are commonly offered at this facility [READ RESPONSE OPTIONS]?<br>*Select all that apply.*	1 Community outreach on PAC availability<br>2 Pre-PAC counselling<br>3 Consultation with nurse or doctor<br>4 Ultrasound<br>5 Evacuation of the uterus using MVA/metal curettage<br>6 Medication<br>7 Providing a referral letter post PAC, in case of continuing complications<br>8 Post PAC contraception<br>9 Other<br>10 Don't know
q210_Other	210 Other. If other, please specify:<br>*Question relevant when: (selected(${q210} , '9'))*	
POST-PAC FAMILY PLANNING<br>*Group relevant when: (selected(${section_two_skip} , '2'))*		
q211 *(required)*	211. If post PAC family planning services are provided at your facility, what methods do you think women are commonly counselled on by your staff?<br>*READ RESPONSE OPTIONS*	1 Male or female sterilization<br>2 IUDs - hormonal/LNG system (e.g. Mirena)<br>3 IUDs - copper (e.g. Copper T)<br>4 Implants<br>5 Contraceptive injections<br>6 Oral contraceptive pills<br>7 Emergency contraceptive pills<br>8 Female condoms<br>9 Male condoms<br>10 Other method<br>11 Not sure which methods
q211_Other	211 Other. If other, please specify:<br>*Question relevant when: (selected(${q211} , '10'))*	
PROPORTION OF WOMEN WITH COMPLICATION TYPE<br>*Group relevant when: (selected(${section_two_skip} , '2')) and (selected(${q103a} , '1'))*		
q212	212. Think about 100 women treated in this facility as INpatients in the last 12 months for post-abortion complications. Of those 100 inpatient women, can you estimate how many were treated for each of the following complications?<br>*READ RESPONSE OPTIONS*	
note_q212	NB: Women can have more than one complication type, so the proportions can sum to more than 100%.	
q212a *(required)*	q212a. Incomplete abortion<br>*Enter 999 if proportion is unknown.*<br>*Response constrained to: (.>=0 and .<=100) or .=999*	
q212b *(required)*	q212b. Sepsis<br>*Enter 999 if proportion is unknown.*<br>*Response constrained to: (.>=0 and .<=100) or .=999*	
q212c *(required)*	q212c. Shock<br>*Enter 999 if proportion is unknown.*<br>*Response constrained to: (.>=0 and .<=100) or .=999*	
q212d *(required)*	q212d. Cervical/vaginal lacerations<br>*Enter 999 if proportion is unknown.*<br>*Response constrained to: (.>=0 and .<=100) or .=999*	
q212e *(required)*	q212e. Uterine laceration/perforation<br>*Enter 999 if proportion is unknown.*<br>*Response constrained to: (.>=0 and .<=100) or .=999*	
group_213_outpatient_complications<br>*Group relevant when: (selected(${section_two_skip} , '2'))*		
q213	213. Now think about 100 women treated in this facility as OUTpatients in the last 12 months for post-abortion complications. Of those 100 outpatient women, can you estimate how many were treated for each of the following complications?<br>*READ RESPONSE OPTIONS*	
note_q213	NB: Women can have more than one complication type, so the proportions can sum to more than 100%.	
q213a *(required)*	q213a. Incomplete abortion<br>*Enter 999 if proportion is unknown.*	

Field	Question	Answer
	Response constrained to: (.>=0 and .<=100) or .=999	
q213b *(required)*	q213b. Sepsis<br>*Enter 999 if proportion is unknown.*<br>*Response constrained to: (.>=0 and .<=100) or .=999*	
q213c *(required)*	q213c. Shock<br>*Enter 999 if proportion is unknown.*<br>*Response constrained to: (.>=0 and .<=100) or .=999*	
q213d *(required)*	q213d. Cervical/vaginal lacerations<br>*Enter 999 if proportion is unknown.*<br>*Response constrained to: (.>=0 and .<=100) or .=999*	
q213e *(required)*	q213e. Uterine laceration/perforation<br>*Enter 999 if proportion is unknown.*<br>*Response constrained to: (.>=0 and .<=100) or .=999*	
group_214_nights_hospitalized<br>*Group relevant when: (selected(${section_two_skip} , '2')) and (selected(${q103a} , '1'))*		
q214	214. Thinking only about women who were hospitalized, can you estimate the average number of nights of hospitalization for each of the following complications?<br>*READ RESPONSE OPTIONS*	
q214a *(required)*	214a. Incomplete abortion<br>*Enter 999 if proportion is unknown.*<br>*Response constrained to: .>=0*	
q214b *(required)*	214b. Sepsis<br>*Enter 999 if proportion is unknown.*<br>*Response constrained to: .>=0*	
q214c *(required)*	214c. Shock<br>*Enter 999 if proportion is unknown.*<br>*Response constrained to: .>=0*	
q214d *(required)*	214d. Cervical/vaginal lacerations<br>*Enter 999 if proportion is unknown.*<br>*Response constrained to: .>=0*	
q214e *(required)*	214e. Uterine laceration/perforation<br>*Enter 999 if proportion is unknown.*<br>*Response constrained to: .>=0*	
q215 *(required)*	215. There are several other post-abortion complications that may occur rarely (e.g., peritonitis, renal failure, heart failure, psychosis, etc.). Out of 1,000 post-abortion complications, can you estimate how many would consist of these rare complications, i.e., complications not listed in my last question?<br>*Enter 99999 if proportion is unknown.*<br>*Question relevant when: (selected(${section_two_skip} , '2'))*<br>*Response constrained to: (.>=0 and .<=1000) or .=99999*	
group_section_three_intro		
section3_start	**III. PAC MANAGEMENT BY FACILITY STAFF**	
section_three_skip	INTERVIEWER: WOULD YOU LIKE TO COMPLETE THIS SECTION NOW OR SKIP THIS SECTION AND RETURN TO IT LATER?<br>*You may need to skip if the participant has indicated that s/he cannot answer the questions in this section.*	1 Skip and come back to this section later.<br>2 Do not skip, complete this section now.
group_301_number_staff<br>*Group relevant when: selected(${section_three_skip} , '2')*		
q301	q301. Can you tell me how many of the following staff types are employed by this facility?<br>*Enter 999 if amount unknown.*	
q301a *(required)*	q301a. Obstetrician/gynecologist<br>*Response constrained to: .>=0*	
q301b *(required)*	q301b. Anesthetist<br>*Response constrained to: .>=0*	
q301c *(required)*	q301c. Doctor/medical officer<br>*Response constrained to: .>=0*	
q301d *(required)*	q301d. Assistant medical officer<br>*Response constrained to: .>=0*	
q301e *(required)*	q301e. Clinical officer<br>*Response constrained to: .>=0*	
q301f *(required)*	q301f. Assistant clinical officer<br>*Response constrained to: .>=0*	
q301g *(required)*	q301g. Nurse/Nursing officer/Assistant nursing officer<br>*Response constrained to: .>=0*	
q301h *(required)*	q301h. Midwife/Enrolled nurse<br>*Response constrained to: .>=0*	

Field	Question	Answer
q301i *(required)*	q301i. Nursing Assistant<br>*Response constrained to: .>=0*	

q301j *(required)*	q301j. Lab Technician<br>*Response constrained to: .>=0*	
q301k *(required)*	q301k. Sonographer<br>*Response constrained to: .>=0*	
q301l *(required)*	q301l. Pharmacist<br>*Response constrained to: .>=0*	
q301m *(required)*	q301m. Pharmacy Assistant<br>*Response constrained to: .>=0*	
q301n *(required)*	q301n. Drug dispenser<br>*Response constrained to: .>=0*	
q301o *(required)*	q301o. Counselor<br>*Do not include nurses who act as counselors.*<br>*Response constrained to: .>=0*	
note_302-305	INTERVIEWER NOTE:<br><br>*For the following questions 302-305, alert the respondent that the questions will be repetitive.*<br><br>*Questions 302 and 303 ask about the staff that see women for incomplete abortion, then sepsis, then shock, and so on. Question 302 asks about outpatient care; while question 303 asks about inpatient care.*<br><br>*Questions 304 and 305 then ask about the number of minutes that each staff person sees a woman who is treated for incomplete abortion, sepsis, shock and so on. Question 304 asks about outpatient care, and question 305 asks about inpatient care.*<br><br>*Any staff members that are not listed as employed here in the first question will be skipped in subsequent questions. Likewise, if you say that a certain kind of staff member doesn't treat patients for the conditions we ask about, then you won't be asked about the time required.*<br>*Question relevant when: selected(${section_three_skip} , '2')*	
group_302a_outstaff_incom_ab<br>*Group relevant when: selected(${section_three_skip} , '2')*		
generated_note_name_201	302a. Can you estimate how many women, out of every 10 women managed as outpatients in this facility for incomplete abortion, are seen by a [LIST EACH TYPE OF HEALTH CARE WORKER]?<br>*Enter a number between 0-10 or 99 if amount unknown.*	
q302aa *(required)*	q302aa. Obstetrician/gynecologist<br>*Question relevant when: ${q301a} >0*<br>*Response constrained to: (.>=0 and .<=10) or .=99*	
q302ab *(required)*	q302ab. Anesthetist<br>*Question relevant when: ${q301b} >0*<br>*Response constrained to: (.>=0 and .<=10) or .=99*	
q302ac *(required)*	q302ac. Doctor/medical officer<br>*Question relevant when: ${q301c} >0*<br>*Response constrained to: (.>=0 and .<=10) or .=99*	
q302ad *(required)*	q302ad. Assistant medical officer<br>*Question relevant when: ${q301d} >0*<br>*Response constrained to: (.>=0 and .<=10) or .=99*	
q302ae *(required)*	q302ae. Clinical officer<br>*Question relevant when: ${q301e} >0*<br>*Response constrained to: (.>=0 and .<=10) or .=99*	
q302af *(required)*	q302af. Assistant clinical officer<br>*Question relevant when: ${q301f} >0*<br>*Response constrained to: (.>=0 and .<=10) or .=99*	
q302ag *(required)*	q302ag. Nurse/Nursing officer/Assistant nursing officer<br>*Question relevant when: ${q301g} >0*<br>*Response constrained to: (.>=0 and .<=10) or .=99*	
q302ah *(required)*	q302ah. Midwife/Enrolled nurse<br>*Question relevant when: ${q301h} >0*<br>*Response constrained to: (.>=0 and .<=10) or .=99*	
q302ai *(required)*	q302ai. Nursing Assistant<br>*Question relevant when: ${q301i} >0*<br>*Response constrained to: (.>=0 and .<=10) or .=99*	
q302aj *(required)*	q302aj. Lab Technician	

Field	Question	Answer
	Question relevant when: ${q301j} >0<br>*Response constrained to: (.>=0 and .<=10) or .=99*	
q302ak *(required)*	q302ak. Sonographer<br>*Question relevant when: ${q301k} >0*<br>*Response constrained to: (.>=0 and .<=10) or .=99*	
q302al *(required)*	q302al. Pharmacist<br>*Question relevant when: ${q301l} >0*<br>*Response constrained to: (.>=0 and .<=10) or .=99*	
q302am *(required)*	q302am. Pharmacy Assistant<br>*Question relevant when: ${q301m} >0*<br>*Response constrained to: (.>=0 and .<=10) or .=99*	
q302an *(required)*	q302an. Drug dispenser<br>*Question relevant when: ${q301n} >0*<br>*Response constrained to: (.>=0 and .<=10) or .=99*	
q302ao *(required)*	q302ao. Counselor<br>*Question relevant when: ${q301o} >0*<br>*Response constrained to: (.>=0 and .<=10) or .=99*	
group_302b_outstaff_sepsis<br>*Group relevant when: selected(${section_three_skip} , '2')*		
generated_note_name_219	302b. Can you estimate how many women, out of every 10 women managed as outpatients at this facility for sepsis, are seen by a [LIST EACH TYPE OF HEALTH CARE WORKER]?<br>*Enter a number between 0-10 or 99 if amount unknown.*	
q302ba *(required)*	q302ba. Obstetrician/gynecologist<br>*Question relevant when: ${q301a} >0*<br>*Response constrained to: (.>=0 and .<=10) or .=99*	
q302bb *(required)*	q302bb. Anesthetist<br>*Question relevant when: ${q301b} >0*<br>*Response constrained to: (.>=0 and .<=10) or .=99*	
q302bc *(required)*	q302bc. Doctor/medical officer<br>*Question relevant when: ${q301c} >0*<br>*Response constrained to: (.>=0 and .<=10) or .=99*	
q302bd *(required)*	q302bd. Assistant medical officer<br>*Question relevant when: ${q301d} >0*<br>*Response constrained to: (.>=0 and .<=10) or .=99*	
q302be *(required)*	q302be. Clinical officer<br>*Question relevant when: ${q301e} >0*<br>*Response constrained to: (.>=0 and .<=10) or .=99*	
q302bf *(required)*	q302bf. Assistant clinical officer<br>*Question relevant when: ${q301f} >0*<br>*Response constrained to: (.>=0 and .<=10) or .=99*	
q302bg *(required)*	q302bg. Nurse/Nursing officer/Assistant nursing officer<br>*Question relevant when: ${q301g} >0*<br>*Response constrained to: (.>=0 and .<=10) or .=99*	
q302bh *(required)*	q302bh. Midwife/Enrolled nurse<br>*Question relevant when: ${q301h} >0*<br>*Response constrained to: (.>=0 and .<=10) or .=99*	
q302bi *(required)*	q302bi. Nursing Assistant<br>*Question relevant when: ${q301i} >0*<br>*Response constrained to: (.>=0 and .<=10) or .=99*	
q302bj *(required)*	q302bj. Lab Technician<br>*Question relevant when: ${q301j} >0*<br>*Response constrained to: (.>=0 and .<=10) or .=99*	
q302bk *(required)*	q302bk. Sonographer<br>*Question relevant when: ${q301k} >0*<br>*Response constrained to: (.>=0 and .<=10) or .=99*	
q302bl *(required)*	q302bl. Pharmacist<br>*Question relevant when: ${q301l} >0*<br>*Response constrained to: (.>=0 and .<=10) or .=99*	
q302bm *(required)*	q302bm. Pharmacy Assistant<br>*Question relevant when: ${q301m} >0*<br>*Response constrained to: (.>=0 and .<=10) or .=99*	
q302bn *(required)*	q302bn. Drug dispenser<br>*Question relevant when: ${q301n} >0*	

Question relevant when: ${q301m} >0

Response constrained to: (.>=0 and .<=10) or .=99

Field	Question	Answer

Field	Question	Answer
q302bo *(required)*	q302bo. Counselor<br>*Question relevant when: ${q301o} >0*<br>*Response constrained to: (.>=0 and .<=10) or .=99*	
group_q302c_outstaff_shock<br>*Group relevant when: selected(${section_three_skip} , '2')*		
generated_note_name_237	302c. Can you estimate how many women, out of every 10 women managed as outpatients at this facility for shock, are seen by a [LIST EACH TYPE OF HEALTH CARE WORKER]?<br>*Enter a number between 0-10 or 99 if amount unknown.*	
q302ca *(required)*	q302ca. Obstetrician/gynecologist<br>*Question relevant when: ${q301a} >0*<br>*Response constrained to: (.>=0 and .<=10) or .=99*	
q302cb *(required)*	q302cb. Anesthetist<br>*Question relevant when: ${q301b} >0*<br>*Response constrained to: (.>=0 and .<=10) or .=99*	
q302cc *(required)*	q302cc. Doctor/medical officer<br>*Question relevant when: ${q301c} >0*<br>*Response constrained to: (.>=0 and .<=10) or .=99*	
q302cd *(required)*	q302cd. Assistant medical officer<br>*Question relevant when: ${q301d} >0*<br>*Response constrained to: (.>=0 and .<=10) or .=99*	
q302ce *(required)*	q302ce. Clinical officer<br>*Question relevant when: ${q301e} >0*<br>*Response constrained to: (.>=0 and .<=10) or .=99*	
q302cf *(required)*	q302cf. Assistant clinical officer<br>*Question relevant when: ${q301f} >0*<br>*Response constrained to: (.>=0 and .<=10) or .=99*	
q302cg *(required)*	q302cg. Nurse/Nursing officer/Assistant nursing officer<br>*Question relevant when: ${q301g} >0*<br>*Response constrained to: (.>=0 and .<=10) or .=99*	
q302ch *(required)*	q302ch. Midwife/Enrolled nurse<br>*Question relevant when: ${q301h} >0*<br>*Response constrained to: (.>=0 and .<=10) or .=99*	
q302ci *(required)*	q302ci. Nursing Assistant<br>*Question relevant when: ${q301i} >0*<br>*Response constrained to: (.>=0 and .<=10) or .=99*	
q302cj *(required)*	q302cj. Lab Technician<br>*Question relevant when: ${q301j} >0*<br>*Response constrained to: (.>=0 and .<=10) or .=99*	
q302ck *(required)*	q302ck. Sonographer<br>*Question relevant when: ${q301k} >0*<br>*Response constrained to: (.>=0 and .<=10) or .=99*	
q302cl *(required)*	q302cl. Pharmacist<br>*Question relevant when: ${q301l} >0*<br>*Response constrained to: (.>=0 and .<=10) or .=99*	
q302cm *(required)*	q302cm. Pharmacy Assistant<br>*Question relevant when: ${q301m} >0*<br>*Response constrained to: (.>=0 and .<=10) or .=99*	
q302cn *(required)*	q302cn. Drug dispenser<br>*Question relevant when: ${q301n} >0*<br>*Response constrained to: (.>=0 and .<=10) or .=99*	
q302co *(required)*	q302co. Counselor<br>*Question relevant when: ${q301o} >0*<br>*Response constrained to: (.>=0 and .<=10) or .=99*	
group_q302d_outstaff_cerv_lac<br>*Group relevant when: selected(${section_three_skip} , '2')*		
generated_note_name_255	302d. Can you estimate how many women, out of every 10 women managed as outpatients at this facility for cervical/vaginal lacerations, are seen by a [LIST EACH TYPE OF HEALTH CARE WORKER]?<br>*Enter a number between 0-10 or 99 if amount unknown.*	
q302da *(required)*	q302da. Obstetrician/gynecologist<br>*Question relevant when: ${q301a} >0*<br>*Response constrained to: (.>=0 and .<=10) or .=99*	

Field	Question	Answer
q302db *(required)*	q302db. Anesthetist<br>*Question relevant when: ${q301b} >0*<br>*Response constrained to: (.>=0 and .<=10) or .=99*	
q302dc *(required)*	q302dc. Doctor/medical officer<br>*Question relevant when: ${q301c} >0*<br>*Response constrained to: (.>=0 and .<=10) or .=99*	
q302dd *(required)*	q302dd. Assistant medical officer<br>*Question relevant when: ${q301d} >0*<br>*Response constrained to: (.>=0 and .<=10) or .=99*	
q302de *(required)*	q302de. Clinical officer<br>*Question relevant when: ${q301e} >0*<br>*Response constrained to: (.>=0 and .<=10) or .=99*	
q302df *(required)*	q302df. Assistant clinical officer<br>*Question relevant when: ${q301f} >0*<br>*Response constrained to: (.>=0 and .<=10) or .=99*	
q302dg *(required)*	q302dg. Nurse/Nursing officer/Assistant nursing officer<br>*Question relevant when: ${q301g} >0*<br>*Response constrained to: (.>=0 and .<=10) or .=99*	
q302dh *(required)*	q302dh. Midwife/Enrolled nurse<br>*Question relevant when: ${q301h} >0*<br>*Response constrained to: (.>=0 and .<=10) or .=99*	
q302di *(required)*	q302di. Nursing Assistant<br>*Question relevant when: ${q301i} >0*<br>*Response constrained to: (.>=0 and .<=10) or .=99*	
q302dj *(required)*	q302dj. Lab Technician<br>*Question relevant when: ${q301j} >0*<br>*Response constrained to: (.>=0 and .<=10) or .=99*	
q302dk *(required)*	q302dk. Sonographer<br>*Question relevant when: ${q301k} >0*<br>*Response constrained to: (.>=0 and .<=10) or .=99*	
q302dl *(required)*	q302dl. Pharmacist<br>*Question relevant when: ${q301l} >0*<br>*Response constrained to: (.>=0 and .<=10) or .=99*	
q302dm *(required)*	q302dm. Pharmacy Assistant<br>*Question relevant when: ${q301m} >0*<br>*Response constrained to: (.>=0 and .<=10) or .=99*	
q302dn *(required)*	q302dn. Drug dispenser<br>*Question relevant when: ${q301n} >0*<br>*Response constrained to: (.>=0 and .<=10) or .=99*	
q302do *(required)*	q302do. Counselor<br>*Question relevant when: ${q301o} >0*<br>*Response constrained to: (.>=0 and .<=10) or .=99*	
group_302e_outstaff_uter_perf<br>*Group relevant when: selected(${section_three_skip} , '2')*		
generated_note_name_273	302e. Can you estimate how many women, out of every 10 women managed as outpatients at this facility for vaginal or uterine perforations, are seen by a [LIST EACH TYPE OF HEALTH CARE WORKER]?<br>*Enter a number between 0-10 or 99 if amount unknown.*	
q302ea *(required)*	q302ea. Obstetrician/gynecologist<br>*Question relevant when: ${q301a} >0*<br>*Response constrained to: (.>=0 and .<=10) or .=99*	
q302eb *(required)*	q302eb. Anesthetist<br>*Question relevant when: ${q301b} >0*<br>*Response constrained to: (.>=0 and .<=10) or .=99*	
q302ec *(required)*	q302ec. Doctor/medical officer<br>*Question relevant when: ${q301c} >0*<br>*Response constrained to: (.>=0 and .<=10) or .=99*	
q302ed *(required)*	q302ed. Assistant medical officer<br>*Question relevant when: ${q301d} >0*<br>*Response constrained to: (.>=0 and .<=10) or .=99*	
q302ee *(required)*	q302ee. Clinical officer<br>*Question relevant when: ${q301e} >0*<br>*Response constrained to: (.>=0 and .<=10) or .=99*	
q302ef *(required)*	q302ef. Assistant clinical officer	

Field	Question	Answer
q302ef *(required)*	q302ef. Assistant clinical officer<br>*Question relevant when: ${q301f} >0*<br>*Response constrained to: (.>=0 and .<=10) or .=99*	
q302eg *(required)*	q302eg. Nurse/Nursing officer/Assistant nursing officer<br>*Question relevant when: ${q301g} >0*<br>*Response constrained to: (.>=0 and .<=10) or .=99*	
q302eh *(required)*	q302eh. Midwife/Enrolled nurse<br>*Question relevant when: ${q301h} >0*<br>*Response constrained to: (.>=0 and .<=10) or .=99*	
q302ei *(required)*	q302ei. Nursing Assistant<br>*Question relevant when: ${q301i} >0*<br>*Response constrained to: (.>=0 and .<=10) or .=99*	
q302ej *(required)*	q302ej. Lab Technician<br>*Question relevant when: ${q301j} >0*<br>*Response constrained to: (.>=0 and .<=10) or .=99*	
q302ek *(required)*	q302ek. Sonographer<br>*Question relevant when: ${q301k} >0*<br>*Response constrained to: (.>=0 and .<=10) or .=99*	
q302el *(required)*	q302el. Pharmacist<br>*Question relevant when: ${q301l} >0*<br>*Response constrained to: (.>=0 and .<=10) or .=99*	
q302em *(required)*	q302em. Pharmacy Assistant<br>*Question relevant when: ${q301m} >0*<br>*Response constrained to: (.>=0 and .<=10) or .=99*	
q302en *(required)*	q302en. Drug dispenser<br>*Question relevant when: ${q301n} >0*<br>*Response constrained to: (.>=0 and .<=10) or .=99*	
q302eo *(required)*	q302eo. Counselor<br>*Question relevant when: ${q301o} >0*<br>*Response constrained to: (.>=0 and .<=10) or .=99*	
group_303a_instaff_incom_ab<br>*Group relevant when: (selected(${section_three_skip} , '2')) and (selected(${q103a} , '1'))*		
generated_note_name_291	303a. Can you estimate how many women, out of every 10 women managed as inpatients in this facility for incomplete abortion, are seen by a [LIST EACH TYPE OF HEALTH CARE WORKER]?<br>*Enter a number between 0-10 or 99 if amount unknown.*	
q303aa *(required)*	q303aa. Obstetrician/gynecologist<br>*Question relevant when: ${q301a} >0*<br>*Response constrained to: (.>=0 and .<=10) or .=99*	
q303ab *(required)*	q303ab. Anesthetist<br>*Question relevant when: ${q301b} >0*<br>*Response constrained to: (.>=0 and .<=10) or .=99*	
q303ac *(required)*	q303ac. Doctor/medical officer<br>*Question relevant when: ${q301c} >0*<br>*Response constrained to: (.>=0 and .<=10) or .=99*	
q303ad *(required)*	q303ad. Assistant medical officer<br>*Question relevant when: ${q301d} >0*<br>*Response constrained to: (.>=0 and .<=10) or .=99*	
q303ae *(required)*	q303ae. Clinical officer<br>*Question relevant when: ${q301e} >0*<br>*Response constrained to: (.>=0 and .<=10) or .=99*	
q303af *(required)*	q303af. Assistant clinical officer<br>*Question relevant when: ${q301f} >0*<br>*Response constrained to: (.>=0 and .<=10) or .=99*	
q303ag *(required)*	q303ag. Nurse/Nursing officer/Assistant nursing officer<br>*Question relevant when: ${q301g} >0*<br>*Response constrained to: (.>=0 and .<=10) or .=99*	
q303ah *(required)*	q303ah. Midwife/Enrolled nurse<br>*Question relevant when: ${q301h} >0*<br>*Response constrained to: (.>=0 and .<=10) or .=99*	
q303ai *(required)*	q303ai. Nursing Assistant<br>*Question relevant when: ${q301i} >0*<br>*Response constrained to: (.>=0 and .<=10) or .=99*	

Field	Question	Answer
q303aj *(required)*	q303aj. Lab Technician<br>*Question relevant when: ${q301j} >0*<br>*Response constrained to: (.>=0 and .<=10) or .=99*	

q303ak *(required)*	q303ak. Sonographer<br>*Question relevant when: ${q301k} >0*<br>*Response constrained to: (.>=0 and .<=10) or .=99*	
q303al *(required)*	q303al. Pharmacist<br>*Question relevant when: ${q301l} >0*<br>*Response constrained to: (.>=0 and .<=10) or .=99*	
q303am *(required)*	q303am. Pharmacy Assistant<br>*Question relevant when: ${q301m} >0*<br>*Response constrained to: (.>=0 and .<=10) or .=99*	
q303an *(required)*	q303an. Drug dispenser<br>*Question relevant when: ${q301n} >0*<br>*Response constrained to: (.>=0 and .<=10) or .=99*	
q303ao *(required)*	q303ao. Counselor<br>*Question relevant when: ${q301o} >0*<br>*Response constrained to: (.>=0 and .<=10) or .=99*	
group_303b_instaff_sepsis<br>*Group relevant when: (selected(${section_three_skip} , '2')) and (selected(${q103a} , '1'))*		
generated_note_name_309	303b. Can you estimate how many women, out of every 10 women managed as inpatients at this facility for sepsis, are seen by a [LIST EACH TYPE OF HEALTH CARE WORKER]?<br>*Enter a number between 0-10 or 99 if amount unknown.*	
q303ba *(required)*	q303ba. Obstetrician/gynecologist<br>*Question relevant when: ${q301a} >0*<br>*Response constrained to: (.>=0 and .<=10) or .=99*	
q303bb *(required)*	q303bb. Anesthetist<br>*Question relevant when: ${q301b} >0*<br>*Response constrained to: (.>=0 and .<=10) or .=99*	
q303bc *(required)*	q303bc. Doctor/medical officer<br>*Question relevant when: ${q301c} >0*<br>*Response constrained to: (.>=0 and .<=10) or .=99*	
q303bd *(required)*	q303bd. Assistant medical officer<br>*Question relevant when: ${q301d} >0*<br>*Response constrained to: (.>=0 and .<=10) or .=99*	
q303be *(required)*	q303be. Clinical officer<br>*Question relevant when: ${q301e} >0*<br>*Response constrained to: (.>=0 and .<=10) or .=99*	
q303bf *(required)*	q303bf. Assistant clinical officer<br>*Question relevant when: ${q301f} >0*<br>*Response constrained to: (.>=0 and .<=10) or .=99*	
q303bg *(required)*	q303bg. Nurse/Nursing officer/Assistant nursing officer<br>*Question relevant when: ${q301g} >0*<br>*Response constrained to: (.>=0 and .<=10) or .=99*	
q303bh *(required)*	q303bh. Midwife/Enrolled nurse<br>*Question relevant when: ${q301h} >0*<br>*Response constrained to: (.>=0 and .<=10) or .=99*	
q303bi *(required)*	q303bi. Nursing Assistant<br>*Question relevant when: ${q301i} >0*<br>*Response constrained to: (.>=0 and .<=10) or .=99*	
q303bj *(required)*	q303bj. Lab Technician<br>*Question relevant when: ${q301j} >0*<br>*Response constrained to: (.>=0 and .<=10) or .=99*	
q303bk *(required)*	q303bk. Sonographer<br>*Question relevant when: ${q301k} >0*<br>*Response constrained to: (.>=0 and .<=10) or .=99*	
q303bl *(required)*	q303bl. Pharmacist<br>*Question relevant when: ${q301l} >0*<br>*Response constrained to: (.>=0 and .<=10) or .=99*	
q303bm *(required)*	q303bm. Pharmacy Assistant<br>*Question relevant when: ${q301m} >0*<br>*Response constrained to: (.>=0 and .<=10) or .=99*	

Field	Question	Answer
q303bn *(required)*	q303bn. Drug dispenser<br>*Question relevant when: ${q301n} >0*<br>*Response constrained to: (.>=0 and .<=10) or .=99*	

Field	Question	Answer
q303bo *(required)*	q303bo. Counselor<br>*Question relevant when: ${q301o} >0*<br>*Response constrained to: (.>=0 and .<=10) or .=99*	
group_q303c_instaff_shock<br>*Group relevant when: (selected(${section_three_skip} , '2')) and (selected(${q103a} , '1'))*		
generated_note_name_327	303c. Can you estimate how many women, out of every 10 women managed as inpatients at this facility for shock, are seen by a [LIST EACH TYPE OF HEALTH CARE WORKER]?<br>*Enter a number between 0-10 or 99 if amount unknown.*	
q303ca *(required)*	q303ca. Obstetrician/gynecologist<br>*Question relevant when: ${q301a} >0*<br>*Response constrained to: (.>=0 and .<=10) or .=99*	
q303cb *(required)*	q303cb. Anesthetist<br>*Question relevant when: ${q301b} >0*<br>*Response constrained to: (.>=0 and .<=10) or .=99*	
q303cc *(required)*	q303cc. Doctor/medical officer<br>*Question relevant when: ${q301c} >0*<br>*Response constrained to: (.>=0 and .<=10) or .=99*	
q303cd *(required)*	q303cd. Assistant medical officer<br>*Question relevant when: ${q301d} >0*<br>*Response constrained to: (.>=0 and .<=10) or .=99*	
q303ce *(required)*	q303ce. Clinical officer<br>*Question relevant when: ${q301e} >0*<br>*Response constrained to: (.>=0 and .<=10) or .=99*	
q303cf *(required)*	q303cf. Assistant clinical officer<br>*Question relevant when: ${q301f} >0*<br>*Response constrained to: (.>=0 and .<=10) or .=99*	
q303cg *(required)*	q303cg. Nurse/Nursing officer/Assistant nursing officer<br>*Question relevant when: ${q301g} >0*<br>*Response constrained to: (.>=0 and .<=10) or .=99*	
q303ch *(required)*	q303ch. Midwife/Enrolled nurse<br>*Question relevant when: ${q301h} >0*<br>*Response constrained to: (.>=0 and .<=10) or .=99*	
q303ci *(required)*	q303ci. Nursing Assistant<br>*Question relevant when: ${q301i} >0*<br>*Response constrained to: (.>=0 and .<=10) or .=99*	
q303cj *(required)*	q303cj. Lab Technician<br>*Question relevant when: ${q301j} >0*<br>*Response constrained to: (.>=0 and .<=10) or .=99*	
q303ck *(required)*	q303ck. Sonographer<br>*Question relevant when: ${q301k} >0*<br>*Response constrained to: (.>=0 and .<=10) or .=99*	
q303cl *(required)*	q303cl. Pharmacist<br>*Question relevant when: ${q301l} >0*<br>*Response constrained to: (.>=0 and .<=10) or .=99*	
q303cm *(required)*	q303cm. Pharmacy Assistant<br>*Question relevant when: ${q301m} >0*<br>*Response constrained to: (.>=0 and .<=10) or .=99*	
q303cn *(required)*	q303cn. Drug dispenser<br>*Question relevant when: ${q301n} >0*<br>*Response constrained to: (.>=0 and .<=10) or .=99*	
q303co *(required)*	q303co. Counselor<br>*Question relevant when: ${q301o} >0*<br>*Response constrained to: (.>=0 and .<=10) or .=99*	
group_q303d_instaff_cerv_lac<br>*Group relevant when: (selected(${section_three_skip} , '2')) and (selected(${q103a} , '1'))*		
generated_note_name_345	303d. Can you estimate how many women, out of every 10 women managed as inpatients at this facility for cervical/vaginal lacerations, are seen by a [LIST EACH TYPE OF HEALTH CARE WORKER]?<br>*Enter a number between 0-10 or 99 if amount unknown.*	
q303da *(required)*	q303da. Obstetrician/gynecologist	

Field	Question	Answer
q303da *(required)*	q303da. Obstetrician/gynecologist<br>*Question relevant when: ${q301a} >0*<br>*Response constrained to: (.>=0 and .<=10) or .=99*	
q303db *(required)*	q303db. Anesthetist<br>*Question relevant when: ${q301b} >0*<br>*Response constrained to: (.>=0 and .<=10) or .=99*	
q303dc *(required)*	q303dc. Doctor/medical officer<br>*Question relevant when: ${q301c} >0*<br>*Response constrained to: (.>=0 and .<=10) or .=99*	
q303dd *(required)*	q303dd. Assistant medical officer<br>*Question relevant when: ${q301d} >0*<br>*Response constrained to: (.>=0 and .<=10) or .=99*	
q303de *(required)*	q303de. Clinical officer<br>*Question relevant when: ${q301e} >0*<br>*Response constrained to: (.>=0 and .<=10) or .=99*	
q303df *(required)*	q303df. Assistant clinical officer<br>*Question relevant when: ${q301f} >0*<br>*Response constrained to: (.>=0 and .<=10) or .=99*	
q303dg *(required)*	q303dg. Nurse/Nursing officer/Assistant nursing officer<br>*Question relevant when: ${q301g} >0*<br>*Response constrained to: (.>=0 and .<=10) or .=99*	
q303dh *(required)*	q303dh. Midwife/Enrolled nurse<br>*Question relevant when: ${q301h} >0*<br>*Response constrained to: (.>=0 and .<=10) or .=99*	
q303di *(required)*	q303di. Nursing Assistant<br>*Question relevant when: ${q301i} >0*<br>*Response constrained to: (.>=0 and .<=10) or .=99*	
q303dj *(required)*	q303dj. Lab Technician<br>*Question relevant when: ${q301j} >0*<br>*Response constrained to: (.>=0 and .<=10) or .=99*	
q303dk *(required)*	q303dk. Sonographer<br>*Question relevant when: ${q301k} >0*<br>*Response constrained to: (.>=0 and .<=10) or .=99*	
q303dl *(required)*	q303dl. Pharmacist<br>*Question relevant when: ${q301l} >0*<br>*Response constrained to: (.>=0 and .<=10) or .=99*	
q303dm *(required)*	q303dm. Pharmacy Assistant<br>*Question relevant when: ${q301m} >0*<br>*Response constrained to: (.>=0 and .<=10) or .=99*	
q303dn *(required)*	q303dn. Drug dispenser<br>*Question relevant when: ${q301n} >0*<br>*Response constrained to: (.>=0 and .<=10) or .=99*	
q303do *(required)*	q303do. Counselor<br>*Question relevant when: ${q301o} >0*<br>*Response constrained to: (.>=0 and .<=10) or .=99*	
group_303e_instaff_uter_perf<br>*Group relevant when: (selected(${section_three_skip} , '2')) and (selected(${q103a} , '1'))*		
generated_note_name_363	303e. Can you estimate how many women, out of every 10 women managed as inpatients at this facility for vaginal or uterine perforations, are seen by a [LIST EACH TYPE OF HEALTH CARE WORKER]?<br>*Enter a number between 0-10 or 99 if amount unknown.*	
q303ea *(required)*	q303ea. Obstetrician/gynecologist<br>*Question relevant when: ${q301a} >0*<br>*Response constrained to: (.>=0 and .<=10) or .=99*	
q303eb *(required)*	q303eb. Anesthetist<br>*Question relevant when: ${q301b} >0*<br>*Response constrained to: (.>=0 and .<=10) or .=99*	
q303ec *(required)*	q303ec. Doctor/medical officer<br>*Question relevant when: ${q301c} >0*<br>*Response constrained to: (.>=0 and .<=10) or .=99*	
q303ed *(required)*	q303ed. Assistant medical officer<br>*Question relevant when: ${q301d} >0*<br>*Response constrained to: (.>=0 and .<=10) or .=99*	
q303ee *(required)*	q303ee. Clinical officer<br>*Question relevant when: ${q301e} >0*	

Field	Question	Answer
	Response constrained to: (.>=0 and .<=10) or .=99	
q303ef *(required)*	q303ef. Assistant clinical officer<br>*Question relevant when: ${q301f} >0*<br>*Response constrained to: (.>=0 and .<=10) or .=99*	
q303eg *(required)*	q303eg. Nurse/Nursing officer/Assistant nursing officer<br>*Question relevant when: ${q301g} >0*<br>*Response constrained to: (.>=0 and .<=10) or .=99*	
q303eh *(required)*	q303eh. Midwife/Enrolled nurse<br>*Question relevant when: ${q301h} >0*<br>*Response constrained to: (.>=0 and .<=10) or .=99*	
q303ei *(required)*	q303ei. Nursing Assistant<br>*Question relevant when: ${q301i} >0*<br>*Response constrained to: (.>=0 and .<=10) or .=99*	
q303ej *(required)*	q303ej. Lab Technician<br>*Question relevant when: ${q301j} >0*<br>*Response constrained to: (.>=0 and .<=10) or .=99*	
q303ek *(required)*	q303ek. Sonographer<br>*Question relevant when: ${q301k} >0*<br>*Response constrained to: (.>=0 and .<=10) or .=99*	
q303el *(required)*	q303el. Pharmacist<br>*Question relevant when: ${q301l} >0*<br>*Response constrained to: (.>=0 and .<=10) or .=99*	
q303em *(required)*	q303em. Pharmacy Assistant<br>*Question relevant when: ${q301m} >0*<br>*Response constrained to: (.>=0 and .<=10) or .=99*	
q303en *(required)*	q303en. Drug dispenser<br>*Question relevant when: ${q301n} >0*<br>*Response constrained to: (.>=0 and .<=10) or .=99*	
q303eo *(required)*	q303eo. Counselor<br>*Question relevant when: ${q301o} >0*<br>*Response constrained to: (.>=0 and .<=10) or .=99*	
group_q304a_outmin_inc_ab<br>*Group relevant when: selected(${section_three_skip} , '2')*		
generated_note_name_381	304a. For women who are managed for incomplete abortion on an outpatient basis, can you estimate how many minutes might be spent by a [LIST EACH HEALTH CARE WORKER]?<br>*Enter 99 if amount unknown.*	
q304aa *(required)*	q304aa. Obstetrician/gynecologist<br>*Question relevant when: ${q301a} >0 and ${q302aa} >0*<br>*Response constrained to: .>=0*	
q304ab *(required)*	q304ab. Anesthetist<br>*Question relevant when: ${q301b} >0 and ${q302ab} >0*<br>*Response constrained to: .>=0*	
q304ac *(required)*	q304ac. Doctor/medical officer<br>*Question relevant when: ${q301c} >0 and ${q302ac} >0*<br>*Response constrained to: .>=0*	
q304ad *(required)*	q304ad. Assistant medical officer<br>*Question relevant when: ${q301d} >0 and ${q302ad} >0*<br>*Response constrained to: .>=0*	
q304ae *(required)*	q304ae. Clinical officer<br>*Question relevant when: ${q301e} >0 and ${q302ae} >0*<br>*Response constrained to: .>=0*	
q304af *(required)*	q304af. Assistant clinical officer<br>*Question relevant when: ${q301f} >0 and ${q302af} >0*<br>*Response constrained to: .>=0*	
q304ag *(required)*	q304ag. Nurse/Nursing officer/Assistant nursing officer<br>*Question relevant when: ${q301g} >0 and ${q302ag} >0*<br>*Response constrained to: .>=0*	
q304ah *(required)*	q304ah. Midwife/Enrolled nurse<br>*Question relevant when: ${q301h} >0 and ${q302ah} >0*<br>*Response constrained to: .>=0*	
q304ai *(required)*	q304ai. Nursing Assistant<br>*Question relevant when: ${q301i} >0 and ${q302ai} >0*	

Field	Question	Answer
	Response constrained to: .>=0	
q304aj *(required)*	q304aj. Lab Technician<br>*Question relevant when: ${q301j} >0 and ${q302aj} >0*<br>*Response constrained to: .>=0*	
q304ak *(required)*	q304ak. Sonographer<br>*Question relevant when: ${q301k} >0 and ${q302ak} >0*<br>*Response constrained to: .>=0*	
q304al *(required)*	q304al. Pharmacist<br>*Question relevant when: ${q301l} >0 and ${q302al} >0*<br>*Response constrained to: .>=0*	
q304am *(required)*	q304am. Pharmacy Assistant<br>*Question relevant when: ${q301m} >0 and ${q302am} >0*<br>*Response constrained to: .>=0*	
q304an *(required)*	q304an. Drug dispenser<br>*Question relevant when: ${q301n} >0 and ${q302an} >0*<br>*Response constrained to: .>=0*	
q304ao *(required)*	q304ao. Counselor<br>*Question relevant when: ${q301o} >0 and ${q302ao} >0*<br>*Response constrained to: .>=0*	
group_q304b_outmin_shock<br>*Group relevant when: selected(${section_three_skip} , '2')*		
generated_note_name_399	304b. For women who are managed for sepsis on an outpatient basis, can you estimate how many minutes might be spent by a [LIST EACH HEALTH CARE WORKER]?<br>*Enter 99 if amount unknown.*	
q304ba *(required)*	q304ba. Obstetrician/gynecologist<br>*Question relevant when: ${q301a} >0 and ${q302ba} >0*<br>*Response constrained to: .>=0*	
q304bb *(required)*	q304bb. Anesthetist<br>*Question relevant when: ${q301b} >0 and ${q302bb} >0*<br>*Response constrained to: .>=0*	
q304bc *(required)*	q304bc. Doctor/medical officer<br>*Question relevant when: ${q301c} >0 and ${q302bc} >0*<br>*Response constrained to: .>=0*	
q304bd *(required)*	q304bd. Assistant medical officer<br>*Question relevant when: ${q301d} >0 and ${q302bd} >0*<br>*Response constrained to: .>=0*	
q304be *(required)*	q304be. Clinical officer<br>*Question relevant when: ${q301e} >0 and ${q302be} >0*<br>*Response constrained to: .>=0*	
q304bf *(required)*	q304bf. Assistant clinical officer<br>*Question relevant when: ${q301f} >0 and ${q302bf} >0*<br>*Response constrained to: .>=0*	
q304bg *(required)*	q304bg. Nurse/Nursing officer/Assistant nursing officer<br>*Question relevant when: ${q301g} >0 and ${q302bg} >0*<br>*Response constrained to: .>=0*	
q304bh *(required)*	q304bh. Midwife/Enrolled nurse<br>*Question relevant when: ${q301h} >0 and ${q302bh} >0*<br>*Response constrained to: .>=0*	
q304bi *(required)*	q304bi. Nursing Assistant<br>*Question relevant when: ${q301i} >0 and ${q302bi} >0*<br>*Response constrained to: .>=0*	
q304bj *(required)*	q304bj. Lab Technician<br>*Question relevant when: ${q301j} >0 and ${q302bj} >0*<br>*Response constrained to: .>=0*	
q304bk *(required)*	q304bk. Sonographer<br>*Question relevant when: ${q301k} >0 and ${q302bk} >0*<br>*Response constrained to: .>=0*	
q304bl *(required)*	q304bl. Pharmacist<br>*Question relevant when: ${q301l} >0 and ${q302bl} >0*<br>*Response constrained to: .>=0*	
q304bm *(required)*	q304bm. Pharmacy Assistant<br>*Question relevant when: ${q301m} >0 and ${q302bm} >0*	

Field	Question	Answer
	Response constrained to: .>=0	
q304bn *(required)*	q304bn. Drug dispenser<br>*Question relevant when: ${q301n} >0 and ${q302bn} >0*<br>*Response constrained to: .>=0*	

q304bo *(required)*	q304bo. Counselor<br>*Question relevant when: ${q301o} >0 and ${q302bo} >0*<br>*Response constrained to: .>=0*	
group_q304c_outmin_sepsis<br>*Group relevant when: selected(${section_three_skip} , '2')*		
generated_note_name_417	304c. For women who are managed for shock on an outpatient basis, can you estimate how many minutes might be spent by a [LIST EACH HEALTH CARE WORKER]?<br>*Enter 99 if amount unknown.*	
q304ca *(required)*	q304ca. Obstetrician/gynecologist<br>*Question relevant when: ${q301a} >0 and ${q302ca} >0*<br>*Response constrained to: .>=0*	
q304cb *(required)*	q304cb. Anesthetist<br>*Question relevant when: ${q301b} >0 and ${q302cb} >0*<br>*Response constrained to: .>=0*	
q304cc *(required)*	q304cc. Doctor/medical officer<br>*Question relevant when: ${q301c} >0 and ${q302cc} >0*<br>*Response constrained to: .>=0*	
q304cd *(required)*	q304cd. Assistant medical officer<br>*Question relevant when: ${q301d} >0 and ${q302cd} >0*<br>*Response constrained to: .>=0*	
q304ce *(required)*	q304ce. Clinical officer<br>*Question relevant when: ${q301e} >0 and ${q302ce} >0*<br>*Response constrained to: .>=0*	
q304cf *(required)*	q304cf. Assistant clinical officer<br>*Question relevant when: ${q301f} >0 and ${q302cf} >0*<br>*Response constrained to: .>=0*	
q304cg *(required)*	q304cg. Nurse/Nursing officer/Assistant nursing officer<br>*Question relevant when: ${q301g} >0 and ${q302cg} >0*<br>*Response constrained to: .>=0*	
q304ch *(required)*	q304ch. Midwife/Enrolled nurse<br>*Question relevant when: ${q301h} >0 and ${q302ch} >0*<br>*Response constrained to: .>=0*	
q304ci *(required)*	q304ci. Nursing Assistant<br>*Question relevant when: ${q301i} >0 and ${q302ci} >0*<br>*Response constrained to: .>=0*	
q304cj *(required)*	q304cj. Lab Technician<br>*Question relevant when: ${q301j} >0 and ${q302cj} >0*<br>*Response constrained to: .>=0*	
q304ck *(required)*	q304ck. Sonographer<br>*Question relevant when: ${q301k} >0 and ${q302ck} >0*<br>*Response constrained to: .>=0*	
q304cl *(required)*	q304cl. Pharmacist<br>*Question relevant when: ${q301l} >0 and ${q302cl} >0*<br>*Response constrained to: .>=0*	
q304cm *(required)*	q304cm. Pharmacy Assistant<br>*Question relevant when: ${q301m} >0 and ${q302cm} >0*<br>*Response constrained to: .>=0*	
q304cn *(required)*	q304cn. Drug dispenser<br>*Question relevant when: ${q301n} >0 and ${q302cn} >0*<br>*Response constrained to: .>=0*	
q304co *(required)*	q304co. Counselor<br>*Question relevant when: ${q301o} >0 and ${q302co} >0*<br>*Response constrained to: .>=0*	
group_q304d_outmin_cerv_lac<br>*Group relevant when: selected(${section_three_skip} , '2')*		
generated_note_name_435	304d. For women who are managed for cervical/vaginal lacerations on an outpatient basis, can you estimate how many minutes might be spent by a [LIST EACH HEALTH CARE WORKER]?<br>*Enter 99 if amount unknown.*	

Field	Question	Answer
q304da *(required)*	q304da. Obstetrician/gynecologist<br>*Question relevant when: ${q301a} >0 and ${q302da} >0*<br>*Response constrained to: .>=0*	
q304db *(required)*	q304db. Anesthetist<br>*Question relevant when: ${q301b} >0 and ${q302db} >0*<br>*Response constrained to: .>=0*	
q304dc *(required)*	q304dc. Doctor/medical officer<br>*Question relevant when: ${q301c} >0 and ${q302dc} >0*<br>*Response constrained to: .>=0*	
q304dd *(required)*	q304dd. Assistant medical officer<br>*Question relevant when: ${q301d} >0 and ${q302dd} >0*<br>*Response constrained to: .>=0*	
q304de *(required)*	q304de. Clinical officer<br>*Question relevant when: ${q301e} >0 and ${q302de} >0*<br>*Response constrained to: .>=0*	
q304df *(required)*	q304df. Assistant clinical officer<br>*Question relevant when: ${q301f} >0 and ${q302df} >0*<br>*Response constrained to: .>=0*	
q304dg *(required)*	q304dg. Nurse/Nursing officer/Assistant nursing officer<br>*Question relevant when: ${q301g} >0 and ${q302dg} >0*<br>*Response constrained to: .>=0*	
q304dh *(required)*	q304dh. Midwife/Enrolled nurse<br>*Question relevant when: ${q301h} >0 and ${q302dh} >0*<br>*Response constrained to: .>=0*	
q304di *(required)*	q304di. Nursing Assistant<br>*Question relevant when: ${q301i} >0 and ${q302di} >0*<br>*Response constrained to: .>=0*	
q304dj *(required)*	q304dj. Lab Technician<br>*Question relevant when: ${q301j} >0 and ${q302dj} >0*<br>*Response constrained to: .>=0*	
q304dk *(required)*	q304dk. Sonographer<br>*Question relevant when: ${q301k} >0 and ${q302dk} >0*<br>*Response constrained to: .>=0*	
q304dl *(required)*	q304dl. Pharmacist<br>*Question relevant when: ${q301l} >0 and ${q302dl} >0*<br>*Response constrained to: .>=0*	
q304dm *(required)*	q304dm. Pharmacy Assistant<br>*Question relevant when: ${q301m} >0 and ${q302dm} >0*<br>*Response constrained to: .>=0*	
q304dn *(required)*	q304dn. Drug dispenser<br>*Question relevant when: ${q301n} >0 and ${q302dn} >0*<br>*Response constrained to: .>=0*	
q304do *(required)*	q304do. Counselor<br>*Question relevant when: ${q301o} >0 and ${q302do} >0*<br>*Response constrained to: .>=0*	
group_q304e_outmin_uter_perf<br>*Group relevant when: selected(${section_three_skip} , '2')*		
generated_note_name_453	304e. For women who are managed for vaginal or uterine perforations on an outpatient basis, can you estimate how many minutes might be spent by a [LIST EACH HEALTH CARE WORKER]?<br>*Enter 99 if amount unknown.*	
q304ea *(required)*	q304ea. Obstetrician/gynecologist<br>*Question relevant when: ${q301a} >0 and ${q302ea} >0*<br>*Response constrained to: .>=0*	
q304eb *(required)*	q304eb. Anesthetist<br>*Question relevant when: ${q301b} >0 and ${q302eb} >0*<br>*Response constrained to: .>=0*	
q304ec *(required)*	q304ec. Doctor/medical officer<br>*Question relevant when: ${q301c} >0 and ${q302ec} >0*<br>*Response constrained to: .>=0*	
q304ed *(required)*	q304ed. Assistant medical officer<br>*Question relevant when: ${q301d} >0 and ${q302ed} >0*<br>*Response constrained to: .>=0*	
	q304ee. Clinical officer<br>*Question relevant when: ${q301e} >0 and ${q302ee} >0*	

Field	Question	Answer
q304ee *(required)*	*Response constrained to: .>=0*	
q304ef *(required)*	q304ef. Assistant clinical officer<br>*Question relevant when: ${q301f} >0 and ${q302ef} >0*<br>*Response constrained to: .>=0*	
q304eg *(required)*	q304eg. Nurse/Nursing officer/Assistant nursing officer<br>*Question relevant when: ${q301g} >0 and ${q302eg} >0*<br>*Response constrained to: .>=0*	
q304eh *(required)*	q304eh. Midwife/Enrolled nurse<br>*Question relevant when: ${q301h} >0 and ${q302eh} >0*<br>*Response constrained to: .>=0*	
q304ei *(required)*	q304ei. Nursing Assistant<br>*Question relevant when: ${q301i} >0 and ${q302ei} >0*<br>*Response constrained to: .>=0*	
q304ej *(required)*	q304ej. Lab Technician<br>*Question relevant when: ${q301j} >0 and ${q302ej} >0*<br>*Response constrained to: .>=0*	
q304ek *(required)*	q304ek. Sonographer<br>*Question relevant when: ${q301k} >0 and ${q302ek} >0*<br>*Response constrained to: .>=0*	
q304el *(required)*	q304el. Pharmacist<br>*Question relevant when: ${q301l} >0 and ${q302el} >0*<br>*Response constrained to: .>=0*	
q304em *(required)*	q304em. Pharmacy Assistant<br>*Question relevant when: ${q301m} >0 and ${q302em} >0*<br>*Response constrained to: .>=0*	
q304en *(required)*	q304en. Drug dispenser<br>*Question relevant when: ${q301n} >0 and ${q302en} >0*<br>*Response constrained to: .>=0*	
q304eo *(required)*	q304eo. Counselor<br>*Question relevant when: ${q301o} >0 and ${q302eo} >0*<br>*Response constrained to: .>=0*	
group_q305a_inmin_inc_ab<br>*Group relevant when: (selected(${section_three_skip} , '2')) and (selected(${q103a} , '1'))*		
generated_note_name_471	305a. For women who are managed for incomplete abortion on an inpatient basis, can you estimate how many minutes might be spent by a [LIST EACH HEALTH CARE WORKER]?<br>*Enter 99 if amount unknown.*	
q305aa *(required)*	q305aa. Obstetrician/gynecologist<br>*Question relevant when: ${q301a} >0 and ${q303aa} >0*<br>*Response constrained to: .>=0*	
q305ab *(required)*	q305ab. Anesthetist<br>*Question relevant when: ${q301b} >0 and ${q303ab} >0*<br>*Response constrained to: .>=0*	
q305ac *(required)*	q305ac. Doctor/medical officer<br>*Question relevant when: ${q301c} >0 and ${q303ac} >0*<br>*Response constrained to: .>=0*	
q305ad *(required)*	q305ad. Assistant medical officer<br>*Question relevant when: ${q301d} >0 and ${q303ad} >0*<br>*Response constrained to: .>=0*	
q305ae *(required)*	q305ae. Clinical officer<br>*Question relevant when: ${q301e} >0 and ${q303ae} >0*<br>*Response constrained to: .>=0*	
q305af *(required)*	q305af. Assistant clinical officer<br>*Question relevant when: ${q301f} >0 and ${q303af} >0*<br>*Response constrained to: .>=0*	
q305ag *(required)*	q305ag. Nurse/Nursing officer/Assistant nursing officer<br>*Question relevant when: ${q301g} >0 and ${q303ag} >0*<br>*Response constrained to: .>=0*	
q305ah *(required)*	q305ah. Midwife/Enrolled nurse<br>*Question relevant when: ${q301h} >0 and ${q303ah} >0*<br>*Response constrained to: .>=0*	
q305ai *(required)*	q305ai. Nursing Assistant<br>*Question relevant when: ${q301i} >0 and ${q303ai} >0*	

Field	Question	Answer
	Response constrained to: .>=0	
q305aj *(required)*	q305aj. Lab Technician<br>*Question relevant when: ${q301j} >0 and ${q303aj} >0*<br>*Response constrained to: .>=0*	
q305ak *(required)*	q305ak. Sonographer<br>*Question relevant when: ${q301k} >0 and ${q303ak} >0*<br>*Response constrained to: .>=0*	
q305al *(required)*	q305al. Pharmacist<br>*Question relevant when: ${q301l} >0 and ${q303al} >0*<br>*Response constrained to: .>=0*	
q305am *(required)*	q305am. Pharmacy Assistant<br>*Question relevant when: ${q301m} >0 and ${q303am} >0*<br>*Response constrained to: .>=0*	
q305an *(required)*	q305an. Drug dispenser<br>*Question relevant when: ${q301n} >0 and ${q303an} >0*<br>*Response constrained to: .>=0*	
q305ao *(required)*	q305ao. Counselor<br>*Question relevant when: ${q301o} >0 and ${q303ao} >0*<br>*Response constrained to: .>=0*	
group_q305b_inmin_shock<br>*Group relevant when: (selected(${section_three_skip} , '2')) and (selected(${q103a} , '1'))*		
generated_note_name_489	305b. For women who are managed for sepsis on an inpatient basis, can you estimate how many minutes might be spent by a [LIST EACH HEALTH CARE WORKER]?<br>*Enter 99 if amount unknown.*	
q305ba *(required)*	q305ba. Obstetrician/gynecologist<br>*Question relevant when: ${q301a} >0 and ${q303ba} >0*<br>*Response constrained to: .>=0*	
q305bb *(required)*	q305bb. Anesthetist<br>*Question relevant when: ${q301b} >0 and ${q303bb} >0*<br>*Response constrained to: .>=0*	
q305bc *(required)*	q305bc. Doctor/medical officer<br>*Question relevant when: ${q301c} >0 and ${q303bc} >0*<br>*Response constrained to: .>=0*	
q305bd *(required)*	q305bd. Assistant medical officer<br>*Question relevant when: ${q301d} >0 and ${q303bd} >0*<br>*Response constrained to: .>=0*	
q305be *(required)*	q305be. Clinical officer<br>*Question relevant when: ${q301e} >0 and ${q303be} >0*<br>*Response constrained to: .>=0*	
q305bf *(required)*	q305bf. Assistant clinical officer<br>*Question relevant when: ${q301f} >0 and ${q303bf} >0*<br>*Response constrained to: .>=0*	
q305bg *(required)*	q305bg. Nurse/Nursing officer/Assistant nursing officer<br>*Question relevant when: ${q301g} >0 and ${q303bg} >0*<br>*Response constrained to: .>=0*	
q305bh *(required)*	q305bh. Midwife/Enrolled nurse<br>*Question relevant when: ${q301h} >0 and ${q303bh} >0*<br>*Response constrained to: .>=0*	
q305bi *(required)*	q305bi. Nursing Assistant<br>*Question relevant when: ${q301i} >0 and ${q303bi} >0*<br>*Response constrained to: .>=0*	
q305bj *(required)*	q305bj. Lab Technician<br>*Question relevant when: ${q301j} >0 and ${q303bj} >0*<br>*Response constrained to: .>=0*	
q305bk *(required)*	q305bk. Sonographer<br>*Question relevant when: ${q301k} >0 and ${q303bk} >0*<br>*Response constrained to: .>=0*	
q305bl *(required)*	q305bl. Pharmacist<br>*Question relevant when: ${q301l} >0 and ${q303bl} >0*<br>*Response constrained to: .>=0*	
q305bm *(required)*	q305bm. Pharmacy Assistant<br>*Question relevant when: ${q301m} >0 and ${q303bm} >0*	

Field	Question	Answer
	Response constrained to: .>=0	
q305bn *(required)*	q305bn. Drug dispenser<br>*Question relevant when: ${q301n} >0 and ${q303bn} >0*<br>*Response constrained to: .>=0*	

Field	Question	Answer
q305bo *(required)*	q305bo. Counselor<br>*Question relevant when: ${q301o} >0 and ${q303bo} >0*<br>*Response constrained to: .>=0*	
group_q305c_inmin_sepsis<br>*Group relevant when: (selected(${section_three_skip} , '2')) and (selected(${q103a} , '1'))*		
generated_note_name_507	305c. For women who are managed for shock on an inpatient basis, can you estimate how many minutes might be spent by a [LIST EACH HEALTH CARE WORKER]?<br>*Enter 99 if amount unknown.*	
q305ca *(required)*	q305ca. Obstetrician/gynecologist<br>*Question relevant when: ${q301a} >0 and ${q303ca} >0*<br>*Response constrained to: .>=0*	
q305cb *(required)*	q305cb. Anesthetist<br>*Question relevant when: ${q301b} >0 and ${q303cb} >0*<br>*Response constrained to: .>=0*	
q305cc *(required)*	q305cc. Doctor/medical officer<br>*Question relevant when: ${q301c} >0 and ${q303cc} >0*<br>*Response constrained to: .>=0*	
q305cd *(required)*	q305cd. Assistant medical officer<br>*Question relevant when: ${q301d} >0 and ${q303cd} >0*<br>*Response constrained to: .>=0*	
q305ce *(required)*	q305ce. Clinical officer<br>*Question relevant when: ${q301e} >0 and ${q303ce} >0*<br>*Response constrained to: .>=0*	
q305cf *(required)*	q305cf. Assistant clinical officer<br>*Question relevant when: ${q301f} >0 and ${q303cf} >0*<br>*Response constrained to: .>=0*	
q305cg *(required)*	q305cg. Nurse/Nursing officer/Assistant nursing officer<br>*Question relevant when: ${q301g} >0 and ${q303cg} >0*<br>*Response constrained to: .>=0*	
q305ch *(required)*	q305ch. Midwife/Enrolled nurse<br>*Question relevant when: ${q301h} >0 and ${q303ch} >0*<br>*Response constrained to: .>=0*	
q305ci *(required)*	q305ci. Nursing Assistant<br>*Question relevant when: ${q301i} >0 and ${q303ci} >0*<br>*Response constrained to: .>=0*	
q305cj *(required)*	q305cj. Lab Technician<br>*Question relevant when: ${q301j} >0 and ${q303cj} >0*<br>*Response constrained to: .>=0*	
q305ck *(required)*	q305ck. Sonographer<br>*Question relevant when: ${q301k} >0 and ${q303ck} >0*<br>*Response constrained to: .>=0*	
q305cl *(required)*	q305cl. Pharmacist<br>*Question relevant when: ${q301l} >0 and ${q303cl} >0*<br>*Response constrained to: .>=0*	
q305cm *(required)*	q305cm. Pharmacy Assistant<br>*Question relevant when: ${q301m} >0 and ${q303cm} >0*<br>*Response constrained to: .>=0*	
q305cn *(required)*	q305cn. Drug dispenser<br>*Question relevant when: ${q301n} >0 and ${q303cn} >0*<br>*Response constrained to: .>=0*	
q305co *(required)*	q305co. Counselor<br>*Question relevant when: ${q301o} >0 and ${q303co} >0*<br>*Response constrained to: .>=0*	
group_q305d_inmin_cerv_lac<br>*Group relevant when: (selected(${section_three_skip} , '2')) and (selected(${q103a} , '1'))*		
generated_note_name_525	305d. For women who are managed for cervical/vaginal lacerations on an inpatient basis, can you estimate how many minutes might be spent by a [LIST EACH HEALTH CARE WORKER]?<br>[illegible]	

Enter 99 if amount unknown.

Field	Question	Answer
q305da *(required)*	q305da. Obstetrician/gynecologist<br>*Question relevant when: ${q301a} >0 and ${q303da} >0*<br>*Response constrained to: .>=0*	
q305db *(required)*	q305db. Anesthetist<br>*Question relevant when: ${q301b} >0 and ${q303db} >0*<br>*Response constrained to: .>=0*	

q305dc *(required)*	q305dc. Doctor/medical officer<br>*Question relevant when: ${q301c} >0 and ${q303dc} >0*<br>*Response constrained to: .>=0*	
q305dd *(required)*	q305dd. Assistant medical officer<br>*Question relevant when: ${q301d} >0 and ${q303dd} >0*<br>*Response constrained to: .>=0*	
q305de *(required)*	q305de. Clinical officer<br>*Question relevant when: ${q301e} >0 and ${q303de} >0*<br>*Response constrained to: .>=0*	
q305df *(required)*	q305df. Assistant clinical officer<br>*Question relevant when: ${q301f} >0 and ${q303df} >0*<br>*Response constrained to: .>=0*	
q305dg *(required)*	q305dg. Nurse/Nursing officer/Assistant nursing officer<br>*Question relevant when: ${q301g} >0 and ${q303dg} >0*<br>*Response constrained to: .>=0*	
q305dh *(required)*	q305dh. Midwife/Enrolled nurse<br>*Question relevant when: ${q301h} >0 and ${q303dh} >0*<br>*Response constrained to: .>=0*	
q305di *(required)*	q305di. Nursing Assistant<br>*Question relevant when: ${q301i} >0 and ${q303di} >0*<br>*Response constrained to: .>=0*	
q305dj *(required)*	q305dj. Lab Technician<br>*Question relevant when: ${q301j} >0 and ${q303dj} >0*<br>*Response constrained to: .>=0*	
q305dk *(required)*	q305dk. Sonographer<br>*Question relevant when: ${q301k} >0 and ${q303dk} >0*<br>*Response constrained to: .>=0*	
q305dl *(required)*	q305dl. Pharmacist<br>*Question relevant when: ${q301l} >0 and ${q303dl} >0*<br>*Response constrained to: .>=0*	
q305dm *(required)*	q305dm. Pharmacy Assistant<br>*Question relevant when: ${q301m} >0 and ${q303dm} >0*<br>*Response constrained to: .>=0*	
q305dn *(required)*	q305dn. Drug dispenser<br>*Question relevant when: ${q301n} >0 and ${q303dn} >0*<br>*Response constrained to: .>=0*	
q305do *(required)*	q305do. Counselor<br>*Question relevant when: ${q301o} >0 and ${q303do} >0*<br>*Response constrained to: .>=0*	
group_q305e_inmin_uter_perf<br>*Group relevant when: (selected(${section_three_skip} , '2')) and (selected(${q103a} , '1'))*		
generated_note_name_543	305e. For women who are managed for vaginal or uterine perforations on an inpatient basis, can you estimate how many minutes might be spent by a [LIST EACH HEALTH CARE WORKER]?<br>*Enter 99 if amount unknown.*	
q305ea *(required)*	q305ea. Obstetrician/gynecologist<br>*Question relevant when: ${q301a} >0 and ${q303ea} >0*<br>*Response constrained to: .>=0*	
q305eb *(required)*	q305eb. Anesthetist<br>*Question relevant when: ${q301b} >0 and ${q303eb} >0*<br>*Response constrained to: .>=0*	
q305ec *(required)*	q305ec. Doctor/medical officer<br>*Question relevant when: ${q301c} >0 and ${q303ec} >0*<br>*Response constrained to: .>=0*	
q305ed *(required)*	q305ed. Assistant medical officer<br>*Question relevant when: ${q301d} >0 and ${q303ed} >0*<br>*Response constrained to: .>=0*	
q305ee *(required)*	q305ee. Clinical officer	

Field	Question	Answer
	Question relevant when: ${q301e} >0 and ${q303ee} >0<br>Response constrained to: .>=0	
q305ef (required)	q305ef. Assistant clinical officer<br>Question relevant when: ${q301f} >0 and ${q303ef} >0<br>Response constrained to: .>=0	
q305eg (required)	q305eg. Nurse/Nursing officer/Assistant nursing officer<br>Question relevant when: ${q301g} >0 and ${q303eg} >0<br>Response constrained to: .>=0	
q305eh (required)	q305eh. Midwife/Enrolled nurse<br>Question relevant when: ${q301h} >0 and ${q303eh} >0<br>Response constrained to: .>=0	
q305ei (required)	q305ei. Nursing Assistant<br>Question relevant when: ${q301i} >0 and ${q303ei} >0<br>Response constrained to: .>=0	
q305ej (required)	q305ej. Lab Technician<br>Question relevant when: ${q301j} >0 and ${q303ej} >0<br>Response constrained to: .>=0	
q305ek (required)	q305ek. Sonographer<br>Question relevant when: ${q301k} >0 and ${q303ek} >0<br>Response constrained to: .>=0	
q305el (required)	q305el. Pharmacist<br>Question relevant when: ${q301l} >0 and ${q303el} >0<br>Response constrained to: .>=0	
q305em (required)	q305em. Pharmacy Assistant<br>Question relevant when: ${q301m} >0 and ${q303em} >0<br>Response constrained to: .>=0	
q305en (required)	q305en. Drug dispenser<br>Question relevant when: ${q301n} >0 and ${q303en} >0<br>Response constrained to: .>=0	
q305eo (required)	q305eo. Counselor<br>Question relevant when: ${q301o} >0 and ${q303eo} >0<br>Response constrained to: .>=0	
group_section_four_start		
section4_start	**SECTION IV. STAFFING, STAFF TIME AND COSTS**	
section_four_skip	INTERVIEWER: WOULD YOU LIKE TO COMPLETE THIS SECTION NOW OR SKIP THIS SECTION AND RETURN TO IT LATER?<br>*You may need to skip if the participant has indicated that s/he cannot answer the questions in this section.*	1 Skip and come back to this section later.<br>2 Do not skip, complete this section now.
qintro_401<br>*Group relevant when: (selected(${section_four_skip} , '2'))*		
generated_note_name_565	READ ALOUD: Many countries have laws requiring a minimum standard for working days and leave days per year. Based on your knowledge of your facility's rules or, if there is no set rule, the basic laws in your country, can you please answer the following:	
q401 (required)	401. Can you tell me how many hours per week are staff employed full time by this facility expected to work, excluding any breaks?<br>*Enter 999 if amount unknown.*<br>Response constrained to: (.>=0 and .<=168) or .=999	
group_q402.1_hrs_per_wk_staff<br>*Group relevant when: (selected(${section_four_skip} , '2'))*		
generated_note_name_569	402a-o1. At your facility, can you estimate the number of hours that the following staff members normally work per week?<br>*Enter 999 if amount unknown.*	
q402a1 (required)	q402a1. Obstetrician/gynecologist<br>Question relevant when: ${q301a} >0<br>Response constrained to: (.>0 and .<=168) or .=999	
q402b1 (required)	q402b1. Anesthetist<br>Question relevant when: ${q301b} >0<br>Response constrained to: (.>0 and .<=168) or .=999	
q402c1 (required)	q402c1. Doctor/medical officer<br>Question relevant when: ${q301c} >0<br>Response constrained to: (.>0 and .<=168) or .=999	

Field	Question	Answer
q402d1 *(required)*	q402d1. Assistant medical officer<br>*Question relevant when: ${q301d} >0*<br>*Response constrained to: (.>0 and .<=168) or .=999*	
q402e1 *(required)*	q402e1. Clinical officer<br>*Question relevant when: ${q301e} >0*<br>*Response constrained to: (.>0 and .<=168) or .=999*	
q402f1 *(required)*	q402f1. Assistant clinical officer<br>*Question relevant when: ${q301f} >0*<br>*Response constrained to: (.>0 and .<=168) or .=999*	
q402g1 *(required)*	q402g1. Nurse/Nursing officer/Assistant nursing officer<br>*Question relevant when: ${q301g} >0*<br>*Response constrained to: (.>0 and .<=168) or .=999*	
q402h1 *(required)*	q402h1. Midwife/Enrolled nurse<br>*Question relevant when: ${q301h} >0*<br>*Response constrained to: (.>0 and .<=168) or .=999*	
q402i1 *(required)*	q402i1. Nursing Assistant<br>*Question relevant when: ${q301i} >0*<br>*Response constrained to: (.>0 and .<=168) or .=999*	
q402j1 *(required)*	q402j1. Lab Technician<br>*Question relevant when: ${q301j} >0*<br>*Response constrained to: (.>0 and .<=168) or .=999*	
q402k1 *(required)*	q402k1. Sonographer<br>*Question relevant when: ${q301k} >0*<br>*Response constrained to: (.>0 and .<=168) or .=999*	
q402l1 *(required)*	q402l1. Pharmacist<br>*Question relevant when: ${q301l} >0*<br>*Response constrained to: (.>0 and .<=168) or .=999*	
q402m1 *(required)*	q402m1. Pharmacy Assistant<br>*Question relevant when: ${q301m} >0*<br>*Response constrained to: (.>0 and .<=168) or .=999*	
q402n1 *(required)*	q402n1. Drug dispenser<br>*Question relevant when: ${q301n} >0*<br>*Response constrained to: (.>0 and .<=168) or .=999*	
q402o1 *(required)*	q402o1. Counselor<br>*Question relevant when: ${q301o} >0*<br>*Response constrained to: (.>0 and .<=168) or .=999*	
group_q402.2_wks_per_yr_staff<br>*Group relevant when: (selected(${section_four_skip} , '2'))*		
generated_note_name_587	402a-o2. At your facility, can you estimate the number of weeks that the following staff members normally work per year?<br>*Enter 999 if amount unknown.*	
q402a2 *(required)*	q402a2. Obstetrician/gynecologist<br>*Question relevant when: ${q301a} >0*<br>*Response constrained to: (.>0 and .<=52) or .=999*	
q402b2 *(required)*	q402b2. Anesthetist<br>*Question relevant when: ${q301b} >0*<br>*Response constrained to: (.>0 and .<=52) or .=999*	
q402c2 *(required)*	q402c2. Doctor/medical officer<br>*Question relevant when: ${q301c} >0*<br>*Response constrained to: (.>0 and .<=52) or .=999*	
q402d2 *(required)*	q402d2. Assistant medical officer<br>*Question relevant when: ${q301d} >0*<br>*Response constrained to: (.>0 and .<=52) or .=999*	
q402e2 *(required)*	q402e2. Clinical officer<br>*Question relevant when: ${q301e} >0*<br>*Response constrained to: (.>0 and .<=52) or .=999*	
q402f2 *(required)*	q402f2. Assistant clinical officer<br>*Question relevant when: ${q301f} >0*<br>*Response constrained to: (.>0 and .<=52) or .=999*	
q402g2 *(required)*	q402g2. Nurse/Nursing officer/Assistant nursing officer<br>*Question relevant when: ${q301g} >0*<br>*Response constrained to: (.>0 and .<=52) or .=999*	
q402h2 *(required)*	q402h2. Midwife/Enrolled nurse<br>*Question relevant when: ${q301h} >0*<br>*Response constrained to: (.>0 and .<=52) or .=999*	

Field	Question	Answer
q402i2 *(required)*	q402i2. Nursing Assistant<br>*Question relevant when: ${q301i} >0*<br>*Response constrained to: (.>0 and .<=52) or .=999*	
q402j2 *(required)*	q402j2. Lab Technician<br>*Question relevant when: ${q301j} >0*<br>*Response constrained to: (.>0 and .<=52) or .=999*	
q402k2 *(required)*	q402k2. Sonographer<br>*Question relevant when: ${q301k} >0*<br>*Response constrained to: (.>0 and .<=52) or .=999*	
q402l2 *(required)*	q402l2. Pharmacist<br>*Question relevant when: ${q301l} >0*<br>*Response constrained to: (.>0 and .<=52) or .=999*	
q402m2 *(required)*	q402m2. Pharmacy Assistant<br>*Question relevant when: ${q301m} >0*<br>*Response constrained to: (.>0 and .<=52) or .=999*	
q402n2 *(required)*	q402n2. Drug dispenser<br>*Question relevant when: ${q301n} >0*<br>*Response constrained to: (.>0 and .<=52) or .=999*	
q402o2 *(required)*	q402o2. Counselor<br>*Question relevant when: ${q301o} >0*<br>*Response constrained to: (.>0 and .<=52) or .=999*	
group_q403_admin_time<br>*Group relevant when: (selected(${section_four_skip} , '2'))*		
generated_note_name_605	403. In addition to attending to patients, most medical workers have other duties. Can you estimate the number of work hours per week that each type of medical worker spends on non-patient activities? (For example, completing forms, attending meetings, training, etc.)<br>*Enter 999 if amount unknown.*	
q403a *(required)*	q403a. Obstetrician/gynecologist<br>*Question relevant when: ${q301a} >0*<br>*Response constrained to: (.>=0 and .<=168) or .=999*	
q403b *(required)*	q403b. Anesthetist<br>*Question relevant when: ${q301b} >0*<br>*Response constrained to: (.>=0 and .<=168) or .=999*	
q403c *(required)*	q403c. Doctor/medical officer<br>*Question relevant when: ${q301c} >0*<br>*Response constrained to: (.>=0 and .<=168) or .=999*	
q403d *(required)*	q403d. Assistant medical officer<br>*Question relevant when: ${q301d} >0*<br>*Response constrained to: (.>=0 and .<=168) or .=999*	
q403e *(required)*	q403e. Clinical officer<br>*Question relevant when: ${q301e} >0*<br>*Response constrained to: (.>=0 and .<=168) or .=999*	
q403f *(required)*	q403f. Assistant clinical officer<br>*Question relevant when: ${q301f} >0*<br>*Response constrained to: (.>=0 and .<=168) or .=999*	
q403g *(required)*	q403g. Nurse/Nursing officer/Assistant nursing officer<br>*Question relevant when: ${q301g} >0*<br>*Response constrained to: (.>=0 and .<=168) or .=999*	
q403h *(required)*	q403h. Midwife/Enrolled nurse<br>*Question relevant when: ${q301h} >0*<br>*Response constrained to: (.>=0 and .<=168) or .=999*	
q403i *(required)*	q403i. Nursing Assistant<br>*Question relevant when: ${q301i} >0*<br>*Response constrained to: (.>=0 and .<=168) or .=999*	
q403j *(required)*	q403j. Lab Technician<br>*Question relevant when: ${q301j} >0*<br>*Response constrained to: (.>=0 and .<=168) or .=999*	
q403k *(required)*	q403k. Sonographer<br>*Question relevant when: ${q301k} >0*<br>*Response constrained to: (.>=0 and .<=168) or .=999*	
q403l *(required)*	q403l. Pharmacist<br>*Question relevant when: ${q301l} >0*<br>*Response constrained to: (.>=0 and .<=168) or .=999*	
q403m *(required)*	q403m. Pharmacy Assistant	

Field	Question	Answer
	Question relevant when: ${q301m} >0<br>*Response constrained to: (.>=0 and .<=168) or .=999*	
q403n *(required)*	q403n. Drug dispenser<br>*Question relevant when: ${q301n} >0*<br>*Response constrained to: (.>=0 and .<=168) or .=999*	
q403o *(required)*	q403o. Counselor<br>*Question relevant when: ${q301o} >0*<br>*Response constrained to: (.>=0 and .<=168) or .=999*	

group_q404_remun<br>*Group relevant when: (selected(${section_four_skip} , '2'))*		
generated_note_name_623	404. Please estimate the average monthly remuneration package (in Shillings) for each category of medical worker at your facility. This should include the worker's salary plus any benefits (e.g. pension, insurance subsidies, housing subsidies, duty allowances, hardship allowances, etc.). Do not include the cost of annual leave, sick leave, etc. [READ ONE AT A TIME]<br>*Enter 99999 if amount unknown.*	
q404a *(required)*	q404a. Obstetrician/gynecologist<br>*Question relevant when: ${q301a} >0*<br>*Response constrained to: .>=0*	
q404b *(required)*	q404b. Anesthetist<br>*Question relevant when: ${q301b} >0*<br>*Response constrained to: .>=0*	
q404c *(required)*	q404c. Doctor/medical officer<br>*Question relevant when: ${q301c} >0*<br>*Response constrained to: .>=0*	
q404d *(required)*	q404d. Assistant medical officer<br>*Question relevant when: ${q301d} >0*<br>*Response constrained to: .>=0*	
q404e *(required)*	q404e. Clinical officer<br>*Question relevant when: ${q301e} >0*<br>*Response constrained to: .>=0*	
q404f *(required)*	q404f. Assistant clinical officer<br>*Question relevant when: ${q301f} >0*<br>*Response constrained to: .>=0*	
q404g *(required)*	q404g. Nurse/Nursing officer/Assistant nursing officer<br>*Question relevant when: ${q301g} >0*<br>*Response constrained to: .>=0*	
q404h *(required)*	q404h. Midwife/Enrolled nurse<br>*Question relevant when: ${q301h} >0*<br>*Response constrained to: .>=0*	
q404i *(required)*	q404i. Nursing Assistant<br>*Question relevant when: ${q301i} >0*<br>*Response constrained to: .>=0*	
q404j *(required)*	q404j. Lab Technician<br>*Question relevant when: ${q301j} >0*<br>*Response constrained to: .>=0*	
q404k *(required)*	q404k. Sonographer<br>*Question relevant when: ${q301k} >0*<br>*Response constrained to: .>=0*	
q404l *(required)*	q404l. Pharmacist<br>*Question relevant when: ${q301l} >0*<br>*Response constrained to: .>=0*	
q404m *(required)*	q404m. Pharmacy Assistant<br>*Question relevant when: ${q301m} >0*<br>*Response constrained to: .>=0*	
q404n *(required)*	q404n. Drug dispenser<br>*Question relevant when: ${q301n} >0*<br>*Response constrained to: .>=0*	
q404o *(required)*	q404o. Counselor<br>*Question relevant when: ${q301o} >0*<br>*Response constrained to: .>=0*	
groups_q405.1_no_aux_staff_FT<br>*Group relevant when: (selected(${section_four_skip} , '2'))*		
generated_note_name_641	405a-m1. Now I would like to know how many auxiliary staff are currently employed at your facility. How many of the	

Field	Question	Answer
	following staff types are employed FULL TIME at your facility?<br>*Enter 999 if amount unknown.*	
q405a1 *(required)*	q405a1. Security guards<br>*Response constrained to: .>=0*	
q405b1 *(required)*	q405b1. Cleaners<br>*Response constrained to: .>=0*	
q405c1 *(required)*	q405c1. Receptionists or administrative assistants<br>*Response constrained to: .>=0*	
q405d1 *(required)*	q405d1. Records managers or filing clerks<br>*Response constrained to: .>=0*	
q405e1 *(required)*	q405e1. Supply officers, procurement officers/staff<br>*Response constrained to: .>=0*	
q405f1 *(required)*	q405f1. Maintenance staff<br>*Response constrained to: .>=0*	
q405g1 *(required)*	q405g1. Drivers<br>*Response constrained to: .>=0*	
q405h1 *(required)*	q405h1. Food preparers/cooks<br>*Response constrained to: .>=0*	
q405i1 *(required)*	q405i1. Medical attendant / food servers / general hands<br>*Response constrained to: .>=0*	
q405j1 *(required)*	q405j1. Health officers<br>*Response constrained to: .>=0*	
q405k1 *(required)*	q405k1. Assistant health officers<br>*Response constrained to: .>=0*	
q405l1 *(required)*	q405l1. Community health workers<br>*Response constrained to: .>=0*	
q405m1 *(required)*	q405m1. Accountant<br>*Response constrained to: .>=0*	
q405n1 *(required)*	q405n1. Assistant accountant<br>*Response constrained to: .>=0*	
groups_q405.2_no_aux_staff_PT<br>*Group relevant when: (selected(${section_four_skip} , '2'))*		
generated_note_name_658	405a-m2. Now I would like to know how many auxiliary staff are currently employed at your facility. How many of the following staff types are employed PART TIME at your facility?<br>*Enter 999 if amount unknown.*	
q405a2 *(required)*	q405a2. Security guards<br>*Response constrained to: .>=0*	
q405b2 *(required)*	q405b2. Cleaners<br>*Response constrained to: .>=0*	
q405c2 *(required)*	q405c2. Receptionists or administrative assistants<br>*Response constrained to: .>=0*	
q405d2 *(required)*	q405d2. Records managers or filing clerks<br>*Response constrained to: .>=0*	
q405e2 *(required)*	q405e2. Supply officers, procurement officers/staff<br>*Response constrained to: .>=0*	
q405f2 *(required)*	q405f2. Maintenance staff<br>*Response constrained to: .>=0*	
q405g2 *(required)*	q405g2. Drivers<br>*Response constrained to: .>=0*	
q405h2 *(required)*	q405h2. Food preparers/cooks<br>*Response constrained to: .>=0*	
q405i2 *(required)*	q405i2. Medical attendant / food servers / general hands<br>*Response constrained to: .>=0*	
q405j2 *(required)*	q405j2. Health officers<br>*Response constrained to: .>=0*	
q405k2 *(required)*	q405k2. Assistant health officers<br>*Response constrained to: .>=0*	
q405l2 *(required)*	q405l2. Community health workers<br>*Response constrained to: .>=0*	
q405m2 *(required)*	q405m2. Accountant<br>*Response constrained to: .>=0*	
q405n2 *(required)*	q405n2. Assistant accountant<br>[illegible]	

Field	Question	Answer
	Response constrained to: .>=0	
Auxiliary staff remuneration<br>*Group relevant when: (selected(${section_four_skip} , '2'))*		
generated_note_name_675	406. Please estimate the average monthly remuneration package (in Shillings) for each category of auxiliary worker at your facility. Please consider a full time working schedule, and again, this should include the worker's salary plus any benefits (e.g. pension, insurance subsidies, housing subsidies, duty allowances, hardship allowances, etc.). Do not include the cost of annual leave, sick leave, etc.<br>*Enter 999 if amount unknown.*	

q406a *(required)*	q406a. Security guards<br>*Question relevant when: ${q405a1} > 0 or ${q405a2} > 0*<br>*Response constrained to: .>=0*	
q406b *(required)*	q406b. Cleaners<br>*Question relevant when: ${q405b1} > 0 or ${q405b2} > 0*<br>*Response constrained to: .>=0*	
q406c *(required)*	q406c. Receptionists or administrative assistants<br>*Question relevant when: ${q405c1} > 0 or ${q405c2} > 0*<br>*Response constrained to: .>=0*	
q406d *(required)*	q406d. Records managers or filing clerks<br>*Question relevant when: ${q405d1} > 0 or ${q405d2} > 0*<br>*Response constrained to: .>=0*	
q406e *(required)*	q406e. Supply officers, procurement officers/staff<br>*Question relevant when: ${q405e1} > 0 or ${q405e2} > 0*<br>*Response constrained to: .>=0*	
q406f *(required)*	q406f. Maintenance staff<br>*Question relevant when: ${q405f1} > 0 or ${q405f2} > 0*<br>*Response constrained to: .>=0*	
q406g *(required)*	q406g. Drivers<br>*Question relevant when: ${q405g1} > 0 or ${q405g2} > 0*<br>*Response constrained to: .>=0*	
q406h *(required)*	q406h. Food preparers/cooks<br>*Question relevant when: ${q405h1} > 0 or ${q405h2} > 0*<br>*Response constrained to: .>=0*	
q406i *(required)*	q406i. Medical attendant / food servers / general hands<br>*Question relevant when: ${q405i1} > 0 or ${q405i2} > 0*<br>*Response constrained to: .>=0*	
q406j *(required)*	q406j. Health officers<br>*Question relevant when: ${q405j1} > 0 or ${q405j2} > 0*<br>*Response constrained to: .>=0*	
q406k *(required)*	q406k. Assistant health officers<br>*Question relevant when: ${q405k1} > 0 or ${q405k2} > 0*<br>*Response constrained to: .>=0*	
q406l *(required)*	q406l. Community health workers<br>*Question relevant when: ${q405l1} > 0 or ${q405l2} > 0*<br>*Response constrained to: .>=0*	
q406m *(required)*	q406m. Accountant<br>*Question relevant when: ${q405m1} > 0 or ${q405m2} > 0*<br>*Response constrained to: .>=0*	
q406n *(required)*	q406n. Assistant accountant<br>*Question relevant when: ${q405n1} > 0 or ${q405n2} > 0*<br>*Response constrained to: .>=0*	
group_section_five_start		
section5_start	**SECTION V. BUILDING COSTS**	
section_five_skip	INTERVIEWER: WOULD YOU LIKE TO COMPLETE THIS SECTION NOW OR SKIP THIS SECTION AND RETURN TO IT LATER?<br>*You may need to skip if the participant has indicated that s/he cannot answer the questions in this section.*	1 Skip and come back to this section later.<br>2 Do not skip, complete this section now.
q501	501. Do you have any records or documents showing the cost of constructing and/or equipping this facility or a similar facility?<br>*Question relevant when: (selected(${section_five_skip} , '2'))*	1 Yes<br>0 No<br>99 Don't know
q501.1 *(required)*	501.1 Do the records show costs for constructing and equipping the facility combined, or are those costs shown separately?<br>*Question relevant when: (selected(${section_five_skip} , '2')) and (selected(${q501} , '1'))*	1 Records show construction and equipping facility costs separately

Field	Question	Answer
		2 Records show construction and equipping facility costs as one combined cost
FACILITY COSTS - FROM RECORDS *Group relevant when: (selected(${section_five_skip} , '2')) and (selected(${q501} , '1'))*		
note_records	INTERVIEWER: PLEASE USE FACILITY RECORDS TO ANSWER THE FOLLOWING (AND OBTAIN A COPY OF THE RECORD(S) IF POSSIBLE).<br>*Enter 99999 if unknown.*	
q502 *(required)*	502. Based on records, what is the cost of constructing this facility (or a similar facility)?<br>*Enter 99999 if unknown.*<br>*Question relevant when: (selected(${q501.1} , '1'))*<br>*Response constrained to: .>=0*	
q503 *(required)*	503. What is the year for which that cost estimate is based?<br>*Please enter a year in this format YYYY. Enter 2999 if unknown.*<br>*Question relevant when: (selected(${q501.1} , '1'))*<br>*Response constrained to: regex(., '[1-2][0-9][0-9][0-9]')*	
q504 *(required)*	504. Based on records, what is the cost of fully equipping this facility (or a similar facility)?<br>*Enter 99999 if unknown.*<br>*Question relevant when: (selected(${q501.1} , '1'))*<br>*Response constrained to: .>=0*	
q505 *(required)*	505. What is the year for which that cost estimate is based?<br>*Please enter a year in this format YYYY. Enter 2999 if unknown.*<br>*Question relevant when: (selected(${q501.1} , '1'))*<br>*Response constrained to: regex(., '[1-2][0-9][0-9][0-9]')*	
q506 *(required)*	506. If costs are provided in records for construction and equipping combined, what is the total cost of constructing and fully equipping this facility (or a similar facility)?<br>*Enter 99999 if unknown.*<br>*Question relevant when: (selected(${q501.1} , '2'))*<br>*Response constrained to: .>=0*	
q507 *(required)*	507. What is the year for which that cost estimate is based?<br>*Please enter a year in this format YYYY. Enter 2999 if unknown.*<br>*Question relevant when: (selected(${q501.1} , '2'))*<br>*Response constrained to: regex(., '[1-2][0-9][0-9][0-9]')*	
Photos of facility costs records (if available) *Group relevant when: (selected(${section_five_skip} , '2')) and (selected(${q501} , '1'))*		
records_photo_1	Photo of records 1	
records_photo_2	Photo of records 2	
records_photo_3	Photo of records 3	
FACILITY COST - ESTIMATES *Group relevant when: (selected(${section_five_skip} , '2')) and not(selected(${q501} , '1'))*		
note_no_records	If no records are available to show the cost of construction and equipment, please estimate (in Shillings) the cost for this year:<br>*Enter 99999 if unknown.*	
q508 *(required)*	508. Can you please estimate the cost of constructing this facility or a similar facility (in Shillings) for this year?<br>*Response constrained to: .>=0*	
q509 *(required)*	509. Can you please estimate the cost of equipping this facility or a similar facility (in Shillings) for this year?<br>*Response constrained to: .>=0*	
q510 *(required)*	510. Can you please estimate the average lifetime (or years of useful service) for this facility or a similar facility?<br>*Enter 999 if unknown.*<br>*Question relevant when: (selected(${section_five_skip} , '2'))*<br>*Response constrained to: .>=0*	
group_q511_overhead_cost *Group relevant when: (selected(${section_five_skip} , '2'))*		
generated_note_name_719	511. Can you please estimate the average annual cost of the following overhead expenses for your whole facility (in Shillings)?<br>*Enter 999 if amount unknown.*	
q511a *(required)*	q511a. Building maintenance cost<br>*Response constrained to: .>=0*	
q511b *(required)*	q511b. Total utilities cost (water, natural gas, electricity, telephones, trash, etc.)<br>*Response constrained to: .>=0*	
q511c *(required)*	q511c. Outsourced security service<br>*Response constrained to: .>=0*	
q511d *(required)*	q511d. Outsourced cleaning service<br>*Response constrained to: .>=0*	
q511e *(required)*	q511e. Vehicle maintenance cost, including insurance	

Field	Question	Answer
	Response constrained to: .>=0	
q511f *(required)*	q511f. Travel expenses (transportation, fuel, petrol, accommodation, meals, etc.)	
	Response constrained to: .>=0	
q511g *(required)*	q511g. Audio and visual materials<br>*Response constrained to: .>=0*	
q511h *(required)*	q511h. Educational/Reference materials for medical staff<br>*Response constrained to: .>=0*	
q511i *(required)*	q511i. Printed materials<br>*Response constrained to: .>=0*	

q511j *(required)*	q511j. Food/kitchen services<br>*Response constrained to: .>=0*	
q511k *(required)*	q511k. Sterilization services<br>*Response constrained to: .>=0*	
q511l *(required)*	q511l. Laundry (Linen/bedding/towel services)<br>*Response constrained to: .>=0*	
group_q512_m2_space<br>*Group relevant when: (selected(${section_five_skip} , '2'))*		
q512 *(required)*	512. Can you tell me the total number of square meters of floor space in your facility?<br>*If estimates are not availble, measure the space or enter 999 if not known.*<br>*Response constrained to: .>=0*	
generated_note_name_735	513. IF THE FACILITY HAS A DESIGNATED AREA FOR MATERNAL AND NEWBORN HEALTH SERVICES (E.G. AN OBSTETRICS AND GYNECOLOGICAL WARD), ASK:]	
q513 *(required)*	Can you estimate, within your facility, how many square meters are occupied by the spaces designated for maternal and newborn health services (e.g. the obstetrics and gynecological ward)?<br>*If estimates are not availble, measure the space or enter 999 if not known.*<br>*Response constrained to: .>=0*	
thank_you	**END OF INTERVIEW QUESTIONS.**<br><br>**THANK THE PARTICIPANT FOR THEIR TIME.**<br><br>**THEN COMPLETE THE FOLLOWING TWO ITEMS.**	
q514_interviewer_comments	514. INTERVIEWER COMMENTS - ENTER ANY RELEVANT NOTES AT THE END OF THE INTERVIEW.	
q515_time_end_qa *(required)*	515. ENTER THE END TIME OF THE INTERVIEW.<br>*NB: The default is the current time.*	
q516_GPS_QA2 *(required)*	516. GPS LOCATION CAPTURE<br>*Press the button to capture the GPS location at this point in the survey.*	
End_note	**END OF QUESTIONNAIRE A**<br><br>ON THE NEXT PAGE YOU'LL FIND THIS FACILITY'S ID NUMBER. TAKE NOTE OF THE NUMBER AND THEN, ON THE FOLLOWING PAGE, NAME THIS FORM BY ADDING THE FACILITY ID BEFORE THE FORM NAME.<br><br>FOR EXAMPLE, "20 Tanzania PAC cost study - Question A" WOULD INDICATE FACILITY NUMBER 20.<br><br>IF THE FORM IS FINAL BASED ON THIS INTERVIEW - I.E. THE RESPONDENT HAS ANSWERED ALL OF THE QUESTIONS THAT THEY CAN, THEN LEAVE THE DEFAULT BOX CHECKED. IF THE FORM IS NOT FINAL FOR THIS RESPONDENT, UNTICK THE BOX.<br><br>THEN SAVE AND EXIT.	
facility_id3	The ID for this facility is '[q4i_facility_id]".	
