# Supplementary material for: The health system costs of post abortion care in Tanzania
Source: BMC Health Serv Res. 2021 Jul 22;21:720. doi: 10.1186/s12913-021-06688-7 (PMC8296742; doi:10.1186/s12913-021-06688-7)
Supplement: Supplementary file 1 — Additional file 1. [file 12913_2021_6688_MOESM1_ESM.zip › QB-part 1 consumables 2019.01.28 FINALR3.pdf]

Tanzania PAC cost study – Quest. B part 1 consumables

Field	Question	Answer
about_survey	**Tanzania PAC cost study – Quest. B part 1 consumables**<br><br>INTERVIEWER INSTRUCTIONS:<br>• DO NOT READ TEXT IN ALL CAPS OR HINTS IN *ITALICS* ALOUD TO THE PARTICIPANT.<br>• BEFORE STARTING THE INTERVIEW, CHECK WHICH PARTS OR SECTIONS OF QUESTIONNAIRE B HAVE NOT BEEN COMPLETED.<br>• MAKE SURE THE PERSON YOU ARE ABOUT TO INTERVIEW HAS PROVIDED CONSENT.	
introduction	QUESTIONNAIRE INTRODUCTION: In this interview, I'm going to ask you a series of questions about what supplies (or "consumables), equipment, lab tests and medications are used when treating women with post abortion complications. We will do the interview in four parts – one for each of the item types. We can pause for breaks between the different parts.<br><br>For each part of the interview, I'll first present a list of items and ask which items are used for the five types of postabortion complications. Those are: uncomplicated incomplete abortion, sepsis, shock, cervical and vaginal lacerations, and vaginal and uterine perforations.<br><br>Then, if you tell me that an item is used at your facility, I'll separately ask how it is used (e.g. what proportion of women get it, how much they get, etc.).<br><br>At the end of interview, for any item that is used, I'll ask about prices for buying the items. If you don't have that information, I can obtain it from someone else at your facility after the interview.<br><br>Before we begin, I'm going to capture some information about where we are and document that I'm doing the interview with you. Remember that your name won't be used with the results later.	
COVER PAGE		
q1_interviewer_name_con *(required)*	q1. NAME OF INTERVIEWER	

Field	Question	Answer
q2_facility_name_con *(required)*	q2. SELECT: NAME OF FACILITY	1 Amana Hospital - Regional Referral Hospital<br>2 Bugara Dispensary<br>3 Bukiriro Health Center<br>4 Matemwe PHCU+<br>5 Ilagala Health Center<br>6 Iporoto Dispensary<br>7 Junguni PHCU<br>8 Kasulu District Hospital<br>9 Kifaru Dispensary<br>10 Kimara Health Center<br>11 Kimeya Health Center<br>12 Kintinku Health Center<br>13 Kishogo Dispensary<br>14 Kitahana Dispensary<br>15 Mahonda PHCU+<br>16 Majohe Dispensary<br>17 Marie Stopes Dispensary<br>18 Mbagala Kuu Dispensary<br>19 Mbagala Roundtable Health Center<br>20 Micheweni PHCC<br>21 Mission Mbagala Dispensary<br>22 Mnazi Mmoja Hospital<br>23 Mteke Dispensary<br>24 Mugana DD Hospital<br>25 Muhimbili National Hospital<br>26 Muyenje Dispensary<br>27 Muzdalfa Kiwalani Dispensary<br>28 Mwandiga Dispensary<br>29 Narumu Govt Dispensary<br>30 Nkwenda Health Center<br>31 Ntungwa Dispensary<br>32 Pamila Dispensary<br>33 Rusesa Health Center<br>34 Rusohoko Dispensary<br>35 Ruvuma Regional Hospital<br>36 Semeni Dispensary<br>37 Siha Health Center<br>38 Tabata A Dispensary<br>39 Tumaini Mission Health Center<br>40 Chukwani PHCU+
q3_GPS_QB_con *(required)*	q3. CAPTURE THE GPS LOCATION<br>*Press the button to capture the GPS location at this point in the survey.*	
RESPONDENTS (1-5)		
cover_page_note_1	INTERVIEWER INSTRUCTIONS: INDICATE NAMES, JOB TITLES AND CONTACT INFORMATION FOR UP TO 5 RESPONDENTS OF QUESTIONNAIRE A.<br>*NB: Information for only the first respondent is required in order to proceed to the next page. Ask for the information as a question if not already known prior to the interview.*	
group_respondent1	RESPONDENT 1	
q6a_name_con *(required)*	q6a. Name of 1st respondent<br>*First and last name*	
q6b_date_con *(required)*	q6b. Date of interview with 1st respondent<br>*Default is today's date.*	
q6c_title_con *(required)*	q6c. Designation/title of 1st respondent<br>*Job title and designation (e.g. Medical officer in charge)*	
q6d_phone_con	q6d. Phone number of 1st respondent<br>*NB: Not required, but you might need this to follow up with questions.*	
group_respondent2_con	RESPONDENT 2	
q7a_name_con	q7a. Name of 2nd respondent<br>*First and last name*	
q7b_date_con	q7b. Date of interview with 2nd respondent<br>*Default is today's date.*	

Field	Question	Answer
q7c_title_con	q7c. Designation/title of 2nd respondent<br>*Job title and designation (e.g. Medical officer in charge)*	
q7d_phone_con	q7d. Phone number of 2nd respondent<br>*NB: Not required, but you might need this to follow up with questions.*	
group_respondent3_con	RESPONDENT 3	
q8a_name_con	q8a. Name of 3rd respondent<br>*First and last name*	
q8b_date_con	q8b. Date of interview with 3rd respondent<br>*Default is today's date.*	
q8c_title_con	q8c. Designation/title of 3rd respondent<br>*Job title and designation (e.g. Medical officer in charge)*	
q8d_phone_con	q8d. Phone number of 3rd respondent<br>*NB: Not required, but you might need this to follow up with questions.*	
group_respondent4_con	RESPONDENT 4	
q9a_name_con	q9a. Name of 4th respondent<br>*First and last name*	
q9b_date_con	q9b. Date of interview with 4th respondent<br>*Default is today's date.*	
q9c_title_con	q9c. Designation/title of 4th respondent<br>*Job title and designation (e.g. Medical officer in charge)*	
q9d_phone_con	q9d. Phone number of 4th respondent<br>*NB: Not required, but you might need this to follow up with questions.*	
group_respondent5_con	RESPONDENT 5	
q10a_name_con	q10a. Name of 5th respondent<br>*First and last name*	
q10b_date_con	q10b. Date of interview with 5th respondent<br>*Default is today's date.*	
q10c_title_con	q10c. Designation/title of 5th respondent<br>*Job title and designation (e.g. Medical officer in charge)*	
q10d_phone_con	q10d. Phone number of 5th respondent<br>*NB: Not required, but you might need this to follow up with questions.*	
q10_interviewer_comments_con	q11. INTERVIEWER COMMENTS<br>*Enter any relevant notes prior to the interview.*	
q12_time_start_qa_con *(required)*	q12. ENTER START TIME OF INTERVIEW<br>*NB: The default is the current time.*	
introduction_con	QUESTIONNAIRE INTRODUCTION: Ok now we can begin the interview. Remember that all the questions in this part of the interview are about supplies, or consumable items, that are used to provide PAC at your facility.	
group_section_one_intro		
section1_start	**SECTION I. FULL LISTING OF ITEMS BY COMPLICATION TYPE**	
section_one_skip_con *(required)*	INTERVIEWER: WOULD YOU LIKE TO COMPLETE THIS SECTION NOW OR SKIP THIS SECTION AND RETURN TO IT LATER?<br>*You may need to skip if the participant has indicated that s/he cannot answer the questions in this section.*	1 Do not skip, complete this section now.<br>2 Skip and come back to this section later.
A. Consumables - Full list (1)<br>*Group relevant when: selected(${section_one_skip_con} , '1')*		
q101_consumables_note1	101. For each of the following single-use supply items, can you tell me if the item is used for post abortion care at your facility? I'm going to ask about each of the five complication types separately. Is [ITEM] used for managing women with [COMPLICATION TYPE]?	
note_101_cleaning	***Cleaning, disinfectants, sterilization, etc.***	
q101_C1_full_list *(required)*	q101_C1. Alcohol, denatured 70%<br>*Select all that apply.*<br>*Response constrained to: if(selected(., 6) or selected(., 99), count-selected(.) = 1, count-selected(.) >= 1)*	1 Incomplete abortion<br>2 Sepsis<br>3 Shock<br>4 Cervical/vaginal lacerations<br>5 Vaginal/uterine perforation<br>6 Not used at this facility<br>99 Don't know
q101_C2_full_list *(required)*	q101_C2. Chlorine (JIK) or Glutaraldehyde solution<br>*Select all that apply.*<br>*Response constrained to: if(selected(., 6) or selected(., 99), count-selected(.) = 1, count-selected(.) >= 1)*	1 Incomplete abortion<br>2 Sepsis<br>3 Shock<br>4 Cervical/vaginal lacerations<br>5 Vaginal/uterine perforation<br>6 Not used at this facility<br>99 Don't know

Field	Question	Answer
q101_C3_full_list *(required)*	q101_C3. Clean water<br>*Select all that apply.*<br>*Response constrained to: if(selected(., 6) or selected(., 99), count-selected(.) = 1, count-selected(.) >= 1)*	1 Incomplete abortion<br>2 Sepsis<br>3 Shock<br>4 Cervical/vaginal lacerations<br>5 Vaginal/uterine perforation<br>6 Not used at this facility<br>99 Don't know
q101_C4_full_list *(required)*	q101_C4. Dettol solution or other water-based antiseptic<br>*Select all that apply.*<br>*Response constrained to: if(selected(., 6) or selected(., 99), count-selected(.) = 1, count-selected(.) >= 1)*	1 Incomplete abortion<br>2 Sepsis<br>3 Shock<br>4 Cervical/vaginal lacerations<br>5 Vaginal/uterine perforation<br>6 Not used at this facility<br>99 Don't know
q101_C5_full_list *(required)*	q101_C5. Cetrimide 15% ("Savlon")<br>*Select all that apply.*<br>*Response constrained to: if(selected(., 6) or selected(., 99), count-selected(.) = 1, count-selected(.) >= 1)*	1 Incomplete abortion<br>2 Sepsis<br>3 Shock<br>4 Cervical/vaginal lacerations<br>5 Vaginal/uterine perforation<br>6 Not used at this facility<br>99 Don't know
q101_C6_full_list *(required)*	q101_C6. Hand wash (liquid)<br>*Select all that apply.*<br>*Response constrained to: if(selected(., 6) or selected(., 99), count-selected(.) = 1, count-selected(.) >= 1)*	1 Incomplete abortion<br>2 Sepsis<br>3 Shock<br>4 Cervical/vaginal lacerations<br>5 Vaginal/uterine perforation<br>6 Not used at this facility<br>99 Don't know
q101_C7_full_list *(required)*	q101_C7. Soap (bar)<br>*Select all that apply.*<br>*Response constrained to: if(selected(., 6) or selected(., 99), count-selected(.) = 1, count-selected(.) >= 1)*	1 Incomplete abortion<br>2 Sepsis<br>3 Shock<br>4 Cervical/vaginal lacerations<br>5 Vaginal/uterine perforation<br>6 Not used at this facility<br>99 Don't know
q101_C8_full_list *(required)*	q101_C8. Hand sterilizer (waterless)<br>*Select all that apply.*<br>*Response constrained to: if(selected(., 6) or selected(., 99), count-selected(.) = 1, count-selected(.) >= 1)*	1 Incomplete abortion<br>2 Sepsis<br>3 Shock<br>4 Cervical/vaginal lacerations<br>5 Vaginal/uterine perforation<br>6 Not used at this facility<br>99 Don't know
A. Consumables - Full list (2)<br>*Group relevant when: selected(${section_one_skip_con} , '1')*		
q101_consumables_note2	101 CONTINUED. For each of the following single-use supply items, can you tell me if the item is used for post abortion care at your facility? I'm going to ask about each of the five complication types separately. Is [ITEM] used for managing women with [COMPLICATION TYPE]?	
note_101_IV_needle_syringe	***IV, needles, syringes, etc.***	
q101_C9_full_list *(required)*	q101_C9. Blood giving set with needle<br>*Select all that apply.*<br>*Response constrained to: if(selected(., 6) or selected(., 99), count-selected(.) = 1, count-selected(.) >= 1)*	1 Incomplete abortion<br>2 Sepsis<br>3 Shock<br>4 Cervical/vaginal lacerations<br>5 Vaginal/uterine perforation<br>6 Not used at this facility<br>99 Don't know

Field	Question	Answer
q101_C10_full_list *(required)*	q101_C10. IV cannula<br>*Select all that apply.*<br>*Response constrained to: if(selected(., 6) or selected(., 99), count-selected(.) = 1, count-selected(.) >= 1)*	1 Incomplete abortion<br>2 Sepsis<br>3 Shock<br>4 Cervical/vaginal lacerations<br>5 Vaginal/uterine perforation<br>6 Not used at this facility<br>99 Don't know
q101_C11_full_list *(required)*	q101_C11. IV set<br>*Select all that apply.*<br>*Response constrained to: if(selected(., 6) or selected(., 99), count-selected(.) = 1, count-selected(.) >= 1)*	1 Incomplete abortion<br>2 Sepsis<br>3 Shock<br>4 Cervical/vaginal lacerations<br>5 Vaginal/uterine perforation<br>6 Not used at this facility<br>99 Don't know
q101_C12_full_list *(required)*	q101_C12. Needle luer tip 18Gx1.1/2" (1.2x38mm)<br>*Select all that apply.*<br>*Response constrained to: if(selected(., 6) or selected(., 99), count-selected(.) = 1, count-selected(.) >= 1)*	1 Incomplete abortion<br>2 Sepsis<br>3 Shock<br>4 Cervical/vaginal lacerations<br>5 Vaginal/uterine perforation<br>6 Not used at this facility<br>99 Don't know
q101_C13_full_list *(required)*	q101_C13. Needle luer tip 21Gx1.1/2" (0.8x38mm)<br>*Select all that apply.*<br>*Response constrained to: if(selected(., 6) or selected(., 99), count-selected(.) = 1, count-selected(.) >= 1)*	1 Incomplete abortion<br>2 Sepsis<br>3 Shock<br>4 Cervical/vaginal lacerations<br>5 Vaginal/uterine perforation<br>6 Not used at this facility<br>99 Don't know
q101_C14_full_list *(required)*	q101_C14. Needle, suture, round body<br>*Select all that apply.*<br>*Response constrained to: if(selected(., 6) or selected(., 99), count-selected(.) = 1, count-selected(.) >= 1)*	1 Incomplete abortion<br>2 Sepsis<br>3 Shock<br>4 Cervical/vaginal lacerations<br>5 Vaginal/uterine perforation<br>6 Not used at this facility<br>99 Don't know
q101_C15_full_list *(required)*	q101_C15. Spinal needle<br>*Select all that apply.*<br>*Response constrained to: if(selected(., 6) or selected(., 99), count-selected(.) = 1, count-selected(.) >= 1)*	1 Incomplete abortion<br>2 Sepsis<br>3 Shock<br>4 Cervical/vaginal lacerations<br>5 Vaginal/uterine perforation<br>6 Not used at this facility<br>99 Don't know
q101_C16_full_list *(required)*	q101_C16. Syringe 10ml, with bypacked needle 21Gx1.5"<br>*Select all that apply.*<br>*Response constrained to: if(selected(., 6) or selected(., 99), count-selected(.) = 1, count-selected(.) >= 1)*	1 Incomplete abortion<br>2 Sepsis<br>3 Shock<br>4 Cervical/vaginal lacerations<br>5 Vaginal/uterine perforation<br>6 Not used at this facility<br>99 Don't know
q101_C17_full_list *(required)*	q101_C17. Syringe 5ml, with bypacked needle 21Gx1.5"<br>*Select all that apply.*<br>*Response constrained to: if(selected(., 6) or selected(., 99), count-selected(.) = 1, count-selected(.) >= 1)*	1 Incomplete abortion<br>2 Sepsis<br>3 Shock<br>4 Cervical/vaginal lacerations<br>5 Vaginal/uterine perforation<br>6 Not used at this facility<br>99 Don't know

Field	Question	Answer
q101_C18_full_list *(required)*	q101_C18. Syringe luer tip 10ml, no needle<br>*Select all that apply.*<br>*Response constrained to: if(selected(., 6) or selected(., 99), count-selected(.) = 1, count-selected(.) >= 1)*	1 Incomplete abortion<br>2 Sepsis<br>3 Shock<br>4 Cervical/vaginal lacerations<br>5 Vaginal/uterine perforation<br>6 Not used at this facility<br>99 Don't know
q101_C19_full_list *(required)*	q101_C19. Syringe luer tip 5ml, no needle<br>*Select all that apply.*<br>*Response constrained to: if(selected(., 6) or selected(., 99), count-selected(.) = 1, count-selected(.) >= 1)*	1 Incomplete abortion<br>2 Sepsis<br>3 Shock<br>4 Cervical/vaginal lacerations<br>5 Vaginal/uterine perforation<br>6 Not used at this facility<br>99 Don't know
q101_C20_full_list *(required)*	q101_C20. Venous catheter G18 + injection port and lock tip<br>*Select all that apply.*<br>*Response constrained to: if(selected(., 6) or selected(., 99), count-selected(.) = 1, count-selected(.) >= 1)*	1 Incomplete abortion<br>2 Sepsis<br>3 Shock<br>4 Cervical/vaginal lacerations<br>5 Vaginal/uterine perforation<br>6 Not used at this facility<br>99 Don't know
A. Consumables - Full list (3)<br>*Group relevant when: selected(${section_one_skip_con} , '1')*		
q101_consumables_note3	101 CONTINUED. For each of the following single-use supply items, can you tell me if the item is used for post abortion care at your facility? I'm going to ask about each of the five complication types separately. Is [ITEM] used for managing women with [COMPLICATION TYPE]?	
note_101_bandages_etc	***Bandages, absorbants, wound care, etc.***	
q101_C21_full_list *(required)*	q101_C21. Adhesive tape<br>*Select all that apply.*<br>*Response constrained to: if(selected(., 6) or selected(., 99), count-selected(.) = 1, count-selected(.) >= 1)*	1 Incomplete abortion<br>2 Sepsis<br>3 Shock<br>4 Cervical/vaginal lacerations<br>5 Vaginal/uterine perforation<br>6 Not used at this facility<br>99 Don't know
q101_C22_full_list *(required)*	q101_C22. Cotton swab<br>*Select all that apply.*<br>*Response constrained to: if(selected(., 6) or selected(., 99), count-selected(.) = 1, count-selected(.) >= 1)*	1 Incomplete abortion<br>2 Sepsis<br>3 Shock<br>4 Cervical/vaginal lacerations<br>5 Vaginal/uterine perforation<br>6 Not used at this facility<br>99 Don't know
q101_C23_full_list *(required)*	q101_C23. Dressing pad absorbent sterile<br>*Select all that apply.*<br>*Response constrained to: if(selected(., 6) or selected(., 99), count-selected(.) = 1, count-selected(.) >= 1)*	1 Incomplete abortion<br>2 Sepsis<br>3 Shock<br>4 Cervical/vaginal lacerations<br>5 Vaginal/uterine perforation<br>6 Not used at this facility<br>99 Don't know
q101_C24_full_list *(required)*	q101_C24. Gauze compress, non sterile<br>*Select all that apply.*<br>*Response constrained to: if(selected(., 6) or selected(., 99), count-selected(.) = 1, count-selected(.) >= 1)*	1 Incomplete abortion<br>2 Sepsis<br>3 Shock<br>4 Cervical/vaginal lacerations<br>5 Vaginal/uterine perforation<br>6 Not used at this facility<br>99 Don't know

Field	Question	Answer
q101_C25_full_list *(required)*	q101_C25. Gauze compress, sterile<br>*Select all that apply.*<br>*Response constrained to: if(selected(., 6) or selected(., 99), count-selected(.) = 1, count-selected(.) >= 1)*	1 Incomplete abortion<br>2 Sepsis<br>3 Shock<br>4 Cervical/vaginal lacerations<br>5 Vaginal/uterine perforation<br>6 Not used at this facility<br>99 Don't know
q101_C26_full_list *(required)*	q101_C26. Linen saver<br>*Select all that apply.*<br>*Response constrained to: if(selected(., 6) or selected(., 99), count-selected(.) = 1, count-selected(.) >= 1)*	1 Incomplete abortion<br>2 Sepsis<br>3 Shock<br>4 Cervical/vaginal lacerations<br>5 Vaginal/uterine perforation<br>6 Not used at this facility<br>99 Don't know
q101_C27_full_list *(required)*	q101_C27. Sanitary Pad<br>*Select all that apply.*<br>*Response constrained to: if(selected(., 6) or selected(., 99), count-selected(.) = 1, count-selected(.) >= 1)*	1 Incomplete abortion<br>2 Sepsis<br>3 Shock<br>4 Cervical/vaginal lacerations<br>5 Vaginal/uterine perforation<br>6 Not used at this facility<br>99 Don't know
q101_C28_full_list *(required)*	q101_C28. Surgical plaster<br>*Select all that apply.*<br>*Response constrained to: if(selected(., 6) or selected(., 99), count-selected(.) = 1, count-selected(.) >= 1)*	1 Incomplete abortion<br>2 Sepsis<br>3 Shock<br>4 Cervical/vaginal lacerations<br>5 Vaginal/uterine perforation<br>6 Not used at this facility<br>99 Don't know
A. Consumables - Full list (4)<br>*Group relevant when: selected(${section_one_skip_con} , '1')*		
q101_consumables_note4	101 CONTINUED. For each of the following single-use supply items, can you tell me if the item is used for post abortion care at your facility? I'm going to ask about each of the five complication types separately. Is [ITEM] used for managing women with [COMPLICATION TYPE]?	
note_101_protective_etc	***Protective supplies***	
q101_C29_full_list *(required)*	q101_C29. Apron (disposable)<br>*Select all that apply.*<br>*Response constrained to: if(selected(., 6) or selected(., 99), count-selected(.) = 1, count-selected(.) >= 1)*	1 Incomplete abortion<br>2 Sepsis<br>3 Shock<br>4 Cervical/vaginal lacerations<br>5 Vaginal/uterine perforation<br>6 Not used at this facility<br>99 Don't know
q101_C30_full_list *(required)*	q101_C30. Gown (disposable)<br>*Select all that apply.*<br>*Response constrained to: if(selected(., 6) or selected(., 99), count-selected(.) = 1, count-selected(.) >= 1)*	1 Incomplete abortion<br>2 Sepsis<br>3 Shock<br>4 Cervical/vaginal lacerations<br>5 Vaginal/uterine perforation<br>6 Not used at this facility<br>99 Don't know
q101_C31_full_list *(required)*	q101_C31. Latex examination glove, without powder<br>*Select all that apply.*<br>*Response constrained to: if(selected(., 6) or selected(., 99), count-selected(.) = 1, count-selected(.) >= 1)*	1 Incomplete abortion<br>2 Sepsis<br>3 Shock<br>4 Cervical/vaginal lacerations<br>5 Vaginal/uterine perforation<br>6 Not used at this facility<br>99 Don't know

Field	Question	Answer
q101_C32_full_list *(required)*	q101_C32. Latex examination glove, with powder<br>*Select all that apply.*<br>*Response constrained to: if(selected(., 6) or selected(., 99), count-selected(.) = 1, count-selected(.) >= 1)*	1 Incomplete abortion<br>2 Sepsis<br>3 Shock<br>4 Cervical/vaginal lacerations<br>5 Vaginal/uterine perforation<br>6 Not used at this facility<br>99 Don't know
q101_C33_full_list *(required)*	q101_C33. Non-latex (e.g. nitrile) examination glove<br>*Select all that apply.*<br>*Response constrained to: if(selected(., 6) or selected(., 99), count-selected(.) = 1, count-selected(.) >= 1)*	1 Incomplete abortion<br>2 Sepsis<br>3 Shock<br>4 Cervical/vaginal lacerations<br>5 Vaginal/uterine perforation<br>6 Not used at this facility<br>99 Don't know
q101_C34_full_list *(required)*	q101_C34. Sterile surgical glove<br>*Select all that apply.*<br>*Response constrained to: if(selected(., 6) or selected(., 99), count-selected(.) = 1, count-selected(.) >= 1)*	1 Incomplete abortion<br>2 Sepsis<br>3 Shock<br>4 Cervical/vaginal lacerations<br>5 Vaginal/uterine perforation<br>6 Not used at this facility<br>99 Don't know
q101_C35_full_list *(required)*	q101_C35. Mask (disposable)<br>*Select all that apply.*<br>*Response constrained to: if(selected(., 6) or selected(., 99), count-selected(.) = 1, count-selected(.) >= 1)*	1 Incomplete abortion<br>2 Sepsis<br>3 Shock<br>4 Cervical/vaginal lacerations<br>5 Vaginal/uterine perforation<br>6 Not used at this facility<br>99 Don't know
q101_C36_full_list *(required)*	q101_C36. Surgical cap<br>*Select all that apply.*<br>*Response constrained to: if(selected(., 6) or selected(., 99), count-selected(.) = 1, count-selected(.) >= 1)*	1 Incomplete abortion<br>2 Sepsis<br>3 Shock<br>4 Cervical/vaginal lacerations<br>5 Vaginal/uterine perforation<br>6 Not used at this facility<br>99 Don't know
A. Consumables - Full list (5)<br>*Group relevant when: selected(${section_one_skip_con} , '1')*		
q101_consumables_note5	101 CONTINUED. For each of the following single-use supply items, can you tell me if the item is used for post abortion care at your facility? I'm going to ask about each of the five complication types separately. Is [ITEM] used for managing women with [COMPLICATION TYPE]?	
note_101_surgery_etc	***Surgery supplies***	
q101_C37_full_list *(required)*	q101_C37. Scalpel blade, single use<br>*Select all that apply.*<br>*Response constrained to: if(selected(., 6) or selected(., 99), count-selected(.) = 1, count-selected(.) >= 1)*	1 Incomplete abortion<br>2 Sepsis<br>3 Shock<br>4 Cervical/vaginal lacerations<br>5 Vaginal/uterine perforation<br>6 Not used at this facility<br>99 Don't know
q101_C38_full_list *(required)*	q101_C38. Surgical drain<br>*Select all that apply.*<br>*Response constrained to: if(selected(., 6) or selected(., 99), count-selected(.) = 1, count-selected(.) >= 1)*	1 Incomplete abortion<br>2 Sepsis<br>3 Shock<br>4 Cervical/vaginal lacerations<br>5 Vaginal/uterine perforation<br>6 Not used at this facility<br>99 Don't know

Field	Question	Answer
q101_C39_full_list *(required)*	q101_C39. Suture string, chromic catgut (0)<br>*Select all that apply.*<br>*Response constrained to: if(selected(., 6) or selected(., 99), count-selected(.) = 1, count-selected(.) >= 1)*	1 Incomplete abortion<br>2 Sepsis<br>3 Shock<br>4 Cervical/vaginal lacerations<br>5 Vaginal/uterine perforation<br>6 Not used at this facility<br>99 Don't know
q101_C40_full_list *(required)*	q101_C40. Suture string, silk (non absorbable and non-synthetic)<br>*Select all that apply.*<br>*Response constrained to: if(selected(., 6) or selected(., 99), count-selected(.) = 1, count-selected(.) >= 1)*	1 Incomplete abortion<br>2 Sepsis<br>3 Shock<br>4 Cervical/vaginal lacerations<br>5 Vaginal/uterine perforation<br>6 Not used at this facility<br>99 Don't know
q101_C41_full_list *(required)*	q101_C41. Suture string, vicryl, no needle included<br>*Select all that apply.*<br>*Response constrained to: if(selected(., 6) or selected(., 99), count-selected(.) = 1, count-selected(.) >= 1)*	1 Incomplete abortion<br>2 Sepsis<br>3 Shock<br>4 Cervical/vaginal lacerations<br>5 Vaginal/uterine perforation<br>6 Not used at this facility<br>99 Don't know
q101_C42_full_list *(required)*	q101_C42. Suture string, vicryl with needle<br>*Select all that apply.*<br>*Response constrained to: if(selected(., 6) or selected(., 99), count-selected(.) = 1, count-selected(.) >= 1)*	1 Incomplete abortion<br>2 Sepsis<br>3 Shock<br>4 Cervical/vaginal lacerations<br>5 Vaginal/uterine perforation<br>6 Not used at this facility<br>99 Don't know
A. Consumables - Full list (6)<br>*Group relevant when: selected(${section_one_skip_con} , '1')*		
q101_consumables_note6	101 CONTINUED. For each of the following single-use supply items, can you tell me if the item is used for post abortion care at your facility? I'm going to ask about each of the five complication types separately. Is [ITEM] used for managing women with [COMPLICATION TYPE]?	
note_101_urine_etc	***Urine, bladder***	
q101_C43_full_list *(required)*	q101_C43. Bladder/urinary catheter<br>*Select all that apply.*<br>*Response constrained to: if(selected(., 6) or selected(., 99), count-selected(.) = 1, count-selected(.) >= 1)*	1 Incomplete abortion<br>2 Sepsis<br>3 Shock<br>4 Cervical/vaginal lacerations<br>5 Vaginal/uterine perforation<br>6 Not used at this facility<br>99 Don't know
q101_C44_full_list *(required)*	q101_C44. Foley's catheter<br>*Select all that apply.*<br>*Response constrained to: if(selected(., 6) or selected(., 99), count-selected(.) = 1, count-selected(.) >= 1)*	1 Incomplete abortion<br>2 Sepsis<br>3 Shock<br>4 Cervical/vaginal lacerations<br>5 Vaginal/uterine perforation<br>6 Not used at this facility<br>99 Don't know
q101_C45_full_list *(required)*	q101_C45. Urine bag with valve and drain, 2000 ml<br>*Select all that apply.*<br>*Response constrained to: if(selected(., 6) or selected(., 99), count-selected(.) = 1, count-selected(.) >= 1)*	1 Incomplete abortion<br>2 Sepsis<br>3 Shock<br>4 Cervical/vaginal lacerations<br>5 Vaginal/uterine perforation<br>6 Not used at this facility<br>99 Don't know
A. Consumables - Full list (7)<br>*Group relevant when: selected(${section_one_skip_con} , '1')*		
q101_consumables_note7	101 CONTINUED. For each of the following single-use supply items, can you tell me if the item is used for post abortion care at your facility? I'm going to ask about each of the five complication types separately. Is [ITEM] used for managing women with [COMPLICATION TYPE]?	

Field	Question	Answer
note_101_other_etc	***Other supplies***	
q101_C46_full_list *(required)*	q101_C46. Silicone oil for lubrication of MVA aspirator<br>*Select all that apply.*<br>*Response constrained to: if(selected(., 6) or selected(., 99), count-selected(.) = 1, count-selected(.) >= 1)*	1 Incomplete abortion<br>2 Sepsis<br>3 Shock<br>4 Cervical/vaginal lacerations<br>5 Vaginal/uterine perforation<br>6 Not used at this facility<br>99 Don't know
q101_C47_full_list *(required)*	q101_C47. Speculum, Bivalve/Cuscos (reusable)<br>*Select all that apply.*<br>*Response constrained to: if(selected(., 6) or selected(., 99), count-selected(.) = 1, count-selected(.) >= 1)*	1 Incomplete abortion<br>2 Sepsis<br>3 Shock<br>4 Cervical/vaginal lacerations<br>5 Vaginal/uterine perforation<br>6 Not used at this facility<br>99 Don't know
q101_C48_full_list *(required)*	q101_C48. Speculum, Sim's (disposable)<br>*Select all that apply.*<br>*Response constrained to: if(selected(., 6) or selected(., 99), count-selected(.) = 1, count-selected(.) >= 1)*	1 Incomplete abortion<br>2 Sepsis<br>3 Shock<br>4 Cervical/vaginal lacerations<br>5 Vaginal/uterine perforation<br>6 Not used at this facility<br>99 Don't know
q101_consumables_other *(required)*	q101_Consum_Other. Are there any single-use supply items that we have not mentioned that are used for postabortion care at your facility?<br>*Question relevant when: selected(${section_one_skip_con} , '1')*	1 Yes<br>0 No<br>99 Don't know
A. Consumables - Full list - specify (8)<br>*Group relevant when: selected(${q101_consumables_other} , '1') and selected(${section_one_skip_con} , '1')*		
note_101_other_suggestion	Please list the "other" items here.	
q101_C49_full_list_other	q101_C49. Other consumble 1. Please specify:	
q101_C50_full_list_other	q101_C50. Other consumable 2. Please specify:	
q101_C51_full_list_other	q101_C51. Other consumable 3. Please specify:	
q101_C52_full_list_other	q101_C52. Other consumable 4. Please specify:	
q101_C53_full_list_other	q101_C53. Other consumable 5. Please specify:	
A. Consumables - Full list - usage (8b)<br>*Group relevant when: selected(${q101_consumables_other} , '1') and selected(${section_one_skip_con} , '1')*		
note_101_other_suggestion_b	For each other supply item, please tell me which of the five post abortion complication types it is used for.	
q101_C49_full_list *(required)*	q101_C49. Other consumable 1: "[q101_C49_full_list_other]"<br>*Select all that apply.*<br>*Question relevant when: string-length(${q101_C49_full_list_other}) > 0 and selected(${section_one_skip_con} , '1')*<br>*Response constrained to: if(selected(., 6) or selected(., 99), count-selected(.) = 1, count-selected(.) >= 1)*	1 Incomplete abortion<br>2 Sepsis<br>3 Shock<br>4 Cervical/vaginal lacerations<br>5 Vaginal/uterine perforation<br>6 Not used at this facility<br>99 Don't know
q101_C50_full_list *(required)*	q101_C50. Other consumable 2: "[q101_C50_full_list_other]"<br>*Select all that apply.*<br>*Question relevant when: string-length(${q101_C50_full_list_other}) > 0 and selected(${section_one_skip_con} , '1')*<br>*Response constrained to: if(selected(., 6) or selected(., 99), count-selected(.) = 1, count-selected(.) >= 1)*	1 Incomplete abortion<br>2 Sepsis<br>3 Shock<br>4 Cervical/vaginal lacerations<br>5 Vaginal/uterine perforation<br>6 Not used at this facility<br>99 Don't know
q101_C51_full_list *(required)*	q101_C51. Other consumable 3: "[q101_C51_full_list_other]"<br>*Select all that apply.*<br>*Question relevant when: string-length(${q101_C51_full_list_other}) > 0 and selected(${section_one_skip_con} , '1')*<br>*Response constrained to: if(selected(., 6) or selected(., 99), count-selected(.) = 1, count-selected(.) >= 1)*	1 Incomplete abortion<br>2 Sepsis<br>3 Shock<br>4 Cervical/vaginal lacerations<br>5 Vaginal/uterine perforation<br>6 Not used at this facility<br>99 Don't know

Field	Question	Answer
q101_C52_full_list *(required)*	q101_C52. Other consumable 4: "[q101_C52_full_list_other]"<br>*Select all that apply.*<br>*Question relevant when: string-length(${q101_C52_full_list_other}) > 0 and selected(${section_one_skip_con} , '1')*<br>*Response constrained to: if(selected(., 6) or selected(., 99), count-selected(.) = 1, count-selected(.) >= 1)*	1 Incomplete abortion<br>2 Sepsis<br>3 Shock<br>4 Cervical/vaginal lacerations<br>5 Vaginal/uterine perforation<br>6 Not used at this facility<br>99 Don't know
q101_C53_full_list *(required)*	q101_C53. Other consumable 5: "[q101_C53_full_list_other]"<br>*Select all that apply.*<br>*Question relevant when: string-length(${q101_C53_full_list_other}) > 0 and selected(${section_one_skip_con} , '1')*<br>*Response constrained to: if(selected(., 6) or selected(., 99), count-selected(.) = 1, count-selected(.) >= 1)*	1 Incomplete abortion<br>2 Sepsis<br>3 Shock<br>4 Cervical/vaginal lacerations<br>5 Vaginal/uterine perforation<br>6 Not used at this facility<br>99 Don't know
group_section_two_intro		
section2_start	**SECTION II. INCOMPLETE ABORTION - USAGE OF ALL ITEMS**	
section_two_skip_con *(required)*	INTERVIEWER: WOULD YOU LIKE TO COMPLETE THIS SECTION NOW OR SKIP THIS SECTION AND RETURN TO IT LATER?<br>*You may need to skip if the participant has indicated that s/he cannot answer the questions in this section.*	1 Do not skip, complete this section now.<br>2 Skip and come back to this section later.
group_section_two_introB<br>*Group relevant when: selected(${section_two_skip_con} , '1')*		
section2_start2	In this section of the interview, we will review all of the items that you said are used for management of "uncomplicated" incomplete abortion. For each item that is used, I'm going to ask questions on how many women need it and how much of it is used.	
section2_start3	INTERVIEWER: ENTER WHOLE NUMBERS OR DECIMALS. DO NOT TYPE PERCENT SIGNS. ENTER 999 FOR ANY THAT ARE UNKNOWN.	
A. Consumables - Incomplete Abortion (1)<br>*Group relevant when: selected(${section_two_skip_con} , '1')*		
note_201_cleaning	***Cleaning, disinfectants, sterilization, etc.***	
q201_C1	q201_C1. Alcohol, denatured 70%<br>*Question relevant when: selected(${q101_C1_full_list} , '1')*	
q201_C1a *(required)*	q201_C1a. What percent of patients require this item?<br>*Question relevant when: selected(${q101_C1_full_list} , '1')*<br>*Response constrained to: .>0 and .<=100 or .=999*	
q201_C1b *(required)*	q201_C1b. How many units of this item are required per patient (throughout the entire treatment provided for complication)?<br>*Smallest unit is mililiter (ml).*<br>*Question relevant when: selected(${q101_C1_full_list} , '1')*<br>*Response constrained to: .>0*	
q201_C2	q201_C2. Chlorine (JIK) or Glutaraldehyde solution<br>*Question relevant when: selected(${q101_C2_full_list} , '1')*	
q201_C2a *(required)*	q201_C2a. What percent of patients require this item?<br>*Question relevant when: selected(${q101_C2_full_list} , '1')*<br>*Response constrained to: .>0 and .<=100 or .=999*	
q201_C2b *(required)*	q201_C2b. How many units of this item are required per patient (throughout the entire treatment provided for complication)?<br>*Smallest unit is mililiter (ml)*<br>*Question relevant when: selected(${q101_C2_full_list} , '1')*<br>*Response constrained to: .>0*	
q201_C3	q201_C3. Clean water<br>*Question relevant when: selected(${q101_C3_full_list} , '1')*	
q201_C3a *(required)*	q201_C3a. What percent of patients require this item?<br>*Question relevant when: selected(${q101_C3_full_list} , '1')*<br>*Response constrained to: .>0 and .<=100 or .=999*	
q201_C3b *(required)*	q201_C3b. How many units of this item are required per patient (throughout the entire treatment provided for complication)?<br>*Smallest unit is litre (ltr)*<br>*Question relevant when: selected(${q101_C3_full_list} , '1')*<br>*Response constrained to: .>0*	
q201_C4	q201_C4. Dettol solution or other water-based antiseptic<br>*Question relevant when: selected(${q101_C4_full_list} , '1')*	

Field	Question	Answer
q201_C4a *(required)*	q201_C4a. What percent of patients require this item?<br>*Question relevant when: selected(${q101_C4_full_list} , '1')*<br>*Response constrained to: .>0 and .<=100 or .=999*	
q201_C4b *(required)*	q201_C4b. How many units of this item are required per patient (throughout the entire treatment provided for complication)?<br>*Smallest unit is milliliter (ml)*<br>*Question relevant when: selected(${q101_C4_full_list} , '1')*<br>*Response constrained to: .>0*	
q201_C5	q201_C5. Cetrimide 15% ("Savlon")<br>*Question relevant when: selected(${q101_C5_full_list} , '1')*	
q201_C5a *(required)*	q201_C5a. What percent of patients require this item?<br>*Question relevant when: selected(${q101_C5_full_list} , '1')*<br>*Response constrained to: .>0 and .<=100 or .=999*	
q201_C5b *(required)*	q201_C5b. How many units of this item are required per patient (throughout the entire treatment provided for complication)?<br>*Smallest unit is milliliter (ml)*<br>*Question relevant when: selected(${q101_C5_full_list} , '1')*<br>*Response constrained to: .>0*	
q201_C6	q201_C6. Hand wash (liquid)<br>*Question relevant when: selected(${q101_C6_full_list} , '1')*	
q201_C6a *(required)*	q201_C6a. What percent of patients require this item?<br>*Question relevant when: selected(${q101_C6_full_list} , '1')*<br>*Response constrained to: .>0 and .<=100 or .=999*	
q201_C6b *(required)*	q201_C6b. How many units of this item are required per patient (throughout the entire treatment provided for complication)?<br>*Smallest unit is milliliter (ml)*<br>*Question relevant when: selected(${q101_C6_full_list} , '1')*<br>*Response constrained to: .>0*	
q201_C7	q201_C7. Soap (bar)<br>*Question relevant when: selected(${q101_C7_full_list} , '1')*	
q201_C7a *(required)*	q201_C7a. What percent of patients require this item?<br>*Question relevant when: selected(${q101_C7_full_list} , '1')*<br>*Response constrained to: .>0 and .<=100 or .=999*	
q201_C7b *(required)*	q201_C7b. How many units of this item are required per patient (throughout the entire treatment provided for complication)?<br>*Smallest unit is 1 bar of soap*<br>*Question relevant when: selected(${q101_C7_full_list} , '1')*<br>*Response constrained to: .>0*	
q201_C8	q201_C8. Hand sterilizer (waterless)<br>*Question relevant when: selected(${q101_C8_full_list} , '1')*	
q201_C8a *(required)*	q201_C8a. What percent of patients require this item?<br>*Question relevant when: selected(${q101_C8_full_list} , '1')*<br>*Response constrained to: .>0 and .<=100 or .=999*	
q201_C8b *(required)*	q201_C8b. How many units of this item are required per patient (throughout the entire treatment provided for complication)?<br>*Smallest unit is milliliter (ml)*<br>*Question relevant when: selected(${q101_C8_full_list} , '1')*<br>*Response constrained to: .>0*	
A. Consumables - Incomplete Abortion (2)<br>*Group relevant when: selected(${section_two_skip_con} , '1')*		
note_201_IV_needle_syringe	***IV, needles, syringes, etc.***	
q201_C9	q201_C9. Blood giving set with needle<br>*Question relevant when: selected(${q101_C9_full_list} , '1')*	
q201_C9a *(required)*	q201_C9a. What percent of patients require this item?<br>*Question relevant when: selected(${q101_C9_full_list} , '1')*<br>*Response constrained to: .>0 and .<=100 or .=999*	
q201_C9b *(required)*	q201_C9b. How many units of this item are required per patient (throughout the entire treatment provided for complication)?<br>*Smallest unit is one set.*<br>*Question relevant when: selected(${q101_C9_full_list} , '1')*<br>*Response constrained to: .>0*	
q201_C10	q201_C10. IV cannula<br>*Question relevant when: selected(${q101_C10_full_list} , '1')*	

Field	Question	Answer
q201_C10a *(required)*	q201_C10a. What percent of patients require this item?<br>*Question relevant when: selected(${q101_C10_full_list} , '1')*<br>*Response constrained to: .>0 and .<=100 or .=999*	
q201_C10b *(required)*	q201_C10b. How many units of this item are required per patient (throughout the entire treatment provided for complication)?<br>*Smallest unit is one cannula.*<br>*Question relevant when: selected(${q101_C10_full_list} , '1')*<br>*Response constrained to: .>0*	
q201_C11	q201_C11. IV set<br>*Question relevant when: selected(${q101_C11_full_list} , '1')*	
q201_C11a *(required)*	q201_C11a. What percent of patients require this item?<br>*Question relevant when: selected(${q101_C11_full_list} , '1')*<br>*Response constrained to: .>0 and .<=100 or .=999*	
q201_C11b *(required)*	q201_C11b. How many units of this item are required per patient (throughout the entire treatment provided for complication)?<br>*Smallest unit is one set.*<br>*Question relevant when: selected(${q101_C11_full_list} , '1')*<br>*Response constrained to: .>0*	
q201_C12	q201_C12. Needle luer tip 18Gx1.1/2" (1.2x38mm)<br>*Question relevant when: selected(${q101_C12_full_list} , '1')*	
q201_C12a *(required)*	q201_C12a. What percent of patients require this item?<br>*Question relevant when: selected(${q101_C12_full_list} , '1')*<br>*Response constrained to: .>0 and .<=100 or .=999*	
q201_C12b *(required)*	q201_C12b. How many units of this item are required per patient (throughout the entire treatment provided for complication)?<br>*Smallest unit is one needle.*<br>*Question relevant when: selected(${q101_C12_full_list} , '1')*<br>*Response constrained to: .>0*	
q201_C13	q201_C13. Needle luer tip 21Gx1.1/2" (0.8x38mm)<br>*Question relevant when: selected(${q101_C13_full_list} , '1')*	
q201_C13a *(required)*	q201_C13a. What percent of patients require this item?<br>*Question relevant when: selected(${q101_C13_full_list} , '1')*<br>*Response constrained to: .>0 and .<=100 or .=999*	
q201_C13b *(required)*	q201_C13b. How many units of this item are required per patient (throughout the entire treatment provided for complication)?<br>*Smallest unit is one needle.*<br>*Question relevant when: selected(${q101_C13_full_list} , '1')*<br>*Response constrained to: .>0*	
q201_C14	q201_C14. Needle, suture, round body<br>*Question relevant when: selected(${q101_C14_full_list} , '1')*	
q201_C14a *(required)*	q201_C14a. What percent of patients require this item?<br>*Question relevant when: selected(${q101_C14_full_list} , '1')*<br>*Response constrained to: .>0 and .<=100 or .=999*	
q201_C14b *(required)*	q201_C14b. How many units of this item are required per patient (throughout the entire treatment provided for complication)?<br>*Smallest unit is one needle.*<br>*Question relevant when: selected(${q101_C14_full_list} , '1')*<br>*Response constrained to: .>0*	
q201_C15	q201_C15. Spinal needle<br>*Question relevant when: selected(${q101_C15_full_list} , '1')*	
q201_C15a *(required)*	q201_C15a. What percent of patients require this item?<br>*Question relevant when: selected(${q101_C15_full_list} , '1')*<br>*Response constrained to: .>0 and .<=100 or .=999*	
q201_C15b *(required)*	q201_C15b. How many units of this item are required per patient (throughout the entire treatment provided for complication)?<br>*Smallest unit is one needle.*<br>*Question relevant when: selected(${q101_C15_full_list} , '1')*<br>*Response constrained to: .>0*	
q201_C16	q201_C16. Syringe 10ml, with bypacked needle 21Gx1.5"<br>*Question relevant when: selected(${q101_C16_full_list} , '1')*	
q201_C16a *(required)*	q201_C16a. What percent of patients require this item?<br>*Question relevant when: selected(${q101_C16_full_list} , '1')*<br>*Response constrained to: .>0 and .<=100 or .=999*	

Field	Question	Answer
q201_C16b *(required)*	q201_C16b. How many units of this item are required per patient (throughout the entire treatment provided for complication)?<br>*Smallest unit is one syringe.*<br>*Question relevant when: selected(${q101_C16_full_list} , '1')*<br>*Response constrained to: .>0*	
q201_C17	q201_C17. Syringe 5ml, with bypacked needle 21Gx1.5"<br>*Question relevant when: selected(${q101_C17_full_list} , '1')*	
q201_C17a *(required)*	q201_C17a. What percent of patients require this item?<br>*Question relevant when: selected(${q101_C17_full_list} , '1')*<br>*Response constrained to: .>0 and .<=100 or .=999*	
q201_C17b *(required)*	q201_C17b. How many units of this item are required per patient (throughout the entire treatment provided for complication)?<br>*Smallest unit is one syringe.*<br>*Question relevant when: selected(${q101_C17_full_list} , '1')*<br>*Response constrained to: .>0*	
q201_C18	q201_C18. Syringe luer tip 10ml, no needle<br>*Question relevant when: selected(${q101_C18_full_list} , '1')*	
q201_C18a *(required)*	q201_C18a. What percent of patients require this item?<br>*Question relevant when: selected(${q101_C18_full_list} , '1')*<br>*Response constrained to: .>0 and .<=100 or .=999*	
q201_C18b *(required)*	q201_C18b. How many units of this item are required per patient (throughout the entire treatment provided for complication)?<br>*Smallest unit is one syringe.*<br>*Question relevant when: selected(${q101_C18_full_list} , '1')*<br>*Response constrained to: .>0*	
q201_C19	q201_C19. Syringe luer tip 5ml, no needle<br>*Question relevant when: selected(${q101_C19_full_list} , '1')*	
q201_C19a *(required)*	q201_C19a. What percent of patients require this item?<br>*Question relevant when: selected(${q101_C19_full_list} , '1')*<br>*Response constrained to: .>0 and .<=100 or .=999*	
q201_C19b *(required)*	q201_C19b. How many units of this item are required per patient (throughout the entire treatment provided for complication)?<br>*Smallest unit is one syringe.*<br>*Question relevant when: selected(${q101_C19_full_list} , '1')*<br>*Response constrained to: .>0*	
q201_C20	q201_C20. Venous catheter G18 + injection port and lock tip<br>*Question relevant when: selected(${q101_C20_full_list} , '1')*	
q201_C20a *(required)*	q201_C20a. What percent of patients require this item?<br>*Question relevant when: selected(${q101_C20_full_list} , '1')*<br>*Response constrained to: .>0 and .<=100 or .=999*	
q201_C20b *(required)*	q201_C20b. How many units of this item are required per patient (throughout the entire treatment provided for complication)?<br>*Smallest unit is one catheter.*<br>*Question relevant when: selected(${q101_C20_full_list} , '1')*<br>*Response constrained to: .>0*	
A. Consumables - Incomplete Abortion (3)<br>*Group relevant when: selected(${section_two_skip_con} , '1')*		
note_201_bandages_etc	***Bandages, absorbants, wound care, etc.***	
q201_C21	q201_C21. Adhesive tape<br>*Question relevant when: selected(${q101_C21_full_list} , '1')*	
q201_C21a *(required)*	q201_C21a. What percent of patients require this item?<br>*Question relevant when: selected(${q101_C21_full_list} , '1')*<br>*Response constrained to: .>0 and .<=100 or .=999*	
q201_C21b *(required)*	q201_C21b. How many units of this item are required per patient (throughout the entire treatment provided for complication)?<br>*Smallest unit is one roll of tape.*<br>*Question relevant when: selected(${q101_C21_full_list} , '1')*<br>*Response constrained to: .>0*	
q201_C22	q201_C22. Cotton swab<br>*Question relevant when: selected(${q101_C22_full_list} , '1')*	
q201_C22a *(required)*	q201_C22a. What percent of patients require this item?<br>*Question relevant when: selected(${q101_C22_full_list} , '1')*<br>*Response constrained to: .>0 and .<=100 or .=999*	

Field	Question	Answer
q201_C22b *(required)*	q201_C22b. How many units of this item are required per patient (throughout the entire treatment provided for complication)?<br>*Smallest unit is one swab.*<br>*Question relevant when: selected(${q101_C22_full_list} , '1')*<br>*Response constrained to: .>0*	
q201_C23	q201_C23. Dressing pad absorbent sterile<br>*Question relevant when: selected(${q101_C23_full_list} , '1')*	
q201_C23a *(required)*	q201_C23a. What percent of patients require this item?<br>*Question relevant when: selected(${q101_C23_full_list} , '1')*<br>*Response constrained to: .>0 and .<=100 or .=999*	
q201_C23b *(required)*	q201_C23b. How many units of this item are required per patient (throughout the entire treatment provided for complication)?<br>*Smallest unit is one pad.*<br>*Question relevant when: selected(${q101_C23_full_list} , '1')*<br>*Response constrained to: .>0*	
q201_C24	q201_C24. Gauze compress, non sterile<br>*Question relevant when: selected(${q101_C24_full_list} , '1')*	
q201_C24a *(required)*	q201_C24a. What percent of patients require this item?<br>*Question relevant when: selected(${q101_C24_full_list} , '1')*<br>*Response constrained to: .>0 and .<=100 or .=999*	
q201_C24b *(required)*	q201_C24b. How many units of this item are required per patient (throughout the entire treatment provided for complication)?<br>*Smallest unit is one compress.*<br>*Question relevant when: selected(${q101_C24_full_list} , '1')*<br>*Response constrained to: .>0*	
q201_C25	q201_C25. Gauze compress, sterile<br>*Question relevant when: selected(${q101_C25_full_list} , '1')*	
q201_C25a *(required)*	q201_C25a. What percent of patients require this item?<br>*Question relevant when: selected(${q101_C25_full_list} , '1')*<br>*Response constrained to: .>0 and .<=100 or .=999*	
q201_C25b *(required)*	q201_C25b. How many units of this item are required per patient (throughout the entire treatment provided for complication)?<br>*Smallest unit is one compress.*<br>*Question relevant when: selected(${q101_C25_full_list} , '1')*<br>*Response constrained to: .>0*	
q201_C26	q201_C26. Linen saver<br>*Question relevant when: selected(${q101_C26_full_list} , '1')*	
q201_C26a *(required)*	q201_C26a. What percent of patients require this item?<br>*Question relevant when: selected(${q101_C26_full_list} , '1')*<br>*Response constrained to: .>0 and .<=100 or .=999*	
q201_C26b *(required)*	q201_C26b. How many units of this item are required per patient (throughout the entire treatment provided for complication)?<br>*Smallest unit is one saver (absorbant sheet).*<br>*Question relevant when: selected(${q101_C26_full_list} , '1')*<br>*Response constrained to: .>0*	
q201_C27	q201_C27. Sanitary Pad<br>*Question relevant when: selected(${q101_C27_full_list} , '1')*	
q201_C27a *(required)*	q201_C27a. What percent of patients require this item?<br>*Question relevant when: selected(${q101_C27_full_list} , '1')*<br>*Response constrained to: .>0 and .<=100 or .=999*	
q201_C27b *(required)*	q201_C27b. How many units of this item are required per patient (throughout the entire treatment provided for complication)?<br>*Smallest unit is one pad.*<br>*Question relevant when: selected(${q101_C27_full_list} , '1')*<br>*Response constrained to: .>0*	
q201_C28	q201_C28. Surgical plaster<br>*Question relevant when: selected(${q101_C28_full_list} , '1')*	
q201_C28a *(required)*	q201_C28a. What percent of patients require this item?<br>*Question relevant when: selected(${q101_C28_full_list} , '1')*<br>*Response constrained to: .>0 and .<=100 or .=999*	
q201_C28b *(required)*	q201_C28b. How many units of this item are required per patient (throughout the entire treatment provided for complication)?<br>*Smallest unit is one plaster.*<br>*Question relevant when: selected(${q101_C28_full_list} , '1')*	

Field	Question	Answer
	Response constrained to: .>0	

A. Consumables - Incomplete Abortion (4)

Group relevant when: selected(${section_two_skip_con} , '1')

Field	Question	Answer
note_201_protective_etc	***Protective supplies***	
q201_C29	q201_C29. Apron (disposable)<br>*Question relevant when: selected(${q101_C29_full_list} , '1')*	
q201_C29a *(required)*	q201_C29a. What percent of patients require this item?<br>*Question relevant when: selected(${q101_C29_full_list} , '1')*<br>*Response constrained to: .>0 and .<=100 or .=999*	
q201_C29b *(required)*	q201_C29b. How many units of this item are required per patient (throughout the entire treatment provided for complication)?<br>*Smallest unit is one apron.*<br>*Question relevant when: selected(${q101_C29_full_list} , '1')*<br>*Response constrained to: .>0*	
q201_C30	q201_C30. Gown (disposable)<br>*Question relevant when: selected(${q101_C30_full_list} , '1')*	
q201_C30a *(required)*	q201_C30a. What percent of patients require this item?<br>*Question relevant when: selected(${q101_C30_full_list} , '1')*<br>*Response constrained to: .>0 and .<=100 or .=999*	
q201_C30b *(required)*	q201_C30b. How many units of this item are required per patient (throughout the entire treatment provided for complication)?<br>*Smallest unit is one gown.*<br>*Question relevant when: selected(${q101_C30_full_list} , '1')*<br>*Response constrained to: .>0*	
q201_C31	q201_C31. Latex examination glove, without powder<br>*Question relevant when: selected(${q101_C31_full_list} , '1')*	
q201_C31a *(required)*	q201_C31a. What percent of patients require this item?<br>*Question relevant when: selected(${q101_C31_full_list} , '1')*<br>*Response constrained to: .>0 and .<=100 or .=999*	
q201_C31b *(required)*	q201_C31b. How many units of this item are required per patient (throughout the entire treatment provided for complication)?<br>*Smallest unit is one glove.*<br>*Question relevant when: selected(${q101_C31_full_list} , '1')*<br>*Response constrained to: .>0*	
q201_C32	q201_C32. Latex examination glove, with powder<br>*Question relevant when: selected(${q101_C32_full_list} , '1')*	
q201_C32a *(required)*	q201_C32a. What percent of patients require this item?<br>*Question relevant when: selected(${q101_C32_full_list} , '1')*<br>*Response constrained to: .>0 and .<=100 or .=999*	
q201_C32b *(required)*	q201_C32b. How many units of this item are required per patient (throughout the entire treatment provided for complication)?<br>*Smallest unit is one glove.*<br>*Question relevant when: selected(${q101_C32_full_list} , '1')*<br>*Response constrained to: .>0*	
q201_C33	q201_C33. Non-latex (e.g. nitrile) examination glove<br>*Question relevant when: selected(${q101_C33_full_list} , '1')*	
q201_C33a *(required)*	q201_C33a. What percent of patients require this item?<br>*Question relevant when: selected(${q101_C33_full_list} , '1')*<br>*Response constrained to: .>0 and .<=100 or .=999*	
q201_C33b *(required)*	q201_C33b. How many units of this item are required per patient (throughout the entire treatment provided for complication)?<br>*Smallest unit is one glove.*<br>*Question relevant when: selected(${q101_C33_full_list} , '1')*<br>*Response constrained to: .>0*	
q201_C34	q201_C34. Sterile surgical glove<br>*Question relevant when: selected(${q101_C34_full_list} , '1')*	
q201_C34a *(required)*	q201_C34a. What percent of patients require this item?<br>*Question relevant when: selected(${q101_C34_full_list} , '1')*<br>*Response constrained to: .>0 and .<=100 or .=999*	
q201_C34b *(required)*	q201_C34b. How many units of this item are required per patient (throughout the entire treatment provided for complication)?<br>*Smallest unit is one glove.*<br>*Question relevant when: selected(${q101_C34_full_list} , '1')*<br>*Response constrained to: .>0*	
q201_C35	q201_C35. Mask (disposable)	

Field	Question	Answer
	Question relevant when: selected(${q101_C35_full_list} , '1')	
q201_C35a *(required)*	q201_C35a. What percent of patients require this item?<br>*Question relevant when: selected(${q101_C35_full_list} , '1')*<br>*Response constrained to: .>0 and .<=100 or .=999*	
q201_C35b *(required)*	q201_C35b. How many units of this item are required per patient (throughout the entire treatment provided for complication)?<br>*Smallest unit is one mask.*<br>*Question relevant when: selected(${q101_C35_full_list} , '1')*<br>*Response constrained to: .>0*	
q201_C36	q201_C36. Surgical cap<br>*Question relevant when: selected(${q101_C36_full_list} , '1')*	
q201_C36a *(required)*	q201_C36a. What percent of patients require this item?<br>*Question relevant when: selected(${q101_C36_full_list} , '1')*<br>*Response constrained to: .>0 and .<=100 or .=999*	
q201_C36b *(required)*	q201_C36b. How many units of this item are required per patient (throughout the entire treatment provided for complication)?<br>*Smallest unit is one cap.*<br>*Question relevant when: selected(${q101_C36_full_list} , '1')*<br>*Response constrained to: .>0*	
A. Consumables - Incomplete Abortion (5)<br>*Group relevant when: selected(${section_two_skip_con} , '1')*		
note_201_surgery_etc	***Surgery supplies***	
q201_C37	q201_C37. Scalpel blade, single use<br>*Question relevant when: selected(${q101_C37_full_list} , '1')*	
q201_C37a *(required)*	q201_C37a. What percent of patients require this item?<br>*Question relevant when: selected(${q101_C37_full_list} , '1')*<br>*Response constrained to: .>0 and .<=100 or .=999*	
q201_C37b *(required)*	q201_C37b. How many units of this item are required per patient (throughout the entire treatment provided for complication)?<br>*Smallest unit is one blade.*<br>*Question relevant when: selected(${q101_C37_full_list} , '1')*<br>*Response constrained to: .>0*	
q201_C38	q201_C38. Surgical drain<br>*Question relevant when: selected(${q101_C38_full_list} , '1')*	
q201_C38a *(required)*	q201_C38a. What percent of patients require this item?<br>*Question relevant when: selected(${q101_C38_full_list} , '1')*<br>*Response constrained to: .>0 and .<=100 or .=999*	
q201_C38b *(required)*	q201_C38b. How many units of this item are required per patient (throughout the entire treatment provided for complication)?<br>*Smallest unit is one drain.*<br>*Question relevant when: selected(${q101_C38_full_list} , '1')*<br>*Response constrained to: .>0*	
q201_C39	q201_C39. Suture string, chromic catgut (0)<br>*Question relevant when: selected(${q101_C39_full_list} , '1')*	
q201_C39a *(required)*	q201_C39a. What percent of patients require this item?<br>*Question relevant when: selected(${q101_C39_full_list} , '1')*<br>*Response constrained to: .>0 and .<=100 or .=999*	
q201_C39b *(required)*	q201_C39b. How many units of this item are required per patient (throughout the entire treatment provided for complication)?<br>*Smallest unit is one suture set.*<br>*Question relevant when: selected(${q101_C39_full_list} , '1')*<br>*Response constrained to: .>0*	
q201_C40	q201_C40. Suture string, silk (non absorbable and non-synthetic)<br>*Question relevant when: selected(${q101_C40_full_list} , '1')*	
q201_C40a *(required)*	q201_C40a. What percent of patients require this item?<br>*Question relevant when: selected(${q101_C40_full_list} , '1')*<br>*Response constrained to: .>0 and .<=100 or .=999*	
q201_C40b *(required)*	q201_C40b. How many units of this item are required per patient (throughout the entire treatment provided for complication)?<br>*Smallest unit is one suture set.*<br>*Question relevant when: selected(${q101_C40_full_list} , '1')*<br>*Response constrained to: .>0*	
q201_C41	q201_C41. Suture string, vicryl, no needle included<br>*Question relevant when: selected(${q101_C41_full_list} , '1')*	

Field	Question	Answer
q201_C41a *(required)*	q201_C41a. What percent of patients require this item?<br>*Question relevant when: selected(${q101_C41_full_list} , '1')*<br>*Response constrained to: .>0 and .<=100 or .=999*	
q201_C41b *(required)*	q201_C41b. How many units of this item are required per patient (throughout the entire treatment provided for complication)?<br>*Smallest unit is one suture set.*<br>*Question relevant when: selected(${q101_C41_full_list} , '1')*<br>*Response constrained to: .>0*	
q201_C42	q201_C42. Suture string, vicryl with needle<br>*Question relevant when: selected(${q101_C42_full_list} , '1')*	
q201_C42a *(required)*	q201_C42a. What percent of patients require this item?<br>*Question relevant when: selected(${q101_C42_full_list} , '1')*<br>*Response constrained to: .>0 and .<=100 or .=999*	
q201_C42b *(required)*	q201_C42b. How many units of this item are required per patient (throughout the entire treatment provided for complication)?<br>*Smallest unit is one suture set with needle..*<br>*Question relevant when: selected(${q101_C42_full_list} , '1')*<br>*Response constrained to: .>0*	
A. Consumables - Incomplete Abortion (6)<br>*Group relevant when: selected(${section_two_skip_con} , '1')*		
note_201_urine_etc	***Urine, bladder***	
q201_C43	q201_C43. Bladder/urinary catheter<br>*Question relevant when: selected(${q101_C43_full_list} , '1')*	
q201_C43a *(required)*	q201_C43a. What percent of patients require this item?<br>*Question relevant when: selected(${q101_C43_full_list} , '1')*<br>*Response constrained to: .>0 and .<=100 or .=999*	
q201_C43b *(required)*	q201_C43b. How many units of this item are required per patient (throughout the entire treatment provided for complication)?<br>*Smallest unit is one catheter.*<br>*Question relevant when: selected(${q101_C43_full_list} , '1')*<br>*Response constrained to: .>0*	
q201_C44	q201_C44. Foley's catheter<br>*Question relevant when: selected(${q101_C44_full_list} , '1')*	
q201_C44a *(required)*	q201_C44a. What percent of patients require this item?<br>*Question relevant when: selected(${q101_C44_full_list} , '1')*<br>*Response constrained to: .>0 and .<=100 or .=999*	
q201_C44b *(required)*	q201_C44b. How many units of this item are required per patient (throughout the entire treatment provided for complication)?<br>*Smallest unit is one catheter.*<br>*Question relevant when: selected(${q101_C44_full_list} , '1')*<br>*Response constrained to: .>0*	
q201_C45	q201_C45. Urine bag with valve and drain<br>*Question relevant when: selected(${q101_C45_full_list} , '1')*	
q201_C45a *(required)*	q201_C45a. What percent of patients require this item?<br>*Question relevant when: selected(${q101_C45_full_list} , '1')*<br>*Response constrained to: .>0 and .<=100 or .=999*	
q201_C45b *(required)*	q201_C45b. How many units of this item are required per patient (throughout the entire treatment provided for complication)?<br>*Smallest unit is one bag.*<br>*Question relevant when: selected(${q101_C45_full_list} , '1')*<br>*Response constrained to: .>0*	
A. Consumables - Incomplete Abortion (7)<br>*Group relevant when: selected(${section_two_skip_con} , '1')*		
note_201_other_etc	***Other supplies***	
q201_C46	q201_C46. Silicone oil for lubrication of MVA aspirator<br>*Question relevant when: selected(${q101_C46_full_list} , '1')*	
q201_C46a *(required)*	q201_C46a. What percent of patients require this item?<br>*Question relevant when: selected(${q101_C46_full_list} , '1')*<br>*Response constrained to: .>0 and .<=100 or .=999*	
q201_C46b *(required)*	q201_C46b. How many units of this item are required per patient (throughout the entire treatment provided for complication)?<br>*Smallest unit is mililiter (ml)*<br>*Question relevant when: selected(${q101_C46_full_list} , '1')*	

Field	Question	Answer
	Response constrained to: .>0	
q201_C47	q201_C47. Speculum, Bivalve/Cuscos (reusable)<br>*Question relevant when: selected(${q101_C47_full_list} , '1')*	
q201_C47a *(required)*	q201_C47a. What percent of patients require this item?<br>*Question relevant when: selected(${q101_C47_full_list} , '1')*<br>*Response constrained to: .>0 and .<=100 or .=999*	
q201_C47b *(required)*	q201_C47b. How many units of this item are required per patient (throughout the entire treatment provided for complication)?<br>*Smallest unit is one speculum.*<br>*Question relevant when: selected(${q101_C47_full_list} , '1')*<br>*Response constrained to: .>0*	
q201_C48	q201_C48. Speculum, Sim's (disposable)<br>*Question relevant when: selected(${q101_C48_full_list} , '1')*	
q201_C48a *(required)*	q201_C48a. What percent of patients require this item?<br>*Question relevant when: selected(${q101_C48_full_list} , '1')*<br>*Response constrained to: .>0 and .<=100 or .=999*	
q201_C48b *(required)*	q201_C48b. How many units of this item are required per patient (throughout the entire treatment provided for complication)?<br>*Smallest unit is one speculum.*<br>*Question relevant when: selected(${q101_C48_full_list} , '1')*<br>*Response constrained to: .>0*	
A. Consumables - Incomplete Abortion (8)<br>*Group relevant when: selected(${section_two_skip_con} , '1')*		
note_201_suggest_other_etc	***Other supplies - suggestions?***	
q201_C49	q201_C49. Other consumable 1: "[q101_C49_full_list_other]"<br>*Question relevant when: selected(${q101_C49_full_list} , '1')*	
q201_C49a *(required)*	q201_C49a. What percent of patients require this item?<br>*Question relevant when: selected(${q101_C49_full_list} , '1')*<br>*Response constrained to: .>=0 and .<=100 or .=999*	
q201_C49b *(required)*	q201_C49b. How many units of this item are required per patient (throughout the entire treatment provided for complication)?<br>*Question relevant when: selected(${q101_C49_full_list} , '1')*<br>*Response constrained to: .>=0*	
q201_C50	q201_C50. Other consumable 2: "[q101_C50_full_list_other]"<br>*Question relevant when: selected(${q101_C50_full_list} , '1')*	
q201_C50a *(required)*	q201_C50a. What percent of patients require this item?<br>*Question relevant when: selected(${q101_C50_full_list} , '1')*<br>*Response constrained to: .>=0 and .<=100 or .=999*	
q201_C50b *(required)*	q201_C50b. How many units of this item are required per patient (throughout the entire treatment provided for complication)?<br>*Question relevant when: selected(${q101_C50_full_list} , '1')*<br>*Response constrained to: .>=0*	
q201_C51	q201_C51. Other consumable 3: "[q101_C51_full_list_other]"<br>*Question relevant when: selected(${q101_C51_full_list} , '1')*	
q201_C51a *(required)*	q201_C51a. What percent of patients require this item?<br>*Question relevant when: selected(${q101_C51_full_list} , '1')*<br>*Response constrained to: .>=0 and .<=100 or .=999*	
q201_C51b *(required)*	q201_C51b. How many units of this item are required per patient (throughout the entire treatment provided for complication)?<br>*Question relevant when: selected(${q101_C51_full_list} , '1')*<br>*Response constrained to: .>=0*	
q201_C52	q201_C52. Other consumable 4: "[q101_C52_full_list_other]"<br>*Question relevant when: selected(${q101_C52_full_list} , '1')*	
q201_C52a *(required)*	q201_C52a. What percent of patients require this item?<br>*Question relevant when: selected(${q101_C52_full_list} , '1')*<br>*Response constrained to: .>=0 and .<=100 or .=999*	
q201_C52b *(required)*	q201_C52b. How many units of this item are required per patient (throughout the entire treatment provided for complication)?<br>*Question relevant when: selected(${q101_C52_full_list} , '1')*<br>*Response constrained to: .>=0*	
q201_C53	q201_C53. Other consumable 5: "[q101_C53_full_list_other]"<br>*Question relevant when: selected(${q101_C53_full_list} , '1')*	
q201_C53a *(required)*	q201_C53a. What percent of patients require this item?	

Field	Question	Answer
	Question relevant when: selected(${q101_C53_full_list} , '1')<br>*Response constrained to: .>=0 and .<=100 or .=999*	

q201_C53b *(required)*	q201_C53b. How many units of this item are required per patient (throughout the entire treatment provided for complication)?<br>*Question relevant when: selected(${q101_C53_full_list} , '1')*<br>*Response constrained to: .>=0*	
group_section_three_intro		
section3_start	**SECTION III. SEPSIS - USAGE OF ALL ITEMS**	
section_three_skip_con *(required)*	INTERVIEWER: WOULD YOU LIKE TO COMPLETE THIS SECTION NOW OR SKIP THIS SECTION AND RETURN TO IT LATER?<br>*You may need to skip if the participant has indicated that s/he cannot answer the questions in this section.*	1 Do not skip, complete this section now.<br>2 Skip and come back to this section later.
group_section_three_introB<br>*Group relevant when: selected(${section_three_skip_con} , '1')*		
section3_start2	In this section of the interview, we will review all of the items that you said are used for management of sepsis. For each item that is used, I'm going to ask questions on how many women need it and how much of it is used.	
section3_start3	INTERVIEWER: ENTER WHOLE NUMBERS OR DECIMALS. DO NOT TYPE PERCENT SIGNS. ENTER 999 FOR ANY THAT ARE UNKNOWN.	
A. Consumables - Sepsis (1)<br>*Group relevant when: selected(${section_three_skip_con} , '1')*		
note_301_cleaning	***Cleaning, disinfectants, sterilization, etc.***	
q301_C1	q301_C1. Alcohol, denatured 70%<br>*Question relevant when: selected(${q101_C1_full_list} , '2')*	
q301_C1a *(required)*	q301_C1a. What percent of patients require this item?<br>*Question relevant when: selected(${q101_C1_full_list} , '2')*<br>*Response constrained to: .>0 and .<=100 or .=999*	
q301_C1b *(required)*	q301_C1b. How many units of this item are required per patient (throughout the entire treatment provided for complication)?<br>*Smallest unit is mililiter (ml)*<br>*Question relevant when: selected(${q101_C1_full_list} , '2')*<br>*Response constrained to: .>0*	
q301_C2	q301_C2. Chlorine (JIK) or Glutaraldehyde solution<br>*Question relevant when: selected(${q101_C2_full_list} , '2')*	
q301_C2a *(required)*	q301_C2a. What percent of patients require this item?<br>*Question relevant when: selected(${q101_C2_full_list} , '2')*<br>*Response constrained to: .>0 and .<=100 or .=999*	
q301_C2b *(required)*	q301_C2b. How many units of this item are required per patient (throughout the entire treatment provided for complication)?<br>*Smallest unit is mililiter (ml)*<br>*Question relevant when: selected(${q101_C2_full_list} , '2')*<br>*Response constrained to: .>0*	
q301_C3	q301_C3. Clean water<br>*Question relevant when: selected(${q101_C3_full_list} , '2')*	
q301_C3a *(required)*	q301_C3a. What percent of patients require this item?<br>*Question relevant when: selected(${q101_C3_full_list} , '2')*<br>*Response constrained to: .>0 and .<=100 or .=999*	
q301_C3b *(required)*	q301_C3b. How many units of this item are required per patient (throughout the entire treatment provided for complication)?<br>*Smallest unit is litre (ltr)*<br>*Question relevant when: selected(${q101_C3_full_list} , '2')*<br>*Response constrained to: .>0*	
q301_C4	q301_C4. Dettol solution or other water-based antiseptic<br>*Question relevant when: selected(${q101_C4_full_list} , '2')*	
q301_C4a *(required)*	q301_C4a. What percent of patients require this item?<br>*Question relevant when: selected(${q101_C4_full_list} , '2')*<br>*Response constrained to: .>0 and .<=100 or .=999*	
q301_C4b *(required)*	q301_C4b. How many units of this item are required per patient (throughout the entire treatment provided for complication)?<br>*Smallest unit is mililiter (ml)*<br>*Question relevant when: selected(${q101_C4_full_list} , '2')*<br>*Response constrained to: .>0*	
q301_C5	q301_C5. Cetrimide 15% ("Savlon")<br>*Question relevant when: selected(${q101_C5_full_list} , '2')*	
q301_C5a *(required)*	q301_C5a. What percent of patients require this item?	

Field	Question	Answer
	Question relevant when: selected(${q101_C5_full_list} , '2')<br>*Response constrained to: .>0 and .<=100 or .=999*	

q301_C5b *(required)*	q301_C5b. How many units of this item are required per patient (throughout the entire treatment provided for complication)?<br>*Smallest unit is mililiter (ml)*<br>*Question relevant when: selected(${q101_C5_full_list} , '2')*<br>*Response constrained to: .>0*	
q301_C6	q301_C6. Hand wash (liquid)<br>*Question relevant when: selected(${q101_C6_full_list} , '2')*	
q301_C6a *(required)*	q301_C6a. What percent of patients require this item?<br>*Question relevant when: selected(${q101_C6_full_list} , '2')*<br>*Response constrained to: .>0 and .<=100 or .=999*	
q301_C6b *(required)*	q301_C6b. How many units of this item are required per patient (throughout the entire treatment provided for complication)?<br>*Smallest unit is mililiter (ml)*<br>*Question relevant when: selected(${q101_C6_full_list} , '2')*<br>*Response constrained to: .>0*	
q301_C7	q301_C7. Soap (bar)<br>*Question relevant when: selected(${q101_C7_full_list} , '2')*	
q301_C7a *(required)*	q301_C7a. What percent of patients require this item?<br>*Question relevant when: selected(${q101_C7_full_list} , '2')*<br>*Response constrained to: .>0 and .<=100 or .=999*	
q301_C7b *(required)*	q301_C7b. How many units of this item are required per patient (throughout the entire treatment provided for complication)?<br>*Smallest unit is 1 bar of soap*<br>*Question relevant when: selected(${q101_C7_full_list} , '2')*<br>*Response constrained to: .>0*	
q301_C8	q301_C8. Hand sterilizer (waterless)<br>*Question relevant when: selected(${q101_C8_full_list} , '2')*	
q301_C8a *(required)*	q301_C8a. What percent of patients require this item?<br>*Question relevant when: selected(${q101_C8_full_list} , '2')*<br>*Response constrained to: .>0 and .<=100 or .=999*	
q301_C8b *(required)*	q301_C8b. How many units of this item are required per patient (throughout the entire treatment provided for complication)?<br>*Smallest unit is mililiter (ml)*<br>*Question relevant when: selected(${q101_C8_full_list} , '2')*<br>*Response constrained to: .>0*	
A. Consumables - Sepsis (2)<br>*Group relevant when: selected(${section_three_skip_con} , '1')*		
note_301_IV_needle_syringe	***IV, needles, syringes, etc.***	
q301_C9	q301_C9. Blood giving set with needle<br>*Question relevant when: selected(${q101_C9_full_list} , '2')*	
q301_C9a *(required)*	q301_C9a. What percent of patients require this item?<br>*Question relevant when: selected(${q101_C9_full_list} , '2')*<br>*Response constrained to: .>0 and .<=100 or .=999*	
q301_C9b *(required)*	q301_C9b. How many units of this item are required per patient (throughout the entire treatment provided for complication)?<br>*Smallest unit is one set.*<br>*Question relevant when: selected(${q101_C9_full_list} , '2')*<br>*Response constrained to: .>0*	
q301_C10	q301_C10. IV cannula<br>*Question relevant when: selected(${q101_C10_full_list} , '2')*	
q301_C10a *(required)*	q301_C10a. What percent of patients require this item?<br>*Question relevant when: selected(${q101_C10_full_list} , '2')*<br>*Response constrained to: .>0 and .<=100 or .=999*	
q301_C10b *(required)*	q301_C10b. How many units of this item are required per patient (throughout the entire treatment provided for complication)?<br>*Smallest unit is one cannula.*<br>*Question relevant when: selected(${q101_C10_full_list} , '2')*<br>*Response constrained to: .>0*	
q301_C11	q301_C11. IV set<br>*Question relevant when: selected(${q101_C11_full_list} , '2')*	
q301_C11a *(required)*	q301_C11a. What percent of patients require this item?<br>*Question relevant when: selected(${q101_C11_full_list} , '2')*<br>*Response constrained to: .>0 and .<=100 or .=999*	

Field	Question	Answer
q301_C11b *(required)*	q301_C11b. How many units of this item are required per patient (throughout the entire treatment provided for complication)?<br>*Smallest unit is one set.*<br>*Question relevant when: selected(${q101_C11_full_list} , '2')*<br>*Response constrained to: .>0*	
q301_C12	q301_C12. Needle luer tip 18Gx1.1/2" (1.2x38mm)<br>*Question relevant when: selected(${q101_C12_full_list} , '2')*	
q301_C12a *(required)*	q301_C12a. What percent of patients require this item?<br>*Question relevant when: selected(${q101_C12_full_list} , '2')*<br>*Response constrained to: .>0 and .<=100 or .=999*	
q301_C12b *(required)*	q301_C12b. How many units of this item are required per patient (throughout the entire treatment provided for complication)?<br>*Smallest unit is one needle.*<br>*Question relevant when: selected(${q101_C12_full_list} , '2')*<br>*Response constrained to: .>0*	
q301_C13	q301_C13. Needle luer tip 21Gx1.1/2" (0.8x38mm)<br>*Question relevant when: selected(${q101_C13_full_list} , '2')*	
q301_C13a *(required)*	q301_C13a. What percent of patients require this item?<br>*Question relevant when: selected(${q101_C13_full_list} , '2')*<br>*Response constrained to: .>0 and .<=100 or .=999*	
q301_C13b *(required)*	q301_C13b. How many units of this item are required per patient (throughout the entire treatment provided for complication)?<br>*Smallest unit is one needle.*<br>*Question relevant when: selected(${q101_C13_full_list} , '2')*<br>*Response constrained to: .>0*	
q301_C14	q301_C14. Needle, suture, round body<br>*Question relevant when: selected(${q101_C14_full_list} , '2')*	
q301_C14a *(required)*	q301_C14a. What percent of patients require this item?<br>*Question relevant when: selected(${q101_C14_full_list} , '2')*<br>*Response constrained to: .>0 and .<=100 or .=999*	
q301_C14b *(required)*	q301_C14b. How many units of this item are required per patient (throughout the entire treatment provided for complication)?<br>*Smallest unit is one needle.*<br>*Question relevant when: selected(${q101_C14_full_list} , '2')*<br>*Response constrained to: .>0*	
q301_C15	q301_C15. Spinal needle<br>*Question relevant when: selected(${q101_C15_full_list} , '2')*	
q301_C15a *(required)*	q301_C15a. What percent of patients require this item?<br>*Question relevant when: selected(${q101_C15_full_list} , '2')*<br>*Response constrained to: .>0 and .<=100 or .=999*	
q301_C15b *(required)*	q301_C15b. How many units of this item are required per patient (throughout the entire treatment provided for complication)?<br>*Smallest unit is one needle.*<br>*Question relevant when: selected(${q101_C15_full_list} , '2')*<br>*Response constrained to: .>0*	
q301_C16	q301_C16. Syringe 10ml, with bypacked needle 21Gx1.5"<br>*Question relevant when: selected(${q101_C16_full_list} , '2')*	
q301_C16a *(required)*	q301_C16a. What percent of patients require this item?<br>*Question relevant when: selected(${q101_C16_full_list} , '2')*<br>*Response constrained to: .>0 and .<=100 or .=999*	
q301_C16b *(required)*	q301_C16b. How many units of this item are required per patient (throughout the entire treatment provided for complication)?<br>*Smallest unit is one syringe.*<br>*Question relevant when: selected(${q101_C16_full_list} , '2')*<br>*Response constrained to: .>0*	
q301_C17	q301_C17. Syringe 5ml, with bypacked needle 21Gx1.5"<br>*Question relevant when: selected(${q101_C17_full_list} , '2')*	
q301_C17a *(required)*	q301_C17a. What percent of patients require this item?<br>*Question relevant when: selected(${q101_C17_full_list} , '2')*<br>*Response constrained to: .>0 and .<=100 or .=999*	
q301_C17b *(required)*	q301_C17b. How many units of this item are required per patient (throughout the entire treatment provided for complication)?<br>*Smallest unit is one syringe.*<br>*Question relevant when: selected(${q101_C17_full_list} , '2')*	

Field	Question	Answer
	Response constrained to: .>0	

q301_C18	q301_C18. Syringe luer tip 10ml, no needle<br>*Question relevant when: selected(${q101_C18_full_list} , '2')*	
q301_C18a *(required)*	q301_C18a. What percent of patients require this item?<br>*Question relevant when: selected(${q101_C18_full_list} , '2')*<br>*Response constrained to: .>0 and .<=100 or .=999*	
q301_C18b *(required)*	q301_C18b. How many units of this item are required per patient (throughout the entire treatment provided for complication)?<br>*Smallest unit is one syringe.*<br>*Question relevant when: selected(${q101_C18_full_list} , '2')*<br>*Response constrained to: .>0*	
q301_C19	q301_C19. Syringe luer tip 5ml, no needle<br>*Question relevant when: selected(${q101_C19_full_list} , '2')*	
q301_C19a *(required)*	q301_C19a. What percent of patients require this item?<br>*Question relevant when: selected(${q101_C19_full_list} , '2')*<br>*Response constrained to: .>0 and .<=100 or .=999*	
q301_C19b *(required)*	q301_C19b. How many units of this item are required per patient (throughout the entire treatment provided for complication)?<br>*Smallest unit is one syringe.*<br>*Question relevant when: selected(${q101_C19_full_list} , '2')*<br>*Response constrained to: .>0*	
q301_C20	q301_C20. Venous catheter G18 + injection port and lock tip<br>*Question relevant when: selected(${q101_C20_full_list} , '2')*	
q301_C20a *(required)*	q301_C20a. What percent of patients require this item?<br>*Question relevant when: selected(${q101_C20_full_list} , '2')*<br>*Response constrained to: .>0 and .<=100 or .=999*	
q301_C20b *(required)*	q301_C20b. How many units of this item are required per patient (throughout the entire treatment provided for complication)?<br>*Smallest unit is one catheter.*<br>*Question relevant when: selected(${q101_C20_full_list} , '2')*<br>*Response constrained to: .>0*	
A. Consumables - Sepsis (3)<br>*Group relevant when: selected(${section_three_skip_con} , '1')*		
note_301_bandages_etc	***Bandages, absorbants, wound care, etc.***	
q301_C21	q301_C21. Adhesive tape<br>*Question relevant when: selected(${q101_C21_full_list} , '2')*	
q301_C21a *(required)*	q301_C21a. What percent of patients require this item?<br>*Question relevant when: selected(${q101_C21_full_list} , '2')*<br>*Response constrained to: .>0 and .<=100 or .=999*	
q301_C21b *(required)*	q301_C21b. How many units of this item are required per patient (throughout the entire treatment provided for complication)?<br>*Smallest unit is one roll of tape.*<br>*Question relevant when: selected(${q101_C21_full_list} , '2')*<br>*Response constrained to: .>0*	
q301_C22	q301_C22. Cotton swab<br>*Question relevant when: selected(${q101_C22_full_list} , '2')*	
q301_C22a *(required)*	q301_C22a. What percent of patients require this item?<br>*Question relevant when: selected(${q101_C22_full_list} , '2')*<br>*Response constrained to: .>0 and .<=100 or .=999*	
q301_C22b *(required)*	q301_C22b. How many units of this item are required per patient (throughout the entire treatment provided for complication)?<br>*Smallest unit is one swab.*<br>*Question relevant when: selected(${q101_C22_full_list} , '2')*<br>*Response constrained to: .>0*	
q301_C23	q301_C23. Dressing pad absorbent sterile<br>*Question relevant when: selected(${q101_C23_full_list} , '2')*	
q301_C23a *(required)*	q301_C23a. What percent of patients require this item?<br>*Question relevant when: selected(${q101_C23_full_list} , '2')*<br>*Response constrained to: .>0 and .<=100 or .=999*	
q301_C23b *(required)*	q301_C23b. How many units of this item are required per patient (throughout the entire treatment provided for complication)?<br>*Smallest unit is one pad.*<br>*Question relevant when: selected(${q101_C23_full_list} , '2')*<br>*Response constrained to: .>0*	

Field	Question	Answer
q301_C24	q301_C24. Gauze compress, non sterile<br>*Question relevant when: selected(${q101_C24_full_list} , '2')*	

q301_C24a *(required)*	q301_C24a. What percent of patients require this item?<br>*Question relevant when: selected(${q101_C24_full_list} , '2')*<br>*Response constrained to: .>0 and .<=100 or .=999*	
q301_C24b *(required)*	q301_C24b. How many units of this item are required per patient (throughout the entire treatment provided for complication)?<br>*Smallest unit is one compress.*<br>*Question relevant when: selected(${q101_C24_full_list} , '2')*<br>*Response constrained to: .>0*	
q301_C25	q301_C25. Gauze compress, sterile<br>*Question relevant when: selected(${q101_C25_full_list} , '2')*	
q301_C25a *(required)*	q301_C25a. What percent of patients require this item?<br>*Question relevant when: selected(${q101_C25_full_list} , '2')*<br>*Response constrained to: .>0 and .<=100 or .=999*	
q301_C25b *(required)*	q301_C25b. How many units of this item are required per patient (throughout the entire treatment provided for complication)?<br>*Smallest unit is one compress.*<br>*Question relevant when: selected(${q101_C25_full_list} , '2')*<br>*Response constrained to: .>0*	
q301_C26	q301_C26. Linen saver<br>*Question relevant when: selected(${q101_C26_full_list} , '2')*	
q301_C26a *(required)*	q301_C26a. What percent of patients require this item?<br>*Question relevant when: selected(${q101_C26_full_list} , '2')*<br>*Response constrained to: .>0 and .<=100 or .=999*	
q301_C26b *(required)*	q301_C26b. How many units of this item are required per patient (throughout the entire treatment provided for complication)?<br>*Smallest unit is one saver (absorbant sheet).*<br>*Question relevant when: selected(${q101_C26_full_list} , '2')*<br>*Response constrained to: .>0*	
q301_C27	q301_C27. Sanitary Pad<br>*Question relevant when: selected(${q101_C27_full_list} , '2')*	
q301_C27a *(required)*	q301_C27a. What percent of patients require this item?<br>*Question relevant when: selected(${q101_C27_full_list} , '2')*<br>*Response constrained to: .>0 and .<=100 or .=999*	
q301_C27b *(required)*	q301_C27b. How many units of this item are required per patient (throughout the entire treatment provided for complication)?<br>*Smallest unit is one pad.*<br>*Question relevant when: selected(${q101_C27_full_list} , '2')*<br>*Response constrained to: .>0*	
q301_C28	q301_C28. Surgical plaster<br>*Question relevant when: selected(${q101_C28_full_list} , '2')*	
q301_C28a *(required)*	q301_C28a. What percent of patients require this item?<br>*Question relevant when: selected(${q101_C28_full_list} , '2')*<br>*Response constrained to: .>0 and .<=100 or .=999*	
q301_C28b *(required)*	q301_C28b. How many units of this item are required per patient (throughout the entire treatment provided for complication)?<br>*Smallest unit is one plaster.*<br>*Question relevant when: selected(${q101_C28_full_list} , '2')*<br>*Response constrained to: .>0*	
A. Consumables - Sepsis (4)<br>*Group relevant when: selected(${section_three_skip_con} , '1')*		
note_301_protective_etc	***Protective supplies***	
q301_C29	q301_C29. Apron (disposable)<br>*Question relevant when: selected(${q101_C29_full_list} , '2')*	
q301_C29a *(required)*	q301_C29a. What percent of patients require this item?<br>*Question relevant when: selected(${q101_C29_full_list} , '2')*<br>*Response constrained to: .>0 and .<=100 or .=999*	
q301_C29b *(required)*	q301_C29b. How many units of this item are required per patient (throughout the entire treatment provided for complication)?<br>*Smallest unit is one apron.*<br>*Question relevant when: selected(${q101_C29_full_list} , '2')*<br>*Response constrained to: .>0*	
q301_C30	q301_C30. Gown (disposable)<br>*Question relevant when: selected(${q101_C30_full_list} , '2')*	

Field	Question	Answer
q301_C30a *(required)*	q301_C30a. What percent of patients require this item?<br>*Question relevant when: selected(${q101_C30_full_list} , '2')*<br>*Response constrained to: .>0 and .<=100 or .=999*	
q301_C30b *(required)*	q301_C30b. How many units of this item are required per patient (throughout the entire treatment provided for complication)?<br>*Smallest unit is one gown.*<br>*Question relevant when: selected(${q101_C30_full_list} , '2')*<br>*Response constrained to: .>0*	
q301_C31	q301_C31. Latex examination glove, without powder<br>*Question relevant when: selected(${q101_C31_full_list} , '2')*	
q301_C31a *(required)*	q301_C31a. What percent of patients require this item?<br>*Question relevant when: selected(${q101_C31_full_list} , '2')*<br>*Response constrained to: .>0 and .<=100 or .=999*	
q301_C31b *(required)*	q301_C31b. How many units of this item are required per patient (throughout the entire treatment provided for complication)?<br>*Smallest unit is one glove.*<br>*Question relevant when: selected(${q101_C31_full_list} , '2')*<br>*Response constrained to: .>0*	
q301_C32	q301_C32. Latex examination glove, with powder<br>*Question relevant when: selected(${q101_C32_full_list} , '2')*	
q301_C32a *(required)*	q301_C32a. What percent of patients require this item?<br>*Question relevant when: selected(${q101_C32_full_list} , '2')*<br>*Response constrained to: .>0 and .<=100 or .=999*	
q301_C32b *(required)*	q301_C32b. How many units of this item are required per patient (throughout the entire treatment provided for complication)?<br>*Smallest unit is one glove.*<br>*Question relevant when: selected(${q101_C32_full_list} , '2')*<br>*Response constrained to: .>0*	
q301_C33	q301_C33. Non-latex (e.g. nitrile) examination glove<br>*Question relevant when: selected(${q101_C33_full_list} , '2')*	
q301_C33a *(required)*	q301_C33a. What percent of patients require this item?<br>*Question relevant when: selected(${q101_C33_full_list} , '2')*<br>*Response constrained to: .>0 and .<=100 or .=999*	
q301_C33b *(required)*	q301_C33b. How many units of this item are required per patient (throughout the entire treatment provided for complication)?<br>*Smallest unit is one glove.*<br>*Question relevant when: selected(${q101_C33_full_list} , '2')*<br>*Response constrained to: .>0*	
q301_C34	q301_C34. Sterile surgical glove<br>*Question relevant when: selected(${q101_C34_full_list} , '2')*	
q301_C34a *(required)*	q301_C34a. What percent of patients require this item?<br>*Question relevant when: selected(${q101_C34_full_list} , '2')*<br>*Response constrained to: .>0 and .<=100 or .=999*	
q301_C34b *(required)*	q301_C34b. How many units of this item are required per patient (throughout the entire treatment provided for complication)?<br>*Smallest unit is one glove.*<br>*Question relevant when: selected(${q101_C34_full_list} , '2')*<br>*Response constrained to: .>0*	
q301_C35	q301_C35. Mask (disposable)<br>*Question relevant when: selected(${q101_C35_full_list} , '2')*	
q301_C35a *(required)*	q301_C35a. What percent of patients require this item?<br>*Question relevant when: selected(${q101_C35_full_list} , '2')*<br>*Response constrained to: .>0 and .<=100 or .=999*	
q301_C35b *(required)*	q301_C35b. How many units of this item are required per patient (throughout the entire treatment provided for complication)?<br>*Smallest unit is one mask.*<br>*Question relevant when: selected(${q101_C35_full_list} , '2')*<br>*Response constrained to: .>0*	
q301_C36	q301_C36. Surgical cap<br>*Question relevant when: selected(${q101_C36_full_list} , '2')*	
q301_C36a *(required)*	q301_C36a. What percent of patients require this item?<br>*Question relevant when: selected(${q101_C36_full_list} , '2')*<br>*Response constrained to: .>0 and .<=100 or .=999*	

Field	Question	Answer
q301_C36b *(required)*	q301_C36b. How many units of this item are required per patient (throughout the entire treatment provided for complication)?<br>*Smallest unit is one cap.*<br>*Question relevant when: selected(${q101_C36_full_list} , '2')*<br>*Response constrained to: .>0*	
A. Consumables - Sepsis (5)<br>*Group relevant when: selected(${section_three_skip_con} , '1')*		
note_301_surgery_etc	***Surgery supplies***	
q301_C37	q301_C37. Scalpel blade, single use<br>*Question relevant when: selected(${q101_C37_full_list} , '2')*	
q301_C37a *(required)*	q301_C37a. What percent of patients require this item?<br>*Question relevant when: selected(${q101_C37_full_list} , '2')*<br>*Response constrained to: .>0 and .<=100 or .=999*	
q301_C37b *(required)*	q301_C37b. How many units of this item are required per patient (throughout the entire treatment provided for complication)?<br>*Smallest unit is one blade.*<br>*Question relevant when: selected(${q101_C37_full_list} , '2')*<br>*Response constrained to: .>0*	
q301_C38	q301_C38. Surgical drain<br>*Question relevant when: selected(${q101_C38_full_list} , '2')*	
q301_C38a *(required)*	q301_C38a. What percent of patients require this item?<br>*Question relevant when: selected(${q101_C38_full_list} , '2')*<br>*Response constrained to: .>0 and .<=100 or .=999*	
q301_C38b *(required)*	q301_C38b. How many units of this item are required per patient (throughout the entire treatment provided for complication)?<br>*Smallest unit is one drain.*<br>*Question relevant when: selected(${q101_C38_full_list} , '2')*<br>*Response constrained to: .>0*	
q301_C39	q301_C39. Suture string, chromic catgut (0)<br>*Question relevant when: selected(${q101_C39_full_list} , '2')*	
q301_C39a *(required)*	q301_C39a. What percent of patients require this item?<br>*Question relevant when: selected(${q101_C39_full_list} , '2')*<br>*Response constrained to: .>0 and .<=100 or .=999*	
q301_C39b *(required)*	q301_C39b. How many units of this item are required per patient (throughout the entire treatment provided for complication)?<br>*Smallest unit is one suture set.*<br>*Question relevant when: selected(${q101_C39_full_list} , '2')*<br>*Response constrained to: .>0*	
q301_C40	q301_C40. Suture string, silk (non absorbable and non-synthetic)<br>*Question relevant when: selected(${q101_C40_full_list} , '2')*	
q301_C40a *(required)*	q301_C40a. What percent of patients require this item?<br>*Question relevant when: selected(${q101_C40_full_list} , '2')*<br>*Response constrained to: .>0 and .<=100 or .=999*	
q301_C40b *(required)*	q301_C40b. How many units of this item are required per patient (throughout the entire treatment provided for complication)?<br>*Smallest unit is one suture set.*<br>*Question relevant when: selected(${q101_C40_full_list} , '2')*<br>*Response constrained to: .>0*	
q301_C41	q301_C41. Suture string, vicryl, no needle included<br>*Question relevant when: selected(${q101_C41_full_list} , '2')*	
q301_C41a *(required)*	q301_C41a. What percent of patients require this item?<br>*Question relevant when: selected(${q101_C41_full_list} , '2')*<br>*Response constrained to: .>0 and .<=100 or .=999*	
q301_C41b *(required)*	q301_C41b. How many units of this item are required per patient (throughout the entire treatment provided for complication)?<br>*Smallest unit is one suture set.*<br>*Question relevant when: selected(${q101_C41_full_list} , '2')*<br>*Response constrained to: .>0*	
q301_C42	q301_C42. Suture string, vicryl with needle<br>*Question relevant when: selected(${q101_C42_full_list} , '2')*	
q301_C42a *(required)*	q301_C42a. What percent of patients require this item?<br>*Question relevant when: selected(${q101_C42_full_list} , '2')*<br>*Response constrained to: .>0 and .<=100 or .=999*	

Field	Question	Answer
q301_C42b *(required)*	q301_C42b. How many units of this item are required per patient (throughout the entire treatment provided for complication)?<br>*Smallest unit is one suture set with needle..*<br>*Question relevant when: selected(${q101_C42_full_list} , '2')*<br>*Response constrained to: .>0*	
A. Consumables - Sepsis (6)<br>*Group relevant when: selected(${section_three_skip_con} , '1')*		
note_301_urine_etc	***Urine, bladder***	
q301_C43	q301_C43. Bladder/urinary catheter<br>*Question relevant when: selected(${q101_C43_full_list} , '2')*	
q301_C43a *(required)*	q301_C43a. What percent of patients require this item?<br>*Question relevant when: selected(${q101_C43_full_list} , '2')*<br>*Response constrained to: .>0 and .<=100 or .=999*	
q301_C43b *(required)*	q301_C43b. How many units of this item are required per patient (throughout the entire treatment provided for complication)?<br>*Smallest unit is one catheter.*<br>*Question relevant when: selected(${q101_C43_full_list} , '2')*<br>*Response constrained to: .>0*	
q301_C44	q301_C44. Foley's catheter<br>*Question relevant when: selected(${q101_C44_full_list} , '2')*	
q301_C44a *(required)*	q301_C44a. What percent of patients require this item?<br>*Question relevant when: selected(${q101_C44_full_list} , '2')*<br>*Response constrained to: .>0 and .<=100 or .=999*	
q301_C44b *(required)*	q301_C44b. How many units of this item are required per patient (throughout the entire treatment provided for complication)?<br>*Smallest unit is one catheter.*<br>*Question relevant when: selected(${q101_C44_full_list} , '2')*<br>*Response constrained to: .>0*	
q301_C45	q301_C45. Urine bag with valve and drain<br>*Question relevant when: selected(${q101_C45_full_list} , '2')*	
q301_C45a *(required)*	q301_C45a. What percent of patients require this item?<br>*Question relevant when: selected(${q101_C45_full_list} , '2')*<br>*Response constrained to: .>0 and .<=100 or .=999*	
q301_C45b *(required)*	q301_C45b. How many units of this item are required per patient (throughout the entire treatment provided for complication)?<br>*Smallest unit is one bag.*<br>*Question relevant when: selected(${q101_C45_full_list} , '2')*<br>*Response constrained to: .>0*	
A. Consumables - Sepsis (7)<br>*Group relevant when: selected(${section_three_skip_con} , '1')*		
note_301_other_etc	***Other supplies***	
q301_C46	q301_C46. Silicone oil for lubrication of MVA aspirator<br>*Question relevant when: selected(${q101_C46_full_list} , '2')*	
q301_C46a *(required)*	q301_C46a. What percent of patients require this item?<br>*Question relevant when: selected(${q101_C46_full_list} , '2')*<br>*Response constrained to: .>0 and .<=100 or .=999*	
q301_C46b *(required)*	q301_C46b. How many units of this item are required per patient (throughout the entire treatment provided for complication)?<br>*Smallest unit is mililiter (ml)*<br>*Question relevant when: selected(${q101_C46_full_list} , '2')*<br>*Response constrained to: .>0*	
q301_C47	q301_C47. Speculum, Bivalve/Cuscos (reusable)<br>*Question relevant when: selected(${q101_C47_full_list} , '2')*	
q301_C47a *(required)*	q301_C47a. What percent of patients require this item?<br>*Question relevant when: selected(${q101_C47_full_list} , '2')*<br>*Response constrained to: .>0 and .<=100 or .=999*	
q301_C47b *(required)*	q301_C47b. How many units of this item are required per patient (throughout the entire treatment provided for complication)?<br>*Smallest unit is one speculum.*<br>*Question relevant when: selected(${q101_C47_full_list} , '2')*<br>*Response constrained to: .>0*	
q301_C48	q301_C48. Speculum, Sim's (disposable)<br>*Question relevant when: selected(${q101_C48_full_list} , '2')*	

Field	Question	Answer
q301_C48a *(required)*	q301_C48a. What percent of patients require this item?<br>*Question relevant when: selected(${q101_C48_full_list} , '2')*<br>*Response constrained to: .>0 and .<=100 or .=999*	
q301_C48b *(required)*	q301_C48b. How many units of this item are required per patient (throughout the entire treatment provided for complication)?<br>*Smallest unit is one speculum.*<br>*Question relevant when: selected(${q101_C48_full_list} , '2')*<br>*Response constrained to: .>0*	
A. Consumables - Sepsis (8)<br>*Group relevant when: selected(${section_three_skip_con} , '1')*		
note_301_suggest_other_etc	***Other supplies - suggestions?***	
q301_C49	q301_C49. "[q101_C49_full_list_other]"<br>*Question relevant when: selected(${q101_C49_full_list} , '2')*	
q301_C49a *(required)*	q301_C49a. What percent of patients require this item?<br>*Question relevant when: selected(${q101_C49_full_list} , '2')*<br>*Response constrained to: .>0 and .<=100 or .=999*	
q301_C49b *(required)*	q301_C49b. How many units of this item are required per patient (throughout the entire treatment provided for complication)?<br>*Question relevant when: selected(${q101_C49_full_list} , '2')*<br>*Response constrained to: .>0*	
q301_C50	q301_C50. "[q101_C50_full_list_other]"<br>*Question relevant when: selected(${q101_C50_full_list} , '2')*	
q301_C50a *(required)*	q301_C50a. What percent of patients require this item?<br>*Question relevant when: selected(${q101_C50_full_list} , '2')*<br>*Response constrained to: .>0 and .<=100 or .=999*	
q301_C50b *(required)*	q301_C50b. How many units of this item are required per patient (throughout the entire treatment provided for complication)?<br>*Question relevant when: selected(${q101_C50_full_list} , '2')*<br>*Response constrained to: .>0*	
q301_C51	q301_C51. "[q101_C51_full_list_other]"<br>*Question relevant when: selected(${q101_C51_full_list} , '2')*	
q301_C51a *(required)*	q301_C51a. What percent of patients require this item?<br>*Question relevant when: selected(${q101_C51_full_list} , '2')*<br>*Response constrained to: .>0 and .<=100 or .=999*	
q301_C51b *(required)*	q301_C51b. How many units of this item are required per patient (throughout the entire treatment provided for complication)?<br>*Question relevant when: selected(${q101_C51_full_list} , '2')*<br>*Response constrained to: .>0*	
q301_C52	q301_C52. "[q101_C52_full_list_other]"<br>*Question relevant when: selected(${q101_C52_full_list} , '2')*	
q301_C52a *(required)*	q301_C52a. What percent of patients require this item?<br>*Question relevant when: selected(${q101_C52_full_list} , '2')*<br>*Response constrained to: .>0 and .<=100 or .=999*	
q301_C52b *(required)*	q301_C52b. How many units of this item are required per patient (throughout the entire treatment provided for complication)?<br>*Question relevant when: selected(${q101_C52_full_list} , '2')*<br>*Response constrained to: .>0*	
q301_C53	q301_C53. "[q101_C53_full_list_other]"<br>*Question relevant when: selected(${q101_C53_full_list} , '2')*	
q301_C53a *(required)*	q301_C53a. What percent of patients require this item?<br>*Question relevant when: selected(${q101_C53_full_list} , '2')*<br>*Response constrained to: .>0 and .<=100 or .=999*	
q301_C53b *(required)*	q301_C53b. How many units of this item are required per patient (throughout the entire treatment provided for complication)?<br>*Question relevant when: selected(${q101_C53_full_list} , '2')*<br>*Response constrained to: .>0*	
group_section_four_intro		
section4_start	**SECTION IV. SHOCK - USAGE OF ALL ITEMS**	
section_four_skip_con *(required)*	INTERVIEWER: WOULD YOU LIKE TO COMPLETE THIS SECTION NOW OR SKIP THIS SECTION AND RETURN TO IT LATER?<br>*You may need to skip if the participant has indicated that s/he cannot answer the questions in this section.*	1 Do not skip, complete this section now.<br>2 Skip and come back to this section later

		section later.
**Field**	**Question**	**Answer**

Field	Question	Answer
group_section_four_introB *Group relevant when: selected(${section_four_skip_con} , '1')*		
section4_start2	In this section of the interview, we will review all of the items that you said are used for management of shock. For each item that is used, I'm going to ask questions on how many women need it and how much of it is used.	
section4_start3	INTERVIEWER: ENTER WHOLE NUMBERS OR DECIMALS. DO NOT TYPE PERCENT SIGNS. ENTER 999 FOR ANY THAT ARE UNKNOWN.	
A. Consumables - Shock (1) *Group relevant when: selected(${section_four_skip_con} , '1')*		
note_401_cleaning	***Cleaning, disinfectants, sterilization, etc.***	
q401_C1	q401_C1. Alcohol, denatured 70% *Question relevant when: selected(${q101_C1_full_list} , '3')*	
q401_C1a *(required)*	q401_C1a. What percent of patients require this item? *Question relevant when: selected(${q101_C1_full_list} , '3')* *Response constrained to: .>0 and .<=100 or .=999*	
q401_C1b *(required)*	q401_C1b. How many units of this item are required per patient (throughout the entire treatment provided for complication)? *Smallest unit is mililiter (ml)* *Question relevant when: selected(${q101_C1_full_list} , '3')* *Response constrained to: .>0*	
q401_C2	q401_C2. Chlorine (JIK) or Glutaraldehyde solution *Question relevant when: selected(${q101_C2_full_list} , '3')*	
q401_C2a *(required)*	q401_C2a. What percent of patients require this item? *Question relevant when: selected(${q101_C2_full_list} , '3')* *Response constrained to: .>0 and .<=100 or .=999*	
q401_C2b *(required)*	q401_C2b. How many units of this item are required per patient (throughout the entire treatment provided for complication)? *Smallest unit is mililiter (ml)* *Question relevant when: selected(${q101_C2_full_list} , '3')* *Response constrained to: .>0*	
q401_C3	q401_C3. Clean water *Question relevant when: selected(${q101_C3_full_list} , '3')*	
q401_C3a *(required)*	q401_C3a. What percent of patients require this item? *Question relevant when: selected(${q101_C3_full_list} , '3')* *Response constrained to: .>0 and .<=100 or .=999*	
q401_C3b *(required)*	q401_C3b. How many units of this item are required per patient (throughout the entire treatment provided for complication)? *Smallest unit is litre (ltr)* *Question relevant when: selected(${q101_C3_full_list} , '3')* *Response constrained to: .>0*	
q401_C4	q401_C4. Dettol solution or other water-based antiseptic *Question relevant when: selected(${q101_C4_full_list} , '3')*	
q401_C4a *(required)*	q401_C4a. What percent of patients require this item? *Question relevant when: selected(${q101_C4_full_list} , '3')* *Response constrained to: .>0 and .<=100 or .=999*	
q401_C4b *(required)*	q401_C4b. How many units of this item are required per patient (throughout the entire treatment provided for complication)? *Smallest unit is mililiter (ml)* *Question relevant when: selected(${q101_C4_full_list} , '3')* *Response constrained to: .>0*	
q401_C5	q401_C5. Cetrimide 15% ("Savlon") *Question relevant when: selected(${q101_C5_full_list} , '3')*	
q401_C5a *(required)*	q401_C5a. What percent of patients require this item? *Question relevant when: selected(${q101_C5_full_list} , '3')* *Response constrained to: .>0 and .<=100 or .=999*	
q401_C5b *(required)*	q401_C5b. How many units of this item are required per patient (throughout the entire treatment provided for complication)? *Smallest unit is mililiter (ml)* *Question relevant when: selected(${q101_C5_full_list} , '3')* *Response constrained to: .>0*	
q401_C6	q401_C6. Hand wash (liquid) *Question relevant when: selected(${q101_C6_full_list} , '3')*	
q401_C6a *(required)*	q401_C6a. What percent of patients require this item? *Question relevant when: selected(${q101_C6_full_list} , '3')*	

Field	Question	Answer
	Response constrained to: .>0 and .<=100 or .=999	
q401_C6b *(required)*	q401_C6b. How many units of this item are required per patient (throughout the entire treatment provided for complication)?<br>*Smallest unit is mililiter (ml)*<br>*Question relevant when: selected(${q101_C6_full_list} , '3')*<br>*Response constrained to: .>0*	
q401_C7	q401_C7. Soap (bar)<br>*Question relevant when: selected(${q101_C7_full_list} , '3')*	
q401_C7a *(required)*	q401_C7a. What percent of patients require this item?<br>*Question relevant when: selected(${q101_C7_full_list} , '3')*<br>*Response constrained to: .>0 and .<=100 or .=999*	
q401_C7b *(required)*	q401_C7b. How many units of this item are required per patient (throughout the entire treatment provided for complication)?<br>*Smallest unit is 1 bar of soap*<br>*Question relevant when: selected(${q101_C7_full_list} , '3')*<br>*Response constrained to: .>0*	
q401_C8	q401_C8. Hand sterilizer (waterless)<br>*Question relevant when: selected(${q101_C8_full_list} , '3')*	
q401_C8a *(required)*	q401_C8a. What percent of patients require this item?<br>*Question relevant when: selected(${q101_C8_full_list} , '3')*<br>*Response constrained to: .>0 and .<=100 or .=999*	
q401_C8b *(required)*	q401_C8b. How many units of this item are required per patient (throughout the entire treatment provided for complication)?<br>*Smallest unit is mililiter (ml)*<br>*Question relevant when: selected(${q101_C8_full_list} , '3')*<br>*Response constrained to: .>0*	
A. Consumables - Shock (2)<br>*Group relevant when: selected(${section_four_skip_con} , '1')*		
note_401_IV_needle_syringe	***IV, needles, syringes, etc.***	
q401_C9	q401_C9. Blood giving set with needle<br>*Question relevant when: selected(${q101_C9_full_list} , '3')*	
q401_C9a *(required)*	q401_C9a. What percent of patients require this item?<br>*Question relevant when: selected(${q101_C9_full_list} , '3')*<br>*Response constrained to: .>0 and .<=100 or .=999*	
q401_C9b *(required)*	q401_C9b. How many units of this item are required per patient (throughout the entire treatment provided for complication)?<br>*Smallest unit is one set.*<br>*Question relevant when: selected(${q101_C9_full_list} , '3')*<br>*Response constrained to: .>0*	
q401_C10	q401_C10. IV cannula<br>*Question relevant when: selected(${q101_C10_full_list} , '3')*	
q401_C10a *(required)*	q401_C10a. What percent of patients require this item?<br>*Question relevant when: selected(${q101_C10_full_list} , '3')*<br>*Response constrained to: .>0 and .<=100 or .=999*	
q401_C10b *(required)*	q401_C10b. How many units of this item are required per patient (throughout the entire treatment provided for complication)?<br>*Smallest unit is one cannula.*<br>*Question relevant when: selected(${q101_C10_full_list} , '3')*<br>*Response constrained to: .>0*	
q401_C11	q401_C11. IV set<br>*Question relevant when: selected(${q101_C11_full_list} , '3')*	
q401_C11a *(required)*	q401_C11a. What percent of patients require this item?<br>*Question relevant when: selected(${q101_C11_full_list} , '3')*<br>*Response constrained to: .>0 and .<=100 or .=999*	
q401_C11b *(required)*	q401_C11b. How many units of this item are required per patient (throughout the entire treatment provided for complication)?<br>*Smallest unit is one set.*<br>*Question relevant when: selected(${q101_C11_full_list} , '3')*<br>*Response constrained to: .>0*	
q401_C12	q401_C12. Needle luer tip 18Gx1.1/2" (1.2x38mm)<br>*Question relevant when: selected(${q101_C12_full_list} , '3')*	
q401_C12a *(required)*	q401_C12a. What percent of patients require this item?<br>*Question relevant when: selected(${q101_C12_full_list} , '3')*<br>*Response constrained to: .>0 and .<=100 or .=999*	

Field	Question	Answer
q401_C12b *(required)*	q401_C12b. How many units of this item are required per patient (throughout the entire treatment provided for complication)?<br>*Smallest unit is one needle.*<br>*Question relevant when: selected(${q101_C12_full_list} , '3')*<br>*Response constrained to: .>0*	
q401_C13	q401_C13. Needle luer tip 21Gx1.1/2" (0.8x38mm)<br>*Question relevant when: selected(${q101_C13_full_list} , '3')*	
q401_C13a *(required)*	q401_C13a. What percent of patients require this item?<br>*Question relevant when: selected(${q101_C13_full_list} , '3')*<br>*Response constrained to: .>0 and .<=100 or .=999*	
q401_C13b *(required)*	q401_C13b. How many units of this item are required per patient (throughout the entire treatment provided for complication)?<br>*Smallest unit is one needle.*<br>*Question relevant when: selected(${q101_C13_full_list} , '3')*<br>*Response constrained to: .>0*	
q401_C14	q401_C14. Needle, suture, round body<br>*Question relevant when: selected(${q101_C14_full_list} , '3')*	
q401_C14a *(required)*	q401_C14a. What percent of patients require this item?<br>*Question relevant when: selected(${q101_C14_full_list} , '3')*<br>*Response constrained to: .>0 and .<=100 or .=999*	
q401_C14b *(required)*	q401_C14b. How many units of this item are required per patient (throughout the entire treatment provided for complication)?<br>*Smallest unit is one needle.*<br>*Question relevant when: selected(${q101_C14_full_list} , '3')*<br>*Response constrained to: .>0*	
q401_C15	q401_C15. Spinal needle<br>*Question relevant when: selected(${q101_C15_full_list} , '3')*	
q401_C15a *(required)*	q401_C15a. What percent of patients require this item?<br>*Question relevant when: selected(${q101_C15_full_list} , '3')*<br>*Response constrained to: .>0 and .<=100 or .=999*	
q401_C15b *(required)*	q401_C15b. How many units of this item are required per patient (throughout the entire treatment provided for complication)?<br>*Smallest unit is one needle.*<br>*Question relevant when: selected(${q101_C15_full_list} , '3')*<br>*Response constrained to: .>0*	
q401_C16	q401_C16. Syringe 10ml, with bypacked needle 21Gx1.5"<br>*Question relevant when: selected(${q101_C16_full_list} , '3')*	
q401_C16a *(required)*	q401_C16a. What percent of patients require this item?<br>*Question relevant when: selected(${q101_C16_full_list} , '3')*<br>*Response constrained to: .>0 and .<=100 or .=999*	
q401_C16b *(required)*	q401_C16b. How many units of this item are required per patient (throughout the entire treatment provided for complication)?<br>*Smallest unit is one syringe.*<br>*Question relevant when: selected(${q101_C16_full_list} , '3')*<br>*Response constrained to: .>0*	
q401_C17	q401_C17. Syringe 5ml, with bypacked needle 21Gx1.5"<br>*Question relevant when: selected(${q101_C17_full_list} , '3')*	
q401_C17a *(required)*	q401_C17a. What percent of patients require this item?<br>*Question relevant when: selected(${q101_C17_full_list} , '3')*<br>*Response constrained to: .>0 and .<=100 or .=999*	
q401_C17b *(required)*	q401_C17b. How many units of this item are required per patient (throughout the entire treatment provided for complication)?<br>*Smallest unit is one syringe.*<br>*Question relevant when: selected(${q101_C17_full_list} , '3')*<br>*Response constrained to: .>0*	
q401_C18	q401_C18. Syringe luer tip 10ml, no needle<br>*Question relevant when: selected(${q101_C18_full_list} , '3')*	
q401_C18a *(required)*	q401_C18a. What percent of patients require this item?<br>*Question relevant when: selected(${q101_C18_full_list} , '3')*<br>*Response constrained to: .>0 and .<=100 or .=999*	
q401_C18b *(required)*	q401_C18b. How many units of this item are required per patient (throughout the entire treatment provided for complication)?<br>*Smallest unit is one syringe.*<br>*Question relevant when: selected(${q101_C18_full_list} , '3')*	

Field	Question	Answer
	Response constrained to: .>0	

Field	Question	Answer
q401_C19	q401_C19. Syringe luer tip 5ml, no needle<br>*Question relevant when: selected(${q101_C19_full_list} , '3')*	
q401_C19a *(required)*	q401_C19a. What percent of patients require this item?<br>*Question relevant when: selected(${q101_C19_full_list} , '3')*<br>*Response constrained to: .>0 and .<=100 or .=999*	
q401_C19b *(required)*	q401_C19b. How many units of this item are required per patient (throughout the entire treatment provided for complication)?<br>*Smallest unit is one syringe.*<br>*Question relevant when: selected(${q101_C19_full_list} , '3')*<br>*Response constrained to: .>0*	
q401_C20	q401_C20. Venous catheter G18 + injection port and lock tip<br>*Question relevant when: selected(${q101_C20_full_list} , '3')*	
q401_C20a *(required)*	q401_C20a. What percent of patients require this item?<br>*Question relevant when: selected(${q101_C20_full_list} , '3')*<br>*Response constrained to: .>0 and .<=100 or .=999*	
q401_C20b *(required)*	q401_C20b. How many units of this item are required per patient (throughout the entire treatment provided for complication)?<br>*Smallest unit is one catheter.*<br>*Question relevant when: selected(${q101_C20_full_list} , '3')*<br>*Response constrained to: .>0*	
A. Consumables - Shock (3)<br>*Group relevant when: selected(${section_four_skip_con} , '1')*		
note_401_bandages_etc	***Bandages, absorbants, wound care, etc.***	
q401_C21	q401_C21. Adhesive tape<br>*Question relevant when: selected(${q101_C21_full_list} , '3')*	
q401_C21a *(required)*	q401_C21a. What percent of patients require this item?<br>*Question relevant when: selected(${q101_C21_full_list} , '3')*<br>*Response constrained to: .>0 and .<=100 or .=999*	
q401_C21b *(required)*	q401_C21b. How many units of this item are required per patient (throughout the entire treatment provided for complication)?<br>*Smallest unit is one roll of tape.*<br>*Question relevant when: selected(${q101_C21_full_list} , '3')*<br>*Response constrained to: .>0*	
q401_C22	q401_C22. Cotton swab<br>*Question relevant when: selected(${q101_C22_full_list} , '3')*	
q401_C22a *(required)*	q401_C22a. What percent of patients require this item?<br>*Question relevant when: selected(${q101_C22_full_list} , '3')*<br>*Response constrained to: .>0 and .<=100 or .=999*	
q401_C22b *(required)*	q401_C22b. How many units of this item are required per patient (throughout the entire treatment provided for complication)?<br>*Smallest unit is one swab.*<br>*Question relevant when: selected(${q101_C22_full_list} , '3')*<br>*Response constrained to: .>0*	
q401_C23	q401_C23. Dressing pad absorbent sterile<br>*Question relevant when: selected(${q101_C23_full_list} , '3')*	
q401_C23a *(required)*	q401_C23a. What percent of patients require this item?<br>*Question relevant when: selected(${q101_C23_full_list} , '3')*<br>*Response constrained to: .>0 and .<=100 or .=999*	
q401_C23b *(required)*	q401_C23b. How many units of this item are required per patient (throughout the entire treatment provided for complication)?<br>*Smallest unit is one pad.*<br>*Question relevant when: selected(${q101_C23_full_list} , '3')*<br>*Response constrained to: .>0*	
q401_C24	q401_C24. Gauze compress, non sterile<br>*Question relevant when: selected(${q101_C24_full_list} , '3')*	
q401_C24a *(required)*	q401_C24a. What percent of patients require this item?<br>*Question relevant when: selected(${q101_C24_full_list} , '3')*<br>*Response constrained to: .>0 and .<=100 or .=999*	
q401_C24b *(required)*	q401_C24b. How many units of this item are required per patient (throughout the entire treatment provided for complication)?<br>*Smallest unit is one compress.*<br>*Question relevant when: selected(${q101_C24_full_list} , '3')*<br>*Response constrained to: .>0*	

Field	Question	Answer
q401_C25	q401_C25. Gauze compress, sterile<br>*Question relevant when: selected(${q101_C25_full_list} , '3')*	
q401_C25a *(required)*	q401_C25a. What percent of patients require this item?<br>*Question relevant when: selected(${q101_C25_full_list} , '3')*<br>*Response constrained to: .>0 and .<=100 or .=999*	
q401_C25b *(required)*	q401_C25b. How many units of this item are required per patient (throughout the entire treatment provided for complication)?<br>*Smallest unit is one compress.*<br>*Question relevant when: selected(${q101_C25_full_list} , '3')*<br>*Response constrained to: .>0*	
q401_C26	q401_C26. Linen saver<br>*Question relevant when: selected(${q101_C26_full_list} , '3')*	
q401_C26a *(required)*	q401_C26a. What percent of patients require this item?<br>*Question relevant when: selected(${q101_C26_full_list} , '3')*<br>*Response constrained to: .>0 and .<=100 or .=999*	
q401_C26b *(required)*	q401_C26b. How many units of this item are required per patient (throughout the entire treatment provided for complication)?<br>*Smallest unit is one saver (absorbant sheet).*<br>*Question relevant when: selected(${q101_C26_full_list} , '3')*<br>*Response constrained to: .>0*	
q401_C27	q401_C27. Sanitary Pad<br>*Question relevant when: selected(${q101_C27_full_list} , '3')*	
q401_C27a *(required)*	q401_C27a. What percent of patients require this item?<br>*Question relevant when: selected(${q101_C27_full_list} , '3')*<br>*Response constrained to: .>0 and .<=100 or .=999*	
q401_C27b *(required)*	q401_C27b. How many units of this item are required per patient (throughout the entire treatment provided for complication)?<br>*Smallest unit is one pad.*<br>*Question relevant when: selected(${q101_C27_full_list} , '3')*<br>*Response constrained to: .>0*	
q401_C28	q401_C28. Surgical plaster<br>*Question relevant when: selected(${q101_C28_full_list} , '3')*	
q401_C28a *(required)*	q401_C28a. What percent of patients require this item?<br>*Question relevant when: selected(${q101_C28_full_list} , '3')*<br>*Response constrained to: .>0 and .<=100 or .=999*	
q401_C28b *(required)*	q401_C28b. How many units of this item are required per patient (throughout the entire treatment provided for complication)?<br>*Smallest unit is one plaster.*<br>*Question relevant when: selected(${q101_C28_full_list} , '3')*<br>*Response constrained to: .>0*	
A. Consumables - Shock (4)<br>*Group relevant when: selected(${section_four_skip_con} , '1')*		
note_401_protective_etc	***Protective supplies***	
q401_C29	q401_C29. Apron (disposable)<br>*Question relevant when: selected(${q101_C29_full_list} , '3')*	
q401_C29a *(required)*	q401_C29a. What percent of patients require this item?<br>*Question relevant when: selected(${q101_C29_full_list} , '3')*<br>*Response constrained to: .>0 and .<=100 or .=999*	
q401_C29b *(required)*	q401_C29b. How many units of this item are required per patient (throughout the entire treatment provided for complication)?<br>*Smallest unit is one apron.*<br>*Question relevant when: selected(${q101_C29_full_list} , '3')*<br>*Response constrained to: .>0*	
q401_C30	q401_C30. Gown (disposable)<br>*Question relevant when: selected(${q101_C30_full_list} , '3')*	
q401_C30a *(required)*	q401_C30a. What percent of patients require this item?<br>*Question relevant when: selected(${q101_C30_full_list} , '3')*<br>*Response constrained to: .>0 and .<=100 or .=999*	
q401_C30b *(required)*	q401_C30b. How many units of this item are required per patient (throughout the entire treatment provided for complication)?<br>*Smallest unit is one gown.*<br>*Question relevant when: selected(${q101_C30_full_list} , '3')*<br>*Response constrained to: .>0*	
q401_C31	q401_C31. Latex examination glove, without powder<br>*Question relevant when: selected(${q101_C31_full_list} , '3')*	

Field	Question	Answer
q401_C31a *(required)*	q401_C31a. What percent of patients require this item?<br>*Question relevant when: selected(${q101_C31_full_list} , '3')*<br>*Response constrained to: .>0 and .<=100 or .=999*	
q401_C31b *(required)*	q401_C31b. How many units of this item are required per patient (throughout the entire treatment provided for complication)?<br>*Smallest unit is one glove.*<br>*Question relevant when: selected(${q101_C31_full_list} , '3')*<br>*Response constrained to: .>0*	
q401_C32	q401_C32. Latex examination glove, with powder<br>*Question relevant when: selected(${q101_C32_full_list} , '3')*	
q401_C32a *(required)*	q401_C32a. What percent of patients require this item?<br>*Question relevant when: selected(${q101_C32_full_list} , '3')*<br>*Response constrained to: .>0 and .<=100 or .=999*	
q401_C32b *(required)*	q401_C32b. How many units of this item are required per patient (throughout the entire treatment provided for complication)?<br>*Smallest unit is one glove.*<br>*Question relevant when: selected(${q101_C32_full_list} , '3')*<br>*Response constrained to: .>0*	
q401_C33	q401_C33. Non-latex (e.g. nitrile) examination glove<br>*Question relevant when: selected(${q101_C33_full_list} , '3')*	
q401_C33a *(required)*	q401_C33a. What percent of patients require this item?<br>*Question relevant when: selected(${q101_C33_full_list} , '3')*<br>*Response constrained to: .>0 and .<=100 or .=999*	
q401_C33b *(required)*	q401_C33b. How many units of this item are required per patient (throughout the entire treatment provided for complication)?<br>*Smallest unit is one glove.*<br>*Question relevant when: selected(${q101_C33_full_list} , '3')*<br>*Response constrained to: .>0*	
q401_C34	q401_C34. Sterile surgical glove<br>*Question relevant when: selected(${q101_C34_full_list} , '3')*	
q401_C34a *(required)*	q401_C34a. What percent of patients require this item?<br>*Question relevant when: selected(${q101_C34_full_list} , '3')*<br>*Response constrained to: .>0 and .<=100 or .=999*	
q401_C34b *(required)*	q401_C34b. How many units of this item are required per patient (throughout the entire treatment provided for complication)?<br>*Smallest unit is one glove.*<br>*Question relevant when: selected(${q101_C34_full_list} , '3')*<br>*Response constrained to: .>0*	
q401_C35	q401_C35. Mask (disposable)<br>*Question relevant when: selected(${q101_C35_full_list} , '3')*	
q401_C35a *(required)*	q401_C35a. What percent of patients require this item?<br>*Question relevant when: selected(${q101_C35_full_list} , '3')*<br>*Response constrained to: .>0 and .<=100 or .=999*	
q401_C35b *(required)*	q401_C35b. How many units of this item are required per patient (throughout the entire treatment provided for complication)?<br>*Smallest unit is one mask.*<br>*Question relevant when: selected(${q101_C35_full_list} , '3')*<br>*Response constrained to: .>0*	
q401_C36	q401_C36. Surgical cap<br>*Question relevant when: selected(${q101_C36_full_list} , '3')*	
q401_C36a *(required)*	q401_C36a. What percent of patients require this item?<br>*Question relevant when: selected(${q101_C36_full_list} , '3')*<br>*Response constrained to: .>0 and .<=100 or .=999*	
q401_C36b *(required)*	q401_C36b. How many units of this item are required per patient (throughout the entire treatment provided for complication)?<br>*Smallest unit is one cap.*<br>*Question relevant when: selected(${q101_C36_full_list} , '3')*<br>*Response constrained to: .>0*	
A. Consumables - Shock (5)<br>*Group relevant when: selected(${section_four_skip_con} , '1')*		
note_401_surgery_etc	***Surgery supplies***	
q401_C37	q401_C37. Scalpel blade, single use<br>*Question relevant when: selected(${q101_C37_full_list} , '3')*	

Question relevant when: selected(${q101_C37_full_list} , '3')

Field	Question	Answer
q401_C37a *(required)*	q401_C37a. What percent of patients require this item?<br>*Question relevant when: selected(${q101_C37_full_list} , '3')*<br>*Response constrained to: .>0 and .<=100 or .=999*	
q401_C37b *(required)*	q401_C37b. How many units of this item are required per patient (throughout the entire treatment provided for complication)?<br>*Smallest unit is one blade.*<br>*Question relevant when: selected(${q101_C37_full_list} , '3')*<br>*Response constrained to: .>0*	
q401_C38	q401_C38. Surgical drain<br>*Question relevant when: selected(${q101_C38_full_list} , '3')*	
q401_C38a *(required)*	q401_C38a. What percent of patients require this item?<br>*Question relevant when: selected(${q101_C38_full_list} , '3')*<br>*Response constrained to: .>0 and .<=100 or .=999*	
q401_C38b *(required)*	q401_C38b. How many units of this item are required per patient (throughout the entire treatment provided for complication)?<br>*Smallest unit is one drain.*<br>*Question relevant when: selected(${q101_C38_full_list} , '3')*<br>*Response constrained to: .>0*	
q401_C39	q401_C39. Suture string, chromic catgut (0)<br>*Question relevant when: selected(${q101_C39_full_list} , '3')*	
q401_C39a *(required)*	q401_C39a. What percent of patients require this item?<br>*Question relevant when: selected(${q101_C39_full_list} , '3')*<br>*Response constrained to: .>0 and .<=100 or .=999*	
q401_C39b *(required)*	q401_C39b. How many units of this item are required per patient (throughout the entire treatment provided for complication)?<br>*Smallest unit is one suture set.*<br>*Question relevant when: selected(${q101_C39_full_list} , '3')*<br>*Response constrained to: .>0*	
q401_C40	q401_C40. Suture string, silk (non absorbable and non-synthetic)<br>*Question relevant when: selected(${q101_C40_full_list} , '3')*	
q401_C40a *(required)*	q401_C40a. What percent of patients require this item?<br>*Question relevant when: selected(${q101_C40_full_list} , '3')*<br>*Response constrained to: .>0 and .<=100 or .=999*	
q401_C40b *(required)*	q401_C40b. How many units of this item are required per patient (throughout the entire treatment provided for complication)?<br>*Smallest unit is one suture set.*<br>*Question relevant when: selected(${q101_C40_full_list} , '3')*<br>*Response constrained to: .>0*	
q401_C41	q401_C41. Suture string, vicryl, no needle included<br>*Question relevant when: selected(${q101_C41_full_list} , '3')*	
q401_C41a *(required)*	q401_C41a. What percent of patients require this item?<br>*Question relevant when: selected(${q101_C41_full_list} , '3')*<br>*Response constrained to: .>0 and .<=100 or .=999*	
q401_C41b *(required)*	q401_C41b. How many units of this item are required per patient (throughout the entire treatment provided for complication)?<br>*Smallest unit is one suture set.*<br>*Question relevant when: selected(${q101_C41_full_list} , '3')*<br>*Response constrained to: .>0*	
q401_C42	q401_C42. Suture string, vicryl with needle<br>*Question relevant when: selected(${q101_C42_full_list} , '3')*	
q401_C42a *(required)*	q401_C42a. What percent of patients require this item?<br>*Question relevant when: selected(${q101_C42_full_list} , '3')*<br>*Response constrained to: .>0 and .<=100 or .=999*	
q401_C42b *(required)*	q401_C42b. How many units of this item are required per patient (throughout the entire treatment provided for complication)?<br>*Smallest unit is one suture set with needle..*<br>*Question relevant when: selected(${q101_C42_full_list} , '3')*<br>*Response constrained to: .>0*	
A. Consumables - Shock (6)<br>*Group relevant when: selected(${section_four_skip_con} , '1')*		
note_401_urine_etc	***Urine, bladder***	

Field	Question	Answer
q401_C43	q401_C43. Bladder/urinary catheter<br>*Question relevant when: selected(${q101_C43_full_list} , '3')*	
q401_C43a *(required)*	q401_C43a. What percent of patients require this item?<br>*Question relevant when: selected(${q101_C43_full_list} , '3')*<br>*Response constrained to: .>0 and .<=100 or .=999*	
q401_C43b *(required)*	q401_C43b. How many units of this item are required per patient (throughout the entire treatment provided for complication)?<br>*Smallest unit is one catheter.*<br>*Question relevant when: selected(${q101_C43_full_list} , '3')*<br>*Response constrained to: .>0*	
q401_C44	q401_C44. Foley's catheter<br>*Question relevant when: selected(${q101_C44_full_list} , '3')*	
q401_C44a *(required)*	q401_C44a. What percent of patients require this item?<br>*Question relevant when: selected(${q101_C44_full_list} , '3')*<br>*Response constrained to: .>0 and .<=100 or .=999*	
q401_C44b *(required)*	q401_C44b. How many units of this item are required per patient (throughout the entire treatment provided for complication)?<br>*Smallest unit is one catheter.*<br>*Question relevant when: selected(${q101_C44_full_list} , '3')*<br>*Response constrained to: .>0*	
q401_C45	q401_C45. Urine bag with valve and drain<br>*Question relevant when: selected(${q101_C45_full_list} , '3')*	
q401_C45a *(required)*	q401_C45a. What percent of patients require this item?<br>*Question relevant when: selected(${q101_C45_full_list} , '3')*<br>*Response constrained to: .>0 and .<=100 or .=999*	
q401_C45b *(required)*	q401_C45b. How many units of this item are required per patient (throughout the entire treatment provided for complication)?<br>*Smallest unit is one bag.*<br>*Question relevant when: selected(${q101_C45_full_list} , '3')*<br>*Response constrained to: .>0*	
A. Consumables - Shock (7)<br>*Group relevant when: selected(${section_four_skip_con} , '1')*		
note_401_other_etc	***Other supplies***	
q401_C46	q401_C46. Silicone oil for lubrication of MVA aspirator<br>*Question relevant when: selected(${q101_C46_full_list} , '3')*	
q401_C46a *(required)*	q401_C46a. What percent of patients require this item?<br>*Question relevant when: selected(${q101_C46_full_list} , '3')*<br>*Response constrained to: .>0 and .<=100 or .=999*	
q401_C46b *(required)*	q401_C46b. How many units of this item are required per patient (throughout the entire treatment provided for complication)?<br>*Smallest unit is mililiter (ml)*<br>*Question relevant when: selected(${q101_C46_full_list} , '3')*<br>*Response constrained to: .>0*	
q401_C47	q401_C47. Speculum, Bivalve/Cuscos (reusable)<br>*Question relevant when: selected(${q101_C47_full_list} , '3')*	
q401_C47a *(required)*	q401_C47a. What percent of patients require this item?<br>*Question relevant when: selected(${q101_C47_full_list} , '3')*<br>*Response constrained to: .>0 and .<=100 or .=999*	
q401_C47b *(required)*	q401_C47b. How many units of this item are required per patient (throughout the entire treatment provided for complication)?<br>*Smallest unit is one speculum.*<br>*Question relevant when: selected(${q101_C47_full_list} , '3')*<br>*Response constrained to: .>0*	
q401_C48	q401_C48. Speculum, Sim's (disposable)<br>*Question relevant when: selected(${q101_C48_full_list} , '3')*	
q401_C48a *(required)*	q401_C48a. What percent of patients require this item?<br>*Question relevant when: selected(${q101_C48_full_list} , '3')*<br>*Response constrained to: .>0 and .<=100 or .=999*	
q401_C48b *(required)*	q401_C48b. How many units of this item are required per patient (throughout the entire treatment provided for complication)?<br>*Smallest unit is one speculum.*<br>*Question relevant when: selected(${q101_C48_full_list} , '3')*<br>*Response constrained to: .>0*	

A. Consumables - Shock (8)
Group relevant when: selected(${section_four_skip_con} , '1')

Field	Question	Answer
note_401_suggest_other_etc	***Other supplies - suggestions?***	

q401_C49	q401_C49. "[q101_C49_full_list_other]"<br>*Question relevant when: selected(${q101_C49_full_list} , '3')*	
q401_C49a *(required)*	q401_C49a. What percent of patients require this item?<br>*Question relevant when: selected(${q101_C49_full_list} , '3')*<br>*Response constrained to: .>0 and .<=100 or .=999*	
q401_C49b *(required)*	q401_C49b. How many units of this item are required per patient (throughout the entire treatment provided for complication)?<br>*Question relevant when: selected(${q101_C49_full_list} , '3')*<br>*Response constrained to: .>0*	
q401_C50	q401_C50. "[q101_C50_full_list_other]"<br>*Question relevant when: selected(${q101_C50_full_list} , '3')*	
q401_C50a *(required)*	q401_C50a. What percent of patients require this item?<br>*Question relevant when: selected(${q101_C50_full_list} , '3')*<br>*Response constrained to: .>0 and .<=100 or .=999*	
q401_C50b *(required)*	q401_C50b. How many units of this item are required per patient (throughout the entire treatment provided for complication)?<br>*Question relevant when: selected(${q101_C50_full_list} , '3')*<br>*Response constrained to: .>0*	
q401_C51	"[q101_C51_full_list_other]"<br>*Question relevant when: selected(${q101_C51_full_list} , '3')*	
q401_C51a *(required)*	q401_C51a. What percent of patients require this item?<br>*Question relevant when: selected(${q101_C51_full_list} , '3')*<br>*Response constrained to: .>0 and .<=100 or .=999*	
q401_C51b *(required)*	q401_C51b. How many units of this item are required per patient (throughout the entire treatment provided for complication)?<br>*Question relevant when: selected(${q101_C51_full_list} , '3')*<br>*Response constrained to: .>0*	
q401_C52	q401_C52. "[q101_C52_full_list_other]"<br>*Question relevant when: selected(${q101_C52_full_list} , '3')*	
q401_C52a *(required)*	q401_C52a. What percent of patients require this item?<br>*Question relevant when: selected(${q101_C52_full_list} , '3')*<br>*Response constrained to: .>0 and .<=100 or .=999*	
q401_C52b *(required)*	q401_C52b. How many units of this item are required per patient (throughout the entire treatment provided for complication)?<br>*Question relevant when: selected(${q101_C52_full_list} , '3')*<br>*Response constrained to: .>0*	
q401_C53	q401_C53. "[q101_C53_full_list_other]"<br>*Question relevant when: selected(${q101_C53_full_list} , '3')*	
q401_C53a *(required)*	q401_C53a. What percent of patients require this item?<br>*Question relevant when: selected(${q101_C53_full_list} , '3')*<br>*Response constrained to: .>0 and .<=100 or .=999*	
q401_C53b *(required)*	q401_C53b. How many units of this item are required per patient (throughout the entire treatment provided for complication)?<br>*Question relevant when: selected(${q101_C53_full_list} , '3')*<br>*Response constrained to: .>0*	
group_section_five_intro		
section5_start	**SECTION V. LACERATIONS - USAGE OF ALL ITEMS**	
section_five_skip_con *(required)*	INTERVIEWER: WOULD YOU LIKE TO COMPLETE THIS SECTION NOW OR SKIP THIS SECTION AND RETURN TO IT LATER?<br>*You may need to skip if the participant has indicated that s/he cannot answer the questions in this section.*	1 Do not skip, complete this section now.<br>2 Skip and come back to this section later.
group_section_five_introB<br>*Group relevant when: selected(${section_five_skip_con} , '1')*		
section5_start2	In this section of the interview, we will review all of the items that you said are used for management of cervical or vaginal lacerations. For each item that is used, I'm going to ask questions on how many women need it and how much of it is used.	
section5_start3	INTERVIEWER: ENTER WHOLE NUMBERS OR DECIMALS. DO NOT TYPE PERCENT SIGNS. ENTER 999 FOR ANY THAT ARE UNKNOWN.	

A. Consumables - Lacerations (1)

Field	Question	Answer
	Group relevant when: selected(${section_five_skip_con} , '1')	
note_501_cleaning	***Cleaning, disinfectants, sterilization, etc.***	
q501_C1	q501_C1. Alcohol, denatured 70%<br>*Question relevant when: selected(${q101_C1_full_list} , '4')*	

q501_C1a *(required)*	q501_C1a. What percent of patients require this item?<br>*Question relevant when: selected(${q101_C1_full_list} , '4')*<br>*Response constrained to: .>0 and .<=100 or .=999*	
q501_C1b *(required)*	q501_C1b. How many units of this item are required per patient (throughout the entire treatment provided for complication)?<br>*Smallest unit is mililiter (ml)*<br>*Question relevant when: selected(${q101_C1_full_list} , '4')*<br>*Response constrained to: .>0*	
q501_C2	q501_C2. Chlorine (JIK) or Glutaraldehyde solution<br>*Question relevant when: selected(${q101_C2_full_list} , '4')*	
q501_C2a *(required)*	q501_C2a. What percent of patients require this item?<br>*Question relevant when: selected(${q101_C2_full_list} , '4')*<br>*Response constrained to: .>0 and .<=100 or .=999*	
q501_C2b *(required)*	q501_C2b. How many units of this item are required per patient (throughout the entire treatment provided for complication)?<br>*Smallest unit is mililiter (ml)*<br>*Question relevant when: selected(${q101_C2_full_list} , '4')*<br>*Response constrained to: .>0*	
q501_C3	q501_C3. Clean water<br>*Question relevant when: selected(${q101_C3_full_list} , '4')*	
q501_C3a *(required)*	q501_C3a. What percent of patients require this item?<br>*Question relevant when: selected(${q101_C3_full_list} , '4')*<br>*Response constrained to: .>0 and .<=100 or .=999*	
q501_C3b *(required)*	q501_C3b. How many units of this item are required per patient (throughout the entire treatment provided for complication)?<br>*Smallest unit is litre (ltr)*<br>*Question relevant when: selected(${q101_C3_full_list} , '4')*<br>*Response constrained to: .>0*	
q501_C4	q501_C4. Dettol solution or other water-based antiseptic<br>*Question relevant when: selected(${q101_C4_full_list} , '4')*	
q501_C4a *(required)*	q501_C4a. What percent of patients require this item?<br>*Question relevant when: selected(${q101_C4_full_list} , '4')*<br>*Response constrained to: .>0 and .<=100 or .=999*	
q501_C4b *(required)*	q501_C4b. How many units of this item are required per patient (throughout the entire treatment provided for complication)?<br>*Smallest unit is mililiter (ml)*<br>*Question relevant when: selected(${q101_C4_full_list} , '4')*<br>*Response constrained to: .>0*	
q501_C5	q501_C5. Cetrimide 15% ("Savlon")<br>*Question relevant when: selected(${q101_C5_full_list} , '4')*	
q501_C5a *(required)*	q501_C5a. What percent of patients require this item?<br>*Question relevant when: selected(${q101_C5_full_list} , '4')*<br>*Response constrained to: .>0 and .<=100 or .=999*	
q501_C5b *(required)*	q501_C5b. How many units of this item are required per patient (throughout the entire treatment provided for complication)?<br>*Smallest unit is mililiter (ml)*<br>*Question relevant when: selected(${q101_C5_full_list} , '4')*<br>*Response constrained to: .>0*	
q501_C6	q501_C6. Hand wash (liquid)<br>*Question relevant when: selected(${q101_C6_full_list} , '4')*	
q501_C6a *(required)*	q501_C6a. What percent of patients require this item?<br>*Question relevant when: selected(${q101_C6_full_list} , '4')*<br>*Response constrained to: .>0 and .<=100 or .=999*	
q501_C6b *(required)*	q501_C6b. How many units of this item are required per patient (throughout the entire treatment provided for complication)?<br>*Smallest unit is mililiter (ml)*<br>*Question relevant when: selected(${q101_C6_full_list} , '4')*<br>*Response constrained to: .>0*	
q501_C7	q501_C7. Soap (bar)<br>*Question relevant when: selected(${q101_C7_full_list} , '4')*	
q501_C7a *(required)*	q501_C7a. What percent of patients require this item?	

Field	Question	Answer
	Question relevant when: selected(${q101_C7_full_list} , '4')<br>*Response constrained to: .>0 and .<=100 or .=999*	
q501_C7b *(required)*	q501_C7b. How many units of this item are required per patient (throughout the entire treatment provided for complication)?<br>*Smallest unit is 1 bar of soap*<br>*Question relevant when: selected(${q101_C7_full_list} , '4')*<br>*Response constrained to: .>0*	
q501_C8	q501_C8. Hand sterilizer (waterless)<br>*Question relevant when: selected(${q101_C8_full_list} , '4')*	
q501_C8a *(required)*	q501_C8a. What percent of patients require this item?<br>*Question relevant when: selected(${q101_C8_full_list} , '4')*<br>*Response constrained to: .>0 and .<=100 or .=999*	
q501_C8b *(required)*	q501_C8b. How many units of this item are required per patient (throughout the entire treatment provided for complication)?<br>*Smallest unit is mililiter (ml)*<br>*Question relevant when: selected(${q101_C8_full_list} , '4')*<br>*Response constrained to: .>0*	
A. Consumables - Lacerations (2)<br>*Group relevant when: selected(${section_five_skip_con} , '1')*		
IV_needle_syringe	***IV, needles, syringes, etc.***	
q501_C9	q501_C9. Blood giving set with needle<br>*Question relevant when: selected(${q101_C9_full_list} , '4')*	
q501_C9a *(required)*	q501_C9a. What percent of patients require this item?<br>*Question relevant when: selected(${q101_C9_full_list} , '4')*<br>*Response constrained to: .>0 and .<=100 or .=999*	
q501_C9b *(required)*	q501_C9b. How many units of this item are required per patient (throughout the entire treatment provided for complication)?<br>*Smallest unit is one set.*<br>*Question relevant when: selected(${q101_C9_full_list} , '4')*<br>*Response constrained to: .>0*	
q501_C10	q501_C10. IV cannula<br>*Question relevant when: selected(${q101_C10_full_list} , '4')*	
q501_C10a *(required)*	q501_C10a. What percent of patients require this item?<br>*Question relevant when: selected(${q101_C10_full_list} , '4')*<br>*Response constrained to: .>0 and .<=100 or .=999*	
q501_C10b *(required)*	q501_C10b. How many units of this item are required per patient (throughout the entire treatment provided for complication)?<br>*Smallest unit is one cannula.*<br>*Question relevant when: selected(${q101_C10_full_list} , '4')*<br>*Response constrained to: .>0*	
q501_C11	q501_C11. IV set<br>*Question relevant when: selected(${q101_C11_full_list} , '4')*	
q501_C11a *(required)*	q501_C11a. What percent of patients require this item?<br>*Question relevant when: selected(${q101_C11_full_list} , '4')*<br>*Response constrained to: .>0 and .<=100 or .=999*	
q501_C11b *(required)*	q501_C11b. How many units of this item are required per patient (throughout the entire treatment provided for complication)?<br>*Smallest unit is one set.*<br>*Question relevant when: selected(${q101_C11_full_list} , '4')*<br>*Response constrained to: .>0*	
q501_C12	q501_C12. Needle luer tip 18Gx1.1/2" (1.2x38mm)<br>*Question relevant when: selected(${q101_C12_full_list} , '4')*	
q501_C12a *(required)*	q501_C12a. What percent of patients require this item?<br>*Question relevant when: selected(${q101_C12_full_list} , '4')*<br>*Response constrained to: .>0 and .<=100 or .=999*	
q501_C12b *(required)*	q501_C12b. How many units of this item are required per patient (throughout the entire treatment provided for complication)?<br>*Smallest unit is one needle.*<br>*Question relevant when: selected(${q101_C12_full_list} , '4')*<br>*Response constrained to: .>0*	
q501_C13	q501_C13. Needle luer tip 21Gx1.1/2" (0.8x38mm)<br>*Question relevant when: selected(${q101_C13_full_list} , '4')*	
q501_C13a *(required)*	q501_C13a. What percent of patients require this item?	

Field	Question	Answer
	Question relevant when: selected(${q101_C13_full_list} , '4')<br>*Response constrained to: .>0 and .<=100 or .=999*	
q501_C13b *(required)*	q501_C13b. How many units of this item are required per patient (throughout the entire treatment provided for complication)?<br>*Smallest unit is one needle.*<br>*Question relevant when: selected(${q101_C13_full_list} , '4')*<br>*Response constrained to: .>0*	
q501_C14	q501_C14. Needle, suture, round body<br>*Question relevant when: selected(${q101_C14_full_list} , '4')*	
q501_C14a *(required)*	q501_C14a. What percent of patients require this item?<br>*Question relevant when: selected(${q101_C14_full_list} , '4')*<br>*Response constrained to: .>0 and .<=100 or .=999*	
q501_C14b *(required)*	q501_C14b. How many units of this item are required per patient (throughout the entire treatment provided for complication)?<br>*Smallest unit is one needle.*<br>*Question relevant when: selected(${q101_C14_full_list} , '4')*<br>*Response constrained to: .>0*	
q501_C15	q501_C15. Spinal needle<br>*Question relevant when: selected(${q101_C15_full_list} , '4')*	
q501_C15a *(required)*	q501_C15a. What percent of patients require this item?<br>*Question relevant when: selected(${q101_C15_full_list} , '4')*<br>*Response constrained to: .>0 and .<=100 or .=999*	
q501_C15b *(required)*	q501_C15b. How many units of this item are required per patient (throughout the entire treatment provided for complication)?<br>*Smallest unit is one needle.*<br>*Question relevant when: selected(${q101_C15_full_list} , '4')*<br>*Response constrained to: .>0*	
q501_C16	q501_C16. Syringe 10ml, with bypacked needle 21Gx1.5"<br>*Question relevant when: selected(${q101_C16_full_list} , '4')*	
q501_C16a *(required)*	q501_C16a. What percent of patients require this item?<br>*Question relevant when: selected(${q101_C16_full_list} , '4')*<br>*Response constrained to: .>0 and .<=100 or .=999*	
q501_C16b *(required)*	q501_C16b. How many units of this item are required per patient (throughout the entire treatment provided for complication)?<br>*Smallest unit is one syringe.*<br>*Question relevant when: selected(${q101_C16_full_list} , '4')*<br>*Response constrained to: .>0*	
q501_C17	q501_C17. Syringe 5ml, with bypacked needle 21Gx1.5"<br>*Question relevant when: selected(${q101_C17_full_list} , '4')*	
q501_C17a *(required)*	q501_C17a. What percent of patients require this item?<br>*Question relevant when: selected(${q101_C17_full_list} , '4')*<br>*Response constrained to: .>0 and .<=100 or .=999*	
q501_C17b *(required)*	q501_C17b. How many units of this item are required per patient (throughout the entire treatment provided for complication)?<br>*Smallest unit is one syringe.*<br>*Question relevant when: selected(${q101_C17_full_list} , '4')*<br>*Response constrained to: .>0*	
q501_C18	q501_C18. Syringe luer tip 10ml, no needle<br>*Question relevant when: selected(${q101_C18_full_list} , '4')*	
q501_C18a *(required)*	q501_C18a. What percent of patients require this item?<br>*Question relevant when: selected(${q101_C18_full_list} , '4')*<br>*Response constrained to: .>0 and .<=100 or .=999*	
q501_C18b *(required)*	q501_C18b. How many units of this item are required per patient (throughout the entire treatment provided for complication)?<br>*Smallest unit is one syringe.*<br>*Question relevant when: selected(${q101_C18_full_list} , '4')*<br>*Response constrained to: .>0*	
q501_C19	q501_C19. Syringe luer tip 5ml, no needle<br>*Question relevant when: selected(${q101_C19_full_list} , '4')*	
q501_C19a *(required)*	q501_C19a. What percent of patients require this item?<br>*Question relevant when: selected(${q101_C19_full_list} , '4')*<br>*Response constrained to: .>0 and .<=100 or .=999*	
q501_C19b *(required)*	q501_C19b. How many units of this item are required per patient (throughout the entire treatment provided for	

Field	Question	Answer
	complication)?<br>*Smallest unit is one syringe.*<br>*Question relevant when: selected(${q101_C19_full_list} , '4')*<br>*Response constrained to: .>0*	

q501_C20	q501_C20. Venous catheter G18 + injection port and lock tip<br>*Question relevant when: selected(${q101_C20_full_list} , '4')*	
q501_C20a *(required)*	q501_C20a. What percent of patients require this item?<br>*Question relevant when: selected(${q101_C20_full_list} , '4')*<br>*Response constrained to: .>0 and .<=100 or .=999*	
q501_C20b *(required)*	q501_C20b. How many units of this item are required per patient (throughout the entire treatment provided for complication)?<br>*Smallest unit is one catheter.*<br>*Question relevant when: selected(${q101_C20_full_list} , '4')*<br>*Response constrained to: .>0*	
A. Consumables - Lacerations (3)<br>*Group relevant when: selected(${section_five_skip_con} , '1')*		
note_501_bandages_etc	***Bandages, absorbants, wound care, etc.***	
q501_C21	q501_C21. Adhesive tape<br>*Question relevant when: selected(${q101_C21_full_list} , '4')*	
q501_C21a *(required)*	q501_C21a. What percent of patients require this item?<br>*Question relevant when: selected(${q101_C21_full_list} , '4')*<br>*Response constrained to: .>0 and .<=100 or .=999*	
q501_C21b *(required)*	q501_C21b. How many units of this item are required per patient (throughout the entire treatment provided for complication)?<br>*Smallest unit is one roll of tape.*<br>*Question relevant when: selected(${q101_C21_full_list} , '4')*<br>*Response constrained to: .>0*	
q501_C22	q501_C22. Cotton swab<br>*Question relevant when: selected(${q101_C22_full_list} , '4')*	
q501_C22a *(required)*	q501_C22a. What percent of patients require this item?<br>*Question relevant when: selected(${q101_C22_full_list} , '4')*<br>*Response constrained to: .>0 and .<=100 or .=999*	
q501_C22b *(required)*	q501_C22b. How many units of this item are required per patient (throughout the entire treatment provided for complication)?<br>*Smallest unit is one swab.*<br>*Question relevant when: selected(${q101_C22_full_list} , '4')*<br>*Response constrained to: .>0*	
q501_C23	q501_C23. Dressing pad absorbent sterile<br>*Question relevant when: selected(${q101_C23_full_list} , '4')*	
q501_C23a *(required)*	q501_C23a. What percent of patients require this item?<br>*Question relevant when: selected(${q101_C23_full_list} , '4')*<br>*Response constrained to: .>0 and .<=100 or .=999*	
q501_C23b *(required)*	q501_C23b. How many units of this item are required per patient (throughout the entire treatment provided for complication)?<br>*Smallest unit is one pad.*<br>*Question relevant when: selected(${q101_C23_full_list} , '4')*<br>*Response constrained to: .>0*	
q501_C24	q501_C24. Gauze compress, non sterile<br>*Question relevant when: selected(${q101_C24_full_list} , '4')*	
q501_C24a *(required)*	q501_C24a. What percent of patients require this item?<br>*Question relevant when: selected(${q101_C24_full_list} , '4')*<br>*Response constrained to: .>0 and .<=100 or .=999*	
q501_C24b *(required)*	q501_C24b. How many units of this item are required per patient (throughout the entire treatment provided for complication)?<br>*Smallest unit is one compress.*<br>*Question relevant when: selected(${q101_C24_full_list} , '4')*<br>*Response constrained to: .>0*	
q501_C25	q501_C25. Gauze compress, sterile<br>*Question relevant when: selected(${q101_C25_full_list} , '4')*	
q501_C25a *(required)*	q501_C25a. What percent of patients require this item?<br>*Question relevant when: selected(${q101_C25_full_list} , '4')*<br>*Response constrained to: .>0 and .<=100 or .=999*	
q501_C25b *(required)*	q501_C25b. How many units of this item are required per patient (throughout the entire treatment provided for complication)?<br>*Smallest unit is one compress.*	

Field	Question	Answer
	Question relevant when: selected(${q101_C25_full_list} , '4')<br>*Response constrained to: .>0*	
q501_C26	q501_C26. Linen saver<br>*Question relevant when: selected(${q101_C26_full_list} , '4')*	

q501_C26a *(required)*	q501_C26a. What percent of patients require this item?<br>*Question relevant when: selected(${q101_C26_full_list} , '4')*<br>*Response constrained to: .>0 and .<=100 or .=999*	
q501_C26b *(required)*	q501_C26b. How many units of this item are required per patient (throughout the entire treatment provided for complication)?<br>*Smallest unit is one saver (absorbant sheet).*<br>*Question relevant when: selected(${q101_C26_full_list} , '4')*<br>*Response constrained to: .>0*	
q501_C27	q501_C27. Sanitary Pad<br>*Question relevant when: selected(${q101_C27_full_list} , '4')*	
q501_C27a *(required)*	q501_C27a. What percent of patients require this item?<br>*Question relevant when: selected(${q101_C27_full_list} , '4')*<br>*Response constrained to: .>0 and .<=100 or .=999*	
q501_C27b *(required)*	q501_C27b. How many units of this item are required per patient (throughout the entire treatment provided for complication)?<br>*Smallest unit is one pad.*<br>*Question relevant when: selected(${q101_C27_full_list} , '4')*<br>*Response constrained to: .>0*	
q501_C28	q501_C28. Surgical plaster<br>*Question relevant when: selected(${q101_C28_full_list} , '4')*	
q501_C28a *(required)*	q501_C28a. What percent of patients require this item?<br>*Question relevant when: selected(${q101_C28_full_list} , '4')*<br>*Response constrained to: .>0 and .<=100 or .=999*	
q501_C28b *(required)*	q501_C28b. How many units of this item are required per patient (throughout the entire treatment provided for complication)?<br>*Smallest unit is one plaster.*<br>*Question relevant when: selected(${q101_C28_full_list} , '4')*<br>*Response constrained to: .>0*	
A. Consumables - Lacerations (4)<br>*Group relevant when: selected(${section_five_skip_con} , '1')*		
note_501_protective_etc	***Protective supplies***	
q501_C29	q501_C29. Apron (disposable)<br>*Question relevant when: selected(${q101_C29_full_list} , '4')*	
q501_C29a *(required)*	q501_C29a. What percent of patients require this item?<br>*Question relevant when: selected(${q101_C29_full_list} , '4')*<br>*Response constrained to: .>0 and .<=100 or .=999*	
q501_C29b *(required)*	q501_C29b. How many units of this item are required per patient (throughout the entire treatment provided for complication)?<br>*Smallest unit is one apron.*<br>*Question relevant when: selected(${q101_C29_full_list} , '4')*<br>*Response constrained to: .>0*	
q501_C30	q501_C30. Gown (disposable)<br>*Question relevant when: selected(${q101_C30_full_list} , '4')*	
q501_C30a *(required)*	q501_C30a. What percent of patients require this item?<br>*Question relevant when: selected(${q101_C30_full_list} , '4')*<br>*Response constrained to: .>0 and .<=100 or .=999*	
q501_C30b *(required)*	q501_C30b. How many units of this item are required per patient (throughout the entire treatment provided for complication)?<br>*Smallest unit is one gown.*<br>*Question relevant when: selected(${q101_C30_full_list} , '4')*<br>*Response constrained to: .>0*	
q501_C31	q501_C31. Latex examination glove, without powder<br>*Question relevant when: selected(${q101_C31_full_list} , '4')*	
q501_C31a *(required)*	q501_C31a. What percent of patients require this item?<br>*Question relevant when: selected(${q101_C31_full_list} , '4')*<br>*Response constrained to: .>0 and .<=100 or .=999*	
q501_C31b *(required)*	q501_C31b. How many units of this item are required per patient (throughout the entire treatment provided for complication)?<br>*Smallest unit is one glove.*<br>*Question relevant when: selected(${q101_C31_full_list} , '4')*<br>*Response constrained to: .>0*	

Field	Question	Answer
q501_C32	q501_C32. Latex examination glove, with powder<br>*Question relevant when: selected(${q101_C32_full_list} , '4')*	
q501_C32a *(required)*	q501_C32a. What percent of patients require this item?<br>*Question relevant when: selected(${q101_C32_full_list} , '4')*<br>*Response constrained to: .>0 and .<=100 or .=999*	
q501_C32b *(required)*	q501_C32b. How many units of this item are required per patient (throughout the entire treatment provided for complication)?<br>*Smallest unit is one glove.*<br>*Question relevant when: selected(${q101_C32_full_list} , '4')*<br>*Response constrained to: .>0*	
q501_C33	q501_C33. Non-latex (e.g. nitrile) examination glove<br>*Question relevant when: selected(${q101_C33_full_list} , '4')*	
q501_C33a *(required)*	q501_C33a. What percent of patients require this item?<br>*Question relevant when: selected(${q101_C33_full_list} , '4')*<br>*Response constrained to: .>0 and .<=100 or .=999*	
q501_C33b *(required)*	q501_C33b. How many units of this item are required per patient (throughout the entire treatment provided for complication)?<br>*Smallest unit is one glove.*<br>*Question relevant when: selected(${q101_C33_full_list} , '4')*<br>*Response constrained to: .>0*	
q501_C34	q501_C34. Sterile surgical glove<br>*Question relevant when: selected(${q101_C34_full_list} , '4')*	
q501_C34a *(required)*	q501_C34a. What percent of patients require this item?<br>*Question relevant when: selected(${q101_C34_full_list} , '4')*<br>*Response constrained to: .>0 and .<=100 or .=999*	
q501_C34b *(required)*	q501_C34b. How many units of this item are required per patient (throughout the entire treatment provided for complication)?<br>*Smallest unit is one glove.*<br>*Question relevant when: selected(${q101_C34_full_list} , '4')*<br>*Response constrained to: .>0*	
q501_C35	q501_C35. Mask (disposable)<br>*Question relevant when: selected(${q101_C35_full_list} , '4')*	
q501_C35a *(required)*	q501_C35a. What percent of patients require this item?<br>*Question relevant when: selected(${q101_C35_full_list} , '4')*<br>*Response constrained to: .>0 and .<=100 or .=999*	
q501_C35b *(required)*	q501_C35b. How many units of this item are required per patient (throughout the entire treatment provided for complication)?<br>*Smallest unit is one mask.*<br>*Question relevant when: selected(${q101_C35_full_list} , '4')*<br>*Response constrained to: .>0*	
q501_C36	q501_C36. Surgical cap<br>*Question relevant when: selected(${q101_C36_full_list} , '4')*	
q501_C36a *(required)*	q501_C36a. What percent of patients require this item?<br>*Question relevant when: selected(${q101_C36_full_list} , '4')*<br>*Response constrained to: .>0 and .<=100 or .=999*	
q501_C36b *(required)*	q501_C36b. How many units of this item are required per patient (throughout the entire treatment provided for complication)?<br>*Smallest unit is one cap.*<br>*Question relevant when: selected(${q101_C36_full_list} , '4')*<br>*Response constrained to: .>0*	
A. Consumables - Lacerations (5)<br>*Group relevant when: selected(${section_five_skip_con} , '1')*		
note_501_surgery_etc	***Surgery supplies***	
q501_C37	q501_C37. Scalpel blade, single use<br>*Question relevant when: selected(${q101_C37_full_list} , '4')*	
q501_C37a *(required)*	q501_C37a. What percent of patients require this item?<br>*Question relevant when: selected(${q101_C37_full_list} , '4')*<br>*Response constrained to: .>0 and .<=100 or .=999*	
q501_C37b *(required)*	q501_C37b. How many units of this item are required per patient (throughout the entire treatment provided for complication)?<br>*Smallest unit is one blade.*<br>*Question relevant when: selected(${q101_C37_full_list} , '4')*<br>*Response constrained to: .>0*	

Field	Question	Answer
q501_C38	q501_C38. Surgical drain<br>*Question relevant when: selected(${q101_C38_full_list} , '4')*	
q501_C38a *(required)*	q501_C38a. What percent of patients require this item?<br>*Question relevant when: selected(${q101_C38_full_list} , '4')*<br>*Response constrained to: .>0 and .<=100 or .=999*	
q501_C38b *(required)*	q501_C38b. How many units of this item are required per patient (throughout the entire treatment provided for complication)?<br>*Smallest unit is one drain.*<br>*Question relevant when: selected(${q101_C38_full_list} , '4')*<br>*Response constrained to: .>0*	
q501_C39	q501_C39. Suture string, chromic catgut (0)<br>*Question relevant when: selected(${q101_C39_full_list} , '4')*	
q501_C39a *(required)*	q501_C39a. What percent of patients require this item?<br>*Question relevant when: selected(${q101_C39_full_list} , '4')*<br>*Response constrained to: .>0 and .<=100 or .=999*	
q501_C39b *(required)*	q501_C39b. How many units of this item are required per patient (throughout the entire treatment provided for complication)?<br>*Smallest unit is one suture set.*<br>*Question relevant when: selected(${q101_C39_full_list} , '4')*<br>*Response constrained to: .>0*	
q501_C40	q501_C40. Suture string, silk (non absorbable and non-synthetic)<br>*Question relevant when: selected(${q101_C40_full_list} , '4')*	
q501_C40a *(required)*	q501_C40a. What percent of patients require this item?<br>*Question relevant when: selected(${q101_C40_full_list} , '4')*<br>*Response constrained to: .>0 and .<=100 or .=999*	
q501_C40b *(required)*	q501_C40b. How many units of this item are required per patient (throughout the entire treatment provided for complication)?<br>*Smallest unit is one suture set.*<br>*Question relevant when: selected(${q101_C40_full_list} , '4')*<br>*Response constrained to: .>0*	
q501_C41	q501_C41. Suture string, vicryl, no needle included<br>*Question relevant when: selected(${q101_C41_full_list} , '4')*	
q501_C41a *(required)*	q501_C41a. What percent of patients require this item?<br>*Question relevant when: selected(${q101_C41_full_list} , '4')*<br>*Response constrained to: .>0 and .<=100 or .=999*	
q501_C41b *(required)*	q501_C41b. How many units of this item are required per patient (throughout the entire treatment provided for complication)?<br>*Smallest unit is one suture set.*<br>*Question relevant when: selected(${q101_C41_full_list} , '4')*<br>*Response constrained to: .>0*	
q501_C42	q501_C42. Suture string, vicryl with needle<br>*Question relevant when: selected(${q101_C42_full_list} , '4')*	
q501_C42a *(required)*	q501_C42a. What percent of patients require this item?<br>*Question relevant when: selected(${q101_C42_full_list} , '4')*<br>*Response constrained to: .>0 and .<=100 or .=999*	
q501_C42b *(required)*	q501_C42b. How many units of this item are required per patient (throughout the entire treatment provided for complication)?<br>*Smallest unit is one suture set with needle..*<br>*Question relevant when: selected(${q101_C42_full_list} , '4')*<br>*Response constrained to: .>0*	
A. Consumables - Lacerations (6)<br>*Group relevant when: selected(${section_five_skip_con} , '1')*		
note_501_urine_etc	***Urine, bladder***	
q501_C43	q501_C43. Bladder/urinary catheter<br>*Question relevant when: selected(${q101_C43_full_list} , '4')*	
q501_C43a *(required)*	q501_C43a. What percent of patients require this item?<br>*Question relevant when: selected(${q101_C43_full_list} , '4')*<br>*Response constrained to: .>0 and .<=100 or .=999*	
q501_C43b *(required)*	q501_C43b. How many units of this item are required per patient (throughout the entire treatment provided for complication)?<br>*Smallest unit is one catheter.*<br>*Question relevant when: selected(${q101_C43_full_list} , '4')*<br>*Response constrained to: .>0*	

Field	Question	Answer
q501_C44	q501_C44. Foley's catheter<br>*Question relevant when: selected(${q101_C44_full_list} , '4')*	
q501_C44a *(required)*	q501_C44a. What percent of patients require this item?<br>*Question relevant when: selected(${q101_C44_full_list} , '4')*<br>*Response constrained to: .>0 and .<=100 or .=999*	
q501_C44b *(required)*	q501_C44b. How many units of this item are required per patient (throughout the entire treatment provided for complication)?<br>*Smallest unit is one catheter.*<br>*Question relevant when: selected(${q101_C44_full_list} , '4')*<br>*Response constrained to: .>0*	
q501_C45	q501_C45. Urine bag with valve and drain<br>*Question relevant when: selected(${q101_C45_full_list} , '4')*	
q501_C45a *(required)*	q501_C45a. What percent of patients require this item?<br>*Question relevant when: selected(${q101_C45_full_list} , '4')*<br>*Response constrained to: .>0 and .<=100 or .=999*	
q501_C45b *(required)*	q501_C45b. How many units of this item are required per patient (throughout the entire treatment provided for complication)?<br>*Smallest unit is one bag.*<br>*Question relevant when: selected(${q101_C45_full_list} , '4')*<br>*Response constrained to: .>0*	
A. Consumables - Lacerations (7)<br>*Group relevant when: selected(${section_five_skip_con} , '1')*		
note_501_other_etc	***Other supplies***	
q501_C46	q501_C46. Silicone oil for lubrication of MVA aspirator<br>*Question relevant when: selected(${q101_C46_full_list} , '4')*	
q501_C46a *(required)*	q501_C46a. What percent of patients require this item?<br>*Question relevant when: selected(${q101_C46_full_list} , '4')*<br>*Response constrained to: .>0 and .<=100 or .=999*	
q501_C46b *(required)*	q501_C46b. How many units of this item are required per patient (throughout the entire treatment provided for complication)?<br>*Smallest unit is milliliter (ml)*<br>*Question relevant when: selected(${q101_C46_full_list} , '4')*<br>*Response constrained to: .>0*	
q501_C47	q501_C47. Speculum, BValve/Cuscos (reusable)<br>*Question relevant when: selected(${q101_C47_full_list} , '4')*	
q501_C47a *(required)*	q501_C47a. What percent of patients require this item?<br>*Question relevant when: selected(${q101_C47_full_list} , '4')*<br>*Response constrained to: .>0 and .<=100 or .=999*	
q501_C47b *(required)*	q501_C47b. How many units of this item are required per patient (throughout the entire treatment provided for complication)?<br>*Smallest unit is one speculum.*<br>*Question relevant when: selected(${q101_C47_full_list} , '4')*<br>*Response constrained to: .>0*	
q501_C48	q501_C48. Speculum, Sim's (disposable)<br>*Question relevant when: selected(${q101_C48_full_list} , '4')*	
q501_C48a *(required)*	q501_C48a. What percent of patients require this item?<br>*Question relevant when: selected(${q101_C48_full_list} , '4')*<br>*Response constrained to: .>0 and .<=100 or .=999*	
q501_C48b *(required)*	q501_C48b. How many units of this item are required per patient (throughout the entire treatment provided for complication)?<br>*Smallest unit is one speculum.*<br>*Question relevant when: selected(${q101_C48_full_list} , '4')*<br>*Response constrained to: .>0*	
A. Consumables - Lacerations (8)<br>*Group relevant when: selected(${section_five_skip_con} , '1')*		
note_501_suggest_other_etc	***Other supplies - suggestions?***	
q501_C49	q501_C49. "[q101_C49_full_list_other]"<br>*Question relevant when: selected(${q101_C49_full_list} , '4')*	
q501_C49a *(required)*	q501_C49a. What percent of patients require this item?<br>*Question relevant when: selected(${q101_C49_full_list} , '4')*<br>*Response constrained to: .>0 and .<=100 or .=999*	
q501_C49b *(required)*	q501_C49b. How many units of this item are required per patient (throughout the entire treatment provided for complication)?	

Field	Question	Answer
	Question relevant when: selected(${q101_C49_full_list} , '4')<br>*Response constrained to: .>0*	
q501_C50	q501_C50. "[q101_C50_full_list_other]"<br>*Question relevant when: selected(${q101_C50_full_list} , '4')*	
q501_C50a *(required)*	q501_C50a. What percent of patients require this item?<br>*Question relevant when: selected(${q101_C50_full_list} , '4')*<br>*Response constrained to: .>0 and .<=100 or .=999*	
q501_C50b *(required)*	q501_C50b. How many units of this item are required per patient (throughout the entire treatment provided for complication)?<br>*Question relevant when: selected(${q101_C50_full_list} , '4')*<br>*Response constrained to: .>0*	
q501_C51	q501_C51. "[q101_C51_full_list_other]"<br>*Question relevant when: selected(${q101_C51_full_list} , '4')*	
q501_C51a *(required)*	q501_C51a. What percent of patients require this item?<br>*Question relevant when: selected(${q101_C51_full_list} , '4')*<br>*Response constrained to: .>0 and .<=100 or .=999*	
q501_C51b *(required)*	q501_C51b. How many units of this item are required per patient (throughout the entire treatment provided for complication)?<br>*Question relevant when: selected(${q101_C51_full_list} , '4')*<br>*Response constrained to: .>0*	
q501_C52	q501_C52. "[q101_C52_full_list_other]"<br>*Question relevant when: selected(${q101_C52_full_list} , '4')*	
q501_C52a *(required)*	q501_C52a. What percent of patients require this item?<br>*Question relevant when: selected(${q101_C52_full_list} , '4')*<br>*Response constrained to: .>0 and .<=100 or .=999*	
q501_C52b *(required)*	q501_C52b. How many units of this item are required per patient (throughout the entire treatment provided for complication)?<br>*Question relevant when: selected(${q101_C52_full_list} , '4')*<br>*Response constrained to: .>0*	
q501_C53	q501_C53. "[q101_C53_full_list_other]"<br>*Question relevant when: selected(${q101_C53_full_list} , '4')*	
q501_C53a *(required)*	q501_C53a. What percent of patients require this item?<br>*Question relevant when: selected(${q101_C53_full_list} , '4')*<br>*Response constrained to: .>0 and .<=100 or .=999*	
q501_C53b *(required)*	q501_C53b. How many units of this item are required per patient (throughout the entire treatment provided for complication)?<br>*Question relevant when: selected(${q101_C53_full_list} , '4')*<br>*Response constrained to: .>0*	
group_section_six_intro		
section6_start	**SECTION VI. PERFORATIONS - USAGE OF ALL ITEMS**	
section_six_skip_con *(required)*	INTERVIEWER: WOULD YOU LIKE TO COMPLETE THIS SECTION NOW OR SKIP THIS SECTION AND RETURN TO IT LATER?<br>*You may need to skip if the participant has indicated that s/he cannot answer the questions in this section.*	1 Do not skip, complete this section now.<br>2 Skip and come back to this section later.
group_section_six_introB<br>*Group relevant when: selected(${section_six_skip_con} , '1')*		
section6_start2	In this section of the interview, we will review all of the items that you said are used for management of vaginal or uterine perforations. For each item that is used, I'm going to ask questions on how many women need it and how much of it is used.	
section6_start3	INTERVIEWER: ENTER WHOLE NUMBERS OR DECIMALS. DO NOT TYPE PERCENT SIGNS. ENTER 999 FOR ANY THAT ARE UNKNOWN.	
A. Consumables - Perforations (1)<br>*Group relevant when: selected(${section_six_skip_con} , '1')*		
note_601_cleaning	***Cleaning, disinfectants, sterilization, etc.***	
q601_C1	q601_C1. Alcohol, denatured 70%<br>*Question relevant when: selected(${q101_C1_full_list} , '5')*	
q601_C1a *(required)*	q601_C1a. What percent of patients require this item?<br>*Question relevant when: selected(${q101_C1_full_list} , '5')*<br>*Response constrained to: .>0 and .<=100 or .=999*	
q601_C1b *(required)*	q601_C1b. How many units of this item are required per patient (throughout the entire treatment provided for complication)?<br>*Smallest unit is mililiter (ml)*<br>*Question relevant when: selected(${q101_C1_full_list} , '5')*<br>[illegible]	

Field	Question	Answer
	Response constrained to: .>0	
q601_C2	q601_C2. Chlorine (JIK) or Glutaraldehyde solution<br>*Question relevant when: selected(${q101_C2_full_list} , '5')*	

q601_C2a *(required)*	q601_C2a. What percent of patients require this item?<br>*Question relevant when: selected(${q101_C2_full_list} , '5')*<br>*Response constrained to: .>0 and .<=100 or .=999*	
q601_C2b *(required)*	q601_C2b. How many units of this item are required per patient (throughout the entire treatment provided for complication)?<br>*Smallest unit is mililiter (ml)*<br>*Question relevant when: selected(${q101_C2_full_list} , '5')*<br>*Response constrained to: .>0*	
q601_C3	q601_C3. Clean water<br>*Question relevant when: selected(${q101_C3_full_list} , '5')*	
q601_C3a *(required)*	q601_C3a. What percent of patients require this item?<br>*Question relevant when: selected(${q101_C3_full_list} , '5')*<br>*Response constrained to: .>0 and .<=100 or .=999*	
q601_C3b *(required)*	q601_C3b. How many units of this item are required per patient (throughout the entire treatment provided for complication)?<br>*Smallest unit is litre (ltr)*<br>*Question relevant when: selected(${q101_C3_full_list} , '5')*<br>*Response constrained to: .>0*	
q601_C4	q601_C4. Dettol solution or other water-based antiseptic<br>*Question relevant when: selected(${q101_C4_full_list} , '5')*	
q601_C4a *(required)*	q601_C4a. What percent of patients require this item?<br>*Question relevant when: selected(${q101_C4_full_list} , '5')*<br>*Response constrained to: .>0 and .<=100 or .=999*	
q601_C4b *(required)*	q601_C4b. How many units of this item are required per patient (throughout the entire treatment provided for complication)?<br>*Smallest unit is mililiter (ml)*<br>*Question relevant when: selected(${q101_C4_full_list} , '5')*<br>*Response constrained to: .>0*	
q601_C5	q601_C5. Cetrimide 15% ("Savlon")<br>*Question relevant when: selected(${q101_C5_full_list} , '5')*	
q601_C5a *(required)*	q601_C5a. What percent of patients require this item?<br>*Question relevant when: selected(${q101_C5_full_list} , '5')*<br>*Response constrained to: .>0 and .<=100 or .=999*	
q601_C5b *(required)*	q601_C5b. How many units of this item are required per patient (throughout the entire treatment provided for complication)?<br>*Smallest unit is mililiter (ml)*<br>*Question relevant when: selected(${q101_C5_full_list} , '5')*<br>*Response constrained to: .>0*	
q601_C6	q601_C6. Hand wash (liquid)<br>*Question relevant when: selected(${q101_C6_full_list} , '5')*	
q601_C6a *(required)*	q601_C6a. What percent of patients require this item?<br>*Question relevant when: selected(${q101_C6_full_list} , '5')*<br>*Response constrained to: .>0 and .<=100 or .=999*	
q601_C6b *(required)*	q601_C6b. How many units of this item are required per patient (throughout the entire treatment provided for complication)?<br>*Smallest unit is mililiter (ml)*<br>*Question relevant when: selected(${q101_C6_full_list} , '5')*<br>*Response constrained to: .>0*	
q601_C7	q601_C7. Soap (bar)<br>*Question relevant when: selected(${q101_C7_full_list} , '5')*	
q601_C7a *(required)*	q601_C7a. What percent of patients require this item?<br>*Question relevant when: selected(${q101_C7_full_list} , '5')*<br>*Response constrained to: .>0 and .<=100 or .=999*	
q601_C7b *(required)*	q601_C7b. How many units of this item are required per patient (throughout the entire treatment provided for complication)?<br>*Smallest unit is 1 bar of soap*<br>*Question relevant when: selected(${q101_C7_full_list} , '5')*<br>*Response constrained to: .>0*	
q601_C8	q601_C8. Hand sterilizer (waterless)<br>*Question relevant when: selected(${q101_C8_full_list} , '5')*	
q601_C8a *(required)*	q601_C8a. What percent of patients require this item?	

Field	Question	Answer
	Question relevant when: selected(${q101_C8_full_list} , '5')<br>*Response constrained to: .>0 and .<=100 or .=999*	
q601_C8b *(required)*	q601_C8b. How many units of this item are required per patient (throughout the entire treatment provided for complication)?<br>*Smallest unit is mililiter (ml)*<br>*Question relevant when: selected(${q101_C8_full_list} , '5')*<br>*Response constrained to: .>0*	
A. Consumables - Perforations (2)<br>*Group relevant when: selected(${section_six_skip_con} , '1')*		
note_601_IV_needle_syringe	***IV, needles, syringes, etc.***	
q601_C9	q601_C9. Blood giving set with needle<br>*Question relevant when: selected(${q101_C9_full_list} , '5')*	
q601_C9a *(required)*	q601_C9a. What percent of patients require this item?<br>*Question relevant when: selected(${q101_C9_full_list} , '5')*<br>*Response constrained to: .>0 and .<=100 or .=999*	
q601_C9b *(required)*	q601_C9b. How many units of this item are required per patient (throughout the entire treatment provided for complication)?<br>*Smallest unit is one set.*<br>*Question relevant when: selected(${q101_C9_full_list} , '5')*<br>*Response constrained to: .>0*	
q601_C10	q601_C10. IV cannula<br>*Question relevant when: selected(${q101_C10_full_list} , '5')*	
q601_C10a *(required)*	q601_C10a. What percent of patients require this item?<br>*Question relevant when: selected(${q101_C10_full_list} , '5')*<br>*Response constrained to: .>0 and .<=100 or .=999*	
q601_C10b *(required)*	q601_C10b. How many units of this item are required per patient (throughout the entire treatment provided for complication)?<br>*Smallest unit is one cannula.*<br>*Question relevant when: selected(${q101_C10_full_list} , '5')*<br>*Response constrained to: .>0*	
q601_C11	q601_C11. IV set<br>*Question relevant when: selected(${q101_C11_full_list} , '5')*	
q601_C11a *(required)*	q601_C11a. What percent of patients require this item?<br>*Question relevant when: selected(${q101_C11_full_list} , '5')*<br>*Response constrained to: .>0 and .<=100 or .=999*	
q601_C11b *(required)*	q601_C11b. How many units of this item are required per patient (throughout the entire treatment provided for complication)?<br>*Smallest unit is one set.*<br>*Question relevant when: selected(${q101_C11_full_list} , '5')*<br>*Response constrained to: .>0*	
q601_C12	q601_C12. Needle luer tip 18Gx1.1/2" (1.2x38mm)<br>*Question relevant when: selected(${q101_C12_full_list} , '5')*	
q601_C12a *(required)*	q601_C12a. What percent of patients require this item?<br>*Question relevant when: selected(${q101_C12_full_list} , '5')*<br>*Response constrained to: .>0 and .<=100 or .=999*	
q601_C12b *(required)*	q601_C12b. How many units of this item are required per patient (throughout the entire treatment provided for complication)?<br>*Smallest unit is one needle.*<br>*Question relevant when: selected(${q101_C12_full_list} , '5')*<br>*Response constrained to: .>0*	
q601_C13	q601_C13. Needle luer tip 21Gx1.1/2" (0.8x38mm)<br>*Question relevant when: selected(${q101_C13_full_list} , '5')*	
q601_C13a *(required)*	q601_C13a. What percent of patients require this item?<br>*Question relevant when: selected(${q101_C13_full_list} , '5')*<br>*Response constrained to: .>0 and .<=100 or .=999*	
q601_C13b *(required)*	q601_C13b. How many units of this item are required per patient (throughout the entire treatment provided for complication)?<br>*Smallest unit is one needle.*<br>*Question relevant when: selected(${q101_C13_full_list} , '5')*<br>*Response constrained to: .>0*	
q601_C14	q601_C14. Needle, suture, round body<br>*Question relevant when: selected(${q101_C14_full_list} , '5')*	
q601_C14a *(required)*	q601_C14a. What percent of patients require this item?	

Field	Question	Answer
	Question relevant when: selected(${q101_C14_full_list} , '5')<br>*Response constrained to: .>0 and .<=100 or .=999*	
q601_C14b *(required)*	q601_C14b. How many units of this item are required per patient (throughout the entire treatment provided for complication)?<br>*Smallest unit is one needle.*<br>*Question relevant when: selected(${q101_C14_full_list} , '5')*<br>*Response constrained to: .>0*	
q601_C15	q601_C15. Spinal needle<br>*Question relevant when: selected(${q101_C15_full_list} , '5')*	
q601_C15a *(required)*	q601_C15a. What percent of patients require this item?<br>*Question relevant when: selected(${q101_C15_full_list} , '5')*<br>*Response constrained to: .>0 and .<=100 or .=999*	
q601_C15b *(required)*	q601_C15b. How many units of this item are required per patient (throughout the entire treatment provided for complication)?<br>*Smallest unit is one needle.*<br>*Question relevant when: selected(${q101_C15_full_list} , '5')*<br>*Response constrained to: .>0*	
q601_C16	q601_C16. Syringe 10ml, with bypacked needle 21Gx1.5"<br>*Question relevant when: selected(${q101_C16_full_list} , '5')*	
q601_C16a *(required)*	q601_C16a. What percent of patients require this item?<br>*Question relevant when: selected(${q101_C16_full_list} , '5')*<br>*Response constrained to: .>0 and .<=100 or .=999*	
q601_C16b *(required)*	q601_C16b. How many units of this item are required per patient (throughout the entire treatment provided for complication)?<br>*Smallest unit is one syringe.*<br>*Question relevant when: selected(${q101_C16_full_list} , '5')*<br>*Response constrained to: .>0*	
q601_C17	q601_C17. Syringe 5ml, with bypacked needle 21Gx1.5"<br>*Question relevant when: selected(${q101_C17_full_list} , '5')*	
q601_C17a *(required)*	q601_C17a. What percent of patients require this item?<br>*Question relevant when: selected(${q101_C17_full_list} , '5')*<br>*Response constrained to: .>0 and .<=100 or .=999*	
q601_C17b *(required)*	q601_C17b. How many units of this item are required per patient (throughout the entire treatment provided for complication)?<br>*Smallest unit is one syringe.*<br>*Question relevant when: selected(${q101_C17_full_list} , '5')*<br>*Response constrained to: .>0*	
q601_C18	q601_C18. Syringe luer tip 10ml, no needle<br>*Question relevant when: selected(${q101_C18_full_list} , '5')*	
q601_C18a *(required)*	q601_C18a. What percent of patients require this item?<br>*Question relevant when: selected(${q101_C18_full_list} , '5')*<br>*Response constrained to: .>0 and .<=100 or .=999*	
q601_C18b *(required)*	q601_C18b. How many units of this item are required per patient (throughout the entire treatment provided for complication)?<br>*Smallest unit is one syringe.*<br>*Question relevant when: selected(${q101_C18_full_list} , '5')*<br>*Response constrained to: .>0*	
q601_C19	q601_C19. Syringe luer tip 5ml, no needle<br>*Question relevant when: selected(${q101_C19_full_list} , '5')*	
q601_C19a *(required)*	q601_C19a. What percent of patients require this item?<br>*Question relevant when: selected(${q101_C19_full_list} , '5')*<br>*Response constrained to: .>0 and .<=100 or .=999*	
q601_C19b *(required)*	q601_C19b. How many units of this item are required per patient (throughout the entire treatment provided for complication)?<br>*Smallest unit is one syringe.*<br>*Question relevant when: selected(${q101_C19_full_list} , '5')*<br>*Response constrained to: .>0*	
q601_C20	q601_C20. Venous catheter G18 + injection port and lock tip<br>*Question relevant when: selected(${q101_C20_full_list} , '5')*	
q601_C20a *(required)*	q601_C20a. What percent of patients require this item?<br>*Question relevant when: selected(${q101_C20_full_list} , '5')*<br>*Response constrained to: .>0 and .<=100 or .=999*	
q601_C20b *(required)*	q601_C20b. How many units of this item are required per patient (throughout the entire treatment provided for	

Field	Question	Answer
	complication)?<br>*Smallest unit is one catheter.*<br>*Question relevant when: selected(${q101_C20_full_list} , '5')*<br>*Response constrained to: .>0*	

A. Consumables - Perforations (3)<br>*Group relevant when: selected(${section_six_skip_con} , '1')*		
note_601_bandages_etc	***Bandages, absorbants, wound care, etc.***	
q601_C21	q601_C21. Adhesive tape<br>*Question relevant when: selected(${q101_C21_full_list} , '5')*	
q601_C21a *(required)*	q601_C21a. What percent of patients require this item?<br>*Question relevant when: selected(${q101_C21_full_list} , '5')*<br>*Response constrained to: .>0 and .<=100 or .=999*	
q601_C21b *(required)*	q601_C21b. How many units of this item are required per patient (throughout the entire treatment provided for complication)?<br>*Smallest unit is one roll of tape.*<br>*Question relevant when: selected(${q101_C21_full_list} , '5')*<br>*Response constrained to: .>0*	
q601_C22	q601_C22. Cotton swab<br>*Question relevant when: selected(${q101_C22_full_list} , '5')*	
q601_C22a *(required)*	q601_C22a. What percent of patients require this item?<br>*Question relevant when: selected(${q101_C22_full_list} , '5')*<br>*Response constrained to: .>0 and .<=100 or .=999*	
q601_C22b *(required)*	q601_C22b. How many units of this item are required per patient (throughout the entire treatment provided for complication)?<br>*Smallest unit is one swab.*<br>*Question relevant when: selected(${q101_C22_full_list} , '5')*<br>*Response constrained to: .>0*	
q601_C23	q601_C23. Dressing pad absorbent sterile<br>*Question relevant when: selected(${q101_C23_full_list} , '5')*	
q601_C23a *(required)*	q601_C23a. What percent of patients require this item?<br>*Question relevant when: selected(${q101_C23_full_list} , '5')*<br>*Response constrained to: .>0 and .<=100 or .=999*	
q601_C23b *(required)*	q601_C23b. How many units of this item are required per patient (throughout the entire treatment provided for complication)?<br>*Smallest unit is one pad.*<br>*Question relevant when: selected(${q101_C23_full_list} , '5')*<br>*Response constrained to: .>0*	
q601_C24	q601_C24. Gauze compress, non sterile<br>*Question relevant when: selected(${q101_C24_full_list} , '5')*	
q601_C24a *(required)*	q601_C24a. What percent of patients require this item?<br>*Question relevant when: selected(${q101_C24_full_list} , '5')*<br>*Response constrained to: .>0 and .<=100 or .=999*	
q601_C24b *(required)*	q601_C24b. How many units of this item are required per patient (throughout the entire treatment provided for complication)?<br>*Smallest unit is one compress.*<br>*Question relevant when: selected(${q101_C24_full_list} , '5')*<br>*Response constrained to: .>0*	
q601_C25	q601_C25. Gauze compress, sterile<br>*Question relevant when: selected(${q101_C25_full_list} , '5')*	
q601_C25a *(required)*	q601_C25a. What percent of patients require this item?<br>*Question relevant when: selected(${q101_C25_full_list} , '5')*<br>*Response constrained to: .>0 and .<=100 or .=999*	
q601_C25b *(required)*	q601_C25b. How many units of this item are required per patient (throughout the entire treatment provided for complication)?<br>*Smallest unit is one compress.*<br>*Question relevant when: selected(${q101_C25_full_list} , '5')*<br>*Response constrained to: .>0*	
q601_C26	q601_C26. Linen saver<br>*Question relevant when: selected(${q101_C26_full_list} , '5')*	
q601_C26a *(required)*	q601_C26a. What percent of patients require this item?<br>*Question relevant when: selected(${q101_C26_full_list} , '5')*<br>*Response constrained to: .>0 and .<=100 or .=999*	
q601_C26b *(required)*	q601_C26b. How many units of this item are required per patient (throughout the entire treatment provided for	

Field	Question	Answer
	complication)?<br>*Smallest unit is one saver (absorbant sheet).*<br>*Question relevant when: selected(${q101_C26_full_list} , '5')*<br>*Response constrained to: .>0*	
q601_C27	q601_C27. Sanitary Pad<br>*Question relevant when: selected(${q101_C27_full_list} , '5')*	

q601_C27a *(required)*	q601_C27a. What percent of patients require this item?<br>*Question relevant when: selected(${q101_C27_full_list} , '5')*<br>*Response constrained to: .>0 and .<=100 or .=999*	
q601_C27b *(required)*	q601_C27b. How many units of this item are required per patient (throughout the entire treatment provided for complication)?<br>*Smallest unit is one pad.*<br>*Question relevant when: selected(${q101_C27_full_list} , '5')*<br>*Response constrained to: .>0*	
q601_C28	q601_C28. Surgical plaster<br>*Question relevant when: selected(${q101_C28_full_list} , '5')*	
q601_C28a *(required)*	q601_C28a. What percent of patients require this item?<br>*Question relevant when: selected(${q101_C28_full_list} , '5')*<br>*Response constrained to: .>0 and .<=100 or .=999*	
q601_C28b *(required)*	q601_C28b. How many units of this item are required per patient (throughout the entire treatment provided for complication)?<br>*Smallest unit is one plaster.*<br>*Question relevant when: selected(${q101_C28_full_list} , '5')*<br>*Response constrained to: .>0*	
A. Consumables - Perforations (4)<br>*Group relevant when: selected(${section_six_skip_con} , '1')*		
note_601_protective_etc	***Protective supplies***	
q601_C29	q601_C29. Apron (disposable)<br>*Question relevant when: selected(${q101_C29_full_list} , '5')*	
q601_C29a *(required)*	q601_C29a. What percent of patients require this item?<br>*Question relevant when: selected(${q101_C29_full_list} , '5')*<br>*Response constrained to: .>0 and .<=100 or .=999*	
q601_C29b *(required)*	q601_C29b. How many units of this item are required per patient (throughout the entire treatment provided for complication)?<br>*Smallest unit is one apron.*<br>*Question relevant when: selected(${q101_C29_full_list} , '5')*<br>*Response constrained to: .>0*	
q601_C30	q601_C30. Gown (disposable)<br>*Question relevant when: selected(${q101_C30_full_list} , '5')*	
q601_C30a *(required)*	q601_C30a. What percent of patients require this item?<br>*Question relevant when: selected(${q101_C30_full_list} , '5')*<br>*Response constrained to: .>0 and .<=100 or .=999*	
q601_C30b *(required)*	q601_C30b. How many units of this item are required per patient (throughout the entire treatment provided for complication)?<br>*Smallest unit is one gown.*<br>*Question relevant when: selected(${q101_C30_full_list} , '5')*<br>*Response constrained to: .>0*	
q601_C31	q601_C31. Latex examination glove, without powder<br>*Question relevant when: selected(${q101_C31_full_list} , '5')*	
q601_C31a *(required)*	q601_C31a. What percent of patients require this item?<br>*Question relevant when: selected(${q101_C31_full_list} , '5')*<br>*Response constrained to: .>0 and .<=100 or .=999*	
q601_C31b *(required)*	q601_C31b. How many units of this item are required per patient (throughout the entire treatment provided for complication)?<br>*Smallest unit is one glove.*<br>*Question relevant when: selected(${q101_C31_full_list} , '5')*<br>*Response constrained to: .>0*	
q601_C32	q601_C32. Latex examination glove, with powder<br>*Question relevant when: selected(${q101_C32_full_list} , '5')*	
q601_C32a *(required)*	q601_C32a. What percent of patients require this item?<br>*Question relevant when: selected(${q101_C32_full_list} , '5')*<br>*Response constrained to: .>0 and .<=100 or .=999*	
q601_C32b *(required)*	q601_C32b. How many units of this item are required per patient (throughout the entire treatment provided for complication)?<br>*Smallest unit is one glove.*<br>*Question relevant when: selected(${q101_C32_full_list} , '5')*	

Question relevant when: selected(${q101_C32_full_list} , '5')
Response constrained to: .>0

Field	Question	Answer
q601_C33	q601_C33. Non-latex (e.g. nitrile) examination glove<br>*Question relevant when: selected(${q101_C33_full_list} , '5')*	

Field	Question	Answer
q601_C33a *(required)*	q601_C33a. What percent of patients require this item?<br>*Question relevant when: selected(${q101_C33_full_list} , '5')*<br>*Response constrained to: .>0 and .<=100 or .=999*	
q601_C33b *(required)*	q601_C33b. How many units of this item are required per patient (throughout the entire treatment provided for complication)?<br>*Smallest unit is one glove.*<br>*Question relevant when: selected(${q101_C33_full_list} , '5')*<br>*Response constrained to: .>0*	
q601_C34	q601_C34. Sterile surgical glove<br>*Question relevant when: selected(${q101_C34_full_list} , '5')*	
q601_C34a *(required)*	q601_C34a. What percent of patients require this item?<br>*Question relevant when: selected(${q101_C34_full_list} , '5')*<br>*Response constrained to: .>0 and .<=100 or .=999*	
q601_C34b *(required)*	q601_C34b. How many units of this item are required per patient (throughout the entire treatment provided for complication)?<br>*Smallest unit is one glove.*<br>*Question relevant when: selected(${q101_C34_full_list} , '5')*<br>*Response constrained to: .>0*	
q601_C35	q601_C35. Mask (disposable)<br>*Question relevant when: selected(${q101_C35_full_list} , '5')*	
q601_C35a *(required)*	q601_C35a. What percent of patients require this item?<br>*Question relevant when: selected(${q101_C35_full_list} , '5')*<br>*Response constrained to: .>0 and .<=100 or .=999*	
q601_C35b *(required)*	q601_C35b. How many units of this item are required per patient (throughout the entire treatment provided for complication)?<br>*Smallest unit is one mask.*<br>*Question relevant when: selected(${q101_C35_full_list} , '5')*<br>*Response constrained to: .>0*	
q601_C36	q601_C36. Surgical cap<br>*Question relevant when: selected(${q101_C36_full_list} , '5')*	
q601_C36a *(required)*	q601_C36a. What percent of patients require this item?<br>*Question relevant when: selected(${q101_C36_full_list} , '5')*<br>*Response constrained to: .>0 and .<=100 or .=999*	
q601_C36b *(required)*	q601_C36b. How many units of this item are required per patient (throughout the entire treatment provided for complication)?<br>*Smallest unit is one cap.*<br>*Question relevant when: selected(${q101_C36_full_list} , '5')*<br>*Response constrained to: .>0*	
A. Consumables - Perforations (5)<br>*Group relevant when: selected(${section_six_skip_con} , '1')*		
note_601_surgery_etc	***Surgery supplies***	
q601_C37	q601_C37. Scalpel blade, single use<br>*Question relevant when: selected(${q101_C37_full_list} , '5')*	
q601_C37a *(required)*	q601_C37a. What percent of patients require this item?<br>*Question relevant when: selected(${q101_C37_full_list} , '5')*<br>*Response constrained to: .>0 and .<=100 or .=999*	
q601_C37b *(required)*	q601_C37b. How many units of this item are required per patient (throughout the entire treatment provided for complication)?<br>*Smallest unit is one blade.*<br>*Question relevant when: selected(${q101_C37_full_list} , '5')*<br>*Response constrained to: .>0*	
q601_C38	q601_C38. Surgical drain<br>*Question relevant when: selected(${q101_C38_full_list} , '5')*	
q601_C38a *(required)*	q601_C38a. What percent of patients require this item?<br>*Question relevant when: selected(${q101_C38_full_list} , '5')*<br>*Response constrained to: .>0 and .<=100 or .=999*	
q601_C38b *(required)*	q601_C38b. How many units of this item are required per patient (throughout the entire treatment provided for complication)?<br>*Smallest unit is one drain.*<br>*Question relevant when: selected(${q101_C38_full_list} , '5')*	

Field	Question	Answer
	Question relevant when: selected(${q101_C38_full_list} , '5') *Response constrained to: .>0*	
q601_C39	q601_C39. Suture string, chromic catgut (0) *Question relevant when: selected(${q101_C39_full_list} , '5')*	
q601_C39a *(required)*	q601_C39a. What percent of patients require this item? *Question relevant when: selected(${q101_C39_full_list} , '5')* *Response constrained to: .>0 and .<=100 or .=999*	
q601_C39b *(required)*	q601_C39b. How many units of this item are required per patient (throughout the entire treatment provided for complication)? *Smallest unit is one suture set.* *Question relevant when: selected(${q101_C39_full_list} , '5')* *Response constrained to: .>0*	
q601_C40	q601_C40. Suture string, silk (non absorbable and non-synthetic) *Question relevant when: selected(${q101_C40_full_list} , '5')*	
q601_C40a *(required)*	q601_C40a. What percent of patients require this item? *Question relevant when: selected(${q101_C40_full_list} , '5')* *Response constrained to: .>0 and .<=100 or .=999*	
q601_C40b *(required)*	q601_C40b. How many units of this item are required per patient (throughout the entire treatment provided for complication)? *Smallest unit is one suture set.* *Question relevant when: selected(${q101_C40_full_list} , '5')* *Response constrained to: .>0*	
q601_C41	q601_C41. Suture string, vicryl, no needle included *Question relevant when: selected(${q101_C41_full_list} , '5')*	
q601_C41a *(required)*	q601_C41a. What percent of patients require this item? *Question relevant when: selected(${q101_C41_full_list} , '5')* *Response constrained to: .>0 and .<=100 or .=999*	
q601_C41b *(required)*	q601_C41b. How many units of this item are required per patient (throughout the entire treatment provided for complication)? *Smallest unit is one suture set.* *Question relevant when: selected(${q101_C41_full_list} , '5')* *Response constrained to: .>0*	
q601_C42	q601_C42. Suture string, vicryl with needle *Question relevant when: selected(${q101_C42_full_list} , '5')*	
q601_C42a *(required)*	q601_C42a. What percent of patients require this item? *Question relevant when: selected(${q101_C42_full_list} , '5')* *Response constrained to: .>0 and .<=100 or .=999*	
q601_C42b *(required)*	q601_C42b. How many units of this item are required per patient (throughout the entire treatment provided for complication)? *Smallest unit is one suture set with needle..* *Question relevant when: selected(${q101_C42_full_list} , '5')* *Response constrained to: .>0*	
A. Consumables - Perforations (6) *Group relevant when: selected(${section_six_skip_con} , '1')*		
note_601_urine_etc	***Urine, bladder***	
q601_C43	q601_C43. Bladder/urinary catheter *Question relevant when: selected(${q101_C43_full_list} , '5')*	
q601_C43a *(required)*	q601_C43a. What percent of patients require this item? *Question relevant when: selected(${q101_C43_full_list} , '5')* *Response constrained to: .>0 and .<=100 or .=999*	
q601_C43b *(required)*	q601_C43b. How many units of this item are required per patient (throughout the entire treatment provided for complication)? *Smallest unit is one catheter.* *Question relevant when: selected(${q101_C43_full_list} , '5')* *Response constrained to: .>0*	
q601_C44	q601_C44. Foley's catheter *Question relevant when: selected(${q101_C44_full_list} , '5')*	
q601_C44a *(required)*	q601_C44a. What percent of patients require this item? *Question relevant when: selected(${q101_C44_full_list} , '5')* *Response constrained to: .>0 and .<=100 or .=999*	
q601_C44b *(required)*	q601_C44b. How many units of this item are required per patient (throughout the entire treatment provided for complication)? *Smallest unit is one catheter.* *Question relevant when: selected(${q101_C44_full_list} , '5')*	

Field	Question	Answer
	Question relevant when: selected(${q101_C44_full_list} , '5')<br>*Response constrained to: .>0*	
q601_C45	q601_C45. Urine bag with valve and drain<br>*Question relevant when: selected(${q101_C45_full_list} , '5')*	
q601_C45a *(required)*	q601_C45a. What percent of patients require this item?<br>*Question relevant when: selected(${q101_C45_full_list} , '5')*<br>*Response constrained to: .>0 and .<=100 or .=999*	
q601_C45b *(required)*	q601_C45b. How many units of this item are required per patient (throughout the entire treatment provided for complication)?<br>*Smallest unit is one bag.*<br>*Question relevant when: selected(${q101_C45_full_list} , '5')*<br>*Response constrained to: .>0*	
A. Consumables - Perforations (7)<br>*Group relevant when: selected(${section_six_skip_con} , '1')*		
note_601_other_etc	***Other supplies***	
q601_C46	q601_C46. Silicone oil for lubrication of MVA aspirator<br>*Question relevant when: selected(${q101_C46_full_list} , '5')*	
q601_C46a *(required)*	q601_C46a. What percent of patients require this item?<br>*Question relevant when: selected(${q101_C46_full_list} , '5')*<br>*Response constrained to: .>0 and .<=100 or .=999*	
q601_C46b *(required)*	q601_C46b. How many units of this item are required per patient (throughout the entire treatment provided for complication)?<br>*Smallest unit is mililiter (ml)*<br>*Question relevant when: selected(${q101_C46_full_list} , '5')*<br>*Response constrained to: .>0*	
q601_C47	q601_C47. Speculum, BValve/Cuscos (reusable)<br>*Question relevant when: selected(${q101_C47_full_list} , '5')*	
q601_C47a *(required)*	q601_C47a. What percent of patients require this item?<br>*Question relevant when: selected(${q101_C47_full_list} , '5')*<br>*Response constrained to: .>0 and .<=100 or .=999*	
q601_C47b *(required)*	q601_C47b. How many units of this item are required per patient (throughout the entire treatment provided for complication)?<br>*Smallest unit is one speculum.*<br>*Question relevant when: selected(${q101_C47_full_list} , '5')*<br>*Response constrained to: .>0*	
q601_C48	q601_C48. Speculum, Sim's (disposable)<br>*Question relevant when: selected(${q101_C48_full_list} , '5')*	
q601_C48a *(required)*	q601_C48a. What percent of patients require this item?<br>*Question relevant when: selected(${q101_C48_full_list} , '5')*<br>*Response constrained to: .>0 and .<=100 or .=999*	
q601_C48b *(required)*	q601_C48b. How many units of this item are required per patient (throughout the entire treatment provided for complication)?<br>*Smallest unit is one speculum.*<br>*Question relevant when: selected(${q101_C48_full_list} , '5')*<br>*Response constrained to: .>0*	
A. Consumables - Perforations (8)<br>*Group relevant when: selected(${section_six_skip_con} , '1')*		
note_601_suggest_other_etc	***Other supplies - suggestions?***	
q601_C49	q601_C49. "[q101_C49_full_list_other]"<br>*Question relevant when: selected(${q101_C49_full_list} , '5')*	
q601_C49a *(required)*	q601_C49a. What percent of patients require this item?<br>*Question relevant when: selected(${q101_C49_full_list} , '5')*<br>*Response constrained to: .>0 and .<=100 or .=999*	
q601_C49b *(required)*	q601_C49b. How many units of this item are required per patient (throughout the entire treatment provided for complication)?<br>*Question relevant when: selected(${q101_C49_full_list} , '5')*<br>*Response constrained to: .>0*	
q601_C50	q601_C50. "[q101_C50_full_list_other]"<br>*Question relevant when: selected(${q101_C50_full_list} , '5')*	
q601_C50a *(required)*	q601_C50a. What percent of patients require this item?<br>*Question relevant when: selected(${q101_C50_full_list} , '5')*<br>*Response constrained to: .>0 and .<=100 or .=999*	

Field	Question	Answer
	Response constrained to: .>0 and .<=100 or .=999	
q601_C50b *(required)*	q601_C50b. How many units of this item are required per patient (throughout the entire treatment provided for complication)?<br>*Question relevant when: selected(${q101_C50_full_list} , '5')*<br>*Response constrained to: .>0*	
q601_C51	q601_C51. "[q101_C51_full_list_other]"<br>*Question relevant when: selected(${q101_C51_full_list} , '5')*	

q601_C51a *(required)*	q601_C51a. What percent of patients require this item?<br>*Question relevant when: selected(${q101_C51_full_list} , '5')*<br>*Response constrained to: .>0 and .<=100 or .=999*	
q601_C51b *(required)*	q601_C51b. How many units of this item are required per patient (throughout the entire treatment provided for complication)?<br>*Question relevant when: selected(${q101_C51_full_list} , '5')*<br>*Response constrained to: .>0*	
q601_C52	q601_C52. "[q101_C52_full_list_other]"<br>*Question relevant when: selected(${q101_C52_full_list} , '5')*	
q601_C52a *(required)*	q601_C52a. What percent of patients require this item?<br>*Question relevant when: selected(${q101_C52_full_list} , '5')*<br>*Response constrained to: .>0 and .<=100 or .=999*	
q601_C52b *(required)*	q601_C52b. How many units of this item are required per patient (throughout the entire treatment provided for complication)?<br>*Question relevant when: selected(${q101_C52_full_list} , '5')*<br>*Response constrained to: .>0*	
q601_C53	q601_C53. "[q101_C53_full_list_other]"<br>*Question relevant when: selected(${q101_C53_full_list} , '5')*	
q601_C53a *(required)*	q601_C53a. What percent of patients require this item?<br>*Question relevant when: selected(${q101_C53_full_list} , '5')*<br>*Response constrained to: .>0 and .<=100 or .=999*	
q601_C53b *(required)*	q601_C53b. How many units of this item are required per patient (throughout the entire treatment provided for complication)?<br>*Question relevant when: selected(${q101_C53_full_list} , '5')*<br>*Response constrained to: .>0*	
group_section_seven_intro		
section7_start	**SECTION VII. COSTS OF ALL ITEMS**	
section_seven_skip_con *(required)*	INTERVIEWER: WOULD YOU LIKE TO COMPLETE THIS SECTION NOW OR SKIP THIS SECTION AND RETURN TO IT LATER?<br>*You may need to skip if the participant has indicated that s/he cannot answer the questions in this section.*	1 Do not skip, complete this section now.<br>2 Skip and come back to this section later.
group_section_seven_introB<br>*Group relevant when: selected(${section_seven_skip_con} , '1')*		
section7_start2	In this section of the interview, we will review all of the items that you said are used for management of any complication type. For each item that is used, I'm going to ask how much the item costs and how it is procured.	
section7_start3	INTERVIEWER: ENTER 999 FOR ANY THAT ARE UNKNOWN.	
A. Consumables - Cost (1)<br>*Group relevant when: selected(${section_seven_skip_con} , '1')*		
note_701_cleaning	***Cleaning, disinfectants, sterilization, etc.***	
q701_C1	***q701_C1. Alcohol, denatured 70%***<br>*Question relevant when: not(selected(${q101_C1_full_list} , '6')) and not(selected(${q101_C1_full_list} , '99'))*	
q701_C1a *(required)*	q701_C1a. When purchased, how many units of this item typically come in one pack, box, bottle, etc.? (e.g. 100 tablets, 1000 ml's, etc.) (Write 1 if purchased as a single item.)<br>*Smallest unit is mililiter (ml)*<br>*Question relevant when: not(selected(${q101_C1_full_list} , '6')) and not(selected(${q101_C1_full_list} , '99'))*<br>*Response constrained to: .>=0*	
q701_C1b *(required)*	q701_C1b. What is the typical purchase price for that quanity of units (i.e. the quantity noted in the question above)?<br>*Question relevant when: not(selected(${q101_C1_full_list} , '6')) and not(selected(${q101_C1_full_list} , '99'))*<br>*Response constrained to: .>=0*	
q701_C1c *(required)*	q701_C1c. Please specify the currency for the purchase price.<br>*Question relevant when: not(selected(${q101_C1_full_list} , '6')) and not(selected(${q101_C1_full_list} , '99'))*	1 Tanzanian Shillings<br>2 US dollars<br>3 Euros<br>99 Don't know<br>5 Other
q701_C1c.1	q701_C1c.1 If other currency, specify:<br>*Leave blank if not applicable*<br>*Question relevant when: not(selected(${q101_C1_full_list} , '6')) and not(selected(${q101_C1_full_list} , '99'))*	

Field	Question	Answer
	Question relevant when: not(selected(${q101_C1_full_list} , '6')) and not(selected(${q101_C1_full_list} , '99'))	
q701_C1d *(required)*	q701_C1d. Please specify the year of the purchase price.<br>*Question relevant when: not(selected(${q101_C1_full_list} , '6')) and not(selected(${q101_C1_full_list} , '99'))*<br>*Response constrained to: .>=2000 and .<=2019 or .=999*	
q701_C2	***q701_C2. Chlorine (JIK) or Glutaraldehyde solution***<br>*Question relevant when: not(selected(${q101_C2_full_list} , '6')) and not(selected(${q101_C2_full_list} , '99'))*	

Field	Question	Answer
q701_C2a *(required)*	q701_C2a. When purchased, how many units of this item typically come in one pack, box, bottle, etc.? (e.g. 100 tablets, 1000 ml's, etc.) (Write 1 if purchased as a single item.)<br>*Smallest unit is mililiter (ml)*<br>*Question relevant when: not(selected(${q101_C2_full_list} , '6')) and not(selected(${q101_C2_full_list} , '99'))*<br>*Response constrained to: .>=0*	
q701_C2b *(required)*	q701_C2b. What is the typical purchase price for that quanity of units (i.e. the quantity noted in the question above)?<br>*Question relevant when: not(selected(${q101_C2_full_list} , '6')) and not(selected(${q101_C2_full_list} , '99'))*<br>*Response constrained to: .>=0*	
q701_C2c *(required)*	q701_C2c. Please specify the currency for the purchase price.<br>*Question relevant when: not(selected(${q101_C2_full_list} , '6')) and not(selected(${q101_C2_full_list} , '99'))*	1 Tanzanian Shillings<br>2 US dollars<br>3 Euros<br>99 Don't know<br>5 Other
q701_C2c.1	q701_C2c.1 If other currency, specify:<br>*Leave blank if not applicable*<br>*Question relevant when: not(selected(${q101_C2_full_list} , '6')) and not(selected(${q101_C2_full_list} , '99'))*	
q701_C2d *(required)*	q701_C2d. Please specify the year of the purchase price.<br>*Question relevant when: not(selected(${q101_C2_full_list} , '6')) and not(selected(${q101_C2_full_list} , '99'))*<br>*Response constrained to: .>=2000 and .<=2019 or .=999*	
q701_C3	***q701_C3. Clean water***<br>*Question relevant when: not(selected(${q101_C3_full_list} , '6')) and not(selected(${q101_C3_full_list} , '99'))*	
q701_C3a *(required)*	q701_C3a. When purchased, how many units of this item typically come in one pack, box, bottle, etc.? (e.g. 100 tablets, 1000 ml's, etc.) (Write 1 if purchased as a single item.)<br>*Smallest unit is litre (ltr)*<br>*Question relevant when: not(selected(${q101_C3_full_list} , '6')) and not(selected(${q101_C3_full_list} , '99'))*<br>*Response constrained to: .>=0*	
q701_C3b *(required)*	q701_C3b. What is the typical purchase price for that quanity of units (i.e. the quantity noted in the question above)?<br>*Question relevant when: not(selected(${q101_C3_full_list} , '6')) and not(selected(${q101_C3_full_list} , '99'))*<br>*Response constrained to: .>=0*	
q701_C3c *(required)*	q701_C3c. Please specify the currency for the purchase price.<br>*Question relevant when: not(selected(${q101_C3_full_list} , '6')) and not(selected(${q101_C3_full_list} , '99'))*	1 Tanzanian Shillings<br>2 US dollars<br>3 Euros<br>99 Don't know<br>5 Other
q701_C3c.1	q701_C3c.1 If other currency, specify:<br>*Leave blank if not applicable*<br>*Question relevant when: not(selected(${q101_C3_full_list} , '6')) and not(selected(${q101_C3_full_list} , '99'))*	
q701_C3d *(required)*	q701_C3d. Please specify the year of the purchase price.<br>*Question relevant when: not(selected(${q101_C3_full_list} , '6')) and not(selected(${q101_C3_full_list} , '99'))*<br>*Response constrained to: .>=2000 and .<=2019 or .=999*	
q701_C4	***q701_C4. Dettol solution or other water-based antiseptic***<br>*Question relevant when: not(selected(${q101_C4_full_list} , '6')) and not(selected(${q101_C4_full_list} , '99'))*	
q701_C4a *(required)*	q701_C4a. When purchased, how many units of this item typically come in one pack, box, bottle, etc.? (e.g. 100 tablets, 1000 ml's, etc.) (Write 1 if purchased as a single item.)<br>*Smallest unit is mililiter (ml)*<br>*Question relevant when: not(selected(${q101_C4_full_list} , '6')) and not(selected(${q101_C4_full_list} , '99'))*<br>*Response constrained to: .>=0*	
q701_C4b *(required)*	q701_C4b. What is the typical purchase price for that quanity of units (i.e. the quantity noted in the question above)?<br>*Question relevant when: not(selected(${q101_C4_full_list} , '6')) and not(selected(${q101_C4_full_list} , '99'))*<br>*Response constrained to: .>=0*	
q701_C4c *(required)*	q701_C4c. Please specify the currency for the purchase price.<br>*Question relevant when: not(selected(${q101_C4_full_list} , '6')) and not(selected(${q101_C4_full_list} , '99'))*	1 Tanzanian Shillings<br>2 US dollars<br>3 Euros<br>99 Don't know<br>5 Other
q701_C4c.1	q701_C4c.1 If other currency, specify:<br>*Leave blank if not applicable*<br>*Question relevant when: not(selected(${q101_C4_full_list} , '6')) and not(selected(${q101_C4_full_list} , '99'))*	

Field	Question	Answer
	Question relevant when: not(selected(${q101_C4_full_list} , '6')) and not(selected(${q101_C4_full_list} , '99'))	
q701_C4d *(required)*	q701_C4d. Please specify the year of the purchase price.<br>*Question relevant when: not(selected(${q101_C4_full_list} , '6')) and not(selected(${q101_C4_full_list} , '99'))*<br>*Response constrained to: .>=2000 and .<=2019 or .=999*	
q701_C5	***q701_C5. Cetrimide 15% ("Savlon")***<br>*Question relevant when: not(selected(${q101_C5_full_list} , '6')) and not(selected(${q101_C5_full_list} , '99'))*	
q701_C5a *(required)*	q701_C5a. When purchased, how many units of this item typically come in one pack, box, bottle, etc.? (e.g. 100 tablets, 1000 ml's, etc.) (Write 1 if purchased as a single item.)<br>*Smallest unit is mililiter (ml)*<br>*Question relevant when: not(selected(${q101_C5_full_list} , '6')) and not(selected(${q101_C5_full_list} , '99'))*<br>*Response constrained to: .>=0*	
q701_C5b *(required)*	q701_C5b. What is the typical purchase price for that quanity of units (i.e. the quantity noted in the question above)?<br>*Question relevant when: not(selected(${q101_C5_full_list} , '6')) and not(selected(${q101_C5_full_list} , '99'))*<br>*Response constrained to: .>=0*	
q701_C5c *(required)*	q701_C5c. Please specify the currency for the purchase price.<br>*Question relevant when: not(selected(${q101_C5_full_list} , '6')) and not(selected(${q101_C5_full_list} , '99'))*	1 Tanzanian Shillings<br>2 US dollars<br>3 Euros<br>99 Don't know<br>5 Other
q701_C5c.1	q701_C5c.1 If other currency, specify:<br>*Leave blank if not applicable*<br>*Question relevant when: not(selected(${q101_C5_full_list} , '6')) and not(selected(${q101_C5_full_list} , '99'))*	
q701_C5d *(required)*	q701_C5d. Please specify the year of the purchase price.<br>*Question relevant when: not(selected(${q101_C5_full_list} , '6')) and not(selected(${q101_C5_full_list} , '99'))*<br>*Response constrained to: .>=2000 and .<=2019 or .=999*	
q701_C6	***q701_C6. Hand wash (liquid)***<br>*Question relevant when: not(selected(${q101_C6_full_list} , '6')) and not(selected(${q101_C6_full_list} , '99'))*	
q701_C6a *(required)*	q701_C6a. When purchased, how many units of this item typically come in one pack, box, bottle, etc.? (e.g. 100 tablets, 1000 ml's, etc.) (Write 1 if purchased as a single item.)<br>*Smallest unit is mililiter (ml)*<br>*Question relevant when: not(selected(${q101_C6_full_list} , '6')) and not(selected(${q101_C6_full_list} , '99'))*<br>*Response constrained to: .>=0*	
q701_C6b *(required)*	q701_C6b. What is the typical purchase price for that quanity of units (i.e. the quantity noted in the question above)?<br>*Question relevant when: not(selected(${q101_C6_full_list} , '6')) and not(selected(${q101_C6_full_list} , '99'))*<br>*Response constrained to: .>=0*	
q701_C6c *(required)*	q701_C6c. Please specify the currency for the purchase price.<br>*Question relevant when: not(selected(${q101_C6_full_list} , '6')) and not(selected(${q101_C6_full_list} , '99'))*	1 Tanzanian Shillings<br>2 US dollars<br>3 Euros<br>99 Don't know<br>5 Other
q701_C6c.1	q701_C6c.1 If other currency, specify:<br>*Leave blank if not applicable*<br>*Question relevant when: not(selected(${q101_C6_full_list} , '6')) and not(selected(${q101_C6_full_list} , '99'))*	
q701_C6d *(required)*	q701_C6d. Please specify the year of the purchase price.<br>*Question relevant when: not(selected(${q101_C6_full_list} , '6')) and not(selected(${q101_C6_full_list} , '99'))*<br>*Response constrained to: .>=2000 and .<=2019 or .=999*	
q701_C7	***q701_C7. Soap (bar)***<br>*Question relevant when: not(selected(${q101_C7_full_list} , '6')) and not(selected(${q101_C7_full_list} , '99'))*	
q701_C7a *(required)*	q701_C7a. When purchased, how many units of this item typically come in one pack, box, bottle, etc.? (e.g. 100 tablets, 1000 ml's, etc.) (Write 1 if purchased as a single item.)<br>*Smallest unit is 1 bar of soap*<br>*Question relevant when: not(selected(${q101_C7_full_list} , '6')) and not(selected(${q101_C7_full_list} , '99'))*<br>*Response constrained to: .>=0*	
q701_C7b *(required)*	q701_C7b. What is the typical purchase price for that quanity of units (i.e. the quantity noted in the question above)?<br>*Question relevant when: not(selected(${q101_C7_full_list} , '6')) and not(selected(${q101_C7_full_list} , '99'))*<br>*Response constrained to: .>=0*	
q701_C7c *(required)*	q701_C7c. Please specify the currency for the purchase price.<br>*Question relevant when: not(selected(${q101_C7_full_list} , '6')) and not(selected(${q101_C7_full_list} , '99'))*	1 Tanzanian Shillings<br>2 US dollars<br>3 Euros<br>99 Don't know<br>5 Other
q701_C7c.1	q701_C7c.1 If other currency, specify:<br>*Leave blank if not applicable*<br>*Question relevant when: not(selected(${q101_C7_full_list} , '6')) and not(selected(${q101_C7_full_list} , '99'))*	

Field	Question	Answer
	Question relevant when: not(selected(${q101_C7_full_list} , '6')) and not(selected(${q101_C7_full_list} , '99'))	
q701_C7d *(required)*	q701_C7d. Please specify the year of the purchase price.<br>*Question relevant when: not(selected(${q101_C7_full_list} , '6')) and not(selected(${q101_C7_full_list} , '99'))*<br>*Response constrained to: .>=2000 and .<=2019 or .=999*	
q701_C8	***q701_C8. Hand sterilizer (waterless)***<br>*Question relevant when: not(selected(${q101_C8_full_list} , '6')) and not(selected(${q101_C8_full_list} , '99'))*	
q701_C8a *(required)*	q701_C8a. When purchased, how many units of this item typically come in one pack, box, bottle, etc.? (e.g. 100 tablets, 1000 ml's, etc.) (Write 1 if purchased as a single item.)<br>*Smallest unit is mililiter (ml)*<br>*Question relevant when: not(selected(${q101_C8_full_list} , '6')) and not(selected(${q101_C8_full_list} , '99'))*<br>*Response constrained to: .>=0*	
q701_C8b *(required)*	q701_C8b. What is the typical purchase price for that quanity of units (i.e. the quantity noted in the question above)?<br>*Question relevant when: not(selected(${q101_C8_full_list} , '6')) and not(selected(${q101_C8_full_list} , '99'))*<br>*Response constrained to: .>=0*	
q701_C8c *(required)*	q701_C8c. Please specify the currency for the purchase price.<br>*Question relevant when: not(selected(${q101_C8_full_list} , '6')) and not(selected(${q101_C8_full_list} , '99'))*	1 Tanzanian Shillings<br>2 US dollars<br>3 Euros<br>99 Don't know<br>5 Other
q701_C8c.1	q701_C8c.1 If other currency, specify:<br>*Leave blank if not applicable*<br>*Question relevant when: not(selected(${q101_C8_full_list} , '6')) and not(selected(${q101_C8_full_list} , '99'))*	
q701_C8d *(required)*	q701_C8d. Please specify the year of the purchase price.<br>*Question relevant when: not(selected(${q101_C8_full_list} , '6')) and not(selected(${q101_C8_full_list} , '99'))*<br>*Response constrained to: .>=2000 and .<=2019 or .=999*	
A. Consumables - Cost (2)<br>*Group relevant when: selected(${section_seven_skip_con} , '1')*		
note_701_IV_needle_syringe	***IV, needles, syringes, etc.***	
q701_C9	***q701_C9. Blood giving set with needle***<br>*Question relevant when: not(selected(${q101_C9_full_list} , '6')) and not(selected(${q101_C9_full_list} , '99'))*	
q701_C9a *(required)*	q701_C9a. When purchased, how many units of this item typically come in one pack, box, bottle, etc.? (e.g. 100 tablets, 1000 ml's, etc.) (Write 1 if purchased as a single item.)<br>*Smallest unit is one set.*<br>*Question relevant when: not(selected(${q101_C9_full_list} , '6')) and not(selected(${q101_C9_full_list} , '99'))*<br>*Response constrained to: .>=0*	
q701_C9b *(required)*	q701_C9b. What is the typical purchase price for that quanity of units (i.e. the quantity noted in the question above)?<br>*Question relevant when: not(selected(${q101_C9_full_list} , '6')) and not(selected(${q101_C9_full_list} , '99'))*<br>*Response constrained to: .>=0*	
q701_C9c *(required)*	q701_C9c. Please specify the currency for the purchase price.<br>*Question relevant when: not(selected(${q101_C9_full_list} , '6')) and not(selected(${q101_C9_full_list} , '99'))*	1 Tanzanian Shillings<br>2 US dollars<br>3 Euros<br>99 Don't know<br>5 Other
q701_C9c.1	q701_C9c.1 If other currency, specify:<br>*Leave blank if not applicable*<br>*Question relevant when: not(selected(${q101_C9_full_list} , '6')) and not(selected(${q101_C9_full_list} , '99'))*	
q701_C9d *(required)*	q701_C9d. Please specify the year of the purchase price.<br>*Question relevant when: not(selected(${q101_C9_full_list} , '6')) and not(selected(${q101_C9_full_list} , '99'))*<br>*Response constrained to: .>=2000 and .<=2019 or .=999*	
q701_C10	***q701_C10. IV cannula***<br>*Question relevant when: not(selected(${q101_C10_full_list} , '6')) and not(selected(${q101_C10_full_list} , '99'))*	
q701_C10a *(required)*	q701_C10a. When purchased, how many units of this item typically come in one pack, box, bottle, etc.? (e.g. 100 tablets, 1000 ml's, etc.) (Write 1 if purchased as a single item.)<br>*Smallest unit is one cannula.*<br>*Question relevant when: not(selected(${q101_C10_full_list} , '6')) and not(selected(${q101_C10_full_list} , '99'))*<br>*Response constrained to: .>=0*	
q701_C10b *(required)*	q701_C10b. What is the typical purchase price for that quanity of units (i.e. the quantity noted in the question above)?<br>*Question relevant when: not(selected(${q101_C10_full_list} , '6')) and not(selected(${q101_C10_full_list} , '99'))*<br>*Response constrained to: .>=0*	
q701_C10c *(required)*	q701_C10c. Please specify the currency for the purchase price.<br>*Question relevant when: not(selected(${q101_C10_full_list} , '6')) and not(selected(${q101_C10_full_list} , '99'))*	1 Tanzanian Shillings<br>2 US dollars<br>3 Euros<br>99 Don't know

Field	Question	Answer
		5 Other
q701_C10c.1	q701_C10c.1 If other currency, specify:<br>*Leave blank if not applicable*<br>*Question relevant when: not(selected(${q101_C10_full_list} , '6')) and not(selected(${q101_C10_full_list} , '99'))*	
q701_C10d *(required)*	q701_C10d. Please specify the year of the purchase price.<br>*Question relevant when: not(selected(${q101_C10_full_list} , '6')) and not(selected(${q101_C10_full_list} , '99'))*<br>*Response constrained to: .>=2000 and .<=2019 or .=999*	

q701_C11	***q701_C11. IV set***<br>*Question relevant when: not(selected(${q101_C11_full_list} , '6')) and not(selected(${q101_C11_full_list} , '99'))*	
q701_C11a *(required)*	q701_C11a. When purchased, how many units of this item typically come in one pack, box, bottle, etc.? (e.g. 100 tablets, 1000 ml's, etc.) (Write 1 if purchased as a single item.)<br>*Smallest unit is one set.*<br>*Question relevant when: not(selected(${q101_C11_full_list} , '6')) and not(selected(${q101_C11_full_list} , '99'))*<br>*Response constrained to: .>=0*	
q701_C11b *(required)*	q701_C11b. What is the typical purchase price for that quanity of units (i.e. the quantity noted in the question above)?<br>*Question relevant when: not(selected(${q101_C11_full_list} , '6')) and not(selected(${q101_C11_full_list} , '99'))*<br>*Response constrained to: .>=0*	
q701_C11c *(required)*	q701_C11c. Please specify the currency for the purchase price.<br>*Question relevant when: not(selected(${q101_C11_full_list} , '6')) and not(selected(${q101_C11_full_list} , '99'))*	1 Tanzanian Shillings<br>2 US dollars<br>3 Euros<br>99 Don't know<br>5 Other
q701_C11c.1	q701_C11c.1 If other currency, specify:<br>*Leave blank if not applicable*<br>*Question relevant when: not(selected(${q101_C11_full_list} , '6')) and not(selected(${q101_C11_full_list} , '99'))*	
q701_C11d *(required)*	q701_C11d. Please specify the year of the purchase price.<br>*Question relevant when: not(selected(${q101_C11_full_list} , '6')) and not(selected(${q101_C11_full_list} , '99'))*<br>*Response constrained to: .>=2000 and .<=2019 or .=999*	
q701_C12	***q701_C12. Needle luer tip 18Gx1.1/2" (1.2x38mm)***<br>*Question relevant when: not(selected(${q101_C12_full_list} , '6')) and not(selected(${q101_C12_full_list} , '99'))*	
q701_C12a *(required)*	q701_C12a. When purchased, how many units of this item typically come in one pack, box, bottle, etc.? (e.g. 100 tablets, 1000 ml's, etc.) (Write 1 if purchased as a single item.)<br>*Smallest unit is one needle.*<br>*Question relevant when: not(selected(${q101_C12_full_list} , '6')) and not(selected(${q101_C12_full_list} , '99'))*<br>*Response constrained to: .>=0*	
q701_C12b *(required)*	q701_C12b. What is the typical purchase price for that quanity of units (i.e. the quantity noted in the question above)?<br>*Question relevant when: not(selected(${q101_C12_full_list} , '6')) and not(selected(${q101_C12_full_list} , '99'))*<br>*Response constrained to: .>=0*	
q701_C12c *(required)*	q701_C12c. Please specify the currency for the purchase price.<br>*Question relevant when: not(selected(${q101_C12_full_list} , '6')) and not(selected(${q101_C12_full_list} , '99'))*	1 Tanzanian Shillings<br>2 US dollars<br>3 Euros<br>99 Don't know<br>5 Other
q701_C12c.1	q701_C12c.1 If other currency, specify:<br>*Leave blank if not applicable*<br>*Question relevant when: not(selected(${q101_C12_full_list} , '6')) and not(selected(${q101_C12_full_list} , '99'))*	
q701_C12d *(required)*	q701_C12d. Please specify the year of the purchase price.<br>*Question relevant when: not(selected(${q101_C12_full_list} , '6')) and not(selected(${q101_C12_full_list} , '99'))*<br>*Response constrained to: .>=2000 and .<=2019 or .=999*	
q701_C13	***q701_C13. Needle luer tip 21Gx1.1/2" (0.8x38mm)***<br>*Question relevant when: not(selected(${q101_C13_full_list} , '6')) and not(selected(${q101_C13_full_list} , '99'))*	
q701_C13a *(required)*	q701_C13a. When purchased, how many units of this item typically come in one pack, box, bottle, etc.? (e.g. 100 tablets, 1000 ml's, etc.) (Write 1 if purchased as a single item.)<br>*Smallest unit is one needle.*<br>*Question relevant when: not(selected(${q101_C13_full_list} , '6')) and not(selected(${q101_C13_full_list} , '99'))*<br>*Response constrained to: .>=0*	
q701_C13b *(required)*	q701_C13b. What is the typical purchase price for that quanity of units (i.e. the quantity noted in the question above)?<br>*Question relevant when: not(selected(${q101_C13_full_list} , '6')) and not(selected(${q101_C13_full_list} , '99'))*<br>*Response constrained to: .>=0*	
q701_C13c *(required)*	q701_C13c. Please specify the currency for the purchase price.<br>*Question relevant when: not(selected(${q101_C13_full_list} , '6')) and not(selected(${q101_C13_full_list} , '99'))*	1 Tanzanian Shillings<br>2 US dollars<br>3 Euros<br>99 Don't know

Field	Question	Answer
		5 Other
q701_C13c.1	q701_C13c.1 If other currency, specify:<br>*Leave blank if not applicable*<br>*Question relevant when: not(selected(${q101_C13_full_list} , '6')) and not(selected(${q101_C13_full_list} , '99'))*	
q701_C13d *(required)*	q701_C13d. Please specify the year of the purchase price.<br>*Question relevant when: not(selected(${q101_C13_full_list} , '6')) and not(selected(${q101_C13_full_list} , '99'))*<br>*Response constrained to: .>=2000 and .<=2019 or .=999*	

Field	Question	Answer
q701_C14	***q701_C14. Needle, suture, round body***<br>*Question relevant when: not(selected(${q101_C14_full_list} , '6')) and not(selected(${q101_C14_full_list} , '99'))*	
q701_C14a *(required)*	q701_C14a. When purchased, how many units of this item typically come in one pack, box, bottle, etc.? (e.g. 100 tablets, 1000 ml's, etc.) (Write 1 if purchased as a single item.)<br>*Smallest unit is one needle.*<br>*Question relevant when: not(selected(${q101_C14_full_list} , '6')) and not(selected(${q101_C14_full_list} , '99'))*<br>*Response constrained to: .>=0*	
q701_C14b *(required)*	q701_C14b. What is the typical purchase price for that quanity of units (i.e. the quantity noted in the question above)?<br>*Question relevant when: not(selected(${q101_C14_full_list} , '6')) and not(selected(${q101_C14_full_list} , '99'))*<br>*Response constrained to: .>=0*	
q701_C14c *(required)*	q701_C14c. Please specify the currency for the purchase price.<br>*Question relevant when: not(selected(${q101_C14_full_list} , '6')) and not(selected(${q101_C14_full_list} , '99'))*	1 Tanzanian Shillings<br>2 US dollars<br>3 Euros<br>99 Don't know<br>5 Other
q701_C14c.1	q701_C14c.1 If other currency, specify:<br>*Leave blank if not applicable*<br>*Question relevant when: not(selected(${q101_C14_full_list} , '6')) and not(selected(${q101_C14_full_list} , '99'))*	
q701_C14d *(required)*	q701_C14d. Please specify the year of the purchase price.<br>*Question relevant when: not(selected(${q101_C14_full_list} , '6')) and not(selected(${q101_C14_full_list} , '99'))*<br>*Response constrained to: .>=2000 and .<=2019 or .=999*	
q701_C15	***q701_C15. Spinal needle***<br>*Question relevant when: not(selected(${q101_C15_full_list} , '6')) and not(selected(${q101_C15_full_list} , '99'))*	
q701_C15a *(required)*	q701_C15a. When purchased, how many units of this item typically come in one pack, box, bottle, etc.? (e.g. 100 tablets, 1000 ml's, etc.) (Write 1 if purchased as a single item.)<br>*Smallest unit is one needle.*<br>*Question relevant when: not(selected(${q101_C15_full_list} , '6')) and not(selected(${q101_C15_full_list} , '99'))*<br>*Response constrained to: .>=0*	
q701_C15b *(required)*	q701_C15b. What is the typical purchase price for that quanity of units (i.e. the quantity noted in the question above)?<br>*Question relevant when: not(selected(${q101_C15_full_list} , '6')) and not(selected(${q101_C15_full_list} , '99'))*<br>*Response constrained to: .>=0*	
q701_C15c *(required)*	q701_C15c. Please specify the currency for the purchase price.<br>*Question relevant when: not(selected(${q101_C15_full_list} , '6')) and not(selected(${q101_C15_full_list} , '99'))*	1 Tanzanian Shillings<br>2 US dollars<br>3 Euros<br>99 Don't know<br>5 Other
q701_C15c.1	q701_C15c.1 If other currency, specify:<br>*Leave blank if not applicable*<br>*Question relevant when: not(selected(${q101_C15_full_list} , '6')) and not(selected(${q101_C15_full_list} , '99'))*	
q701_C15d *(required)*	q701_C15d. Please specify the year of the purchase price.<br>*Question relevant when: not(selected(${q101_C15_full_list} , '6')) and not(selected(${q101_C15_full_list} , '99'))*<br>*Response constrained to: .>=2000 and .<=2019 or .=999*	
q701_C16	***q701_C16. Syringe 10ml, with bypacked needle 21Gx1.5"***<br>*Question relevant when: not(selected(${q101_C16_full_list} , '6')) and not(selected(${q101_C16_full_list} , '99'))*	
q701_C16a *(required)*	q701_C16a. When purchased, how many units of this item typically come in one pack, box, bottle, etc.? (e.g. 100 tablets, 1000 ml's, etc.) (Write 1 if purchased as a single item.)<br>*Smallest unit is one syringe.*<br>*Question relevant when: not(selected(${q101_C16_full_list} , '6')) and not(selected(${q101_C16_full_list} , '99'))*<br>*Response constrained to: .>=0*	
q701_C16b *(required)*	q701_C16b. What is the typical purchase price for that quanity of units (i.e. the quantity noted in the question above)?<br>*Question relevant when: not(selected(${q101_C16_full_list} , '6')) and not(selected(${q101_C16_full_list} , '99'))*<br>*Response constrained to: .>=0*	
q701_C16c *(required)*	q701_C16c. Please specify the currency for the purchase price.<br>*Question relevant when: not(selected(${q101_C16_full_list} , '6')) and not(selected(${q101_C16_full_list} , '99'))*	1 Tanzanian Shillings<br>2 US dollars<br>3 Euros<br>99 Don't know

Field	Question	Answer
		99 Don't know<br>5 Other
q701_C16c.1	q701_C16c.1 If other currency, specify:<br>*Leave blank if not applicable*<br>*Question relevant when: not(selected(${q101_C16_full_list} , '6')) and not(selected(${q101_C16_full_list} , '99'))*	
q701_C16d *(required)*	q701_C16d. Please specify the year of the purchase price.<br>*Question relevant when: not(selected(${q101_C16_full_list} , '6')) and not(selected(${q101_C16_full_list} , '99'))*<br>*Response constrained to: .>=2000 and .<=2019 or .=999*	

Field	Question	Answer
q701_C17	***q701_C17. Syringe 5ml, with bypacked needle 21Gx1.5"***<br>*Question relevant when: not(selected(${q101_C16_full_list} , '6')) and not(selected(${q101_C16_full_list} , '99'))*	
q701_C17a *(required)*	q701_C17a. When purchased, how many units of this item typically come in one pack, box, bottle, etc.? (e.g. 100 tablets, 1000 ml's, etc.) (Write 1 if purchased as a single item.)<br>*Smallest unit is one syringe.*<br>*Question relevant when: not(selected(${q101_C17_full_list} , '6')) and not(selected(${q101_C17_full_list} , '99'))*<br>*Response constrained to: .>=0*	
q701_C17b *(required)*	q701_C17b. What is the typical purchase price for that quanity of units (i.e. the quantity noted in the question above)?<br>*Question relevant when: not(selected(${q101_C17_full_list} , '6')) and not(selected(${q101_C17_full_list} , '99'))*<br>*Response constrained to: .>=0*	
q701_C17c *(required)*	q701_C17c. Please specify the currency for the purchase price.<br>*Question relevant when: not(selected(${q101_C17_full_list} , '6')) and not(selected(${q101_C17_full_list} , '99'))*	1 Tanzanian Shillings<br>2 US dollars<br>3 Euros<br>99 Don't know<br>5 Other
q701_C17c.1	q701_C17c.1 If other currency, specify:<br>*Leave blank if not applicable*<br>*Question relevant when: not(selected(${q101_C17_full_list} , '6')) and not(selected(${q101_C17_full_list} , '99'))*	
q701_C17d *(required)*	q701_C17d. Please specify the year of the purchase price.<br>*Question relevant when: not(selected(${q101_C17_full_list} , '6')) and not(selected(${q101_C17_full_list} , '99'))*<br>*Response constrained to: .>=2000 and .<=2019 or .=999*	
q701_C18	***q701_C18. Syringe luer tip 10ml, no needle***<br>*Question relevant when: not(selected(${q101_C18_full_list} , '6')) and not(selected(${q101_C18_full_list} , '99'))*	
q701_C18a *(required)*	q701_C18a. When purchased, how many units of this item typically come in one pack, box, bottle, etc.? (e.g. 100 tablets, 1000 ml's, etc.) (Write 1 if purchased as a single item.)<br>*Smallest unit is one syringe.*<br>*Question relevant when: not(selected(${q101_C18_full_list} , '6')) and not(selected(${q101_C18_full_list} , '99'))*<br>*Response constrained to: .>=0*	
q701_C18b *(required)*	q701_C18b. What is the typical purchase price for that quanity of units (i.e. the quantity noted in the question above)?<br>*Question relevant when: not(selected(${q101_C18_full_list} , '6')) and not(selected(${q101_C18_full_list} , '99'))*<br>*Response constrained to: .>=0*	
q701_C18c *(required)*	q701_C18c. Please specify the currency for the purchase price.<br>*Question relevant when: not(selected(${q101_C18_full_list} , '6')) and not(selected(${q101_C18_full_list} , '99'))*	1 Tanzanian Shillings<br>2 US dollars<br>3 Euros<br>99 Don't know<br>5 Other
q701_C18c.1	q701_C18c.1 If other currency, specify:<br>*Leave blank if not applicable*<br>*Question relevant when: not(selected(${q101_C18_full_list} , '6')) and not(selected(${q101_C18_full_list} , '99'))*	
q701_C18d *(required)*	q701_C18d. Please specify the year of the purchase price.<br>*Question relevant when: not(selected(${q101_C18_full_list} , '6')) and not(selected(${q101_C18_full_list} , '99'))*<br>*Response constrained to: .>=2000 and .<=2019 or .=999*	
q701_C19	***q701_C19. Syringe luer tip 5ml, no needle***<br>*Question relevant when: not(selected(${q101_C19_full_list} , '6')) and not(selected(${q101_C19_full_list} , '99'))*	
q701_C19a *(required)*	q701_C19a. When purchased, how many units of this item typically come in one pack, box, bottle, etc.? (e.g. 100 tablets, 1000 ml's, etc.) (Write 1 if purchased as a single item.)<br>*Smallest unit is one syringe.*<br>*Question relevant when: not(selected(${q101_C19_full_list} , '6')) and not(selected(${q101_C19_full_list} , '99'))*<br>*Response constrained to: .>=0*	
q701_C19b *(required)*	q701_C19b. What is the typical purchase price for that quanity of units (i.e. the quantity noted in the question above)?<br>*Question relevant when: not(selected(${q101_C19_full_list} , '6')) and not(selected(${q101_C19_full_list} , '99'))*<br>*Response constrained to: .>=0*	
q701_C19c *(required)*	q701_C19c. Please specify the currency for the purchase price.<br>*Question relevant when: not(selected(${q101_C19_full_list} , '6')) and not(selected(${q101_C19_full_list} , '99'))*	1 Tanzanian Shillings<br>2 US dollars<br>3 Euros

Field	Question	Answer
		99 Don't know<br>5 Other
q701_C19c.1	q701_C19c.1 If other currency, specify:<br>*Leave blank if not applicable*<br>*Question relevant when: not(selected(${q101_C19_full_list} , '6')) and not(selected(${q101_C19_full_list} , '99'))*	
q701_C19d *(required)*	q701_C19d. Please specify the year of the purchase price.<br>*Question relevant when: not(selected(${q101_C19_full_list} , '6')) and not(selected(${q101_C19_full_list} , '99'))*<br>*Response constrained to: .>=2000 and .<=2019 or .=999*	

q701_C20	***q701_C20. Venous catheter G18 + injection port and lock tip***<br>*Question relevant when: not(selected(${q101_C20_full_list} , '6')) and not(selected(${q101_C20_full_list} , '99'))*	
q701_C20a *(required)*	q701_C20a. When purchased, how many units of this item typically come in one pack, box, bottle, etc.? (e.g. 100 tablets, 1000 ml's, etc.) (Write 1 if purchased as a single item.)<br>*Smallest unit is one catheter.*<br>*Question relevant when: not(selected(${q101_C20_full_list} , '6')) and not(selected(${q101_C20_full_list} , '99'))*<br>*Response constrained to: .>=0*	
q701_C20b *(required)*	q701_C20b. What is the typical purchase price for that quanity of units (i.e. the quantity noted in the question above)?<br>*Question relevant when: not(selected(${q101_C20_full_list} , '6')) and not(selected(${q101_C20_full_list} , '99'))*<br>*Response constrained to: .>=0*	
q701_C20c *(required)*	q701_C20c. Please specify the currency for the purchase price.<br>*Question relevant when: not(selected(${q101_C20_full_list} , '6')) and not(selected(${q101_C20_full_list} , '99'))*	1 Tanzanian Shillings<br>2 US dollars<br>3 Euros<br>99 Don't know<br>5 Other
q701_C20c.1	q701_C20c.1 If other currency, specify:<br>*Leave blank if not applicable*<br>*Question relevant when: not(selected(${q101_C20_full_list} , '6')) and not(selected(${q101_C20_full_list} , '99'))*	
q701_C20d *(required)*	q701_C20d. Please specify the year of the purchase price.<br>*Question relevant when: not(selected(${q101_C20_full_list} , '6')) and not(selected(${q101_C20_full_list} , '99'))*<br>*Response constrained to: .>=2000 and .<=2019 or .=999*	
A. Consumables - Cost (3)<br>*Group relevant when: selected(${section_seven_skip_con} , '1')*		
note_701_bandages_etc	***Bandages, absorbants, wound care, etc.***	
q701_C21	***q701_C21. Adhesive tape***<br>*Question relevant when: not(selected(${q101_C21_full_list} , '6')) and not(selected(${q101_C21_full_list} , '99'))*	
q701_C21a *(required)*	q701_C21a. When purchased, how many units of this item typically come in one pack, box, bottle, etc.? (e.g. 100 tablets, 1000 ml's, etc.) (Write 1 if purchased as a single item.)<br>*Smallest unit is one roll of tape.*<br>*Question relevant when: not(selected(${q101_C21_full_list} , '6')) and not(selected(${q101_C21_full_list} , '99'))*<br>*Response constrained to: .>=0*	
q701_C21b *(required)*	q701_C21b. What is the typical purchase price for that quanity of units (i.e. the quantity noted in the question above)?<br>*Question relevant when: not(selected(${q101_C21_full_list} , '6')) and not(selected(${q101_C21_full_list} , '99'))*<br>*Response constrained to: .>=0*	
q701_C21c *(required)*	q701_C21c. Please specify the currency for the purchase price.<br>*Question relevant when: not(selected(${q101_C21_full_list} , '6')) and not(selected(${q101_C21_full_list} , '99'))*	1 Tanzanian Shillings<br>2 US dollars<br>3 Euros<br>99 Don't know<br>5 Other
q701_C21c.1	q701_C21c.1 If other currency, specify:<br>*Leave blank if not applicable*<br>*Question relevant when: not(selected(${q101_C21_full_list} , '6')) and not(selected(${q101_C21_full_list} , '99'))*	
q701_C21d *(required)*	q701_C21d. Please specify the year of the purchase price.<br>*Question relevant when: not(selected(${q101_C21_full_list} , '6')) and not(selected(${q101_C21_full_list} , '99'))*<br>*Response constrained to: .>=2000 and .<=2019 or .=999*	
q701_C22	***q701_C22. Cotton swab***<br>*Question relevant when: not(selected(${q101_C22_full_list} , '6')) and not(selected(${q101_C22_full_list} , '99'))*	
q701_C22a *(required)*	q701_C22a. When purchased, how many units of this item typically come in one pack, box, bottle, etc.? (e.g. 100 tablets, 1000 ml's, etc.) (Write 1 if purchased as a single item.)<br>*Smallest unit is one swab.*<br>*Question relevant when: not(selected(${q101_C22_full_list} , '6')) and not(selected(${q101_C22_full_list} , '99'))*<br>*Response constrained to: .>=0*	
q701_C22b *(required)*	q701_C22b. What is the typical purchase price for that quanity of units (i.e. the quantity noted in the question above)?<br>*Question relevant when: not(selected(${q101_C22_full_list} , '6')) and not(selected(${q101_C22_full_list} , '99'))*<br>*Response constrained to: .>=0*	

Field	Question	Answer
q701_C22c *(required)*	q701_C22c. Please specify the currency for the purchase price.<br>*Question relevant when: not(selected(${q101_C22_full_list} , '6')) and not(selected(${q101_C22_full_list} , '99'))*	1 Tanzanian Shillings<br>2 US dollars<br>3 Euros<br>99 Don't know<br>5 Other
q701_C22c.1	q701_C22c.1 If other currency, specify:<br>*Leave blank if not applicable*<br>*Question relevant when: not(selected(${q101_C22_full_list} , '6')) and not(selected(${q101_C22_full_list} , '99'))*	

q701_C22d *(required)*	q701_C22d. Please specify the year of the purchase price.<br>*Question relevant when: not(selected(${q101_C22_full_list} , '6')) and not(selected(${q101_C22_full_list} , '99'))*<br>*Response constrained to: .>=2000 and .<=2019 or .=999*	
q701_C23	***q701_C23. Dressing pad absorbent sterile***<br>*Question relevant when: not(selected(${q101_C23_full_list} , '6')) and not(selected(${q101_C23_full_list} , '99'))*	
q701_C23a *(required)*	q701_C23a. When purchased, how many units of this item typically come in one pack, box, bottle, etc.? (e.g. 100 tablets, 1000 ml's, etc.) (Write 1 if purchased as a single item.)<br>*Smallest unit is one pad.*<br>*Question relevant when: not(selected(${q101_C23_full_list} , '6')) and not(selected(${q101_C23_full_list} , '99'))*<br>*Response constrained to: .>=0*	
q701_C23b *(required)*	q701_C23b. What is the typical purchase price for that quanity of units (i.e. the quantity noted in the question above)?<br>*Question relevant when: not(selected(${q101_C23_full_list} , '6')) and not(selected(${q101_C23_full_list} , '99'))*<br>*Response constrained to: .>=0*	
q701_C23c *(required)*	q701_C23c. Please specify the currency for the purchase price.<br>*Question relevant when: not(selected(${q101_C23_full_list} , '6')) and not(selected(${q101_C23_full_list} , '99'))*	1 Tanzanian Shillings<br>2 US dollars<br>3 Euros<br>99 Don't know<br>5 Other
q701_C23c.1	q701_C23c.1 If other currency, specify:<br>*Leave blank if not applicable*<br>*Question relevant when: not(selected(${q101_C23_full_list} , '6')) and not(selected(${q101_C23_full_list} , '99'))*	
q701_C23d *(required)*	q701_C23d. Please specify the year of the purchase price.<br>*Question relevant when: not(selected(${q101_C23_full_list} , '6')) and not(selected(${q101_C23_full_list} , '99'))*<br>*Response constrained to: .>=2000 and .<=2019 or .=999*	
q701_C24	***q701_C24. Gauze compress, non sterile***<br>*Question relevant when: not(selected(${q101_C24_full_list} , '6')) and not(selected(${q101_C24_full_list} , '99'))*	
q701_C24a *(required)*	q701_C24a. When purchased, how many units of this item typically come in one pack, box, bottle, etc.? (e.g. 100 tablets, 1000 ml's, etc.) (Write 1 if purchased as a single item.)<br>*Smallest unit is one compress.*<br>*Question relevant when: not(selected(${q101_C24_full_list} , '6')) and not(selected(${q101_C24_full_list} , '99'))*<br>*Response constrained to: .>=0*	
q701_C24b *(required)*	q701_C24b. What is the typical purchase price for that quanity of units (i.e. the quantity noted in the question above)?<br>*Question relevant when: not(selected(${q101_C24_full_list} , '6')) and not(selected(${q101_C24_full_list} , '99'))*<br>*Response constrained to: .>=0*	
q701_C24c *(required)*	q701_C24c. Please specify the currency for the purchase price.<br>*Question relevant when: not(selected(${q101_C24_full_list} , '6')) and not(selected(${q101_C24_full_list} , '99'))*	1 Tanzanian Shillings<br>2 US dollars<br>3 Euros<br>99 Don't know<br>5 Other
q701_C24c.1	q701_C24c.1 If other currency, specify:<br>*Leave blank if not applicable*<br>*Question relevant when: not(selected(${q101_C24_full_list} , '6')) and not(selected(${q101_C24_full_list} , '99'))*	
q701_C24d *(required)*	q701_C24d. Please specify the year of the purchase price.<br>*Question relevant when: not(selected(${q101_C24_full_list} , '6')) and not(selected(${q101_C24_full_list} , '99'))*<br>*Response constrained to: .>=2000 and .<=2019 or .=999*	
q701_C25	***q701_C25. Gauze compress, sterile***<br>*Question relevant when: not(selected(${q101_C25_full_list} , '6')) and not(selected(${q101_C25_full_list} , '99'))*	
q701_C25a *(required)*	q701_C25a. When purchased, how many units of this item typically come in one pack, box, bottle, etc.? (e.g. 100 tablets, 1000 ml's, etc.) (Write 1 if purchased as a single item.)<br>*Smallest unit is one compress.*<br>*Question relevant when: not(selected(${q101_C25_full_list} , '6')) and not(selected(${q101_C25_full_list} , '99'))*<br>*Response constrained to: .>=0*	
q701_C25b *(required)*	q701_C25b. What is the typical purchase price for that quanity of units (i.e. the quantity noted in the question above)?<br>*Question relevant when: not(selected(${q101_C25_full_list} , '6')) and not(selected(${q101_C25_full_list} , '99'))*<br>*Response constrained to: .>=0*	

Field	Question	Answer
q701_C25c (required)	q701_C25c. Please specify the currency for the purchase price.<br>*Question relevant when: not(selected(${q101_C25_full_list} , '6')) and not(selected(${q101_C25_full_list} , '99'))*	1 Tanzanian Shillings<br>2 US dollars<br>3 Euros<br>99 Don't know<br>5 Other
q701_C25c.1	q701_C25c.1 If other currency, specify:<br>*Leave blank if not applicable*<br>*Question relevant when: not(selected(${q101_C25_full_list} , '6')) and not(selected(${q101_C25_full_list} , '99'))*	
q701_C25d (required)	q701_C25d. Please specify the year of the purchase price.<br>*Question relevant when: not(selected(${q101_C25_full_list} , '6')) and not(selected(${q101_C25_full_list} , '99'))*<br>*Response constrained to: .>=2000 and .<=2019 or .=999*	
q701_C26	***q701_C26. Linen saver***<br>*Question relevant when: not(selected(${q101_C26_full_list} , '6')) and not(selected(${q101_C26_full_list} , '99'))*	
q701_C26a (required)	q701_C26a. When purchased, how many units of this item typically come in one pack, box, bottle, etc.? (e.g. 100 tablets, 1000 ml's, etc.) (Write 1 if purchased as a single item.)<br>*Smallest unit is one saver (absorbant sheet).*<br>*Question relevant when: not(selected(${q101_C26_full_list} , '6')) and not(selected(${q101_C26_full_list} , '99'))*<br>*Response constrained to: .>=0*	
q701_C26b (required)	q701_C26b. What is the typical purchase price for that quanity of units (i.e. the quantity noted in the question above)?<br>*Question relevant when: not(selected(${q101_C26_full_list} , '6')) and not(selected(${q101_C26_full_list} , '99'))*<br>*Response constrained to: .>=0*	
q701_C26c (required)	q701_C26c. Please specify the currency for the purchase price.<br>*Question relevant when: not(selected(${q101_C26_full_list} , '6')) and not(selected(${q101_C26_full_list} , '99'))*	1 Tanzanian Shillings<br>2 US dollars<br>3 Euros<br>99 Don't know<br>5 Other
q701_C26c.1	q701_C26c.1 If other currency, specify:<br>*Leave blank if not applicable*<br>*Question relevant when: not(selected(${q101_C26_full_list} , '6')) and not(selected(${q101_C26_full_list} , '99'))*	
q701_C26d (required)	q701_C26d. Please specify the year of the purchase price.<br>*Question relevant when: not(selected(${q101_C26_full_list} , '6')) and not(selected(${q101_C26_full_list} , '99'))*<br>*Response constrained to: .>=2000 and .<=2019 or .=999*	
q701_C27	***q701_C27. Sanitary Pad***<br>*Question relevant when: not(selected(${q101_C27_full_list} , '6')) and not(selected(${q101_C27_full_list} , '99'))*	
q701_C27a (required)	q701_C27a. When purchased, how many units of this item typically come in one pack, box, bottle, etc.? (e.g. 100 tablets, 1000 ml's, etc.) (Write 1 if purchased as a single item.)<br>*Smallest unit is one pad.*<br>*Question relevant when: not(selected(${q101_C27_full_list} , '6')) and not(selected(${q101_C27_full_list} , '99'))*<br>*Response constrained to: .>=0*	
q701_C27b (required)	q701_C27b. What is the typical purchase price for that quanity of units (i.e. the quantity noted in the question above)?<br>*Question relevant when: not(selected(${q101_C27_full_list} , '6')) and not(selected(${q101_C27_full_list} , '99'))*<br>*Response constrained to: .>=0*	
q701_C27c (required)	q701_C27c. Please specify the currency for the purchase price.<br>*Question relevant when: not(selected(${q101_C27_full_list} , '6')) and not(selected(${q101_C27_full_list} , '99'))*	1 Tanzanian Shillings<br>2 US dollars<br>3 Euros<br>99 Don't know<br>5 Other
q701_C27c.1	q701_C27c.1 If other currency, specify:<br>*Leave blank if not applicable*<br>*Question relevant when: not(selected(${q101_C27_full_list} , '6')) and not(selected(${q101_C27_full_list} , '99'))*	
q701_C27d (required)	q701_C27d. Please specify the year of the purchase price.<br>*Question relevant when: not(selected(${q101_C27_full_list} , '6')) and not(selected(${q101_C27_full_list} , '99'))*<br>*Response constrained to: .>=2000 and .<=2019 or .=999*	
q701_C28	***q701_C28. Surgical plaster***<br>*Question relevant when: not(selected(${q101_C28_full_list} , '6')) and not(selected(${q101_C28_full_list} , '99'))*	
q701_C28a (required)	q701_C28a. When purchased, how many units of this item typically come in one pack, box, bottle, etc.? (e.g. 100 tablets, 1000 ml's, etc.) (Write 1 if purchased as a single item.)<br>*Smallest unit is one plaster.*<br>*Question relevant when: not(selected(${q101_C28_full_list} , '6')) and not(selected(${q101_C28_full_list} , '99'))*<br>*Response constrained to: .>=0*	
q701_C28b (required)	q701_C28b. What is the typical purchase price for that quanity of units (i.e. the quantity noted in the question above)?<br>*Question relevant when: not(selected(${q101_C28_full_list} , '6')) and not(selected(${q101_C28_full_list} , '99'))*<br>*Response constrained to: .>=0*	

Field	Question	Answer
q701_C28c *(required)*	q701_C28c. Please specify the currency for the purchase price.<br>*Question relevant when: not(selected(${q101_C28_full_list} , '6')) and not(selected(${q101_C28_full_list} , '99'))*	1 Tanzanian Shillings<br>2 US dollars<br>3 Euros<br>99 Don't know<br>5 Other
q701_C28c.1	q701_C28c.1 If other currency, specify:<br>*Leave blank if not applicable*<br>*Question relevant when: not(selected(${q101_C28_full_list} , '6')) and not(selected(${q101_C28_full_list} , '99'))*	
q701_C28d *(required)*	q701_C28d. Please specify the year of the purchase price.<br>*Question relevant when: not(selected(${q101_C28_full_list} , '6')) and not(selected(${q101_C28_full_list} , '99'))*<br>*Response constrained to: .>=2000 and .<=2019 or .=999*	
A. Consumables - Cost (4)<br>*Group relevant when: selected(${section_seven_skip_con} , '1')*		
note_701_protective_etc	***Protective supplies***	
q701_C29	***q701_C29. Apron (disposable)***<br>*Question relevant when: not(selected(${q101_C29_full_list} , '6')) and not(selected(${q101_C29_full_list} , '99'))*	
q701_C29a *(required)*	q701_C29a. When purchased, how many units of this item typically come in one pack, box, bottle, etc.? (e.g. 100 tablets, 1000 ml's, etc.) (Write 1 if purchased as a single item.)<br>*Smallest unit is one apron.*<br>*Question relevant when: not(selected(${q101_C29_full_list} , '6')) and not(selected(${q101_C29_full_list} , '99'))*<br>*Response constrained to: .>=0*	
q701_C29b *(required)*	q701_C29b. What is the typical purchase price for that quanity of units (i.e. the quantity noted in the question above)?<br>*Question relevant when: not(selected(${q101_C29_full_list} , '6')) and not(selected(${q101_C29_full_list} , '99'))*<br>*Response constrained to: .>=0*	
q701_C29c *(required)*	q701_C29c. Please specify the currency for the purchase price.<br>*Question relevant when: not(selected(${q101_C29_full_list} , '6')) and not(selected(${q101_C29_full_list} , '99'))*	1 Tanzanian Shillings<br>2 US dollars<br>3 Euros<br>99 Don't know<br>5 Other
q701_C29c.1	q701_C29c.1 If other currency, specify:<br>*Leave blank if not applicable*<br>*Question relevant when: not(selected(${q101_C29_full_list} , '6')) and not(selected(${q101_C29_full_list} , '99'))*	
q701_C29d *(required)*	q701_C29d. Please specify the year of the purchase price.<br>*Question relevant when: not(selected(${q101_C29_full_list} , '6')) and not(selected(${q101_C29_full_list} , '99'))*<br>*Response constrained to: .>=2000 and .<=2019 or .=999*	
q701_C30	***q701_C30. Gown (disposable)***<br>*Question relevant when: not(selected(${q101_C30_full_list} , '6')) and not(selected(${q101_C30_full_list} , '99'))*	
q701_C30a *(required)*	q701_C30a. When purchased, how many units of this item typically come in one pack, box, bottle, etc.? (e.g. 100 tablets, 1000 ml's, etc.) (Write 1 if purchased as a single item.)<br>*Smallest unit is one gown.*<br>*Question relevant when: not(selected(${q101_C30_full_list} , '6')) and not(selected(${q101_C30_full_list} , '99'))*<br>*Response constrained to: .>=0*	
q701_C30b *(required)*	q701_C30b. What is the typical purchase price for that quanity of units (i.e. the quantity noted in the question above)?<br>*Question relevant when: not(selected(${q101_C30_full_list} , '6')) and not(selected(${q101_C30_full_list} , '99'))*<br>*Response constrained to: .>=0*	
q701_C30c *(required)*	q701_C30c. Please specify the currency for the purchase price.<br>*Question relevant when: not(selected(${q101_C30_full_list} , '6')) and not(selected(${q101_C30_full_list} , '99'))*	1 Tanzanian Shillings<br>2 US dollars<br>3 Euros<br>99 Don't know<br>5 Other
q701_C30c.1	q701_C30c.1 If other currency, specify:<br>*Leave blank if not applicable*<br>*Question relevant when: not(selected(${q101_C30_full_list} , '6')) and not(selected(${q101_C30_full_list} , '99'))*	
q701_C30d *(required)*	q701_C30d. Please specify the year of the purchase price.<br>*Question relevant when: not(selected(${q101_C30_full_list} , '6')) and not(selected(${q101_C30_full_list} , '99'))*<br>*Response constrained to: .>=2000 and .<=2019 or .=999*	
q701_C31	***q701_C31. Latex examination glove, without powder***<br>*Question relevant when: not(selected(${q101_C31_full_list} , '6')) and not(selected(${q101_C31_full_list} , '99'))*	
q701_C31a *(required)*	q701_C31a. When purchased, how many units of this item typically come in one pack, box, bottle, etc.? (e.g. 100 tablets, 1000 ml's, etc.) (Write 1 if purchased as a single item.)<br>*Smallest unit is one glove.*<br>*Question relevant when: not(selected(${q101_C31_full_list} , '6')) and not(selected(${q101_C31_full_list} , '99'))*<br>*Response constrained to: .>=0*	

Field	Question	Answer
q701_C31b *(required)*	q701_C31b. What is the typical purchase price for that quanity of units (i.e. the quantity noted in the question above)?<br>*Question relevant when: not(selected(${q101_C31_full_list} , '6')) and not(selected(${q101_C31_full_list} , '99'))*<br>*Response constrained to: .>=0*	
q701_C31c *(required)*	q701_C31c. Please specify the currency for the purchase price.<br>*Question relevant when: not(selected(${q101_C31_full_list} , '6')) and not(selected(${q101_C31_full_list} , '99'))*	1 Tanzanian Shillings<br>2 US dollars<br>3 Euros<br>99 Don't know<br>5 Other

q701_C31c.1	q701_C31c.1 If other currency, specify:<br>*Leave blank if not applicable*<br>*Question relevant when: not(selected(${q101_C31_full_list} , '6')) and not(selected(${q101_C31_full_list} , '99'))*	
q701_C31d *(required)*	q701_C31d. Please specify the year of the purchase price.<br>*Question relevant when: not(selected(${q101_C31_full_list} , '6')) and not(selected(${q101_C31_full_list} , '99'))*<br>*Response constrained to: .>=2000 and .<=2019 or .=999*	
q701_C32	***q701_C32. Latex examination glove, with powder***<br>*Question relevant when: not(selected(${q101_C32_full_list} , '6')) and not(selected(${q101_C32_full_list} , '99'))*	
q701_C32a *(required)*	q701_C32a. When purchased, how many units of this item typically come in one pack, box, bottle, etc.? (e.g. 100 tablets, 1000 ml's, etc.) (Write 1 if purchased as a single item.)<br>*Smallest unit is one glove.*<br>*Question relevant when: not(selected(${q101_C32_full_list} , '6')) and not(selected(${q101_C32_full_list} , '99'))*<br>*Response constrained to: .>=0*	
q701_C32b *(required)*	q701_C32b. What is the typical purchase price for that quanity of units (i.e. the quantity noted in the question above)?<br>*Question relevant when: not(selected(${q101_C32_full_list} , '6')) and not(selected(${q101_C32_full_list} , '99'))*<br>*Response constrained to: .>=0*	
q701_C32c *(required)*	q701_C32c. Please specify the currency for the purchase price.<br>*Question relevant when: not(selected(${q101_C32_full_list} , '6')) and not(selected(${q101_C32_full_list} , '99'))*	1 Tanzanian Shillings<br>2 US dollars<br>3 Euros<br>99 Don't know<br>5 Other
q701_C32c.1	q701_C32c.1 If other currency, specify:<br>*Leave blank if not applicable*<br>*Question relevant when: not(selected(${q101_C32_full_list} , '6')) and not(selected(${q101_C32_full_list} , '99'))*	
q701_C32d *(required)*	q701_C32d. Please specify the year of the purchase price.<br>*Question relevant when: not(selected(${q101_C32_full_list} , '6')) and not(selected(${q101_C32_full_list} , '99'))*<br>*Response constrained to: .>=2000 and .<=2019 or .=999*	
q701_C33	***q701_C33. Non-latex (e.g. nitrile) examination glove***<br>*Question relevant when: not(selected(${q101_C33_full_list} , '6')) and not(selected(${q101_C33_full_list} , '99'))*	
q701_C33a *(required)*	q701_C33a. When purchased, how many units of this item typically come in one pack, box, bottle, etc.? (e.g. 100 tablets, 1000 ml's, etc.) (Write 1 if purchased as a single item.)<br>*Smallest unit is one glove.*<br>*Question relevant when: not(selected(${q101_C33_full_list} , '6')) and not(selected(${q101_C33_full_list} , '99'))*<br>*Response constrained to: .>=0*	
q701_C33b *(required)*	q701_C33b. What is the typical purchase price for that quanity of units (i.e. the quantity noted in the question above)?<br>*Question relevant when: not(selected(${q101_C33_full_list} , '6')) and not(selected(${q101_C33_full_list} , '99'))*<br>*Response constrained to: .>=0*	
q701_C33c *(required)*	q701_C33c. Please specify the currency for the purchase price.<br>*Question relevant when: not(selected(${q101_C33_full_list} , '6')) and not(selected(${q101_C33_full_list} , '99'))*	1 Tanzanian Shillings<br>2 US dollars<br>3 Euros<br>99 Don't know<br>5 Other
q701_C33c.1	q701_C33c.1 If other currency, specify:<br>*Leave blank if not applicable*<br>*Question relevant when: not(selected(${q101_C33_full_list} , '6')) and not(selected(${q101_C33_full_list} , '99'))*	
q701_C33d *(required)*	q701_C33d. Please specify the year of the purchase price.<br>*Question relevant when: not(selected(${q101_C33_full_list} , '6')) and not(selected(${q101_C33_full_list} , '99'))*<br>*Response constrained to: .>=2000 and .<=2019 or .=999*	
q701_C34	***q701_C34. Sterile surgical glove***<br>*Question relevant when: not(selected(${q101_C34_full_list} , '6')) and not(selected(${q101_C34_full_list} , '99'))*	
q701_C34a *(required)*	q701_C34a. When purchased, how many units of this item typically come in one pack, box, bottle, etc.? (e.g. 100 tablets, 1000 ml's, etc.) (Write 1 if purchased as a single item.)<br>*Smallest unit is one glove.*<br>*Question relevant when: not(selected(${q101_C34_full_list} , '6')) and not(selected(${q101_C34_full_list} , '99'))*<br>*Response constrained to: .>=0*	
q701_C34b *(required)*	q701_C34b. What is the typical purchase price for that quanity of units (i.e. the quantity noted in the question above)?	

Field	Question	Answer
q701_C34b *(required)*	q701_C34b. What is the typical purchase price for that quanity of units (i.e. the quantity noted in the question above)?<br>*Question relevant when: not(selected(${q101_C34_full_list} , '6')) and not(selected(${q101_C34_full_list} , '99'))*<br>*Response constrained to: .>=0*	
q701_C34c *(required)*	q701_C34c. Please specify the currency for the purchase price.<br>*Question relevant when: not(selected(${q101_C34_full_list} , '6')) and not(selected(${q101_C34_full_list} , '99'))*	1 Tanzanian Shillings<br>2 US dollars<br>3 Euros<br>99 Don't know<br>5 Other
q701_C34c.1	q701_C34c.1 If other currency, specify:<br>*Leave blank if not applicable*<br>*Question relevant when: not(selected(${q101_C34_full_list} , '6')) and not(selected(${q101_C34_full_list} , '99'))*	
q701_C34d *(required)*	q701_C34d. Please specify the year of the purchase price.<br>*Question relevant when: not(selected(${q101_C34_full_list} , '6')) and not(selected(${q101_C34_full_list} , '99'))*<br>*Response constrained to: .>=2000 and .<=2019 or .=999*	
q701_C35	***q701_C35. Mask (disposable)***<br>*Question relevant when: not(selected(${q101_C35_full_list} , '6')) and not(selected(${q101_C35_full_list} , '99'))*	
q701_C35a *(required)*	q701_C35a. When purchased, how many units of this item typically come in one pack, box, bottle, etc.? (e.g. 100 tablets, 1000 ml's, etc.) (Write 1 if purchased as a single item.)<br>*Smallest unit is one mask.*<br>*Question relevant when: not(selected(${q101_C35_full_list} , '6')) and not(selected(${q101_C35_full_list} , '99'))*<br>*Response constrained to: .>=0*	
q701_C35b *(required)*	q701_C35b. What is the typical purchase price for that quanity of units (i.e. the quantity noted in the question above)?<br>*Question relevant when: not(selected(${q101_C35_full_list} , '6')) and not(selected(${q101_C35_full_list} , '99'))*<br>*Response constrained to: .>=0*	
q701_C35c *(required)*	q701_C35c. Please specify the currency for the purchase price.<br>*Question relevant when: not(selected(${q101_C35_full_list} , '6')) and not(selected(${q101_C35_full_list} , '99'))*	1 Tanzanian Shillings<br>2 US dollars<br>3 Euros<br>99 Don't know<br>5 Other
q701_C35c.1	q701_C35c.1 If other currency, specify:<br>*Leave blank if not applicable*<br>*Question relevant when: not(selected(${q101_C35_full_list} , '6')) and not(selected(${q101_C35_full_list} , '99'))*	
q701_C35d *(required)*	q701_C35d. Please specify the year of the purchase price.<br>*Question relevant when: not(selected(${q101_C35_full_list} , '6')) and not(selected(${q101_C35_full_list} , '99'))*<br>*Response constrained to: .>=2000 and .<=2019 or .=999*	
q701_C36	***q701_C36. Surgical cap (disposable)***<br>*Question relevant when: not(selected(${q101_C36_full_list} , '6')) and not(selected(${q101_C36_full_list} , '99'))*	
q701_C36a *(required)*	q701_C36a. When purchased, how many units of this item typically come in one pack, box, bottle, etc.? (e.g. 100 tablets, 1000 ml's, etc.) (Write 1 if purchased as a single item.)<br>*Smallest unit is one cap.*<br>*Question relevant when: not(selected(${q101_C36_full_list} , '6')) and not(selected(${q101_C36_full_list} , '99'))*<br>*Response constrained to: .>=0*	
q701_C36b *(required)*	q701_C36b. What is the typical purchase price for that quanity of units (i.e. the quantity noted in the question above)?<br>*Question relevant when: not(selected(${q101_C36_full_list} , '6')) and not(selected(${q101_C36_full_list} , '99'))*<br>*Response constrained to: .>=0*	
q701_C36c *(required)*	q701_C36c. Please specify the currency for the purchase price.<br>*Question relevant when: not(selected(${q101_C36_full_list} , '6')) and not(selected(${q101_C36_full_list} , '99'))*	1 Tanzanian Shillings<br>2 US dollars<br>3 Euros<br>99 Don't know<br>5 Other
q701_C36c.1	q701_C36c.1 If other currency, specify:<br>*Leave blank if not applicable*<br>*Question relevant when: not(selected(${q101_C36_full_list} , '6')) and not(selected(${q101_C36_full_list} , '99'))*	
q701_C36d *(required)*	q701_C36d. Please specify the year of the purchase price.<br>*Question relevant when: not(selected(${q101_C36_full_list} , '6')) and not(selected(${q101_C36_full_list} , '99'))*<br>*Response constrained to: .>=2000 and .<=2019 or .=999*	
A. Consumables - Cost (5)<br>*Group relevant when: selected(${section_seven_skip_con} , '1')*		
note_701_surgery_etc	***Surgery supplies***	
q701_C37	***q701_C37. Scalpel blade, single use***<br>*Question relevant when: not(selected(${q101_C37_full_list} , '6')) and not(selected(${q101_C37_full_list} , '99'))*	
q701_C37a *(required)*	q701_C37a. When purchased, how many units of this item typically come in one pack, box, bottle, etc.? (e.g. 100 tablets, 1000 ml's, etc.) (Write 1 if purchased as a single item.)	

Field	Question	Answer
	Smallest unit is one blade.<br>*Question relevant when: not(selected(${q101_C37_full_list} , '6')) and not(selected(${q101_C37_full_list} , '99'))*<br>*Response constrained to: .>=0*	
q701_C37b *(required)*	q701_C37b. What is the typical purchase price for that quanity of units (i.e. the quantity noted in the question above)?<br>*Question relevant when: not(selected(${q101_C37_full_list} , '6')) and not(selected(${q101_C37_full_list} , '99'))*<br>*Response constrained to: .>=0*	
q701_C37c *(required)*	q701_C37c. Please specify the currency for the purchase price.<br>*Question relevant when: not(selected(${q101_C37_full_list} , '6')) and not(selected(${q101_C37_full_list} , '99'))*	1 Tanzanian Shillings<br>2 US dollars<br>3 Euros<br>99 Don't know<br>5 Other
q701_C37c.1	q701_C37c.1 If other currency, specify:<br>*Leave blank if not applicable*<br>*Question relevant when: not(selected(${q101_C37_full_list} , '6')) and not(selected(${q101_C37_full_list} , '99'))*	
q701_C37d *(required)*	q701_C37d. Please specify the year of the purchase price.<br>*Question relevant when: not(selected(${q101_C37_full_list} , '6')) and not(selected(${q101_C37_full_list} , '99'))*<br>*Response constrained to: .>=2000 and .<=2019 or .=999*	
q701_C38	***q701_C38. Surgical drain***<br>*Question relevant when: not(selected(${q101_C38_full_list} , '6')) and not(selected(${q101_C38_full_list} , '99'))*	
q701_C38a *(required)*	q701_C38a. When purchased, how many units of this item typically come in one pack, box, bottle, etc.? (e.g. 100 tablets, 1000 ml's, etc.) (Write 1 if purchased as a single item.)<br>*Smallest unit is one drain.*<br>*Question relevant when: not(selected(${q101_C38_full_list} , '6')) and not(selected(${q101_C38_full_list} , '99'))*<br>*Response constrained to: .>=0*	
q701_C38b *(required)*	q701_C38b. What is the typical purchase price for that quanity of units (i.e. the quantity noted in the question above)?<br>*Question relevant when: not(selected(${q101_C38_full_list} , '6')) and not(selected(${q101_C38_full_list} , '99'))*<br>*Response constrained to: .>=0*	
q701_C38c *(required)*	q701_C38c. Please specify the currency for the purchase price.<br>*Question relevant when: not(selected(${q101_C38_full_list} , '6')) and not(selected(${q101_C38_full_list} , '99'))*	1 Tanzanian Shillings<br>2 US dollars<br>3 Euros<br>99 Don't know<br>5 Other
q701_C38c.1	q701_C38c.1 If other currency, specify:<br>*Leave blank if not applicable*<br>*Question relevant when: not(selected(${q101_C38_full_list} , '6')) and not(selected(${q101_C38_full_list} , '99'))*	
q701_C38d *(required)*	q701_C38d. Please specify the year of the purchase price.<br>*Question relevant when: not(selected(${q101_C38_full_list} , '6')) and not(selected(${q101_C38_full_list} , '99'))*<br>*Response constrained to: .>=2000 and .<=2019 or .=999*	
q701_C39	***q701_C39. Suture string, chromic catgut (0)***<br>*Question relevant when: not(selected(${q101_C39_full_list} , '6')) and not(selected(${q101_C39_full_list} , '99'))*	
q701_C39a *(required)*	q701_C39a. When purchased, how many units of this item typically come in one pack, box, bottle, etc.? (e.g. 100 tablets, 1000 ml's, etc.) (Write 1 if purchased as a single item.)<br>*Smallest unit is one suture set.*<br>*Question relevant when: not(selected(${q101_C39_full_list} , '6')) and not(selected(${q101_C39_full_list} , '99'))*<br>*Response constrained to: .>=0*	
q701_C39b *(required)*	q701_C39b. What is the typical purchase price for that quanity of units (i.e. the quantity noted in the question above)?<br>*Question relevant when: not(selected(${q101_C39_full_list} , '6')) and not(selected(${q101_C39_full_list} , '99'))*<br>*Response constrained to: .>=0*	
q701_C39c *(required)*	q701_C39c. Please specify the currency for the purchase price.<br>*Question relevant when: not(selected(${q101_C39_full_list} , '6')) and not(selected(${q101_C39_full_list} , '99'))*	1 Tanzanian Shillings<br>2 US dollars<br>3 Euros<br>99 Don't know<br>5 Other
q701_C39c.1	q701_C39c.1 If other currency, specify:<br>*Leave blank if not applicable*<br>*Question relevant when: not(selected(${q101_C39_full_list} , '6')) and not(selected(${q101_C39_full_list} , '99'))*	
q701_C39d *(required)*	q701_C39d. Please specify the year of the purchase price.<br>*Question relevant when: not(selected(${q101_C39_full_list} , '6')) and not(selected(${q101_C39_full_list} , '99'))*<br>*Response constrained to: .>=2000 and .<=2019 or .=999*	
q701_C40	***q701_C40. Suture string, silk (non absorbable and non-synthetic)***<br>*Question relevant when: not(selected(${q101_C40_full_list} , '6')) and not(selected(${q101_C40_full_list} , '99'))*	

Field	Question	Answer
q701_C40a *(required)*	q701_C40a. When purchased, how many units of this item typically come in one pack, box, bottle, etc.? (e.g. 100 tablets, 1000 ml's, etc.) (Write 1 if purchased as a single item.)<br>*Smallest unit is one suture set.*<br>*Question relevant when: not(selected(${q101_C40_full_list} , '6')) and not(selected(${q101_C40_full_list} , '99'))*<br>*Response constrained to: .>=0*	
q701_C40b *(required)*	q701_C40b. What is the typical purchase price for that quanity of units (i.e. the quantity noted in the question above)?<br>*Question relevant when: not(selected(${q101_C40_full_list} , '6')) and not(selected(${q101_C40_full_list} , '99'))*<br>*Response constrained to: .>=0*	
q701_C40c *(required)*	q701_C40c. Please specify the currency for the purchase price.<br>*Question relevant when: not(selected(${q101_C40_full_list} , '6')) and not(selected(${q101_C40_full_list} , '99'))*	1 Tanzanian Shillings<br>2 US dollars<br>3 Euros<br>99 Don't know<br>5 Other
q701_C40c.1	q701_C40c.1 If other currency, specify:<br>*Leave blank if not applicable*<br>*Question relevant when: not(selected(${q101_C40_full_list} , '6')) and not(selected(${q101_C40_full_list} , '99'))*	
q701_C40d *(required)*	q701_C40d. Please specify the year of the purchase price.<br>*Question relevant when: not(selected(${q101_C40_full_list} , '6')) and not(selected(${q101_C40_full_list} , '99'))*<br>*Response constrained to: .>=2000 and .<=2019 or .=999*	
q701_C41	***q701_C41. Suture string, vicryl, no needle included***<br>*Question relevant when: not(selected(${q101_C41_full_list} , '6')) and not(selected(${q101_C41_full_list} , '99'))*	
q701_C41a *(required)*	q701_C41a. When purchased, how many units of this item typically come in one pack, box, bottle, etc.? (e.g. 100 tablets, 1000 ml's, etc.) (Write 1 if purchased as a single item.)<br>*Smallest unit is one suture set.*<br>*Question relevant when: not(selected(${q101_C41_full_list} , '6')) and not(selected(${q101_C41_full_list} , '99'))*<br>*Response constrained to: .>=0*	
q701_C41b *(required)*	q701_C41b. What is the typical purchase price for that quanity of units (i.e. the quantity noted in the question above)?<br>*Question relevant when: not(selected(${q101_C41_full_list} , '6')) and not(selected(${q101_C41_full_list} , '99'))*<br>*Response constrained to: .>=0*	
q701_C41c *(required)*	q701_C41c. Please specify the currency for the purchase price.<br>*Question relevant when: not(selected(${q101_C41_full_list} , '6')) and not(selected(${q101_C41_full_list} , '99'))*	1 Tanzanian Shillings<br>2 US dollars<br>3 Euros<br>99 Don't know<br>5 Other
q701_C41c.1	q701_C41c.1 If other currency, specify:<br>*Leave blank if not applicable*<br>*Question relevant when: not(selected(${q101_C41_full_list} , '6')) and not(selected(${q101_C41_full_list} , '99'))*	
q701_C41d *(required)*	q701_C41d. Please specify the year of the purchase price.<br>*Question relevant when: not(selected(${q101_C41_full_list} , '6')) and not(selected(${q101_C41_full_list} , '99'))*<br>*Response constrained to: .>=2000 and .<=2019 or .=999*	
q701_C42	***q701_C42. Suture string, vicryl with needle***<br>*Question relevant when: not(selected(${q101_C42_full_list} , '6')) and not(selected(${q101_C42_full_list} , '99'))*	
q701_C42a *(required)*	q701_C42a. When purchased, how many units of this item typically come in one pack, box, bottle, etc.? (e.g. 100 tablets, 1000 ml's, etc.) (Write 1 if purchased as a single item.)<br>*Smallest unit is one suture set with needle..*<br>*Question relevant when: not(selected(${q101_C42_full_list} , '6')) and not(selected(${q101_C42_full_list} , '99'))*<br>*Response constrained to: .>=0*	
q701_C42b *(required)*	q701_C42b. What is the typical purchase price for that quanity of units (i.e. the quantity noted in the question above)?<br>*Question relevant when: not(selected(${q101_C42_full_list} , '6')) and not(selected(${q101_C42_full_list} , '99'))*<br>*Response constrained to: .>=0*	
q701_C42c *(required)*	q701_C42c. Please specify the currency for the purchase price.<br>*Question relevant when: not(selected(${q101_C42_full_list} , '6')) and not(selected(${q101_C42_full_list} , '99'))*	1 Tanzanian Shillings<br>2 US dollars<br>3 Euros<br>99 Don't know<br>5 Other
q701_C42c.1	q701_C42c.1 If other currency, specify:<br>*Leave blank if not applicable*<br>*Question relevant when: not(selected(${q101_C42_full_list} , '6')) and not(selected(${q101_C42_full_list} , '99'))*	
q701_C42d *(required)*	q701_C42d. Please specify the year of the purchase price.<br>*Question relevant when: not(selected(${q101_C42_full_list} , '6')) and not(selected(${q101_C42_full_list} , '99'))*<br>*Response constrained to: .>=2000 and .<=2019 or .=999*	

A. Consumables - Cost (6)

Group relevant when: selected(${section_seven_skip_con} , '1')

Field	Question	Answer
note_701_urine_etc	***Urine, bladder***	
q701_C43	***q701_C43. Bladder/urinary catheter***	
	Question relevant when: not(selected(${q101_C43_full_list} , '6')) and not(selected(${q101_C43_full_list} , '99'))	
q701_C43a *(required)*	q701_C43a. When purchased, how many units of this item typically come in one pack, box, bottle, etc.? (e.g. 100 tablets, 1000 ml's, etc.) (Write 1 if purchased as a single item.)<br>*Smallest unit is one catheter.*<br>*Question relevant when: not(selected(${q101_C43_full_list} , '6')) and not(selected(${q101_C43_full_list} , '99'))*<br>*Response constrained to: .>=0*	

Field	Question	Answer
q701_C43b *(required)*	q701_C43b. What is the typical purchase price for that quanity of units (i.e. the quantity noted in the question above)?<br>*Question relevant when: not(selected(${q101_C43_full_list} , '6')) and not(selected(${q101_C43_full_list} , '99'))*<br>*Response constrained to: .>=0*	
q701_C43c *(required)*	q701_C43c. Please specify the currency for the purchase price.<br>*Question relevant when: not(selected(${q101_C43_full_list} , '6')) and not(selected(${q101_C43_full_list} , '99'))*	1 Tanzanian Shillings<br>2 US dollars<br>3 Euros<br>99 Don't know<br>5 Other
q701_C43c.1	q701_C43c.1 If other currency, specify:<br>*Leave blank if not applicable*<br>*Question relevant when: not(selected(${q101_C43_full_list} , '6')) and not(selected(${q101_C43_full_list} , '99'))*	
q701_C43d *(required)*	q701_C43d. Please specify the year of the purchase price.<br>*Question relevant when: not(selected(${q101_C43_full_list} , '6')) and not(selected(${q101_C43_full_list} , '99'))*<br>*Response constrained to: .>=2000 and .<=2019 or .=999*	
q701_C44	***q701_C44. Foley's catheter***<br>*Question relevant when: not(selected(${q101_C44_full_list} , '6')) and not(selected(${q101_C44_full_list} , '99'))*	
q701_C44a *(required)*	q701_C44a. When purchased, how many units of this item typically come in one pack, box, bottle, etc.? (e.g. 100 tablets, 1000 ml's, etc.) (Write 1 if purchased as a single item.)<br>*Smallest unit is one catheter.*<br>*Question relevant when: not(selected(${q101_C44_full_list} , '6')) and not(selected(${q101_C44_full_list} , '99'))*<br>*Response constrained to: .>=0*	
q701_C44b *(required)*	q701_C44b. What is the typical purchase price for that quanity of units (i.e. the quantity noted in the question above)?<br>*Question relevant when: not(selected(${q101_C44_full_list} , '6')) and not(selected(${q101_C44_full_list} , '99'))*<br>*Response constrained to: .>=0*	
q701_C44c *(required)*	q701_C44c. Please specify the currency for the purchase price.<br>*Question relevant when: not(selected(${q101_C44_full_list} , '6')) and not(selected(${q101_C44_full_list} , '99'))*	1 Tanzanian Shillings<br>2 US dollars<br>3 Euros<br>99 Don't know<br>5 Other
q701_C44c.1	q701_C44c.1 If other currency, specify:<br>*Leave blank if not applicable*<br>*Question relevant when: not(selected(${q101_C44_full_list} , '6')) and not(selected(${q101_C44_full_list} , '99'))*	
q701_C44d *(required)*	q701_C44d. Please specify the year of the purchase price.<br>*Question relevant when: not(selected(${q101_C44_full_list} , '6')) and not(selected(${q101_C44_full_list} , '99'))*<br>*Response constrained to: .>=2000 and .<=2019 or .=999*	
q701_C45	***q701_C45. Urine bag with valve and drain***<br>*Question relevant when: not(selected(${q101_C45_full_list} , '6')) and not(selected(${q101_C45_full_list} , '99'))*	
q701_C45a *(required)*	q701_C45a. When purchased, how many units of this item typically come in one pack, box, bottle, etc.? (e.g. 100 tablets, 1000 ml's, etc.) (Write 1 if purchased as a single item.)<br>*Smallest unit is one bag.*<br>*Question relevant when: not(selected(${q101_C45_full_list} , '6')) and not(selected(${q101_C45_full_list} , '99'))*<br>*Response constrained to: .>=0*	
q701_C45b *(required)*	q701_C45b. What is the typical purchase price for that quanity of units (i.e. the quantity noted in the question above)?<br>*Question relevant when: not(selected(${q101_C45_full_list} , '6')) and not(selected(${q101_C45_full_list} , '99'))*<br>*Response constrained to: .>=0*	
q701_C45c *(required)*	q701_C45c. Please specify the currency for the purchase price.<br>*Question relevant when: not(selected(${q101_C45_full_list} , '6')) and not(selected(${q101_C45_full_list} , '99'))*	1 Tanzanian Shillings<br>2 US dollars<br>3 Euros<br>99 Don't know<br>5 Other
q701_C45c.1	q701_C45c.1 If other currency, specify:<br>*Leave blank if not applicable*<br>*Question relevant when: not(selected(${q101_C45_full_list} , '6')) and not(selected(${q101_C45_full_list} , '99'))*	
q701_C45d *(required)*	q701_C45d. Please specify the year of the purchase price.<br>*Question relevant when: not(selected(${q101_C45_full_list} , '6')) and not(selected(${q101_C45_full_list} , '99'))*	

Field	Question	Answer
A. Consumables - Cost (7)	*Response constrained to: .>=2000 and .<=2019 or .=999*	
Group relevant when: selected(${section_seven_skip_con} , '1')		
note_701_other_etc	***Other supplies***	
q701_C46	***q701_C46. Silicone oil for lubrication of MVA aspirator***<br>*Question relevant when: not(selected(${q101_C46_full_list} , '6')) and not(selected(${q101_C46_full_list} , '99'))*	
q701_C46a *(required)*	q701_C46a. When purchased, how many units of this item typically come in one pack, box, bottle, etc.? (e.g. 100 tablets, 1000 ml's, etc.) (Write 1 if purchased as a single item.)<br>*Smallest unit is mililiter (ml)*<br>*Question relevant when: not(selected(${q101_C46_full_list} , '6')) and not(selected(${q101_C46_full_list} , '99'))*<br>*Response constrained to: .>=0*	
q701_C46b *(required)*	q701_C46b. What is the typical purchase price for that quanity of units (i.e. the quantity noted in the question above)?<br>*Question relevant when: not(selected(${q101_C46_full_list} , '6')) and not(selected(${q101_C46_full_list} , '99'))*<br>*Response constrained to: .>=0*	
q701_C46c *(required)*	q701_C46c. Please specify the currency for the purchase price.<br>*Question relevant when: not(selected(${q101_C46_full_list} , '6')) and not(selected(${q101_C46_full_list} , '99'))*	1 Tanzanian Shillings<br>2 US dollars<br>3 Euros<br>99 Don't know<br>5 Other
q701_C46c.1	q701_C46c.1 If other currency, specify:<br>*Leave blank if not applicable*<br>*Question relevant when: not(selected(${q101_C46_full_list} , '6')) and not(selected(${q101_C46_full_list} , '99'))*	
q701_C46d *(required)*	q701_C46d. Please specify the year of the purchase price.<br>*Question relevant when: not(selected(${q101_C46_full_list} , '6')) and not(selected(${q101_C46_full_list} , '99'))*<br>*Response constrained to: .>=2000 and .<=2019 or .=999*	
q701_C47	***q701_C47. Speculum, BValve/Cuscos (reusable)***<br>*Question relevant when: not(selected(${q101_C47_full_list} , '6')) and not(selected(${q101_C47_full_list} , '99'))*	
q701_C47a *(required)*	q701_C47a. When purchased, how many units of this item typically come in one pack, box, bottle, etc.? (e.g. 100 tablets, 1000 ml's, etc.) (Write 1 if purchased as a single item.)<br>*Smallest unit is one speculum.*<br>*Question relevant when: not(selected(${q101_C47_full_list} , '6')) and not(selected(${q101_C47_full_list} , '99'))*<br>*Response constrained to: .>=0*	
q701_C47b *(required)*	q701_C47b. What is the typical purchase price for that quanity of units (i.e. the quantity noted in the question above)?<br>*Question relevant when: not(selected(${q101_C47_full_list} , '6')) and not(selected(${q101_C47_full_list} , '99'))*<br>*Response constrained to: .>=0*	
q701_C47c *(required)*	q701_C47c. Please specify the currency for the purchase price.<br>*Question relevant when: not(selected(${q101_C47_full_list} , '6')) and not(selected(${q101_C47_full_list} , '99'))*	1 Tanzanian Shillings<br>2 US dollars<br>3 Euros<br>99 Don't know<br>5 Other
q701_C47c.1	q701_C47c.1 If other currency, specify:<br>*Leave blank if not applicable*<br>*Question relevant when: not(selected(${q101_C47_full_list} , '6')) and not(selected(${q101_C47_full_list} , '99'))*	
q701_C47d *(required)*	q701_C47d. Please specify the year of the purchase price.<br>*Question relevant when: not(selected(${q101_C47_full_list} , '6')) and not(selected(${q101_C47_full_list} , '99'))*<br>*Response constrained to: .>=2000 and .<=2019 or .=999*	
q701_C48	***q701_C48. Speculum, Sim's (disposable)***<br>*Question relevant when: not(selected(${q101_C48_full_list} , '6')) and not(selected(${q101_C48_full_list} , '99'))*	
q701_C48a *(required)*	q701_C48a. When purchased, how many units of this item typically come in one pack, box, bottle, etc.? (e.g. 100 tablets, 1000 ml's, etc.) (Write 1 if purchased as a single item.)<br>*Smallest unit is one speculum.*<br>*Question relevant when: not(selected(${q101_C48_full_list} , '6')) and not(selected(${q101_C48_full_list} , '99'))*<br>*Response constrained to: .>=0*	
q701_C48b *(required)*	q701_C48b. What is the typical purchase price for that quanity of units (i.e. the quantity noted in the question above)?<br>*Question relevant when: not(selected(${q101_C48_full_list} , '6')) and not(selected(${q101_C48_full_list} , '99'))*<br>*Response constrained to: .>=0*	
q701_C48c *(required)*	q701_C48c. Please specify the currency for the purchase price.<br>*Question relevant when: not(selected(${q101_C48_full_list} , '6')) and not(selected(${q101_C48_full_list} , '99'))*	1 Tanzanian Shillings<br>2 US dollars<br>3 Euros<br>99 Don't know<br>5 Other

Field	Question	Answer
q701_C48c.1	q701_C48c.1 If other currency, specify:<br>*Leave blank if not applicable*<br>*Question relevant when: not(selected(${q101_C48_full_list} , '6')) and not(selected(${q101_C48_full_list} , '99'))*	
q701_C48d *(required)*	q701_C48d. Please specify the year of the purchase price.<br>*Question relevant when: not(selected(${q101_C48_full_list} , '6')) and not(selected(${q101_C48_full_list} , '99'))*<br>*Response constrained to: .>=2000 and .<=2019 or .=999*	
A. Consumables - Cost (8)<br>*Group relevant when: selected(${section_seven_skip_con} , '1')*		
note_701_suggest_other_etc	***Other supplies - suggestions?***	

Field	Question	Answer
q701_C49	***q701_C49. "[q101_C49_full_list_other]"***<br>*Question relevant when: not(selected(${q101_C49_full_list} , '6')) and not(selected(${q101_C49_full_list} , '99')) and string-length(${q101_C49_full_list_other}) > 0*	
q701_C49a *(required)*	q701_C49a. When purchased, how many units of this item typically come in one pack, box, bottle, etc.? (e.g. 100 tablets, 1000 ml's, etc.) (Write 1 if purchased as a single item.)<br>*Question relevant when: not(selected(${q101_C49_full_list} , '6')) and not(selected(${q101_C49_full_list} , '99')) and string-length(${q101_C49_full_list_other}) > 0*<br>*Response constrained to: .>=0*	
q701_C49b *(required)*	q701_C49b. What is the typical purchase price for that quanity of units (i.e. the quantity noted in the question above)?<br>*Question relevant when: not(selected(${q101_C49_full_list} , '6')) and not(selected(${q101_C49_full_list} , '99')) and string-length(${q101_C49_full_list_other}) > 0*<br>*Response constrained to: .>=0*	
q701_C49c *(required)*	q701_C49c. Please specify the currency for the purchase price.<br>*Question relevant when: not(selected(${q101_C49_full_list} , '6')) and not(selected(${q101_C49_full_list} , '99')) and string-length(${q101_C49_full_list_other}) > 0*	1 Tanzanian Shillings<br>2 US dollars<br>3 Euros<br>99 Don't know<br>5 Other
q701_C49c.1	q701_C49c.1 If other currency, specify:<br>*Leave blank if not applicable*<br>*Question relevant when: not(selected(${q101_C49_full_list} , '6')) and not(selected(${q101_C49_full_list} , '99')) and string-length(${q101_C49_full_list_other}) > 0*	
q701_C49d *(required)*	q701_C49d. Please specify the year of the purchase price.<br>*Question relevant when: not(selected(${q101_C49_full_list} , '6')) and not(selected(${q101_C49_full_list} , '99')) and string-length(${q101_C49_full_list_other}) > 0*<br>*Response constrained to: .>=2000 and .<=2019 or .=999*	
q701_C50	***q701_C50. "[q101_C50_full_list_other]"***<br>*Question relevant when: not(selected(${q101_C49_full_list} , '6')) and not(selected(${q101_C49_full_list} , '99')) and string-length(${q101_C49_full_list_other}) > 0*	
q701_C50a *(required)*	q701_C50a. When purchased, how many units of this item typically come in one pack, box, bottle, etc.? (e.g. 100 tablets, 1000 ml's, etc.) (Write 1 if purchased as a single item.)<br>*Question relevant when: not(selected(${q101_C50_full_list} , '6')) and not(selected(${q101_C50_full_list} , '99')) and string-length(${q101_C50_full_list_other}) > 0*<br>*Response constrained to: .>=0*	
q701_C50b *(required)*	q701_C50b. What is the typical purchase price for that quanity of units (i.e. the quantity noted in the question above)?<br>*Question relevant when: not(selected(${q101_C50_full_list} , '6')) and not(selected(${q101_C50_full_list} , '99')) and string-length(${q101_C50_full_list_other}) > 0*<br>*Response constrained to: .>=0*	
q701_C50c *(required)*	q701_C50c. Please specify the currency for the purchase price.<br>*Question relevant when: not(selected(${q101_C50_full_list} , '6')) and not(selected(${q101_C50_full_list} , '99')) and string-length(${q101_C50_full_list_other}) > 0*	1 Tanzanian Shillings<br>2 US dollars<br>3 Euros<br>99 Don't know<br>5 Other
q701_C50c.1	q701_C50c.1 If other currency, specify:<br>*Leave blank if not applicable*<br>*Question relevant when: not(selected(${q101_C50_full_list} , '6')) and not(selected(${q101_C50_full_list} , '99')) and string-length(${q101_C50_full_list_other}) > 0*	
q701_C50d *(required)*	q701_C50d. Please specify the year of the purchase price.<br>*Question relevant when: not(selected(${q101_C50_full_list} , '6')) and not(selected(${q101_C50_full_list} , '99')) and string-length(${q101_C50_full_list_other}) > 0*<br>*Response constrained to: .>=2000 and .<=2019 or .=999*	
q701_C51	***q701_C51. "[q101_C51_full_list_other]"***<br>*Question relevant when: not(selected(${q101_C51_full_list} , '6')) and not(selected(${q101_C51_full_list} , '99')) and string-length(${q101_C51_full_list_other}) > 0*	
q701_C51a *(required)*	q701_C51a. When purchased, how many units of this item typically come in one pack, box, bottle, etc.? (e.g. 100 tablets	

Field	Question	Answer
q701_C51a *(required)*	q701_C51a. When purchased, how many units of this item typically come in one pack, box, bottle, etc.? (e.g. 100 tablets, 1000 ml's, etc.) (Write 1 if purchased as a single item.)<br>*Question relevant when: not(selected(${q101_C51_full_list} , '6')) and not(selected(${q101_C51_full_list} , '99')) and string-length(${q101_C51_full_list_other}) > 0*<br>*Response constrained to: .>=0*	
q701_C51b *(required)*	q701_C51b. What is the typical purchase price for that quanity of units (i.e. the quantity noted in the question above)?<br>*Question relevant when: not(selected(${q101_C51_full_list} , '6')) and not(selected(${q101_C51_full_list} , '99')) and string-length(${q101_C51_full_list_other}) > 0*<br>*Response constrained to: .>=0*	
q701_C51c *(required)*	q701_C51c. Please specify the currency for the purchase price.<br>*Question relevant when: not(selected(${q101_C51_full_list} , '6')) and not(selected(${q101_C51_full_list} , '99')) and string-length(${q101_C51_full_list_other}) > 0*	1 Tanzanian Shillings<br>2 US dollars<br>3 Euros<br>99 Don't know<br>5 Other
q701_C51c.1	q701_C51c.1 If other currency, specify:<br>*Leave blank if not applicable*<br>*Question relevant when: not(selected(${q101_C51_full_list} , '6')) and not(selected(${q101_C51_full_list} , '99')) and string-length(${q101_C51_full_list_other}) > 0*	
q701_C51d *(required)*	q701_C51d. Please specify the year of the purchase price.<br>*Question relevant when: not(selected(${q101_C51_full_list} , '6')) and not(selected(${q101_C51_full_list} , '99')) and string-length(${q101_C51_full_list_other}) > 0*<br>*Response constrained to: .>=2000 and .<=2019 or .=999*	
q701_C52	***q701_C52. "[q101_C52_full_list_other]"***<br>*Question relevant when: not(selected(${q101_C52_full_list} , '6')) and not(selected(${q101_C52_full_list} , '99')) and string-length(${q101_C52_full_list_other}) > 0*	
q701_C52a *(required)*	q701_C52a. When purchased, how many units of this item typically come in one pack, box, bottle, etc.? (e.g. 100 tablets, 1000 ml's, etc.) (Write 1 if purchased as a single item.)<br>*Question relevant when: not(selected(${q101_C52_full_list} , '6')) and not(selected(${q101_C52_full_list} , '99')) and string-length(${q101_C52_full_list_other}) > 0*<br>*Response constrained to: .>=0*	
q701_C52b *(required)*	q701_C52b. What is the typical purchase price for that quanity of units (i.e. the quantity noted in the question above)?<br>*Question relevant when: not(selected(${q101_C52_full_list} , '6')) and not(selected(${q101_C52_full_list} , '99')) and string-length(${q101_C52_full_list_other}) > 0*<br>*Response constrained to: .>=0*	
q701_C52c *(required)*	q701_C52c. Please specify the currency for the purchase price.<br>*Question relevant when: not(selected(${q101_C52_full_list} , '6')) and not(selected(${q101_C52_full_list} , '99')) and string-length(${q101_C52_full_list_other}) > 0*	1 Tanzanian Shillings<br>2 US dollars<br>3 Euros<br>99 Don't know<br>5 Other
q701_C52c.1	q701_C52c.1 If other currency, specify:<br>*Leave blank if not applicable*<br>*Question relevant when: not(selected(${q101_C52_full_list} , '6')) and not(selected(${q101_C52_full_list} , '99')) and string-length(${q101_C52_full_list_other}) > 0*	
q701_C52d *(required)*	q701_C52d. Please specify the year of the purchase price.<br>*Question relevant when: not(selected(${q101_C52_full_list} , '6')) and not(selected(${q101_C52_full_list} , '99')) and string-length(${q101_C52_full_list_other}) > 0*<br>*Response constrained to: .>=2000 and .<=2019 or .=999*	
q701_C53	***q701_C53. "[q101_C53_full_list_other]"***<br>*Question relevant when: not(selected(${q101_C53_full_list} , '6')) and not(selected(${q101_C53_full_list} , '99')) and string-length(${q101_C53_full_list_other}) > 0*	
q701_C53a *(required)*	q701_C53a. When purchased, how many units of this item typically come in one pack, box, bottle, etc.? (e.g. 100 tablets, 1000 ml's, etc.) (Write 1 if purchased as a single item.)<br>*Question relevant when: not(selected(${q101_C53_full_list} , '6')) and not(selected(${q101_C53_full_list} , '99')) and string-length(${q101_C53_full_list_other}) > 0*<br>*Response constrained to: .>=0*	
q701_C53b *(required)*	q701_C53b. What is the typical purchase price for that quanity of units (i.e. the quantity noted in the question above)?<br>*Question relevant when: not(selected(${q101_C53_full_list} , '6')) and not(selected(${q101_C53_full_list} , '99')) and string-length(${q101_C53_full_list_other}) > 0*<br>*Response constrained to: .>=0*	
q701_C53c *(required)*	q701_C53c. Please specify the currency for the purchase price.<br>*Question relevant when: not(selected(${q101_C53_full_list} , '6')) and not(selected(${q101_C53_full_list} , '99')) and string-length(${q101_C53_full_list_other}) > 0*	1 Tanzanian Shillings<br>2 US dollars<br>3 Euros<br>[illegible]

Field	Question	Answer
		99 Don't know<br>5 Other
q701_C53c.1	q701_C53c.1 If other currency, specify:<br>*Leave blank if not applicable*<br>*Question relevant when: not(selected(${q101_C53_full_list} , '6')) and not(selected(${q101_C53_full_list} , '99')) and string-length(${q101_C53_full_list_other}) > 0*	
q701_C53d *(required)*	q701_C53d. Please specify the year of the purchase price.<br>*Question relevant when: not(selected(${q101_C53_full_list} , '6')) and not(selected(${q101_C53_full_list} , '99')) and string-length(${q101_C53_full_list_other}) > 0*<br>*Response constrained to: .>=2000 and .<=2019 or .=999*	

GPS_QB2_con *(required)*	GPS location capture<br>*Press the button to capture the GPS location at this point in the survey.*	
q705_time_end_qb_con *(required)*	ENTER THE END TIME OF THE INTERVIEW<br>*NB: The default is the current time.*	
thank_you	**END OF INTERVIEW QUESTIONS.**<br><br>**THANK THE PARTICIPANT FOR THEIR TIME.**<br><br>**THEN COMPLETE THE FOLLOWING TWO ITEMS.**	
q706_interviewer_comments_con	INTERVIEW COMMENTS - ENTER ANY RELEVANT NOTES AT THE END OF THE INTERVIEW. PLEASE ALSO EXPLAIN HOW COSTS WERE COLLECTED, I.E. VIA INTERVIEW, SITE REPRESENTATIVE FILLING OUT FORM, OR EXTRACTION FROM RECORDS.	
End_note	**End of Questionnaire B part 1**<br><br>ON THE NEXT PAGE YOU'LL FIND THIS FACILITY'S ID NUMBER. TAKE NOTE OF THE NUMBER, AND THEN, ON THE FOLLOWING PAGE, NAME THIS FORM BY ADDING THE FACILITY ID BEFORE THE FORM NAME.<br><br>FOR EXAMPLE, "20 Tanzania PAC cost study – Quest. B part 1 consumables".<br><br>IF THIS INTERVIEW IS COMPLETE - I.E. THE RESPONDENT HAS ANSWERED ALL OF THE QUESTIONS THAT THEY CAN ON THIS FORM, THEN LEAVE THE DEFAULT BOX CHECKED. IF THE FORM IS NOT FINAL FOR THIS FACILITY, UNTICK THE BOX.<br><br>THEN SAVE AND EXIT.	
facility_id_con2	The ID for this facility is '[facility_id_con]".	
