# Supplementary material for: The health system costs of post abortion care in Tanzania
Source: BMC Health Serv Res. 2021 Jul 22;21:720. doi: 10.1186/s12913-021-06688-7 (PMC8296742; doi:10.1186/s12913-021-06688-7)
Supplement: Supplementary file 1 — Additional file 1. [file 12913_2021_6688_MOESM1_ESM.zip › QB-part 2 equipment 2019.01.28 FINALR3.pdf]

Tanzania PAC cost study – Quest. B part 2 equipment

Field	Question	Answer
about_survey	**Tanzania PAC cost study – Quest. B part 2 equipment**<br><br>INTERVIEWER INSTRUCTIONS:<br>• DO NOT READ TEXT IN ALL CAPS OR HINTS IN *ITALICS* ALOUD TO THE PARTICIPANT.<br>• BEFORE STARTING THE INTERVIEW, CHECK WHICH PARTS OR SECTIONS OF QUESTIONNAIRE B HAVE NOT BEEN COMPLETED.<br>• MAKE SURE THE PERSON YOU ARE ABOUT TO INTERVIEW HAS PROVIDED CONSENT.	
introduction	QUESTIONNAIRE INTRODUCTION: We're now ready to complete part 2 of the interview. In this part of the interview we'll focus on equipment used at your facility.<br><br>As with the other interview parts, I'll first present a list of items and ask which items are used for the five types of postabortion complications. Those are: uncomplicated incomplete abortion, sepsis, shock, cervical and vaginal lacerations, and vaginal and uterine perforations. Then, if you tell me that an item is used at your facility, I'll separately ask how it is used (e.g. what proportion of women get it, how much they get, etc.).<br><br>At the end of interview, for any item that is used, I'll ask about prices for buying the items. If you don't have that information, I can obtain it from someone else at your facility after the interview.<br><br>Before we begin, I'm going to capture a some information about where we are and document that I'm doing the interview with you. Remember that your name won't be used with the results later.	
COVER PAGE		
q1_interviewer_name_equ *(required)*	q1. NAME OF INTERVIEWER	
q2_facility_name_equ *(required)*	q2. SELECT: NAME OF FACILITY	facilityid | facility_name
q3_GPS_QB_equ *(required)*	q3. CAPTURE THE GPS LOCATION<br>*Press the button to capture the GPS location at this point in the survey.*	
RESPONDENTS (1-5)		
cover_page_note_1_equ	INTERVIEWER INSTRUCTIONS: INDICATE NAMES, JOB TITLES AND CONTACT INFORMATION FOR UP TO 5 RESPONDENTS OF QUESTIONNAIRE A.<br>*NB: Information for only the first respondent is required in order to proceed to the next page. Ask for the information as a question if not already known prior to the interview.*	
group_respondent1_equ	RESPONDENT 1	
q4a_name_equ *(required)*	q4a. Name of 1st respondent<br>*First and last name*	
q4b_date_equ *(required)*	q4b. Date of interview with 1st respondent<br>*Default is today's date.*	
q4c_title_equ *(required)*	q4c. Designation/title of 1st respondent<br>*Job title and designation (e.g. Medical officer in charge)*	
q4d_phone_equ	q4d. Phone number of 1st respondent<br>*NB: Not required, but you might need this to follow up with questions.*	
group_respondent2_equ	RESPONDENT 2	
q5a_name_equ	q5a. Name of 2nd respondent<br>*First and last name*	
q5b_date_equ	q5b. Date of interview with 2nd respondent<br>*Default is today's date.*	
q5c_title_equ	q5c. Designation/title of 2nd respondent<br>*Job title and designation (e.g. Medical officer in charge)*	
q5d_phone_equ	q5d. Phone number of 2nd respondent<br>*NB: Not required, but you might need this to follow up with questions.*	
group_respondent3_equ	RESPONDENT 3	
q6a_name_equ	q6a. Name of 3rd respondent<br>*First and last name*	
q6b_date_equ	q6b. Date of interview with 3rd respondent<br>*Default is today's date.*	
q6c_title_equ	q6c. Designation/title of 3rd respondent<br>*Job title and designation (e.g. Medical officer in charge)*	
q6d_phone_equ	q6d. Phone number of 3rd respondent<br>*NB: Not required, but you might need this to follow up with questions.*	
group_respondent4_equ	RESPONDENT 4	
q8a_name_equ	q8a. Name of 4th respondent<br>*First and last name*	
q8b_date_equ	q8b. Date of interview with 4th respondent<br>*Default is today's date.*	

Field	Question	Answer
q8c_title_equ	q8c. Designation/title of 4th respondent<br>*Job title and designation (e.g. Medical officer in charge)*	
q8d_phone_equ	q8d. Phone number of 4th respondent<br>*NB: Not required, but you might need this to follow up with questions.*	
group_respondent5_equ	RESPONDENT 5	
q9a_name_equ	q9a. Name of 5th respondent<br>*First and last name*	
q9b_date_equ	q9b. Date of interview with 5th respondent<br>*Default is today's date.*	
q9c_title_equ	q9c. Designation/title of 5th respondent<br>*Job title and designation (e.g. Medical officer in charge)*	
q9d_phone_equ	q9d. Phone number of 5th respondent<br>*NB: Not required, but you might need this to follow up with questions.*	
q10_interviewer_comments_equ	q10. INTERVIEWER COMMENTS<br>*Enter any relevant notes prior to the interview.*	
q11_time_start_qa *(required)*	q11. ENTER START TIME OF INTERVIEW<br>*NB: The default is the current time.*	
introduction_equ	QUESTIONNAIRE INTRODUCTION: Ok now we can begin the interview. Remember that all the questions in this part of the interview are about equipment that is used to provide PAC at your facility.	
group_section_one_intro		
section1_start	**SECTION I. FULL LISTING OF ITEMS BY COMPLICATION TYPE**	
section_one_skip_equ	INTERVIEWER: WOULD YOU LIKE TO COMPLETE THIS SECTION NOW OR SKIP THIS SECTION AND RETURN TO IT LATER?<br>*You may need to skip if the participant has indicated that s/he cannot answer the questions in this section.*	1 Do not skip, complete this section now.<br>2 Skip and come back to this section later.
B. Small equipment - Full list (1)<br>*Group relevant when: selected(${section_one_skip_equ} , '1')*		
q102_equip_note1	102 For each of the following reusable, small equipment items, can you tell me if the item is used for post abortion care at your facility? I'm going to ask about each of the five complication types separately. Is [ITEM] used for managing women with [COMPLICATION TYPE]?	
note_102_evac	***Evacuation***	
q102_E1_full_list *(required)*	q102_E1. MVA aspirator<br>*Select all that apply.*<br>*Response constrained to: if(selected(., 6) or selected(., 99), count-selected(.) = 1, count-selected(.) >= 1)*	1 Incomplete abortion<br>2 Sepsis<br>3 Shock<br>4 Cervical/vaginal lacerations<br>5 Vaginal/uterine perforation<br>6 Not used at this facility<br>99 Don't know
q102_E2_full_list *(required)*	q102_E2. Cannula set<br>*Select all that apply.*<br>*Response constrained to: if(selected(., 6) or selected(., 99), count-selected(.) = 1, count-selected(.) >= 1)*	1 Incomplete abortion<br>2 Sepsis<br>3 Shock<br>4 Cervical/vaginal lacerations<br>5 Vaginal/uterine perforation<br>6 Not used at this facility<br>99 Don't know
q102_E3_full_list *(required)*	q102_E3. Uterine curette set - plastic<br>*Select all that apply.*<br>*Response constrained to: if(selected(., 6) or selected(., 99), count-selected(.) = 1, count-selected(.) >= 1)*	1 Incomplete abortion<br>2 Sepsis<br>3 Shock<br>4 Cervical/vaginal lacerations<br>5 Vaginal/uterine perforation<br>6 Not used at this facility<br>99 Don't know
q102_E4_full_list *(required)*	q102_E4. Uterine currette set - metal<br>*Select all that apply.*<br>*Response constrained to: if(selected(., 6) or selected(., 99), count-selected(.) = 1, count-selected(.) >= 1)*	1 Incomplete abortion<br>2 Sepsis<br>3 Shock<br>4 Cervical/vaginal lacerations<br>5 Vaginal/uterine perforation<br>6 Not used at this facility<br>99 Don't know

Field	Question	Answer
q102_E5_full_list *(required)*	q102_E5. Dilators (Hegar's Cervical dilators)<br>*Select all that apply.*<br>*Response constrained to: if(selected(., 6) or selected(., 99), count-selected(.) = 1, count-selected(.) >= 1)*	1 Incomplete abortion<br>2 Sepsis<br>3 Shock<br>4 Cervical/vaginal lacerations<br>5 Vaginal/uterine perforation<br>6 Not used at this facility<br>99 Don't know
B. Small equipment - Full list (2)<br>*Group relevant when: selected(${section_one_skip_equ} , '1')*		
q102_equip_note2	102 CONTINUED. For each of the following reusable, small equipment items, can you tell me if the item is used for post abortion care at your facility? I'm going to ask about each of the five complication types separately. Is [ITEM] used for managing women with [COMPLICATION TYPE]?	
note_102_forceps	***Forceps, speculums, dilators, etc.***	
q102_E6_full_list *(required)*	q102_E6. Forceps, artery/Kocher<br>*Select all that apply.*<br>*Response constrained to: if(selected(., 6) or selected(., 99), count-selected(.) = 1, count-selected(.) >= 1)*	1 Incomplete abortion<br>2 Sepsis<br>3 Shock<br>4 Cervical/vaginal lacerations<br>5 Vaginal/uterine perforation<br>6 Not used at this facility<br>99 Don't know
q102_E7_full_list *(required)*	q102_E7. Needle holder<br>*Select all that apply.*<br>*Response constrained to: if(selected(., 6) or selected(., 99), count-selected(.) = 1, count-selected(.) >= 1)*	1 Incomplete abortion<br>2 Sepsis<br>3 Shock<br>4 Cervical/vaginal lacerations<br>5 Vaginal/uterine perforation<br>6 Not used at this facility<br>99 Don't know
q102_E8_full_list *(required)*	q102_E8. Ovum forceps<br>*Select all that apply.*<br>*Response constrained to: if(selected(., 6) or selected(., 99), count-selected(.) = 1, count-selected(.) >= 1)*	1 Incomplete abortion<br>2 Sepsis<br>3 Shock<br>4 Cervical/vaginal lacerations<br>5 Vaginal/uterine perforation<br>6 Not used at this facility<br>99 Don't know
q102_E9_full_list *(required)*	q102_E9. Ring forceps<br>*Select all that apply.*<br>*Response constrained to: if(selected(., 6) or selected(., 99), count-selected(.) = 1, count-selected(.) >= 1)*	1 Incomplete abortion<br>2 Sepsis<br>3 Shock<br>4 Cervical/vaginal lacerations<br>5 Vaginal/uterine perforation<br>6 Not used at this facility<br>99 Don't know
q102_E10_full_list *(required)*	q102_E10. Speculum, Bivalve/Cuscos (reusable)<br>*Select all that apply.*<br>*Response constrained to: if(selected(., 6) or selected(., 99), count-selected(.) = 1, count-selected(.) >= 1)*	1 Incomplete abortion<br>2 Sepsis<br>3 Shock<br>4 Cervical/vaginal lacerations<br>5 Vaginal/uterine perforation<br>6 Not used at this facility<br>99 Don't know
q102_E11_full_list *(required)*	q102_E11. Speculum, Sim's (reusable)<br>*Select all that apply.*<br>*Response constrained to: if(selected(., 6) or selected(., 99), count-selected(.) = 1, count-selected(.) >= 1)*	1 Incomplete abortion<br>2 Sepsis<br>3 Shock<br>4 Cervical/vaginal lacerations<br>5 Vaginal/uterine perforation<br>6 Not used at this facility<br>99 Don't know

Field	Question	Answer
q102_E12_full_list *(required)*	q102_E12. Tenaculum<br>*Select all that apply.*<br>*Response constrained to: if(selected(., 6) or selected(., 99), count-selected(.) = 1, count-selected(.) >= 1)*	1 Incomplete abortion<br>2 Sepsis<br>3 Shock<br>4 Cervical/vaginal lacerations<br>5 Vaginal/uterine perforation<br>6 Not used at this facility<br>99 Don't know
B. Small equipment - Full list (3)<br>*Group relevant when: selected(${section_one_skip_equ} , '1')*		
q102_equip_note3	102 CONTINUED. For each of the following reusable, small equipment items, can you tell me if the item is used for post abortion care at your facility? I'm going to ask about each of the five complication types separately. Is [ITEM] used for managing women with [COMPLICATION TYPE]?	
note_102_linens	***Linens***	
q102_E13_full_list *(required)*	q102_E13. Towel<br>*Select all that apply.*<br>*Response constrained to: if(selected(., 6) or selected(., 99), count-selected(.) = 1, count-selected(.) >= 1)*	1 Incomplete abortion<br>2 Sepsis<br>3 Shock<br>4 Cervical/vaginal lacerations<br>5 Vaginal/uterine perforation<br>6 Not used at this facility<br>99 Don't know
q102_E14_full_list *(required)*	q102_E14. Sheet<br>*Select all that apply.*<br>*Response constrained to: if(selected(., 6) or selected(., 99), count-selected(.) = 1, count-selected(.) >= 1)*	1 Incomplete abortion<br>2 Sepsis<br>3 Shock<br>4 Cervical/vaginal lacerations<br>5 Vaginal/uterine perforation<br>6 Not used at this facility<br>99 Don't know
q102_E15_full_list *(required)*	q102_E15. Blanket<br>*Select all that apply.*<br>*Response constrained to: if(selected(., 6) or selected(., 99), count-selected(.) = 1, count-selected(.) >= 1)*	1 Incomplete abortion<br>2 Sepsis<br>3 Shock<br>4 Cervical/vaginal lacerations<br>5 Vaginal/uterine perforation<br>6 Not used at this facility<br>99 Don't know
q102_E16_full_list *(required)*	q102_E16. Pillow<br>*Select all that apply.*<br>*Response constrained to: if(selected(., 6) or selected(., 99), count-selected(.) = 1, count-selected(.) >= 1)*	1 Incomplete abortion<br>2 Sepsis<br>3 Shock<br>4 Cervical/vaginal lacerations<br>5 Vaginal/uterine perforation<br>6 Not used at this facility<br>99 Don't know
B. Small equipment - Full list (4)<br>*Group relevant when: selected(${section_one_skip_equ} , '1')*		
q102_equip_note4	102 CONTINUED. For each of the following reusable, small equipment items, can you tell me if the item is used for post abortion care at your facility? I'm going to ask about each of the five complication types separately. Is [ITEM] used for managing women with [COMPLICATION TYPE]?	
note_102_protective	***Protective supplies***	
q102_E17_full_list *(required)*	q102_E17. Apron (reusable)<br>*Select all that apply.*<br>*Response constrained to: if(selected(., 6) or selected(., 99), count-selected(.) = 1, count-selected(.) >= 1)*	1 Incomplete abortion<br>2 Sepsis<br>3 Shock<br>4 Cervical/vaginal lacerations<br>5 Vaginal/uterine perforation<br>6 Not used at this facility<br>99 Don't know
q102_E18_full_list *(required)*	q102_E18. Gown (reusable)<br>*Select all that apply.*<br>*Response constrained to: if(selected(., 6) or selected(., 99), count-selected(.) = 1, count-selected(.) >= 1)*	1 Incomplete abortion<br>2 Sepsis<br>3 Shock<br>4 Cervical/vaginal lacerations<br>5 Vaginal/uterine perforation<br>6 Not used at this facility<br>99 Don't know

Field	Question	Answer
q102_E19_full_list *(required)*	q102_E19. Heavy duty gloves<br>*Select all that apply.*<br>*Response constrained to: if(selected(., 6) or selected(., 99), count-selected(.) = 1, count-selected(.) >= 1)*	1 Incomplete abortion<br>2 Sepsis<br>3 Shock<br>4 Cervical/vaginal lacerations<br>5 Vaginal/uterine perforation<br>6 Not used at this facility<br>99 Don't know
q102_E20_full_list *(required)*	q102_E20. Mask (reusable)<br>*Select all that apply.*<br>*Response constrained to: if(selected(., 6) or selected(., 99), count-selected(.) = 1, count-selected(.) >= 1)*	1 Incomplete abortion<br>2 Sepsis<br>3 Shock<br>4 Cervical/vaginal lacerations<br>5 Vaginal/uterine perforation<br>6 Not used at this facility<br>99 Don't know
q102_E21_full_list *(required)*	q102_E21. Protective glasses<br>*Select all that apply.*<br>*Response constrained to: if(selected(., 6) or selected(., 99), count-selected(.) = 1, count-selected(.) >= 1)*	1 Incomplete abortion<br>2 Sepsis<br>3 Shock<br>4 Cervical/vaginal lacerations<br>5 Vaginal/uterine perforation<br>6 Not used at this facility<br>99 Don't know
B. Small equipment - Full list (5)<br>*Group relevant when: selected(${section_one_skip_equ} , '1')*		
q102_equip_note5	102 CONTINUED. For each of the following reusable, small equipment items, can you tell me if the item is used for post abortion care at your facility? I'm going to ask about each of the five complication types separately. Is [ITEM] used for managing women with [COMPLICATION TYPE]?	
note_102_receivers	***Receivers***	
q102_E22_full_list *(required)*	q102_E22. Kidney dish - small<br>*Select all that apply.*<br>*Response constrained to: if(selected(., 6) or selected(., 99), count-selected(.) = 1, count-selected(.) >= 1)*	1 Incomplete abortion<br>2 Sepsis<br>3 Shock<br>4 Cervical/vaginal lacerations<br>5 Vaginal/uterine perforation<br>6 Not used at this facility<br>99 Don't know
q102_E23_full_list *(required)*	q102_E23. Kidney dish - medium<br>*Select all that apply.*<br>*Response constrained to: if(selected(., 6) or selected(., 99), count-selected(.) = 1, count-selected(.) >= 1)*	1 Incomplete abortion<br>2 Sepsis<br>3 Shock<br>4 Cervical/vaginal lacerations<br>5 Vaginal/uterine perforation<br>6 Not used at this facility<br>99 Don't know
q102_E24_full_list *(required)*	q102_E24. Kidney dish - large<br>*Select all that apply.*<br>*Response constrained to: if(selected(., 6) or selected(., 99), count-selected(.) = 1, count-selected(.) >= 1)*	1 Incomplete abortion<br>2 Sepsis<br>3 Shock<br>4 Cervical/vaginal lacerations<br>5 Vaginal/uterine perforation<br>6 Not used at this facility<br>99 Don't know
q102_E25_full_list *(required)*	q102_E25. Receiving bowl - small<br>*Select all that apply.*<br>*Response constrained to: if(selected(., 6) or selected(., 99), count-selected(.) = 1, count-selected(.) >= 1)*	1 Incomplete abortion<br>2 Sepsis<br>3 Shock<br>4 Cervical/vaginal lacerations<br>5 Vaginal/uterine perforation<br>6 Not used at this facility<br>99 Don't know

Field	Question	Answer
q102_E26_full_list *(required)*	q102_E26. Receiving bowl - medium<br>*Select all that apply.*<br>*Response constrained to: if(selected(., 6) or selected(., 99), count-selected(.) = 1, count-selected(.) >= 1)*	1 Incomplete abortion<br>2 Sepsis<br>3 Shock<br>4 Cervical/vaginal lacerations<br>5 Vaginal/uterine perforation<br>6 Not used at this facility<br>99 Don't know
q102_E27_full_list *(required)*	q102_E27. Receiving bowl - large<br>*Select all that apply.*<br>*Response constrained to: if(selected(., 6) or selected(., 99), count-selected(.) = 1, count-selected(.) >= 1)*	1 Incomplete abortion<br>2 Sepsis<br>3 Shock<br>4 Cervical/vaginal lacerations<br>5 Vaginal/uterine perforation<br>6 Not used at this facility<br>99 Don't know
B. Small equipment - Full list (6)<br>*Group relevant when: selected(${section_one_skip_equ} , '1')*		
q102_equip_note6	102 CONTINUED. For each of the following reusable, small equipment items, can you tell me if the item is used for post abortion care at your facility? I'm going to ask about each of the five complication types separately. Is [ITEM] used for managing women with [COMPLICATION TYPE]?	
note_102_surgery	***Surgery***	
q102_E28_full_list *(required)*	q102_E28. Scalpel, reusable<br>*Select all that apply.*<br>*Response constrained to: if(selected(., 6) or selected(., 99), count-selected(.) = 1, count-selected(.) >= 1)*	1 Incomplete abortion<br>2 Sepsis<br>3 Shock<br>4 Cervical/vaginal lacerations<br>5 Vaginal/uterine perforation<br>6 Not used at this facility<br>99 Don't know
q102_E29_full_list *(required)*	q102_E29. Surgical scissors<br>*Select all that apply.*<br>*Response constrained to: if(selected(., 6) or selected(., 99), count-selected(.) = 1, count-selected(.) >= 1)*	1 Incomplete abortion<br>2 Sepsis<br>3 Shock<br>4 Cervical/vaginal lacerations<br>5 Vaginal/uterine perforation<br>6 Not used at this facility<br>99 Don't know
B. Small equipment - Full list (7)<br>*Group relevant when: selected(${section_one_skip_equ} , '1')*		
q102_equip_note7	102 CONTINUED. For each of the following reusable, small equipment items, can you tell me if the item is used for post abortion care at your facility? I'm going to ask about each of the five complication types separately. Is [ITEM] used for managing women with [COMPLICATION TYPE]?	
note_102_other	***Other small equipment***	
q102_E30_full_list *(required)*	q102_E30. Head lamp/batteries<br>*Select all that apply.*<br>*Response constrained to: if(selected(., 6) or selected(., 99), count-selected(.) = 1, count-selected(.) >= 1)*	1 Incomplete abortion<br>2 Sepsis<br>3 Shock<br>4 Cervical/vaginal lacerations<br>5 Vaginal/uterine perforation<br>6 Not used at this facility<br>99 Don't know
q102_E31_full_list *(required)*	q102_E31. Metal catheter<br>*Select all that apply.*<br>*Response constrained to: if(selected(., 6) or selected(., 99), count-selected(.) = 1, count-selected(.) >= 1)*	1 Incomplete abortion<br>2 Sepsis<br>3 Shock<br>4 Cervical/vaginal lacerations<br>5 Vaginal/uterine perforation<br>6 Not used at this facility<br>99 Don't know
q102_E32_full_list *(required)*	q102_E32. Urine collection flask<br>*Select all that apply.*<br>*Response constrained to: if(selected(., 6) or selected(., 99), count-selected(.) = 1, count-selected(.) >= 1)*	1 Incomplete abortion<br>2 Sepsis<br>3 Shock<br>4 Cervical/vaginal lacerations<br>5 Vaginal/uterine perforation<br>6 Not used at this facility<br>99 Don't know

Field	Question	Answer
q102_equipment_other *(required)*	q102_Equip_Other. Are there any small equipment (i.e. resuable) items that we have not mentioned that are used for postabortion care at your facility?<br>*Question relevant when: selected(${section_one_skip_equ} , '1')*	1 Yes<br>0 No<br>99 Don't know
B. Small equipment - Full list - specify (8)<br>*Group relevant when: selected(${q102_equipment_other} , '1') and selected(${section_one_skip_equ} , '1')*		
note_102_other_suggestion	Please list the "other" equipment items here.	
q102_E33_full_list_other	q102_E33. Other equipment 1. Please specify:	
q102_E34_full_list_other	q102_E34. Other equipment 2. Please specify:	
q102_E35_full_list_other	q102_E35. Other equipment 3. Please specify:	
q102_E36_full_list_other	q102_E36. Other equipment 3. Please specify:	
q102_E37_full_list_other	q102_E37. Other equipment 5. Please specify:	
B. Small equipment - Full list - usage of other items (8b)<br>*Group relevant when: selected(${q102_equipment_other} , '1') and selected(${section_one_skip_equ} , '1')*		
note_102_other_suggestion_b	For each "other" item that you listed, please tell me which of the five post abortion complication types it is used for.	
q102_E33_full_list *(required)*	q102_E33. Other equipment 1: "[q102_E33_full_list_other]"<br>*Select all that apply.*<br>*Question relevant when: string-length(${q102_E33_full_list_other}) > 0 and selected(${section_one_skip_equ} , '1')*<br>*Response constrained to: if(selected(., 6) or selected(., 99), count-selected(.) = 1, count-selected(.) >= 1)*	1 Incomplete abortion<br>2 Sepsis<br>3 Shock<br>4 Cervical/vaginal lacerations<br>5 Vaginal/uterine perforation<br>6 Not used at this facility<br>99 Don't know
q102_E34_full_list *(required)*	q102_E34. Other equipment 2: "[q102_E34_full_list_other]"<br>*Select all that apply.*<br>*Question relevant when: string-length(${q102_E34_full_list_other}) > 0 and selected(${section_one_skip_equ} , '1')*<br>*Response constrained to: if(selected(., 6) or selected(., 99), count-selected(.) = 1, count-selected(.) >= 1)*	1 Incomplete abortion<br>2 Sepsis<br>3 Shock<br>4 Cervical/vaginal lacerations<br>5 Vaginal/uterine perforation<br>6 Not used at this facility<br>99 Don't know
q102_E35_full_list *(required)*	q102_E35. Other equipment 3: "[q102_E35_full_list_other]"<br>*Select all that apply.*<br>*Question relevant when: string-length(${q102_E35_full_list_other}) > 0 and selected(${section_one_skip_equ} , '1')*<br>*Response constrained to: if(selected(., 6) or selected(., 99), count-selected(.) = 1, count-selected(.) >= 1)*	1 Incomplete abortion<br>2 Sepsis<br>3 Shock<br>4 Cervical/vaginal lacerations<br>5 Vaginal/uterine perforation<br>6 Not used at this facility<br>99 Don't know
q102_E36_full_list *(required)*	q102_E36. Other equipment 4: "[q102_E36_full_list_other]"<br>*Select all that apply.*<br>*Question relevant when: string-length(${q102_E36_full_list_other}) > 0 and selected(${section_one_skip_equ} , '1')*<br>*Response constrained to: if(selected(., 6) or selected(., 99), count-selected(.) = 1, count-selected(.) >= 1)*	1 Incomplete abortion<br>2 Sepsis<br>3 Shock<br>4 Cervical/vaginal lacerations<br>5 Vaginal/uterine perforation<br>6 Not used at this facility<br>99 Don't know
q102_E37_full_list *(required)*	q102_E37. Other equipment 5: "[q102_E37_full_list_other]"<br>*Select all that apply.*<br>*Question relevant when: string-length(${q102_E37_full_list_other}) > 0 and selected(${section_one_skip_equ} , '1')*<br>*Response constrained to: if(selected(., 6) or selected(., 99), count-selected(.) = 1, count-selected(.) >= 1)*	1 Incomplete abortion<br>2 Sepsis<br>3 Shock<br>4 Cervical/vaginal lacerations<br>5 Vaginal/uterine perforation<br>6 Not used at this facility<br>99 Don't know
group_section_two_intro		
section2_start	**SECTION II. INCOMPLETE ABORTION - USAGE OF ALL ITEMS**	
section_two_skip_equ	INTERVIEWER: WOULD YOU LIKE TO COMPLETE THIS SECTION NOW OR SKIP THIS SECTION AND RETURN TO IT LATER?<br>*You may need to skip if the participant has indicated that s/he cannot answer the questions in this section.*	1 Do not skip, complete this section now.<br>2 Skip and come back to this section later.
group_section_two_introB<br>*Group relevant when: selected(${section_two_skip_equ} , '1')*		
section2_start2	In this section of the interview, we will review all of the items that you said are used for management of "uncomplicated" incomplete abortion. For each item that is used, I'm going to ask questions on how many women need it and how much of it is used.	

Field	Question	Answer
section2_start3	INTERVIEWER: ENTER WHOLE NUMBERS OR DECIMALS. DO NOT TYPE PERCENT SIGNS. ENTER 999 FOR ANY THAT ARE UNKNOWN.	
B. Small equipment - Incomplete Abortion (1)<br>*Group relevant when: selected(${section_two_skip_equ} , '1')*		
note_202_evac	***Evacuation***	
q202_E1	q202_E1. MVA aspirator<br>*Question relevant when: selected(${q102_E1_full_list} , '1')*	
q202_E1a *(required)*	q202_E1a. What percent of patients require this item?<br>*Question relevant when: selected(${q102_E1_full_list} , '1')*<br>*Response constrained to: .>0 and .<=100 or .=999*	
q202_E1b *(required)*	q202_E1b. How many units of this item are required per patient (throughout the entire treatment provided for complication)?<br>*Smallest unit is one aspirator.*<br>*Question relevant when: selected(${q102_E1_full_list} , '1')*<br>*Response constrained to: .>=1*	
q202_E2	q202_E2. Cannula set<br>*Question relevant when: selected(${q102_E2_full_list} , '1')*	
q202_E2a *(required)*	q202_E2a. What percent of patients require this item?<br>*Question relevant when: selected(${q102_E2_full_list} , '1')*<br>*Response constrained to: .>0 and .<=100 or .=999*	
q202_E2b *(required)*	q202_E2b. How many units of this item are required per patient (throughout the entire treatment provided for complication)?<br>*Smallest unit is one set.*<br>*Question relevant when: selected(${q102_E2_full_list} , '1')*<br>*Response constrained to: .>=1*	
q202_E3	q202_E3. Uterine curette set - plastic<br>*Question relevant when: selected(${q102_E3_full_list} , '1')*	
q202_E3a *(required)*	q202_E3a. What percent of patients require this item?<br>*Question relevant when: selected(${q102_E3_full_list} , '1')*<br>*Response constrained to: .>0 and .<=100 or .=999*	
q202_E3b *(required)*	q202_E3b. How many units of this item are required per patient (throughout the entire treatment provided for complication)?<br>*Smallest unit is one set.*<br>*Question relevant when: selected(${q102_E3_full_list} , '1')*<br>*Response constrained to: .>=1*	
q202_E4	q202_E4. Uterine currette set - metal<br>*Question relevant when: selected(${q102_E4_full_list} , '1')*	
q202_E4a *(required)*	q202_E4a. What percent of patients require this item?<br>*Question relevant when: selected(${q102_E4_full_list} , '1')*<br>*Response constrained to: .>0 and .<=100 or .=999*	
q202_E4b *(required)*	q202_E4b. How many units of this item are required per patient (throughout the entire treatment provided for complication)?<br>*Smallest unit is one set.*<br>*Question relevant when: selected(${q102_E4_full_list} , '1')*<br>*Response constrained to: .>=1*	
q202_E5	q202_E5. Dilators (Hegar's Cervical dilators)<br>*Question relevant when: selected(${q102_E5_full_list} , '1')*	
q202_E5a *(required)*	q202_E5a. What percent of patients require this item?<br>*Question relevant when: selected(${q102_E5_full_list} , '1')*<br>*Response constrained to: .>0 and .<=100 or .=999*	
q202_E5b *(required)*	q202_E5b. How many units of this item are required per patient (throughout the entire treatment provided for complication)?<br>*Smallest unit is one set.*<br>*Question relevant when: selected(${q102_E5_full_list} , '1')*<br>*Response constrained to: .>=1*	
B. Small equipment - Incomplete Abortion (2)<br>*Group relevant when: selected(${section_two_skip_equ} , '1')*		
note_202_forceps	***Forceps, speculums, dilators, etc.***	
q202_E6	q202_E6. Forceps, artery/Kocher<br>*Question relevant when: selected(${q102_E6_full_list} , '1')*	
q202_E6a *(required)*	q202_E6a. What percent of patients require this item?<br>*Question relevant when: selected(${q102_E6_full_list} , '1')*<br>*Response constrained to: .>0 and .<=100 or .=999*	

Field	Question	Answer
q202_E6b *(required)*	q202_E6b. How many units of this item are required per patient (throughout the entire treatment provided for complication)?<br>*Smallest unit is one forceps.*<br>*Question relevant when: selected(${q102_E6_full_list} , '1')*<br>*Response constrained to: .>=1*	
q202_E7	q202_E7. Needle holder<br>*Question relevant when: selected(${q102_E7_full_list} , '1')*	
q202_E7a *(required)*	q202_E7a. What percent of patients require this item?<br>*Question relevant when: selected(${q102_E7_full_list} , '1')*<br>*Response constrained to: .>0 and .<=100 or .=999*	
q202_E7b *(required)*	q202_E7b. How many units of this item are required per patient (throughout the entire treatment provided for complication)?<br>*Smallest unit is one holder.*<br>*Question relevant when: selected(${q102_E7_full_list} , '1')*<br>*Response constrained to: .>=1*	
q202_E8	q202_E8. Ovum forceps<br>*Question relevant when: selected(${q102_E8_full_list} , '1')*	
q202_E8a *(required)*	q202_E8a. What percent of patients require this item?<br>*Question relevant when: selected(${q102_E8_full_list} , '1')*<br>*Response constrained to: .>0 and .<=100 or .=999*	
q202_E8b *(required)*	q202_E8b. How many units of this item are required per patient (throughout the entire treatment provided for complication)?<br>*Smallest unit is one forceps.*<br>*Question relevant when: selected(${q102_E8_full_list} , '1')*<br>*Response constrained to: .>=1*	
q202_E9	q202_E9. Ring forceps<br>*Question relevant when: selected(${q102_E9_full_list} , '1')*	
q202_E9a *(required)*	q202_E9a. What percent of patients require this item?<br>*Question relevant when: selected(${q102_E9_full_list} , '1')*<br>*Response constrained to: .>0 and .<=100 or .=999*	
q202_E9b *(required)*	q202_E9b. How many units of this item are required per patient (throughout the entire treatment provided for complication)?<br>*Smallest unit is one forceps.*<br>*Question relevant when: selected(${q102_E9_full_list} , '1')*<br>*Response constrained to: .>=1*	
q202_E10	q202_E10. Speculum, Bivalve/Cuscos (reusable)<br>*Question relevant when: selected(${q102_E10_full_list} , '1')*	
q202_E10a *(required)*	q202_E10a. What percent of patients require this item?<br>*Question relevant when: selected(${q102_E10_full_list} , '1')*<br>*Response constrained to: .>0 and .<=100 or .=999*	
q202_E10b *(required)*	q202_E10b. How many units of this item are required per patient (throughout the entire treatment provided for complication)?<br>*Smallest unit is one speculum.*<br>*Question relevant when: selected(${q102_E10_full_list} , '1')*<br>*Response constrained to: .>=1*	
q202_E11	q202_E11. Speculum, Sim's (reusable)<br>*Question relevant when: selected(${q102_E11_full_list} , '1')*	
q202_E11a *(required)*	q202_E11a. What percent of patients require this item?<br>*Question relevant when: selected(${q102_E11_full_list} , '1')*<br>*Response constrained to: .>0 and .<=100 or .=999*	
q202_E11b *(required)*	q202_E11b. How many units of this item are required per patient (throughout the entire treatment provided for complication)?<br>*Smallest unit is one speculum.*<br>*Question relevant when: selected(${q102_E11_full_list} , '1')*<br>*Response constrained to: .>=1*	
q202_E12	q202_E12. Tenaculum<br>*Question relevant when: selected(${q102_E12_full_list} , '1')*	
q202_E12a *(required)*	q202_E12a. What percent of patients require this item?<br>*Question relevant when: selected(${q102_E12_full_list} , '1')*<br>*Response constrained to: .>0 and .<=100 or .=999*	
q202_E12b *(required)*	q202_E12b. How many units of this item are required per patient (throughout the entire treatment provided for complication)?<br>*Smallest unit is one tenaculum.*<br>*Question relevant when: selected(${q102_E12_full_list} , '1')*<br>*Response constrained to: .>=1*	

Field	Question	Answer
B. Small equipment - Incomplete Abortion (3)<br>*Group relevant when: selected(${section_two_skip_equ} , '1')*		
note_202_linens	***Linens***	
q202_E13	q202_E13. Towel<br>*Question relevant when: selected(${q102_E13_full_list} , '1')*	
q202_E13a *(required)*	q202_E13a. What percent of patients require this item?<br>*Question relevant when: selected(${q102_E13_full_list} , '1')*<br>*Response constrained to: .>0 and .<=100 or .=999*	
q202_E13b *(required)*	q202_E13b. How many units of this item are required per patient (throughout the entire treatment provided for complication)?<br>*Smallest unit is one towel.*<br>*Question relevant when: selected(${q102_E13_full_list} , '1')*<br>*Response constrained to: .>=1*	
q202_E14	q202_E14. Sheet<br>*Question relevant when: selected(${q102_E14_full_list} , '1')*	
q202_E14a *(required)*	q202_E14a. What percent of patients require this item?<br>*Question relevant when: selected(${q102_E14_full_list} , '1')*<br>*Response constrained to: .>0 and .<=100 or .=999*	
q202_E14b *(required)*	q202_E14b. How many units of this item are required per patient (throughout the entire treatment provided for complication)?<br>*Smallest unit is one sheet.*<br>*Question relevant when: selected(${q102_E14_full_list} , '1')*<br>*Response constrained to: .>=1*	
q202_E15	q202_E15. Blanket<br>*Question relevant when: selected(${q102_E15_full_list} , '1')*	
q202_E15a *(required)*	q202_E15a. What percent of patients require this item?<br>*Question relevant when: selected(${q102_E15_full_list} , '1')*<br>*Response constrained to: .>0 and .<=100 or .=999*	
q202_E15b *(required)*	q202_E15b. How many units of this item are required per patient (throughout the entire treatment provided for complication)?<br>*Smallest unit is one blanket.*<br>*Question relevant when: selected(${q102_E15_full_list} , '1')*<br>*Response constrained to: .>=1*	
q202_E16	q202_E16. Pillow<br>*Question relevant when: selected(${q102_E16_full_list} , '1')*	
q202_E16a *(required)*	q202_E16a. What percent of patients require this item?<br>*Question relevant when: selected(${q102_E16_full_list} , '1')*<br>*Response constrained to: .>0 and .<=100 or .=999*	
q202_E16b *(required)*	q202_E16b. How many units of this item are required per patient (throughout the entire treatment provided for complication)?<br>*Smallest unit is one pillow.*<br>*Question relevant when: selected(${q102_E16_full_list} , '1')*<br>*Response constrained to: .>=1*	
B. Small equipment - Incomplete Abortion (4)<br>*Group relevant when: selected(${section_two_skip_equ} , '1')*		
note_202_protective	***Protective supplies***	
q202_E17	q202_E17. Apron (reusable)<br>*Question relevant when: selected(${q102_E17_full_list} , '1')*	
q202_E17a *(required)*	q202_E17a. What percent of patients require this item?<br>*Question relevant when: selected(${q102_E17_full_list} , '1')*<br>*Response constrained to: .>0 and .<=100 or .=999*	
q202_E17b *(required)*	q202_E17b. How many units of this item are required per patient (throughout the entire treatment provided for complication)?<br>*Smallest unit is one apron.*<br>*Question relevant when: selected(${q102_E17_full_list} , '1')*<br>*Response constrained to: .>=1*	
q202_E18	q202_E18. Gown (reusable)<br>*Question relevant when: selected(${q102_E18_full_list} , '1')*	
q202_E18a *(required)*	q202_E18a. What percent of patients require this item?<br>*Question relevant when: selected(${q102_E18_full_list} , '1')*<br>*Response constrained to: .>0 and .<=100 or .=999*	

Field	Question	Answer
q202_E18b *(required)*	q202_E18b. How many units of this item are required per patient (throughout the entire treatment provided for complication)?<br>*Smallest unit is one gown.*<br>*Question relevant when: selected(${q102_E18_full_list} , '1')*<br>*Response constrained to: .>=1*	
q202_E19	q202_E19. Heavy duty gloves<br>*Question relevant when: selected(${q102_E19_full_list} , '1')*	
q202_E19a *(required)*	q202_E19a. What percent of patients require this item?<br>*Question relevant when: selected(${q102_E19_full_list} , '1')*<br>*Response constrained to: .>0 and .<=100 or .=999*	
q202_E19b *(required)*	q202_E19b. How many units of this item are required per patient (throughout the entire treatment provided for complication)?<br>*Smallest unit is one glove.*<br>*Question relevant when: selected(${q102_E19_full_list} , '1')*<br>*Response constrained to: .>=1*	
q202_E20	q202_E20. Mask (reusable)<br>*Question relevant when: selected(${q102_E20_full_list} , '1')*	
q202_E20a *(required)*	q202_E20a. What percent of patients require this item?<br>*Question relevant when: selected(${q102_E20_full_list} , '1')*<br>*Response constrained to: .>0 and .<=100 or .=999*	
q202_E20b *(required)*	q202_E20b. How many units of this item are required per patient (throughout the entire treatment provided for complication)?<br>*Smallest unit is one mask.*<br>*Question relevant when: selected(${q102_E20_full_list} , '1')*<br>*Response constrained to: .>=1*	
q202_E21	q202_E21. Protective glasses<br>*Question relevant when: selected(${q102_E21_full_list} , '1')*	
q202_E21a *(required)*	q202_E21a. What percent of patients require this item?<br>*Question relevant when: selected(${q102_E21_full_list} , '1')*<br>*Response constrained to: .>0 and .<=100 or .=999*	
q202_E21b *(required)*	q202_E21b. How many units of this item are required per patient (throughout the entire treatment provided for complication)?<br>*Smallest unit is one pair of glasses.*<br>*Question relevant when: selected(${q102_E21_full_list} , '1')*<br>*Response constrained to: .>=1*	
B. Small equipment - Incomplete Abortion (5)<br>*Group relevant when: selected(${section_two_skip_equ} , '1')*		
note_202_receivers	***Receivers***	
q202_E22	q202_E22. Kidney dish - small<br>*Question relevant when: selected(${q102_E22_full_list} , '1')*	
q202_E22a *(required)*	q202_E22a. What percent of patients require this item?<br>*Question relevant when: selected(${q102_E22_full_list} , '1')*<br>*Response constrained to: .>0 and .<=100 or .=999*	
q202_E22b *(required)*	q202_E22b. How many units of this item are required per patient (throughout the entire treatment provided for complication)?<br>*Smallest unit is one dish.*<br>*Question relevant when: selected(${q102_E22_full_list} , '1')*<br>*Response constrained to: .>=1*	
q202_E23	q202_E23. Kidney dish - medium<br>*Question relevant when: selected(${q102_E23_full_list} , '1')*	
q202_E23a *(required)*	q202_E23a. What percent of patients require this item?<br>*Question relevant when: selected(${q102_E23_full_list} , '1')*<br>*Response constrained to: .>0 and .<=100 or .=999*	
q202_E23b *(required)*	q202_E23b. How many units of this item are required per patient (throughout the entire treatment provided for complication)?<br>*Smallest unit is one dish.*<br>*Question relevant when: selected(${q102_E23_full_list} , '1')*<br>*Response constrained to: .>=1*	
q202_E24	q202_E24. Kidney dish - large<br>*Question relevant when: selected(${q102_E24_full_list} , '1')*	
q202_E24a *(required)*	q202_E24a. What percent of patients require this item?<br>*Question relevant when: selected(${q102_E24_full_list} , '1')*<br>*Response constrained to: .>0 and .<=100 or .=999*	

Field	Question	Answer
q202_E24b *(required)*	q202_E24b. How many units of this item are required per patient (throughout the entire treatment provided for complication)?<br>*Smallest unit is one dish.*<br>*Question relevant when: selected(${q102_E24_full_list} , '1')*<br>*Response constrained to: .>=1*	
q202_E25	q202_E25. Receiving bowl - small<br>*Question relevant when: selected(${q102_E25_full_list} , '1')*	
q202_E25a *(required)*	q202_E25a. What percent of patients require this item?<br>*Question relevant when: selected(${q102_E25_full_list} , '1')*<br>*Response constrained to: .>0 and .<=100 or .=999*	
q202_E25b *(required)*	q202_E25b. How many units of this item are required per patient (throughout the entire treatment provided for complication)?<br>*Smallest unit is one dish.*<br>*Question relevant when: selected(${q102_E25_full_list} , '1')*<br>*Response constrained to: .>=1*	
q202_E26	q202_E26. Receiving bowl - medium<br>*Question relevant when: selected(${q102_E26_full_list} , '1')*	
q202_E26a *(required)*	q202_E26a. What percent of patients require this item?<br>*Question relevant when: selected(${q102_E26_full_list} , '1')*<br>*Response constrained to: .>0 and .<=100 or .=999*	
q202_E26b *(required)*	q202_E26b. How many units of this item are required per patient (throughout the entire treatment provided for complication)?<br>*Smallest unit is one dish.*<br>*Question relevant when: selected(${q102_E26_full_list} , '1')*<br>*Response constrained to: .>=1*	
q202_E27	q202_E27. Receiving bowl - large<br>*Question relevant when: selected(${q102_E27_full_list} , '1')*	
q202_E27a *(required)*	q202_E27a. What percent of patients require this item?<br>*Question relevant when: selected(${q102_E27_full_list} , '1')*<br>*Response constrained to: .>0 and .<=100 or .=999*	
q202_E27b *(required)*	q202_E27b. How many units of this item are required per patient (throughout the entire treatment provided for complication)?<br>*Smallest unit is one dish.*<br>*Question relevant when: selected(${q102_E27_full_list} , '1')*<br>*Response constrained to: .>=1*	
B. Small equipment - Incomplete Abortion (6)<br>*Group relevant when: selected(${section_two_skip_equ} , '1')*		
note_202_surgery	***Surgery***	
q202_E28	q202_E28. Scalpel, reusable<br>*Question relevant when: selected(${q102_E28_full_list} , '1')*	
q202_E28a *(required)*	q202_E28a. What percent of patients require this item?<br>*Question relevant when: selected(${q102_E28_full_list} , '1')*<br>*Response constrained to: .>0 and .<=100 or .=999*	
q202_E28b *(required)*	q202_E28b. How many units of this item are required per patient (throughout the entire treatment provided for complication)?<br>*Smallest unit is one scalpel.*<br>*Question relevant when: selected(${q102_E28_full_list} , '1')*<br>*Response constrained to: .>=1*	
q202_E29	q202_E29. Surgical scissors<br>*Question relevant when: selected(${q102_E29_full_list} , '1')*	
q202_E29a *(required)*	q202_E29a. What percent of patients require this item?<br>*Question relevant when: selected(${q102_E29_full_list} , '1')*<br>*Response constrained to: .>0 and .<=100 or .=999*	
q202_E29b *(required)*	q202_E29b. How many units of this item are required per patient (throughout the entire treatment provided for complication)?<br>*Smallest unit is one pair of scissors.*<br>*Question relevant when: selected(${q102_E29_full_list} , '1')*<br>*Response constrained to: .>=1*	
B. Small equipment - Incomplete Abortion (7)<br>*Group relevant when: selected(${section_two_skip_equ} , '1')*		
note_202_other	***Other small equipment***	
q202_E30	q202_E30. Head lamp/batteries<br>*Question relevant when: selected(${q102_E30_full_list} , '1')*	

Field	Question	Answer
q202_E30a *(required)*	q202_E30a. What percent of patients require this item?<br>*Question relevant when: selected(${q102_E30_full_list} , '1')*<br>*Response constrained to: .>0 and .<=100 or .=999*	
q202_E30b *(required)*	q202_E30b. How many units of this item are required per patient (throughout the entire treatment provided for complication)?<br>*Smallest unit is one lamp/battery set.*<br>*Question relevant when: selected(${q102_E30_full_list} , '1')*<br>*Response constrained to: .>=1*	
q202_E31	q202_E31. Metal catheter<br>*Question relevant when: selected(${q102_E31_full_list} , '1')*	
q202_E31a *(required)*	q202_E31a. What percent of patients require this item?<br>*Question relevant when: selected(${q102_E31_full_list} , '1')*<br>*Response constrained to: .>0 and .<=100 or .=999*	
q202_E31b *(required)*	q202_E31b. How many units of this item are required per patient (throughout the entire treatment provided for complication)?<br>*Smallest unit is one catheter.*<br>*Question relevant when: selected(${q102_E31_full_list} , '1')*<br>*Response constrained to: .>=1*	
q202_E32	q202_E32. Urine collection flask<br>*Question relevant when: selected(${q102_E32_full_list} , '1')*	
q202_E32a *(required)*	q202_E32a. What percent of patients require this item?<br>*Question relevant when: selected(${q102_E32_full_list} , '1')*<br>*Response constrained to: .>0 and .<=100 or .=999*	
q202_E32b *(required)*	q202_E32b. How many units of this item are required per patient (throughout the entire treatment provided for complication)?<br>*Smallest unit is one flask.*<br>*Question relevant when: selected(${q102_E32_full_list} , '1')*<br>*Response constrained to: .>=1*	
B. Small equipment - Incomplete Abortion (8)<br>*Group relevant when: selected(${section_two_skip_equ} , '1')*		
note_202_other_suggestion	***Other equipment - suggestions?***	
q202_E33	q202_E33. Equipment other 1: "[q102_E33_full_list_other]"<br>*Question relevant when: selected(${q102_E33_full_list} , '1')*	
q202_E33a *(required)*	q202_E33a. What percent of patients require this item?<br>*Question relevant when: selected(${q102_E33_full_list} , '1')*<br>*Response constrained to: .>0 and .<=100 or .=999*	
q202_E33b *(required)*	q202_E33b. How many units of this item are required per patient (throughout the entire treatment provided for complication)?<br>*Question relevant when: selected(${q102_E33_full_list} , '1')*<br>*Response constrained to: .>=1*	
q202_E34	q202_E34. Equipment other 2: "[q102_E34_full_list_other]"<br>*Question relevant when: selected(${q102_E34_full_list} , '1')*	
q202_E34a *(required)*	q202_E34a. What percent of patients require this item?<br>*Question relevant when: selected(${q102_E34_full_list} , '1')*<br>*Response constrained to: .>0 and .<=100 or .=999*	
q202_E34b *(required)*	q202_E34b. How many units of this item are required per patient (throughout the entire treatment provided for complication)?<br>*Question relevant when: selected(${q102_E34_full_list} , '1')*<br>*Response constrained to: .>=1*	
q202_E35	q202_E35. Equipment other 3: "[q102_E35_full_list_other]"<br>*Question relevant when: selected(${q102_E35_full_list} , '1')*	
q202_E35a *(required)*	q202_E35a. What percent of patients require this item?<br>*Question relevant when: selected(${q102_E35_full_list} , '1')*<br>*Response constrained to: .>0 and .<=100 or .=999*	
q202_E35b *(required)*	q202_E35b. How many units of this item are required per patient (throughout the entire treatment provided for complication)?<br>*Question relevant when: selected(${q102_E35_full_list} , '1')*<br>*Response constrained to: .>=1*	
q202_E36	q202_E36. Equipment other 4: "[q102_E36_full_list_other]"<br>*Question relevant when: selected(${q102_E36_full_list} , '1')*	
q202_E36a *(required)*	q202_E36a. What percent of patients require this item?<br>*Question relevant when: selected(${q102_E36_full_list} , '1')*<br>*Response constrained to: .>0 and .<=100 or .=999*	

Field	Question	Answer
q202_E36b *(required)*	q202_E36b. How many units of this item are required per patient (throughout the entire treatment provided for complication)?<br>*Question relevant when: selected(${q102_E36_full_list} , '1')*<br>*Response constrained to: .>=1*	
q202_E37	q202_E37. Equipment other 5: "[q102_E37_full_list_other]"<br>*Question relevant when: selected(${q102_E37_full_list} , '1')*	
q202_E37a *(required)*	q202_E37a. What percent of patients require this item?<br>*Question relevant when: selected(${q102_E37_full_list} , '1')*<br>*Response constrained to: .>0 and .<=100 or .=999*	
q202_E37b *(required)*	q202_E37b. How many units of this item are required per patient (throughout the entire treatment provided for complication)?<br>*Question relevant when: selected(${q102_E37_full_list} , '1')*<br>*Response constrained to: .>=1*	
group_section_three_intro		
section3_start	**SECTION III. SEPSIS - USAGE OF ALL ITEMS**	
section_three_skip_equ	INTERVIEWER: WOULD YOU LIKE TO COMPLETE THIS SECTION NOW OR SKIP THIS SECTION AND RETURN TO IT LATER?<br>*You may need to skip if the participant has indicated that s/he cannot answer the questions in this section.*	1 Do not skip, complete this section now.<br>2 Skip and come back to this section later.
group_section_three_introB<br>*Group relevant when: selected(${section_three_skip_equ} , '1')*		
section3_start2	In this section of the interview, we will review all of the items that you said are used for management of sepsis. For each item that is used, I'm going to ask questions on how many women need it and how much of it is used.	
section3_start3	INTERVIEWER: ENTER WHOLE NUMBERS OR DECIMALS. DO NOT TYPE PERCENT SIGNS. ENTER 999 FOR ANY THAT ARE UNKNOWN.	
B. Small equipment - Sepsis (1)<br>*Group relevant when: selected(${section_three_skip_equ} , '1')*		
note_302_evac	***Evacuation***	
q302_E1	q302_E1. MVA aspirator<br>*Question relevant when: selected(${q102_E1_full_list} , '2')*	
q302_E1a *(required)*	q302_E1a. What percent of patients require this item?<br>*Question relevant when: selected(${q102_E1_full_list} , '2')*<br>*Response constrained to: .>0 and .<=100 or .=999*	
q302_E1b *(required)*	q302_E1b. How many units of this item are required per patient (throughout the entire treatment provided for complication)?<br>*Smallest unit is one aspirator.*<br>*Question relevant when: selected(${q102_E1_full_list} , '2')*<br>*Response constrained to: .>=1*	
q302_E2	q302_E2. Cannula set<br>*Question relevant when: selected(${q102_E2_full_list} , '2')*	
q302_E2a *(required)*	q302_E2a. What percent of patients require this item?<br>*Question relevant when: selected(${q102_E2_full_list} , '2')*<br>*Response constrained to: .>0 and .<=100 or .=999*	
q302_E2b *(required)*	q302_E2b. How many units of this item are required per patient (throughout the entire treatment provided for complication)?<br>*Smallest unit is one set.*<br>*Question relevant when: selected(${q102_E2_full_list} , '2')*<br>*Response constrained to: .>=1*	
q302_E3	q302_E3. Uterine curette set - plastic<br>*Question relevant when: selected(${q102_E3_full_list} , '2')*	
q302_E3a *(required)*	q302_E3a. What percent of patients require this item?<br>*Question relevant when: selected(${q102_E3_full_list} , '2')*<br>*Response constrained to: .>0 and .<=100 or .=999*	
q302_E3b *(required)*	q302_E3b. How many units of this item are required per patient (throughout the entire treatment provided for complication)?<br>*Smallest unit is one set.*<br>*Question relevant when: selected(${q102_E3_full_list} , '2')*<br>*Response constrained to: .>=1*	
q302_E4	q302_E4. Uterine currette set - metal<br>*Question relevant when: selected(${q102_E4_full_list} , '2')*	
q302_E4a *(required)*	q302_E4a. What percent of patients require this item?<br>*Question relevant when: selected(${q102_E4_full_list} , '2')*<br>*Response constrained to: .>0 and .<=100 or .=999*	

Field	Question	Answer
q302_E4b *(required)*	q302_E4b. How many units of this item are required per patient (throughout the entire treatment provided for complication)?<br>*Smallest unit is one set.*<br>*Question relevant when: selected(${q102_E4_full_list} , '2')*<br>*Response constrained to: .>=1*	
q302_E5	q302_E5. Dilators (Hegar's Cervical dilators)<br>*Question relevant when: selected(${q102_E5_full_list} , '2')*	
q302_E5a *(required)*	q302_E5a. What percent of patients require this item?<br>*Question relevant when: selected(${q102_E5_full_list} , '2')*<br>*Response constrained to: .>0 and .<=100 or .=999*	
q302_E5b *(required)*	q302_E5b. How many units of this item are required per patient (throughout the entire treatment provided for complication)?<br>*Smallest unit is one set.*<br>*Question relevant when: selected(${q102_E5_full_list} , '2')*<br>*Response constrained to: .>=1*	
B. Small equipment - Sepsis (2)<br>*Group relevant when: selected(${section_three_skip_equ} , '1')*		
note_302_forceps	***Forceps, speculums, dilators, etc.***	
q302_E6	q302_E6. Forceps, artery/Kocher<br>*Question relevant when: selected(${q102_E6_full_list} , '2')*	
q302_E6a *(required)*	q302_E6a. What percent of patients require this item?<br>*Question relevant when: selected(${q102_E6_full_list} , '2')*<br>*Response constrained to: .>0 and .<=100 or .=999*	
q302_E6b *(required)*	q302_E6b. How many units of this item are required per patient (throughout the entire treatment provided for complication)?<br>*Smallest unit is one forceps.*<br>*Question relevant when: selected(${q102_E6_full_list} , '2')*<br>*Response constrained to: .>=1*	
q302_E7	q302_E7. Needle holder<br>*Question relevant when: selected(${q102_E7_full_list} , '2')*	
q302_E7a *(required)*	q302_E7a. What percent of patients require this item?<br>*Question relevant when: selected(${q102_E7_full_list} , '2')*<br>*Response constrained to: .>0 and .<=100 or .=999*	
q302_E7b *(required)*	q302_E7b. How many units of this item are required per patient (throughout the entire treatment provided for complication)?<br>*Smallest unit is one holder.*<br>*Question relevant when: selected(${q102_E7_full_list} , '2')*<br>*Response constrained to: .>=1*	
q302_E8	q302_E8. Ovum forceps<br>*Question relevant when: selected(${q102_E8_full_list} , '2')*	
q302_E8a *(required)*	q302_E8a. What percent of patients require this item?<br>*Question relevant when: selected(${q102_E8_full_list} , '2')*<br>*Response constrained to: .>0 and .<=100 or .=999*	
q302_E8b *(required)*	q302_E8b. How many units of this item are required per patient (throughout the entire treatment provided for complication)?<br>*Smallest unit is one forceps.*<br>*Question relevant when: selected(${q102_E8_full_list} , '2')*<br>*Response constrained to: .>=1*	
q302_E9	q302_E9. Ring forceps<br>*Question relevant when: selected(${q102_E9_full_list} , '2')*	
q302_E9a *(required)*	q302_E9a. What percent of patients require this item?<br>*Question relevant when: selected(${q102_E9_full_list} , '2')*<br>*Response constrained to: .>0 and .<=100 or .=999*	
q302_E9b *(required)*	q302_E9b. How many units of this item are required per patient (throughout the entire treatment provided for complication)?<br>*Smallest unit is one forceps.*<br>*Question relevant when: selected(${q102_E9_full_list} , '2')*<br>*Response constrained to: .>=1*	
q302_E10	q302_E10. Speculum, Bivalve/Cuscos (reusable)<br>*Question relevant when: selected(${q102_E10_full_list} , '2')*	
q302_E10a *(required)*	q302_E10a. What percent of patients require this item?<br>*Question relevant when: selected(${q102_E10_full_list} , '2')*<br>*Response constrained to: .>0 and .<=100 or .=999*	

Field	Question	Answer
q302_E10b *(required)*	q302_E10b. How many units of this item are required per patient (throughout the entire treatment provided for complication)?<br>*Smallest unit is one speculum.*<br>*Question relevant when: selected(${q102_E10_full_list} , '2')*<br>*Response constrained to: .>=1*	
q302_E11	q302_E11. Speculum, Sim's (reusable)<br>*Question relevant when: selected(${q102_E11_full_list} , '2')*	
q302_E11a *(required)*	q302_E11a. What percent of patients require this item?<br>*Question relevant when: selected(${q102_E11_full_list} , '2')*<br>*Response constrained to: .>0 and .<=100 or .=999*	
q302_E11b *(required)*	q302_E11b. How many units of this item are required per patient (throughout the entire treatment provided for complication)?<br>*Smallest unit is one speculum.*<br>*Question relevant when: selected(${q102_E11_full_list} , '2')*<br>*Response constrained to: .>=1*	
q302_E12	q302_E12. Tenaculum<br>*Question relevant when: selected(${q102_E12_full_list} , '2')*	
q302_E12a *(required)*	q302_E12a. What percent of patients require this item?<br>*Question relevant when: selected(${q102_E12_full_list} , '2')*<br>*Response constrained to: .>0 and .<=100 or .=999*	
q302_E12b *(required)*	q302_E12b. How many units of this item are required per patient (throughout the entire treatment provided for complication)?<br>*Smallest unit is one tenaculum.*<br>*Question relevant when: selected(${q102_E12_full_list} , '2')*<br>*Response constrained to: .>=1*	
B. Small equipment - Sepsis (3)<br>*Group relevant when: selected(${section_three_skip_equ} , '1')*		
note_302_linens	***Linens***	
q302_E13	q302_E13. Towel<br>*Question relevant when: selected(${q102_E13_full_list} , '2')*	
q302_E13a *(required)*	q302_E13a. What percent of patients require this item?<br>*Question relevant when: selected(${q102_E13_full_list} , '2')*<br>*Response constrained to: .>0 and .<=100 or .=999*	
q302_E13b *(required)*	q302_E13b. How many units of this item are required per patient (throughout the entire treatment provided for complication)?<br>*Smallest unit is one towel.*<br>*Question relevant when: selected(${q102_E13_full_list} , '2')*<br>*Response constrained to: .>=1*	
q302_E14	q302_E14. Sheet<br>*Question relevant when: selected(${q102_E14_full_list} , '2')*	
q302_E14a *(required)*	q302_E14a. What percent of patients require this item?<br>*Question relevant when: selected(${q102_E14_full_list} , '2')*<br>*Response constrained to: .>0 and .<=100 or .=999*	
q302_E14b *(required)*	q302_E14b. How many units of this item are required per patient (throughout the entire treatment provided for complication)?<br>*Smallest unit is one sheet.*<br>*Question relevant when: selected(${q102_E14_full_list} , '2')*<br>*Response constrained to: .>=1*	
q302_E15	q302_E15. Blanket<br>*Question relevant when: selected(${q102_E15_full_list} , '2')*	
q302_E15a *(required)*	q302_E15a. What percent of patients require this item?<br>*Question relevant when: selected(${q102_E15_full_list} , '2')*<br>*Response constrained to: .>0 and .<=100 or .=999*	
q302_E15b *(required)*	q302_E15b. How many units of this item are required per patient (throughout the entire treatment provided for complication)?<br>*Smallest unit is one blanket.*<br>*Question relevant when: selected(${q102_E15_full_list} , '2')*<br>*Response constrained to: .>=1*	
q302_E16	q302_E16. Pillow<br>*Question relevant when: selected(${q102_E16_full_list} , '2')*	
q302_E16a *(required)*	q302_E16a. What percent of patients require this item?<br>*Question relevant when: selected(${q102_E16_full_list} , '2')*<br>*Response constrained to: .>0 and .<=100 or .=999*	

Field	Question	Answer
q302_E16b *(required)*	q302_E16b. How many units of this item are required per patient (throughout the entire treatment provided for complication)?<br>*Smallest unit is one pillow.*<br>*Question relevant when: selected(${q102_E16_full_list} , '2')*<br>*Response constrained to: .>=1*	
B. Small equipment - Sepsis (4)<br>*Group relevant when: selected(${section_three_skip_equ} , '1')*		
note_302_protective	***Protective supplies***	
q302_E17	q302_E17. Apron (reusable)<br>*Question relevant when: selected(${q102_E17_full_list} , '2')*	
q302_E17a *(required)*	q302_E17a. What percent of patients require this item?<br>*Question relevant when: selected(${q102_E17_full_list} , '2')*<br>*Response constrained to: .>0 and .<=100 or .=999*	
q302_E17b *(required)*	q302_E17b. How many units of this item are required per patient (throughout the entire treatment provided for complication)?<br>*Smallest unit is one apron.*<br>*Question relevant when: selected(${q102_E17_full_list} , '2')*<br>*Response constrained to: .>=1*	
q302_E18	q302_E18. Gown (reusable)<br>*Question relevant when: selected(${q102_E18_full_list} , '2')*	
q302_E18a *(required)*	q302_E18a. What percent of patients require this item?<br>*Question relevant when: selected(${q102_E18_full_list} , '2')*<br>*Response constrained to: .>0 and .<=100 or .=999*	
q302_E18b *(required)*	q302_E18b. How many units of this item are required per patient (throughout the entire treatment provided for complication)?<br>*Smallest unit is one gown.*<br>*Question relevant when: selected(${q102_E18_full_list} , '2')*<br>*Response constrained to: .>=1*	
q302_E19	q302_E19. Heavy duty gloves<br>*Question relevant when: selected(${q102_E19_full_list} , '2')*	
q302_E19a *(required)*	q302_E19a. What percent of patients require this item?<br>*Question relevant when: selected(${q102_E19_full_list} , '2')*<br>*Response constrained to: .>0 and .<=100 or .=999*	
q302_E19b *(required)*	q302_E19b. How many units of this item are required per patient (throughout the entire treatment provided for complication)?<br>*Smallest unit is one glove.*<br>*Question relevant when: selected(${q102_E19_full_list} , '2')*<br>*Response constrained to: .>=1*	
q302_E20	q302_E20. Mask (reusable)<br>*Question relevant when: selected(${q102_E20_full_list} , '2')*	
q302_E20a *(required)*	q302_E20a. What percent of patients require this item?<br>*Question relevant when: selected(${q102_E20_full_list} , '2')*<br>*Response constrained to: .>0 and .<=100 or .=999*	
q302_E20b *(required)*	q302_E20b. How many units of this item are required per patient (throughout the entire treatment provided for complication)?<br>*Smallest unit is one mask.*<br>*Question relevant when: selected(${q102_E20_full_list} , '2')*<br>*Response constrained to: .>=1*	
q302_E21	q302_E21. Protective glasses<br>*Question relevant when: selected(${q102_E21_full_list} , '2')*	
q302_E21a *(required)*	q302_E21a. What percent of patients require this item?<br>*Question relevant when: selected(${q102_E21_full_list} , '2')*<br>*Response constrained to: .>0 and .<=100 or .=999*	
q302_E21b *(required)*	q302_E21b. How many units of this item are required per patient (throughout the entire treatment provided for complication)?<br>*Smallest unit is one pair of glasses.*<br>*Question relevant when: selected(${q102_E21_full_list} , '2')*<br>*Response constrained to: .>=1*	
B. Small equipment - Sepsis (5)		
note_302_receivers	***Receivers***<br>*Question relevant when: selected(${section_three_skip_equ} , '1')*	
q302_E22	q302_E22. Kidney dish - small<br>*Question relevant when: selected(${q102_E22_full_list} , '2')*	

Field	Question	Answer
q302_E22a *(required)*	q302_E22a. What percent of patients require this item?<br>*Question relevant when: selected(${q102_E22_full_list} , '2')*<br>*Response constrained to: .>0 and .<=100 or .=999*	
q302_E22b *(required)*	q302_E22b. How many units of this item are required per patient (throughout the entire treatment provided for complication)?<br>*Smallest unit is one dish.*<br>*Question relevant when: selected(${q102_E22_full_list} , '2')*<br>*Response constrained to: .>=1*	
q302_E23	q302_E23. Kidney dish - medium<br>*Question relevant when: selected(${q102_E23_full_list} , '2')*	
q302_E23a *(required)*	q302_E23a. What percent of patients require this item?<br>*Question relevant when: selected(${q102_E23_full_list} , '2')*<br>*Response constrained to: .>0 and .<=100 or .=999*	
q302_E23b *(required)*	q302_E23b. How many units of this item are required per patient (throughout the entire treatment provided for complication)?<br>*Smallest unit is one dish.*<br>*Question relevant when: selected(${q102_E23_full_list} , '2')*<br>*Response constrained to: .>=1*	
q302_E24	q302_E24. Kidney dish - large<br>*Question relevant when: selected(${q102_E24_full_list} , '2')*	
q302_E24a *(required)*	q302_E24a. What percent of patients require this item?<br>*Question relevant when: selected(${q102_E24_full_list} , '2')*<br>*Response constrained to: .>0 and .<=100 or .=999*	
q302_E24b *(required)*	q302_E24b. How many units of this item are required per patient (throughout the entire treatment provided for complication)?<br>*Smallest unit is one dish.*<br>*Question relevant when: selected(${q102_E24_full_list} , '2')*<br>*Response constrained to: .>=1*	
q302_E25	q302_E25. Receiving bowl - small<br>*Question relevant when: selected(${q102_E25_full_list} , '2')*	
q302_E25a *(required)*	q302_E25a. What percent of patients require this item?<br>*Question relevant when: selected(${q102_E25_full_list} , '2')*<br>*Response constrained to: .>0 and .<=100 or .=999*	
q302_E25b *(required)*	q302_E25b. How many units of this item are required per patient (throughout the entire treatment provided for complication)?<br>*Smallest unit is one dish.*<br>*Question relevant when: selected(${q102_E25_full_list} , '2')*<br>*Response constrained to: .>=1*	
q302_E26	q302_E26. Receiving bowl - medium<br>*Question relevant when: selected(${q102_E26_full_list} , '2')*	
q302_E26a *(required)*	q302_E26a. What percent of patients require this item?<br>*Question relevant when: selected(${q102_E26_full_list} , '2')*<br>*Response constrained to: .>0 and .<=100 or .=999*	
q302_E26b *(required)*	q302_E26b. How many units of this item are required per patient (throughout the entire treatment provided for complication)?<br>*Smallest unit is one dish.*<br>*Question relevant when: selected(${q102_E26_full_list} , '2')*<br>*Response constrained to: .>=1*	
q302_E27	q302_E27. Receiving bowl - large<br>*Question relevant when: selected(${q102_E27_full_list} , '2')*	
q302_E27a *(required)*	q302_E27a. What percent of patients require this item?<br>*Question relevant when: selected(${q102_E27_full_list} , '2')*<br>*Response constrained to: .>0 and .<=100 or .=999*	
q302_E27b *(required)*	q302_E27b. How many units of this item are required per patient (throughout the entire treatment provided for complication)?<br>*Smallest unit is one dish.*<br>*Question relevant when: selected(${q102_E27_full_list} , '2')*<br>*Response constrained to: .>=1*	
B. Small equipment - Sepsis (6)<br>*Group relevant when: selected(${section_three_skip_equ} , '1')*		
note_302_surgery	***Surgery***	
q302_E28	q302_E28. Scalpel, reusable<br>*Question relevant when: selected(${q102_E28_full_list} , '2')*	

Field	Question	Answer
q302_E28a *(required)*	q302_E28a. What percent of patients require this item?<br>*Question relevant when: selected(${q102_E28_full_list} , '2')*<br>*Response constrained to: .>0 and .<=100 or .=999*	
q302_E28b *(required)*	q302_E28b. How many units of this item are required per patient (throughout the entire treatment provided for complication)?<br>*Smallest unit is one scalpel.*<br>*Question relevant when: selected(${q102_E28_full_list} , '2')*<br>*Response constrained to: .>=1*	
q302_E29	q302_E29. Surgical scissors<br>*Question relevant when: selected(${q102_E29_full_list} , '2')*	
q302_E29a *(required)*	q302_E29a. What percent of patients require this item?<br>*Question relevant when: selected(${q102_E29_full_list} , '2')*<br>*Response constrained to: .>0 and .<=100 or .=999*	
q302_E29b *(required)*	q302_E29b. How many units of this item are required per patient (throughout the entire treatment provided for complication)?<br>*Smallest unit is one pair of scissors.*<br>*Question relevant when: selected(${q102_E29_full_list} , '2')*<br>*Response constrained to: .>=1*	
B. Small equipment - Sepsis (7)<br>*Group relevant when: selected(${section_three_skip_equ} , '1')*		
note_302_other	***Other small equipment***	
q302_E30	q302_E30. Head lamp/batteries<br>*Question relevant when: selected(${q102_E30_full_list} , '2')*	
q302_E30a *(required)*	q302_E30a. What percent of patients require this item?<br>*Question relevant when: selected(${q102_E30_full_list} , '2')*<br>*Response constrained to: .>0 and .<=100 or .=999*	
q302_E30b *(required)*	q302_E30b. How many units of this item are required per patient (throughout the entire treatment provided for complication)?<br>*Smallest unit is one lamp/battery set.*<br>*Question relevant when: selected(${q102_E30_full_list} , '2')*<br>*Response constrained to: .>=1*	
q302_E31	q302_E31. Metal catheter<br>*Question relevant when: selected(${q102_E31_full_list} , '2')*	
q302_E31a *(required)*	q302_E31a. What percent of patients require this item?<br>*Question relevant when: selected(${q102_E31_full_list} , '2')*<br>*Response constrained to: .>0 and .<=100 or .=999*	
q302_E31b *(required)*	q302_E31b. How many units of this item are required per patient (throughout the entire treatment provided for complication)?<br>*Smallest unit is one catheter.*<br>*Question relevant when: selected(${q102_E31_full_list} , '2')*<br>*Response constrained to: .>=1*	
q302_E32	q302_E32. Urine collection flask<br>*Question relevant when: selected(${q102_E32_full_list} , '2')*	
q302_E32a *(required)*	q302_E32a. What percent of patients require this item?<br>*Question relevant when: selected(${q102_E32_full_list} , '2')*<br>*Response constrained to: .>0 and .<=100 or .=999*	
q302_E32b *(required)*	q302_E32b. How many units of this item are required per patient (throughout the entire treatment provided for complication)?<br>*Smallest unit is one flask.*<br>*Question relevant when: selected(${q102_E32_full_list} , '2')*<br>*Response constrained to: .>=1*	
B. Small equipment - Sepsis (8)<br>*Group relevant when: selected(${section_three_skip_equ} , '1')*		
note_302_other_suggestion	***Other equipment - suggestions?***	
q302_E33	q302_E33. Equipment other 1: "[q102_E33_full_list_other]"<br>*Question relevant when: selected(${q102_E33_full_list} , '2')*	
q302_E33a *(required)*	q302_E33a. What percent of patients require this item?<br>*Question relevant when: selected(${q102_E33_full_list} , '2')*<br>*Response constrained to: .>0 and .<=100 or .=999*	
q302_E33b *(required)*	q302_E33b. How many units of this item are required per patient (throughout the entire treatment provided for complication)?<br>*Question relevant when: selected(${q102_E33_full_list} , '2')*<br>*Response constrained to: .>=1*	

Field	Question	Answer
q302_E34	q302_E34. Equipment other 2: "[q102_E34_full_list_other]"<br>*Question relevant when: selected(${q102_E34_full_list} , '2')*	

Field	Question	Answer
q302_E34a *(required)*	q302_E34a. What percent of patients require this item?<br>*Question relevant when: selected(${q102_E34_full_list} , '2')*<br>*Response constrained to: .>0 and .<=100 or .=999*	
q302_E34b *(required)*	q302_E34b. How many units of this item are required per patient (throughout the entire treatment provided for complication)?<br>*Question relevant when: selected(${q102_E34_full_list} , '2')*<br>*Response constrained to: .>=1*	
q302_E35	q302_E35. Equipment other 3: "[q102_E35_full_list_other]"<br>*Question relevant when: selected(${q102_E35_full_list} , '2')*	
q302_E35a *(required)*	q302_E35a. What percent of patients require this item?<br>*Question relevant when: selected(${q102_E35_full_list} , '2')*<br>*Response constrained to: .>0 and .<=100 or .=999*	
q302_E35b *(required)*	q302_E35b. How many units of this item are required per patient (throughout the entire treatment provided for complication)?<br>*Question relevant when: selected(${q102_E35_full_list} , '2')*<br>*Response constrained to: .>=1*	
q302_E36	q302_E36. Equipment other 4: "[q102_E36_full_list_other]"<br>*Question relevant when: selected(${q102_E36_full_list} , '2')*	
q302_E36a *(required)*	q302_E36a. What percent of patients require this item?<br>*Question relevant when: selected(${q102_E36_full_list} , '2')*<br>*Response constrained to: .>0 and .<=100 or .=999*	
q302_E36b *(required)*	q302_E36b. How many units of this item are required per patient (throughout the entire treatment provided for complication)?<br>*Question relevant when: selected(${q102_E36_full_list} , '2')*<br>*Response constrained to: .>=1*	
q302_E37	q302_E37. Equipment other 5: "[q102_E37_full_list_other]"<br>*Question relevant when: selected(${q102_E37_full_list} , '2')*	
q302_E37a *(required)*	q302_E37a. What percent of patients require this item?<br>*Question relevant when: selected(${q102_E37_full_list} , '2')*<br>*Response constrained to: .>0 and .<=100 or .=999*	
q302_E37b *(required)*	q302_E37b. How many units of this item are required per patient (throughout the entire treatment provided for complication)?<br>*Question relevant when: selected(${q102_E37_full_list} , '2')*<br>*Response constrained to: .>=1*	
group_section_four_intro		
section4_start	**SECTION IV. SHOCK - USAGE OF ALL ITEMS**	
section_four_skip_equ	INTERVIEWER: WOULD YOU LIKE TO COMPLETE THIS SECTION NOW OR SKIP THIS SECTION AND RETURN TO IT LATER?<br>*You may need to skip if the participant has indicated that s/he cannot answer the questions in this section.*	1 Do not skip, complete this section now.<br>2 Skip and come back to this section later.
group_section_four_introB<br>*Group relevant when: selected(${section_four_skip_equ} , '1')*		
section4_start2	In this section of the interview, we will review all of the items that you said are used for management of shock. For each item that is used, I'm going to ask questions on how many women need it and how much of it is used.	
section4_start3	INTERVIEWER: ENTER WHOLE NUMBERS OR DECIMALS. DO NOT TYPE PERCENT SIGNS. ENTER 999 FOR ANY THAT ARE UNKNOWN.	
B. Small equipment - Shock (1)<br>*Group relevant when: selected(${section_four_skip_equ} , '1')*		
note_402_evac	***Evacuation***	
q402_E1	q402_E1. MVA aspirator<br>*Question relevant when: selected(${q102_E1_full_list} , '3')*	
q402_E1a *(required)*	q402_E1a. What percent of patients require this item?<br>*Question relevant when: selected(${q102_E1_full_list} , '3')*<br>*Response constrained to: .>0 and .<=100 or .=999*	
q402_E1b *(required)*	q402_E1b. How many units of this item are required per patient (throughout the entire treatment provided for complication)?<br>*Smallest unit is one aspirator.*<br>*Question relevant when: selected(${q102_E1_full_list} , '3')*<br>*Response constrained to: .>=1*	
q402_E2	q402_E2. Cannula set<br>*Question relevant when: selected(${q102_E2_full_list} , '3')*	

Field	Question	Answer
q402_E2a *(required)*	q402_E2a. What percent of patients require this item?<br>*Question relevant when: selected(${q102_E2_full_list} , '3')*<br>*Response constrained to: .>0 and .<=100 or .=999*	
q402_E2b *(required)*	q402_E2b. How many units of this item are required per patient (throughout the entire treatment provided for complication)?<br>*Smallest unit is one set.*<br>*Question relevant when: selected(${q102_E2_full_list} , '3')*<br>*Response constrained to: .>=1*	
q402_E3	q402_E3. Uterine curette set - plastic<br>*Question relevant when: selected(${q102_E3_full_list} , '3')*	
q402_E3a *(required)*	q402_E3a. What percent of patients require this item?<br>*Question relevant when: selected(${q102_E3_full_list} , '3')*<br>*Response constrained to: .>0 and .<=100 or .=999*	
q402_E3b *(required)*	q402_E3b. How many units of this item are required per patient (throughout the entire treatment provided for complication)?<br>*Smallest unit is one set.*<br>*Question relevant when: selected(${q102_E3_full_list} , '3')*<br>*Response constrained to: .>=1*	
q402_E4	q402_E4. Uterine currette set - metal<br>*Question relevant when: selected(${q102_E4_full_list} , '3')*	
q402_E4a *(required)*	q402_E4a. What percent of patients require this item?<br>*Question relevant when: selected(${q102_E4_full_list} , '3')*<br>*Response constrained to: .>0 and .<=100 or .=999*	
q402_E4b *(required)*	q402_E4b. How many units of this item are required per patient (throughout the entire treatment provided for complication)?<br>*Smallest unit is one set.*<br>*Question relevant when: selected(${q102_E4_full_list} , '3')*<br>*Response constrained to: .>=1*	
q402_E5	q402_E5. Dilators (Hegar's Cervical dilators)<br>*Question relevant when: selected(${q102_E5_full_list} , '3')*	
q402_E5a *(required)*	q402_E5a. What percent of patients require this item?<br>*Question relevant when: selected(${q102_E5_full_list} , '3')*<br>*Response constrained to: .>0 and .<=100 or .=999*	
q402_E5b *(required)*	q402_E5b. How many units of this item are required per patient (throughout the entire treatment provided for complication)?<br>*Smallest unit is one set.*<br>*Question relevant when: selected(${q102_E5_full_list} , '3')*<br>*Response constrained to: .>=1*	
B. Small equipment - Shock (2)<br>*Group relevant when: selected(${section_four_skip_equ} , '1')*		
note_402_forceps	***Forceps, speculums, dilators, etc.***	
q402_E6	q402_E6. Forceps, artery/Kocher<br>*Question relevant when: selected(${q102_E6_full_list} , '3')*	
q402_E6a *(required)*	q402_E6a. What percent of patients require this item?<br>*Question relevant when: selected(${q102_E6_full_list} , '3')*<br>*Response constrained to: .>0 and .<=100 or .=999*	
q402_E6b *(required)*	q402_E6b. How many units of this item are required per patient (throughout the entire treatment provided for complication)?<br>*Smallest unit is one forceps.*<br>*Question relevant when: selected(${q102_E6_full_list} , '3')*<br>*Response constrained to: .>=1*	
q402_E7	q402_E7. Needle holder<br>*Question relevant when: selected(${q102_E7_full_list} , '3')*	
q402_E7a *(required)*	q402_E7a. What percent of patients require this item?<br>*Question relevant when: selected(${q102_E7_full_list} , '3')*<br>*Response constrained to: .>0 and .<=100 or .=999*	
q402_E7b *(required)*	q402_E7b. How many units of this item are required per patient (throughout the entire treatment provided for complication)?<br>*Smallest unit is one holder.*<br>*Question relevant when: selected(${q102_E7_full_list} , '3')*<br>*Response constrained to: .>=1*	
q402_E8	q402_E8. Ovum forceps<br>*Question relevant when: selected(${q102_E8_full_list} , '3')*	

Field	Question	Answer
q402_E8a *(required)*	q402_E8a. What percent of patients require this item?<br>*Question relevant when: selected(${q102_E8_full_list} , '3')*<br>*Response constrained to: .>0 and .<=100 or .=999*	
q402_E8b *(required)*	q402_E8b. How many units of this item are required per patient (throughout the entire treatment provided for complication)?<br>*Smallest unit is one forceps.*<br>*Question relevant when: selected(${q102_E8_full_list} , '3')*<br>*Response constrained to: .>=1*	
q402_E9	q402_E9. Ring forceps<br>*Question relevant when: selected(${q102_E9_full_list} , '3')*	
q402_E9a *(required)*	q402_E9a. What percent of patients require this item?<br>*Question relevant when: selected(${q102_E9_full_list} , '3')*<br>*Response constrained to: .>0 and .<=100 or .=999*	
q402_E9b *(required)*	q402_E9b. How many units of this item are required per patient (throughout the entire treatment provided for complication)?<br>*Smallest unit is one forceps.*<br>*Question relevant when: selected(${q102_E9_full_list} , '3')*<br>*Response constrained to: .>=1*	
q402_E10	q402_E10. Speculum, Bivalve/Cuscos (reusable)<br>*Question relevant when: selected(${q102_E10_full_list} , '3')*	
q402_E10a *(required)*	q402_E10a. What percent of patients require this item?<br>*Question relevant when: selected(${q102_E10_full_list} , '3')*<br>*Response constrained to: .>0 and .<=100 or .=999*	
q402_E10b *(required)*	q402_E10b. How many units of this item are required per patient (throughout the entire treatment provided for complication)?<br>*Smallest unit is one speculum.*<br>*Question relevant when: selected(${q102_E10_full_list} , '3')*<br>*Response constrained to: .>=1*	
q402_E11	q402_E11. Speculum, Sim's (reusable)<br>*Question relevant when: selected(${q102_E11_full_list} , '3')*	
q402_E11a *(required)*	q402_E11a. What percent of patients require this item?<br>*Question relevant when: selected(${q102_E11_full_list} , '3')*<br>*Response constrained to: .>0 and .<=100 or .=999*	
q402_E11b *(required)*	q402_E11b. How many units of this item are required per patient (throughout the entire treatment provided for complication)?<br>*Smallest unit is one speculum.*<br>*Question relevant when: selected(${q102_E11_full_list} , '3')*<br>*Response constrained to: .>=1*	
q402_E12	q402_E12. Tenaculum<br>*Question relevant when: selected(${q102_E12_full_list} , '3')*	
q402_E12a *(required)*	q402_E12a. What percent of patients require this item?<br>*Question relevant when: selected(${q102_E12_full_list} , '3')*<br>*Response constrained to: .>0 and .<=100 or .=999*	
q402_E12b *(required)*	q402_E12b. How many units of this item are required per patient (throughout the entire treatment provided for complication)?<br>*Smallest unit is one tenaculum.*<br>*Question relevant when: selected(${q102_E12_full_list} , '3')*<br>*Response constrained to: .>=1*	
B. Small equipment - Shock (3)<br>*Group relevant when: selected(${section_four_skip_equ} , '1')*		
note_402_linens	***Linens***	
q402_E13	q402_E13. Towel<br>*Question relevant when: selected(${q102_E13_full_list} , '3')*	
q402_E13a *(required)*	q402_E13a. What percent of patients require this item?<br>*Question relevant when: selected(${q102_E13_full_list} , '3')*<br>*Response constrained to: .>0 and .<=100 or .=999*	
q402_E13b *(required)*	q402_E13b. How many units of this item are required per patient (throughout the entire treatment provided for complication)?<br>*Smallest unit is one towel.*<br>*Question relevant when: selected(${q102_E13_full_list} , '3')*<br>*Response constrained to: .>=1*	
q402_E14	q402_E14. Sheet<br>*Question relevant when: selected(${q102_E14_full_list} , '3')*	

Field	Question	Answer
q402_E14a *(required)*	q402_E14a. What percent of patients require this item?<br>*Question relevant when: selected(${q102_E14_full_list} , '3')*<br>*Response constrained to: .>0 and .<=100 or .=999*	
q402_E14b *(required)*	q402_E14b. How many units of this item are required per patient (throughout the entire treatment provided for complication)?<br>*Smallest unit is one sheet.*<br>*Question relevant when: selected(${q102_E14_full_list} , '3')*<br>*Response constrained to: .>=1*	
q402_E15	q402_E15. Blanket<br>*Question relevant when: selected(${q102_E15_full_list} , '3')*	
q402_E15a *(required)*	q402_E15a. What percent of patients require this item?<br>*Question relevant when: selected(${q102_E15_full_list} , '3')*<br>*Response constrained to: .>0 and .<=100 or .=999*	
q402_E15b *(required)*	q402_E15b. How many units of this item are required per patient (throughout the entire treatment provided for complication)?<br>*Smallest unit is one blanket.*<br>*Question relevant when: selected(${q102_E15_full_list} , '3')*<br>*Response constrained to: .>=1*	
q402_E16	q402_E16. Pillow<br>*Question relevant when: selected(${q102_E16_full_list} , '3')*	
q402_E16a *(required)*	q402_E16a. What percent of patients require this item?<br>*Question relevant when: selected(${q102_E16_full_list} , '3')*<br>*Response constrained to: .>0 and .<=100 or .=999*	
q402_E16b *(required)*	q402_E16b. How many units of this item are required per patient (throughout the entire treatment provided for complication)?<br>*Smallest unit is one pillow.*<br>*Question relevant when: selected(${q102_E16_full_list} , '3')*<br>*Response constrained to: .>=1*	
B. Small equipment - Shock (4)<br>*Group relevant when: selected(${section_four_skip_equ} , '1')*		
note_402_protective	***Protective supplies***	
q402_E17	q402_E17. Apron (reusable)<br>*Question relevant when: selected(${q102_E17_full_list} , '3')*	
q402_E17a *(required)*	q402_E17a. What percent of patients require this item?<br>*Question relevant when: selected(${q102_E17_full_list} , '3')*<br>*Response constrained to: .>0 and .<=100 or .=999*	
q402_E17b *(required)*	q402_E17b. How many units of this item are required per patient (throughout the entire treatment provided for complication)?<br>*Smallest unit is one apron.*<br>*Question relevant when: selected(${q102_E17_full_list} , '3')*<br>*Response constrained to: .>=1*	
q402_E18	q402_E18. Gown (reusable)<br>*Question relevant when: selected(${q102_E18_full_list} , '3')*	
q402_E18a *(required)*	q402_E18a. What percent of patients require this item?<br>*Question relevant when: selected(${q102_E18_full_list} , '3')*<br>*Response constrained to: .>0 and .<=100 or .=999*	
q402_E18b *(required)*	q402_E18b. How many units of this item are required per patient (throughout the entire treatment provided for complication)?<br>*Smallest unit is one gown.*<br>*Question relevant when: selected(${q102_E18_full_list} , '3')*<br>*Response constrained to: .>=1*	
q402_E19	q402_E19. Heavy duty gloves<br>*Question relevant when: selected(${q102_E19_full_list} , '3')*	
q402_E19a *(required)*	q402_E19a. What percent of patients require this item?<br>*Question relevant when: selected(${q102_E19_full_list} , '3')*<br>*Response constrained to: .>0 and .<=100 or .=999*	
q402_E19b *(required)*	q402_E19b. How many units of this item are required per patient (throughout the entire treatment provided for complication)?<br>*Smallest unit is one glove.*<br>*Question relevant when: selected(${q102_E19_full_list} , '3')*<br>*Response constrained to: .>=1*	
q402_E20	q402_E20. Mask (reusable)<br>*Question relevant when: selected(${q102_E20_full_list} , '3')*	

Field	Question	Answer
q402_E20a *(required)*	q402_E20a. What percent of patients require this item?<br>*Question relevant when: selected(${q102_E20_full_list} , '3')*<br>*Response constrained to: .>0 and .<=100 or .=999*	
q402_E20b *(required)*	q402_E20b. How many units of this item are required per patient (throughout the entire treatment provided for complication)?<br>*Smallest unit is one mask.*<br>*Question relevant when: selected(${q102_E20_full_list} , '3')*<br>*Response constrained to: .>=1*	
q402_E21	q402_E21. Protective glasses<br>*Question relevant when: selected(${q102_E21_full_list} , '3')*	
q402_E21a *(required)*	q402_E21a. What percent of patients require this item?<br>*Question relevant when: selected(${q102_E21_full_list} , '3')*<br>*Response constrained to: .>0 and .<=100 or .=999*	
q402_E21b *(required)*	q402_E21b. How many units of this item are required per patient (throughout the entire treatment provided for complication)?<br>*Smallest unit is one pair of glasses.*<br>*Question relevant when: selected(${q102_E21_full_list} , '3')*<br>*Response constrained to: .>=1*	
B. Small equipment - Shock (5)<br>*Group relevant when: selected(${section_four_skip_equ} , '1')*		
note_402_receivers	***Receivers***	
q402_E22	q402_E22. Kidney dish - small<br>*Question relevant when: selected(${q102_E22_full_list} , '3')*	
q402_E22a *(required)*	q402_E22a. What percent of patients require this item?<br>*Question relevant when: selected(${q102_E22_full_list} , '3')*<br>*Response constrained to: .>0 and .<=100 or .=999*	
q402_E22b *(required)*	q402_E22b. How many units of this item are required per patient (throughout the entire treatment provided for complication)?<br>*Smallest unit is one dish.*<br>*Question relevant when: selected(${q102_E22_full_list} , '3')*<br>*Response constrained to: .>=1*	
q402_E23	q402_E23. Kidney dish - medium<br>*Question relevant when: selected(${q102_E23_full_list} , '3')*	
q402_E23a *(required)*	q402_E23a. What percent of patients require this item?<br>*Question relevant when: selected(${q102_E23_full_list} , '3')*<br>*Response constrained to: .>0 and .<=100 or .=999*	
q402_E23b *(required)*	q402_E23b. How many units of this item are required per patient (throughout the entire treatment provided for complication)?<br>*Smallest unit is one dish.*<br>*Question relevant when: selected(${q102_E23_full_list} , '3')*<br>*Response constrained to: .>=1*	
q402_E24	q402_E24. Kidney dish - large<br>*Question relevant when: selected(${q102_E24_full_list} , '3')*	
q402_E24a *(required)*	q402_E24a. What percent of patients require this item?<br>*Question relevant when: selected(${q102_E24_full_list} , '3')*<br>*Response constrained to: .>0 and .<=100 or .=999*	
q402_E24b *(required)*	q402_E24b. How many units of this item are required per patient (throughout the entire treatment provided for complication)?<br>*Smallest unit is one dish.*<br>*Question relevant when: selected(${q102_E24_full_list} , '3')*<br>*Response constrained to: .>=1*	
q402_E25	q402_E25. Receiving bowl - small<br>*Question relevant when: selected(${q102_E25_full_list} , '3')*	
q402_E25a *(required)*	q402_E25a. What percent of patients require this item?<br>*Question relevant when: selected(${q102_E25_full_list} , '3')*<br>*Response constrained to: .>0 and .<=100 or .=999*	
q402_E25b *(required)*	q402_E25b. How many units of this item are required per patient (throughout the entire treatment provided for complication)?<br>*Smallest unit is one dish.*<br>*Question relevant when: selected(${q102_E25_full_list} , '3')*<br>*Response constrained to: .>=1*	
q402_E26	q402_E26. Receiving bowl - medium<br>*Question relevant when: selected(${q102_E26_full_list} , '3')*	

Field	Question	Answer
q402_E26a *(required)*	q402_E26a. What percent of patients require this item?<br>*Question relevant when: selected(${q102_E26_full_list} , '3')*<br>*Response constrained to: .>0 and .<=100 or .=999*	
q402_E26b *(required)*	q402_E26b. How many units of this item are required per patient (throughout the entire treatment provided for complication)?<br>*Smallest unit is one dish.*<br>*Question relevant when: selected(${q102_E26_full_list} , '3')*<br>*Response constrained to: .>=1*	
q402_E27	q402_E27. Receiving bowl - large<br>*Question relevant when: selected(${q102_E27_full_list} , '3')*	
q402_E27a *(required)*	q402_E27a. What percent of patients require this item?<br>*Question relevant when: selected(${q102_E27_full_list} , '3')*<br>*Response constrained to: .>0 and .<=100 or .=999*	
q402_E27b *(required)*	q402_E27b. How many units of this item are required per patient (throughout the entire treatment provided for complication)?<br>*Smallest unit is one dish.*<br>*Question relevant when: selected(${q102_E27_full_list} , '3')*<br>*Response constrained to: .>=1*	
B. Small equipment - Shock (6)<br>*Group relevant when: selected(${section_four_skip_equ} , '1')*		
note_402_surgery	***Surgery***	
q402_E28	q402_E28. Scalpel, reusable<br>*Question relevant when: selected(${q102_E28_full_list} , '3')*	
q402_E28a *(required)*	q402_E28a. What percent of patients require this item?<br>*Question relevant when: selected(${q102_E28_full_list} , '3')*<br>*Response constrained to: .>0 and .<=100 or .=999*	
q402_E28b *(required)*	q402_E28b. How many units of this item are required per patient (throughout the entire treatment provided for complication)?<br>*Smallest unit is one scalpel.*<br>*Question relevant when: selected(${q102_E28_full_list} , '3')*<br>*Response constrained to: .>=1*	
q402_E29	q402_E29. Surgical scissors<br>*Question relevant when: selected(${q102_E29_full_list} , '3')*	
q402_E29a *(required)*	q402_E29a. What percent of patients require this item?<br>*Question relevant when: selected(${q102_E29_full_list} , '3')*<br>*Response constrained to: .>0 and .<=100 or .=999*	
q402_E29b *(required)*	q402_E29b. How many units of this item are required per patient (throughout the entire treatment provided for complication)?<br>*Smallest unit is one pair of scissors.*<br>*Question relevant when: selected(${q102_E29_full_list} , '3')*<br>*Response constrained to: .>=1*	
B. Small equipment - Shock (7)<br>*Group relevant when: selected(${section_four_skip_equ} , '1')*		
note_402_other	***Other small equipment***	
q402_E30	q402_E30. Head lamp/batteries<br>*Question relevant when: selected(${q102_E30_full_list} , '3')*	
q402_E30a *(required)*	q402_E30a. What percent of patients require this item?<br>*Question relevant when: selected(${q102_E30_full_list} , '3')*<br>*Response constrained to: .>0 and .<=100 or .=999*	
q402_E30b *(required)*	q402_E30b. How many units of this item are required per patient (throughout the entire treatment provided for complication)?<br>*Smallest unit is one lamp/battery set.*<br>*Question relevant when: selected(${q102_E30_full_list} , '3')*<br>*Response constrained to: .>=1*	
q402_E31	q402_E31. Metal catheter<br>*Question relevant when: selected(${q102_E31_full_list} , '3')*	
q402_E31a *(required)*	q402_E31a. What percent of patients require this item?<br>*Question relevant when: selected(${q102_E31_full_list} , '3')*<br>*Response constrained to: .>0 and .<=100 or .=999*	
q402_E31b *(required)*	q402_E31b. How many units of this item are required per patient (throughout the entire treatment provided for complication)?<br>*Smallest unit is one catheter.*<br>*Question relevant when: selected(${q102_E31_full_list} , '3')*	

Field	Question	Answer
	Response constrained to: .>=1	
q402_E32	q402_E32. Urine collection flask<br>*Question relevant when: selected(${q102_E32_full_list} , '3')*	
q402_E32a *(required)*	q402_E32a. What percent of patients require this item?<br>*Question relevant when: selected(${q102_E32_full_list} , '3')*<br>*Response constrained to: .>0 and .<=100 or .=999*	
q402_E32b *(required)*	q402_E32b. How many units of this item are required per patient (throughout the entire treatment provided for complication)?<br>*Smallest unit is one flask.*<br>*Question relevant when: selected(${q102_E32_full_list} , '3')*<br>*Response constrained to: .>=1*	
B. Small equipment - Shock (8)<br>*Group relevant when: selected(${section_four_skip_equ} , '1')*		
note_402_other_suggestion	***Other equipment - suggestions?***	
q402_E33	q402_E33. Equipment other 1: "[q102_E33_full_list_other]"<br>*Question relevant when: selected(${q102_E33_full_list} , '3')*	
q402_E33a *(required)*	q402_E33a. What percent of patients require this item?<br>*Question relevant when: selected(${q102_E33_full_list} , '3')*<br>*Response constrained to: .>0 and .<=100 or .=999*	
q402_E33b *(required)*	q402_E33b. How many units of this item are required per patient (throughout the entire treatment provided for complication)?<br>*Question relevant when: selected(${q102_E33_full_list} , '3')*<br>*Response constrained to: .>=1*	
q402_E34	q402_E34. Equipment other 2: "[q102_E34_full_list_other]"<br>*Question relevant when: selected(${q102_E34_full_list} , '3')*	
q402_E34a *(required)*	q402_E34a. What percent of patients require this item?<br>*Question relevant when: selected(${q102_E34_full_list} , '3')*<br>*Response constrained to: .>0 and .<=100 or .=999*	
q402_E34b *(required)*	q402_E34b. How many units of this item are required per patient (throughout the entire treatment provided for complication)?<br>*Question relevant when: selected(${q102_E34_full_list} , '3')*<br>*Response constrained to: .>=1*	
q402_E35	q402_E35. Equipment other 3: "[q102_E35_full_list_other]"<br>*Question relevant when: selected(${q102_E35_full_list} , '3')*	
q402_E35a *(required)*	q402_E35a. What percent of patients require this item?<br>*Question relevant when: selected(${q102_E35_full_list} , '3')*<br>*Response constrained to: .>0 and .<=100 or .=999*	
q402_E35b *(required)*	q402_E35b. How many units of this item are required per patient (throughout the entire treatment provided for complication)?<br>*Question relevant when: selected(${q102_E35_full_list} , '3')*<br>*Response constrained to: .>=1*	
q402_E36	q402_E36. Equipment other 4: "[q102_E36_full_list_other]"<br>*Question relevant when: selected(${q102_E36_full_list} , '3')*	
q402_E36a *(required)*	q402_E36a. What percent of patients require this item?<br>*Question relevant when: selected(${q102_E36_full_list} , '3')*<br>*Response constrained to: .>0 and .<=100 or .=999*	
q402_E36b *(required)*	q402_E36b. How many units of this item are required per patient (throughout the entire treatment provided for complication)?<br>*Question relevant when: selected(${q102_E36_full_list} , '3')*<br>*Response constrained to: .>=1*	
q402_E37	q402_E37. Equipment other 5: "[q102_E37_full_list_other]"<br>*Question relevant when: selected(${q102_E37_full_list} , '3')*	
q402_E37a *(required)*	q402_E37a. What percent of patients require this item?<br>*Question relevant when: selected(${q102_E37_full_list} , '3')*<br>*Response constrained to: .>0 and .<=100 or .=999*	
q402_E37b *(required)*	q402_E37b. How many units of this item are required per patient (throughout the entire treatment provided for complication)?<br>*Question relevant when: selected(${q102_E37_full_list} , '3')*<br>*Response constrained to: .>=1*	
group_section_five_intro		
section5_start	SECTION V. LACERATIONS - USAGE OF ALL ITEMS	

section5_start	SECTION V. LACERATIONS - USAGE OF ALL ITEMS	
**Field**	**Question**	**Answer**
section_five_skip_equ	INTERVIEWER: WOULD YOU LIKE TO COMPLETE THIS SECTION NOW OR SKIP THIS SECTION AND RETURN TO IT LATER?<br>*You may need to skip if the participant has indicated that s/he cannot answer the questions in this section.*	1 Do not skip, complete this section now.<br>2 Skip and come back to this section later.
group_section_five_introB<br>*Group relevant when: selected(${section_five_skip_equ} , '1')*		
section5_start2	In this section of the interview, we will review all of the items that you said are used for management of cervical or vaginal lacerations. For each item that is used, I'm going to ask questions on how many women need it and how much of it is used.	
section5_start3	INTERVIEWER: ENTER WHOLE NUMBERS OR DECIMALS. DO NOT TYPE PERCENT SIGNS. ENTER 999 FOR ANY THAT ARE UNKNOWN.	
B. Small equipment - Lacerations (1)<br>*Group relevant when: selected(${section_five_skip_equ} , '1')*		
note_502_evac	***Evacuation***	
q502_E1	q502_E1. MVA aspirator<br>*Question relevant when: selected(${q102_E1_full_list} , '4')*	
q502_E1a *(required)*	q502_E1a. What percent of patients require this item?<br>*Question relevant when: selected(${q102_E1_full_list} , '4')*<br>*Response constrained to: .>0 and .<=100 or .=999*	
q502_E1b *(required)*	q502_E1b. How many units of this item are required per patient (throughout the entire treatment provided for complication)?<br>*Smallest unit is one aspirator.*<br>*Question relevant when: selected(${q102_E1_full_list} , '4')*<br>*Response constrained to: .>=1*	
q502_E2	q502_E2. Cannula set<br>*Question relevant when: selected(${q102_E2_full_list} , '4')*	
q502_E2a *(required)*	q502_E2a. What percent of patients require this item?<br>*Question relevant when: selected(${q102_E2_full_list} , '4')*<br>*Response constrained to: .>0 and .<=100 or .=999*	
q502_E2b *(required)*	q502_E2b. How many units of this item are required per patient (throughout the entire treatment provided for complication)?<br>*Smallest unit is one set.*<br>*Question relevant when: selected(${q102_E2_full_list} , '4')*<br>*Response constrained to: .>=1*	
q502_E3	q502_E3. Uterine curette set - plastic<br>*Question relevant when: selected(${q102_E3_full_list} , '4')*	
q502_E3a *(required)*	q502_E3a. What percent of patients require this item?<br>*Question relevant when: selected(${q102_E3_full_list} , '4')*<br>*Response constrained to: .>0 and .<=100 or .=999*	
q502_E3b *(required)*	q502_E3b. How many units of this item are required per patient (throughout the entire treatment provided for complication)?<br>*Smallest unit is one set.*<br>*Question relevant when: selected(${q102_E3_full_list} , '4')*<br>*Response constrained to: .>=1*	
q502_E4	q502_E4. Uterine currette set - metal<br>*Question relevant when: selected(${q102_E4_full_list} , '4')*	
q502_E4a *(required)*	q502_E4a. What percent of patients require this item?<br>*Question relevant when: selected(${q102_E4_full_list} , '4')*<br>*Response constrained to: .>0 and .<=100 or .=999*	
q502_E4b *(required)*	q502_E4b. How many units of this item are required per patient (throughout the entire treatment provided for complication)?<br>*Smallest unit is one set.*<br>*Question relevant when: selected(${q102_E4_full_list} , '4')*<br>*Response constrained to: .>=1*	
q502_E5	q502_E5. Dilators (Hegar's Cervical dilators)<br>*Question relevant when: selected(${q102_E5_full_list} , '4')*	
q502_E5a *(required)*	q502_E5a. What percent of patients require this item?<br>*Question relevant when: selected(${q102_E5_full_list} , '4')*<br>*Response constrained to: .>0 and .<=100 or .=999*	
q502_E5b *(required)*	q502_E5b. How many units of this item are required per patient (throughout the entire treatment provided for	

Field	Question	Answer
	complication)?<br>*Smallest unit is one set.*<br>*Question relevant when: selected(${q102_E5_full_list} , '4')*<br>*Response constrained to: .>=1*	

B. Small equipment - Lacerations (2)<br>*Group relevant when: selected(${section_five_skip_equ} , '1')*		
note_502_forceps	***Forceps, speculums, dilators, etc.***	
q502_E6	q502_E6. Forceps, artery/Kocher<br>*Question relevant when: selected(${q102_E6_full_list} , '4')*	
q502_E6a *(required)*	q502_E6a. What percent of patients require this item?<br>*Question relevant when: selected(${q102_E6_full_list} , '4')*<br>*Response constrained to: .>0 and .<=100 or .=999*	
q502_E6b *(required)*	q502_E6b. How many units of this item are required per patient (throughout the entire treatment provided for complication)?<br>*Smallest unit is one forceps.*<br>*Question relevant when: selected(${q102_E6_full_list} , '4')*<br>*Response constrained to: .>=1*	
q502_E7	q502_E7. Needle holder<br>*Question relevant when: selected(${q102_E7_full_list} , '4')*	
q502_E7a *(required)*	q502_E7a. What percent of patients require this item?<br>*Question relevant when: selected(${q102_E7_full_list} , '4')*<br>*Response constrained to: .>0 and .<=100 or .=999*	
q502_E7b *(required)*	q502_E7b. How many units of this item are required per patient (throughout the entire treatment provided for complication)?<br>*Smallest unit is one holder.*<br>*Question relevant when: selected(${q102_E7_full_list} , '4')*<br>*Response constrained to: .>=1*	
q502_E8	q502_E8. Ovum forceps<br>*Question relevant when: selected(${q102_E8_full_list} , '4')*	
q502_E8a *(required)*	q502_E8a. What percent of patients require this item?<br>*Question relevant when: selected(${q102_E8_full_list} , '4')*<br>*Response constrained to: .>0 and .<=100 or .=999*	
q502_E8b *(required)*	q502_E8b. How many units of this item are required per patient (throughout the entire treatment provided for complication)?<br>*Smallest unit is one forceps.*<br>*Question relevant when: selected(${q102_E8_full_list} , '4')*<br>*Response constrained to: .>=1*	
q502_E9	q502_E9. Ring forceps<br>*Question relevant when: selected(${q102_E9_full_list} , '4')*	
q502_E9a *(required)*	q502_E9a. What percent of patients require this item?<br>*Question relevant when: selected(${q102_E9_full_list} , '4')*<br>*Response constrained to: .>0 and .<=100 or .=999*	
q502_E9b *(required)*	q502_E9b. How many units of this item are required per patient (throughout the entire treatment provided for complication)?<br>*Smallest unit is one forceps.*<br>*Question relevant when: selected(${q102_E9_full_list} , '4')*<br>*Response constrained to: .>=1*	
q502_E10	q502_E10. Speculum, BValve/Cuscos (reusable)<br>*Question relevant when: selected(${q102_E10_full_list} , '4')*	
q502_E10a *(required)*	q502_E10a. What percent of patients require this item?<br>*Question relevant when: selected(${q102_E10_full_list} , '4')*<br>*Response constrained to: .>0 and .<=100 or .=999*	
q502_E10b *(required)*	q502_E10b. How many units of this item are required per patient (throughout the entire treatment provided for complication)?<br>*Smallest unit is one speculum.*<br>*Question relevant when: selected(${q102_E10_full_list} , '4')*<br>*Response constrained to: .>=1*	
q502_E11	q502_E11. Speculum, Sim's (reusable)<br>*Question relevant when: selected(${q102_E11_full_list} , '4')*	
q502_E11a *(required)*	q502_E11a. What percent of patients require this item?<br>*Question relevant when: selected(${q102_E11_full_list} , '4')*<br>*Response constrained to: .>0 and .<=100 or .=999*	
q502_E11b *(required)*	q502_E11b. How many units of this item are required per patient (throughout the entire treatment provided for complication)?	

Field	Question	Answer
	Smallest unit is one speculum.<br>*Question relevant when: selected(${q102_E11_full_list} , '4')*<br>*Response constrained to: .>=1*	
q502_E12	q502_E12. Tenaculum<br>*Question relevant when: selected(${q102_E12_full_list} , '4')*	

q502_E12a *(required)*	q502_E12a. What percent of patients require this item?<br>*Question relevant when: selected(${q102_E12_full_list} , '4')*<br>*Response constrained to: .>0 and .<=100 or .=999*	
q502_E12b *(required)*	q502_E12b. How many units of this item are required per patient (throughout the entire treatment provided for complication)?<br>*Smallest unit is one tenaculum.*<br>*Question relevant when: selected(${q102_E12_full_list} , '4')*<br>*Response constrained to: .>=1*	
B. Small equipment - Lacerations (3)<br>*Group relevant when: selected(${section_five_skip_equ} , '1')*		
note_502_linens	***Linens***	
q502_E13	q502_E13. Towel<br>*Question relevant when: selected(${q102_E13_full_list} , '4')*	
q502_E13a *(required)*	q502_E13a. What percent of patients require this item?<br>*Question relevant when: selected(${q102_E13_full_list} , '4')*<br>*Response constrained to: .>0 and .<=100 or .=999*	
q502_E13b *(required)*	q502_E13b. How many units of this item are required per patient (throughout the entire treatment provided for complication)?<br>*Smallest unit is one towel.*<br>*Question relevant when: selected(${q102_E13_full_list} , '4')*<br>*Response constrained to: .>=1*	
q502_E14	q502_E14. Sheet<br>*Question relevant when: selected(${q102_E14_full_list} , '4')*	
q502_E14a *(required)*	q502_E14a. What percent of patients require this item?<br>*Question relevant when: selected(${q102_E14_full_list} , '4')*<br>*Response constrained to: .>0 and .<=100 or .=999*	
q502_E14b *(required)*	q502_E14b. How many units of this item are required per patient (throughout the entire treatment provided for complication)?<br>*Smallest unit is one sheet.*<br>*Question relevant when: selected(${q102_E14_full_list} , '4')*<br>*Response constrained to: .>=1*	
q502_E15	q502_E15. Blanket<br>*Question relevant when: selected(${q102_E15_full_list} , '4')*	
q502_E15a *(required)*	q502_E15a. What percent of patients require this item?<br>*Question relevant when: selected(${q102_E15_full_list} , '4')*<br>*Response constrained to: .>0 and .<=100 or .=999*	
q502_E15b *(required)*	q502_E15b. How many units of this item are required per patient (throughout the entire treatment provided for complication)?<br>*Smallest unit is one blanket.*<br>*Question relevant when: selected(${q102_E15_full_list} , '4')*<br>*Response constrained to: .>=1*	
q502_E16	q502_E16. Pillow<br>*Question relevant when: selected(${q102_E16_full_list} , '4')*	
q502_E16a *(required)*	q502_E16a. What percent of patients require this item?<br>*Question relevant when: selected(${q102_E16_full_list} , '4')*<br>*Response constrained to: .>0 and .<=100 or .=999*	
q502_E16b *(required)*	q502_E16b. How many units of this item are required per patient (throughout the entire treatment provided for complication)?<br>*Smallest unit is one pillow.*<br>*Question relevant when: selected(${q102_E16_full_list} , '4')*<br>*Response constrained to: .>=1*	
B. Small equipment - Lacerations (4)<br>*Group relevant when: selected(${section_five_skip_equ} , '1')*		
note_502_protective	***Protective supplies***	
q502_E17	q502_E17. Apron (reusable)<br>*Question relevant when: selected(${q102_E17_full_list} , '4')*	
q502_E17a *(required)*	q502_E17a. What percent of patients require this item?<br>*Question relevant when: selected(${q102_E17_full_list} , '4')*<br>*Response constrained to: .>0 and .<=100 or .=999*	
q502_E17b *(required)*	q502_E17b. How many units of this item are required per patient (throughout the entire treatment provided for	

Field	Question	Answer
	complication)?<br>*Smallest unit is one apron.*<br>*Question relevant when: selected(${q102_E17_full_list} , '4')*<br>*Response constrained to: .>=1*	

Field	Question	Answer
q502_E18	q502_E18. Gown (reusable)<br>*Question relevant when: selected(${q102_E18_full_list} , '4')*	
q502_E18a *(required)*	q502_E18a. What percent of patients require this item?<br>*Question relevant when: selected(${q102_E18_full_list} , '4')*<br>*Response constrained to: .>0 and .<=100 or .=999*	
q502_E18b *(required)*	q502_E18b. How many units of this item are required per patient (throughout the entire treatment provided for complication)?<br>*Smallest unit is one gown.*<br>*Question relevant when: selected(${q102_E18_full_list} , '4')*<br>*Response constrained to: .>=1*	
q502_E19	q502_E19. Heavy duty gloves<br>*Question relevant when: selected(${q102_E19_full_list} , '4')*	
q502_E19a *(required)*	q502_E19a. What percent of patients require this item?<br>*Question relevant when: selected(${q102_E19_full_list} , '4')*<br>*Response constrained to: .>0 and .<=100 or .=999*	
q502_E19b *(required)*	q502_E19b. How many units of this item are required per patient (throughout the entire treatment provided for complication)?<br>*Smallest unit is one glove.*<br>*Question relevant when: selected(${q102_E19_full_list} , '4')*<br>*Response constrained to: .>=1*	
q502_E20	q502_E20. Mask (reusable)<br>*Question relevant when: selected(${q102_E20_full_list} , '4')*	
q502_E20a *(required)*	q502_E20a. What percent of patients require this item?<br>*Question relevant when: selected(${q102_E20_full_list} , '4')*<br>*Response constrained to: .>0 and .<=100 or .=999*	
q502_E20b *(required)*	q502_E20b. How many units of this item are required per patient (throughout the entire treatment provided for complication)?<br>*Smallest unit is one mask.*<br>*Question relevant when: selected(${q102_E20_full_list} , '4')*<br>*Response constrained to: .>=1*	
q502_E21	q502_E21. Protective glasses<br>*Question relevant when: selected(${q102_E21_full_list} , '4')*	
q502_E21a *(required)*	q502_E21a. What percent of patients require this item?<br>*Question relevant when: selected(${q102_E21_full_list} , '4')*<br>*Response constrained to: .>0 and .<=100 or .=999*	
q502_E21b *(required)*	q502_E21b. How many units of this item are required per patient (throughout the entire treatment provided for complication)?<br>*Smallest unit is one pair of glasses.*<br>*Question relevant when: selected(${q102_E21_full_list} , '4')*<br>*Response constrained to: .>=1*	
B. Small equipment - Lacerations (5)<br>*Group relevant when: selected(${section_five_skip_equ} , '1')*		
note_502_receivers	***Receivers***	
q502_E22	q502_E22. Kidney dish - small<br>*Question relevant when: selected(${q102_E22_full_list} , '4')*	
q502_E22a *(required)*	q502_E22a. What percent of patients require this item?<br>*Question relevant when: selected(${q102_E22_full_list} , '4')*<br>*Response constrained to: .>0 and .<=100 or .=999*	
q502_E22b *(required)*	q502_E22b. How many units of this item are required per patient (throughout the entire treatment provided for complication)?<br>*Smallest unit is one dish.*<br>*Question relevant when: selected(${q102_E22_full_list} , '4')*<br>*Response constrained to: .>=1*	
q502_E23	q502_E23. Kidney dish - medium<br>*Question relevant when: selected(${q102_E23_full_list} , '4')*	
q502_E23a *(required)*	q502_E23a. What percent of patients require this item?<br>*Question relevant when: selected(${q102_E23_full_list} , '4')*<br>*Response constrained to: .>0 and .<=100 or .=999*	
q502_E23b *(required)*	q502_E23b. How many units of this item are required per patient (throughout the entire treatment provided for complication)?	

Field	Question	Answer
	Smallest unit is one dish.<br>*Question relevant when: selected(${q102_E23_full_list} , '4')*<br>*Response constrained to: .>=1*	
q502_E24	q502_E24. Kidney dish - large<br>*Question relevant when: selected(${q102_E24_full_list} , '4')*	

q502_E24a *(required)*	q502_E24a. What percent of patients require this item?<br>*Question relevant when: selected(${q102_E24_full_list} , '4')*<br>*Response constrained to: .>0 and .<=100 or .=999*	
q502_E24b *(required)*	q502_E24b. How many units of this item are required per patient (throughout the entire treatment provided for complication)?<br>*Smallest unit is one dish.*<br>*Question relevant when: selected(${q102_E24_full_list} , '4')*<br>*Response constrained to: .>=1*	
q502_E25	q502_E25. Receiving bowl - small<br>*Question relevant when: selected(${q102_E25_full_list} , '4')*	
q502_E25a *(required)*	q502_E25a. What percent of patients require this item?<br>*Question relevant when: selected(${q102_E25_full_list} , '4')*<br>*Response constrained to: .>0 and .<=100 or .=999*	
q502_E25b *(required)*	q502_E25b. How many units of this item are required per patient (throughout the entire treatment provided for complication)?<br>*Smallest unit is one dish.*<br>*Question relevant when: selected(${q102_E25_full_list} , '4')*<br>*Response constrained to: .>=1*	
q502_E26	q502_E26. Receiving bowl - medium<br>*Question relevant when: selected(${q102_E26_full_list} , '4')*	
q502_E26a *(required)*	q502_E26a. What percent of patients require this item?<br>*Question relevant when: selected(${q102_E26_full_list} , '4')*<br>*Response constrained to: .>0 and .<=100 or .=999*	
q502_E26b *(required)*	q502_E26b. How many units of this item are required per patient (throughout the entire treatment provided for complication)?<br>*Smallest unit is one dish.*<br>*Question relevant when: selected(${q102_E26_full_list} , '4')*<br>*Response constrained to: .>=1*	
q502_E27	q502_E27. Receiving bowl - large<br>*Question relevant when: selected(${q102_E27_full_list} , '4')*	
q502_E27a *(required)*	q502_E27a. What percent of patients require this item?<br>*Question relevant when: selected(${q102_E27_full_list} , '4')*<br>*Response constrained to: .>0 and .<=100 or .=999*	
q502_E27b *(required)*	q502_E27b. How many units of this item are required per patient (throughout the entire treatment provided for complication)?<br>*Smallest unit is one dish.*<br>*Question relevant when: selected(${q102_E27_full_list} , '4')*<br>*Response constrained to: .>=1*	
B. Small equipment - Lacerations (6)<br>*Group relevant when: selected(${section_five_skip_equ} , '1')*		
note_502_surgery	***Surgery***	
q502_E28	q502_E28. Scalpel, reusable<br>*Question relevant when: selected(${q102_E28_full_list} , '4')*	
q502_E28a *(required)*	q502_E28a. What percent of patients require this item?<br>*Question relevant when: selected(${q102_E28_full_list} , '4')*<br>*Response constrained to: .>0 and .<=100 or .=999*	
q502_E28b *(required)*	q502_E28b. How many units of this item are required per patient (throughout the entire treatment provided for complication)?<br>*Smallest unit is one scalpel.*<br>*Question relevant when: selected(${q102_E28_full_list} , '4')*<br>*Response constrained to: .>=1*	
q502_E29	q502_E29. Surgical scissors<br>*Question relevant when: selected(${q102_E29_full_list} , '4')*	
q502_E29a *(required)*	q502_E29a. What percent of patients require this item?<br>*Question relevant when: selected(${q102_E29_full_list} , '4')*<br>*Response constrained to: .>0 and .<=100 or .=999*	
q502_E29b *(required)*	q502_E29b. How many units of this item are required per patient (throughout the entire treatment provided for complication)?<br>*Smallest unit is one pair of scissors.*<br>*Question relevant when: selected(${q102_E29_full_list} , '4')*	

Field	Question	Answer
	Response constrained to: .>=1	
B. Small equipment - Lacerations (7) *Group relevant when: selected(${section_five_skip_equ} , '1')*		
note_502_other	***Other small equipment***	

Field	Question	Answer
q502_E30	q502_E30. Head lamp/batteries<br>*Question relevant when: selected(${q102_E30_full_list} , '4')*	
q502_E30a *(required)*	q502_E30a. What percent of patients require this item?<br>*Question relevant when: selected(${q102_E30_full_list} , '4')*<br>*Response constrained to: .>0 and .<=100 or .=999*	
q502_E30b *(required)*	q502_E30b. How many units of this item are required per patient (throughout the entire treatment provided for complication)?<br>*Smallest unit is one lamp/battery set.*<br>*Question relevant when: selected(${q102_E30_full_list} , '4')*<br>*Response constrained to: .>=1*	
q502_E31	q502_E31. Metal catheter<br>*Question relevant when: selected(${q102_E31_full_list} , '4')*	
q502_E31a *(required)*	q502_E31a. What percent of patients require this item?<br>*Question relevant when: selected(${q102_E31_full_list} , '4')*<br>*Response constrained to: .>0 and .<=100 or .=999*	
q502_E31b *(required)*	q502_E31b. How many units of this item are required per patient (throughout the entire treatment provided for complication)?<br>*Smallest unit is one catheter.*<br>*Question relevant when: selected(${q102_E31_full_list} , '4')*<br>*Response constrained to: .>=1*	
q502_E32	q502_E32. Urine collection flask<br>*Question relevant when: selected(${q102_E32_full_list} , '4')*	
q502_E32a *(required)*	q502_E32a. What percent of patients require this item?<br>*Question relevant when: selected(${q102_E32_full_list} , '4')*<br>*Response constrained to: .>0 and .<=100 or .=999*	
q502_E32b *(required)*	q502_E32b. How many units of this item are required per patient (throughout the entire treatment provided for complication)?<br>*Smallest unit is one flask.*<br>*Question relevant when: selected(${q102_E32_full_list} , '4')*<br>*Response constrained to: .>=1*	
B. Small equipment - Lacerations (8) *Group relevant when: selected(${section_five_skip_equ} , '1')*		
note_502_other_suggestion	***Other equipment - suggestions?***	
q502_E33	q502_E33. Equipment other 1: "[q102_E33_full_list_other]"<br>*Question relevant when: selected(${q102_E33_full_list} , '4')*	
q502_E33a *(required)*	q502_E33a. What percent of patients require this item?<br>*Question relevant when: selected(${q102_E33_full_list} , '4')*<br>*Response constrained to: .>0 and .<=100 or .=999*	
q502_E33b *(required)*	q502_E33b. How many units of this item are required per patient (throughout the entire treatment provided for complication)?<br>*Question relevant when: selected(${q102_E33_full_list} , '4')*<br>*Response constrained to: .>=1*	
q502_E34	q502_E34. Equipment other 2: "[q102_E34_full_list_other]"<br>*Question relevant when: selected(${q102_E34_full_list} , '4')*	
q502_E34a *(required)*	q502_E34a. What percent of patients require this item?<br>*Question relevant when: selected(${q102_E34_full_list} , '4')*<br>*Response constrained to: .>0 and .<=100 or .=999*	
q502_E34b *(required)*	q502_E34b. How many units of this item are required per patient (throughout the entire treatment provided for complication)?<br>*Question relevant when: selected(${q102_E34_full_list} , '4')*<br>*Response constrained to: .>=1*	
q502_E35	q502_E35. Equipment other 3: "[q102_E35_full_list_other]"<br>*Question relevant when: selected(${q102_E35_full_list} , '4')*	
q502_E35a *(required)*	q502_E35a. What percent of patients require this item?<br>*Question relevant when: selected(${q102_E35_full_list} , '4')*<br>*Response constrained to: .>0 and .<=100 or .=999*	
q502_E35b *(required)*	q502_E35b. How many units of this item are required per patient (throughout the entire treatment provided for complication)?<br>*Question relevant when: selected(${q102_E35_full_list} , '4')*	

Field	Question	Answer
	Response constrained to: .>=1	
q502_E36	q502_E36. Equipment other 4: "[q102_E36_full_list_other]"<br>*Question relevant when: selected(${q102_E36_full_list} , '4')*	
q502_E36a *(required)*	q502_E36a. What percent of patients require this item?<br>*Question relevant when: selected(${q102_E36_full_list} , '4')*<br>*Response constrained to: .>0 and .<=100 or .=999*	
q502_E36b *(required)*	q502_E36b. How many units of this item are required per patient (throughout the entire treatment provided for complication)?<br>*Question relevant when: selected(${q102_E36_full_list} , '4')*<br>*Response constrained to: .>=1*	
q502_E37	q502_E37. Equipment other 5: "[q102_E37_full_list_other]"<br>*Question relevant when: selected(${q102_E37_full_list} , '4')*	
q502_E37a *(required)*	q502_E37a. What percent of patients require this item?<br>*Question relevant when: selected(${q102_E37_full_list} , '4')*<br>*Response constrained to: .>0 and .<=100 or .=999*	
q502_E37b *(required)*	q502_E37b. How many units of this item are required per patient (throughout the entire treatment provided for complication)?<br>*Question relevant when: selected(${q102_E37_full_list} , '4')*<br>*Response constrained to: .>=1*	
group_section_six_intro		
section6_start	**SECTION VI. PERFORATIONS - USAGE OF ALL ITEMS**	
section_six_skip_equ	INTERVIEWER: WOULD YOU LIKE TO COMPLETE THIS SECTION NOW OR SKIP THIS SECTION AND RETURN TO IT LATER?<br>*You may need to skip if the participant has indicated that s/he cannot answer the questions in this section.*	1 Do not skip, complete this section now.<br>2 Skip and come back to this section later.
group_section_six_introB<br>*Group relevant when: selected(${section_six_skip_equ} , '1')*		
section6_start2	In this section of the interview, we will review all of the items that you said are used for management of vaginal or uterine perforations. For each item that is used, I'm going to ask questions on how many women need it and how much of it is used.	
section6_start3	INTERVIEWER: ENTER WHOLE NUMBERS OR DECIMALS. DO NOT TYPE PERCENT SIGNS. ENTER 999 FOR ANY THAT ARE UNKNOWN.	
B. Small equipment - Perforations (1)<br>*Group relevant when: selected(${section_six_skip_equ} , '1')*		
note_602_evac	***Evacuation***	
q602_E1	q602_E1. MVA aspirator<br>*Question relevant when: selected(${q102_E1_full_list} , '5')*	
q602_E1a *(required)*	q602_E1a. What percent of patients require this item?<br>*Question relevant when: selected(${q102_E1_full_list} , '5')*<br>*Response constrained to: .>0 and .<=100 or .=999*	
q602_E1b *(required)*	q602_E1b. How many units of this item are required per patient (throughout the entire treatment provided for complication)?<br>*Smallest unit is one aspirator.*<br>*Question relevant when: selected(${q102_E1_full_list} , '5')*<br>*Response constrained to: .>=1*	
q602_E2	q602_E2. Cannula set<br>*Question relevant when: selected(${q102_E2_full_list} , '5')*	
q602_E2a *(required)*	q602_E2a. What percent of patients require this item?<br>*Question relevant when: selected(${q102_E2_full_list} , '5')*<br>*Response constrained to: .>0 and .<=100 or .=999*	
q602_E2b *(required)*	q602_E2b. How many units of this item are required per patient (throughout the entire treatment provided for complication)?<br>*Smallest unit is one set.*<br>*Question relevant when: selected(${q102_E2_full_list} , '5')*<br>*Response constrained to: .>=1*	
q602_E3	q602_E3. Uterine curette set - plastic<br>*Question relevant when: selected(${q102_E3_full_list} , '5')*	
q602_E3a *(required)*	q602_E3a. What percent of patients require this item?<br>*Question relevant when: selected(${q102_E3_full_list} , '5')*<br>*Response constrained to: .>0 and .<=100 or .=999*	
q602_E3b *(required)*	q602_E3b. How many units of this item are required per patient (throughout the entire treatment provided for complication)?	

Field	Question	Answer
	Smallest unit is one set.<br>*Question relevant when: selected(${q102_E3_full_list} , '5')*	
	Response constrained to: .>=1	
q602_E4	q602_E4. Uterine currette set - metal<br>*Question relevant when: selected(${q102_E4_full_list} , '5')*	
q602_E4a *(required)*	q602_E4a. What percent of patients require this item?<br>*Question relevant when: selected(${q102_E4_full_list} , '5')*<br>*Response constrained to: .>0 and .<=100 or .=999*	
q602_E4b *(required)*	q602_E4b. How many units of this item are required per patient (throughout the entire treatment provided for complication)?<br>*Smallest unit is one set.*<br>*Question relevant when: selected(${q102_E4_full_list} , '5')*<br>*Response constrained to: .>=1*	
q602_E5	q602_E5. Dilators (Hegar's Cervical dilators)<br>*Question relevant when: selected(${q102_E5_full_list} , '5')*	
q602_E5a *(required)*	q602_E5a. What percent of patients require this item?<br>*Question relevant when: selected(${q102_E5_full_list} , '5')*<br>*Response constrained to: .>0 and .<=100 or .=999*	
q602_E5b *(required)*	q602_E5b. How many units of this item are required per patient (throughout the entire treatment provided for complication)?<br>*Smallest unit is one set.*<br>*Question relevant when: selected(${q102_E5_full_list} , '5')*<br>*Response constrained to: .>=1*	
B. Small equipment - Perforations (2)<br>*Group relevant when: selected(${section_six_skip_equ} , '1')*		
note_602_forceps	***Forceps, speculums, dilators, etc.***	
q602_E6	q602_E6. Forceps, artery/Kocher<br>*Question relevant when: selected(${q102_E6_full_list} , '5')*	
q602_E6a *(required)*	q602_E6a. What percent of patients require this item?<br>*Question relevant when: selected(${q102_E6_full_list} , '5')*<br>*Response constrained to: .>0 and .<=100 or .=999*	
q602_E6b *(required)*	q602_E6b. How many units of this item are required per patient (throughout the entire treatment provided for complication)?<br>*Smallest unit is one forceps.*<br>*Question relevant when: selected(${q102_E6_full_list} , '5')*<br>*Response constrained to: .>=1*	
q602_E7	q602_E7. Needle holder<br>*Question relevant when: selected(${q102_E7_full_list} , '5')*	
q602_E7a *(required)*	q602_E7a. What percent of patients require this item?<br>*Question relevant when: selected(${q102_E7_full_list} , '5')*<br>*Response constrained to: .>0 and .<=100 or .=999*	
q602_E7b *(required)*	q602_E7b. How many units of this item are required per patient (throughout the entire treatment provided for complication)?<br>*Smallest unit is one holder.*<br>*Question relevant when: selected(${q102_E7_full_list} , '5')*<br>*Response constrained to: .>=1*	
q602_E8	q602_E8. Ovum forceps<br>*Question relevant when: selected(${q102_E8_full_list} , '5')*	
q602_E8a *(required)*	q602_E8a. What percent of patients require this item?<br>*Question relevant when: selected(${q102_E8_full_list} , '5')*<br>*Response constrained to: .>0 and .<=100 or .=999*	
q602_E8b *(required)*	q602_E8b. How many units of this item are required per patient (throughout the entire treatment provided for complication)?<br>*Smallest unit is one forceps.*<br>*Question relevant when: selected(${q102_E8_full_list} , '5')*<br>*Response constrained to: .>=1*	
q602_E9	q602_E9. Ring forceps<br>*Question relevant when: selected(${q102_E9_full_list} , '5')*	
q602_E9a *(required)*	q602_E9a. What percent of patients require this item?<br>*Question relevant when: selected(${q102_E9_full_list} , '5')*<br>*Response constrained to: .>0 and .<=100 or .=999*	
q602_E9b *(required)*	q602_E9b. How many units of this item are required per patient (throughout the entire treatment provided for complication)?<br>*Smallest unit is one forceps.*<br>*Question relevant when: selected(${q102_E9_full_list} , '5')*	

Field	Question	Answer
	Response constrained to: .>=1	
q602_E10	q602_E10. Speculum, BValve/Cuscos (reusable)<br>*Question relevant when: selected(${q102_E10_full_list} , '5')*	
q602_E10a *(required)*	q602_E10a. What percent of patients require this item?<br>*Question relevant when: selected(${q102_E10_full_list} , '5')*<br>*Response constrained to: .>0 and .<=100 or .=999*	
q602_E10b *(required)*	q602_E10b. How many units of this item are required per patient (throughout the entire treatment provided for complication)?<br>*Smallest unit is one speculum.*<br>*Question relevant when: selected(${q102_E10_full_list} , '5')*<br>*Response constrained to: .>=1*	
q602_E11	q602_E11. Speculum, Sim's (reusable)<br>*Question relevant when: selected(${q102_E11_full_list} , '5')*	
q602_E11a *(required)*	q602_E11a. What percent of patients require this item?<br>*Question relevant when: selected(${q102_E11_full_list} , '5')*<br>*Response constrained to: .>0 and .<=100 or .=999*	
q602_E11b *(required)*	q602_E11b. How many units of this item are required per patient (throughout the entire treatment provided for complication)?<br>*Smallest unit is one speculum.*<br>*Question relevant when: selected(${q102_E11_full_list} , '5')*<br>*Response constrained to: .>=1*	
q602_E12	q602_E12. Tenaculum<br>*Question relevant when: selected(${q102_E12_full_list} , '5')*	
q602_E12a *(required)*	q602_E12a. What percent of patients require this item?<br>*Question relevant when: selected(${q102_E12_full_list} , '5')*<br>*Response constrained to: .>0 and .<=100 or .=999*	
q602_E12b *(required)*	q602_E12b. How many units of this item are required per patient (throughout the entire treatment provided for complication)?<br>*Smallest unit is one tenaculum.*<br>*Question relevant when: selected(${q102_E12_full_list} , '5')*<br>*Response constrained to: .>=1*	
B. Small equipment - Perforations (3)<br>*Group relevant when: selected(${section_six_skip_equ} , '1')*		
note_602_linens	***Linens***	
q602_E13	q602_E13. Towel<br>*Question relevant when: selected(${q102_E13_full_list} , '5')*	
q602_E13a *(required)*	q602_E13a. What percent of patients require this item?<br>*Question relevant when: selected(${q102_E13_full_list} , '5')*<br>*Response constrained to: .>0 and .<=100 or .=999*	
q602_E13b *(required)*	q602_E13b. How many units of this item are required per patient (throughout the entire treatment provided for complication)?<br>*Smallest unit is one towel.*<br>*Question relevant when: selected(${q102_E13_full_list} , '5')*<br>*Response constrained to: .>=1*	
q602_E14	q602_E14. Sheet<br>*Question relevant when: selected(${q102_E14_full_list} , '5')*	
q602_E14a *(required)*	q602_E14a. What percent of patients require this item?<br>*Question relevant when: selected(${q102_E14_full_list} , '5')*<br>*Response constrained to: .>0 and .<=100 or .=999*	
q602_E14b *(required)*	q602_E14b. How many units of this item are required per patient (throughout the entire treatment provided for complication)?<br>*Smallest unit is one sheet.*<br>*Question relevant when: selected(${q102_E14_full_list} , '5')*<br>*Response constrained to: .>=1*	
q602_E15	q602_E15. Blanket<br>*Question relevant when: selected(${q102_E15_full_list} , '5')*	
q602_E15a *(required)*	q602_E15a. What percent of patients require this item?<br>*Question relevant when: selected(${q102_E15_full_list} , '5')*<br>*Response constrained to: .>0 and .<=100 or .=999*	
q602_E15b *(required)*	q602_E15b. How many units of this item are required per patient (throughout the entire treatment provided for complication)?<br>*Smallest unit is one blanket.*	

Field	Question	Answer
	Question relevant when: selected(${q102_E15_full_list} , '5')<br>*Response constrained to: .>=1*	
q602_E16	q602_E16. Pillow<br>*Question relevant when: selected(${q102_E16_full_list} , '5')*	
q602_E16a *(required)*	q602_E16a. What percent of patients require this item?<br>*Question relevant when: selected(${q102_E16_full_list} , '5')*<br>*Response constrained to: .>0 and .<=100 or .=999*	
q602_E16b *(required)*	q602_E16b. How many units of this item are required per patient (throughout the entire treatment provided for complication)?<br>*Smallest unit is one pillow.*<br>*Question relevant when: selected(${q102_E16_full_list} , '5')*<br>*Response constrained to: .>=1*	
B. Small equipment - Perforations (4)<br>*Group relevant when: selected(${section_six_skip_equ} , '1')*		
note_602_protective	***Protective supplies***	
q602_E17	q602_E17. Apron (reusable)<br>*Question relevant when: selected(${q102_E17_full_list} , '5')*	
q602_E17a *(required)*	q602_E17a. What percent of patients require this item?<br>*Question relevant when: selected(${q102_E17_full_list} , '5')*<br>*Response constrained to: .>0 and .<=100 or .=999*	
q602_E17b *(required)*	q602_E17b. How many units of this item are required per patient (throughout the entire treatment provided for complication)?<br>*Smallest unit is one apron.*<br>*Question relevant when: selected(${q102_E17_full_list} , '5')*<br>*Response constrained to: .>=1*	
q602_E18	q602_E18. Gown (reusable)<br>*Question relevant when: selected(${q102_E18_full_list} , '5')*	
q602_E18a *(required)*	q602_E18a. What percent of patients require this item?<br>*Question relevant when: selected(${q102_E18_full_list} , '5')*<br>*Response constrained to: .>0 and .<=100 or .=999*	
q602_E18b *(required)*	q602_E18b. How many units of this item are required per patient (throughout the entire treatment provided for complication)?<br>*Smallest unit is one gown.*<br>*Question relevant when: selected(${q102_E18_full_list} , '5')*<br>*Response constrained to: .>=1*	
q602_E19	q602_E19. Heavy duty gloves<br>*Question relevant when: selected(${q102_E19_full_list} , '5')*	
q602_E19a *(required)*	q602_E19a. What percent of patients require this item?<br>*Question relevant when: selected(${q102_E19_full_list} , '5')*<br>*Response constrained to: .>0 and .<=100 or .=999*	
q602_E19b *(required)*	q602_E19b. How many units of this item are required per patient (throughout the entire treatment provided for complication)?<br>*Smallest unit is one glove.*<br>*Question relevant when: selected(${q102_E19_full_list} , '5')*<br>*Response constrained to: .>=1*	
q602_E20	q602_E20. Mask (reusable)<br>*Question relevant when: selected(${q102_E20_full_list} , '5')*	
q602_E20a *(required)*	q602_E20a. What percent of patients require this item?<br>*Question relevant when: selected(${q102_E20_full_list} , '5')*<br>*Response constrained to: .>0 and .<=100 or .=999*	
q602_E20b *(required)*	q602_E20b. How many units of this item are required per patient (throughout the entire treatment provided for complication)?<br>*Smallest unit is one mask.*<br>*Question relevant when: selected(${q102_E20_full_list} , '5')*<br>*Response constrained to: .>=1*	
q602_E21	q602_E21. Protective glasses<br>*Question relevant when: selected(${q102_E21_full_list} , '5')*	
q602_E21a *(required)*	q602_E21a. What percent of patients require this item?<br>*Question relevant when: selected(${q102_E21_full_list} , '5')*<br>*Response constrained to: .>0 and .<=100 or .=999*	
q602_E21b *(required)*	q602_E21b. How many units of this item are required per patient (throughout the entire treatment provided for complication)?<br>*Smallest unit is one pair of glasses.*	

Field	Question	Answer
	Question relevant when: selected(${q102_E21_full_list} , '5')<br>*Response constrained to: .>=1*	
B. Small equipment - Perforations (5)<br>*Group relevant when: selected(${section_six_skip_equ} , '1')*		
note_602_receivers	***Receivers***	

q602_E22	q602_E22. Kidney dish - small<br>*Question relevant when: selected(${q102_E22_full_list} , '5')*	
q602_E22a *(required)*	q602_E22a. What percent of patients require this item?<br>*Question relevant when: selected(${q102_E22_full_list} , '5')*<br>*Response constrained to: .>0 and .<=100 or .=999*	
q602_E22b *(required)*	q602_E22b. How many units of this item are required per patient (throughout the entire treatment provided for complication)?<br>*Smallest unit is one dish.*<br>*Question relevant when: selected(${q102_E22_full_list} , '5')*<br>*Response constrained to: .>=1*	
q602_E23	q602_E23. Kidney dish - medium<br>*Question relevant when: selected(${q102_E23_full_list} , '5')*	
q602_E23a *(required)*	q602_E23a. What percent of patients require this item?<br>*Question relevant when: selected(${q102_E23_full_list} , '5')*<br>*Response constrained to: .>0 and .<=100 or .=999*	
q602_E23b *(required)*	q602_E23b. How many units of this item are required per patient (throughout the entire treatment provided for complication)?<br>*Smallest unit is one dish.*<br>*Question relevant when: selected(${q102_E23_full_list} , '5')*<br>*Response constrained to: .>=1*	
q602_E24	q602_E24. Kidney dish - large<br>*Question relevant when: selected(${q102_E24_full_list} , '5')*	
q602_E24a *(required)*	q602_E24a. What percent of patients require this item?<br>*Question relevant when: selected(${q102_E24_full_list} , '5')*<br>*Response constrained to: .>0 and .<=100 or .=999*	
q602_E24b *(required)*	q602_E24b. How many units of this item are required per patient (throughout the entire treatment provided for complication)?<br>*Smallest unit is one dish.*<br>*Question relevant when: selected(${q102_E24_full_list} , '5')*<br>*Response constrained to: .>=1*	
q602_E25	q602_E25. Receiving bowl - small<br>*Question relevant when: selected(${q102_E25_full_list} , '5')*	
q602_E25a *(required)*	q602_E25a. What percent of patients require this item?<br>*Question relevant when: selected(${q102_E25_full_list} , '5')*<br>*Response constrained to: .>0 and .<=100 or .=999*	
q602_E25b *(required)*	q602_E25b. How many units of this item are required per patient (throughout the entire treatment provided for complication)?<br>*Smallest unit is one dish.*<br>*Question relevant when: selected(${q102_E25_full_list} , '5')*<br>*Response constrained to: .>=1*	
q602_E26	q602_E26. Receiving bowl - medium<br>*Question relevant when: selected(${q102_E26_full_list} , '5')*	
q602_E26a *(required)*	q602_E26a. What percent of patients require this item?<br>*Question relevant when: selected(${q102_E26_full_list} , '5')*<br>*Response constrained to: .>0 and .<=100 or .=999*	
q602_E26b *(required)*	q602_E26b. How many units of this item are required per patient (throughout the entire treatment provided for complication)?<br>*Smallest unit is one dish.*<br>*Question relevant when: selected(${q102_E26_full_list} , '5')*<br>*Response constrained to: .>=1*	
q602_E27	q602_E27. Receiving bowl - large<br>*Question relevant when: selected(${q102_E27_full_list} , '5')*	
q602_E27a *(required)*	q602_E27a. What percent of patients require this item?<br>*Question relevant when: selected(${q102_E27_full_list} , '5')*<br>*Response constrained to: .>0 and .<=100 or .=999*	
q602_E27b *(required)*	q602_E27b. How many units of this item are required per patient (throughout the entire treatment provided for complication)?<br>*Smallest unit is one dish.*<br>*Question relevant when: selected(${q102_E27_full_list} , '5')*	

Field	Question	Answer
	Response constrained to: .>=1	
B. Small equipment - Perforations (6)		
Group relevant when: selected(${section_six_skip_equ} , '1')		
note_602_surgery	***Surgery***	
q602_E28	q602_E28. Scalpel, reusable<br>*Question relevant when: selected(${q102_E28_full_list} , '5')*	
q602_E28a (required)	q602_E28a. What percent of patients require this item?<br>*Question relevant when: selected(${q102_E28_full_list} , '5')*<br>*Response constrained to: .>0 and .<=100 or .=999*	
q602_E28b (required)	q602_E28b. How many units of this item are required per patient (throughout the entire treatment provided for complication)?<br>*Smallest unit is one scalpel.*<br>*Question relevant when: selected(${q102_E28_full_list} , '5')*<br>*Response constrained to: .>=1*	
q602_E29	q602_E29. Surgical scissors<br>*Question relevant when: selected(${q102_E29_full_list} , '5')*	
q602_E29a (required)	q602_E29a. What percent of patients require this item?<br>*Question relevant when: selected(${q102_E29_full_list} , '5')*<br>*Response constrained to: .>0 and .<=100 or .=999*	
q602_E29b (required)	q602_E29b. How many units of this item are required per patient (throughout the entire treatment provided for complication)?<br>*Smallest unit is one pair of scissors.*<br>*Question relevant when: selected(${q102_E29_full_list} , '5')*<br>*Response constrained to: .>=1*	
B. Small equipment - Perforations (7)		
Group relevant when: selected(${section_six_skip_equ} , '1')		
note_602_other	***Other small equipment***	
q602_E30	q602_E30. Head lamp/batteries<br>*Question relevant when: selected(${q102_E30_full_list} , '5')*	
q602_E30a (required)	q602_E30a. What percent of patients require this item?<br>*Question relevant when: selected(${q102_E30_full_list} , '5')*<br>*Response constrained to: .>0 and .<=100 or .=999*	
q602_E30b (required)	q602_E30b. How many units of this item are required per patient (throughout the entire treatment provided for complication)?<br>*Smallest unit is one lamp/battery set.*<br>*Question relevant when: selected(${q102_E30_full_list} , '5')*<br>*Response constrained to: .>=1*	
q602_E31	q602_E31. Metal catheter<br>*Question relevant when: selected(${q102_E31_full_list} , '5')*	
q602_E31a (required)	q602_E31a. What percent of patients require this item?<br>*Question relevant when: selected(${q102_E31_full_list} , '5')*<br>*Response constrained to: .>0 and .<=100 or .=999*	
q602_E31b (required)	q602_E31b. How many units of this item are required per patient (throughout the entire treatment provided for complication)?<br>*Smallest unit is one catheter.*<br>*Question relevant when: selected(${q102_E31_full_list} , '5')*<br>*Response constrained to: .>=1*	
q602_E32	q602_E32. Urine collection flask<br>*Question relevant when: selected(${q102_E32_full_list} , '5')*	
q602_E32a (required)	q602_E32a. What percent of patients require this item?<br>*Question relevant when: selected(${q102_E32_full_list} , '5')*<br>*Response constrained to: .>0 and .<=100 or .=999*	
q602_E32b (required)	q602_E32b. How many units of this item are required per patient (throughout the entire treatment provided for complication)?<br>*Smallest unit is one flask.*<br>*Question relevant when: selected(${q102_E32_full_list} , '5')*<br>*Response constrained to: .>=1*	
B. Small equipment - Perforations (8)		
Group relevant when: selected(${section_six_skip_equ} , '1')		
note_602_other_suggestion	***Other equipment - suggestions?***	
q602_E33	q602_E33. Equipment other 1: "[q102_E33_full_list_other]"<br>*Question relevant when: selected(${q102_E33_full_list} , '5')*	
q602_E33a (required)	q602_E33a. What percent of patients require this item?<br>*Question relevant when: selected(${q102_E33_full_list} , '5')*<br>[illegible]	

Response constrained to: .>0 and .<=100 or .=999

Field	Question	Answer
q602_E33b *(required)*	q602_E33b. How many units of this item are required per patient (throughout the entire treatment provided for complication)?<br>*Question relevant when: selected(${q102_E33_full_list} , '5')*<br>*Response constrained to: .>=1*	
q602_E34	q602_E34. Equipment other 2: "[q102_E34_full_list_other]"<br>*Question relevant when: selected(${q102_E34_full_list} , '5')*	

q602_E34a *(required)*	q602_E34a. What percent of patients require this item?<br>*Question relevant when: selected(${q102_E34_full_list} , '5')*<br>*Response constrained to: .>0 and .<=100 or .=999*	
q602_E34b *(required)*	q602_E34b. How many units of this item are required per patient (throughout the entire treatment provided for complication)?<br>*Question relevant when: selected(${q102_E34_full_list} , '5')*<br>*Response constrained to: .>=1*	
q602_E35	q602_E35. Equipment other 3: "[q102_E35_full_list_other]"<br>*Question relevant when: selected(${q102_E35_full_list} , '5')*	
q602_E35a *(required)*	q602_E35a. What percent of patients require this item?<br>*Question relevant when: selected(${q102_E35_full_list} , '5')*<br>*Response constrained to: .>0 and .<=100 or .=999*	
q602_E35b *(required)*	q602_E35b. How many units of this item are required per patient (throughout the entire treatment provided for complication)?<br>*Question relevant when: selected(${q102_E35_full_list} , '5')*<br>*Response constrained to: .>=1*	
q602_E36	q602_E36. Equipment other 4: "[q102_E36_full_list_other]"<br>*Question relevant when: selected(${q102_E36_full_list} , '5')*	
q602_E36a *(required)*	q602_E36a. What percent of patients require this item?<br>*Question relevant when: selected(${q102_E36_full_list} , '5')*<br>*Response constrained to: .>0 and .<=100 or .=999*	
q602_E36b *(required)*	q602_E36b. How many units of this item are required per patient (throughout the entire treatment provided for complication)?<br>*Question relevant when: selected(${q102_E36_full_list} , '5')*<br>*Response constrained to: .>=1*	
q602_E37	q602_E37. Equipment other 5: "[q102_E37_full_list_other]"<br>*Question relevant when: selected(${q102_E37_full_list} , '5')*	
q602_E37a *(required)*	q602_E37a. What percent of patients require this item?<br>*Question relevant when: selected(${q102_E37_full_list} , '5')*<br>*Response constrained to: .>0 and .<=100 or .=999*	
q602_E37b *(required)*	q602_E37b. How many units of this item are required per patient (throughout the entire treatment provided for complication)?<br>*Question relevant when: selected(${q102_E37_full_list} , '5')*<br>*Response constrained to: .>=1*	
group_section_seven_intro		
section7_start	**SECTION VII. COSTS OF ALL ITEMS**	
section_seven_skip_equ	INTERVIEWER: WOULD YOU LIKE TO COMPLETE THIS SECTION NOW OR SKIP THIS SECTION AND RETURN TO IT LATER?<br>*You may need to skip if the participant has indicated that s/he cannot answer the questions in this section.*	1 Do not skip, complete this section now.<br>2 Skip and come back to this section later.
group_section_seven_introB<br>*Group relevant when: selected(${section_seven_skip_equ} , '1')*		
section7_start2	In this section of the interview, we will review all of the items that you said are used for management of any complication type. For each item that is used, I'm going to ask how much the item costs and how it is procured.	
section7_start3	INTERVIEWER: ENTER 999 FOR ANY THAT ARE UNKNOWN.	
B. Small equipment - Cost (1)<br>*Group relevant when: selected(${section_seven_skip_equ} , '1')*		
note_702_evac	***Evacuation***	
q702_E1	***q702_E1. MVA aspirator***<br>*Question relevant when: not(selected(${q102_E1_full_list} , '6')) and not(selected(${q102_E1_full_list} , '99'))*	
q702_E1a *(required)*	q702_E1a. When purchased, how many units of this item typically come in one pack, box, bottle, etc.? (e.g. 100 tablets, 1000 ml's, etc.) (Write 1 if purchased as a single item.)<br>*Smallest unit is one aspirator.*<br>*Question relevant when: not(selected(${q102_E1_full_list} , '6')) and not(selected(${q102_E1_full_list} , '99'))*<br>*Response constrained to: .>=0*	
q702_E1b *(required)*	q702_E1b. What is the typical purchase price for that quanity of units (i.e. the quantity noted in the question above)?<br>*Question relevant when: not(selected(${q102_E1_full_list} , '6')) and not(selected(${q102_E1_full_list} , '99'))*	

Field	Question	Answer
	Response constrained to: .>=0	
q702_E1c *(required)*	q702_E1c. Please specify the currency for the purchase price.<br>*Question relevant when: not(selected(${q102_E1_full_list} , '6')) and not(selected(${q102_E1_full_list} , '99'))*	1 Tanzanian Shillings<br>2 US dollars<br>3 Euros<br>4 Other<br>99 Don't know

Field	Question	Answer
q702_E1c.1	q702_E1c.1 If other currency, specify:<br>*Leave blank if not applicable*<br>*Question relevant when: not(selected(${q102_E1_full_list} , '6')) and not(selected(${q102_E1_full_list} , '99'))*	
q702_E1d *(required)*	q702_E1d. Please specify the year of the purchase price.<br>*Question relevant when: not(selected(${q102_E1_full_list} , '6')) and not(selected(${q102_E1_full_list} , '99'))*<br>*Response constrained to: .>=2000 and .<=2019 or .=999*	
q702_E1e *(required)*	q702_E1e. How often is the item replaced?<br>*Question relevant when: not(selected(${q102_E1_full_list} , '6')) and not(selected(${q102_E1_full_list} , '99'))*	1 After each use<br>2 Daily<br>3 Weekly<br>4 Monthly<br>5 Yearly<br>6 Item is replaced after other number of uses, weeks, years, etc. (specify how often in next question)<br>99 Don't know
q702_E1e.1	q702_E1e.1. If "other number of uses", specify how often:<br>*Remember to indicate "uses," "weeks", etc. with number.*<br>*Question relevant when: not(selected(${q102_E1_full_list} , '6')) and not(selected(${q102_E1_full_list} , '99'))*	
q702_E2	***q702_E2. Cannula set***<br>*Question relevant when: not(selected(${q102_E2_full_list} , '6')) and not(selected(${q102_E2_full_list} , '99'))*	
q702_E2a *(required)*	q702_E2a. When purchased, how many units of this item typically come in one pack, box, bottle, etc.? (e.g. 100 tablets, 1000 ml's, etc.) (Write 1 if purchased as a single item.)<br>*Smallest unit is one set.*<br>*Question relevant when: not(selected(${q102_E2_full_list} , '6')) and not(selected(${q102_E2_full_list} , '99'))*<br>*Response constrained to: .>=0*	
q702_E2b *(required)*	q702_E2b. What is the typical purchase price for that quanity of units (i.e. the quantity noted in the question above)?<br>*Question relevant when: not(selected(${q102_E2_full_list} , '6')) and not(selected(${q102_E2_full_list} , '99'))*<br>*Response constrained to: .>=0*	
q702_E2c *(required)*	q702_E2c. Please specify the currency for the purchase price.<br>*Question relevant when: not(selected(${q102_E2_full_list} , '6')) and not(selected(${q102_E2_full_list} , '99'))*	1 Tanzanian Shillings<br>2 US dollars<br>3 Euros<br>4 Other<br>99 Don't know
q702_E2c.1	q702_E2c.1 If other currency, specify:<br>*Leave blank if not applicable*<br>*Question relevant when: not(selected(${q102_E2_full_list} , '6')) and not(selected(${q102_E2_full_list} , '99'))*	
q702_E2d *(required)*	q702_E2d. Please specify the year of the purchase price.<br>*Question relevant when: not(selected(${q102_E2_full_list} , '6')) and not(selected(${q102_E2_full_list} , '99'))*<br>*Response constrained to: .>=2000 and .<=2019 or .=999*	
q702_E2e *(required)*	q702_E2e. How often is the item replaced?<br>*Question relevant when: not(selected(${q102_E2_full_list} , '6')) and not(selected(${q102_E2_full_list} , '99'))*	1 After each use<br>2 Daily<br>3 Weekly<br>4 Monthly<br>5 Yearly<br>6 Item is replaced after other number of uses, weeks, years, etc. (specify how often in next question)<br>99 Don't know
q702_E2e.1	q702_E2e.1. If "other number of uses", specify how often:<br>*Remember to indicate "uses," "weeks", etc. with number.*<br>*Question relevant when: not(selected(${q102_E2_full_list} , '6')) and not(selected(${q102_E2_full_list} , '99'))*	
q702_E3	***q702_E3. Uterine curette set - plastic***<br>*Question relevant when: not(selected(${q102_E3_full_list} , '6')) and not(selected(${q102_E3_full_list} , '99'))*	
q702_E3a *(required)*	q702_E3a. When purchased, how many units of this item typically come in one pack, box, bottle, etc.? (e.g. 100 tablets, 1000 ml's, etc.) (Write 1 if purchased as a single item.)<br>*Smallest unit is one set.*	

Field	Question	Answer
	Question relevant when: not(selected(${q102_E3_full_list} , '6')) and not(selected(${q102_E3_full_list} , '99'))<br>*Response constrained to: .>=0*	
q702_E3b *(required)*	q702_E3b. What is the typical purchase price for that quanity of units (i.e. the quantity noted in the question above)?<br>*Question relevant when: not(selected(${q102_E3_full_list} , '6')) and not(selected(${q102_E3_full_list} , '99'))*<br>*Response constrained to: .>=0*	

Field	Question	Answer
q702_E3c *(required)*	q702_E3c. Please specify the currency for the purchase price.<br>*Question relevant when: not(selected(${q102_E3_full_list} , '6')) and not(selected(${q102_E3_full_list} , '99'))*	1 Tanzanian Shillings<br>2 US dollars<br>3 Euros<br>4 Other<br>99 Don't know
q702_E3c.1	q702_E3c.1 If other currency, specify:<br>*Leave blank if not applicable*<br>*Question relevant when: not(selected(${q102_E3_full_list} , '6')) and not(selected(${q102_E3_full_list} , '99'))*	
q702_E3d *(required)*	q702_E3d. Please specify the year of the purchase price.<br>*Question relevant when: not(selected(${q102_E3_full_list} , '6')) and not(selected(${q102_E3_full_list} , '99'))*<br>*Response constrained to: .>=2000 and .<=2019 or .=999*	
q702_E3e *(required)*	q702_E3e. How often is the item replaced?<br>*Question relevant when: not(selected(${q102_E3_full_list} , '6')) and not(selected(${q102_E3_full_list} , '99'))*	1 After each use<br>2 Daily<br>3 Weekly<br>4 Monthly<br>5 Yearly<br>6 Item is replaced after other number of uses, weeks, years, etc. (specify how often in next question)<br>99 Don't know
q702_E3e.1	q702_E3e.1. If "other number of uses", specify how often:<br>*Remember to indicate "uses," "weeks", etc. with number.*<br>*Question relevant when: not(selected(${q102_E3_full_list} , '6')) and not(selected(${q102_E3_full_list} , '99'))*	
q702_E4	***q702_E4. Uterine currette set - metal***<br>*Question relevant when: not(selected(${q102_E4_full_list} , '6')) and not(selected(${q102_E4_full_list} , '99'))*	
q702_E4a *(required)*	q702_E4a. When purchased, how many units of this item typically come in one pack, box, bottle, etc.? (e.g. 100 tablets, 1000 ml's, etc.) (Write 1 if purchased as a single item.)<br>*Smallest unit is one set.*<br>*Question relevant when: not(selected(${q102_E4_full_list} , '6')) and not(selected(${q102_E4_full_list} , '99'))*<br>*Response constrained to: .>=0*	
q702_E4b *(required)*	q702_E4b. What is the typical purchase price for that quanity of units (i.e. the quantity noted in the question above)?<br>*Question relevant when: not(selected(${q102_E4_full_list} , '6')) and not(selected(${q102_E4_full_list} , '99'))*<br>*Response constrained to: .>=0*	
q702_E4c *(required)*	q702_E4c. Please specify the currency for the purchase price.<br>*Question relevant when: not(selected(${q102_E4_full_list} , '6')) and not(selected(${q102_E4_full_list} , '99'))*	1 Tanzanian Shillings<br>2 US dollars<br>3 Euros<br>4 Other<br>99 Don't know
q702_E4c.1	q702_E4c.1 If other currency, specify:<br>*Leave blank if not applicable*<br>*Question relevant when: not(selected(${q102_E4_full_list} , '6')) and not(selected(${q102_E4_full_list} , '99'))*	
q702_E4d *(required)*	q702_E4d. Please specify the year of the purchase price.<br>*Question relevant when: not(selected(${q102_E4_full_list} , '6')) and not(selected(${q102_E4_full_list} , '99'))*<br>*Response constrained to: .>=2000 and .<=2019 or .=999*	
q702_E4e *(required)*	q702_E4e. How often is the item replaced?<br>*Question relevant when: not(selected(${q102_E4_full_list} , '6')) and not(selected(${q102_E4_full_list} , '99'))*	1 After each use<br>2 Daily<br>3 Weekly<br>4 Monthly<br>5 Yearly<br>6 Item is replaced after other number of uses, weeks, years, etc. (specify how often in next question)<br>99 Don't know
q702_E4e.1	q702_E4e.1. If "other number of uses", specify how often:<br>*Remember to indicate "uses," "weeks", etc. with number.*<br>[illegible]	

Question relevant when: not(selected(${q102_E4_full_list} , '6')) and not(selected(${q102_E4_full_list} , '99'))

Field	Question	Answer
q702_E5	***q702_E5. Dilators (Hegar's Cervical dilators)***<br>*Question relevant when: not(selected(${q102_E5_full_list} , '6')) and not(selected(${q102_E5_full_list} , '99'))*	
q702_E5a *(required)*	q702_E5a. When purchased, how many units of this item typically come in one pack, box, bottle, etc.? (e.g. 100 tablets, 1000 ml's, etc.) (Write 1 if purchased as a single item.)<br>*Smallest unit is one set.*<br>*Question relevant when: not(selected(${q102_E5_full_list} , '6')) and not(selected(${q102_E5_full_list} , '99'))*<br>*Response constrained to: .>=0*	
q702_E5b *(required)*	q702_E5b. What is the typical purchase price for that quanity of units (i.e. the quantity noted in the question above)?<br>*Question relevant when: not(selected(${q102_E5_full_list} , '6')) and not(selected(${q102_E5_full_list} , '99'))*<br>*Response constrained to: .>=0*	
q702_E5c *(required)*	q702_E5c. Please specify the currency for the purchase price.<br>*Question relevant when: not(selected(${q102_E5_full_list} , '6')) and not(selected(${q102_E5_full_list} , '99'))*	1 Tanzanian Shillings<br>2 US dollars<br>3 Euros<br>4 Other<br>99 Don't know
q702_E5c.1	q702_E5c.1 If other currency, specify:<br>*Leave blank if not applicable*<br>*Question relevant when: not(selected(${q102_E5_full_list} , '6')) and not(selected(${q102_E5_full_list} , '99'))*	
q702_E5d *(required)*	q702_E5d. Please specify the year of the purchase price.<br>*Question relevant when: not(selected(${q102_E5_full_list} , '6')) and not(selected(${q102_E5_full_list} , '99'))*<br>*Response constrained to: .>=2000 and .<=2019 or .=999*	
q702_E5e *(required)*	q702_E5e. How often is the item replaced?<br>*Question relevant when: not(selected(${q102_E5_full_list} , '6')) and not(selected(${q102_E5_full_list} , '99'))*	1 After each use<br>2 Daily<br>3 Weekly<br>4 Monthly<br>5 Yearly<br>6 Item is replaced after other number of uses, weeks, years, etc. (specify how often in next question)<br>99 Don't know
q702_E5e.1	q702_E5e.1. If "other number of uses", specify how often:<br>*Remember to indicate "uses," "weeks", etc. with number.*<br>*Question relevant when: not(selected(${q102_E5_full_list} , '6')) and not(selected(${q102_E5_full_list} , '99'))*	
B. Small equipment - Cost (2)<br>*Group relevant when: selected(${section_seven_skip_equ} , '1')*		
note_702_forceps	***Forceps, speculums, dilators, etc.***	
q702_E6	***q702_E6. Forceps, artery/Kocher***<br>*Question relevant when: not(selected(${q102_E6_full_list} , '6')) and not(selected(${q102_E6_full_list} , '99'))*	
q702_E6a *(required)*	q702_E6a. When purchased, how many units of this item typically come in one pack, box, bottle, etc.? (e.g. 100 tablets, 1000 ml's, etc.) (Write 1 if purchased as a single item.)<br>*Smallest unit is one forceps.*<br>*Question relevant when: not(selected(${q102_E6_full_list} , '6')) and not(selected(${q102_E6_full_list} , '99'))*<br>*Response constrained to: .>=0*	
q702_E6b *(required)*	q702_E6b. What is the typical purchase price for that quanity of units (i.e. the quantity noted in the question above)?<br>*Question relevant when: not(selected(${q102_E6_full_list} , '6')) and not(selected(${q102_E6_full_list} , '99'))*<br>*Response constrained to: .>=0*	
q702_E6c *(required)*	q702_E6c. Please specify the currency for the purchase price.<br>*Question relevant when: not(selected(${q102_E6_full_list} , '6')) and not(selected(${q102_E6_full_list} , '99'))*	1 Tanzanian Shillings<br>2 US dollars<br>3 Euros<br>4 Other<br>99 Don't know
q702_E6c.1	q702_E6c.1 If other currency, specify:<br>*Leave blank if not applicable*<br>*Question relevant when: not(selected(${q102_E6_full_list} , '6')) and not(selected(${q102_E6_full_list} , '99'))*	
q702_E6d *(required)*	q702_E6d. Please specify the year of the purchase price.<br>*Question relevant when: not(selected(${q102_E6_full_list} , '6')) and not(selected(${q102_E6_full_list} , '99'))*<br>*Response constrained to: .>=2000 and .<=2019 or .=999*	

Field	Question	Answer
q702_E6e *(required)*	q702_E6e. How often is the item replaced?<br>*Question relevant when: not(selected(${q102_E6_full_list} , '6')) and not(selected(${q102_E6_full_list} , '99'))*	1 After each use<br>2 Daily<br>3 Weekly<br>4 Monthly<br>5 Yearly<br>6 Item is replaced after other number of uses, weeks, years, etc. (specify how often in next question)<br>99 Don't know
q702_E6e.1	q702_E6e.1. If "other number of uses", specify how often:<br>*Remember to indicate "uses," "weeks", etc. with number.*<br>*Question relevant when: not(selected(${q102_E6_full_list} , '6')) and not(selected(${q102_E6_full_list} , '99'))*	
q702_E7	***q702_E7. Needle holder***<br>*Question relevant when: not(selected(${q102_E7_full_list} , '6')) and not(selected(${q102_E7_full_list} , '99'))*	
q702_E7a *(required)*	q702_E7a. When purchased, how many units of this item typically come in one pack, box, bottle, etc.? (e.g. 100 tablets, 1000 ml's, etc.) (Write 1 if purchased as a single item.)<br>*Smallest unit is one holder.*<br>*Question relevant when: not(selected(${q102_E7_full_list} , '6')) and not(selected(${q102_E7_full_list} , '99'))*<br>*Response constrained to: .>=0*	
q702_E7b *(required)*	q702_E7b. What is the typical purchase price for that quanity of units (i.e. the quantity noted in the question above)?<br>*Question relevant when: not(selected(${q102_E7_full_list} , '6')) and not(selected(${q102_E7_full_list} , '99'))*<br>*Response constrained to: .>=0*	
q702_E7c *(required)*	q702_E7c. Please specify the currency for the purchase price.<br>*Question relevant when: not(selected(${q102_E7_full_list} , '6')) and not(selected(${q102_E7_full_list} , '99'))*	1 Tanzanian Shillings<br>2 US dollars<br>3 Euros<br>4 Other<br>99 Don't know
q702_E7c.1	q702_E7c.1 If other currency, specify:<br>*Leave blank if not applicable*<br>*Question relevant when: not(selected(${q102_E7_full_list} , '6')) and not(selected(${q102_E7_full_list} , '99'))*	
q702_E7d *(required)*	q702_E7d. Please specify the year of the purchase price.<br>*Question relevant when: not(selected(${q102_E7_full_list} , '6')) and not(selected(${q102_E7_full_list} , '99'))*<br>*Response constrained to: .>=2000 and .<=2019 or .=999*	
q702_E7e *(required)*	q702_E7e. How often is the item replaced?<br>*Question relevant when: not(selected(${q102_E7_full_list} , '6')) and not(selected(${q102_E7_full_list} , '99'))*	1 After each use<br>2 Daily<br>3 Weekly<br>4 Monthly<br>5 Yearly<br>6 Item is replaced after other number of uses, weeks, years, etc. (specify how often in next question)<br>99 Don't know
q702_E7e.1	q702_E7e.1. If "other number of uses", specify how often:<br>*Remember to indicate "uses," "weeks", etc. with number.*<br>*Question relevant when: not(selected(${q102_E7_full_list} , '6')) and not(selected(${q102_E7_full_list} , '99'))*	
q702_E8	***q702_E8. Ovum forceps***<br>*Question relevant when: not(selected(${q102_E8_full_list} , '6')) and not(selected(${q102_E8_full_list} , '99'))*	
q702_E8a *(required)*	q702_E8a. When purchased, how many units of this item typically come in one pack, box, bottle, etc.? (e.g. 100 tablets, 1000 ml's, etc.) (Write 1 if purchased as a single item.)<br>*Smallest unit is one forceps.*<br>*Question relevant when: not(selected(${q102_E8_full_list} , '6')) and not(selected(${q102_E8_full_list} , '99'))*<br>*Response constrained to: .>=0*	
q702_E8b *(required)*	q702_E8b. What is the typical purchase price for that quanity of units (i.e. the quantity noted in the question above)?<br>*Question relevant when: not(selected(${q102_E8_full_list} , '6')) and not(selected(${q102_E8_full_list} , '99'))*<br>*Response constrained to: .>=0*	
q702_E8c *(required)*	q702_E8c. Please specify the currency for the purchase price.	1 Tanzanian Shillings

Field	Question	Answer
q702_E8c (required)	q702_E8c. Please specify the currency for the purchase price.<br>*Question relevant when: not(selected(${q102_E8_full_list} , '6')) and not(selected(${q102_E8_full_list} , '99'))*	1 Tanzanian Shillings<br>2 US dollars<br>3 Euros<br>4 Other<br>99 Don't know
q702_E8c.1	q702_E8c.1 If other currency, specify:<br>*Leave blank if not applicable*<br>*Question relevant when: not(selected(${q102_E8_full_list} , '6')) and not(selected(${q102_E8_full_list} , '99'))*	
q702_E8d (required)	q702_E8d. Please specify the year of the purchase price.<br>*Question relevant when: not(selected(${q102_E8_full_list} , '6')) and not(selected(${q102_E8_full_list} , '99'))*<br>*Response constrained to: .>=2000 and .<=2019 or .=999*	
q702_E8e (required)	q702_E8e. How often is the item replaced?<br>*Question relevant when: not(selected(${q102_E8_full_list} , '6')) and not(selected(${q102_E8_full_list} , '99'))*	1 After each use<br>2 Daily<br>3 Weekly<br>4 Monthly<br>5 Yearly<br>6 Item is replaced after other number of uses, weeks, years, etc. (specify how often in next question)<br>99 Don't know
q702_E8e.1	q702_E8e.1. If "other number of uses", specify how often:<br>*Remember to indicate "uses," "weeks", etc. with number.*<br>*Question relevant when: not(selected(${q102_E8_full_list} , '6')) and not(selected(${q102_E8_full_list} , '99'))*	
q702_E9	***q702_E9. Ring forceps***<br>*Question relevant when: not(selected(${q102_E9_full_list} , '6')) and not(selected(${q102_E9_full_list} , '99'))*	
q702_E9a (required)	q702_E9a. When purchased, how many units of this item typically come in one pack, box, bottle, etc.? (e.g. 100 tablets, 1000 ml's, etc.) (Write 1 if purchased as a single item.)<br>*Smallest unit is one forceps.*<br>*Question relevant when: not(selected(${q102_E9_full_list} , '6')) and not(selected(${q102_E9_full_list} , '99'))*<br>*Response constrained to: .>=0*	
q702_E9b (required)	q702_E9b. What is the typical purchase price for that quanity of units (i.e. the quantity noted in the question above)?<br>*Question relevant when: not(selected(${q102_E9_full_list} , '6')) and not(selected(${q102_E9_full_list} , '99'))*<br>*Response constrained to: .>=0*	
q702_E9c (required)	q702_E9c. Please specify the currency for the purchase price.<br>*Question relevant when: not(selected(${q102_E9_full_list} , '6')) and not(selected(${q102_E9_full_list} , '99'))*	1 Tanzanian Shillings<br>2 US dollars<br>3 Euros<br>4 Other<br>99 Don't know
q702_E9c.1	q702_E9c.1 If other currency, specify:<br>*Leave blank if not applicable*<br>*Question relevant when: not(selected(${q102_E9_full_list} , '6')) and not(selected(${q102_E9_full_list} , '99'))*	
q702_E9d (required)	q702_E9d. Please specify the year of the purchase price.<br>*Question relevant when: not(selected(${q102_E9_full_list} , '6')) and not(selected(${q102_E9_full_list} , '99'))*<br>*Response constrained to: .>=2000 and .<=2019 or .=999*	
q702_E9e (required)	q702_E9e. How often is the item replaced?<br>*Question relevant when: not(selected(${q102_E9_full_list} , '6')) and not(selected(${q102_E9_full_list} , '99'))*	1 After each use<br>2 Daily<br>3 Weekly<br>4 Monthly<br>5 Yearly<br>6 Item is replaced after other number of uses, weeks, years, etc. (specify how often in next question)<br>99 Don't know
q702_E9e.1	q702_E9e.1. If "other number of uses", specify how often:<br>*Remember to indicate "uses," "weeks", etc. with number.*<br>*Question relevant when: not(selected(${q102_E9_full_list} , '6')) and not(selected(${q102_E9_full_list} , '99'))*	
q702_E10	***q702_E10. Speculum, BValve/Cuscos (reusable)***<br>*Question relevant when: not(selected(${q102_E10_full_list} , '6')) and not(selected(${q102_E10_full_list} , '99'))*	
q702_E10a (required)	q702_E10a. When purchased, how many units of this item typically come in one pack, box, bottle, etc.? (e.g. 100 tablets, 1000 ml's, etc.) (Write 1 if purchased as a single item.)<br>*Smallest unit is one speculum.*	

Field	Question	Answer
	Question relevant when: not(selected(${q102_E10_full_list} , '6')) and not(selected(${q102_E10_full_list} , '99'))<br>*Response constrained to: .>=0*	
q702_E10b *(required)*	q702_E10b. What is the typical purchase price for that quanity of units (i.e. the quantity noted in the question above)?<br>*Question relevant when: not(selected(${q102_E10_full_list} , '6')) and not(selected(${q102_E10_full_list} , '99'))*<br>*Response constrained to: .>=0*	
q702_E10c *(required)*	q702_E10c. Please specify the currency for the purchase price.<br>*Question relevant when: not(selected(${q102_E10_full_list} , '6')) and not(selected(${q102_E10_full_list} , '99'))*	1 Tanzanian Shillings<br>2 US dollars<br>3 Euros<br>4 Other<br>99 Don't know
q702_E10c.1	q702_E10c.1 If other currency, specify:<br>*Leave blank if not applicable*<br>*Question relevant when: not(selected(${q102_E10_full_list} , '6')) and not(selected(${q102_E10_full_list} , '99'))*	
q702_E10d *(required)*	q702_E10d. Please specify the year of the purchase price.<br>*Question relevant when: not(selected(${q102_E10_full_list} , '6')) and not(selected(${q102_E10_full_list} , '99'))*<br>*Response constrained to: .>=2000 and .<=2019 or .=999*	
q702_E10e *(required)*	q702_E10e. How often is the item replaced?<br>*Question relevant when: not(selected(${q102_E10_full_list} , '6')) and not(selected(${q102_E10_full_list} , '99'))*	1 After each use<br>2 Daily<br>3 Weekly<br>4 Monthly<br>5 Yearly<br>6 Item is replaced after other number of uses, weeks, years, etc. (specify how often in next question)<br>99 Don't know
q702_E10e.1	q702_E10e.1. If "other number of uses", specify how often:<br>*Remember to indicate "uses," "weeks", etc. with number.*<br>*Question relevant when: not(selected(${q102_E10_full_list} , '6')) and not(selected(${q102_E10_full_list} , '99'))*	
q702_E11	***q702_E11. Speculum, Sim's (reusable)***<br>*Question relevant when: not(selected(${q102_E11_full_list} , '6')) and not(selected(${q102_E11_full_list} , '99'))*	
q702_E11a *(required)*	q702_E11a. When purchased, how many units of this item typically come in one pack, box, bottle, etc.? (e.g. 100 tablets, 1000 ml's, etc.) (Write 1 if purchased as a single item.)<br>*Smallest unit is one speculum.*<br>*Question relevant when: not(selected(${q102_E11_full_list} , '6')) and not(selected(${q102_E11_full_list} , '99'))*<br>*Response constrained to: .>=0*	
q702_E11b *(required)*	q702_E11b. What is the typical purchase price for that quanity of units (i.e. the quantity noted in the question above)?<br>*Question relevant when: not(selected(${q102_E11_full_list} , '6')) and not(selected(${q102_E11_full_list} , '99'))*<br>*Response constrained to: .>=0*	
q702_E11c *(required)*	q702_E11c. Please specify the currency for the purchase price.<br>*Question relevant when: not(selected(${q102_E11_full_list} , '6')) and not(selected(${q102_E11_full_list} , '99'))*	1 Tanzanian Shillings<br>2 US dollars<br>3 Euros<br>4 Other<br>99 Don't know
q702_E11c.1	q702_E11c.1 If other currency, specify:<br>*Leave blank if not applicable*<br>*Question relevant when: not(selected(${q102_E11_full_list} , '6')) and not(selected(${q102_E11_full_list} , '99'))*	
q702_E11d *(required)*	q702_E11d. Please specify the year of the purchase price.<br>*Question relevant when: not(selected(${q102_E11_full_list} , '6')) and not(selected(${q102_E11_full_list} , '99'))*<br>*Response constrained to: .>=2000 and .<=2019 or .=999*	
q702_E11e *(required)*	q702_E11e. How often is the item replaced?<br>*Question relevant when: not(selected(${q102_E11_full_list} , '6')) and not(selected(${q102_E11_full_list} , '99'))*	1 After each use<br>2 Daily<br>3 Weekly<br>4 Monthly<br>5 Yearly<br>6 Item is replaced after other number of uses, weeks, years, etc. (specify how often in next question)<br>99 Don't know
q702_E11e.1	q702_E11e.1. If "other number of uses", specify how often:<br>*Remember to indicate "uses," "weeks", etc. with number.*<br>[illegible]	

Question relevant when: not(selected(${q102_E11_full_list} , '6')) and not(selected(${q102_E11_full_list} , '99'))

Field	Question	Answer
q702_E12	***q702_E12. Tenaculum***<br>*Question relevant when: not(selected(${q102_E12_full_list} , '6')) and not(selected(${q102_E12_full_list} , '99'))*	
q702_E12a *(required)*	q702_E12a. When purchased, how many units of this item typically come in one pack, box, bottle, etc.? (e.g. 100 tablets, 1000 ml's, etc.) (Write 1 if purchased as a single item.)<br>*Smallest unit is one tenaculum.*<br>*Question relevant when: not(selected(${q102_E12_full_list} , '6')) and not(selected(${q102_E12_full_list} , '99'))*<br>*Response constrained to: .>=0*	
q702_E12b *(required)*	q702_E12b. What is the typical purchase price for that quanity of units (i.e. the quantity noted in the question above)?<br>*Question relevant when: not(selected(${q102_E12_full_list} , '6')) and not(selected(${q102_E12_full_list} , '99'))*<br>*Response constrained to: .>=0*	
q702_E12c *(required)*	q702_E12c. Please specify the currency for the purchase price.<br>*Question relevant when: not(selected(${q102_E12_full_list} , '6')) and not(selected(${q102_E12_full_list} , '99'))*	1 Tanzanian Shillings<br>2 US dollars<br>3 Euros<br>4 Other<br>99 Don't know
q702_E12c.1	q702_E12c.1 If other currency, specify:<br>*Leave blank if not applicable*<br>*Question relevant when: not(selected(${q102_E12_full_list} , '6')) and not(selected(${q102_E12_full_list} , '99'))*	
q702_E12d *(required)*	q702_E12d. Please specify the year of the purchase price.<br>*Question relevant when: not(selected(${q102_E12_full_list} , '6')) and not(selected(${q102_E12_full_list} , '99'))*<br>*Response constrained to: .>=2000 and .<=2019 or .=999*	
q702_E12e *(required)*	q702_E12e. How often is the item replaced?<br>*Question relevant when: not(selected(${q102_E12_full_list} , '6')) and not(selected(${q102_E12_full_list} , '99'))*	1 After each use<br>2 Daily<br>3 Weekly<br>4 Monthly<br>5 Yearly<br>6 Item is replaced after other number of uses, weeks, years, etc. (specify how often in next question)<br>99 Don't know
q702_E12e.1	q702_E12e.1. If "other number of uses", specify how often:<br>*Remember to indicate "uses," "weeks", etc. with number.*<br>*Question relevant when: not(selected(${q102_E12_full_list} , '6')) and not(selected(${q102_E12_full_list} , '99'))*	
B. Small equipment - Cost (3)<br>*Group relevant when: selected(${section_seven_skip_equ} , '1')*		
note_702_linens	***Linens***	
q702_E13	***q702_E13. Towel***<br>*Question relevant when: not(selected(${q102_E13_full_list} , '6')) and not(selected(${q102_E13_full_list} , '99'))*	
q702_E13a *(required)*	q702_E13a. When purchased, how many units of this item typically come in one pack, box, bottle, etc.? (e.g. 100 tablets, 1000 ml's, etc.) (Write 1 if purchased as a single item.)<br>*Smallest unit is one towel.*<br>*Question relevant when: not(selected(${q102_E13_full_list} , '6')) and not(selected(${q102_E13_full_list} , '99'))*<br>*Response constrained to: .>=0*	
q702_E13b *(required)*	q702_E13b. What is the typical purchase price for that quanity of units (i.e. the quantity noted in the question above)?<br>*Question relevant when: not(selected(${q102_E13_full_list} , '6')) and not(selected(${q102_E13_full_list} , '99'))*<br>*Response constrained to: .>=0*	
q702_E13c *(required)*	q702_E13c. Please specify the currency for the purchase price.<br>*Question relevant when: not(selected(${q102_E13_full_list} , '6')) and not(selected(${q102_E13_full_list} , '99'))*	1 Tanzanian Shillings<br>2 US dollars<br>3 Euros<br>4 Other<br>99 Don't know
q702_E13c.1	q702_E13c.1 If other currency, specify:<br>*Leave blank if not applicable*<br>*Question relevant when: not(selected(${q102_E13_full_list} , '6')) and not(selected(${q102_E13_full_list} , '99'))*	
q702_E13d *(required)*	q702_E13d. Please specify the year of the purchase price.<br>*Question relevant when: not(selected(${q102_E13_full_list} , '6')) and not(selected(${q102_E13_full_list} , '99'))*<br>*Response constrained to: .>=2000 and .<=2019 or .=999*	

Field	Question	Answer
q702_E13e *(required)*	q702_E13e. How often is the item replaced?<br>*Question relevant when: not(selected(${q102_E13_full_list} , '6')) and not(selected(${q102_E13_full_list} , '99'))*	1 After each use<br>2 Daily<br>3 Weekly<br>4 Monthly<br>5 Yearly<br>6 Item is replaced after other number of uses, weeks, years, etc. (specify how often in next question)<br>99 Don't know
q702_E13e.1	q702_E13e.1. If "other number of uses", specify how often:<br>*Remember to indicate "uses," "weeks", etc. with number.*<br>*Question relevant when: not(selected(${q102_E13_full_list} , '6')) and not(selected(${q102_E13_full_list} , '99'))*	
q702_E14	***q702_E14. Sheet***<br>*Question relevant when: not(selected(${q102_E14_full_list} , '6')) and not(selected(${q102_E14_full_list} , '99'))*	
q702_E14a *(required)*	q702_E14a. When purchased, how many units of this item typically come in one pack, box, bottle, etc.? (e.g. 100 tablets, 1000 ml's, etc.) (Write 1 if purchased as a single item.)<br>*Smallest unit is one sheet.*<br>*Question relevant when: not(selected(${q102_E14_full_list} , '6')) and not(selected(${q102_E14_full_list} , '99'))*<br>*Response constrained to: .>=0*	
q702_E14b *(required)*	q702_E14b. What is the typical purchase price for that quanity of units (i.e. the quantity noted in the question above)?<br>*Question relevant when: not(selected(${q102_E14_full_list} , '6')) and not(selected(${q102_E14_full_list} , '99'))*<br>*Response constrained to: .>=0*	
q702_E14c *(required)*	q702_E14c. Please specify the currency for the purchase price.<br>*Question relevant when: not(selected(${q102_E14_full_list} , '6')) and not(selected(${q102_E14_full_list} , '99'))*	1 Tanzanian Shillings<br>2 US dollars<br>3 Euros<br>4 Other<br>99 Don't know
q702_E14c.1	q702_E14c.1 If other currency, specify:<br>*Leave blank if not applicable*<br>*Question relevant when: not(selected(${q102_E14_full_list} , '6')) and not(selected(${q102_E14_full_list} , '99'))*	
q702_E14d *(required)*	q702_E14d. Please specify the year of the purchase price.<br>*Question relevant when: not(selected(${q102_E14_full_list} , '6')) and not(selected(${q102_E14_full_list} , '99'))*<br>*Response constrained to: .>=2000 and .<=2019 or .=999*	
q702_E14e *(required)*	q702_E14e. How often is the item replaced?<br>*Question relevant when: not(selected(${q102_E14_full_list} , '6')) and not(selected(${q102_E14_full_list} , '99'))*	1 After each use<br>2 Daily<br>3 Weekly<br>4 Monthly<br>5 Yearly<br>6 Item is replaced after other number of uses, weeks, years, etc. (specify how often in next question)<br>99 Don't know
q702_E14e.1	q702_E14e.1. If "other number of uses", specify how often:<br>*Remember to indicate "uses," "weeks", etc. with number.*<br>*Question relevant when: not(selected(${q102_E14_full_list} , '6')) and not(selected(${q102_E14_full_list} , '99'))*	
q702_E15	***q702_E15. Blanket***<br>*Question relevant when: not(selected(${q102_E15_full_list} , '6')) and not(selected(${q102_E15_full_list} , '99'))*	
q702_E15a *(required)*	q702_E15a. When purchased, how many units of this item typically come in one pack, box, bottle, etc.? (e.g. 100 tablets, 1000 ml's, etc.) (Write 1 if purchased as a single item.)<br>*Smallest unit is one blanket.*<br>*Question relevant when: not(selected(${q102_E15_full_list} , '6')) and not(selected(${q102_E15_full_list} , '99'))*<br>*Response constrained to: .>=0*	
q702_E15b *(required)*	q702_E15b. What is the typical purchase price for that quanity of units (i.e. the quantity noted in the question above)?<br>*Question relevant when: not(selected(${q102_E15_full_list} , '6')) and not(selected(${q102_E15_full_list} , '99'))*<br>*Response constrained to: .>=0*	
q702_E15c *(required)*	q702_E15c. Please specify the currency for the purchase price.	1 Tanzanian Shillings

Field	Question	Answer
q702_E15c (required)	q702_E15c. Please specify the currency for the purchase price.<br>*Question relevant when: not(selected(${q102_E15_full_list} , '6')) and not(selected(${q102_E15_full_list} , '99'))*	1 Tanzanian Shillings<br>2 US dollars<br>3 Euros<br>4 Other<br>99 Don't know
q702_E15c.1	q702_E15c.1 If other currency, specify:<br>*Leave blank if not applicable*<br>*Question relevant when: not(selected(${q102_E15_full_list} , '6')) and not(selected(${q102_E15_full_list} , '99'))*	
q702_E15d (required)	q702_E15d. Please specify the year of the purchase price.<br>*Question relevant when: not(selected(${q102_E15_full_list} , '6')) and not(selected(${q102_E15_full_list} , '99'))*<br>*Response constrained to: .>=2000 and .<=2019 or .=999*	
q702_E15e (required)	q702_E15e. How often is the item replaced?<br>*Question relevant when: not(selected(${q102_E15_full_list} , '6')) and not(selected(${q102_E15_full_list} , '99'))*	1 After each use<br>2 Daily<br>3 Weekly<br>4 Monthly<br>5 Yearly<br>6 Item is replaced after other number of uses, weeks, years, etc. (specify how often in next question)<br>99 Don't know
q702_E15e.1	q702_E15e.1. If "other number of uses", specify how often:<br>*Remember to indicate "uses," "weeks", etc. with number.*<br>*Question relevant when: not(selected(${q102_E15_full_list} , '6')) and not(selected(${q102_E15_full_list} , '99'))*	
q702_E16	***q702_E16. Pillow***<br>*Question relevant when: not(selected(${q102_E16_full_list} , '6')) and not(selected(${q102_E16_full_list} , '99'))*	
q702_E16a (required)	q702_E16a. When purchased, how many units of this item typically come in one pack, box, bottle, etc.? (e.g. 100 tablets, 1000 ml's, etc.) (Write 1 if purchased as a single item.)<br>*Smallest unit is one pillow.*<br>*Question relevant when: not(selected(${q102_E16_full_list} , '6')) and not(selected(${q102_E16_full_list} , '99'))*<br>*Response constrained to: .>=0*	
q702_E16b (required)	q702_E16b. What is the typical purchase price for that quanity of units (i.e. the quantity noted in the question above)?<br>*Question relevant when: not(selected(${q102_E16_full_list} , '6')) and not(selected(${q102_E16_full_list} , '99'))*<br>*Response constrained to: .>=0*	
q702_E16c (required)	q702_E16c. Please specify the currency for the purchase price.<br>*Question relevant when: not(selected(${q102_E16_full_list} , '6')) and not(selected(${q102_E16_full_list} , '99'))*	1 Tanzanian Shillings<br>2 US dollars<br>3 Euros<br>4 Other<br>99 Don't know
q702_E16c.1	q702_E16c.1 If other currency, specify:<br>*Leave blank if not applicable*<br>*Question relevant when: not(selected(${q102_E16_full_list} , '6')) and not(selected(${q102_E16_full_list} , '99'))*	
q702_E16d (required)	q702_E16d. Please specify the year of the purchase price.<br>*Question relevant when: not(selected(${q102_E16_full_list} , '6')) and not(selected(${q102_E16_full_list} , '99'))*<br>*Response constrained to: .>=2000 and .<=2019 or .=999*	
q702_E16e (required)	q702_E16e. How often is the item replaced?<br>*Question relevant when: not(selected(${q102_E16_full_list} , '6')) and not(selected(${q102_E16_full_list} , '99'))*	1 After each use<br>2 Daily<br>3 Weekly<br>4 Monthly<br>5 Yearly<br>6 Item is replaced after other number of uses, weeks, years, etc. (specify how often in next question)<br>99 Don't know
q702_E16e.1	q702_E16e.1. If "other number of uses", specify how often:<br>*Remember to indicate "uses," "weeks", etc. with number.*<br>*Question relevant when: not(selected(${q102_E16_full_list} , '6')) and not(selected(${q102_E16_full_list} , '99'))*	
B. Small equipment - Cost (4)<br>*Group relevant when: selected(${section_seven_skip_equ} , '1')*		
note_702_protective	***Protective supplies***	
q702_E17	***q702_E17. Apron (reusable)***<br>*Question relevant when: not(selected(${q102_E17_full_list} , '6')) and not(selected(${q102_E17_full_list} , '99'))*	

[illegible]

Field	Question	Answer
q702_E17a *(required)*	q702_E17a. When purchased, how many units of this item typically come in one pack, box, bottle, etc.? (e.g. 100 tablets, 1000 ml's, etc.) (Write 1 if purchased as a single item.)<br>*Smallest unit is one apron.*<br>*Question relevant when: not(selected(${q102_E17_full_list} , '6')) and not(selected(${q102_E17_full_list} , '99'))*<br>*Response constrained to: .>=0*	

Field	Question	Answer
q702_E17b *(required)*	q702_E17b. What is the typical purchase price for that quanity of units (i.e. the quantity noted in the question above)?<br>*Question relevant when: not(selected(${q102_E17_full_list} , '6')) and not(selected(${q102_E17_full_list} , '99'))*<br>*Response constrained to: .>=0*	
q702_E17c *(required)*	q702_E17c. Please specify the currency for the purchase price.<br>*Question relevant when: not(selected(${q102_E17_full_list} , '6')) and not(selected(${q102_E17_full_list} , '99'))*	1 Tanzanian Shillings<br>2 US dollars<br>3 Euros<br>4 Other<br>99 Don't know
q702_E17c.1	q702_E17c.1 If other currency, specify:<br>*Leave blank if not applicable*<br>*Question relevant when: not(selected(${q102_E17_full_list} , '6')) and not(selected(${q102_E17_full_list} , '99'))*	
q702_E17d *(required)*	q702_E17d. Please specify the year of the purchase price.<br>*Question relevant when: not(selected(${q102_E17_full_list} , '6')) and not(selected(${q102_E17_full_list} , '99'))*<br>*Response constrained to: .>=2000 and .<=2019 or .=999*	
q702_E17e *(required)*	q702_E17e. How often is the item replaced?<br>*Question relevant when: not(selected(${q102_E17_full_list} , '6')) and not(selected(${q102_E17_full_list} , '99'))*	1 After each use<br>2 Daily<br>3 Weekly<br>4 Monthly<br>5 Yearly<br>6 Item is replaced after other number of uses, weeks, years, etc. (specify how often in next question)<br>99 Don't know
q702_E17e.1	q702_E17e.1. If "other number of uses", specify how often:<br>*Remember to indicate "uses," "weeks", etc. with number.*<br>*Question relevant when: not(selected(${q102_E17_full_list} , '6')) and not(selected(${q102_E17_full_list} , '99'))*	
q702_E18	***q702_E18. Gown (reusable)***<br>*Question relevant when: not(selected(${q102_E18_full_list} , '6')) and not(selected(${q102_E18_full_list} , '99'))*	
q702_E18a *(required)*	q702_E18a. When purchased, how many units of this item typically come in one pack, box, bottle, etc.? (e.g. 100 tablets, 1000 ml's, etc.) (Write 1 if purchased as a single item.)<br>*Smallest unit is one gown.*<br>*Question relevant when: not(selected(${q102_E18_full_list} , '6')) and not(selected(${q102_E18_full_list} , '99'))*<br>*Response constrained to: .>=0*	
q702_E18b *(required)*	q702_E18b. What is the typical purchase price for that quanity of units (i.e. the quantity noted in the question above)?<br>*Question relevant when: not(selected(${q102_E18_full_list} , '6')) and not(selected(${q102_E18_full_list} , '99'))*<br>*Response constrained to: .>=0*	
q702_E18c *(required)*	q702_E18c. Please specify the currency for the purchase price.<br>*Question relevant when: not(selected(${q102_E18_full_list} , '6')) and not(selected(${q102_E18_full_list} , '99'))*	1 Tanzanian Shillings<br>2 US dollars<br>3 Euros<br>4 Other<br>99 Don't know
q702_E18c.1	q702_E18c.1 If other currency, specify:<br>*Leave blank if not applicable*<br>*Question relevant when: not(selected(${q102_E18_full_list} , '6')) and not(selected(${q102_E18_full_list} , '99'))*	
q702_E18d *(required)*	q702_E18d. Please specify the year of the purchase price.<br>*Question relevant when: not(selected(${q102_E18_full_list} , '6')) and not(selected(${q102_E18_full_list} , '99'))*<br>*Response constrained to: .>=2000 and .<=2019 or .=999*	
q702_E18e *(required)*	q702_E18e. How often is the item replaced?<br>*Question relevant when: not(selected(${q102_E18_full_list} , '6')) and not(selected(${q102_E18_full_list} , '99'))*	1 After each use<br>2 Daily<br>3 Weekly<br>4 Monthly<br>5 Yearly<br>6 Item is replaced after other number of uses, weeks, years, etc. (specify how often in next question)

Field	Question	Answer
		99 Don't know
q702_E18e.1	q702_E18e.1. If "other number of uses", specify how often:<br>*Remember to indicate "uses," "weeks", etc. with number.*<br>*Question relevant when: not(selected(${q102_E18_full_list} , '6')) and not(selected(${q102_E18_full_list} , '99'))*	
q702_E19	***q702_E19. Heavy duty gloves***<br>*Question relevant when: not(selected(${q102_E19_full_list} , '6')) and not(selected(${q102_E19_full_list} , '99'))*	

q702_E19a *(required)*	q702_E19a. When purchased, how many units of this item typically come in one pack, box, bottle, etc.? (e.g. 100 tablets, 1000 ml's, etc.) (Write 1 if purchased as a single item.)<br>*Smallest unit is one glove.*<br>*Question relevant when: not(selected(${q102_E19_full_list} , '6')) and not(selected(${q102_E19_full_list} , '99'))*<br>*Response constrained to: .>=0*	
q702_E19b *(required)*	q702_E19b. What is the typical purchase price for that quanity of units (i.e. the quantity noted in the question above)?<br>*Question relevant when: not(selected(${q102_E19_full_list} , '6')) and not(selected(${q102_E19_full_list} , '99'))*<br>*Response constrained to: .>=0*	
q702_E19c *(required)*	q702_E19c. Please specify the currency for the purchase price.<br>*Question relevant when: not(selected(${q102_E19_full_list} , '6')) and not(selected(${q102_E19_full_list} , '99'))*	1 Tanzanian Shillings<br>2 US dollars<br>3 Euros<br>4 Other<br>99 Don't know
q702_E19c.1	q702_E19c.1 If other currency, specify:<br>*Leave blank if not applicable*<br>*Question relevant when: not(selected(${q102_E19_full_list} , '6')) and not(selected(${q102_E19_full_list} , '99'))*	
q702_E19d *(required)*	q702_E19d. Please specify the year of the purchase price.<br>*Question relevant when: not(selected(${q102_E19_full_list} , '6')) and not(selected(${q102_E19_full_list} , '99'))*<br>*Response constrained to: .>=2000 and .<=2019 or .=999*	
q702_E19e *(required)*	q702_E19e. How often is the item replaced?<br>*Question relevant when: not(selected(${q102_E19_full_list} , '6')) and not(selected(${q102_E19_full_list} , '99'))*	1 After each use<br>2 Daily<br>3 Weekly<br>4 Monthly<br>5 Yearly<br>6 Item is replaced after other number of uses, weeks, years, etc. (specify how often in next question)<br>99 Don't know
q702_E19e.1	q702_E19e.1. If "other number of uses", specify how often:<br>*Remember to indicate "uses," "weeks", etc. with number.*<br>*Question relevant when: not(selected(${q102_E19_full_list} , '6')) and not(selected(${q102_E19_full_list} , '99'))*	
q702_E20	***q702_E20. Mask (reusable)***<br>*Question relevant when: not(selected(${q102_E19_full_list} , '6')) and not(selected(${q102_E19_full_list} , '99'))*	
q702_E20a *(required)*	q702_E20a. When purchased, how many units of this item typically come in one pack, box, bottle, etc.? (e.g. 100 tablets, 1000 ml's, etc.) (Write 1 if purchased as a single item.)<br>*Smallest unit is one mask.*<br>*Question relevant when: not(selected(${q102_E20_full_list} , '6')) and not(selected(${q102_E20_full_list} , '99'))*<br>*Response constrained to: .>=0*	
q702_E20b *(required)*	q702_E20b. What is the typical purchase price for that quanity of units (i.e. the quantity noted in the question above)?<br>*Question relevant when: not(selected(${q102_E20_full_list} , '6')) and not(selected(${q102_E20_full_list} , '99'))*<br>*Response constrained to: .>=0*	
q702_E20c *(required)*	q702_E20c. Please specify the currency for the purchase price.<br>*Question relevant when: not(selected(${q102_E20_full_list} , '6')) and not(selected(${q102_E20_full_list} , '99'))*	1 Tanzanian Shillings<br>2 US dollars<br>3 Euros<br>4 Other<br>99 Don't know
q702_E20c.1	q702_E20c.1 If other currency, specify:<br>*Leave blank if not applicable*<br>*Question relevant when: not(selected(${q102_E20_full_list} , '6')) and not(selected(${q102_E20_full_list} , '99'))*	
q702_E20d *(required)*	q702_E20d. Please specify the year of the purchase price.<br>*Question relevant when: not(selected(${q102_E20_full_list} , '6')) and not(selected(${q102_E20_full_list} , '99'))*<br>*Response constrained to: .>=2000 and .<=2019 or .=999*	
q702_E20e *(required)*	q702_E20e. How often is the item replaced?<br>*Question relevant when: not(selected(${q102_E20_full_list} , '6')) and not(selected(${q102_E20_full_list} , '99'))*	1 After each use<br>2 Daily<br>3 Weekly<br>4 Monthly<br>5 Yearly

Field	Question	Answer
		5 Yearly<br>6 Item is replaced after other number of uses, weeks, years, etc. (specify how often in next question)<br>99 Don't know

q702_E20e.1	q702_E20e.1. If "other number of uses", specify how often:<br>*Remember to indicate "uses," "weeks", etc. with number.*<br>*Question relevant when: not(selected(${q102_E20_full_list} , '6')) and not(selected(${q102_E20_full_list} , '99'))*	
q702_E21	***q702_E21. Protective glasses***<br>*Question relevant when: not(selected(${q102_E21_full_list} , '6')) and not(selected(${q102_E21_full_list} , '99'))*	
q702_E21a *(required)*	q702_E21a. When purchased, how many units of this item typically come in one pack, box, bottle, etc.? (e.g. 100 tablets, 1000 ml's, etc.) (Write 1 if purchased as a single item.)<br>*Smallest unit is one pair of glasses.*<br>*Question relevant when: not(selected(${q102_E21_full_list} , '6')) and not(selected(${q102_E21_full_list} , '99'))*<br>*Response constrained to: .>=0*	
q702_E21b *(required)*	q702_E21b. What is the typical purchase price for that quanity of units (i.e. the quantity noted in the question above)?<br>*Question relevant when: not(selected(${q102_E21_full_list} , '6')) and not(selected(${q102_E21_full_list} , '99'))*<br>*Response constrained to: .>=0*	
q702_E21c *(required)*	q702_E21c. Please specify the currency for the purchase price.<br>*Question relevant when: not(selected(${q102_E21_full_list} , '6')) and not(selected(${q102_E21_full_list} , '99'))*	1 Tanzanian Shillings<br>2 US dollars<br>3 Euros<br>4 Other<br>99 Don't know
q702_E21c.1	q702_E21c.1 If other currency, specify:<br>*Leave blank if not applicable*<br>*Question relevant when: not(selected(${q102_E21_full_list} , '6')) and not(selected(${q102_E21_full_list} , '99'))*	
q702_E21d *(required)*	q702_E21d. Please specify the year of the purchase price.<br>*Question relevant when: not(selected(${q102_E21_full_list} , '6')) and not(selected(${q102_E21_full_list} , '99'))*<br>*Response constrained to: .>=2000 and .<=2019 or .=999*	
q702_E21e *(required)*	q702_E21e. How often is the item replaced?<br>*Question relevant when: not(selected(${q102_E21_full_list} , '6')) and not(selected(${q102_E21_full_list} , '99'))*	1 After each use<br>2 Daily<br>3 Weekly<br>4 Monthly<br>5 Yearly<br>6 Item is replaced after other number of uses, weeks, years, etc. (specify how often in next question)<br>99 Don't know
q702_E21e.1	q702_E21e.1. If "other number of uses", specify how often:<br>*Remember to indicate "uses," "weeks", etc. with number.*<br>*Question relevant when: not(selected(${q102_E21_full_list} , '6')) and not(selected(${q102_E21_full_list} , '99'))*	
B. Small equipment - Cost (5)<br>*Group relevant when: selected(${section_seven_skip_equ} , '1')*		
note_702_receivers	***Receivers***	
q702_E22	***q702_E22. Kidney dish - small***<br>*Question relevant when: not(selected(${q102_E22_full_list} , '6')) and not(selected(${q102_E22_full_list} , '99'))*	
q702_E22a *(required)*	q702_E22a. When purchased, how many units of this item typically come in one pack, box, bottle, etc.? (e.g. 100 tablets, 1000 ml's, etc.) (Write 1 if purchased as a single item.)<br>*Smallest unit is one dish.*<br>*Question relevant when: not(selected(${q102_E22_full_list} , '6')) and not(selected(${q102_E22_full_list} , '99'))*<br>*Response constrained to: .>=0*	
q702_E22b *(required)*	q702_E22b. What is the typical purchase price for that quanity of units (i.e. the quantity noted in the question above)?<br>*Question relevant when: not(selected(${q102_E22_full_list} , '6')) and not(selected(${q102_E22_full_list} , '99'))*<br>*Response constrained to: .>=0*	
q702_E22c *(required)*	q702_E22c. Please specify the currency for the purchase price.<br>*Question relevant when: not(selected(${q102_E22_full_list} , '6')) and not(selected(${q102_E22_full_list} , '99'))*	1 Tanzanian Shillings<br>2 US dollars<br>3 Euros<br>4 Other<br>99 Don't know
q702_E22c.1	q702_E22c.1 If other currency, specify:<br>*Leave blank if not applicable*<br>*Question relevant when: not(selected(${q102_E22_full_list} , '6')) and not(selected(${q102_E22_full_list} , '99'))*	

Field	Question	Answer
q702_E22d *(required)*	q702_E22d. Please specify the year of the purchase price.<br>*Question relevant when: not(selected(${q102_E22_full_list} , '6')) and not(selected(${q102_E22_full_list} , '99'))*<br>*Response constrained to: .>=2000 and .<=2019 or .=999*	
q702_E22e *(required)*	q702_E22e. How often is the item replaced?<br>*Question relevant when: not(selected(${q102_E22_full_list} , '6')) and not(selected(${q102_E22_full_list} , '99'))*	1 After each use<br>2 Daily<br>3 Weekly<br>4 Monthly<br>5 Yearly<br>6 Item is replaced after other number of uses, weeks, years, etc. (specify how often in next question)<br>99 Don't know
q702_E22e.1	q702_E22e.1. If "other number of uses", specify how often:<br>*Remember to indicate "uses," "weeks", etc. with number.*<br>*Question relevant when: not(selected(${q102_E22_full_list} , '6')) and not(selected(${q102_E22_full_list} , '99'))*	
q702_E23	***q702_E23. Kidney dish - medium***<br>*Question relevant when: not(selected(${q102_E23_full_list} , '6')) and not(selected(${q102_E23_full_list} , '99'))*	
q702_E23a *(required)*	q702_E23a. When purchased, how many units of this item typically come in one pack, box, bottle, etc.? (e.g. 100 tablets, 1000 ml's, etc.) (Write 1 if purchased as a single item.)<br>*Smallest unit is one dish.*<br>*Question relevant when: not(selected(${q102_E23_full_list} , '6')) and not(selected(${q102_E23_full_list} , '99'))*<br>*Response constrained to: .>=0*	
q702_E23b *(required)*	q702_E23b. What is the typical purchase price for that quanity of units (i.e. the quantity noted in the question above)?<br>*Question relevant when: not(selected(${q102_E23_full_list} , '6')) and not(selected(${q102_E23_full_list} , '99'))*<br>*Response constrained to: .>=0*	
q702_E23c *(required)*	q702_E23c. Please specify the currency for the purchase price.<br>*Question relevant when: not(selected(${q102_E23_full_list} , '6')) and not(selected(${q102_E23_full_list} , '99'))*	1 Tanzanian Shillings<br>2 US dollars<br>3 Euros<br>4 Other<br>99 Don't know
q702_E23c.1	q702_E23c.1 If other currency, specify:<br>*Leave blank if not applicable*<br>*Question relevant when: not(selected(${q102_E23_full_list} , '6')) and not(selected(${q102_E23_full_list} , '99'))*	
q702_E23d *(required)*	q702_E23d. Please specify the year of the purchase price.<br>*Question relevant when: not(selected(${q102_E23_full_list} , '6')) and not(selected(${q102_E23_full_list} , '99'))*<br>*Response constrained to: .>=2000 and .<=2019 or .=999*	
q702_E23e *(required)*	q702_E23e. How often is the item replaced?<br>*Question relevant when: not(selected(${q102_E23_full_list} , '6')) and not(selected(${q102_E23_full_list} , '99'))*	1 After each use<br>2 Daily<br>3 Weekly<br>4 Monthly<br>5 Yearly<br>6 Item is replaced after other number of uses, weeks, years, etc. (specify how often in next question)<br>99 Don't know
q702_E23e.1	q702_E23e.1. If "other number of uses", specify how often:<br>*Remember to indicate "uses," "weeks", etc. with number.*<br>*Question relevant when: not(selected(${q102_E23_full_list} , '6')) and not(selected(${q102_E23_full_list} , '99'))*	
q702_E24	***q702_E24. Kidney dish - large***<br>*Question relevant when: not(selected(${q102_E24_full_list} , '6')) and not(selected(${q102_E24_full_list} , '99'))*	
q702_E24a *(required)*	q702_E24a. When purchased, how many units of this item typically come in one pack, box, bottle, etc.? (e.g. 100 tablets, 1000 ml's, etc.) (Write 1 if purchased as a single item.)<br>*Smallest unit is one dish.*<br>*Question relevant when: not(selected(${q102_E24_full_list} , '6')) and not(selected(${q102_E24_full_list} , '99'))*<br>*Response constrained to: .>=0*	
q702_E24b *(required)*	q702_E24b. What is the typical purchase price for that quanity of units (i.e. the quantity noted in the question above)?<br>*Question relevant when: not(selected(${q102_E24_full_list} , '6')) and not(selected(${q102_E24_full_list} , '99'))*<br>*Response constrained to: .>=0*	
q702_E24c *(required)*	q702_E24c. Please specify the currency for the purchase price.	1 Tanzanian Shillings

Field	Question	Answer
q702_E24c (required)	q702_E24c. Please specify the currency for the purchase price.<br>*Question relevant when: not(selected(${q102_E24_full_list} , '6')) and not(selected(${q102_E24_full_list} , '99'))*	1 Tanzanian Shillings<br>2 US dollars<br>3 Euros<br>4 Other<br>99 Don't know

Field	Question	Answer
q702_E24c.1	q702_E24c.1 If other currency, specify:<br>*Leave blank if not applicable*<br>*Question relevant when: not(selected(${q102_E24_full_list} , '6')) and not(selected(${q102_E24_full_list} , '99'))*	
q702_E24d (required)	q702_E24d. Please specify the year of the purchase price.<br>*Question relevant when: not(selected(${q102_E24_full_list} , '6')) and not(selected(${q102_E24_full_list} , '99'))*<br>*Response constrained to: .>=2000 and .<=2019 or .=999*	
q702_E24e (required)	q702_E24e. How often is the item replaced?<br>*Question relevant when: not(selected(${q102_E24_full_list} , '6')) and not(selected(${q102_E24_full_list} , '99'))*	1 After each use<br>2 Daily<br>3 Weekly<br>4 Monthly<br>5 Yearly<br>6 Item is replaced after other number of uses, weeks, years, etc. (specify how often in next question)<br>99 Don't know
q702_E24e.1	q702_E24e.1. If "other number of uses", specify how often:<br>*Remember to indicate "uses," "weeks", etc. with number.*<br>*Question relevant when: not(selected(${q102_E24_full_list} , '6')) and not(selected(${q102_E24_full_list} , '99'))*	
q702_E25	***q702_E25. Receiving bowl - small***<br>*Question relevant when: not(selected(${q102_E25_full_list} , '6')) and not(selected(${q102_E25_full_list} , '99'))*	
q702_E25a (required)	q702_E25a. When purchased, how many units of this item typically come in one pack, box, bottle, etc.? (e.g. 100 tablets, 1000 ml's, etc.) (Write 1 if purchased as a single item.)<br>*Smallest unit is one dish.*<br>*Question relevant when: not(selected(${q102_E25_full_list} , '6')) and not(selected(${q102_E25_full_list} , '99'))*<br>*Response constrained to: .>=0*	
q702_E25b (required)	q702_E25b. What is the typical purchase price for that quanity of units (i.e. the quantity noted in the question above)?<br>*Question relevant when: not(selected(${q102_E25_full_list} , '6')) and not(selected(${q102_E25_full_list} , '99'))*<br>*Response constrained to: .>=0*	
q702_E25c (required)	q702_E25c. Please specify the currency for the purchase price.<br>*Question relevant when: not(selected(${q102_E25_full_list} , '6')) and not(selected(${q102_E25_full_list} , '99'))*	1 Tanzanian Shillings<br>2 US dollars<br>3 Euros<br>4 Other<br>99 Don't know
q702_E25c.1	q702_E25c.1 If other currency, specify:<br>*Leave blank if not applicable*<br>*Question relevant when: not(selected(${q102_E25_full_list} , '6')) and not(selected(${q102_E25_full_list} , '99'))*	
q702_E25d (required)	q702_E25d. Please specify the year of the purchase price.<br>*Question relevant when: not(selected(${q102_E25_full_list} , '6')) and not(selected(${q102_E25_full_list} , '99'))*<br>*Response constrained to: .>=2000 and .<=2019 or .=999*	
q702_E25e (required)	q702_E25e. How often is the item replaced?<br>*Question relevant when: not(selected(${q102_E25_full_list} , '6')) and not(selected(${q102_E25_full_list} , '99'))*	1 After each use<br>2 Daily<br>3 Weekly<br>4 Monthly<br>5 Yearly<br>6 Item is replaced after other number of uses, weeks, years, etc. (specify how often in next question)<br>99 Don't know
q702_E25e.1	q702_E25e.1. If "other number of uses", specify how often:<br>*Remember to indicate "uses," "weeks", etc. with number.*<br>*Question relevant when: not(selected(${q102_E25_full_list} , '6')) and not(selected(${q102_E25_full_list} , '99'))*	
q702_E26	***q702_E26. Receiving bowl - medium***<br>*Question relevant when: not(selected(${q102_E26_full_list} , '6')) and not(selected(${q102_E26_full_list} , '99'))*	
q702_E26a (required)	q702_E26a. When purchased, how many units of this item typically come in one pack, box, bottle, etc.? (e.g. 100 tablets, 1000 ml's, etc.) (Write 1 if purchased as a single item.)<br>*Smallest unit is one dish.*	

Field	Question	Answer
	Question relevant when: not(selected(${q102_E26_full_list} , '6')) and not(selected(${q102_E26_full_list} , '99'))<br>*Response constrained to: .>=0*	
q702_E26b *(required)*	q702_E26b. What is the typical purchase price for that quanity of units (i.e. the quantity noted in the question above)?<br>*Question relevant when: not(selected(${q102_E26_full_list} , '6')) and not(selected(${q102_E26_full_list} , '99'))*<br>*Response constrained to: .>=0*	
q702_E26c *(required)*	q702_E26c. Please specify the currency for the purchase price.<br>*Question relevant when: not(selected(${q102_E26_full_list} , '6')) and not(selected(${q102_E26_full_list} , '99'))*	1 Tanzanian Shillings<br>2 US dollars<br>3 Euros<br>4 Other<br>99 Don't know
q702_E26c.1	q702_E26c.1 If other currency, specify:<br>*Leave blank if not applicable*<br>*Question relevant when: not(selected(${q102_E26_full_list} , '6')) and not(selected(${q102_E26_full_list} , '99'))*	
q702_E26d *(required)*	q702_E26d. Please specify the year of the purchase price.<br>*Question relevant when: not(selected(${q102_E26_full_list} , '6')) and not(selected(${q102_E26_full_list} , '99'))*<br>*Response constrained to: .>=2000 and .<=2019 or .=999*	
q702_E26e *(required)*	q702_E26e. How often is the item replaced?<br>*Question relevant when: not(selected(${q102_E26_full_list} , '6')) and not(selected(${q102_E26_full_list} , '99'))*	1 After each use<br>2 Daily<br>3 Weekly<br>4 Monthly<br>5 Yearly<br>6 Item is replaced after other number of uses, weeks, years, etc. (specify how often in next question)<br>99 Don't know
q702_E26e.1	q702_E26e.1. If "other number of uses", specify how often:<br>*Remember to indicate "uses," "weeks", etc. with number.*<br>*Question relevant when: not(selected(${q102_E26_full_list} , '6')) and not(selected(${q102_E26_full_list} , '99'))*	
q702_E27	***q702_E27. Receiving bowl - large***<br>*Question relevant when: not(selected(${q102_E27_full_list} , '6')) and not(selected(${q102_E27_full_list} , '99'))*	
q702_E27a *(required)*	q702_E27a. When purchased, how many units of this item typically come in one pack, box, bottle, etc.? (e.g. 100 tablets, 1000 ml's, etc.) (Write 1 if purchased as a single item.)<br>*Smallest unit is one dish.*<br>*Question relevant when: not(selected(${q102_E27_full_list} , '6')) and not(selected(${q102_E27_full_list} , '99'))*<br>*Response constrained to: .>=0*	
q702_E27b *(required)*	q702_E27b. What is the typical purchase price for that quanity of units (i.e. the quantity noted in the question above)?<br>*Question relevant when: not(selected(${q102_E27_full_list} , '6')) and not(selected(${q102_E27_full_list} , '99'))*<br>*Response constrained to: .>=0*	
q702_E27c *(required)*	q702_E27c. Please specify the currency for the purchase price.<br>*Question relevant when: not(selected(${q102_E27_full_list} , '6')) and not(selected(${q102_E27_full_list} , '99'))*	1 Tanzanian Shillings<br>2 US dollars<br>3 Euros<br>4 Other<br>99 Don't know
q702_E27c.1	q702_E27c.1 If other currency, specify:<br>*Leave blank if not applicable*<br>*Question relevant when: not(selected(${q102_E27_full_list} , '6')) and not(selected(${q102_E27_full_list} , '99'))*	
q702_E27d *(required)*	q702_E27d. Please specify the year of the purchase price.<br>*Question relevant when: not(selected(${q102_E27_full_list} , '6')) and not(selected(${q102_E27_full_list} , '99'))*<br>*Response constrained to: .>=2000 and .<=2019 or .=999*	
q702_E27e *(required)*	q702_E27e. How often is the item replaced?<br>*Question relevant when: not(selected(${q102_E27_full_list} , '6')) and not(selected(${q102_E27_full_list} , '99'))*	1 After each use<br>2 Daily<br>3 Weekly<br>4 Monthly<br>5 Yearly<br>6 Item is replaced after other number of uses, weeks, years, etc. (specify how often in next question)<br>99 Don't know
q702_E27e.1	q702_E27e.1. If "other number of uses", specify how often:<br>*Remember to indicate "uses," "weeks", etc. with number.*<br>[illegible]	

Question relevant when: not(selected(${q102_E27_full_list} , '6')) and not(selected(${q102_E27_full_list} , '99'))

8. Small equipment - Cost (6)

Group relevant when: selected(${section_seven_skip_equ} , '1')

Field	Question	Answer
note_702_surgery	***Surgery***	
q702_E28	***q702_E28. Scalpel, reusable***<br>*Question relevant when: not(selected(${q102_E28_full_list} , '6')) and not(selected(${q102_E28_full_list} , '99'))*	

q702_E28a *(required)*	q702_E28a. When purchased, how many units of this item typically come in one pack, box, bottle, etc.? (e.g. 100 tablets, 1000 ml's, etc.) (Write 1 if purchased as a single item.)<br>*Smallest unit is one scalpel.*<br>*Question relevant when: not(selected(${q102_E28_full_list} , '6')) and not(selected(${q102_E28_full_list} , '99'))*<br>*Response constrained to: .>=0*	
q702_E28b *(required)*	q702_E28b. What is the typical purchase price for that quanity of units (i.e. the quantity noted in the question above)?<br>*Question relevant when: not(selected(${q102_E28_full_list} , '6')) and not(selected(${q102_E28_full_list} , '99'))*<br>*Response constrained to: .>=0*	
q702_E28c *(required)*	q702_E28c. Please specify the currency for the purchase price.<br>*Question relevant when: not(selected(${q102_E28_full_list} , '6')) and not(selected(${q102_E28_full_list} , '99'))*	1 Tanzanian Shillings<br>2 US dollars<br>3 Euros<br>4 Other<br>99 Don't know
q702_E28c.1	q702_E28c.1 If other currency, specify:<br>*Leave blank if not applicable*<br>*Question relevant when: not(selected(${q102_E28_full_list} , '6')) and not(selected(${q102_E28_full_list} , '99'))*	
q702_E28d *(required)*	q702_E28d. Please specify the year of the purchase price.<br>*Question relevant when: not(selected(${q102_E28_full_list} , '6')) and not(selected(${q102_E28_full_list} , '99'))*<br>*Response constrained to: .>=2000 and .<=2019 or .=999*	
q702_E28e *(required)*	q702_E28e. How often is the item replaced?<br>*Question relevant when: not(selected(${q102_E28_full_list} , '6')) and not(selected(${q102_E28_full_list} , '99'))*	1 After each use<br>2 Daily<br>3 Weekly<br>4 Monthly<br>5 Yearly<br>6 Item is replaced after other number of uses, weeks, years, etc. (specify how often in next question)<br>99 Don't know
q702_E28e.1	q702_E28e.1. If "other number of uses", specify how often:<br>*Remember to indicate "uses," "weeks", etc. with number.*<br>*Question relevant when: not(selected(${q102_E28_full_list} , '6')) and not(selected(${q102_E28_full_list} , '99'))*	
q702_E29	***q702_E29. Surgical scissors***<br>*Question relevant when: not(selected(${q102_E29_full_list} , '6')) and not(selected(${q102_E29_full_list} , '99'))*	
q702_E29a *(required)*	q702_E29a. When purchased, how many units of this item typically come in one pack, box, bottle, etc.? (e.g. 100 tablets, 1000 ml's, etc.) (Write 1 if purchased as a single item.)<br>*Smallest unit is one pair of scissors.*<br>*Question relevant when: not(selected(${q102_E29_full_list} , '6')) and not(selected(${q102_E29_full_list} , '99'))*<br>*Response constrained to: .>=0*	
q702_E29b *(required)*	q702_E29b. What is the typical purchase price for that quanity of units (i.e. the quantity noted in the question above)?<br>*Question relevant when: not(selected(${q102_E29_full_list} , '6')) and not(selected(${q102_E29_full_list} , '99'))*<br>*Response constrained to: .>=0*	
q702_E29c *(required)*	q702_E29c. Please specify the currency for the purchase price.<br>*Question relevant when: not(selected(${q102_E29_full_list} , '6')) and not(selected(${q102_E29_full_list} , '99'))*	1 Tanzanian Shillings<br>2 US dollars<br>3 Euros<br>4 Other<br>99 Don't know
q702_E29c.1	q702_E29c.1 If other currency, specify:<br>*Leave blank if not applicable*<br>*Question relevant when: not(selected(${q102_E29_full_list} , '6')) and not(selected(${q102_E29_full_list} , '99'))*	
q702_E29d *(required)*	q702_E29d. Please specify the year of the purchase price.<br>*Question relevant when: not(selected(${q102_E29_full_list} , '6')) and not(selected(${q102_E29_full_list} , '99'))*<br>*Response constrained to: .>=2000 and .<=2019 or .=999*	
q702_E29e *(required)*	q702_E29e. How often is the item replaced?<br>*Question relevant when: not(selected(${q102_E29_full_list} , '6')) and not(selected(${q102_E29_full_list} , '99'))*	1 After each use<br>2 Daily<br>3 Weekly<br>4 Monthly<br>5 Yearly

Field	Question	Answer
		5 Yearly<br>6 Item is replaced after other number of uses, weeks, years, etc. (specify how often in next question)<br>99 Don't know

Field	Question	Answer
q702_E29e.1	q702_E29e.1. If "other number of uses", specify how often:<br>*Remember to indicate "uses," "weeks", etc. with number.*<br>*Question relevant when: not(selected(${q102_E29_full_list} , '6')) and not(selected(${q102_E29_full_list} , '99'))*	
B. Small equipment - Cost (7)<br>*Group relevant when: selected(${section_seven_skip_equ} , '1')*		
note_702_other	***Other small equipment***	
q702_E30	***q702_E30. Head lamp/batteries***<br>*Question relevant when: not(selected(${q102_E30_full_list} , '6')) and not(selected(${q102_E30_full_list} , '99'))*	
q702_E30a *(required)*	q702_E30a. When purchased, how many units of this item typically come in one pack, box, bottle, etc.? (e.g. 100 tablets, 1000 ml's, etc.) (Write 1 if purchased as a single item.)<br>*Smallest unit is one lamp/battery set.*<br>*Question relevant when: not(selected(${q102_E30_full_list} , '6')) and not(selected(${q102_E30_full_list} , '99'))*<br>*Response constrained to: .>=0*	
q702_E30b *(required)*	q702_E30b. What is the typical purchase price for that quanity of units (i.e. the quantity noted in the question above)?<br>*Question relevant when: not(selected(${q102_E30_full_list} , '6')) and not(selected(${q102_E30_full_list} , '99'))*<br>*Response constrained to: .>=0*	
q702_E30c *(required)*	q702_E30c. Please specify the currency for the purchase price.<br>*Question relevant when: not(selected(${q102_E30_full_list} , '6')) and not(selected(${q102_E30_full_list} , '99'))*	1 Tanzanian Shillings<br>2 US dollars<br>3 Euros<br>4 Other<br>99 Don't know
q702_E30c.1	q702_E30c.1 If other currency, specify:<br>*Leave blank if not applicable*<br>*Question relevant when: not(selected(${q102_E30_full_list} , '6')) and not(selected(${q102_E30_full_list} , '99'))*	
q702_E30d *(required)*	q702_E30d. Please specify the year of the purchase price.<br>*Question relevant when: not(selected(${q102_E30_full_list} , '6')) and not(selected(${q102_E30_full_list} , '99'))*<br>*Response constrained to: .>=2000 and .<=2019 or .=999*	
q702_E30e *(required)*	q702_E30e. How often is the item replaced?<br>*Question relevant when: not(selected(${q102_E30_full_list} , '6')) and not(selected(${q102_E30_full_list} , '99'))*	1 After each use<br>2 Daily<br>3 Weekly<br>4 Monthly<br>5 Yearly<br>6 Item is replaced after other number of uses, weeks, years, etc. (specify how often in next question)<br>99 Don't know
q702_E30e.1	q702_E30e.1. If "other number of uses", specify how often:<br>*Remember to indicate "uses," "weeks", etc. with number.*<br>*Question relevant when: not(selected(${q102_E30_full_list} , '6')) and not(selected(${q102_E30_full_list} , '99'))*	
q702_E31	***q702_E31. Metal catheter***<br>*Question relevant when: not(selected(${q102_E31_full_list} , '6')) and not(selected(${q102_E31_full_list} , '99'))*	
q702_E31a *(required)*	q702_E31a. When purchased, how many units of this item typically come in one pack, box, bottle, etc.? (e.g. 100 tablets, 1000 ml's, etc.) (Write 1 if purchased as a single item.)<br>*Smallest unit is one catheter.*<br>*Question relevant when: not(selected(${q102_E31_full_list} , '6')) and not(selected(${q102_E31_full_list} , '99'))*<br>*Response constrained to: .>=0*	
q702_E31b *(required)*	q702_E31b. What is the typical purchase price for that quanity of units (i.e. the quantity noted in the question above)?<br>*Question relevant when: not(selected(${q102_E31_full_list} , '6')) and not(selected(${q102_E31_full_list} , '99'))*<br>*Response constrained to: .>=0*	
q702_E31c *(required)*	q702_E31c. Please specify the currency for the purchase price.<br>*Question relevant when: not(selected(${q102_E31_full_list} , '6')) and not(selected(${q102_E31_full_list} , '99'))*	1 Tanzanian Shillings<br>2 US dollars<br>3 Euros<br>4 Other<br>99 Don't know
q702_E31c.1	q702_E31c.1 If other currency, specify:<br>*Leave blank if not applicable*<br>*Question relevant when: not(selected(${q102_E31_full_list} , '6')) and not(selected(${q102_E31_full_list} , '99'))*	

Field	Question	Answer
q702_E31d *(required)*	q702_E31d. Please specify the year of the purchase price.<br>*Question relevant when: not(selected(${q102_E31_full_list} , '6')) and not(selected(${q102_E31_full_list} , '99'))*<br>*Response constrained to: .>=2000 and .<=2019 or .=999*	
q702_E31e *(required)*	q702_E31e. How often is the item replaced?<br>*Question relevant when: not(selected(${q102_E31_full_list} , '6')) and not(selected(${q102_E31_full_list} , '99'))*	1 After each use<br>2 Daily<br>3 Weekly<br>4 Monthly<br>5 Yearly<br>6 Item is replaced after other number of uses, weeks, years, etc. (specify how often in next question)<br>99 Don't know
q702_E31e.1	q702_E31e.1. If "other number of uses", specify how often:<br>*Remember to indicate "uses," "weeks", etc. with number.*<br>*Question relevant when: not(selected(${q102_E31_full_list} , '6')) and not(selected(${q102_E31_full_list} , '99'))*	
q702_E32	***q702_E32. Urine collection flask***<br>*Question relevant when: not(selected(${q102_E32_full_list} , '6')) and not(selected(${q102_E32_full_list} , '99'))*	
q702_E32a *(required)*	q702_E32a. When purchased, how many units of this item typically come in one pack, box, bottle, etc.? (e.g. 100 tablets, 1000 ml's, etc.) (Write 1 if purchased as a single item.)<br>*Smallest unit is one flask.*<br>*Question relevant when: not(selected(${q102_E32_full_list} , '6')) and not(selected(${q102_E32_full_list} , '99'))*<br>*Response constrained to: .>=0*	
q702_E32b *(required)*	q702_E32b. What is the typical purchase price for that quanity of units (i.e. the quantity noted in the question above)?<br>*Question relevant when: not(selected(${q102_E32_full_list} , '6')) and not(selected(${q102_E32_full_list} , '99'))*<br>*Response constrained to: .>=0*	
q702_E32c *(required)*	q702_E32c. Please specify the currency for the purchase price.<br>*Question relevant when: not(selected(${q102_E32_full_list} , '6')) and not(selected(${q102_E32_full_list} , '99'))*	1 Tanzanian Shillings<br>2 US dollars<br>3 Euros<br>4 Other<br>99 Don't know
q702_E32c.1	q702_E32c.1 If other currency, specify:<br>*Leave blank if not applicable*<br>*Question relevant when: not(selected(${q102_E32_full_list} , '6')) and not(selected(${q102_E32_full_list} , '99'))*	
q702_E32d *(required)*	q702_E32d. Please specify the year of the purchase price.<br>*Question relevant when: not(selected(${q102_E32_full_list} , '6')) and not(selected(${q102_E32_full_list} , '99'))*<br>*Response constrained to: .>=2000 and .<=2019 or .=999*	
q702_E32e *(required)*	q702_E32e. How often is the item replaced?<br>*Question relevant when: not(selected(${q102_E32_full_list} , '6')) and not(selected(${q102_E32_full_list} , '99'))*	1 After each use<br>2 Daily<br>3 Weekly<br>4 Monthly<br>5 Yearly<br>6 Item is replaced after other number of uses, weeks, years, etc. (specify how often in next question)<br>99 Don't know
q702_E32e.1	q702_E32e.1. If "other number of uses", specify how often:<br>*Remember to indicate "uses," "weeks", etc. with number.*<br>*Question relevant when: not(selected(${q102_E32_full_list} , '6')) and not(selected(${q102_E32_full_list} , '99'))*	
B. Small equipment - Cost (8)<br>*Group relevant when: selected(${section_seven_skip_equ} , '1')*		
note_702_other_suggestion	***Other equipment - suggestions?***	
q702_E33	***q702_E33. Equipment other 1: "[q102_E33_full_list_other]"***<br>*Question relevant when: not(selected(${q102_E33_full_list} , '6')) and not(selected(${q102_E33_full_list} , '99')) and string-length(${q102_E33_full_list_other}) >0*	
q702_E33a *(required)*	q702_E33a. When purchased, how many units of this item typically come in one pack, box, bottle, etc.? (e.g. 100 tablets, 1000 ml's, etc.) (Write 1 if purchased as a single item.)<br>*Question relevant when: not(selected(${q102_E33_full_list} , '6')) and not(selected(${q102_E33_full_list} , '99')) and string-length(${q102_E33_full_list_other}) >0*<br>[illegible]	

Response constrained to: .>=0

Field	Question	Answer
q702_E33b *(required)*	q702_E33b. What is the typical purchase price for that quanity of units (i.e. the quantity noted in the question above)?<br>*Question relevant when: not(selected(${q102_E33_full_list} , '6')) and not(selected(${q102_E33_full_list} , '99')) and string-length(${q102_E33_full_list_other}) >0*<br>*Response constrained to: .>=0*	
q702_E33c *(required)*	q702_E33c. Please specify the currency for the purchase price.<br>*Question relevant when: not(selected(${q102_E33_full_list} , '6')) and not(selected(${q102_E33_full_list} , '99')) and string-length(${q102_E33_full_list_other}) >0*	1 Tanzanian Shillings<br>2 US dollars<br>3 Euros<br>4 Other<br>99 Don't know
q702_E33c.1	q702_E33c.1 If other currency, specify:<br>*Leave blank if not applicable*<br>*Question relevant when: not(selected(${q102_E33_full_list} , '6')) and not(selected(${q102_E33_full_list} , '99')) and string-length(${q102_E33_full_list_other}) >0*	
q702_E33d *(required)*	q702_E33d. Please specify the year of the purchase price.<br>*Question relevant when: not(selected(${q102_E33_full_list} , '6')) and not(selected(${q102_E33_full_list} , '99')) and string-length(${q102_E33_full_list_other}) >0*<br>*Response constrained to: .>=2000 and .<=2019 or .=999*	
q702_E33e *(required)*	q702_E33e. How often is the item replaced?<br>*Question relevant when: not(selected(${q102_E33_full_list} , '6')) and not(selected(${q102_E33_full_list} , '99')) and string-length(${q102_E33_full_list_other}) >0*	1 After each use<br>2 Daily<br>3 Weekly<br>4 Monthly<br>5 Yearly<br>6 Item is replaced after other number of uses, weeks, years, etc. (specify how often in next question)<br>99 Don't know
q702_E33e.1	q702_E33e.1. If "other number of uses", specify how often:<br>*Remember to indicate "uses," "weeks", etc. with number.*<br>*Question relevant when: not(selected(${q102_E33_full_list} , '6')) and not(selected(${q102_E33_full_list} , '99')) and string-length(${q102_E33_full_list_other}) >0*	
q702_E34	***q702_E34. Equipment other 2: "[q102_E34_full_list_other]"***<br>*Question relevant when: not(selected(${q102_E34_full_list} , '6')) and not(selected(${q102_E34_full_list} , '99')) and string-length(${q102_E34_full_list_other}) >0*	
q702_E34a *(required)*	q702_E34a. When purchased, how many units of this item typically come in one pack, box, bottle, etc.? (e.g. 100 tablets, 1000 ml's, etc.) (Write 1 if purchased as a single item.)<br>*Question relevant when: not(selected(${q102_E34_full_list} , '6')) and not(selected(${q102_E34_full_list} , '99')) and string-length(${q102_E34_full_list_other}) >0*<br>*Response constrained to: .>=0*	
q702_E34b *(required)*	q702_E34b. What is the typical purchase price for that quanity of units (i.e. the quantity noted in the question above)?<br>*Question relevant when: not(selected(${q102_E34_full_list} , '6')) and not(selected(${q102_E34_full_list} , '99')) and string-length(${q102_E34_full_list_other}) >0*<br>*Response constrained to: .>=0*	
q702_E34c *(required)*	q702_E34c. Please specify the currency for the purchase price.<br>*Question relevant when: not(selected(${q102_E34_full_list} , '6')) and not(selected(${q102_E34_full_list} , '99')) and string-length(${q102_E34_full_list_other}) >0*	1 Tanzanian Shillings<br>2 US dollars<br>3 Euros<br>4 Other<br>99 Don't know
q702_E34c.1	q702_E34c.1 If other currency, specify:<br>*Leave blank if not applicable*<br>*Question relevant when: not(selected(${q102_E34_full_list} , '6')) and not(selected(${q102_E34_full_list} , '99')) and string-length(${q102_E34_full_list_other}) >0*	
q702_E34d *(required)*	q702_E34d. Please specify the year of the purchase price.<br>*Question relevant when: not(selected(${q102_E34_full_list} , '6')) and not(selected(${q102_E34_full_list} , '99')) and string-length(${q102_E34_full_list_other}) >0*<br>*Response constrained to: .>=2000 and .<=2019 or .=999*	
q702_E34e *(required)*	q702_E34e. How often is the item replaced?<br>*Question relevant when: not(selected(${q102_E34_full_list} , '6')) and not(selected(${q102_E34_full_list} , '99')) and string-length(${q102_E34_full_list_other}) >0*	1 After each use<br>2 Daily<br>3 Weekly<br>4 Monthly<br>5 Yearly

Field	Question	Answer
		6 Item is replaced after other number of uses, weeks, years, etc. (specify how often in next question)<br>99 Don't know

Field	Question	Answer
q702_E34e.1	q702_E34e.1. If "other number of uses", specify how often:<br>*Remember to indicate "uses," "weeks", etc. with number.*<br>*Question relevant when: not(selected(${q102_E34_full_list} , '6')) and not(selected(${q102_E34_full_list} , '99')) and string-length(${q102_E34_full_list_other}) >0*	
q702_E35	***q702_E35. Equipment other 3: "[q102_E35_full_list_other]"***<br>*Question relevant when: not(selected(${q102_E35_full_list} , '6')) and not(selected(${q102_E35_full_list} , '99')) and string-length(${q102_E35_full_list_other}) >0*	
q702_E35a *(required)*	q702_E35a. When purchased, how many units of this item typically come in one pack, box, bottle, etc.? (e.g. 100 tablets, 1000 ml's, etc.) (Write 1 if purchased as a single item.)<br>*Question relevant when: not(selected(${q102_E35_full_list} , '6')) and not(selected(${q102_E35_full_list} , '99')) and string-length(${q102_E35_full_list_other}) >0*<br>*Response constrained to: .>=0*	
q702_E35b *(required)*	q702_E35b. What is the typical purchase price for that quanity of units (i.e. the quantity noted in the question above)?<br>*Question relevant when: not(selected(${q102_E35_full_list} , '6')) and not(selected(${q102_E35_full_list} , '99')) and string-length(${q102_E35_full_list_other}) >0*<br>*Response constrained to: .>=0*	
q702_E35c *(required)*	q702_E35c. Please specify the currency for the purchase price.<br>*Question relevant when: not(selected(${q102_E35_full_list} , '6')) and not(selected(${q102_E35_full_list} , '99')) and string-length(${q102_E35_full_list_other}) >0*	1 Tanzanian Shillings<br>2 US dollars<br>3 Euros<br>4 Other<br>99 Don't know
q702_E35c.1	q702_E35c.1 If other currency, specify:<br>*Leave blank if not applicable*<br>*Question relevant when: not(selected(${q102_E35_full_list} , '6')) and not(selected(${q102_E35_full_list} , '99')) and string-length(${q102_E35_full_list_other}) >0*	
q702_E35d *(required)*	q702_E35d. Please specify the year of the purchase price.<br>*Question relevant when: not(selected(${q102_E35_full_list} , '6')) and not(selected(${q102_E35_full_list} , '99')) and string-length(${q102_E35_full_list_other}) >0*<br>*Response constrained to: .>=2000 and .<=2019 or .=999*	
q702_E35e *(required)*	q702_E35e. How often is the item replaced?<br>*Question relevant when: not(selected(${q102_E35_full_list} , '6')) and not(selected(${q102_E35_full_list} , '99')) and string-length(${q102_E35_full_list_other}) >0*	1 After each use<br>2 Daily<br>3 Weekly<br>4 Monthly<br>5 Yearly<br>6 Item is replaced after other number of uses, weeks, years, etc. (specify how often in next question)<br>99 Don't know
q702_E35e.1	q702_E35e.1. If "other number of uses", specify how often:<br>*Remember to indicate "uses," "weeks", etc. with number.*<br>*Question relevant when: not(selected(${q102_E35_full_list} , '6')) and not(selected(${q102_E35_full_list} , '99')) and string-length(${q102_E35_full_list_other}) >0*	
q702_E36	***q702_E36. Equipment other 4: "[q102_E36_full_list_other]"***<br>*Question relevant when: not(selected(${q102_E36_full_list} , '6')) and not(selected(${q102_E36_full_list} , '99')) and string-length(${q102_E36_full_list_other}) >0*	
q702_E36a *(required)*	q702_E36a. When purchased, how many units of this item typically come in one pack, box, bottle, etc.? (e.g. 100 tablets, 1000 ml's, etc.) (Write 1 if purchased as a single item.)<br>*Question relevant when: not(selected(${q102_E36_full_list} , '6')) and not(selected(${q102_E36_full_list} , '99')) and string-length(${q102_E36_full_list_other}) >0*<br>*Response constrained to: .>=0*	
q702_E36b *(required)*	q702_E36b. What is the typical purchase price for that quanity of units (i.e. the quantity noted in the question above)?<br>*Question relevant when: not(selected(${q102_E36_full_list} , '6')) and not(selected(${q102_E36_full_list} , '99')) and string-length(${q102_E36_full_list_other}) >0*<br>*Response constrained to: .>=0*	
q702_E36c *(required)*	q702_E36c. Please specify the currency for the purchase price.<br>*Question relevant when: not(selected(${q102_E36_full_list} , '6')) and not(selected(${q102_E36_full_list} , '99')) and*	1 Tanzanian Shillings<br>2 US dollars

Field	Question	Answer
	string-length(${q102_E36_full_list_other}) >0	3 Euros<br>4 Other<br>99 Don't know
q702_E36c.1	q702_E36c.1 If other currency, specify:<br>*Leave blank if not applicable*<br>*Question relevant when: not(selected(${q102_E36_full_list} , '6')) and not(selected(${q102_E36_full_list} , '99')) and string-length(${q102_E36_full_list_other}) >0*	

Field	Question	Answer
q702_E36d *(required)*	q702_E36d. Please specify the year of the purchase price.<br>*Question relevant when: not(selected(${q102_E36_full_list} , '6')) and not(selected(${q102_E36_full_list} , '99')) and string-length(${q102_E36_full_list_other}) >0*<br>*Response constrained to: .>=2000 and .<=2019 or .=999*	
q702_E36e *(required)*	q702_E36e. How often is the item replaced?<br>*Question relevant when: not(selected(${q102_E36_full_list} , '6')) and not(selected(${q102_E36_full_list} , '99')) and string-length(${q102_E36_full_list_other}) >0*	1 After each use<br>2 Daily<br>3 Weekly<br>4 Monthly<br>5 Yearly<br>6 Item is replaced after other number of uses, weeks, years, etc. (specify how often in next question)<br>99 Don't know
q702_E36e.1	q702_E36e.1. If "other number of uses", specify how often:<br>*Remember to indicate "uses," "weeks", etc. with number.*<br>*Question relevant when: not(selected(${q102_E36_full_list} , '6')) and not(selected(${q102_E36_full_list} , '99')) and string-length(${q102_E36_full_list_other}) >0*	
q702_E37	***q702_E37. Equipment other 5: "[q102_E37_full_list_other]"***<br>*Question relevant when: not(selected(${q102_E37_full_list} , '6')) and not(selected(${q102_E37_full_list} , '99')) and string-length(${q102_E37_full_list_other}) >0*	
q702_E37a *(required)*	q702_E37a. When purchased, how many units of this item typically come in one pack, box, bottle, etc.? (e.g. 100 tablets, 1000 ml's, etc.) (Write 1 if purchased as a single item.)<br>*Question relevant when: not(selected(${q102_E37_full_list} , '6')) and not(selected(${q102_E37_full_list} , '99')) and string-length(${q102_E37_full_list_other}) >0*<br>*Response constrained to: .>=0*	
q702_E37b *(required)*	q702_E37b. What is the typical purchase price for that quanity of units (i.e. the quantity noted in the question above)?<br>*Question relevant when: not(selected(${q102_E37_full_list} , '6')) and not(selected(${q102_E37_full_list} , '99')) and string-length(${q102_E37_full_list_other}) >0*<br>*Response constrained to: .>=0*	
q702_E37c *(required)*	q702_E37c. Please specify the currency for the purchase price.<br>*Question relevant when: not(selected(${q102_E37_full_list} , '6')) and not(selected(${q102_E37_full_list} , '99')) and string-length(${q102_E37_full_list_other}) >0*	1 Tanzanian Shillings<br>2 US dollars<br>3 Euros<br>4 Other<br>99 Don't know
q702_E37c.1	q702_E37c.1 If other currency, specify:<br>*Leave blank if not applicable*<br>*Question relevant when: not(selected(${q102_E37_full_list} , '6')) and not(selected(${q102_E37_full_list} , '99')) and string-length(${q102_E37_full_list_other}) >0*	
q702_E37d *(required)*	q702_E37d. Please specify the year of the purchase price.<br>*Question relevant when: not(selected(${q102_E37_full_list} , '6')) and not(selected(${q102_E37_full_list} , '99')) and string-length(${q102_E37_full_list_other}) >0*<br>*Response constrained to: .>=2000 and .<=2019 or .=999*	
q702_E37e *(required)*	q702_E37e. How often is the item replaced?<br>*Question relevant when: not(selected(${q102_E37_full_list} , '6')) and not(selected(${q102_E37_full_list} , '99')) and string-length(${q102_E37_full_list_other}) >0*	1 After each use<br>2 Daily<br>3 Weekly<br>4 Monthly<br>5 Yearly<br>6 Item is replaced after other number of uses, weeks, years, etc. (specify how often in next question)<br>99 Don't know
q702_E37e.1	q702_E37e.1. If "other number of uses", specify how often:<br>*Remember to indicate "uses," "weeks", etc. with number.*<br>*Question relevant when: not(selected(${q102_E37_full_list} , '6')) and not(selected(${q102_E37_full_list} , '99')) and string-length(${q102_E37_full_list_other}) >0*	

Field	Question	Answer
GPS_QB2_equ *(required)*	GPS location capture<br>*Press the button to capture the GPS location at this point in the survey.*	
q705_time_end_qb_equ *(required)*	ENTER THE END TIME OF THE INTERVIEW<br>*NB: The default is the current time.*	

thank_you	**END OF INTERVIEW QUESTIONS.**<br><br>**THANK THE PARTICIPANT FOR THEIR TIME.**<br><br>**THEN COMPLETE THE FOLLOWING TWO ITEMS.**	
q706_interviewer_comments_equ	INTERVIEW COMMENTS - ENTER ANY RELEVANT NOTES AT THE END OF THE INTERVIEW. PLEASE ALSO EXPLAIN HOW COSTS WERE COLLECTED, I.E. VIA INTERVIEW, SITE REPRESENTATIVE FILLING OUT FORM, OR EXTRACTION FROM RECORDS.	
End_note	**End of Questionnaire B part 2**<br><br>ON THE NEXT PAGE YOU'LL FIND THIS FACILITY'S ID NUMBER. TAKE NOTE OF THE NUMBER, AND THEN, ON THE FOLLOWING PAGE, NAME THIS FORM BY ADDING THE FACILITY ID BEFORE THE FORM NAME.<br><br>FOR EXAMPLE, "20 Tanzania PAC cost study – Quest. B part 2 equipment".<br><br>IF THIS INTERVIEW IS COMPLETE - I.E. THE RESPONDENT HAS ANSWERED ALL OF THE QUESTIONS THAT THEY CAN ON THIS FORM, THEN LEAVE THE DEFAULT BOX CHECKED. IF THE FORM IS NOT FINAL FOR THIS FACILITY, UNTICK THE BOX.<br><br>THEN SAVE AND EXIT.	
facility_id_equ2	The ID for this facility is '[facility_id_equ]".	
