# Supplementary material for: The health system costs of post abortion care in Tanzania
Source: BMC Health Serv Res. 2021 Jul 22;21:720. doi: 10.1186/s12913-021-06688-7 (PMC8296742; doi:10.1186/s12913-021-06688-7)
Supplement: Supplementary file 1 — Additional file 1. [file 12913_2021_6688_MOESM1_ESM.zip › QB-part 3 labs 2019.01.28 FINALR3.pdf]

Tanzania PAC cost study – Quest. B part 3 labs

Field	Question	Answer
about_survey	**Tanzania PAC cost study – Quest. B part 3 labs**<br><br>INTERVIEWER INSTRUCTIONS:<br>• DO NOT READ TEXT IN ALL CAPS OR HINTS IN *ITALICS* ALOUD TO THE PARTICIPANT.<br>• BEFORE STARTING THE INTERVIEW, CHECK WHICH PARTS OR SECTIONS OF QUESTIONNAIRE B HAVE NOT BEEN COMPLETED.<br>• MAKE SURE THE PERSON YOU ARE ABOUT TO INTERVIEW HAS PROVIDED CONSENT.	
introduction	QUESTIONNAIRE INTRODUCTION: We're now ready to complete part 3 of the interview. In this part of the interview we'll focus on laboratory tests done at your facility or at an off-site laboratory.<br><br>As with the other interview parts, I'll first present a list of items and ask which items are used for the five types of postabortion complications. Those are: uncomplicated incomplete abortion, sepsis, shock, cervical and vaginal lacerations, and vaginal and uterine perforations.<br><br>Then, if you tell me that an the item is used at your facility, I'll separately ask how it is used (e.g. what proportion of women get it, how much they get, etc.).<br><br>At the end of interview, for any item that is used, I'll ask about prices for buying the items. If you don't have that information, I can obtain it from someone else at your facility after the interview.<br><br>Before we begin, I'm going to capture a some information about where we are and document that I'm doing the interview with you. Remember that your name won't be used with the results later.	
COVER PAGE		
q1_interviewer_name_lab *(required)*	q1. NAME OF INTERVIEWER	
q2_facility_name_lab *(required)*	q2. SELECT: NAME OF FACILITY	facilityid | facility_name
q3_GPS_QB_lab *(required)*	q3. CAPTURE THE GPS LOCATION<br>*Press the button to capture the GPS location at this point in the survey.*	
RESPONDENTS (1-5)		
cover_page_note_1_lab	INTERVIEWER INSTRUCTIONS: INDICATE NAMES, JOB TITLES AND CONTACT INFORMATION FOR UP TO 5 RESPONDENTS OF QUESTIONNAIRE A.<br>*NB: Information for only the first respondent is required in order to proceed to the next page. Ask for the information as a question if not already known prior to the interview.*	
group_respondent1_lab	RESPONDENT 1	
q4a_name_lab *(required)*	q4a. Name of 1st respondent<br>*First and last name*	
q4b_date_lab *(required)*	q4b. Date of interview with 1st respondent<br>*Default is today's date.*	
q4c_title_lab *(required)*	q4c. Designation/title of 1st respondent<br>*Job title and designation (e.g. Medical officer in charge)*	
q4d_phone_lab	q4d. Phone number of 1st respondent<br>*NB: Not required, but you might need this to follow up with questions.*	
group_respondent2_lab	RESPONDENT 2	
q5a_name_lab	q5a. Name of 2nd respondent<br>*First and last name*	
q5b_date_lab	q5b. Date of interview with 2nd respondent<br>*Default is today's date.*	
q5c_title_lab	q5c. Designation/title of 2nd respondent<br>*Job title and designation (e.g. Medical officer in charge)*	
q5d_phone_lab	q5d. Phone number of 2nd respondent<br>*NB: Not required, but you might need this to follow up with questions.*	
group_respondent3_lab	RESPONDENT 3	
q6a_name_lab	q6a. Name of 3rd respondent<br>*First and last name*	
q6b_date_lab	q6b. Date of interview with 3rd respondent<br>*Default is today's date.*	
q6c_title_lab	q6c. Designation/title of 3rd respondent<br>*Job title and designation (e.g. Medical officer in charge)*	
q6d_phone_lab	q6d. Phone number of 3rd respondent<br>*NB: Not required, but you might need this to follow up with questions.*	
group_respondent4_lab	RESPONDENT 4	
q7a_name_lab	q7a. Name of 4th respondent<br>*First and last name*	

Field	Question	Answer
q7b_date_lab	q7b. Date of interview with 4th respondent<br>*Default is today's date.*	
q7c_title_lab	q7c. Designation/title of 4th respondent<br>*Job title and designation (e.g. Medical officer in charge)*	
q7d_phone_lab	q7d. Phone number of 4th respondent<br>*NB: Not required, but you might need this to follow up with questions.*	
group_respondent5_lab	RESPONDENT 5	
q8a_name_lab	q8a. Name of 5th respondent<br>*First and last name*	
q8b_date_lab	q8b. Date of interview with 5th respondent<br>*Default is today's date.*	
q8c_title_lab	q8c. Designation/title of 5th respondent<br>*Job title and designation (e.g. Medical officer in charge)*	
q8d_phone_lab	q8d. Phone number of 5th respondent<br>*NB: Not required, but you might need this to follow up with questions.*	
q9_interviewer_comments_lab	q9. INTERVIEWER COMMENTS<br>*Enter any relevant notes prior to the interview.*	
q10_time_start_qa_lab *(required)*	q10. ENTER START TIME OF INTERVIEW<br>*NB: The default is the current time.*	
introduction_lab	QUESTIONNAIRE INTRODUCTION: Ok now we can begin the interview. Remember that all the questions in this part of the interview are about laboratory tests that are done at your facility or off-site as part of the PAC services at your facility.	
group_section_one_intro		
section1_start	**SECTION I. FULL LISTING OF ITEMS BY COMPLICATION TYPE**	
section_one_skip_lab	INTERVIEWER: WOULD YOU LIKE TO COMPLETE THIS SECTION NOW OR SKIP THIS SECTION AND RETURN TO IT LATER?<br>*You may need to skip if the participant has indicated that s/he cannot answer the questions in this section.*	1 Do not skip, complete this section now.<br>2 Skip and come back to this section later.
C. Laboratory tests, supplies - Full list (1)<br>*Group relevant when: selected(${section_one_skip_lab} , '1')*		
q103_lab_note1	103 For each of the following on-site and offsite laboratory tests and investigations, can you tell me if the item is used for post abortion care at your facility? I'm going to ask about each of the five complication types separately. Is [ITEM] used for managing women with [COMPLICATION TYPE]?	
note_103_blood	***Blood tests***	
q103_L1_full_list *(required)*	q103_L1. Blood glucose strip<br>*Select all that apply.*<br>*Response constrained to: if(selected(., 6) or selected(., 99), count-selected(.) = 1, count-selected(.) >= 1)*	1 Incomplete abortion<br>2 Sepsis<br>3 Shock<br>4 Cervical/vaginal lacerations<br>5 Vaginal/uterine perforation<br>6 Not used at this facility<br>99 Don't know
q103_L2_full_list *(required)*	q103_L2. Blood group test, A/B monoclonal<br>*Select all that apply.*<br>*Response constrained to: if(selected(., 6) or selected(., 99), count-selected(.) = 1, count-selected(.) >= 1)*	1 Incomplete abortion<br>2 Sepsis<br>3 Shock<br>4 Cervical/vaginal lacerations<br>5 Vaginal/uterine perforation<br>6 Not used at this facility<br>99 Don't know
q103_L3_full_list *(required)*	q103_L3. Full blood count<br>*Select all that apply.*<br>*Response constrained to: if(selected(., 6) or selected(., 99), count-selected(.) = 1, count-selected(.) >= 1)*	1 Incomplete abortion<br>2 Sepsis<br>3 Shock<br>4 Cervical/vaginal lacerations<br>5 Vaginal/uterine perforation<br>6 Not used at this facility<br>99 Don't know
q103_L4_full_list *(required)*	q103_L4. Hematocrit test<br>*Select all that apply.*<br>*Response constrained to: if(selected(., 6) or selected(., 99), count-selected(.) = 1, count-selected(.) >= 1)*	1 Incomplete abortion<br>2 Sepsis<br>3 Shock<br>4 Cervical/vaginal lacerations<br>5 Vaginal/uterine perforation<br>6 Not used at this facility<br>99 Don't know

Field	Question	Answer
q103_L5_full_list *(required)*	q103_L5. Hemoglobin test<br>*Select all that apply.*<br>*Response constrained to: if(selected(., 6) or selected(., 99), count-selected(.) = 1, count-selected(.) >= 1)*	1 Incomplete abortion<br>2 Sepsis<br>3 Shock<br>4 Cervical/vaginal lacerations<br>5 Vaginal/uterine perforation<br>6 Not used at this facility<br>99 Don't know
q103_L6_full_list *(required)*	q103_L6. Rhesus factor test<br>*Select all that apply.*<br>*Response constrained to: if(selected(., 6) or selected(., 99), count-selected(.) = 1, count-selected(.) >= 1)*	1 Incomplete abortion<br>2 Sepsis<br>3 Shock<br>4 Cervical/vaginal lacerations<br>5 Vaginal/uterine perforation<br>6 Not used at this facility<br>99 Don't know
q103_L7_full_list *(required)*	q103_L7. Test of bleeding time<br>*Select all that apply.*<br>*Response constrained to: if(selected(., 6) or selected(., 99), count-selected(.) = 1, count-selected(.) >= 1)*	1 Incomplete abortion<br>2 Sepsis<br>3 Shock<br>4 Cervical/vaginal lacerations<br>5 Vaginal/uterine perforation<br>6 Not used at this facility<br>99 Don't know
q103_L8_full_list *(required)*	q103_L8. White blood cell count<br>*Select all that apply.*<br>*Response constrained to: if(selected(., 6) or selected(., 99), count-selected(.) = 1, count-selected(.) >= 1)*	1 Incomplete abortion<br>2 Sepsis<br>3 Shock<br>4 Cervical/vaginal lacerations<br>5 Vaginal/uterine perforation<br>6 Not used at this facility<br>99 Don't know
C. Laboratory tests, supplies - Full list (2)<br>*Group relevant when: selected(${section_one_skip_lab} , '1')*		
q103_lab_note2	103 CONTINUED. For each of the following on-site and offsite laboratory tests and investigations, can you tell me if the item is used for post abortion care at your facility? I'm going to ask about each of the five complication types separately. Is [ITEM] used for managing women with [COMPLICATION TYPE]?	
note_103_HIV_STI	***HIV and STI tests***	
q103_L9_full_list *(required)*	q103_L9. HIV test<br>*Select all that apply.*<br>*Response constrained to: if(selected(., 6) or selected(., 99), count-selected(.) = 1, count-selected(.) >= 1)*	1 Incomplete abortion<br>2 Sepsis<br>3 Shock<br>4 Cervical/vaginal lacerations<br>5 Vaginal/uterine perforation<br>6 Not used at this facility<br>99 Don't know
q103_L10_full_list *(required)*	q103_L10. Rapid test for Hepatitis C<br>*Select all that apply.*<br>*Response constrained to: if(selected(., 6) or selected(., 99), count-selected(.) = 1, count-selected(.) >= 1)*	1 Incomplete abortion<br>2 Sepsis<br>3 Shock<br>4 Cervical/vaginal lacerations<br>5 Vaginal/uterine perforation<br>6 Not used at this facility<br>99 Don't know
q103_L11_full_list *(required)*	q103_L11. Syphilis test<br>*Select all that apply.*<br>*Response constrained to: if(selected(., 6) or selected(., 99), count-selected(.) = 1, count-selected(.) >= 1)*	1 Incomplete abortion<br>2 Sepsis<br>3 Shock<br>4 Cervical/vaginal lacerations<br>5 Vaginal/uterine perforation<br>6 Not used at this facility<br>99 Don't know
C. Laboratory tests, supplies - Full list (3)<br>*Group relevant when: selected(${section_one_skip_lab} , '1')*		
q103_lab_note3	103 CONTINUED. For each of the following on-site and offsite laboratory tests and investigations, can you tell me if the item is used for post abortion care at your facility? I'm going to ask about each of the five complication types separately. Is [ITEM] used for managing women with [COMPLICATION TYPE]?	
note_103_malaria	***Malaria***	

Field	Question	Answer
q103_L12_full_list *(required)*	q103_L12. Rapid test for Malaria<br>*Select all that apply.*<br>*Response constrained to: if(selected(., 6) or selected(., 99), count-selected(.) = 1, count-selected(.) >= 1)*	1 Incomplete abortion<br>2 Sepsis<br>3 Shock<br>4 Cervical/vaginal lacerations<br>5 Vaginal/uterine perforation<br>6 Not used at this facility<br>99 Don't know
q103_L13_full_list *(required)*	q103_L13. Thick blood smear (for malaria)<br>*Select all that apply.*<br>*Response constrained to: if(selected(., 6) or selected(., 99), count-selected(.) = 1, count-selected(.) >= 1)*	1 Incomplete abortion<br>2 Sepsis<br>3 Shock<br>4 Cervical/vaginal lacerations<br>5 Vaginal/uterine perforation<br>6 Not used at this facility<br>99 Don't know
C. Laboratory tests, supplies - Full list (4)<br>*Group relevant when: selected(${section_one_skip_lab} , '1')*		
q103_lab_note4	103 CONTINUED. For each of the following on-site and offsite laboratory tests and investigations, can you tell me if the item is used for post abortion care at your facility? I'm going to ask about each of the five complication types separately. Is [ITEM] used for managing women with [COMPLICATION TYPE]?	
note_103_Pregnancy	***Pregnancy***	
q103_L14_full_list *(required)*	q103_L14. Pregnancy test - blood<br>*Select all that apply.*<br>*Response constrained to: if(selected(., 6) or selected(., 99), count-selected(.) = 1, count-selected(.) >= 1)*	1 Incomplete abortion<br>2 Sepsis<br>3 Shock<br>4 Cervical/vaginal lacerations<br>5 Vaginal/uterine perforation<br>6 Not used at this facility<br>99 Don't know
q103_L15_full_list *(required)*	q103_L15. Pregnancy test - urine<br>*Select all that apply.*<br>*Response constrained to: if(selected(., 6) or selected(., 99), count-selected(.) = 1, count-selected(.) >= 1)*	1 Incomplete abortion<br>2 Sepsis<br>3 Shock<br>4 Cervical/vaginal lacerations<br>5 Vaginal/uterine perforation<br>6 Not used at this facility<br>99 Don't know
q103_L16_full_list *(required)*	q103_L16. Ultrasound<br>*Select all that apply.*<br>*Response constrained to: if(selected(., 6) or selected(., 99), count-selected(.) = 1, count-selected(.) >= 1)*	1 Incomplete abortion<br>2 Sepsis<br>3 Shock<br>4 Cervical/vaginal lacerations<br>5 Vaginal/uterine perforation<br>6 Not used at this facility<br>99 Don't know
C. Laboratory tests, supplies - Full list (5)<br>*Group relevant when: selected(${section_one_skip_lab} , '1')*		
q103_lab_note5	103 CONTINUED. For each of the following on-site and offsite laboratory tests and investigations, can you tell me if the item is used for post abortion care at your facility? I'm going to ask about each of the five complication types separately. Is [ITEM] used for managing women with [COMPLICATION TYPE]?	
note_103_Urine	***Urine***	
q103_L17_full_list *(required)*	q103_L17. Urine dipstick<br>*Select all that apply.*<br>*Response constrained to: if(selected(., 6) or selected(., 99), count-selected(.) = 1, count-selected(.) >= 1)*	1 Incomplete abortion<br>2 Sepsis<br>3 Shock<br>4 Cervical/vaginal lacerations<br>5 Vaginal/uterine perforation<br>6 Not used at this facility<br>99 Don't know
q103_lab_other *(required)*	q103_Lab_Other. Are there any on-site or off-site laboratory tests that we have not mentioned that are used for postabortion care at your facility?<br>*Question relevant when: selected(${section_one_skip_lab} , '1')*	1 Yes<br>0 No<br>99 Don't know
C. Laboratory tests, supplies - Full list - specify (6)<br>*Group relevant when: selected(${q103_lab_other} , '1') and selected(${section_one_skip_lab} , '1')*		
note_103_other_suggestion	Please list the "other" laboratory items here.	
q103_L18_full_list_other	q103_L18. Other lab test 1. Please specify:	

Field	Question	Answer
q103_L19_full_list_other	q103_L19. Other lab test 2. Please specify:	
q103_L20_full_list_other	q103_L20. Other lab test 3. Please specify:	
q103_L21_full_list_other	q103_L21. Other lab test 4. Please specify:	
q103_L22_full_list_other	q103_L22. Other lab test 5. Please specify:	
C. Laboratory tests, supplies - Full list - usage (6b)<br>*Group relevant when: selected(${q103_lab_other} , '1') and selected(${section_one_skip_lab} , '1')*		
note_103_other_suggestion_b	For each other laboratory item, please tell me which of the five post abortion complication types it is used for.	
q103_L18_full_list *(required)*	q103_L18. Other lab test 1: "[q103_L18_full_list_other]"<br>*Select all that apply.*<br>*Question relevant when: string-length(${q103_L18_full_list_other}) > 0 and selected(${section_one_skip_lab} , '1')*<br>*Response constrained to: if(selected(., 6) or selected(., 99), count-selected(.) = 1, count-selected(.) >= 1)*	1 Incomplete abortion<br>2 Sepsis<br>3 Shock<br>4 Cervical/vaginal lacerations<br>5 Vaginal/uterine perforation<br>6 Not used at this facility<br>99 Don't know
q103_L19_full_list *(required)*	q103_L19. Other lab test 2: "[q103_L19_full_list_other]"<br>*Select all that apply.*<br>*Question relevant when: string-length(${q103_L19_full_list_other}) > 0 and selected(${section_one_skip_lab} , '1')*<br>*Response constrained to: if(selected(., 6) or selected(., 99), count-selected(.) = 1, count-selected(.) >= 1)*	1 Incomplete abortion<br>2 Sepsis<br>3 Shock<br>4 Cervical/vaginal lacerations<br>5 Vaginal/uterine perforation<br>6 Not used at this facility<br>99 Don't know
q103_L20_full_list *(required)*	q103_L20. Other lab test 3: "[q103_L20_full_list_other]"<br>*Select all that apply.*<br>*Question relevant when: string-length(${q103_L20_full_list_other}) > 0 and selected(${section_one_skip_lab} , '1')*<br>*Response constrained to: if(selected(., 6) or selected(., 99), count-selected(.) = 1, count-selected(.) >= 1)*	1 Incomplete abortion<br>2 Sepsis<br>3 Shock<br>4 Cervical/vaginal lacerations<br>5 Vaginal/uterine perforation<br>6 Not used at this facility<br>99 Don't know
q103_L21_full_list *(required)*	q103_L21. Other lab test 4: "[q103_L21_full_list_other]"<br>*Select all that apply.*<br>*Question relevant when: string-length(${q103_L21_full_list_other}) > 0 and selected(${section_one_skip_lab} , '1')*<br>*Response constrained to: if(selected(., 6) or selected(., 99), count-selected(.) = 1, count-selected(.) >= 1)*	1 Incomplete abortion<br>2 Sepsis<br>3 Shock<br>4 Cervical/vaginal lacerations<br>5 Vaginal/uterine perforation<br>6 Not used at this facility<br>99 Don't know
q103_L22_full_list *(required)*	q103_L22. Other lab test 5: "[q103_L22_full_list_other]"<br>*Select all that apply.*<br>*Question relevant when: string-length(${q103_L22_full_list_other}) > 0 and selected(${section_one_skip_lab} , '1')*<br>*Response constrained to: if(selected(., 6) or selected(., 99), count-selected(.) = 1, count-selected(.) >= 1)*	1 Incomplete abortion<br>2 Sepsis<br>3 Shock<br>4 Cervical/vaginal lacerations<br>5 Vaginal/uterine perforation<br>6 Not used at this facility<br>99 Don't know
group_section_two_intro		
section2_start	**SECTION II. INCOMPLETE ABORTION - USAGE OF ALL ITEMS**	
section_two_skip_lab	INTERVIEWER: WOULD YOU LIKE TO COMPLETE THIS SECTION NOW OR SKIP THIS SECTION AND RETURN TO IT LATER?<br>*You may need to skip if the participant has indicated that s/he cannot answer the questions in this section.*	1 Do not skip, complete this section now.<br>2 Skip and come back to this section later.
group_section_two_introB<br>*Group relevant when: selected(${section_two_skip_lab} , '1')*		
section2_start2	In this section of the interview, we will review all of the items that you said are used for management of "uncomplicated" incomplete abortion. For each item that is used, I'm going to ask questions on how many women need it and how much of it is used.	
section2_start3	INTERVIEWER: ENTER WHOLE NUMBERS OR DECIMALS. DO NOT TYPE PERCENT SIGNS. ENTER 999 FOR ANY THAT ARE UNKNOWN.	
C. Laboratory tests, supplies - Incomplete Abortion (1)<br>*Group relevant when: selected(${section_two_skip_lab} , '1')*		
note_203_blood	***Blood tests***	
q203_L1	q203_L1. Blood glucose strip<br>*Question relevant when: selected(${q103_L1_full_list} , '1')*	

Field	Question	Answer
q203_L1a *(required)*	q203_L1a. What percent of patients require this item?<br>*Question relevant when: selected(${q103_L1_full_list} , '1')*<br>*Response constrained to: .>0 and .<=100 or .=999*	
q203_L1b *(required)*	q203_L1b. How many units of this test or exam are required per patient (for all treatment provided for complication)?<br>*Smallest unit is one strip.*<br>*Question relevant when: selected(${q103_L1_full_list} , '1')*<br>*Response constrained to: .>0*	
q203_L1c *(required)*	q203_L1c. Is this test or exam done at your facility (on-site) or at an off-site laboratory?<br>*Question relevant when: selected(${q103_L1_full_list} , '1')*	1 On-site<br>2 Off-site<br>99 Don't know
q203_L2	q203_L2. Blood group test, A/B monoclonal<br>*Question relevant when: selected(${q103_L2_full_list} , '1')*	
q203_L2a *(required)*	q203_L2a. What percent of patients require this item?<br>*Question relevant when: selected(${q103_L2_full_list} , '1')*<br>*Response constrained to: .>0 and .<=100 or .=999*	
q203_L2b *(required)*	q203_L2b. How many units of this test or exam are required per patient (for all treatment provided for complication)?<br>*Smallest unit is one test.*<br>*Question relevant when: selected(${q103_L2_full_list} , '1')*<br>*Response constrained to: .>0*	
q203_L2c *(required)*	q203_L2c. Is this test or exam done at your facility (on-site) or at an off-site laboratory?<br>*Question relevant when: selected(${q103_L2_full_list} , '1')*	1 On-site<br>2 Off-site<br>99 Don't know
q203_L3	q203_L3. Full blood count<br>*Question relevant when: selected(${q103_L3_full_list} , '1')*	
q203_L3a *(required)*	q203_L3a. What percent of patients require this item?<br>*Question relevant when: selected(${q103_L3_full_list} , '1')*<br>*Response constrained to: .>0 and .<=100 or .=999*	
q203_L3b *(required)*	q203_L3b. How many units of this test or exam are required per patient (for all treatment provided for complication)?<br>*Smallest unit is one test.*<br>*Question relevant when: selected(${q103_L3_full_list} , '1')*<br>*Response constrained to: .>0*	
q203_L3c *(required)*	q203_L3c. Is this test or exam done at your facility (on-site) or at an off-site laboratory?<br>*Question relevant when: selected(${q103_L3_full_list} , '1')*	1 On-site<br>2 Off-site<br>99 Don't know
q203_L4	q203_L4. Hematocrit test<br>*Question relevant when: selected(${q103_L4_full_list} , '1')*	
q203_L4a *(required)*	q203_L4a. What percent of patients require this item?<br>*Question relevant when: selected(${q103_L4_full_list} , '1')*<br>*Response constrained to: .>0 and .<=100 or .=999*	
q203_L4b *(required)*	q203_L4b. How many units of this test or exam are required per patient (for all treatment provided for complication)?<br>*Smallest unit is one test.*<br>*Question relevant when: selected(${q103_L4_full_list} , '1')*<br>*Response constrained to: .>0*	
q203_L4c *(required)*	q203_L4c. Is this test or exam done at your facility (on-site) or at an off-site laboratory?<br>*Question relevant when: selected(${q103_L4_full_list} , '1')*	1 On-site<br>2 Off-site<br>99 Don't know
q203_L5	q203_L5. Hemoglobin test<br>*Question relevant when: selected(${q103_L5_full_list} , '1')*	
q203_L5a *(required)*	q203_L5a. What percent of patients require this item?<br>*Question relevant when: selected(${q103_L5_full_list} , '1')*<br>*Response constrained to: .>0 and .<=100 or .=999*	
q203_L5b *(required)*	q203_L5b. How many units of this test or exam are required per patient (for all treatment provided for complication)?<br>*Smallest unit is one test.*<br>*Question relevant when: selected(${q103_L5_full_list} , '1')*<br>*Response constrained to: .>0*	
q203_L5c *(required)*	q203_L5c. Is this test or exam done at your facility (on-site) or at an off-site laboratory?<br>*Question relevant when: selected(${q103_L5_full_list} , '1')*	1 On-site<br>2 Off-site<br>99 Don't know
q203_L6	q203_L6. Rhesus factor test<br>*Question relevant when: selected(${q103_L6_full_list} , '1')*	
q203_L6a *(required)*	q203_L6a. What percent of patients require this item?<br>*Question relevant when: selected(${q103_L6_full_list} , '1')*<br>*Response constrained to: .>0 and .<=100 or .=999*	

Field	Question	Answer
q203_L6b *(required)*	q203_L6b. How many units of this test or exam are required per patient (for all treatment provided for complication)?<br>*Smallest unit is one test.*<br>*Question relevant when: selected(${q103_L6_full_list} , '1')*<br>*Response constrained to: .>0*	
q203_L6c *(required)*	q203_L6c. Is this test or exam done at your facility (on-site) or at an off-site laboratory?<br>*Question relevant when: selected(${q103_L6_full_list} , '1')*	1 On-site<br>2 Off-site<br>99 Don't know
q203_L7	q203_L7. Test of bleeding time<br>*Question relevant when: selected(${q103_L7_full_list} , '1')*	
q203_L7a *(required)*	q203_L7a. What percent of patients require this item?<br>*Question relevant when: selected(${q103_L7_full_list} , '1')*<br>*Response constrained to: .>0 and .<=100 or .=999*	
q203_L7b *(required)*	q203_L7b. How many units of this test or exam are required per patient (for all treatment provided for complication)?<br>*Smallest unit is one test.*<br>*Question relevant when: selected(${q103_L7_full_list} , '1')*<br>*Response constrained to: .>0*	
q203_L7c *(required)*	q203_L7c. Is this test or exam done at your facility (on-site) or at an off-site laboratory?<br>*Question relevant when: selected(${q103_L7_full_list} , '1')*	1 On-site<br>2 Off-site<br>99 Don't know
q203_L8	q203_L8. White blood cell count<br>*Question relevant when: selected(${q103_L8_full_list} , '1')*	
q203_L8a *(required)*	q203_L8a. What percent of patients require this item?<br>*Question relevant when: selected(${q103_L8_full_list} , '1')*<br>*Response constrained to: .>0 and .<=100 or .=999*	
q203_L8b *(required)*	q203_L8b. How many units of this test or exam are required per patient (for all treatment provided for complication)?<br>*Smallest unit is one test.*<br>*Question relevant when: selected(${q103_L8_full_list} , '1')*<br>*Response constrained to: .>0*	
q203_L8c *(required)*	q203_L8c. Is this test or exam done at your facility (on-site) or at an off-site laboratory?<br>*Question relevant when: selected(${q103_L8_full_list} , '1')*	1 On-site<br>2 Off-site<br>99 Don't know
C. Laboratory tests, supplies - Incomplete Abortion (2)<br>*Group relevant when: selected(${section_two_skip_lab} , '1')*		
note_203_HIV_STI	***HIV and STI tests***	
q203_L9	q203_L9. HIV test<br>*Question relevant when: selected(${q103_L9_full_list} , '1')*	
q203_L9a *(required)*	q203_L9a. What percent of patients require this item?<br>*Question relevant when: selected(${q103_L9_full_list} , '1')*<br>*Response constrained to: .>0 and .<=100 or .=999*	
q203_L9b *(required)*	q203_L9b. How many units of this test or exam are required per patient (for all treatment provided for complication)?<br>*Smallest unit is one test.*<br>*Question relevant when: selected(${q103_L9_full_list} , '1')*<br>*Response constrained to: .>0*	
q203_L9c *(required)*	q203_L9c. Is this test or exam done at your facility (on-site) or at an off-site laboratory?<br>*Question relevant when: selected(${q103_L9_full_list} , '1')*	1 On-site<br>2 Off-site<br>99 Don't know
q203_L10	q203_L10. Rapid test for Hepatitis C<br>*Question relevant when: selected(${q103_L10_full_list} , '1')*	
q203_L10a *(required)*	q203_L10a. What percent of patients require this item?<br>*Question relevant when: selected(${q103_L10_full_list} , '1')*<br>*Response constrained to: .>0 and .<=100 or .=999*	
q203_L10b *(required)*	q203_L10b. How many units of this test or exam are required per patient (for all treatment provided for complication)?<br>*Smallest unit is one test.*<br>*Question relevant when: selected(${q103_L10_full_list} , '1')*<br>*Response constrained to: .>0*	
q203_L10c *(required)*	q203_L10c. Is this test or exam done at your facility (on-site) or at an off-site laboratory?<br>*Question relevant when: selected(${q103_L10_full_list} , '1')*	1 On-site<br>2 Off-site<br>99 Don't know
q203_L11	q203_L11. Syphilis test<br>*Question relevant when: selected(${q103_L11_full_list} , '1')*	
q203_L11a *(required)*	q203_L11a. What percent of patients require this item?<br>*Question relevant when: selected(${q103_L11_full_list} , '1')*<br>*Response constrained to: .>0 and .<=100 or .=999*	

Field	Question	Answer
q203_L11b *(required)*	q203_L11b. How many units of this test or exam are required per patient (for all treatment provided for complication)?<br>*Smallest unit is one test.*<br>*Question relevant when: selected(${q103_L11_full_list} , '1')*<br>*Response constrained to: .>0*	
q203_L11c *(required)*	q203_L11c. Is this test or exam done at your facility (on-site) or at an off-site laboratory?<br>*Question relevant when: selected(${q103_L11_full_list} , '1')*	1 On-site<br>2 Off-site<br>99 Don't know
C. Laboratory tests, supplies - Incomplete Abortion (3)<br>*Group relevant when: selected(${section_two_skip_lab} , '1')*		
note_203_malaria	***Malaria***	
q203_L12	q203_L12. Rapid test for Malaria<br>*Question relevant when: selected(${q103_L12_full_list} , '1')*	
q203_L12a *(required)*	q203_L12a. What percent of patients require this item?<br>*Question relevant when: selected(${q103_L12_full_list} , '1')*<br>*Response constrained to: .>0 and .<=100 or .=999*	
q203_L12b *(required)*	q203_L12b. How many units of this test or exam are required per patient (for all treatment provided for complication)?<br>*Smallest unit is one test.*<br>*Question relevant when: selected(${q103_L12_full_list} , '1')*<br>*Response constrained to: .>0*	
q203_L12c *(required)*	q203_L12c. Is this test or exam done at your facility (on-site) or at an off-site laboratory?<br>*Question relevant when: selected(${q103_L12_full_list} , '1')*	1 On-site<br>2 Off-site<br>99 Don't know
q203_L13	q203_L13. Thick blood smear (for malaria)<br>*Question relevant when: selected(${q103_L13_full_list} , '1')*	
q203_L13a *(required)*	q203_L13a. What percent of patients require this item?<br>*Question relevant when: selected(${q103_L13_full_list} , '1')*<br>*Response constrained to: .>0 and .<=100 or .=999*	
q203_L13b *(required)*	q203_L13b. How many units of this test or exam are required per patient (for all treatment provided for complication)?<br>*Smallest unit is one test.*<br>*Question relevant when: selected(${q103_L13_full_list} , '1')*<br>*Response constrained to: .>0*	
q203_L13c *(required)*	q203_L13c. Is this test or exam done at your facility (on-site) or at an off-site laboratory?<br>*Question relevant when: selected(${q103_L13_full_list} , '1')*	1 On-site<br>2 Off-site<br>99 Don't know
C. Laboratory tests, supplies - Incomplete Abortion (4)<br>*Group relevant when: selected(${section_two_skip_lab} , '1')*		
note_203_Pregnancy	***Pregnancy***	
q203_L14	q203_L14. Pregnancy test - blood<br>*Question relevant when: selected(${q103_L14_full_list} , '1')*	
q203_L14a *(required)*	q203_L14a. What percent of patients require this item?<br>*Question relevant when: selected(${q103_L14_full_list} , '1')*<br>*Response constrained to: .>0 and .<=100 or .=999*	
q203_L14b *(required)*	q203_L14b. How many units of this test or exam are required per patient (for all treatment provided for complication)?<br>*Smallest unit is one test.*<br>*Question relevant when: selected(${q103_L14_full_list} , '1')*<br>*Response constrained to: .>0*	
q203_L14c *(required)*	q203_L14c. Is this test or exam done at your facility (on-site) or at an off-site laboratory?<br>*Question relevant when: selected(${q103_L14_full_list} , '1')*	1 On-site<br>2 Off-site<br>99 Don't know
q203_L15	q203_L15. Pregnancy test - urine<br>*Question relevant when: selected(${q103_L15_full_list} , '1')*	
q203_L15a *(required)*	q203_L15a. What percent of patients require this item?<br>*Question relevant when: selected(${q103_L15_full_list} , '1')*<br>*Response constrained to: .>0 and .<=100 or .=999*	
q203_L15b *(required)*	q203_L15b. How many units of this test or exam are required per patient (for all treatment provided for complication)?<br>*Smallest unit is one test.*<br>*Question relevant when: selected(${q103_L15_full_list} , '1')*<br>*Response constrained to: .>0*	
q203_L15c *(required)*	q203_L15c. Is this test or exam done at your facility (on-site) or at an off-site laboratory?<br>*Question relevant when: selected(${q103_L15_full_list} , '1')*	1 On-site<br>2 Off-site<br>99 Don't know
q203_L16	q203_L16. Ultrasound<br>*Question relevant when: selected(${q103_L16_full_list} , '1')*	

Field	Question	Answer
q203_L16a *(required)*	q203_L16a. What percent of patients require this item?<br>*Question relevant when: selected(${q103_L16_full_list} , '1')*<br>*Response constrained to: .>0 and .<=100 or .=999*	
q203_L16b *(required)*	q203_L16b. How many units of this test or exam are required per patient (for all treatment provided for complication)?<br>*Smallest unit is one scan.*<br>*Question relevant when: selected(${q103_L16_full_list} , '1')*<br>*Response constrained to: .>0*	
q203_L16c *(required)*	q203_L16c. Is this test or exam done at your facility (on-site) or at an off-site laboratory?<br>*Question relevant when: selected(${q103_L16_full_list} , '1')*	1 On-site<br>2 Off-site<br>99 Don't know
C. Laboratory tests, supplies - Incomplete Abortion(5)<br>*Group relevant when: selected(${section_two_skip_lab} , '1')*		
note_203_Urine	***Urine***	
q203_L17	q203_L17. Urine dipstick<br>*Question relevant when: selected(${q103_L17_full_list} , '1')*	
q203_L17a *(required)*	q203_L17a. What percent of patients require this item?<br>*Question relevant when: selected(${q103_L17_full_list} , '1')*<br>*Response constrained to: .>0 and .<=100 or .=999*	
q203_L17b *(required)*	q203_L17b. How many units of this test or exam are required per patient (for all treatment provided for complication)?<br>*Smallest unit is one dipstick.*<br>*Question relevant when: selected(${q103_L17_full_list} , '1')*<br>*Response constrained to: .>0*	
q203_L17c *(required)*	q203_L17c. Is this test or exam done at your facility (on-site) or at an off-site laboratory?<br>*Question relevant when: selected(${q103_L17_full_list} , '1')*	1 On-site<br>2 Off-site<br>99 Don't know
C. Laboratory tests, supplies - Incomplete Abortion (6)<br>*Group relevant when: selected(${section_two_skip_lab} , '1')*		
note_203_Other	***Other lab tests - suggestions?***	
q203_L18	q203_L18. Lab test other 1: "[q103_L18_full_list_other]"<br>*Question relevant when: selected(${q103_L18_full_list} , '1')*	
q203_L18a *(required)*	q203_L18a. What percent of patients require this item?<br>*Question relevant when: selected(${q103_L18_full_list} , '1')*<br>*Response constrained to: .>0 and .<=100 or .=999*	
q203_L18b *(required)*	q203_L18b. How many units of this test or exam are required per patient (for all treatment provided for complication)?<br>*Question relevant when: selected(${q103_L18_full_list} , '1')*<br>*Response constrained to: .>0*	
q203_L18c *(required)*	q203_L18c. Is this test or exam done at your facility (on-site) or at an off-site laboratory?<br>*Question relevant when: selected(${q103_L18_full_list} , '1')*	1 On-site<br>2 Off-site<br>99 Don't know
q203_L19	q203_L19. Lab test other 2: "[q103_L19_full_list_other]"<br>*Question relevant when: selected(${q103_L19_full_list} , '1')*	
q203_L19a *(required)*	q203_L19a. What percent of patients require this item?<br>*Question relevant when: selected(${q103_L19_full_list} , '1')*<br>*Response constrained to: .>0 and .<=100 or .=999*	
q203_L19b *(required)*	q203_L19b. How many units of this test or exam are required per patient (for all treatment provided for complication)?<br>*Question relevant when: selected(${q103_L19_full_list} , '1')*<br>*Response constrained to: .>0*	
q203_L19c *(required)*	q203_L19c. Is this test or exam done at your facility (on-site) or at an off-site laboratory?<br>*Question relevant when: selected(${q103_L19_full_list} , '1')*	1 On-site<br>2 Off-site<br>99 Don't know
q203_L20	q203_L20. Lab test other 3: "[q103_L20_full_list_other]"<br>*Question relevant when: selected(${q103_L20_full_list} , '1')*	
q203_L20a *(required)*	q203_L20a. What percent of patients require this item?<br>*Question relevant when: selected(${q103_L20_full_list} , '1')*<br>*Response constrained to: .>0 and .<=100 or .=999*	
q203_L20b *(required)*	q203_L20b. How many units of this test or exam are required per patient (for all treatment provided for complication)?<br>*Question relevant when: selected(${q103_L20_full_list} , '1')*<br>*Response constrained to: .>0*	
q203_L20c *(required)*	q203_L20c. Is this test or exam done at your facility (on-site) or at an off-site laboratory?<br>*Question relevant when: selected(${q103_L20_full_list} , '1')*	1 On-site<br>2 Off-site<br>99 Don't know

Field	Question	Answer
q203_L21	q203_L21. Lab test other 4: "[q103_L21_full_list_other]"<br>*Question relevant when: selected(${q103_L21_full_list} , '1')*	
q203_L21a *(required)*	q203_L21a. What percent of patients require this item?<br>*Question relevant when: selected(${q103_L21_full_list} , '1')*<br>*Response constrained to: .>0 and .<=100 or .=999*	
q203_L21b *(required)*	q203_L21b. How many units of this test or exam are required per patient (for all treatment provided for complication)?<br>*Question relevant when: selected(${q103_L21_full_list} , '1')*<br>*Response constrained to: .>0*	
q203_L21c *(required)*	q203_L21c. Is this test or exam done at your facility (on-site) or at an off-site laboratory?<br>*Question relevant when: selected(${q103_L21_full_list} , '1')*	1 On-site<br>2 Off-site<br>99 Don't know
q203_L22	q203_L22. Lab test other 5: "[q103_L22_full_list_other]"<br>*Question relevant when: selected(${q103_L22_full_list} , '1')*	
q203_L22a *(required)*	q203_L22a. What percent of patients require this item?<br>*Question relevant when: selected(${q103_L22_full_list} , '1')*<br>*Response constrained to: .>0 and .<=100 or .=999*	
q203_L22b *(required)*	q203_L22b. How many units of this test or exam are required per patient (for all treatment provided for complication)?<br>*Question relevant when: selected(${q103_L22_full_list} , '1')*<br>*Response constrained to: .>0*	
q203_L22c *(required)*	q203_L22c. Is this test or exam done at your facility (on-site) or at an off-site laboratory?<br>*Question relevant when: selected(${q103_L22_full_list} , '1')*	1 On-site<br>2 Off-site<br>99 Don't know
group_section_three_intro		
section3_start	**SECTION III. SEPSIS - USAGE OF ALL ITEMS**	
section_three_skip_lab	INTERVIEWER: WOULD YOU LIKE TO COMPLETE THIS SECTION NOW OR SKIP THIS SECTION AND RETURN TO IT LATER?<br>*You may need to skip if the participant has indicated that s/he cannot answer the questions in this section.*	1 Do not skip, complete this section now.<br>2 Skip and come back to this section later.
group_section_three_introB<br>*Group relevant when: selected(${section_three_skip_lab} , '1')*		
section3_start2	In this section of the interview, we will review all of the items that you said are used for management of sepsis. For each item that is used, I'm going to ask questions on how many women need it and how much of it is used.	
section3_start3	INTERVIEWER: ENTER WHOLE NUMBERS OR DECIMALS. DO NOT TYPE PERCENT SIGNS. ENTER 999 FOR ANY THAT ARE UNKNOWN.	
C. Laboratory tests, supplies - Sepsis (1)<br>*Group relevant when: selected(${section_three_skip_lab} , '1')*		
note_303_blood	***Blood tests***	
q303_L1	q303_L1. Blood glucose strip<br>*Question relevant when: selected(${q103_L1_full_list} , '2')*	
q303_L1a *(required)*	q303_L1a. What percent of patients require this item?<br>*Question relevant when: selected(${q103_L1_full_list} , '2')*<br>*Response constrained to: .>0 and .<=100 or .=999*	
q303_L1b *(required)*	q303_L1b. How many units of this test or exam are required per patient (for all treatment provided for complication)?<br>*Smallest unit is one strip.*<br>*Question relevant when: selected(${q103_L1_full_list} , '2')*<br>*Response constrained to: .>0*	
q303_L1c *(required)*	q303_L1c. Is this test or exam done at your facility (on-site) or at an off-site laboratory?<br>*Question relevant when: selected(${q103_L1_full_list} , '2')*	1 On-site<br>2 Off-site<br>99 Don't know
q303_L2	q303_L2. Blood group test, A/B monoclonal<br>*Question relevant when: selected(${q103_L2_full_list} , '2')*	
q303_L2a *(required)*	q303_L2a. What percent of patients require this item?<br>*Question relevant when: selected(${q103_L2_full_list} , '2')*<br>*Response constrained to: .>0 and .<=100 or .=999*	
q303_L2b *(required)*	q303_L2b. How many units of this test or exam are required per patient (for all treatment provided for complication)?<br>*Smallest unit is one test.*<br>*Question relevant when: selected(${q103_L2_full_list} , '2')*<br>*Response constrained to: .>0*	
q303_L2c *(required)*	q303_L2c. Is this test or exam done at your facility (on-site) or at an off-site laboratory?<br>*Question relevant when: selected(${q103_L2_full_list} , '2')*	1 On-site<br>2 Off-site<br>99 Don't know
q303_L3	q303_L3. Full blood count<br>*Question relevant when: selected(${q103_L3_full_list} , '2')*	

Field	Question	Answer
q303_L3a *(required)*	q303_L3a. What percent of patients require this item?<br>*Question relevant when: selected(${q103_L3_full_list} , '2')*<br>*Response constrained to: .>0 and .<=100 or .=999*	
q303_L3b *(required)*	q303_L3b. How many units of this test or exam are required per patient (for all treatment provided for complication)?<br>*Smallest unit is one test.*<br>*Question relevant when: selected(${q103_L3_full_list} , '2')*<br>*Response constrained to: .>0*	
q303_L3c *(required)*	q303_L3c. Is this test or exam done at your facility (on-site) or at an off-site laboratory?<br>*Question relevant when: selected(${q103_L3_full_list} , '2')*	1 On-site<br>2 Off-site<br>99 Don't know
q303_L4	q303_L4. Hematocrit test<br>*Question relevant when: selected(${q103_L4_full_list} , '2')*	
q303_L4a *(required)*	q303_L4a. What percent of patients require this item?<br>*Question relevant when: selected(${q103_L4_full_list} , '2')*<br>*Response constrained to: .>0 and .<=100 or .=999*	
q303_L4b *(required)*	q303_L4b. How many units of this test or exam are required per patient (for all treatment provided for complication)?<br>*Smallest unit is one test.*<br>*Question relevant when: selected(${q103_L4_full_list} , '2')*<br>*Response constrained to: .>0*	
q303_L4c *(required)*	q303_L4c. Is this test or exam done at your facility (on-site) or at an off-site laboratory?<br>*Question relevant when: selected(${q103_L4_full_list} , '2')*	1 On-site<br>2 Off-site<br>99 Don't know
q303_L5	q303_L5. Hemoglobin test<br>*Question relevant when: selected(${q103_L5_full_list} , '2')*	
q303_L5a *(required)*	q303_L5a. What percent of patients require this item?<br>*Question relevant when: selected(${q103_L5_full_list} , '2')*<br>*Response constrained to: .>0 and .<=100 or .=999*	
q303_L5b *(required)*	q303_L5b. How many units of this test or exam are required per patient (for all treatment provided for complication)?<br>*Smallest unit is one test.*<br>*Question relevant when: selected(${q103_L5_full_list} , '2')*<br>*Response constrained to: .>0*	
q303_L5c *(required)*	q303_L5c. Is this test or exam done at your facility (on-site) or at an off-site laboratory?<br>*Question relevant when: selected(${q103_L5_full_list} , '2')*	1 On-site<br>2 Off-site<br>99 Don't know
q303_L6	q303_L6. Rhesus factor test<br>*Question relevant when: selected(${q103_L6_full_list} , '2')*	
q303_L6a *(required)*	q303_L6a. What percent of patients require this item?<br>*Question relevant when: selected(${q103_L6_full_list} , '2')*<br>*Response constrained to: .>0 and .<=100 or .=999*	
q303_L6b *(required)*	q303_L6b. How many units of this test or exam are required per patient (for all treatment provided for complication)?<br>*Smallest unit is one test.*<br>*Question relevant when: selected(${q103_L6_full_list} , '2')*<br>*Response constrained to: .>0*	
q303_L6c *(required)*	q303_L6c. Is this test or exam done at your facility (on-site) or at an off-site laboratory?<br>*Question relevant when: selected(${q103_L6_full_list} , '2')*	1 On-site<br>2 Off-site<br>99 Don't know
q303_L7	q303_L7. Test of bleeding time<br>*Question relevant when: selected(${q103_L7_full_list} , '2')*	
q303_L7a *(required)*	q303_L7a. What percent of patients require this item?<br>*Question relevant when: selected(${q103_L7_full_list} , '2')*<br>*Response constrained to: .>0 and .<=100 or .=999*	
q303_L7b *(required)*	q303_L7b. How many units of this test or exam are required per patient (for all treatment provided for complication)?<br>*Smallest unit is one test.*<br>*Question relevant when: selected(${q103_L7_full_list} , '2')*<br>*Response constrained to: .>0*	
q303_L7c *(required)*	q303_L7c. Is this test or exam done at your facility (on-site) or at an off-site laboratory?<br>*Question relevant when: selected(${q103_L7_full_list} , '2')*	1 On-site<br>2 Off-site<br>99 Don't know
q303_L8	q303_L8. White blood cell count<br>*Question relevant when: selected(${q103_L8_full_list} , '2')*	
q303_L8a *(required)*	q303_L8a. What percent of patients require this item?<br>*Question relevant when: selected(${q103_L8_full_list} , '2')*	

Response constrained to: .>0 and .<=100 or .=999

Field	Question	Answer
q303_L8b *(required)*	q303_L8b. How many units of this test or exam are required per patient (for all treatment provided for complication)?<br>*Smallest unit is one test.*<br>*Question relevant when: selected(${q103_L8_full_list} , '2')*<br>*Response constrained to: .>0*	
q303_L8c *(required)*	q303_L8c. Is this test or exam done at your facility (on-site) or at an off-site laboratory?<br>*Question relevant when: selected(${q103_L8_full_list} , '2')*	1 On-site<br>2 Off-site<br>99 Don't know
C. Laboratory tests, supplies - Sepsis (2)<br>*Group relevant when: selected(${section_three_skip_lab} , '1')*		
note_303_HIV_STI	***HIV and STI tests***	
q303_L9	q303_L9. HIV test<br>*Question relevant when: selected(${q103_L9_full_list} , '2')*	
q303_L9a *(required)*	q303_L9a. What percent of patients require this item?<br>*Question relevant when: selected(${q103_L9_full_list} , '2')*<br>*Response constrained to: .>0 and .<=100 or .=999*	
q303_L9b *(required)*	q303_L9b. How many units of this test or exam are required per patient (for all treatment provided for complication)?<br>*Smallest unit is one test.*<br>*Question relevant when: selected(${q103_L9_full_list} , '2')*<br>*Response constrained to: .>0*	
q303_L9c *(required)*	q303_L9c. Is this test or exam done at your facility (on-site) or at an off-site laboratory?<br>*Question relevant when: selected(${q103_L9_full_list} , '2')*	1 On-site<br>2 Off-site<br>99 Don't know
q303_L10	q303_L10. Rapid test for Hepatitis C<br>*Question relevant when: selected(${q103_L10_full_list} , '2')*	
q303_L10a *(required)*	q303_L10a. What percent of patients require this item?<br>*Question relevant when: selected(${q103_L10_full_list} , '2')*<br>*Response constrained to: .>0 and .<=100 or .=999*	
q303_L10b *(required)*	q303_L10b. How many units of this test or exam are required per patient (for all treatment provided for complication)?<br>*Smallest unit is one test.*<br>*Question relevant when: selected(${q103_L10_full_list} , '2')*<br>*Response constrained to: .>0*	
q303_L10c *(required)*	q303_L10c. Is this test or exam done at your facility (on-site) or at an off-site laboratory?<br>*Question relevant when: selected(${q103_L10_full_list} , '2')*	1 On-site<br>2 Off-site<br>99 Don't know
q303_L11	q303_L11. Syphilis test<br>*Question relevant when: selected(${q103_L11_full_list} , '2')*	
q303_L11a *(required)*	q303_L11a. What percent of patients require this item?<br>*Question relevant when: selected(${q103_L11_full_list} , '2')*<br>*Response constrained to: .>0 and .<=100 or .=999*	
q303_L11b *(required)*	q303_L11b. How many units of this test or exam are required per patient (for all treatment provided for complication)?<br>*Smallest unit is one test.*<br>*Question relevant when: selected(${q103_L11_full_list} , '2')*<br>*Response constrained to: .>0*	
q303_L11c *(required)*	q303_L11c. Is this test or exam done at your facility (on-site) or at an off-site laboratory?<br>*Question relevant when: selected(${q103_L11_full_list} , '2')*	1 On-site<br>2 Off-site<br>99 Don't know
C. Laboratory tests, supplies - Sepsis (3)<br>*Group relevant when: selected(${section_three_skip_lab} , '1')*		
note_303_malaria	***Malaria***	
q303_L12	q303_L12. Rapid test for Malaria<br>*Question relevant when: selected(${q103_L12_full_list} , '2')*	
q303_L12a *(required)*	q303_L12a. What percent of patients require this item?<br>*Question relevant when: selected(${q103_L12_full_list} , '2')*<br>*Response constrained to: .>0 and .<=100 or .=999*	
q303_L12b *(required)*	q303_L12b. How many units of this test or exam are required per patient (for all treatment provided for complication)?<br>*Smallest unit is one test.*<br>*Question relevant when: selected(${q103_L12_full_list} , '2')*<br>*Response constrained to: .>0*	
q303_L12c *(required)*	q303_L12c. Is this test or exam done at your facility (on-site) or at an off-site laboratory?<br>*Question relevant when: selected(${q103_L12_full_list} , '2')*	1 On-site<br>2 Off-site

Field	Question	Answer
		99 Don't know
q303_L13	q303_L13. Thick blood smear (for malaria)	
	Question relevant when: selected(${q103_L13_full_list} , '2')	

q303_L13a *(required)*	q303_L13a. What percent of patients require this item?<br>*Question relevant when: selected(${q103_L13_full_list} , '2')*<br>*Response constrained to: .>0 and .<=100 or .=999*	
q303_L13b *(required)*	q303_L13b. How many units of this test or exam are required per patient (for all treatment provided for complication)?<br>*Smallest unit is one test.*<br>*Question relevant when: selected(${q103_L13_full_list} , '2')*<br>*Response constrained to: .>0*	
q303_L13c *(required)*	q303_L13c. Is this test or exam done at your facility (on-site) or at an off-site laboratory?<br>*Question relevant when: selected(${q103_L13_full_list} , '2')*	1 On-site<br>2 Off-site<br>99 Don't know
C. Laboratory tests, supplies - Sepsis (4)<br>*Group relevant when: selected(${section_three_skip_lab} , '1')*		
note_303_Pregnancy	***Pregnancy***	
q303_L14	q303_L14. Pregnancy test - blood<br>*Question relevant when: selected(${q103_L14_full_list} , '2')*	
q303_L14a *(required)*	q303_L14a. What percent of patients require this item?<br>*Question relevant when: selected(${q103_L14_full_list} , '2')*<br>*Response constrained to: .>0 and .<=100 or .=999*	
q303_L14b *(required)*	q303_L14b. How many units of this test or exam are required per patient (for all treatment provided for complication)?<br>*Smallest unit is one test.*<br>*Question relevant when: selected(${q103_L14_full_list} , '2')*<br>*Response constrained to: .>0*	
q303_L14c *(required)*	q303_L14c. Is this test or exam done at your facility (on-site) or at an off-site laboratory?<br>*Question relevant when: selected(${q103_L14_full_list} , '2')*	1 On-site<br>2 Off-site<br>99 Don't know
q303_L15	q303_L15. Pregnancy test - urine<br>*Question relevant when: selected(${q103_L15_full_list} , '2')*	
q303_L15a *(required)*	q303_L15a. What percent of patients require this item?<br>*Question relevant when: selected(${q103_L15_full_list} , '2')*<br>*Response constrained to: .>0 and .<=100 or .=999*	
q303_L15b *(required)*	q303_L15b. How many units of this test or exam are required per patient (for all treatment provided for complication)?<br>*Smallest unit is one test.*<br>*Question relevant when: selected(${q103_L15_full_list} , '2')*<br>*Response constrained to: .>0*	
q303_L15c *(required)*	q303_L15c. Is this test or exam done at your facility (on-site) or at an off-site laboratory?<br>*Question relevant when: selected(${q103_L15_full_list} , '2')*	1 On-site<br>2 Off-site<br>99 Don't know
q303_L16	q303_L16. Ultrasound<br>*Question relevant when: selected(${q103_L16_full_list} , '2')*	
q303_L16a *(required)*	q303_L16a. What percent of patients require this item?<br>*Question relevant when: selected(${q103_L16_full_list} , '2')*<br>*Response constrained to: .>0 and .<=100 or .=999*	
q303_L16b *(required)*	q303_L16b. How many units of this test or exam are required per patient (for all treatment provided for complication)?<br>*Smallest unit is one scan.*<br>*Question relevant when: selected(${q103_L16_full_list} , '2')*<br>*Response constrained to: .>0*	
q303_L16c *(required)*	q303_L16c. Is this test or exam done at your facility (on-site) or at an off-site laboratory?<br>*Question relevant when: selected(${q103_L16_full_list} , '2')*	1 On-site<br>2 Off-site<br>99 Don't know
C. Laboratory tests, supplies - Sepsis(5)<br>*Group relevant when: selected(${section_three_skip_lab} , '1')*		
note_303_Urine	***Urine***	
q303_L17	q303_L17. Urine dipstick<br>*Question relevant when: selected(${q103_L17_full_list} , '2')*	
q303_L17a *(required)*	q303_L17a. What percent of patients require this item?<br>*Question relevant when: selected(${q103_L17_full_list} , '2')*<br>*Response constrained to: .>0 and .<=100 or .=999*	
q303_L17b *(required)*	q303_L17b. How many units of this test or exam are required per patient (for all treatment provided for complication)?<br>*Smallest unit is one dipstick.*<br>*Question relevant when: selected(${q103_L17_full_list} , '2')*	

	Response constrained to: .>0	

Field	Question	Answer
q303_L17c *(required)*	q303_L17c. Is this test or exam done at your facility (on-site) or at an off-site laboratory?<br>*Question relevant when: selected(${q103_L17_full_list} , '2')*	1 On-site<br>2 Off-site<br>99 Don't know

C. Laboratory tests, supplies - Sepsis (6)<br>*Group relevant when: selected(${section_three_skip_lab} , '1')*		
note_303_Other	***Other lab tests - suggestions?***	
q303_L18	q303_L18. Lab test other 1: "[q103_L18_full_list_other]"<br>*Question relevant when: selected(${q103_L18_full_list} , '2')*	
q303_L18a *(required)*	q303_L18a. What percent of patients require this item?<br>*Question relevant when: selected(${q103_L18_full_list} , '2')*<br>*Response constrained to: .>0 and .<=100 or .=999*	
q303_L18b *(required)*	q303_L18b. How many units of this test or exam are required per patient (for all treatment provided for complication)?<br>*Question relevant when: selected(${q103_L18_full_list} , '2')*<br>*Response constrained to: .>0*	
q303_L18c *(required)*	q303_L18c. Is this test or exam done at your facility (on-site) or at an off-site laboratory?<br>*Question relevant when: selected(${q103_L18_full_list} , '2')*	1 On-site<br>2 Off-site<br>99 Don't know
q303_L19	q303_L19. Lab test other 2: "[q103_L19_full_list_other]"<br>*Question relevant when: selected(${q103_L19_full_list} , '2')*	
q303_L19a *(required)*	q303_L19a. What percent of patients require this item?<br>*Question relevant when: selected(${q103_L19_full_list} , '2')*<br>*Response constrained to: .>0 and .<=100 or .=999*	
q303_L19b *(required)*	q303_L19b. How many units of this test or exam are required per patient (for all treatment provided for complication)?<br>*Question relevant when: selected(${q103_L19_full_list} , '2')*<br>*Response constrained to: .>0*	
q303_L19c *(required)*	q303_L19c. Is this test or exam done at your facility (on-site) or at an off-site laboratory?<br>*Question relevant when: selected(${q103_L19_full_list} , '2')*	1 On-site<br>2 Off-site<br>99 Don't know
q303_L20	q303_L20. Lab test other 3: "[q103_L20_full_list_other]"<br>*Question relevant when: selected(${q103_L20_full_list} , '2')*	
q303_L20a *(required)*	q303_L20a. What percent of patients require this item?<br>*Question relevant when: selected(${q103_L20_full_list} , '2')*<br>*Response constrained to: .>0 and .<=100 or .=999*	
q303_L20b *(required)*	q303_L20b. How many units of this test or exam are required per patient (for all treatment provided for complication)?<br>*Question relevant when: selected(${q103_L20_full_list} , '2')*<br>*Response constrained to: .>0*	
q303_L20c *(required)*	q303_L20c. Is this test or exam done at your facility (on-site) or at an off-site laboratory?<br>*Question relevant when: selected(${q103_L20_full_list} , '2')*	1 On-site<br>2 Off-site<br>99 Don't know
q303_L21	q303_L21. Lab test other 4: "[q103_L21_full_list_other]"<br>*Question relevant when: selected(${q103_L21_full_list} , '2')*	
q303_L21a *(required)*	q303_L21a. What percent of patients require this item?<br>*Question relevant when: selected(${q103_L21_full_list} , '2')*<br>*Response constrained to: .>0 and .<=100 or .=999*	
q303_L21b *(required)*	q303_L21b. How many units of this test or exam are required per patient (for all treatment provided for complication)?<br>*Question relevant when: selected(${q103_L21_full_list} , '2')*<br>*Response constrained to: .>0*	
q303_L21c *(required)*	q303_L21c. Is this test or exam done at your facility (on-site) or at an off-site laboratory?<br>*Question relevant when: selected(${q103_L21_full_list} , '2')*	1 On-site<br>2 Off-site<br>99 Don't know
q303_L22	q303_L22. Lab test other 5: "[q103_L22_full_list_other]"<br>*Question relevant when: selected(${q103_L22_full_list} , '2')*	
q303_L22a *(required)*	q303_L22a. What percent of patients require this item?<br>*Question relevant when: selected(${q103_L22_full_list} , '2')*<br>*Response constrained to: .>0 and .<=100 or .=999*	
q303_L22b *(required)*	q303_L22b. How many units of this test or exam are required per patient (for all treatment provided for complication)?<br>*Question relevant when: selected(${q103_L22_full_list} , '2')*<br>*Response constrained to: .>0*	
q303_L22c *(required)*	q303_L22c. Is this test or exam done at your facility (on-site) or at an off-site laboratory?<br>*Question relevant when: selected(${q103_L22_full_list} , '2')*	1 On-site<br>2 Off-site<br>99 Don't know

group_section_four_intro

group_section_four_intro

Field	Question	Answer
section4_start	SECTION IV. SHOCK - USAGE OF ALL ITEMS	
section_four_skip_lab	INTERVIEWER: WOULD YOU LIKE TO COMPLETE THIS SECTION NOW OR SKIP THIS SECTION AND RETURN TO IT LATER?<br>*You may need to skip if the participant has indicated that s/he cannot answer the questions in this section.*	1 Do not skip, complete this section now.<br>2 Skip and come back to this section later.
group_section_four_introB<br>*Group relevant when: selected(${section_four_skip_lab} , '1')*		
section4_start2	In this section of the interview, we will review all of the items that you said are used for management of shock. For each item that is used, I'm going to ask questions on how many women need it and how much of it is used.	
section4_start3	INTERVIEWER: ENTER WHOLE NUMBERS OR DECIMALS. DO NOT TYPE PERCENT SIGNS. ENTER 999 FOR ANY THAT ARE UNKNOWN.	
C. Laboratory tests, supplies - Shock (1)<br>*Group relevant when: selected(${section_four_skip_lab} , '1')*		
note_403_blood	***Blood tests***	
q403_L1	q403_L1. Blood glucose strip<br>*Question relevant when: selected(${q103_L1_full_list} , '3')*	
q403_L1a (required)	q403_L1a. What percent of patients require this item?<br>*Question relevant when: selected(${q103_L1_full_list} , '3')*<br>*Response constrained to: .>0 and .<=100 or .=999*	
q403_L1b (required)	q403_L1b. How many units of this test or exam are required per patient (for all treatment provided for complication)?<br>*Smallest unit is one strip.*<br>*Question relevant when: selected(${q103_L1_full_list} , '3')*<br>*Response constrained to: .>0*	
q403_L1c (required)	q403_L1c. Is this test or exam done at your facility (on-site) or at an off-site laboratory?<br>*Question relevant when: selected(${q103_L1_full_list} , '3')*	1 On-site<br>2 Off-site<br>99 Don't know
q403_L2	q403_L2. Blood group test, A/B monoclonal<br>*Question relevant when: selected(${q103_L2_full_list} , '3')*	
q403_L2a (required)	q403_L2a. What percent of patients require this item?<br>*Question relevant when: selected(${q103_L2_full_list} , '3')*<br>*Response constrained to: .>0 and .<=100 or .=999*	
q403_L2b (required)	q403_L2b. How many units of this test or exam are required per patient (for all treatment provided for complication)?<br>*Smallest unit is one test.*<br>*Question relevant when: selected(${q103_L2_full_list} , '3')*<br>*Response constrained to: .>0*	
q403_L2c (required)	q403_L2c. Is this test or exam done at your facility (on-site) or at an off-site laboratory?<br>*Question relevant when: selected(${q103_L2_full_list} , '3')*	1 On-site<br>2 Off-site<br>99 Don't know
q403_L3	q403_L3. Full blood count<br>*Question relevant when: selected(${q103_L3_full_list} , '3')*	
q403_L3a (required)	q403_L3a. What percent of patients require this item?<br>*Question relevant when: selected(${q103_L3_full_list} , '3')*<br>*Response constrained to: .>0 and .<=100 or .=999*	
q403_L3b (required)	q403_L3b. How many units of this test or exam are required per patient (for all treatment provided for complication)?<br>*Smallest unit is one test.*<br>*Question relevant when: selected(${q103_L3_full_list} , '3')*<br>*Response constrained to: .>0*	
q403_L3c (required)	q403_L3c. Is this test or exam done at your facility (on-site) or at an off-site laboratory?<br>*Question relevant when: selected(${q103_L3_full_list} , '3')*	1 On-site<br>2 Off-site<br>99 Don't know
q403_L4	q403_L4. Hematocrit test<br>*Question relevant when: selected(${q103_L4_full_list} , '3')*	
q403_L4a (required)	q403_L4a. What percent of patients require this item?<br>*Question relevant when: selected(${q103_L4_full_list} , '3')*<br>*Response constrained to: .>0 and .<=100 or .=999*	
q403_L4b (required)	q403_L4b. How many units of this test or exam are required per patient (for all treatment provided for complication)?<br>*Smallest unit is one test.*<br>*Question relevant when: selected(${q103_L4_full_list} , '3')*<br>*Response constrained to: .>0*	
q403_L4c (required)	q403_L4c. Is this test or exam done at your facility (on-site) or at an off-site laboratory?<br>*Question relevant when: selected(${q103_L4_full_list} , '3')*	1 On-site<br>2 Off-site

Field	Question	Answer
		99 Don't know
q403_L5	q403_L5. Hemoglobin test<br>*Question relevant when: selected(${q103_L5_full_list} , '3')*	
q403_L5a *(required)*	q403_L5a. What percent of patients require this item?<br>*Question relevant when: selected(${q103_L5_full_list} , '3')*<br>*Response constrained to: .>0 and .<=100 or .=999*	
q403_L5b *(required)*	q403_L5b. How many units of this test or exam are required per patient (for all treatment provided for complication)?<br>*Smallest unit is one test.*<br>*Question relevant when: selected(${q103_L5_full_list} , '3')*<br>*Response constrained to: .>0*	
q403_L5c *(required)*	q403_L5c. Is this test or exam done at your facility (on-site) or at an off-site laboratory?<br>*Question relevant when: selected(${q103_L5_full_list} , '3')*	1 On-site<br>2 Off-site<br>99 Don't know
q403_L6	q403_L6. Rhesus factor test<br>*Question relevant when: selected(${q103_L6_full_list} , '3')*	
q403_L6a *(required)*	q403_L6a. What percent of patients require this item?<br>*Question relevant when: selected(${q103_L6_full_list} , '3')*<br>*Response constrained to: .>0 and .<=100 or .=999*	
q403_L6b *(required)*	q403_L6b. How many units of this test or exam are required per patient (for all treatment provided for complication)?<br>*Smallest unit is one test.*<br>*Question relevant when: selected(${q103_L6_full_list} , '3')*<br>*Response constrained to: .>0*	
q403_L6c *(required)*	q403_L6c. Is this test or exam done at your facility (on-site) or at an off-site laboratory?<br>*Question relevant when: selected(${q103_L6_full_list} , '3')*	1 On-site<br>2 Off-site<br>99 Don't know
q403_L7	q403_L7. Test of bleeding time<br>*Question relevant when: selected(${q103_L7_full_list} , '3')*	
q403_L7a *(required)*	q403_L7a. What percent of patients require this item?<br>*Question relevant when: selected(${q103_L7_full_list} , '3')*<br>*Response constrained to: .>0 and .<=100 or .=999*	
q403_L7b *(required)*	q403_L7b. How many units of this test or exam are required per patient (for all treatment provided for complication)?<br>*Smallest unit is one test.*<br>*Question relevant when: selected(${q103_L7_full_list} , '3')*<br>*Response constrained to: .>0*	
q403_L7c *(required)*	q403_L7c. Is this test or exam done at your facility (on-site) or at an off-site laboratory?<br>*Question relevant when: selected(${q103_L7_full_list} , '3')*	1 On-site<br>2 Off-site<br>99 Don't know
q403_L8	q403_L8. White blood cell count<br>*Question relevant when: selected(${q103_L8_full_list} , '3')*	
q403_L8a *(required)*	q403_L8a. What percent of patients require this item?<br>*Question relevant when: selected(${q103_L8_full_list} , '3')*<br>*Response constrained to: .>0 and .<=100 or .=999*	
q403_L8b *(required)*	q403_L8b. How many units of this test or exam are required per patient (for all treatment provided for complication)?<br>*Smallest unit is one test.*<br>*Question relevant when: selected(${q103_L8_full_list} , '3')*<br>*Response constrained to: .>0*	
q403_L8c *(required)*	q403_L8c. Is this test or exam done at your facility (on-site) or at an off-site laboratory?<br>*Question relevant when: selected(${q103_L8_full_list} , '3')*	1 On-site<br>2 Off-site<br>99 Don't know
C. Laboratory tests, supplies - Shock (2)<br>*Group relevant when: selected(${section_four_skip_lab} , '1')*		
note_403_HIV_STI	***HIV and STI tests***	
q403_L9	q403_L9. HIV test<br>*Question relevant when: selected(${q103_L9_full_list} , '3')*	
q403_L9a *(required)*	q403_L9a. What percent of patients require this item?<br>*Question relevant when: selected(${q103_L9_full_list} , '3')*<br>*Response constrained to: .>0 and .<=100 or .=999*	
q403_L9b *(required)*	q403_L9b. How many units of this test or exam are required per patient (for all treatment provided for complication)?<br>*Smallest unit is one test.*<br>*Question relevant when: selected(${q103_L9_full_list} , '3')*<br>*Response constrained to: .>0*	
q403_L9c *(required)*	q403_L9c. Is this test or exam done at your facility (on-site) or at an off-site laboratory?<br>*Question relevant when: selected(${q103_L9_full_list} , '3')*	1 On-site<br>2 Off-site

Field	Question	Answer
		99 Don't know
q403_L10	q403_L10. Rapid test for Hepatitis C<br>*Question relevant when: selected(${q103_L10_full_list} , '3')*	
q403_L10a *(required)*	q403_L10a. What percent of patients require this item?<br>*Question relevant when: selected(${q103_L10_full_list} , '3')*<br>*Response constrained to: .>0 and .<=100 or .=999*	
q403_L10b *(required)*	q403_L10b. How many units of this test or exam are required per patient (for all treatment provided for complication)?<br>*Smallest unit is one test.*<br>*Question relevant when: selected(${q103_L10_full_list} , '3')*<br>*Response constrained to: .>0*	
q403_L10c *(required)*	q403_L10c. Is this test or exam done at your facility (on-site) or at an off-site laboratory?<br>*Question relevant when: selected(${q103_L10_full_list} , '3')*	1 On-site<br>2 Off-site<br>99 Don't know
q403_L11	q403_L11. Syphilis test<br>*Question relevant when: selected(${q103_L11_full_list} , '3')*	
q403_L11a *(required)*	q403_L11a. What percent of patients require this item?<br>*Question relevant when: selected(${q103_L11_full_list} , '3')*<br>*Response constrained to: .>0 and .<=100 or .=999*	
q403_L11b *(required)*	q403_L11b. How many units of this test or exam are required per patient (for all treatment provided for complication)?<br>*Smallest unit is one test.*<br>*Question relevant when: selected(${q103_L11_full_list} , '3')*<br>*Response constrained to: .>0*	
q403_L11c *(required)*	q403_L11c. Is this test or exam done at your facility (on-site) or at an off-site laboratory?<br>*Question relevant when: selected(${q103_L11_full_list} , '3')*	1 On-site<br>2 Off-site<br>99 Don't know
C. Laboratory tests, supplies - Shock (3)<br>*Group relevant when: selected(${section_four_skip_lab} , '1')*		
note_403_malaria	***Malaria***	
q403_L12	q403_L12. Rapid test for Malaria<br>*Question relevant when: selected(${q103_L12_full_list} , '3')*	
q403_L12a *(required)*	q403_L12a. What percent of patients require this item?<br>*Question relevant when: selected(${q103_L12_full_list} , '3')*<br>*Response constrained to: .>0 and .<=100 or .=999*	
q403_L12b *(required)*	q403_L12b. How many units of this test or exam are required per patient (for all treatment provided for complication)?<br>*Smallest unit is one test.*<br>*Question relevant when: selected(${q103_L12_full_list} , '3')*<br>*Response constrained to: .>0*	
q403_L12c *(required)*	q403_L12c. Is this test or exam done at your facility (on-site) or at an off-site laboratory?<br>*Question relevant when: selected(${q103_L12_full_list} , '3')*	1 On-site<br>2 Off-site<br>99 Don't know
q403_L13	q403_L13. Thick blood smear (for malaria)<br>*Question relevant when: selected(${q103_L13_full_list} , '3')*	
q403_L13a *(required)*	q403_L13a. What percent of patients require this item?<br>*Question relevant when: selected(${q103_L13_full_list} , '3')*<br>*Response constrained to: .>0 and .<=100 or .=999*	
q403_L13b *(required)*	q403_L13b. How many units of this test or exam are required per patient (for all treatment provided for complication)?<br>*Smallest unit is one test.*<br>*Question relevant when: selected(${q103_L13_full_list} , '3')*<br>*Response constrained to: .>0*	
q403_L13c *(required)*	q403_L13c. Is this test or exam done at your facility (on-site) or at an off-site laboratory?<br>*Question relevant when: selected(${q103_L13_full_list} , '3')*	1 On-site<br>2 Off-site<br>99 Don't know
C. Laboratory tests, supplies - Shock (4)<br>*Group relevant when: selected(${section_four_skip_lab} , '1')*		
note_403_Pregnancy	***Pregnancy***	
q403_L14	q403_L14. Pregnancy test - blood<br>*Question relevant when: selected(${q103_L14_full_list} , '3')*	
q403_L14a *(required)*	q403_L14a. What percent of patients require this item?<br>*Question relevant when: selected(${q103_L14_full_list} , '3')*<br>*Response constrained to: .>0 and .<=100 or .=999*	
q403_L14b *(required)*	q403_L14b. How many units of this test or exam are required per patient (for all treatment provided for complication)?<br>*Smallest unit is one test.*<br>*Question relevant when: selected(${q103_L14_full_list} , '3')*	

Field	Question	Answer
	Response constrained to: .>0	
q403_L14c *(required)*	q403_L14c. Is this test or exam done at your facility (on-site) or at an off-site laboratory?<br>*Question relevant when: selected(${q103_L14_full_list} , '3')*	1 On-site<br>2 Off-site<br>99 Don't know

q403_L15	q403_L15. Pregnancy test - urine<br>*Question relevant when: selected(${q103_L15_full_list} , '3')*	
q403_L15a *(required)*	q403_L15a. What percent of patients require this item?<br>*Question relevant when: selected(${q103_L15_full_list} , '3')*<br>*Response constrained to: .>0 and .<=100 or .=999*	
q403_L15b *(required)*	q403_L15b. How many units of this test or exam are required per patient (for all treatment provided for complication)?<br>*Smallest unit is one test.*<br>*Question relevant when: selected(${q103_L15_full_list} , '3')*<br>*Response constrained to: .>0*	
q403_L15c *(required)*	q403_L15c. Is this test or exam done at your facility (on-site) or at an off-site laboratory?<br>*Question relevant when: selected(${q103_L15_full_list} , '3')*	1 On-site<br>2 Off-site<br>99 Don't know
q403_L16	q403_L16. Ultrasound<br>*Question relevant when: selected(${q103_L16_full_list} , '3')*	
q403_L16a *(required)*	q403_L16a. What percent of patients require this item?<br>*Question relevant when: selected(${q103_L16_full_list} , '3')*<br>*Response constrained to: .>0 and .<=100 or .=999*	
q403_L16b *(required)*	q403_L16b. How many units of this test or exam are required per patient (for all treatment provided for complication)?<br>*Smallest unit is one scan.*<br>*Question relevant when: selected(${q103_L16_full_list} , '3')*<br>*Response constrained to: .>0*	
q403_L16c *(required)*	q403_L16c. Is this test or exam done at your facility (on-site) or at an off-site laboratory?<br>*Question relevant when: selected(${q103_L16_full_list} , '3')*	1 On-site<br>2 Off-site<br>99 Don't know
C. Laboratory tests, supplies - Shock(5)<br>*Group relevant when: selected(${section_four_skip_lab} , '1')*		
note_403_Urine	***Urine***	
q403_L17	q403_L17. Urine dipstick<br>*Question relevant when: selected(${q103_L17_full_list} , '3')*	
q403_L17a *(required)*	q403_L17a. What percent of patients require this item?<br>*Question relevant when: selected(${q103_L17_full_list} , '3')*<br>*Response constrained to: .>0 and .<=100 or .=999*	
q403_L17b *(required)*	q403_L17b. How many units of this test or exam are required per patient (for all treatment provided for complication)?<br>*Smallest unit is one dipstick.*<br>*Question relevant when: selected(${q103_L17_full_list} , '3')*<br>*Response constrained to: .>0*	
q403_L17c *(required)*	q403_L17c. Is this test or exam done at your facility (on-site) or at an off-site laboratory?<br>*Question relevant when: selected(${q103_L17_full_list} , '3')*	1 On-site<br>2 Off-site<br>99 Don't know
C. Laboratory tests, supplies - Shock (6)<br>*Group relevant when: selected(${section_four_skip_lab} , '1')*		
note_403_Other	***Other lab tests - suggestions?***	
q403_L18	q403_L18. Lab test other 1: "[q103_L18_full_list_other]"<br>*Question relevant when: selected(${q103_L18_full_list} , '3')*	
q403_L18a *(required)*	q403_L18a. What percent of patients require this item?<br>*Question relevant when: selected(${q103_L18_full_list} , '3')*<br>*Response constrained to: .>0 and .<=100 or .=999*	
q403_L18b *(required)*	q403_L18b. How many units of this test or exam are required per patient (for all treatment provided for complication)?<br>*Question relevant when: selected(${q103_L18_full_list} , '3')*<br>*Response constrained to: .>0*	
q403_L18c *(required)*	q403_L18c. Is this test or exam done at your facility (on-site) or at an off-site laboratory?<br>*Question relevant when: selected(${q103_L18_full_list} , '3')*	1 On-site<br>2 Off-site<br>99 Don't know
q403_L19	q403_L19. Lab test other 2: "[q103_L19_full_list_other]"<br>*Question relevant when: selected(${q103_L19_full_list} , '3')*	
q403_L19a *(required)*	q403_L19a. What percent of patients require this item?<br>*Question relevant when: selected(${q103_L19_full_list} , '3')*<br>*Response constrained to: .>0 and .<=100 or .=999*	
q403_L19b *(required)*	q403_L19b. How many units of this test or exam are required per patient (for all treatment provided for complication)?<br>*Question relevant when: selected(${q103_L19_full_list} , '3')*	

Field	Question	Answer
	Response constrained to: .>0	
q403_L19c *(required)*	q403_L19c. Is this test or exam done at your facility (on-site) or at an off-site laboratory?<br>*Question relevant when: selected(${q103_L19_full_list} , '3')*	1 On-site<br>2 Off-site<br>99 Don't know
q403_L20	q403_L20. Lab test other 3: "[q103_L20_full_list_other]"<br>*Question relevant when: selected(${q103_L20_full_list} , '3')*	
q403_L20a *(required)*	q403_L20a. What percent of patients require this item?<br>*Question relevant when: selected(${q103_L20_full_list} , '3')*<br>*Response constrained to: .>0 and .<=100 or .=999*	
q403_L20b *(required)*	q403_L20b. How many units of this test or exam are required per patient (for all treatment provided for complication)?<br>*Question relevant when: selected(${q103_L20_full_list} , '3')*<br>*Response constrained to: .>0*	
q403_L20c *(required)*	q403_L20c. Is this test or exam done at your facility (on-site) or at an off-site laboratory?<br>*Question relevant when: selected(${q103_L20_full_list} , '3')*	1 On-site<br>2 Off-site<br>99 Don't know
q403_L21	q403_L21. Lab test other 4: "[q103_L21_full_list_other]"<br>*Question relevant when: selected(${q103_L21_full_list} , '3')*	
q403_L21a *(required)*	q403_L21a. What percent of patients require this item?<br>*Question relevant when: selected(${q103_L21_full_list} , '3')*<br>*Response constrained to: .>0 and .<=100 or .=999*	
q403_L21b *(required)*	q403_L21b. How many units of this test or exam are required per patient (for all treatment provided for complication)?<br>*Question relevant when: selected(${q103_L21_full_list} , '3')*<br>*Response constrained to: .>0*	
q403_L21c *(required)*	q403_L21c. Is this test or exam done at your facility (on-site) or at an off-site laboratory?<br>*Question relevant when: selected(${q103_L21_full_list} , '3')*	1 On-site<br>2 Off-site<br>99 Don't know
q403_L22	q403_L22. Lab test other 5: "[q103_L22_full_list_other]"<br>*Question relevant when: selected(${q103_L22_full_list} , '3')*	
q403_L22a *(required)*	q403_L22a. What percent of patients require this item?<br>*Question relevant when: selected(${q103_L22_full_list} , '3')*<br>*Response constrained to: .>0 and .<=100 or .=999*	
q403_L22b *(required)*	q403_L22b. How many units of this test or exam are required per patient (for all treatment provided for complication)?<br>*Question relevant when: selected(${q103_L22_full_list} , '3')*<br>*Response constrained to: .>0*	
q403_L22c *(required)*	q403_L22c. Is this test or exam done at your facility (on-site) or at an off-site laboratory?<br>*Question relevant when: selected(${q103_L22_full_list} , '3')*	1 On-site<br>2 Off-site<br>99 Don't know
group_section_five_intro		
section5_start	**SECTION V. LACERATIONS - USAGE OF ALL ITEMS**	
section_five_skip_lab	INTERVIEWER: WOULD YOU LIKE TO COMPLETE THIS SECTION NOW OR SKIP THIS SECTION AND RETURN TO IT LATER?<br>*You may need to skip if the participant has indicated that s/he cannot answer the questions in this section.*	1 Do not skip, complete this section now.<br>2 Skip and come back to this section later.
group_section_five_introB<br>*Group relevant when: selected(${section_five_skip_lab} , '1')*		
section5_start2	In this section of the interview, we will review all of the items that you said are used for management of cervical or vaginal lacerations. For each item that is used, I'm going to ask questions on how many women need it and how much of it is used.	
section5_start3	INTERVIEWER: ENTER WHOLE NUMBERS OR DECIMALS. DO NOT TYPE PERCENT SIGNS. ENTER 999 FOR ANY THAT ARE UNKNOWN.	
C. Laboratory tests, supplies - Lacerations (1)<br>*Group relevant when: selected(${section_five_skip_lab} , '1')*		
note_503_blood	***Blood tests***	
q503_L1	q503_L1. Blood glucose strip<br>*Question relevant when: selected(${q103_L1_full_list} , '4')*	
q503_L1a *(required)*	q503_L1a. What percent of patients require this item?<br>*Question relevant when: selected(${q103_L1_full_list} , '4')*<br>*Response constrained to: .>0 and .<=100 or .=999*	
q503_L1b *(required)*	q503_L1b. How many units of this test or exam are required per patient (for all treatment provided for complication)?	

Field	Question	Answer
	Smallest unit is one strip.<br>*Question relevant when: selected(${q103_L1_full_list} , '4')*<br>*Response constrained to: .>0*	
q503_L1c *(required)*	q503_L1c. Is this test or exam done at your facility (on-site) or at an off-site laboratory?<br>*Question relevant when: selected(${q103_L1_full_list} , '4')*	1 On-site<br>2 Off-site<br>99 Don't know
q503_L2	q503_L2. Blood group test, A/B monoclonal<br>*Question relevant when: selected(${q103_L2_full_list} , '4')*	
q503_L2a *(required)*	q503_L2a. What percent of patients require this item?<br>*Question relevant when: selected(${q103_L2_full_list} , '4')*<br>*Response constrained to: .>0 and .<=100 or .=999*	
q503_L2b *(required)*	q503_L2b. How many units of this test or exam are required per patient (for all treatment provided for complication)?<br>*Smallest unit is one test.*<br>*Question relevant when: selected(${q103_L2_full_list} , '4')*<br>*Response constrained to: .>0*	
q503_L2c *(required)*	q503_L2c. Is this test or exam done at your facility (on-site) or at an off-site laboratory?<br>*Question relevant when: selected(${q103_L2_full_list} , '4')*	1 On-site<br>2 Off-site<br>99 Don't know
q503_L3	q503_L3. Full blood count<br>*Question relevant when: selected(${q103_L3_full_list} , '4')*	
q503_L3a *(required)*	q503_L3a. What percent of patients require this item?<br>*Question relevant when: selected(${q103_L3_full_list} , '4')*<br>*Response constrained to: .>0 and .<=100 or .=999*	
q503_L3b *(required)*	q503_L3b. How many units of this test or exam are required per patient (for all treatment provided for complication)?<br>*Smallest unit is one test.*<br>*Question relevant when: selected(${q103_L3_full_list} , '4')*<br>*Response constrained to: .>0*	
q503_L3c *(required)*	q503_L3c. Is this test or exam done at your facility (on-site) or at an off-site laboratory?<br>*Question relevant when: selected(${q103_L3_full_list} , '4')*	1 On-site<br>2 Off-site<br>99 Don't know
q503_L4	q503_L4. Hematocrit test<br>*Question relevant when: selected(${q103_L4_full_list} , '4')*	
q503_L4a *(required)*	q503_L4a. What percent of patients require this item?<br>*Question relevant when: selected(${q103_L4_full_list} , '4')*<br>*Response constrained to: .>0 and .<=100 or .=999*	
q503_L4b *(required)*	q503_L4b. How many units of this test or exam are required per patient (for all treatment provided for complication)?<br>*Smallest unit is one test.*<br>*Question relevant when: selected(${q103_L4_full_list} , '4')*<br>*Response constrained to: .>0*	
q503_L4c *(required)*	q503_L4c. Is this test or exam done at your facility (on-site) or at an off-site laboratory?<br>*Question relevant when: selected(${q103_L4_full_list} , '4')*	1 On-site<br>2 Off-site<br>99 Don't know
q503_L5	q503_L5. Hemoglobin test<br>*Question relevant when: selected(${q103_L5_full_list} , '4')*	
q503_L5a *(required)*	q503_L5a. What percent of patients require this item?<br>*Question relevant when: selected(${q103_L5_full_list} , '4')*<br>*Response constrained to: .>0 and .<=100 or .=999*	
q503_L5b *(required)*	q503_L5b. How many units of this test or exam are required per patient (for all treatment provided for complication)?<br>*Smallest unit is one test.*<br>*Question relevant when: selected(${q103_L5_full_list} , '4')*<br>*Response constrained to: .>0*	
q503_L5c *(required)*	q503_L5c. Is this test or exam done at your facility (on-site) or at an off-site laboratory?<br>*Question relevant when: selected(${q103_L5_full_list} , '4')*	1 On-site<br>2 Off-site<br>99 Don't know
q503_L6	q503_L6. Rhesus factor test<br>*Question relevant when: selected(${q103_L6_full_list} , '4')*	
q503_L6a *(required)*	q503_L6a. What percent of patients require this item?<br>*Question relevant when: selected(${q103_L6_full_list} , '4')*<br>*Response constrained to: .>0 and .<=100 or .=999*	
q503_L6b *(required)*	q503_L6b. How many units of this test or exam are required per patient (for all treatment provided for complication)?<br>*Smallest unit is one test.*<br>*Question relevant when: selected(${q103_L6_full_list} , '4')*<br>*Response constrained to: .>0*	

Field	Question	Answer
	Response constrained to: .>0	
q503_L6c *(required)*	q503_L6c. Is this test or exam done at your facility (on-site) or at an off-site laboratory?<br>*Question relevant when: selected(${q103_L6_full_list} , '4')*	1 On-site<br>2 Off-site<br>99 Don't know

q503_L7	q503_L7. Test of bleeding time<br>*Question relevant when: selected(${q103_L7_full_list} , '4')*	
q503_L7a *(required)*	q503_L7a. What percent of patients require this item?<br>*Question relevant when: selected(${q103_L7_full_list} , '4')*<br>*Response constrained to: .>0 and .<=100 or .=999*	
q503_L7b *(required)*	q503_L7b. How many units of this test or exam are required per patient (for all treatment provided for complication)?<br>*Smallest unit is one test.*<br>*Question relevant when: selected(${q103_L7_full_list} , '4')*<br>*Response constrained to: .>0*	
q503_L7c *(required)*	q503_L7c. Is this test or exam done at your facility (on-site) or at an off-site laboratory?<br>*Question relevant when: selected(${q103_L7_full_list} , '4')*	1 On-site<br>2 Off-site<br>99 Don't know
q503_L8	q503_L8. White blood cell count<br>*Question relevant when: selected(${q103_L8_full_list} , '4')*	
q503_L8a *(required)*	q503_L8a. What percent of patients require this item?<br>*Question relevant when: selected(${q103_L8_full_list} , '4')*<br>*Response constrained to: .>0 and .<=100 or .=999*	
q503_L8b *(required)*	q503_L8b. How many units of this test or exam are required per patient (for all treatment provided for complication)?<br>*Smallest unit is one test.*<br>*Question relevant when: selected(${q103_L8_full_list} , '4')*<br>*Response constrained to: .>0*	
q503_L8c *(required)*	q503_L8c. Is this test or exam done at your facility (on-site) or at an off-site laboratory?<br>*Question relevant when: selected(${q103_L8_full_list} , '4')*	1 On-site<br>2 Off-site<br>99 Don't know
C. Laboratory tests, supplies - Lacerations (2)<br>*Group relevant when: selected(${section_five_skip_lab} , '1')*		
note_503_HIV_STI	***HIV and STI tests***	
q503_L9	q503_L9. HIV test<br>*Question relevant when: selected(${q103_L9_full_list} , '4')*	
q503_L9a *(required)*	q503_L9a. What percent of patients require this item?<br>*Question relevant when: selected(${q103_L9_full_list} , '4')*<br>*Response constrained to: .>0 and .<=100 or .=999*	
q503_L9b *(required)*	q503_L9b. How many units of this test or exam are required per patient (for all treatment provided for complication)?<br>*Smallest unit is one test.*<br>*Question relevant when: selected(${q103_L9_full_list} , '4')*<br>*Response constrained to: .>0*	
q503_L9c *(required)*	q503_L9c. Is this test or exam done at your facility (on-site) or at an off-site laboratory?<br>*Question relevant when: selected(${q103_L9_full_list} , '4')*	1 On-site<br>2 Off-site<br>99 Don't know
q503_L10	q503_L10. Rapid test for Hepatitis C<br>*Question relevant when: selected(${q103_L10_full_list} , '4')*	
q503_L10a *(required)*	q503_L10a. What percent of patients require this item?<br>*Question relevant when: selected(${q103_L10_full_list} , '4')*<br>*Response constrained to: .>0 and .<=100 or .=999*	
q503_L10b *(required)*	q503_L10b. How many units of this test or exam are required per patient (for all treatment provided for complication)?<br>*Smallest unit is one test.*<br>*Question relevant when: selected(${q103_L10_full_list} , '4')*<br>*Response constrained to: .>0*	
q503_L10c *(required)*	q503_L10c. Is this test or exam done at your facility (on-site) or at an off-site laboratory?<br>*Question relevant when: selected(${q103_L10_full_list} , '4')*	1 On-site<br>2 Off-site<br>99 Don't know
q503_L11	q503_L11. Syphilis test<br>*Question relevant when: selected(${q103_L11_full_list} , '4')*	
q503_L11a *(required)*	q503_L11a. What percent of patients require this item?<br>*Question relevant when: selected(${q103_L11_full_list} , '4')*<br>*Response constrained to: .>0 and .<=100 or .=999*	
q503_L11b *(required)*	q503_L11b. How many units of this test or exam are required per patient (for all treatment provided for complication)?<br>*Smallest unit is one test.*<br>*Question relevant when: selected(${q103_L11_full_list} , '4')*<br>*Response constrained to: .>0*	

Field	Question	Answer
q503_L11c *(required)*	q503_L11c. Is this test or exam done at your facility (on-site) or at an off-site laboratory?<br>*Question relevant when: selected(${q103_L11_full_list} , '4')*	1 On-site<br>2 Off-site<br>99 Don't know

C. Laboratory tests, supplies - Lacerations (3)<br>*Group relevant when: selected(${section_five_skip_lab} , '1')*		
note_503_malaria	***Malaria***	
q503_L12	q503_L12. Rapid test for Malaria<br>*Question relevant when: selected(${q103_L12_full_list} , '4')*	
q503_L12a *(required)*	q503_L12a. What percent of patients require this item?<br>*Question relevant when: selected(${q103_L12_full_list} , '4')*<br>*Response constrained to: .>0 and .<=100 or .=999*	
q503_L12b *(required)*	q503_L12b. How many units of this test or exam are required per patient (for all treatment provided for complication)?<br>*Smallest unit is one test.*<br>*Question relevant when: selected(${q103_L12_full_list} , '4')*<br>*Response constrained to: .>0*	
q503_L12c *(required)*	q503_L12c. Is this test or exam done at your facility (on-site) or at an off-site laboratory?<br>*Question relevant when: selected(${q103_L12_full_list} , '4')*	1 On-site<br>2 Off-site<br>99 Don't know
q503_L13	q503_L13. Thick blood smear (for malaria)<br>*Question relevant when: selected(${q103_L13_full_list} , '4')*	
q503_L13a *(required)*	q503_L13a. What percent of patients require this item?<br>*Question relevant when: selected(${q103_L13_full_list} , '4')*<br>*Response constrained to: .>0 and .<=100 or .=999*	
q503_L13b *(required)*	q503_L13b. How many units of this test or exam are required per patient (for all treatment provided for complication)?<br>*Smallest unit is one test.*<br>*Question relevant when: selected(${q103_L13_full_list} , '4')*<br>*Response constrained to: .>0*	
q503_L13c *(required)*	q503_L13c. Is this test or exam done at your facility (on-site) or at an off-site laboratory?<br>*Question relevant when: selected(${q103_L13_full_list} , '4')*	1 On-site<br>2 Off-site<br>99 Don't know
C. Laboratory tests, supplies - Lacerations (4)<br>*Group relevant when: selected(${section_five_skip_lab} , '1')*		
note_503_Pregnancy	***Pregnancy***	
q503_L14	q503_L14. Pregnancy test - blood<br>*Question relevant when: selected(${q103_L14_full_list} , '4')*	
q503_L14a *(required)*	q503_L14a. What percent of patients require this item?<br>*Question relevant when: selected(${q103_L14_full_list} , '4')*<br>*Response constrained to: .>0 and .<=100 or .=999*	
q503_L14b *(required)*	q503_L14b. How many units of this test or exam are required per patient (for all treatment provided for complication)?<br>*Smallest unit is one test.*<br>*Question relevant when: selected(${q103_L14_full_list} , '4')*<br>*Response constrained to: .>0*	
q503_L14c *(required)*	q503_L14c. Is this test or exam done at your facility (on-site) or at an off-site laboratory?<br>*Question relevant when: selected(${q103_L14_full_list} , '4')*	1 On-site<br>2 Off-site<br>99 Don't know
q503_L15	q503_L15. Pregnancy test - urine<br>*Question relevant when: selected(${q103_L15_full_list} , '4')*	
q503_L15a *(required)*	q503_L15a. What percent of patients require this item?<br>*Question relevant when: selected(${q103_L15_full_list} , '4')*<br>*Response constrained to: .>0 and .<=100 or .=999*	
q503_L15b *(required)*	q503_L15b. How many units of this test or exam are required per patient (for all treatment provided for complication)?<br>*Smallest unit is one test.*<br>*Question relevant when: selected(${q103_L15_full_list} , '4')*<br>*Response constrained to: .>0*	
q503_L15c *(required)*	q503_L15c. Is this test or exam done at your facility (on-site) or at an off-site laboratory?<br>*Question relevant when: selected(${q103_L15_full_list} , '4')*	1 On-site<br>2 Off-site<br>99 Don't know
q503_L16	q503_L16. Ultrasound<br>*Question relevant when: selected(${q103_L16_full_list} , '4')*	
q503_L16a *(required)*	q503_L16a. What percent of patients require this item?<br>*Question relevant when: selected(${q103_L16_full_list} , '4')*<br>*Response constrained to: .>0 and .<=100 or .=999*	
[illegible]	[illegible]	

Field	Question	Answer
q503_L16b *(required)*	q503_L16b. How many units of this test or exam are required per patient (for all treatment provided for complication)?<br>*Smallest unit is one scan.*<br>*Question relevant when: selected(${q103_L16_full_list} , '4')*<br>*Response constrained to: .>0*	
q503_L16c *(required)*	q503_L16c. Is this test or exam done at your facility (on-site) or at an off-site laboratory?<br>*Question relevant when: selected(${q103_L16_full_list} , '4')*	1 On-site<br>2 Off-site<br>99 Don't know
C. Laboratory tests, supplies - Lacerations(5)<br>*Group relevant when: selected(${section_five_skip_lab} , '1')*		
note_503_Urine	***Urine***	
q503_L17	q503_L17. Urine dipstick<br>*Question relevant when: selected(${q103_L17_full_list} , '4')*	
q503_L17a *(required)*	q503_L17a. What percent of patients require this item?<br>*Question relevant when: selected(${q103_L17_full_list} , '4')*<br>*Response constrained to: .>0 and .<=100 or .=999*	
q503_L17b *(required)*	q503_L17b. How many units of this test or exam are required per patient (for all treatment provided for complication)?<br>*Smallest unit is one dipstick.*<br>*Question relevant when: selected(${q103_L17_full_list} , '4')*<br>*Response constrained to: .>0*	
q503_L17c *(required)*	q503_L17c. Is this test or exam done at your facility (on-site) or at an off-site laboratory?<br>*Question relevant when: selected(${q103_L17_full_list} , '4')*	1 On-site<br>2 Off-site<br>99 Don't know
C. Laboratory tests, supplies - Lacerations (6)<br>*Group relevant when: selected(${section_five_skip_lab} , '1')*		
note_503_Other	***Other lab tests - suggestions?***	
q503_L18	q503_L18. Lab test other 1: "[q103_L18_full_list_other]"<br>*Question relevant when: selected(${q103_L18_full_list} , '4')*	
q503_L18a *(required)*	q503_L18a. What percent of patients require this item?<br>*Question relevant when: selected(${q103_L18_full_list} , '4')*<br>*Response constrained to: .>0 and .<=100 or .=999*	
q503_L18b *(required)*	q503_L18b. How many units of this test or exam are required per patient (for all treatment provided for complication)?<br>*Question relevant when: selected(${q103_L18_full_list} , '4')*<br>*Response constrained to: .>0*	
q503_L18c *(required)*	q503_L18c. Is this test or exam done at your facility (on-site) or at an off-site laboratory?<br>*Question relevant when: selected(${q103_L18_full_list} , '4')*	1 On-site<br>2 Off-site<br>99 Don't know
q503_L19	q503_L19. Lab test other 2: "[q103_L19_full_list_other]"<br>*Question relevant when: selected(${q103_L19_full_list} , '4')*	
q503_L19a *(required)*	q503_L19a. What percent of patients require this item?<br>*Question relevant when: selected(${q103_L19_full_list} , '4')*<br>*Response constrained to: .>0 and .<=100 or .=999*	
q503_L19b *(required)*	q503_L19b. How many units of this test or exam are required per patient (for all treatment provided for complication)?<br>*Question relevant when: selected(${q103_L19_full_list} , '4')*<br>*Response constrained to: .>0*	
q503_L19c *(required)*	q503_L19c. Is this test or exam done at your facility (on-site) or at an off-site laboratory?<br>*Question relevant when: selected(${q103_L19_full_list} , '4')*	1 On-site<br>2 Off-site<br>99 Don't know
q503_L20	q503_L20. Lab test other 3: "[q103_L20_full_list_other]"<br>*Question relevant when: selected(${q103_L20_full_list} , '4')*	
q503_L20a *(required)*	q503_L20a. What percent of patients require this item?<br>*Question relevant when: selected(${q103_L20_full_list} , '4')*<br>*Response constrained to: .>0 and .<=100 or .=999*	
q503_L20b *(required)*	q503_L20b. How many units of this test or exam are required per patient (for all treatment provided for complication)?<br>*Question relevant when: selected(${q103_L20_full_list} , '4')*<br>*Response constrained to: .>0*	
q503_L20c *(required)*	q503_L20c. Is this test or exam done at your facility (on-site) or at an off-site laboratory?<br>*Question relevant when: selected(${q103_L20_full_list} , '4')*	1 On-site<br>2 Off-site<br>99 Don't know
q503_L21	q503_L21. Lab test other 4: "[q103_L21_full_list_other]"<br>*Question relevant when: selected(${q103_L21_full_list} , '4')*	
q503_L21a *(required)*	q503_L21a. What percent of patients require this item?<br>*Question relevant when: selected(${q103_L21_full_list} , '4')*	

Field	Question	Answer
	Response constrained to: .>0 and .<=100 or .=999	
q503_L21b *(required)*	q503_L21b. How many units of this test or exam are required per patient (for all treatment provided for complication)?<br>*Question relevant when: selected(${q103_L21_full_list} , '4')*<br>*Response constrained to: .>0*	
q503_L21c *(required)*	q503_L21c. Is this test or exam done at your facility (on-site) or at an off-site laboratory?<br>*Question relevant when: selected(${q103_L21_full_list} , '4')*	1 On-site<br>2 Off-site<br>99 Don't know
q503_L22	q503_L22. Lab test other 5: "[q103_L22_full_list_other]"<br>*Question relevant when: selected(${q103_L22_full_list} , '4')*	
q503_L22a *(required)*	q503_L22a. What percent of patients require this item?<br>*Question relevant when: selected(${q103_L22_full_list} , '4')*<br>*Response constrained to: .>0 and .<=100 or .=999*	
q503_L22b *(required)*	q503_L22b. How many units of this test or exam are required per patient (for all treatment provided for complication)?<br>*Question relevant when: selected(${q103_L22_full_list} , '4')*<br>*Response constrained to: .>0*	
q503_L22c *(required)*	q503_L22c. Is this test or exam done at your facility (on-site) or at an off-site laboratory?<br>*Question relevant when: selected(${q103_L22_full_list} , '4')*	1 On-site<br>2 Off-site<br>99 Don't know
group_section_six_intro		
section6_start	**SECTION VI. PERFORATIONS - USAGE OF ALL ITEMS**	
section_six_skip_lab	INTERVIEWER: WOULD YOU LIKE TO COMPLETE THIS SECTION NOW OR SKIP THIS SECTION AND RETURN TO IT LATER?<br>*You may need to skip if the participant has indicated that s/he cannot answer the questions in this section.*	1 Do not skip, complete this section now.<br>2 Skip and come back to this section later.
group_section_six_introB<br>*Group relevant when: selected(${section_six_skip_lab} , '1')*		
section6_start2	In this section of the interview, we will review all of the items that you said are used for management of vaginal or uterine perforations. For each item that is used, I'm going to ask questions on how many women need it and how much of it is used.	
section6_start3	INTERVIEWER: ENTER WHOLE NUMBERS OR DECIMALS. DO NOT TYPE PERCENT SIGNS. ENTER 999 FOR ANY THAT ARE UNKNOWN.	
C. Laboratory tests, supplies - Perforations (1)<br>*Group relevant when: selected(${section_six_skip_lab} , '1')*		
note_603_blood	***Blood tests***	
q603_L1	q603_L1. Blood glucose strip<br>*Question relevant when: selected(${q103_L1_full_list} , '5')*	
q603_L1a *(required)*	q603_L1a. What percent of patients require this item?<br>*Question relevant when: selected(${q103_L1_full_list} , '5')*<br>*Response constrained to: .>0 and .<=100 or .=999*	
q603_L1b *(required)*	q603_L1b. How many units of this test or exam are required per patient (for all treatment provided for complication)?<br>*Smallest unit is one strip.*<br>*Question relevant when: selected(${q103_L1_full_list} , '5')*<br>*Response constrained to: .>0*	
q603_L1c *(required)*	q603_L1c. Is this test or exam done at your facility (on-site) or at an off-site laboratory?<br>*Question relevant when: selected(${q103_L1_full_list} , '5')*	1 On-site<br>2 Off-site<br>99 Don't know
q603_L2	q603_L2. Blood group test, A/B monoclonal<br>*Question relevant when: selected(${q103_L2_full_list} , '5')*	
q603_L2a *(required)*	q603_L2a. What percent of patients require this item?<br>*Question relevant when: selected(${q103_L2_full_list} , '5')*<br>*Response constrained to: .>0 and .<=100 or .=999*	
q603_L2b *(required)*	q603_L2b. How many units of this test or exam are required per patient (for all treatment provided for complication)?<br>*Smallest unit is one test.*<br>*Question relevant when: selected(${q103_L2_full_list} , '5')*<br>*Response constrained to: .>0*	
q603_L2c *(required)*	q603_L2c. Is this test or exam done at your facility (on-site) or at an off-site laboratory?<br>*Question relevant when: selected(${q103_L2_full_list} , '5')*	1 On-site<br>2 Off-site<br>99 Don't know
q603_L3	q603_L3. Full blood count<br>*Question relevant when: selected(${q103_L3_full_list} , '5')*	
q603_L3a *(required)*	q603_L3a. What percent of patients require this item?<br>*Question relevant when: selected(${q103_L3_full_list} , '5')*<br>*Response constrained to: .>0 and .<=100 or .=999*	

Response constrained to: .>0 and .<=100 or .=999

Field	Question	Answer
q603_L3b *(required)*	q603_L3b. How many units of this test or exam are required per patient (for all treatment provided for complication)?<br>*Smallest unit is one test.*<br>*Question relevant when: selected(${q103_L3_full_list} , '5')*<br>*Response constrained to: .>0*	
q603_L3c *(required)*	q603_L3c. Is this test or exam done at your facility (on-site) or at an off-site laboratory?<br>*Question relevant when: selected(${q103_L3_full_list} , '5')*	1 On-site<br>2 Off-site<br>99 Don't know
q603_L4	q603_L4. Hematocrit test<br>*Question relevant when: selected(${q103_L4_full_list} , '5')*	
q603_L4a *(required)*	q603_L4a. What percent of patients require this item?<br>*Question relevant when: selected(${q103_L4_full_list} , '5')*<br>*Response constrained to: .>0 and .<=100 or .=999*	
q603_L4b *(required)*	q603_L4b. How many units of this test or exam are required per patient (for all treatment provided for complication)?<br>*Smallest unit is one test.*<br>*Question relevant when: selected(${q103_L4_full_list} , '5')*<br>*Response constrained to: .>0*	
q603_L4c *(required)*	q603_L4c. Is this test or exam done at your facility (on-site) or at an off-site laboratory?<br>*Question relevant when: selected(${q103_L4_full_list} , '5')*	1 On-site<br>2 Off-site<br>99 Don't know
q603_L5	q603_L5. Hemoglobin test<br>*Question relevant when: selected(${q103_L5_full_list} , '5')*	
q603_L5a *(required)*	q603_L5a. What percent of patients require this item?<br>*Question relevant when: selected(${q103_L5_full_list} , '5')*<br>*Response constrained to: .>0 and .<=100 or .=999*	
q603_L5b *(required)*	q603_L5b. How many units of this test or exam are required per patient (for all treatment provided for complication)?<br>*Smallest unit is one test.*<br>*Question relevant when: selected(${q103_L5_full_list} , '5')*<br>*Response constrained to: .>0*	
q603_L5c *(required)*	q603_L5c. Is this test or exam done at your facility (on-site) or at an off-site laboratory?<br>*Question relevant when: selected(${q103_L5_full_list} , '5')*	1 On-site<br>2 Off-site<br>99 Don't know
q603_L6	q603_L6. Rhesus factor test<br>*Question relevant when: selected(${q103_L6_full_list} , '5')*	
q603_L6a *(required)*	q603_L6a. What percent of patients require this item?<br>*Question relevant when: selected(${q103_L6_full_list} , '5')*<br>*Response constrained to: .>0 and .<=100 or .=999*	
q603_L6b *(required)*	q603_L6b. How many units of this test or exam are required per patient (for all treatment provided for complication)?<br>*Smallest unit is one test.*<br>*Question relevant when: selected(${q103_L6_full_list} , '5')*<br>*Response constrained to: .>0*	
q603_L6c *(required)*	q603_L6c. Is this test or exam done at your facility (on-site) or at an off-site laboratory?<br>*Question relevant when: selected(${q103_L6_full_list} , '5')*	1 On-site<br>2 Off-site<br>99 Don't know
q603_L7	q603_L7. Test of bleeding time<br>*Question relevant when: selected(${q103_L7_full_list} , '5')*	
q603_L7a *(required)*	q603_L7a. What percent of patients require this item?<br>*Question relevant when: selected(${q103_L7_full_list} , '5')*<br>*Response constrained to: .>0 and .<=100 or .=999*	
q603_L7b *(required)*	q603_L7b. How many units of this test or exam are required per patient (for all treatment provided for complication)?<br>*Smallest unit is one test.*<br>*Question relevant when: selected(${q103_L7_full_list} , '5')*<br>*Response constrained to: .>0*	
q603_L7c *(required)*	q603_L7c. Is this test or exam done at your facility (on-site) or at an off-site laboratory?<br>*Question relevant when: selected(${q103_L7_full_list} , '5')*	1 On-site<br>2 Off-site<br>99 Don't know
q603_L8	q603_L8. White blood cell count<br>*Question relevant when: selected(${q103_L8_full_list} , '5')*	
q603_L8a *(required)*	q603_L8a. What percent of patients require this item?<br>*Question relevant when: selected(${q103_L8_full_list} , '5')*<br>*Response constrained to: .>0 and .<=100 or .=999*	
q603_L8b *(required)*	q603_L8b. How many units of this test or exam are required per patient (for all treatment provided for complication)?<br>*Smallest unit is one test.*<br>*Question relevant when: selected(${q103_L8_full_list} , '5')*<br>*Response constrained to: .>0*	

Field	Question	Answer
	Response constrained to: .>0	
q603_L8c *(required)*	q603_L8c. Is this test or exam done at your facility (on-site) or at an off-site laboratory?<br>*Question relevant when: selected(${q103_L8_full_list} , '5')*	1 On-site<br>2 Off-site<br>99 Don't know

C. Laboratory tests, supplies - Perforations (2)<br>*Group relevant when: selected(${section_six_skip_lab} , '1')*		
note_603_HIV_STI	***HIV and STI tests***	
q603_L9	q603_L9. HIV test<br>*Question relevant when: selected(${q103_L9_full_list} , '5')*	
q603_L9a *(required)*	q603_L9a. What percent of patients require this item?<br>*Question relevant when: selected(${q103_L9_full_list} , '5')*<br>*Response constrained to: .>0 and .<=100 or .=999*	
q603_L9b *(required)*	q603_L9b. How many units of this test or exam are required per patient (for all treatment provided for complication)?<br>*Smallest unit is one test.*<br>*Question relevant when: selected(${q103_L9_full_list} , '5')*<br>*Response constrained to: .>0*	
q603_L9c *(required)*	q603_L9c. Is this test or exam done at your facility (on-site) or at an off-site laboratory?<br>*Question relevant when: selected(${q103_L9_full_list} , '5')*	1 On-site<br>2 Off-site<br>99 Don't know
q603_L10	q603_L10. Rapid test for Hepatitis C<br>*Question relevant when: selected(${q103_L10_full_list} , '5')*	
q603_L10a *(required)*	q603_L10a. What percent of patients require this item?<br>*Question relevant when: selected(${q103_L10_full_list} , '5')*<br>*Response constrained to: .>0 and .<=100 or .=999*	
q603_L10b *(required)*	q603_L10b. How many units of this test or exam are required per patient (for all treatment provided for complication)?<br>*Smallest unit is one test.*<br>*Question relevant when: selected(${q103_L10_full_list} , '5')*<br>*Response constrained to: .>0*	
q603_L10c *(required)*	q603_L10c. Is this test or exam done at your facility (on-site) or at an off-site laboratory?<br>*Question relevant when: selected(${q103_L10_full_list} , '5')*	1 On-site<br>2 Off-site<br>99 Don't know
q603_L11	q603_L11. Syphilis test<br>*Question relevant when: selected(${q103_L11_full_list} , '5')*	
q603_L11a *(required)*	q603_L11a. What percent of patients require this item?<br>*Question relevant when: selected(${q103_L11_full_list} , '5')*<br>*Response constrained to: .>0 and .<=100 or .=999*	
q603_L11b *(required)*	q603_L11b. How many units of this test or exam are required per patient (for all treatment provided for complication)?<br>*Smallest unit is one test.*<br>*Question relevant when: selected(${q103_L11_full_list} , '5')*<br>*Response constrained to: .>0*	
q603_L11c *(required)*	q603_L11c. Is this test or exam done at your facility (on-site) or at an off-site laboratory?<br>*Question relevant when: selected(${q103_L11_full_list} , '5')*	1 On-site<br>2 Off-site<br>99 Don't know
C. Laboratory tests, supplies - Perforations (3)<br>*Group relevant when: selected(${section_six_skip_lab} , '1')*		
note_603_malaria	***Malaria***	
q603_L12	q603_L12. Rapid test for Malaria<br>*Question relevant when: selected(${q103_L12_full_list} , '5')*	
q603_L12a *(required)*	q603_L12a. What percent of patients require this item?<br>*Question relevant when: selected(${q103_L12_full_list} , '5')*<br>*Response constrained to: .>0 and .<=100 or .=999*	
q603_L12b *(required)*	q603_L12b. How many units of this test or exam are required per patient (for all treatment provided for complication)?<br>*Smallest unit is one test.*<br>*Question relevant when: selected(${q103_L12_full_list} , '5')*<br>*Response constrained to: .>0*	
q603_L12c *(required)*	q603_L12c. Is this test or exam done at your facility (on-site) or at an off-site laboratory?<br>*Question relevant when: selected(${q103_L12_full_list} , '5')*	1 On-site<br>2 Off-site<br>99 Don't know
q603_L13	q603_L13. Thick blood smear (for malaria)<br>*Question relevant when: selected(${q103_L13_full_list} , '5')*	
q603_L13a *(required)*	q603_L13a. What percent of patients require this item?<br>*Question relevant when: selected(${q103_L13_full_list} , '5')*<br>*Response constrained to: .>0 and .<=100 or .=999*	
[illegible]	[illegible]	

Field	Question	Answer
q603_L13b *(required)*	q603_L13b. How many units of this test or exam are required per patient (for all treatment provided for complication)?<br>*Smallest unit is one test.*<br>*Question relevant when: selected(${q103_L13_full_list} , '5')*<br>*Response constrained to: .>0*	

Field	Question	Answer
q603_L13c *(required)*	q603_L13c. Is this test or exam done at your facility (on-site) or at an off-site laboratory?<br>*Question relevant when: selected(${q103_L13_full_list} , '5')*	1 On-site<br>2 Off-site<br>99 Don't know
C. Laboratory tests, supplies - Perforations (4)<br>*Group relevant when: selected(${section_six_skip_lab} , '1')*		
note_603_Pregnancy	***Pregnancy***	
q603_L14	q603_L14. Pregnancy test - blood<br>*Question relevant when: selected(${q103_L14_full_list} , '5')*	
q603_L14a *(required)*	q603_L14a. What percent of patients require this item?<br>*Question relevant when: selected(${q103_L14_full_list} , '5')*<br>*Response constrained to: .>0 and .<=100 or .=999*	
q603_L14b *(required)*	q603_L14b. How many units of this test or exam are required per patient (for all treatment provided for complication)?<br>*Smallest unit is one test.*<br>*Question relevant when: selected(${q103_L14_full_list} , '5')*<br>*Response constrained to: .>0*	
q603_L14c *(required)*	q603_L14c. Is this test or exam done at your facility (on-site) or at an off-site laboratory?<br>*Question relevant when: selected(${q103_L14_full_list} , '5')*	1 On-site<br>2 Off-site<br>99 Don't know
q603_L15	q603_L15. Pregnancy test - urine<br>*Question relevant when: selected(${q103_L15_full_list} , '5')*	
q603_L15a *(required)*	q603_L15a. What percent of patients require this item?<br>*Question relevant when: selected(${q103_L15_full_list} , '5')*<br>*Response constrained to: .>0 and .<=100 or .=999*	
q603_L15b *(required)*	q603_L15b. How many units of this test or exam are required per patient (for all treatment provided for complication)?<br>*Smallest unit is one test.*<br>*Question relevant when: selected(${q103_L15_full_list} , '5')*<br>*Response constrained to: .>0*	
q603_L15c *(required)*	q603_L15c. Is this test or exam done at your facility (on-site) or at an off-site laboratory?<br>*Question relevant when: selected(${q103_L15_full_list} , '5')*	1 On-site<br>2 Off-site<br>99 Don't know
q603_L16	q603_L16. Ultrasound<br>*Question relevant when: selected(${q103_L16_full_list} , '5')*	
q603_L16a *(required)*	q603_L16a. What percent of patients require this item?<br>*Question relevant when: selected(${q103_L16_full_list} , '5')*<br>*Response constrained to: .>0 and .<=100 or .=999*	
q603_L16b *(required)*	q603_L16b. How many units of this test or exam are required per patient (for all treatment provided for complication)?<br>*Smallest unit is one scan.*<br>*Question relevant when: selected(${q103_L16_full_list} , '5')*<br>*Response constrained to: .>0*	
q603_L16c *(required)*	q603_L16c. Is this test or exam done at your facility (on-site) or at an off-site laboratory?<br>*Question relevant when: selected(${q103_L16_full_list} , '5')*	1 On-site<br>2 Off-site<br>99 Don't know
C. Laboratory tests, supplies - Perforations(5)<br>*Group relevant when: selected(${section_six_skip_lab} , '1')*		
note_603_Urine	***Urine***	
q603_L17	q603_L17. Urine dipstick<br>*Question relevant when: selected(${q103_L17_full_list} , '5')*	
q603_L17a *(required)*	q603_L17a. What percent of patients require this item?<br>*Question relevant when: selected(${q103_L17_full_list} , '5')*<br>*Response constrained to: .>0 and .<=100 or .=999*	
q603_L17b *(required)*	q603_L17b. How many units of this test or exam are required per patient (for all treatment provided for complication)?<br>*Smallest unit is one dipstick.*<br>*Question relevant when: selected(${q103_L17_full_list} , '5')*<br>*Response constrained to: .>0*	
q603_L17c *(required)*	q603_L17c. Is this test or exam done at your facility (on-site) or at an off-site laboratory?<br>*Question relevant when: selected(${q103_L17_full_list} , '5')*	1 On-site<br>2 Off-site<br>99 Don't know
C. Laboratory tests, supplies - Perforations (6)<br>*Group relevant when: selected(${section_six_skip_lab} , '1')*		

Field	Question	Answer
note_603_Other	***Other lab tests - suggestions?***	
q603_L18	q603_L18. Lab test other 1: "[q103_L18_full_list_other]"<br>*Question relevant when: selected(${q103_L18_full_list} , '5')*	

Field	Question	Answer
q603_L18a *(required)*	q603_L18a. What percent of patients require this item?<br>*Question relevant when: selected(${q103_L18_full_list} , '5')*<br>*Response constrained to: .>0 and .<=100 or .=999*	
q603_L18b *(required)*	q603_L18b. How many units of this test or exam are required per patient (for all treatment provided for complication)?<br>*Question relevant when: selected(${q103_L18_full_list} , '5')*<br>*Response constrained to: .>0*	
q603_L18c *(required)*	q603_L18c. Is this test or exam done at your facility (on-site) or at an off-site laboratory?<br>*Question relevant when: selected(${q103_L18_full_list} , '5')*	1 On-site<br>2 Off-site<br>99 Don't know
q603_L19	q603_L19. Lab test other 2: "[q103_L19_full_list_other]"<br>*Question relevant when: selected(${q103_L19_full_list} , '5')*	
q603_L19a *(required)*	q603_L19a. What percent of patients require this item?<br>*Question relevant when: selected(${q103_L19_full_list} , '5')*<br>*Response constrained to: .>0 and .<=100 or .=999*	
q603_L19b *(required)*	q603_L19b. How many units of this test or exam are required per patient (for all treatment provided for complication)?<br>*Question relevant when: selected(${q103_L19_full_list} , '5')*<br>*Response constrained to: .>0*	
q603_L19c *(required)*	q603_L19c. Is this test or exam done at your facility (on-site) or at an off-site laboratory?<br>*Question relevant when: selected(${q103_L19_full_list} , '5')*	1 On-site<br>2 Off-site<br>99 Don't know
q603_L20	q603_L20. Lab test other 3: "[q103_L20_full_list_other]"<br>*Question relevant when: selected(${q103_L20_full_list} , '5')*	
q603_L20a *(required)*	q603_L20a. What percent of patients require this item?<br>*Question relevant when: selected(${q103_L20_full_list} , '5')*<br>*Response constrained to: .>0 and .<=100 or .=999*	
q603_L20b *(required)*	q603_L20b. How many units of this test or exam are required per patient (for all treatment provided for complication)?<br>*Question relevant when: selected(${q103_L20_full_list} , '5')*<br>*Response constrained to: .>0*	
q603_L20c *(required)*	q603_L20c. Is this test or exam done at your facility (on-site) or at an off-site laboratory?<br>*Question relevant when: selected(${q103_L20_full_list} , '5')*	1 On-site<br>2 Off-site<br>99 Don't know
q603_L21	q603_L21. Lab test other 4: "[q103_L21_full_list_other]"<br>*Question relevant when: selected(${q103_L21_full_list} , '5')*	
q603_L21a *(required)*	q603_L21a. What percent of patients require this item?<br>*Question relevant when: selected(${q103_L21_full_list} , '5')*<br>*Response constrained to: .>0 and .<=100 or .=999*	
q603_L21b *(required)*	q603_L21b. How many units of this test or exam are required per patient (for all treatment provided for complication)?<br>*Question relevant when: selected(${q103_L21_full_list} , '5')*<br>*Response constrained to: .>0*	
q603_L21c *(required)*	q603_L21c. Is this test or exam done at your facility (on-site) or at an off-site laboratory?<br>*Question relevant when: selected(${q103_L21_full_list} , '5')*	1 On-site<br>2 Off-site<br>99 Don't know
q603_L22	q603_L22. Lab test other 5: "[q103_L22_full_list_other]"<br>*Question relevant when: selected(${q103_L22_full_list} , '5')*	
q603_L22a *(required)*	q603_L22a. What percent of patients require this item?<br>*Question relevant when: selected(${q103_L22_full_list} , '5')*<br>*Response constrained to: .>0 and .<=100 or .=999*	
q603_L22b *(required)*	q603_L22b. How many units of this test or exam are required per patient (for all treatment provided for complication)?<br>*Question relevant when: selected(${q103_L22_full_list} , '5')*<br>*Response constrained to: .>0*	
q603_L22c *(required)*	q603_L22c. Is this test or exam done at your facility (on-site) or at an off-site laboratory?<br>*Question relevant when: selected(${q103_L22_full_list} , '5')*	1 On-site<br>2 Off-site<br>99 Don't know
group_section_seven_intro		
section7_start	**SECTION VII. COSTS OF ALL ITEMS**	
section_seven_skip_lab	INTERVIEWER: WOULD YOU LIKE TO COMPLETE THIS SECTION NOW OR SKIP THIS SECTION AND RETURN TO IT LATER?<br>*You may need to skip if the participant has indicated that s/he cannot answer the questions in this section.*	1 Do not skip, complete this section now.<br>2 Skip and come back to this

Field	Question	Answer
group_section_seven_introB		section later.
Group relevant when: selected(${section_seven_skip_lab} , '1')		

section7_start2	In this section of the interview, we will review all of the items that you said are used for management of any complication type. For each item that is used, I'm going to ask how much the item costs and how it is procured.	
section7_start3	INTERVIEWER: ENTER 999 FOR ANY THAT ARE UNKNOWN.	
C. Laboratory tests, supplies - Cost (1)<br>*Group relevant when: selected(${section_seven_skip_lab} , '1')*		
note_703_blood	***Blood tests***	
q703_L1	***q703_L1. Blood glucose strip***<br>*Question relevant when: not(selected(${q103_L1_full_list} , '6')) and not(selected(${q103_L1_full_list} , '99'))*	
q703_L1a *(required)*	q703_L1a. When purchased, how many units of this item typically come in one pack, box, bottle, etc.? (e.g. 100 tablets, 1000 ml's, etc.) (Write 1 if purchased as a single item.)<br>*Smallest unit is one strip.*<br>*Question relevant when: not(selected(${q103_L1_full_list} , '6')) and not(selected(${q103_L1_full_list} , '99'))*<br>*Response constrained to: .>=0*	
q703_L1b *(required)*	q703_L1b. What is the typical purchase price for that quanity of units (i.e. the quantity noted in the question above)?<br>*Question relevant when: not(selected(${q103_L1_full_list} , '6')) and not(selected(${q103_L1_full_list} , '99'))*<br>*Response constrained to: .>=0*	
q703_L1c *(required)*	q703_L1c. Please specify the currency for the purchase price.<br>*Question relevant when: not(selected(${q103_L1_full_list} , '6')) and not(selected(${q103_L1_full_list} , '99'))*	1 Tanzanian Shillings<br>2 US dollars<br>3 Euros<br>4 Other<br>99 Don't know
q703_L1c.1	q703_L1c.1 If other currency, specify:<br>*Leave blank if not applicable*<br>*Question relevant when: not(selected(${q103_L1_full_list} , '6')) and not(selected(${q103_L1_full_list} , '99'))*	
q703_L1d *(required)*	q703_L1d. Please specify the year of the purchase price.<br>*Question relevant when: not(selected(${q103_L1_full_list} , '6')) and not(selected(${q103_L1_full_list} , '99'))*<br>*Response constrained to: .>=2000 and .<=2019 or .=999*	
q703_L2	***q703_L2. Blood group test, A/B monoclonal***<br>*Question relevant when: not(selected(${q103_L2_full_list} , '6')) and not(selected(${q103_L2_full_list} , '99'))*	
q703_L2a *(required)*	q703_L2a. When purchased, how many units of this item typically come in one pack, box, bottle, etc.? (e.g. 100 tablets, 1000 ml's, etc.) (Write 1 if purchased as a single item.)<br>*Smallest unit is one test.*<br>*Question relevant when: not(selected(${q103_L2_full_list} , '6')) and not(selected(${q103_L2_full_list} , '99'))*<br>*Response constrained to: .>=0*	
q703_L2b *(required)*	q703_L2b. What is the typical purchase price for that quanity of units (i.e. the quantity noted in the question above)?<br>*Question relevant when: not(selected(${q103_L2_full_list} , '6')) and not(selected(${q103_L2_full_list} , '99'))*<br>*Response constrained to: .>=0*	
q703_L2c *(required)*	q703_L2c. Please specify the currency for the purchase price.<br>*Question relevant when: not(selected(${q103_L2_full_list} , '6')) and not(selected(${q103_L2_full_list} , '99'))*	1 Tanzanian Shillings<br>2 US dollars<br>3 Euros<br>4 Other<br>99 Don't know
q703_L2c.1	q703_L2c.1 If other currency, specify:<br>*Leave blank if not applicable*<br>*Question relevant when: not(selected(${q103_L2_full_list} , '6')) and not(selected(${q103_L2_full_list} , '99'))*	
q703_L2d *(required)*	q703_L2d. Please specify the year of the purchase price.<br>*Question relevant when: not(selected(${q103_L2_full_list} , '6')) and not(selected(${q103_L2_full_list} , '99'))*<br>*Response constrained to: .>=2000 and .<=2019 or .=999*	
q703_L3	***q703_L3. Full blood count***<br>*Question relevant when: not(selected(${q103_L3_full_list} , '6')) and not(selected(${q103_L3_full_list} , '99'))*	
q703_L3a *(required)*	q703_L3a. When purchased, how many units of this item typically come in one pack, box, bottle, etc.? (e.g. 100 tablets, 1000 ml's, etc.) (Write 1 if purchased as a single item.)<br>*Smallest unit is one test.*<br>*Question relevant when: not(selected(${q103_L3_full_list} , '6')) and not(selected(${q103_L3_full_list} , '99'))*<br>*Response constrained to: .>=0*	
q703_L3b *(required)*	q703_L3b. What is the typical purchase price for that quanity of units (i.e. the quantity noted in the question above)?<br>*Question relevant when: not(selected(${q103_L3_full_list} , '6')) and not(selected(${q103_L3_full_list} , '99'))*<br>*Response constrained to: .>=0*	
q703_L3c *(required)*	q703_L3c. Please specify the currency for the purchase price.<br>*Question relevant when: not(selected(${q103_L3_full_list} , '6')) and not(selected(${q103_L3_full_list} , '99'))*	1 Tanzanian Shillings<br>2 US dollars

Field	Question	Answer
		3 Euros<br>4 Other<br>99 Don't know

Field	Question	Answer
q703_L3c.1	q703_L3c.1 If other currency, specify:<br>*Leave blank if not applicable*<br>*Question relevant when: not(selected(${q103_L3_full_list} , '6')) and not(selected(${q103_L3_full_list} , '99'))*	
q703_L3d *(required)*	q703_L3d. Please specify the year of the purchase price.<br>*Question relevant when: not(selected(${q103_L3_full_list} , '6')) and not(selected(${q103_L3_full_list} , '99'))*<br>*Response constrained to: .>=2000 and .<=2019 or .=999*	
q703_L4	***q703_L4. Hematocrit test***<br>*Question relevant when: not(selected(${q103_L4_full_list} , '6')) and not(selected(${q103_L4_full_list} , '99'))*	
q703_L4a *(required)*	q703_L4a. When purchased, how many units of this item typically come in one pack, box, bottle, etc.? (e.g. 100 tablets, 1000 ml's, etc.) (Write 1 if purchased as a single item.)<br>*Smallest unit is one test.*<br>*Question relevant when: not(selected(${q103_L4_full_list} , '6')) and not(selected(${q103_L4_full_list} , '99'))*<br>*Response constrained to: .>=0*	
q703_L4b *(required)*	q703_L4b. What is the typical purchase price for that quanity of units (i.e. the quantity noted in the question above)?<br>*Question relevant when: not(selected(${q103_L4_full_list} , '6')) and not(selected(${q103_L4_full_list} , '99'))*<br>*Response constrained to: .>=0*	
q703_L4c *(required)*	q703_L4c. Please specify the currency for the purchase price.<br>*Question relevant when: not(selected(${q103_L4_full_list} , '6')) and not(selected(${q103_L4_full_list} , '99'))*	1 Tanzanian Shillings<br>2 US dollars<br>3 Euros<br>4 Other<br>99 Don't know
q703_L4c.1	q703_L4c.1 If other currency, specify:<br>*Leave blank if not applicable*<br>*Question relevant when: not(selected(${q103_L4_full_list} , '6')) and not(selected(${q103_L4_full_list} , '99'))*	
q703_L4d *(required)*	q703_L4d. Please specify the year of the purchase price.<br>*Question relevant when: not(selected(${q103_L4_full_list} , '6')) and not(selected(${q103_L4_full_list} , '99'))*<br>*Response constrained to: .>=2000 and .<=2019 or .=999*	
q703_L5	***q703_L5. Hemoglobin test***<br>*Question relevant when: not(selected(${q103_L5_full_list} , '6')) and not(selected(${q103_L5_full_list} , '99'))*	
q703_L5a *(required)*	q703_L5a. When purchased, how many units of this item typically come in one pack, box, bottle, etc.? (e.g. 100 tablets, 1000 ml's, etc.) (Write 1 if purchased as a single item.)<br>*Smallest unit is one test.*<br>*Question relevant when: not(selected(${q103_L5_full_list} , '6')) and not(selected(${q103_L5_full_list} , '99'))*<br>*Response constrained to: .>=0*	
q703_L5b *(required)*	q703_L5b. What is the typical purchase price for that quanity of units (i.e. the quantity noted in the question above)?<br>*Question relevant when: not(selected(${q103_L5_full_list} , '6')) and not(selected(${q103_L5_full_list} , '99'))*<br>*Response constrained to: .>=0*	
q703_L5c *(required)*	q703_L5c. Please specify the currency for the purchase price.<br>*Question relevant when: not(selected(${q103_L5_full_list} , '6')) and not(selected(${q103_L5_full_list} , '99'))*	1 Tanzanian Shillings<br>2 US dollars<br>3 Euros<br>4 Other<br>99 Don't know
q703_L5c.1	q703_L5c.1 If other currency, specify:<br>*Leave blank if not applicable*<br>*Question relevant when: not(selected(${q103_L5_full_list} , '6')) and not(selected(${q103_L5_full_list} , '99'))*	
q703_L5d *(required)*	q703_L5d. Please specify the year of the purchase price.<br>*Question relevant when: not(selected(${q103_L5_full_list} , '6')) and not(selected(${q103_L5_full_list} , '99'))*<br>*Response constrained to: .>=2000 and .<=2019 or .=999*	
q703_L6	***q703_L6. Rhesus factor test***<br>*Question relevant when: not(selected(${q103_L6_full_list} , '6')) and not(selected(${q103_L6_full_list} , '99'))*	
q703_L6a *(required)*	q703_L6a. When purchased, how many units of this item typically come in one pack, box, bottle, etc.? (e.g. 100 tablets, 1000 ml's, etc.) (Write 1 if purchased as a single item.)<br>*Smallest unit is one test.*<br>*Question relevant when: not(selected(${q103_L6_full_list} , '6')) and not(selected(${q103_L6_full_list} , '99'))*<br>*Response constrained to: .>=0*	
q703_L6b *(required)*	q703_L6b. What is the typical purchase price for that quanity of units (i.e. the quantity noted in the question above)?<br>*Question relevant when: not(selected(${q103_L6_full_list} , '6')) and not(selected(${q103_L6_full_list} , '99'))*<br>*Response constrained to: .>=0*	
q703_L6c *(required)*	q703_L6c. Please specify the currency for the purchase price.<br>*Question relevant when: not(selected(${q103_L6_full_list} , '6')) and not(selected(${q103_L6_full_list} , '99'))*	1 Tanzanian Shillings<br>2 US dollars

Field	Question	Answer
		3 Euros<br>4 Other<br>99 Don't know

q703_L6c.1	q703_L6c.1 If other currency, specify:<br>*Leave blank if not applicable*<br>*Question relevant when: not(selected(${q103_L6_full_list} , '6')) and not(selected(${q103_L6_full_list} , '99'))*	
q703_L6d *(required)*	q703_L6d. Please specify the year of the purchase price.<br>*Question relevant when: not(selected(${q103_L6_full_list} , '6')) and not(selected(${q103_L6_full_list} , '99'))*<br>*Response constrained to: .>=2000 and .<=2019 or .=999*	
q703_L7	***q703_L7. Test of bleeding time***<br>*Question relevant when: not(selected(${q103_L7_full_list} , '6')) and not(selected(${q103_L7_full_list} , '99'))*	
q703_L7a *(required)*	q703_L7a. When purchased, how many units of this item typically come in one pack, box, bottle, etc.? (e.g. 100 tablets, 1000 ml's, etc.) (Write 1 if purchased as a single item.)<br>*Smallest unit is one test.*<br>*Question relevant when: not(selected(${q103_L7_full_list} , '6')) and not(selected(${q103_L7_full_list} , '99'))*<br>*Response constrained to: .>=0*	
q703_L7b *(required)*	q703_L7b. What is the typical purchase price for that quanity of units (i.e. the quantity noted in the question above)?<br>*Question relevant when: not(selected(${q103_L7_full_list} , '6')) and not(selected(${q103_L7_full_list} , '99'))*<br>*Response constrained to: .>=0*	
q703_L7c *(required)*	q703_L7c. Please specify the currency for the purchase price.<br>*Question relevant when: not(selected(${q103_L7_full_list} , '6')) and not(selected(${q103_L7_full_list} , '99'))*	1 Tanzanian Shillings<br>2 US dollars<br>3 Euros<br>4 Other<br>99 Don't know
q703_L7c.1	q703_L7c.1 If other currency, specify:<br>*Leave blank if not applicable*<br>*Question relevant when: not(selected(${q103_L7_full_list} , '6')) and not(selected(${q103_L7_full_list} , '99'))*	
q703_L7d *(required)*	q703_L7d. Please specify the year of the purchase price.<br>*Question relevant when: not(selected(${q103_L7_full_list} , '6')) and not(selected(${q103_L7_full_list} , '99'))*<br>*Response constrained to: .>=2000 and .<=2019 or .=999*	
q703_L8	***q703_L8. White blood cell count***<br>*Question relevant when: not(selected(${q103_L8_full_list} , '6')) and not(selected(${q103_L8_full_list} , '99'))*	
q703_L8a *(required)*	q703_L8a. When purchased, how many units of this item typically come in one pack, box, bottle, etc.? (e.g. 100 tablets, 1000 ml's, etc.) (Write 1 if purchased as a single item.)<br>*Smallest unit is one test.*<br>*Question relevant when: not(selected(${q103_L8_full_list} , '6')) and not(selected(${q103_L8_full_list} , '99'))*<br>*Response constrained to: .>=0*	
q703_L8b *(required)*	q703_L8b. What is the typical purchase price for that quanity of units (i.e. the quantity noted in the question above)?<br>*Question relevant when: not(selected(${q103_L8_full_list} , '6')) and not(selected(${q103_L8_full_list} , '99'))*<br>*Response constrained to: .>=0*	
q703_L8c *(required)*	q703_L8c. Please specify the currency for the purchase price.<br>*Question relevant when: not(selected(${q103_L8_full_list} , '6')) and not(selected(${q103_L8_full_list} , '99'))*	1 Tanzanian Shillings<br>2 US dollars<br>3 Euros<br>4 Other<br>99 Don't know
q703_L8c.1	q703_L8c.1 If other currency, specify:<br>*Leave blank if not applicable*<br>*Question relevant when: not(selected(${q103_L8_full_list} , '6')) and not(selected(${q103_L8_full_list} , '99'))*	
q703_L8d *(required)*	q703_L8d. Please specify the year of the purchase price.<br>*Question relevant when: not(selected(${q103_L8_full_list} , '6')) and not(selected(${q103_L8_full_list} , '99'))*<br>*Response constrained to: .>=2000 and .<=2019 or .=999*	
C. Laboratory tests, supplies - Cost (2)<br>*Group relevant when: selected(${section_seven_skip_lab} , '1')*		
note_703_HIV_STI	***HIV and STI tests***	
q703_L9	***q703_L9. HIV test***<br>*Question relevant when: not(selected(${q103_L9_full_list} , '6')) and not(selected(${q103_L9_full_list} , '99'))*	
q703_L9a *(required)*	q703_L9a. When purchased, how many units of this item typically come in one pack, box, bottle, etc.? (e.g. 100 tablets, 1000 ml's, etc.) (Write 1 if purchased as a single item.)<br>*Smallest unit is one test.*<br>*Question relevant when: not(selected(${q103_L9_full_list} , '6')) and not(selected(${q103_L9_full_list} , '99'))*<br>*Response constrained to: .>=0*	
q703_L9b *(required)*	q703_L9b. What is the typical purchase price for that quanity of units (i.e. the quantity noted in the question above)?<br>*Question relevant when: not(selected(${q103_L9_full_list} , '6')) and not(selected(${q103_L9_full_list} , '99'))*<br>[illegible]	

Response constrained to: .>=0

Field	Question	Answer
q703_L9c *(required)*	q703_L9c. Please specify the currency for the purchase price.<br>*Question relevant when: not(selected(${q103_L9_full_list} , '6')) and not(selected(${q103_L9_full_list} , '99'))*	1 Tanzanian Shillings<br>2 US dollars<br>3 Euros<br>4 Other<br>99 Don't know
q703_L9c.1	q703_L9c.1 If other currency, specify:<br>*Leave blank if not applicable*<br>*Question relevant when: not(selected(${q103_L9_full_list} , '6')) and not(selected(${q103_L9_full_list} , '99'))*	
q703_L9d *(required)*	q703_L9d. Please specify the year of the purchase price.<br>*Question relevant when: not(selected(${q103_L9_full_list} , '6')) and not(selected(${q103_L9_full_list} , '99'))*<br>*Response constrained to: .>=2000 and .<=2019 or .=999*	
q703_L10	***q703_L10. Rapid test for Hepatitis C***<br>*Question relevant when: not(selected(${q103_L10_full_list} , '6')) and not(selected(${q103_L10_full_list} , '99'))*	
q703_L10a *(required)*	q703_L10a. When purchased, how many units of this item typically come in one pack, box, bottle, etc.? (e.g. 100 tablets, 1000 ml's, etc.) (Write 1 if purchased as a single item.)<br>*Smallest unit is one test.*<br>*Question relevant when: not(selected(${q103_L10_full_list} , '6')) and not(selected(${q103_L10_full_list} , '99'))*<br>*Response constrained to: .>=0*	
q703_L10b *(required)*	q703_L10b. What is the typical purchase price for that quanity of units (i.e. the quantity noted in the question above)?<br>*Question relevant when: not(selected(${q103_L10_full_list} , '6')) and not(selected(${q103_L10_full_list} , '99'))*<br>*Response constrained to: .>=0*	
q703_L10c *(required)*	q703_L10c. Please specify the currency for the purchase price.<br>*Question relevant when: not(selected(${q103_L10_full_list} , '6')) and not(selected(${q103_L10_full_list} , '99'))*	1 Tanzanian Shillings<br>2 US dollars<br>3 Euros<br>4 Other<br>99 Don't know
q703_L10c.1	q703_L10c.1 If other currency, specify:<br>*Leave blank if not applicable*<br>*Question relevant when: not(selected(${q103_L10_full_list} , '6')) and not(selected(${q103_L10_full_list} , '99'))*	
q703_L10d *(required)*	q703_L10d. Please specify the year of the purchase price.<br>*Question relevant when: not(selected(${q103_L10_full_list} , '6')) and not(selected(${q103_L10_full_list} , '99'))*<br>*Response constrained to: .>=2000 and .<=2019 or .=999*	
q703_L11	***q703_L11. Syphilis test***<br>*Question relevant when: not(selected(${q103_L11_full_list} , '6')) and not(selected(${q103_L11_full_list} , '99'))*	
q703_L11a *(required)*	q703_L11a. When purchased, how many units of this item typically come in one pack, box, bottle, etc.? (e.g. 100 tablets, 1000 ml's, etc.) (Write 1 if purchased as a single item.)<br>*Smallest unit is one test.*<br>*Question relevant when: not(selected(${q103_L11_full_list} , '6')) and not(selected(${q103_L11_full_list} , '99'))*<br>*Response constrained to: .>=0*	
q703_L11b *(required)*	q703_L11b. What is the typical purchase price for that quanity of units (i.e. the quantity noted in the question above)?<br>*Question relevant when: not(selected(${q103_L11_full_list} , '6')) and not(selected(${q103_L11_full_list} , '99'))*<br>*Response constrained to: .>=0*	
q703_L11c *(required)*	q703_L11c. Please specify the currency for the purchase price.<br>*Question relevant when: not(selected(${q103_L11_full_list} , '6')) and not(selected(${q103_L11_full_list} , '99'))*	1 Tanzanian Shillings<br>2 US dollars<br>3 Euros<br>4 Other<br>99 Don't know
q703_L11c.1	q703_L11c.1 If other currency, specify:<br>*Leave blank if not applicable*<br>*Question relevant when: not(selected(${q103_L11_full_list} , '6')) and not(selected(${q103_L11_full_list} , '99'))*	
q703_L11d *(required)*	q703_L11d. Please specify the year of the purchase price.<br>*Question relevant when: not(selected(${q103_L11_full_list} , '6')) and not(selected(${q103_L11_full_list} , '99'))*<br>*Response constrained to: .>=2000 and .<=2019 or .=999*	
C. Laboratory tests, supplies - Cost (3)<br>*Group relevant when: selected(${section_seven_skip_lab} , '1')*		
note_703_malaria	***Malaria***	
q703_L12	***q703_L12. Rapid test for Malaria***<br>*Question relevant when: not(selected(${q103_L12_full_list} , '6')) and not(selected(${q103_L12_full_list} , '99'))*	
q703_L12a *(required)*	q703_L12a. When purchased, how many units of this item typically come in one pack, box, bottle, etc.? (e.g. 100 tablets,	

Field	Question	Answer
	1000 ml's, etc.) (Write 1 if purchased as a single item.)<br>*Smallest unit is one test.*	
	Question relevant when: not(selected(${q103_L12_full_list} , '6')) and not(selected(${q103_L12_full_list} , '99'))<br>*Response constrained to: .>=0*	

q703_L12b *(required)*	q703_L12b. What is the typical purchase price for that quanity of units (i.e. the quantity noted in the question above)?<br>*Question relevant when: not(selected(${q103_L12_full_list} , '6')) and not(selected(${q103_L12_full_list} , '99'))*<br>*Response constrained to: .>=0*	
q703_L12c *(required)*	q703_L12c. Please specify the currency for the purchase price.<br>*Question relevant when: not(selected(${q103_L12_full_list} , '6')) and not(selected(${q103_L12_full_list} , '99'))*	1 Tanzanian Shillings<br>2 US dollars<br>3 Euros<br>4 Other<br>99 Don't know
q703_L12c.1	q703_L12c.1 If other currency, specify:<br>*Leave blank if not applicable*<br>*Question relevant when: not(selected(${q103_L12_full_list} , '6')) and not(selected(${q103_L12_full_list} , '99'))*	
q703_L12d *(required)*	q703_L12d. Please specify the year of the purchase price.<br>*Question relevant when: not(selected(${q103_L12_full_list} , '6')) and not(selected(${q103_L12_full_list} , '99'))*<br>*Response constrained to: .>=2000 and .<=2019 or .=999*	
q703_L13	***q703_L13. Thick blood smear (for malaria)***<br>*Question relevant when: not(selected(${q103_L13_full_list} , '6')) and not(selected(${q103_L13_full_list} , '99'))*	
q703_L13a *(required)*	q703_L13a. When purchased, how many units of this item typically come in one pack, box, bottle, etc.? (e.g. 100 tablets, 1000 ml's, etc.) (Write 1 if purchased as a single item.)<br>*Smallest unit is one test.*<br>*Question relevant when: not(selected(${q103_L13_full_list} , '6')) and not(selected(${q103_L13_full_list} , '99'))*<br>*Response constrained to: .>=0*	
q703_L13b *(required)*	q703_L13b. What is the typical purchase price for that quanity of units (i.e. the quantity noted in the question above)?<br>*Question relevant when: not(selected(${q103_L13_full_list} , '6')) and not(selected(${q103_L13_full_list} , '99'))*<br>*Response constrained to: .>=0*	
q703_L13c *(required)*	q703_L13c. Please specify the currency for the purchase price.<br>*Question relevant when: not(selected(${q103_L13_full_list} , '6')) and not(selected(${q103_L13_full_list} , '99'))*	1 Tanzanian Shillings<br>2 US dollars<br>3 Euros<br>4 Other<br>99 Don't know
q703_L13c.1	q703_L13c.1 If other currency, specify:<br>*Leave blank if not applicable*<br>*Question relevant when: not(selected(${q103_L13_full_list} , '6')) and not(selected(${q103_L13_full_list} , '99'))*	
q703_L13d *(required)*	q703_L13d. Please specify the year of the purchase price.<br>*Question relevant when: not(selected(${q103_L13_full_list} , '6')) and not(selected(${q103_L13_full_list} , '99'))*<br>*Response constrained to: .>=2000 and .<=2019 or .=999*	
C. Laboratory tests, supplies - Cost (4)<br>*Group relevant when: selected(${section_seven_skip_lab} , '1')*		
note_703_Pregnancy	***Pregnancy***	
q703_L14	***q703_L14. Pregnancy test - blood***<br>*Question relevant when: not(selected(${q103_L14_full_list} , '6')) and not(selected(${q103_L14_full_list} , '99'))*	
q703_L14a *(required)*	q703_L14a. When purchased, how many units of this item typically come in one pack, box, bottle, etc.? (e.g. 100 tablets, 1000 ml's, etc.) (Write 1 if purchased as a single item.)<br>*Smallest unit is one test.*<br>*Question relevant when: not(selected(${q103_L14_full_list} , '6')) and not(selected(${q103_L14_full_list} , '99'))*<br>*Response constrained to: .>=0*	
q703_L14b *(required)*	q703_L14b. What is the typical purchase price for that quanity of units (i.e. the quantity noted in the question above)?<br>*Question relevant when: not(selected(${q103_L14_full_list} , '6')) and not(selected(${q103_L14_full_list} , '99'))*<br>*Response constrained to: .>=0*	
q703_L14c *(required)*	q703_L14c. Please specify the currency for the purchase price.<br>*Question relevant when: not(selected(${q103_L14_full_list} , '6')) and not(selected(${q103_L14_full_list} , '99'))*	1 Tanzanian Shillings<br>2 US dollars<br>3 Euros<br>4 Other<br>99 Don't know
q703_L14c.1	q703_L14c.1 If other currency, specify:<br>*Leave blank if not applicable*<br>*Question relevant when: not(selected(${q103_L14_full_list} , '6')) and not(selected(${q103_L14_full_list} , '99'))*	
q703_L14d *(required)*	q703_L14d. Please specify the year of the purchase price.<br>*Question relevant when: not(selected(${q103_L14_full_list} , '6')) and not(selected(${q103_L14_full_list} , '99'))*<br>*Response constrained to: .>=2000 and .<=2019 or .=999*	

Field	Question	Answer
q703_L15	***q703_L15. Pregnancy test - urine***<br>*Question relevant when: not(selected(${q103_L15_full_list} , '6')) and not(selected(${q103_L15_full_list} , '99'))*	
q703_L15a *(required)*	q703_L15a. When purchased, how many units of this item typically come in one pack, box, bottle, etc.? (e.g. 100 tablets, 1000 ml's, etc.) (Write 1 if purchased as a single item.)<br>*Smallest unit is one test.*<br>*Question relevant when: not(selected(${q103_L15_full_list} , '6')) and not(selected(${q103_L15_full_list} , '99'))*<br>*Response constrained to: .>=0*	
q703_L15b *(required)*	q703_L15b. What is the typical purchase price for that quanity of units (i.e. the quantity noted in the question above)?<br>*Question relevant when: not(selected(${q103_L15_full_list} , '6')) and not(selected(${q103_L15_full_list} , '99'))*<br>*Response constrained to: .>=0*	
q703_L15c *(required)*	q703_L15c. Please specify the currency for the purchase price.<br>*Question relevant when: not(selected(${q103_L15_full_list} , '6')) and not(selected(${q103_L15_full_list} , '99'))*	1 Tanzanian Shillings<br>2 US dollars<br>3 Euros<br>4 Other<br>99 Don't know
q703_L15c.1	q703_L15c.1 If other currency, specify:<br>*Leave blank if not applicable*<br>*Question relevant when: not(selected(${q103_L15_full_list} , '6')) and not(selected(${q103_L15_full_list} , '99'))*	
q703_L15d *(required)*	q703_L15d. Please specify the year of the purchase price.<br>*Question relevant when: not(selected(${q103_L15_full_list} , '6')) and not(selected(${q103_L15_full_list} , '99'))*<br>*Response constrained to: .>=2000 and .<=2019 or .=999*	
q703_L16	***q703_L16. Ultrasound***<br>*Question relevant when: not(selected(${q103_L16_full_list} , '6')) and not(selected(${q103_L16_full_list} , '99'))*	
q703_L16a *(required)*	q703_L16a. When purchased, how many units of this item typically come in one pack, box, bottle, etc.? (e.g. 100 tablets, 1000 ml's, etc.) (Write 1 if purchased as a single item.)<br>*Smallest unit is one scan.*<br>*Question relevant when: not(selected(${q103_L16_full_list} , '6')) and not(selected(${q103_L16_full_list} , '99'))*<br>*Response constrained to: .>=0*	
q703_L16b *(required)*	q703_L16b. What is the typical purchase price for that quanity of units (i.e. the quantity noted in the question above)?<br>*Question relevant when: not(selected(${q103_L16_full_list} , '6')) and not(selected(${q103_L16_full_list} , '99'))*<br>*Response constrained to: .>=0*	
q703_L16c *(required)*	q703_L16c. Please specify the currency for the purchase price.<br>*Question relevant when: not(selected(${q103_L16_full_list} , '6')) and not(selected(${q103_L16_full_list} , '99'))*	1 Tanzanian Shillings<br>2 US dollars<br>3 Euros<br>4 Other<br>99 Don't know
q703_L16c.1	q703_L16c.1 If other currency, specify:<br>*Leave blank if not applicable*<br>*Question relevant when: not(selected(${q103_L16_full_list} , '6')) and not(selected(${q103_L16_full_list} , '99'))*	
q703_L16d *(required)*	q703_L16d. Please specify the year of the purchase price.<br>*Question relevant when: not(selected(${q103_L16_full_list} , '6')) and not(selected(${q103_L16_full_list} , '99'))*<br>*Response constrained to: .>=2000 and .<=2019 or .=999*	
C. Laboratory tests, supplies - Cost(5)<br>*Group relevant when: selected(${section_seven_skip_lab} , '1')*		
note_703_Urine	***Urine***	
q703_L17	***q703_L17. Urine dipstick***<br>*Question relevant when: not(selected(${q103_L17_full_list} , '6')) and not(selected(${q103_L17_full_list} , '99'))*	
q703_L17a *(required)*	q703_L17a. When purchased, how many units of this item typically come in one pack, box, bottle, etc.? (e.g. 100 tablets, 1000 ml's, etc.) (Write 1 if purchased as a single item.)<br>*Smallest unit is one test.*<br>*Question relevant when: not(selected(${q103_L17_full_list} , '6')) and not(selected(${q103_L17_full_list} , '99'))*<br>*Response constrained to: .>=0*	
q703_L17b *(required)*	q703_L17b. What is the typical purchase price for that quanity of units (i.e. the quantity noted in the question above)?<br>*Question relevant when: not(selected(${q103_L17_full_list} , '6')) and not(selected(${q103_L17_full_list} , '99'))*<br>*Response constrained to: .>=0*	
q703_L17c *(required)*	q703_L17c. Please specify the currency for the purchase price.<br>*Question relevant when: not(selected(${q103_L17_full_list} , '6')) and not(selected(${q103_L17_full_list} , '99'))*	1 Tanzanian Shillings<br>2 US dollars<br>3 Euros<br>4 Other<br>99 Don't know
q703_L17c.1	q703_L17c.1 If other currency, specify:	

Field	Question	Answer
	Leave blank if not applicable<br>*Question relevant when: not(selected(${q103_L17_full_list} , '6')) and not(selected(${q103_L17_full_list} , '99'))*	
q703_L17d *(required)*	q703_L17d. Please specify the year of the purchase price.<br>*Question relevant when: not(selected(${q103_L17_full_list} , '6')) and not(selected(${q103_L17_full_list} , '99'))*<br>*Response constrained to: .>=2000 and .<=2019 or .=999*	

C. Laboratory tests, supplies - Cost (6)<br>*Group relevant when: selected(${section_seven_skip_lab} , '1')*		
note_703_Other	***Other lab tests - suggestions?***	
q703_L18	***q703_L18. Lab test other 1: "[q103_L18_full_list_other]"***<br>*Question relevant when: not(selected(${q103_L18_full_list} , '6')) and not(selected(${q103_L18_full_list} , '99')) and string-length(${q103_L18_full_list_other}) > 0*	
q703_L18a *(required)*	q703_L18a. When purchased, how many units of this item typically come in one pack, box, bottle, etc.? (e.g. 100 tablets, 1000 ml's, etc.) (Write 1 if purchased as a single item.)<br>*Question relevant when: not(selected(${q103_L18_full_list} , '6')) and not(selected(${q103_L18_full_list} , '99')) and string-length(${q103_L18_full_list_other}) > 0*<br>*Response constrained to: .>=0*	
q703_L18b *(required)*	q703_L18b. What is the typical purchase price for that quanity of units (i.e. the quantity noted in the question above)?<br>*Question relevant when: not(selected(${q103_L18_full_list} , '6')) and not(selected(${q103_L18_full_list} , '99')) and string-length(${q103_L18_full_list_other}) > 0*<br>*Response constrained to: .>=0*	
q703_L18c *(required)*	q703_L18c. Please specify the currency for the purchase price.<br>*Question relevant when: not(selected(${q103_L18_full_list} , '6')) and not(selected(${q103_L18_full_list} , '99')) and string-length(${q103_L18_full_list_other}) > 0*	1 Tanzanian Shillings<br>2 US dollars<br>3 Euros<br>4 Other<br>99 Don't know
q703_L18c.1	q703_L18c.1 If other currency, specify:<br>*Leave blank if not applicable*<br>*Question relevant when: not(selected(${q103_L18_full_list} , '6')) and not(selected(${q103_L18_full_list} , '99')) and string-length(${q103_L18_full_list_other}) > 0*	
q703_L18d *(required)*	q703_L18d. Please specify the year of the purchase price.<br>*Question relevant when: not(selected(${q103_L18_full_list} , '6')) and not(selected(${q103_L18_full_list} , '99')) and string-length(${q103_L18_full_list_other}) > 0*<br>*Response constrained to: .>=2000 and .<=2019 or .=999*	
q703_L19	***q703_L19. Lab test other 2: "[q103_L19_full_list_other]"***<br>*Question relevant when: not(selected(${q103_L19_full_list} , '6')) and not(selected(${q103_L19_full_list} , '99')) and string-length(${q103_L19_full_list_other}) > 0*	
q703_L19a *(required)*	q703_L19a. When purchased, how many units of this item typically come in one pack, box, bottle, etc.? (e.g. 100 tablets, 1000 ml's, etc.) (Write 1 if purchased as a single item.)<br>*Question relevant when: not(selected(${q103_L19_full_list} , '6')) and not(selected(${q103_L19_full_list} , '99')) and string-length(${q103_L19_full_list_other}) > 0*<br>*Response constrained to: .>=0*	
q703_L19b *(required)*	q703_L19b. What is the typical purchase price for that quanity of units (i.e. the quantity noted in the question above)?<br>*Question relevant when: not(selected(${q103_L19_full_list} , '6')) and not(selected(${q103_L19_full_list} , '99')) and string-length(${q103_L19_full_list_other}) > 0*<br>*Response constrained to: .>=0*	
q703_L19c *(required)*	q703_L19c. Please specify the currency for the purchase price.<br>*Question relevant when: not(selected(${q103_L19_full_list} , '6')) and not(selected(${q103_L19_full_list} , '99')) and string-length(${q103_L19_full_list_other}) > 0*	1 Tanzanian Shillings<br>2 US dollars<br>3 Euros<br>4 Other<br>99 Don't know
q703_L19c.1	q703_L19c.1 If other currency, specify:<br>*Leave blank if not applicable*<br>*Question relevant when: not(selected(${q103_L19_full_list} , '6')) and not(selected(${q103_L19_full_list} , '99')) and string-length(${q103_L19_full_list_other}) > 0*	
q703_L19d *(required)*	q703_L19d. Please specify the year of the purchase price.<br>*Question relevant when: not(selected(${q103_L19_full_list} , '6')) and not(selected(${q103_L19_full_list} , '99')) and string-length(${q103_L19_full_list_other}) > 0*<br>*Response constrained to: .>=2000 and .<=2019 or .=999*	
q703_L20	***q703_L20. Lab test other 3: "[q103_L20_full_list_other]"***<br>*Question relevant when: not(selected(${q103_L20_full_list} , '6')) and not(selected(${q103_L20_full_list} , '99')) and string-length(${q103_L20_full_list_other}) > 0*	
q703_L20a *(required)*	q703_L20a. When purchased, how many units of this item typically come in one pack, box, bottle, etc.? (e.g. 100 tablets, 1000 ml's, etc.) (Write 1 if purchased as a single item.)	

Field	Question	Answer
	Question relevant when: not(selected(${q103_L20_full_list} , '6')) and not(selected(${q103_L20_full_list} , '99')) and string-length(${q103_L20_full_list_other}) > 0<br>*Response constrained to: .>=0*	
q703_L20b *(required)*	q703_L20b. What is the typical purchase price for that quanity of units (i.e. the quantity noted in the question above)?<br>*Question relevant when: not(selected(${q103_L20_full_list} , '6')) and not(selected(${q103_L20_full_list} , '99')) and string-length(${q103_L20_full_list_other}) > 0*<br>*Response constrained to: .>=0*	
q703_L20c *(required)*	q703_L20c. Please specify the currency for the purchase price.<br>*Question relevant when: not(selected(${q103_L20_full_list} , '6')) and not(selected(${q103_L20_full_list} , '99')) and string-length(${q103_L20_full_list_other}) > 0*	1 Tanzanian Shillings<br>2 US dollars<br>3 Euros<br>4 Other<br>99 Don't know
q703_L20c.1	q703_L20c.1 If other currency, specify:<br>*Leave blank if not applicable*<br>*Question relevant when: not(selected(${q103_L20_full_list} , '6')) and not(selected(${q103_L20_full_list} , '99')) and string-length(${q103_L20_full_list_other}) > 0*	
q703_L20d *(required)*	q703_L20d. Please specify the year of the purchase price.<br>*Question relevant when: not(selected(${q103_L20_full_list} , '6')) and not(selected(${q103_L20_full_list} , '99')) and string-length(${q103_L20_full_list_other}) > 0*<br>*Response constrained to: .>=2000 and .<=2019 or .=999*	
q703_L21	***q703_L21. Lab test other 4: "[q103_L21_full_list_other]"***<br>*Question relevant when: not(selected(${q103_L21_full_list} , '6')) and not(selected(${q103_L21_full_list} , '99')) and string-length(${q103_L21_full_list_other}) > 0*	
q703_L21a *(required)*	q703_L21a. When purchased, how many units of this item typically come in one pack, box, bottle, etc.? (e.g. 100 tablets, 1000 ml's, etc.) (Write 1 if purchased as a single item.)<br>*Question relevant when: not(selected(${q103_L21_full_list} , '6')) and not(selected(${q103_L21_full_list} , '99')) and string-length(${q103_L21_full_list_other}) > 0*<br>*Response constrained to: .>=0*	
q703_L21b *(required)*	q703_L21b. What is the typical purchase price for that quanity of units (i.e. the quantity noted in the question above)?<br>*Question relevant when: not(selected(${q103_L21_full_list} , '6')) and not(selected(${q103_L21_full_list} , '99')) and string-length(${q103_L21_full_list_other}) > 0*<br>*Response constrained to: .>=0*	
q703_L21c *(required)*	q703_L21c. Please specify the currency for the purchase price.<br>*Question relevant when: not(selected(${q103_L21_full_list} , '6')) and not(selected(${q103_L21_full_list} , '99')) and string-length(${q103_L21_full_list_other}) > 0*	1 Tanzanian Shillings<br>2 US dollars<br>3 Euros<br>4 Other<br>99 Don't know
q703_L21c.1	q703_L21c.1 If other currency, specify:<br>*Leave blank if not applicable*<br>*Question relevant when: not(selected(${q103_L21_full_list} , '6')) and not(selected(${q103_L21_full_list} , '99')) and string-length(${q103_L21_full_list_other}) > 0*	
q703_L21d *(required)*	q703_L21d. Please specify the year of the purchase price.<br>*Question relevant when: not(selected(${q103_L21_full_list} , '6')) and not(selected(${q103_L21_full_list} , '99')) and string-length(${q103_L21_full_list_other}) > 0*<br>*Response constrained to: .>=2000 and .<=2019 or .=999*	
q703_L22	***q703_L22. Lab test other 5: "[q103_L22_full_list_other]"***<br>*Question relevant when: not(selected(${q103_L22_full_list} , '6')) and not(selected(${q103_L22_full_list} , '99')) and string-length(${q103_L22_full_list_other}) > 0*	
q703_L22a *(required)*	q703_L22a. When purchased, how many units of this item typically come in one pack, box, bottle, etc.? (e.g. 100 tablets, 1000 ml's, etc.) (Write 1 if purchased as a single item.)<br>*Question relevant when: not(selected(${q103_L22_full_list} , '6')) and not(selected(${q103_L22_full_list} , '99')) and string-length(${q103_L22_full_list_other}) > 0*<br>*Response constrained to: .>=0*	
q703_L22b *(required)*	q703_L22b. What is the typical purchase price for that quanity of units (i.e. the quantity noted in the question above)?<br>*Question relevant when: not(selected(${q103_L22_full_list} , '6')) and not(selected(${q103_L22_full_list} , '99')) and string-length(${q103_L22_full_list_other}) > 0*<br>*Response constrained to: .>=0*	
q703_L22c *(required)*	q703_L22c. Please specify the currency for the purchase price.<br>*Question relevant when: not(selected(${q103_L22_full_list} , '6')) and not(selected(${q103_L22_full_list} , '99')) and string-length(${q103_L22_full_list_other}) > 0*	1 Tanzanian Shillings<br>2 US dollars<br>3 Euros<br>4 Other

Field	Question	Answer
		99 Don't know
q703_L22c.1	q703_L22c.1 If other currency, specify:<br>*Leave blank if not applicable*<br>*Question relevant when: not(selected(${q103_L22_full_list} , '6')) and not(selected(${q103_L22_full_list} , '99')) and string-length(${q103_L22_full_list_other}) > 0*	

q703_L22d *(required)*	q703_L22d. Please specify the year of the purchase price.<br>*Question relevant when: not(selected(${q103_L22_full_list} , '6')) and not(selected(${q103_L22_full_list} , '99')) and string-length(${q103_L22_full_list_other}) > 0*<br>*Response constrained to: .>=2000 and .<=2019 or .=999*	
GPS_QB2_lab *(required)*	GPS location capture<br>*Press the button to capture the GPS location at this point in the survey.*	
q705_time_end_qb_lab *(required)*	ENTER THE END TIME OF THE INTERVIEW<br>*NB: The default is the current time.*	
thank_you	**END OF INTERVIEW QUESTIONS.**<br><br>**THANK THE PARTICIPANT FOR THEIR TIME.**<br><br>**THEN COMPLETE THE FOLLOWING TWO ITEMS.**	
q706_interviewer_comments_lab	INTERVIEW COMMENTS - ENTER ANY RELEVANT NOTES AT THE END OF THE INTERVIEW. PLEASE ALSO EXPLAIN HOW COSTS WERE COLLECTED, I.E. VIA INTERVIEW, SITE REPRESENTATIVE FILLING OUT FORM, OR EXTRACTION FROM RECORDS.	
End_note	**End of Questionnaire B part 3**<br><br>ON THE NEXT PAGE YOU'LL FIND THIS FACILITY'S ID NUMBER. TAKE NOTE OF THE NUMBER, AND THEN, ON THE FOLLOWING PAGE, NAME THIS FORM BY ADDING THE FACILITY ID BEFORE THE FORM NAME.<br>FOR EXAMPLE, "20 Tanzania PAC cost study – Quest. B part 3 labs".<br><br>IF THIS INTERVIEW IS COMPLETE - I.E. THE RESPONDENT HAS ANSWERED ALL OF THE QUESTIONS THAT THEY CAN ON THIS FORM, THEN LEAVE THE DEFAULT BOX CHECKED. IF THE FORM IS NOT FINAL FOR THIS FACILITY, UNTICK THE BOX.<br><br>THEN SAVE AND EXIT.	
facility_id_lab2	The ID for this facility is '[facility_id_lab]".	
