# Supplementary material for: The health system costs of post abortion care in Tanzania
Source: BMC Health Serv Res. 2021 Jul 22;21:720. doi: 10.1186/s12913-021-06688-7 (PMC8296742; doi:10.1186/s12913-021-06688-7)
Supplement: Supplementary file 1 — Additional file 1. [file 12913_2021_6688_MOESM1_ESM.zip › QB-part 4 medications 2019.01.28 FINALR3.pdf]

## Tanzania PAC cost study – Quest. B part 4 medications

| Field | Question | Answer |
|---|---|---|
| about_survey | **Tanzania PAC cost study – Quest. B part 4 medications**<br><br>INTERVIEWER INSTRUCTIONS:<br>• DO NOT READ TEXT IN ALL CAPS OR HINTS IN *ITALICS* ALOUD TO THE PARTICIPANT.<br>• BEFORE STARTING THE INTERVIEW, CHECK WHICH PARTS OR SECTIONS OF QUESTIONNAIRE B HAVE NOT BEEN COMPLETED.<br>• MAKE SURE THE PERSON YOU ARE ABOUT TO INTERVIEW HAS PROVIDED CONSENT. | |
| introduction | QUESTIONNAIRE INTRODUCTION: We're now ready to complete part 4 of the interview. In this part of the interview we'll focus on medications used at your facility.<br><br>As with the other interview parts, I'll first present a list of items and ask which items are used for the five types of postabortion complications. Those are: uncomplicated incomplete abortion, sepsis, shock, cervical and vaginal lacerations, and vaginal and uterine perforations.<br><br>Then, if you tell me that an item is used at your facility, I'll separately ask how it is used (e.g. what proportion of women get it, how much they get, etc.).<br><br>At the end of interview, for any item that is used, I'll ask about prices for buying the items. If you don't have that information, I can obtain it from someone else at your facility after the interview.<br><br>Before we begin, I'm going to capture a some information about where we are and document that I'm doing the interview with you. Remember that your name won't be used with the results later. | |
| COVER PAGE | | |
| q1_interviewer_name_med *(required)* | q1. NAME OF INTERVIEWER | |
| q2_facility_name_med *(required)* | q2. SELECT: NAME OF FACILITY | facilityid facility_name |
| q3_GPS_QB_med *(required)* | q3. CAPTURE THE GPS LOCATION<br>*Press the button to capture the GPS location at this point in the survey.* | |
| RESPONDENTS (1-5) | | |
| cover_page_note_1_med | INTERVIEWER INSTRUCTIONS: INDICATE NAMES, JOB TITLES AND CONTACT INFORMATION FOR UP TO 5 RESPONDENTS OF QUESTIONNAIRE A.<br>*NB: Information for only the first respondent is required in order to proceed to the next page. Ask for the information as a question if not already known prior to the interview.* | |
| group_respondent1_med | RESPONDENT 1 | |
| q4a_name_med *(required)* | q4a. Name of 1st respondent<br>*First and last name* | |
| q4b_date_med *(required)* | q4b. Date of interview with 1st respondent<br>*Default is today's date.* | |
| q4c_title_med *(required)* | q4c. Designation/title of 1st respondent<br>*Job title and designation (e.g. Medical officer in charge)* | |
| q4d_phone_med | q4d. Phone number of 1st respondent<br>*NB: Not required, but you might need this to follow up with questions.* | |
| group_respondent2_med | RESPONDENT 2 | |
| q5a_name_med | q5a. Name of 2nd respondent<br>*First and last name* | |
| q5b_date_med | q5b. Date of interview with 2nd respondent<br>*Default is today's date.* | |
| q5c_title_med | q5c. Designation/title of 2nd respondent<br>*Job title and designation (e.g. Medical officer in charge)* | |
| q5d_phone_med | q5d. Phone number of 2nd respondent<br>*NB: Not required, but you might need this to follow up with questions.* | |
| group_respondent3_med | RESPONDENT 3 | |
| q6a_name_med | q6a. Name of 3rd respondent<br>*First and last name* | |
| q6b_date_med | q6b. Date of interview with 3rd respondent<br>*Default is today's date.* | |
| q6c_title_med | q6c. Designation/title of 3rd respondent<br>*Job title and designation (e.g. Medical officer in charge)* | |
| q6d_phone_med | q6d. Phone number of 3rd respondent<br>*NB: Not required, but you might need this to follow up with questions.* | |
| group_respondent4_med | RESPONDENT 4 | |
| q7a_name_med | q7a. Name of 4th respondent<br>*First and last name* | |

| Field | Question | Answer |
|---|---|---|
| q7b_date_med | q7b. Date of interview with 4th respondent<br>*Default is today's date.* | |
| q7c_title_med | q7c. Designation/title of 4th respondent<br>*Job title and designation (e.g. Medical officer in charge)* | |
| q7d_phone_med | q7d. Phone number of 4th respondent<br>*NB: Not required, but you might need this to follow up with questions.* | |
| group_respondent5_med | RESPONDENT 5 | |
| q8a_name_med | q8a. Name of 5th respondent<br>*First and last name* | |
| q8b_date_med | q8b. Date of interview with 5th respondent<br>*Default is today's date.* | |
| q8c_title_med | q8c. Designation/title of 5th respondent<br>*Job title and designation (e.g. Medical officer in charge)* | |
| q8d_phone_med | q8d. Phone number of 5th respondent<br>*NB: Not required, but you might need this to follow up with questions.* | |
| q9_interviewer_comments_med | q9. INTERVIEWER COMMENTS<br>*Enter any relevant notes prior to the interview.* | |
| q10_time_start_qa_med *(required)* | q10. ENTER START TIME OF INTERVIEW<br>*NB: The default is the current time.* | |
| introduction_med | QUESTIONNAIRE INTRODUCTION: Ok now we can begin the interview. Remember that all the questions in this part of the interview are about medications that are used to provide PAC at your facility. | |
| group_section_one_intro | | |
| section1_start | **SECTION I. FULL LISTING OF ITEMS BY COMPLICATION TYPE** | |
| section_one_skip_med | INTERVIEWER: WOULD YOU LIKE TO COMPLETE THIS SECTION NOW OR SKIP THIS SECTION AND RETURN TO IT LATER?<br>*You may need to skip if the participant has indicated that s/he cannot answer the questions in this section.* | 1 Do not skip, complete this section now.<br>2 Skip and come back to this section later. |
| D. Medications - Full list (1)<br>*Group relevant when: selected( ${section_one_skip_med} , '1')* | | |
| q104_med_note1 | 104 For each of the following medications and other medical products, can you tell me if the item is used for post abortion care at your facility? I'm going to ask about each of the five complication types separately. Is [ITEM] used for managing women with [COMPLICATION TYPE]? | |
| note_104_analgesic | ***Analgesic, anti-inflammatory, narcotic, etc.*** | |
| q104_M1_full_list *(required)* | q104_M1. Acetylsalicylic acid (aspirin)<br>*Select all that apply.*<br>*Response constrained to: if(selected(., 6) or selected(., 99), count-selected(.) = 1, count-selected(.) >= 1)* | 1 Incomplete abortion<br>2 Sepsis<br>3 Shock<br>4 Cervical/vaginal lacerations<br>5 Vaginal/uterine perforation<br>6 Not used at this facility<br>99 Don't know |
| q104_M2_full_list *(required)* | q104_M2. Paracetamol<br>*Select all that apply.*<br>*Response constrained to: if(selected(., 6) or selected(., 99), count-selected(.) = 1, count-selected(.) >= 1)* | 1 Incomplete abortion<br>2 Sepsis<br>3 Shock<br>4 Cervical/vaginal lacerations<br>5 Vaginal/uterine perforation<br>6 Not used at this facility<br>99 Don't know |
| q104_M3_full_list *(required)* | q104_M3. Pethidine hydrochloride<br>*Select all that apply.*<br>*Response constrained to: if(selected(., 6) or selected(., 99), count-selected(.) = 1, count-selected(.) >= 1)* | 1 Incomplete abortion<br>2 Sepsis<br>3 Shock<br>4 Cervical/vaginal lacerations<br>5 Vaginal/uterine perforation<br>6 Not used at this facility<br>99 Don't know |
| q104_M4_full_list *(required)* | q104_M4. Tramadol Hydrochloride<br>*Select all that apply.*<br>*Response constrained to: if(selected(., 6) or selected(., 99), count-selected(.) = 1, count-selected(.) >= 1)* | 1 Incomplete abortion<br>2 Sepsis<br>3 Shock<br>4 Cervical/vaginal lacerations<br>5 Vaginal/uterine perforation<br>6 Not used at this facility<br>99 Don't know |

| Field | Question | Answer |
|---|---|---|
| q104_M5_full_list *(required)* | q104_M5. Ibuprofen<br>*Select all that apply.*<br>*Response constrained to: if(selected(., 6) or selected(., 99), count-selected(.) = 1, count-selected(.) >= 1)* | 1 Incomplete abortion<br>2 Sepsis<br>3 Shock<br>4 Cervical/vaginal lacerations<br>5 Vaginal/uterine perforation<br>6 Not used at this facility<br>99 Don't know |
| q104_M6_full_list *(required)* | q104_M6. Diclofenac<br>*Select all that apply.*<br>*Response constrained to: if(selected(., 6) or selected(., 99), count-selected(.) = 1, count-selected(.) >= 1)* | 1 Incomplete abortion<br>2 Sepsis<br>3 Shock<br>4 Cervical/vaginal lacerations<br>5 Vaginal/uterine perforation<br>6 Not used at this facility<br>99 Don't know |
| q104_M7_full_list *(required)* | q104_M7. Diclofenac sodium<br>*Select all that apply.*<br>*Response constrained to: if(selected(., 6) or selected(., 99), count-selected(.) = 1, count-selected(.) >= 1)* | 1 Incomplete abortion<br>2 Sepsis<br>3 Shock<br>4 Cervical/vaginal lacerations<br>5 Vaginal/uterine perforation<br>6 Not used at this facility<br>99 Don't know |
| q104_M8_full_list *(required)* | q104_M8. Dypron (dipyrone), 250mg/ml (Metamizole)<br>*Select all that apply.*<br>*Response constrained to: if(selected(., 6) or selected(., 99), count-selected(.) = 1, count-selected(.) >= 1)* | 1 Incomplete abortion<br>2 Sepsis<br>3 Shock<br>4 Cervical/vaginal lacerations<br>5 Vaginal/uterine perforation<br>6 Not used at this facility<br>99 Don't know |
| q104_M9_full_list *(required)* | q104_M9. Fentanyl citrate, 50 mcg/ml<br>*Select all that apply.*<br>*Response constrained to: if(selected(., 6) or selected(., 99), count-selected(.) = 1, count-selected(.) >= 1)* | 1 Incomplete abortion<br>2 Sepsis<br>3 Shock<br>4 Cervical/vaginal lacerations<br>5 Vaginal/uterine perforation<br>6 Not used at this facility<br>99 Don't know |
| q104_analgesic_other *(required)* | q104_(analgesic)_Other. Are there any pain medications (i.e. analgesics) that we have not mentioned that are used for postabortion care at your facility?<br>*Question relevant when: selected( ${section_one_skip_med} , '1')* | 1 Yes<br>0 No<br>99 Don't know |
| D. Medications - Full list - specify (1 other)<br>*Group relevant when: selected( ${q104_analgesic_other} , '1') and selected( ${section_one_skip_med} , '1')* | | |
| note_104_analgesic_other | Please list the "other" analgesic items here. | |
| q104_M10_full_list_other | q104_M10. Other 1. Please specify: | |
| q104_M11_full_list_other | q104_M11. Other 2. Please specify: | |
| q104_M12_full_list_other | q104_M12. Other 3. Please specify: | |
| q104_M13_full_list_other | q104_M13. Other 4. Please specify: | |
| q104_M14_full_list_other | q104_M14. Other 5. Please specify: | |
| D. Medications - Full list - usage (1 other)<br>*Group relevant when: selected( ${q104_analgesic_other} , '1') and selected( ${section_one_skip_med} , '1')* | | |
| note_104_analgesic_other_b | For each "other" analgesic item, please tell me which of the five post abortion complication types it is used for. | |
| q104_M10_full_list *(required)* | q104_M10. Other 1: "[q104_M10_full_list_other]"<br>*Select all that apply.*<br>*Question relevant when: string-length( ${q104_M10_full_list_other} ) > 0 and selected( ${section_one_skip_med} , '1')*<br>*Response constrained to: if(selected(., 6) or selected(., 99), count-selected(.) = 1, count-selected(.) >= 1)* | 1 Incomplete abortion<br>2 Sepsis<br>3 Shock<br>4 Cervical/vaginal lacerations<br>5 Vaginal/uterine perforation<br>6 Not used at this facility<br>99 Don't know |

| Field | Question | Answer |
|---|---|---|
| q104_M11_full_list *(required)* | q104_M11. Other 2: "[q104_M11_full_list_other]"<br>*Select all that apply.*<br>*Question relevant when: string-length( ${q104_M11_full_list_other} ) > 0 and selected( ${section_one_skip_med} , '1')*<br>*Response constrained to: if(selected(., 6) or selected(., 99), count-selected(.) = 1, count-selected(.) >= 1)* | 1 Incomplete abortion<br>2 Sepsis<br>3 Shock<br>4 Cervical/vaginal lacerations<br>5 Vaginal/uterine perforation<br>6 Not used at this facility<br>99 Don't know |
| q104_M12_full_list *(required)* | q104_M12. Other 3: "[q104_M12_full_list_other]"<br>*Select all that apply.*<br>*Question relevant when: string-length( ${q104_M12_full_list_other} ) > 0 and selected( ${section_one_skip_med} , '1')*<br>*Response constrained to: if(selected(., 6) or selected(., 99), count-selected(.) = 1, count-selected(.) >= 1)* | 1 Incomplete abortion<br>2 Sepsis<br>3 Shock<br>4 Cervical/vaginal lacerations<br>5 Vaginal/uterine perforation<br>6 Not used at this facility<br>99 Don't know |
| q104_M13_full_list *(required)* | q104_M13. Other 4: "[q104_M13_full_list_other]"<br>*Select all that apply.*<br>*Question relevant when: string-length( ${q104_M13_full_list_other} ) > 0 and selected( ${section_one_skip_med} , '1')*<br>*Response constrained to: if(selected(., 6) or selected(., 99), count-selected(.) = 1, count-selected(.) >= 1)* | 1 Incomplete abortion<br>2 Sepsis<br>3 Shock<br>4 Cervical/vaginal lacerations<br>5 Vaginal/uterine perforation<br>6 Not used at this facility<br>99 Don't know |
| q104_M14_full_list *(required)* | q104_M14. Other 5: "[q104_M14_full_list_other]"<br>*Select all that apply.*<br>*Question relevant when: string-length( ${q104_M14_full_list_other} ) > 0 and selected( ${section_one_skip_med} , '1')*<br>*Response constrained to: if(selected(., 6) or selected(., 99), count-selected(.) = 1, count-selected(.) >= 1)* | 1 Incomplete abortion<br>2 Sepsis<br>3 Shock<br>4 Cervical/vaginal lacerations<br>5 Vaginal/uterine perforation<br>6 Not used at this facility<br>99 Don't know |
| D. Medications - Full list (2)<br>*Group relevant when: selected( ${section_one_skip_med} , '1')* | | |
| q104_med_note2 | 104 CONTINUED. For each of the following medications and other medical products, can you tell me if the item is used for post abortion care at your facility? I'm going to ask about each of the five complication types separately. Is [ITEM] used for managing women with [COMPLICATION TYPE]? | |
| note_104_anesthetic | ***Anesthetic - local, general, etc. (and oxygen)*** | |
| q104_M15_full_list *(required)* | q104_M15. Halothane<br>*Select all that apply.*<br>*Response constrained to: if(selected(., 6) or selected(., 99), count-selected(.) = 1, count-selected(.) >= 1)* | 1 Incomplete abortion<br>2 Sepsis<br>3 Shock<br>4 Cervical/vaginal lacerations<br>5 Vaginal/uterine perforation<br>6 Not used at this facility<br>99 Don't know |
| q104_M16_full_list *(required)* | q104_M16. Ketamine (Ketalar), 50mg/ml<br>*Select all that apply.*<br>*Response constrained to: if(selected(., 6) or selected(., 99), count-selected(.) = 1, count-selected(.) >= 1)* | 1 Incomplete abortion<br>2 Sepsis<br>3 Shock<br>4 Cervical/vaginal lacerations<br>5 Vaginal/uterine perforation<br>6 Not used at this facility<br>99 Don't know |
| q104_M17_full_list *(required)* | q104_M17. Propofol (Diprivan), 10mg/ml<br>*Select all that apply.*<br>*Response constrained to: if(selected(., 6) or selected(., 99), count-selected(.) = 1, count-selected(.) >= 1)* | 1 Incomplete abortion<br>2 Sepsis<br>3 Shock<br>4 Cervical/vaginal lacerations<br>5 Vaginal/uterine perforation<br>6 Not used at this facility<br>99 Don't know |

| Field | Question | Answer |
|---|---|---|
| q104_M18_full_list *(required)* | q104_M18. Suxamethonium chloride<br>*Select all that apply.*<br>*Response constrained to: if(selected(., 6) or selected(., 99), count-selected(.) = 1, count-selected(.) >= 1)* | 1 Incomplete abortion<br>2 Sepsis<br>3 Shock<br>4 Cervical/vaginal lacerations<br>5 Vaginal/uterine perforation<br>6 Not used at this facility<br>99 Don't know |
| q104_M19_full_list *(required)* | q104_M19. Thiopental sodium (Intraval)<br>*Select all that apply.*<br>*Response constrained to: if(selected(., 6) or selected(., 99), count-selected(.) = 1, count-selected(.) >= 1)* | 1 Incomplete abortion<br>2 Sepsis<br>3 Shock<br>4 Cervical/vaginal lacerations<br>5 Vaginal/uterine perforation<br>6 Not used at this facility<br>99 Don't know |
| q104_M20_full_list *(required)* | q104_M20. Oxygen<br>*Select all that apply.*<br>*Response constrained to: if(selected(., 6) or selected(., 99), count-selected(.) = 1, count-selected(.) >= 1)* | 1 Incomplete abortion<br>2 Sepsis<br>3 Shock<br>4 Cervical/vaginal lacerations<br>5 Vaginal/uterine perforation<br>6 Not used at this facility<br>99 Don't know |
| q104_M21_full_list *(required)* | q104_M21. Bupivacaine HCL 0.5% (Marcaine)<br>*Select all that apply.*<br>*Response constrained to: if(selected(., 6) or selected(., 99), count-selected(.) = 1, count-selected(.) >= 1)* | 1 Incomplete abortion<br>2 Sepsis<br>3 Shock<br>4 Cervical/vaginal lacerations<br>5 Vaginal/uterine perforation<br>6 Not used at this facility<br>99 Don't know |
| q104_M22_full_list *(required)* | q104_M22. Lidocaine HCl 2% + epinephrin 1:100,000<br>*Select all that apply.*<br>*Response constrained to: if(selected(., 6) or selected(., 99), count-selected(.) = 1, count-selected(.) >= 1)* | 1 Incomplete abortion<br>2 Sepsis<br>3 Shock<br>4 Cervical/vaginal lacerations<br>5 Vaginal/uterine perforation<br>6 Not used at this facility<br>99 Don't know |
| q104_M23_full_list *(required)* | q104_M23. Lidocaine HCl 5% + dextrose 7.5%<br>*Select all that apply.*<br>*Response constrained to: if(selected(., 6) or selected(., 99), count-selected(.) = 1, count-selected(.) >= 1)* | 1 Incomplete abortion<br>2 Sepsis<br>3 Shock<br>4 Cervical/vaginal lacerations<br>5 Vaginal/uterine perforation<br>6 Not used at this facility<br>99 Don't know |
| q104_M24_full_list *(required)* | q104_M24. Lidocaine HCl, 1%<br>*Select all that apply.*<br>*Response constrained to: if(selected(., 6) or selected(., 99), count-selected(.) = 1, count-selected(.) >= 1)* | 1 Incomplete abortion<br>2 Sepsis<br>3 Shock<br>4 Cervical/vaginal lacerations<br>5 Vaginal/uterine perforation<br>6 Not used at this facility<br>99 Don't know |
| q104_anesthetic_other *(required)* | q104_(anesthetics)_Other. Are there any anesthetics that we have not mentioned that are used for postabortion care at your facility?<br>*Question relevant when: selected( ${section_one_skip_med} , '1')* | 1 Yes<br>0 No<br>99 Don't know |
| D. Medications - Full list - specify (2 other)<br>*Group relevant when: selected( ${q104_anesthetic_other} , '1') and selected( ${section_one_skip_med} , '1')* | | |
| note_104_anesthetic_other | Please list the "other" anesthetic items here. | |
| q104_M25_full_list_other | q104_M25. Other 1. Please specify: | |
| q104_M26_full_list_other | q104_M26. Other 2. Please specify: | |
| q104_M27_full_list_other | q104_M27. Other 3. Please specify: | |
| q104_M28_full_list_other | q104_M28. Other 4. Please specify: | |
| q104_M29_full_list_other | q104_M29. Other 5. Please specify: | |

| Field | Question | Answer |
|---|---|---|
| D. Medications - Full list - usage (2 other)<br>*Group relevant when: selected( ${q104_anesthetic_other} , '1') and selected( ${section_one_skip_med} , '1')* | | |
| note_104_anesthetic_other_b | For each "other" anesthetic item, please tell me which of the five post abortion complication types it is used for. | |
| q104_M25_full_list *(required)* | q104_M25. Other 1: "[q104_M25_full_list_other]"<br>*Select all that apply.*<br>*Question relevant when: string-length( ${q104_M25_full_list_other} ) > 0 and selected( ${section_one_skip_med} , '1')*<br>*Response constrained to: if(selected(., 6) or selected(., 99), count-selected(.) = 1, count-selected(.) >= 1)* | 1 Incomplete abortion<br>2 Sepsis<br>3 Shock<br>4 Cervical/vaginal lacerations<br>5 Vaginal/uterine perforation<br>6 Not used at this facility<br>99 Don't know |
| q104_M26_full_list *(required)* | q104_M26. Other 2: "[q104_M26_full_list_other]"<br>*Select all that apply.*<br>*Question relevant when: string-length( ${q104_M26_full_list_other} ) > 0 and selected( ${section_one_skip_med} , '1')*<br>*Response constrained to: if(selected(., 6) or selected(., 99), count-selected(.) = 1, count-selected(.) >= 1)* | 1 Incomplete abortion<br>2 Sepsis<br>3 Shock<br>4 Cervical/vaginal lacerations<br>5 Vaginal/uterine perforation<br>6 Not used at this facility<br>99 Don't know |
| q104_M27_full_list *(required)* | q104_M27. Other 3: "[q104_M27_full_list_other]"<br>*Select all that apply.*<br>*Question relevant when: string-length( ${q104_M27_full_list_other} ) > 0 and selected( ${section_one_skip_med} , '1')*<br>*Response constrained to: if(selected(., 6) or selected(., 99), count-selected(.) = 1, count-selected(.) >= 1)* | 1 Incomplete abortion<br>2 Sepsis<br>3 Shock<br>4 Cervical/vaginal lacerations<br>5 Vaginal/uterine perforation<br>6 Not used at this facility<br>99 Don't know |
| q104_M28_full_list *(required)* | q104_M28. Other 4: "[q104_M28_full_list_other]"<br>*Select all that apply.*<br>*Question relevant when: string-length( ${q104_M28_full_list_other} ) > 0 and selected( ${section_one_skip_med} , '1')*<br>*Response constrained to: if(selected(., 6) or selected(., 99), count-selected(.) = 1, count-selected(.) >= 1)* | 1 Incomplete abortion<br>2 Sepsis<br>3 Shock<br>4 Cervical/vaginal lacerations<br>5 Vaginal/uterine perforation<br>6 Not used at this facility<br>99 Don't know |
| q104_M29_full_list *(required)* | q104_M29. Other 5: "[q104_M29_full_list_other]"<br>*Select all that apply.*<br>*Question relevant when: string-length( ${q104_M29_full_list_other} ) > 0 and selected( ${section_one_skip_med} , '1')*<br>*Response constrained to: if(selected(., 6) or selected(., 99), count-selected(.) = 1, count-selected(.) >= 1)* | 1 Incomplete abortion<br>2 Sepsis<br>3 Shock<br>4 Cervical/vaginal lacerations<br>5 Vaginal/uterine perforation<br>6 Not used at this facility<br>99 Don't know |
| D. Medications - Full list (3)<br>*Group relevant when: selected( ${section_one_skip_med} , '1')* | | |
| q104_med_note3 | 104 CONTINUED. For each of the following medications and other medical products, can you tell me if the item is used for post abortion care at your facility? I'm going to ask about each of the five complication types separately. Is [ITEM] used for managing women with [COMPLICATION TYPE]? | |
| note_104_antibiotics | ***Antibiotics*** | |
| q104_M30_full_list *(required)* | q104_M30. Amoxicillin<br>*Select all that apply.*<br>*Response constrained to: if(selected(., 6) or selected(., 99), count-selected(.) = 1, count-selected(.) >= 1)* | 1 Incomplete abortion<br>2 Sepsis<br>3 Shock<br>4 Cervical/vaginal lacerations<br>5 Vaginal/uterine perforation<br>6 Not used at this facility<br>99 Don't know |
| q104_M31_full_list *(required)* | q104_M31. Amoxicillin + Clauvulenic Acid 635mg<br>*Select all that apply.*<br>*Response constrained to: if(selected(., 6) or selected(., 99), count-selected(.) = 1, count-selected(.) >= 1)* | 1 Incomplete abortion<br>2 Sepsis<br>3 Shock<br>4 Cervical/vaginal lacerations<br>5 Vaginal/uterine perforation<br>6 Not used at this facility<br>99 Don't know |

| Field | Question | Answer |
|---|---|---|
| q104_M32_full_list *(required)* | q104_M32. Ampicillin<br>*Select all that apply.*<br>*Response constrained to: if(selected(., 6) or selected(., 99), count-selected(.) = 1, count-selected(.) >= 1)* | 1 Incomplete abortion<br>2 Sepsis<br>3 Shock<br>4 Cervical/vaginal lacerations<br>5 Vaginal/uterine perforation<br>6 Not used at this facility<br>99 Don't know |
| q104_M33_full_list *(required)* | q104_M33. Ampicillin (injection)<br>*Select all that apply.*<br>*Response constrained to: if(selected(., 6) or selected(., 99), count-selected(.) = 1, count-selected(.) >= 1)* | 1 Incomplete abortion<br>2 Sepsis<br>3 Shock<br>4 Cervical/vaginal lacerations<br>5 Vaginal/uterine perforation<br>6 Not used at this facility<br>99 Don't know |
| q104_M34_full_list *(required)* | q104_M34. Ampicillin/sulbactam (Unasyn)<br>*Select all that apply.*<br>*Response constrained to: if(selected(., 6) or selected(., 99), count-selected(.) = 1, count-selected(.) >= 1)* | 1 Incomplete abortion<br>2 Sepsis<br>3 Shock<br>4 Cervical/vaginal lacerations<br>5 Vaginal/uterine perforation<br>6 Not used at this facility<br>99 Don't know |
| q104_M35_full_list *(required)* | q104_M35. Ceftriaxone<br>*Select all that apply.*<br>*Response constrained to: if(selected(., 6) or selected(., 99), count-selected(.) = 1, count-selected(.) >= 1)* | 1 Incomplete abortion<br>2 Sepsis<br>3 Shock<br>4 Cervical/vaginal lacerations<br>5 Vaginal/uterine perforation<br>6 Not used at this facility<br>99 Don't know |
| q104_M36_full_list *(required)* | q104_M36. Chloramphenicol Sodium Succinate (injection)<br>*Select all that apply.*<br>*Response constrained to: if(selected(., 6) or selected(., 99), count-selected(.) = 1, count-selected(.) >= 1)* | 1 Incomplete abortion<br>2 Sepsis<br>3 Shock<br>4 Cervical/vaginal lacerations<br>5 Vaginal/uterine perforation<br>6 Not used at this facility<br>99 Don't know |
| q104_M37_full_list *(required)* | q104_M37. Ciprofloxacin<br>*Select all that apply.*<br>*Response constrained to: if(selected(., 6) or selected(., 99), count-selected(.) = 1, count-selected(.) >= 1)* | 1 Incomplete abortion<br>2 Sepsis<br>3 Shock<br>4 Cervical/vaginal lacerations<br>5 Vaginal/uterine perforation<br>6 Not used at this facility<br>99 Don't know |
| q104_M38_full_list *(required)* | q104_M38. Doxycycline<br>*Select all that apply.*<br>*Response constrained to: if(selected(., 6) or selected(., 99), count-selected(.) = 1, count-selected(.) >= 1)* | 1 Incomplete abortion<br>2 Sepsis<br>3 Shock<br>4 Cervical/vaginal lacerations<br>5 Vaginal/uterine perforation<br>6 Not used at this facility<br>99 Don't know |
| q104_M39_full_list *(required)* | q104_M39. Erythromycin<br>*Select all that apply.*<br>*Response constrained to: if(selected(., 6) or selected(., 99), count-selected(.) = 1, count-selected(.) >= 1)* | 1 Incomplete abortion<br>2 Sepsis<br>3 Shock<br>4 Cervical/vaginal lacerations<br>5 Vaginal/uterine perforation<br>6 Not used at this facility<br>99 Don't know |

| Field | Question | Answer |
|---|---|---|
| q104_M40_full_list *(required)* | q104_M40. Gentamycin<br>*Select all that apply.*<br>*Response constrained to: if(selected(., 6) or selected(., 99), count-selected(.) = 1, count-selected(.) >= 1)* | 1 Incomplete abortion<br>2 Sepsis<br>3 Shock<br>4 Cervical/vaginal lacerations<br>5 Vaginal/uterine perforation<br>6 Not used at this facility<br>99 Don't know |
| q104_M41_full_list *(required)* | q104_M41. Metronidazole (for infusion)<br>*Select all that apply.*<br>*Response constrained to: if(selected(., 6) or selected(., 99), count-selected(.) = 1, count-selected(.) >= 1)* | 1 Incomplete abortion<br>2 Sepsis<br>3 Shock<br>4 Cervical/vaginal lacerations<br>5 Vaginal/uterine perforation<br>6 Not used at this facility<br>99 Don't know |
| q104_M42_full_list *(required)* | q104_M42. Metronidazole (injection)<br>*Select all that apply.*<br>*Response constrained to: if(selected(., 6) or selected(., 99), count-selected(.) = 1, count-selected(.) >= 1)* | 1 Incomplete abortion<br>2 Sepsis<br>3 Shock<br>4 Cervical/vaginal lacerations<br>5 Vaginal/uterine perforation<br>6 Not used at this facility<br>99 Don't know |
| q104_M43_full_list *(required)* | q104_M43. Metronidazole (oral)<br>*Select all that apply.*<br>*Response constrained to: if(selected(., 6) or selected(., 99), count-selected(.) = 1, count-selected(.) >= 1)* | 1 Incomplete abortion<br>2 Sepsis<br>3 Shock<br>4 Cervical/vaginal lacerations<br>5 Vaginal/uterine perforation<br>6 Not used at this facility<br>99 Don't know |
| q104_M44_full_list *(required)* | q104_M44. Penicillin<br>*Select all that apply.*<br>*Response constrained to: if(selected(., 6) or selected(., 99), count-selected(.) = 1, count-selected(.) >= 1)* | 1 Incomplete abortion<br>2 Sepsis<br>3 Shock<br>4 Cervical/vaginal lacerations<br>5 Vaginal/uterine perforation<br>6 Not used at this facility<br>99 Don't know |
| q104_antibiotic_other *(required)* | q104_(antibiotic)_Other. Are there any antibiotics that we have not mentioned that are used for postabortion care at your facility?<br>*Question relevant when: selected( ${section_one_skip_med} , '1')* | 1 Yes<br>0 No<br>99 Don't know |
| D. Medications - Full list - specify (3 other)<br>*Group relevant when: selected( ${q104_antibiotic_other} , '1') and selected( ${section_one_skip_med} , '1')* | | |
| note_104_antibiotic_other | Please list the "other" antibiotic items here. | |
| q104_M45_full_list_other | q104_M45. Other 1. Please specify: | |
| q104_M46_full_list_other | q104_M46. Other 2. Please specify: | |
| q104_M47_full_list_other | q104_M47. Other 3. Please specify: | |
| q104_M48_full_list_other | q104_M48. Other 4. Please specify: | |
| q104_M49_full_list_other | q104_M49. Other 5. Please specify: | |
| D. Medications - Full list - usage (3 other)<br>*Group relevant when: selected( ${q104_antibiotic_other} , '1') and selected( ${section_one_skip_med} , '1')* | | |
| note_104_antibiotic_other_b | For each "other" antibiotic item, please tell me which of the five post abortion complication types it is used for. | |
| q104_M45_full_list *(required)* | q104_M45. Other 1: "[q104_M45_full_list_other]"<br>*Select all that apply.*<br>*Question relevant when: string-length( ${q104_M45_full_list_other} ) > 0 and selected( ${section_one_skip_med} , '1')*<br>*Response constrained to: if(selected(., 6) or selected(., 99), count-selected(.) = 1, count-selected(.) >= 1)* | 1 Incomplete abortion<br>2 Sepsis<br>3 Shock<br>4 Cervical/vaginal lacerations<br>5 Vaginal/uterine perforation<br>6 Not used at this facility<br>99 Don't know |

| Field | Question | Answer |
|---|---|---|
| q104_M46_full_list *(required)* | q104_M46. Other 2: "[q104_M46_full_list_other]"<br>*Select all that apply.*<br>*Question relevant when: string-length( ${q104_M46_full_list_other} ) > 0 and selected( ${section_one_skip_med} , '1')*<br>*Response constrained to: if(selected(., 6) or selected(., 99), count-selected(.) = 1, count-selected(.) >= 1)* | 1 Incomplete abortion<br>2 Sepsis<br>3 Shock<br>4 Cervical/vaginal lacerations<br>5 Vaginal/uterine perforation<br>6 Not used at this facility<br>99 Don't know |
| q104_M47_full_list *(required)* | q104_M47. Other 3: "[q104_M47_full_list_other]"<br>*Select all that apply.*<br>*Question relevant when: string-length( ${q104_M47_full_list_other} ) > 0 and selected( ${section_one_skip_med} , '1')*<br>*Response constrained to: if(selected(., 6) or selected(., 99), count-selected(.) = 1, count-selected(.) >= 1)* | 1 Incomplete abortion<br>2 Sepsis<br>3 Shock<br>4 Cervical/vaginal lacerations<br>5 Vaginal/uterine perforation<br>6 Not used at this facility<br>99 Don't know |
| q104_M48_full_list *(required)* | q104_M48. Other 4: "[q104_M48_full_list_other]"<br>*Select all that apply.*<br>*Question relevant when: string-length( ${q104_M48_full_list_other} ) > 0 and selected( ${section_one_skip_med} , '1')*<br>*Response constrained to: if(selected(., 6) or selected(., 99), count-selected(.) = 1, count-selected(.) >= 1)* | 1 Incomplete abortion<br>2 Sepsis<br>3 Shock<br>4 Cervical/vaginal lacerations<br>5 Vaginal/uterine perforation<br>6 Not used at this facility<br>99 Don't know |
| q104_M49_full_list *(required)* | q104_M49. Other 5: "[q104_M49_full_list_other]"<br>*Select all that apply.*<br>*Question relevant when: string-length( ${q104_M49_full_list_other} ) > 0 and selected( ${section_one_skip_med} , '1')*<br>*Response constrained to: if(selected(., 6) or selected(., 99), count-selected(.) = 1, count-selected(.) >= 1)* | 1 Incomplete abortion<br>2 Sepsis<br>3 Shock<br>4 Cervical/vaginal lacerations<br>5 Vaginal/uterine perforation<br>6 Not used at this facility<br>99 Don't know |
| D. Medications - Full list (4)<br>*Group relevant when: selected( ${section_one_skip_med} , '1')* | | |
| q104_med_note4 | 104 CONTINUED. For each of the following medications and other medical products, can you tell me if the item is used for post abortion care at your facility? I'm going to ask about each of the five complication types separately. Is [ITEM] used for managing women with [COMPLICATION TYPE]? | |
| note_104_antiseptic | ***Antiseptic*** | |
| q104_M50_full_list *(required)* | q104_M50. Cetrimide 15% ("Savlon")<br>*Select all that apply.*<br>*Response constrained to: if(selected(., 6) or selected(., 99), count-selected(.) = 1, count-selected(.) >= 1)* | 1 Incomplete abortion<br>2 Sepsis<br>3 Shock<br>4 Cervical/vaginal lacerations<br>5 Vaginal/uterine perforation<br>6 Not used at this facility<br>99 Don't know |
| q104_M51_full_list *(required)* | q104_M51. Povidone-iodine solution, 7.5%<br>*Select all that apply.*<br>*Response constrained to: if(selected(., 6) or selected(., 99), count-selected(.) = 1, count-selected(.) >= 1)* | 1 Incomplete abortion<br>2 Sepsis<br>3 Shock<br>4 Cervical/vaginal lacerations<br>5 Vaginal/uterine perforation<br>6 Not used at this facility<br>99 Don't know |
| q104_antiseptic_other *(required)* | q104_(antiseptic)_Other. Are there any antiseptics that we have not mentioned that are used for postabortion care at your facility?<br>*Question relevant when: selected( ${section_one_skip_med} , '1')* | 1 Yes<br>0 No<br>99 Don't know |
| D. Medications - Full list - specify (4 other)<br>*Group relevant when: selected( ${q104_antiseptic_other} , '1') and selected( ${section_one_skip_med} , '1')* | | |
| note_104_antiseptic_other | Please list the "other" antiseptic items here. | |
| q104_M52_full_list_other | q104_M52. Other 1. Please specify: | |
| q104_M53_full_list_other | q104_M53. Other 2. Please specify: | |
| q104_M54_full_list_other | q104_M54. Other 3. Please specify: | |
| q104_M54.1_full_list_other | q104_M54.1 Other 4. Please specify: | |
| q104_M54.2_full_list_other | q104_M54.2 Other 5. Please specify: | |
| D. Medications - Full list - usage (4 other)<br>*Group relevant when: selected( ${q104_antiseptic_other} , '1') and selected( ${section_one_skip_med} , '1')* | | |

| Field | Question | Answer |
|---|---|---|
| note_104_antiseptic_other_b | For each "other" antiseptic item, please tell me which of the five post abortion complication types it is used for. (or all or none). | |
| q104_M52_full_list *(required)* | q104_M52. Other 1: "[q104_M52_full_list_other]"<br>*Select all that apply.*<br>*Question relevant when: string-length( ${q104_M52_full_list_other} ) > 0 and selected( ${section_one_skip_med} , '1')*<br>*Response constrained to: if(selected(., 6) or selected(., 99), count-selected(.) = 1, count-selected(.) >= 1)* | 1 Incomplete abortion<br>2 Sepsis<br>3 Shock<br>4 Cervical/vaginal lacerations<br>5 Vaginal/uterine perforation<br>6 Not used at this facility<br>99 Don't know |
| q104_M53_full_list *(required)* | q104_M53. Other 2: "[q104_M53_full_list_other]"<br>*Select all that apply.*<br>*Question relevant when: string-length( ${q104_M53_full_list_other} ) > 0 and selected( ${section_one_skip_med} , '1')*<br>*Response constrained to: if(selected(., 6) or selected(., 99), count-selected(.) = 1, count-selected(.) >= 1)* | 1 Incomplete abortion<br>2 Sepsis<br>3 Shock<br>4 Cervical/vaginal lacerations<br>5 Vaginal/uterine perforation<br>6 Not used at this facility<br>99 Don't know |
| q104_M54_full_list *(required)* | q104_M54. Other 3: "[q104_M54_full_list_other]"<br>*Select all that apply.*<br>*Question relevant when: string-length( ${q104_M54_full_list_other} ) > 0 and selected( ${section_one_skip_med} , '1')*<br>*Response constrained to: if(selected(., 6) or selected(., 99), count-selected(.) = 1, count-selected(.) >= 1)* | 1 Incomplete abortion<br>2 Sepsis<br>3 Shock<br>4 Cervical/vaginal lacerations<br>5 Vaginal/uterine perforation<br>6 Not used at this facility<br>99 Don't know |
| q104_M54.1_full_list *(required)* | q104_M54.1 Other 4: "[q104_M54.1_full_list_other]"<br>*Select all that apply.*<br>*Question relevant when: string-length( ${q104_M54.1_full_list_other} ) > 0 and selected( ${section_one_skip_med} , '1')*<br>*Response constrained to: if(selected(., 6) or selected(., 99), count-selected(.) = 1, count-selected(.) >= 1)* | 1 Incomplete abortion<br>2 Sepsis<br>3 Shock<br>4 Cervical/vaginal lacerations<br>5 Vaginal/uterine perforation<br>6 Not used at this facility<br>99 Don't know |
| q104_M54.2_full_list *(required)* | q104_M54.2 Other 5: "[q104_M54.2_full_list_other]"<br>*Select all that apply.*<br>*Question relevant when: string-length( ${q104_M54.2_full_list_other} ) > 0 and selected( ${section_one_skip_med} , '1')*<br>*Response constrained to: if(selected(., 6) or selected(., 99), count-selected(.) = 1, count-selected(.) >= 1)* | 1 Incomplete abortion<br>2 Sepsis<br>3 Shock<br>4 Cervical/vaginal lacerations<br>5 Vaginal/uterine perforation<br>6 Not used at this facility<br>99 Don't know |
| D. Medications - Full list (5)<br>*Group relevant when: selected( ${section_one_skip_med} , '1')* | | |
| q104_med_note5 | 104 CONTINUED. For each of the following medications and other medical products, can you tell me if the item is used for post abortion care at your facility? I'm going to ask about each of the five complication types separately. Is [ITEM] used for managing women with [COMPLICATION TYPE]? | |
| note_104_antispas | ***Antispasmodic, anxiolytic, sedative, etc*** | |
| q104_M55_full_list *(required)* | q104_M55. Hyoscine butylbromide / scopolamine butilhisocin (Buscopan )<br>*Select all that apply.*<br>*Response constrained to: if(selected(., 6) or selected(., 99), count-selected(.) = 1, count-selected(.) >= 1)* | 1 Incomplete abortion<br>2 Sepsis<br>3 Shock<br>4 Cervical/vaginal lacerations<br>5 Vaginal/uterine perforation<br>6 Not used at this facility<br>99 Don't know |
| q104_M56_full_list *(required)* | q104_M56. Atropine sulfate (or sulphate)<br>*Select all that apply.*<br>*Response constrained to: if(selected(., 6) or selected(., 99), count-selected(.) = 1, count-selected(.) >= 1)* | 1 Incomplete abortion<br>2 Sepsis<br>3 Shock<br>4 Cervical/vaginal lacerations<br>5 Vaginal/uterine perforation<br>6 Not used at this facility<br>99 Don't know |

| Field | Question | Answer |
|---|---|---|
| q104_M57_full_list *(required)* | q104_M57. Papaverine HCl<br>*Select all that apply.*<br>*Response constrained to: if(selected(., 6) or selected(., 99), count-selected(.) = 1, count-selected(.) >= 1)* | 1 Incomplete abortion<br>2 Sepsis<br>3 Shock<br>4 Cervical/vaginal lacerations<br>5 Vaginal/uterine perforation<br>6 Not used at this facility<br>99 Don't know |
| q104_M58_full_list *(required)* | q104_M58. Diazepam (injection)<br>*Select all that apply.*<br>*Response constrained to: if(selected(., 6) or selected(., 99), count-selected(.) = 1, count-selected(.) >= 1)* | 1 Incomplete abortion<br>2 Sepsis<br>3 Shock<br>4 Cervical/vaginal lacerations<br>5 Vaginal/uterine perforation<br>6 Not used at this facility<br>99 Don't know |
| q104_M59_full_list *(required)* | q104_M59. Diazepam (oral)<br>*Select all that apply.*<br>*Response constrained to: if(selected(., 6) or selected(., 99), count-selected(.) = 1, count-selected(.) >= 1)* | 1 Incomplete abortion<br>2 Sepsis<br>3 Shock<br>4 Cervical/vaginal lacerations<br>5 Vaginal/uterine perforation<br>6 Not used at this facility<br>99 Don't know |
| q104_antispasmodic_other *(required)* | q104_(antispasmodic_etc)_Other. Are there any antispasmodics that we have not mentioned that are used for postabortion care at your facility?<br>*Question relevant when: selected( ${section_one_skip_med} , '1')* | 1 Yes<br>0 No<br>99 Don't know |
| D. Medications - Full list - specify (5 other)<br>*Group relevant when: selected( ${q104_antispasmodic_other} , '1') and selected( ${section_one_skip_med} , '1')* | | |
| note_104_antispasmodic_other | Please list the "other" antispasmodic, anxiolytic, sedative, etc items here. | |
| q104_M60_full_list_other | q104_M60. Other 1. Please specify: | |
| q104_M61_full_list_other | q104_M61. Other 2. Please specify: | |
| q104_M62_full_list_other | q104_M62. Other 3. Please specify: | |
| q104_M63_full_list_other | q104_M63. Other 4. Please specify: | |
| q104_M64_full_list_other | q104_M64. Other 5. Please specify: | |
| D. Medications - Full list - usage (5 other)<br>*Group relevant when: selected( ${q104_antispasmodic_other} , '1') and selected( ${section_one_skip_med} , '1')* | | |
| note_104_antispasmodic_other_b | For each "other" antispasmodic, anxiolytic, sedative, etc item, please tell me which of the five post abortion complication types it is used for. | |
| q104_M60_full_list *(required)* | q104_M60. Other 1: "[q104_M60_full_list_other]"<br>*Select all that apply.*<br>*Question relevant when: string-length( ${q104_M60_full_list_other} ) > 0 and selected( ${section_one_skip_med} , '1')*<br>*Response constrained to: if(selected(., 6) or selected(., 99), count-selected(.) = 1, count-selected(.) >= 1)* | 1 Incomplete abortion<br>2 Sepsis<br>3 Shock<br>4 Cervical/vaginal lacerations<br>5 Vaginal/uterine perforation<br>6 Not used at this facility<br>99 Don't know |
| q104_M61_full_list *(required)* | q104_M61. Other 2: "[q104_M61_full_list_other]"<br>*Select all that apply.*<br>*Question relevant when: string-length( ${q104_M61_full_list_other} ) > 0 and selected( ${section_one_skip_med} , '1')*<br>*Response constrained to: if(selected(., 6) or selected(., 99), count-selected(.) = 1, count-selected(.) >= 1)* | 1 Incomplete abortion<br>2 Sepsis<br>3 Shock<br>4 Cervical/vaginal lacerations<br>5 Vaginal/uterine perforation<br>6 Not used at this facility<br>99 Don't know |
| q104_M62_full_list *(required)* | q104_M62. Other 3: "[q104_M62_full_list_other]"<br>*Select all that apply.*<br>*Question relevant when: string-length( ${q104_M62_full_list_other} ) > 0 and selected( ${section_one_skip_med} , '1')*<br>*Response constrained to: if(selected(., 6) or selected(., 99), count-selected(.) = 1, count-selected(.) >= 1)* | 1 Incomplete abortion<br>2 Sepsis<br>3 Shock<br>4 Cervical/vaginal lacerations<br>5 Vaginal/uterine perforation<br>6 Not used at this facility<br>99 Don't know |

| Field | Question | Answer |
|---|---|---|
| q104_M63_full_list *(required)* | q104_M63. Other 4: "[q104_M63_full_list_other]"<br>*Select all that apply.*<br>*Question relevant when: string-length( ${q104_M63_full_list_other} ) > 0 and selected( ${section_one_skip_med} , '1')*<br>*Response constrained to: if(selected(., 6) or selected(., 99), count-selected(.) = 1, count-selected(.) >= 1)* | 1 Incomplete abortion<br>2 Sepsis<br>3 Shock<br>4 Cervical/vaginal lacerations<br>5 Vaginal/uterine perforation<br>6 Not used at this facility<br>99 Don't know |
| q104_M64_full_list *(required)* | q104_M64. Other 5: "[q104_M64_full_list_other]"<br>*Select all that apply.*<br>*Question relevant when: string-length( ${q104_M64_full_list_other} ) > 0 and selected( ${section_one_skip_med} , '1')*<br>*Response constrained to: if(selected(., 6) or selected(., 99), count-selected(.) = 1, count-selected(.) >= 1)* | 1 Incomplete abortion<br>2 Sepsis<br>3 Shock<br>4 Cervical/vaginal lacerations<br>5 Vaginal/uterine perforation<br>6 Not used at this facility<br>99 Don't know |
| D. Medications - Full list (6)<br>*Group relevant when: selected( ${section_one_skip_med} , '1')* | | |
| q104_med_note6 | 104 CONTINUED. For each of the following medications and other medical products, can you tell me if the item is used for post abortion care at your facility? I'm going to ask about each of the five complication types separately. Is [ITEM] used for managing women with [COMPLICATION TYPE]? | |
| note_104_adrenergic | ***Adrenergic*** | |
| q104_M65_full_list *(required)* | q104_M65. Ephedrine HCl<br>*Select all that apply.*<br>*Response constrained to: if(selected(., 6) or selected(., 99), count-selected(.) = 1, count-selected(.) >= 1)* | 1 Incomplete abortion<br>2 Sepsis<br>3 Shock<br>4 Cervical/vaginal lacerations<br>5 Vaginal/uterine perforation<br>6 Not used at this facility<br>99 Don't know |
| q104_M66_full_list *(required)* | q104_M66. Epinephrine<br>*Select all that apply.*<br>*Response constrained to: if(selected(., 6) or selected(., 99), count-selected(.) = 1, count-selected(.) >= 1)* | 1 Incomplete abortion<br>2 Sepsis<br>3 Shock<br>4 Cervical/vaginal lacerations<br>5 Vaginal/uterine perforation<br>6 Not used at this facility<br>99 Don't know |
| q104_andrenergic_other *(required)* | q104_(adrenergic)_Other. Are there any andrenergic drugs that we have not mentioned that are used for postabortion care at your facility?<br>*Question relevant when: selected( ${section_one_skip_med} , '1')* | 1 Yes<br>0 No<br>99 Don't know |
| D. Medications - Full list - specify (6 Other)<br>*Group relevant when: selected( ${q104_andrenergic_other} , '1') and selected( ${section_one_skip_med} , '1')* | | |
| note_104_andrenergic_other | Please list the "other" adrenergic items here. | |
| q104_M67_full_list_other | q104_M67. Other 1. Please specify: | |
| q104_M68_full_list_other | q104_M68. Other 2. Please specify: | |
| q104_M69_full_list_other | q104_M69. Other 3. Please specify: | |
| q104_M69.1_full_list_other | q104_M69.1 Other 4. Please specify: | |
| q104_M69.2_full_list_other | q104_M69.2 Other 5. Please specify: | |
| D. Medications - Full list - usage (6 Other)<br>*Group relevant when: selected( ${q104_andrenergic_other} , '1') and selected( ${section_one_skip_med} , '1')* | | |
| note_104_andrenergic_other_b | For each "other" adrenergic item, please tell me which of the five post abortion complication types it is used for. | |
| q104_M67_full_list *(required)* | q104_M67. Other 1: "[q104_M67_full_list_other]"<br>*Select all that apply.*<br>*Question relevant when: string-length( ${q104_M67_full_list_other} ) > 0 and selected( ${section_one_skip_med} , '1')*<br>*Response constrained to: if(selected(., 6) or selected(., 99), count-selected(.) = 1, count-selected(.) >= 1)* | 1 Incomplete abortion<br>2 Sepsis<br>3 Shock<br>4 Cervical/vaginal lacerations<br>5 Vaginal/uterine perforation<br>6 Not used at this facility<br>99 Don't know |

| Field | Question | Answer |
|---|---|---|
| q104_M68_full_list *(required)* | q104_M68. Other 2: "[q104_M68_full_list_other]"<br>*Select all that apply.*<br>*Question relevant when: string-length( ${q104_M68_full_list_other} ) > 0 and selected( ${section_one_skip_med} , '1')*<br>*Response constrained to: if(selected(., 6) or selected(., 99), count-selected(.) = 1, count-selected(.) >= 1)* | 1 Incomplete abortion<br>2 Sepsis<br>3 Shock<br>4 Cervical/vaginal lacerations<br>5 Vaginal/uterine perforation<br>6 Not used at this facility<br>99 Don't know |
| q104_M69_full_list *(required)* | q104_M69. Other 3: "[q104_M69_full_list_other]"<br>*Select all that apply.*<br>*Question relevant when: string-length( ${q104_M69_full_list_other} ) > 0 and selected( ${section_one_skip_med} , '1')*<br>*Response constrained to: if(selected(., 6) or selected(., 99), count-selected(.) = 1, count-selected(.) >= 1)* | 1 Incomplete abortion<br>2 Sepsis<br>3 Shock<br>4 Cervical/vaginal lacerations<br>5 Vaginal/uterine perforation<br>6 Not used at this facility<br>99 Don't know |
| q104_M69.1_full_list *(required)* | q104_M69.1 Other 4: "[q104_M69.1_full_list_other]"<br>*Select all that apply.*<br>*Question relevant when: string-length( ${q104_M69.1_full_list_other} ) > 0 and selected( ${section_one_skip_med} , '1')*<br>*Response constrained to: if(selected(., 6) or selected(., 99), count-selected(.) = 1, count-selected(.) >= 1)* | 1 Incomplete abortion<br>2 Sepsis<br>3 Shock<br>4 Cervical/vaginal lacerations<br>5 Vaginal/uterine perforation<br>6 Not used at this facility<br>99 Don't know |
| q104_M69.2_full_list *(required)* | q104_M69.2 Other 5: "[q104_M69.2_full_list_other]"<br>*Select all that apply.*<br>*Question relevant when: string-length( ${q104_M69.2_full_list_other} ) > 0 and selected( ${section_one_skip_med} , '1')*<br>*Response constrained to: if(selected(., 6) or selected(., 99), count-selected(.) = 1, count-selected(.) >= 1)* | 1 Incomplete abortion<br>2 Sepsis<br>3 Shock<br>4 Cervical/vaginal lacerations<br>5 Vaginal/uterine perforation<br>6 Not used at this facility<br>99 Don't know |
| D. Medications - Full list (7)<br>*Group relevant when: selected( ${section_one_skip_med} , '1')* | | |
| q104_med_note7 | 104 CONTINUED. For each of the following medications and other medical products, can you tell me if the item is used for post abortion care at your facility? I'm going to ask about each of the five complication types separately. Is [ITEM] used for managing women with [COMPLICATION TYPE]? | |
| note_104_blood | ***Blood products*** | |
| q104_M70_full_list *(required)* | q104_M70. Unit of packed red blood cells<br>*Select all that apply.*<br>*Response constrained to: if(selected(., 6) or selected(., 99), count-selected(.) = 1, count-selected(.) >= 1)* | 1 Incomplete abortion<br>2 Sepsis<br>3 Shock<br>4 Cervical/vaginal lacerations<br>5 Vaginal/uterine perforation<br>6 Not used at this facility<br>99 Don't know |
| q104_M71_full_list *(required)* | q104_M71. Unit of platelets<br>*Select all that apply.*<br>*Response constrained to: if(selected(., 6) or selected(., 99), count-selected(.) = 1, count-selected(.) >= 1)* | 1 Incomplete abortion<br>2 Sepsis<br>3 Shock<br>4 Cervical/vaginal lacerations<br>5 Vaginal/uterine perforation<br>6 Not used at this facility<br>99 Don't know |
| q104_M72_full_list *(required)* | q104_M72. Unit of whole blood<br>*Select all that apply.*<br>*Response constrained to: if(selected(., 6) or selected(., 99), count-selected(.) = 1, count-selected(.) >= 1)* | 1 Incomplete abortion<br>2 Sepsis<br>3 Shock<br>4 Cervical/vaginal lacerations<br>5 Vaginal/uterine perforation<br>6 Not used at this facility<br>99 Don't know |
| q104_bloods_other *(required)* | q104_(blood)_Other. Are there any blood products that we have not mentioned that are used for postabortion care at your facility?<br>*Question relevant when: selected( ${section_one_skip_med} , '1')* | 1 Yes<br>0 No<br>99 Don't know |
| D. Medications - Full list - specify (7 Other)<br>*Group relevant when: selected( ${q104_bloods_other} , '1') and selected( ${section_one_skip_med} , '1')* | | |
| note_104_bloods_other | Please list the "other" blood products items here. | |

| Field | Question | Answer |
|---|---|---|
| q104_M73_full_list_other | q104_M73. Other 1. Please specify: | |
| q104_M74_full_list_other | q104_M74. Other 2. Please specify: | |
| q104_M75_full_list_other | q104_M75. Other 3. Please specify: | |
| q104_M75.1_full_list_other | q104_M75.1 Other 4. Please specify: | |
| q104_M75.2_full_list_other | q104_M75.2 Other 5. Please specify: | |
| D. Medications - Full list - usage (7 Other)<br>*Group relevant when: selected( ${q104_bloods_other} , '1') and selected( ${section_one_skip_med} , '1')* | | |
| note_104_bloods_other_b | For each "other" blood product item, please tell me which of the five post abortion complication types it is used for. | |
| q104_M73_full_list *(required)* | q104_M73. Other 1: "[q104_M73_full_list_other]"<br>*Select all that apply.*<br>*Question relevant when: string-length( ${q104_M73_full_list_other} ) > 0 and selected( ${section_one_skip_med} , '1')*<br>*Response constrained to: if(selected(., 6) or selected(., 99), count-selected(.) = 1, count-selected(.) >= 1)* | 1 Incomplete abortion<br>2 Sepsis<br>3 Shock<br>4 Cervical/vaginal lacerations<br>5 Vaginal/uterine perforation<br>6 Not used at this facility<br>99 Don't know |
| q104_M74_full_list *(required)* | q104_M74. Other 2: "[q104_M74_full_list_other]"<br>*Select all that apply.*<br>*Question relevant when: string-length( ${q104_M74_full_list_other} ) > 0 and selected( ${section_one_skip_med} , '1')*<br>*Response constrained to: if(selected(., 6) or selected(., 99), count-selected(.) = 1, count-selected(.) >= 1)* | 1 Incomplete abortion<br>2 Sepsis<br>3 Shock<br>4 Cervical/vaginal lacerations<br>5 Vaginal/uterine perforation<br>6 Not used at this facility<br>99 Don't know |
| q104_M75_full_list *(required)* | q104_M75. Other 3: "[q104_M75_full_list_other]"<br>*Select all that apply.*<br>*Question relevant when: string-length( ${q104_M75_full_list_other} ) > 0 and selected( ${section_one_skip_med} , '1')*<br>*Response constrained to: if(selected(., 6) or selected(., 99), count-selected(.) = 1, count-selected(.) >= 1)* | 1 Incomplete abortion<br>2 Sepsis<br>3 Shock<br>4 Cervical/vaginal lacerations<br>5 Vaginal/uterine perforation<br>6 Not used at this facility<br>99 Don't know |
| q104_M75.1_full_list *(required)* | q104_M75.1 Other 4: "[q104_M75.1_full_list_other]"<br>*Select all that apply.*<br>*Question relevant when: string-length( ${q104_M75.1_full_list_other} ) > 0 and selected( ${section_one_skip_med} , '1')*<br>*Response constrained to: if(selected(., 6) or selected(., 99), count-selected(.) = 1, count-selected(.) >= 1)* | 1 Incomplete abortion<br>2 Sepsis<br>3 Shock<br>4 Cervical/vaginal lacerations<br>5 Vaginal/uterine perforation<br>6 Not used at this facility<br>99 Don't know |
| q104_M75.2_full_list *(required)* | q104_M75.2 Other 5: "[q104_M75.2_full_list_other]"<br>*Select all that apply.*<br>*Question relevant when: string-length( ${q104_M75.2_full_list_other} ) > 0 and selected( ${section_one_skip_med} , '1')*<br>*Response constrained to: if(selected(., 6) or selected(., 99), count-selected(.) = 1, count-selected(.) >= 1)* | 1 Incomplete abortion<br>2 Sepsis<br>3 Shock<br>4 Cervical/vaginal lacerations<br>5 Vaginal/uterine perforation<br>6 Not used at this facility<br>99 Don't know |
| D. Medications - Full list (8)<br>*Group relevant when: selected( ${section_one_skip_med} , '1')* | | |
| q104_med_note8 | 104 CONTINUED. For each of the following medications and other medical products, can you tell me if the item is used for post abortion care at your facility? I'm going to ask about each of the five complication types separately. Is [ITEM] used for managing women with [COMPLICATION TYPE]? | |
| note_104_hemostatic | ***Hemostatics*** | |
| q104_M76_full_list *(required)* | q104_M76. Dicynone / Dicynene (Etamsylate)<br>*Select all that apply.*<br>*Response constrained to: if(selected(., 6) or selected(., 99), count-selected(.) = 1, count-selected(.) >= 1)* | 1 Incomplete abortion<br>2 Sepsis<br>3 Shock<br>4 Cervical/vaginal lacerations<br>5 Vaginal/uterine perforation<br>6 Not used at this facility<br>99 Don't know |

| Field | Question | Answer |
|---|---|---|
| q104_M77_full_list *(required)* | q104_M77. Tranexamic acid (Kapron, Cyklokapron)<br>*Select all that apply.*<br>*Response constrained to: if(selected(., 6) or selected(., 99), count-selected(.) = 1, count-selected(.) >= 1)* | 1 Incomplete abortion<br>2 Sepsis<br>3 Shock<br>4 Cervical/vaginal lacerations<br>5 Vaginal/uterine perforation<br>6 Not used at this facility<br>99 Don't know |
| q104_M78_full_list *(required)* | q104_M78. Polygeline 4%<br>*Select all that apply.*<br>*Response constrained to: if(selected(., 6) or selected(., 99), count-selected(.) = 1, count-selected(.) >= 1)* | 1 Incomplete abortion<br>2 Sepsis<br>3 Shock<br>4 Cervical/vaginal lacerations<br>5 Vaginal/uterine perforation<br>6 Not used at this facility<br>99 Don't know |
| q104_hemostatic_other *(required)* | q104_(hemostatic)_Other. Are there any other hemostatic drugs that we have not mentioned that are used for postabortion care at your facility?<br>*Question relevant when: selected( ${section_one_skip_med} , '1')* | 1 Yes<br>0 No<br>99 Don't know |
| D. Medications - Full list - specify (8 Other)<br>*Group relevant when: selected( ${q104_hemostatic_other} , '1') and selected( ${section_one_skip_med} , '1')* | | |
| note_104_hemostatic_other | Please list the "other" hemostatic items here. | |
| q104_M79_full_list_other | q104_M79. Other 1. Please specify: | |
| q104_M80_full_list_other | q104_M80. Other 2. Please specify: | |
| q104_M81_full_list_other | q104_M81. Other 3. Please specify: | |
| q104_M81.1_full_list_other | q104_M81.1 Other 4. Please specify: | |
| q104_M81.2_full_list_other | q104_M81.2 Other 5. Please specify: | |
| D. Medications - Full list - usage (8 Other)<br>*Group relevant when: selected( ${q104_hemostatic_other} , '1') and selected( ${section_one_skip_med} , '1')* | | |
| note_104_hemostatic_other_b | For each "other" hemostatic item, please tell me which of the five post abortion complication types it is used for. | |
| q104_M79_full_list *(required)* | q104_M79. Other 1: "[q104_M79_full_list_other]"<br>*Select all that apply.*<br>*Question relevant when: string-length( ${q104_M79_full_list_other} ) > 0 and selected( ${section_one_skip_med} , '1')*<br>*Response constrained to: if(selected(., 6) or selected(., 99), count-selected(.) = 1, count-selected(.) >= 1)* | 1 Incomplete abortion<br>2 Sepsis<br>3 Shock<br>4 Cervical/vaginal lacerations<br>5 Vaginal/uterine perforation<br>6 Not used at this facility<br>99 Don't know |
| q104_M80_full_list *(required)* | q104_M80. Other 2: "[q104_M80_full_list_other]"<br>*Select all that apply.*<br>*Question relevant when: string-length( ${q104_M80_full_list_other} ) > 0 and selected( ${section_one_skip_med} , '1')*<br>*Response constrained to: if(selected(., 6) or selected(., 99), count-selected(.) = 1, count-selected(.) >= 1)* | 1 Incomplete abortion<br>2 Sepsis<br>3 Shock<br>4 Cervical/vaginal lacerations<br>5 Vaginal/uterine perforation<br>6 Not used at this facility<br>99 Don't know |
| q104_M81_full_list *(required)* | q104_M81. Other 3: "[q104_M81_full_list_other]"<br>*Select all that apply.*<br>*Question relevant when: string-length( ${q104_M81_full_list_other} ) > 0 and selected( ${section_one_skip_med} , '1')*<br>*Response constrained to: if(selected(., 6) or selected(., 99), count-selected(.) = 1, count-selected(.) >= 1)* | 1 Incomplete abortion<br>2 Sepsis<br>3 Shock<br>4 Cervical/vaginal lacerations<br>5 Vaginal/uterine perforation<br>6 Not used at this facility<br>99 Don't know |
| q104_M81.1_full_list *(required)* | q104_M81.1 Other 4: "[q104_M81.1_full_list_other]"<br>*Select all that apply.*<br>*Question relevant when: string-length( ${q104_M81.1_full_list_other} ) > 0 and selected( ${section_one_skip_med} , '1')*<br>*Response constrained to: if(selected(., 6) or selected(., 99), count-selected(.) = 1, count-selected(.) >= 1)* | 1 Incomplete abortion<br>2 Sepsis<br>3 Shock<br>4 Cervical/vaginal lacerations<br>5 Vaginal/uterine perforation<br>6 Not used at this facility<br>99 Don't know |

| Field | Question | Answer |
|---|---|---|
| q104_M81.2_full_list *(required)* | q104_M81.2 Other 5: "[q104_M81.2_full_list_other]"<br>*Select all that apply.*<br>*Question relevant when: string-length( ${q104_M81.2_full_list_other} ) > 0 and selected( ${section_one_skip_med} , '1')*<br>*Response constrained to: if(selected(., 6) or selected(., 99), count-selected(.) = 1, count-selected(.) >= 1)* | 1 Incomplete abortion<br>2 Sepsis<br>3 Shock<br>4 Cervical/vaginal lacerations<br>5 Vaginal/uterine perforation<br>6 Not used at this facility<br>99 Don't know |
| D. Medications - Full list (9)<br>*Group relevant when: selected( ${section_one_skip_med} , '1')* | | |
| q104_med_note9 | 104 CONTINUED. For each of the following medications and other medical products, can you tell me if the item is used for post abortion care at your facility? I'm going to ask about each of the five complication types separately. Is [ITEM] used for managing women with [COMPLICATION TYPE]? | |
| note_104_saline | ***Saline, glucose, water, etc*** | |
| q104_M82_full_list *(required)* | q104_M82. IV solution, dextrose 2.5% in NaCl 0.45%<br>*Select all that apply.*<br>*Response constrained to: if(selected(., 6) or selected(., 99), count-selected(.) = 1, count-selected(.) >= 1)* | 1 Incomplete abortion<br>2 Sepsis<br>3 Shock<br>4 Cervical/vaginal lacerations<br>5 Vaginal/uterine perforation<br>6 Not used at this facility<br>99 Don't know |
| q104_M83_full_list *(required)* | q104_M83. IV solution, glucose (5%), isotonic<br>*Select all that apply.*<br>*Response constrained to: if(selected(., 6) or selected(., 99), count-selected(.) = 1, count-selected(.) >= 1)* | 1 Incomplete abortion<br>2 Sepsis<br>3 Shock<br>4 Cervical/vaginal lacerations<br>5 Vaginal/uterine perforation<br>6 Not used at this facility<br>99 Don't know |
| q104_M84_full_list *(required)* | q104_M84. Ringers lactate (prepared onsite)<br>*Select all that apply.*<br>*Response constrained to: if(selected(., 6) or selected(., 99), count-selected(.) = 1, count-selected(.) >= 1)* | 1 Incomplete abortion<br>2 Sepsis<br>3 Shock<br>4 Cervical/vaginal lacerations<br>5 Vaginal/uterine perforation<br>6 Not used at this facility<br>99 Don't know |
| q104_M85_full_list *(required)* | q104_M85. Ringers lactate (bought from pharmacy/MSD)<br>*Select all that apply.*<br>*Response constrained to: if(selected(., 6) or selected(., 99), count-selected(.) = 1, count-selected(.) >= 1)* | 1 Incomplete abortion<br>2 Sepsis<br>3 Shock<br>4 Cervical/vaginal lacerations<br>5 Vaginal/uterine perforation<br>6 Not used at this facility<br>99 Don't know |
| q104_M86_full_list *(required)* | q104_M86. Saline, 0.9% (Normal Saline) (prepared onsite)<br>*Select all that apply.*<br>*Response constrained to: if(selected(., 6) or selected(., 99), count-selected(.) = 1, count-selected(.) >= 1)* | 1 Incomplete abortion<br>2 Sepsis<br>3 Shock<br>4 Cervical/vaginal lacerations<br>5 Vaginal/uterine perforation<br>6 Not used at this facility<br>99 Don't know |
| q104_M87_full_list *(required)* | q104_M87. Saline, 0.9% (Normal Saline) (bought from pharmacy/MSD)<br>*Select all that apply.*<br>*Response constrained to: if(selected(., 6) or selected(., 99), count-selected(.) = 1, count-selected(.) >= 1)* | 1 Incomplete abortion<br>2 Sepsis<br>3 Shock<br>4 Cervical/vaginal lacerations<br>5 Vaginal/uterine perforation<br>6 Not used at this facility<br>99 Don't know |

| Field | Question | Answer |
| --- | --- | --- |
| q104_M88_full_list *(required)* | q104_M88. Solution for Infusion, glucose 5% + NaCl 0.9% (prepared onsite)<br>*Select all that apply.*<br>*Response constrained to: if(selected(., 6) or selected(., 99), count-selected(.) = 1, count-selected(.) >= 1)* | 1 Incomplete abortion<br>2 Sepsis<br>3 Shock<br>4 Cervical/vaginal lacerations<br>5 Vaginal/uterine perforation<br>6 Not used at this facility<br>99 Don't know |
| q104_M89_full_list *(required)* | q104_M89. Solution for Infusion, glucose 5% + NaCl 0.9% (bought from pharmacy or MSD)<br>*Select all that apply.*<br>*Response constrained to: if(selected(., 6) or selected(., 99), count-selected(.) = 1, count-selected(.) >= 1)* | 1 Incomplete abortion<br>2 Sepsis<br>3 Shock<br>4 Cervical/vaginal lacerations<br>5 Vaginal/uterine perforation<br>6 Not used at this facility<br>99 Don't know |
| q104_M90_full_list *(required)* | q104_M90. Water for injection<br>*Select all that apply.*<br>*Response constrained to: if(selected(., 6) or selected(., 99), count-selected(.) = 1, count-selected(.) >= 1)* | 1 Incomplete abortion<br>2 Sepsis<br>3 Shock<br>4 Cervical/vaginal lacerations<br>5 Vaginal/uterine perforation<br>6 Not used at this facility<br>99 Don't know |
| q104_saline_other *(required)* | q104_(saline_etc)_Other. Are there any other items that include saline, glucose, water, etc. that we have not mentioned that are used for postabortion care at your facility?<br>*Question relevant when: selected( ${section_one_skip_med} , '1')* | 1 Yes<br>0 No<br>99 Don't know |
| D. Medications - Full list - specify (9 Other)<br>*Group relevant when: selected( ${q104_saline_other} , '1') and selected( ${section_one_skip_med} , '1')* | | |
| note_104_saline_other | Please list the "other" saline, glucose, water, etc. items here. | |
| q104_M91_full_list_other | q104_M91. Other 1. Please specify: | |
| q104_M92_full_list_other | q104_M92. Other 2. Please specify: | |
| q104_M93_full_list_other | q104_M93. Other 3. Please specify: | |
| q104_M94_full_list_other | q104_M94. Other 4. Please specify: | |
| q104_M95_full_list_other | q104_M95. Other 5. Please specify: | |
| D. Medications - Full list - usage (9 Other)<br>*Group relevant when: selected( ${q104_saline_other} , '1') and selected( ${section_one_skip_med} , '1')* | | |
| note_104_saline_other_b | For each "other" saline, glucose, water, etc. item, please tell me which of the five post abortion complication types it is used for. | |
| q104_M91_full_list *(required)* | q104_M91. Other 1: "[q104_M91_full_list_other]"<br>*Select all that apply.*<br>*Question relevant when: string-length( ${q104_M91_full_list_other} ) > 0 and selected( ${section_one_skip_med} , '1')*<br>*Response constrained to: if(selected(., 6) or selected(., 99), count-selected(.) = 1, count-selected(.) >= 1)* | 1 Incomplete abortion<br>2 Sepsis<br>3 Shock<br>4 Cervical/vaginal lacerations<br>5 Vaginal/uterine perforation<br>6 Not used at this facility<br>99 Don't know |
| q104_M92_full_list *(required)* | q104_M92. Other 2: "[q104_M92_full_list_other]"<br>*Select all that apply.*<br>*Question relevant when: string-length( ${q104_M92_full_list_other} ) > 0 and selected( ${section_one_skip_med} , '1')*<br>*Response constrained to: if(selected(., 6) or selected(., 99), count-selected(.) = 1, count-selected(.) >= 1)* | 1 Incomplete abortion<br>2 Sepsis<br>3 Shock<br>4 Cervical/vaginal lacerations<br>5 Vaginal/uterine perforation<br>6 Not used at this facility<br>99 Don't know |
| q104_M93_full_list *(required)* | q104_M93. Other 3: "[q104_M93_full_list_other]"<br>*Select all that apply.*<br>*Question relevant when: string-length( ${q104_M93_full_list_other} ) > 0 and selected( ${section_one_skip_med} , '1')*<br>*Response constrained to: if(selected(., 6) or selected(., 99), count-selected(.) = 1, count-selected(.) >= 1)* | 1 Incomplete abortion<br>2 Sepsis<br>3 Shock<br>4 Cervical/vaginal lacerations<br>5 Vaginal/uterine perforation<br>6 Not used at this facility<br>99 Don't know |

| Field | Question | Answer |
|---|---|---|
| q104_M94_full_list *(required)* | q104_M94. Other 4: "[q104_M94_full_list_other]"<br>*Select all that apply.*<br>*Question relevant when: string-length( ${q104_M94_full_list_other} ) > 0 and selected( ${section_one_skip_med} , '1')*<br>*Response constrained to: if(selected(., 6) or selected(., 99), count-selected(.) = 1, count-selected(.) >= 1)* | 1 Incomplete abortion<br>2 Sepsis<br>3 Shock<br>4 Cervical/vaginal lacerations<br>5 Vaginal/uterine perforation<br>6 Not used at this facility<br>99 Don't know |
| q104_M95_full_list *(required)* | q104_M95. Other 5: "[q104_M95_full_list_other]"<br>*Select all that apply.*<br>*Question relevant when: string-length( ${q104_M95_full_list_other} ) > 0 and selected( ${section_one_skip_med} , '1')*<br>*Response constrained to: if(selected(., 6) or selected(., 99), count-selected(.) = 1, count-selected(.) >= 1)* | 1 Incomplete abortion<br>2 Sepsis<br>3 Shock<br>4 Cervical/vaginal lacerations<br>5 Vaginal/uterine perforation<br>6 Not used at this facility<br>99 Don't know |
| D. Medications - Full list (10)<br>*Group relevant when: selected( ${section_one_skip_med} , '1')* | | |
| q104_med_note10 | 104 CONTINUED. For each of the following medications and other medical products, can you tell me if the item is used for post abortion care at your facility? I'm going to ask about each of the five complication types separately. Is [ITEM] used for managing women with [COMPLICATION TYPE]? | |
| note_104_prost_utero | ***Prostaglandin, uterotonic, etc.*** | |
| q104_M96_full_list *(required)* | q104_M96. Misoprostol<br>*Select all that apply.*<br>*Response constrained to: if(selected(., 6) or selected(., 99), count-selected(.) = 1, count-selected(.) >= 1)* | 1 Incomplete abortion<br>2 Sepsis<br>3 Shock<br>4 Cervical/vaginal lacerations<br>5 Vaginal/uterine perforation<br>6 Not used at this facility<br>99 Don't know |
| q104_M97_full_list *(required)* | q104_M97. Ergometrine<br>*Select all that apply.*<br>*Response constrained to: if(selected(., 6) or selected(., 99), count-selected(.) = 1, count-selected(.) >= 1)* | 1 Incomplete abortion<br>2 Sepsis<br>3 Shock<br>4 Cervical/vaginal lacerations<br>5 Vaginal/uterine perforation<br>6 Not used at this facility<br>99 Don't know |
| q104_M98_full_list *(required)* | q104_M98. Methylergonovine Maleate (Methergine)<br>*Select all that apply.*<br>*Response constrained to: if(selected(., 6) or selected(., 99), count-selected(.) = 1, count-selected(.) >= 1)* | 1 Incomplete abortion<br>2 Sepsis<br>3 Shock<br>4 Cervical/vaginal lacerations<br>5 Vaginal/uterine perforation<br>6 Not used at this facility<br>99 Don't know |
| q104_M99_full_list *(required)* | q104_M99. Mifepristone<br>*Select all that apply.*<br>*Response constrained to: if(selected(., 6) or selected(., 99), count-selected(.) = 1, count-selected(.) >= 1)* | 1 Incomplete abortion<br>2 Sepsis<br>3 Shock<br>4 Cervical/vaginal lacerations<br>5 Vaginal/uterine perforation<br>6 Not used at this facility<br>99 Don't know |
| q104_M100_full_list *(required)* | q104_M100. Oxytocin<br>*Select all that apply.*<br>*Response constrained to: if(selected(., 6) or selected(., 99), count-selected(.) = 1, count-selected(.) >= 1)* | 1 Incomplete abortion<br>2 Sepsis<br>3 Shock<br>4 Cervical/vaginal lacerations<br>5 Vaginal/uterine perforation<br>6 Not used at this facility<br>99 Don't know |
| q104_uterotonic_other *(required)* | q104_(prostaglandin_etc)_Other. Are there any other prostaglandin or uterotonic drugs that we have not mentioned that are used for postabortion care at your facility?<br>*Question relevant when: selected( ${section_one_skip_med} , '1')* | 1 Yes<br>0 No<br>99 Don't know |
| D. Medications - Full list - specify (10 Other)<br>*Group relevant when: selected( ${q104_uterotonic_other} , '1') and selected( ${section_one_skip_med} , '1')* | | |
| note_104_uterotonic_other | Please list the "other" prostaglandin, uterotonic, etc. items here. | |

| Field | Question | Answer |
|---|---|---|
| q104_M101_full_list_other | q104_M101. Other 1. Please specify: | |
| q104_M102_full_list_other | q104_M102. Other 2. Please specify: | |
| q104_M103_full_list_other | q104_M103. Other 3. Please specify: | |
| q104_M104_full_list_other | q104_M104. Other 4. Please specify: | |
| q104_M105_full_list_other | q104_M105. Other 5. Please specify: | |
| D. Medications - Full list - usage (10 Other)<br>*Group relevant when: selected( ${q104_uterotonic_other} , '1') and selected( ${section_one_skip_med} , '1')* | | |
| note_104_uterotonic_other_b | For each "other" prostaglandin, uterotonic, etc. item, please tell me which of the five post abortion complication types it is used for. | |
| q104_M101_full_list *(required)* | q104_M101. Other 1: "[q104_M101_full_list_other]"<br>*Select all that apply.*<br>*Question relevant when: string-length( ${q104_M101_full_list_other} ) > 0 and selected( ${section_one_skip_med} , '1')*<br>*Response constrained to: if(selected(., 6) or selected(., 99), count-selected(.) = 1, count-selected(.) >= 1)* | 1 Incomplete abortion<br>2 Sepsis<br>3 Shock<br>4 Cervical/vaginal lacerations<br>5 Vaginal/uterine perforation<br>6 Not used at this facility<br>99 Don't know |
| q104_M102_full_list *(required)* | q104_M102. Other 2: "[q104_M102_full_list_other]"<br>*Select all that apply.*<br>*Question relevant when: string-length( ${q104_M102_full_list_other} ) > 0 and selected( ${section_one_skip_med} , '1')*<br>*Response constrained to: if(selected(., 6) or selected(., 99), count-selected(.) = 1, count-selected(.) >= 1)* | 1 Incomplete abortion<br>2 Sepsis<br>3 Shock<br>4 Cervical/vaginal lacerations<br>5 Vaginal/uterine perforation<br>6 Not used at this facility<br>99 Don't know |
| q104_M103_full_list *(required)* | q104_M103. Other 3: "[q104_M103_full_list_other]"<br>*Select all that apply.*<br>*Question relevant when: string-length( ${q104_M103_full_list_other} ) > 0 and selected( ${section_one_skip_med} , '1')*<br>*Response constrained to: if(selected(., 6) or selected(., 99), count-selected(.) = 1, count-selected(.) >= 1)* | 1 Incomplete abortion<br>2 Sepsis<br>3 Shock<br>4 Cervical/vaginal lacerations<br>5 Vaginal/uterine perforation<br>6 Not used at this facility<br>99 Don't know |
| q104_M104_full_list *(required)* | q104_M104. Other 4: "[q104_M104_full_list_other]"<br>*Select all that apply.*<br>*Question relevant when: string-length( ${q104_M104_full_list_other} ) > 0 and selected( ${section_one_skip_med} , '1')*<br>*Response constrained to: if(selected(., 6) or selected(., 99), count-selected(.) = 1, count-selected(.) >= 1)* | 1 Incomplete abortion<br>2 Sepsis<br>3 Shock<br>4 Cervical/vaginal lacerations<br>5 Vaginal/uterine perforation<br>6 Not used at this facility<br>99 Don't know |
| q104_M105_full_list *(required)* | q104_M105. Other 5: "[q104_M105_full_list_other]"<br>*Select all that apply.*<br>*Question relevant when: string-length( ${q104_M105_full_list_other} ) > 0 and selected( ${section_one_skip_med} , '1')*<br>*Response constrained to: if(selected(., 6) or selected(., 99), count-selected(.) = 1, count-selected(.) >= 1)* | 1 Incomplete abortion<br>2 Sepsis<br>3 Shock<br>4 Cervical/vaginal lacerations<br>5 Vaginal/uterine perforation<br>6 Not used at this facility<br>99 Don't know |
| D. Medications - Full list (11)<br>*Group relevant when: selected( ${section_one_skip_med} , '1')* | | |
| q104_med_note11 | 104 CONTINUED. For each of the following medications and other medical products, can you tell me if the item is used for post abortion care at your facility? I'm going to ask about each of the five complication types separately. Is [ITEM] used for managing women with [COMPLICATION TYPE]? | |
| note_104_vitamin | ***Vitamin, immunological, etc.*** | |
| q104_M106_full_list *(required)* | q104_M106. Ferrous sulphate (tablets)<br>*Select all that apply.*<br>*Response constrained to: if(selected(., 6) or selected(., 99), count-selected(.) = 1, count-selected(.) >= 1)* | 1 Incomplete abortion<br>2 Sepsis<br>3 Shock<br>4 Cervical/vaginal lacerations<br>5 Vaginal/uterine perforation<br>6 Not used at this facility<br>99 Don't know |

| Field | Question | Answer |
|---|---|---|
| q104_M107_full_list *(required)* | q104_M107. Ferrous sulphate + folic acid (Ferrotone capsules)<br>*Select all that apply.*<br>*Response constrained to: if(selected(., 6) or selected(., 99), count-selected(.) = 1, count-selected(.) >= 1)* | 1 Incomplete abortion<br>2 Sepsis<br>3 Shock<br>4 Cervical/vaginal lacerations<br>5 Vaginal/uterine perforation<br>6 Not used at this facility<br>99 Don't know |
| q104_M108_full_list *(required)* | q104_M108. Folic acid (Tablets)<br>*Select all that apply.*<br>*Response constrained to: if(selected(., 6) or selected(., 99), count-selected(.) = 1, count-selected(.) >= 1)* | 1 Incomplete abortion<br>2 Sepsis<br>3 Shock<br>4 Cervical/vaginal lacerations<br>5 Vaginal/uterine perforation<br>6 Not used at this facility<br>99 Don't know |
| q104_M109_full_list *(required)* | q104_M109. Serum anti-tetanus<br>*Select all that apply.*<br>*Response constrained to: if(selected(., 6) or selected(., 99), count-selected(.) = 1, count-selected(.) >= 1)* | 1 Incomplete abortion<br>2 Sepsis<br>3 Shock<br>4 Cervical/vaginal lacerations<br>5 Vaginal/uterine perforation<br>6 Not used at this facility<br>99 Don't know |
| q104_M110_full_list *(required)* | q104_M110. Anti D (Rh)-Immunoglobulin<br>*Select all that apply.*<br>*Response constrained to: if(selected(., 6) or selected(., 99), count-selected(.) = 1, count-selected(.) >= 1)* | 1 Incomplete abortion<br>2 Sepsis<br>3 Shock<br>4 Cervical/vaginal lacerations<br>5 Vaginal/uterine perforation<br>6 Not used at this facility<br>99 Don't know |
| q104_vitamin_other *(required)* | q104_(vitmain)_Other. Are there any other vitamins, vaccines, or other supplements that we have not mentioned that are used for postabortion care at your facility?<br>*Question relevant when: selected( ${section_one_skip_med} , '1')* | 1 Yes<br>0 No<br>99 Don't know |
| D. Medications - Full list - specify (11 Other)<br>*Group relevant when: selected( ${q104_vitamin_other} , '1') and selected( ${section_one_skip_med} , '1')* | | |
| note_104_vitamin_other | Please list the "other" vitamin, vaccines or other supplement items here. | |
| q104_M111_full_list_other | q104_M111. Other 1. Please specify: | |
| q104_M112_full_list_other | q104_M112. Other 2. Please specify: | |
| q104_M113_full_list_other | q104_M113. Other 3. Please specify: | |
| q104_M114_full_list_other | q104_M114. Other 4. Please specify: | |
| q104_M115_full_list_other | q104_M115. Other 5. Please specify: | |
| D. Medications - Full list - usage (11 Other)<br>*Group relevant when: selected( ${q104_vitamin_other} , '1') and selected( ${section_one_skip_med} , '1')* | | |
| note_104_vitamin_other_b | For each "other" vitamin, vaccines or other supplement item, please tell me which of the five post abortion complication types it is used for. | |
| q104_M111_full_list *(required)* | q104_M111. Other 1: "[q104_M111_full_list_other]"<br>*Select all that apply.*<br>*Question relevant when: string-length( ${q104_M111_full_list_other} ) > 0 and selected( ${section_one_skip_med} , '1')*<br>*Response constrained to: if(selected(., 6) or selected(., 99), count-selected(.) = 1, count-selected(.) >= 1)* | 1 Incomplete abortion<br>2 Sepsis<br>3 Shock<br>4 Cervical/vaginal lacerations<br>5 Vaginal/uterine perforation<br>6 Not used at this facility<br>99 Don't know |
| q104_M112_full_list *(required)* | q104_M112. Other 2: "[q104_M112_full_list_other]"<br>*Select all that apply.*<br>*Question relevant when: string-length( ${q104_M112_full_list_other} ) > 0 and selected( ${section_one_skip_med} , '1')*<br>*Response constrained to: if(selected(., 6) or selected(., 99), count-selected(.) = 1, count-selected(.) >= 1)* | 1 Incomplete abortion<br>2 Sepsis<br>3 Shock<br>4 Cervical/vaginal lacerations<br>5 Vaginal/uterine perforation<br>6 Not used at this facility<br>99 Don't know |

| Field | Question | Answer |
|---|---|---|
| q104_M113_full_list *(required)* | q104_M113. Other 3: "[q104_M113_full_list_other]"<br>*Select all that apply.*<br>*Question relevant when: string-length( ${q104_M113_full_list_other} ) > 0 and selected( ${section_one_skip_med} , '1')*<br>*Response constrained to: if(selected(., 6) or selected(., 99), count-selected(.) = 1, count-selected(.) >= 1)* | 1 Incomplete abortion<br>2 Sepsis<br>3 Shock<br>4 Cervical/vaginal lacerations<br>5 Vaginal/uterine perforation<br>6 Not used at this facility<br>99 Don't know |
| q104_M114_full_list *(required)* | q104_M114. Other 4: "[q104_M114_full_list_other]"<br>*Select all that apply.*<br>*Question relevant when: string-length( ${q104_M114_full_list_other} ) > 0 and selected( ${section_one_skip_med} , '1')*<br>*Response constrained to: if(selected(., 6) or selected(., 99), count-selected(.) = 1, count-selected(.) >= 1)* | 1 Incomplete abortion<br>2 Sepsis<br>3 Shock<br>4 Cervical/vaginal lacerations<br>5 Vaginal/uterine perforation<br>6 Not used at this facility<br>99 Don't know |
| q104_M115_full_list *(required)* | q104_M115. Other 5: "[q104_M115_full_list_other]"<br>*Select all that apply.*<br>*Question relevant when: string-length( ${q104_M115_full_list_other} ) > 0 and selected( ${section_one_skip_med} , '1')*<br>*Response constrained to: if(selected(., 6) or selected(., 99), count-selected(.) = 1, count-selected(.) >= 1)* | 1 Incomplete abortion<br>2 Sepsis<br>3 Shock<br>4 Cervical/vaginal lacerations<br>5 Vaginal/uterine perforation<br>6 Not used at this facility<br>99 Don't know |
| q104_meds_other *(required)* | q104_(other)_Other. Are there any medications or medical products (excluding contraception) that we have not mentioned that are used for postabortion care at your facility?<br>*Question relevant when: selected( ${section_one_skip_med} , '1')* | 1 Yes<br>0 No<br>99 Don't know |
| D. Medications - Full list - specify (12)<br>*Group relevant when: selected( ${q104_meds_other} , '1') and selected( ${section_one_skip_med} , '1')* | | |
| note_104_other_suggestion | Please list the "other" medications or medical product items (excluding contraception) here. | |
| note_104_Other | ***Other medication, any class*** | |
| q104_M116_full_list_other | q104_M116. Other 1. Please specify: | |
| q104_M117_full_list_other | q104_M117. Other 2. Please specify: | |
| q104_M118_full_list_other | q104_M118. Other 3. Please specify: | |
| q104_M119_full_list_other | q104_M119. Other 4. Please specify: | |
| q104_M120_full_list_other | q104_M120. Other 5. Please specify: | |
| D. Medications - Full list - usage (12)<br>*Group relevant when: selected( ${q104_meds_other} , '1') and selected( ${section_one_skip_med} , '1')* | | |
| note_104_other_suggestion_b | For each "other" medications or medical product items (excluding contraception), please tell me which of the five post abortion complication types it is used for. | |
| note_104_Other_b | ***Other medication, any class*** | |
| q104_M116_full_list *(required)* | q104_M116. Other 1: "[q104_M116_full_list_other]"<br>*Select all that apply.*<br>*Question relevant when: string-length( ${q104_M116_full_list_other} ) > 0 and selected( ${section_one_skip_med} , '1')*<br>*Response constrained to: if(selected(., 6) or selected(., 99), count-selected(.) = 1, count-selected(.) >= 1)* | 1 Incomplete abortion<br>2 Sepsis<br>3 Shock<br>4 Cervical/vaginal lacerations<br>5 Vaginal/uterine perforation<br>6 Not used at this facility<br>99 Don't know |
| q104_M117_full_list *(required)* | q104_M117. Other 2: "[q104_M117_full_list_other]"<br>*Select all that apply.*<br>*Question relevant when: string-length( ${q104_M117_full_list_other} ) > 0 and selected( ${section_one_skip_med} , '1')*<br>*Response constrained to: if(selected(., 6) or selected(., 99), count-selected(.) = 1, count-selected(.) >= 1)* | 1 Incomplete abortion<br>2 Sepsis<br>3 Shock<br>4 Cervical/vaginal lacerations<br>5 Vaginal/uterine perforation<br>6 Not used at this facility<br>99 Don't know |
| q104_M118_full_list *(required)* | q104_M118. Other 3: "[q104_M118_full_list_other]"<br>*Select all that apply.*<br>*Question relevant when: string-length( ${q104_M118_full_list_other} ) > 0 and selected( ${section_one_skip_med} , '1')*<br>*Response constrained to: if(selected(., 6) or selected(., 99), count-selected(.) = 1, count-selected(.) >= 1)* | 1 Incomplete abortion<br>2 Sepsis<br>3 Shock<br>4 Cervical/vaginal lacerations<br>5 Vaginal/uterine perforation<br>6 Not used at this facility<br>99 Don't know |

| Field | Question | Answer |
|---|---|---|
| q104_M119_full_list *(required)* | q104_M119. Other 4: "[q104_M119_full_list_other]"<br>*Select all that apply.*<br>*Question relevant when: string-length( ${q104_M119_full_list_other} ) > 0 and selected( ${section_one_skip_med} , '1')*<br>*Response constrained to: if(selected(., 6) or selected(., 99), count-selected(.) = 1, count-selected(.) >= 1)* | 1 Incomplete abortion<br>2 Sepsis<br>3 Shock<br>4 Cervical/vaginal lacerations<br>5 Vaginal/uterine perforation<br>6 Not used at this facility<br>99 Don't know |
| q104_M120_full_list *(required)* | q104_M120. Other 5: "[q104_M120_full_list_other]"<br>*Select all that apply.*<br>*Question relevant when: string-length( ${q104_M120_full_list_other} ) > 0 and selected( ${section_one_skip_med} , '1')*<br>*Response constrained to: if(selected(., 6) or selected(., 99), count-selected(.) = 1, count-selected(.) >= 1)* | 1 Incomplete abortion<br>2 Sepsis<br>3 Shock<br>4 Cervical/vaginal lacerations<br>5 Vaginal/uterine perforation<br>6 Not used at this facility<br>99 Don't know |
| group_section_two_intro | | |
| section2_start | **SECTION II. INCOMPLETE ABORTION - USAGE OF ALL ITEMS** | |
| section_two_skip_med | INTERVIEWER: WOULD YOU LIKE TO COMPLETE THIS SECTION NOW OR SKIP THIS SECTION AND RETURN TO IT LATER?<br>*You may need to skip if the participant has indicated that s/he cannot answer the questions in this section.* | 1 Do not skip, complete this section now.<br>2 Skip and come back to this section later. |
| group_section_two_introB<br>*Group relevant when: selected( ${section_two_skip_med} , '1')* | | |
| section2_start2 | In this section of the interview, we will review all of the items that you said are used for management of "uncomplicated" incomplete abortion. For each item that is used, I'm going to ask questions on how many women need it and how much of it is used. | |
| section2_start3 | INTERVIEWER: ENTER WHOLE NUMBERS OR DECIMALS. DO NOT TYPE PERCENT SIGNS. ENTER 999 FOR ANY THAT ARE UNKNOWN. | |
| <p><strong><em>D. Medications (1)</em></strong></p><br>*Group relevant when: selected( ${section_two_skip_med} , '1')* | | |
| q204_med_note1 | 204 For each of the following medications and other medical products that are used for incomplete abortion, can you tell me …? | |
| note_204_analgesic | ***Analgesic, anti-inflammatory, narcotic, etc.*** | |
| q204_M1 | ***q204_M1. Acetylsalicylic acid (aspirin)***<br>*Question relevant when: selected( ${q104_M1_full_list} , '1')* | |
| q204_M1a *(required)* | q204_M1a. What percent of patients require this item?<br>*Question relevant when: selected( ${q104_M1_full_list} , '1')*<br>*Response constrained to: .>0 and .<=100 or .=999* | |
| q204_M1b *(required)* | q204_M1b. How is the item administered, or given, to the patient?<br>*Question relevant when: selected( ${q104_M1_full_list} , '1')* | 1 Oral - solid (e.g. tablet)<br>2 Oral - liquid<br>3 Sublingual<br>4 Intravenous (IV)<br>5 Intramusculuar injection (IM)<br>6 Subcutaneous<br>7 Rectal<br>8 Inhaled (e.g. gas)<br>9 Inserted (e.g. vaginal pessaries)<br>10 Topical - cream<br>11 Topical - drops (e.g. eye drops)<br>12 Other<br>99 Don't know |

| Field | Question | Answer |
|---|---|---|
| q204_M1c *(required)* | q204_M1c. What is the item's smallest unit of measurement (mg, mcg, ml, g, etc)?<br>*Question relevant when: selected( ${q104_M1_full_list} , '1')* | microgram — microgram (mcg)<br>miligram — miligram (mg)<br>miligram_dissolved_in_liquid — miligram (mg) dissolved in liquid (e.g. 2 mg per 3 militers)<br>gram — gram (g)<br>militer — militer (ml)<br>centiliter — centiliter (cl)<br>litre — litre (l)<br>ml_of _solution — ml of solution (e.g. ml of 10% solution)<br>litre_of_solution — litre of solution (e.g. 1 litre of 5% solution)<br>cubic_meter — cubic meter (m^3)<br>other — other<br>dont_know — don't know |
| q204_M1c.1 | q204_M1c.1 If other unit of measurement or route of administration, specify here:<br>*Leave blank if not applicable*<br>*Question relevant when: selected( ${q104_M1_full_list} , '1')* | |
| q204_M1d *(required)* | q204_M1d. In total, how many units are used to treat a patient during all of her care?<br>*Remember to add all units! E.g. 5 days x 200 mg twice per day = 2,000mg*<br>*Question relevant when: selected( ${q104_M1_full_list} , '1')*<br>*Response constrained to: .>0* | |
| q204_M2 | ***q204_M2. Paracetamol***<br>*Question relevant when: selected( ${q104_M2_full_list} , '1')* | |
| q204_M2a *(required)* | q204_M2a. What percent of patients require this item?<br>*Question relevant when: selected( ${q104_M2_full_list} , '1')*<br>*Response constrained to: .>0 and .<=100 or .=999* | |
| q204_M2b *(required)* | q204_M2b. How is the item administered, or given, to the patient?<br>*Question relevant when: selected( ${q104_M2_full_list} , '1')* | 1 Oral - solid (e.g. tablet)<br>2 Oral - liquid<br>3 Sublingual<br>4 Intravenous (IV)<br>5 Intramusculuar injection (IM)<br>6 Subcutaneous<br>7 Rectal<br>8 Inhaled (e.g. gas)<br>9 Inserted (e.g. vaginal pessaries)<br>10 Topical - cream<br>11 Topical - drops (e.g. eye drops)<br>12 Other<br>99 Don't know |

| Field | Question | Answer |
|---|---|---|
| q204_M2c *(required)* | q204_M2c. What is the item's smallest unit of measurement (mg, mcg, ml, g, etc)?<br>*Question relevant when: selected( ${q104_M2_full_list} , '1')* | microgram — microgram (mcg)<br>miligram — miligram (mg)<br>miligram_dissolved_in_liquid — miligram (mg) dissolved in liquid (e.g. 2 mg per 3 militers)<br>gram — gram (g)<br>militer — militer (ml)<br>centiliter — centiliter (cl)<br>litre — litre (l)<br>ml_of _solution — ml of solution (e.g. ml of 10% solution)<br>litre_of_solution — litre of solution (e.g. 1 litre of 5% solution)<br>cubic_meter — cubic meter (m^3)<br>other — other<br>dont_know — don't know |
| q204_M2c.1 | q204_M2c.1 If other unit of measurement or route of administration, specify here:<br>*Leave blank if not applicable*<br>*Question relevant when: selected( ${q104_M2_full_list} , '1')* | |
| q204_M2d *(required)* | q204_M2d. In total, how many units are used to treat a patient during all of her care?<br>*Remember to add all units! E.g. 5 days x 200 mg twice per day = 2,000mg*<br>*Question relevant when: selected( ${q104_M2_full_list} , '1')*<br>*Response constrained to: .>0* | |
| q204_M3 | ***q204_M3. Pethidine hydrochloride***<br>*Question relevant when: selected( ${q104_M3_full_list} , '1')* | |
| q204_M3a *(required)* | q204_M3a. What percent of patients require this item?<br>*Question relevant when: selected( ${q104_M3_full_list} , '1')*<br>*Response constrained to: .>0 and .<=100 or .=999* | |
| q204_M3b *(required)* | q204_M3b. How is the item administered, or given, to the patient?<br>*Question relevant when: selected( ${q104_M3_full_list} , '1')* | 1 Oral - solid (e.g. tablet)<br>2 Oral - liquid<br>3 Sublingual<br>4 Intravenous (IV)<br>5 Intramusculuar injection (IM)<br>6 Subcutaneous<br>7 Rectal<br>8 Inhaled (e.g. gas)<br>9 Inserted (e.g. vaginal pessaries)<br>10 Topical - cream<br>11 Topical - drops (e.g. eye drops)<br>12 Other<br>99 Don't know |

| Field | Question | Answer |
|---|---|---|
| q204_M3c *(required)* | q204_M3c. What is the item's smallest unit of measurement (mg, mcg, ml, g, etc)?<br>*Question relevant when: selected( ${q104_M3_full_list} , '1')* | microgram: microgram (mcg)<br>miligram: miligram (mg)<br>miligram_dissolved_in_liquid: miligram (mg) dissolved in liquid (e.g. 2 mg per 3 militers)<br>gram: gram (g)<br>militer: militer (ml)<br>centiliter: centiliter (cl)<br>litre: litre (l)<br>ml_of _solution: ml of solution (e.g. ml of 10% solution)<br>litre_of_solution: litre of solution (e.g. 1 litre of 5% solution)<br>cubic_meter: cubic meter (m^3)<br>other: other<br>dont_know: don't know |
| q204_M3c.1 | q204_M3c.1 If other unit of measurement or route of administration, specify here:<br>*Leave blank if not applicable*<br>*Question relevant when: selected( ${q104_M3_full_list} , '1')* | |
| q204_M3d *(required)* | q204_M3d. In total, how many units are used to treat a patient during all of her care?<br>*Remember to add all units! E.g. 5 days x 200 mg twice per day = 2,000mg*<br>*Question relevant when: selected( ${q104_M3_full_list} , '1')*<br>*Response constrained to: .>0* | |
| q204_M4 | ***q204_M4. Tramadol Hydrochloride***<br>*Question relevant when: selected( ${q104_M4_full_list} , '1')* | |
| q204_M4a *(required)* | q204_M4a. What percent of patients require this item?<br>*Question relevant when: selected( ${q104_M4_full_list} , '1')*<br>*Response constrained to: .>0 and .<=100 or .=999* | |
| q204_M4b *(required)* | q204_M4b. How is the item administered, or given, to the patient?<br>*Question relevant when: selected( ${q104_M4_full_list} , '1')* | 1 Oral - solid (e.g. tablet)<br>2 Oral - liquid<br>3 Sublingual<br>4 Intravenous (IV)<br>5 Intramuscluar injection (IM)<br>6 Subcutaneous<br>7 Rectal<br>8 Inhaled (e.g. gas)<br>9 Inserted (e.g. vaginal pessaries)<br>10 Topical - cream<br>11 Topical - drops (e.g. eye drops)<br>12 Other<br>99 Don't know |

| Field | Question | Answer |
|---|---|---|
| q204_M4c *(required)* | q204_M4c. What is the item's smallest unit of measurement (mg, mcg, ml, g, etc)?<br>*Question relevant when: selected( ${q104_M4_full_list} , '1')* | microgram — microgram (mcg)<br>miligram — miligram (mg)<br>miligram_dissolved_in_liquid — miligram (mg) dissolved in liquid (e.g. 2 mg per 3 militers)<br>gram — gram (g)<br>militer — militer (ml)<br>centiliter — centiliter (cl)<br>litre — litre (l)<br>ml_of _solution — ml of solution (e.g. ml of 10% solution)<br>litre_of_solution — litre of solution (e.g. 1 litre of 5% solution)<br>cubic_meter — cubic meter (m^3)<br>other — other<br>dont_know — don't know |
| q204_M4c.1 | q204_M4c.1 If other unit of measurement or route of administration, specify here:<br>*Leave blank if not applicable*<br>*Question relevant when: selected( ${q104_M4_full_list} , '1')* | |
| q204_M4d *(required)* | q204_M4d. In total, how many units are used to treat a patient during all of her care?<br>*Remember to add all units! E.g. 5 days x 200 mg twice per day = 2,000mg*<br>*Question relevant when: selected( ${q104_M4_full_list} , '1')*<br>*Response constrained to: .>0* | |
| q204_M5 | ***q204_M5. Ibuprofen***<br>*Question relevant when: selected( ${q104_M5_full_list} , '1')* | |
| q204_M5a *(required)* | q204_M5a. What percent of patients require this item?<br>*Question relevant when: selected( ${q104_M5_full_list} , '1')*<br>*Response constrained to: .>0 and .<=100 or .=999* | |
| q204_M5b *(required)* | q204_M5b. How is the item administered, or given, to the patient?<br>*Question relevant when: selected( ${q104_M5_full_list} , '1')* | 1 Oral - solid (e.g. tablet)<br>2 Oral - liquid<br>3 Sublingual<br>4 Intravenous (IV)<br>5 Intramusculuar injection (IM)<br>6 Subcutaneous<br>7 Rectal<br>8 Inhaled (e.g. gas)<br>9 Inserted (e.g. vaginal pessaries)<br>10 Topical - cream<br>11 Topical - drops (e.g. eye drops)<br>12 Other<br>99 Don't know |

| Field | Question | Answer |
|---|---|---|
| q204_M5c *(required)* | q204_M5c. What is the item's smallest unit of measurement (mg, mcg, ml, g, etc)?<br>*Question relevant when: selected( ${q104_M5_full_list} , '1')* | microgram — microgram (mcg)<br>miligram — miligram (mg)<br>miligram_dissolved_in_liquid — miligram (mg) dissolved in liquid (e.g. 2 mg per 3 militers)<br>gram — gram (g)<br>militer — militer (ml)<br>centiliter — centiliter (cl)<br>litre — litre (l)<br>ml_of _solution — ml of solution (e.g. ml of 10% solution)<br>litre_of_solution — litre of solution (e.g. 1 litre of 5% solution)<br>cubic_meter — cubic meter (m^3)<br>other — other<br>dont_know — don't know |
| q204_M5c.1 | q204_M5c.1 If other unit of measurement or route of administration, specify here:<br>*Leave blank if not applicable*<br>*Question relevant when: selected( ${q104_M5_full_list} , '1')* | |
| q204_M5d *(required)* | q204_M5d. In total, how many units are used to treat a patient during all of her care?<br>*Remember to add all units! E.g. 5 days x 200 mg twice per day = 2,000mg*<br>*Question relevant when: selected( ${q104_M5_full_list} , '1')*<br>*Response constrained to: .>0* | |
| q204_M6 | ***q204_M6. Diclofenac***<br>*Question relevant when: selected( ${q104_M6_full_list} , '1')* | |
| q204_M6a *(required)* | q204_M6a. What percent of patients require this item?<br>*Question relevant when: selected( ${q104_M6_full_list} , '1')*<br>*Response constrained to: .>0 and .<=100 or .=999* | |
| q204_M6b *(required)* | q204_M6b. How is the item administered, or given, to the patient?<br>*Question relevant when: selected( ${q104_M6_full_list} , '1')* | 1 Oral - solid (e.g. tablet)<br>2 Oral - liquid<br>3 Sublingual<br>4 Intravenous (IV)<br>5 Intramuscular injection (IM)<br>6 Subcutaneous<br>7 Rectal<br>8 Inhaled (e.g. gas)<br>9 Inserted (e.g. vaginal pessaries)<br>10 Topical - cream<br>11 Topical - drops (e.g. eye drops)<br>12 Other<br>99 Don't know |

| Field | Question | Answer |
|---|---|---|
| q204_M6c *(required)* | q204_M6c. What is the item's smallest unit of measurement (mg, mcg, ml, g, etc)?<br>*Question relevant when: selected( ${q104_M6_full_list} , '1')* | microgram — microgram (mcg)<br>miligram — miligram (mg)<br>miligram_dissolved_in_liquid — miligram (mg) dissolved in liquid (e.g. 2 mg per 3 militers)<br>gram — gram (g)<br>militer — militer (ml)<br>centiliter — centiliter (cl)<br>litre — litre (l)<br>ml_of _solution — ml of solution (e.g. ml of 10% solution)<br>litre_of_solution — litre of solution (e.g. 1 litre of 5% solution)<br>cubic_meter — cubic meter (m^3)<br>other — other<br>dont_know — don't know |
| q204_M6c.1 | q204_M6c.1 If other unit of measurement or route of administration, specify here:<br>*Leave blank if not applicable*<br>*Question relevant when: selected( ${q104_M6_full_list} , '1')* | |
| q204_M6d *(required)* | q204_M6d. In total, how many units are used to treat a patient during all of her care?<br>*Remember to add all units! E.g. 5 days x 200 mg twice per day = 2,000mg*<br>*Question relevant when: selected( ${q104_M6_full_list} , '1')*<br>*Response constrained to: .>0* | |
| q204_M7 | ***q204_M7. Diclofenac sodium***<br>*Question relevant when: selected( ${q104_M7_full_list} , '1')* | |
| q204_M7a *(required)* | q204_M7a. What percent of patients require this item?<br>*Question relevant when: selected( ${q104_M7_full_list} , '1')*<br>*Response constrained to: .>0 and .<=100 or .=999* | |
| q204_M7b *(required)* | q204_M7b. How is the item administered, or given, to the patient?<br>*Question relevant when: selected( ${q104_M7_full_list} , '1')* | 1 Oral - solid (e.g. tablet)<br>2 Oral - liquid<br>3 Sublingual<br>4 Intravenous (IV)<br>5 Intramusculuar injection (IM)<br>6 Subcutaneous<br>7 Rectal<br>8 Inhaled (e.g. gas)<br>9 Inserted (e.g. vaginal pessaries)<br>10 Topical - cream<br>11 Topical - drops (e.g. eye drops)<br>12 Other<br>99 Don't know |

| Field | Question | Answer |
|---|---|---|
| q204_M7c *(required)* | q204_M7c. What is the item's smallest unit of measurement (mg, mcg, ml, g, etc)?<br>*Question relevant when: selected( ${q104_M7_full_list} , '1')* | \| \| microgram \| microgram (mcg) \|<br>\| \| miligram \| miligram (mg) \|<br>\| \| miligram_dissolved_in_liquid \| miligram (mg) dissolved in liquid (e.g. 2 mg per 3 militers) \|<br>\| \| gram \| gram (g) \|<br>\| \| militer \| militer (ml) \|<br>\| \| centiliter \| centiliter (cl) \|<br>\| \| litre \| litre (l) \|<br>\| \| ml_of _solution \| ml of solution (e.g. ml of 10% solution) \|<br>\| \| litre_of_solution \| litre of solution (e.g. 1 litre of 5% solution) \|<br>\| \| cubic_meter \| cubic meter (m^3) \|<br>\| \| other \| other \|<br>\| \| dont_know \| don't know \| |
| q204_M7c.1 | q204_M7c.1 If other unit of measurement or route of administration, specify here:<br>*Leave blank if not applicable*<br>*Question relevant when: selected( ${q104_M7_full_list} , '1')* | |
| q204_M7d *(required)* | q204_M7d. In total, how many units are used to treat a patient during all of her care?<br>*Remember to add all units! E.g. 5 days x 200 mg twice per day = 2,000mg*<br>*Question relevant when: selected( ${q104_M7_full_list} , '1')*<br>*Response constrained to: .>0* | |
| q204_M8 | ***q204_M8. Dypron (dipyrone), 250mg/ml (Metamizole)***<br>*Question relevant when: selected( ${q104_M8_full_list} , '1')* | |
| q204_M8a *(required)* | q204_M8a. What percent of patients require this item?<br>*Question relevant when: selected( ${q104_M8_full_list} , '1')*<br>*Response constrained to: .>0 and .<=100 or .=999* | |
| q204_M8b *(required)* | q204_M8b. How is the item administered, or given, to the patient?<br>*Question relevant when: selected( ${q104_M8_full_list} , '1')* | \| \| 1 \| Oral - solid (e.g. tablet) \|<br>\| \| 2 \| Oral - liquid \|<br>\| \| 3 \| Sublingual \|<br>\| \| 4 \| Intravenous (IV) \|<br>\| \| 5 \| Intramusculuar injection (IM) \|<br>\| \| 6 \| Subcutaneous \|<br>\| \| 7 \| Rectal \|<br>\| \| 8 \| Inhaled (e.g. gas) \|<br>\| \| 9 \| Inserted (e.g. vaginal pessaries) \|<br>\| \| 10 \| Topical - cream \|<br>\| \| 11 \| Topical - drops (e.g. eye drops) \|<br>\| \| 12 \| Other \|<br>\| \| 99 \| Don't know \| |

| Field | Question | Answer |
|---|---|---|
| q204_M8c *(required)* | q204_M8c. What is the item's smallest unit of measurement (mg, mcg, ml, g, etc)?<br>*Question relevant when: selected( ${q104_M8_full_list} , '1')* | \| \| microgram \| microgram (mcg) \|<br>\| \| miligram \| miligram (mg) \|<br>\| \| miligram_dissolved_in_liquid \| miligram (mg) dissolved in liquid (e.g. 2 mg per 3 militers) \|<br>\| \| gram \| gram (g) \|<br>\| \| militer \| militer (ml) \|<br>\| \| centiliter \| centiliter (cl) \|<br>\| \| litre \| litre (l) \|<br>\| \| ml_of _solution \| ml of solution (e.g. ml of 10% solution) \|<br>\| \| litre_of_solution \| litre of solution (e.g. 1 litre of 5% solution) \|<br>\| \| cubic_meter \| cubic meter (m^3) \|<br>\| \| other \| other \|<br>\| \| dont_know \| don't know \| |
| q204_M8c.1 | q204_M8c.1 If other unit of measurement or route of administration, specify here:<br>*Leave blank if not applicable*<br>*Question relevant when: selected( ${q104_M8_full_list} , '1')* | |
| q204_M8d *(required)* | q204_M8d. In total, how many units are used to treat a patient during all of her care?<br>*Remember to add all units! E.g. 5 days x 200 mg twice per day = 2,000mg*<br>*Question relevant when: selected( ${q104_M8_full_list} , '1')*<br>*Response constrained to: .>0* | |
| q204_M9 | ***q204_M9. Fentanyl citrate, 50 mcg/ml***<br>*Question relevant when: selected( ${q104_M9_full_list} , '1')* | |
| q204_M9a *(required)* | q204_M9a. What percent of patients require this item?<br>*Question relevant when: selected( ${q104_M9_full_list} , '1')*<br>*Response constrained to: .>0 and .<=100 or .=999* | |
| q204_M9b *(required)* | q204_M9b. How is the item administered, or given, to the patient?<br>*Question relevant when: selected( ${q104_M9_full_list} , '1')* | 1 Oral - solid (e.g. tablet)<br>2 Oral - liquid<br>3 Sublingual<br>4 Intravenous (IV)<br>5 Intramusculuar injection (IM)<br>6 Subcutaneous<br>7 Rectal<br>8 Inhaled (e.g. gas)<br>9 Inserted (e.g. vaginal pessaries)<br>10 Topical - cream<br>11 Topical - drops (e.g. eye drops)<br>12 Other<br>99 Don't know |

| Field | Question | Answer |
|---|---|---|
| q204_M9c *(required)* | q204_M9c. What is the item's smallest unit of measurement (mg, mcg, ml, g, etc)? *Question relevant when: selected( ${q104_M9_full_list} , '1')* | microgram — microgram (mcg)<br>miligram — miligram (mg)<br>miligram_dissolved_in_liquid — miligram (mg) dissolved in liquid (e.g. 2 mg per 3 militers)<br>gram — gram (g)<br>militer — militer (ml)<br>centiliter — centiliter (cl)<br>litre — litre (l)<br>ml_of _solution — ml of solution (e.g. ml of 10% solution)<br>litre_of_solution — litre of solution (e.g. 1 litre of 5% solution)<br>cubic_meter — cubic meter (m^3)<br>other — other<br>dont_know — don't know |
| q204_M9c.1 | q204_M9c.1 If other unit of measurement or route of administration, specify here: *Leave blank if not applicable* *Question relevant when: selected( ${q104_M9_full_list} , '1')* | |
| q204_M9d *(required)* | q204_M9d. In total, how many units are used to treat a patient during all of her care? *Remember to add all units! E.g. 5 days x 200 mg twice per day = 2,000mg* *Question relevant when: selected( ${q104_M9_full_list} , '1')* *Response constrained to: .>0* | |
| q204_M10 | ***q204_M10. Analgesic - other 1: "[q104_M10_full_list_other]"*** *Question relevant when: selected( ${q104_M10_full_list} , '1')* | |
| q204_M10a *(required)* | q204_M10a. What percent of patients require this item? *Question relevant when: selected( ${q104_M10_full_list} , '1')* *Response constrained to: .>0 and .<=100 or .=999* | |
| q204_M10b *(required)* | q204_M10b. How is the item administered, or given, to the patient? *Question relevant when: selected( ${q104_M10_full_list} , '1')* | 1 Oral - solid (e.g. tablet)<br>2 Oral - liquid<br>3 Sublingual<br>4 Intravenous (IV)<br>5 Intramusculuar injection (IM)<br>6 Subcutaneous<br>7 Rectal<br>8 Inhaled (e.g. gas)<br>9 Inserted (e.g. vaginal pessaries)<br>10 Topical - cream<br>11 Topical - drops (e.g. eye drops)<br>12 Other<br>99 Don't know |

| Field | Question | Answer |
|---|---|---|
| q204_M10c *(required)* | q204_M10c. What is the item's smallest unit of measurement (mg, mcg, ml, g, etc)?<br>*Question relevant when: selected( ${q104_M10_full_list} , '1')* | microgram — microgram (mcg)<br>miligram — miligram (mg)<br>miligram_dissolved_in_liquid — miligram (mg) dissolved in liquid (e.g. 2 mg per 3 militers)<br>gram — gram (g)<br>militer — militer (ml)<br>centiliter — centiliter (cl)<br>litre — litre (l)<br>ml_of _solution — ml of solution (e.g. ml of 10% solution)<br>litre_of_solution — litre of solution (e.g. 1 litre of 5% solution)<br>cubic_meter — cubic meter (m^3)<br>other — other<br>dont_know — don't know |
| q204_M10c.1 | q204_M10c.1 If other unit of measurement or route of administration, specify here:<br>*Leave blank if not applicable*<br>*Question relevant when: selected( ${q104_M10_full_list} , '1')* | |
| q204_M10d *(required)* | q204_M10d. In total, how many units are used to treat a patient during all of her care?<br>*Remember to add all units! E.g. 5 days x 200 mg twice per day = 2,000mg*<br>*Question relevant when: selected( ${q104_M10_full_list} , '1')*<br>*Response constrained to: .>0* | |
| q204_M11 | ***q204_M11. Analgesic - other 2: "[q104_M11_full_list_other]"***<br>*Question relevant when: selected( ${q104_M11_full_list} , '1')* | |
| q204_M11a *(required)* | q204_M11a. What percent of patients require this item?<br>*Question relevant when: selected( ${q104_M11_full_list} , '1')*<br>*Response constrained to: .>0 and .<=100 or .=999* | |
| q204_M11b *(required)* | q204_M11b. How is the item administered, or given, to the patient?<br>*Question relevant when: selected( ${q104_M11_full_list} , '1')* | 1 Oral - solid (e.g. tablet)<br>2 Oral - liquid<br>3 Sublingual<br>4 Intravenous (IV)<br>5 Intramuscular injection (IM)<br>6 Subcutaneous<br>7 Rectal<br>8 Inhaled (e.g. gas)<br>9 Inserted (e.g. vaginal pessaries)<br>10 Topical - cream<br>11 Topical - drops (e.g. eye drops)<br>12 Other<br>99 Don't know |

| Field | Question | Answer |
|---|---|---|
| q204_M11c *(required)* | q204_M11c. What is the item's smallest unit of measurement (mg, mcg, ml, g, etc)?<br>*Question relevant when: selected( ${q104_M11_full_list} , '1')* | \| \| microgram \| microgram (mcg) \|<br>\| \| miligram \| miligram (mg) \|<br>\| \| miligram_dissolved_in_liquid \| miligram (mg) dissolved in liquid (e.g. 2 mg per 3 militers) \|<br>\| \| gram \| gram (g) \|<br>\| \| militer \| militer (ml) \|<br>\| \| centiliter \| centiliter (cl) \|<br>\| \| litre \| litre (l) \|<br>\| \| ml_of _solution \| ml of solution (e.g. ml of 10% solution) \|<br>\| \| litre_of_solution \| litre of solution (e.g. 1 litre of 5% solution) \|<br>\| \| cubic_meter \| cubic meter (m^3) \|<br>\| \| other \| other \|<br>\| \| dont_know \| don't know \| |
| q204_M11c.1 | q204_M11c.1 If other unit of measurement or route of administration, specify here:<br>*Leave blank if not applicable*<br>*Question relevant when: selected( ${q104_M11_full_list} , '1')* | |
| q204_M11d *(required)* | q204_M11d. In total, how many units are used to treat a patient during all of her care?<br>*Remember to add all units! E.g. 5 days x 200 mg twice per day = 2,000mg*<br>*Question relevant when: selected( ${q104_M11_full_list} , '1')*<br>*Response constrained to: .>0* | |
| q204_M12 | ***q204_M12. Analgesic - other 3: "[q104_M12_full_list_other]"***<br>*Question relevant when: selected( ${q104_M12_full_list} , '1')* | |
| q204_M12a *(required)* | q204_M12a. What percent of patients require this item?<br>*Question relevant when: selected( ${q104_M12_full_list} , '1')*<br>*Response constrained to: .>0 and .<=100 or .=999* | |
| q204_M12b *(required)* | q204_M12b. How is the item administered, or given, to the patient?<br>*Question relevant when: selected( ${q104_M12_full_list} , '1')* | \| \| 1 \| Oral - solid (e.g. tablet) \|<br>\| \| 2 \| Oral - liquid \|<br>\| \| 3 \| Sublingual \|<br>\| \| 4 \| Intravenous (IV) \|<br>\| \| 5 \| Intramusculuar injection (IM) \|<br>\| \| 6 \| Subcutaneous \|<br>\| \| 7 \| Rectal \|<br>\| \| 8 \| Inhaled (e.g. gas) \|<br>\| \| 9 \| Inserted (e.g. vaginal pessaries) \|<br>\| \| 10 \| Topical - cream \|<br>\| \| 11 \| Topical - drops (e.g. eye drops) \|<br>\| \| 12 \| Other \|<br>\| \| 99 \| Don't know \| |

| Field | Question | Answer |
|---|---|---|
| q204_M12c *(required)* | q204_M12c. What is the item's smallest unit of measurement (mg, mcg, ml, g, etc)?<br>*Question relevant when: selected( ${q104_M12_full_list} , '1')* | microgram — microgram (mcg)<br>miligram — miligram (mg)<br>miligram_dissolved_in_liquid — miligram (mg) dissolved in liquid (e.g. 2 mg per 3 militers)<br>gram — gram (g)<br>militer — militer (ml)<br>centiliter — centiliter (cl)<br>litre — litre (l)<br>ml_of _solution — ml of solution (e.g. ml of 10% solution)<br>litre_of_solution — litre of solution (e.g. 1 litre of 5% solution)<br>cubic_meter — cubic meter (m^3)<br>other — other<br>dont_know — don't know |
| q204_M12c.1 | q204_M12c.1 If other unit of measurement or route of administration, specify here:<br>*Leave blank if not applicable*<br>*Question relevant when: selected( ${q104_M12_full_list} , '1')* | |
| q204_M12d *(required)* | q204_M12d. In total, how many units are used to treat a patient during all of her care?<br>*Remember to add all units! E.g. 5 days x 200 mg twice per day = 2,000mg*<br>*Question relevant when: selected( ${q104_M12_full_list} , '1')*<br>*Response constrained to: .>0* | |
| q204_M13 | ***q204_M13. Analgesic - other 4: "[q104_M13_full_list_other]"***<br>*Question relevant when: selected( ${q104_M13_full_list} , '1')* | |
| q204_M13a *(required)* | q204_M13a. What percent of patients require this item?<br>*Question relevant when: selected( ${q104_M13_full_list} , '1')*<br>*Response constrained to: .>0 and .<=100 or .=999* | |
| q204_M13b *(required)* | q204_M13b. How is the item administered, or given, to the patient?<br>*Question relevant when: selected( ${q104_M13_full_list} , '1')* | 1 Oral - solid (e.g. tablet)<br>2 Oral - liquid<br>3 Sublingual<br>4 Intravenous (IV)<br>5 Intramusculuar injection (IM)<br>6 Subcutaneous<br>7 Rectal<br>8 Inhaled (e.g. gas)<br>9 Inserted (e.g. vaginal pessaries)<br>10 Topical - cream<br>11 Topical - drops (e.g. eye drops)<br>12 Other<br>99 Don't know |

| Field | Question | Answer |
|---|---|---|
| q204_M13c *(required)* | q204_M13c. What is the item's smallest unit of measurement (mg, mcg, ml, g, etc)?<br>*Question relevant when: selected( ${q104_M13_full_list} , '1')* | microgram — microgram (mcg)<br>miligram — miligram (mg)<br>miligram_dissolved_in_liquid — miligram (mg) dissolved in liquid (e.g. 2 mg per 3 militers)<br>gram — gram (g)<br>militer — militer (ml)<br>centiliter — centiliter (cl)<br>litre — litre (l)<br>ml_of _solution — ml of solution (e.g. ml of 10% solution)<br>litre_of_solution — litre of solution (e.g. 1 litre of 5% solution)<br>cubic_meter — cubic meter (m^3)<br>other — other<br>dont_know — don't know |
| q204_M13c.1 | q204_M13c.1 If other unit of measurement or route of administration, specify here:<br>*Leave blank if not applicable*<br>*Question relevant when: selected( ${q104_M13_full_list} , '1')* | |
| q204_M13d *(required)* | q204_M13d. In total, how many units are used to treat a patient during all of her care?<br>*Remember to add all units! E.g. 5 days x 200 mg twice per day = 2,000mg*<br>*Question relevant when: selected( ${q104_M13_full_list} , '1')*<br>*Response constrained to: .>0* | |
| q204_M14 | ***q204_M14. Analgesic - other 5: "[q104_M14_full_list_other]"***<br>*Question relevant when: selected( ${q104_M14_full_list} , '1')* | |
| q204_M14a *(required)* | q204_M14a. What percent of patients require this item?<br>*Question relevant when: selected( ${q104_M14_full_list} , '1')*<br>*Response constrained to: .>0 and .<=100 or .=999* | |
| q204_M14b *(required)* | q204_M14b. How is the item administered, or given, to the patient?<br>*Question relevant when: selected( ${q104_M14_full_list} , '1')* | 1 Oral - solid (e.g. tablet)<br>2 Oral - liquid<br>3 Sublingual<br>4 Intravenous (IV)<br>5 Intramusculuar injection (IM)<br>6 Subcutaneous<br>7 Rectal<br>8 Inhaled (e.g. gas)<br>9 Inserted (e.g. vaginal pessaries)<br>10 Topical - cream<br>11 Topical - drops (e.g. eye drops)<br>12 Other<br>99 Don't know |

| Field | Question | Answer |
|---|---|---|
| q204_M14c *(required)* | q204_M14c. What is the item's smallest unit of measurement (mg, mcg, ml, g, etc)?<br>*Question relevant when: selected( ${q104_M14_full_list} , '1')* | microgram: microgram (mcg)<br>miligram: miligram (mg)<br>miligram_dissolved_in_liquid: miligram (mg) dissolved in liquid (e.g. 2 mg per 3 militers)<br>gram: gram (g)<br>militer: militer (ml)<br>centiliter: centiliter (cl)<br>litre: litre (l)<br>ml_of _solution: ml of solution (e.g. ml of 10% solution)<br>litre_of_solution: litre of solution (e.g. 1 litre of 5% solution)<br>cubic_meter: cubic meter (m^3)<br>other: other<br>dont_know: don't know |
| q204_M14c.1 | q204_M14c.1 If other unit of measurement or route of administration, specify here:<br>*Leave blank if not applicable*<br>*Question relevant when: selected( ${q104_M14_full_list} , '1')* | |
| q204_M14d *(required)* | q204_M14d. In total, how many units are used to treat a patient during all of her care?<br>*Remember to add all units! E.g. 5 days x 200 mg twice per day = 2,000mg*<br>*Question relevant when: selected( ${q104_M14_full_list} , '1')*<br>*Response constrained to: .>0* | |
| <p><strong><em>D. Medications (2)</em></strong></p><br>*Group relevant when: selected( ${section_two_skip_med} , '1')* | | |
| q204_med_note2 | 204 For each of the following medications and other medical products that are used for incomplete abortion, can you tell me …? | |
| note_204_anesthetic | ***Anesthetic - local, general, etc. (and oxygen)*** | |
| q204_M15 | ***q204_M15. Halothane***<br>*Question relevant when: selected( ${q104_M15_full_list} , '1')* | |
| q204_M15a *(required)* | q204_M15a. What percent of patients require this item?<br>*Question relevant when: selected( ${q104_M15_full_list} , '1')*<br>*Response constrained to: .>0 and .<=100 or .=999* | |
| q204_M15b *(required)* | q204_M15b. How is the item administered, or given, to the patient?<br>*Question relevant when: selected( ${q104_M15_full_list} , '1')* | 1 Oral - solid (e.g. tablet)<br>2 Oral - liquid<br>3 Sublingual<br>4 Intravenous (IV)<br>5 Intramusculuar injection (IM)<br>6 Subcutaneous<br>7 Rectal<br>8 Inhaled (e.g. gas)<br>9 Inserted (e.g. vaginal pessaries)<br>10 Topical - cream<br>11 Topical - drops (e.g. eye drops)<br>12 Other<br>99 Don't know |

| Field | Question | Answer |
|---|---|---|
| q204_M15c *(required)* | q204_M15c. What is the item's smallest unit of measurement (mg, mcg, ml, g, etc)?<br>*Question relevant when: selected( ${q104_M15_full_list} , '1')* | microgram — microgram (mcg)<br>miligram — miligram (mg)<br>miligram_dissolved_in_liquid — miligram (mg) dissolved in liquid (e.g. 2 mg per 3 militers)<br>gram — gram (g)<br>militer — militer (ml)<br>centiliter — centiliter (cl)<br>litre — litre (l)<br>ml_of _solution — ml of solution (e.g. ml of 10% solution)<br>litre_of_solution — litre of solution (e.g. 1 litre of 5% solution)<br>cubic_meter — cubic meter (m^3)<br>other — other<br>dont_know — don't know |
| q204_M15c.1 | q204_M15c.1 If other unit of measurement or route of administration, specify here:<br>*Leave blank if not applicable*<br>*Question relevant when: selected( ${q104_M15_full_list} , '1')* | |
| q204_M15d *(required)* | q204_M15d. In total, how many units are used to treat a patient during all of her care?<br>*Remember to add all units! E.g. 5 days x 200 mg twice per day = 2,000mg*<br>*Question relevant when: selected( ${q104_M15_full_list} , '1')*<br>*Response constrained to: .>0* | |
| q204_M16 | ***q204_M16. Ketamine (Ketalar), 50mg/ml***<br>*Question relevant when: selected( ${q104_M16_full_list} , '1')* | |
| q204_M16a *(required)* | q204_M16a. What percent of patients require this item?<br>*Question relevant when: selected( ${q104_M16_full_list} , '1')*<br>*Response constrained to: .>0 and .<=100 or .=999* | |
| q204_M16b *(required)* | q204_M16b. How is the item administered, or given, to the patient?<br>*Question relevant when: selected( ${q104_M16_full_list} , '1')* | 1 Oral - solid (e.g. tablet)<br>2 Oral - liquid<br>3 Sublingual<br>4 Intravenous (IV)<br>5 Intramusculuar injection (IM)<br>6 Subcutaneous<br>7 Rectal<br>8 Inhaled (e.g. gas)<br>9 Inserted (e.g. vaginal pessaries)<br>10 Topical - cream<br>11 Topical - drops (e.g. eye drops)<br>12 Other<br>99 Don't know |

| Field | Question | Answer |
|---|---|---|
| q204_M16c *(required)* | q204_M16c. What is the item's smallest unit of measurement (mg, mcg, ml, g, etc)?<br>*Question relevant when: selected( ${q104_M16_full_list} , '1')* | microgram — microgram (mcg)<br>miligram — miligram (mg)<br>miligram_dissolved_in_liquid — miligram (mg) dissolved in liquid (e.g. 2 mg per 3 militers)<br>gram — gram (g)<br>militer — militer (ml)<br>centiliter — centiliter (cl)<br>litre — litre (l)<br>ml_of _solution — ml of solution (e.g. ml of 10% solution)<br>litre_of_solution — litre of solution (e.g. 1 litre of 5% solution)<br>cubic_meter — cubic meter (m^3)<br>other — other<br>dont_know — don't know |
| q204_M16c.1 | q204_M16c.1 If other unit of measurement or route of administration, specify here:<br>*Leave blank if not applicable*<br>*Question relevant when: selected( ${q104_M16_full_list} , '1')* | |
| q204_M16d *(required)* | q204_M16d. In total, how many units are used to treat a patient during all of her care?<br>*Remember to add all units! E.g. 5 days x 200 mg twice per day = 2,000mg*<br>*Question relevant when: selected( ${q104_M16_full_list} , '1')*<br>*Response constrained to: .>0* | |
| q204_M17 | ***q204_M17. Propofol (Diprivan), 10mg/ml***<br>*Question relevant when: selected( ${q104_M17_full_list} , '1')* | |
| q204_M17a *(required)* | q204_M17a. What percent of patients require this item?<br>*Question relevant when: selected( ${q104_M17_full_list} , '1')*<br>*Response constrained to: .>0 and .<=100 or .=999* | |
| q204_M17b *(required)* | q204_M17b. How is the item administered, or given, to the patient?<br>*Question relevant when: selected( ${q104_M17_full_list} , '1')* | 1 Oral - solid (e.g. tablet)<br>2 Oral - liquid<br>3 Sublingual<br>4 Intravenous (IV)<br>5 Intramusculuar injection (IM)<br>6 Subcutaneous<br>7 Rectal<br>8 Inhaled (e.g. gas)<br>9 Inserted (e.g. vaginal pessaries)<br>10 Topical - cream<br>11 Topical - drops (e.g. eye drops)<br>12 Other<br>99 Don't know |

| Field | Question | Answer |
|---|---|---|
| q204_M17c *(required)* | q204_M17c. What is the item's smallest unit of measurement (mg, mcg, ml, g, etc)?<br>*Question relevant when: selected( ${q104_M17_full_list} , '1')* | microgram — microgram (mcg)<br>miligram — miligram (mg)<br>miligram_dissolved_in_liquid — miligram (mg) dissolved in liquid (e.g. 2 mg per 3 militers)<br>gram — gram (g)<br>militer — militer (ml)<br>centiliter — centiliter (cl)<br>litre — litre (l)<br>ml_of _solution — ml of solution (e.g. ml of 10% solution)<br>litre_of_solution — litre of solution (e.g. 1 litre of 5% solution)<br>cubic_meter — cubic meter (m^3)<br>other — other<br>dont_know — don't know |
| q204_M17c.1 | q204_M17c.1 If other unit of measurement or route of administration, specify here:<br>*Leave blank if not applicable*<br>*Question relevant when: selected( ${q104_M17_full_list} , '1')* | |
| q204_M17d *(required)* | q204_M17d. In total, how many units are used to treat a patient during all of her care?<br>*Remember to add all units! E.g. 5 days x 200 mg twice per day = 2,000mg*<br>*Question relevant when: selected( ${q104_M17_full_list} , '1')*<br>*Response constrained to: .>0* | |
| q204_M18 | ***q204_M18. Suxamethonium chloride***<br>*Question relevant when: selected( ${q104_M18_full_list} , '1')* | |
| q204_M18a *(required)* | q204_M18a. What percent of patients require this item?<br>*Question relevant when: selected( ${q104_M18_full_list} , '1')*<br>*Response constrained to: .>0 and .<=100 or .=999* | |
| q204_M18b *(required)* | q204_M18b. How is the item administered, or given, to the patient?<br>*Question relevant when: selected( ${q104_M18_full_list} , '1')* | 1 Oral - solid (e.g. tablet)<br>2 Oral - liquid<br>3 Sublingual<br>4 Intravenous (IV)<br>5 Intramusculuar injection (IM)<br>6 Subcutaneous<br>7 Rectal<br>8 Inhaled (e.g. gas)<br>9 Inserted (e.g. vaginal pessaries)<br>10 Topical - cream<br>11 Topical - drops (e.g. eye drops)<br>12 Other<br>99 Don't know |

| Field | Question | Answer |
|---|---|---|
| q204_M18c *(required)* | q204_M18c. What is the item's smallest unit of measurement (mg, mcg, ml, g, etc)?<br>*Question relevant when: selected( ${q104_M18_full_list} , '1')* | <table><tr><td></td><td>microgram</td><td>microgram (mcg)</td></tr><tr><td></td><td>miligram</td><td>miligram (mg)</td></tr><tr><td></td><td>miligram_dissolved_in_liquid</td><td>miligram (mg) dissolved in liquid (e.g. 2 mg per 3 militers)</td></tr><tr><td></td><td>gram</td><td>gram (g)</td></tr><tr><td></td><td>militer</td><td>militer (ml)</td></tr><tr><td></td><td>centiliter</td><td>centiliter (cl)</td></tr><tr><td></td><td>litre</td><td>litre (l)</td></tr><tr><td></td><td>ml_of _solution</td><td>ml of solution (e.g. ml of 10% solution)</td></tr><tr><td></td><td>litre_of_solution</td><td>litre of solution (e.g. 1 litre of 5% solution)</td></tr><tr><td></td><td>cubic_meter</td><td>cubic meter (m^3)</td></tr><tr><td></td><td>other</td><td>other</td></tr><tr><td></td><td>dont_know</td><td>don't know</td></tr></table> |
| q204_M18c.1 | q204_M18c.1 If other unit of measurement or route of administration, specify here:<br>*Leave blank if not applicable*<br>*Question relevant when: selected( ${q104_M18_full_list} , '1')* | |
| q204_M18d *(required)* | q204_M18d. In total, how many units are used to treat a patient during all of her care?<br>*Remember to add all units! E.g. 5 days x 200 mg twice per day = 2,000mg*<br>*Question relevant when: selected( ${q104_M18_full_list} , '1')*<br>*Response constrained to: .>0* | |
| q204_M19 | ***q204_M19. Thiopental sodium (Intraval)***<br>*Question relevant when: selected( ${q104_M19_full_list} , '1')* | |
| q204_M19a *(required)* | q204_M19a. What percent of patients require this item?<br>*Question relevant when: selected( ${q104_M19_full_list} , '1')*<br>*Response constrained to: .>0 and .<=100 or .=999* | |
| q204_M19b *(required)* | q204_M19b. How is the item administered, or given, to the patient?<br>*Question relevant when: selected( ${q104_M19_full_list} , '1')* | <table><tr><td>1</td><td>Oral - solid (e.g. tablet)</td></tr><tr><td>2</td><td>Oral - liquid</td></tr><tr><td>3</td><td>Sublingual</td></tr><tr><td>4</td><td>Intravenous (IV)</td></tr><tr><td>5</td><td>Intramusculuar injection (IM)</td></tr><tr><td>6</td><td>Subcutaneous</td></tr><tr><td>7</td><td>Rectal</td></tr><tr><td>8</td><td>Inhaled (e.g. gas)</td></tr><tr><td>9</td><td>Inserted (e.g. vaginal pessaries)</td></tr><tr><td>10</td><td>Topical - cream</td></tr><tr><td>11</td><td>Topical - drops (e.g. eye drops)</td></tr><tr><td>12</td><td>Other</td></tr><tr><td>99</td><td>Don't know</td></tr></table> |

| Field | Question | Answer |
|---|---|---|
| q204_M19c *(required)* | q204_M19c. What is the item's smallest unit of measurement (mg, mcg, ml, g, etc)?<br>*Question relevant when: selected( ${q104_M19_full_list} , '1')* | microgram — microgram (mcg)<br>miligram — miligram (mg)<br>miligram_dissolved_in_liquid — miligram (mg) dissolved in liquid (e.g. 2 mg per 3 militers)<br>gram — gram (g)<br>militer — militer (ml)<br>centiliter — centiliter (cl)<br>litre — litre (l)<br>ml_of _solution — ml of solution (e.g. ml of 10% solution)<br>litre_of_solution — litre of solution (e.g. 1 litre of 5% solution)<br>cubic_meter — cubic meter (m^3)<br>other — other<br>dont_know — don't know |
| q204_M19c.1 | q204_M19c.1 If other unit of measurement or route of administration, specify here:<br>*Leave blank if not applicable*<br>*Question relevant when: selected( ${q104_M19_full_list} , '1')* | |
| q204_M19d *(required)* | q204_M19d. In total, how many units are used to treat a patient during all of her care?<br>*Remember to add all units! E.g. 5 days x 200 mg twice per day = 2,000mg*<br>*Question relevant when: selected( ${q104_M19_full_list} , '1')*<br>*Response constrained to: .>0* | |
| q204_M20 | ***q204_M20. Oxygen***<br>*Question relevant when: selected( ${q104_M20_full_list} , '1')* | |
| q204_M20a *(required)* | q204_M20a. What percent of patients require this item?<br>*Question relevant when: selected( ${q104_M20_full_list} , '1')*<br>*Response constrained to: .>0 and .<=100 or .=999* | |
| q204_M20b *(required)* | q204_M20b. How is the item administered, or given, to the patient?<br>*Question relevant when: selected( ${q104_M20_full_list} , '1')* | 1 Oral - solid (e.g. tablet)<br>2 Oral - liquid<br>3 Sublingual<br>4 Intravenous (IV)<br>5 Intramusculuar injection (IM)<br>6 Subcutaneous<br>7 Rectal<br>8 Inhaled (e.g. gas)<br>9 Inserted (e.g. vaginal pessaries)<br>10 Topical - cream<br>11 Topical - drops (e.g. eye drops)<br>12 Other<br>99 Don't know |

| Field | Question | Answer |
|---|---|---|
| q204_M20c *(required)* | q204_M20c. What is the item's smallest unit of measurement (mg, mcg, ml, g, etc)?<br>*Question relevant when: selected( ${q104_M20_full_list} , '1')* | microgram — microgram (mcg)<br>miligram — miligram (mg)<br>miligram_dissolved_in_liquid — miligram (mg) dissolved in liquid (e.g. 2 mg per 3 militers)<br>gram — gram (g)<br>militer — militer (ml)<br>centiliter — centiliter (cl)<br>litre — litre (l)<br>ml_of _solution — ml of solution (e.g. ml of 10% solution)<br>litre_of_solution — litre of solution (e.g. 1 litre of 5% solution)<br>cubic_meter — cubic meter (m^3)<br>other — other<br>dont_know — don't know |
| q204_M20c.1 | q204_M20c.1 If other unit of measurement or route of administration, specify here:<br>*Leave blank if not applicable*<br>*Question relevant when: selected( ${q104_M20_full_list} , '1')* | |
| q204_M20d *(required)* | q204_M20d. In total, how many units are used to treat a patient during all of her care?<br>*Remember to add all units! E.g. 5 days x 200 mg twice per day = 2,000mg*<br>*Question relevant when: selected( ${q104_M20_full_list} , '1')*<br>*Response constrained to: .>0* | |
| q204_M21 | ***q204_M21. Bupivacaine HCL 0.5% (Marcaine)***<br>*Question relevant when: selected( ${q104_M21_full_list} , '1')* | |
| q204_M21a *(required)* | q204_M21a. What percent of patients require this item?<br>*Question relevant when: selected( ${q104_M21_full_list} , '1')*<br>*Response constrained to: .>0 and .<=100 or .=999* | |
| q204_M21b *(required)* | q204_M21b. How is the item administered, or given, to the patient?<br>*Question relevant when: selected( ${q104_M21_full_list} , '1')* | 1 Oral - solid (e.g. tablet)<br>2 Oral - liquid<br>3 Sublingual<br>4 Intravenous (IV)<br>5 Intramusculuar injection (IM)<br>6 Subcutaneous<br>7 Rectal<br>8 Inhaled (e.g. gas)<br>9 Inserted (e.g. vaginal pessaries)<br>10 Topical - cream<br>11 Topical - drops (e.g. eye drops)<br>12 Other<br>99 Don't know |

| Field | Question | Answer |
|---|---|---|
| q204_M21c *(required)* | q204_M21c. What is the item's smallest unit of measurement (mg, mcg, ml, g, etc)?<br>*Question relevant when: selected( ${q104_M21_full_list} , '1')* | microgram — microgram (mcg)<br>miligram — miligram (mg)<br>miligram_dissolved_in_liquid — miligram (mg) dissolved in liquid (e.g. 2 mg per 3 militers)<br>gram — gram (g)<br>militer — militer (ml)<br>centiliter — centiliter (cl)<br>litre — litre (l)<br>ml_of _solution — ml of solution (e.g. ml of 10% solution)<br>litre_of_solution — litre of solution (e.g. 1 litre of 5% solution)<br>cubic_meter — cubic meter (m^3)<br>other — other<br>dont_know — don't know |
| q204_M21c.1 | q204_M21c.1 If other unit of measurement or route of administration, specify here:<br>*Leave blank if not applicable*<br>*Question relevant when: selected( ${q104_M21_full_list} , '1')* | |
| q204_M21d *(required)* | q204_M21d. In total, how many units are used to treat a patient during all of her care?<br>*Remember to add all units! E.g. 5 days x 200 mg twice per day = 2,000mg*<br>*Question relevant when: selected( ${q104_M21_full_list} , '1')*<br>*Response constrained to: .>0* | |
| q204_M22 | ***q204_M22. Lidocaine HCl 2% + epinephrin 1:100,000***<br>*Question relevant when: selected( ${q104_M22_full_list} , '1')* | |
| q204_M22a *(required)* | q204_M22a. What percent of patients require this item?<br>*Question relevant when: selected( ${q104_M22_full_list} , '1')*<br>*Response constrained to: .>0 and .<=100 or .=999* | |
| q204_M22b *(required)* | q204_M22b. How is the item administered, or given, to the patient?<br>*Question relevant when: selected( ${q104_M22_full_list} , '1')* | 1 Oral - solid (e.g. tablet)<br>2 Oral - liquid<br>3 Sublingual<br>4 Intravenous (IV)<br>5 Intramusculuar injection (IM)<br>6 Subcutaneous<br>7 Rectal<br>8 Inhaled (e.g. gas)<br>9 Inserted (e.g. vaginal pessaries)<br>10 Topical - cream<br>11 Topical - drops (e.g. eye drops)<br>12 Other<br>99 Don't know |

| Field | Question | Answer |
|---|---|---|
| q204_M22c *(required)* | q204_M22c. What is the item's smallest unit of measurement (mg, mcg, ml, g, etc)?<br>*Question relevant when: selected( ${q104_M22_full_list} , '1')* | microgram — microgram (mcg)<br>miligram — miligram (mg)<br>miligram_dissolved_in_liquid — miligram (mg) dissolved in liquid (e.g. 2 mg per 3 militers)<br>gram — gram (g)<br>militer — militer (ml)<br>centiliter — centiliter (cl)<br>litre — litre (l)<br>ml_of _solution — ml of solution (e.g. ml of 10% solution)<br>litre_of_solution — litre of solution (e.g. 1 litre of 5% solution)<br>cubic_meter — cubic meter (m^3)<br>other — other<br>dont_know — don't know |
| q204_M22c.1 | q204_M22c.1 If other unit of measurement or route of administration, specify here:<br>*Leave blank if not applicable*<br>*Question relevant when: selected( ${q104_M22_full_list} , '1')* | |
| q204_M22d *(required)* | q204_M22d. In total, how many units are used to treat a patient during all of her care?<br>*Remember to add all units! E.g. 5 days x 200 mg twice per day = 2,000mg*<br>*Question relevant when: selected( ${q104_M22_full_list} , '1')*<br>*Response constrained to: .>0* | |
| q204_M23 | ***q204_M23. Lidocaine HCl 5% + dextrose 7.5%***<br>*Question relevant when: selected( ${q104_M23_full_list} , '1')* | |
| q204_M23a *(required)* | q204_M23a. What percent of patients require this item?<br>*Question relevant when: selected( ${q104_M23_full_list} , '1')*<br>*Response constrained to: .>0 and .<=100 or .=999* | |
| q204_M23b *(required)* | q204_M23b. How is the item administered, or given, to the patient?<br>*Question relevant when: selected( ${q104_M23_full_list} , '1')* | 1 Oral - solid (e.g. tablet)<br>2 Oral - liquid<br>3 Sublingual<br>4 Intravenous (IV)<br>5 Intramuscular injection (IM)<br>6 Subcutaneous<br>7 Rectal<br>8 Inhaled (e.g. gas)<br>9 Inserted (e.g. vaginal pessaries)<br>10 Topical - cream<br>11 Topical - drops (e.g. eye drops)<br>12 Other<br>99 Don't know |

| Field | Question | Answer |
|---|---|---|
| q204_M23c *(required)* | q204_M23c. What is the item's smallest unit of measurement (mg, mcg, ml, g, etc)? *Question relevant when: selected( ${q104_M23_full_list} , '1')* | microgram — microgram (mcg)<br>miligram — miligram (mg)<br>miligram_dissolved_in_liquid — miligram (mg) dissolved in liquid (e.g. 2 mg per 3 militers)<br>gram — gram (g)<br>militer — militer (ml)<br>centiliter — centiliter (cl)<br>litre — litre (l)<br>ml_of _solution — ml of solution (e.g. ml of 10% solution)<br>litre_of_solution — litre of solution (e.g. 1 litre of 5% solution)<br>cubic_meter — cubic meter (m^3)<br>other — other<br>dont_know — don't know |
| q204_M23c.1 | q204_M23c.1 If other unit of measurement or route of administration, specify here: *Leave blank if not applicable* *Question relevant when: selected( ${q104_M23_full_list} , '1')* | |
| q204_M23d *(required)* | q204_M23d. In total, how many units are used to treat a patient during all of her care? *Remember to add all units! E.g. 5 days x 200 mg twice per day = 2,000mg* *Question relevant when: selected( ${q104_M23_full_list} , '1')* *Response constrained to: .>0* | |
| q204_M24 | ***q204_M24. Lidocaine HCl, 1%*** *Question relevant when: selected( ${q104_M24_full_list} , '1')* | |
| q204_M24a *(required)* | q204_M24a. What percent of patients require this item? *Question relevant when: selected( ${q104_M24_full_list} , '1')* *Response constrained to: .>0 and .<=100 or .=999* | |
| q204_M24b *(required)* | q204_M24b. How is the item administered, or given, to the patient? *Question relevant when: selected( ${q104_M24_full_list} , '1')* | 1 Oral - solid (e.g. tablet)<br>2 Oral - liquid<br>3 Sublingual<br>4 Intravenous (IV)<br>5 Intramusculuar injection (IM)<br>6 Subcutaneous<br>7 Rectal<br>8 Inhaled (e.g. gas)<br>9 Inserted (e.g. vaginal pessaries)<br>10 Topical - cream<br>11 Topical - drops (e.g. eye drops)<br>12 Other<br>99 Don't know |

| Field | Question | Answer |
|---|---|---|
| q204_M24c *(required)* | q204_M24c. What is the item's smallest unit of measurement (mg, mcg, ml, g, etc)?<br>*Question relevant when: selected( ${q104_M24_full_list} , '1')* | microgram — microgram (mcg)<br>miligram — miligram (mg)<br>miligram_dissolved_in_liquid — miligram (mg) dissolved in liquid (e.g. 2 mg per 3 militers)<br>gram — gram (g)<br>militer — militer (ml)<br>centiliter — centiliter (cl)<br>litre — litre (l)<br>ml_of _solution — ml of solution (e.g. ml of 10% solution)<br>litre_of_solution — litre of solution (e.g. 1 litre of 5% solution)<br>cubic_meter — cubic meter (m^3)<br>other — other<br>dont_know — don't know |
| q204_M24c.1 | q204_M24c.1 If other unit of measurement or route of administration, specify here:<br>*Leave blank if not applicable*<br>*Question relevant when: selected( ${q104_M24_full_list} , '1')* | |
| q204_M24d *(required)* | q204_M24d. In total, how many units are used to treat a patient during all of her care?<br>*Remember to add all units! E.g. 5 days x 200 mg twice per day = 2,000mg*<br>*Question relevant when: selected( ${q104_M24_full_list} , '1')*<br>*Response constrained to: .>0* | |
| q204_M25 | ***q204_M25. Anesthetic - other 1: "[q104_M25_full_list_other]"***<br>*Question relevant when: selected( ${q104_M25_full_list} , '1')* | |
| q204_M25a *(required)* | q204_M25a. What percent of patients require this item?<br>*Question relevant when: selected( ${q104_M25_full_list} , '1')*<br>*Response constrained to: .>0 and .<=100 or .=999* | |
| q204_M25b *(required)* | q204_M25b. How is the item administered, or given, to the patient?<br>*Question relevant when: selected( ${q104_M25_full_list} , '1')* | 1 Oral - solid (e.g. tablet)<br>2 Oral - liquid<br>3 Sublingual<br>4 Intravenous (IV)<br>5 Intramuscular injection (IM)<br>6 Subcutaneous<br>7 Rectal<br>8 Inhaled (e.g. gas)<br>9 Inserted (e.g. vaginal pessaries)<br>10 Topical - cream<br>11 Topical - drops (e.g. eye drops)<br>12 Other<br>99 Don't know |

| Field | Question | Answer |
|---|---|---|
| q204_M25c *(required)* | q204_M25c. What is the item's smallest unit of measurement (mg, mcg, ml, g, etc)?<br>*Question relevant when: selected( ${q104_M25_full_list} , '1')* | microgram — microgram (mcg)<br>miligram — miligram (mg)<br>miligram_dissolved_in_liquid — miligram (mg) dissolved in liquid (e.g. 2 mg per 3 militers)<br>gram — gram (g)<br>militer — militer (ml)<br>centiliter — centiliter (cl)<br>litre — litre (l)<br>ml_of _solution — ml of solution (e.g. ml of 10% solution)<br>litre_of_solution — litre of solution (e.g. 1 litre of 5% solution)<br>cubic_meter — cubic meter (m^3)<br>other — other<br>dont_know — don't know |
| q204_M25c.1 | q204_M25c.1 If other unit of measurement or route of administration, specify here:<br>*Leave blank if not applicable*<br>*Question relevant when: selected( ${q104_M25_full_list} , '1')* | |
| q204_M25d *(required)* | q204_M25d. In total, how many units are used to treat a patient during all of her care?<br>*Remember to add all units! E.g. 5 days x 200 mg twice per day = 2,000mg*<br>*Question relevant when: selected( ${q104_M25_full_list} , '1')*<br>*Response constrained to: .>0* | |
| q204_M26 | ***q204_M26. Anesthetic - other 2: "[q104_M26_full_list_other]"***<br>*Question relevant when: selected( ${q104_M26_full_list} , '1')* | |
| q204_M26a *(required)* | q204_M26a. What percent of patients require this item?<br>*Question relevant when: selected( ${q104_M26_full_list} , '1')*<br>*Response constrained to: .>0 and .<=100 or .=999* | |
| q204_M26b *(required)* | q204_M26b. How is the item administered, or given, to the patient?<br>*Question relevant when: selected( ${q104_M26_full_list} , '1')* | 1 Oral - solid (e.g. tablet)<br>2 Oral - liquid<br>3 Sublingual<br>4 Intravenous (IV)<br>5 Intramusculuar injection (IM)<br>6 Subcutaneous<br>7 Rectal<br>8 Inhaled (e.g. gas)<br>9 Inserted (e.g. vaginal pessaries)<br>10 Topical - cream<br>11 Topical - drops (e.g. eye drops)<br>12 Other<br>99 Don't know |

| Field | Question | Answer |
|---|---|---|
| q204_M26c *(required)* | q204_M26c. What is the item's smallest unit of measurement (mg, mcg, ml, g, etc)?<br>*Question relevant when: selected( ${q104_M26_full_list} , '1')* | microgram — microgram (mcg)<br>miligram — miligram (mg)<br>miligram_dissolved_in_liquid — miligram (mg) dissolved in liquid (e.g. 2 mg per 3 militers)<br>gram — gram (g)<br>militer — militer (ml)<br>centiliter — centiliter (cl)<br>litre — litre (l)<br>ml_of _solution — ml of solution (e.g. ml of 10% solution)<br>litre_of_solution — litre of solution (e.g. 1 litre of 5% solution)<br>cubic_meter — cubic meter (m^3)<br>other — other<br>dont_know — don't know |
| q204_M26c.1 | q204_M26c.1 If other unit of measurement or route of administration, specify here:<br>*Leave blank if not applicable*<br>*Question relevant when: selected( ${q104_M26_full_list} , '1')* | |
| q204_M26d *(required)* | q204_M26d. In total, how many units are used to treat a patient during all of her care?<br>*Remember to add all units! E.g. 5 days x 200 mg twice per day = 2,000mg*<br>*Question relevant when: selected( ${q104_M26_full_list} , '1')*<br>*Response constrained to: .>0* | |
| q204_M27 | ***q204_M27. Anesthetic - other 3: "[q104_M27_full_list_other]"***<br>*Question relevant when: selected( ${q104_M27_full_list} , '1')* | |
| q204_M27a *(required)* | q204_M27a. What percent of patients require this item?<br>*Question relevant when: selected( ${q104_M27_full_list} , '1')*<br>*Response constrained to: .>0 and .<=100 or .=999* | |
| q204_M27b *(required)* | q204_M27b. How is the item administered, or given, to the patient?<br>*Question relevant when: selected( ${q104_M27_full_list} , '1')* | 1 Oral - solid (e.g. tablet)<br>2 Oral - liquid<br>3 Sublingual<br>4 Intravenous (IV)<br>5 Intramusculuar injection (IM)<br>6 Subcutaneous<br>7 Rectal<br>8 Inhaled (e.g. gas)<br>9 Inserted (e.g. vaginal pessaries)<br>10 Topical - cream<br>11 Topical - drops (e.g. eye drops)<br>12 Other<br>99 Don't know |

| Field | Question | Answer |
|---|---|---|
| q204_M27c *(required)* | q204_M27c. What is the item's smallest unit of measurement (mg, mcg, ml, g, etc)?<br>*Question relevant when: selected( ${q104_M27_full_list} , '1')* | microgram — microgram (mcg)<br>miligram — miligram (mg)<br>miligram_dissolved_in_liquid — miligram (mg) dissolved in liquid (e.g. 2 mg per 3 militers)<br>gram — gram (g)<br>militer — militer (ml)<br>centiliter — centiliter (cl)<br>litre — litre (l)<br>ml_of _solution — ml of solution (e.g. ml of 10% solution)<br>litre_of_solution — litre of solution (e.g. 1 litre of 5% solution)<br>cubic_meter — cubic meter (m^3)<br>other — other<br>dont_know — don't know |
| q204_M27c.1 | q204_M27c.1 If other unit of measurement or route of administration, specify here:<br>*Leave blank if not applicable*<br>*Question relevant when: selected( ${q104_M27_full_list} , '1')* | |
| q204_M27d *(required)* | q204_M27d. In total, how many units are used to treat a patient during all of her care?<br>*Remember to add all units! E.g. 5 days x 200 mg twice per day = 2,000mg*<br>*Question relevant when: selected( ${q104_M27_full_list} , '1')*<br>*Response constrained to: .>0* | |
| q204_M28 | ***q204_M28. Anesthetic - other 4: "[q104_M28_full_list_other]"***<br>*Question relevant when: selected( ${q104_M28_full_list} , '1')* | |
| q204_M28a *(required)* | q204_M28a. What percent of patients require this item?<br>*Question relevant when: selected( ${q104_M28_full_list} , '1')*<br>*Response constrained to: .>0 and .<=100 or .=999* | |
| q204_M28b *(required)* | q204_M28b. How is the item administered, or given, to the patient?<br>*Question relevant when: selected( ${q104_M28_full_list} , '1')* | 1 Oral - solid (e.g. tablet)<br>2 Oral - liquid<br>3 Sublingual<br>4 Intravenous (IV)<br>5 Intramusculuar injection (IM)<br>6 Subcutaneous<br>7 Rectal<br>8 Inhaled (e.g. gas)<br>9 Inserted (e.g. vaginal pessaries)<br>10 Topical - cream<br>11 Topical - drops (e.g. eye drops)<br>12 Other<br>99 Don't know |

| Field | Question | Answer |
|---|---|---|
| q204_M28c *(required)* | q204_M28c. What is the item's smallest unit of measurement (mg, mcg, ml, g, etc)?<br>*Question relevant when: selected( ${q104_M28_full_list} , '1')* | microgram — microgram (mcg)<br>miligram — miligram (mg)<br>miligram_dissolved_in_liquid — miligram (mg) dissolved in liquid (e.g. 2 mg per 3 militers)<br>gram — gram (g)<br>militer — militer (ml)<br>centiliter — centiliter (cl)<br>litre — litre (l)<br>ml_of _solution — ml of solution (e.g. ml of 10% solution)<br>litre_of_solution — litre of solution (e.g. 1 litre of 5% solution)<br>cubic_meter — cubic meter (m^3)<br>other — other<br>dont_know — don't know |
| q204_M28c.1 | q204_M28c.1 If other unit of measurement or route of administration, specify here:<br>*Leave blank if not applicable*<br>*Question relevant when: selected( ${q104_M28_full_list} , '1')* | |
| q204_M28d *(required)* | q204_M28d. In total, how many units are used to treat a patient during all of her care?<br>*Remember to add all units! E.g. 5 days x 200 mg twice per day = 2,000mg*<br>*Question relevant when: selected( ${q104_M28_full_list} , '1')*<br>*Response constrained to: .>0* | |
| q204_M29 | ***q204_M29. Anesthetic - other 5: "[q104_M29_full_list_other]"***<br>*Question relevant when: selected( ${q104_M29_full_list} , '1')* | |
| q204_M29a *(required)* | q204_M29a. What percent of patients require this item?<br>*Question relevant when: selected( ${q104_M29_full_list} , '1')*<br>*Response constrained to: .>0 and .<=100 or .=999* | |
| q204_M29b *(required)* | q204_M29b. How is the item administered, or given, to the patient?<br>*Question relevant when: selected( ${q104_M29_full_list} , '1')* | 1 Oral - solid (e.g. tablet)<br>2 Oral - liquid<br>3 Sublingual<br>4 Intravenous (IV)<br>5 Intramusculuar injection (IM)<br>6 Subcutaneous<br>7 Rectal<br>8 Inhaled (e.g. gas)<br>9 Inserted (e.g. vaginal pessaries)<br>10 Topical - cream<br>11 Topical - drops (e.g. eye drops)<br>12 Other<br>99 Don't know |

| Field | Question | Answer |
|---|---|---|
| q204_M29c *(required)* | q204_M29c. What is the item's smallest unit of measurement (mg, mcg, ml, g, etc)?<br>*Question relevant when: selected( ${q104_M29_full_list} , '1')* | microgram — microgram (mcg)<br>miligram — miligram (mg)<br>miligram_dissolved_in_liquid — miligram (mg) dissolved in liquid (e.g. 2 mg per 3 militers)<br>gram — gram (g)<br>militer — militer (ml)<br>centiliter — centiliter (cl)<br>litre — litre (l)<br>ml_of _solution — ml of solution (e.g. ml of 10% solution)<br>litre_of_solution — litre of solution (e.g. 1 litre of 5% solution)<br>cubic_meter — cubic meter (m^3)<br>other — other<br>dont_know — don't know |
| q204_M29c.1 | q204_M29c.1 If other unit of measurement or route of administration, specify here:<br>*Leave blank if not applicable*<br>*Question relevant when: selected( ${q104_M29_full_list} , '1')* | |
| q204_M29d *(required)* | q204_M29d. In total, how many units are used to treat a patient during all of her care?<br>*Remember to add all units! E.g. 5 days x 200 mg twice per day = 2,000mg*<br>*Question relevant when: selected( ${q104_M29_full_list} , '1')*<br>*Response constrained to: .>0* | |
| <p><strong><em>D. Medications (3)</em></strong></p><br>*Group relevant when: selected( ${section_two_skip_med} , '1')* | | |
| q204_med_note3 | 204 CONTINUED For each of the following medications and other medical products that are used for incomplete abortion, can you tell me …? | |
| note_204_antibiotics | ***Antibiotics*** | |
| q204_M30 | ***q204_M30. Amoxicillin***<br>*Question relevant when: selected( ${q104_M30_full_list} , '1')* | |
| q204_M30a *(required)* | q204_M30a. What percent of patients require this item?<br>*Question relevant when: selected( ${q104_M30_full_list} , '1')*<br>*Response constrained to: .>0 and .<=100 or .=999* | |
| q204_M30b *(required)* | q204_M30b. How is the item administered, or given, to the patient?<br>*Question relevant when: selected( ${q104_M30_full_list} , '1')* | 1 Oral - solid (e.g. tablet)<br>2 Oral - liquid<br>3 Sublingual<br>4 Intravenous (IV)<br>5 Intramusculuar injection (IM)<br>6 Subcutaneous<br>7 Rectal<br>8 Inhaled (e.g. gas)<br>9 Inserted (e.g. vaginal pessaries)<br>10 Topical - cream<br>11 Topical - drops (e.g. eye drops)<br>12 Other<br>99 Don't know |

| Field | Question | Answer |
|---|---|---|
| q204_M30c *(required)* | q204_M30c. What is the item's smallest unit of measurement (mg, mcg, ml, g, etc)?<br>*Question relevant when: selected( ${q104_M30_full_list} , '1')* | microgram — microgram (mcg)<br>miligram — miligram (mg)<br>miligram_dissolved_in_liquid — miligram (mg) dissolved in liquid (e.g. 2 mg per 3 militers)<br>gram — gram (g)<br>militer — militer (ml)<br>centiliter — centiliter (cl)<br>litre — litre (l)<br>ml_of _solution — ml of solution (e.g. ml of 10% solution)<br>litre_of_solution — litre of solution (e.g. 1 litre of 5% solution)<br>cubic_meter — cubic meter (m^3)<br>other — other<br>dont_know — don't know |
| q204_M30c.1 | q204_M30c.1 If other unit of measurement or route of administration, specify here:<br>*Leave blank if not applicable*<br>*Question relevant when: selected( ${q104_M30_full_list} , '1')* | |
| q204_M30d *(required)* | q204_M30d. In total, how many units are used to treat a patient during all of her care?<br>*Remember to add all units! E.g. 5 days x 200 mg twice per day = 2,000mg*<br>*Question relevant when: selected( ${q104_M30_full_list} , '1')*<br>*Response constrained to: .>0* | |
| q204_M31 | ***q204_M31. Amoxicillin + Clauvulenic Acid 635mg***<br>*Question relevant when: selected( ${q104_M31_full_list} , '1')* | |
| q204_M31a *(required)* | q204_M31a. What percent of patients require this item?<br>*Question relevant when: selected( ${q104_M31_full_list} , '1')*<br>*Response constrained to: .>0 and .<=100 or .=999* | |
| q204_M31b *(required)* | q204_M31b. How is the item administered, or given, to the patient?<br>*Question relevant when: selected( ${q104_M31_full_list} , '1')* | 1 Oral - solid (e.g. tablet)<br>2 Oral - liquid<br>3 Sublingual<br>4 Intravenous (IV)<br>5 Intramusculuar injection (IM)<br>6 Subcutaneous<br>7 Rectal<br>8 Inhaled (e.g. gas)<br>9 Inserted (e.g. vaginal pessaries)<br>10 Topical - cream<br>11 Topical - drops (e.g. eye drops)<br>12 Other<br>99 Don't know |

| Field | Question | Answer |
|---|---|---|
| q204_M31c *(required)* | q204_M31c. What is the item's smallest unit of measurement (mg, mcg, ml, g, etc)?<br>*Question relevant when: selected( ${q104_M31_full_list} , '1')* | microgram — microgram (mcg)<br>miligram — miligram (mg)<br>miligram_dissolved_in_liquid — miligram (mg) dissolved in liquid (e.g. 2 mg per 3 militers)<br>gram — gram (g)<br>militer — militer (ml)<br>centiliter — centiliter (cl)<br>litre — litre (l)<br>ml_of _solution — ml of solution (e.g. ml of 10% solution)<br>litre_of_solution — litre of solution (e.g. 1 litre of 5% solution)<br>cubic_meter — cubic meter (m^3)<br>other — other<br>dont_know — don't know |
| q204_M31c.1 | q204_M31c.1 If other unit of measurement or route of administration, specify here:<br>*Leave blank if not applicable*<br>*Question relevant when: selected( ${q104_M31_full_list} , '1')* | |
| q204_M31d *(required)* | q204_M31d. In total, how many units are used to treat a patient during all of her care?<br>*Remember to add all units! E.g. 5 days x 200 mg twice per day = 2,000mg*<br>*Question relevant when: selected( ${q104_M31_full_list} , '1')*<br>*Response constrained to: .>0* | |
| q204_M32 | ***q204_M32. Ampicillin***<br>*Question relevant when: selected( ${q104_M32_full_list} , '1')* | |
| q204_M32a *(required)* | q204_M32a. What percent of patients require this item?<br>*Question relevant when: selected( ${q104_M32_full_list} , '1')*<br>*Response constrained to: .>0 and .<=100 or .=999* | |
| q204_M32b *(required)* | q204_M32b. How is the item administered, or given, to the patient?<br>*Question relevant when: selected( ${q104_M32_full_list} , '1')* | 1 Oral - solid (e.g. tablet)<br>2 Oral - liquid<br>3 Sublingual<br>4 Intravenous (IV)<br>5 Intramusculuar injection (IM)<br>6 Subcutaneous<br>7 Rectal<br>8 Inhaled (e.g. gas)<br>9 Inserted (e.g. vaginal pessaries)<br>10 Topical - cream<br>11 Topical - drops (e.g. eye drops)<br>12 Other<br>99 Don't know |

| Field | Question | Answer |
|---|---|---|
| q204_M32c *(required)* | q204_M32c. What is the item's smallest unit of measurement (mg, mcg, ml, g, etc)?<br>*Question relevant when: selected( ${q104_M32_full_list} , '1')* | microgram — microgram (mcg)<br>miligram — miligram (mg)<br>miligram_dissolved_in_liquid — miligram (mg) dissolved in liquid (e.g. 2 mg per 3 militers)<br>gram — gram (g)<br>militer — militer (ml)<br>centiliter — centiliter (cl)<br>litre — litre (l)<br>ml_of _solution — ml of solution (e.g. ml of 10% solution)<br>litre_of_solution — litre of solution (e.g. 1 litre of 5% solution)<br>cubic_meter — cubic meter (m^3)<br>other — other<br>dont_know — don't know |
| q204_M32c.1 | q204_M32c.1 If other unit of measurement or route of administration, specify here:<br>*Leave blank if not applicable*<br>*Question relevant when: selected( ${q104_M32_full_list} , '1')* | |
| q204_M32d *(required)* | q204_M32d. In total, how many units are used to treat a patient during all of her care?<br>*Remember to add all units! E.g. 5 days x 200 mg twice per day = 2,000mg*<br>*Question relevant when: selected( ${q104_M32_full_list} , '1')*<br>*Response constrained to: .>0* | |
| q204_M33 | ***q204_M33. Ampicillin (injection)***<br>*Question relevant when: selected( ${q104_M33_full_list} , '1')* | |
| q204_M33a *(required)* | q204_M33a. What percent of patients require this item?<br>*Question relevant when: selected( ${q104_M33_full_list} , '1')*<br>*Response constrained to: .>0 and .<=100 or .=999* | |
| q204_M33b *(required)* | q204_M33b. How is the item administered, or given, to the patient?<br>*Question relevant when: selected( ${q104_M33_full_list} , '1')* | 1 Oral - solid (e.g. tablet)<br>2 Oral - liquid<br>3 Sublingual<br>4 Intravenous (IV)<br>5 Intramusculuar injection (IM)<br>6 Subcutaneous<br>7 Rectal<br>8 Inhaled (e.g. gas)<br>9 Inserted (e.g. vaginal pessaries)<br>10 Topical - cream<br>11 Topical - drops (e.g. eye drops)<br>12 Other<br>99 Don't know |

| Field | Question | Answer |
|---|---|---|
| q204_M33c *(required)* | q204_M33c. What is the item's smallest unit of measurement (mg, mcg, ml, g, etc)?<br>*Question relevant when: selected( ${q104_M33_full_list} , '1')* | microgram — microgram (mcg)<br>miligram — miligram (mg)<br>miligram_dissolved_in_liquid — miligram (mg) dissolved in liquid (e.g. 2 mg per 3 militers)<br>gram — gram (g)<br>militer — militer (ml)<br>centiliter — centiliter (cl)<br>litre — litre (l)<br>ml_of _solution — ml of solution (e.g. ml of 10% solution)<br>litre_of_solution — litre of solution (e.g. 1 litre of 5% solution)<br>cubic_meter — cubic meter (m^3)<br>other — other<br>dont_know — don't know |
| q204_M33c.1 | q204_M33c.1 If other unit of measurement or route of administration, specify here:<br>*Leave blank if not applicable*<br>*Question relevant when: selected( ${q104_M33_full_list} , '1')* | |
| q204_M33d *(required)* | q204_M33d. In total, how many units are used to treat a patient during all of her care?<br>*Remember to add all units! E.g. 5 days x 200 mg twice per day = 2,000mg*<br>*Question relevant when: selected( ${q104_M33_full_list} , '1')*<br>*Response constrained to: .>0* | |
| q204_M34 | ***q204_M34. Ampicillin/sulbactam (Unasyn)***<br>*Question relevant when: selected( ${q104_M34_full_list} , '1')* | |
| q204_M34a *(required)* | q204_M34a. What percent of patients require this item?<br>*Question relevant when: selected( ${q104_M34_full_list} , '1')*<br>*Response constrained to: .>0 and .<=100 or .=999* | |
| q204_M34b *(required)* | q204_M34b. How is the item administered, or given, to the patient?<br>*Question relevant when: selected( ${q104_M34_full_list} , '1')* | 1 Oral - solid (e.g. tablet)<br>2 Oral - liquid<br>3 Sublingual<br>4 Intravenous (IV)<br>5 Intramusculuar injection (IM)<br>6 Subcutaneous<br>7 Rectal<br>8 Inhaled (e.g. gas)<br>9 Inserted (e.g. vaginal pessaries)<br>10 Topical - cream<br>11 Topical - drops (e.g. eye drops)<br>12 Other<br>99 Don't know |

| Field | Question | Answer |
|---|---|---|
| q204_M34c *(required)* | q204_M34c. What is the item's smallest unit of measurement (mg, mcg, ml, g, etc)?<br>*Question relevant when: selected( ${q104_M34_full_list} , '1')* | microgram — microgram (mcg)<br>miligram — miligram (mg)<br>miligram_dissolved_in_liquid — miligram (mg) dissolved in liquid (e.g. 2 mg per 3 militers)<br>gram — gram (g)<br>militer — militer (ml)<br>centiliter — centiliter (cl)<br>litre — litre (l)<br>ml_of _solution — ml of solution (e.g. ml of 10% solution)<br>litre_of_solution — litre of solution (e.g. 1 litre of 5% solution)<br>cubic_meter — cubic meter (m^3)<br>other — other<br>dont_know — don't know |
| q204_M34c.1 | q204_M34c.1 If other unit of measurement or route of administration, specify here:<br>*Leave blank if not applicable*<br>*Question relevant when: selected( ${q104_M34_full_list} , '1')* | |
| q204_M34d *(required)* | q204_M34d. In total, how many units are used to treat a patient during all of her care?<br>*Remember to add all units! E.g. 5 days x 200 mg twice per day = 2,000mg*<br>*Question relevant when: selected( ${q104_M34_full_list} , '1')*<br>*Response constrained to: .>0* | |
| q204_M35 | ***q204_M35. Ceftriaxone***<br>*Question relevant when: selected( ${q104_M35_full_list} , '1')* | |
| q204_M35a *(required)* | q204_M35a. What percent of patients require this item?<br>*Question relevant when: selected( ${q104_M35_full_list} , '1')*<br>*Response constrained to: .>0 and .<=100 or .=999* | |
| q204_M35b *(required)* | q204_M35b. How is the item administered, or given, to the patient?<br>*Question relevant when: selected( ${q104_M35_full_list} , '1')* | 1 Oral - solid (e.g. tablet)<br>2 Oral - liquid<br>3 Sublingual<br>4 Intravenous (IV)<br>5 Intramusculuar injection (IM)<br>6 Subcutaneous<br>7 Rectal<br>8 Inhaled (e.g. gas)<br>9 Inserted (e.g. vaginal pessaries)<br>10 Topical - cream<br>11 Topical - drops (e.g. eye drops)<br>12 Other<br>99 Don't know |

| Field | Question | Answer |
|---|---|---|
| q204_M35c *(required)* | q204_M35c. What is the item's smallest unit of measurement (mg, mcg, ml, g, etc)?<br>*Question relevant when: selected( ${q104_M35_full_list} , '1')* | microgram — microgram (mcg)<br>miligram — miligram (mg)<br>miligram_dissolved_in_liquid — miligram (mg) dissolved in liquid (e.g. 2 mg per 3 militers)<br>gram — gram (g)<br>militer — militer (ml)<br>centiliter — centiliter (cl)<br>litre — litre (l)<br>ml_of _solution — ml of solution (e.g. ml of 10% solution)<br>litre_of_solution — litre of solution (e.g. 1 litre of 5% solution)<br>cubic_meter — cubic meter (m^3)<br>other — other<br>dont_know — don't know |
| q204_M35c.1 | q204_M35c.1 If other unit of measurement or route of administration, specify here:<br>*Leave blank if not applicable*<br>*Question relevant when: selected( ${q104_M35_full_list} , '1')* | |
| q204_M35d *(required)* | q204_M35d. In total, how many units are used to treat a patient during all of her care?<br>*Remember to add all units! E.g. 5 days x 200 mg twice per day = 2,000mg*<br>*Question relevant when: selected( ${q104_M35_full_list} , '1')*<br>*Response constrained to: .>0* | |
| q204_M36 | ***q204_M36. Chloramphenicol Sodium Succinate (injection)***<br>*Question relevant when: selected( ${q104_M36_full_list} , '1')* | |
| q204_M36a *(required)* | q204_M36a. What percent of patients require this item?<br>*Question relevant when: selected( ${q104_M36_full_list} , '1')*<br>*Response constrained to: .>0 and .<=100 or .=999* | |
| q204_M36b *(required)* | q204_M36b. How is the item administered, or given, to the patient?<br>*Question relevant when: selected( ${q104_M36_full_list} , '1')* | 1 Oral - solid (e.g. tablet)<br>2 Oral - liquid<br>3 Sublingual<br>4 Intravenous (IV)<br>5 Intramusculuar injection (IM)<br>6 Subcutaneous<br>7 Rectal<br>8 Inhaled (e.g. gas)<br>9 Inserted (e.g. vaginal pessaries)<br>10 Topical - cream<br>11 Topical - drops (e.g. eye drops)<br>12 Other<br>99 Don't know |

| Field | Question | Answer |
|---|---|---|
| q204_M36c *(required)* | q204_M36c. What is the item's smallest unit of measurement (mg, mcg, ml, g, etc)?<br>*Question relevant when: selected( ${q104_M36_full_list} , '1')* | microgram: microgram (mcg)<br>miligram: miligram (mg)<br>miligram_dissolved_in_liquid: miligram (mg) dissolved in liquid (e.g. 2 mg per 3 militers)<br>gram: gram (g)<br>militer: militer (ml)<br>centiliter: centiliter (cl)<br>litre: litre (l)<br>ml_of _solution: ml of solution (e.g. ml of 10% solution)<br>litre_of_solution: litre of solution (e.g. 1 litre of 5% solution)<br>cubic_meter: cubic meter (m^3)<br>other: other<br>dont_know: don't know |
| q204_M36c.1 | q204_M36c.1 If other unit of measurement or route of administration, specify here:<br>*Leave blank if not applicable*<br>*Question relevant when: selected( ${q104_M36_full_list} , '1')* | |
| q204_M36d *(required)* | q204_M36d. In total, how many units are used to treat a patient during all of her care?<br>*Remember to add all units! E.g. 5 days x 200 mg twice per day = 2,000mg*<br>*Question relevant when: selected( ${q104_M36_full_list} , '1')*<br>*Response constrained to: .>0* | |
| q204_M37 | ***q204_M37. Ciprofloxacin***<br>*Question relevant when: selected( ${q104_M37_full_list} , '1')* | |
| q204_M37a *(required)* | q204_M37a. What percent of patients require this item?<br>*Question relevant when: selected( ${q104_M37_full_list} , '1')*<br>*Response constrained to: .>0 and .<=100 or .=999* | |
| q204_M37b *(required)* | q204_M37b. How is the item administered, or given, to the patient?<br>*Question relevant when: selected( ${q104_M37_full_list} , '1')* | 1 Oral - solid (e.g. tablet)<br>2 Oral - liquid<br>3 Sublingual<br>4 Intravenous (IV)<br>5 Intramusculuar injection (IM)<br>6 Subcutaneous<br>7 Rectal<br>8 Inhaled (e.g. gas)<br>9 Inserted (e.g. vaginal pessaries)<br>10 Topical - cream<br>11 Topical - drops (e.g. eye drops)<br>12 Other<br>99 Don't know |

| Field | Question | Answer |
|---|---|---|
| q204_M37c *(required)* | q204_M37c. What is the item's smallest unit of measurement (mg, mcg, ml, g, etc)?<br>*Question relevant when: selected( ${q104_M37_full_list} , '1')* | microgram — microgram (mcg)<br>miligram — miligram (mg)<br>miligram_dissolved_in_liquid — miligram (mg) dissolved in liquid (e.g. 2 mg per 3 militers)<br>gram — gram (g)<br>militer — militer (ml)<br>centiliter — centiliter (cl)<br>litre — litre (l)<br>ml_of _solution — ml of solution (e.g. ml of 10% solution)<br>litre_of_solution — litre of solution (e.g. 1 litre of 5% solution)<br>cubic_meter — cubic meter (m^3)<br>other — other<br>dont_know — don't know |
| q204_M37c.1 | q204_M37c.1 If other unit of measurement or route of administration, specify here:<br>*Leave blank if not applicable*<br>*Question relevant when: selected( ${q104_M37_full_list} , '1')* | |
| q204_M37d *(required)* | q204_M37d. In total, how many units are used to treat a patient during all of her care?<br>*Remember to add all units! E.g. 5 days x 200 mg twice per day = 2,000mg*<br>*Question relevant when: selected( ${q104_M37_full_list} , '1')*<br>*Response constrained to: .>0* | |
| q204_M38 | ***q204_M38. Doxycycline***<br>*Question relevant when: selected( ${q104_M38_full_list} , '1')* | |
| q204_M38a *(required)* | q204_M38a. What percent of patients require this item?<br>*Question relevant when: selected( ${q104_M38_full_list} , '1')*<br>*Response constrained to: .>0 and .<=100 or .=999* | |
| q204_M38b *(required)* | q204_M38b. How is the item administered, or given, to the patient?<br>*Question relevant when: selected( ${q104_M38_full_list} , '1')* | 1 Oral - solid (e.g. tablet)<br>2 Oral - liquid<br>3 Sublingual<br>4 Intravenous (IV)<br>5 Intramusculuar injection (IM)<br>6 Subcutaneous<br>7 Rectal<br>8 Inhaled (e.g. gas)<br>9 Inserted (e.g. vaginal pessaries)<br>10 Topical - cream<br>11 Topical - drops (e.g. eye drops)<br>12 Other<br>99 Don't know |

| Field | Question | Answer |
|---|---|---|
| q204_M38c *(required)* | q204_M38c. What is the item's smallest unit of measurement (mg, mcg, ml, g, etc)?<br>*Question relevant when: selected( ${q104_M38_full_list} , '1')* | microgram — microgram (mcg)<br>miligram — miligram (mg)<br>miligram_dissolved_in_liquid — miligram (mg) dissolved in liquid (e.g. 2 mg per 3 militers)<br>gram — gram (g)<br>militer — militer (ml)<br>centiliter — centiliter (cl)<br>litre — litre (l)<br>ml_of _solution — ml of solution (e.g. ml of 10% solution)<br>litre_of_solution — litre of solution (e.g. 1 litre of 5% solution)<br>cubic_meter — cubic meter (m^3)<br>other — other<br>dont_know — don't know |
| q204_M38c.1 | q204_M38c.1 If other unit of measurement or route of administration, specify here:<br>*Leave blank if not applicable*<br>*Question relevant when: selected( ${q104_M38_full_list} , '1')* | |
| q204_M38d *(required)* | q204_M38d. In total, how many units are used to treat a patient during all of her care?<br>*Remember to add all units! E.g. 5 days x 200 mg twice per day = 2,000mg*<br>*Question relevant when: selected( ${q104_M38_full_list} , '1')*<br>*Response constrained to: .>0* | |
| q204_M39 | ***q204_M39. Erythromycin***<br>*Question relevant when: selected( ${q104_M39_full_list} , '1')* | |
| q204_M39a *(required)* | q204_M39a. What percent of patients require this item?<br>*Question relevant when: selected( ${q104_M39_full_list} , '1')*<br>*Response constrained to: .>0 and .<=100 or .=999* | |
| q204_M39b *(required)* | q204_M39b. How is the item administered, or given, to the patient?<br>*Question relevant when: selected( ${q104_M39_full_list} , '1')* | 1 Oral - solid (e.g. tablet)<br>2 Oral - liquid<br>3 Sublingual<br>4 Intravenous (IV)<br>5 Intramusculuar injection (IM)<br>6 Subcutaneous<br>7 Rectal<br>8 Inhaled (e.g. gas)<br>9 Inserted (e.g. vaginal pessaries)<br>10 Topical - cream<br>11 Topical - drops (e.g. eye drops)<br>12 Other<br>99 Don't know |

| Field | Question | Answer |
|---|---|---|
| q204_M39c *(required)* | q204_M39c. What is the item's smallest unit of measurement (mg, mcg, ml, g, etc)? *Question relevant when: selected( ${q104_M39_full_list} , '1')* | microgram — microgram (mcg)<br>miligram — miligram (mg)<br>miligram_dissolved_in_liquid — miligram (mg) dissolved in liquid (e.g. 2 mg per 3 militers)<br>gram — gram (g)<br>militer — militer (ml)<br>centiliter — centiliter (cl)<br>litre — litre (l)<br>ml_of _solution — ml of solution (e.g. ml of 10% solution)<br>litre_of_solution — litre of solution (e.g. 1 litre of 5% solution)<br>cubic_meter — cubic meter (m^3)<br>other — other<br>dont_know — don't know |
| q204_M39c.1 | q204_M39c.1 If other unit of measurement or route of administration, specify here: *Leave blank if not applicable* *Question relevant when: selected( ${q104_M39_full_list} , '1')* | |
| q204_M39d *(required)* | q204_M39d. In total, how many units are used to treat a patient during all of her care? *Remember to add all units! E.g. 5 days x 200 mg twice per day = 2,000mg* *Question relevant when: selected( ${q104_M39_full_list} , '1')* *Response constrained to: .>0* | |
| q204_M40 | ***q204_M40. Gentamycin*** *Question relevant when: selected( ${q104_M40_full_list} , '1')* | |
| q204_M40a *(required)* | q204_M40a. What percent of patients require this item? *Question relevant when: selected( ${q104_M40_full_list} , '1')* *Response constrained to: .>0 and .<=100 or .=999* | |
| q204_M40b *(required)* | q204_M40b. How is the item administered, or given, to the patient? *Question relevant when: selected( ${q104_M40_full_list} , '1')* | 1 Oral - solid (e.g. tablet)<br>2 Oral - liquid<br>3 Sublingual<br>4 Intravenous (IV)<br>5 Intramusculuar injection (IM)<br>6 Subcutaneous<br>7 Rectal<br>8 Inhaled (e.g. gas)<br>9 Inserted (e.g. vaginal pessaries)<br>10 Topical - cream<br>11 Topical - drops (e.g. eye drops)<br>12 Other<br>99 Don't know |

| Field | Question | Answer |
|---|---|---|
| q204_M40c *(required)* | q204_M40c. What is the item's smallest unit of measurement (mg, mcg, ml, g, etc)?<br>*Question relevant when: selected( ${q104_M40_full_list} , '1')* | microgram — microgram (mcg)<br>miligram — miligram (mg)<br>miligram_dissolved_in_liquid — miligram (mg) dissolved in liquid (e.g. 2 mg per 3 militers)<br>gram — gram (g)<br>militer — militer (ml)<br>centiliter — centiliter (cl)<br>litre — litre (l)<br>ml_of _solution — ml of solution (e.g. ml of 10% solution)<br>litre_of_solution — litre of solution (e.g. 1 litre of 5% solution)<br>cubic_meter — cubic meter (m^3)<br>other — other<br>dont_know — don't know |
| q204_M40c.1 | q204_M40c.1 If other unit of measurement or route of administration, specify here:<br>*Leave blank if not applicable*<br>*Question relevant when: selected( ${q104_M40_full_list} , '1')* | |
| q204_M40d *(required)* | q204_M40d. In total, how many units are used to treat a patient during all of her care?<br>*Remember to add all units! E.g. 5 days x 200 mg twice per day = 2,000mg*<br>*Question relevant when: selected( ${q104_M40_full_list} , '1')*<br>*Response constrained to: .>0* | |
| q204_M41 | ***q204_M41. Metronidazole (for infusion)***<br>*Question relevant when: selected( ${q104_M41_full_list} , '1')* | |
| q204_M41a *(required)* | q204_M41a. What percent of patients require this item?<br>*Question relevant when: selected( ${q104_M41_full_list} , '1')*<br>*Response constrained to: .>0 and .<=100 or .=999* | |
| q204_M41b *(required)* | q204_M41b. How is the item administered, or given, to the patient?<br>*Question relevant when: selected( ${q104_M41_full_list} , '1')* | 1 Oral - solid (e.g. tablet)<br>2 Oral - liquid<br>3 Sublingual<br>4 Intravenous (IV)<br>5 Intramusculuar injection (IM)<br>6 Subcutaneous<br>7 Rectal<br>8 Inhaled (e.g. gas)<br>9 Inserted (e.g. vaginal pessaries)<br>10 Topical - cream<br>11 Topical - drops (e.g. eye drops)<br>12 Other<br>99 Don't know |

| Field | Question | Answer |
|---|---|---|
| q204_M41c *(required)* | q204_M41c. What is the item's smallest unit of measurement (mg, mcg, ml, g, etc)?<br>*Question relevant when: selected( ${q104_M41_full_list} , '1')* | microgram — microgram (mcg)<br>miligram — miligram (mg)<br>miligram_dissolved_in_liquid — miligram (mg) dissolved in liquid (e.g. 2 mg per 3 militers)<br>gram — gram (g)<br>militer — militer (ml)<br>centiliter — centiliter (cl)<br>litre — litre (l)<br>ml_of _solution — ml of solution (e.g. ml of 10% solution)<br>litre_of_solution — litre of solution (e.g. 1 litre of 5% solution)<br>cubic_meter — cubic meter (m^3)<br>other — other<br>dont_know — don't know |
| q204_M41c.1 | q204_M41c.1 If other unit of measurement or route of administration, specify here:<br>*Leave blank if not applicable*<br>*Question relevant when: selected( ${q104_M41_full_list} , '1')* | |
| q204_M41d *(required)* | q204_M41d. In total, how many units are used to treat a patient during all of her care?<br>*Remember to add all units! E.g. 5 days x 200 mg twice per day = 2,000mg*<br>*Question relevant when: selected( ${q104_M41_full_list} , '1')*<br>*Response constrained to: .>0* | |
| q204_M42 | ***q204_M42. Metronidazole (injection)***<br>*Question relevant when: selected( ${q104_M42_full_list} , '1')* | |
| q204_M42a *(required)* | q204_M42a. What percent of patients require this item?<br>*Question relevant when: selected( ${q104_M42_full_list} , '1')*<br>*Response constrained to: .>0 and .<=100 or .=999* | |
| q204_M42b *(required)* | q204_M42b. How is the item administered, or given, to the patient?<br>*Question relevant when: selected( ${q104_M42_full_list} , '1')* | 1 Oral - solid (e.g. tablet)<br>2 Oral - liquid<br>3 Sublingual<br>4 Intravenous (IV)<br>5 Intramusculuar injection (IM)<br>6 Subcutaneous<br>7 Rectal<br>8 Inhaled (e.g. gas)<br>9 Inserted (e.g. vaginal pessaries)<br>10 Topical - cream<br>11 Topical - drops (e.g. eye drops)<br>12 Other<br>99 Don't know |

| Field | Question | Answer |
|---|---|---|
| q204_M42c *(required)* | q204_M42c. What is the item's smallest unit of measurement (mg, mcg, ml, g, etc)?<br>*Question relevant when: selected( ${q104_M42_full_list} , '1')* | microgram — microgram (mcg)<br>miligram — miligram (mg)<br>miligram_dissolved_in_liquid — miligram (mg) dissolved in liquid (e.g. 2 mg per 3 militers)<br>gram — gram (g)<br>militer — militer (ml)<br>centiliter — centiliter (cl)<br>litre — litre (l)<br>ml_of _solution — ml of solution (e.g. ml of 10% solution)<br>litre_of_solution — litre of solution (e.g. 1 litre of 5% solution)<br>cubic_meter — cubic meter (m^3)<br>other — other<br>dont_know — don't know |
| q204_M42c.1 | q204_M42c.1 If other unit of measurement or route of administration, specify here:<br>*Leave blank if not applicable*<br>*Question relevant when: selected( ${q104_M42_full_list} , '1')* | |
| q204_M42d *(required)* | q204_M42d. In total, how many units are used to treat a patient during all of her care?<br>*Remember to add all units! E.g. 5 days x 200 mg twice per day = 2,000mg*<br>*Question relevant when: selected( ${q104_M42_full_list} , '1')*<br>*Response constrained to: .>0* | |
| q204_M43 | ***q204_M43. Metronidazole (oral)***<br>*Question relevant when: selected( ${q104_M43_full_list} , '1')* | |
| q204_M43a *(required)* | q204_M43a. What percent of patients require this item?<br>*Question relevant when: selected( ${q104_M43_full_list} , '1')*<br>*Response constrained to: .>0 and .<=100 or .=999* | |
| q204_M43b *(required)* | q204_M43b. How is the item administered, or given, to the patient?<br>*Question relevant when: selected( ${q104_M43_full_list} , '1')* | 1 Oral - solid (e.g. tablet)<br>2 Oral - liquid<br>3 Sublingual<br>4 Intravenous (IV)<br>5 Intramusculuar injection (IM)<br>6 Subcutaneous<br>7 Rectal<br>8 Inhaled (e.g. gas)<br>9 Inserted (e.g. vaginal pessaries)<br>10 Topical - cream<br>11 Topical - drops (e.g. eye drops)<br>12 Other<br>99 Don't know |

| Field | Question | Answer |
|---|---|---|
| q204_M43c *(required)* | q204_M43c. What is the item's smallest unit of measurement (mg, mcg, ml, g, etc)?<br>*Question relevant when: selected( ${q104_M43_full_list} , '1')* | microgram — microgram (mcg)<br>miligram — miligram (mg)<br>miligram_dissolved_in_liquid — miligram (mg) dissolved in liquid (e.g. 2 mg per 3 militers)<br>gram — gram (g)<br>militer — militer (ml)<br>centiliter — centiliter (cl)<br>litre — litre (l)<br>ml_of _solution — ml of solution (e.g. ml of 10% solution)<br>litre_of_solution — litre of solution (e.g. 1 litre of 5% solution)<br>cubic_meter — cubic meter (m^3)<br>other — other<br>dont_know — don't know |
| q204_M43c.1 | q204_M43c.1 If other unit of measurement or route of administration, specify here:<br>*Leave blank if not applicable*<br>*Question relevant when: selected( ${q104_M43_full_list} , '1')* | |
| q204_M43d *(required)* | q204_M43d. In total, how many units are used to treat a patient during all of her care?<br>*Remember to add all units! E.g. 5 days x 200 mg twice per day = 2,000mg*<br>*Question relevant when: selected( ${q104_M43_full_list} , '1')*<br>*Response constrained to: .>0* | |
| q204_M44 | ***q204_M44. Penicillin***<br>*Question relevant when: selected( ${q104_M44_full_list} , '1')* | |
| q204_M44a *(required)* | q204_M44a. What percent of patients require this item?<br>*Question relevant when: selected( ${q104_M44_full_list} , '1')*<br>*Response constrained to: .>0 and .<=100 or .=999* | |
| q204_M44b *(required)* | q204_M44b. How is the item administered, or given, to the patient?<br>*Question relevant when: selected( ${q104_M44_full_list} , '1')* | 1 Oral - solid (e.g. tablet)<br>2 Oral - liquid<br>3 Sublingual<br>4 Intravenous (IV)<br>5 Intramusculuar injection (IM)<br>6 Subcutaneous<br>7 Rectal<br>8 Inhaled (e.g. gas)<br>9 Inserted (e.g. vaginal pessaries)<br>10 Topical - cream<br>11 Topical - drops (e.g. eye drops)<br>12 Other<br>99 Don't know |

| Field | Question | Answer |
|---|---|---|
| q204_M44c *(required)* | q204_M44c. What is the item's smallest unit of measurement (mg, mcg, ml, g, etc)?<br>*Question relevant when: selected( ${q104_M44_full_list} , '1')* | <table><tr><td></td><td>microgram</td><td>microgram (mcg)</td></tr><tr><td></td><td>miligram</td><td>miligram (mg)</td></tr><tr><td></td><td>miligram_dissolved_in_liquid</td><td>miligram (mg) dissolved in liquid (e.g. 2 mg per 3 militers)</td></tr><tr><td></td><td>gram</td><td>gram (g)</td></tr><tr><td></td><td>militer</td><td>militer (ml)</td></tr><tr><td></td><td>centiliter</td><td>centiliter (cl)</td></tr><tr><td></td><td>litre</td><td>litre (l)</td></tr><tr><td></td><td>ml_of _solution</td><td>ml of solution (e.g. ml of 10% solution)</td></tr><tr><td></td><td>litre_of_solution</td><td>litre of solution (e.g. 1 litre of 5% solution)</td></tr><tr><td></td><td>cubic_meter</td><td>cubic meter (m^3)</td></tr><tr><td></td><td>other</td><td>other</td></tr><tr><td></td><td>dont_know</td><td>don't know</td></tr></table> |
| q204_M44c.1 | q204_M44c.1 If other unit of measurement or route of administration, specify here:<br>*Leave blank if not applicable*<br>*Question relevant when: selected( ${q104_M44_full_list} , '1')* | |
| q204_M44d *(required)* | q204_M44d. In total, how many units are used to treat a patient during all of her care?<br>*Remember to add all units! E.g. 5 days x 200 mg twice per day = 2,000mg*<br>*Question relevant when: selected( ${q104_M44_full_list} , '1')*<br>*Response constrained to: .>0* | |
| q204_M45 | ***q204_M45. Antibiotic - other 1: "[q104_M45_full_list_other]"***<br>*Question relevant when: selected( ${q104_M45_full_list} , '1')* | |
| q204_M45a *(required)* | q204_M45a. What percent of patients require this item?<br>*Question relevant when: selected( ${q104_M45_full_list} , '1')*<br>*Response constrained to: .>0 and .<=100 or .=999* | |
| q204_M45b *(required)* | q204_M45b. How is the item administered, or given, to the patient?<br>*Question relevant when: selected( ${q104_M45_full_list} , '1')* | <table><tr><td></td><td>1</td><td>Oral - solid (e.g. tablet)</td></tr><tr><td></td><td>2</td><td>Oral - liquid</td></tr><tr><td></td><td>3</td><td>Sublingual</td></tr><tr><td></td><td>4</td><td>Intravenous (IV)</td></tr><tr><td></td><td>5</td><td>Intramusculuar injection (IM)</td></tr><tr><td></td><td>6</td><td>Subcutaneous</td></tr><tr><td></td><td>7</td><td>Rectal</td></tr><tr><td></td><td>8</td><td>Inhaled (e.g. gas)</td></tr><tr><td></td><td>9</td><td>Inserted (e.g. vaginal pessaries)</td></tr><tr><td></td><td>10</td><td>Topical - cream</td></tr><tr><td></td><td>11</td><td>Topical - drops (e.g. eye drops)</td></tr><tr><td></td><td>12</td><td>Other</td></tr><tr><td></td><td>99</td><td>Don't know</td></tr></table> |

| Field | Question | Answer |
|---|---|---|
| q204_M45c *(required)* | q204_M45c. What is the item's smallest unit of measurement (mg, mcg, ml, g, etc)?<br>*Question relevant when: selected( ${q104_M45_full_list} , '1')* | microgram — microgram (mcg)<br>miligram — miligram (mg)<br>miligram_dissolved_in_liquid — miligram (mg) dissolved in liquid (e.g. 2 mg per 3 militers)<br>gram — gram (g)<br>militer — militer (ml)<br>centiliter — centiliter (cl)<br>litre — litre (l)<br>ml_of _solution — ml of solution (e.g. ml of 10% solution)<br>litre_of_solution — litre of solution (e.g. 1 litre of 5% solution)<br>cubic_meter — cubic meter (m^3)<br>other — other<br>dont_know — don't know |
| q204_M45c.1 | q204_M45c.1 If other unit of measurement or route of administration, specify here:<br>*Leave blank if not applicable*<br>*Question relevant when: selected( ${q104_M45_full_list} , '1')* | |
| q204_M45d *(required)* | q204_M45d. In total, how many units are used to treat a patient during all of her care?<br>*Remember to add all units! E.g. 5 days x 200 mg twice per day = 2,000mg*<br>*Question relevant when: selected( ${q104_M45_full_list} , '1')*<br>*Response constrained to: .>0* | |
| q204_M46 | ***q204_M46. Antibiotic - other 2: "[q104_M46_full_list_other]"***<br>*Question relevant when: selected( ${q104_M46_full_list} , '1')* | |
| q204_M46a *(required)* | q204_M46a. What percent of patients require this item?<br>*Question relevant when: selected( ${q104_M46_full_list} , '1')*<br>*Response constrained to: .>0 and .<=100 or .=999* | |
| q204_M46b *(required)* | q204_M46b. How is the item administered, or given, to the patient?<br>*Question relevant when: selected( ${q104_M46_full_list} , '1')* | 1 Oral - solid (e.g. tablet)<br>2 Oral - liquid<br>3 Sublingual<br>4 Intravenous (IV)<br>5 Intramusculuar injection (IM)<br>6 Subcutaneous<br>7 Rectal<br>8 Inhaled (e.g. gas)<br>9 Inserted (e.g. vaginal pessaries)<br>10 Topical - cream<br>11 Topical - drops (e.g. eye drops)<br>12 Other<br>99 Don't know |

| Field | Question | Answer |
|---|---|---|
| q204_M46c *(required)* | q204_M46c. What is the item's smallest unit of measurement (mg, mcg, ml, g, etc)?<br>*Question relevant when: selected( ${q104_M46_full_list} , '1')* | microgram — microgram (mcg)<br>miligram — miligram (mg)<br>miligram_dissolved_in_liquid — miligram (mg) dissolved in liquid (e.g. 2 mg per 3 militers)<br>gram — gram (g)<br>militer — militer (ml)<br>centiliter — centiliter (cl)<br>litre — litre (l)<br>ml_of _solution — ml of solution (e.g. ml of 10% solution)<br>litre_of_solution — litre of solution (e.g. 1 litre of 5% solution)<br>cubic_meter — cubic meter (m^3)<br>other — other<br>dont_know — don't know |
| q204_M46c.1 | q204_M46c.1 If other unit of measurement or route of administration, specify here:<br>*Leave blank if not applicable*<br>*Question relevant when: selected( ${q104_M46_full_list} , '1')* | |
| q204_M46d *(required)* | q204_M46d. In total, how many units are used to treat a patient during all of her care?<br>*Remember to add all units! E.g. 5 days x 200 mg twice per day = 2,000mg*<br>*Question relevant when: selected( ${q104_M46_full_list} , '1')*<br>*Response constrained to: .>0* | |
| q204_M47 | ***q204_M47. Antibiotic - other 3: "[q104_M47_full_list_other]"***<br>*Question relevant when: selected( ${q104_M47_full_list} , '1')* | |
| q204_M47a *(required)* | q204_M47a. What percent of patients require this item?<br>*Question relevant when: selected( ${q104_M47_full_list} , '1')*<br>*Response constrained to: .>0 and .<=100 or .=999* | |
| q204_M47b *(required)* | q204_M47b. How is the item administered, or given, to the patient?<br>*Question relevant when: selected( ${q104_M47_full_list} , '1')* | 1 Oral - solid (e.g. tablet)<br>2 Oral - liquid<br>3 Sublingual<br>4 Intravenous (IV)<br>5 Intramusculuar injection (IM)<br>6 Subcutaneous<br>7 Rectal<br>8 Inhaled (e.g. gas)<br>9 Inserted (e.g. vaginal pessaries)<br>10 Topical - cream<br>11 Topical - drops (e.g. eye drops)<br>12 Other<br>99 Don't know |

| Field | Question | Answer |
|---|---|---|
| q204_M47c *(required)* | q204_M47c. What is the item's smallest unit of measurement (mg, mcg, ml, g, etc)?<br>*Question relevant when: selected( ${q104_M47_full_list} , '1')* | microgram — microgram (mcg)<br>miligram — miligram (mg)<br>miligram_dissolved_in_liquid — miligram (mg) dissolved in liquid (e.g. 2 mg per 3 militers)<br>gram — gram (g)<br>militer — militer (ml)<br>centiliter — centiliter (cl)<br>litre — litre (l)<br>ml_of _solution — ml of solution (e.g. ml of 10% solution)<br>litre_of_solution — litre of solution (e.g. 1 litre of 5% solution)<br>cubic_meter — cubic meter (m^3)<br>other — other<br>dont_know — don't know |
| q204_M47c.1 | q204_M47c.1 If other unit of measurement or route of administration, specify here:<br>*Leave blank if not applicable*<br>*Question relevant when: selected( ${q104_M47_full_list} , '1')* | |
| q204_M47d *(required)* | q204_M47d. In total, how many units are used to treat a patient during all of her care?<br>*Remember to add all units! E.g. 5 days x 200 mg twice per day = 2,000mg*<br>*Question relevant when: selected( ${q104_M47_full_list} , '1')*<br>*Response constrained to: .>0* | |
| q204_M48 | ***q204_M48. Antibiotic - other 4: "[q104_M48_full_list_other]"***<br>*Question relevant when: selected( ${q104_M48_full_list} , '1')* | |
| q204_M48a *(required)* | q204_M48a. What percent of patients require this item?<br>*Question relevant when: selected( ${q104_M48_full_list} , '1')*<br>*Response constrained to: .>0 and .<=100 or .=999* | |
| q204_M48b *(required)* | q204_M48b. How is the item administered, or given, to the patient?<br>*Question relevant when: selected( ${q104_M48_full_list} , '1')* | 1 Oral - solid (e.g. tablet)<br>2 Oral - liquid<br>3 Sublingual<br>4 Intravenous (IV)<br>5 Intramusculuar injection (IM)<br>6 Subcutaneous<br>7 Rectal<br>8 Inhaled (e.g. gas)<br>9 Inserted (e.g. vaginal pessaries)<br>10 Topical - cream<br>11 Topical - drops (e.g. eye drops)<br>12 Other<br>99 Don't know |

| Field | Question | Answer |
|---|---|---|
| q204_M48c *(required)* | q204_M48c. What is the item's smallest unit of measurement (mg, mcg, ml, g, etc)?<br>*Question relevant when: selected( ${q104_M48_full_list} , '1')* | microgram — microgram (mcg)<br>miligram — miligram (mg)<br>miligram_dissolved_in_liquid — miligram (mg) dissolved in liquid (e.g. 2 mg per 3 militers)<br>gram — gram (g)<br>militer — militer (ml)<br>centiliter — centiliter (cl)<br>litre — litre (l)<br>ml_of _solution — ml of solution (e.g. ml of 10% solution)<br>litre_of_solution — litre of solution (e.g. 1 litre of 5% solution)<br>cubic_meter — cubic meter (m^3)<br>other — other<br>dont_know — don't know |
| q204_M48c.1 | q204_M48c.1 If other unit of measurement or route of administration, specify here:<br>*Leave blank if not applicable*<br>*Question relevant when: selected( ${q104_M48_full_list} , '1')* | |
| q204_M48d *(required)* | q204_M48d. In total, how many units are used to treat a patient during all of her care?<br>*Remember to add all units! E.g. 5 days x 200 mg twice per day = 2,000mg*<br>*Question relevant when: selected( ${q104_M48_full_list} , '1')*<br>*Response constrained to: .>0* | |
| q204_M49 | ***q204_M49. Antibiotic - other 5: "[q104_M49_full_list_other]"***<br>*Question relevant when: selected( ${q104_M49_full_list} , '1')* | |
| q204_M49a *(required)* | q204_M49a. What percent of patients require this item?<br>*Question relevant when: selected( ${q104_M49_full_list} , '1')*<br>*Response constrained to: .>0 and .<=100 or .=999* | |
| q204_M49b *(required)* | q204_M49b. How is the item administered, or given, to the patient?<br>*Question relevant when: selected( ${q104_M49_full_list} , '1')* | 1 Oral - solid (e.g. tablet)<br>2 Oral - liquid<br>3 Sublingual<br>4 Intravenous (IV)<br>5 Intramusculuar injection (IM)<br>6 Subcutaneous<br>7 Rectal<br>8 Inhaled (e.g. gas)<br>9 Inserted (e.g. vaginal pessaries)<br>10 Topical - cream<br>11 Topical - drops (e.g. eye drops)<br>12 Other<br>99 Don't know |

| Field | Question | Answer |
|---|---|---|
| q204_M49c *(required)* | q204_M49c. What is the item's smallest unit of measurement (mg, mcg, ml, g, etc)?<br>*Question relevant when: selected( ${q104_M49_full_list} , '1')* | microgram — microgram (mcg)<br>miligram — miligram (mg)<br>miligram_dissolved_in_liquid — miligram (mg) dissolved in liquid (e.g. 2 mg per 3 militers)<br>gram — gram (g)<br>militer — militer (ml)<br>centiliter — centiliter (cl)<br>litre — litre (l)<br>ml_of _solution — ml of solution (e.g. ml of 10% solution)<br>litre_of_solution — litre of solution (e.g. 1 litre of 5% solution)<br>cubic_meter — cubic meter (m^3)<br>other — other<br>dont_know — don't know |
| q204_M49c.1 | q204_M49c.1 If other unit of measurement or route of administration, specify here:<br>*Leave blank if not applicable*<br>*Question relevant when: selected( ${q104_M49_full_list} , '1')* | |
| q204_M49d *(required)* | q204_M49d. In total, how many units are used to treat a patient during all of her care?<br>*Remember to add all units! E.g. 5 days x 200 mg twice per day = 2,000mg*<br>*Question relevant when: selected( ${q104_M49_full_list} , '1')*<br>*Response constrained to: .>0* | |
| <p><strong><em>D. Medications (4)</em></strong></p><br>*Group relevant when: selected( ${section_two_skip_med} , '1')* | | |
| q204_med_note4 | 204 CONTINUED For each of the following medications and other medical products that are used for incomplete abortion, can you tell me …? | |
| note_204_antiseptic | ***Antiseptic*** | |
| q204_M50 | ***q204_M50. Cetrimide 15% ("Savlon")***<br>*Question relevant when: selected( ${q104_M50_full_list} , '1')* | |
| q204_M50a *(required)* | q204_M50a. What percent of patients require this item?<br>*Question relevant when: selected( ${q104_M50_full_list} , '1')*<br>*Response constrained to: .>0 and .<=100 or .=999* | |
| q204_M50b *(required)* | q204_M50b. How is the item administered, or given, to the patient?<br>*Question relevant when: selected( ${q104_M50_full_list} , '1')* | 1 Oral - solid (e.g. tablet)<br>2 Oral - liquid<br>3 Sublingual<br>4 Intravenous (IV)<br>5 Intramusculuar injection (IM)<br>6 Subcutaneous<br>7 Rectal<br>8 Inhaled (e.g. gas)<br>9 Inserted (e.g. vaginal pessaries)<br>10 Topical - cream<br>11 Topical - drops (e.g. eye drops)<br>12 Other<br>99 Don't know |

| Field | Question | Answer |
|---|---|---|
| q204_M50c *(required)* | q204_M50c. What is the item's smallest unit of measurement (mg, mcg, ml, g, etc)?<br>*Question relevant when: selected( ${q104_M50_full_list} , '1')* | microgram — microgram (mcg)<br>miligram — miligram (mg)<br>miligram_dissolved_in_liquid — miligram (mg) dissolved in liquid (e.g. 2 mg per 3 militers)<br>gram — gram (g)<br>militer — militer (ml)<br>centiliter — centiliter (cl)<br>litre — litre (l)<br>ml_of _solution — ml of solution (e.g. ml of 10% solution)<br>litre_of_solution — litre of solution (e.g. 1 litre of 5% solution)<br>cubic_meter — cubic meter (m^3)<br>other — other<br>dont_know — don't know |
| q204_M50c.1 | q204_M50c.1 If other unit of measurement or route of administration, specify here:<br>*Leave blank if not applicable*<br>*Question relevant when: selected( ${q104_M50_full_list} , '1')* | |
| q204_M50d *(required)* | q204_M50d. In total, how many units are used to treat a patient during all of her care?<br>*Remember to add all units! E.g. 5 days x 200 mg twice per day = 2,000mg*<br>*Question relevant when: selected( ${q104_M50_full_list} , '1')*<br>*Response constrained to: .>0* | |
| q204_M51 | ***q204_M51. Povidone-iodine solution, 7.5%***<br>*Question relevant when: selected( ${q104_M51_full_list} , '1')* | |
| q204_M51a *(required)* | q204_M51a. What percent of patients require this item?<br>*Question relevant when: selected( ${q104_M51_full_list} , '1')*<br>*Response constrained to: .>0 and .<=100 or .=999* | |
| q204_M51b *(required)* | q204_M51b. How is the item administered, or given, to the patient?<br>*Question relevant when: selected( ${q104_M51_full_list} , '1')* | 1 Oral - solid (e.g. tablet)<br>2 Oral - liquid<br>3 Sublingual<br>4 Intravenous (IV)<br>5 Intramusculuar injection (IM)<br>6 Subcutaneous<br>7 Rectal<br>8 Inhaled (e.g. gas)<br>9 Inserted (e.g. vaginal pessaries)<br>10 Topical - cream<br>11 Topical - drops (e.g. eye drops)<br>12 Other<br>99 Don't know |

| Field | Question | Answer |
|---|---|---|
| q204_M51c *(required)* | q204_M51c. What is the item's smallest unit of measurement (mg, mcg, ml, g, etc)?<br>*Question relevant when: selected( ${q104_M51_full_list} , '1')* | microgram — microgram (mcg)<br>miligram — miligram (mg)<br>miligram_dissolved_in_liquid — miligram (mg) dissolved in liquid (e.g. 2 mg per 3 militers)<br>gram — gram (g)<br>militer — militer (ml)<br>centiliter — centiliter (cl)<br>litre — litre (l)<br>ml_of _solution — ml of solution (e.g. ml of 10% solution)<br>litre_of_solution — litre of solution (e.g. 1 litre of 5% solution)<br>cubic_meter — cubic meter (m^3)<br>other — other<br>dont_know — don't know |
| q204_M51c.1 | q204_M51c.1 If other unit of measurement or route of administration, specify here:<br>*Leave blank if not applicable*<br>*Question relevant when: selected( ${q104_M51_full_list} , '1')* | |
| q204_M51d *(required)* | q204_M51d. In total, how many units are used to treat a patient during all of her care?<br>*Remember to add all units! E.g. 5 days x 200 mg twice per day = 2,000mg*<br>*Question relevant when: selected( ${q104_M51_full_list} , '1')*<br>*Response constrained to: .>0* | |
| q204_M52 | ***q204_M52. Antiseptic - other 1: "[q104_M52_full_list_other]"***<br>*Question relevant when: selected( ${q104_M52_full_list} , '1')* | |
| q204_M52a *(required)* | q204_M52a. What percent of patients require this item?<br>*Question relevant when: selected( ${q104_M52_full_list} , '1')*<br>*Response constrained to: .>0 and .<=100 or .=999* | |
| q204_M52b *(required)* | q204_M52b. How is the item administered, or given, to the patient?<br>*Question relevant when: selected( ${q104_M52_full_list} , '1')* | 1 Oral - solid (e.g. tablet)<br>2 Oral - liquid<br>3 Sublingual<br>4 Intravenous (IV)<br>5 Intramusculuar injection (IM)<br>6 Subcutaneous<br>7 Rectal<br>8 Inhaled (e.g. gas)<br>9 Inserted (e.g. vaginal pessaries)<br>10 Topical - cream<br>11 Topical - drops (e.g. eye drops)<br>12 Other<br>99 Don't know |

| Field | Question | Answer |
|---|---|---|
| q204_M52c *(required)* | q204_M52c. What is the item's smallest unit of measurement (mg, mcg, ml, g, etc)? *Question relevant when: selected( ${q104_M52_full_list} , '1')* | microgram: microgram (mcg)<br>miligram: miligram (mg)<br>miligram_dissolved_in_liquid: miligram (mg) dissolved in liquid (e.g. 2 mg per 3 militers)<br>gram: gram (g)<br>militer: militer (ml)<br>centiliter: centiliter (cl)<br>litre: litre (l)<br>ml_of _solution: ml of solution (e.g. ml of 10% solution)<br>litre_of_solution: litre of solution (e.g. 1 litre of 5% solution)<br>cubic_meter: cubic meter (m^3)<br>other: other<br>dont_know: don't know |
| q204_M52c.1 | q204_M52c.1 If other unit of measurement or route of administration, specify here: *Leave blank if not applicable* *Question relevant when: selected( ${q104_M52_full_list} , '1')* | |
| q204_M52d *(required)* | q204_M52d. In total, how many units are used to treat a patient during all of her care? *Remember to add all units! E.g. 5 days x 200 mg twice per day = 2,000mg* *Question relevant when: selected( ${q104_M52_full_list} , '1')* *Response constrained to: .>0* | |
| q204_M53 | ***q204_M53. Antiseptic - other 2: "[q104_M53_full_list_other]"*** *Question relevant when: selected( ${q104_M53_full_list} , '1')* | |
| q204_M53a *(required)* | q204_M53a. What percent of patients require this item? *Question relevant when: selected( ${q104_M53_full_list} , '1')* *Response constrained to: .>0 and .<=100 or .=999* | |
| q204_M53b *(required)* | q204_M53b. How is the item administered, or given, to the patient? *Question relevant when: selected( ${q104_M53_full_list} , '1')* | 1 Oral - solid (e.g. tablet)<br>2 Oral - liquid<br>3 Sublingual<br>4 Intravenous (IV)<br>5 Intramusculuar injection (IM)<br>6 Subcutaneous<br>7 Rectal<br>8 Inhaled (e.g. gas)<br>9 Inserted (e.g. vaginal pessaries)<br>10 Topical - cream<br>11 Topical - drops (e.g. eye drops)<br>12 Other<br>99 Don't know |

| Field | Question | Answer |
|---|---|---|
| q204_M53c *(required)* | q204_M53c. What is the item's smallest unit of measurement (mg, mcg, ml, g, etc)?<br>*Question relevant when: selected( ${q104_M53_full_list} , '1')* | microgram — microgram (mcg)<br>miligram — miligram (mg)<br>miligram_dissolved_in_liquid — miligram (mg) dissolved in liquid (e.g. 2 mg per 3 militers)<br>gram — gram (g)<br>militer — militer (ml)<br>centiliter — centiliter (cl)<br>litre — litre (l)<br>ml_of _solution — ml of solution (e.g. ml of 10% solution)<br>litre_of_solution — litre of solution (e.g. 1 litre of 5% solution)<br>cubic_meter — cubic meter (m^3)<br>other — other<br>dont_know — don't know |
| q204_M53c.1 | q204_M53c.1 If other unit of measurement or route of administration, specify here:<br>*Leave blank if not applicable*<br>*Question relevant when: selected( ${q104_M53_full_list} , '1')* | |
| q204_M53d *(required)* | q204_M53d. In total, how many units are used to treat a patient during all of her care?<br>*Remember to add all units! E.g. 5 days x 200 mg twice per day = 2,000mg*<br>*Question relevant when: selected( ${q104_M53_full_list} , '1')*<br>*Response constrained to: .>0* | |
| q204_M54 | ***q204_M54. Antiseptic - other 3: "[q104_M54_full_list_other]"***<br>*Question relevant when: selected( ${q104_M54_full_list} , '1')* | |
| q204_M54a *(required)* | q204_M54a. What percent of patients require this item?<br>*Question relevant when: selected( ${q104_M54_full_list} , '1')*<br>*Response constrained to: .>0 and .<=100 or .=999* | |
| q204_M54b *(required)* | q204_M54b. How is the item administered, or given, to the patient?<br>*Question relevant when: selected( ${q104_M54_full_list} , '1')* | 1 Oral - solid (e.g. tablet)<br>2 Oral - liquid<br>3 Sublingual<br>4 Intravenous (IV)<br>5 Intramusculuar injection (IM)<br>6 Subcutaneous<br>7 Rectal<br>8 Inhaled (e.g. gas)<br>9 Inserted (e.g. vaginal pessaries)<br>10 Topical - cream<br>11 Topical - drops (e.g. eye drops)<br>12 Other<br>99 Don't know |

| Field | Question | Answer |
|---|---|---|
| q204_M54c *(required)* | q204_M54c. What is the item's smallest unit of measurement (mg, mcg, ml, g, etc)? *Question relevant when: selected( ${q104_M54_full_list} , '1')* | microgram — microgram (mcg)<br>miligram — miligram (mg)<br>miligram_dissolved_in_liquid — miligram (mg) dissolved in liquid (e.g. 2 mg per 3 militers)<br>gram — gram (g)<br>militer — militer (ml)<br>centiliter — centiliter (cl)<br>litre — litre (l)<br>ml_of _solution — ml of solution (e.g. ml of 10% solution)<br>litre_of_solution — litre of solution (e.g. 1 litre of 5% solution)<br>cubic_meter — cubic meter (m^3)<br>other — other<br>dont_know — don't know |
| q204_M54c.1 | q204_M54c.1 If other unit of measurement or route of administration, specify here: *Leave blank if not applicable* *Question relevant when: selected( ${q104_M54_full_list} , '1')* | |
| q204_M54d *(required)* | q204_M54d. In total, how many units are used to treat a patient during all of her care? *Remember to add all units! E.g. 5 days x 200 mg twice per day = 2,000mg* *Question relevant when: selected( ${q104_M54_full_list} , '1')* *Response constrained to: .>0* | |
| q204_M54.1 | ***q204_M54.1. Antiseptic - other 4: "[q104_M54.1_full_list_other]"*** *Question relevant when: selected( ${q104_M54.1_full_list} , '1')* | |
| q204_M54.1a *(required)* | q204_M54.1a. What percent of patients require this item? *Question relevant when: selected( ${q104_M54.1_full_list} , '1')* *Response constrained to: .>0 and .<=100 or .=999* | |
| q204_M54.1b *(required)* | q204_M54.1b. How is the item administered, or given, to the patient? *Question relevant when: selected( ${q104_M54.1_full_list} , '1')* | 1 Oral - solid (e.g. tablet)<br>2 Oral - liquid<br>3 Sublingual<br>4 Intravenous (IV)<br>5 Intramusculuar injection (IM)<br>6 Subcutaneous<br>7 Rectal<br>8 Inhaled (e.g. gas)<br>9 Inserted (e.g. vaginal pessaries)<br>10 Topical - cream<br>11 Topical - drops (e.g. eye drops)<br>12 Other<br>99 Don't know |

| Field | Question | Answer |
|---|---|---|
| q204_M54.1c *(required)* | q204_M54.1c. What is the item's smallest unit of measurement (mg, mcg, ml, g, etc)?<br>*Question relevant when: selected( ${q104_M54.1_full_list} , '1')* | microgram — microgram (mcg)<br>miligram — miligram (mg)<br>miligram_dissolved_in_liquid — miligram (mg) dissolved in liquid (e.g. 2 mg per 3 militers)<br>gram — gram (g)<br>militer — militer (ml)<br>centiliter — centiliter (cl)<br>litre — litre (l)<br>ml_of _solution — ml of solution (e.g. ml of 10% solution)<br>litre_of_solution — litre of solution (e.g. 1 litre of 5% solution)<br>cubic_meter — cubic meter (m^3)<br>other — other<br>dont_know — don't know |
| q204_M54.1c.1 | q204_M54.1c.1 If other unit of measurement or route of administration, specify here:<br>*Leave blank if not applicable*<br>*Question relevant when: selected( ${q104_M54.1_full_list} , '1')* | |
| q204_M54.1d *(required)* | q204_M54.1d. In total, how many units are used to treat a patient during all of her care?<br>*Remember to add all units! E.g. 5 days x 200 mg twice per day = 2,000mg*<br>*Question relevant when: selected( ${q104_M54.1_full_list} , '1')*<br>*Response constrained to: .>0* | |
| q204_M54.2 | ***q204_M54.2. Antiseptic - other 5: "[q104_M54.2_full_list_other]"***<br>*Question relevant when: selected( ${q104_M54.2_full_list} , '1')* | |
| q204_M54.2a *(required)* | q204_M54.2a. What percent of patients require this item?<br>*Question relevant when: selected( ${q104_M54.2_full_list} , '1')*<br>*Response constrained to: .>0 and .<=100 or .=999* | |
| q204_M54.2b *(required)* | q204_M54.2b. How is the item administered, or given, to the patient?<br>*Question relevant when: selected( ${q104_M54.2_full_list} , '1')* | 1 Oral - solid (e.g. tablet)<br>2 Oral - liquid<br>3 Sublingual<br>4 Intravenous (IV)<br>5 Intramusculuar injection (IM)<br>6 Subcutaneous<br>7 Rectal<br>8 Inhaled (e.g. gas)<br>9 Inserted (e.g. vaginal pessaries)<br>10 Topical - cream<br>11 Topical - drops (e.g. eye drops)<br>12 Other<br>99 Don't know |

| Field | Question | Answer |
|---|---|---|
| q204_M54.2c *(required)* | q204_M54.2c. What is the item's smallest unit of measurement (mg, mcg, ml, g, etc)?<br>*Question relevant when: selected( ${q104_M54.2_full_list} , '1')* | | microgram | microgram (mcg) |<br>| | miligram | miligram (mg) |<br>| | miligram_dissolved_in_liquid | miligram (mg) dissolved in liquid (e.g. 2 mg per 3 militers) |<br>| | gram | gram (g) |<br>| | militer | militer (ml) |<br>| | centiliter | centiliter (cl) |<br>| | litre | litre (l) |<br>| | ml_of _solution | ml of solution (e.g. ml of 10% solution) |<br>| | litre_of_solution | litre of solution (e.g. 1 litre of 5% solution) |<br>| | cubic_meter | cubic meter (m^3) |<br>| | other | other |<br>| | dont_know | don't know |<br> |
| q204_M54.2c.1 | q204_M54.2c.1 If other unit of measurement or route of administration, specify here:<br>*Leave blank if not applicable*<br>*Question relevant when: selected( ${q104_M54.2_full_list} , '1')* | |
| q204_M54.2d *(required)* | q204_M54.2d. In total, how many units are used to treat a patient during all of her care?<br>*Remember to add all units! E.g. 5 days x 200 mg twice per day = 2,000mg*<br>*Question relevant when: selected( ${q104_M54.2_full_list} , '1')*<br>*Response constrained to: .>0* | |
| <p><strong><em>D. Medications (5)</em></strong></p><br>*Group relevant when: selected( ${section_two_skip_med} , '1')* | | |
| q204_med_notE4 | 204 CONTINUED For each of the following medications and other medical products that are used for incomplete abortion, can you tell me …? | |
| note_204_antispas | ***Antispasmodic, anxiolytic, sedative, etc*** | |
| q204_M55 | ***q204_M55. Hyoscine butylbromide / scopolamine butilhisocin (Buscopan )***<br>*Question relevant when: selected( ${q104_M55_full_list} , '1')* | |
| q204_M55a *(required)* | q204_M55a. What percent of patients require this item?<br>*Question relevant when: selected( ${q104_M55_full_list} , '1')*<br>*Response constrained to: .>0 and .<=100 or .=999* | |
| q204_M55b *(required)* | q204_M55b. How is the item administered, or given, to the patient?<br>*Question relevant when: selected( ${q104_M55_full_list} , '1')* | 1 Oral - solid (e.g. tablet)<br>2 Oral - liquid<br>3 Sublingual<br>4 Intravenous (IV)<br>5 Intramusculuar injection (IM)<br>6 Subcutaneous<br>7 Rectal<br>8 Inhaled (e.g. gas)<br>9 Inserted (e.g. vaginal pessaries)<br>10 Topical - cream<br>11 Topical - drops (e.g. eye drops)<br>12 Other<br>99 Don't know |

| Field | Question | Answer |
|---|---|---|
| q204_M55c *(required)* | q204_M55c. What is the item's smallest unit of measurement (mg, mcg, ml, g, etc)?<br>*Question relevant when: selected( ${q104_M55_full_list} , '1')* | microgram: microgram (mcg)<br>miligram: miligram (mg)<br>miligram_dissolved_in_liquid: miligram (mg) dissolved in liquid (e.g. 2 mg per 3 militers)<br>gram: gram (g)<br>militer: militer (ml)<br>centiliter: centiliter (cl)<br>litre: litre (l)<br>ml_of _solution: ml of solution (e.g. ml of 10% solution)<br>litre_of_solution: litre of solution (e.g. 1 litre of 5% solution)<br>cubic_meter: cubic meter (m^3)<br>other: other<br>dont_know: don't know |
| q204_M55c.1 | q204_M55c.1 If other unit of measurement or route of administration, specify here:<br>*Leave blank if not applicable*<br>*Question relevant when: selected( ${q104_M55_full_list} , '1')* | |
| q204_M55d *(required)* | q204_M55d. In total, how many units are used to treat a patient during all of her care?<br>*Remember to add all units! E.g. 5 days x 200 mg twice per day = 2,000mg*<br>*Question relevant when: selected( ${q104_M55_full_list} , '1')*<br>*Response constrained to: .>0* | |
| q204_M56 | ***q204_M56. Atropine sulfate (or sulphate)***<br>*Question relevant when: selected( ${q104_M56_full_list} , '1')* | |
| q204_M56a *(required)* | q204_M56a. What percent of patients require this item?<br>*Question relevant when: selected( ${q104_M56_full_list} , '1')*<br>*Response constrained to: .>0 and .<=100 or .=999* | |
| q204_M56b *(required)* | q204_M56b. How is the item administered, or given, to the patient?<br>*Question relevant when: selected( ${q104_M56_full_list} , '1')* | 1 Oral - solid (e.g. tablet)<br>2 Oral - liquid<br>3 Sublingual<br>4 Intravenous (IV)<br>5 Intramusculuar injection (IM)<br>6 Subcutaneous<br>7 Rectal<br>8 Inhaled (e.g. gas)<br>9 Inserted (e.g. vaginal pessaries)<br>10 Topical - cream<br>11 Topical - drops (e.g. eye drops)<br>12 Other<br>99 Don't know |

| Field | Question | Answer |
|---|---|---|
| q204_M56c *(required)* | q204_M56c. What is the item's smallest unit of measurement (mg, mcg, ml, g, etc)?<br>*Question relevant when: selected( ${q104_M56_full_list} , '1')* | microgram — microgram (mcg)<br>miligram — miligram (mg)<br>miligram_dissolved_in_liquid — miligram (mg) dissolved in liquid (e.g. 2 mg per 3 militers)<br>gram — gram (g)<br>militer — militer (ml)<br>centiliter — centiliter (cl)<br>litre — litre (l)<br>ml_of _solution — ml of solution (e.g. ml of 10% solution)<br>litre_of_solution — litre of solution (e.g. 1 litre of 5% solution)<br>cubic_meter — cubic meter (m^3)<br>other — other<br>dont_know — don't know |
| q204_M56c.1 | q204_M56c.1 If other unit of measurement or route of administration, specify here:<br>*Leave blank if not applicable*<br>*Question relevant when: selected( ${q104_M56_full_list} , '1')* | |
| q204_M56d *(required)* | q204_M56d. In total, how many units are used to treat a patient during all of her care?<br>*Remember to add all units! E.g. 5 days x 200 mg twice per day = 2,000mg*<br>*Question relevant when: selected( ${q104_M56_full_list} , '1')*<br>*Response constrained to: .>0* | |
| q204_M57 | ***q204_M57. Papaverine HCl***<br>*Question relevant when: selected( ${q104_M57_full_list} , '1')* | |
| q204_M57a *(required)* | q204_M57a. What percent of patients require this item?<br>*Question relevant when: selected( ${q104_M57_full_list} , '1')*<br>*Response constrained to: .>0 and .<=100 or .=999* | |
| q204_M57b *(required)* | q204_M57b. How is the item administered, or given, to the patient?<br>*Question relevant when: selected( ${q104_M57_full_list} , '1')* | 1 Oral - solid (e.g. tablet)<br>2 Oral - liquid<br>3 Sublingual<br>4 Intravenous (IV)<br>5 Intramusculuar injection (IM)<br>6 Subcutaneous<br>7 Rectal<br>8 Inhaled (e.g. gas)<br>9 Inserted (e.g. vaginal pessaries)<br>10 Topical - cream<br>11 Topical - drops (e.g. eye drops)<br>12 Other<br>99 Don't know |

| Field | Question | Answer |
|---|---|---|
| q204_M57c *(required)* | q204_M57c. What is the item's smallest unit of measurement (mg, mcg, ml, g, etc)?<br>*Question relevant when: selected( ${q104_M57_full_list} , '1')* | microgram — microgram (mcg)<br>miligram — miligram (mg)<br>miligram_dissolved_in_liquid — miligram (mg) dissolved in liquid (e.g. 2 mg per 3 militers)<br>gram — gram (g)<br>militer — militer (ml)<br>centiliter — centiliter (cl)<br>litre — litre (l)<br>ml_of _solution — ml of solution (e.g. ml of 10% solution)<br>litre_of_solution — litre of solution (e.g. 1 litre of 5% solution)<br>cubic_meter — cubic meter (m^3)<br>other — other<br>dont_know — don't know |
| q204_M57c.1 | q204_M57c.1 If other unit of measurement or route of administration, specify here:<br>*Leave blank if not applicable*<br>*Question relevant when: selected( ${q104_M57_full_list} , '1')* | |
| q204_M57d *(required)* | q204_M57d. In total, how many units are used to treat a patient during all of her care?<br>*Remember to add all units! E.g. 5 days x 200 mg twice per day = 2,000mg*<br>*Question relevant when: selected( ${q104_M57_full_list} , '1')*<br>*Response constrained to: .>0* | |
| q204_M58 | ***q204_M58. Diazepam (injection)***<br>*Question relevant when: selected( ${q104_M58_full_list} , '1')* | |
| q204_M58a *(required)* | q204_M58a. What percent of patients require this item?<br>*Question relevant when: selected( ${q104_M58_full_list} , '1')*<br>*Response constrained to: .>0 and .<=100 or .=999* | |
| q204_M58b *(required)* | q204_M58b. How is the item administered, or given, to the patient?<br>*Question relevant when: selected( ${q104_M58_full_list} , '1')* | 1 Oral - solid (e.g. tablet)<br>2 Oral - liquid<br>3 Sublingual<br>4 Intravenous (IV)<br>5 Intramusculuar injection (IM)<br>6 Subcutaneous<br>7 Rectal<br>8 Inhaled (e.g. gas)<br>9 Inserted (e.g. vaginal pessaries)<br>10 Topical - cream<br>11 Topical - drops (e.g. eye drops)<br>12 Other<br>99 Don't know |

| Field | Question | Answer |
|---|---|---|
| q204_M58c *(required)* | q204_M58c. What is the item's smallest unit of measurement (mg, mcg, ml, g, etc)?<br>*Question relevant when: selected( ${q104_M58_full_list} , '1')* | microgram — microgram (mcg)<br>miligram — miligram (mg)<br>miligram_dissolved_in_liquid — miligram (mg) dissolved in liquid (e.g. 2 mg per 3 militers)<br>gram — gram (g)<br>militer — militer (ml)<br>centiliter — centiliter (cl)<br>litre — litre (l)<br>ml_of _solution — ml of solution (e.g. ml of 10% solution)<br>litre_of_solution — litre of solution (e.g. 1 litre of 5% solution)<br>cubic_meter — cubic meter (m^3)<br>other — other<br>dont_know — don't know |
| q204_M58c.1 | q204_M58c.1 If other unit of measurement or route of administration, specify here:<br>*Leave blank if not applicable*<br>*Question relevant when: selected( ${q104_M58_full_list} , '1')* | |
| q204_M58d *(required)* | q204_M58d. In total, how many units are used to treat a patient during all of her care?<br>*Remember to add all units! E.g. 5 days x 200 mg twice per day = 2,000mg*<br>*Question relevant when: selected( ${q104_M58_full_list} , '1')*<br>*Response constrained to: .>0* | |
| q204_M59 | ***q204_M59. Diazepam (oral)***<br>*Question relevant when: selected( ${q104_M59_full_list} , '1')* | |
| q204_M59a *(required)* | q204_M59a. What percent of patients require this item?<br>*Question relevant when: selected( ${q104_M59_full_list} , '1')*<br>*Response constrained to: .>0 and .<=100 or .=999* | |
| q204_M59b *(required)* | q204_M59b. How is the item administered, or given, to the patient?<br>*Question relevant when: selected( ${q104_M59_full_list} , '1')* | 1 Oral - solid (e.g. tablet)<br>2 Oral - liquid<br>3 Sublingual<br>4 Intravenous (IV)<br>5 Intramusculuar injection (IM)<br>6 Subcutaneous<br>7 Rectal<br>8 Inhaled (e.g. gas)<br>9 Inserted (e.g. vaginal pessaries)<br>10 Topical - cream<br>11 Topical - drops (e.g. eye drops)<br>12 Other<br>99 Don't know |

| Field | Question | Answer |
|---|---|---|
| q204_M59c *(required)* | q204_M59c. What is the item's smallest unit of measurement (mg, mcg, ml, g, etc)?<br>*Question relevant when: selected( ${q104_M59_full_list} , '1')* | microgram — microgram (mcg)<br>miligram — miligram (mg)<br>miligram_dissolved_in_liquid — miligram (mg) dissolved in liquid (e.g. 2 mg per 3 militers)<br>gram — gram (g)<br>militer — militer (ml)<br>centiliter — centiliter (cl)<br>litre — litre (l)<br>ml_of _solution — ml of solution (e.g. ml of 10% solution)<br>litre_of_solution — litre of solution (e.g. 1 litre of 5% solution)<br>cubic_meter — cubic meter (m^3)<br>other — other<br>dont_know — don't know |
| q204_M59c.1 | q204_M59c.1 If other unit of measurement or route of administration, specify here:<br>*Leave blank if not applicable*<br>*Question relevant when: selected( ${q104_M59_full_list} , '1')* | |
| q204_M59d *(required)* | q204_M59d. In total, how many units are used to treat a patient during all of her care?<br>*Remember to add all units! E.g. 5 days x 200 mg twice per day = 2,000mg*<br>*Question relevant when: selected( ${q104_M59_full_list} , '1')*<br>*Response constrained to: .>0* | |
| q204_M60 | ***q204_M60. Antispasmodic, anxiolytic, sedative, etc - Other 1: "[q104_M60_full_list_other]"***<br>*Question relevant when: selected( ${q104_M60_full_list} , '1')* | |
| q204_M60a *(required)* | q204_M60a. What percent of patients require this item?<br>*Question relevant when: selected( ${q104_M60_full_list} , '1')*<br>*Response constrained to: .>0 and .<=100 or .=999* | |
| q204_M60b *(required)* | q204_M60b. How is the item administered, or given, to the patient?<br>*Question relevant when: selected( ${q104_M60_full_list} , '1')* | 1 Oral - solid (e.g. tablet)<br>2 Oral - liquid<br>3 Sublingual<br>4 Intravenous (IV)<br>5 Intramusculuar injection (IM)<br>6 Subcutaneous<br>7 Rectal<br>8 Inhaled (e.g. gas)<br>9 Inserted (e.g. vaginal pessaries)<br>10 Topical - cream<br>11 Topical - drops (e.g. eye drops)<br>12 Other<br>99 Don't know |

| Field | Question | Answer |
|---|---|---|
| q204_M60c *(required)* | q204_M60c. What is the item's smallest unit of measurement (mg, mcg, ml, g, etc)?<br>*Question relevant when: selected( ${q104_M60_full_list} , '1')* | microgram — microgram (mcg)<br>miligram — miligram (mg)<br>miligram_dissolved_in_liquid — miligram (mg) dissolved in liquid (e.g. 2 mg per 3 militers)<br>gram — gram (g)<br>militer — militer (ml)<br>centiliter — centiliter (cl)<br>litre — litre (l)<br>ml_of _solution — ml of solution (e.g. ml of 10% solution)<br>litre_of_solution — litre of solution (e.g. 1 litre of 5% solution)<br>cubic_meter — cubic meter (m^3)<br>other — other<br>dont_know — don't know |
| q204_M60c.1 | q204_M60c.1 If other unit of measurement or route of administration, specify here:<br>*Leave blank if not applicable*<br>*Question relevant when: selected( ${q104_M60_full_list} , '1')* | |
| q204_M60d *(required)* | q204_M60d. In total, how many units are used to treat a patient during all of her care?<br>*Remember to add all units! E.g. 5 days x 200 mg twice per day = 2,000mg*<br>*Question relevant when: selected( ${q104_M60_full_list} , '1')*<br>*Response constrained to: .>0* | |
| q204_M61 | ***q204_M61. Antispasmodic, anxiolytic, sedative, etc - Other 2: "[q104_M61_full_list_other]"***<br>*Question relevant when: selected( ${q104_M61_full_list} , '1')* | |
| q204_M61a *(required)* | q204_M61a. What percent of patients require this item?<br>*Question relevant when: selected( ${q104_M61_full_list} , '1')*<br>*Response constrained to: .>0 and .<=100 or .=999* | |
| q204_M61b *(required)* | q204_M61b. How is the item administered, or given, to the patient?<br>*Question relevant when: selected( ${q104_M61_full_list} , '1')* | 1 Oral - solid (e.g. tablet)<br>2 Oral - liquid<br>3 Sublingual<br>4 Intravenous (IV)<br>5 Intramusculuar injection (IM)<br>6 Subcutaneous<br>7 Rectal<br>8 Inhaled (e.g. gas)<br>9 Inserted (e.g. vaginal pessaries)<br>10 Topical - cream<br>11 Topical - drops (e.g. eye drops)<br>12 Other<br>99 Don't know |

| Field | Question | Answer |
|---|---|---|
| q204_M61c *(required)* | q204_M61c. What is the item's smallest unit of measurement (mg, mcg, ml, g, etc)?<br>*Question relevant when: selected( ${q104_M61_full_list} , '1')* | microgram — microgram (mcg)<br>miligram — miligram (mg)<br>miligram_dissolved_in_liquid — miligram (mg) dissolved in liquid (e.g. 2 mg per 3 militers)<br>gram — gram (g)<br>militer — militer (ml)<br>centiliter — centiliter (cl)<br>litre — litre (l)<br>ml_of _solution — ml of solution (e.g. ml of 10% solution)<br>litre_of_solution — litre of solution (e.g. 1 litre of 5% solution)<br>cubic_meter — cubic meter (m^3)<br>other — other<br>dont_know — don't know |
| q204_M61c.1 | q204_M61c.1 If other unit of measurement or route of administration, specify here:<br>*Leave blank if not applicable*<br>*Question relevant when: selected( ${q104_M61_full_list} , '1')* | |
| q204_M61d *(required)* | q204_M61d. In total, how many units are used to treat a patient during all of her care?<br>*Remember to add all units! E.g. 5 days x 200 mg twice per day = 2,000mg*<br>*Question relevant when: selected( ${q104_M61_full_list} , '1')*<br>*Response constrained to: .>0* | |
| q204_M62 | ***q204_M62. Antispasmodic, anxiolytic, sedative, etc - Other 3: "[q104_M62_full_list_other]"***<br>*Question relevant when: selected( ${q104_M62_full_list} , '1')* | |
| q204_M62a *(required)* | q204_M62a. What percent of patients require this item?<br>*Question relevant when: selected( ${q104_M62_full_list} , '1')*<br>*Response constrained to: .>0 and .<=100 or .=999* | |
| q204_M62b *(required)* | q204_M62b. How is the item administered, or given, to the patient?<br>*Question relevant when: selected( ${q104_M62_full_list} , '1')* | 1 Oral - solid (e.g. tablet)<br>2 Oral - liquid<br>3 Sublingual<br>4 Intravenous (IV)<br>5 Intramusculuar injection (IM)<br>6 Subcutaneous<br>7 Rectal<br>8 Inhaled (e.g. gas)<br>9 Inserted (e.g. vaginal pessaries)<br>10 Topical - cream<br>11 Topical - drops (e.g. eye drops)<br>12 Other<br>99 Don't know |

| Field | Question | Answer |
|---|---|---|
| q204_M62c *(required)* | q204_M62c. What is the item's smallest unit of measurement (mg, mcg, ml, g, etc)?<br>*Question relevant when: selected( ${q104_M62_full_list} , '1')* | microgram — microgram (mcg)<br>miligram — miligram (mg)<br>miligram_dissolved_in_liquid — miligram (mg) dissolved in liquid (e.g. 2 mg per 3 militers)<br>gram — gram (g)<br>militer — militer (ml)<br>centiliter — centiliter (cl)<br>litre — litre (l)<br>ml_of _solution — ml of solution (e.g. ml of 10% solution)<br>litre_of_solution — litre of solution (e.g. 1 litre of 5% solution)<br>cubic_meter — cubic meter (m^3)<br>other — other<br>dont_know — don't know |
| q204_M62c.1 | q204_M62c.1 If other unit of measurement or route of administration, specify here:<br>*Leave blank if not applicable*<br>*Question relevant when: selected( ${q104_M62_full_list} , '1')* | |
| q204_M62d *(required)* | q204_M62d. In total, how many units are used to treat a patient during all of her care?<br>*Remember to add all units! E.g. 5 days x 200 mg twice per day = 2,000mg*<br>*Question relevant when: selected( ${q104_M62_full_list} , '1')*<br>*Response constrained to: .>0* | |
| q204_M63 | ***q204_M63. Antispasmodic, anxiolytic, sedative, etc - Other 4: "[q104_M63_full_list_other]"***<br>*Question relevant when: selected( ${q104_M63_full_list} , '1')* | |
| q204_M63a *(required)* | q204_M63a. What percent of patients require this item?<br>*Question relevant when: selected( ${q104_M63_full_list} , '1')*<br>*Response constrained to: .>0 and .<=100 or .=999* | |
| q204_M63b *(required)* | q204_M63b. How is the item administered, or given, to the patient?<br>*Question relevant when: selected( ${q104_M63_full_list} , '1')* | 1 Oral - solid (e.g. tablet)<br>2 Oral - liquid<br>3 Sublingual<br>4 Intravenous (IV)<br>5 Intramusculuar injection (IM)<br>6 Subcutaneous<br>7 Rectal<br>8 Inhaled (e.g. gas)<br>9 Inserted (e.g. vaginal pessaries)<br>10 Topical - cream<br>11 Topical - drops (e.g. eye drops)<br>12 Other<br>99 Don't know |

| Field | Question | Answer |
|---|---|---|
| q204_M63c *(required)* | q204_M63c. What is the item's smallest unit of measurement (mg, mcg, ml, g, etc)?<br>*Question relevant when: selected( ${q104_M63_full_list} , '1')* | \| \| microgram \| microgram (mcg) \|<br>\| \| miligram \| miligram (mg) \|<br>\| \| miligram_dissolved_in_liquid \| miligram (mg) dissolved in liquid (e.g. 2 mg per 3 militers) \|<br>\| \| gram \| gram (g) \|<br>\| \| militer \| militer (ml) \|<br>\| \| centiliter \| centiliter (cl) \|<br>\| \| litre \| litre (l) \|<br>\| \| ml_of _solution \| ml of solution (e.g. ml of 10% solution) \|<br>\| \| litre_of_solution \| litre of solution (e.g. 1 litre of 5% solution) \|<br>\| \| cubic_meter \| cubic meter (m^3) \|<br>\| \| other \| other \|<br>\| \| dont_know \| don't know \| |
| q204_M63c.1 | q204_M63c.1 If other unit of measurement or route of administration, specify here:<br>*Leave blank if not applicable*<br>*Question relevant when: selected( ${q104_M63_full_list} , '1')* | |
| q204_M63d *(required)* | q204_M63d. In total, how many units are used to treat a patient during all of her care?<br>*Remember to add all units! E.g. 5 days x 200 mg twice per day = 2,000mg*<br>*Question relevant when: selected( ${q104_M63_full_list} , '1')*<br>*Response constrained to: .>0* | |
| q204_M64 | ***q204_M64. Antispasmodic, anxiolytic, sedative, etc - Other 5: "[q104_M64_full_list_other]"***<br>*Question relevant when: selected( ${q104_M64_full_list} , '1')* | |
| q204_M64a *(required)* | q204_M64a. What percent of patients require this item?<br>*Question relevant when: selected( ${q104_M64_full_list} , '1')*<br>*Response constrained to: .>0 and .<=100 or .=999* | |
| q204_M64b *(required)* | q204_M64b. How is the item administered, or given, to the patient?<br>*Question relevant when: selected( ${q104_M64_full_list} , '1')* | \| \| 1 \| Oral - solid (e.g. tablet) \|<br>\| \| 2 \| Oral - liquid \|<br>\| \| 3 \| Sublingual \|<br>\| \| 4 \| Intravenous (IV) \|<br>\| \| 5 \| Intramusculuar injection (IM) \|<br>\| \| 6 \| Subcutaneous \|<br>\| \| 7 \| Rectal \|<br>\| \| 8 \| Inhaled (e.g. gas) \|<br>\| \| 9 \| Inserted (e.g. vaginal pessaries) \|<br>\| \| 10 \| Topical - cream \|<br>\| \| 11 \| Topical - drops (e.g. eye drops) \|<br>\| \| 12 \| Other \|<br>\| \| 99 \| Don't know \| |

| Field | Question | Answer |
|---|---|---|
| q204_M64c *(required)* | q204_M64c. What is the item's smallest unit of measurement (mg, mcg, ml, g, etc)?<br>*Question relevant when: selected( ${q104_M64_full_list} , '1')* | microgram — microgram (mcg)<br>miligram — miligram (mg)<br>miligram_dissolved_in_liquid — miligram (mg) dissolved in liquid (e.g. 2 mg per 3 militers)<br>gram — gram (g)<br>militer — militer (ml)<br>centiliter — centiliter (cl)<br>litre — litre (l)<br>ml_of _solution — ml of solution (e.g. ml of 10% solution)<br>litre_of_solution — litre of solution (e.g. 1 litre of 5% solution)<br>cubic_meter — cubic meter (m^3)<br>other — other<br>dont_know — don't know |
| q204_M64c.1 | q204_M64c.1 If other unit of measurement or route of administration, specify here:<br>*Leave blank if not applicable*<br>*Question relevant when: selected( ${q104_M64_full_list} , '1')* | |
| q204_M64d *(required)* | q204_M64d. In total, how many units are used to treat a patient during all of her care?<br>*Remember to add all units! E.g. 5 days x 200 mg twice per day = 2,000mg*<br>*Question relevant when: selected( ${q104_M64_full_list} , '1')*<br>*Response constrained to: .>0* | |
| <p><strong><em>D. Medications (6)</em></strong></p><br>*Group relevant when: selected( ${section_two_skip_med} , '1')* | | |
| q204_med_notE5 | 204 CONTINUED For each of the following medications and other medical products that are used for incomplete abortion, can you tell me …? | |
| note_204_adrenergic | ***Adrenergic*** | |
| q204_M65 | ***q204_M65. Ephedrine HCl***<br>*Question relevant when: selected( ${q104_M65_full_list} , '1')* | |
| q204_M65a *(required)* | q204_M65a. What percent of patients require this item?<br>*Question relevant when: selected( ${q104_M65_full_list} , '1')*<br>*Response constrained to: .>0 and .<=100 or .=999* | |
| q204_M65b *(required)* | q204_M65b. How is the item administered, or given, to the patient?<br>*Question relevant when: selected( ${q104_M65_full_list} , '1')* | 1 Oral - solid (e.g. tablet)<br>2 Oral - liquid<br>3 Sublingual<br>4 Intravenous (IV)<br>5 Intramusculuar injection (IM)<br>6 Subcutaneous<br>7 Rectal<br>8 Inhaled (e.g. gas)<br>9 Inserted (e.g. vaginal pessaries)<br>10 Topical - cream<br>11 Topical - drops (e.g. eye drops)<br>12 Other |

| Field | Question | Answer |
|---|---|---|
| | | 99 Don't know |

| Field | Question | Answer |
|---|---|---|
| q204_M65c *(required)* | q204_M65c. What is the item's smallest unit of measurement (mg, mcg, ml, g, etc)? *Question relevant when: selected( ${q104_M65_full_list} , '1')* | microgram — microgram (mcg)<br>miligram — miligram (mg)<br>miligram_dissolved_in_liquid — miligram (mg) dissolved in liquid (e.g. 2 mg per 3 militers)<br>gram — gram (g)<br>militer — militer (ml)<br>centiliter — centiliter (cl)<br>litre — litre (l)<br>ml_of _solution — ml of solution (e.g. ml of 10% solution)<br>litre_of_solution — litre of solution (e.g. 1 litre of 5% solution)<br>cubic_meter — cubic meter (m^3)<br>other — other<br>dont_know — don't know |
| q204_M65c.1 | q204_M65c.1 If other unit of measurement or route of administration, specify here: *Leave blank if not applicable* *Question relevant when: selected( ${q104_M65_full_list} , '1')* | |
| q204_M65d *(required)* | q204_M65d. In total, how many units are used to treat a patient during all of her care? *Remember to add all units! E.g. 5 days x 200 mg twice per day = 2,000mg* *Question relevant when: selected( ${q104_M65_full_list} , '1')* *Response constrained to: .>0* | |
| q204_M66 | ***q204_M66. Epinephrine*** *Question relevant when: selected( ${q104_M66_full_list} , '1')* | |
| q204_M66a *(required)* | q204_M66a. What percent of patients require this item? *Question relevant when: selected( ${q104_M66_full_list} , '1')* *Response constrained to: .>0 and .<=100 or .=999* | |
| q204_M66b *(required)* | q204_M66b. How is the item administered, or given, to the patient? *Question relevant when: selected( ${q104_M66_full_list} , '1')* | 1 Oral - solid (e.g. tablet)<br>2 Oral - liquid<br>3 Sublingual<br>4 Intravenous (IV)<br>5 Intramusculuar injection (IM)<br>6 Subcutaneous<br>7 Rectal<br>8 Inhaled (e.g. gas)<br>9 Inserted (e.g. vaginal pessaries)<br>10 Topical - cream<br>11 Topical - drops (e.g. eye drops)<br>12 Other<br>99 Don't know |

| Field | Question | Answer |
|---|---|---|
| q204_M66c *(required)* | q204_M66c. What is the item's smallest unit of measurement (mg, mcg, ml, g, etc)?<br>*Question relevant when: selected( ${q104_M66_full_list} , '1')* | microgram — microgram (mcg)<br>miligram — miligram (mg)<br>miligram_dissolved_in_liquid — miligram (mg) dissolved in liquid (e.g. 2 mg per 3 militers)<br>gram — gram (g)<br>militer — militer (ml)<br>centiliter — centiliter (cl)<br>litre — litre (l)<br>ml_of _solution — ml of solution (e.g. ml of 10% solution)<br>litre_of_solution — litre of solution (e.g. 1 litre of 5% solution)<br>cubic_meter — cubic meter (m^3)<br>other — other<br>dont_know — don't know |
| q204_M66c.1 | q204_M66c.1 If other unit of measurement or route of administration, specify here:<br>*Leave blank if not applicable*<br>*Question relevant when: selected( ${q104_M66_full_list} , '1')* | |
| q204_M66d *(required)* | q204_M66d. In total, how many units are used to treat a patient during all of her care?<br>*Remember to add all units! E.g. 5 days x 200 mg twice per day = 2,000mg*<br>*Question relevant when: selected( ${q104_M66_full_list} , '1')*<br>*Response constrained to: .>0* | |
| q204_M67 | ***q204_M67. Adrenergic - other 1: "[q104_M67_full_list_other]"***<br>*Question relevant when: selected( ${q104_M67_full_list} , '1')* | |
| q204_M67a *(required)* | q204_M67a. What percent of patients require this item?<br>*Question relevant when: selected( ${q104_M67_full_list} , '1')*<br>*Response constrained to: .>0 and .<=100 or .=999* | |
| q204_M67b *(required)* | q204_M67b. How is the item administered, or given, to the patient?<br>*Question relevant when: selected( ${q104_M67_full_list} , '1')* | 1 Oral - solid (e.g. tablet)<br>2 Oral - liquid<br>3 Sublingual<br>4 Intravenous (IV)<br>5 Intramusculuar injection (IM)<br>6 Subcutaneous<br>7 Rectal<br>8 Inhaled (e.g. gas)<br>9 Inserted (e.g. vaginal pessaries)<br>10 Topical - cream<br>11 Topical - drops (e.g. eye drops)<br>12 Other<br>99 Don't know |

| Field | Question | Answer |
|---|---|---|
| q204_M67c *(required)* | q204_M67c. What is the item's smallest unit of measurement (mg, mcg, ml, g, etc)?<br>*Question relevant when: selected( ${q104_M67_full_list} , '1')* | microgram — microgram (mcg)<br>miligram — miligram (mg)<br>miligram_dissolved_in_liquid — miligram (mg) dissolved in liquid (e.g. 2 mg per 3 militers)<br>gram — gram (g)<br>militer — militer (ml)<br>centiliter — centiliter (cl)<br>litre — litre (l)<br>ml_of _solution — ml of solution (e.g. ml of 10% solution)<br>litre_of_solution — litre of solution (e.g. 1 litre of 5% solution)<br>cubic_meter — cubic meter (m^3)<br>other — other<br>dont_know — don't know |
| q204_M67c.1 | q204_M67c.1 If other unit of measurement or route of administration, specify here:<br>*Leave blank if not applicable*<br>*Question relevant when: selected( ${q104_M67_full_list} , '1')* | |
| q204_M67d *(required)* | q204_M67d. In total, how many units are used to treat a patient during all of her care?<br>*Remember to add all units! E.g. 5 days x 200 mg twice per day = 2,000mg*<br>*Question relevant when: selected( ${q104_M67_full_list} , '1')*<br>*Response constrained to: .>0* | |
| q204_M68 | ***q204_M68. Adrenergic - other 2: "[q104_M68_full_list_other]"***<br>*Question relevant when: selected( ${q104_M68_full_list} , '1')* | |
| q204_M68a *(required)* | q204_M68a. What percent of patients require this item?<br>*Question relevant when: selected( ${q104_M68_full_list} , '1')*<br>*Response constrained to: .>0 and .<=100 or .=999* | |
| q204_M68b *(required)* | q204_M68b. How is the item administered, or given, to the patient?<br>*Question relevant when: selected( ${q104_M68_full_list} , '1')* | 1 Oral - solid (e.g. tablet)<br>2 Oral - liquid<br>3 Sublingual<br>4 Intravenous (IV)<br>5 Intramusculuar injection (IM)<br>6 Subcutaneous<br>7 Rectal<br>8 Inhaled (e.g. gas)<br>9 Inserted (e.g. vaginal pessaries)<br>10 Topical - cream<br>11 Topical - drops (e.g. eye drops)<br>12 Other<br>99 Don't know |

| Field | Question | Answer |
|---|---|---|

| Field | Question | Answer | |
|---|---|---|---|
| q204_M68c *(required)* | q204_M68c. What is the item's smallest unit of measurement (mg, mcg, ml, g, etc)?<br>*Question relevant when: selected( ${q104_M68_full_list} , '1')* | microgram | microgram (mcg) |
| | | miligram | miligram (mg) |
| | | miligram_dissolved_in_liquid | miligram (mg) dissolved in liquid (e.g. 2 mg per 3 militers) |
| | | gram | gram (g) |
| | | militer | militer (ml) |
| | | centiliter | centiliter (cl) |
| | | litre | litre (l) |
| | | ml_of _solution | ml of solution (e.g. ml of 10% solution) |
| | | litre_of_solution | litre of solution (e.g. 1 litre of 5% solution) |
| | | cubic_meter | cubic meter (m^3) |
| | | other | other |
| | | dont_know | don't know |
| q204_M68c.1 | q204_M68c.1 If other unit of measurement or route of administration, specify here:<br>*Leave blank if not applicable*<br>*Question relevant when: selected( ${q104_M68_full_list} , '1')* | | |
| q204_M68d *(required)* | q204_M68d. In total, how many units are used to treat a patient during all of her care?<br>*Remember to add all units! E.g. 5 days x 200 mg twice per day = 2,000mg*<br>*Question relevant when: selected( ${q104_M68_full_list} , '1')*<br>*Response constrained to: .>0* | | |
| q204_M69 | ***q204_M69. Adrenergic - other 3: "[q104_M69_full_list_other]"***<br>*Question relevant when: selected( ${q104_M69_full_list} , '1')* | | |
| q204_M69a *(required)* | q204_M69a. What percent of patients require this item?<br>*Question relevant when: selected( ${q104_M69_full_list} , '1')*<br>*Response constrained to: .>0 and .<=100 or .=999* | | |
| q204_M69b *(required)* | q204_M69b. How is the item administered, or given, to the patient?<br>*Question relevant when: selected( ${q104_M69_full_list} , '1')* | 1 | Oral - solid (e.g. tablet) |
| | | 2 | Oral - liquid |
| | | 3 | Sublingual |
| | | 4 | Intravenous (IV) |
| | | 5 | Intramusculuar injection (IM) |
| | | 6 | Subcutaneous |
| | | 7 | Rectal |
| | | 8 | Inhaled (e.g. gas) |
| | | 9 | Inserted (e.g. vaginal pessaries) |
| | | 10 | Topical - cream |
| | | 11 | Topical - drops (e.g. eye drops) |
| | | 12 | Other |
| | | 99 | Don't know |

| Field | Question | Answer |
|---|---|---|
| q204_M69c *(required)* | q204_M69c. What is the item's smallest unit of measurement (mg, mcg, ml, g, etc)?<br>*Question relevant when: selected( ${q104_M69_full_list} , '1')* | microgram — microgram (mcg)<br>miligram — miligram (mg)<br>miligram_dissolved_in_liquid — miligram (mg) dissolved in liquid (e.g. 2 mg per 3 militers)<br>gram — gram (g)<br>militer — militer (ml)<br>centiliter — centiliter (cl)<br>litre — litre (l)<br>ml_of _solution — ml of solution (e.g. ml of 10% solution)<br>litre_of_solution — litre of solution (e.g. 1 litre of 5% solution)<br>cubic_meter — cubic meter (m^3)<br>other — other<br>dont_know — don't know |
| q204_M69c.1 | q204_M69c.1 If other unit of measurement or route of administration, specify here:<br>*Leave blank if not applicable*<br>*Question relevant when: selected( ${q104_M69_full_list} , '1')* | |
| q204_M69d *(required)* | q204_M69d. In total, how many units are used to treat a patient during all of her care?<br>*Remember to add all units! E.g. 5 days x 200 mg twice per day = 2,000mg*<br>*Question relevant when: selected( ${q104_M69_full_list} , '1')*<br>*Response constrained to: .>0* | |
| q204_M69.1 | ***q204_M69.1. Adrenergic - other 4: "[q104_M69.1_full_list_other]"***<br>*Question relevant when: selected( ${q104_M69.1_full_list} , '1')* | |
| q204_M69.1a *(required)* | q204_M69.1a. What percent of patients require this item?<br>*Question relevant when: selected( ${q104_M69.1_full_list} , '1')*<br>*Response constrained to: .>0 and .<=100 or .=999* | |
| q204_M69.1b *(required)* | q204_M69.1b. How is the item administered, or given, to the patient?<br>*Question relevant when: selected( ${q104_M69.1_full_list} , '1')* | 1 Oral - solid (e.g. tablet)<br>2 Oral - liquid<br>3 Sublingual<br>4 Intravenous (IV)<br>5 Intramusculuar injection (IM)<br>6 Subcutaneous<br>7 Rectal<br>8 Inhaled (e.g. gas)<br>9 Inserted (e.g. vaginal pessaries)<br>10 Topical - cream<br>11 Topical - drops (e.g. eye drops)<br>12 Other<br>99 Don't know |

| Field | Question | Answer |
|---|---|---|
| q204_M69.1c *(required)* | q204_M69.1c. What is the item's smallest unit of measurement (mg, mcg, ml, g, etc)?<br>*Question relevant when: selected( ${q104_M69.1_full_list} , '1')* | microgram — microgram (mcg)<br>miligram — miligram (mg)<br>miligram_dissolved_in_liquid — miligram (mg) dissolved in liquid (e.g. 2 mg per 3 militers)<br>gram — gram (g)<br>militer — militer (ml)<br>centiliter — centiliter (cl)<br>litre — litre (l)<br>ml_of _solution — ml of solution (e.g. ml of 10% solution)<br>litre_of_solution — litre of solution (e.g. 1 litre of 5% solution)<br>cubic_meter — cubic meter (m^3)<br>other — other<br>dont_know — don't know |
| q204_M69.1c.1 | q204_M69.1c.1 If other unit of measurement or route of administration, specify here:<br>*Leave blank if not applicable*<br>*Question relevant when: selected( ${q104_M69.1_full_list} , '1')* | |
| q204_M69.1d *(required)* | q204_M69.1d. In total, how many units are used to treat a patient during all of her care?<br>*Remember to add all units! E.g. 5 days x 200 mg twice per day = 2,000mg*<br>*Question relevant when: selected( ${q104_M69.1_full_list} , '1')*<br>*Response constrained to: .>0* | |
| q204_M69.2 | ***q204_M69.2. Adrenergic - other 5: "[q104_M69.2_full_list_other]"***<br>*Question relevant when: selected( ${q104_M69.2_full_list} , '1')* | |
| q204_M69.2a *(required)* | q204_M69.2a. What percent of patients require this item?<br>*Question relevant when: selected( ${q104_M69.2_full_list} , '1')*<br>*Response constrained to: .>0 and .<=100 or .=999* | |
| q204_M69.2b *(required)* | q204_M69.2b. How is the item administered, or given, to the patient?<br>*Question relevant when: selected( ${q104_M69.2_full_list} , '1')* | 1 Oral - solid (e.g. tablet)<br>2 Oral - liquid<br>3 Sublingual<br>4 Intravenous (IV)<br>5 Intramusculuar injection (IM)<br>6 Subcutaneous<br>7 Rectal<br>8 Inhaled (e.g. gas)<br>9 Inserted (e.g. vaginal pessaries)<br>10 Topical - cream<br>11 Topical - drops (e.g. eye drops)<br>12 Other<br>99 Don't know |

| Field | Question | Answer |
|---|---|---|
| q204_M69.2c *(required)* | q204_M69.2c. What is the item's smallest unit of measurement (mg, mcg, ml, g, etc)?<br>*Question relevant when: selected( ${q104_M69.2_full_list} , '1')* | microgram: microgram (mcg)<br>miligram: miligram (mg)<br>miligram_dissolved_in_liquid: miligram (mg) dissolved in liquid (e.g. 2 mg per 3 militers)<br>gram: gram (g)<br>militer: militer (ml)<br>centiliter: centiliter (cl)<br>litre: litre (l)<br>ml_of _solution: ml of solution (e.g. ml of 10% solution)<br>litre_of_solution: litre of solution (e.g. 1 litre of 5% solution)<br>cubic_meter: cubic meter (m^3)<br>other: other<br>dont_know: don't know |
| q204_M69.2c.1 | q204_M69.2c.1 If other unit of measurement or route of administration, specify here:<br>*Leave blank if not applicable*<br>*Question relevant when: selected( ${q104_M69.2_full_list} , '1')* | |
| q204_M69.2d *(required)* | q204_M69.2d. In total, how many units are used to treat a patient during all of her care?<br>*Remember to add all units! E.g. 5 days x 200 mg twice per day = 2,000mg*<br>*Question relevant when: selected( ${q104_M69.2_full_list} , '1')*<br>*Response constrained to: .>0* | |
| <p><strong><em>D. Medications (7)</em></strong></p><br>*Group relevant when: selected( ${section_two_skip_med} , '1')* | | |
| q204_med_notE6 | 204 CONTINUED For each of the following medications and other medical products that are used for incomplete abortion, can you tell me …? | |
| note_204_blood | ***Blood products*** | |
| q204_M70 | ***q204_M70. Unit of packed red blood cells***<br>*Question relevant when: selected( ${q104_M70_full_list} , '1')* | |
| q204_M70a *(required)* | q204_M70a. What percent of patients require this item?<br>*Question relevant when: selected( ${q104_M70_full_list} , '1')*<br>*Response constrained to: .>0 and .<=100 or .=999* | |
| q204_M70b *(required)* | q204_M70b. How is the item administered, or given, to the patient?<br>*Question relevant when: selected( ${q104_M70_full_list} , '1')* | 1 Oral - solid (e.g. tablet)<br>2 Oral - liquid<br>3 Sublingual<br>4 Intravenous (IV)<br>5 Intramusculuar injection (IM)<br>6 Subcutaneous<br>7 Rectal<br>8 Inhaled (e.g. gas)<br>9 Inserted (e.g. vaginal pessaries)<br>10 Topical - cream<br>11 Topical - drops (e.g. eye drops)<br>12 Other |

| Field | Question | Answer |
|---|---|---|
| | | 99 Don't know |
| q204_M70c *(required)* | q204_M70c. What is the item's smallest unit of measurement (mg, mcg, ml, g, etc)? *Question relevant when: selected( ${q104_M70_full_list} , '1')* | microgram — microgram (mcg)<br>miligram — miligram (mg)<br>miligram_dissolved_in_liquid — miligram (mg) dissolved in liquid (e.g. 2 mg per 3 militers)<br>gram — gram (g)<br>militer — militer (ml)<br>centiliter — centiliter (cl)<br>litre — litre (l)<br>ml_of _solution — ml of solution (e.g. ml of 10% solution)<br>litre_of_solution — litre of solution (e.g. 1 litre of 5% solution)<br>cubic_meter — cubic meter (m^3)<br>other — other<br>dont_know — don't know |
| q204_M70c.1 | q204_M70c.1 If other unit of measurement or route of administration, specify here: *Leave blank if not applicable* *Question relevant when: selected( ${q104_M70_full_list} , '1')* | |
| q204_M70d *(required)* | q204_M70d. In total, how many units are used to treat a patient during all of her care? *Remember to add all units! E.g. 5 days x 200 mg twice per day = 2,000mg* *Question relevant when: selected( ${q104_M70_full_list} , '1')* *Response constrained to: .>0* | |
| q204_M71 | ***q204_M71. Unit of platelets*** *Question relevant when: selected( ${q104_M71_full_list} , '1')* | |
| q204_M71a *(required)* | q204_M71a. What percent of patients require this item? *Question relevant when: selected( ${q104_M71_full_list} , '1')* *Response constrained to: .>0 and .<=100 or .=999* | |
| q204_M71b *(required)* | q204_M71b. How is the item administered, or given, to the patient? *Question relevant when: selected( ${q104_M71_full_list} , '1')* | 1 Oral - solid (e.g. tablet)<br>2 Oral - liquid<br>3 Sublingual<br>4 Intravenous (IV)<br>5 Intramusculuar injection (IM)<br>6 Subcutaneous<br>7 Rectal<br>8 Inhaled (e.g. gas)<br>9 Inserted (e.g. vaginal pessaries)<br>10 Topical - cream<br>11 Topical - drops (e.g. eye drops)<br>12 Other<br>99 Don't know |

| Field | Question | Answer |
|---|---|---|

| Field | Question | Answer | | |
|---|---|---|---|---|
| q204_M71c *(required)* | q204_M71c. What is the item's smallest unit of measurement (mg, mcg, ml, g, etc)?<br>*Question relevant when: selected( ${q104_M71_full_list} , '1')* | | microgram | microgram (mcg) |
| | | | miligram | miligram (mg) |
| | | | miligram_dissolved_in_liquid | miligram (mg) dissolved in liquid (e.g. 2 mg per 3 militers) |
| | | | gram | gram (g) |
| | | | militer | militer (ml) |
| | | | centiliter | centiliter (cl) |
| | | | litre | litre (l) |
| | | | ml_of _solution | ml of solution (e.g. ml of 10% solution) |
| | | | litre_of_solution | litre of solution (e.g. 1 litre of 5% solution) |
| | | | cubic_meter | cubic meter (m^3) |
| | | | other | other |
| | | | dont_know | don't know |
| q204_M71c.1 | q204_M71c.1 If other unit of measurement or route of administration, specify here:<br>*Leave blank if not applicable*<br>*Question relevant when: selected( ${q104_M71_full_list} , '1')* | | | |
| q204_M71d *(required)* | q204_M71d. In total, how many units are used to treat a patient during all of her care?<br>*Remember to add all units! E.g. 5 days x 200 mg twice per day = 2,000mg*<br>*Question relevant when: selected( ${q104_M71_full_list} , '1')*<br>*Response constrained to: .>0* | | | |
| q204_M72 | ***q204_M72. Unit of whole blood***<br>*Question relevant when: selected( ${q104_M72_full_list} , '1')* | | | |
| q204_M72a *(required)* | q204_M72a. What percent of patients require this item?<br>*Question relevant when: selected( ${q104_M72_full_list} , '1')*<br>*Response constrained to: .>0 and .<=100 or .=999* | | | |
| q204_M72b *(required)* | q204_M72b. How is the item administered, or given, to the patient?<br>*Question relevant when: selected( ${q104_M72_full_list} , '1')* | | 1 | Oral - solid (e.g. tablet) |
| | | | 2 | Oral - liquid |
| | | | 3 | Sublingual |
| | | | 4 | Intravenous (IV) |
| | | | 5 | Intramusculuar injection (IM) |
| | | | 6 | Subcutaneous |
| | | | 7 | Rectal |
| | | | 8 | Inhaled (e.g. gas) |
| | | | 9 | Inserted (e.g. vaginal pessaries) |
| | | | 10 | Topical - cream |
| | | | 11 | Topical - drops (e.g. eye drops) |
| | | | 12 | Other |
| | | | 99 | Don't know |

| Field | Question | Answer |
|---|---|---|
| q204_M72c *(required)* | q204_M72c. What is the item's smallest unit of measurement (mg, mcg, ml, g, etc)?<br>*Question relevant when: selected( ${q104_M72_full_list} , '1')* | microgram: microgram (mcg)<br>miligram: miligram (mg)<br>miligram_dissolved_in_liquid: miligram (mg) dissolved in liquid (e.g. 2 mg per 3 militers)<br>gram: gram (g)<br>militer: militer (ml)<br>centiliter: centiliter (cl)<br>litre: litre (l)<br>ml_of _solution: ml of solution (e.g. ml of 10% solution)<br>litre_of_solution: litre of solution (e.g. 1 litre of 5% solution)<br>cubic_meter: cubic meter (m^3)<br>other: other<br>dont_know: don't know |
| q204_M72c.1 | q204_M72c.1 If other unit of measurement or route of administration, specify here:<br>*Leave blank if not applicable*<br>*Question relevant when: selected( ${q104_M72_full_list} , '1')* | |
| q204_M72d *(required)* | q204_M72d. In total, how many units are used to treat a patient during all of her care?<br>*Remember to add all units! E.g. 5 days x 200 mg twice per day = 2,000mg*<br>*Question relevant when: selected( ${q104_M72_full_list} , '1')*<br>*Response constrained to: .>0* | |
| q204_M73 | ***q204_M73. Blood product - other 1: "[q104_M73_full_list_other]"***<br>*Question relevant when: selected( ${q104_M73_full_list} , '1')* | |
| q204_M73a *(required)* | q204_M73a. What percent of patients require this item?<br>*Question relevant when: selected( ${q104_M73_full_list} , '1')*<br>*Response constrained to: .>0 and .<=100 or .=999* | |
| q204_M73b *(required)* | q204_M73b. How is the item administered, or given, to the patient?<br>*Question relevant when: selected( ${q104_M73_full_list} , '1')* | 1 Oral - solid (e.g. tablet)<br>2 Oral - liquid<br>3 Sublingual<br>4 Intravenous (IV)<br>5 Intramusculuar injection (IM)<br>6 Subcutaneous<br>7 Rectal<br>8 Inhaled (e.g. gas)<br>9 Inserted (e.g. vaginal pessaries)<br>10 Topical - cream<br>11 Topical - drops (e.g. eye drops)<br>12 Other<br>99 Don't know |

| Field | Question | Answer |
|---|---|---|
| q204_M73c *(required)* | q204_M73c. What is the item's smallest unit of measurement (mg, mcg, ml, g, etc)?<br>*Question relevant when: selected( ${q104_M73_full_list} , '1')* | microgram — microgram (mcg)<br>miligram — miligram (mg)<br>miligram_dissolved_in_liquid — miligram (mg) dissolved in liquid (e.g. 2 mg per 3 militers)<br>gram — gram (g)<br>militer — militer (ml)<br>centiliter — centiliter (cl)<br>litre — litre (l)<br>ml_of _solution — ml of solution (e.g. ml of 10% solution)<br>litre_of_solution — litre of solution (e.g. 1 litre of 5% solution)<br>cubic_meter — cubic meter (m^3)<br>other — other<br>dont_know — don't know |
| q204_M73c.1 | q204_M73c.1 If other unit of measurement or route of administration, specify here:<br>*Leave blank if not applicable*<br>*Question relevant when: selected( ${q104_M73_full_list} , '1')* | |
| q204_M73d *(required)* | q204_M73d. In total, how many units are used to treat a patient during all of her care?<br>*Remember to add all units! E.g. 5 days x 200 mg twice per day = 2,000mg*<br>*Question relevant when: selected( ${q104_M73_full_list} , '1')*<br>*Response constrained to: .>0* | |
| q204_M74 | ***q204_M74. Blood product - other 2: "[q104_M74_full_list_other]"***<br>*Question relevant when: selected( ${q104_M74_full_list} , '1')* | |
| q204_M74a *(required)* | q204_M74a. What percent of patients require this item?<br>*Question relevant when: selected( ${q104_M74_full_list} , '1')*<br>*Response constrained to: .>0 and .<=100 or .=999* | |
| q204_M74b *(required)* | q204_M74b. How is the item administered, or given, to the patient?<br>*Question relevant when: selected( ${q104_M74_full_list} , '1')* | 1 Oral - solid (e.g. tablet)<br>2 Oral - liquid<br>3 Sublingual<br>4 Intravenous (IV)<br>5 Intramusculuar injection (IM)<br>6 Subcutaneous<br>7 Rectal<br>8 Inhaled (e.g. gas)<br>9 Inserted (e.g. vaginal pessaries)<br>10 Topical - cream<br>11 Topical - drops (e.g. eye drops)<br>12 Other<br>99 Don't know |

| Field | Question | Answer |
|---|---|---|
| q204_M74c *(required)* | q204_M74c. What is the item's smallest unit of measurement (mg, mcg, ml, g, etc)?<br>*Question relevant when: selected( ${q104_M74_full_list} , '1')* | microgram: microgram (mcg)<br>miligram: miligram (mg)<br>miligram_dissolved_in_liquid: miligram (mg) dissolved in liquid (e.g. 2 mg per 3 militers)<br>gram: gram (g)<br>militer: militer (ml)<br>centiliter: centiliter (cl)<br>litre: litre (l)<br>ml_of _solution: ml of solution (e.g. ml of 10% solution)<br>litre_of_solution: litre of solution (e.g. 1 litre of 5% solution)<br>cubic_meter: cubic meter (m^3)<br>other: other<br>dont_know: don't know |
| q204_M74c.1 | q204_M74c.1 If other unit of measurement or route of administration, specify here:<br>*Leave blank if not applicable*<br>*Question relevant when: selected( ${q104_M74_full_list} , '1')* | |
| q204_M74d *(required)* | q204_M74d. In total, how many units are used to treat a patient during all of her care?<br>*Remember to add all units! E.g. 5 days x 200 mg twice per day = 2,000mg*<br>*Question relevant when: selected( ${q104_M74_full_list} , '1')*<br>*Response constrained to: .>0* | |
| q204_M75 | ***q204_M75. Blood product - other 3: "[q104_M75_full_list_other]"***<br>*Question relevant when: selected( ${q104_M75_full_list} , '1')* | |
| q204_M75a *(required)* | q204_M75a. What percent of patients require this item?<br>*Question relevant when: selected( ${q104_M75_full_list} , '1')*<br>*Response constrained to: .>0 and .<=100 or .=999* | |
| q204_M75b *(required)* | q204_M75b. How is the item administered, or given, to the patient?<br>*Question relevant when: selected( ${q104_M75_full_list} , '1')* | 1 Oral - solid (e.g. tablet)<br>2 Oral - liquid<br>3 Sublingual<br>4 Intravenous (IV)<br>5 Intramusculuar injection (IM)<br>6 Subcutaneous<br>7 Rectal<br>8 Inhaled (e.g. gas)<br>9 Inserted (e.g. vaginal pessaries)<br>10 Topical - cream<br>11 Topical - drops (e.g. eye drops)<br>12 Other<br>99 Don't know |

| Field | Question | Answer |
|---|---|---|
| q204_M75c *(required)* | q204_M75c. What is the item's smallest unit of measurement (mg, mcg, ml, g, etc)?<br>*Question relevant when: selected( ${q104_M75_full_list} , '1')* | microgram — microgram (mcg)<br>miligram — miligram (mg)<br>miligram_dissolved_in_liquid — miligram (mg) dissolved in liquid (e.g. 2 mg per 3 militers)<br>gram — gram (g)<br>militer — militer (ml)<br>centiliter — centiliter (cl)<br>litre — litre (l)<br>ml_of _solution — ml of solution (e.g. ml of 10% solution)<br>litre_of_solution — litre of solution (e.g. 1 litre of 5% solution)<br>cubic_meter — cubic meter (m^3)<br>other — other<br>dont_know — don't know |
| q204_M75c.1 | q204_M75c.1 If other unit of measurement or route of administration, specify here:<br>*Leave blank if not applicable*<br>*Question relevant when: selected( ${q104_M75_full_list} , '1')* | |
| q204_M75d *(required)* | q204_M75d. In total, how many units are used to treat a patient during all of her care?<br>*Remember to add all units! E.g. 5 days x 200 mg twice per day = 2,000mg*<br>*Question relevant when: selected( ${q104_M75_full_list} , '1')*<br>*Response constrained to: .>0* | |
| q204_M75.1 | ***q204_M75.1. Blood product - other 4: "[q104_M75.1_full_list_other]"***<br>*Question relevant when: selected( ${q104_M75.1_full_list} , '1')* | |
| q204_M75.1a *(required)* | q204_M75.1a. What percent of patients require this item?<br>*Question relevant when: selected( ${q104_M75.1_full_list} , '1')*<br>*Response constrained to: .>0 and .<=100 or .=999* | |
| q204_M75.1b *(required)* | q204_M75.1b. How is the item administered, or given, to the patient?<br>*Question relevant when: selected( ${q104_M75.1_full_list} , '1')* | 1 Oral - solid (e.g. tablet)<br>2 Oral - liquid<br>3 Sublingual<br>4 Intravenous (IV)<br>5 Intramusculuar injection (IM)<br>6 Subcutaneous<br>7 Rectal<br>8 Inhaled (e.g. gas)<br>9 Inserted (e.g. vaginal pessaries)<br>10 Topical - cream<br>11 Topical - drops (e.g. eye drops)<br>12 Other<br>99 Don't know |

| Field | Question | Answer |
|---|---|---|
| q204_M75.1c *(required)* | q204_M75.1c. What is the item's smallest unit of measurement (mg, mcg, ml, g, etc)?<br>*Question relevant when: selected( ${q104_M75.1_full_list} , '1')* | microgram — microgram (mcg)<br>miligram — miligram (mg)<br>miligram_dissolved_in_liquid — miligram (mg) dissolved in liquid (e.g. 2 mg per 3 militers)<br>gram — gram (g)<br>militer — militer (ml)<br>centiliter — centiliter (cl)<br>litre — litre (l)<br>ml_of _solution — ml of solution (e.g. ml of 10% solution)<br>litre_of_solution — litre of solution (e.g. 1 litre of 5% solution)<br>cubic_meter — cubic meter (m^3)<br>other — other<br>dont_know — don't know |
| q204_M75.1c.1 | q204_M75.1c.1 If other unit of measurement or route of administration, specify here:<br>*Leave blank if not applicable*<br>*Question relevant when: selected( ${q104_M75.1_full_list} , '1')* | |
| q204_M75.1d *(required)* | q204_M75.1d. In total, how many units are used to treat a patient during all of her care?<br>*Remember to add all units! E.g. 5 days x 200 mg twice per day = 2,000mg*<br>*Question relevant when: selected( ${q104_M75.1_full_list} , '1')*<br>*Response constrained to: .>0* | |
| q204_M75.2 | ***q204_M75.2. Blood product - other 5: "[q104_M75.2_full_list_other]"***<br>*Question relevant when: selected( ${q104_M75.2_full_list} , '1')* | |
| q204_M75.2a *(required)* | q204_M75.2a. What percent of patients require this item?<br>*Question relevant when: selected( ${q104_M75.2_full_list} , '1')*<br>*Response constrained to: .>0 and .<=100 or .=999* | |
| q204_M75.2b *(required)* | q204_M75.2b. How is the item administered, or given, to the patient?<br>*Question relevant when: selected( ${q104_M75.2_full_list} , '1')* | 1 Oral - solid (e.g. tablet)<br>2 Oral - liquid<br>3 Sublingual<br>4 Intravenous (IV)<br>5 Intramusculuar injection (IM)<br>6 Subcutaneous<br>7 Rectal<br>8 Inhaled (e.g. gas)<br>9 Inserted (e.g. vaginal pessaries)<br>10 Topical - cream<br>11 Topical - drops (e.g. eye drops)<br>12 Other<br>99 Don't know |

| Field | Question | Answer |
|---|---|---|
| q204_M75.2c *(required)* | q204_M75.2c. What is the item's smallest unit of measurement (mg, mcg, ml, g, etc)? *Question relevant when: selected( ${q104_M75.2_full_list} , '1')* | microgram — microgram (mcg)<br>miligram — miligram (mg)<br>miligram_dissolved_in_liquid — miligram (mg) dissolved in liquid (e.g. 2 mg per 3 militers)<br>gram — gram (g)<br>militer — militer (ml)<br>centiliter — centiliter (cl)<br>litre — litre (l)<br>ml_of _solution — ml of solution (e.g. ml of 10% solution)<br>litre_of_solution — litre of solution (e.g. 1 litre of 5% solution)<br>cubic_meter — cubic meter (m^3)<br>other — other<br>dont_know — don't know |
| q204_M75.2c.1 | q204_M75.2c.1 If other unit of measurement or route of administration, specify here: *Leave blank if not applicable* *Question relevant when: selected( ${q104_M75.2_full_list} , '1')* | |
| q204_M75.2d *(required)* | q204_M75.2d. In total, how many units are used to treat a patient during all of her care? *Remember to add all units! E.g. 5 days x 200 mg twice per day = 2,000mg* *Question relevant when: selected( ${q104_M75.2_full_list} , '1')* *Response constrained to: .>0* | |
| <p><strong><em>D. Medications (8)</em></strong></p> *Group relevant when: selected( ${section_two_skip_med} , '1')* | | |
| q204_med_note8 | 204 CONTINUED For each of the following medications and other medical products that are used for incomplete abortion, can you tell me …? | |
| note_204_hemostatic | ***Hemostatics*** | |
| q204_M76 | ***q204_M76. Dicynone / Dicynene (Etamsylate)*** *Question relevant when: selected( ${q104_M76_full_list} , '1')* | |
| q204_M76a *(required)* | q204_M76a. What percent of patients require this item? *Question relevant when: selected( ${q104_M76_full_list} , '1')* *Response constrained to: .>0 and .<=100 or .=999* | |
| q204_M76b *(required)* | q204_M76b. How is the item administered, or given, to the patient? *Question relevant when: selected( ${q104_M76_full_list} , '1')* | 1 Oral - solid (e.g. tablet)<br>2 Oral - liquid<br>3 Sublingual<br>4 Intravenous (IV)<br>5 Intramusculuar injection (IM)<br>6 Subcutaneous<br>7 Rectal<br>8 Inhaled (e.g. gas)<br>9 Inserted (e.g. vaginal pessaries)<br>10 Topical - cream<br>11 Topical - drops (e.g. eye drops)<br>12 Other |

| Field | Question | Answer |
|---|---|---|
| | | 99 Don't know |

| Field | Question | Answer | | |
|---|---|---|---|---|
| q204_M76c *(required)* | q204_M76c. What is the item's smallest unit of measurement (mg, mcg, ml, g, etc)? *Question relevant when: selected( ${q104_M76_full_list} , '1')* | | microgram | microgram (mcg) |
| | | | miligram | miligram (mg) |
| | | | miligram_dissolved_in_liquid | miligram (mg) dissolved in liquid (e.g. 2 mg per 3 militers) |
| | | | gram | gram (g) |
| | | | militer | militer (ml) |
| | | | centiliter | centiliter (cl) |
| | | | litre | litre (l) |
| | | | ml_of _solution | ml of solution (e.g. ml of 10% solution) |
| | | | litre_of_solution | litre of solution (e.g. 1 litre of 5% solution) |
| | | | cubic_meter | cubic meter (m^3) |
| | | | other | other |
| | | | dont_know | don't know |
| q204_M76c.1 | q204_M76c.1 If other unit of measurement or route of administration, specify here: *Leave blank if not applicable* *Question relevant when: selected( ${q104_M76_full_list} , '1')* | | | |
| q204_M76d *(required)* | q204_M76d. In total, how many units are used to treat a patient during all of her care? *Remember to add all units! E.g. 5 days x 200 mg twice per day = 2,000mg* *Question relevant when: selected( ${q104_M76_full_list} , '1')* *Response constrained to: .>0* | | | |
| q204_M77 | ***q204_M77. Tranexamic acid (Kapron, Cyklokapron)*** *Question relevant when: selected( ${q104_M77_full_list} , '1')* | | | |
| q204_M77a *(required)* | q204_M77a. What percent of patients require this item? *Question relevant when: selected( ${q104_M77_full_list} , '1')* *Response constrained to: .>0 and .<=100 or .=999* | | | |
| q204_M77b *(required)* | q204_M77b. How is the item administered, or given, to the patient? *Question relevant when: selected( ${q104_M77_full_list} , '1')* | | 1 | Oral - solid (e.g. tablet) |
| | | | 2 | Oral - liquid |
| | | | 3 | Sublingual |
| | | | 4 | Intravenous (IV) |
| | | | 5 | Intramusculuar injection (IM) |
| | | | 6 | Subcutaneous |
| | | | 7 | Rectal |
| | | | 8 | Inhaled (e.g. gas) |
| | | | 9 | Inserted (e.g. vaginal pessaries) |
| | | | 10 | Topical - cream |
| | | | 11 | Topical - drops (e.g. eye drops) |
| | | | 12 | Other |
| | | | 99 | Don't know |

| Field | Question | Answer |
|---|---|---|
| q204_M77c *(required)* | q204_M77c. What is the item's smallest unit of measurement (mg, mcg, ml, g, etc)?<br>*Question relevant when: selected( ${q104_M77_full_list} , '1')* | microgram — microgram (mcg)<br>miligram — miligram (mg)<br>miligram_dissolved_in_liquid — miligram (mg) dissolved in liquid (e.g. 2 mg per 3 militers)<br>gram — gram (g)<br>militer — militer (ml)<br>centiliter — centiliter (cl)<br>litre — litre (l)<br>ml_of _solution — ml of solution (e.g. ml of 10% solution)<br>litre_of_solution — litre of solution (e.g. 1 litre of 5% solution)<br>cubic_meter — cubic meter (m^3)<br>other — other<br>dont_know — don't know |
| q204_M77c.1 | q204_M77c.1 If other unit of measurement or route of administration, specify here:<br>*Leave blank if not applicable*<br>*Question relevant when: selected( ${q104_M77_full_list} , '1')* | |
| q204_M77d *(required)* | q204_M77d. In total, how many units are used to treat a patient during all of her care?<br>*Remember to add all units! E.g. 5 days x 200 mg twice per day = 2,000mg*<br>*Question relevant when: selected( ${q104_M77_full_list} , '1')*<br>*Response constrained to: .>0* | |
| q204_M78 | ***q204_M78. Polygeline 4%***<br>*Question relevant when: selected( ${q104_M78_full_list} , '1')* | |
| q204_M78a *(required)* | q204_M78a. What percent of patients require this item?<br>*Question relevant when: selected( ${q104_M78_full_list} , '1')*<br>*Response constrained to: .>0 and .<=100 or .=999* | |
| q204_M78b *(required)* | q204_M78b. How is the item administered, or given, to the patient?<br>*Question relevant when: selected( ${q104_M78_full_list} , '1')* | 1 Oral - solid (e.g. tablet)<br>2 Oral - liquid<br>3 Sublingual<br>4 Intravenous (IV)<br>5 Intramusculuar injection (IM)<br>6 Subcutaneous<br>7 Rectal<br>8 Inhaled (e.g. gas)<br>9 Inserted (e.g. vaginal pessaries)<br>10 Topical - cream<br>11 Topical - drops (e.g. eye drops)<br>12 Other<br>99 Don't know |

| Field | Question | Answer |
|---|---|---|
| q204_M78c *(required)* | q204_M78c. What is the item's smallest unit of measurement (mg, mcg, ml, g, etc)?<br>*Question relevant when: selected( ${q104_M78_full_list} , '1')* | microgram — microgram (mcg)<br>miligram — miligram (mg)<br>miligram_dissolved_in_liquid — miligram (mg) dissolved in liquid (e.g. 2 mg per 3 militers)<br>gram — gram (g)<br>militer — militer (ml)<br>centiliter — centiliter (cl)<br>litre — litre (l)<br>ml_of _solution — ml of solution (e.g. ml of 10% solution)<br>litre_of_solution — litre of solution (e.g. 1 litre of 5% solution)<br>cubic_meter — cubic meter (m^3)<br>other — other<br>dont_know — don't know |
| q204_M78c.1 | q204_M78c.1 If other unit of measurement or route of administration, specify here:<br>*Leave blank if not applicable*<br>*Question relevant when: selected( ${q104_M78_full_list} , '1')* | |
| q204_M78d *(required)* | q204_M78d. In total, how many units are used to treat a patient during all of her care?<br>*Remember to add all units! E.g. 5 days x 200 mg twice per day = 2,000mg*<br>*Question relevant when: selected( ${q104_M78_full_list} , '1')*<br>*Response constrained to: .>0* | |
| q204_M79 | ***q204_M79. Hemostatic - other 1: "[q104_M79_full_list_other]"***<br>*Question relevant when: selected( ${q104_M79_full_list} , '1')* | |
| q204_M79a *(required)* | q204_M79a. What percent of patients require this item?<br>*Question relevant when: selected( ${q104_M79_full_list} , '1')*<br>*Response constrained to: .>0 and .<=100 or .=999* | |
| q204_M79b *(required)* | q204_M79b. How is the item administered, or given, to the patient?<br>*Question relevant when: selected( ${q104_M79_full_list} , '1')* | 1 Oral - solid (e.g. tablet)<br>2 Oral - liquid<br>3 Sublingual<br>4 Intravenous (IV)<br>5 Intramusculuar injection (IM)<br>6 Subcutaneous<br>7 Rectal<br>8 Inhaled (e.g. gas)<br>9 Inserted (e.g. vaginal pessaries)<br>10 Topical - cream<br>11 Topical - drops (e.g. eye drops)<br>12 Other<br>99 Don't know |

| Field | Question | Answer |
|---|---|---|
| q204_M79c *(required)* | q204_M79c. What is the item's smallest unit of measurement (mg, mcg, ml, g, etc)?<br>*Question relevant when: selected( ${q104_M79_full_list} , '1')* | microgram — microgram (mcg)<br>miligram — miligram (mg)<br>miligram_dissolved_in_liquid — miligram (mg) dissolved in liquid (e.g. 2 mg per 3 militers)<br>gram — gram (g)<br>militer — militer (ml)<br>centiliter — centiliter (cl)<br>litre — litre (l)<br>ml_of _solution — ml of solution (e.g. ml of 10% solution)<br>litre_of_solution — litre of solution (e.g. 1 litre of 5% solution)<br>cubic_meter — cubic meter (m^3)<br>other — other<br>dont_know — don't know |
| q204_M79c.1 | q204_M79c.1 If other unit of measurement or route of administration, specify here:<br>*Leave blank if not applicable*<br>*Question relevant when: selected( ${q104_M79_full_list} , '1')* | |
| q204_M79d *(required)* | q204_M79d. In total, how many units are used to treat a patient during all of her care?<br>*Remember to add all units! E.g. 5 days x 200 mg twice per day = 2,000mg*<br>*Question relevant when: selected( ${q104_M79_full_list} , '1')*<br>*Response constrained to: .>0* | |
| q204_M80 | ***q204_M80. Hemostatic - other 2: "[q104_M80_full_list_other]"***<br>*Question relevant when: selected( ${q104_M80_full_list} , '1')* | |
| q204_M80a *(required)* | q204_M80a. What percent of patients require this item?<br>*Question relevant when: selected( ${q104_M80_full_list} , '1')*<br>*Response constrained to: .>0 and .<=100 or .=999* | |
| q204_M80b *(required)* | q204_M80b. How is the item administered, or given, to the patient?<br>*Question relevant when: selected( ${q104_M80_full_list} , '1')* | 1 Oral - solid (e.g. tablet)<br>2 Oral - liquid<br>3 Sublingual<br>4 Intravenous (IV)<br>5 Intramusculuar injection (IM)<br>6 Subcutaneous<br>7 Rectal<br>8 Inhaled (e.g. gas)<br>9 Inserted (e.g. vaginal pessaries)<br>10 Topical - cream<br>11 Topical - drops (e.g. eye drops)<br>12 Other<br>99 Don't know |

| Field | Question | Answer |
|---|---|---|
| q204_M80c *(required)* | q204_M80c. What is the item's smallest unit of measurement (mg, mcg, ml, g, etc)?<br>*Question relevant when: selected( ${q104_M80_full_list} , '1')* | microgram — microgram (mcg)<br>miligram — miligram (mg)<br>miligram_dissolved_in_liquid — miligram (mg) dissolved in liquid (e.g. 2 mg per 3 militers)<br>gram — gram (g)<br>militer — militer (ml)<br>centiliter — centiliter (cl)<br>litre — litre (l)<br>ml_of _solution — ml of solution (e.g. ml of 10% solution)<br>litre_of_solution — litre of solution (e.g. 1 litre of 5% solution)<br>cubic_meter — cubic meter (m^3)<br>other — other<br>dont_know — don't know |
| q204_M80c.1 | q204_M80c.1 If other unit of measurement or route of administration, specify here:<br>*Leave blank if not applicable*<br>*Question relevant when: selected( ${q104_M80_full_list} , '1')* | |
| q204_M80d *(required)* | q204_M80d. In total, how many units are used to treat a patient during all of her care?<br>*Remember to add all units! E.g. 5 days x 200 mg twice per day = 2,000mg*<br>*Question relevant when: selected( ${q104_M80_full_list} , '1')*<br>*Response constrained to: .>0* | |
| q204_M81 | ***q204_M81. Hemostatic - other 3: "[q104_M81_full_list_other]"***<br>*Question relevant when: selected( ${q104_M81_full_list} , '1')* | |
| q204_M81a *(required)* | q204_M81a. What percent of patients require this item?<br>*Question relevant when: selected( ${q104_M81_full_list} , '1')*<br>*Response constrained to: .>0 and .<=100 or .=999* | |
| q204_M81b *(required)* | q204_M81b. How is the item administered, or given, to the patient?<br>*Question relevant when: selected( ${q104_M81_full_list} , '1')* | 1 Oral - solid (e.g. tablet)<br>2 Oral - liquid<br>3 Sublingual<br>4 Intravenous (IV)<br>5 Intramusculuar injection (IM)<br>6 Subcutaneous<br>7 Rectal<br>8 Inhaled (e.g. gas)<br>9 Inserted (e.g. vaginal pessaries)<br>10 Topical - cream<br>11 Topical - drops (e.g. eye drops)<br>12 Other<br>99 Don't know |

| Field | Question | Answer |
|---|---|---|
| q204_M81c *(required)* | q204_M81c. What is the item's smallest unit of measurement (mg, mcg, ml, g, etc)?<br>*Question relevant when: selected( ${q104_M81_full_list} , '1')* | microgram — microgram (mcg)<br>miligram — miligram (mg)<br>miligram_dissolved_in_liquid — miligram (mg) dissolved in liquid (e.g. 2 mg per 3 militers)<br>gram — gram (g)<br>militer — militer (ml)<br>centiliter — centiliter (cl)<br>litre — litre (l)<br>ml_of _solution — ml of solution (e.g. ml of 10% solution)<br>litre_of_solution — litre of solution (e.g. 1 litre of 5% solution)<br>cubic_meter — cubic meter (m^3)<br>other — other<br>dont_know — don't know |
| q204_M81c.1 | q204_M81c.1 If other unit of measurement or route of administration, specify here:<br>*Leave blank if not applicable*<br>*Question relevant when: selected( ${q104_M81_full_list} , '1')* | |
| q204_M81d *(required)* | q204_M81d. In total, how many units are used to treat a patient during all of her care?<br>*Remember to add all units! E.g. 5 days x 200 mg twice per day = 2,000mg*<br>*Question relevant when: selected( ${q104_M81_full_list} , '1')*<br>*Response constrained to: .>0* | |
| q204_M81.1 | ***q204_M81.1. Hemostatic - other 4: "[q104_M81.1_full_list_other]"***<br>*Question relevant when: selected( ${q104_M81.1_full_list} , '1')* | |
| q204_M81.1a *(required)* | q204_M81.1a. What percent of patients require this item?<br>*Question relevant when: selected( ${q104_M81.1_full_list} , '1')*<br>*Response constrained to: .>0 and .<=100 or .=999* | |
| q204_M81.1b *(required)* | q204_M81.1b. How is the item administered, or given, to the patient?<br>*Question relevant when: selected( ${q104_M81.1_full_list} , '1')* | 1 Oral - solid (e.g. tablet)<br>2 Oral - liquid<br>3 Sublingual<br>4 Intravenous (IV)<br>5 Intramusculuar injection (IM)<br>6 Subcutaneous<br>7 Rectal<br>8 Inhaled (e.g. gas)<br>9 Inserted (e.g. vaginal pessaries)<br>10 Topical - cream<br>11 Topical - drops (e.g. eye drops)<br>12 Other<br>99 Don't know |

| Field | Question | Answer |
|---|---|---|
| q204_M81.1c *(required)* | q204_M81.1c. What is the item's smallest unit of measurement (mg, mcg, ml, g, etc)?<br>*Question relevant when: selected( ${q104_M81.1_full_list} , '1')* | microgram: microgram (mcg)<br>miligram: miligram (mg)<br>miligram_dissolved_in_liquid: miligram (mg) dissolved in liquid (e.g. 2 mg per 3 militers)<br>gram: gram (g)<br>militer: militer (ml)<br>centiliter: centiliter (cl)<br>litre: litre (l)<br>ml_of _solution: ml of solution (e.g. ml of 10% solution)<br>litre_of_solution: litre of solution (e.g. 1 litre of 5% solution)<br>cubic_meter: cubic meter (m^3)<br>other: other<br>dont_know: don't know |
| q204_M81.1c.1 | q204_M81.1c.1 If other unit of measurement or route of administration, specify here:<br>*Leave blank if not applicable*<br>*Question relevant when: selected( ${q104_M81.1_full_list} , '1')* | |
| q204_M81.1d *(required)* | q204_M81.1d. In total, how many units are used to treat a patient during all of her care?<br>*Remember to add all units! E.g. 5 days x 200 mg twice per day = 2,000mg*<br>*Question relevant when: selected( ${q104_M81.1_full_list} , '1')*<br>*Response constrained to: .>0* | |
| q204_M81.2 | ***q204_M81.2. Hemostatic - other 5: "[q104_M81.2_full_list_other]"***<br>*Question relevant when: selected( ${q104_M81.2_full_list} , '1')* | |
| q204_M81.2a *(required)* | q204_M81.2a. What percent of patients require this item?<br>*Question relevant when: selected( ${q104_M81.2_full_list} , '1')*<br>*Response constrained to: .>0 and .<=100 or .=999* | |
| q204_M81.2b *(required)* | q204_M81.2b. How is the item administered, or given, to the patient?<br>*Question relevant when: selected( ${q104_M81.2_full_list} , '1')* | 1 Oral - solid (e.g. tablet)<br>2 Oral - liquid<br>3 Sublingual<br>4 Intravenous (IV)<br>5 Intramusculuar injection (IM)<br>6 Subcutaneous<br>7 Rectal<br>8 Inhaled (e.g. gas)<br>9 Inserted (e.g. vaginal pessaries)<br>10 Topical - cream<br>11 Topical - drops (e.g. eye drops)<br>12 Other<br>99 Don't know |

| Field | Question | Answer |
|---|---|---|
| q204_M81.2c *(required)* | q204_M81.2c. What is the item's smallest unit of measurement (mg, mcg, ml, g, etc)?<br>*Question relevant when: selected( ${q104_M81.2_full_list} , '1')* | microgram — microgram (mcg)<br>miligram — miligram (mg)<br>miligram_dissolved_in_liquid — miligram (mg) dissolved in liquid (e.g. 2 mg per 3 militers)<br>gram — gram (g)<br>militer — militer (ml)<br>centiliter — centiliter (cl)<br>litre — litre (l)<br>ml_of _solution — ml of solution (e.g. ml of 10% solution)<br>litre_of_solution — litre of solution (e.g. 1 litre of 5% solution)<br>cubic_meter — cubic meter (m^3)<br>other — other<br>dont_know — don't know |
| q204_M81.2c.1 | q204_M81.2c.1 If other unit of measurement or route of administration, specify here:<br>*Leave blank if not applicable*<br>*Question relevant when: selected( ${q104_M81.2_full_list} , '1')* | |
| q204_M81.2d *(required)* | q204_M81.2d. In total, how many units are used to treat a patient during all of her care?<br>*Remember to add all units! E.g. 5 days x 200 mg twice per day = 2,000mg*<br>*Question relevant when: selected( ${q104_M81.2_full_list} , '1')*<br>*Response constrained to: .>0* | |
| <p><strong><em>D. Medications (9)</em></strong></p><br>*Group relevant when: selected( ${section_two_skip_med} , '1')* | | |
| q204_med_notE8 | 204 CONTINUED For each of the following medications and other medical products that are used for incomplete abortion, can you tell me …? | |
| note_204_saline | ***Saline, glucose, water, etc*** | |
| q204_M82 | ***q204_M82. IV solution, dextrose 2.5% in NaCl 0.45%***<br>*Question relevant when: selected( ${q104_M82_full_list} , '1')* | |
| q204_M82a *(required)* | q204_M82a. What percent of patients require this item?<br>*Question relevant when: selected( ${q104_M82_full_list} , '1')*<br>*Response constrained to: .>0 and .<=100 or .=999* | |
| q204_M82b *(required)* | q204_M82b. How is the item administered, or given, to the patient?<br>*Question relevant when: selected( ${q104_M82_full_list} , '1')* | 1 Oral - solid (e.g. tablet)<br>2 Oral - liquid<br>3 Sublingual<br>4 Intravenous (IV)<br>5 Intramusculuar injection (IM)<br>6 Subcutaneous<br>7 Rectal<br>8 Inhaled (e.g. gas)<br>9 Inserted (e.g. vaginal pessaries)<br>10 Topical - cream<br>11 Topical - drops (e.g. eye drops)<br>12 Other |

| Field | Question | Answer |
|---|---|---|
| | | 99 Don't know |

| Field | Question | Answer |
|---|---|---|
| q204_M82c *(required)* | q204_M82c. What is the item's smallest unit of measurement (mg, mcg, ml, g, etc)?<br>*Question relevant when: selected( ${q104_M82_full_list} , '1')* | microgram — microgram (mcg)<br>miligram — miligram (mg)<br>miligram_dissolved_in_liquid — miligram (mg) dissolved in liquid (e.g. 2 mg per 3 militers)<br>gram — gram (g)<br>militer — militer (ml)<br>centiliter — centiliter (cl)<br>litre — litre (l)<br>ml_of _solution — ml of solution (e.g. ml of 10% solution)<br>litre_of_solution — litre of solution (e.g. 1 litre of 5% solution)<br>cubic_meter — cubic meter (m^3)<br>other — other<br>dont_know — don't know |
| q204_M82c.1 | q204_M82c.1 If other unit of measurement or route of administration, specify here:<br>*Leave blank if not applicable*<br>*Question relevant when: selected( ${q104_M82_full_list} , '1')* | |
| q204_M82d *(required)* | q204_M82d. In total, how many units are used to treat a patient during all of her care?<br>*Remember to add all units! E.g. 5 days x 200 mg twice per day = 2,000mg*<br>*Question relevant when: selected( ${q104_M82_full_list} , '1')*<br>*Response constrained to: .>0* | |
| q204_M83 | ***q204_M83. IV solution, glucose (5%), isotonic***<br>*Question relevant when: selected( ${q104_M83_full_list} , '1')* | |
| q204_M83a *(required)* | q204_M83a. What percent of patients require this item?<br>*Question relevant when: selected( ${q104_M83_full_list} , '1')*<br>*Response constrained to: .>0 and .<=100 or .=999* | |
| q204_M83b *(required)* | q204_M83b. How is the item administered, or given, to the patient?<br>*Question relevant when: selected( ${q104_M83_full_list} , '1')* | 1 Oral - solid (e.g. tablet)<br>2 Oral - liquid<br>3 Sublingual<br>4 Intravenous (IV)<br>5 Intramusculuar injection (IM)<br>6 Subcutaneous<br>7 Rectal<br>8 Inhaled (e.g. gas)<br>9 Inserted (e.g. vaginal pessaries)<br>10 Topical - cream<br>11 Topical - drops (e.g. eye drops)<br>12 Other<br>99 Don't know |

| Field | Question | Answer |
|---|---|---|
| q204_M83c *(required)* | q204_M83c. What is the item's smallest unit of measurement (mg, mcg, ml, g, etc)?<br>*Question relevant when: selected( ${q104_M83_full_list} , '1')* | microgram: microgram (mcg)<br>miligram: miligram (mg)<br>miligram_dissolved_in_liquid: miligram (mg) dissolved in liquid (e.g. 2 mg per 3 militers)<br>gram: gram (g)<br>militer: militer (ml)<br>centiliter: centiliter (cl)<br>litre: litre (l)<br>ml_of _solution: ml of solution (e.g. ml of 10% solution)<br>litre_of_solution: litre of solution (e.g. 1 litre of 5% solution)<br>cubic_meter: cubic meter (m^3)<br>other: other<br>dont_know: don't know |
| q204_M83c.1 | q204_M83c.1 If other unit of measurement or route of administration, specify here:<br>*Leave blank if not applicable*<br>*Question relevant when: selected( ${q104_M83_full_list} , '1')* | |
| q204_M83d *(required)* | q204_M83d. In total, how many units are used to treat a patient during all of her care?<br>*Remember to add all units! E.g. 5 days x 200 mg twice per day = 2,000mg*<br>*Question relevant when: selected( ${q104_M83_full_list} , '1')*<br>*Response constrained to: .>0* | |
| q204_M84 | ***q204_M84. Ringers lactate (prepared onsite)***<br>*Question relevant when: selected( ${q104_M84_full_list} , '1')* | |
| q204_M84a *(required)* | q204_M84a. What percent of patients require this item?<br>*Question relevant when: selected( ${q104_M84_full_list} , '1')*<br>*Response constrained to: .>0 and .<=100 or .=999* | |
| q204_M84b *(required)* | q204_M84b. How is the item administered, or given, to the patient?<br>*Question relevant when: selected( ${q104_M84_full_list} , '1')* | 1 Oral - solid (e.g. tablet)<br>2 Oral - liquid<br>3 Sublingual<br>4 Intravenous (IV)<br>5 Intramusculuar injection (IM)<br>6 Subcutaneous<br>7 Rectal<br>8 Inhaled (e.g. gas)<br>9 Inserted (e.g. vaginal pessaries)<br>10 Topical - cream<br>11 Topical - drops (e.g. eye drops)<br>12 Other<br>99 Don't know |

| Field | Question | Answer |
|---|---|---|
| q204_M84c *(required)* | q204_M84c. What is the item's smallest unit of measurement (mg, mcg, ml, g, etc)? <br> *Question relevant when: selected( ${q104_M84_full_list} , '1')* | microgram — microgram (mcg)<br>miligram — miligram (mg)<br>miligram_dissolved_in_liquid — miligram (mg) dissolved in liquid (e.g. 2 mg per 3 militers)<br>gram — gram (g)<br>militer — militer (ml)<br>centiliter — centiliter (cl)<br>litre — litre (l)<br>ml_of _solution — ml of solution (e.g. ml of 10% solution)<br>litre_of_solution — litre of solution (e.g. 1 litre of 5% solution)<br>cubic_meter — cubic meter (m^3)<br>other — other<br>dont_know — don't know |
| q204_M84c.1 | q204_M84c.1 If other unit of measurement or route of administration, specify here: <br> *Leave blank if not applicable* <br> *Question relevant when: selected( ${q104_M84_full_list} , '1')* | |
| q204_M84d *(required)* | q204_M84d. In total, how many units are used to treat a patient during all of her care? <br> *Remember to add all units! E.g. 5 days x 200 mg twice per day = 2,000mg* <br> *Question relevant when: selected( ${q104_M84_full_list} , '1')* <br> *Response constrained to: .>0* | |
| q204_M85 | ***q204_M85. Ringers lactate (bought from pharmacy/MSD)*** <br> *Question relevant when: selected( ${q104_M85_full_list} , '1')* | |
| q204_M85a *(required)* | q204_M85a. What percent of patients require this item? <br> *Question relevant when: selected( ${q104_M85_full_list} , '1')* <br> *Response constrained to: .>0 and .<=100 or .=999* | |
| q204_M85b *(required)* | q204_M85b. How is the item administered, or given, to the patient? <br> *Question relevant when: selected( ${q104_M85_full_list} , '1')* | 1 Oral - solid (e.g. tablet)<br>2 Oral - liquid<br>3 Sublingual<br>4 Intravenous (IV)<br>5 Intramusculuar injection (IM)<br>6 Subcutaneous<br>7 Rectal<br>8 Inhaled (e.g. gas)<br>9 Inserted (e.g. vaginal pessaries)<br>10 Topical - cream<br>11 Topical - drops (e.g. eye drops)<br>12 Other<br>99 Don't know |

| Field | Question | Answer |
|---|---|---|
| q204_M85c *(required)* | q204_M85c. What is the item's smallest unit of measurement (mg, mcg, ml, g, etc)?<br>*Question relevant when: selected( ${q104_M85_full_list} , '1')* | microgram: microgram (mcg)<br>miligram: miligram (mg)<br>miligram_dissolved_in_liquid: miligram (mg) dissolved in liquid (e.g. 2 mg per 3 militers)<br>gram: gram (g)<br>militer: militer (ml)<br>centiliter: centiliter (cl)<br>litre: litre (l)<br>ml_of _solution: ml of solution (e.g. ml of 10% solution)<br>litre_of_solution: litre of solution (e.g. 1 litre of 5% solution)<br>cubic_meter: cubic meter (m^3)<br>other: other<br>dont_know: don't know |
| q204_M85c.1 | q204_M85c.1 If other unit of measurement or route of administration, specify here:<br>*Leave blank if not applicable*<br>*Question relevant when: selected( ${q104_M85_full_list} , '1')* | |
| q204_M85d *(required)* | q204_M85d. In total, how many units are used to treat a patient during all of her care?<br>*Remember to add all units! E.g. 5 days x 200 mg twice per day = 2,000mg*<br>*Question relevant when: selected( ${q104_M85_full_list} , '1')*<br>*Response constrained to: .>0* | |
| q204_M86 | ***q204_M86. Saline, 0.9% (Normal Saline) (prepared onsite)***<br>*Question relevant when: selected( ${q104_M86_full_list} , '1')* | |
| q204_M86a *(required)* | q204_M86a. What percent of patients require this item?<br>*Question relevant when: selected( ${q104_M86_full_list} , '1')*<br>*Response constrained to: .>0 and .<=100 or .=999* | |
| q204_M86b *(required)* | q204_M86b. How is the item administered, or given, to the patient?<br>*Question relevant when: selected( ${q104_M86_full_list} , '1')* | 1 Oral - solid (e.g. tablet)<br>2 Oral - liquid<br>3 Sublingual<br>4 Intravenous (IV)<br>5 Intramusculuar injection (IM)<br>6 Subcutaneous<br>7 Rectal<br>8 Inhaled (e.g. gas)<br>9 Inserted (e.g. vaginal pessaries)<br>10 Topical - cream<br>11 Topical - drops (e.g. eye drops)<br>12 Other<br>99 Don't know |

| Field | Question | Answer |
|---|---|---|
| q204_M86c *(required)* | q204_M86c. What is the item's smallest unit of measurement (mg, mcg, ml, g, etc)?<br>*Question relevant when: selected( ${q104_M86_full_list} , '1')* | microgram — microgram (mcg)<br>miligram — miligram (mg)<br>miligram_dissolved_in_liquid — miligram (mg) dissolved in liquid (e.g. 2 mg per 3 militers)<br>gram — gram (g)<br>militer — militer (ml)<br>centiliter — centiliter (cl)<br>litre — litre (l)<br>ml_of _solution — ml of solution (e.g. ml of 10% solution)<br>litre_of_solution — litre of solution (e.g. 1 litre of 5% solution)<br>cubic_meter — cubic meter (m^3)<br>other — other<br>dont_know — don't know |
| q204_M86c.1 | q204_M86c.1 If other unit of measurement or route of administration, specify here:<br>*Leave blank if not applicable*<br>*Question relevant when: selected( ${q104_M86_full_list} , '1')* | |
| q204_M86d *(required)* | q204_M86d. In total, how many units are used to treat a patient during all of her care?<br>*Remember to add all units! E.g. 5 days x 200 mg twice per day = 2,000mg*<br>*Question relevant when: selected( ${q104_M86_full_list} , '1')*<br>*Response constrained to: .>0* | |
| q204_M87 | ***q204_M87. Saline, 0.9% (Normal Saline) (bought from pharmacy/MSD)***<br>*Question relevant when: selected( ${q104_M87_full_list} , '1')* | |
| q204_M87a *(required)* | q204_M87a. What percent of patients require this item?<br>*Question relevant when: selected( ${q104_M87_full_list} , '1')*<br>*Response constrained to: .>0 and .<=100 or .=999* | |
| q204_M87b *(required)* | q204_M87b. How is the item administered, or given, to the patient?<br>*Question relevant when: selected( ${q104_M87_full_list} , '1')* | 1 Oral - solid (e.g. tablet)<br>2 Oral - liquid<br>3 Sublingual<br>4 Intravenous (IV)<br>5 Intramusculuar injection (IM)<br>6 Subcutaneous<br>7 Rectal<br>8 Inhaled (e.g. gas)<br>9 Inserted (e.g. vaginal pessaries)<br>10 Topical - cream<br>11 Topical - drops (e.g. eye drops)<br>12 Other<br>99 Don't know |

| Field | Question | Answer |
|---|---|---|
| q204_M87c *(required)* | q204_M87c. What is the item's smallest unit of measurement (mg, mcg, ml, g, etc)?<br>*Question relevant when: selected( ${q104_M87_full_list} , '1')* | microgram — microgram (mcg)<br>miligram — miligram (mg)<br>miligram_dissolved_in_liquid — miligram (mg) dissolved in liquid (e.g. 2 mg per 3 militers)<br>gram — gram (g)<br>militer — militer (ml)<br>centiliter — centiliter (cl)<br>litre — litre (l)<br>ml_of _solution — ml of solution (e.g. ml of 10% solution)<br>litre_of_solution — litre of solution (e.g. 1 litre of 5% solution)<br>cubic_meter — cubic meter (m^3)<br>other — other<br>dont_know — don't know |
| q204_M87c.1 | q204_M87c.1 If other unit of measurement or route of administration, specify here:<br>*Leave blank if not applicable*<br>*Question relevant when: selected( ${q104_M87_full_list} , '1')* | |
| q204_M87d *(required)* | q204_M87d. In total, how many units are used to treat a patient during all of her care?<br>*Remember to add all units! E.g. 5 days x 200 mg twice per day = 2,000mg*<br>*Question relevant when: selected( ${q104_M87_full_list} , '1')*<br>*Response constrained to: .>0* | |
| q204_M88 | ***q204_M88. Solution for Infusion, glucose 5% + NaCl 0.9% (prepared onsite)***<br>*Question relevant when: selected( ${q104_M88_full_list} , '1')* | |
| q204_M88a *(required)* | q204_M88a. What percent of patients require this item?<br>*Question relevant when: selected( ${q104_M88_full_list} , '1')*<br>*Response constrained to: .>0 and .<=100 or .=999* | |
| q204_M88b *(required)* | q204_M88b. How is the item administered, or given, to the patient?<br>*Question relevant when: selected( ${q104_M88_full_list} , '1')* | 1 Oral - solid (e.g. tablet)<br>2 Oral - liquid<br>3 Sublingual<br>4 Intravenous (IV)<br>5 Intramusculuar injection (IM)<br>6 Subcutaneous<br>7 Rectal<br>8 Inhaled (e.g. gas)<br>9 Inserted (e.g. vaginal pessaries)<br>10 Topical - cream<br>11 Topical - drops (e.g. eye drops)<br>12 Other<br>99 Don't know |

| Field | Question | Answer |
|---|---|---|
| q204_M88c *(required)* | q204_M88c. What is the item's smallest unit of measurement (mg, mcg, ml, g, etc)?<br>*Question relevant when: selected( ${q104_M88_full_list} , '1')* | microgram — microgram (mcg)<br>miligram — miligram (mg)<br>miligram_dissolved_in_liquid — miligram (mg) dissolved in liquid (e.g. 2 mg per 3 militers)<br>gram — gram (g)<br>militer — militer (ml)<br>centiliter — centiliter (cl)<br>litre — litre (l)<br>ml_of _solution — ml of solution (e.g. ml of 10% solution)<br>litre_of_solution — litre of solution (e.g. 1 litre of 5% solution)<br>cubic_meter — cubic meter (m^3)<br>other — other<br>dont_know — don't know |
| q204_M88c.1 | q204_M88c.1 If other unit of measurement or route of administration, specify here:<br>*Leave blank if not applicable*<br>*Question relevant when: selected( ${q104_M88_full_list} , '1')* | |
| q204_M88d *(required)* | q204_M88d. In total, how many units are used to treat a patient during all of her care?<br>*Remember to add all units! E.g. 5 days x 200 mg twice per day = 2,000mg*<br>*Question relevant when: selected( ${q104_M88_full_list} , '1')*<br>*Response constrained to: .>0* | |
| q204_M89 | ***q204_M89. Solution for Infusion, glucose 5% + NaCl 0.9% (bought from pharmacy or MSD)***<br>*Question relevant when: selected( ${q104_M89_full_list} , '1')* | |
| q204_M89a *(required)* | q204_M89a. What percent of patients require this item?<br>*Question relevant when: selected( ${q104_M89_full_list} , '1')*<br>*Response constrained to: .>0 and .<=100 or .=999* | |
| q204_M89b *(required)* | q204_M89b. How is the item administered, or given, to the patient?<br>*Question relevant when: selected( ${q104_M89_full_list} , '1')* | 1 Oral - solid (e.g. tablet)<br>2 Oral - liquid<br>3 Sublingual<br>4 Intravenous (IV)<br>5 Intramusculuar injection (IM)<br>6 Subcutaneous<br>7 Rectal<br>8 Inhaled (e.g. gas)<br>9 Inserted (e.g. vaginal pessaries)<br>10 Topical - cream<br>11 Topical - drops (e.g. eye drops)<br>12 Other<br>99 Don't know |

| Field | Question | Answer |
|---|---|---|
| q204_M89c *(required)* | q204_M89c. What is the item's smallest unit of measurement (mg, mcg, ml, g, etc)?<br>*Question relevant when: selected( ${q104_M89_full_list} , '1')* | microgram — microgram (mcg)<br>miligram — miligram (mg)<br>miligram_dissolved_in_liquid — miligram (mg) dissolved in liquid (e.g. 2 mg per 3 militers)<br>gram — gram (g)<br>militer — militer (ml)<br>centiliter — centiliter (cl)<br>litre — litre (l)<br>ml_of _solution — ml of solution (e.g. ml of 10% solution)<br>litre_of_solution — litre of solution (e.g. 1 litre of 5% solution)<br>cubic_meter — cubic meter (m^3)<br>other — other<br>dont_know — don't know |
| q204_M89c.1 | q204_M89c.1 If other unit of measurement or route of administration, specify here:<br>*Leave blank if not applicable*<br>*Question relevant when: selected( ${q104_M89_full_list} , '1')* | |
| q204_M89d *(required)* | q204_M89d. In total, how many units are used to treat a patient during all of her care?<br>*Remember to add all units! E.g. 5 days x 200 mg twice per day = 2,000mg*<br>*Question relevant when: selected( ${q104_M89_full_list} , '1')*<br>*Response constrained to: .>0* | |
| q204_M90 | ***q204_M90. Water for injection***<br>*Question relevant when: selected( ${q104_M90_full_list} , '1')* | |
| q204_M90a *(required)* | q204_M90a. What percent of patients require this item?<br>*Question relevant when: selected( ${q104_M90_full_list} , '1')*<br>*Response constrained to: .>0 and .<=100 or .=999* | |
| q204_M90b *(required)* | q204_M90b. How is the item administered, or given, to the patient?<br>*Question relevant when: selected( ${q104_M90_full_list} , '1')* | 1 Oral - solid (e.g. tablet)<br>2 Oral - liquid<br>3 Sublingual<br>4 Intravenous (IV)<br>5 Intramusculuar injection (IM)<br>6 Subcutaneous<br>7 Rectal<br>8 Inhaled (e.g. gas)<br>9 Inserted (e.g. vaginal pessaries)<br>10 Topical - cream<br>11 Topical - drops (e.g. eye drops)<br>12 Other<br>99 Don't know |

| Field | Question | Answer |
|---|---|---|
| q204_M90c *(required)* | q204_M90c. What is the item's smallest unit of measurement (mg, mcg, ml, g, etc)? *Question relevant when: selected( ${q104_M90_full_list} , '1')* | microgram: microgram (mcg); miligram: miligram (mg); miligram_dissolved_in_liquid: miligram (mg) dissolved in liquid (e.g. 2 mg per 3 militers); gram: gram (g); militer: militer (ml); centiliter: centiliter (cl); litre: litre (l); ml_of _solution: ml of solution (e.g. ml of 10% solution); litre_of_solution: litre of solution (e.g. 1 litre of 5% solution); cubic_meter: cubic meter (m^3); other: other; dont_know: don't know |
| q204_M90c.1 | q204_M90c.1 If other unit of measurement or route of administration, specify here: *Leave blank if not applicable* *Question relevant when: selected( ${q104_M90_full_list} , '1')* | |
| q204_M90d *(required)* | q204_M90d. In total, how many units are used to treat a patient during all of her care? *Remember to add all units! E.g. 5 days x 200 mg twice per day = 2,000mg* *Question relevant when: selected( ${q104_M90_full_list} , '1')* *Response constrained to: .>0* | |
| q204_M91 | ***q204_M91. Saline, glucose, water, etc. - Other 1: "[q104_M91_full_list_other]"*** *Question relevant when: selected( ${q104_M91_full_list} , '1')* | |
| q204_M91a *(required)* | q204_M91a. What percent of patients require this item? *Question relevant when: selected( ${q104_M91_full_list} , '1')* *Response constrained to: .>0 and .<=100 or .=999* | |
| q204_M91b *(required)* | q204_M91b. How is the item administered, or given, to the patient? *Question relevant when: selected( ${q104_M91_full_list} , '1')* | 1 Oral - solid (e.g. tablet); 2 Oral - liquid; 3 Sublingual; 4 Intravenous (IV); 5 Intramusculuar injection (IM); 6 Subcutaneous; 7 Rectal; 8 Inhaled (e.g. gas); 9 Inserted (e.g. vaginal pessaries); 10 Topical - cream; 11 Topical - drops (e.g. eye drops); 12 Other; 99 Don't know |

| Field | Question | Answer |
|---|---|---|
| q204_M91c *(required)* | q204_M91c. What is the item's smallest unit of measurement (mg, mcg, ml, g, etc)?<br>*Question relevant when: selected( ${q104_M91_full_list} , '1')* | microgram — microgram (mcg)<br>miligram — miligram (mg)<br>miligram_dissolved_in_liquid — miligram (mg) dissolved in liquid (e.g. 2 mg per 3 militers)<br>gram — gram (g)<br>militer — militer (ml)<br>centiliter — centiliter (cl)<br>litre — litre (l)<br>ml_of _solution — ml of solution (e.g. ml of 10% solution)<br>litre_of_solution — litre of solution (e.g. 1 litre of 5% solution)<br>cubic_meter — cubic meter (m^3)<br>other — other<br>dont_know — don't know |
| q204_M91c.1 | q204_M91c.1 If other unit of measurement or route of administration, specify here:<br>*Leave blank if not applicable*<br>*Question relevant when: selected( ${q104_M91_full_list} , '1')* | |
| q204_M91d *(required)* | q204_M91d. In total, how many units are used to treat a patient during all of her care?<br>*Remember to add all units! E.g. 5 days x 200 mg twice per day = 2,000mg*<br>*Question relevant when: selected( ${q104_M91_full_list} , '1')*<br>*Response constrained to: .>0* | |
| q204_M92 | ***q204_M92. Saline, glucose, water, etc. - Other 2: "[q104_M92_full_list_other]"***<br>*Question relevant when: selected( ${q104_M92_full_list} , '1')* | |
| q204_M92a *(required)* | q204_M92a. What percent of patients require this item?<br>*Question relevant when: selected( ${q104_M92_full_list} , '1')*<br>*Response constrained to: .>0 and .<=100 or .=999* | |
| q204_M92b *(required)* | q204_M92b. How is the item administered, or given, to the patient?<br>*Question relevant when: selected( ${q104_M92_full_list} , '1')* | 1 Oral - solid (e.g. tablet)<br>2 Oral - liquid<br>3 Sublingual<br>4 Intravenous (IV)<br>5 Intramusculuar injection (IM)<br>6 Subcutaneous<br>7 Rectal<br>8 Inhaled (e.g. gas)<br>9 Inserted (e.g. vaginal pessaries)<br>10 Topical - cream<br>11 Topical - drops (e.g. eye drops)<br>12 Other<br>99 Don't know |

| Field | Question | Answer |
|---|---|---|
| q204_M92c *(required)* | q204_M92c. What is the item's smallest unit of measurement (mg, mcg, ml, g, etc)?<br>*Question relevant when: selected( ${q104_M92_full_list} , '1')* | microgram — microgram (mcg)<br>miligram — miligram (mg)<br>miligram_dissolved_in_liquid — miligram (mg) dissolved in liquid (e.g. 2 mg per 3 militers)<br>gram — gram (g)<br>militer — militer (ml)<br>centiliter — centiliter (cl)<br>litre — litre (l)<br>ml_of _solution — ml of solution (e.g. ml of 10% solution)<br>litre_of_solution — litre of solution (e.g. 1 litre of 5% solution)<br>cubic_meter — cubic meter (m^3)<br>other — other<br>dont_know — don't know |
| q204_M92c.1 | q204_M92c.1 If other unit of measurement or route of administration, specify here:<br>*Leave blank if not applicable*<br>*Question relevant when: selected( ${q104_M92_full_list} , '1')* | |
| q204_M92d *(required)* | q204_M92d. In total, how many units are used to treat a patient during all of her care?<br>*Remember to add all units! E.g. 5 days x 200 mg twice per day = 2,000mg*<br>*Question relevant when: selected( ${q104_M92_full_list} , '1')*<br>*Response constrained to: .>0* | |
| q204_M93 | ***q204_M93. Saline, glucose, water, etc. - Other 3: "[q104_M93_full_list_other]"***<br>*Question relevant when: selected( ${q104_M93_full_list} , '1')* | |
| q204_M93a *(required)* | q204_M93a. What percent of patients require this item?<br>*Question relevant when: selected( ${q104_M93_full_list} , '1')*<br>*Response constrained to: .>0 and .<=100 or .=999* | |
| q204_M93b *(required)* | q204_M93b. How is the item administered, or given, to the patient?<br>*Question relevant when: selected( ${q104_M93_full_list} , '1')* | 1 Oral - solid (e.g. tablet)<br>2 Oral - liquid<br>3 Sublingual<br>4 Intravenous (IV)<br>5 Intramusculuar injection (IM)<br>6 Subcutaneous<br>7 Rectal<br>8 Inhaled (e.g. gas)<br>9 Inserted (e.g. vaginal pessaries)<br>10 Topical - cream<br>11 Topical - drops (e.g. eye drops)<br>12 Other<br>99 Don't know |

| Field | Question | Answer |
|---|---|---|
| q204_M93c *(required)* | q204_M93c. What is the item's smallest unit of measurement (mg, mcg, ml, g, etc)?<br>*Question relevant when: selected( ${q104_M93_full_list} , '1')* | microgram: microgram (mcg)<br>miligram: miligram (mg)<br>miligram_dissolved_in_liquid: miligram (mg) dissolved in liquid (e.g. 2 mg per 3 militers)<br>gram: gram (g)<br>militer: militer (ml)<br>centiliter: centiliter (cl)<br>litre: litre (l)<br>ml_of _solution: ml of solution (e.g. ml of 10% solution)<br>litre_of_solution: litre of solution (e.g. 1 litre of 5% solution)<br>cubic_meter: cubic meter (m^3)<br>other: other<br>dont_know: don't know |
| q204_M93c.1 | q204_M93c.1 If other unit of measurement or route of administration, specify here:<br>*Leave blank if not applicable*<br>*Question relevant when: selected( ${q104_M93_full_list} , '1')* | |
| q204_M93d *(required)* | q204_M93d. In total, how many units are used to treat a patient during all of her care?<br>*Remember to add all units! E.g. 5 days x 200 mg twice per day = 2,000mg*<br>*Question relevant when: selected( ${q104_M93_full_list} , '1')*<br>*Response constrained to: .>0* | |
| q204_M94 | ***q204_M94. Saline, glucose, water, etc. - Other 4: "[q104_M94_full_list_other]"***<br>*Question relevant when: selected( ${q104_M94_full_list} , '1')* | |
| q204_M94a *(required)* | q204_M94a. What percent of patients require this item?<br>*Question relevant when: selected( ${q104_M94_full_list} , '1')*<br>*Response constrained to: .>0 and .<=100 or .=999* | |
| q204_M94b *(required)* | q204_M94b. How is the item administered, or given, to the patient?<br>*Question relevant when: selected( ${q104_M94_full_list} , '1')* | 1 Oral - solid (e.g. tablet)<br>2 Oral - liquid<br>3 Sublingual<br>4 Intravenous (IV)<br>5 Intramusculuar injection (IM)<br>6 Subcutaneous<br>7 Rectal<br>8 Inhaled (e.g. gas)<br>9 Inserted (e.g. vaginal pessaries)<br>10 Topical - cream<br>11 Topical - drops (e.g. eye drops)<br>12 Other<br>99 Don't know |

| Field | Question | Answer |
|---|---|---|
| q204_M94c *(required)* | q204_M94c. What is the item's smallest unit of measurement (mg, mcg, ml, g, etc)?<br>*Question relevant when: selected( ${q104_M94_full_list} , '1')* | microgram — microgram (mcg)<br>miligram — miligram (mg)<br>miligram_dissolved_in_liquid — miligram (mg) dissolved in liquid (e.g. 2 mg per 3 militers)<br>gram — gram (g)<br>militer — militer (ml)<br>centiliter — centiliter (cl)<br>litre — litre (l)<br>ml_of _solution — ml of solution (e.g. ml of 10% solution)<br>litre_of_solution — litre of solution (e.g. 1 litre of 5% solution)<br>cubic_meter — cubic meter (m^3)<br>other — other<br>dont_know — don't know |
| q204_M94c.1 | q204_M94c.1 If other unit of measurement or route of administration, specify here:<br>*Leave blank if not applicable*<br>*Question relevant when: selected( ${q104_M94_full_list} , '1')* | |
| q204_M94d *(required)* | q204_M94d. In total, how many units are used to treat a patient during all of her care?<br>*Remember to add all units! E.g. 5 days x 200 mg twice per day = 2,000mg*<br>*Question relevant when: selected( ${q104_M94_full_list} , '1')*<br>*Response constrained to: .>0* | |
| q204_M95 | ***q204_M95. Saline, glucose, water, etc. - Other 5: "[q104_M95_full_list_other]"***<br>*Question relevant when: selected( ${q104_M95_full_list} , '1')* | |
| q204_M95a *(required)* | q204_M95a. What percent of patients require this item?<br>*Question relevant when: selected( ${q104_M95_full_list} , '1')*<br>*Response constrained to: .>0 and .<=100 or .=999* | |
| q204_M95b *(required)* | q204_M95b. How is the item administered, or given, to the patient?<br>*Question relevant when: selected( ${q104_M95_full_list} , '1')* | 1 Oral - solid (e.g. tablet)<br>2 Oral - liquid<br>3 Sublingual<br>4 Intravenous (IV)<br>5 Intramusculuar injection (IM)<br>6 Subcutaneous<br>7 Rectal<br>8 Inhaled (e.g. gas)<br>9 Inserted (e.g. vaginal pessaries)<br>10 Topical - cream<br>11 Topical - drops (e.g. eye drops)<br>12 Other<br>99 Don't know |

| Field | Question | Answer |
|---|---|---|
| q204_M95c *(required)* | q204_M95c. What is the item's smallest unit of measurement (mg, mcg, ml, g, etc)?<br>*Question relevant when: selected( ${q104_M95_full_list} , '1')* | microgram — microgram (mcg)<br>miligram — miligram (mg)<br>miligram_dissolved_in_liquid — miligram (mg) dissolved in liquid (e.g. 2 mg per 3 militers)<br>gram — gram (g)<br>militer — militer (ml)<br>centiliter — centiliter (cl)<br>litre — litre (l)<br>ml_of _solution — ml of solution (e.g. ml of 10% solution)<br>litre_of_solution — litre of solution (e.g. 1 litre of 5% solution)<br>cubic_meter — cubic meter (m^3)<br>other — other<br>dont_know — don't know |
| q204_M95c.1 | q204_M95c.1 If other unit of measurement or route of administration, specify here:<br>*Leave blank if not applicable*<br>*Question relevant when: selected( ${q104_M95_full_list} , '1')* | |
| q204_M95d *(required)* | q204_M95d. In total, how many units are used to treat a patient during all of her care?<br>*Remember to add all units! E.g. 5 days x 200 mg twice per day = 2,000mg*<br>*Question relevant when: selected( ${q104_M95_full_list} , '1')*<br>*Response constrained to: .>0* | |
| <p><strong><em>D. Medications (10)</em></strong></p><br>*Group relevant when: selected( ${section_two_skip_med} , '1')* | | |
| q204_med_notE9 | 204 CONTINUED For each of the following medications and other medical products that are used for incomplete abortion, can you tell me …? | |
| note_204_prost_utero | ***Prostaglandin, uterotonic, etc.*** | |
| q204_M96 | ***q204_M96. Misoprostol***<br>*Question relevant when: selected( ${q104_M96_full_list} , '1')* | |
| q204_M96a *(required)* | q204_M96a. What percent of patients require this item?<br>*Question relevant when: selected( ${q104_M96_full_list} , '1')*<br>*Response constrained to: .>0 and .<=100 or .=999* | |
| q204_M96b *(required)* | q204_M96b. How is the item administered, or given, to the patient?<br>*Question relevant when: selected( ${q104_M96_full_list} , '1')* | 1 Oral - solid (e.g. tablet)<br>2 Oral - liquid<br>3 Sublingual<br>4 Intravenous (IV)<br>5 Intramusculuar injection (IM)<br>6 Subcutaneous<br>7 Rectal<br>8 Inhaled (e.g. gas)<br>9 Inserted (e.g. vaginal pessaries)<br>10 Topical - cream<br>11 Topical - drops (e.g. eye drops) |

| Field | Question | Answer |
|---|---|---|
| | | 12 Other<br>99 Don't know |

| Field | Question | Answer |
|---|---|---|
| q204_M96c *(required)* | q204_M96c. What is the item's smallest unit of measurement (mg, mcg, ml, g, etc)?<br>*Question relevant when: selected( ${q104_M96_full_list} , '1')* | microgram — microgram (mcg)<br>miligram — miligram (mg)<br>miligram_dissolved_in_liquid — miligram (mg) dissolved in liquid (e.g. 2 mg per 3 militers)<br>gram — gram (g)<br>militer — militer (ml)<br>centiliter — centiliter (cl)<br>litre — litre (l)<br>ml_of _solution — ml of solution (e.g. ml of 10% solution)<br>litre_of_solution — litre of solution (e.g. 1 litre of 5% solution)<br>cubic_meter — cubic meter (m^3)<br>other — other<br>dont_know — don't know |
| q204_M96c.1 | q204_M96c.1 If other unit of measurement or route of administration, specify here:<br>*Leave blank if not applicable*<br>*Question relevant when: selected( ${q104_M96_full_list} , '1')* | |
| q204_M96d *(required)* | q204_M96d. In total, how many units are used to treat a patient during all of her care?<br>*Remember to add all units! E.g. 5 days x 200 mg twice per day = 2,000mg*<br>*Question relevant when: selected( ${q104_M96_full_list} , '1')*<br>*Response constrained to: .>0* | |
| q204_M97 | ***q204_M97. Ergometrine***<br>*Question relevant when: selected( ${q104_M97_full_list} , '1')* | |
| q204_M97a *(required)* | q204_M97a. What percent of patients require this item?<br>*Question relevant when: selected( ${q104_M97_full_list} , '1')*<br>*Response constrained to: .>0 and .<=100 or .=999* | |
| q204_M97b *(required)* | q204_M97b. How is the item administered, or given, to the patient?<br>*Question relevant when: selected( ${q104_M97_full_list} , '1')* | 1 Oral - solid (e.g. tablet)<br>2 Oral - liquid<br>3 Sublingual<br>4 Intravenous (IV)<br>5 Intramusculuar injection (IM)<br>6 Subcutaneous<br>7 Rectal<br>8 Inhaled (e.g. gas)<br>9 Inserted (e.g. vaginal pessaries)<br>10 Topical - cream<br>11 Topical - drops (e.g. eye drops)<br>12 Other<br>99 Don't know |

| Field | Question | Answer |
|---|---|---|
| q204_M97c *(required)* | q204_M97c. What is the item's smallest unit of measurement (mg, mcg, ml, g, etc)?<br>*Question relevant when: selected( ${q104_M97_full_list} , '1')* | microgram — microgram (mcg)<br>miligram — miligram (mg)<br>miligram_dissolved_in_liquid — miligram (mg) dissolved in liquid (e.g. 2 mg per 3 militers)<br>gram — gram (g)<br>militer — militer (ml)<br>centiliter — centiliter (cl)<br>litre — litre (l)<br>ml_of _solution — ml of solution (e.g. ml of 10% solution)<br>litre_of_solution — litre of solution (e.g. 1 litre of 5% solution)<br>cubic_meter — cubic meter (m^3)<br>other — other<br>dont_know — don't know |
| q204_M97c.1 | q204_M97c.1 If other unit of measurement or route of administration, specify here:<br>*Leave blank if not applicable*<br>*Question relevant when: selected( ${q104_M97_full_list} , '1')* | |
| q204_M97d *(required)* | q204_M97d. In total, how many units are used to treat a patient during all of her care?<br>*Remember to add all units! E.g. 5 days x 200 mg twice per day = 2,000mg*<br>*Question relevant when: selected( ${q104_M97_full_list} , '1')*<br>*Response constrained to: .>0* | |
| q204_M98 | ***q204_M98. Methylergonovine Maleate (Methergine)***<br>*Question relevant when: selected( ${q104_M98_full_list} , '1')* | |
| q204_M98a *(required)* | q204_M98a. What percent of patients require this item?<br>*Question relevant when: selected( ${q104_M98_full_list} , '1')*<br>*Response constrained to: .>0 and .<=100 or .=999* | |
| q204_M98b *(required)* | q204_M98b. How is the item administered, or given, to the patient?<br>*Question relevant when: selected( ${q104_M98_full_list} , '1')* | 1 Oral - solid (e.g. tablet)<br>2 Oral - liquid<br>3 Sublingual<br>4 Intravenous (IV)<br>5 Intramusculuar injection (IM)<br>6 Subcutaneous<br>7 Rectal<br>8 Inhaled (e.g. gas)<br>9 Inserted (e.g. vaginal pessaries)<br>10 Topical - cream<br>11 Topical - drops (e.g. eye drops)<br>12 Other<br>99 Don't know |

| Field | Question | Answer |
|---|---|---|
| q204_M98c *(required)* | q204_M98c. What is the item's smallest unit of measurement (mg, mcg, ml, g, etc)? *Question relevant when: selected( ${q104_M98_full_list} , '1')* | microgram: microgram (mcg)<br>miligram: miligram (mg)<br>miligram_dissolved_in_liquid: miligram (mg) dissolved in liquid (e.g. 2 mg per 3 militers)<br>gram: gram (g)<br>militer: militer (ml)<br>centiliter: centiliter (cl)<br>litre: litre (l)<br>ml_of _solution: ml of solution (e.g. ml of 10% solution)<br>litre_of_solution: litre of solution (e.g. 1 litre of 5% solution)<br>cubic_meter: cubic meter (m^3)<br>other: other<br>dont_know: don't know |
| q204_M98c.1 | q204_M98c.1 If other unit of measurement or route of administration, specify here: *Leave blank if not applicable* *Question relevant when: selected( ${q104_M98_full_list} , '1')* | |
| q204_M98d *(required)* | q204_M98d. In total, how many units are used to treat a patient during all of her care? *Remember to add all units! E.g. 5 days x 200 mg twice per day = 2,000mg* *Question relevant when: selected( ${q104_M98_full_list} , '1')* *Response constrained to: .>0* | |
| q204_M99 | ***q204_M99. Mifepristone*** *Question relevant when: selected( ${q104_M99_full_list} , '1')* | |
| q204_M99a *(required)* | q204_M99a. What percent of patients require this item? *Question relevant when: selected( ${q104_M99_full_list} , '1')* *Response constrained to: .>0 and .<=100 or .=999* | |
| q204_M99b *(required)* | q204_M99b. How is the item administered, or given, to the patient? *Question relevant when: selected( ${q104_M99_full_list} , '1')* | 1 Oral - solid (e.g. tablet)<br>2 Oral - liquid<br>3 Sublingual<br>4 Intravenous (IV)<br>5 Intramusculuar injection (IM)<br>6 Subcutaneous<br>7 Rectal<br>8 Inhaled (e.g. gas)<br>9 Inserted (e.g. vaginal pessaries)<br>10 Topical - cream<br>11 Topical - drops (e.g. eye drops)<br>12 Other<br>99 Don't know |

| Field | Question | Answer |
|---|---|---|
| q204_M99c *(required)* | q204_M99c. What is the item's smallest unit of measurement (mg, mcg, ml, g, etc)?<br>*Question relevant when: selected( ${q104_M99_full_list} , '1')* | microgram — microgram (mcg)<br>miligram — miligram (mg)<br>miligram_dissolved_in_liquid — miligram (mg) dissolved in liquid (e.g. 2 mg per 3 militers)<br>gram — gram (g)<br>militer — militer (ml)<br>centiliter — centiliter (cl)<br>litre — litre (l)<br>ml_of _solution — ml of solution (e.g. ml of 10% solution)<br>litre_of_solution — litre of solution (e.g. 1 litre of 5% solution)<br>cubic_meter — cubic meter (m^3)<br>other — other<br>dont_know — don't know |
| q204_M99c.1 | q204_M99c.1 If other unit of measurement or route of administration, specify here:<br>*Leave blank if not applicable*<br>*Question relevant when: selected( ${q104_M99_full_list} , '1')* | |
| q204_M99d *(required)* | q204_M99d. In total, how many units are used to treat a patient during all of her care?<br>*Remember to add all units! E.g. 5 days x 200 mg twice per day = 2,000mg*<br>*Question relevant when: selected( ${q104_M99_full_list} , '1')*<br>*Response constrained to: .>0* | |
| q204_M100 | ***q204_M100. Oxytocin***<br>*Question relevant when: selected( ${q104_M100_full_list} , '1')* | |
| q204_M100a *(required)* | q204_M100a. What percent of patients require this item?<br>*Question relevant when: selected( ${q104_M100_full_list} , '1')*<br>*Response constrained to: .>0 and .<=100 or .=999* | |
| q204_M100b *(required)* | q204_M100b. How is the item administered, or given, to the patient?<br>*Question relevant when: selected( ${q104_M100_full_list} , '1')* | 1 Oral - solid (e.g. tablet)<br>2 Oral - liquid<br>3 Sublingual<br>4 Intravenous (IV)<br>5 Intramusculuar injection (IM)<br>6 Subcutaneous<br>7 Rectal<br>8 Inhaled (e.g. gas)<br>9 Inserted (e.g. vaginal pessaries)<br>10 Topical - cream<br>11 Topical - drops (e.g. eye drops)<br>12 Other<br>99 Don't know |

| Field | Question | Answer |
|---|---|---|
| q204_M100c *(required)* | q204_M100c. What is the item's smallest unit of measurement (mg, mcg, ml, g, etc)?<br>*Question relevant when: selected( ${q104_M100_full_list} , '1')* | <table><tr><td></td><td>microgram</td><td>microgram (mcg)</td></tr><tr><td></td><td>miligram</td><td>miligram (mg)</td></tr><tr><td></td><td>miligram_dissolved_in_liquid</td><td>miligram (mg) dissolved in liquid (e.g. 2 mg per 3 militers)</td></tr><tr><td></td><td>gram</td><td>gram (g)</td></tr><tr><td></td><td>militer</td><td>militer (ml)</td></tr><tr><td></td><td>centiliter</td><td>centiliter (cl)</td></tr><tr><td></td><td>litre</td><td>litre (l)</td></tr><tr><td></td><td>ml_of _solution</td><td>ml of solution (e.g. ml of 10% solution)</td></tr><tr><td></td><td>litre_of_solution</td><td>litre of solution (e.g. 1 litre of 5% solution)</td></tr><tr><td></td><td>cubic_meter</td><td>cubic meter (m^3)</td></tr><tr><td></td><td>other</td><td>other</td></tr><tr><td></td><td>dont_know</td><td>don't know</td></tr></table> |
| q204_M100c.1 | q204_M100c.1 If other unit of measurement or route of administration, specify here:<br>*Leave blank if not applicable*<br>*Question relevant when: selected( ${q104_M100_full_list} , '1')* | |
| q204_M100d *(required)* | q204_M100d. In total, how many units are used to treat a patient during all of her care?<br>*Remember to add all units! E.g. 5 days x 200 mg twice per day = 2,000mg*<br>*Question relevant when: selected( ${q104_M100_full_list} , '1')*<br>*Response constrained to: .>0* | |
| q204_M101 | ***q204_M101. Prostaglandin, uterotonic, etc. - Other 1: "[q104_M101_full_list_other]"***<br>*Question relevant when: selected( ${q104_M101_full_list} , '1')* | |
| q204_M101a *(required)* | q204_M101a. What percent of patients require this item?<br>*Question relevant when: selected( ${q104_M101_full_list} , '1')*<br>*Response constrained to: .>0 and .<=100 or .=999* | |
| q204_M101b *(required)* | q204_M101b. How is the item administered, or given, to the patient?<br>*Question relevant when: selected( ${q104_M101_full_list} , '1')* | <table><tr><td>1</td><td>Oral - solid (e.g. tablet)</td></tr><tr><td>2</td><td>Oral - liquid</td></tr><tr><td>3</td><td>Sublingual</td></tr><tr><td>4</td><td>Intravenous (IV)</td></tr><tr><td>5</td><td>Intramusculuar injection (IM)</td></tr><tr><td>6</td><td>Subcutaneous</td></tr><tr><td>7</td><td>Rectal</td></tr><tr><td>8</td><td>Inhaled (e.g. gas)</td></tr><tr><td>9</td><td>Inserted (e.g. vaginal pessaries)</td></tr><tr><td>10</td><td>Topical - cream</td></tr><tr><td>11</td><td>Topical - drops (e.g. eye drops)</td></tr><tr><td>12</td><td>Other</td></tr><tr><td>99</td><td>Don't know</td></tr></table> |

| Field | Question | Answer |
|---|---|---|
| q204_M101c *(required)* | q204_M101c. What is the item's smallest unit of measurement (mg, mcg, ml, g, etc)?<br>*Question relevant when: selected( ${q104_M101_full_list} , '1')* | microgram — microgram (mcg)<br>miligram — miligram (mg)<br>miligram_dissolved_in_liquid — miligram (mg) dissolved in liquid (e.g. 2 mg per 3 militers)<br>gram — gram (g)<br>militer — militer (ml)<br>centiliter — centiliter (cl)<br>litre — litre (l)<br>ml_of _solution — ml of solution (e.g. ml of 10% solution)<br>litre_of_solution — litre of solution (e.g. 1 litre of 5% solution)<br>cubic_meter — cubic meter (m^3)<br>other — other<br>dont_know — don't know |
| q204_M101c.1 | q204_M101c.1 If other unit of measurement or route of administration, specify here:<br>*Leave blank if not applicable*<br>*Question relevant when: selected( ${q104_M101_full_list} , '1')* | |
| q204_M101d *(required)* | q204_M101d. In total, how many units are used to treat a patient during all of her care?<br>*Remember to add all units! E.g. 5 days x 200 mg twice per day = 2,000mg*<br>*Question relevant when: selected( ${q104_M101_full_list} , '1')*<br>*Response constrained to: .>0* | |
| q204_M102 | ***q204_M102. Prostaglandin, uterotonic, etc. - Other 2: "[q104_M102_full_list_other]"***<br>*Question relevant when: selected( ${q104_M102_full_list} , '1')* | |
| q204_M102a *(required)* | q204_M102a. What percent of patients require this item?<br>*Question relevant when: selected( ${q104_M102_full_list} , '1')*<br>*Response constrained to: .>0 and .<=100 or .=999* | |
| q204_M102b *(required)* | q204_M102b. How is the item administered, or given, to the patient?<br>*Question relevant when: selected( ${q104_M102_full_list} , '1')* | 1 Oral - solid (e.g. tablet)<br>2 Oral - liquid<br>3 Sublingual<br>4 Intravenous (IV)<br>5 Intramusculuar injection (IM)<br>6 Subcutaneous<br>7 Rectal<br>8 Inhaled (e.g. gas)<br>9 Inserted (e.g. vaginal pessaries)<br>10 Topical - cream<br>11 Topical - drops (e.g. eye drops)<br>12 Other<br>99 Don't know |

| Field | Question | Answer |
|---|---|---|
| q204_M102c *(required)* | q204_M102c. What is the item's smallest unit of measurement (mg, mcg, ml, g, etc)?<br>*Question relevant when: selected( ${q104_M102_full_list} , '1')* | microgram: microgram (mcg)<br>miligram: miligram (mg)<br>miligram_dissolved_in_liquid: miligram (mg) dissolved in liquid (e.g. 2 mg per 3 militers)<br>gram: gram (g)<br>militer: militer (ml)<br>centiliter: centiliter (cl)<br>litre: litre (l)<br>ml_of _solution: ml of solution (e.g. ml of 10% solution)<br>litre_of_solution: litre of solution (e.g. 1 litre of 5% solution)<br>cubic_meter: cubic meter (m^3)<br>other: other<br>dont_know: don't know |
| q204_M102c.1 | q204_M102c.1 If other unit of measurement or route of administration, specify here:<br>*Leave blank if not applicable*<br>*Question relevant when: selected( ${q104_M102_full_list} , '1')* | |
| q204_M102d *(required)* | q204_M102d. In total, how many units are used to treat a patient during all of her care?<br>*Remember to add all units! E.g. 5 days x 200 mg twice per day = 2,000mg*<br>*Question relevant when: selected( ${q104_M102_full_list} , '1')*<br>*Response constrained to: .>0* | |
| q204_M103 | ***q204_M103. Prostaglandin, uterotonic, etc. - Other 3: "[q104_M103_full_list_other]"***<br>*Question relevant when: selected( ${q104_M104_full_list} , '1')* | |
| q204_M103a *(required)* | q204_M103a. What percent of patients require this item?<br>*Question relevant when: selected( ${q104_M104_full_list} , '1')*<br>*Response constrained to: .>0 and .<=100 or .=999* | |
| q204_M103b *(required)* | q204_M103b. How is the item administered, or given, to the patient?<br>*Question relevant when: selected( ${q104_M104_full_list} , '1')* | 1 Oral - solid (e.g. tablet)<br>2 Oral - liquid<br>3 Sublingual<br>4 Intravenous (IV)<br>5 Intramusculuar injection (IM)<br>6 Subcutaneous<br>7 Rectal<br>8 Inhaled (e.g. gas)<br>9 Inserted (e.g. vaginal pessaries)<br>10 Topical - cream<br>11 Topical - drops (e.g. eye drops)<br>12 Other<br>99 Don't know |

| Field | Question | Answer |
|---|---|---|
| q204_M103c *(required)* | q204_M103c. What is the item's smallest unit of measurement (mg, mcg, ml, g, etc)?<br>*Question relevant when: selected( ${q104_M104_full_list} , '1')* | microgram — microgram (mcg)<br>miligram — miligram (mg)<br>miligram_dissolved_in_liquid — miligram (mg) dissolved in liquid (e.g. 2 mg per 3 militers)<br>gram — gram (g)<br>militer — militer (ml)<br>centiliter — centiliter (cl)<br>litre — litre (l)<br>ml_of _solution — ml of solution (e.g. ml of 10% solution)<br>litre_of_solution — litre of solution (e.g. 1 litre of 5% solution)<br>cubic_meter — cubic meter (m^3)<br>other — other<br>dont_know — don't know |
| q204_M103c.1 | q204_M103c.1 If other unit of measurement or route of administration, specify here:<br>*Leave blank if not applicable*<br>*Question relevant when: selected( ${q104_M104_full_list} , '1')* | |
| q204_M103d *(required)* | q204_M103d. In total, how many units are used to treat a patient during all of her care?<br>*Remember to add all units! E.g. 5 days x 200 mg twice per day = 2,000mg*<br>*Question relevant when: selected( ${q104_M104_full_list} , '1')*<br>*Response constrained to: .>0* | |
| q204_M104 | ***q204_M104. Prostaglandin, uterotonic, etc. - Other 4: "[q104_M104_full_list_other]"***<br>*Question relevant when: selected( ${q104_M104_full_list} , '1')* | |
| q204_M104a *(required)* | q204_M104a. What percent of patients require this item?<br>*Question relevant when: selected( ${q104_M104_full_list} , '1')*<br>*Response constrained to: .>0 and .<=100 or .=999* | |
| q204_M104b *(required)* | q204_M104b. How is the item administered, or given, to the patient?<br>*Question relevant when: selected( ${q104_M104_full_list} , '1')* | 1 Oral - solid (e.g. tablet)<br>2 Oral - liquid<br>3 Sublingual<br>4 Intravenous (IV)<br>5 Intramuscular injection (IM)<br>6 Subcutaneous<br>7 Rectal<br>8 Inhaled (e.g. gas)<br>9 Inserted (e.g. vaginal pessaries)<br>10 Topical - cream<br>11 Topical - drops (e.g. eye drops)<br>12 Other<br>99 Don't know |

| Field | Question | Answer |
|---|---|---|
| q204_M104c *(required)* | q204_M104c. What is the item's smallest unit of measurement (mg, mcg, ml, g, etc)?<br>*Question relevant when: selected( ${q104_M104_full_list} , '1')* | microgram — microgram (mcg)<br>miligram — miligram (mg)<br>miligram_dissolved_in_liquid — miligram (mg) dissolved in liquid (e.g. 2 mg per 3 militers)<br>gram — gram (g)<br>militer — militer (ml)<br>centiliter — centiliter (cl)<br>litre — litre (l)<br>ml_of _solution — ml of solution (e.g. ml of 10% solution)<br>litre_of_solution — litre of solution (e.g. 1 litre of 5% solution)<br>cubic_meter — cubic meter (m^3)<br>other — other<br>dont_know — don't know |
| q204_M104c.1 | q204_M104c.1 If other unit of measurement or route of administration, specify here:<br>*Leave blank if not applicable*<br>*Question relevant when: selected( ${q104_M104_full_list} , '1')* | |
| q204_M104d *(required)* | q204_M104d. In total, how many units are used to treat a patient during all of her care?<br>*Remember to add all units! E.g. 5 days x 200 mg twice per day = 2,000mg*<br>*Question relevant when: selected( ${q104_M104_full_list} , '1')*<br>*Response constrained to: .>0* | |
| q204_M105 | ***q204_M105. Prostaglandin, uterotonic, etc. - Other 5: "[q104_M105_full_list_other]"***<br>*Question relevant when: selected( ${q104_M105_full_list} , '1')* | |
| q204_M105a *(required)* | q204_M105a. What percent of patients require this item?<br>*Question relevant when: selected( ${q104_M105_full_list} , '1')*<br>*Response constrained to: .>0 and .<=100 or .=999* | |
| q204_M105b *(required)* | q204_M105b. How is the item administered, or given, to the patient?<br>*Question relevant when: selected( ${q104_M105_full_list} , '1')* | 1 Oral - solid (e.g. tablet)<br>2 Oral - liquid<br>3 Sublingual<br>4 Intravenous (IV)<br>5 Intramusculuar injection (IM)<br>6 Subcutaneous<br>7 Rectal<br>8 Inhaled (e.g. gas)<br>9 Inserted (e.g. vaginal pessaries)<br>10 Topical - cream<br>11 Topical - drops (e.g. eye drops)<br>12 Other<br>99 Don't know |

| Field | Question | Answer |
|---|---|---|
| q204_M105c *(required)* | q204_M105c. What is the item's smallest unit of measurement (mg, mcg, ml, g, etc)?<br>*Question relevant when: selected( ${q104_M105_full_list} , '1')* | microgram — microgram (mcg)<br>miligram — miligram (mg)<br>miligram_dissolved_in_liquid — miligram (mg) dissolved in liquid (e.g. 2 mg per 3 militers)<br>gram — gram (g)<br>militer — militer (ml)<br>centiliter — centiliter (cl)<br>litre — litre (l)<br>ml_of _solution — ml of solution (e.g. ml of 10% solution)<br>litre_of_solution — litre of solution (e.g. 1 litre of 5% solution)<br>cubic_meter — cubic meter (m^3)<br>other — other<br>dont_know — don't know |
| q204_M105c.1 | q204_M105c.1 If other unit of measurement or route of administration, specify here:<br>*Leave blank if not applicable*<br>*Question relevant when: selected( ${q104_M105_full_list} , '1')* | |
| q204_M105d *(required)* | q204_M105d. In total, how many units are used to treat a patient during all of her care?<br>*Remember to add all units! E.g. 5 days x 200 mg twice per day = 2,000mg*<br>*Question relevant when: selected( ${q104_M105_full_list} , '1')*<br>*Response constrained to: .>0* | |
| <p><strong><em>D. Medications (11)</em></strong></p><br>*Group relevant when: selected( ${section_two_skip_med} , '1')* | | |
| q204_med_notE10 | 204 CONTINUED For each of the following medications and other medical products that are used for incomplete abortion, can you tell me …? | |
| note_204_vitamin | ***Vitamin, immunological, etc.*** | |
| q204_M106 | ***q204_M106. Ferrous sulphate (tablets)***<br>*Question relevant when: selected( ${q104_M106_full_list} , '1')* | |
| q204_M106a *(required)* | q204_M106a. What percent of patients require this item?<br>*Question relevant when: selected( ${q104_M106_full_list} , '1')*<br>*Response constrained to: .>0 and .<=100 or .=999* | |
| q204_M106b *(required)* | q204_M106b. How is the item administered, or given, to the patient?<br>*Question relevant when: selected( ${q104_M106_full_list} , '1')* | 1 Oral - solid (e.g. tablet)<br>2 Oral - liquid<br>3 Sublingual<br>4 Intravenous (IV)<br>5 Intramusculuar injection (IM)<br>6 Subcutaneous<br>7 Rectal<br>8 Inhaled (e.g. gas)<br>9 Inserted (e.g. vaginal pessaries)<br>10 Topical - cream<br>11 Topical - drops (e.g. eye drops) |

| Field | Question | Answer |
|---|---|---|
| | | 12 Other<br>99 Don't know |

| Field | Question | Answer |
|---|---|---|
| q204_M106c *(required)* | q204_M106c. What is the item's smallest unit of measurement (mg, mcg, ml, g, etc)?<br>*Question relevant when: selected( ${q104_M106_full_list} , '1')* | microgram — microgram (mcg)<br>miligram — miligram (mg)<br>miligram_dissolved_in_liquid — miligram (mg) dissolved in liquid (e.g. 2 mg per 3 militers)<br>gram — gram (g)<br>militer — militer (ml)<br>centiliter — centiliter (cl)<br>litre — litre (l)<br>ml_of _solution — ml of solution (e.g. ml of 10% solution)<br>litre_of_solution — litre of solution (e.g. 1 litre of 5% solution)<br>cubic_meter — cubic meter (m^3)<br>other — other<br>dont_know — don't know |
| q204_M106c.1 | q204_M106c.1 If other unit of measurement or route of administration, specify here:<br>*Leave blank if not applicable*<br>*Question relevant when: selected( ${q104_M106_full_list} , '1')* | |
| q204_M106d *(required)* | q204_M106d. In total, how many units are used to treat a patient during all of her care?<br>*Remember to add all units! E.g. 5 days x 200 mg twice per day = 2,000mg*<br>*Question relevant when: selected( ${q104_M106_full_list} , '1')*<br>*Response constrained to: .>0* | |
| q204_M107 | ***q204_M107. Ferrous sulphate + folic acid (Ferrotone capsules)***<br>*Question relevant when: selected( ${q104_M107_full_list} , '1')* | |
| q204_M107a *(required)* | q204_M107a. What percent of patients require this item?<br>*Question relevant when: selected( ${q104_M107_full_list} , '1')*<br>*Response constrained to: .>0 and .<=100 or .=999* | |
| q204_M107b *(required)* | q204_M107b. How is the item administered, or given, to the patient?<br>*Question relevant when: selected( ${q104_M107_full_list} , '1')* | 1 Oral - solid (e.g. tablet)<br>2 Oral - liquid<br>3 Sublingual<br>4 Intravenous (IV)<br>5 Intramusculuar injection (IM)<br>6 Subcutaneous<br>7 Rectal<br>8 Inhaled (e.g. gas)<br>9 Inserted (e.g. vaginal pessaries)<br>10 Topical - cream<br>11 Topical - drops (e.g. eye drops)<br>12 Other<br>99 Don't know |

| Field | Question | Answer |
|---|---|---|
| q204_M107c *(required)* | q204_M107c. What is the item's smallest unit of measurement (mg, mcg, ml, g, etc)?<br>*Question relevant when: selected( ${q104_M107_full_list} , '1')* | microgram — microgram (mcg)<br>miligram — miligram (mg)<br>miligram_dissolved_in_liquid — miligram (mg) dissolved in liquid (e.g. 2 mg per 3 militers)<br>gram — gram (g)<br>militer — militer (ml)<br>centiliter — centiliter (cl)<br>litre — litre (l)<br>ml_of _solution — ml of solution (e.g. ml of 10% solution)<br>litre_of_solution — litre of solution (e.g. 1 litre of 5% solution)<br>cubic_meter — cubic meter (m^3)<br>other — other<br>dont_know — don't know |
| q204_M107c.1 | q204_M107c.1 If other unit of measurement or route of administration, specify here:<br>*Leave blank if not applicable*<br>*Question relevant when: selected( ${q104_M107_full_list} , '1')* | |
| q204_M107d *(required)* | q204_M107d. In total, how many units are used to treat a patient during all of her care?<br>*Remember to add all units! E.g. 5 days x 200 mg twice per day = 2,000mg*<br>*Question relevant when: selected( ${q104_M107_full_list} , '1')*<br>*Response constrained to: .>0* | |
| q204_M108 | ***q204_M108. Folic acid (Tablets)***<br>*Question relevant when: selected( ${q104_M108_full_list} , '1')* | |
| q204_M108a *(required)* | q204_M108a. What percent of patients require this item?<br>*Question relevant when: selected( ${q104_M108_full_list} , '1')*<br>*Response constrained to: .>0 and .<=100 or .=999* | |
| q204_M108b *(required)* | q204_M108b. How is the item administered, or given, to the patient?<br>*Question relevant when: selected( ${q104_M108_full_list} , '1')* | 1 Oral - solid (e.g. tablet)<br>2 Oral - liquid<br>3 Sublingual<br>4 Intravenous (IV)<br>5 Intramusculuar injection (IM)<br>6 Subcutaneous<br>7 Rectal<br>8 Inhaled (e.g. gas)<br>9 Inserted (e.g. vaginal pessaries)<br>10 Topical - cream<br>11 Topical - drops (e.g. eye drops)<br>12 Other<br>99 Don't know |

| Field | Question | Answer |
|---|---|---|
| q204_M108c *(required)* | q204_M108c. What is the item's smallest unit of measurement (mg, mcg, ml, g, etc)?<br>*Question relevant when: selected( ${q104_M108_full_list} , '1')* | microgram — microgram (mcg)<br>miligram — miligram (mg)<br>miligram_dissolved_in_liquid — miligram (mg) dissolved in liquid (e.g. 2 mg per 3 militers)<br>gram — gram (g)<br>militer — militer (ml)<br>centiliter — centiliter (cl)<br>litre — litre (l)<br>ml_of _solution — ml of solution (e.g. ml of 10% solution)<br>litre_of_solution — litre of solution (e.g. 1 litre of 5% solution)<br>cubic_meter — cubic meter (m^3)<br>other — other<br>dont_know — don't know |
| q204_M108c.1 | q204_M108c.1 If other unit of measurement or route of administration, specify here:<br>*Leave blank if not applicable*<br>*Question relevant when: selected( ${q104_M108_full_list} , '1')* | |
| q204_M108d *(required)* | q204_M108d. In total, how many units are used to treat a patient during all of her care?<br>*Remember to add all units! E.g. 5 days x 200 mg twice per day = 2,000mg*<br>*Question relevant when: selected( ${q104_M108_full_list} , '1')*<br>*Response constrained to: .>0* | |
| q204_M109 | ***q204_M109. Serum anti-tetanus***<br>*Question relevant when: selected( ${q104_M109_full_list} , '1')* | |
| q204_M109a *(required)* | q204_M109a. What percent of patients require this item?<br>*Question relevant when: selected( ${q104_M109_full_list} , '1')*<br>*Response constrained to: .>0 and .<=100 or .=999* | |
| q204_M109b *(required)* | q204_M109b. How is the item administered, or given, to the patient?<br>*Question relevant when: selected( ${q104_M109_full_list} , '1')* | 1 Oral - solid (e.g. tablet)<br>2 Oral - liquid<br>3 Sublingual<br>4 Intravenous (IV)<br>5 Intramusculuar injection (IM)<br>6 Subcutaneous<br>7 Rectal<br>8 Inhaled (e.g. gas)<br>9 Inserted (e.g. vaginal pessaries)<br>10 Topical - cream<br>11 Topical - drops (e.g. eye drops)<br>12 Other<br>99 Don't know |

| Field | Question | Answer |
|---|---|---|
| q204_M109c *(required)* | q204_M109c. What is the item's smallest unit of measurement (mg, mcg, ml, g, etc)?<br>*Question relevant when: selected( ${q104_M109_full_list} , '1')* | microgram — microgram (mcg)<br>miligram — miligram (mg)<br>miligram_dissolved_in_liquid — miligram (mg) dissolved in liquid (e.g. 2 mg per 3 militers)<br>gram — gram (g)<br>militer — militer (ml)<br>centiliter — centiliter (cl)<br>litre — litre (l)<br>ml_of _solution — ml of solution (e.g. ml of 10% solution)<br>litre_of_solution — litre of solution (e.g. 1 litre of 5% solution)<br>cubic_meter — cubic meter (m^3)<br>other — other<br>dont_know — don't know |
| q204_M109c.1 | q204_M109c.1 If other unit of measurement or route of administration, specify here:<br>*Leave blank if not applicable*<br>*Question relevant when: selected( ${q104_M109_full_list} , '1')* | |
| q204_M109d *(required)* | q204_M109d. In total, how many units are used to treat a patient during all of her care?<br>*Remember to add all units! E.g. 5 days x 200 mg twice per day = 2,000mg*<br>*Question relevant when: selected( ${q104_M109_full_list} , '1')*<br>*Response constrained to: .>0* | |
| q204_M110 | ***q204_M110. Anti D (Rh)-Immunoglobulin***<br>*Question relevant when: selected( ${q104_M110_full_list} , '1')* | |
| q204_M110a *(required)* | q204_M110a. What percent of patients require this item?<br>*Question relevant when: selected( ${q104_M110_full_list} , '1')*<br>*Response constrained to: .>0 and .<=100 or .=999* | |
| q204_M110b *(required)* | q204_M110b. How is the item administered, or given, to the patient?<br>*Question relevant when: selected( ${q104_M110_full_list} , '1')* | 1 Oral - solid (e.g. tablet)<br>2 Oral - liquid<br>3 Sublingual<br>4 Intravenous (IV)<br>5 Intramusculuar injection (IM)<br>6 Subcutaneous<br>7 Rectal<br>8 Inhaled (e.g. gas)<br>9 Inserted (e.g. vaginal pessaries)<br>10 Topical - cream<br>11 Topical - drops (e.g. eye drops)<br>12 Other<br>99 Don't know |

| Field | Question | Answer |
|---|---|---|
| q204_M110c *(required)* | q204_M110c. What is the item's smallest unit of measurement (mg, mcg, ml, g, etc)?<br>*Question relevant when: selected( ${q104_M110_full_list} , '1')* | microgram: microgram (mcg)<br>miligram: miligram (mg)<br>miligram_dissolved_in_liquid: miligram (mg) dissolved in liquid (e.g. 2 mg per 3 militers)<br>gram: gram (g)<br>militer: militer (ml)<br>centiliter: centiliter (cl)<br>litre: litre (l)<br>ml_of _solution: ml of solution (e.g. ml of 10% solution)<br>litre_of_solution: litre of solution (e.g. 1 litre of 5% solution)<br>cubic_meter: cubic meter (m^3)<br>other: other<br>dont_know: don't know |
| q204_M110c.1 | q204_M110c.1 If other unit of measurement or route of administration, specify here:<br>*Leave blank if not applicable*<br>*Question relevant when: selected( ${q104_M110_full_list} , '1')* | |
| q204_M110d *(required)* | q204_M110d. In total, how many units are used to treat a patient during all of her care?<br>*Remember to add all units! E.g. 5 days x 200 mg twice per day = 2,000mg*<br>*Question relevant when: selected( ${q104_M110_full_list} , '1')*<br>*Response constrained to: .>0* | |
| q204_M111 | ***q204_M111. Vitamin, immunological, etc. - Other 1: "[q104_M111_full_list_other]"***<br>*Question relevant when: selected( ${q104_M111_full_list} , '1')* | |
| q204_M111a *(required)* | q204_M111a. What percent of patients require this item?<br>*Question relevant when: selected( ${q104_M111_full_list} , '1')*<br>*Response constrained to: .>0 and .<=100 or .=999* | |
| q204_M111b *(required)* | q204_M111b. How is the item administered, or given, to the patient?<br>*Question relevant when: selected( ${q104_M111_full_list} , '1')* | 1 Oral - solid (e.g. tablet)<br>2 Oral - liquid<br>3 Sublingual<br>4 Intravenous (IV)<br>5 Intramusculuar injection (IM)<br>6 Subcutaneous<br>7 Rectal<br>8 Inhaled (e.g. gas)<br>9 Inserted (e.g. vaginal pessaries)<br>10 Topical - cream<br>11 Topical - drops (e.g. eye drops)<br>12 Other<br>99 Don't know |

| Field | Question | Answer |
|---|---|---|
| q204_M111c *(required)* | q204_M111c. What is the item's smallest unit of measurement (mg, mcg, ml, g, etc)?<br>*Question relevant when: selected( ${q104_M111_full_list} , '1')* | microgram — microgram (mcg)<br>miligram — miligram (mg)<br>miligram_dissolved_in_liquid — miligram (mg) dissolved in liquid (e.g. 2 mg per 3 militers)<br>gram — gram (g)<br>militer — militer (ml)<br>centiliter — centiliter (cl)<br>litre — litre (l)<br>ml_of _solution — ml of solution (e.g. ml of 10% solution)<br>litre_of_solution — litre of solution (e.g. 1 litre of 5% solution)<br>cubic_meter — cubic meter (m^3)<br>other — other<br>dont_know — don't know |
| q204_M111c.1 | q204_M111c.1 If other unit of measurement or route of administration, specify here:<br>*Leave blank if not applicable*<br>*Question relevant when: selected( ${q104_M111_full_list} , '1')* | |
| q204_M111d *(required)* | q204_M111d. In total, how many units are used to treat a patient during all of her care?<br>*Remember to add all units! E.g. 5 days x 200 mg twice per day = 2,000mg*<br>*Question relevant when: selected( ${q104_M111_full_list} , '1')*<br>*Response constrained to: .>0* | |
| q204_M112 | ***q204_M112. Vitamin, immunological, etc. - Other 2: "[q104_M112_full_list_other]"***<br>*Question relevant when: selected( ${q104_M112_full_list} , '1')* | |
| q204_M112a *(required)* | q204_M112a. What percent of patients require this item?<br>*Question relevant when: selected( ${q104_M112_full_list} , '1')*<br>*Response constrained to: .>0 and .<=100 or .=999* | |
| q204_M112b *(required)* | q204_M112b. How is the item administered, or given, to the patient?<br>*Question relevant when: selected( ${q104_M112_full_list} , '1')* | 1 Oral - solid (e.g. tablet)<br>2 Oral - liquid<br>3 Sublingual<br>4 Intravenous (IV)<br>5 Intramusculuar injection (IM)<br>6 Subcutaneous<br>7 Rectal<br>8 Inhaled (e.g. gas)<br>9 Inserted (e.g. vaginal pessaries)<br>10 Topical - cream<br>11 Topical - drops (e.g. eye drops)<br>12 Other<br>99 Don't know |

| Field | Question | Answer |
|---|---|---|
| q204_M112c *(required)* | q204_M112c. What is the item's smallest unit of measurement (mg, mcg, ml, g, etc)?<br>*Question relevant when: selected( ${q104_M112_full_list} , '1')* | \| \| microgram \| microgram (mcg) \|<br>\| \| miligram \| miligram (mg) \|<br>\| \| miligram_dissolved_in_liquid \| miligram (mg) dissolved in liquid (e.g. 2 mg per 3 militers) \|<br>\| \| gram \| gram (g) \|<br>\| \| militer \| militer (ml) \|<br>\| \| centiliter \| centiliter (cl) \|<br>\| \| litre \| litre (l) \|<br>\| \| ml_of _solution \| ml of solution (e.g. ml of 10% solution) \|<br>\| \| litre_of_solution \| litre of solution (e.g. 1 litre of 5% solution) \|<br>\| \| cubic_meter \| cubic meter (m^3) \|<br>\| \| other \| other \|<br>\| \| dont_know \| don't know \| |
| q204_M112c.1 | q204_M112c.1 If other unit of measurement or route of administration, specify here:<br>*Leave blank if not applicable*<br>*Question relevant when: selected( ${q104_M112_full_list} , '1')* | |
| q204_M112d *(required)* | q204_M112d. In total, how many units are used to treat a patient during all of her care?<br>*Remember to add all units! E.g. 5 days x 200 mg twice per day = 2,000mg*<br>*Question relevant when: selected( ${q104_M112_full_list} , '1')*<br>*Response constrained to: .>0* | |
| q204_M113 | ***q204_M113. Vitamin, immunological, etc. - Other 3: "[q104_M113_full_list_other]"***<br>*Question relevant when: selected( ${q104_M113_full_list} , '1')* | |
| q204_M113a *(required)* | q204_M113a. What percent of patients require this item?<br>*Question relevant when: selected( ${q104_M113_full_list} , '1')*<br>*Response constrained to: .>0 and .<=100 or .=999* | |
| q204_M113b *(required)* | q204_M113b. How is the item administered, or given, to the patient?<br>*Question relevant when: selected( ${q104_M113_full_list} , '1')* | \| \| 1 \| Oral - solid (e.g. tablet) \|<br>\| \| 2 \| Oral - liquid \|<br>\| \| 3 \| Sublingual \|<br>\| \| 4 \| Intravenous (IV) \|<br>\| \| 5 \| Intramusculuar injection (IM) \|<br>\| \| 6 \| Subcutaneous \|<br>\| \| 7 \| Rectal \|<br>\| \| 8 \| Inhaled (e.g. gas) \|<br>\| \| 9 \| Inserted (e.g. vaginal pessaries) \|<br>\| \| 10 \| Topical - cream \|<br>\| \| 11 \| Topical - drops (e.g. eye drops) \|<br>\| \| 12 \| Other \|<br>\| \| 99 \| Don't know \| |

| Field | Question | Answer |
|---|---|---|
| q204_M113c *(required)* | q204_M113c. What is the item's smallest unit of measurement (mg, mcg, ml, g, etc)?<br>*Question relevant when: selected( ${q104_M113_full_list} , '1')* | microgram — microgram (mcg)<br>miligram — miligram (mg)<br>miligram_dissolved_in_liquid — miligram (mg) dissolved in liquid (e.g. 2 mg per 3 militers)<br>gram — gram (g)<br>militer — militer (ml)<br>centiliter — centiliter (cl)<br>litre — litre (l)<br>ml_of _solution — ml of solution (e.g. ml of 10% solution)<br>litre_of_solution — litre of solution (e.g. 1 litre of 5% solution)<br>cubic_meter — cubic meter (m^3)<br>other — other<br>dont_know — don't know |
| q204_M113c.1 | q204_M113c.1 If other unit of measurement or route of administration, specify here:<br>*Leave blank if not applicable*<br>*Question relevant when: selected( ${q104_M113_full_list} , '1')* | |
| q204_M113d *(required)* | q204_M113d. In total, how many units are used to treat a patient during all of her care?<br>*Remember to add all units! E.g. 5 days x 200 mg twice per day = 2,000mg*<br>*Question relevant when: selected( ${q104_M113_full_list} , '1')*<br>*Response constrained to: .>0* | |
| q204_M114 | ***q204_M114. Vitamin, immunological, etc. - Other 4: "[q104_M114_full_list_other]"***<br>*Question relevant when: selected( ${q104_M114_full_list} , '1')* | |
| q204_M114a *(required)* | q204_M114a. What percent of patients require this item?<br>*Question relevant when: selected( ${q104_M114_full_list} , '1')*<br>*Response constrained to: .>0 and .<=100 or .=999* | |
| q204_M114b *(required)* | q204_M114b. How is the item administered, or given, to the patient?<br>*Question relevant when: selected( ${q104_M114_full_list} , '1')* | 1 Oral - solid (e.g. tablet)<br>2 Oral - liquid<br>3 Sublingual<br>4 Intravenous (IV)<br>5 Intramusculuar injection (IM)<br>6 Subcutaneous<br>7 Rectal<br>8 Inhaled (e.g. gas)<br>9 Inserted (e.g. vaginal pessaries)<br>10 Topical - cream<br>11 Topical - drops (e.g. eye drops)<br>12 Other<br>99 Don't know |

| Field | Question | Answer |
|---|---|---|
| q204_M114c *(required)* | q204_M114c. What is the item's smallest unit of measurement (mg, mcg, ml, g, etc)?<br>*Question relevant when: selected( ${q104_M114_full_list} , '1')* | microgram — microgram (mcg)<br>miligram — miligram (mg)<br>miligram_dissolved_in_liquid — miligram (mg) dissolved in liquid (e.g. 2 mg per 3 militers)<br>gram — gram (g)<br>militer — militer (ml)<br>centiliter — centiliter (cl)<br>litre — litre (l)<br>ml_of _solution — ml of solution (e.g. ml of 10% solution)<br>litre_of_solution — litre of solution (e.g. 1 litre of 5% solution)<br>cubic_meter — cubic meter (m^3)<br>other — other<br>dont_know — don't know |
| q204_M114c.1 | q204_M114c.1 If other unit of measurement or route of administration, specify here:<br>*Leave blank if not applicable*<br>*Question relevant when: selected( ${q104_M114_full_list} , '1')* | |
| q204_M114d *(required)* | q204_M114d. In total, how many units are used to treat a patient during all of her care?<br>*Remember to add all units! E.g. 5 days x 200 mg twice per day = 2,000mg*<br>*Question relevant when: selected( ${q104_M114_full_list} , '1')*<br>*Response constrained to: .>0* | |
| q204_M115 | ***q204_M115. Vitamin, immunological, etc. - Other 5: "[q104_M115_full_list_other]"***<br>*Question relevant when: selected( ${q104_M115_full_list} , '1')* | |
| q204_M115a *(required)* | q204_M115a. What percent of patients require this item?<br>*Question relevant when: selected( ${q104_M115_full_list} , '1')*<br>*Response constrained to: .>0 and .<=100 or .=999* | |
| q204_M115b *(required)* | q204_M115b. How is the item administered, or given, to the patient?<br>*Question relevant when: selected( ${q104_M115_full_list} , '1')* | 1 Oral - solid (e.g. tablet)<br>2 Oral - liquid<br>3 Sublingual<br>4 Intravenous (IV)<br>5 Intramusculuar injection (IM)<br>6 Subcutaneous<br>7 Rectal<br>8 Inhaled (e.g. gas)<br>9 Inserted (e.g. vaginal pessaries)<br>10 Topical - cream<br>11 Topical - drops (e.g. eye drops)<br>12 Other<br>99 Don't know |

| Field | Question | Answer |
|---|---|---|
| q204_M115c *(required)* | q204_M115c. What is the item's smallest unit of measurement (mg, mcg, ml, g, etc)?<br>*Question relevant when: selected( ${q104_M115_full_list} , '1')* | microgram — microgram (mcg)<br>miligram — miligram (mg)<br>miligram_dissolved_in_liquid — miligram (mg) dissolved in liquid (e.g. 2 mg per 3 militers)<br>gram — gram (g)<br>militer — militer (ml)<br>centiliter — centiliter (cl)<br>litre — litre (l)<br>ml_of _solution — ml of solution (e.g. ml of 10% solution)<br>litre_of_solution — litre of solution (e.g. 1 litre of 5% solution)<br>cubic_meter — cubic meter (m^3)<br>other — other<br>dont_know — don't know |
| q204_M115c.1 | q204_M115c.1 If other unit of measurement or route of administration, specify here:<br>*Leave blank if not applicable*<br>*Question relevant when: selected( ${q104_M115_full_list} , '1')* | |
| q204_M115d *(required)* | q204_M115d. In total, how many units are used to treat a patient during all of her care?<br>*Remember to add all units! E.g. 5 days x 200 mg twice per day = 2,000mg*<br>*Question relevant when: selected( ${q104_M115_full_list} , '1')*<br>*Response constrained to: .>0* | |
| <p><strong><em>D. Medications (12)</em></strong></p><br>*Group relevant when: selected( ${section_two_skip_med} , '1')* | | |
| q204_med_notE11 | 204 CONTINUED For each of the following medications and other medical products that are used for incomplete abortion, can you tell me …? | |
| note_204_Other | ***Other medication, any class*** | |
| q204_M116 | ***q204_M116. Medication - any class - Other 1: "[q104_M116_full_list_other]"***<br>*Question relevant when: selected( ${q104_M116_full_list} , '1')* | |
| q204_M116a *(required)* | q204_M116a. What percent of patients require this item?<br>*Question relevant when: selected( ${q104_M116_full_list} , '1')*<br>*Response constrained to: .>0 and .<=100 or .=999* | |
| q204_M116b *(required)* | q204_M116b. How is the item administered, or given, to the patient?<br>*Question relevant when: selected( ${q104_M116_full_list} , '1')* | 1 Oral - solid (e.g. tablet)<br>2 Oral - liquid<br>3 Sublingual<br>4 Intravenous (IV)<br>5 Intramusculuar injection (IM)<br>6 Subcutaneous<br>7 Rectal<br>8 Inhaled (e.g. gas)<br>9 Inserted (e.g. vaginal pessaries)<br>10 Topical - cream<br>11 Topical - drops (e.g. eye drops) |

| Field | Question | Answer |
|---|---|---|
| | | 12 Other<br>99 Don't know |

| Field | Question | Answer |
|---|---|---|
| q204_M116c *(required)* | q204_M116c. What is the item's smallest unit of measurement (mg, mcg, ml, g, etc)?<br>*Question relevant when: selected( ${q104_M116_full_list} , '1')* | microgram — microgram (mcg)<br>miligram — miligram (mg)<br>miligram_dissolved_in_liquid — miligram (mg) dissolved in liquid (e.g. 2 mg per 3 militers)<br>gram — gram (g)<br>militer — militer (ml)<br>centiliter — centiliter (cl)<br>litre — litre (l)<br>ml_of _solution — ml of solution (e.g. ml of 10% solution)<br>litre_of_solution — litre of solution (e.g. 1 litre of 5% solution)<br>cubic_meter — cubic meter (m^3)<br>other — other<br>dont_know — don't know |
| q204_M116c.1 | q204_M116c.1 If other unit of measurement or route of administration, specify here:<br>*Leave blank if not applicable*<br>*Question relevant when: selected( ${q104_M116_full_list} , '1')* | |
| q204_M116d *(required)* | q204_M116d. In total, how many units are used to treat a patient during all of her care?<br>*Remember to add all units! E.g. 5 days x 200 mg twice per day = 2,000mg*<br>*Question relevant when: selected( ${q104_M116_full_list} , '1')*<br>*Response constrained to: .>0* | |
| q204_M117 | ***q204_M117. Medication - any class - Other 2: "[q104_M117_full_list_other]"***<br>*Question relevant when: selected( ${q104_M117_full_list} , '1')* | |
| q204_M117a *(required)* | q204_M117a. What percent of patients require this item?<br>*Question relevant when: selected( ${q104_M117_full_list} , '1')*<br>*Response constrained to: .>0 and .<=100 or .=999* | |
| q204_M117b *(required)* | q204_M117b. How is the item administered, or given, to the patient?<br>*Question relevant when: selected( ${q104_M117_full_list} , '1')* | 1 Oral - solid (e.g. tablet)<br>2 Oral - liquid<br>3 Sublingual<br>4 Intravenous (IV)<br>5 Intramusculuar injection (IM)<br>6 Subcutaneous<br>7 Rectal<br>8 Inhaled (e.g. gas)<br>9 Inserted (e.g. vaginal pessaries)<br>10 Topical - cream<br>11 Topical - drops (e.g. eye drops)<br>12 Other<br>99 Don't know |

| Field | Question | Answer |
|---|---|---|
| q204_M117c *(required)* | q204_M117c. What is the item's smallest unit of measurement (mg, mcg, ml, g, etc)?<br>*Question relevant when: selected( ${q104_M117_full_list} , '1')* | microgram — microgram (mcg)<br>miligram — miligram (mg)<br>miligram_dissolved_in_liquid — miligram (mg) dissolved in liquid (e.g. 2 mg per 3 militers)<br>gram — gram (g)<br>militer — militer (ml)<br>centiliter — centiliter (cl)<br>litre — litre (l)<br>ml_of _solution — ml of solution (e.g. ml of 10% solution)<br>litre_of_solution — litre of solution (e.g. 1 litre of 5% solution)<br>cubic_meter — cubic meter (m^3)<br>other — other<br>dont_know — don't know |
| q204_M117c.1 | q204_M117c.1 If other unit of measurement or route of administration, specify here:<br>*Leave blank if not applicable*<br>*Question relevant when: selected( ${q104_M117_full_list} , '1')* | |
| q204_M117d *(required)* | q204_M117d. In total, how many units are used to treat a patient during all of her care?<br>*Remember to add all units! E.g. 5 days x 200 mg twice per day = 2,000mg*<br>*Question relevant when: selected( ${q104_M117_full_list} , '1')*<br>*Response constrained to: .>0* | |
| q204_M118 | ***q204_M118. Medication - any class - Other 3: "[q104_M118_full_list_other]"***<br>*Question relevant when: selected( ${q104_M118_full_list} , '1')* | |
| q204_M118a *(required)* | q204_M118a. What percent of patients require this item?<br>*Question relevant when: selected( ${q104_M118_full_list} , '1')*<br>*Response constrained to: .>0 and .<=100 or .=999* | |
| q204_M118b *(required)* | q204_M118b. How is the item administered, or given, to the patient?<br>*Question relevant when: selected( ${q104_M118_full_list} , '1')* | 1 Oral - solid (e.g. tablet)<br>2 Oral - liquid<br>3 Sublingual<br>4 Intravenous (IV)<br>5 Intramusculuar injection (IM)<br>6 Subcutaneous<br>7 Rectal<br>8 Inhaled (e.g. gas)<br>9 Inserted (e.g. vaginal pessaries)<br>10 Topical - cream<br>11 Topical - drops (e.g. eye drops)<br>12 Other<br>99 Don't know |

| Field | Question | Answer |
|---|---|---|
| q204_M118c *(required)* | q204_M118c. What is the item's smallest unit of measurement (mg, mcg, ml, g, etc)?<br>*Question relevant when: selected( ${q104_M118_full_list} , '1')* | microgram — microgram (mcg)<br>miligram — miligram (mg)<br>miligram_dissolved_in_liquid — miligram (mg) dissolved in liquid (e.g. 2 mg per 3 militers)<br>gram — gram (g)<br>militer — militer (ml)<br>centiliter — centiliter (cl)<br>litre — litre (l)<br>ml_of _solution — ml of solution (e.g. ml of 10% solution)<br>litre_of_solution — litre of solution (e.g. 1 litre of 5% solution)<br>cubic_meter — cubic meter (m^3)<br>other — other<br>dont_know — don't know |
| q204_M118c.1 | q204_M118c.1 If other unit of measurement or route of administration, specify here:<br>*Leave blank if not applicable*<br>*Question relevant when: selected( ${q104_M118_full_list} , '1')* | |
| q204_M118d *(required)* | q204_M118d. In total, how many units are used to treat a patient during all of her care?<br>*Remember to add all units! E.g. 5 days x 200 mg twice per day = 2,000mg*<br>*Question relevant when: selected( ${q104_M118_full_list} , '1')*<br>*Response constrained to: .>0* | |
| q204_M119 | ***q204_M119. Medication - any class - Other 4: "[q104_M119_full_list_other]"***<br>*Question relevant when: selected( ${q104_M119_full_list} , '1')* | |
| q204_M119a *(required)* | q204_M119a. What percent of patients require this item?<br>*Question relevant when: selected( ${q104_M119_full_list} , '1')*<br>*Response constrained to: .>0 and .<=100 or .=999* | |
| q204_M119b *(required)* | q204_M119b. How is the item administered, or given, to the patient?<br>*Question relevant when: selected( ${q104_M119_full_list} , '1')* | 1 Oral - solid (e.g. tablet)<br>2 Oral - liquid<br>3 Sublingual<br>4 Intravenous (IV)<br>5 Intramusculuar injection (IM)<br>6 Subcutaneous<br>7 Rectal<br>8 Inhaled (e.g. gas)<br>9 Inserted (e.g. vaginal pessaries)<br>10 Topical - cream<br>11 Topical - drops (e.g. eye drops)<br>12 Other<br>99 Don't know |

| Field | Question | Answer |
|---|---|---|
| q204_M119c *(required)* | q204_M119c. What is the item's smallest unit of measurement (mg, mcg, ml, g, etc)?<br>*Question relevant when: selected( ${q104_M119_full_list} , '1')* | microgram: microgram (mcg)<br>miligram: miligram (mg)<br>miligram_dissolved_in_liquid: miligram (mg) dissolved in liquid (e.g. 2 mg per 3 militers)<br>gram: gram (g)<br>militer: militer (ml)<br>centiliter: centiliter (cl)<br>litre: litre (l)<br>ml_of _solution: ml of solution (e.g. ml of 10% solution)<br>litre_of_solution: litre of solution (e.g. 1 litre of 5% solution)<br>cubic_meter: cubic meter (m^3)<br>other: other<br>dont_know: don't know |
| q204_M119c.1 | q204_M119c.1 If other unit of measurement or route of administration, specify here:<br>*Leave blank if not applicable*<br>*Question relevant when: selected( ${q104_M119_full_list} , '1')* | |
| q204_M119d *(required)* | q204_M119d. In total, how many units are used to treat a patient during all of her care?<br>*Remember to add all units! E.g. 5 days x 200 mg twice per day = 2,000mg*<br>*Question relevant when: selected( ${q104_M119_full_list} , '1')*<br>*Response constrained to: .>0* | |
| q204_M120 | ***q204_M120. Medication - any class - Other 5: "[q104_M120_full_list_other]"***<br>*Question relevant when: selected( ${q104_M120_full_list} , '1')* | |
| q204_M120a *(required)* | q204_M120a. What percent of patients require this item?<br>*Question relevant when: selected( ${q104_M120_full_list} , '1')*<br>*Response constrained to: .>0 and .<=100 or .=999* | |
| q204_M120b *(required)* | q204_M120b. How is the item administered, or given, to the patient?<br>*Question relevant when: selected( ${q104_M120_full_list} , '1')* | 1 Oral - solid (e.g. tablet)<br>2 Oral - liquid<br>3 Sublingual<br>4 Intravenous (IV)<br>5 Intramusculuar injection (IM)<br>6 Subcutaneous<br>7 Rectal<br>8 Inhaled (e.g. gas)<br>9 Inserted (e.g. vaginal pessaries)<br>10 Topical - cream<br>11 Topical - drops (e.g. eye drops)<br>12 Other<br>99 Don't know |

| Field | Question | Answer |
|---|---|---|
| q204_M120c *(required)* | q204_M120c. What is the item's smallest unit of measurement (mg, mcg, ml, g, etc)?<br>*Question relevant when: selected( ${q104_M120_full_list} , '1')* | microgram — microgram (mcg)<br>miligram — miligram (mg)<br>miligram_dissolved_in_liquid — miligram (mg) dissolved in liquid (e.g. 2 mg per 3 militers)<br>gram — gram (g)<br>militer — militer (ml)<br>centiliter — centiliter (cl)<br>litre — litre (l)<br>ml_of _solution — ml of solution (e.g. ml of 10% solution)<br>litre_of_solution — litre of solution (e.g. 1 litre of 5% solution)<br>cubic_meter — cubic meter (m^3)<br>other — other<br>dont_know — don't know |
| q204_M120c.1 | q204_M120c.1 If other unit of measurement or route of administration, specify here:<br>*Leave blank if not applicable*<br>*Question relevant when: selected( ${q104_M120_full_list} , '1')* | |
| q204_M120d *(required)* | q204_M120d. In total, how many units are used to treat a patient during all of her care?<br>*Remember to add all units! E.g. 5 days x 200 mg twice per day = 2,000mg*<br>*Question relevant when: selected( ${q104_M120_full_list} , '1')*<br>*Response constrained to: .>0* | |
| group_section_three_intro | | |
| section3_start | **SECTION III. SEPSIS - USAGE OF ALL ITEMS** | |
| section_three_skip_med | INTERVIEWER: WOULD YOU LIKE TO COMPLETE THIS SECTION NOW OR SKIP THIS SECTION AND RETURN TO IT LATER?<br>*You may need to skip if the participant has indicated that s/he cannot answer the questions in this section.* | 1 Do not skip, complete this section now.<br>2 Skip and come back to this section later. |
| group_section_three_introB<br>*Group relevant when: selected( ${section_three_skip_med} , '1')* | | |
| section3_start2 | In this section of the interview, we will review all of the items that you said are used for management of sepsis. For each item that is used, I'm going to ask questions on how many women need it and how much of it is used. | |
| section3_start3 | INTERVIEWER: ENTER WHOLE NUMBERS OR DECIMALS. DO NOT TYPE PERCENT SIGNS. ENTER 999 FOR ANY THAT ARE UNKNOWN. | |
| <p><strong><em>D. Medications - Sepsis (1)</em></strong></p><br>*Group relevant when: selected( ${section_three_skip_med} , '1')* | | |
| q304_med_note1 | 304 For each of the following medications and other medical products that are used for sepsis, can you tell me …? | |
| note_304_analgesic | ***Analgesic, anti-inflammatory, narcotic, etc.*** | |
| q304_M1 | ***q304_M1. Acetylsalicylic acid (aspirin)***<br>*Question relevant when: selected( ${q104_M1_full_list} , '2')* | |
| q304_M1a *(required)* | q304_M1a. What percent of patients require this item?<br>*Question relevant when: selected( ${q104_M1_full_list} , '2')*<br>*Response constrained to: .>0 and .<=100 or .=999* | |

| Field | Question | Answer |
|---|---|---|
| q304_M1b *(required)* | q304_M1b. How is the item administered, or given, to the patient?<br>*Question relevant when: selected( ${q104_M1_full_list} , '2')* | 1 Oral - solid (e.g. tablet)<br>2 Oral - liquid<br>3 Sublingual<br>4 Intravenous (IV)<br>5 Intramusculuar injection (IM)<br>6 Subcutaneous<br>7 Rectal<br>8 Inhaled (e.g. gas)<br>9 Inserted (e.g. vaginal pessaries)<br>10 Topical - cream<br>11 Topical - drops (e.g. eye drops)<br>12 Other<br>99 Don't know |
| q304_M1c *(required)* | q304_M1c. What is the item's smallest unit of measurement (mg, mcg, ml, g, etc)?<br>*Question relevant when: selected( ${q104_M1_full_list} , '2')* | microgram — microgram (mcg)<br>miligram — miligram (mg)<br>miligram_dissolved_in_liquid — miligram (mg) dissolved in liquid (e.g. 2 mg per 3 militers)<br>gram — gram (g)<br>militer — militer (ml)<br>centiliter — centiliter (cl)<br>litre — litre (l)<br>ml_of _solution — ml of solution (e.g. ml of 10% solution)<br>litre_of_solution — litre of solution (e.g. 1 litre of 5% solution)<br>cubic_meter — cubic meter (m^3)<br>other — other<br>dont_know — don't know |
| q304_M1c.1 | q304_M1c.1 If other unit of measurement or route of administration, specify here:<br>*Leave blank if not applicable*<br>*Question relevant when: selected( ${q104_M1_full_list} , '2')* | |
| q304_M1d *(required)* | q304_M1d. In total, how many units are used to treat a patient during all of her care?<br>*Remember to add all units! E.g. 5 days x 200 mg twice per day = 2,000mg*<br>*Question relevant when: selected( ${q104_M1_full_list} , '2')*<br>*Response constrained to: .>0* | |
| q304_M2 | ***q304_M2. Paracetamol***<br>*Question relevant when: selected( ${q104_M2_full_list} , '2')* | |
| q304_M2a *(required)* | q304_M2a. What percent of patients require this item?<br>*Question relevant when: selected( ${q104_M2_full_list} , '2')*<br>*Response constrained to: .>0 and .<=100 or .=999* | |

| Field | Question | Answer |
|---|---|---|
| q304_M2b *(required)* | q304_M2b. How is the item administered, or given, to the patient?<br>*Question relevant when: selected( ${q104_M2_full_list} , '2')* | 1 Oral - solid (e.g. tablet)<br>2 Oral - liquid<br>3 Sublingual<br>4 Intravenous (IV)<br>5 Intramusculuar injection (IM)<br>6 Subcutaneous<br>7 Rectal<br>8 Inhaled (e.g. gas)<br>9 Inserted (e.g. vaginal pessaries)<br>10 Topical - cream<br>11 Topical - drops (e.g. eye drops)<br>12 Other<br>99 Don't know |
| q304_M2c *(required)* | q304_M2c. What is the item's smallest unit of measurement (mg, mcg, ml, g, etc)?<br>*Question relevant when: selected( ${q104_M2_full_list} , '2')* | microgram: microgram (mcg)<br>miligram: miligram (mg)<br>miligram_dissolved_in_liquid: miligram (mg) dissolved in liquid (e.g. 2 mg per 3 militers)<br>gram: gram (g)<br>militer: militer (ml)<br>centiliter: centiliter (cl)<br>litre: litre (l)<br>ml_of _solution: ml of solution (e.g. ml of 10% solution)<br>litre_of_solution: litre of solution (e.g. 1 litre of 5% solution)<br>cubic_meter: cubic meter (m^3)<br>other: other<br>dont_know: don't know |
| q304_M2c.1 | q304_M2c.1 If other unit of measurement or route of administration, specify here:<br>*Leave blank if not applicable*<br>*Question relevant when: selected( ${q104_M2_full_list} , '2')* | |
| q304_M2d *(required)* | q304_M2d. In total, how many units are used to treat a patient during all of her care?<br>*Remember to add all units! E.g. 5 days x 200 mg twice per day = 2,000mg*<br>*Question relevant when: selected( ${q104_M2_full_list} , '2')*<br>*Response constrained to: .>0* | |
| q304_M3 | ***q304_M3. Pethidine hydrochloride***<br>*Question relevant when: selected( ${q104_M3_full_list} , '2')* | |
| q304_M3a *(required)* | q304_M3a. What percent of patients require this item?<br>*Question relevant when: selected( ${q104_M3_full_list} , '2')*<br>*Response constrained to: .>0 and .<=100 or .=999* | |

| Field | Question | Answer |
|---|---|---|
| q304_M3b *(required)* | q304_M3b. How is the item administered, or given, to the patient?<br>*Question relevant when: selected( ${q104_M3_full_list} , '2')* | 1 Oral - solid (e.g. tablet)<br>2 Oral - liquid<br>3 Sublingual<br>4 Intravenous (IV)<br>5 Intramusculuar injection (IM)<br>6 Subcutaneous<br>7 Rectal<br>8 Inhaled (e.g. gas)<br>9 Inserted (e.g. vaginal pessaries)<br>10 Topical - cream<br>11 Topical - drops (e.g. eye drops)<br>12 Other<br>99 Don't know |
| q304_M3c *(required)* | q304_M3c. What is the item's smallest unit of measurement (mg, mcg, ml, g, etc)?<br>*Question relevant when: selected( ${q104_M3_full_list} , '2')* | microgram — microgram (mcg)<br>miligram — miligram (mg)<br>miligram_dissolved_in_liquid — miligram (mg) dissolved in liquid (e.g. 2 mg per 3 militers)<br>gram — gram (g)<br>militer — militer (ml)<br>centiliter — centiliter (cl)<br>litre — litre (l)<br>ml_of _solution — ml of solution (e.g. ml of 10% solution)<br>litre_of_solution — litre of solution (e.g. 1 litre of 5% solution)<br>cubic_meter — cubic meter (m^3)<br>other — other<br>dont_know — don't know |
| q304_M3c.1 | q304_M3c.1 If other unit of measurement or route of administration, specify here:<br>*Leave blank if not applicable*<br>*Question relevant when: selected( ${q104_M3_full_list} , '2')* | |
| q304_M3d *(required)* | q304_M3d. In total, how many units are used to treat a patient during all of her care?<br>*Remember to add all units! E.g. 5 days x 200 mg twice per day = 2,000mg*<br>*Question relevant when: selected( ${q104_M3_full_list} , '2')*<br>*Response constrained to: .>0* | |
| q304_M4 | ***q304_M4. Tramadol Hydrochloride***<br>*Question relevant when: selected( ${q104_M4_full_list} , '2')* | |
| q304_M4a *(required)* | q304_M4a. What percent of patients require this item?<br>*Question relevant when: selected( ${q104_M4_full_list} , '2')*<br>*Response constrained to: .>0 and .<=100 or .=999* | |

| Field | Question | Answer |
|---|---|---|
| q304_M4b *(required)* | q304_M4b. How is the item administered, or given, to the patient?<br>*Question relevant when: selected( ${q104_M4_full_list} , '2')* | 1 Oral - solid (e.g. tablet)<br>2 Oral - liquid<br>3 Sublingual<br>4 Intravenous (IV)<br>5 Intramusculuar injection (IM)<br>6 Subcutaneous<br>7 Rectal<br>8 Inhaled (e.g. gas)<br>9 Inserted (e.g. vaginal pessaries)<br>10 Topical - cream<br>11 Topical - drops (e.g. eye drops)<br>12 Other<br>99 Don't know |
| q304_M4c *(required)* | q304_M4c. What is the item's smallest unit of measurement (mg, mcg, ml, g, etc)?<br>*Question relevant when: selected( ${q104_M4_full_list} , '2')* | microgram: microgram (mcg)<br>miligram: miligram (mg)<br>miligram_dissolved_in_liquid: miligram (mg) dissolved in liquid (e.g. 2 mg per 3 militers)<br>gram: gram (g)<br>militer: militer (ml)<br>centiliter: centiliter (cl)<br>litre: litre (l)<br>ml_of _solution: ml of solution (e.g. ml of 10% solution)<br>litre_of_solution: litre of solution (e.g. 1 litre of 5% solution)<br>cubic_meter: cubic meter (m^3)<br>other: other<br>dont_know: don't know |
| q304_M4c.1 | q304_M4c.1 If other unit of measurement or route of administration, specify here:<br>*Leave blank if not applicable*<br>*Question relevant when: selected( ${q104_M4_full_list} , '2')* | |
| q304_M4d *(required)* | q304_M4d. In total, how many units are used to treat a patient during all of her care?<br>*Remember to add all units! E.g. 5 days x 200 mg twice per day = 2,000mg*<br>*Question relevant when: selected( ${q104_M4_full_list} , '2')*<br>*Response constrained to: .>0* | |
| q304_M5 | ***q304_M5. Ibuprofen***<br>*Question relevant when: selected( ${q104_M5_full_list} , '2')* | |
| q304_M5a *(required)* | q304_M5a. What percent of patients require this item?<br>*Question relevant when: selected( ${q104_M5_full_list} , '2')*<br>*Response constrained to: .>0 and .<=100 or .=999* | |

| Field | Question | Answer |
|---|---|---|
| q304_M5b *(required)* | q304_M5b. How is the item administered, or given, to the patient?<br>*Question relevant when: selected( ${q104_M5_full_list} , '2')* | 1 Oral - solid (e.g. tablet)<br>2 Oral - liquid<br>3 Sublingual<br>4 Intravenous (IV)<br>5 Intramusculuar injection (IM)<br>6 Subcutaneous<br>7 Rectal<br>8 Inhaled (e.g. gas)<br>9 Inserted (e.g. vaginal pessaries)<br>10 Topical - cream<br>11 Topical - drops (e.g. eye drops)<br>12 Other<br>99 Don't know |
| q304_M5c *(required)* | q304_M5c. What is the item's smallest unit of measurement (mg, mcg, ml, g, etc)?<br>*Question relevant when: selected( ${q104_M5_full_list} , '2')* | microgram — microgram (mcg)<br>miligram — miligram (mg)<br>miligram_dissolved_in_liquid — miligram (mg) dissolved in liquid (e.g. 2 mg per 3 militers)<br>gram — gram (g)<br>militer — militer (ml)<br>centiliter — centiliter (cl)<br>litre — litre (l)<br>ml_of _solution — ml of solution (e.g. ml of 10% solution)<br>litre_of_solution — litre of solution (e.g. 1 litre of 5% solution)<br>cubic_meter — cubic meter (m^3)<br>other — other<br>dont_know — don't know |
| q304_M5c.1 | q304_M5c.1 If other unit of measurement or route of administration, specify here:<br>*Leave blank if not applicable*<br>*Question relevant when: selected( ${q104_M5_full_list} , '2')* | |
| q304_M5d *(required)* | q304_M5d. In total, how many units are used to treat a patient during all of her care?<br>*Remember to add all units! E.g. 5 days x 200 mg twice per day = 2,000mg*<br>*Question relevant when: selected( ${q104_M5_full_list} , '2')*<br>*Response constrained to: .>0* | |
| q304_M6 | ***q304_M6. Diclofenac***<br>*Question relevant when: selected( ${q104_M6_full_list} , '2')* | |
| q304_M6a *(required)* | q304_M6a. What percent of patients require this item?<br>*Question relevant when: selected( ${q104_M6_full_list} , '2')*<br>*Response constrained to: .>0 and .<=100 or .=999* | |

| Field | Question | Answer |
|---|---|---|
| q304_M6b *(required)* | q304_M6b. How is the item administered, or given, to the patient?<br>*Question relevant when: selected( ${q104_M6_full_list} , '2')* | 1 Oral - solid (e.g. tablet)<br>2 Oral - liquid<br>3 Sublingual<br>4 Intravenous (IV)<br>5 Intramusculuar injection (IM)<br>6 Subcutaneous<br>7 Rectal<br>8 Inhaled (e.g. gas)<br>9 Inserted (e.g. vaginal pessaries)<br>10 Topical - cream<br>11 Topical - drops (e.g. eye drops)<br>12 Other<br>99 Don't know |
| q304_M6c *(required)* | q304_M6c. What is the item's smallest unit of measurement (mg, mcg, ml, g, etc)?<br>*Question relevant when: selected( ${q104_M6_full_list} , '2')* | microgram — microgram (mcg)<br>miligram — miligram (mg)<br>miligram_dissolved_in_liquid — miligram (mg) dissolved in liquid (e.g. 2 mg per 3 militers)<br>gram — gram (g)<br>militer — militer (ml)<br>centiliter — centiliter (cl)<br>litre — litre (l)<br>ml_of _solution — ml of solution (e.g. ml of 10% solution)<br>litre_of_solution — litre of solution (e.g. 1 litre of 5% solution)<br>cubic_meter — cubic meter (m^3)<br>other — other<br>dont_know — don't know |
| q304_M6c.1 | q304_M6c.1 If other unit of measurement or route of administration, specify here:<br>*Leave blank if not applicable*<br>*Question relevant when: selected( ${q104_M6_full_list} , '2')* | |
| q304_M6d *(required)* | q304_M6d. In total, how many units are used to treat a patient during all of her care?<br>*Remember to add all units! E.g. 5 days x 200 mg twice per day = 2,000mg*<br>*Question relevant when: selected( ${q104_M6_full_list} , '2')*<br>*Response constrained to: .>0* | |
| q304_M7 | ***q304_M7. Diclofenac sodium***<br>*Question relevant when: selected( ${q104_M7_full_list} , '2')* | |
| q304_M7a *(required)* | q304_M7a. What percent of patients require this item?<br>*Question relevant when: selected( ${q104_M7_full_list} , '2')*<br>*Response constrained to: .>0 and .<=100 or .=999* | |

| Field | Question | Answer |
|---|---|---|
| q304_M7b *(required)* | q304_M7b. How is the item administered, or given, to the patient?<br>*Question relevant when: selected( ${q104_M7_full_list} , '2')* | 1 Oral - solid (e.g. tablet)<br>2 Oral - liquid<br>3 Sublingual<br>4 Intravenous (IV)<br>5 Intramusculuar injection (IM)<br>6 Subcutaneous<br>7 Rectal<br>8 Inhaled (e.g. gas)<br>9 Inserted (e.g. vaginal pessaries)<br>10 Topical - cream<br>11 Topical - drops (e.g. eye drops)<br>12 Other<br>99 Don't know |
| q304_M7c *(required)* | q304_M7c. What is the item's smallest unit of measurement (mg, mcg, ml, g, etc)?<br>*Question relevant when: selected( ${q104_M7_full_list} , '2')* | microgram — microgram (mcg)<br>miligram — miligram (mg)<br>miligram_dissolved_in_liquid — miligram (mg) dissolved in liquid (e.g. 2 mg per 3 militers)<br>gram — gram (g)<br>militer — militer (ml)<br>centiliter — centiliter (cl)<br>litre — litre (l)<br>ml_of _solution — ml of solution (e.g. ml of 10% solution)<br>litre_of_solution — litre of solution (e.g. 1 litre of 5% solution)<br>cubic_meter — cubic meter (m^3)<br>other — other<br>dont_know — don't know |
| q304_M7c.1 | q304_M7c.1 If other unit of measurement or route of administration, specify here:<br>*Leave blank if not applicable*<br>*Question relevant when: selected( ${q104_M7_full_list} , '2')* | |
| q304_M7d *(required)* | q304_M7d. In total, how many units are used to treat a patient during all of her care?<br>*Remember to add all units! E.g. 5 days x 200 mg twice per day = 2,000mg*<br>*Question relevant when: selected( ${q104_M7_full_list} , '2')*<br>*Response constrained to: .>0* | |
| q304_M8 | ***q304_M8. Dypron (dipyrone), 250mg/ml (Metamizole)***<br>*Question relevant when: selected( ${q104_M8_full_list} , '2')* | |
| q304_M8a *(required)* | q304_M8a. What percent of patients require this item?<br>*Question relevant when: selected( ${q104_M8_full_list} , '2')*<br>*Response constrained to: .>0 and .<=100 or .=999* | |

| Field | Question | Answer |
|---|---|---|
| q304_M8b *(required)* | q304_M8b. How is the item administered, or given, to the patient?<br>*Question relevant when: selected( ${q104_M8_full_list} , '2')* | 1 Oral - solid (e.g. tablet)<br>2 Oral - liquid<br>3 Sublingual<br>4 Intravenous (IV)<br>5 Intramusculuar injection (IM)<br>6 Subcutaneous<br>7 Rectal<br>8 Inhaled (e.g. gas)<br>9 Inserted (e.g. vaginal pessaries)<br>10 Topical - cream<br>11 Topical - drops (e.g. eye drops)<br>12 Other<br>99 Don't know |
| q304_M8c *(required)* | q304_M8c. What is the item's smallest unit of measurement (mg, mcg, ml, g, etc)?<br>*Question relevant when: selected( ${q104_M8_full_list} , '2')* | microgram — microgram (mcg)<br>miligram — miligram (mg)<br>miligram_dissolved_in_liquid — miligram (mg) dissolved in liquid (e.g. 2 mg per 3 militers)<br>gram — gram (g)<br>militer — militer (ml)<br>centiliter — centiliter (cl)<br>litre — litre (l)<br>ml_of _solution — ml of solution (e.g. ml of 10% solution)<br>litre_of_solution — litre of solution (e.g. 1 litre of 5% solution)<br>cubic_meter — cubic meter (m^3)<br>other — other<br>dont_know — don't know |
| q304_M8c.1 | q304_M8c.1 If other unit of measurement or route of administration, specify here:<br>*Leave blank if not applicable*<br>*Question relevant when: selected( ${q104_M8_full_list} , '2')* | |
| q304_M8d *(required)* | q304_M8d. In total, how many units are used to treat a patient during all of her care?<br>*Remember to add all units! E.g. 5 days x 200 mg twice per day = 2,000mg*<br>*Question relevant when: selected( ${q104_M8_full_list} , '2')*<br>*Response constrained to: .>0* | |
| q304_M9 | ***q304_M9. Fentanyl citrate, 50 mcg/ml***<br>*Question relevant when: selected( ${q104_M9_full_list} , '2')* | |
| q304_M9a *(required)* | q304_M9a. What percent of patients require this item?<br>*Question relevant when: selected( ${q104_M9_full_list} , '2')*<br>*Response constrained to: .>0 and .<=100 or .=999* | |

| Field | Question | Answer |
|---|---|---|
| q304_M9b *(required)* | q304_M9b. How is the item administered, or given, to the patient?<br>*Question relevant when: selected( ${q104_M9_full_list} , '2')* | 1 Oral - solid (e.g. tablet)<br>2 Oral - liquid<br>3 Sublingual<br>4 Intravenous (IV)<br>5 Intramusculuar injection (IM)<br>6 Subcutaneous<br>7 Rectal<br>8 Inhaled (e.g. gas)<br>9 Inserted (e.g. vaginal pessaries)<br>10 Topical - cream<br>11 Topical - drops (e.g. eye drops)<br>12 Other<br>99 Don't know |
| q304_M9c *(required)* | q304_M9c. What is the item's smallest unit of measurement (mg, mcg, ml, g, etc)?<br>*Question relevant when: selected( ${q104_M9_full_list} , '2')* | microgram — microgram (mcg)<br>miligram — miligram (mg)<br>miligram_dissolved_in_liquid — miligram (mg) dissolved in liquid (e.g. 2 mg per 3 militers)<br>gram — gram (g)<br>militer — militer (ml)<br>centiliter — centiliter (cl)<br>litre — litre (l)<br>ml_of _solution — ml of solution (e.g. ml of 10% solution)<br>litre_of_solution — litre of solution (e.g. 1 litre of 5% solution)<br>cubic_meter — cubic meter (m^3)<br>other — other<br>dont_know — don't know |
| q304_M9c.1 | q304_M9c.1 If other unit of measurement or route of administration, specify here:<br>*Leave blank if not applicable*<br>*Question relevant when: selected( ${q104_M9_full_list} , '2')* | |
| q304_M9d *(required)* | q304_M9d. In total, how many units are used to treat a patient during all of her care?<br>*Remember to add all units! E.g. 5 days x 200 mg twice per day = 2,000mg*<br>*Question relevant when: selected( ${q104_M9_full_list} , '2')*<br>*Response constrained to: .>0* | |
| q304_M10 | ***q304_M10. Analgesic - other 1: "[q104_M10_full_list_other]"***<br>*Question relevant when: selected( ${q104_M10_full_list} , '2')* | |
| q304_M10a *(required)* | q304_M10a. What percent of patients require this item?<br>*Question relevant when: selected( ${q104_M10_full_list} , '2')*<br>*Response constrained to: .>0 and .<=100 or .=999* | |

| Field | Question | Answer |
|---|---|---|
| q304_M10b *(required)* | q304_M10b. How is the item administered, or given, to the patient?<br>*Question relevant when: selected( ${q104_M10_full_list} , '2')* | 1 Oral - solid (e.g. tablet)<br>2 Oral - liquid<br>3 Sublingual<br>4 Intravenous (IV)<br>5 Intramusculuar injection (IM)<br>6 Subcutaneous<br>7 Rectal<br>8 Inhaled (e.g. gas)<br>9 Inserted (e.g. vaginal pessaries)<br>10 Topical - cream<br>11 Topical - drops (e.g. eye drops)<br>12 Other<br>99 Don't know |
| q304_M10c *(required)* | q304_M10c. What is the item's smallest unit of measurement (mg, mcg, ml, g, etc)?<br>*Question relevant when: selected( ${q104_M10_full_list} , '2')* | microgram — microgram (mcg)<br>miligram — miligram (mg)<br>miligram_dissolved_in_liquid — miligram (mg) dissolved in liquid (e.g. 2 mg per 3 militers)<br>gram — gram (g)<br>militer — militer (ml)<br>centiliter — centiliter (cl)<br>litre — litre (l)<br>ml_of _solution — ml of solution (e.g. ml of 10% solution)<br>litre_of_solution — litre of solution (e.g. 1 litre of 5% solution)<br>cubic_meter — cubic meter (m^3)<br>other — other<br>dont_know — don't know |
| q304_M10c.1 | q304_M10c.1 If other unit of measurement or route of administration, specify here:<br>*Leave blank if not applicable*<br>*Question relevant when: selected( ${q104_M10_full_list} , '2')* | |
| q304_M10d *(required)* | q304_M10d. In total, how many units are used to treat a patient during all of her care?<br>*Remember to add all units! E.g. 5 days x 200 mg twice per day = 2,000mg*<br>*Question relevant when: selected( ${q104_M10_full_list} , '2')*<br>*Response constrained to: .>0* | |
| q304_M11 | ***q304_M11. Analgesic - other 2: "[q104_M11_full_list_other]"***<br>*Question relevant when: selected( ${q104_M11_full_list} , '2')* | |
| q304_M11a *(required)* | q304_M11a. What percent of patients require this item?<br>*Question relevant when: selected( ${q104_M11_full_list} , '2')*<br>*Response constrained to: .>0 and .<=100 or .=999* | |

| Field | Question | Answer |
|---|---|---|
| q304_M11b *(required)* | q304_M11b. How is the item administered, or given, to the patient?<br>*Question relevant when: selected( ${q104_M11_full_list} , '2')* | 1 Oral - solid (e.g. tablet)<br>2 Oral - liquid<br>3 Sublingual<br>4 Intravenous (IV)<br>5 Intramusculuar injection (IM)<br>6 Subcutaneous<br>7 Rectal<br>8 Inhaled (e.g. gas)<br>9 Inserted (e.g. vaginal pessaries)<br>10 Topical - cream<br>11 Topical - drops (e.g. eye drops)<br>12 Other<br>99 Don't know |
| q304_M11c *(required)* | q304_M11c. What is the item's smallest unit of measurement (mg, mcg, ml, g, etc)?<br>*Question relevant when: selected( ${q104_M11_full_list} , '2')* | microgram — microgram (mcg)<br>miligram — miligram (mg)<br>miligram_dissolved_in_liquid — miligram (mg) dissolved in liquid (e.g. 2 mg per 3 militers)<br>gram — gram (g)<br>militer — militer (ml)<br>centiliter — centiliter (cl)<br>litre — litre (l)<br>ml_of _solution — ml of solution (e.g. ml of 10% solution)<br>litre_of_solution — litre of solution (e.g. 1 litre of 5% solution)<br>cubic_meter — cubic meter (m^3)<br>other — other<br>dont_know — don't know |
| q304_M11c.1 | q304_M11c.1 If other unit of measurement or route of administration, specify here:<br>*Leave blank if not applicable*<br>*Question relevant when: selected( ${q104_M11_full_list} , '2')* | |
| q304_M11d *(required)* | q304_M11d. In total, how many units are used to treat a patient during all of her care?<br>*Remember to add all units! E.g. 5 days x 200 mg twice per day = 2,000mg*<br>*Question relevant when: selected( ${q104_M11_full_list} , '2')*<br>*Response constrained to: .>0* | |
| q304_M12 | ***q304_M12. Analgesic - other 3: "[q104_M12_full_list_other]"***<br>*Question relevant when: selected( ${q104_M12_full_list} , '2')* | |
| q304_M12a *(required)* | q304_M12a. What percent of patients require this item?<br>*Question relevant when: selected( ${q104_M12_full_list} , '2')*<br>*Response constrained to: .>0 and .<=100 or .=999* | |

| Field | Question | Answer |
|---|---|---|
| q304_M12b *(required)* | q304_M12b. How is the item administered, or given, to the patient?<br>*Question relevant when: selected( ${q104_M12_full_list} , '2')* | 1 Oral - solid (e.g. tablet)<br>2 Oral - liquid<br>3 Sublingual<br>4 Intravenous (IV)<br>5 Intramusculuar injection (IM)<br>6 Subcutaneous<br>7 Rectal<br>8 Inhaled (e.g. gas)<br>9 Inserted (e.g. vaginal pessaries)<br>10 Topical - cream<br>11 Topical - drops (e.g. eye drops)<br>12 Other<br>99 Don't know |
| q304_M12c *(required)* | q304_M12c. What is the item's smallest unit of measurement (mg, mcg, ml, g, etc)?<br>*Question relevant when: selected( ${q104_M12_full_list} , '2')* | microgram — microgram (mcg)<br>miligram — miligram (mg)<br>miligram_dissolved_in_liquid — miligram (mg) dissolved in liquid (e.g. 2 mg per 3 militers)<br>gram — gram (g)<br>militer — militer (ml)<br>centiliter — centiliter (cl)<br>litre — litre (l)<br>ml_of _solution — ml of solution (e.g. ml of 10% solution)<br>litre_of_solution — litre of solution (e.g. 1 litre of 5% solution)<br>cubic_meter — cubic meter (m^3)<br>other — other<br>dont_know — don't know |
| q304_M12c.1 | q304_M12c.1 If other unit of measurement or route of administration, specify here:<br>*Leave blank if not applicable*<br>*Question relevant when: selected( ${q104_M12_full_list} , '2')* | |
| q304_M12d *(required)* | q304_M12d. In total, how many units are used to treat a patient during all of her care?<br>*Remember to add all units! E.g. 5 days x 200 mg twice per day = 2,000mg*<br>*Question relevant when: selected( ${q104_M12_full_list} , '2')*<br>*Response constrained to: .>0* | |
| q304_M13 | ***q304_M13. Analgesic - other 4: "[q104_M13_full_list_other]"***<br>*Question relevant when: selected( ${q104_M13_full_list} , '2')* | |
| q304_M13a *(required)* | q304_M13a. What percent of patients require this item?<br>*Question relevant when: selected( ${q104_M13_full_list} , '2')*<br>*Response constrained to: .>0 and .<=100 or .=999* | |

| Field | Question | Answer |
|---|---|---|
| q304_M13b *(required)* | q304_M13b. How is the item administered, or given, to the patient?<br>*Question relevant when: selected( ${q104_M13_full_list} , '2')* | 1 Oral - solid (e.g. tablet)<br>2 Oral - liquid<br>3 Sublingual<br>4 Intravenous (IV)<br>5 Intramusculuar injection (IM)<br>6 Subcutaneous<br>7 Rectal<br>8 Inhaled (e.g. gas)<br>9 Inserted (e.g. vaginal pessaries)<br>10 Topical - cream<br>11 Topical - drops (e.g. eye drops)<br>12 Other<br>99 Don't know |
| q304_M13c *(required)* | q304_M13c. What is the item's smallest unit of measurement (mg, mcg, ml, g, etc)?<br>*Question relevant when: selected( ${q104_M13_full_list} , '2')* | microgram — microgram (mcg)<br>miligram — miligram (mg)<br>miligram_dissolved_in_liquid — miligram (mg) dissolved in liquid (e.g. 2 mg per 3 militers)<br>gram — gram (g)<br>militer — militer (ml)<br>centiliter — centiliter (cl)<br>litre — litre (l)<br>ml_of _solution — ml of solution (e.g. ml of 10% solution)<br>litre_of_solution — litre of solution (e.g. 1 litre of 5% solution)<br>cubic_meter — cubic meter (m^3)<br>other — other<br>dont_know — don't know |
| q304_M13c.1 | q304_M13c.1 If other unit of measurement or route of administration, specify here:<br>*Leave blank if not applicable*<br>*Question relevant when: selected( ${q104_M13_full_list} , '2')* | |
| q304_M13d *(required)* | q304_M13d. In total, how many units are used to treat a patient during all of her care?<br>*Remember to add all units! E.g. 5 days x 200 mg twice per day = 2,000mg*<br>*Question relevant when: selected( ${q104_M13_full_list} , '2')*<br>*Response constrained to: .>0* | |
| q304_M14 | ***q304_M14. Analgesic - other 5: "[q104_M14_full_list_other]"***<br>*Question relevant when: selected( ${q104_M14_full_list} , '2')* | |
| q304_M14a *(required)* | q304_M14a. What percent of patients require this item?<br>*Question relevant when: selected( ${q104_M14_full_list} , '2')*<br>*Response constrained to: .>0 and .<=100 or .=999* | |

| Field | Question | Answer |
|---|---|---|
| q304_M14b *(required)* | q304_M14b. How is the item administered, or given, to the patient? *Question relevant when: selected( ${q104_M14_full_list} , '2')* | 1 Oral - solid (e.g. tablet)<br>2 Oral - liquid<br>3 Sublingual<br>4 Intravenous (IV)<br>5 Intramusculuar injection (IM)<br>6 Subcutaneous<br>7 Rectal<br>8 Inhaled (e.g. gas)<br>9 Inserted (e.g. vaginal pessaries)<br>10 Topical - cream<br>11 Topical - drops (e.g. eye drops)<br>12 Other<br>99 Don't know |
| q304_M14c *(required)* | q304_M14c. What is the item's smallest unit of measurement (mg, mcg, ml, g, etc)? *Question relevant when: selected( ${q104_M14_full_list} , '2')* | microgram — microgram (mcg)<br>miligram — miligram (mg)<br>miligram_dissolved_in_liquid — miligram (mg) dissolved in liquid (e.g. 2 mg per 3 militers)<br>gram — gram (g)<br>militer — militer (ml)<br>centiliter — centiliter (cl)<br>litre — litre (l)<br>ml_of _solution — ml of solution (e.g. ml of 10% solution)<br>litre_of_solution — litre of solution (e.g. 1 litre of 5% solution)<br>cubic_meter — cubic meter (m^3)<br>other — other<br>dont_know — don't know |
| q304_M14c.1 | q304_M14c.1 If other unit of measurement or route of administration, specify here: *Leave blank if not applicable* *Question relevant when: selected( ${q104_M14_full_list} , '2')* | |
| q304_M14d *(required)* | q304_M14d. In total, how many units are used to treat a patient during all of her care? *Remember to add all units! E.g. 5 days x 200 mg twice per day = 2,000mg* *Question relevant when: selected( ${q104_M14_full_list} , '2')* *Response constrained to: .>0* | |
| <p><strong><em>D. Medications - Sepsis (2)</em></strong></p> *Group relevant when: selected( ${section_three_skip_med} , '1')* | | |
| q304_med_note2 | 304 For each of the following medications and other medical products that are used for sepsis, can you tell me …? | |
| note_304_anesthetic | ***Anesthetic - local, general, etc. (and oxygen)*** | |
| q304_M15 | ***q304_M15. Halothane*** *Question relevant when: selected( ${q104_M15_full_list} , '2')* | |
| q304_M15a *(required)* | q304_M15a. What percent of patients require this item? *Question relevant when: selected( ${q104_M15_full_list} , '2')* | |

| Field | Question | Answer |
|---|---|---|
| | *Response constrained to: .>0 and .<=100 or .=999* | |

| Field | Question | Answer |
|---|---|---|
| q304_M15b *(required)* | q304_M15b. How is the item administered, or given, to the patient?<br>*Question relevant when: selected( ${q104_M15_full_list} , '2')* | 1 Oral - solid (e.g. tablet)<br>2 Oral - liquid<br>3 Sublingual<br>4 Intravenous (IV)<br>5 Intramusculuar injection (IM)<br>6 Subcutaneous<br>7 Rectal<br>8 Inhaled (e.g. gas)<br>9 Inserted (e.g. vaginal pessaries)<br>10 Topical - cream<br>11 Topical - drops (e.g. eye drops)<br>12 Other<br>99 Don't know |
| q304_M15c *(required)* | q304_M15c. What is the item's smallest unit of measurement (mg, mcg, ml, g, etc)?<br>*Question relevant when: selected( ${q104_M15_full_list} , '2')* | microgram — microgram (mcg)<br>miligram — miligram (mg)<br>miligram_dissolved_in_liquid — miligram (mg) dissolved in liquid (e.g. 2 mg per 3 militers)<br>gram — gram (g)<br>militer — militer (ml)<br>centiliter — centiliter (cl)<br>litre — litre (l)<br>ml_of _solution — ml of solution (e.g. ml of 10% solution)<br>litre_of_solution — litre of solution (e.g. 1 litre of 5% solution)<br>cubic_meter — cubic meter (m^3)<br>other — other<br>dont_know — don't know |
| q304_M15c.1 | q304_M15c.1 If other unit of measurement or route of administration, specify here:<br>*Leave blank if not applicable*<br>*Question relevant when: selected( ${q104_M15_full_list} , '2')* | |
| q304_M15d *(required)* | q304_M15d. In total, how many units are used to treat a patient during all of her care?<br>*Remember to add all units! E.g. 5 days x 200 mg twice per day = 2,000mg*<br>*Question relevant when: selected( ${q104_M15_full_list} , '2')*<br>*Response constrained to: .>0* | |
| q304_M16 | ***q304_M16. Ketamine (Ketalar), 50mg/ml***<br>*Question relevant when: selected( ${q104_M16_full_list} , '2')* | |
| q304_M16a *(required)* | q304_M16a. What percent of patients require this item?<br>*Question relevant when: selected( ${q104_M16_full_list} , '2')*<br>*Response constrained to: .>0 and .<=100 or .=999* | |

| Field | Question | Answer |
|---|---|---|
| q304_M16b *(required)* | q304_M16b. How is the item administered, or given, to the patient?<br>*Question relevant when: selected( ${q104_M16_full_list} , '2')* | 1 Oral - solid (e.g. tablet)<br>2 Oral - liquid<br>3 Sublingual<br>4 Intravenous (IV)<br>5 Intramusculuar injection (IM)<br>6 Subcutaneous<br>7 Rectal<br>8 Inhaled (e.g. gas)<br>9 Inserted (e.g. vaginal pessaries)<br>10 Topical - cream<br>11 Topical - drops (e.g. eye drops)<br>12 Other<br>99 Don't know |
| q304_M16c *(required)* | q304_M16c. What is the item's smallest unit of measurement (mg, mcg, ml, g, etc)?<br>*Question relevant when: selected( ${q104_M16_full_list} , '2')* | microgram — microgram (mcg)<br>miligram — miligram (mg)<br>miligram_dissolved_in_liquid — miligram (mg) dissolved in liquid (e.g. 2 mg per 3 militers)<br>gram — gram (g)<br>militer — militer (ml)<br>centiliter — centiliter (cl)<br>litre — litre (l)<br>ml_of _solution — ml of solution (e.g. ml of 10% solution)<br>litre_of_solution — litre of solution (e.g. 1 litre of 5% solution)<br>cubic_meter — cubic meter (m^3)<br>other — other<br>dont_know — don't know |
| q304_M16c.1 | q304_M16c.1 If other unit of measurement or route of administration, specify here:<br>*Leave blank if not applicable*<br>*Question relevant when: selected( ${q104_M16_full_list} , '2')* | |
| q304_M16d *(required)* | q304_M16d. In total, how many units are used to treat a patient during all of her care?<br>*Remember to add all units! E.g. 5 days x 200 mg twice per day = 2,000mg*<br>*Question relevant when: selected( ${q104_M16_full_list} , '2')*<br>*Response constrained to: .>0* | |
| q304_M17 | ***q304_M17. Propofol (Diprivan), 10mg/ml***<br>*Question relevant when: selected( ${q104_M17_full_list} , '2')* | |
| q304_M17a *(required)* | q304_M17a. What percent of patients require this item?<br>*Question relevant when: selected( ${q104_M17_full_list} , '2')*<br>*Response constrained to: .>0 and .<=100 or .=999* | |

| Field | Question | Answer |
|---|---|---|
| q304_M17b *(required)* | q304_M17b. How is the item administered, or given, to the patient?<br>*Question relevant when: selected( ${q104_M17_full_list} , '2')* | 1 Oral - solid (e.g. tablet)<br>2 Oral - liquid<br>3 Sublingual<br>4 Intravenous (IV)<br>5 Intramusculuar injection (IM)<br>6 Subcutaneous<br>7 Rectal<br>8 Inhaled (e.g. gas)<br>9 Inserted (e.g. vaginal pessaries)<br>10 Topical - cream<br>11 Topical - drops (e.g. eye drops)<br>12 Other<br>99 Don't know |
| q304_M17c *(required)* | q304_M17c. What is the item's smallest unit of measurement (mg, mcg, ml, g, etc)?<br>*Question relevant when: selected( ${q104_M17_full_list} , '2')* | microgram — microgram (mcg)<br>miligram — miligram (mg)<br>miligram_dissolved_in_liquid — miligram (mg) dissolved in liquid (e.g. 2 mg per 3 militers)<br>gram — gram (g)<br>militer — militer (ml)<br>centiliter — centiliter (cl)<br>litre — litre (l)<br>ml_of _solution — ml of solution (e.g. ml of 10% solution)<br>litre_of_solution — litre of solution (e.g. 1 litre of 5% solution)<br>cubic_meter — cubic meter (m^3)<br>other — other<br>dont_know — don't know |
| q304_M17c.1 | q304_M17c.1 If other unit of measurement or route of administration, specify here:<br>*Leave blank if not applicable*<br>*Question relevant when: selected( ${q104_M17_full_list} , '2')* | |
| q304_M17d *(required)* | q304_M17d. In total, how many units are used to treat a patient during all of her care?<br>*Remember to add all units! E.g. 5 days x 200 mg twice per day = 2,000mg*<br>*Question relevant when: selected( ${q104_M17_full_list} , '2')*<br>*Response constrained to: .>0* | |
| q304_M18 | ***q304_M18. Suxamethonium chloride***<br>*Question relevant when: selected( ${q104_M18_full_list} , '2')* | |
| q304_M18a *(required)* | q304_M18a. What percent of patients require this item?<br>*Question relevant when: selected( ${q104_M18_full_list} , '2')*<br>*Response constrained to: .>0 and .<=100 or .=999* | |

| Field | Question | Answer |
|---|---|---|
| q304_M18b *(required)* | q304_M18b. How is the item administered, or given, to the patient?<br>*Question relevant when: selected( ${q104_M18_full_list} , '2')* | 1 Oral - solid (e.g. tablet)<br>2 Oral - liquid<br>3 Sublingual<br>4 Intravenous (IV)<br>5 Intramusculuar injection (IM)<br>6 Subcutaneous<br>7 Rectal<br>8 Inhaled (e.g. gas)<br>9 Inserted (e.g. vaginal pessaries)<br>10 Topical - cream<br>11 Topical - drops (e.g. eye drops)<br>12 Other<br>99 Don't know |
| q304_M18c *(required)* | q304_M18c. What is the item's smallest unit of measurement (mg, mcg, ml, g, etc)?<br>*Question relevant when: selected( ${q104_M18_full_list} , '2')* | microgram — microgram (mcg)<br>miligram — miligram (mg)<br>miligram_dissolved_in_liquid — miligram (mg) dissolved in liquid (e.g. 2 mg per 3 militers)<br>gram — gram (g)<br>militer — militer (ml)<br>centiliter — centiliter (cl)<br>litre — litre (l)<br>ml_of _solution — ml of solution (e.g. ml of 10% solution)<br>litre_of_solution — litre of solution (e.g. 1 litre of 5% solution)<br>cubic_meter — cubic meter (m^3)<br>other — other<br>dont_know — don't know |
| q304_M18c.1 | q304_M18c.1 If other unit of measurement or route of administration, specify here:<br>*Leave blank if not applicable*<br>*Question relevant when: selected( ${q104_M18_full_list} , '2')* | |
| q304_M18d *(required)* | q304_M18d. In total, how many units are used to treat a patient during all of her care?<br>*Remember to add all units! E.g. 5 days x 200 mg twice per day = 2,000mg*<br>*Question relevant when: selected( ${q104_M18_full_list} , '2')*<br>*Response constrained to: .>0* | |
| q304_M19 | ***q304_M19. Thiopental sodium (Intraval)***<br>*Question relevant when: selected( ${q104_M19_full_list} , '2')* | |
| q304_M19a *(required)* | q304_M19a. What percent of patients require this item?<br>*Question relevant when: selected( ${q104_M19_full_list} , '2')*<br>*Response constrained to: .>0 and .<=100 or .=999* | |

| Field | Question | Answer |
|---|---|---|
| q304_M19b *(required)* | q304_M19b. How is the item administered, or given, to the patient?<br>*Question relevant when: selected( ${q104_M19_full_list} , '2')* | 1 Oral - solid (e.g. tablet)<br>2 Oral - liquid<br>3 Sublingual<br>4 Intravenous (IV)<br>5 Intramusculuar injection (IM)<br>6 Subcutaneous<br>7 Rectal<br>8 Inhaled (e.g. gas)<br>9 Inserted (e.g. vaginal pessaries)<br>10 Topical - cream<br>11 Topical - drops (e.g. eye drops)<br>12 Other<br>99 Don't know |
| q304_M19c *(required)* | q304_M19c. What is the item's smallest unit of measurement (mg, mcg, ml, g, etc)?<br>*Question relevant when: selected( ${q104_M19_full_list} , '2')* | microgram — microgram (mcg)<br>miligram — miligram (mg)<br>miligram_dissolved_in_liquid — miligram (mg) dissolved in liquid (e.g. 2 mg per 3 militers)<br>gram — gram (g)<br>militer — militer (ml)<br>centiliter — centiliter (cl)<br>litre — litre (l)<br>ml_of _solution — ml of solution (e.g. ml of 10% solution)<br>litre_of_solution — litre of solution (e.g. 1 litre of 5% solution)<br>cubic_meter — cubic meter (m^3)<br>other — other<br>dont_know — don't know |
| q304_M19c.1 | q304_M19c.1 If other unit of measurement or route of administration, specify here:<br>*Leave blank if not applicable*<br>*Question relevant when: selected( ${q104_M19_full_list} , '2')* | |
| q304_M19d *(required)* | q304_M19d. In total, how many units are used to treat a patient during all of her care?<br>*Remember to add all units! E.g. 5 days x 200 mg twice per day = 2,000mg*<br>*Question relevant when: selected( ${q104_M19_full_list} , '2')*<br>*Response constrained to: .>0* | |
| q304_M20 | ***q304_M20. Oxygen***<br>*Question relevant when: selected( ${q104_M20_full_list} , '2')* | |
| q304_M20a *(required)* | q304_M20a. What percent of patients require this item?<br>*Question relevant when: selected( ${q104_M20_full_list} , '2')*<br>*Response constrained to: .>0 and .<=100 or .=999* | |

| Field | Question | Answer |
|---|---|---|
| q304_M20b *(required)* | q304_M20b. How is the item administered, or given, to the patient?<br>*Question relevant when: selected( ${q104_M20_full_list} , '2')* | 1 Oral - solid (e.g. tablet)<br>2 Oral - liquid<br>3 Sublingual<br>4 Intravenous (IV)<br>5 Intramusculuar injection (IM)<br>6 Subcutaneous<br>7 Rectal<br>8 Inhaled (e.g. gas)<br>9 Inserted (e.g. vaginal pessaries)<br>10 Topical - cream<br>11 Topical - drops (e.g. eye drops)<br>12 Other<br>99 Don't know |
| q304_M20c *(required)* | q304_M20c. What is the item's smallest unit of measurement (mg, mcg, ml, g, etc)?<br>*Question relevant when: selected( ${q104_M20_full_list} , '2')* | microgram — microgram (mcg)<br>miligram — miligram (mg)<br>miligram_dissolved_in_liquid — miligram (mg) dissolved in liquid (e.g. 2 mg per 3 militers)<br>gram — gram (g)<br>militer — militer (ml)<br>centiliter — centiliter (cl)<br>litre — litre (l)<br>ml_of _solution — ml of solution (e.g. ml of 10% solution)<br>litre_of_solution — litre of solution (e.g. 1 litre of 5% solution)<br>cubic_meter — cubic meter (m^3)<br>other — other<br>dont_know — don't know |
| q304_M20c.1 | q304_M20c.1 If other unit of measurement or route of administration, specify here:<br>*Leave blank if not applicable*<br>*Question relevant when: selected( ${q104_M20_full_list} , '2')* | |
| q304_M20d *(required)* | q304_M20d. In total, how many units are used to treat a patient during all of her care?<br>*Remember to add all units! E.g. 5 days x 200 mg twice per day = 2,000mg*<br>*Question relevant when: selected( ${q104_M20_full_list} , '2')*<br>*Response constrained to: .>0* | |
| q304_M21 | ***q304_M21. Bupivacaine HCL 0.5% (Marcaine)***<br>*Question relevant when: selected( ${q104_M21_full_list} , '2')* | |
| q304_M21a *(required)* | q304_M21a. What percent of patients require this item?<br>*Question relevant when: selected( ${q104_M21_full_list} , '2')*<br>*Response constrained to: .>0 and .<=100 or .=999* | |

| Field | Question | Answer |
|---|---|---|
| q304_M21b *(required)* | q304_M21b. How is the item administered, or given, to the patient?<br>*Question relevant when: selected( ${q104_M21_full_list} , '2')* | 1 Oral - solid (e.g. tablet)<br>2 Oral - liquid<br>3 Sublingual<br>4 Intravenous (IV)<br>5 Intramusculuar injection (IM)<br>6 Subcutaneous<br>7 Rectal<br>8 Inhaled (e.g. gas)<br>9 Inserted (e.g. vaginal pessaries)<br>10 Topical - cream<br>11 Topical - drops (e.g. eye drops)<br>12 Other<br>99 Don't know |
| q304_M21c *(required)* | q304_M21c. What is the item's smallest unit of measurement (mg, mcg, ml, g, etc)?<br>*Question relevant when: selected( ${q104_M21_full_list} , '2')* | microgram — microgram (mcg)<br>miligram — miligram (mg)<br>miligram_dissolved_in_liquid — miligram (mg) dissolved in liquid (e.g. 2 mg per 3 militers)<br>gram — gram (g)<br>militer — militer (ml)<br>centiliter — centiliter (cl)<br>litre — litre (l)<br>ml_of _solution — ml of solution (e.g. ml of 10% solution)<br>litre_of_solution — litre of solution (e.g. 1 litre of 5% solution)<br>cubic_meter — cubic meter (m^3)<br>other — other<br>dont_know — don't know |
| q304_M21c.1 | q304_M21c.1 If other unit of measurement or route of administration, specify here:<br>*Leave blank if not applicable*<br>*Question relevant when: selected( ${q104_M21_full_list} , '2')* | |
| q304_M21d *(required)* | q304_M21d. In total, how many units are used to treat a patient during all of her care?<br>*Remember to add all units! E.g. 5 days x 200 mg twice per day = 2,000mg*<br>*Question relevant when: selected( ${q104_M21_full_list} , '2')*<br>*Response constrained to: .>0* | |
| q304_M22 | ***q304_M22. Lidocaine HCl 2% + epinephrin 1:100,000***<br>*Question relevant when: selected( ${q104_M22_full_list} , '2')* | |
| q304_M22a *(required)* | q304_M22a. What percent of patients require this item?<br>*Question relevant when: selected( ${q104_M22_full_list} , '2')*<br>*Response constrained to: .>0 and .<=100 or .=999* | |

<table>
<tr><th>Field</th><th>Question</th><th>Answer</th></tr>
<tr><td>q304_M22b (required)</td><td>q304_M22b. How is the item administered, or given, to the patient?<br>Question relevant when: selected( ${q104_M22_full_list} , '2')</td><td><table>
<tr><td>1</td><td>Oral - solid (e.g. tablet)</td></tr>
<tr><td>2</td><td>Oral - liquid</td></tr>
<tr><td>3</td><td>Sublingual</td></tr>
<tr><td>4</td><td>Intravenous (IV)</td></tr>
<tr><td>5</td><td>Intramusculuar injection (IM)</td></tr>
<tr><td>6</td><td>Subcutaneous</td></tr>
<tr><td>7</td><td>Rectal</td></tr>
<tr><td>8</td><td>Inhaled (e.g. gas)</td></tr>
<tr><td>9</td><td>Inserted (e.g. vaginal pessaries)</td></tr>
<tr><td>10</td><td>Topical - cream</td></tr>
<tr><td>11</td><td>Topical - drops (e.g. eye drops)</td></tr>
<tr><td>12</td><td>Other</td></tr>
<tr><td>99</td><td>Don't know</td></tr>
</table></td></tr>
<tr><td>q304_M22c (required)</td><td>q304_M22c. What is the item's smallest unit of measurement (mg, mcg, ml, g, etc)?<br>Question relevant when: selected( ${q104_M22_full_list} , '2')</td><td><table>
<tr><td>microgram</td><td>microgram (mcg)</td></tr>
<tr><td>miligram</td><td>miligram (mg)</td></tr>
<tr><td>miligram_dissolved_in_liquid</td><td>miligram (mg) dissolved in liquid (e.g. 2 mg per 3 militers)</td></tr>
<tr><td>gram</td><td>gram (g)</td></tr>
<tr><td>militer</td><td>militer (ml)</td></tr>
<tr><td>centiliter</td><td>centiliter (cl)</td></tr>
<tr><td>litre</td><td>litre (l)</td></tr>
<tr><td>ml_of _solution</td><td>ml of solution (e.g. ml of 10% solution)</td></tr>
<tr><td>litre_of_solution</td><td>litre of solution (e.g. 1 litre of 5% solution)</td></tr>
<tr><td>cubic_meter</td><td>cubic meter (m^3)</td></tr>
<tr><td>other</td><td>other</td></tr>
<tr><td>dont_know</td><td>don't know</td></tr>
</table></td></tr>
<tr><td>q304_M22c.1</td><td>q304_M22c.1 If other unit of measurement or route of administration, specify here:<br>Leave blank if not applicable<br>Question relevant when: selected( ${q104_M22_full_list} , '2')</td><td></td></tr>
<tr><td>q304_M22d (required)</td><td>q304_M22d. In total, how many units are used to treat a patient during all of her care?<br>Remember to add all units! E.g. 5 days x 200 mg twice per day = 2,000mg<br>Question relevant when: selected( ${q104_M22_full_list} , '2')<br>Response constrained to: .>0</td><td></td></tr>
<tr><td>q304_M23</td><td>q304_M23. Lidocaine HCl 5% + dextrose 7.5%<br>Question relevant when: selected( ${q104_M23_full_list} , '2')</td><td></td></tr>
<tr><td>q304_M23a (required)</td><td>q304_M23a. What percent of patients require this item?<br>Question relevant when: selected( ${q104_M23_full_list} , '2')<br>Response constrained to: .>0 and .<=100 or .=999</td><td></td></tr>
</table>

| Field | Question | Answer |
|---|---|---|
| q304_M23b *(required)* | q304_M23b. How is the item administered, or given, to the patient?<br>*Question relevant when: selected( ${q104_M23_full_list} , '2')* | 1 Oral - solid (e.g. tablet)<br>2 Oral - liquid<br>3 Sublingual<br>4 Intravenous (IV)<br>5 Intramusculuar injection (IM)<br>6 Subcutaneous<br>7 Rectal<br>8 Inhaled (e.g. gas)<br>9 Inserted (e.g. vaginal pessaries)<br>10 Topical - cream<br>11 Topical - drops (e.g. eye drops)<br>12 Other<br>99 Don't know |
| q304_M23c *(required)* | q304_M23c. What is the item's smallest unit of measurement (mg, mcg, ml, g, etc)?<br>*Question relevant when: selected( ${q104_M23_full_list} , '2')* | microgram — microgram (mcg)<br>miligram — miligram (mg)<br>miligram_dissolved_in_liquid — miligram (mg) dissolved in liquid (e.g. 2 mg per 3 militers)<br>gram — gram (g)<br>militer — militer (ml)<br>centiliter — centiliter (cl)<br>litre — litre (l)<br>ml_of _solution — ml of solution (e.g. ml of 10% solution)<br>litre_of_solution — litre of solution (e.g. 1 litre of 5% solution)<br>cubic_meter — cubic meter (m^3)<br>other — other<br>dont_know — don't know |
| q304_M23c.1 | q304_M23c.1 If other unit of measurement or route of administration, specify here:<br>*Leave blank if not applicable*<br>*Question relevant when: selected( ${q104_M23_full_list} , '2')* | |
| q304_M23d *(required)* | q304_M23d. In total, how many units are used to treat a patient during all of her care?<br>*Remember to add all units! E.g. 5 days x 200 mg twice per day = 2,000mg*<br>*Question relevant when: selected( ${q104_M23_full_list} , '2')*<br>*Response constrained to: .>0* | |
| q304_M24 | ***q304_M24. Lidocaine HCl, 1%***<br>*Question relevant when: selected( ${q104_M24_full_list} , '2')* | |
| q304_M24a *(required)* | q304_M24a. What percent of patients require this item?<br>*Question relevant when: selected( ${q104_M24_full_list} , '2')*<br>*Response constrained to: .>0 and .<=100 or .=999* | |

| Field | Question | Answer |
|---|---|---|
| q304_M24b *(required)* | q304_M24b. How is the item administered, or given, to the patient?<br>*Question relevant when: selected( ${q104_M24_full_list} , '2')* | 1 Oral - solid (e.g. tablet)<br>2 Oral - liquid<br>3 Sublingual<br>4 Intravenous (IV)<br>5 Intramusculuar injection (IM)<br>6 Subcutaneous<br>7 Rectal<br>8 Inhaled (e.g. gas)<br>9 Inserted (e.g. vaginal pessaries)<br>10 Topical - cream<br>11 Topical - drops (e.g. eye drops)<br>12 Other<br>99 Don't know |
| q304_M24c *(required)* | q304_M24c. What is the item's smallest unit of measurement (mg, mcg, ml, g, etc)?<br>*Question relevant when: selected( ${q104_M24_full_list} , '2')* | microgram — microgram (mcg)<br>miligram — miligram (mg)<br>miligram_dissolved_in_liquid — miligram (mg) dissolved in liquid (e.g. 2 mg per 3 militers)<br>gram — gram (g)<br>militer — militer (ml)<br>centiliter — centiliter (cl)<br>litre — litre (l)<br>ml_of _solution — ml of solution (e.g. ml of 10% solution)<br>litre_of_solution — litre of solution (e.g. 1 litre of 5% solution)<br>cubic_meter — cubic meter (m^3)<br>other — other<br>dont_know — don't know |
| q304_M24c.1 | q304_M24c.1 If other unit of measurement or route of administration, specify here:<br>*Leave blank if not applicable*<br>*Question relevant when: selected( ${q104_M24_full_list} , '2')* | |
| q304_M24d *(required)* | q304_M24d. In total, how many units are used to treat a patient during all of her care?<br>*Remember to add all units! E.g. 5 days x 200 mg twice per day = 2,000mg*<br>*Question relevant when: selected( ${q104_M24_full_list} , '2')*<br>*Response constrained to: .>0* | |
| q304_M25 | ***q304_M25. Anesthetic - other 1: "[q104_M25_full_list_other]"***<br>*Question relevant when: selected( ${q104_M25_full_list} , '2')* | |
| q304_M25a *(required)* | q304_M25a. What percent of patients require this item?<br>*Question relevant when: selected( ${q104_M25_full_list} , '2')*<br>*Response constrained to: .>0 and .<=100 or .=999* | |

| Field | Question | Answer |
|---|---|---|
| q304_M25b *(required)* | q304_M25b. How is the item administered, or given, to the patient?<br>*Question relevant when: selected( ${q104_M25_full_list} , '2')* | 1 Oral - solid (e.g. tablet)<br>2 Oral - liquid<br>3 Sublingual<br>4 Intravenous (IV)<br>5 Intramusculuar injection (IM)<br>6 Subcutaneous<br>7 Rectal<br>8 Inhaled (e.g. gas)<br>9 Inserted (e.g. vaginal pessaries)<br>10 Topical - cream<br>11 Topical - drops (e.g. eye drops)<br>12 Other<br>99 Don't know |
| q304_M25c *(required)* | q304_M25c. What is the item's smallest unit of measurement (mg, mcg, ml, g, etc)?<br>*Question relevant when: selected( ${q104_M25_full_list} , '2')* | microgram — microgram (mcg)<br>miligram — miligram (mg)<br>miligram_dissolved_in_liquid — miligram (mg) dissolved in liquid (e.g. 2 mg per 3 militers)<br>gram — gram (g)<br>militer — militer (ml)<br>centiliter — centiliter (cl)<br>litre — litre (l)<br>ml_of _solution — ml of solution (e.g. ml of 10% solution)<br>litre_of_solution — litre of solution (e.g. 1 litre of 5% solution)<br>cubic_meter — cubic meter (m^3)<br>other — other<br>dont_know — don't know |
| q304_M25c.1 | q304_M25c.1 If other unit of measurement or route of administration, specify here:<br>*Leave blank if not applicable*<br>*Question relevant when: selected( ${q104_M25_full_list} , '2')* | |
| q304_M25d *(required)* | q304_M25d. In total, how many units are used to treat a patient during all of her care?<br>*Remember to add all units! E.g. 5 days x 200 mg twice per day = 2,000mg*<br>*Question relevant when: selected( ${q104_M25_full_list} , '2')*<br>*Response constrained to: .>0* | |
| q304_M26 | ***q304_M26. Anesthetic - other 2: "[q104_M26_full_list_other]"***<br>*Question relevant when: selected( ${q104_M26_full_list} , '2')* | |
| q304_M26a *(required)* | q304_M26a. What percent of patients require this item?<br>*Question relevant when: selected( ${q104_M26_full_list} , '2')*<br>*Response constrained to: .>0 and .<=100 or .=999* | |

| Field | Question | Answer |
|---|---|---|
| q304_M26b *(required)* | q304_M26b. How is the item administered, or given, to the patient?<br>*Question relevant when: selected( ${q104_M26_full_list} , '2')* | 1 Oral - solid (e.g. tablet)<br>2 Oral - liquid<br>3 Sublingual<br>4 Intravenous (IV)<br>5 Intramusculuar injection (IM)<br>6 Subcutaneous<br>7 Rectal<br>8 Inhaled (e.g. gas)<br>9 Inserted (e.g. vaginal pessaries)<br>10 Topical - cream<br>11 Topical - drops (e.g. eye drops)<br>12 Other<br>99 Don't know |
| q304_M26c *(required)* | q304_M26c. What is the item's smallest unit of measurement (mg, mcg, ml, g, etc)?<br>*Question relevant when: selected( ${q104_M26_full_list} , '2')* | microgram — microgram (mcg)<br>miligram — miligram (mg)<br>miligram_dissolved_in_liquid — miligram (mg) dissolved in liquid (e.g. 2 mg per 3 militers)<br>gram — gram (g)<br>militer — militer (ml)<br>centiliter — centiliter (cl)<br>litre — litre (l)<br>ml_of _solution — ml of solution (e.g. ml of 10% solution)<br>litre_of_solution — litre of solution (e.g. 1 litre of 5% solution)<br>cubic_meter — cubic meter (m^3)<br>other — other<br>dont_know — don't know |
| q304_M26c.1 | q304_M26c.1 If other unit of measurement or route of administration, specify here:<br>*Leave blank if not applicable*<br>*Question relevant when: selected( ${q104_M26_full_list} , '2')* | |
| q304_M26d *(required)* | q304_M26d. In total, how many units are used to treat a patient during all of her care?<br>*Remember to add all units! E.g. 5 days x 200 mg twice per day = 2,000mg*<br>*Question relevant when: selected( ${q104_M26_full_list} , '2')*<br>*Response constrained to: .>0* | |
| q304_M27 | ***q304_M27. Anesthetic - other 3: "[q104_M27_full_list_other]"***<br>*Question relevant when: selected( ${q104_M27_full_list} , '2')* | |
| q304_M27a *(required)* | q304_M27a. What percent of patients require this item?<br>*Question relevant when: selected( ${q104_M27_full_list} , '2')*<br>*Response constrained to: .>0 and .<=100 or .=999* | |

| Field | Question | Answer |
|---|---|---|
| q304_M27b *(required)* | q304_M27b. How is the item administered, or given, to the patient?<br>*Question relevant when: selected( ${q104_M27_full_list} , '2')* | 1 Oral - solid (e.g. tablet)<br>2 Oral - liquid<br>3 Sublingual<br>4 Intravenous (IV)<br>5 Intramusculuar injection (IM)<br>6 Subcutaneous<br>7 Rectal<br>8 Inhaled (e.g. gas)<br>9 Inserted (e.g. vaginal pessaries)<br>10 Topical - cream<br>11 Topical - drops (e.g. eye drops)<br>12 Other<br>99 Don't know |
| q304_M27c *(required)* | q304_M27c. What is the item's smallest unit of measurement (mg, mcg, ml, g, etc)?<br>*Question relevant when: selected( ${q104_M27_full_list} , '2')* | microgram — microgram (mcg)<br>miligram — miligram (mg)<br>miligram_dissolved_in_liquid — miligram (mg) dissolved in liquid (e.g. 2 mg per 3 militers)<br>gram — gram (g)<br>militer — militer (ml)<br>centiliter — centiliter (cl)<br>litre — litre (l)<br>ml_of _solution — ml of solution (e.g. ml of 10% solution)<br>litre_of_solution — litre of solution (e.g. 1 litre of 5% solution)<br>cubic_meter — cubic meter (m^3)<br>other — other<br>dont_know — don't know |
| q304_M27c.1 | q304_M27c.1 If other unit of measurement or route of administration, specify here:<br>*Leave blank if not applicable*<br>*Question relevant when: selected( ${q104_M27_full_list} , '2')* | |
| q304_M27d *(required)* | q304_M27d. In total, how many units are used to treat a patient during all of her care?<br>*Remember to add all units! E.g. 5 days x 200 mg twice per day = 2,000mg*<br>*Question relevant when: selected( ${q104_M27_full_list} , '2')*<br>*Response constrained to: .>0* | |
| q304_M28 | ***q304_M28. Anesthetic - other 4: "[q104_M28_full_list_other]"***<br>*Question relevant when: selected( ${q104_M28_full_list} , '2')* | |
| q304_M28a *(required)* | q304_M28a. What percent of patients require this item?<br>*Question relevant when: selected( ${q104_M28_full_list} , '2')*<br>*Response constrained to: .>0 and .<=100 or .=999* | |

| Field | Question | Answer |
|---|---|---|
| q304_M28b *(required)* | q304_M28b. How is the item administered, or given, to the patient?<br>*Question relevant when: selected( ${q104_M28_full_list} , '2')* | 1 Oral - solid (e.g. tablet)<br>2 Oral - liquid<br>3 Sublingual<br>4 Intravenous (IV)<br>5 Intramusculuar injection (IM)<br>6 Subcutaneous<br>7 Rectal<br>8 Inhaled (e.g. gas)<br>9 Inserted (e.g. vaginal pessaries)<br>10 Topical - cream<br>11 Topical - drops (e.g. eye drops)<br>12 Other<br>99 Don't know |
| q304_M28c *(required)* | q304_M28c. What is the item's smallest unit of measurement (mg, mcg, ml, g, etc)?<br>*Question relevant when: selected( ${q104_M28_full_list} , '2')* | microgram — microgram (mcg)<br>miligram — miligram (mg)<br>miligram_dissolved_in_liquid — miligram (mg) dissolved in liquid (e.g. 2 mg per 3 militers)<br>gram — gram (g)<br>militer — militer (ml)<br>centiliter — centiliter (cl)<br>litre — litre (l)<br>ml_of _solution — ml of solution (e.g. ml of 10% solution)<br>litre_of_solution — litre of solution (e.g. 1 litre of 5% solution)<br>cubic_meter — cubic meter (m^3)<br>other — other<br>dont_know — don't know |
| q304_M28c.1 | q304_M28c.1 If other unit of measurement or route of administration, specify here:<br>*Leave blank if not applicable*<br>*Question relevant when: selected( ${q104_M28_full_list} , '2')* | |
| q304_M28d *(required)* | q304_M28d. In total, how many units are used to treat a patient during all of her care?<br>*Remember to add all units! E.g. 5 days x 200 mg twice per day = 2,000mg*<br>*Question relevant when: selected( ${q104_M28_full_list} , '2')*<br>*Response constrained to: .>0* | |
| q304_M29 | ***q304_M29. Anesthetic - other 5: "[q104_M29_full_list_other]"***<br>*Question relevant when: selected( ${q104_M29_full_list} , '2')* | |
| q304_M29a *(required)* | q304_M29a. What percent of patients require this item?<br>*Question relevant when: selected( ${q104_M29_full_list} , '2')*<br>*Response constrained to: .>0 and .<=100 or .=999* | |

| Field | Question | Answer |
|---|---|---|
| q304_M29b *(required)* | q304_M29b. How is the item administered, or given, to the patient?<br>*Question relevant when: selected( ${q104_M29_full_list} , '2')* | 1 Oral - solid (e.g. tablet)<br>2 Oral - liquid<br>3 Sublingual<br>4 Intravenous (IV)<br>5 Intramusculuar injection (IM)<br>6 Subcutaneous<br>7 Rectal<br>8 Inhaled (e.g. gas)<br>9 Inserted (e.g. vaginal pessaries)<br>10 Topical - cream<br>11 Topical - drops (e.g. eye drops)<br>12 Other<br>99 Don't know |
| q304_M29c *(required)* | q304_M29c. What is the item's smallest unit of measurement (mg, mcg, ml, g, etc)?<br>*Question relevant when: selected( ${q104_M29_full_list} , '2')* | microgram — microgram (mcg)<br>miligram — miligram (mg)<br>miligram_dissolved_in_liquid — miligram (mg) dissolved in liquid (e.g. 2 mg per 3 militers)<br>gram — gram (g)<br>militer — militer (ml)<br>centiliter — centiliter (cl)<br>litre — litre (l)<br>ml_of _solution — ml of solution (e.g. ml of 10% solution)<br>litre_of_solution — litre of solution (e.g. 1 litre of 5% solution)<br>cubic_meter — cubic meter (m^3)<br>other — other<br>dont_know — don't know |
| q304_M29c.1 | q304_M29c.1 If other unit of measurement or route of administration, specify here:<br>*Leave blank if not applicable*<br>*Question relevant when: selected( ${q104_M29_full_list} , '2')* | |
| q304_M29d *(required)* | q304_M29d. In total, how many units are used to treat a patient during all of her care?<br>*Remember to add all units! E.g. 5 days x 200 mg twice per day = 2,000mg*<br>*Question relevant when: selected( ${q104_M29_full_list} , '2')*<br>*Response constrained to: .>0* | |
| <p><strong><em>D. Medications - Sepsis (3)</em></strong></p><br>*Group relevant when: selected( ${section_three_skip_med} , '1')* | | |
| q304_med_note3 | 304 CONTINUED For each of the following medications and other medical products that are used for sepsis, can you tell me …? | |
| note_304_antibiotics | ***Antibiotics*** | |
| q304_M30 | ***q304_M30. Amoxicillin***<br>[illegible] | |

| Field | Question | Answer |
|---|---|---|
| | *Question relevant when: selected( ${q104_M30_full_list} , '2')* | |
| q304_M30a *(required)* | q304_M30a. What percent of patients require this item?<br>*Question relevant when: selected( ${q104_M30_full_list} , '2')*<br>*Response constrained to: .>0 and .<=100 or .=999* | |

| Field | Question | Answer |
|---|---|---|
| q304_M30b *(required)* | q304_M30b. How is the item administered, or given, to the patient?<br>*Question relevant when: selected( ${q104_M30_full_list} , '2')* | 1 Oral - solid (e.g. tablet)<br>2 Oral - liquid<br>3 Sublingual<br>4 Intravenous (IV)<br>5 Intramusculuar injection (IM)<br>6 Subcutaneous<br>7 Rectal<br>8 Inhaled (e.g. gas)<br>9 Inserted (e.g. vaginal pessaries)<br>10 Topical - cream<br>11 Topical - drops (e.g. eye drops)<br>12 Other<br>99 Don't know |
| q304_M30c *(required)* | q304_M30c. What is the item's smallest unit of measurement (mg, mcg, ml, g, etc)?<br>*Question relevant when: selected( ${q104_M30_full_list} , '2')* | microgram — microgram (mcg)<br>miligram — miligram (mg)<br>miligram_dissolved_in_liquid — miligram (mg) dissolved in liquid (e.g. 2 mg per 3 militers)<br>gram — gram (g)<br>militer — militer (ml)<br>centiliter — centiliter (cl)<br>litre — litre (l)<br>ml_of _solution — ml of solution (e.g. ml of 10% solution)<br>litre_of_solution — litre of solution (e.g. 1 litre of 5% solution)<br>cubic_meter — cubic meter (m^3)<br>other — other<br>dont_know — don't know |
| q304_M30c.1 | q304_M30c.1 If other unit of measurement or route of administration, specify here:<br>*Leave blank if not applicable*<br>*Question relevant when: selected( ${q104_M30_full_list} , '2')* | |
| q304_M30d *(required)* | q304_M30d. In total, how many units are used to treat a patient during all of her care?<br>*Remember to add all units! E.g. 5 days x 200 mg twice per day = 2,000mg*<br>*Question relevant when: selected( ${q104_M30_full_list} , '2')*<br>*Response constrained to: .>0* | |
| q304_M31 | ***q304_M31. Amoxicillin + Clauvulenic Acid 635mg***<br>*Question relevant when: selected( ${q104_M31_full_list} , '2')* | |
| q304_M31a *(required)* | q304_M31a. What percent of patients require this item?<br>*Question relevant when: selected( ${q104_M31_full_list} , '2')*<br>*Response constrained to: .>0 and .<=100 or .=999* | |

| Field | Question | Answer |
|---|---|---|
| q304_M31b *(required)* | q304_M31b. How is the item administered, or given, to the patient?<br>*Question relevant when: selected( ${q104_M31_full_list} , '2')* | 1 Oral - solid (e.g. tablet)<br>2 Oral - liquid<br>3 Sublingual<br>4 Intravenous (IV)<br>5 Intramusculuar injection (IM)<br>6 Subcutaneous<br>7 Rectal<br>8 Inhaled (e.g. gas)<br>9 Inserted (e.g. vaginal pessaries)<br>10 Topical - cream<br>11 Topical - drops (e.g. eye drops)<br>12 Other<br>99 Don't know |
| q304_M31c *(required)* | q304_M31c. What is the item's smallest unit of measurement (mg, mcg, ml, g, etc)?<br>*Question relevant when: selected( ${q104_M31_full_list} , '2')* | microgram: microgram (mcg)<br>miligram: miligram (mg)<br>miligram_dissolved_in_liquid: miligram (mg) dissolved in liquid (e.g. 2 mg per 3 militers)<br>gram: gram (g)<br>militer: militer (ml)<br>centiliter: centiliter (cl)<br>litre: litre (l)<br>ml_of _solution: ml of solution (e.g. ml of 10% solution)<br>litre_of_solution: litre of solution (e.g. 1 litre of 5% solution)<br>cubic_meter: cubic meter (m^3)<br>other: other<br>dont_know: don't know |
| q304_M31c.1 | q304_M31c.1 If other unit of measurement or route of administration, specify here:<br>*Leave blank if not applicable*<br>*Question relevant when: selected( ${q104_M31_full_list} , '2')* | |
| q304_M31d *(required)* | q304_M31d. In total, how many units are used to treat a patient during all of her care?<br>*Remember to add all units! E.g. 5 days x 200 mg twice per day = 2,000mg*<br>*Question relevant when: selected( ${q104_M31_full_list} , '2')*<br>*Response constrained to: .>0* | |
| q304_M32 | ***q304_M32. Ampicillin***<br>*Question relevant when: selected( ${q104_M32_full_list} , '2')* | |
| q304_M32a *(required)* | q304_M32a. What percent of patients require this item?<br>*Question relevant when: selected( ${q104_M32_full_list} , '2')*<br>*Response constrained to: .>0 and .<=100 or .=999* | |

| Field | Question | Answer |
|---|---|---|
| q304_M32b *(required)* | q304_M32b. How is the item administered, or given, to the patient?<br>*Question relevant when: selected( ${q104_M32_full_list} , '2')* | 1 Oral - solid (e.g. tablet)<br>2 Oral - liquid<br>3 Sublingual<br>4 Intravenous (IV)<br>5 Intramusculuar injection (IM)<br>6 Subcutaneous<br>7 Rectal<br>8 Inhaled (e.g. gas)<br>9 Inserted (e.g. vaginal pessaries)<br>10 Topical - cream<br>11 Topical - drops (e.g. eye drops)<br>12 Other<br>99 Don't know |
| q304_M32c *(required)* | q304_M32c. What is the item's smallest unit of measurement (mg, mcg, ml, g, etc)?<br>*Question relevant when: selected( ${q104_M32_full_list} , '2')* | microgram — microgram (mcg)<br>miligram — miligram (mg)<br>miligram_dissolved_in_liquid — miligram (mg) dissolved in liquid (e.g. 2 mg per 3 militers)<br>gram — gram (g)<br>militer — militer (ml)<br>centiliter — centiliter (cl)<br>litre — litre (l)<br>ml_of _solution — ml of solution (e.g. ml of 10% solution)<br>litre_of_solution — litre of solution (e.g. 1 litre of 5% solution)<br>cubic_meter — cubic meter (m^3)<br>other — other<br>dont_know — don't know |
| q304_M32c.1 | q304_M32c.1 If other unit of measurement or route of administration, specify here:<br>*Leave blank if not applicable*<br>*Question relevant when: selected( ${q104_M32_full_list} , '2')* | |
| q304_M32d *(required)* | q304_M32d. In total, how many units are used to treat a patient during all of her care?<br>*Remember to add all units! E.g. 5 days x 200 mg twice per day = 2,000mg*<br>*Question relevant when: selected( ${q104_M32_full_list} , '2')*<br>*Response constrained to: .>0* | |
| q304_M33 | ***q304_M33. Ampicillin (injection)***<br>*Question relevant when: selected( ${q104_M33_full_list} , '2')* | |
| q304_M33a *(required)* | q304_M33a. What percent of patients require this item?<br>*Question relevant when: selected( ${q104_M33_full_list} , '2')*<br>*Response constrained to: .>0 and .<=100 or .=999* | |

| Field | Question | Answer |
|---|---|---|
| q304_M33b *(required)* | q304_M33b. How is the item administered, or given, to the patient?<br>*Question relevant when: selected( ${q104_M33_full_list} , '2')* | 1 Oral - solid (e.g. tablet)<br>2 Oral - liquid<br>3 Sublingual<br>4 Intravenous (IV)<br>5 Intramusculuar injection (IM)<br>6 Subcutaneous<br>7 Rectal<br>8 Inhaled (e.g. gas)<br>9 Inserted (e.g. vaginal pessaries)<br>10 Topical - cream<br>11 Topical - drops (e.g. eye drops)<br>12 Other<br>99 Don't know |
| q304_M33c *(required)* | q304_M33c. What is the item's smallest unit of measurement (mg, mcg, ml, g, etc)?<br>*Question relevant when: selected( ${q104_M33_full_list} , '2')* | microgram — microgram (mcg)<br>miligram — miligram (mg)<br>miligram_dissolved_in_liquid — miligram (mg) dissolved in liquid (e.g. 2 mg per 3 militers)<br>gram — gram (g)<br>militer — militer (ml)<br>centiliter — centiliter (cl)<br>litre — litre (l)<br>ml_of _solution — ml of solution (e.g. ml of 10% solution)<br>litre_of_solution — litre of solution (e.g. 1 litre of 5% solution)<br>cubic_meter — cubic meter (m^3)<br>other — other<br>dont_know — don't know |
| q304_M33c.1 | q304_M33c.1 If other unit of measurement or route of administration, specify here:<br>*Leave blank if not applicable*<br>*Question relevant when: selected( ${q104_M33_full_list} , '2')* | |
| q304_M33d *(required)* | q304_M33d. In total, how many units are used to treat a patient during all of her care?<br>*Remember to add all units! E.g. 5 days x 200 mg twice per day = 2,000mg*<br>*Question relevant when: selected( ${q104_M33_full_list} , '2')*<br>*Response constrained to: .>0* | |
| q304_M34 | ***q304_M34. Ampicillin/sulbactam (Unasyn)***<br>*Question relevant when: selected( ${q104_M34_full_list} , '2')* | |
| q304_M34a *(required)* | q304_M34a. What percent of patients require this item?<br>*Question relevant when: selected( ${q104_M34_full_list} , '2')*<br>*Response constrained to: .>0 and .<=100 or .=999* | |

| Field | Question | Answer |
|---|---|---|
| q304_M34b *(required)* | q304_M34b. How is the item administered, or given, to the patient?<br>*Question relevant when: selected( ${q104_M34_full_list} , '2')* | 1 Oral - solid (e.g. tablet)<br>2 Oral - liquid<br>3 Sublingual<br>4 Intravenous (IV)<br>5 Intramusculuar injection (IM)<br>6 Subcutaneous<br>7 Rectal<br>8 Inhaled (e.g. gas)<br>9 Inserted (e.g. vaginal pessaries)<br>10 Topical - cream<br>11 Topical - drops (e.g. eye drops)<br>12 Other<br>99 Don't know |
| q304_M34c *(required)* | q304_M34c. What is the item's smallest unit of measurement (mg, mcg, ml, g, etc)?<br>*Question relevant when: selected( ${q104_M34_full_list} , '2')* | microgram — microgram (mcg)<br>miligram — miligram (mg)<br>miligram_dissolved_in_liquid — miligram (mg) dissolved in liquid (e.g. 2 mg per 3 militers)<br>gram — gram (g)<br>militer — militer (ml)<br>centiliter — centiliter (cl)<br>litre — litre (l)<br>ml_of _solution — ml of solution (e.g. ml of 10% solution)<br>litre_of_solution — litre of solution (e.g. 1 litre of 5% solution)<br>cubic_meter — cubic meter (m^3)<br>other — other<br>dont_know — don't know |
| q304_M34c.1 | q304_M34c.1 If other unit of measurement or route of administration, specify here:<br>*Leave blank if not applicable*<br>*Question relevant when: selected( ${q104_M34_full_list} , '2')* | |
| q304_M34d *(required)* | q304_M34d. In total, how many units are used to treat a patient during all of her care?<br>*Remember to add all units! E.g. 5 days x 200 mg twice per day = 2,000mg*<br>*Question relevant when: selected( ${q104_M34_full_list} , '2')*<br>*Response constrained to: .>0* | |
| q304_M35 | ***q304_M35. Ceftriaxone***<br>*Question relevant when: selected( ${q104_M35_full_list} , '2')* | |
| q304_M35a *(required)* | q304_M35a. What percent of patients require this item?<br>*Question relevant when: selected( ${q104_M35_full_list} , '2')*<br>*Response constrained to: .>0 and .<=100 or .=999* | |

| Field | Question | Answer |
|---|---|---|
| q304_M35b *(required)* | q304_M35b. How is the item administered, or given, to the patient?<br>*Question relevant when: selected( ${q104_M35_full_list} , '2')* | 1 Oral - solid (e.g. tablet)<br>2 Oral - liquid<br>3 Sublingual<br>4 Intravenous (IV)<br>5 Intramusculuar injection (IM)<br>6 Subcutaneous<br>7 Rectal<br>8 Inhaled (e.g. gas)<br>9 Inserted (e.g. vaginal pessaries)<br>10 Topical - cream<br>11 Topical - drops (e.g. eye drops)<br>12 Other<br>99 Don't know |
| q304_M35c *(required)* | q304_M35c. What is the item's smallest unit of measurement (mg, mcg, ml, g, etc)?<br>*Question relevant when: selected( ${q104_M35_full_list} , '2')* | microgram — microgram (mcg)<br>miligram — miligram (mg)<br>miligram_dissolved_in_liquid — miligram (mg) dissolved in liquid (e.g. 2 mg per 3 militers)<br>gram — gram (g)<br>militer — militer (ml)<br>centiliter — centiliter (cl)<br>litre — litre (l)<br>ml_of _solution — ml of solution (e.g. ml of 10% solution)<br>litre_of_solution — litre of solution (e.g. 1 litre of 5% solution)<br>cubic_meter — cubic meter (m^3)<br>other — other<br>dont_know — don't know |
| q304_M35c.1 | q304_M35c.1 If other unit of measurement or route of administration, specify here:<br>*Leave blank if not applicable*<br>*Question relevant when: selected( ${q104_M35_full_list} , '2')* | |
| q304_M35d *(required)* | q304_M35d. In total, how many units are used to treat a patient during all of her care?<br>*Remember to add all units! E.g. 5 days x 200 mg twice per day = 2,000mg*<br>*Question relevant when: selected( ${q104_M35_full_list} , '2')*<br>*Response constrained to: .>0* | |
| q304_M36 | ***q304_M36. Chloramphenicol Sodium Succinate (injection)***<br>*Question relevant when: selected( ${q104_M36_full_list} , '2')* | |
| q304_M36a *(required)* | q304_M36a. What percent of patients require this item?<br>*Question relevant when: selected( ${q104_M36_full_list} , '2')*<br>*Response constrained to: .>0 and .<=100 or .=999* | |

| Field | Question | Answer |
|---|---|---|
| q304_M36b *(required)* | q304_M36b. How is the item administered, or given, to the patient? *Question relevant when: selected( ${q104_M36_full_list} , '2')* | 1 Oral - solid (e.g. tablet)<br>2 Oral - liquid<br>3 Sublingual<br>4 Intravenous (IV)<br>5 Intramusculuar injection (IM)<br>6 Subcutaneous<br>7 Rectal<br>8 Inhaled (e.g. gas)<br>9 Inserted (e.g. vaginal pessaries)<br>10 Topical - cream<br>11 Topical - drops (e.g. eye drops)<br>12 Other<br>99 Don't know |
| q304_M36c *(required)* | q304_M36c. What is the item's smallest unit of measurement (mg, mcg, ml, g, etc)? *Question relevant when: selected( ${q104_M36_full_list} , '2')* | microgram — microgram (mcg)<br>miligram — miligram (mg)<br>miligram_dissolved_in_liquid — miligram (mg) dissolved in liquid (e.g. 2 mg per 3 militers)<br>gram — gram (g)<br>militer — militer (ml)<br>centiliter — centiliter (cl)<br>litre — litre (l)<br>ml_of _solution — ml of solution (e.g. ml of 10% solution)<br>litre_of_solution — litre of solution (e.g. 1 litre of 5% solution)<br>cubic_meter — cubic meter (m^3)<br>other — other<br>dont_know — don't know |
| q304_M36c.1 | q304_M36c.1 If other unit of measurement or route of administration, specify here: *Leave blank if not applicable* *Question relevant when: selected( ${q104_M36_full_list} , '2')* | |
| q304_M36d *(required)* | q304_M36d. In total, how many units are used to treat a patient during all of her care? *Remember to add all units! E.g. 5 days x 200 mg twice per day = 2,000mg* *Question relevant when: selected( ${q104_M36_full_list} , '2')* *Response constrained to: .>0* | |
| q304_M37 | ***q304_M37. Ciprofloxacin*** *Question relevant when: selected( ${q104_M37_full_list} , '2')* | |
| q304_M37a *(required)* | q304_M37a. What percent of patients require this item? *Question relevant when: selected( ${q104_M37_full_list} , '2')* *Response constrained to: .>0 and .<=100 or .=999* | |

| Field | Question | Answer |
|---|---|---|
| q304_M37b *(required)* | q304_M37b. How is the item administered, or given, to the patient?<br>*Question relevant when: selected( ${q104_M37_full_list} , '2')* | 1 Oral - solid (e.g. tablet)<br>2 Oral - liquid<br>3 Sublingual<br>4 Intravenous (IV)<br>5 Intramusculuar injection (IM)<br>6 Subcutaneous<br>7 Rectal<br>8 Inhaled (e.g. gas)<br>9 Inserted (e.g. vaginal pessaries)<br>10 Topical - cream<br>11 Topical - drops (e.g. eye drops)<br>12 Other<br>99 Don't know |
| q304_M37c *(required)* | q304_M37c. What is the item's smallest unit of measurement (mg, mcg, ml, g, etc)?<br>*Question relevant when: selected( ${q104_M37_full_list} , '2')* | microgram — microgram (mcg)<br>miligram — miligram (mg)<br>miligram_dissolved_in_liquid — miligram (mg) dissolved in liquid (e.g. 2 mg per 3 militers)<br>gram — gram (g)<br>militer — militer (ml)<br>centiliter — centiliter (cl)<br>litre — litre (l)<br>ml_of _solution — ml of solution (e.g. ml of 10% solution)<br>litre_of_solution — litre of solution (e.g. 1 litre of 5% solution)<br>cubic_meter — cubic meter (m^3)<br>other — other<br>dont_know — don't know |
| q304_M37c.1 | q304_M37c.1 If other unit of measurement or route of administration, specify here:<br>*Leave blank if not applicable*<br>*Question relevant when: selected( ${q104_M37_full_list} , '2')* | |
| q304_M37d *(required)* | q304_M37d. In total, how many units are used to treat a patient during all of her care?<br>*Remember to add all units! E.g. 5 days x 200 mg twice per day = 2,000mg*<br>*Question relevant when: selected( ${q104_M37_full_list} , '2')*<br>*Response constrained to: .>0* | |
| q304_M38 | ***q304_M38. Doxycycline***<br>*Question relevant when: selected( ${q104_M38_full_list} , '2')* | |
| q304_M38a *(required)* | q304_M38a. What percent of patients require this item?<br>*Question relevant when: selected( ${q104_M38_full_list} , '2')*<br>*Response constrained to: .>0 and .<=100 or .=999* | |

| Field | Question | Answer |
|---|---|---|
| q304_M38b *(required)* | q304_M38b. How is the item administered, or given, to the patient?<br>*Question relevant when: selected( ${q104_M38_full_list} , '2')* | 1 Oral - solid (e.g. tablet)<br>2 Oral - liquid<br>3 Sublingual<br>4 Intravenous (IV)<br>5 Intramusculuar injection (IM)<br>6 Subcutaneous<br>7 Rectal<br>8 Inhaled (e.g. gas)<br>9 Inserted (e.g. vaginal pessaries)<br>10 Topical - cream<br>11 Topical - drops (e.g. eye drops)<br>12 Other<br>99 Don't know |
| q304_M38c *(required)* | q304_M38c. What is the item's smallest unit of measurement (mg, mcg, ml, g, etc)?<br>*Question relevant when: selected( ${q104_M38_full_list} , '2')* | microgram — microgram (mcg)<br>miligram — miligram (mg)<br>miligram_dissolved_in_liquid — miligram (mg) dissolved in liquid (e.g. 2 mg per 3 militers)<br>gram — gram (g)<br>militer — militer (ml)<br>centiliter — centiliter (cl)<br>litre — litre (l)<br>ml_of _solution — ml of solution (e.g. ml of 10% solution)<br>litre_of_solution — litre of solution (e.g. 1 litre of 5% solution)<br>cubic_meter — cubic meter (m^3)<br>other — other<br>dont_know — don't know |
| q304_M38c.1 | q304_M38c.1 If other unit of measurement or route of administration, specify here:<br>*Leave blank if not applicable*<br>*Question relevant when: selected( ${q104_M38_full_list} , '2')* | |
| q304_M38d *(required)* | q304_M38d. In total, how many units are used to treat a patient during all of her care?<br>*Remember to add all units! E.g. 5 days x 200 mg twice per day = 2,000mg*<br>*Question relevant when: selected( ${q104_M38_full_list} , '2')*<br>*Response constrained to: .>0* | |
| q304_M39 | ***q304_M39. Erythromycin***<br>*Question relevant when: selected( ${q104_M39_full_list} , '2')* | |
| q304_M39a *(required)* | q304_M39a. What percent of patients require this item?<br>*Question relevant when: selected( ${q104_M39_full_list} , '2')*<br>*Response constrained to: .>0 and .<=100 or .=999* | |

| Field | Question | Answer |
|---|---|---|
| q304_M39b *(required)* | q304_M39b. How is the item administered, or given, to the patient? *Question relevant when: selected( ${q104_M39_full_list} , '2')* | 1 Oral - solid (e.g. tablet)<br>2 Oral - liquid<br>3 Sublingual<br>4 Intravenous (IV)<br>5 Intramusculuar injection (IM)<br>6 Subcutaneous<br>7 Rectal<br>8 Inhaled (e.g. gas)<br>9 Inserted (e.g. vaginal pessaries)<br>10 Topical - cream<br>11 Topical - drops (e.g. eye drops)<br>12 Other<br>99 Don't know |
| q304_M39c *(required)* | q304_M39c. What is the item's smallest unit of measurement (mg, mcg, ml, g, etc)? *Question relevant when: selected( ${q104_M39_full_list} , '2')* | microgram — microgram (mcg)<br>miligram — miligram (mg)<br>miligram_dissolved_in_liquid — miligram (mg) dissolved in liquid (e.g. 2 mg per 3 militers)<br>gram — gram (g)<br>militer — militer (ml)<br>centiliter — centiliter (cl)<br>litre — litre (l)<br>ml_of _solution — ml of solution (e.g. ml of 10% solution)<br>litre_of_solution — litre of solution (e.g. 1 litre of 5% solution)<br>cubic_meter — cubic meter (m^3)<br>other — other<br>dont_know — don't know |
| q304_M39c.1 | q304_M39c.1 If other unit of measurement or route of administration, specify here: *Leave blank if not applicable* *Question relevant when: selected( ${q104_M39_full_list} , '2')* | |
| q304_M39d *(required)* | q304_M39d. In total, how many units are used to treat a patient during all of her care? *Remember to add all units! E.g. 5 days x 200 mg twice per day = 2,000mg* *Question relevant when: selected( ${q104_M39_full_list} , '2')* *Response constrained to: .>0* | |
| q304_M40 | ***q304_M40. Gentamycin*** *Question relevant when: selected( ${q104_M40_full_list} , '2')* | |
| q304_M40a *(required)* | q304_M40a. What percent of patients require this item? *Question relevant when: selected( ${q104_M40_full_list} , '2')* *Response constrained to: .>0 and .<=100 or .=999* | |

| Field | Question | Answer |
|---|---|---|
| q304_M40b *(required)* | q304_M40b. How is the item administered, or given, to the patient?<br>*Question relevant when: selected( ${q104_M40_full_list} , '2')* | 1 Oral - solid (e.g. tablet)<br>2 Oral - liquid<br>3 Sublingual<br>4 Intravenous (IV)<br>5 Intramusculuar injection (IM)<br>6 Subcutaneous<br>7 Rectal<br>8 Inhaled (e.g. gas)<br>9 Inserted (e.g. vaginal pessaries)<br>10 Topical - cream<br>11 Topical - drops (e.g. eye drops)<br>12 Other<br>99 Don't know |
| q304_M40c *(required)* | q304_M40c. What is the item's smallest unit of measurement (mg, mcg, ml, g, etc)?<br>*Question relevant when: selected( ${q104_M40_full_list} , '2')* | microgram — microgram (mcg)<br>miligram — miligram (mg)<br>miligram_dissolved_in_liquid — miligram (mg) dissolved in liquid (e.g. 2 mg per 3 militers)<br>gram — gram (g)<br>militer — militer (ml)<br>centiliter — centiliter (cl)<br>litre — litre (l)<br>ml_of _solution — ml of solution (e.g. ml of 10% solution)<br>litre_of_solution — litre of solution (e.g. 1 litre of 5% solution)<br>cubic_meter — cubic meter (m^3)<br>other — other<br>dont_know — don't know |
| q304_M40c.1 | q304_M40c.1 If other unit of measurement or route of administration, specify here:<br>*Leave blank if not applicable*<br>*Question relevant when: selected( ${q104_M40_full_list} , '2')* | |
| q304_M40d *(required)* | q304_M40d. In total, how many units are used to treat a patient during all of her care?<br>*Remember to add all units! E.g. 5 days x 200 mg twice per day = 2,000mg*<br>*Question relevant when: selected( ${q104_M40_full_list} , '2')*<br>*Response constrained to: .>0* | |
| q304_M41 | ***q304_M41. Metronidazole (for infusion)***<br>*Question relevant when: selected( ${q104_M41_full_list} , '2')* | |
| q304_M41a *(required)* | q304_M41a. What percent of patients require this item?<br>*Question relevant when: selected( ${q104_M41_full_list} , '2')*<br>*Response constrained to: .>0 and .<=100 or .=999* | |

| Field | Question | Answer |
|---|---|---|
| q304_M41b *(required)* | q304_M41b. How is the item administered, or given, to the patient?<br>*Question relevant when: selected( ${q104_M41_full_list} , '2')* | 1 Oral - solid (e.g. tablet)<br>2 Oral - liquid<br>3 Sublingual<br>4 Intravenous (IV)<br>5 Intramusculuar injection (IM)<br>6 Subcutaneous<br>7 Rectal<br>8 Inhaled (e.g. gas)<br>9 Inserted (e.g. vaginal pessaries)<br>10 Topical - cream<br>11 Topical - drops (e.g. eye drops)<br>12 Other<br>99 Don't know |
| q304_M41c *(required)* | q304_M41c. What is the item's smallest unit of measurement (mg, mcg, ml, g, etc)?<br>*Question relevant when: selected( ${q104_M41_full_list} , '2')* | microgram — microgram (mcg)<br>miligram — miligram (mg)<br>miligram_dissolved_in_liquid — miligram (mg) dissolved in liquid (e.g. 2 mg per 3 militers)<br>gram — gram (g)<br>militer — militer (ml)<br>centiliter — centiliter (cl)<br>litre — litre (l)<br>ml_of _solution — ml of solution (e.g. ml of 10% solution)<br>litre_of_solution — litre of solution (e.g. 1 litre of 5% solution)<br>cubic_meter — cubic meter (m^3)<br>other — other<br>dont_know — don't know |
| q304_M41c.1 | q304_M41c.1 If other unit of measurement or route of administration, specify here:<br>*Leave blank if not applicable*<br>*Question relevant when: selected( ${q104_M41_full_list} , '2')* | |
| q304_M41d *(required)* | q304_M41d. In total, how many units are used to treat a patient during all of her care?<br>*Remember to add all units! E.g. 5 days x 200 mg twice per day = 2,000mg*<br>*Question relevant when: selected( ${q104_M41_full_list} , '2')*<br>*Response constrained to: .>0* | |
| q304_M42 | ***q304_M42. Metronidazole (injection)***<br>*Question relevant when: selected( ${q104_M42_full_list} , '2')* | |
| q304_M42a *(required)* | q304_M42a. What percent of patients require this item?<br>*Question relevant when: selected( ${q104_M42_full_list} , '2')*<br>*Response constrained to: .>0 and .<=100 or .=999* | |

| Field | Question | Answer |
|---|---|---|
| q304_M42b *(required)* | q304_M42b. How is the item administered, or given, to the patient?<br>*Question relevant when: selected( ${q104_M42_full_list} , '2')* | 1 Oral - solid (e.g. tablet)<br>2 Oral - liquid<br>3 Sublingual<br>4 Intravenous (IV)<br>5 Intramusculuar injection (IM)<br>6 Subcutaneous<br>7 Rectal<br>8 Inhaled (e.g. gas)<br>9 Inserted (e.g. vaginal pessaries)<br>10 Topical - cream<br>11 Topical - drops (e.g. eye drops)<br>12 Other<br>99 Don't know |
| q304_M42c *(required)* | q304_M42c. What is the item's smallest unit of measurement (mg, mcg, ml, g, etc)?<br>*Question relevant when: selected( ${q104_M42_full_list} , '2')* | microgram — microgram (mcg)<br>miligram — miligram (mg)<br>miligram_dissolved_in_liquid — miligram (mg) dissolved in liquid (e.g. 2 mg per 3 militers)<br>gram — gram (g)<br>militer — militer (ml)<br>centiliter — centiliter (cl)<br>litre — litre (l)<br>ml_of _solution — ml of solution (e.g. ml of 10% solution)<br>litre_of_solution — litre of solution (e.g. 1 litre of 5% solution)<br>cubic_meter — cubic meter (m^3)<br>other — other<br>dont_know — don't know |
| q304_M42c.1 | q304_M42c.1 If other unit of measurement or route of administration, specify here:<br>*Leave blank if not applicable*<br>*Question relevant when: selected( ${q104_M42_full_list} , '2')* | |
| q304_M42d *(required)* | q304_M42d. In total, how many units are used to treat a patient during all of her care?<br>*Remember to add all units! E.g. 5 days x 200 mg twice per day = 2,000mg*<br>*Question relevant when: selected( ${q104_M42_full_list} , '2')*<br>*Response constrained to: .>0* | |
| q304_M43 | ***q304_M43. Metronidazole (oral)***<br>*Question relevant when: selected( ${q104_M43_full_list} , '2')* | |
| q304_M43a *(required)* | q304_M43a. What percent of patients require this item?<br>*Question relevant when: selected( ${q104_M43_full_list} , '2')*<br>*Response constrained to: .>0 and .<=100 or .=999* | |

| Field | Question | Answer |
|---|---|---|
| q304_M43b *(required)* | q304_M43b. How is the item administered, or given, to the patient?<br>*Question relevant when: selected( ${q104_M43_full_list} , '2')* | 1 Oral - solid (e.g. tablet)<br>2 Oral - liquid<br>3 Sublingual<br>4 Intravenous (IV)<br>5 Intramusculuar injection (IM)<br>6 Subcutaneous<br>7 Rectal<br>8 Inhaled (e.g. gas)<br>9 Inserted (e.g. vaginal pessaries)<br>10 Topical - cream<br>11 Topical - drops (e.g. eye drops)<br>12 Other<br>99 Don't know |
| q304_M43c *(required)* | q304_M43c. What is the item's smallest unit of measurement (mg, mcg, ml, g, etc)?<br>*Question relevant when: selected( ${q104_M43_full_list} , '2')* | microgram: microgram (mcg)<br>miligram: miligram (mg)<br>miligram_dissolved_in_liquid: miligram (mg) dissolved in liquid (e.g. 2 mg per 3 militers)<br>gram: gram (g)<br>militer: militer (ml)<br>centiliter: centiliter (cl)<br>litre: litre (l)<br>ml_of _solution: ml of solution (e.g. ml of 10% solution)<br>litre_of_solution: litre of solution (e.g. 1 litre of 5% solution)<br>cubic_meter: cubic meter (m^3)<br>other: other<br>dont_know: don't know |
| q304_M43c.1 | q304_M43c.1 If other unit of measurement or route of administration, specify here:<br>*Leave blank if not applicable*<br>*Question relevant when: selected( ${q104_M43_full_list} , '2')* | |
| q304_M43d *(required)* | q304_M43d. In total, how many units are used to treat a patient during all of her care?<br>*Remember to add all units! E.g. 5 days x 200 mg twice per day = 2,000mg*<br>*Question relevant when: selected( ${q104_M43_full_list} , '2')*<br>*Response constrained to: .>0* | |
| q304_M44 | ***q304_M44. Penicillin***<br>*Question relevant when: selected( ${q104_M44_full_list} , '2')* | |
| q304_M44a *(required)* | q304_M44a. What percent of patients require this item?<br>*Question relevant when: selected( ${q104_M44_full_list} , '2')*<br>*Response constrained to: .>0 and .<=100 or .=999* | |

| Field | Question | Answer |
|---|---|---|
| q304_M44b *(required)* | q304_M44b. How is the item administered, or given, to the patient?<br>*Question relevant when: selected( ${q104_M44_full_list} , '2')* | 1 Oral - solid (e.g. tablet)<br>2 Oral - liquid<br>3 Sublingual<br>4 Intravenous (IV)<br>5 Intramusculuar injection (IM)<br>6 Subcutaneous<br>7 Rectal<br>8 Inhaled (e.g. gas)<br>9 Inserted (e.g. vaginal pessaries)<br>10 Topical - cream<br>11 Topical - drops (e.g. eye drops)<br>12 Other<br>99 Don't know |
| q304_M44c *(required)* | q304_M44c. What is the item's smallest unit of measurement (mg, mcg, ml, g, etc)?<br>*Question relevant when: selected( ${q104_M44_full_list} , '2')* | microgram — microgram (mcg)<br>miligram — miligram (mg)<br>miligram_dissolved_in_liquid — miligram (mg) dissolved in liquid (e.g. 2 mg per 3 militers)<br>gram — gram (g)<br>militer — militer (ml)<br>centiliter — centiliter (cl)<br>litre — litre (l)<br>ml_of _solution — ml of solution (e.g. ml of 10% solution)<br>litre_of_solution — litre of solution (e.g. 1 litre of 5% solution)<br>cubic_meter — cubic meter (m^3)<br>other — other<br>dont_know — don't know |
| q304_M44c.1 | q304_M44c.1 If other unit of measurement or route of administration, specify here:<br>*Leave blank if not applicable*<br>*Question relevant when: selected( ${q104_M44_full_list} , '2')* | |
| q304_M44d *(required)* | q304_M44d. In total, how many units are used to treat a patient during all of her care?<br>*Remember to add all units! E.g. 5 days x 200 mg twice per day = 2,000mg*<br>*Question relevant when: selected( ${q104_M44_full_list} , '2')*<br>*Response constrained to: .>0* | |
| q304_M45 | ***q304_M45. Antibiotic - other 1: "[q104_M45_full_list_other]"***<br>*Question relevant when: selected( ${q104_M45_full_list} , '2')* | |
| q304_M45a *(required)* | q304_M45a. What percent of patients require this item?<br>*Question relevant when: selected( ${q104_M45_full_list} , '2')*<br>*Response constrained to: .>0 and .<=100 or .=999* | |

| Field | Question | Answer |
|---|---|---|
| q304_M45b *(required)* | q304_M45b. How is the item administered, or given, to the patient? *Question relevant when: selected( ${q104_M45_full_list} , '2')* | 1 Oral - solid (e.g. tablet)<br>2 Oral - liquid<br>3 Sublingual<br>4 Intravenous (IV)<br>5 Intramusculuar injection (IM)<br>6 Subcutaneous<br>7 Rectal<br>8 Inhaled (e.g. gas)<br>9 Inserted (e.g. vaginal pessaries)<br>10 Topical - cream<br>11 Topical - drops (e.g. eye drops)<br>12 Other<br>99 Don't know |
| q304_M45c *(required)* | q304_M45c. What is the item's smallest unit of measurement (mg, mcg, ml, g, etc)? *Question relevant when: selected( ${q104_M45_full_list} , '2')* | microgram — microgram (mcg)<br>miligram — miligram (mg)<br>miligram_dissolved_in_liquid — miligram (mg) dissolved in liquid (e.g. 2 mg per 3 militers)<br>gram — gram (g)<br>militer — militer (ml)<br>centiliter — centiliter (cl)<br>litre — litre (l)<br>ml_of _solution — ml of solution (e.g. ml of 10% solution)<br>litre_of_solution — litre of solution (e.g. 1 litre of 5% solution)<br>cubic_meter — cubic meter (m^3)<br>other — other<br>dont_know — don't know |
| q304_M45c.1 | q304_M45c.1 If other unit of measurement or route of administration, specify here: *Leave blank if not applicable* *Question relevant when: selected( ${q104_M45_full_list} , '2')* | |
| q304_M45d *(required)* | q304_M45d. In total, how many units are used to treat a patient during all of her care? *Remember to add all units! E.g. 5 days x 200 mg twice per day = 2,000mg* *Question relevant when: selected( ${q104_M45_full_list} , '2')* *Response constrained to: .>0* | |
| q304_M46 | ***q304_M46. Antibiotic - other 2: "[q104_M46_full_list_other]"*** *Question relevant when: selected( ${q104_M46_full_list} , '2')* | |
| q304_M46a *(required)* | q304_M46a. What percent of patients require this item? *Question relevant when: selected( ${q104_M46_full_list} , '2')* *Response constrained to: .>0 and .<=100 or .=999* | |

<table>
<tr><th>Field</th><th>Question</th><th>Answer</th></tr>
<tr><td>q304_M46b (required)</td><td>q304_M46b. How is the item administered, or given, to the patient?<br>Question relevant when: selected( ${q104_M46_full_list} , '2')</td><td><table><tr><td></td><td>1</td><td>Oral - solid (e.g. tablet)</td></tr><tr><td></td><td>2</td><td>Oral - liquid</td></tr><tr><td></td><td>3</td><td>Sublingual</td></tr><tr><td></td><td>4</td><td>Intravenous (IV)</td></tr><tr><td></td><td>5</td><td>Intramusculuar injection (IM)</td></tr><tr><td></td><td>6</td><td>Subcutaneous</td></tr><tr><td></td><td>7</td><td>Rectal</td></tr><tr><td></td><td>8</td><td>Inhaled (e.g. gas)</td></tr><tr><td></td><td>9</td><td>Inserted (e.g. vaginal pessaries)</td></tr><tr><td></td><td>10</td><td>Topical - cream</td></tr><tr><td></td><td>11</td><td>Topical - drops (e.g. eye drops)</td></tr><tr><td></td><td>12</td><td>Other</td></tr><tr><td></td><td>99</td><td>Don't know</td></tr></table></td></tr>
<tr><td>q304_M46c (required)</td><td>q304_M46c. What is the item's smallest unit of measurement (mg, mcg, ml, g, etc)?<br>Question relevant when: selected( ${q104_M46_full_list} , '2')</td><td><table><tr><td></td><td>microgram</td><td>microgram (mcg)</td></tr><tr><td></td><td>miligram</td><td>miligram (mg)</td></tr><tr><td></td><td>miligram_dissolved_in_liquid</td><td>miligram (mg) dissolved in liquid (e.g. 2 mg per 3 militers)</td></tr><tr><td></td><td>gram</td><td>gram (g)</td></tr><tr><td></td><td>militer</td><td>militer (ml)</td></tr><tr><td></td><td>centiliter</td><td>centiliter (cl)</td></tr><tr><td></td><td>litre</td><td>litre (l)</td></tr><tr><td></td><td>ml_of _solution</td><td>ml of solution (e.g. ml of 10% solution)</td></tr><tr><td></td><td>litre_of_solution</td><td>litre of solution (e.g. 1 litre of 5% solution)</td></tr><tr><td></td><td>cubic_meter</td><td>cubic meter (m^3)</td></tr><tr><td></td><td>other</td><td>other</td></tr><tr><td></td><td>dont_know</td><td>don't know</td></tr></table></td></tr>
<tr><td>q304_M46c.1</td><td>q304_M46c.1 If other unit of measurement or route of administration, specify here:<br>Leave blank if not applicable<br>Question relevant when: selected( ${q104_M46_full_list} , '2')</td><td></td></tr>
<tr><td>q304_M46d (required)</td><td>q304_M46d. In total, how many units are used to treat a patient during all of her care?<br>Remember to add all units! E.g. 5 days x 200 mg twice per day = 2,000mg<br>Question relevant when: selected( ${q104_M46_full_list} , '2')<br>Response constrained to: .>0</td><td></td></tr>
<tr><td>q304_M47</td><td>q304_M47. Antibiotic - other 3: "[q104_M47_full_list_other]"<br>Question relevant when: selected( ${q104_M47_full_list} , '2')</td><td></td></tr>
<tr><td>q304_M47a (required)</td><td>q304_M47a. What percent of patients require this item?<br>Question relevant when: selected( ${q104_M47_full_list} , '2')<br>Response constrained to: .>0 and .<=100 or .=999</td><td></td></tr>
</table>

| Field | Question | Answer |
|---|---|---|
| q304_M47b *(required)* | q304_M47b. How is the item administered, or given, to the patient?<br>*Question relevant when: selected( ${q104_M47_full_list} , '2')* | 1 Oral - solid (e.g. tablet)<br>2 Oral - liquid<br>3 Sublingual<br>4 Intravenous (IV)<br>5 Intramusculuar injection (IM)<br>6 Subcutaneous<br>7 Rectal<br>8 Inhaled (e.g. gas)<br>9 Inserted (e.g. vaginal pessaries)<br>10 Topical - cream<br>11 Topical - drops (e.g. eye drops)<br>12 Other<br>99 Don't know |
| q304_M47c *(required)* | q304_M47c. What is the item's smallest unit of measurement (mg, mcg, ml, g, etc)?<br>*Question relevant when: selected( ${q104_M47_full_list} , '2')* | microgram: microgram (mcg)<br>miligram: miligram (mg)<br>miligram_dissolved_in_liquid: miligram (mg) dissolved in liquid (e.g. 2 mg per 3 militers)<br>gram: gram (g)<br>militer: militer (ml)<br>centiliter: centiliter (cl)<br>litre: litre (l)<br>ml_of _solution: ml of solution (e.g. ml of 10% solution)<br>litre_of_solution: litre of solution (e.g. 1 litre of 5% solution)<br>cubic_meter: cubic meter (m^3)<br>other: other<br>dont_know: don't know |
| q304_M47c.1 | q304_M47c.1 If other unit of measurement or route of administration, specify here:<br>*Leave blank if not applicable*<br>*Question relevant when: selected( ${q104_M47_full_list} , '2')* | |
| q304_M47d *(required)* | q304_M47d. In total, how many units are used to treat a patient during all of her care?<br>*Remember to add all units! E.g. 5 days x 200 mg twice per day = 2,000mg*<br>*Question relevant when: selected( ${q104_M47_full_list} , '2')*<br>*Response constrained to: .>0* | |
| q304_M48 | ***q304_M48. Antibiotic - other 4: "[q104_M48_full_list_other]"***<br>*Question relevant when: selected( ${q104_M48_full_list} , '2')* | |
| q304_M48a *(required)* | q304_M48a. What percent of patients require this item?<br>*Question relevant when: selected( ${q104_M48_full_list} , '2')*<br>*Response constrained to: .>0 and .<=100 or .=999* | |

| Field | Question | Answer |
|---|---|---|
| q304_M48b *(required)* | q304_M48b. How is the item administered, or given, to the patient?<br>*Question relevant when: selected( ${q104_M48_full_list} , '2')* | 1 Oral - solid (e.g. tablet)<br>2 Oral - liquid<br>3 Sublingual<br>4 Intravenous (IV)<br>5 Intramusculuar injection (IM)<br>6 Subcutaneous<br>7 Rectal<br>8 Inhaled (e.g. gas)<br>9 Inserted (e.g. vaginal pessaries)<br>10 Topical - cream<br>11 Topical - drops (e.g. eye drops)<br>12 Other<br>99 Don't know |
| q304_M48c *(required)* | q304_M48c. What is the item's smallest unit of measurement (mg, mcg, ml, g, etc)?<br>*Question relevant when: selected( ${q104_M48_full_list} , '2')* | microgram — microgram (mcg)<br>miligram — miligram (mg)<br>miligram_dissolved_in_liquid — miligram (mg) dissolved in liquid (e.g. 2 mg per 3 militers)<br>gram — gram (g)<br>militer — militer (ml)<br>centiliter — centiliter (cl)<br>litre — litre (l)<br>ml_of _solution — ml of solution (e.g. ml of 10% solution)<br>litre_of_solution — litre of solution (e.g. 1 litre of 5% solution)<br>cubic_meter — cubic meter (m^3)<br>other — other<br>dont_know — don't know |
| q304_M48c.1 | q304_M48c.1 If other unit of measurement or route of administration, specify here:<br>*Leave blank if not applicable*<br>*Question relevant when: selected( ${q104_M48_full_list} , '2')* | |
| q304_M48d *(required)* | q304_M48d. In total, how many units are used to treat a patient during all of her care?<br>*Remember to add all units! E.g. 5 days x 200 mg twice per day = 2,000mg*<br>*Question relevant when: selected( ${q104_M48_full_list} , '2')*<br>*Response constrained to: .>0* | |
| q304_M49 | ***q304_M49. Antibiotic - other 5: "[q104_M49_full_list_other]"***<br>*Question relevant when: selected( ${q104_M49_full_list} , '2')* | |
| q304_M49a *(required)* | q304_M49a. What percent of patients require this item?<br>*Question relevant when: selected( ${q104_M49_full_list} , '2')*<br>*Response constrained to: .>0 and .<=100 or .=999* | |

| Field | Question | Answer |
|---|---|---|
| q304_M49b *(required)* | q304_M49b. How is the item administered, or given, to the patient?<br>*Question relevant when: selected( ${q104_M49_full_list} , '2')* | 1 Oral - solid (e.g. tablet)<br>2 Oral - liquid<br>3 Sublingual<br>4 Intravenous (IV)<br>5 Intramusculuar injection (IM)<br>6 Subcutaneous<br>7 Rectal<br>8 Inhaled (e.g. gas)<br>9 Inserted (e.g. vaginal pessaries)<br>10 Topical - cream<br>11 Topical - drops (e.g. eye drops)<br>12 Other<br>99 Don't know |
| q304_M49c *(required)* | q304_M49c. What is the item's smallest unit of measurement (mg, mcg, ml, g, etc)?<br>*Question relevant when: selected( ${q104_M49_full_list} , '2')* | microgram — microgram (mcg)<br>miligram — miligram (mg)<br>miligram_dissolved_in_liquid — miligram (mg) dissolved in liquid (e.g. 2 mg per 3 militers)<br>gram — gram (g)<br>militer — militer (ml)<br>centiliter — centiliter (cl)<br>litre — litre (l)<br>ml_of _solution — ml of solution (e.g. ml of 10% solution)<br>litre_of_solution — litre of solution (e.g. 1 litre of 5% solution)<br>cubic_meter — cubic meter (m^3)<br>other — other<br>dont_know — don't know |
| q304_M49c.1 | q304_M49c.1 If other unit of measurement or route of administration, specify here:<br>*Leave blank if not applicable*<br>*Question relevant when: selected( ${q104_M49_full_list} , '2')* | |
| q304_M49d *(required)* | q304_M49d. In total, how many units are used to treat a patient during all of her care?<br>*Remember to add all units! E.g. 5 days x 200 mg twice per day = 2,000mg*<br>*Question relevant when: selected( ${q104_M49_full_list} , '2')*<br>*Response constrained to: .>0* | |
| <p><strong><em>D. Medications - Sepsis (4)</em></strong></p><br>*Group relevant when: selected( ${section_three_skip_med} , '1')* | | |
| q304_med_note4 | 304 CONTINUED For each of the following medications and other medical products that are used for sepsis, can you tell me …? | |
| note_304_antiseptic | ***Antiseptic*** | |
| q304_M50 | ***q304_M50. Cetrimide 15% ("Savlon")*** | |

| | *Question relevant when: selected( ${q104_M50_full_list} , '2')* | |
|---|---|---|

| Field | Question | Answer |
|---|---|---|
| q304_M50a *(required)* | q304_M50a. What percent of patients require this item?<br>*Question relevant when: selected( ${q104_M50_full_list} , '2')*<br>*Response constrained to: .>0 and .<=100 or .=999* | |

| Field | Question | Answer |
|---|---|---|
| q304_M50b *(required)* | q304_M50b. How is the item administered, or given, to the patient?<br>*Question relevant when: selected( ${q104_M50_full_list} , '2')* | 1 Oral - solid (e.g. tablet)<br>2 Oral - liquid<br>3 Sublingual<br>4 Intravenous (IV)<br>5 Intramusculuar injection (IM)<br>6 Subcutaneous<br>7 Rectal<br>8 Inhaled (e.g. gas)<br>9 Inserted (e.g. vaginal pessaries)<br>10 Topical - cream<br>11 Topical - drops (e.g. eye drops)<br>12 Other<br>99 Don't know |
| q304_M50c *(required)* | q304_M50c. What is the item's smallest unit of measurement (mg, mcg, ml, g, etc)?<br>*Question relevant when: selected( ${q104_M50_full_list} , '2')* | microgram — microgram (mcg)<br>miligram — miligram (mg)<br>miligram_dissolved_in_liquid — miligram (mg) dissolved in liquid (e.g. 2 mg per 3 militers)<br>gram — gram (g)<br>militer — militer (ml)<br>centiliter — centiliter (cl)<br>litre — litre (l)<br>ml_of _solution — ml of solution (e.g. ml of 10% solution)<br>litre_of_solution — litre of solution (e.g. 1 litre of 5% solution)<br>cubic_meter — cubic meter (m^3)<br>other — other<br>dont_know — don't know |
| q304_M50c.1 | q304_M50c.1 If other unit of measurement or route of administration, specify here:<br>*Leave blank if not applicable*<br>*Question relevant when: selected( ${q104_M50_full_list} , '2')* | |
| q304_M50d *(required)* | q304_M50d. In total, how many units are used to treat a patient during all of her care?<br>*Remember to add all units! E.g. 5 days x 200 mg twice per day = 2,000mg*<br>*Question relevant when: selected( ${q104_M50_full_list} , '2')*<br>*Response constrained to: .>0* | |
| q304_M51 | ***q304_M51. Povidone-iodine solution, 7.5%***<br>*Question relevant when: selected( ${q104_M51_full_list} , '2')* | |
| q304_M51a *(required)* | q304_M51a. What percent of patients require this item?<br>*Question relevant when: selected( ${q104_M51_full_list} , '2')*<br>*Response constrained to: .>0 and .<=100 or .=999* | |

| Field | Question | Answer |
|---|---|---|
| q304_M51b *(required)* | q304_M51b. How is the item administered, or given, to the patient?<br>*Question relevant when: selected( ${q104_M51_full_list} , '2')* | 1 Oral - solid (e.g. tablet)<br>2 Oral - liquid<br>3 Sublingual<br>4 Intravenous (IV)<br>5 Intramusculuar injection (IM)<br>6 Subcutaneous<br>7 Rectal<br>8 Inhaled (e.g. gas)<br>9 Inserted (e.g. vaginal pessaries)<br>10 Topical - cream<br>11 Topical - drops (e.g. eye drops)<br>12 Other<br>99 Don't know |
| q304_M51c *(required)* | q304_M51c. What is the item's smallest unit of measurement (mg, mcg, ml, g, etc)?<br>*Question relevant when: selected( ${q104_M51_full_list} , '2')* | microgram — microgram (mcg)<br>miligram — miligram (mg)<br>miligram_dissolved_in_liquid — miligram (mg) dissolved in liquid (e.g. 2 mg per 3 militers)<br>gram — gram (g)<br>militer — militer (ml)<br>centiliter — centiliter (cl)<br>litre — litre (l)<br>ml_of _solution — ml of solution (e.g. ml of 10% solution)<br>litre_of_solution — litre of solution (e.g. 1 litre of 5% solution)<br>cubic_meter — cubic meter (m^3)<br>other — other<br>dont_know — don't know |
| q304_M51c.1 | q304_M51c.1 If other unit of measurement or route of administration, specify here:<br>*Leave blank if not applicable*<br>*Question relevant when: selected( ${q104_M51_full_list} , '2')* | |
| q304_M51d *(required)* | q304_M51d. In total, how many units are used to treat a patient during all of her care?<br>*Remember to add all units! E.g. 5 days x 200 mg twice per day = 2,000mg*<br>*Question relevant when: selected( ${q104_M51_full_list} , '2')*<br>*Response constrained to: .>0* | |
| q304_M52 | ***q304_M52. Antiseptic - other 1: "[q104_M52_full_list_other]"***<br>*Question relevant when: selected( ${q104_M52_full_list} , '2')* | |
| q304_M52a *(required)* | q304_M52a. What percent of patients require this item?<br>*Question relevant when: selected( ${q104_M52_full_list} , '2')*<br>*Response constrained to: .>0 and .<=100 or .=999* | |

| Field | Question | Answer |
|---|---|---|
| q304_M52b *(required)* | q304_M52b. How is the item administered, or given, to the patient?<br>*Question relevant when: selected( ${q104_M52_full_list} , '2')* | 1 Oral - solid (e.g. tablet)<br>2 Oral - liquid<br>3 Sublingual<br>4 Intravenous (IV)<br>5 Intramusculuar injection (IM)<br>6 Subcutaneous<br>7 Rectal<br>8 Inhaled (e.g. gas)<br>9 Inserted (e.g. vaginal pessaries)<br>10 Topical - cream<br>11 Topical - drops (e.g. eye drops)<br>12 Other<br>99 Don't know |
| q304_M52c *(required)* | q304_M52c. What is the item's smallest unit of measurement (mg, mcg, ml, g, etc)?<br>*Question relevant when: selected( ${q104_M52_full_list} , '2')* | microgram — microgram (mcg)<br>miligram — miligram (mg)<br>miligram_dissolved_in_liquid — miligram (mg) dissolved in liquid (e.g. 2 mg per 3 militers)<br>gram — gram (g)<br>militer — militer (ml)<br>centiliter — centiliter (cl)<br>litre — litre (l)<br>ml_of _solution — ml of solution (e.g. ml of 10% solution)<br>litre_of_solution — litre of solution (e.g. 1 litre of 5% solution)<br>cubic_meter — cubic meter (m^3)<br>other — other<br>dont_know — don't know |
| q304_M52c.1 | q304_M52c.1 If other unit of measurement or route of administration, specify here:<br>*Leave blank if not applicable*<br>*Question relevant when: selected( ${q104_M52_full_list} , '2')* | |
| q304_M52d *(required)* | q304_M52d. In total, how many units are used to treat a patient during all of her care?<br>*Remember to add all units! E.g. 5 days x 200 mg twice per day = 2,000mg*<br>*Question relevant when: selected( ${q104_M52_full_list} , '2')*<br>*Response constrained to: .>0* | |
| q304_M53 | ***q304_M53. Antiseptic - other 2: "[q104_M53_full_list_other]"***<br>*Question relevant when: selected( ${q104_M53_full_list} , '2')* | |
| q304_M53a *(required)* | q304_M53a. What percent of patients require this item?<br>*Question relevant when: selected( ${q104_M53_full_list} , '2')*<br>*Response constrained to: .>0 and .<=100 or .=999* | |

| Field | Question | Answer |
| --- | --- | --- |
| q304_M53b *(required)* | q304_M53b. How is the item administered, or given, to the patient?<br>*Question relevant when: selected( ${q104_M53_full_list} , '2')* | 1 Oral - solid (e.g. tablet)<br>2 Oral - liquid<br>3 Sublingual<br>4 Intravenous (IV)<br>5 Intramusculuar injection (IM)<br>6 Subcutaneous<br>7 Rectal<br>8 Inhaled (e.g. gas)<br>9 Inserted (e.g. vaginal pessaries)<br>10 Topical - cream<br>11 Topical - drops (e.g. eye drops)<br>12 Other<br>99 Don't know |
| q304_M53c *(required)* | q304_M53c. What is the item's smallest unit of measurement (mg, mcg, ml, g, etc)?<br>*Question relevant when: selected( ${q104_M53_full_list} , '2')* | microgram — microgram (mcg)<br>miligram — miligram (mg)<br>miligram_dissolved_in_liquid — miligram (mg) dissolved in liquid (e.g. 2 mg per 3 militers)<br>gram — gram (g)<br>militer — militer (ml)<br>centiliter — centiliter (cl)<br>litre — litre (l)<br>ml_of _solution — ml of solution (e.g. ml of 10% solution)<br>litre_of_solution — litre of solution (e.g. 1 litre of 5% solution)<br>cubic_meter — cubic meter (m^3)<br>other — other<br>dont_know — don't know |
| q304_M53c.1 | q304_M53c.1 If other unit of measurement or route of administration, specify here:<br>*Leave blank if not applicable*<br>*Question relevant when: selected( ${q104_M53_full_list} , '2')* | |
| q304_M53d *(required)* | q304_M53d. In total, how many units are used to treat a patient during all of her care?<br>*Remember to add all units! E.g. 5 days x 200 mg twice per day = 2,000mg*<br>*Question relevant when: selected( ${q104_M53_full_list} , '2')*<br>*Response constrained to: .>0* | |
| q304_M54 | ***q304_M54. Antiseptic - other 3: "[q104_M54_full_list_other]"***<br>*Question relevant when: selected( ${q104_M54_full_list} , '2')* | |
| q304_M54a *(required)* | q304_M54a. What percent of patients require this item?<br>*Question relevant when: selected( ${q104_M54_full_list} , '2')*<br>*Response constrained to: .>0 and .<=100 or .=999* | |

| Field | Question | Answer |
|---|---|---|
| q304_M54b *(required)* | q304_M54b. How is the item administered, or given, to the patient?<br>*Question relevant when: selected( ${q104_M54_full_list} , '2')* | 1 Oral - solid (e.g. tablet)<br>2 Oral - liquid<br>3 Sublingual<br>4 Intravenous (IV)<br>5 Intramusculuar injection (IM)<br>6 Subcutaneous<br>7 Rectal<br>8 Inhaled (e.g. gas)<br>9 Inserted (e.g. vaginal pessaries)<br>10 Topical - cream<br>11 Topical - drops (e.g. eye drops)<br>12 Other<br>99 Don't know |
| q304_M54c *(required)* | q304_M54c. What is the item's smallest unit of measurement (mg, mcg, ml, g, etc)?<br>*Question relevant when: selected( ${q104_M54_full_list} , '2')* | microgram: microgram (mcg)<br>miligram: miligram (mg)<br>miligram_dissolved_in_liquid: miligram (mg) dissolved in liquid (e.g. 2 mg per 3 militers)<br>gram: gram (g)<br>militer: militer (ml)<br>centiliter: centiliter (cl)<br>litre: litre (l)<br>ml_of _solution: ml of solution (e.g. ml of 10% solution)<br>litre_of_solution: litre of solution (e.g. 1 litre of 5% solution)<br>cubic_meter: cubic meter (m^3)<br>other: other<br>dont_know: don't know |
| q304_M54c.1 | q304_M54c.1 If other unit of measurement or route of administration, specify here:<br>*Leave blank if not applicable*<br>*Question relevant when: selected( ${q104_M54_full_list} , '2')* | |
| q304_M54d *(required)* | q304_M54d. In total, how many units are used to treat a patient during all of her care?<br>*Remember to add all units! E.g. 5 days x 200 mg twice per day = 2,000mg*<br>*Question relevant when: selected( ${q104_M54_full_list} , '2')*<br>*Response constrained to: .>0* | |
| q304_M54.1 | ***q304_M54.1. Antiseptic - other 4: "[q104_M54.1_full_list_other]"***<br>*Question relevant when: selected( ${q104_M54.1_full_list} , '2')* | |
| q304_M54.1a *(required)* | q304_M54.1a. What percent of patients require this item?<br>*Question relevant when: selected( ${q104_M54.1_full_list} , '2')*<br>*Response constrained to: .>0 and .<=100 or .=999* | |

| Field | Question | Answer |
|---|---|---|
| q304_M54.1b *(required)* | q304_M54.1b. How is the item administered, or given, to the patient?<br>*Question relevant when: selected( ${q104_M54.1_full_list} , '2')* | 1 Oral - solid (e.g. tablet)<br>2 Oral - liquid<br>3 Sublingual<br>4 Intravenous (IV)<br>5 Intramusculuar injection (IM)<br>6 Subcutaneous<br>7 Rectal<br>8 Inhaled (e.g. gas)<br>9 Inserted (e.g. vaginal pessaries)<br>10 Topical - cream<br>11 Topical - drops (e.g. eye drops)<br>12 Other<br>99 Don't know |
| q304_M54.1c *(required)* | q304_M54.1c. What is the item's smallest unit of measurement (mg, mcg, ml, g, etc)?<br>*Question relevant when: selected( ${q104_M54.1_full_list} , '2')* | microgram — microgram (mcg)<br>miligram — miligram (mg)<br>miligram_dissolved_in_liquid — miligram (mg) dissolved in liquid (e.g. 2 mg per 3 militers)<br>gram — gram (g)<br>militer — militer (ml)<br>centiliter — centiliter (cl)<br>litre — litre (l)<br>ml_of _solution — ml of solution (e.g. ml of 10% solution)<br>litre_of_solution — litre of solution (e.g. 1 litre of 5% solution)<br>cubic_meter — cubic meter (m^3)<br>other — other<br>dont_know — don't know |
| q304_M54.1c.1 | q304_M54.1c.1 If other unit of measurement or route of administration, specify here:<br>*Leave blank if not applicable*<br>*Question relevant when: selected( ${q104_M54.1_full_list} , '2')* | |
| q304_M54.1d *(required)* | q304_M54.1d. In total, how many units are used to treat a patient during all of her care?<br>*Remember to add all units! E.g. 5 days x 200 mg twice per day = 2,000mg*<br>*Question relevant when: selected( ${q104_M54.1_full_list} , '2')*<br>*Response constrained to: .>0* | |
| q304_M54.2 | ***q304_M54.2. Antiseptic - other 5: "[q104_M54.2_full_list_other]"***<br>*Question relevant when: selected( ${q104_M54.2_full_list} , '2')* | |
| q304_M54.2a *(required)* | q304_M54.2a. What percent of patients require this item?<br>*Question relevant when: selected( ${q104_M54.2_full_list} , '2')*<br>*Response constrained to: .>0 and .<=100 or .=999* | |

| Field | Question | Answer |
|---|---|---|
| q304_M54.2b *(required)* | q304_M54.2b. How is the item administered, or given, to the patient?<br>*Question relevant when: selected( ${q104_M54.2_full_list} , '2')* | 1 Oral - solid (e.g. tablet)<br>2 Oral - liquid<br>3 Sublingual<br>4 Intravenous (IV)<br>5 Intramusculuar injection (IM)<br>6 Subcutaneous<br>7 Rectal<br>8 Inhaled (e.g. gas)<br>9 Inserted (e.g. vaginal pessaries)<br>10 Topical - cream<br>11 Topical - drops (e.g. eye drops)<br>12 Other<br>99 Don't know |
| q304_M54.2c *(required)* | q304_M54.2c. What is the item's smallest unit of measurement (mg, mcg, ml, g, etc)?<br>*Question relevant when: selected( ${q104_M54.2_full_list} , '2')* | microgram — microgram (mcg)<br>miligram — miligram (mg)<br>miligram_dissolved_in_liquid — miligram (mg) dissolved in liquid (e.g. 2 mg per 3 militers)<br>gram — gram (g)<br>militer — militer (ml)<br>centiliter — centiliter (cl)<br>litre — litre (l)<br>ml_of _solution — ml of solution (e.g. ml of 10% solution)<br>litre_of_solution — litre of solution (e.g. 1 litre of 5% solution)<br>cubic_meter — cubic meter (m^3)<br>other — other<br>dont_know — don't know |
| q304_M54.2c.1 | q304_M54.2c.1 If other unit of measurement or route of administration, specify here:<br>*Leave blank if not applicable*<br>*Question relevant when: selected( ${q104_M54.2_full_list} , '2')* | |
| q304_M54.2d *(required)* | q304_M54.2d. In total, how many units are used to treat a patient during all of her care?<br>*Remember to add all units! E.g. 5 days x 200 mg twice per day = 2,000mg*<br>*Question relevant when: selected( ${q104_M54.2_full_list} , '2')*<br>*Response constrained to: .>0* | |
| <p><strong><em>D. Medications - Sepsis (5)</em></strong></p><br>*Group relevant when: selected( ${section_three_skip_med} , '1')* | | |
| q304_med_notE4 | 304 CONTINUED For each of the following medications and other medical products that are used for sepsis, can you tell me …? | |
| note_304_antispas | ***Antispasmodic, anxiolytic, sedative, etc*** | |
| q304_M55 | ***q304_M55. Hyoscine butylbromide / scopolamine butilhisocin (Buscopan )***<br>[illegible] | |

Question relevant when: selected( ${q104_M55_full_list} , '2')

| Field | Question | Answer |
|---|---|---|
| q304_M55a *(required)* | q304_M55a. What percent of patients require this item?<br>*Question relevant when: selected( ${q104_M55_full_list} , '2')*<br>*Response constrained to: .>0 and .<=100 or .=999* | |

| | | |
|---|---|---|
| q304_M55b *(required)* | q304_M55b. How is the item administered, or given, to the patient?<br>*Question relevant when: selected( ${q104_M55_full_list} , '2')* | 1 Oral - solid (e.g. tablet)<br>2 Oral - liquid<br>3 Sublingual<br>4 Intravenous (IV)<br>5 Intramusculuar injection (IM)<br>6 Subcutaneous<br>7 Rectal<br>8 Inhaled (e.g. gas)<br>9 Inserted (e.g. vaginal pessaries)<br>10 Topical - cream<br>11 Topical - drops (e.g. eye drops)<br>12 Other<br>99 Don't know |
| q304_M55c *(required)* | q304_M55c. What is the item's smallest unit of measurement (mg, mcg, ml, g, etc)?<br>*Question relevant when: selected( ${q104_M55_full_list} , '2')* | microgram — microgram (mcg)<br>miligram — miligram (mg)<br>miligram_dissolved_in_liquid — miligram (mg) dissolved in liquid (e.g. 2 mg per 3 militers)<br>gram — gram (g)<br>militer — militer (ml)<br>centiliter — centiliter (cl)<br>litre — litre (l)<br>ml_of _solution — ml of solution (e.g. ml of 10% solution)<br>litre_of_solution — litre of solution (e.g. 1 litre of 5% solution)<br>cubic_meter — cubic meter (m^3)<br>other — other<br>dont_know — don't know |
| q304_M55c.1 | q304_M55c.1 If other unit of measurement or route of administration, specify here:<br>*Leave blank if not applicable*<br>*Question relevant when: selected( ${q104_M55_full_list} , '2')* | |
| q304_M55d *(required)* | q304_M55d. In total, how many units are used to treat a patient during all of her care?<br>*Remember to add all units! E.g. 5 days x 200 mg twice per day = 2,000mg*<br>*Question relevant when: selected( ${q104_M55_full_list} , '2')*<br>*Response constrained to: .>0* | |
| q304_M56 | ***q304_M56. Atropine sulfate (or sulphate)***<br>*Question relevant when: selected( ${q104_M56_full_list} , '2')* | |
| q304_M56a *(required)* | q304_M56a. What percent of patients require this item?<br>*Question relevant when: selected( ${q104_M56_full_list} , '2')*<br>*Response constrained to: .>0 and .<=100 or .=999* | |

| Field | Question | Answer |
|---|---|---|
| q304_M56b *(required)* | q304_M56b. How is the item administered, or given, to the patient?<br>*Question relevant when: selected( ${q104_M56_full_list} , '2')* | 1 Oral - solid (e.g. tablet)<br>2 Oral - liquid<br>3 Sublingual<br>4 Intravenous (IV)<br>5 Intramusculuar injection (IM)<br>6 Subcutaneous<br>7 Rectal<br>8 Inhaled (e.g. gas)<br>9 Inserted (e.g. vaginal pessaries)<br>10 Topical - cream<br>11 Topical - drops (e.g. eye drops)<br>12 Other<br>99 Don't know |
| q304_M56c *(required)* | q304_M56c. What is the item's smallest unit of measurement (mg, mcg, ml, g, etc)?<br>*Question relevant when: selected( ${q104_M56_full_list} , '2')* | microgram — microgram (mcg)<br>miligram — miligram (mg)<br>miligram_dissolved_in_liquid — miligram (mg) dissolved in liquid (e.g. 2 mg per 3 militers)<br>gram — gram (g)<br>militer — militer (ml)<br>centiliter — centiliter (cl)<br>litre — litre (l)<br>ml_of _solution — ml of solution (e.g. ml of 10% solution)<br>litre_of_solution — litre of solution (e.g. 1 litre of 5% solution)<br>cubic_meter — cubic meter (m^3)<br>other — other<br>dont_know — don't know |
| q304_M56c.1 | q304_M56c.1 If other unit of measurement or route of administration, specify here:<br>*Leave blank if not applicable*<br>*Question relevant when: selected( ${q104_M56_full_list} , '2')* | |
| q304_M56d *(required)* | q304_M56d. In total, how many units are used to treat a patient during all of her care?<br>*Remember to add all units! E.g. 5 days x 200 mg twice per day = 2,000mg*<br>*Question relevant when: selected( ${q104_M56_full_list} , '2')*<br>*Response constrained to: .>0* | |
| q304_M57 | ***q304_M57. Papaverine HCl***<br>*Question relevant when: selected( ${q104_M57_full_list} , '2')* | |
| q304_M57a *(required)* | q304_M57a. What percent of patients require this item?<br>*Question relevant when: selected( ${q104_M57_full_list} , '2')*<br>*Response constrained to: .>0 and .<=100 or .=999* | |

| Field | Question | Answer |
|---|---|---|
| q304_M57b *(required)* | q304_M57b. How is the item administered, or given, to the patient? *Question relevant when: selected( ${q104_M57_full_list} , '2')* | 1 Oral - solid (e.g. tablet)<br>2 Oral - liquid<br>3 Sublingual<br>4 Intravenous (IV)<br>5 Intramusculuar injection (IM)<br>6 Subcutaneous<br>7 Rectal<br>8 Inhaled (e.g. gas)<br>9 Inserted (e.g. vaginal pessaries)<br>10 Topical - cream<br>11 Topical - drops (e.g. eye drops)<br>12 Other<br>99 Don't know |
| q304_M57c *(required)* | q304_M57c. What is the item's smallest unit of measurement (mg, mcg, ml, g, etc)? *Question relevant when: selected( ${q104_M57_full_list} , '2')* | microgram — microgram (mcg)<br>miligram — miligram (mg)<br>miligram_dissolved_in_liquid — miligram (mg) dissolved in liquid (e.g. 2 mg per 3 militers)<br>gram — gram (g)<br>militer — militer (ml)<br>centiliter — centiliter (cl)<br>litre — litre (l)<br>ml_of _solution — ml of solution (e.g. ml of 10% solution)<br>litre_of_solution — litre of solution (e.g. 1 litre of 5% solution)<br>cubic_meter — cubic meter (m^3)<br>other — other<br>dont_know — don't know |
| q304_M57c.1 | q304_M57c.1 If other unit of measurement or route of administration, specify here: *Leave blank if not applicable* *Question relevant when: selected( ${q104_M57_full_list} , '2')* | |
| q304_M57d *(required)* | q304_M57d. In total, how many units are used to treat a patient during all of her care? *Remember to add all units! E.g. 5 days x 200 mg twice per day = 2,000mg* *Question relevant when: selected( ${q104_M57_full_list} , '2')* *Response constrained to: .>0* | |
| q304_M58 | ***q304_M58. Diazepam (injection)*** *Question relevant when: selected( ${q104_M58_full_list} , '2')* | |
| q304_M58a *(required)* | q304_M58a. What percent of patients require this item? *Question relevant when: selected( ${q104_M58_full_list} , '2')* *Response constrained to: .>0 and .<=100 or .=999* | |

| Field | Question | Answer |
|---|---|---|
| q304_M58b *(required)* | q304_M58b. How is the item administered, or given, to the patient?<br>*Question relevant when: selected( ${q104_M58_full_list} , '2')* | 1 Oral - solid (e.g. tablet)<br>2 Oral - liquid<br>3 Sublingual<br>4 Intravenous (IV)<br>5 Intramusculuar injection (IM)<br>6 Subcutaneous<br>7 Rectal<br>8 Inhaled (e.g. gas)<br>9 Inserted (e.g. vaginal pessaries)<br>10 Topical - cream<br>11 Topical - drops (e.g. eye drops)<br>12 Other<br>99 Don't know |
| q304_M58c *(required)* | q304_M58c. What is the item's smallest unit of measurement (mg, mcg, ml, g, etc)?<br>*Question relevant when: selected( ${q104_M58_full_list} , '2')* | microgram — microgram (mcg)<br>miligram — miligram (mg)<br>miligram_dissolved_in_liquid — miligram (mg) dissolved in liquid (e.g. 2 mg per 3 militers)<br>gram — gram (g)<br>militer — militer (ml)<br>centiliter — centiliter (cl)<br>litre — litre (l)<br>ml_of _solution — ml of solution (e.g. ml of 10% solution)<br>litre_of_solution — litre of solution (e.g. 1 litre of 5% solution)<br>cubic_meter — cubic meter (m^3)<br>other — other<br>dont_know — don't know |
| q304_M58c.1 | q304_M58c.1 If other unit of measurement or route of administration, specify here:<br>*Leave blank if not applicable*<br>*Question relevant when: selected( ${q104_M58_full_list} , '2')* | |
| q304_M58d *(required)* | q304_M58d. In total, how many units are used to treat a patient during all of her care?<br>*Remember to add all units! E.g. 5 days x 200 mg twice per day = 2,000mg*<br>*Question relevant when: selected( ${q104_M58_full_list} , '2')*<br>*Response constrained to: .>0* | |
| q304_M59 | ***q304_M59. Diazepam (oral)***<br>*Question relevant when: selected( ${q104_M59_full_list} , '2')* | |
| q304_M59a *(required)* | q304_M59a. What percent of patients require this item?<br>*Question relevant when: selected( ${q104_M59_full_list} , '2')*<br>*Response constrained to: .>0 and .<=100 or .=999* | |

| Field | Question | Answer |
|---|---|---|
| q304_M59b *(required)* | q304_M59b. How is the item administered, or given, to the patient?<br>*Question relevant when: selected( ${q104_M59_full_list} , '2')* | 1 Oral - solid (e.g. tablet)<br>2 Oral - liquid<br>3 Sublingual<br>4 Intravenous (IV)<br>5 Intramusculuar injection (IM)<br>6 Subcutaneous<br>7 Rectal<br>8 Inhaled (e.g. gas)<br>9 Inserted (e.g. vaginal pessaries)<br>10 Topical - cream<br>11 Topical - drops (e.g. eye drops)<br>12 Other<br>99 Don't know |
| q304_M59c *(required)* | q304_M59c. What is the item's smallest unit of measurement (mg, mcg, ml, g, etc)?<br>*Question relevant when: selected( ${q104_M59_full_list} , '2')* | microgram — microgram (mcg)<br>miligram — miligram (mg)<br>miligram_dissolved_in_liquid — miligram (mg) dissolved in liquid (e.g. 2 mg per 3 militers)<br>gram — gram (g)<br>militer — militer (ml)<br>centiliter — centiliter (cl)<br>litre — litre (l)<br>ml_of _solution — ml of solution (e.g. ml of 10% solution)<br>litre_of_solution — litre of solution (e.g. 1 litre of 5% solution)<br>cubic_meter — cubic meter (m^3)<br>other — other<br>dont_know — don't know |
| q304_M59c.1 | q304_M59c.1 If other unit of measurement or route of administration, specify here:<br>*Leave blank if not applicable*<br>*Question relevant when: selected( ${q104_M59_full_list} , '2')* | |
| q304_M59d *(required)* | q304_M59d. In total, how many units are used to treat a patient during all of her care?<br>*Remember to add all units! E.g. 5 days x 200 mg twice per day = 2,000mg*<br>*Question relevant when: selected( ${q104_M59_full_list} , '2')*<br>*Response constrained to: .>0* | |
| q304_M60 | ***q304_M60. Antispasmodic, anxiolytic, sedative, etc - Other 1: "[q104_M60_full_list_other]"***<br>*Question relevant when: selected( ${q104_M60_full_list} , '2')* | |
| q304_M60a *(required)* | q304_M60a. What percent of patients require this item?<br>*Question relevant when: selected( ${q104_M60_full_list} , '2')*<br>*Response constrained to: .>0 and .<=100 or .=999* | |

| Field | Question | Answer |
|---|---|---|

| Field | Question | Answer |
|---|---|---|
| q304_M60b *(required)* | q304_M60b. How is the item administered, or given, to the patient?<br>*Question relevant when: selected( ${q104_M60_full_list} , '2')* | 1 Oral - solid (e.g. tablet)<br>2 Oral - liquid<br>3 Sublingual<br>4 Intravenous (IV)<br>5 Intramusculuar injection (IM)<br>6 Subcutaneous<br>7 Rectal<br>8 Inhaled (e.g. gas)<br>9 Inserted (e.g. vaginal pessaries)<br>10 Topical - cream<br>11 Topical - drops (e.g. eye drops)<br>12 Other<br>99 Don't know |
| q304_M60c *(required)* | q304_M60c. What is the item's smallest unit of measurement (mg, mcg, ml, g, etc)?<br>*Question relevant when: selected( ${q104_M60_full_list} , '2')* | microgram — microgram (mcg)<br>miligram — miligram (mg)<br>miligram_dissolved_in_liquid — miligram (mg) dissolved in liquid (e.g. 2 mg per 3 militers)<br>gram — gram (g)<br>militer — militer (ml)<br>centiliter — centiliter (cl)<br>litre — litre (l)<br>ml_of _solution — ml of solution (e.g. ml of 10% solution)<br>litre_of_solution — litre of solution (e.g. 1 litre of 5% solution)<br>cubic_meter — cubic meter (m^3)<br>other — other<br>dont_know — don't know |
| q304_M60c.1 | q304_M60c.1 If other unit of measurement or route of administration, specify here:<br>*Leave blank if not applicable*<br>*Question relevant when: selected( ${q104_M60_full_list} , '2')* | |
| q304_M60d *(required)* | q304_M60d. In total, how many units are used to treat a patient during all of her care?<br>*Remember to add all units! E.g. 5 days x 200 mg twice per day = 2,000mg*<br>*Question relevant when: selected( ${q104_M60_full_list} , '2')*<br>*Response constrained to: .>0* | |
| q304_M61 | ***q304_M61. Antispasmodic, anxiolytic, sedative, etc - Other 2: "[q104_M61_full_list_other]"***<br>*Question relevant when: selected( ${q104_M61_full_list} , '2')* | |
| q304_M61a *(required)* | q304_M61a. What percent of patients require this item?<br>*Question relevant when: selected( ${q104_M61_full_list} , '2')*<br>*Response constrained to: .>0 and .<=100 or .=999* | |

| Field | Question | Answer |
|---|---|---|
| q304_M61b *(required)* | q304_M61b. How is the item administered, or given, to the patient?<br>*Question relevant when: selected( ${q104_M61_full_list} , '2')* | 1 Oral - solid (e.g. tablet)<br>2 Oral - liquid<br>3 Sublingual<br>4 Intravenous (IV)<br>5 Intramusculuar injection (IM)<br>6 Subcutaneous<br>7 Rectal<br>8 Inhaled (e.g. gas)<br>9 Inserted (e.g. vaginal pessaries)<br>10 Topical - cream<br>11 Topical - drops (e.g. eye drops)<br>12 Other<br>99 Don't know |
| q304_M61c *(required)* | q304_M61c. What is the item's smallest unit of measurement (mg, mcg, ml, g, etc)?<br>*Question relevant when: selected( ${q104_M61_full_list} , '2')* | microgram — microgram (mcg)<br>miligram — miligram (mg)<br>miligram_dissolved_in_liquid — miligram (mg) dissolved in liquid (e.g. 2 mg per 3 militers)<br>gram — gram (g)<br>militer — militer (ml)<br>centiliter — centiliter (cl)<br>litre — litre (l)<br>ml_of _solution — ml of solution (e.g. ml of 10% solution)<br>litre_of_solution — litre of solution (e.g. 1 litre of 5% solution)<br>cubic_meter — cubic meter (m^3)<br>other — other<br>dont_know — don't know |
| q304_M61c.1 | q304_M61c.1 If other unit of measurement or route of administration, specify here:<br>*Leave blank if not applicable*<br>*Question relevant when: selected( ${q104_M61_full_list} , '2')* | |
| q304_M61d *(required)* | q304_M61d. In total, how many units are used to treat a patient during all of her care?<br>*Remember to add all units! E.g. 5 days x 200 mg twice per day = 2,000mg*<br>*Question relevant when: selected( ${q104_M61_full_list} , '2')*<br>*Response constrained to: .>0* | |
| q304_M62 | ***q304_M62. Antispasmodic, anxiolytic, sedative, etc - Other 3: "[q104_M62_full_list_other]"***<br>*Question relevant when: selected( ${q104_M62_full_list} , '2')* | |
| q304_M62a *(required)* | q304_M62a. What percent of patients require this item?<br>*Question relevant when: selected( ${q104_M62_full_list} , '2')*<br>*Response constrained to: .>0 and .<=100 or .=999* | |

| Field | Question | Answer |
|---|---|---|
| q304_M62b *(required)* | q304_M62b. How is the item administered, or given, to the patient?<br>*Question relevant when: selected( ${q104_M62_full_list} , '2')* | 1 Oral - solid (e.g. tablet)<br>2 Oral - liquid<br>3 Sublingual<br>4 Intravenous (IV)<br>5 Intramusculuar injection (IM)<br>6 Subcutaneous<br>7 Rectal<br>8 Inhaled (e.g. gas)<br>9 Inserted (e.g. vaginal pessaries)<br>10 Topical - cream<br>11 Topical - drops (e.g. eye drops)<br>12 Other<br>99 Don't know |
| q304_M62c *(required)* | q304_M62c. What is the item's smallest unit of measurement (mg, mcg, ml, g, etc)?<br>*Question relevant when: selected( ${q104_M62_full_list} , '2')* | microgram — microgram (mcg)<br>miligram — miligram (mg)<br>miligram_dissolved_in_liquid — miligram (mg) dissolved in liquid (e.g. 2 mg per 3 militers)<br>gram — gram (g)<br>militer — militer (ml)<br>centiliter — centiliter (cl)<br>litre — litre (l)<br>ml_of _solution — ml of solution (e.g. ml of 10% solution)<br>litre_of_solution — litre of solution (e.g. 1 litre of 5% solution)<br>cubic_meter — cubic meter (m^3)<br>other — other<br>dont_know — don't know |
| q304_M62c.1 | q304_M62c.1 If other unit of measurement or route of administration, specify here:<br>*Leave blank if not applicable*<br>*Question relevant when: selected( ${q104_M62_full_list} , '2')* | |
| q304_M62d *(required)* | q304_M62d. In total, how many units are used to treat a patient during all of her care?<br>*Remember to add all units! E.g. 5 days x 200 mg twice per day = 2,000mg*<br>*Question relevant when: selected( ${q104_M62_full_list} , '2')*<br>*Response constrained to: .>0* | |
| q304_M63 | ***q304_M63. Antispasmodic, anxiolytic, sedative, etc - Other 4: "[q104_M63_full_list_other]"***<br>*Question relevant when: selected( ${q104_M63_full_list} , '2')* | |
| q304_M63a *(required)* | q304_M63a. What percent of patients require this item?<br>*Question relevant when: selected( ${q104_M63_full_list} , '2')*<br>*Response constrained to: .>0 and .<=100 or .=999* | |

| Field | Question | Answer |
|---|---|---|
| q304_M63b *(required)* | q304_M63b. How is the item administered, or given, to the patient?<br>*Question relevant when: selected( ${q104_M63_full_list} , '2')* | 1 Oral - solid (e.g. tablet)<br>2 Oral - liquid<br>3 Sublingual<br>4 Intravenous (IV)<br>5 Intramusculuar injection (IM)<br>6 Subcutaneous<br>7 Rectal<br>8 Inhaled (e.g. gas)<br>9 Inserted (e.g. vaginal pessaries)<br>10 Topical - cream<br>11 Topical - drops (e.g. eye drops)<br>12 Other<br>99 Don't know |
| q304_M63c *(required)* | q304_M63c. What is the item's smallest unit of measurement (mg, mcg, ml, g, etc)?<br>*Question relevant when: selected( ${q104_M63_full_list} , '2')* | microgram — microgram (mcg)<br>miligram — miligram (mg)<br>miligram_dissolved_in_liquid — miligram (mg) dissolved in liquid (e.g. 2 mg per 3 militers)<br>gram — gram (g)<br>militer — militer (ml)<br>centiliter — centiliter (cl)<br>litre — litre (l)<br>ml_of _solution — ml of solution (e.g. ml of 10% solution)<br>litre_of_solution — litre of solution (e.g. 1 litre of 5% solution)<br>cubic_meter — cubic meter (m^3)<br>other — other<br>dont_know — don't know |
| q304_M63c.1 | q304_M63c.1 If other unit of measurement or route of administration, specify here:<br>*Leave blank if not applicable*<br>*Question relevant when: selected( ${q104_M63_full_list} , '2')* | |
| q304_M63d *(required)* | q304_M63d. In total, how many units are used to treat a patient during all of her care?<br>*Remember to add all units! E.g. 5 days x 200 mg twice per day = 2,000mg*<br>*Question relevant when: selected( ${q104_M63_full_list} , '2')*<br>*Response constrained to: .>0* | |
| q304_M64 | ***q304_M64. Antispasmodic, anxiolytic, sedative, etc - Other 5: "[q104_M64_full_list_other]"***<br>*Question relevant when: selected( ${q104_M64_full_list} , '2')* | |
| q304_M64a *(required)* | q304_M64a. What percent of patients require this item?<br>*Question relevant when: selected( ${q104_M64_full_list} , '2')*<br>*Response constrained to: .>0 and .<=100 or .=999* | |

| Field | Question | Answer |
|---|---|---|
| q304_M64b *(required)* | q304_M64b. How is the item administered, or given, to the patient?<br>*Question relevant when: selected( ${q104_M64_full_list} , '2')* | 1 Oral - solid (e.g. tablet)<br>2 Oral - liquid<br>3 Sublingual<br>4 Intravenous (IV)<br>5 Intramusculuar injection (IM)<br>6 Subcutaneous<br>7 Rectal<br>8 Inhaled (e.g. gas)<br>9 Inserted (e.g. vaginal pessaries)<br>10 Topical - cream<br>11 Topical - drops (e.g. eye drops)<br>12 Other<br>99 Don't know |
| q304_M64c *(required)* | q304_M64c. What is the item's smallest unit of measurement (mg, mcg, ml, g, etc)?<br>*Question relevant when: selected( ${q104_M64_full_list} , '2')* | microgram: microgram (mcg)<br>miligram: miligram (mg)<br>miligram_dissolved_in_liquid: miligram (mg) dissolved in liquid (e.g. 2 mg per 3 militers)<br>gram: gram (g)<br>militer: militer (ml)<br>centiliter: centiliter (cl)<br>litre: litre (l)<br>ml_of _solution: ml of solution (e.g. ml of 10% solution)<br>litre_of_solution: litre of solution (e.g. 1 litre of 5% solution)<br>cubic_meter: cubic meter (m^3)<br>other: other<br>dont_know: don't know |
| q304_M64c.1 | q304_M64c.1 If other unit of measurement or route of administration, specify here:<br>*Leave blank if not applicable*<br>*Question relevant when: selected( ${q104_M64_full_list} , '2')* | |
| q304_M64d *(required)* | q304_M64d. In total, how many units are used to treat a patient during all of her care?<br>*Remember to add all units! E.g. 5 days x 200 mg twice per day = 2,000mg*<br>*Question relevant when: selected( ${q104_M64_full_list} , '2')*<br>*Response constrained to: .>0* | |
| <p><strong><em>D. Medications - Sepsis (6)</em></strong></p><br>*Group relevant when: selected( ${section_three_skip_med} , '1')* | | |
| q304_med_notE5 | 304 CONTINUED For each of the following medications and other medical products that are used for sepsis, can you tell me …? | |
| note_304_adrenergic | ***Adrenergic*** | |
| [illegible] | [illegible] | |

| Field | Question | Answer |
|---|---|---|
| q304_M65 | ***q304_M65. Ephedrine HCl*** *Question relevant when: selected( ${q104_M65_full_list} , '2')* | |
| q304_M65a *(required)* | q304_M65a. What percent of patients require this item? *Question relevant when: selected( ${q104_M65_full_list} , '2')* *Response constrained to: .>0 and .<=100 or .=999* | |
| q304_M65b *(required)* | q304_M65b. How is the item administered, or given, to the patient? *Question relevant when: selected( ${q104_M65_full_list} , '2')* | 1 Oral - solid (e.g. tablet)<br>2 Oral - liquid<br>3 Sublingual<br>4 Intravenous (IV)<br>5 Intramusculuar injection (IM)<br>6 Subcutaneous<br>7 Rectal<br>8 Inhaled (e.g. gas)<br>9 Inserted (e.g. vaginal pessaries)<br>10 Topical - cream<br>11 Topical - drops (e.g. eye drops)<br>12 Other<br>99 Don't know |
| q304_M65c *(required)* | q304_M65c. What is the item's smallest unit of measurement (mg, mcg, ml, g, etc)? *Question relevant when: selected( ${q104_M65_full_list} , '2')* | microgram — microgram (mcg)<br>miligram — miligram (mg)<br>miligram_dissolved_in_liquid — miligram (mg) dissolved in liquid (e.g. 2 mg per 3 militers)<br>gram — gram (g)<br>militer — militer (ml)<br>centiliter — centiliter (cl)<br>litre — litre (l)<br>ml_of _solution — ml of solution (e.g. ml of 10% solution)<br>litre_of_solution — litre of solution (e.g. 1 litre of 5% solution)<br>cubic_meter — cubic meter (m^3)<br>other — other<br>dont_know — don't know |
| q304_M65c.1 | q304_M65c.1 If other unit of measurement or route of administration, specify here: *Leave blank if not applicable* *Question relevant when: selected( ${q104_M65_full_list} , '2')* | |
| q304_M65d *(required)* | q304_M65d. In total, how many units are used to treat a patient during all of her care? *Remember to add all units! E.g. 5 days x 200 mg twice per day = 2,000mg* *Question relevant when: selected( ${q104_M65_full_list} , '2')* *Response constrained to: .>0* | |
| q304_M66 | ***q304_M66. Epinephrine*** *Question relevant when: selected( ${q104_M66_full_list} , '2')* | |
| q304_M66a *(required)* | q304_M66a. What percent of patients require this item? *Question relevant when: selected( ${q104_M66_full_list} , '2')* *Response constrained to: .>0 and .<=100 or .=999* | |

| Field | Question | Answer |
|---|---|---|
| q304_M66b *(required)* | q304_M66b. How is the item administered, or given, to the patient? *Question relevant when: selected( ${q104_M66_full_list} , '2')* | 1 Oral - solid (e.g. tablet)<br>2 Oral - liquid<br>3 Sublingual<br>4 Intravenous (IV)<br>5 Intramusculuar injection (IM)<br>6 Subcutaneous<br>7 Rectal<br>8 Inhaled (e.g. gas)<br>9 Inserted (e.g. vaginal pessaries)<br>10 Topical - cream<br>11 Topical - drops (e.g. eye drops)<br>12 Other<br>99 Don't know |
| q304_M66c *(required)* | q304_M66c. What is the item's smallest unit of measurement (mg, mcg, ml, g, etc)? *Question relevant when: selected( ${q104_M66_full_list} , '2')* | microgram — microgram (mcg)<br>miligram — miligram (mg)<br>miligram_dissolved_in_liquid — miligram (mg) dissolved in liquid (e.g. 2 mg per 3 militers)<br>gram — gram (g)<br>militer — militer (ml)<br>centiliter — centiliter (cl)<br>litre — litre (l)<br>ml_of _solution — ml of solution (e.g. ml of 10% solution)<br>litre_of_solution — litre of solution (e.g. 1 litre of 5% solution)<br>cubic_meter — cubic meter (m^3)<br>other — other<br>dont_know — don't know |
| q304_M66c.1 | q304_M66c.1 If other unit of measurement or route of administration, specify here: *Leave blank if not applicable* *Question relevant when: selected( ${q104_M66_full_list} , '2')* | |
| q304_M66d *(required)* | q304_M66d. In total, how many units are used to treat a patient during all of her care? *Remember to add all units! E.g. 5 days x 200 mg twice per day = 2,000mg* *Question relevant when: selected( ${q104_M66_full_list} , '2')* *Response constrained to: .>0* | |
| q304_M67 | ***q304_M67. Adrenergic - other 1: "[q104_M67_full_list_other]"*** *Question relevant when: selected( ${q104_M67_full_list} , '2')* | |
| q304_M67a *(required)* | q304_M67a. What percent of patients require this item? *Question relevant when: selected( ${q104_M67_full_list} , '2')* *Response constrained to: .>0 and .<=100 or .=999* | |

| Field | Question | Answer |
|---|---|---|
| q304_M67b *(required)* | q304_M67b. How is the item administered, or given, to the patient?<br>*Question relevant when: selected( ${q104_M67_full_list} , '2')* | 1 Oral - solid (e.g. tablet)<br>2 Oral - liquid<br>3 Sublingual<br>4 Intravenous (IV)<br>5 Intramusculuar injection (IM)<br>6 Subcutaneous<br>7 Rectal<br>8 Inhaled (e.g. gas)<br>9 Inserted (e.g. vaginal pessaries)<br>10 Topical - cream<br>11 Topical - drops (e.g. eye drops)<br>12 Other<br>99 Don't know |
| q304_M67c *(required)* | q304_M67c. What is the item's smallest unit of measurement (mg, mcg, ml, g, etc)?<br>*Question relevant when: selected( ${q104_M67_full_list} , '2')* | microgram — microgram (mcg)<br>miligram — miligram (mg)<br>miligram_dissolved_in_liquid — miligram (mg) dissolved in liquid (e.g. 2 mg per 3 militers)<br>gram — gram (g)<br>militer — militer (ml)<br>centiliter — centiliter (cl)<br>litre — litre (l)<br>ml_of _solution — ml of solution (e.g. ml of 10% solution)<br>litre_of_solution — litre of solution (e.g. 1 litre of 5% solution)<br>cubic_meter — cubic meter (m^3)<br>other — other<br>dont_know — don't know |
| q304_M67c.1 | q304_M67c.1 If other unit of measurement or route of administration, specify here:<br>*Leave blank if not applicable*<br>*Question relevant when: selected( ${q104_M67_full_list} , '2')* | |
| q304_M67d *(required)* | q304_M67d. In total, how many units are used to treat a patient during all of her care?<br>*Remember to add all units! E.g. 5 days x 200 mg twice per day = 2,000mg*<br>*Question relevant when: selected( ${q104_M67_full_list} , '2')*<br>*Response constrained to: .>0* | |
| q304_M68 | ***q304_M68. Adrenergic - other 2: "[q104_M68_full_list_other]"***<br>*Question relevant when: selected( ${q104_M68_full_list} , '2')* | |
| q304_M68a *(required)* | q304_M68a. What percent of patients require this item?<br>*Question relevant when: selected( ${q104_M68_full_list} , '2')*<br>*Response constrained to: .>0 and .<=100 or .=999* | |

| Field | Question | Answer |
|---|---|---|
| q304_M68b *(required)* | q304_M68b. How is the item administered, or given, to the patient?<br>*Question relevant when: selected( ${q104_M68_full_list} , '2')* | 1 Oral - solid (e.g. tablet)<br>2 Oral - liquid<br>3 Sublingual<br>4 Intravenous (IV)<br>5 Intramusculuar injection (IM)<br>6 Subcutaneous<br>7 Rectal<br>8 Inhaled (e.g. gas)<br>9 Inserted (e.g. vaginal pessaries)<br>10 Topical - cream<br>11 Topical - drops (e.g. eye drops)<br>12 Other<br>99 Don't know |
| q304_M68c *(required)* | q304_M68c. What is the item's smallest unit of measurement (mg, mcg, ml, g, etc)?<br>*Question relevant when: selected( ${q104_M68_full_list} , '2')* | microgram — microgram (mcg)<br>miligram — miligram (mg)<br>miligram_dissolved_in_liquid — miligram (mg) dissolved in liquid (e.g. 2 mg per 3 militers)<br>gram — gram (g)<br>militer — militer (ml)<br>centiliter — centiliter (cl)<br>litre — litre (l)<br>ml_of _solution — ml of solution (e.g. ml of 10% solution)<br>litre_of_solution — litre of solution (e.g. 1 litre of 5% solution)<br>cubic_meter — cubic meter (m^3)<br>other — other<br>dont_know — don't know |
| q304_M68c.1 | q304_M68c.1 If other unit of measurement or route of administration, specify here:<br>*Leave blank if not applicable*<br>*Question relevant when: selected( ${q104_M68_full_list} , '2')* | |
| q304_M68d *(required)* | q304_M68d. In total, how many units are used to treat a patient during all of her care?<br>*Remember to add all units! E.g. 5 days x 200 mg twice per day = 2,000mg*<br>*Question relevant when: selected( ${q104_M68_full_list} , '2')*<br>*Response constrained to: .>0* | |
| q304_M69 | ***q304_M69. Adrenergic - other 3: "[q104_M69_full_list_other]"***<br>*Question relevant when: selected( ${q104_M69_full_list} , '2')* | |
| q304_M69a *(required)* | q304_M69a. What percent of patients require this item?<br>*Question relevant when: selected( ${q104_M69_full_list} , '2')*<br>*Response constrained to: .>0 and .<=100 or .=999* | |

| Field | Question | Answer |
|---|---|---|
| q304_M69b *(required)* | q304_M69b. How is the item administered, or given, to the patient?<br>*Question relevant when: selected( ${q104_M69_full_list} , '2')* | 1 Oral - solid (e.g. tablet)<br>2 Oral - liquid<br>3 Sublingual<br>4 Intravenous (IV)<br>5 Intramusculuar injection (IM)<br>6 Subcutaneous<br>7 Rectal<br>8 Inhaled (e.g. gas)<br>9 Inserted (e.g. vaginal pessaries)<br>10 Topical - cream<br>11 Topical - drops (e.g. eye drops)<br>12 Other<br>99 Don't know |
| q304_M69c *(required)* | q304_M69c. What is the item's smallest unit of measurement (mg, mcg, ml, g, etc)?<br>*Question relevant when: selected( ${q104_M69_full_list} , '2')* | microgram — microgram (mcg)<br>miligram — miligram (mg)<br>miligram_dissolved_in_liquid — miligram (mg) dissolved in liquid (e.g. 2 mg per 3 militers)<br>gram — gram (g)<br>militer — militer (ml)<br>centiliter — centiliter (cl)<br>litre — litre (l)<br>ml_of _solution — ml of solution (e.g. ml of 10% solution)<br>litre_of_solution — litre of solution (e.g. 1 litre of 5% solution)<br>cubic_meter — cubic meter (m^3)<br>other — other<br>dont_know — don't know |
| q304_M69c.1 | q304_M69c.1 If other unit of measurement or route of administration, specify here:<br>*Leave blank if not applicable*<br>*Question relevant when: selected( ${q104_M69_full_list} , '2')* | |
| q304_M69d *(required)* | q304_M69d. In total, how many units are used to treat a patient during all of her care?<br>*Remember to add all units! E.g. 5 days x 200 mg twice per day = 2,000mg*<br>*Question relevant when: selected( ${q104_M69_full_list} , '2')*<br>*Response constrained to: .>0* | |
| q304_M69.1 | ***q304_M69.1. Adrenergic - other 4: "[q104_M69.1_full_list_other]"***<br>*Question relevant when: selected( ${q104_M69.1_full_list} , '2')* | |
| q304_M69.1a *(required)* | q304_M69.1a. What percent of patients require this item?<br>*Question relevant when: selected( ${q104_M69.1_full_list} , '2')*<br>*Response constrained to: .>0 and .<=100 or .=999* | |

| Field | Question | Answer |
|---|---|---|
| q304_M69.1b *(required)* | q304_M69.1b. How is the item administered, or given, to the patient? *Question relevant when: selected( ${q104_M69.1_full_list} , '2')* | 1 Oral - solid (e.g. tablet)<br>2 Oral - liquid<br>3 Sublingual<br>4 Intravenous (IV)<br>5 Intramusculuar injection (IM)<br>6 Subcutaneous<br>7 Rectal<br>8 Inhaled (e.g. gas)<br>9 Inserted (e.g. vaginal pessaries)<br>10 Topical - cream<br>11 Topical - drops (e.g. eye drops)<br>12 Other<br>99 Don't know |
| q304_M69.1c *(required)* | q304_M69.1c. What is the item's smallest unit of measurement (mg, mcg, ml, g, etc)? *Question relevant when: selected( ${q104_M69.1_full_list} , '2')* | microgram — microgram (mcg)<br>miligram — miligram (mg)<br>miligram_dissolved_in_liquid — miligram (mg) dissolved in liquid (e.g. 2 mg per 3 militers)<br>gram — gram (g)<br>militer — militer (ml)<br>centiliter — centiliter (cl)<br>litre — litre (l)<br>ml_of _solution — ml of solution (e.g. ml of 10% solution)<br>litre_of_solution — litre of solution (e.g. 1 litre of 5% solution)<br>cubic_meter — cubic meter (m^3)<br>other — other<br>dont_know — don't know |
| q304_M69.1c.1 | q304_M69.1c.1 If other unit of measurement or route of administration, specify here: *Leave blank if not applicable* *Question relevant when: selected( ${q104_M69.1_full_list} , '2')* | |
| q304_M69.1d *(required)* | q304_M69.1d. In total, how many units are used to treat a patient during all of her care? *Remember to add all units! E.g. 5 days x 200 mg twice per day = 2,000mg* *Question relevant when: selected( ${q104_M69.1_full_list} , '2')* *Response constrained to: .>0* | |
| q304_M69.2 | ***q304_M69.2. Adrenergic - other 5: "[q104_M69.2_full_list_other]"*** *Question relevant when: selected( ${q104_M69.2_full_list} , '2')* | |
| q304_M69.2a *(required)* | q304_M69.2a. What percent of patients require this item? *Question relevant when: selected( ${q104_M69.2_full_list} , '2')* *Response constrained to: .>0 and .<=100 or .=999* | |

| Field | Question | Answer |
|---|---|---|

| Field | Question | Answer |
|---|---|---|
| q304_M69.2b *(required)* | q304_M69.2b. How is the item administered, or given, to the patient?<br>*Question relevant when: selected( ${q104_M69.2_full_list} , '2')* | 1 Oral - solid (e.g. tablet)<br>2 Oral - liquid<br>3 Sublingual<br>4 Intravenous (IV)<br>5 Intramusculuar injection (IM)<br>6 Subcutaneous<br>7 Rectal<br>8 Inhaled (e.g. gas)<br>9 Inserted (e.g. vaginal pessaries)<br>10 Topical - cream<br>11 Topical - drops (e.g. eye drops)<br>12 Other<br>99 Don't know |
| q304_M69.2c *(required)* | q304_M69.2c. What is the item's smallest unit of measurement (mg, mcg, ml, g, etc)?<br>*Question relevant when: selected( ${q104_M69.2_full_list} , '2')* | microgram — microgram (mcg)<br>miligram — miligram (mg)<br>miligram_dissolved_in_liquid — miligram (mg) dissolved in liquid (e.g. 2 mg per 3 militers)<br>gram — gram (g)<br>militer — militer (ml)<br>centiliter — centiliter (cl)<br>litre — litre (l)<br>ml_of _solution — ml of solution (e.g. ml of 10% solution)<br>litre_of_solution — litre of solution (e.g. 1 litre of 5% solution)<br>cubic_meter — cubic meter (m^3)<br>other — other<br>dont_know — don't know |
| q304_M69.2c.1 | q304_M69.2c.1 If other unit of measurement or route of administration, specify here:<br>*Leave blank if not applicable*<br>*Question relevant when: selected( ${q104_M69.2_full_list} , '2')* | |
| q304_M69.2d *(required)* | q304_M69.2d. In total, how many units are used to treat a patient during all of her care?<br>*Remember to add all units! E.g. 5 days x 200 mg twice per day = 2,000mg*<br>*Question relevant when: selected( ${q104_M69.2_full_list} , '2')*<br>*Response constrained to: .>0* | |
| <p><strong><em>D. Medications - Sepsis (7)</em></strong></p><br>*Group relevant when: selected( ${section_three_skip_med} , '1')* | | |
| q304_med_notE6 | 304 CONTINUED For each of the following medications and other medical products that are used for sepsis, can you tell me …? | |
| note_304_blood | ***Blood products*** | |

| Field | Question | Answer |
|---|---|---|
| q304_M70 | ***q304_M70. Unit of packed red blood cells***<br>*Question relevant when: selected( ${q104_M70_full_list} , '2')* | |
| q304_M70a *(required)* | q304_M70a. What percent of patients require this item?<br>*Question relevant when: selected( ${q104_M70_full_list} , '2')*<br>*Response constrained to: .>0 and .<=100 or .=999* | |

| Field | Question | Answer |
|---|---|---|
| q304_M70b *(required)* | q304_M70b. How is the item administered, or given, to the patient?<br>*Question relevant when: selected( ${q104_M70_full_list} , '2')* | 1 Oral - solid (e.g. tablet)<br>2 Oral - liquid<br>3 Sublingual<br>4 Intravenous (IV)<br>5 Intramusculuar injection (IM)<br>6 Subcutaneous<br>7 Rectal<br>8 Inhaled (e.g. gas)<br>9 Inserted (e.g. vaginal pessaries)<br>10 Topical - cream<br>11 Topical - drops (e.g. eye drops)<br>12 Other<br>99 Don't know |
| q304_M70c *(required)* | q304_M70c. What is the item's smallest unit of measurement (mg, mcg, ml, g, etc)?<br>*Question relevant when: selected( ${q104_M70_full_list} , '2')* | microgram — microgram (mcg)<br>miligram — miligram (mg)<br>miligram_dissolved_in_liquid — miligram (mg) dissolved in liquid (e.g. 2 mg per 3 militers)<br>gram — gram (g)<br>militer — militer (ml)<br>centiliter — centiliter (cl)<br>litre — litre (l)<br>ml_of _solution — ml of solution (e.g. ml of 10% solution)<br>litre_of_solution — litre of solution (e.g. 1 litre of 5% solution)<br>cubic_meter — cubic meter (m^3)<br>other — other<br>dont_know — don't know |
| q304_M70c.1 | q304_M70c.1 If other unit of measurement or route of administration, specify here:<br>*Leave blank if not applicable*<br>*Question relevant when: selected( ${q104_M70_full_list} , '2')* | |
| q304_M70d *(required)* | q304_M70d. In total, how many units are used to treat a patient during all of her care?<br>*Remember to add all units! E.g. 5 days x 200 mg twice per day = 2,000mg*<br>*Question relevant when: selected( ${q104_M70_full_list} , '2')*<br>*Response constrained to: .>0* | |
| q304_M71 | ***q304_M71. Unit of platelets***<br>*Question relevant when: selected( ${q104_M71_full_list} , '2')* | |
| q304_M71a *(required)* | q304_M71a. What percent of patients require this item?<br>*Question relevant when: selected( ${q104_M71_full_list} , '2')*<br>*Response constrained to: .>0 and .<=100 or .=999* | |

| Field | Question | Answer |
|---|---|---|
| q304_M71b *(required)* | q304_M71b. How is the item administered, or given, to the patient?<br>*Question relevant when: selected( ${q104_M71_full_list} , '2')* | 1 Oral - solid (e.g. tablet)<br>2 Oral - liquid<br>3 Sublingual<br>4 Intravenous (IV)<br>5 Intramusculuar injection (IM)<br>6 Subcutaneous<br>7 Rectal<br>8 Inhaled (e.g. gas)<br>9 Inserted (e.g. vaginal pessaries)<br>10 Topical - cream<br>11 Topical - drops (e.g. eye drops)<br>12 Other<br>99 Don't know |
| q304_M71c *(required)* | q304_M71c. What is the item's smallest unit of measurement (mg, mcg, ml, g, etc)?<br>*Question relevant when: selected( ${q104_M71_full_list} , '2')* | microgram — microgram (mcg)<br>miligram — miligram (mg)<br>miligram_dissolved_in_liquid — miligram (mg) dissolved in liquid (e.g. 2 mg per 3 militers)<br>gram — gram (g)<br>militer — militer (ml)<br>centiliter — centiliter (cl)<br>litre — litre (l)<br>ml_of _solution — ml of solution (e.g. ml of 10% solution)<br>litre_of_solution — litre of solution (e.g. 1 litre of 5% solution)<br>cubic_meter — cubic meter (m^3)<br>other — other<br>dont_know — don't know |
| q304_M71c.1 | q304_M71c.1 If other unit of measurement or route of administration, specify here:<br>*Leave blank if not applicable*<br>*Question relevant when: selected( ${q104_M71_full_list} , '2')* | |
| q304_M71d *(required)* | q304_M71d. In total, how many units are used to treat a patient during all of her care?<br>*Remember to add all units! E.g. 5 days x 200 mg twice per day = 2,000mg*<br>*Question relevant when: selected( ${q104_M71_full_list} , '2')*<br>*Response constrained to: .>0* | |
| q304_M72 | ***q304_M72. Unit of whole blood***<br>*Question relevant when: selected( ${q104_M72_full_list} , '2')* | |
| q304_M72a *(required)* | q304_M72a. What percent of patients require this item?<br>*Question relevant when: selected( ${q104_M72_full_list} , '2')*<br>*Response constrained to: .>0 and .<=100 or .=999* | |

| Field | Question | Answer |
|---|---|---|
| q304_M72b *(required)* | q304_M72b. How is the item administered, or given, to the patient?<br>*Question relevant when: selected( ${q104_M72_full_list} , '2')* | 1 Oral - solid (e.g. tablet)<br>2 Oral - liquid<br>3 Sublingual<br>4 Intravenous (IV)<br>5 Intramusculuar injection (IM)<br>6 Subcutaneous<br>7 Rectal<br>8 Inhaled (e.g. gas)<br>9 Inserted (e.g. vaginal pessaries)<br>10 Topical - cream<br>11 Topical - drops (e.g. eye drops)<br>12 Other<br>99 Don't know |
| q304_M72c *(required)* | q304_M72c. What is the item's smallest unit of measurement (mg, mcg, ml, g, etc)?<br>*Question relevant when: selected( ${q104_M72_full_list} , '2')* | microgram: microgram (mcg)<br>miligram: miligram (mg)<br>miligram_dissolved_in_liquid: miligram (mg) dissolved in liquid (e.g. 2 mg per 3 militers)<br>gram: gram (g)<br>militer: militer (ml)<br>centiliter: centiliter (cl)<br>litre: litre (l)<br>ml_of _solution: ml of solution (e.g. ml of 10% solution)<br>litre_of_solution: litre of solution (e.g. 1 litre of 5% solution)<br>cubic_meter: cubic meter (m^3)<br>other: other<br>dont_know: don't know |
| q304_M72c.1 | q304_M72c.1 If other unit of measurement or route of administration, specify here:<br>*Leave blank if not applicable*<br>*Question relevant when: selected( ${q104_M72_full_list} , '2')* | |
| q304_M72d *(required)* | q304_M72d. In total, how many units are used to treat a patient during all of her care?<br>*Remember to add all units! E.g. 5 days x 200 mg twice per day = 2,000mg*<br>*Question relevant when: selected( ${q104_M72_full_list} , '2')*<br>*Response constrained to: .>0* | |
| q304_M73 | ***q304_M73. Blood product - other 1: "[q104_M73_full_list_other]"***<br>*Question relevant when: selected( ${q104_M73_full_list} , '2')* | |
| q304_M73a *(required)* | q304_M73a. What percent of patients require this item?<br>*Question relevant when: selected( ${q104_M73_full_list} , '2')*<br>*Response constrained to: .>0 and .<=100 or .=999* | |

| Field | Question | Answer |
|---|---|---|
| q304_M73b *(required)* | q304_M73b. How is the item administered, or given, to the patient?<br>*Question relevant when: selected( ${q104_M73_full_list} , '2')* | 1 Oral - solid (e.g. tablet)<br>2 Oral - liquid<br>3 Sublingual<br>4 Intravenous (IV)<br>5 Intramusculuar injection (IM)<br>6 Subcutaneous<br>7 Rectal<br>8 Inhaled (e.g. gas)<br>9 Inserted (e.g. vaginal pessaries)<br>10 Topical - cream<br>11 Topical - drops (e.g. eye drops)<br>12 Other<br>99 Don't know |
| q304_M73c *(required)* | q304_M73c. What is the item's smallest unit of measurement (mg, mcg, ml, g, etc)?<br>*Question relevant when: selected( ${q104_M73_full_list} , '2')* | microgram: microgram (mcg)<br>miligram: miligram (mg)<br>miligram_dissolved_in_liquid: miligram (mg) dissolved in liquid (e.g. 2 mg per 3 militers)<br>gram: gram (g)<br>militer: militer (ml)<br>centiliter: centiliter (cl)<br>litre: litre (l)<br>ml_of _solution: ml of solution (e.g. ml of 10% solution)<br>litre_of_solution: litre of solution (e.g. 1 litre of 5% solution)<br>cubic_meter: cubic meter (m^3)<br>other: other<br>dont_know: don't know |
| q304_M73c.1 | q304_M73c.1 If other unit of measurement or route of administration, specify here:<br>*Leave blank if not applicable*<br>*Question relevant when: selected( ${q104_M73_full_list} , '2')* | |
| q304_M73d *(required)* | q304_M73d. In total, how many units are used to treat a patient during all of her care?<br>*Remember to add all units! E.g. 5 days x 200 mg twice per day = 2,000mg*<br>*Question relevant when: selected( ${q104_M73_full_list} , '2')*<br>*Response constrained to: .>0* | |
| q304_M74 | ***q304_M74. Blood product - other 2: "[q104_M74_full_list_other]"***<br>*Question relevant when: selected( ${q104_M74_full_list} , '2')* | |
| q304_M74a *(required)* | q304_M74a. What percent of patients require this item?<br>*Question relevant when: selected( ${q104_M74_full_list} , '2')*<br>*Response constrained to: .>0 and .<=100 or .=999* | |

| Field | Question | Answer |
|---|---|---|
| q304_M74b *(required)* | q304_M74b. How is the item administered, or given, to the patient?<br>*Question relevant when: selected( ${q104_M74_full_list} , '2')* | 1 Oral - solid (e.g. tablet)<br>2 Oral - liquid<br>3 Sublingual<br>4 Intravenous (IV)<br>5 Intramusculuar injection (IM)<br>6 Subcutaneous<br>7 Rectal<br>8 Inhaled (e.g. gas)<br>9 Inserted (e.g. vaginal pessaries)<br>10 Topical - cream<br>11 Topical - drops (e.g. eye drops)<br>12 Other<br>99 Don't know |
| q304_M74c *(required)* | q304_M74c. What is the item's smallest unit of measurement (mg, mcg, ml, g, etc)?<br>*Question relevant when: selected( ${q104_M74_full_list} , '2')* | microgram — microgram (mcg)<br>miligram — miligram (mg)<br>miligram_dissolved_in_liquid — miligram (mg) dissolved in liquid (e.g. 2 mg per 3 militers)<br>gram — gram (g)<br>militer — militer (ml)<br>centiliter — centiliter (cl)<br>litre — litre (l)<br>ml_of _solution — ml of solution (e.g. ml of 10% solution)<br>litre_of_solution — litre of solution (e.g. 1 litre of 5% solution)<br>cubic_meter — cubic meter (m^3)<br>other — other<br>dont_know — don't know |
| q304_M74c.1 | q304_M74c.1 If other unit of measurement or route of administration, specify here:<br>*Leave blank if not applicable*<br>*Question relevant when: selected( ${q104_M74_full_list} , '2')* | |
| q304_M74d *(required)* | q304_M74d. In total, how many units are used to treat a patient during all of her care?<br>*Remember to add all units! E.g. 5 days x 200 mg twice per day = 2,000mg*<br>*Question relevant when: selected( ${q104_M74_full_list} , '2')*<br>*Response constrained to: .>0* | |
| q304_M75 | ***q304_M75. Blood product - other 3: "[q104_M75_full_list_other]"***<br>*Question relevant when: selected( ${q104_M75_full_list} , '2')* | |
| q304_M75a *(required)* | q304_M75a. What percent of patients require this item?<br>*Question relevant when: selected( ${q104_M75_full_list} , '2')*<br>*Response constrained to: .>0 and .<=100 or .=999* | |

| Field | Question | Answer |
|---|---|---|
| q304_M75b *(required)* | q304_M75b. How is the item administered, or given, to the patient?<br>*Question relevant when: selected( ${q104_M75_full_list} , '2')* | 1 Oral - solid (e.g. tablet)<br>2 Oral - liquid<br>3 Sublingual<br>4 Intravenous (IV)<br>5 Intramusculuar injection (IM)<br>6 Subcutaneous<br>7 Rectal<br>8 Inhaled (e.g. gas)<br>9 Inserted (e.g. vaginal pessaries)<br>10 Topical - cream<br>11 Topical - drops (e.g. eye drops)<br>12 Other<br>99 Don't know |
| q304_M75c *(required)* | q304_M75c. What is the item's smallest unit of measurement (mg, mcg, ml, g, etc)?<br>*Question relevant when: selected( ${q104_M75_full_list} , '2')* | microgram — microgram (mcg)<br>miligram — miligram (mg)<br>miligram_dissolved_in_liquid — miligram (mg) dissolved in liquid (e.g. 2 mg per 3 militers)<br>gram — gram (g)<br>militer — militer (ml)<br>centiliter — centiliter (cl)<br>litre — litre (l)<br>ml_of _solution — ml of solution (e.g. ml of 10% solution)<br>litre_of_solution — litre of solution (e.g. 1 litre of 5% solution)<br>cubic_meter — cubic meter (m^3)<br>other — other<br>dont_know — don't know |
| q304_M75c.1 | q304_M75c.1 If other unit of measurement or route of administration, specify here:<br>*Leave blank if not applicable*<br>*Question relevant when: selected( ${q104_M75_full_list} , '2')* | |
| q304_M75d *(required)* | q304_M75d. In total, how many units are used to treat a patient during all of her care?<br>*Remember to add all units! E.g. 5 days x 200 mg twice per day = 2,000mg*<br>*Question relevant when: selected( ${q104_M75_full_list} , '2')*<br>*Response constrained to: .>0* | |
| q304_M75.1 | ***q304_M75.1. Blood product - other 4: "[q104_M75.1_full_list_other]"***<br>*Question relevant when: selected( ${q104_M75.1_full_list} , '2')* | |
| q304_M75.1a *(required)* | q304_M75.1a. What percent of patients require this item?<br>*Question relevant when: selected( ${q104_M75.1_full_list} , '2')*<br>*Response constrained to: .>0 and .<=100 or .=999* | |

| Field | Question | Answer |
|---|---|---|
| q304_M75.1b *(required)* | q304_M75.1b. How is the item administered, or given, to the patient?<br>*Question relevant when: selected( ${q104_M75.1_full_list} , '2')* | 1 Oral - solid (e.g. tablet)<br>2 Oral - liquid<br>3 Sublingual<br>4 Intravenous (IV)<br>5 Intramusculuar injection (IM)<br>6 Subcutaneous<br>7 Rectal<br>8 Inhaled (e.g. gas)<br>9 Inserted (e.g. vaginal pessaries)<br>10 Topical - cream<br>11 Topical - drops (e.g. eye drops)<br>12 Other<br>99 Don't know |
| q304_M75.1c *(required)* | q304_M75.1c. What is the item's smallest unit of measurement (mg, mcg, ml, g, etc)?<br>*Question relevant when: selected( ${q104_M75.1_full_list} , '2')* | microgram — microgram (mcg)<br>miligram — miligram (mg)<br>miligram_dissolved_in_liquid — miligram (mg) dissolved in liquid (e.g. 2 mg per 3 militers)<br>gram — gram (g)<br>militer — militer (ml)<br>centiliter — centiliter (cl)<br>litre — litre (l)<br>ml_of _solution — ml of solution (e.g. ml of 10% solution)<br>litre_of_solution — litre of solution (e.g. 1 litre of 5% solution)<br>cubic_meter — cubic meter (m^3)<br>other — other<br>dont_know — don't know |
| q304_M75.1c.1 | q304_M75.1c.1 If other unit of measurement or route of administration, specify here:<br>*Leave blank if not applicable*<br>*Question relevant when: selected( ${q104_M75.1_full_list} , '2')* | |
| q304_M75.1d *(required)* | q304_M75.1d. In total, how many units are used to treat a patient during all of her care?<br>*Remember to add all units! E.g. 5 days x 200 mg twice per day = 2,000mg*<br>*Question relevant when: selected( ${q104_M75.1_full_list} , '2')*<br>*Response constrained to: .>0* | |
| q304_M75.2 | ***q304_M75.2. Blood product - other 5: "[q104_M75.2_full_list_other]"***<br>*Question relevant when: selected( ${q104_M75.2_full_list} , '2')* | |
| q304_M75.2a *(required)* | q304_M75.2a. What percent of patients require this item?<br>*Question relevant when: selected( ${q104_M75.2_full_list} , '2')*<br>*Response constrained to: .>0 and .<=100 or .=999* | |

| Field | Question | Answer |
|---|---|---|
| q304_M75.2b *(required)* | q304_M75.2b. How is the item administered, or given, to the patient?<br>*Question relevant when: selected( ${q104_M75.2_full_list} , '2')* | 1 Oral - solid (e.g. tablet)<br>2 Oral - liquid<br>3 Sublingual<br>4 Intravenous (IV)<br>5 Intramusculuar injection (IM)<br>6 Subcutaneous<br>7 Rectal<br>8 Inhaled (e.g. gas)<br>9 Inserted (e.g. vaginal pessaries)<br>10 Topical - cream<br>11 Topical - drops (e.g. eye drops)<br>12 Other<br>99 Don't know |
| q304_M75.2c *(required)* | q304_M75.2c. What is the item's smallest unit of measurement (mg, mcg, ml, g, etc)?<br>*Question relevant when: selected( ${q104_M75.2_full_list} , '2')* | microgram — microgram (mcg)<br>miligram — miligram (mg)<br>miligram_dissolved_in_liquid — miligram (mg) dissolved in liquid (e.g. 2 mg per 3 militers)<br>gram — gram (g)<br>militer — militer (ml)<br>centiliter — centiliter (cl)<br>litre — litre (l)<br>ml_of _solution — ml of solution (e.g. ml of 10% solution)<br>litre_of_solution — litre of solution (e.g. 1 litre of 5% solution)<br>cubic_meter — cubic meter (m^3)<br>other — other<br>dont_know — don't know |
| q304_M75.2c.1 | q304_M75.2c.1 If other unit of measurement or route of administration, specify here:<br>*Leave blank if not applicable*<br>*Question relevant when: selected( ${q104_M75.2_full_list} , '2')* | |
| q304_M75.2d *(required)* | q304_M75.2d. In total, how many units are used to treat a patient during all of her care?<br>*Remember to add all units! E.g. 5 days x 200 mg twice per day = 2,000mg*<br>*Question relevant when: selected( ${q104_M75.2_full_list} , '2')*<br>*Response constrained to: .>0* | |
| <p><strong><em>D. Medications - Sepsis (8)</em></strong></p><br>*Group relevant when: selected( ${section_three_skip_med} , '1')* | | |
| q304_med_note8 | 304 CONTINUED For each of the following medications and other medical products that are used for sepsis, can you tell me …? | |
| note_304_hemostatic | ***Hemostatics*** | |

| Field | Question | Answer |
|---|---|---|
| q304_M76 | ***q304_M76. Dicynone / Dicynene (Etamsylate)***<br>*Question relevant when: selected( ${q104_M76_full_list} , '2')* | |
| q304_M76a *(required)* | q304_M76a. What percent of patients require this item?<br>*Question relevant when: selected( ${q104_M76_full_list} , '2')*<br>*Response constrained to: .>0 and .<=100 or .=999* | |

| Field | Question | Answer |
|---|---|---|
| q304_M76b *(required)* | q304_M76b. How is the item administered, or given, to the patient?<br>*Question relevant when: selected( ${q104_M76_full_list} , '2')* | 1 Oral - solid (e.g. tablet)<br>2 Oral - liquid<br>3 Sublingual<br>4 Intravenous (IV)<br>5 Intramusculuar injection (IM)<br>6 Subcutaneous<br>7 Rectal<br>8 Inhaled (e.g. gas)<br>9 Inserted (e.g. vaginal pessaries)<br>10 Topical - cream<br>11 Topical - drops (e.g. eye drops)<br>12 Other<br>99 Don't know |
| q304_M76c *(required)* | q304_M76c. What is the item's smallest unit of measurement (mg, mcg, ml, g, etc)?<br>*Question relevant when: selected( ${q104_M76_full_list} , '2')* | microgram — microgram (mcg)<br>miligram — miligram (mg)<br>miligram_dissolved_in_liquid — miligram (mg) dissolved in liquid (e.g. 2 mg per 3 militers)<br>gram — gram (g)<br>militer — militer (ml)<br>centiliter — centiliter (cl)<br>litre — litre (l)<br>ml_of _solution — ml of solution (e.g. ml of 10% solution)<br>litre_of_solution — litre of solution (e.g. 1 litre of 5% solution)<br>cubic_meter — cubic meter (m^3)<br>other — other<br>dont_know — don't know |
| q304_M76c.1 | q304_M76c.1 If other unit of measurement or route of administration, specify here:<br>*Leave blank if not applicable*<br>*Question relevant when: selected( ${q104_M76_full_list} , '2')* | |
| q304_M76d *(required)* | q304_M76d. In total, how many units are used to treat a patient during all of her care?<br>*Remember to add all units! E.g. 5 days x 200 mg twice per day = 2,000mg*<br>*Question relevant when: selected( ${q104_M76_full_list} , '2')*<br>*Response constrained to: .>0* | |
| q304_M77 | ***q304_M77. Tranexamic acid (Kapron, Cyklokapron)***<br>*Question relevant when: selected( ${q104_M77_full_list} , '2')* | |
| [illegible] | q304_M77a. What percent of patients require this item?<br>*Question relevant when: selected( ${q104_M77_full_list} , '2')*<br>*Response constrained to: .>0 and .<=100 or .=999* | |

q304_M77a *(required)*

| Field | Question | Answer |
|---|---|---|
| q304_M77b *(required)* | q304_M77b. How is the item administered, or given, to the patient?<br>*Question relevant when: selected( ${q104_M77_full_list} , '2')* | 1 Oral - solid (e.g. tablet)<br>2 Oral - liquid<br>3 Sublingual<br>4 Intravenous (IV)<br>5 Intramusculuar injection (IM)<br>6 Subcutaneous<br>7 Rectal<br>8 Inhaled (e.g. gas)<br>9 Inserted (e.g. vaginal pessaries)<br>10 Topical - cream<br>11 Topical - drops (e.g. eye drops)<br>12 Other<br>99 Don't know |
| q304_M77c *(required)* | q304_M77c. What is the item's smallest unit of measurement (mg, mcg, ml, g, etc)?<br>*Question relevant when: selected( ${q104_M77_full_list} , '2')* | microgram — microgram (mcg)<br>miligram — miligram (mg)<br>miligram_dissolved_in_liquid — miligram (mg) dissolved in liquid (e.g. 2 mg per 3 militers)<br>gram — gram (g)<br>militer — militer (ml)<br>centiliter — centiliter (cl)<br>litre — litre (l)<br>ml_of _solution — ml of solution (e.g. ml of 10% solution)<br>litre_of_solution — litre of solution (e.g. 1 litre of 5% solution)<br>cubic_meter — cubic meter (m^3)<br>other — other<br>dont_know — don't know |
| q304_M77c.1 | q304_M77c.1 If other unit of measurement or route of administration, specify here:<br>*Leave blank if not applicable*<br>*Question relevant when: selected( ${q104_M77_full_list} , '2')* | |
| q304_M77d *(required)* | q304_M77d. In total, how many units are used to treat a patient during all of her care?<br>*Remember to add all units! E.g. 5 days x 200 mg twice per day = 2,000mg*<br>*Question relevant when: selected( ${q104_M77_full_list} , '2')*<br>*Response constrained to: .>0* | |
| q304_M78 | ***q304_M78. Polygeline 4%***<br>*Question relevant when: selected( ${q104_M78_full_list} , '2')* | |
| q304_M78a *(required)* | q304_M78a. What percent of patients require this item?<br>*Question relevant when: selected( ${q104_M78_full_list} , '2')*<br>*Response constrained to: .>0 and .<=100 or .=999* | |

| Field | Question | Answer |
|---|---|---|
| q304_M78b *(required)* | q304_M78b. How is the item administered, or given, to the patient?<br>*Question relevant when: selected( ${q104_M78_full_list} , '2')* | 1 Oral - solid (e.g. tablet)<br>2 Oral - liquid<br>3 Sublingual<br>4 Intravenous (IV)<br>5 Intramusculuar injection (IM)<br>6 Subcutaneous<br>7 Rectal<br>8 Inhaled (e.g. gas)<br>9 Inserted (e.g. vaginal pessaries)<br>10 Topical - cream<br>11 Topical - drops (e.g. eye drops)<br>12 Other<br>99 Don't know |
| q304_M78c *(required)* | q304_M78c. What is the item's smallest unit of measurement (mg, mcg, ml, g, etc)?<br>*Question relevant when: selected( ${q104_M78_full_list} , '2')* | microgram — microgram (mcg)<br>miligram — miligram (mg)<br>miligram_dissolved_in_liquid — miligram (mg) dissolved in liquid (e.g. 2 mg per 3 militers)<br>gram — gram (g)<br>militer — militer (ml)<br>centiliter — centiliter (cl)<br>litre — litre (l)<br>ml_of _solution — ml of solution (e.g. ml of 10% solution)<br>litre_of_solution — litre of solution (e.g. 1 litre of 5% solution)<br>cubic_meter — cubic meter (m^3)<br>other — other<br>dont_know — don't know |
| q304_M78c.1 | q304_M78c.1 If other unit of measurement or route of administration, specify here:<br>*Leave blank if not applicable*<br>*Question relevant when: selected( ${q104_M78_full_list} , '2')* | |
| q304_M78d *(required)* | q304_M78d. In total, how many units are used to treat a patient during all of her care?<br>*Remember to add all units! E.g. 5 days x 200 mg twice per day = 2,000mg*<br>*Question relevant when: selected( ${q104_M78_full_list} , '2')*<br>*Response constrained to: .>0* | |
| q304_M79 | ***q304_M79. Hemostatic - other 1: "[q104_M79_full_list_other]"***<br>*Question relevant when: selected( ${q104_M79_full_list} , '2')* | |
| q304_M79a *(required)* | q304_M79a. What percent of patients require this item?<br>*Question relevant when: selected( ${q104_M79_full_list} , '2')*<br>*Response constrained to: .>0 and .<=100 or .=999* | |

| Field | Question | Answer |
|---|---|---|
| q304_M79b *(required)* | q304_M79b. How is the item administered, or given, to the patient?<br>*Question relevant when: selected( ${q104_M79_full_list} , '2')* | 1 Oral - solid (e.g. tablet)<br>2 Oral - liquid<br>3 Sublingual<br>4 Intravenous (IV)<br>5 Intramusculuar injection (IM)<br>6 Subcutaneous<br>7 Rectal<br>8 Inhaled (e.g. gas)<br>9 Inserted (e.g. vaginal pessaries)<br>10 Topical - cream<br>11 Topical - drops (e.g. eye drops)<br>12 Other<br>99 Don't know |
| q304_M79c *(required)* | q304_M79c. What is the item's smallest unit of measurement (mg, mcg, ml, g, etc)?<br>*Question relevant when: selected( ${q104_M79_full_list} , '2')* | microgram — microgram (mcg)<br>miligram — miligram (mg)<br>miligram_dissolved_in_liquid — miligram (mg) dissolved in liquid (e.g. 2 mg per 3 militers)<br>gram — gram (g)<br>militer — militer (ml)<br>centiliter — centiliter (cl)<br>litre — litre (l)<br>ml_of _solution — ml of solution (e.g. ml of 10% solution)<br>litre_of_solution — litre of solution (e.g. 1 litre of 5% solution)<br>cubic_meter — cubic meter (m^3)<br>other — other<br>dont_know — don't know |
| q304_M79c.1 | q304_M79c.1 If other unit of measurement or route of administration, specify here:<br>*Leave blank if not applicable*<br>*Question relevant when: selected( ${q104_M79_full_list} , '2')* | |
| q304_M79d *(required)* | q304_M79d. In total, how many units are used to treat a patient during all of her care?<br>*Remember to add all units! E.g. 5 days x 200 mg twice per day = 2,000mg*<br>*Question relevant when: selected( ${q104_M79_full_list} , '2')*<br>*Response constrained to: .>0* | |
| q304_M80 | ***q304_M80. Hemostatic - other 2: "[q104_M80_full_list_other]"***<br>*Question relevant when: selected( ${q104_M80_full_list} , '2')* | |
| q304_M80a *(required)* | q304_M80a. What percent of patients require this item?<br>*Question relevant when: selected( ${q104_M80_full_list} , '2')*<br>*Response constrained to: .>0 and .<=100 or .=999* | |

| Field | Question | Answer |
|---|---|---|
| q304_M80b *(required)* | q304_M80b. How is the item administered, or given, to the patient?<br>*Question relevant when: selected( ${q104_M80_full_list} , '2')* | 1 Oral - solid (e.g. tablet)<br>2 Oral - liquid<br>3 Sublingual<br>4 Intravenous (IV)<br>5 Intramusculuar injection (IM)<br>6 Subcutaneous<br>7 Rectal<br>8 Inhaled (e.g. gas)<br>9 Inserted (e.g. vaginal pessaries)<br>10 Topical - cream<br>11 Topical - drops (e.g. eye drops)<br>12 Other<br>99 Don't know |
| q304_M80c *(required)* | q304_M80c. What is the item's smallest unit of measurement (mg, mcg, ml, g, etc)?<br>*Question relevant when: selected( ${q104_M80_full_list} , '2')* | microgram — microgram (mcg)<br>miligram — miligram (mg)<br>miligram_dissolved_in_liquid — miligram (mg) dissolved in liquid (e.g. 2 mg per 3 militers)<br>gram — gram (g)<br>militer — militer (ml)<br>centiliter — centiliter (cl)<br>litre — litre (l)<br>ml_of _solution — ml of solution (e.g. ml of 10% solution)<br>litre_of_solution — litre of solution (e.g. 1 litre of 5% solution)<br>cubic_meter — cubic meter (m^3)<br>other — other<br>dont_know — don't know |
| q304_M80c.1 | q304_M80c.1 If other unit of measurement or route of administration, specify here:<br>*Leave blank if not applicable*<br>*Question relevant when: selected( ${q104_M80_full_list} , '2')* | |
| q304_M80d *(required)* | q304_M80d. In total, how many units are used to treat a patient during all of her care?<br>*Remember to add all units! E.g. 5 days x 200 mg twice per day = 2,000mg*<br>*Question relevant when: selected( ${q104_M80_full_list} , '2')*<br>*Response constrained to: .>0* | |
| q304_M81 | ***q304_M81. Hemostatic - other 3: "[q104_M81_full_list_other]"***<br>*Question relevant when: selected( ${q104_M81_full_list} , '2')* | |
| q304_M81a *(required)* | q304_M81a. What percent of patients require this item?<br>*Question relevant when: selected( ${q104_M81_full_list} , '2')*<br>*Response constrained to: .>0 and .<=100 or .=999* | |

| Field | Question | Answer |
|---|---|---|
| q304_M81b *(required)* | q304_M81b. How is the item administered, or given, to the patient?<br>*Question relevant when: selected( ${q104_M81_full_list} , '2')* | 1 Oral - solid (e.g. tablet)<br>2 Oral - liquid<br>3 Sublingual<br>4 Intravenous (IV)<br>5 Intramusculuar injection (IM)<br>6 Subcutaneous<br>7 Rectal<br>8 Inhaled (e.g. gas)<br>9 Inserted (e.g. vaginal pessaries)<br>10 Topical - cream<br>11 Topical - drops (e.g. eye drops)<br>12 Other<br>99 Don't know |
| q304_M81c *(required)* | q304_M81c. What is the item's smallest unit of measurement (mg, mcg, ml, g, etc)?<br>*Question relevant when: selected( ${q104_M81_full_list} , '2')* | microgram – microgram (mcg)<br>miligram – miligram (mg)<br>miligram_dissolved_in_liquid – miligram (mg) dissolved in liquid (e.g. 2 mg per 3 militers)<br>gram – gram (g)<br>militer – militer (ml)<br>centiliter – centiliter (cl)<br>litre – litre (l)<br>ml_of _solution – ml of solution (e.g. ml of 10% solution)<br>litre_of_solution – litre of solution (e.g. 1 litre of 5% solution)<br>cubic_meter – cubic meter (m^3)<br>other – other<br>dont_know – don't know |
| q304_M81c.1 | q304_M81c.1 If other unit of measurement or route of administration, specify here:<br>*Leave blank if not applicable*<br>*Question relevant when: selected( ${q104_M81_full_list} , '2')* | |
| q304_M81d *(required)* | q304_M81d. In total, how many units are used to treat a patient during all of her care?<br>*Remember to add all units! E.g. 5 days x 200 mg twice per day = 2,000mg*<br>*Question relevant when: selected( ${q104_M81_full_list} , '2')*<br>*Response constrained to: .>0* | |
| q304_M81.1 | ***q304_M81.1. Hemostatic - other 4: "[q104_M81.1_full_list_other]"***<br>*Question relevant when: selected( ${q104_M81.1_full_list} , '2')* | |
| q304_M81.1a *(required)* | q304_M81.1a. What percent of patients require this item?<br>*Question relevant when: selected( ${q104_M81.1_full_list} , '2')*<br>*Response constrained to: .>0 and .<=100 or .=999* | |

| Field | Question | Answer |
|---|---|---|
| q304_M81.1b *(required)* | q304_M81.1b. How is the item administered, or given, to the patient?<br>*Question relevant when: selected( ${q104_M81.1_full_list} , '2')* | 1 Oral - solid (e.g. tablet)<br>2 Oral - liquid<br>3 Sublingual<br>4 Intravenous (IV)<br>5 Intramusculuar injection (IM)<br>6 Subcutaneous<br>7 Rectal<br>8 Inhaled (e.g. gas)<br>9 Inserted (e.g. vaginal pessaries)<br>10 Topical - cream<br>11 Topical - drops (e.g. eye drops)<br>12 Other<br>99 Don't know |
| q304_M81.1c *(required)* | q304_M81.1c. What is the item's smallest unit of measurement (mg, mcg, ml, g, etc)?<br>*Question relevant when: selected( ${q104_M81.1_full_list} , '2')* | microgram — microgram (mcg)<br>miligram — miligram (mg)<br>miligram_dissolved_in_liquid — miligram (mg) dissolved in liquid (e.g. 2 mg per 3 militers)<br>gram — gram (g)<br>militer — militer (ml)<br>centiliter — centiliter (cl)<br>litre — litre (l)<br>ml_of _solution — ml of solution (e.g. ml of 10% solution)<br>litre_of_solution — litre of solution (e.g. 1 litre of 5% solution)<br>cubic_meter — cubic meter (m^3)<br>other — other<br>dont_know — don't know |
| q304_M81.1c.1 | q304_M81.1c.1 If other unit of measurement or route of administration, specify here:<br>*Leave blank if not applicable*<br>*Question relevant when: selected( ${q104_M81.1_full_list} , '2')* | |
| q304_M81.1d *(required)* | q304_M81.1d. In total, how many units are used to treat a patient during all of her care?<br>*Remember to add all units! E.g. 5 days x 200 mg twice per day = 2,000mg*<br>*Question relevant when: selected( ${q104_M81.1_full_list} , '2')*<br>*Response constrained to: .>0* | |
| q304_M81.2 | ***q304_M81.2. Hemostatic - other 5: "[q104_M81.2_full_list_other]"***<br>*Question relevant when: selected( ${q104_M81.2_full_list} , '2')* | |
| q304_M81.2a *(required)* | q304_M81.2a. What percent of patients require this item?<br>*Question relevant when: selected( ${q104_M81.2_full_list} , '2')*<br>*Response constrained to: .>0 and .<=100 or .=999* | |

| Field | Question | Answer |
|---|---|---|
| q304_M81.2b *(required)* | q304_M81.2b. How is the item administered, or given, to the patient?<br>*Question relevant when: selected( ${q104_M81.2_full_list} , '2')* | 1 Oral - solid (e.g. tablet)<br>2 Oral - liquid<br>3 Sublingual<br>4 Intravenous (IV)<br>5 Intramusculuar injection (IM)<br>6 Subcutaneous<br>7 Rectal<br>8 Inhaled (e.g. gas)<br>9 Inserted (e.g. vaginal pessaries)<br>10 Topical - cream<br>11 Topical - drops (e.g. eye drops)<br>12 Other<br>99 Don't know |
| q304_M81.2c *(required)* | q304_M81.2c. What is the item's smallest unit of measurement (mg, mcg, ml, g, etc)?<br>*Question relevant when: selected( ${q104_M81.2_full_list} , '2')* | microgram — microgram (mcg)<br>miligram — miligram (mg)<br>miligram_dissolved_in_liquid — miligram (mg) dissolved in liquid (e.g. 2 mg per 3 militers)<br>gram — gram (g)<br>militer — militer (ml)<br>centiliter — centiliter (cl)<br>litre — litre (l)<br>ml_of _solution — ml of solution (e.g. ml of 10% solution)<br>litre_of_solution — litre of solution (e.g. 1 litre of 5% solution)<br>cubic_meter — cubic meter (m^3)<br>other — other<br>dont_know — don't know |
| q304_M81.2c.1 | q304_M81.2c.1 If other unit of measurement or route of administration, specify here:<br>*Leave blank if not applicable*<br>*Question relevant when: selected( ${q104_M81.2_full_list} , '2')* | |
| q304_M81.2d *(required)* | q304_M81.2d. In total, how many units are used to treat a patient during all of her care?<br>*Remember to add all units! E.g. 5 days x 200 mg twice per day = 2,000mg*<br>*Question relevant when: selected( ${q104_M81.2_full_list} , '2')*<br>*Response constrained to: .>0* | |
| <p><strong><em>D. Medications - Sepsis (9)</em></strong></p><br>*Group relevant when: selected( ${section_three_skip_med} , '1')* | | |
| q304_med_notE8 | 304 CONTINUED For each of the following medications and other medical products that are used for sepsis, can you tell me …? | |
| note_304_saline | ***Saline, glucose, water, etc*** | |

| Field | Question | Answer |
|---|---|---|
| q304_M82 | ***q304_M82. IV solution, dextrose 2.5% in NaCl 0.45%***<br>*Question relevant when: selected( ${q104_M82_full_list} , '2')* | |
| q304_M82a *(required)* | q304_M82a. What percent of patients require this item?<br>*Question relevant when: selected( ${q104_M82_full_list} , '2')*<br>*Response constrained to: .>0 and .<=100 or .=999* | |

| | | |
|---|---|---|
| q304_M82b *(required)* | q304_M82b. How is the item administered, or given, to the patient?<br>*Question relevant when: selected( ${q104_M82_full_list} , '2')* | 1 Oral - solid (e.g. tablet)<br>2 Oral - liquid<br>3 Sublingual<br>4 Intravenous (IV)<br>5 Intramusculuar injection (IM)<br>6 Subcutaneous<br>7 Rectal<br>8 Inhaled (e.g. gas)<br>9 Inserted (e.g. vaginal pessaries)<br>10 Topical - cream<br>11 Topical - drops (e.g. eye drops)<br>12 Other<br>99 Don't know |
| q304_M82c *(required)* | q304_M82c. What is the item's smallest unit of measurement (mg, mcg, ml, g, etc)?<br>*Question relevant when: selected( ${q104_M82_full_list} , '2')* | microgram — microgram (mcg)<br>miligram — miligram (mg)<br>miligram_dissolved_in_liquid — miligram (mg) dissolved in liquid (e.g. 2 mg per 3 militers)<br>gram — gram (g)<br>militer — militer (ml)<br>centiliter — centiliter (cl)<br>litre — litre (l)<br>ml_of _solution — ml of solution (e.g. ml of 10% solution)<br>litre_of_solution — litre of solution (e.g. 1 litre of 5% solution)<br>cubic_meter — cubic meter (m^3)<br>other — other<br>dont_know — don't know |
| q304_M82c.1 | q304_M82c.1 If other unit of measurement or route of administration, specify here:<br>*Leave blank if not applicable*<br>*Question relevant when: selected( ${q104_M82_full_list} , '2')* | |
| q304_M82d *(required)* | q304_M82d. In total, how many units are used to treat a patient during all of her care?<br>*Remember to add all units! E.g. 5 days x 200 mg twice per day = 2,000mg*<br>*Question relevant when: selected( ${q104_M82_full_list} , '2')*<br>*Response constrained to: .>0* | |
| q304_M83 | ***q304_M83. IV solution, glucose (5%), isotonic***<br>*Question relevant when: selected( ${q104_M83_full_list} , '2')* | |
| q304_M83a *(required)* | q304_M83a. What percent of patients require this item?<br>*Question relevant when: selected( ${q104_M83_full_list} , '2')*<br>*Response constrained to: .>0 and .<=100 or .=999* | |

| Field | Question | Answer |
|---|---|---|
| q304_M83b *(required)* | q304_M83b. How is the item administered, or given, to the patient?<br>*Question relevant when: selected( ${q104_M83_full_list} , '2')* | 1 Oral - solid (e.g. tablet)<br>2 Oral - liquid<br>3 Sublingual<br>4 Intravenous (IV)<br>5 Intramusculuar injection (IM)<br>6 Subcutaneous<br>7 Rectal<br>8 Inhaled (e.g. gas)<br>9 Inserted (e.g. vaginal pessaries)<br>10 Topical - cream<br>11 Topical - drops (e.g. eye drops)<br>12 Other<br>99 Don't know |
| q304_M83c *(required)* | q304_M83c. What is the item's smallest unit of measurement (mg, mcg, ml, g, etc)?<br>*Question relevant when: selected( ${q104_M83_full_list} , '2')* | microgram — microgram (mcg)<br>miligram — miligram (mg)<br>miligram_dissolved_in_liquid — miligram (mg) dissolved in liquid (e.g. 2 mg per 3 militers)<br>gram — gram (g)<br>militer — militer (ml)<br>centiliter — centiliter (cl)<br>litre — litre (l)<br>ml_of _solution — ml of solution (e.g. ml of 10% solution)<br>litre_of_solution — litre of solution (e.g. 1 litre of 5% solution)<br>cubic_meter — cubic meter (m^3)<br>other — other<br>dont_know — don't know |
| q304_M83c.1 | q304_M83c.1 If other unit of measurement or route of administration, specify here:<br>*Leave blank if not applicable*<br>*Question relevant when: selected( ${q104_M83_full_list} , '2')* | |
| q304_M83d *(required)* | q304_M83d. In total, how many units are used to treat a patient during all of her care?<br>*Remember to add all units! E.g. 5 days x 200 mg twice per day = 2,000mg*<br>*Question relevant when: selected( ${q104_M83_full_list} , '2')*<br>*Response constrained to: .>0* | |
| q304_M84 | ***q304_M84. Ringers lactate (prepared onsite)***<br>*Question relevant when: selected( ${q104_M84_full_list} , '2')* | |
| q304_M84a *(required)* | q304_M84a. What percent of patients require this item?<br>*Question relevant when: selected( ${q104_M84_full_list} , '2')*<br>*Response constrained to: .>0 and .<=100 or .=999* | |

| Field | Question | Answer |
|---|---|---|
| q304_M84b *(required)* | q304_M84b. How is the item administered, or given, to the patient? *Question relevant when: selected( ${q104_M84_full_list} , '2')* | 1 Oral - solid (e.g. tablet)<br>2 Oral - liquid<br>3 Sublingual<br>4 Intravenous (IV)<br>5 Intramusculuar injection (IM)<br>6 Subcutaneous<br>7 Rectal<br>8 Inhaled (e.g. gas)<br>9 Inserted (e.g. vaginal pessaries)<br>10 Topical - cream<br>11 Topical - drops (e.g. eye drops)<br>12 Other<br>99 Don't know |
| q304_M84c *(required)* | q304_M84c. What is the item's smallest unit of measurement (mg, mcg, ml, g, etc)? *Question relevant when: selected( ${q104_M84_full_list} , '2')* | microgram — microgram (mcg)<br>miligram — miligram (mg)<br>miligram_dissolved_in_liquid — miligram (mg) dissolved in liquid (e.g. 2 mg per 3 militers)<br>gram — gram (g)<br>militer — militer (ml)<br>centiliter — centiliter (cl)<br>litre — litre (l)<br>ml_of _solution — ml of solution (e.g. ml of 10% solution)<br>litre_of_solution — litre of solution (e.g. 1 litre of 5% solution)<br>cubic_meter — cubic meter (m^3)<br>other — other<br>dont_know — don't know |
| q304_M84c.1 | q304_M84c.1 If other unit of measurement or route of administration, specify here: *Leave blank if not applicable* *Question relevant when: selected( ${q104_M84_full_list} , '2')* | |
| q304_M84d *(required)* | q304_M84d. In total, how many units are used to treat a patient during all of her care? *Remember to add all units! E.g. 5 days x 200 mg twice per day = 2,000mg* *Question relevant when: selected( ${q104_M84_full_list} , '2')* *Response constrained to: .>0* | |
| q304_M85 | ***q304_M85. Ringers lactate (bought from pharmacy/MSD)*** *Question relevant when: selected( ${q104_M85_full_list} , '2')* | |
| | q304_M85a. What percent of patients require this item? *Question relevant when: selected( ${q104_M85_full_list} , '2')* *Response constrained to: .>0 and .<=100 or .=999* | |

| Field | Question | Answer |
|---|---|---|
| q304_M85a *(required)* | | |
| q304_M85b *(required)* | q304_M85b. How is the item administered, or given, to the patient?<br>*Question relevant when: selected( ${q104_M85_full_list} , '2')* | 1 Oral - solid (e.g. tablet)<br>2 Oral - liquid<br>3 Sublingual<br>4 Intravenous (IV)<br>5 Intramusculuar injection (IM)<br>6 Subcutaneous<br>7 Rectal<br>8 Inhaled (e.g. gas)<br>9 Inserted (e.g. vaginal pessaries)<br>10 Topical - cream<br>11 Topical - drops (e.g. eye drops)<br>12 Other<br>99 Don't know |
| q304_M85c *(required)* | q304_M85c. What is the item's smallest unit of measurement (mg, mcg, ml, g, etc)?<br>*Question relevant when: selected( ${q104_M85_full_list} , '2')* | microgram — microgram (mcg)<br>miligram — miligram (mg)<br>miligram_dissolved_in_liquid — miligram (mg) dissolved in liquid (e.g. 2 mg per 3 militers)<br>gram — gram (g)<br>militer — militer (ml)<br>centiliter — centiliter (cl)<br>litre — litre (l)<br>ml_of _solution — ml of solution (e.g. ml of 10% solution)<br>litre_of_solution — litre of solution (e.g. 1 litre of 5% solution)<br>cubic_meter — cubic meter (m^3)<br>other — other<br>dont_know — don't know |
| q304_M85c.1 | q304_M85c.1 If other unit of measurement or route of administration, specify here:<br>*Leave blank if not applicable*<br>*Question relevant when: selected( ${q104_M85_full_list} , '2')* | |
| q304_M85d *(required)* | q304_M85d. In total, how many units are used to treat a patient during all of her care?<br>*Remember to add all units! E.g. 5 days x 200 mg twice per day = 2,000mg*<br>*Question relevant when: selected( ${q104_M85_full_list} , '2')*<br>*Response constrained to: .>0* | |
| q304_M86 | ***q304_M86. Saline, 0.9% (Normal Saline) (prepared onsite)***<br>*Question relevant when: selected( ${q104_M86_full_list} , '2')* | |
| q304_M86a *(required)* | q304_M86a. What percent of patients require this item?<br>*Question relevant when: selected( ${q104_M86_full_list} , '2')*<br>*Response constrained to: .>0 and .<=100 or .=999* | |

| Field | Question | Answer |
|---|---|---|
| q304_M86b *(required)* | q304_M86b. How is the item administered, or given, to the patient? *Question relevant when: selected( ${q104_M86_full_list} , '2')* | 1 Oral - solid (e.g. tablet)<br>2 Oral - liquid<br>3 Sublingual<br>4 Intravenous (IV)<br>5 Intramusculuar injection (IM)<br>6 Subcutaneous<br>7 Rectal<br>8 Inhaled (e.g. gas)<br>9 Inserted (e.g. vaginal pessaries)<br>10 Topical - cream<br>11 Topical - drops (e.g. eye drops)<br>12 Other<br>99 Don't know |
| q304_M86c *(required)* | q304_M86c. What is the item's smallest unit of measurement (mg, mcg, ml, g, etc)? *Question relevant when: selected( ${q104_M86_full_list} , '2')* | microgram: microgram (mcg)<br>miligram: miligram (mg)<br>miligram_dissolved_in_liquid: miligram (mg) dissolved in liquid (e.g. 2 mg per 3 militers)<br>gram: gram (g)<br>militer: militer (ml)<br>centiliter: centiliter (cl)<br>litre: litre (l)<br>ml_of _solution: ml of solution (e.g. ml of 10% solution)<br>litre_of_solution: litre of solution (e.g. 1 litre of 5% solution)<br>cubic_meter: cubic meter (m^3)<br>other: other<br>dont_know: don't know |
| q304_M86c.1 | q304_M86c.1 If other unit of measurement or route of administration, specify here: *Leave blank if not applicable* *Question relevant when: selected( ${q104_M86_full_list} , '2')* | |
| q304_M86d *(required)* | q304_M86d. In total, how many units are used to treat a patient during all of her care? *Remember to add all units! E.g. 5 days x 200 mg twice per day = 2,000mg* *Question relevant when: selected( ${q104_M86_full_list} , '2')* *Response constrained to: .>0* | |
| q304_M87 | ***q304_M87. Saline, 0.9% (Normal Saline) (bought from pharmacy/MSD)*** *Question relevant when: selected( ${q104_M87_full_list} , '2')* | |
| q304_M87a *(required)* | q304_M87a. What percent of patients require this item? *Question relevant when: selected( ${q104_M87_full_list} , '2')* *Response constrained to: .>0 and .<=100 or .=999* | |

| Field | Question | Answer |
|---|---|---|
| q304_M87b *(required)* | q304_M87b. How is the item administered, or given, to the patient?<br>*Question relevant when: selected( ${q104_M87_full_list} , '2')* | 1 Oral - solid (e.g. tablet)<br>2 Oral - liquid<br>3 Sublingual<br>4 Intravenous (IV)<br>5 Intramusculuar injection (IM)<br>6 Subcutaneous<br>7 Rectal<br>8 Inhaled (e.g. gas)<br>9 Inserted (e.g. vaginal pessaries)<br>10 Topical - cream<br>11 Topical - drops (e.g. eye drops)<br>12 Other<br>99 Don't know |
| q304_M87c *(required)* | q304_M87c. What is the item's smallest unit of measurement (mg, mcg, ml, g, etc)?<br>*Question relevant when: selected( ${q104_M87_full_list} , '2')* | microgram — microgram (mcg)<br>miligram — miligram (mg)<br>miligram_dissolved_in_liquid — miligram (mg) dissolved in liquid (e.g. 2 mg per 3 militers)<br>gram — gram (g)<br>militer — militer (ml)<br>centiliter — centiliter (cl)<br>litre — litre (l)<br>ml_of _solution — ml of solution (e.g. ml of 10% solution)<br>litre_of_solution — litre of solution (e.g. 1 litre of 5% solution)<br>cubic_meter — cubic meter (m^3)<br>other — other<br>dont_know — don't know |
| q304_M87c.1 | q304_M87c.1 If other unit of measurement or route of administration, specify here:<br>*Leave blank if not applicable*<br>*Question relevant when: selected( ${q104_M87_full_list} , '2')* | |
| q304_M87d *(required)* | q304_M87d. In total, how many units are used to treat a patient during all of her care?<br>*Remember to add all units! E.g. 5 days x 200 mg twice per day = 2,000mg*<br>*Question relevant when: selected( ${q104_M87_full_list} , '2')*<br>*Response constrained to: .>0* | |
| q304_M88 | ***q304_M88. Solution for Infusion, glucose 5% + NaCl 0.9% (prepared onsite)***<br>*Question relevant when: selected( ${q104_M88_full_list} , '2')* | |
| q304_M88a *(required)* | q304_M88a. What percent of patients require this item?<br>*Question relevant when: selected( ${q104_M88_full_list} , '2')*<br>*Response constrained to: .>0 and .<=100 or .=999* | |

| Field | Question | Answer |
|---|---|---|
| q304_M88b *(required)* | q304_M88b. How is the item administered, or given, to the patient?<br>*Question relevant when: selected( ${q104_M88_full_list} , '2')* | 1 Oral - solid (e.g. tablet)<br>2 Oral - liquid<br>3 Sublingual<br>4 Intravenous (IV)<br>5 Intramusculuar injection (IM)<br>6 Subcutaneous<br>7 Rectal<br>8 Inhaled (e.g. gas)<br>9 Inserted (e.g. vaginal pessaries)<br>10 Topical - cream<br>11 Topical - drops (e.g. eye drops)<br>12 Other<br>99 Don't know |
| q304_M88c *(required)* | q304_M88c. What is the item's smallest unit of measurement (mg, mcg, ml, g, etc)?<br>*Question relevant when: selected( ${q104_M88_full_list} , '2')* | microgram — microgram (mcg)<br>miligram — miligram (mg)<br>miligram_dissolved_in_liquid — miligram (mg) dissolved in liquid (e.g. 2 mg per 3 militers)<br>gram — gram (g)<br>militer — militer (ml)<br>centiliter — centiliter (cl)<br>litre — litre (l)<br>ml_of _solution — ml of solution (e.g. ml of 10% solution)<br>litre_of_solution — litre of solution (e.g. 1 litre of 5% solution)<br>cubic_meter — cubic meter (m^3)<br>other — other<br>dont_know — don't know |
| q304_M88c.1 | q304_M88c.1 If other unit of measurement or route of administration, specify here:<br>*Leave blank if not applicable*<br>*Question relevant when: selected( ${q104_M88_full_list} , '2')* | |
| q304_M88d *(required)* | q304_M88d. In total, how many units are used to treat a patient during all of her care?<br>*Remember to add all units! E.g. 5 days x 200 mg twice per day = 2,000mg*<br>*Question relevant when: selected( ${q104_M88_full_list} , '2')*<br>*Response constrained to: .>0* | |
| q304_M89 | ***q304_M89. Solution for Infusion, glucose 5% + NaCl 0.9% (bought from pharmacy or MSD)***<br>*Question relevant when: selected( ${q104_M89_full_list} , '2')* | |
| q304_M89a *(required)* | q304_M89a. What percent of patients require this item?<br>*Question relevant when: selected( ${q104_M89_full_list} , '2')*<br>*Response constrained to: .>0 and .<=100 or .=999* | |

| Field | Question | Answer |
|---|---|---|
| q304_M89b *(required)* | q304_M89b. How is the item administered, or given, to the patient?<br>*Question relevant when: selected( ${q104_M89_full_list} , '2')* | 1 Oral - solid (e.g. tablet)<br>2 Oral - liquid<br>3 Sublingual<br>4 Intravenous (IV)<br>5 Intramuscular injection (IM)<br>6 Subcutaneous<br>7 Rectal<br>8 Inhaled (e.g. gas)<br>9 Inserted (e.g. vaginal pessaries)<br>10 Topical - cream<br>11 Topical - drops (e.g. eye drops)<br>12 Other<br>99 Don't know |
| q304_M89c *(required)* | q304_M89c. What is the item's smallest unit of measurement (mg, mcg, ml, g, etc)?<br>*Question relevant when: selected( ${q104_M89_full_list} , '2')* | microgram — microgram (mcg)<br>miligram — miligram (mg)<br>miligram_dissolved_in_liquid — miligram (mg) dissolved in liquid (e.g. 2 mg per 3 militers)<br>gram — gram (g)<br>militer — militer (ml)<br>centiliter — centiliter (cl)<br>litre — litre (l)<br>ml_of _solution — ml of solution (e.g. ml of 10% solution)<br>litre_of_solution — litre of solution (e.g. 1 litre of 5% solution)<br>cubic_meter — cubic meter (m^3)<br>other — other<br>dont_know — don't know |
| q304_M89c.1 | q304_M89c.1 If other unit of measurement or route of administration, specify here:<br>*Leave blank if not applicable*<br>*Question relevant when: selected( ${q104_M89_full_list} , '2')* | |
| q304_M89d *(required)* | q304_M89d. In total, how many units are used to treat a patient during all of her care?<br>*Remember to add all units! E.g. 5 days x 200 mg twice per day = 2,000mg*<br>*Question relevant when: selected( ${q104_M89_full_list} , '2')*<br>*Response constrained to: .>0* | |
| q304_M90 | ***q304_M90. Water for injection***<br>*Question relevant when: selected( ${q104_M90_full_list} , '2')* | |
| q304_M90a *(required)* | q304_M90a. What percent of patients require this item?<br>*Question relevant when: selected( ${q104_M90_full_list} , '2')*<br>*Response constrained to: .>0 and .<=100 or .=999* | |

| Field | Question | Answer |
|---|---|---|
| q304_M90b *(required)* | q304_M90b. How is the item administered, or given, to the patient?<br>*Question relevant when: selected( ${q104_M90_full_list} , '2')* | 1 Oral - solid (e.g. tablet)<br>2 Oral - liquid<br>3 Sublingual<br>4 Intravenous (IV)<br>5 Intramusculuar injection (IM)<br>6 Subcutaneous<br>7 Rectal<br>8 Inhaled (e.g. gas)<br>9 Inserted (e.g. vaginal pessaries)<br>10 Topical - cream<br>11 Topical - drops (e.g. eye drops)<br>12 Other<br>99 Don't know |
| q304_M90c *(required)* | q304_M90c. What is the item's smallest unit of measurement (mg, mcg, ml, g, etc)?<br>*Question relevant when: selected( ${q104_M90_full_list} , '2')* | microgram — microgram (mcg)<br>miligram — miligram (mg)<br>miligram_dissolved_in_liquid — miligram (mg) dissolved in liquid (e.g. 2 mg per 3 militers)<br>gram — gram (g)<br>militer — militer (ml)<br>centiliter — centiliter (cl)<br>litre — litre (l)<br>ml_of _solution — ml of solution (e.g. ml of 10% solution)<br>litre_of_solution — litre of solution (e.g. 1 litre of 5% solution)<br>cubic_meter — cubic meter (m^3)<br>other — other<br>dont_know — don't know |
| q304_M90c.1 | q304_M90c.1 If other unit of measurement or route of administration, specify here:<br>*Leave blank if not applicable*<br>*Question relevant when: selected( ${q104_M90_full_list} , '2')* | |
| q304_M90d *(required)* | q304_M90d. In total, how many units are used to treat a patient during all of her care?<br>*Remember to add all units! E.g. 5 days x 200 mg twice per day = 2,000mg*<br>*Question relevant when: selected( ${q104_M90_full_list} , '2')*<br>*Response constrained to: .>0* | |
| q304_M91 | ***q304_M91. Saline, glucose, water, etc. - Other 1: "[q104_M91_full_list_other]"***<br>*Question relevant when: selected( ${q104_M91_full_list} , '2')* | |
| q304_M91a *(required)* | q304_M91a. What percent of patients require this item?<br>*Question relevant when: selected( ${q104_M91_full_list} , '2')*<br>*Response constrained to: .>0 and .<=100 or .=999* | |

| Field | Question | Answer |
|---|---|---|
| q304_M91b *(required)* | q304_M91b. How is the item administered, or given, to the patient?<br>*Question relevant when: selected( ${q104_M91_full_list} , '2')* | 1 Oral - solid (e.g. tablet)<br>2 Oral - liquid<br>3 Sublingual<br>4 Intravenous (IV)<br>5 Intramusculuar injection (IM)<br>6 Subcutaneous<br>7 Rectal<br>8 Inhaled (e.g. gas)<br>9 Inserted (e.g. vaginal pessaries)<br>10 Topical - cream<br>11 Topical - drops (e.g. eye drops)<br>12 Other<br>99 Don't know |
| q304_M91c *(required)* | q304_M91c. What is the item's smallest unit of measurement (mg, mcg, ml, g, etc)?<br>*Question relevant when: selected( ${q104_M91_full_list} , '2')* | microgram — microgram (mcg)<br>miligram — miligram (mg)<br>miligram_dissolved_in_liquid — miligram (mg) dissolved in liquid (e.g. 2 mg per 3 militers)<br>gram — gram (g)<br>militer — militer (ml)<br>centiliter — centiliter (cl)<br>litre — litre (l)<br>ml_of _solution — ml of solution (e.g. ml of 10% solution)<br>litre_of_solution — litre of solution (e.g. 1 litre of 5% solution)<br>cubic_meter — cubic meter (m^3)<br>other — other<br>dont_know — don't know |
| q304_M91c.1 | q304_M91c.1 If other unit of measurement or route of administration, specify here:<br>*Leave blank if not applicable*<br>*Question relevant when: selected( ${q104_M91_full_list} , '2')* | |
| q304_M91d *(required)* | q304_M91d. In total, how many units are used to treat a patient during all of her care?<br>*Remember to add all units! E.g. 5 days x 200 mg twice per day = 2,000mg*<br>*Question relevant when: selected( ${q104_M91_full_list} , '2')*<br>*Response constrained to: .>0* | |
| q304_M92 | ***q304_M92. Saline, glucose, water, etc. - Other 2: "[q104_M92_full_list_other]"***<br>*Question relevant when: selected( ${q104_M92_full_list} , '2')* | |
| q304_M92a *(required)* | q304_M92a. What percent of patients require this item?<br>*Question relevant when: selected( ${q104_M92_full_list} , '2')*<br>*Response constrained to: .>0 and .<=100 or .=999* | |

| Field | Question | Answer |
|---|---|---|
| q304_M92b *(required)* | q304_M92b. How is the item administered, or given, to the patient?<br>*Question relevant when: selected( ${q104_M92_full_list} , '2')* | 1 Oral - solid (e.g. tablet)<br>2 Oral - liquid<br>3 Sublingual<br>4 Intravenous (IV)<br>5 Intramusculuar injection (IM)<br>6 Subcutaneous<br>7 Rectal<br>8 Inhaled (e.g. gas)<br>9 Inserted (e.g. vaginal pessaries)<br>10 Topical - cream<br>11 Topical - drops (e.g. eye drops)<br>12 Other<br>99 Don't know |
| q304_M92c *(required)* | q304_M92c. What is the item's smallest unit of measurement (mg, mcg, ml, g, etc)?<br>*Question relevant when: selected( ${q104_M92_full_list} , '2')* | microgram — microgram (mcg)<br>miligram — miligram (mg)<br>miligram_dissolved_in_liquid — miligram (mg) dissolved in liquid (e.g. 2 mg per 3 militers)<br>gram — gram (g)<br>militer — militer (ml)<br>centiliter — centiliter (cl)<br>litre — litre (l)<br>ml_of _solution — ml of solution (e.g. ml of 10% solution)<br>litre_of_solution — litre of solution (e.g. 1 litre of 5% solution)<br>cubic_meter — cubic meter (m^3)<br>other — other<br>dont_know — don't know |
| q304_M92c.1 | q304_M92c.1 If other unit of measurement or route of administration, specify here:<br>*Leave blank if not applicable*<br>*Question relevant when: selected( ${q104_M92_full_list} , '2')* | |
| q304_M92d *(required)* | q304_M92d. In total, how many units are used to treat a patient during all of her care?<br>*Remember to add all units! E.g. 5 days x 200 mg twice per day = 2,000mg*<br>*Question relevant when: selected( ${q104_M92_full_list} , '2')*<br>*Response constrained to: .>0* | |
| q304_M93 | ***q304_M93. Saline, glucose, water, etc. - Other 3: "[q104_M93_full_list_other]"***<br>*Question relevant when: selected( ${q104_M93_full_list} , '2')* | |
| q304_M93a *(required)* | q304_M93a. What percent of patients require this item?<br>*Question relevant when: selected( ${q104_M93_full_list} , '2')*<br>*Response constrained to: .>0 and .<=100 or .=999* | |

| Field | Question | Answer |
|---|---|---|
| q304_M93b *(required)* | q304_M93b. How is the item administered, or given, to the patient?<br>*Question relevant when: selected( ${q104_M93_full_list} , '2')* | 1 Oral - solid (e.g. tablet)<br>2 Oral - liquid<br>3 Sublingual<br>4 Intravenous (IV)<br>5 Intramusculuar injection (IM)<br>6 Subcutaneous<br>7 Rectal<br>8 Inhaled (e.g. gas)<br>9 Inserted (e.g. vaginal pessaries)<br>10 Topical - cream<br>11 Topical - drops (e.g. eye drops)<br>12 Other<br>99 Don't know |
| q304_M93c *(required)* | q304_M93c. What is the item's smallest unit of measurement (mg, mcg, ml, g, etc)?<br>*Question relevant when: selected( ${q104_M93_full_list} , '2')* | microgram — microgram (mcg)<br>miligram — miligram (mg)<br>miligram_dissolved_in_liquid — miligram (mg) dissolved in liquid (e.g. 2 mg per 3 militers)<br>gram — gram (g)<br>militer — militer (ml)<br>centiliter — centiliter (cl)<br>litre — litre (l)<br>ml_of _solution — ml of solution (e.g. ml of 10% solution)<br>litre_of_solution — litre of solution (e.g. 1 litre of 5% solution)<br>cubic_meter — cubic meter (m^3)<br>other — other<br>dont_know — don't know |
| q304_M93c.1 | q304_M93c.1 If other unit of measurement or route of administration, specify here:<br>*Leave blank if not applicable*<br>*Question relevant when: selected( ${q104_M93_full_list} , '2')* | |
| q304_M93d *(required)* | q304_M93d. In total, how many units are used to treat a patient during all of her care?<br>*Remember to add all units! E.g. 5 days x 200 mg twice per day = 2,000mg*<br>*Question relevant when: selected( ${q104_M93_full_list} , '2')*<br>*Response constrained to: .>0* | |
| q304_M94 | ***q304_M94. Saline, glucose, water, etc. - Other 4: "[q104_M94_full_list_other]"***<br>*Question relevant when: selected( ${q104_M94_full_list} , '2')* | |
| q304_M94a *(required)* | q304_M94a. What percent of patients require this item?<br>*Question relevant when: selected( ${q104_M94_full_list} , '2')*<br>*Response constrained to: .>0 and .<=100 or .=999* | |

| Field | Question | Answer |
|---|---|---|
| q304_M94b *(required)* | q304_M94b. How is the item administered, or given, to the patient?<br>*Question relevant when: selected( ${q104_M94_full_list} , '2')* | 1 Oral - solid (e.g. tablet)<br>2 Oral - liquid<br>3 Sublingual<br>4 Intravenous (IV)<br>5 Intramusculuar injection (IM)<br>6 Subcutaneous<br>7 Rectal<br>8 Inhaled (e.g. gas)<br>9 Inserted (e.g. vaginal pessaries)<br>10 Topical - cream<br>11 Topical - drops (e.g. eye drops)<br>12 Other<br>99 Don't know |
| q304_M94c *(required)* | q304_M94c. What is the item's smallest unit of measurement (mg, mcg, ml, g, etc)?<br>*Question relevant when: selected( ${q104_M94_full_list} , '2')* | microgram — microgram (mcg)<br>miligram — miligram (mg)<br>miligram_dissolved_in_liquid — miligram (mg) dissolved in liquid (e.g. 2 mg per 3 militers)<br>gram — gram (g)<br>militer — militer (ml)<br>centiliter — centiliter (cl)<br>litre — litre (l)<br>ml_of _solution — ml of solution (e.g. ml of 10% solution)<br>litre_of_solution — litre of solution (e.g. 1 litre of 5% solution)<br>cubic_meter — cubic meter (m^3)<br>other — other<br>dont_know — don't know |
| q304_M94c.1 | q304_M94c.1 If other unit of measurement or route of administration, specify here:<br>*Leave blank if not applicable*<br>*Question relevant when: selected( ${q104_M94_full_list} , '2')* | |
| q304_M94d *(required)* | q304_M94d. In total, how many units are used to treat a patient during all of her care?<br>*Remember to add all units! E.g. 5 days x 200 mg twice per day = 2,000mg*<br>*Question relevant when: selected( ${q104_M94_full_list} , '2')*<br>*Response constrained to: .>0* | |
| q304_M95 | ***q304_M95. Saline, glucose, water, etc. - Other 5: "[q104_M95_full_list_other]"***<br>*Question relevant when: selected( ${q104_M95_full_list} , '2')* | |
| q304_M95a *(required)* | q304_M95a. What percent of patients require this item?<br>*Question relevant when: selected( ${q104_M95_full_list} , '2')*<br>*Response constrained to: .>0 and .<=100 or .=999* | |

| Field | Question | Answer |
|---|---|---|
| q304_M95b *(required)* | q304_M95b. How is the item administered, or given, to the patient?<br>*Question relevant when: selected( ${q104_M95_full_list} , '2')* | 1 Oral - solid (e.g. tablet)<br>2 Oral - liquid<br>3 Sublingual<br>4 Intravenous (IV)<br>5 Intramusculuar injection (IM)<br>6 Subcutaneous<br>7 Rectal<br>8 Inhaled (e.g. gas)<br>9 Inserted (e.g. vaginal pessaries)<br>10 Topical - cream<br>11 Topical - drops (e.g. eye drops)<br>12 Other<br>99 Don't know |
| q304_M95c *(required)* | q304_M95c. What is the item's smallest unit of measurement (mg, mcg, ml, g, etc)?<br>*Question relevant when: selected( ${q104_M95_full_list} , '2')* | microgram — microgram (mcg)<br>miligram — miligram (mg)<br>miligram_dissolved_in_liquid — miligram (mg) dissolved in liquid (e.g. 2 mg per 3 militers)<br>gram — gram (g)<br>militer — militer (ml)<br>centiliter — centiliter (cl)<br>litre — litre (l)<br>ml_of _solution — ml of solution (e.g. ml of 10% solution)<br>litre_of_solution — litre of solution (e.g. 1 litre of 5% solution)<br>cubic_meter — cubic meter (m^3)<br>other — other<br>dont_know — don't know |
| q304_M95c.1 | q304_M95c.1 If other unit of measurement or route of administration, specify here:<br>*Leave blank if not applicable*<br>*Question relevant when: selected( ${q104_M95_full_list} , '2')* | |
| q304_M95d *(required)* | q304_M95d. In total, how many units are used to treat a patient during all of her care?<br>*Remember to add all units! E.g. 5 days x 200 mg twice per day = 2,000mg*<br>*Question relevant when: selected( ${q104_M95_full_list} , '2')*<br>*Response constrained to: .>0* | |
| <p><strong><em>D. Medications - Sepsis (10)</em></strong></p><br>*Group relevant when: selected( ${section_three_skip_med} , '1')* | | |
| q304_med_notE9 | 304 CONTINUED For each of the following medications and other medical products that are used for sepsis, can you tell me …? | |
| note_304_prost_utero | ***Prostaglandin, uterotonic, etc.*** | |

| Field | Question | Answer |
|---|---|---|
| q304_M96 | ***q304_M96. Misoprostol***<br>*Question relevant when: selected( ${q104_M96_full_list} , '2')* | |
| q304_M96a *(required)* | q304_M96a. What percent of patients require this item?<br>*Question relevant when: selected( ${q104_M96_full_list} , '2')*<br>*Response constrained to: .>0 and .<=100 or .=999* | |

| Field | Question | Answer |
|---|---|---|
| q304_M96b *(required)* | q304_M96b. How is the item administered, or given, to the patient?<br>*Question relevant when: selected( ${q104_M96_full_list} , '2')* | 1 Oral - solid (e.g. tablet)<br>2 Oral - liquid<br>3 Sublingual<br>4 Intravenous (IV)<br>5 Intramusculuar injection (IM)<br>6 Subcutaneous<br>7 Rectal<br>8 Inhaled (e.g. gas)<br>9 Inserted (e.g. vaginal pessaries)<br>10 Topical - cream<br>11 Topical - drops (e.g. eye drops)<br>12 Other<br>99 Don't know |
| q304_M96c *(required)* | q304_M96c. What is the item's smallest unit of measurement (mg, mcg, ml, g, etc)?<br>*Question relevant when: selected( ${q104_M96_full_list} , '2')* | microgram — microgram (mcg)<br>miligram — miligram (mg)<br>miligram_dissolved_in_liquid — miligram (mg) dissolved in liquid (e.g. 2 mg per 3 militers)<br>gram — gram (g)<br>militer — militer (ml)<br>centiliter — centiliter (cl)<br>litre — litre (l)<br>ml_of _solution — ml of solution (e.g. ml of 10% solution)<br>litre_of_solution — litre of solution (e.g. 1 litre of 5% solution)<br>cubic_meter — cubic meter (m^3)<br>other — other<br>dont_know — don't know |
| q304_M96c.1 | q304_M96c.1 If other unit of measurement or route of administration, specify here:<br>*Leave blank if not applicable*<br>*Question relevant when: selected( ${q104_M96_full_list} , '2')* | |
| q304_M96d *(required)* | q304_M96d. In total, how many units are used to treat a patient during all of her care?<br>*Remember to add all units! E.g. 5 days x 200 mg twice per day = 2,000mg*<br>*Question relevant when: selected( ${q104_M96_full_list} , '2')*<br>*Response constrained to: .>0* | |
| q304_M97 | ***q304_M97. Ergometrine***<br>*Question relevant when: selected( ${q104_M97_full_list} , '2')* | |
| q304_M97a *(required)* | q304_M97a. What percent of patients require this item?<br>*Question relevant when: selected( ${q104_M97_full_list} , '2')*<br>*Response constrained to: .>0 and .<=100 or .=999* | |

| Field | Question | Answer |
|---|---|---|
| q304_M97b *(required)* | q304_M97b. How is the item administered, or given, to the patient?<br>*Question relevant when: selected( ${q104_M97_full_list} , '2')* | 1 Oral - solid (e.g. tablet)<br>2 Oral - liquid<br>3 Sublingual<br>4 Intravenous (IV)<br>5 Intramusculuar injection (IM)<br>6 Subcutaneous<br>7 Rectal<br>8 Inhaled (e.g. gas)<br>9 Inserted (e.g. vaginal pessaries)<br>10 Topical - cream<br>11 Topical - drops (e.g. eye drops)<br>12 Other<br>99 Don't know |
| q304_M97c *(required)* | q304_M97c. What is the item's smallest unit of measurement (mg, mcg, ml, g, etc)?<br>*Question relevant when: selected( ${q104_M97_full_list} , '2')* | microgram — microgram (mcg)<br>miligram — miligram (mg)<br>miligram_dissolved_in_liquid — miligram (mg) dissolved in liquid (e.g. 2 mg per 3 militers)<br>gram — gram (g)<br>militer — militer (ml)<br>centiliter — centiliter (cl)<br>litre — litre (l)<br>ml_of _solution — ml of solution (e.g. ml of 10% solution)<br>litre_of_solution — litre of solution (e.g. 1 litre of 5% solution)<br>cubic_meter — cubic meter (m^3)<br>other — other<br>dont_know — don't know |
| q304_M97c.1 | q304_M97c.1 If other unit of measurement or route of administration, specify here:<br>*Leave blank if not applicable*<br>*Question relevant when: selected( ${q104_M97_full_list} , '2')* | |
| q304_M97d *(required)* | q304_M97d. In total, how many units are used to treat a patient during all of her care?<br>*Remember to add all units! E.g. 5 days x 200 mg twice per day = 2,000mg*<br>*Question relevant when: selected( ${q104_M97_full_list} , '2')*<br>*Response constrained to: .>0* | |
| q304_M98 | ***q304_M98. Methylergonovine Maleate (Methergine)***<br>*Question relevant when: selected( ${q104_M98_full_list} , '2')* | |
| q304_M98a *(required)* | q304_M98a. What percent of patients require this item?<br>*Question relevant when: selected( ${q104_M98_full_list} , '2')*<br>*Response constrained to: .>0 and .<=100 or .=999* | |

| Field | Question | Answer |
|---|---|---|
| q304_M98b *(required)* | q304_M98b. How is the item administered, or given, to the patient?<br>*Question relevant when: selected( ${q104_M98_full_list} , '2')* | 1 Oral - solid (e.g. tablet)<br>2 Oral - liquid<br>3 Sublingual<br>4 Intravenous (IV)<br>5 Intramusculuar injection (IM)<br>6 Subcutaneous<br>7 Rectal<br>8 Inhaled (e.g. gas)<br>9 Inserted (e.g. vaginal pessaries)<br>10 Topical - cream<br>11 Topical - drops (e.g. eye drops)<br>12 Other<br>99 Don't know |
| q304_M98c *(required)* | q304_M98c. What is the item's smallest unit of measurement (mg, mcg, ml, g, etc)?<br>*Question relevant when: selected( ${q104_M98_full_list} , '2')* | microgram — microgram (mcg)<br>miligram — miligram (mg)<br>miligram_dissolved_in_liquid — miligram (mg) dissolved in liquid (e.g. 2 mg per 3 militers)<br>gram — gram (g)<br>militer — militer (ml)<br>centiliter — centiliter (cl)<br>litre — litre (l)<br>ml_of _solution — ml of solution (e.g. ml of 10% solution)<br>litre_of_solution — litre of solution (e.g. 1 litre of 5% solution)<br>cubic_meter — cubic meter (m^3)<br>other — other<br>dont_know — don't know |
| q304_M98c.1 | q304_M98c.1 If other unit of measurement or route of administration, specify here:<br>*Leave blank if not applicable*<br>*Question relevant when: selected( ${q104_M98_full_list} , '2')* | |
| q304_M98d *(required)* | q304_M98d. In total, how many units are used to treat a patient during all of her care?<br>*Remember to add all units! E.g. 5 days x 200 mg twice per day = 2,000mg*<br>*Question relevant when: selected( ${q104_M98_full_list} , '2')*<br>*Response constrained to: .>0* | |
| q304_M99 | ***q304_M99. Mifepristone***<br>*Question relevant when: selected( ${q104_M99_full_list} , '2')* | |
| q304_M99a *(required)* | q304_M99a. What percent of patients require this item?<br>*Question relevant when: selected( ${q104_M99_full_list} , '2')*<br>*Response constrained to: .>0 and .<=100 or .=999* | |

| Field | Question | Answer |
|---|---|---|
| q304_M99b *(required)* | q304_M99b. How is the item administered, or given, to the patient?<br>*Question relevant when: selected( ${q104_M99_full_list} , '2')* | 1 Oral - solid (e.g. tablet)<br>2 Oral - liquid<br>3 Sublingual<br>4 Intravenous (IV)<br>5 Intramusculuar injection (IM)<br>6 Subcutaneous<br>7 Rectal<br>8 Inhaled (e.g. gas)<br>9 Inserted (e.g. vaginal pessaries)<br>10 Topical - cream<br>11 Topical - drops (e.g. eye drops)<br>12 Other<br>99 Don't know |
| q304_M99c *(required)* | q304_M99c. What is the item's smallest unit of measurement (mg, mcg, ml, g, etc)?<br>*Question relevant when: selected( ${q104_M99_full_list} , '2')* | microgram — microgram (mcg)<br>miligram — miligram (mg)<br>miligram_dissolved_in_liquid — miligram (mg) dissolved in liquid (e.g. 2 mg per 3 militers)<br>gram — gram (g)<br>militer — militer (ml)<br>centiliter — centiliter (cl)<br>litre — litre (l)<br>ml_of _solution — ml of solution (e.g. ml of 10% solution)<br>litre_of_solution — litre of solution (e.g. 1 litre of 5% solution)<br>cubic_meter — cubic meter (m^3)<br>other — other<br>dont_know — don't know |
| q304_M99c.1 | q304_M99c.1 If other unit of measurement or route of administration, specify here:<br>*Leave blank if not applicable*<br>*Question relevant when: selected( ${q104_M99_full_list} , '2')* | |
| q304_M99d *(required)* | q304_M99d. In total, how many units are used to treat a patient during all of her care?<br>*Remember to add all units! E.g. 5 days x 200 mg twice per day = 2,000mg*<br>*Question relevant when: selected( ${q104_M99_full_list} , '2')*<br>*Response constrained to: .>0* | |
| q304_M100 | ***q304_M100. Oxytocin***<br>*Question relevant when: selected( ${q104_M100_full_list} , '2')* | |
| q304_M100a *(required)* | q304_M100a. What percent of patients require this item?<br>*Question relevant when: selected( ${q104_M100_full_list} , '2')*<br>*Response constrained to: .>0 and .<=100 or .=999* | |

| Field | Question | Answer |
|---|---|---|
| q304_M100b *(required)* | q304_M100b. How is the item administered, or given, to the patient?<br>*Question relevant when: selected( ${q104_M100_full_list} , '2')* | 1 Oral - solid (e.g. tablet)<br>2 Oral - liquid<br>3 Sublingual<br>4 Intravenous (IV)<br>5 Intramusculuar injection (IM)<br>6 Subcutaneous<br>7 Rectal<br>8 Inhaled (e.g. gas)<br>9 Inserted (e.g. vaginal pessaries)<br>10 Topical - cream<br>11 Topical - drops (e.g. eye drops)<br>12 Other<br>99 Don't know |
| q304_M100c *(required)* | q304_M100c. What is the item's smallest unit of measurement (mg, mcg, ml, g, etc)?<br>*Question relevant when: selected( ${q104_M100_full_list} , '2')* | microgram: microgram (mcg)<br>miligram: miligram (mg)<br>miligram_dissolved_in_liquid: miligram (mg) dissolved in liquid (e.g. 2 mg per 3 militers)<br>gram: gram (g)<br>militer: militer (ml)<br>centiliter: centiliter (cl)<br>litre: litre (l)<br>ml_of _solution: ml of solution (e.g. ml of 10% solution)<br>litre_of_solution: litre of solution (e.g. 1 litre of 5% solution)<br>cubic_meter: cubic meter (m^3)<br>other: other<br>dont_know: don't know |
| q304_M100c.1 | q304_M100c.1 If other unit of measurement or route of administration, specify here:<br>*Leave blank if not applicable*<br>*Question relevant when: selected( ${q104_M100_full_list} , '2')* | |
| q304_M100d *(required)* | q304_M100d. In total, how many units are used to treat a patient during all of her care?<br>*Remember to add all units! E.g. 5 days x 200 mg twice per day = 2,000mg*<br>*Question relevant when: selected( ${q104_M100_full_list} , '2')*<br>*Response constrained to: .>0* | |
| q304_M101 | ***q304_M101. Prostaglandin, uterotonic, etc. - Other 1: "[q104_M101_full_list_other]"***<br>*Question relevant when: selected( ${q104_M101_full_list} , '2')* | |
| q304_M101a *(required)* | q304_M101a. What percent of patients require this item?<br>*Question relevant when: selected( ${q104_M101_full_list} , '2')*<br>*Response constrained to: .>0 and .<=100 or .=999* | |

| Field | Question | Answer |
|---|---|---|

| Field | Question | Answer |
|---|---|---|
| q304_M101b *(required)* | q304_M101b. How is the item administered, or given, to the patient?<br>*Question relevant when: selected( ${q104_M101_full_list} , '2')* | 1 Oral - solid (e.g. tablet)<br>2 Oral - liquid<br>3 Sublingual<br>4 Intravenous (IV)<br>5 Intramusculuar injection (IM)<br>6 Subcutaneous<br>7 Rectal<br>8 Inhaled (e.g. gas)<br>9 Inserted (e.g. vaginal pessaries)<br>10 Topical - cream<br>11 Topical - drops (e.g. eye drops)<br>12 Other<br>99 Don't know |
| q304_M101c *(required)* | q304_M101c. What is the item's smallest unit of measurement (mg, mcg, ml, g, etc)?<br>*Question relevant when: selected( ${q104_M101_full_list} , '2')* | microgram — microgram (mcg)<br>miligram — miligram (mg)<br>miligram_dissolved_in_liquid — miligram (mg) dissolved in liquid (e.g. 2 mg per 3 militers)<br>gram — gram (g)<br>militer — militer (ml)<br>centiliter — centiliter (cl)<br>litre — litre (l)<br>ml_of _solution — ml of solution (e.g. ml of 10% solution)<br>litre_of_solution — litre of solution (e.g. 1 litre of 5% solution)<br>cubic_meter — cubic meter (m^3)<br>other — other<br>dont_know — don't know |
| q304_M101c.1 | q304_M101c.1 If other unit of measurement or route of administration, specify here:<br>*Leave blank if not applicable*<br>*Question relevant when: selected( ${q104_M101_full_list} , '2')* | |
| q304_M101d *(required)* | q304_M101d. In total, how many units are used to treat a patient during all of her care?<br>*Remember to add all units! E.g. 5 days x 200 mg twice per day = 2,000mg*<br>*Question relevant when: selected( ${q104_M101_full_list} , '2')*<br>*Response constrained to: .>0* | |
| q304_M102 | ***q304_M102. Prostaglandin, uterotonic, etc. - Other 2: "[q104_M102_full_list_other]"***<br>*Question relevant when: selected( ${q104_M102_full_list} , '2')* | |
| q304_M102a *(required)* | q304_M102a. What percent of patients require this item?<br>*Question relevant when: selected( ${q104_M102_full_list} , '2')*<br>*Response constrained to: .>0 and .<=100 or .=999* | |

| Field | Question | Answer |
|---|---|---|
| q304_M102b *(required)* | q304_M102b. How is the item administered, or given, to the patient?<br>*Question relevant when: selected( ${q104_M102_full_list} , '2')* | 1 Oral - solid (e.g. tablet)<br>2 Oral - liquid<br>3 Sublingual<br>4 Intravenous (IV)<br>5 Intramusculuar injection (IM)<br>6 Subcutaneous<br>7 Rectal<br>8 Inhaled (e.g. gas)<br>9 Inserted (e.g. vaginal pessaries)<br>10 Topical - cream<br>11 Topical - drops (e.g. eye drops)<br>12 Other<br>99 Don't know |
| q304_M102c *(required)* | q304_M102c. What is the item's smallest unit of measurement (mg, mcg, ml, g, etc)?<br>*Question relevant when: selected( ${q104_M102_full_list} , '2')* | microgram — microgram (mcg)<br>miligram — miligram (mg)<br>miligram_dissolved_in_liquid — miligram (mg) dissolved in liquid (e.g. 2 mg per 3 militers)<br>gram — gram (g)<br>militer — militer (ml)<br>centiliter — centiliter (cl)<br>litre — litre (l)<br>ml_of _solution — ml of solution (e.g. ml of 10% solution)<br>litre_of_solution — litre of solution (e.g. 1 litre of 5% solution)<br>cubic_meter — cubic meter (m^3)<br>other — other<br>dont_know — don't know |
| q304_M102c.1 | q304_M102c.1 If other unit of measurement or route of administration, specify here:<br>*Leave blank if not applicable*<br>*Question relevant when: selected( ${q104_M102_full_list} , '2')* | |
| q304_M102d *(required)* | q304_M102d. In total, how many units are used to treat a patient during all of her care?<br>*Remember to add all units! E.g. 5 days x 200 mg twice per day = 2,000mg*<br>*Question relevant when: selected( ${q104_M102_full_list} , '2')*<br>*Response constrained to: .>0* | |
| q304_M103 | ***q304_M103. Prostaglandin, uterotonic, etc. - Other 3: "[q104_M103_full_list_other]"***<br>*Question relevant when: selected( ${q104_M104_full_list} , '2')* | |
| q304_M103a *(required)* | q304_M103a. What percent of patients require this item?<br>*Question relevant when: selected( ${q104_M104_full_list} , '2')*<br>*Response constrained to: .>0 and .<=100 or .=999* | |

| Field | Question | Answer |
|---|---|---|
| q304_M103b *(required)* | q304_M103b. How is the item administered, or given, to the patient? *Question relevant when: selected( ${q104_M104_full_list} , '2')* | 1 Oral - solid (e.g. tablet)<br>2 Oral - liquid<br>3 Sublingual<br>4 Intravenous (IV)<br>5 Intramusculuar injection (IM)<br>6 Subcutaneous<br>7 Rectal<br>8 Inhaled (e.g. gas)<br>9 Inserted (e.g. vaginal pessaries)<br>10 Topical - cream<br>11 Topical - drops (e.g. eye drops)<br>12 Other<br>99 Don't know |
| q304_M103c *(required)* | q304_M103c. What is the item's smallest unit of measurement (mg, mcg, ml, g, etc)? *Question relevant when: selected( ${q104_M104_full_list} , '2')* | microgram — microgram (mcg)<br>miligram — miligram (mg)<br>miligram_dissolved_in_liquid — miligram (mg) dissolved in liquid (e.g. 2 mg per 3 militers)<br>gram — gram (g)<br>militer — militer (ml)<br>centiliter — centiliter (cl)<br>litre — litre (l)<br>ml_of _solution — ml of solution (e.g. ml of 10% solution)<br>litre_of_solution — litre of solution (e.g. 1 litre of 5% solution)<br>cubic_meter — cubic meter (m^3)<br>other — other<br>dont_know — don't know |
| q304_M103c.1 | q304_M103c.1 If other unit of measurement or route of administration, specify here: *Leave blank if not applicable* *Question relevant when: selected( ${q104_M104_full_list} , '2')* | |
| q304_M103d *(required)* | q304_M103d. In total, how many units are used to treat a patient during all of her care? *Remember to add all units! E.g. 5 days x 200 mg twice per day = 2,000mg* *Question relevant when: selected( ${q104_M104_full_list} , '2')* *Response constrained to: .>0* | |
| q304_M104 | ***q304_M104. Prostaglandin, uterotonic, etc. - Other 4: "[q104_M104_full_list_other]"*** *Question relevant when: selected( ${q104_M104_full_list} , '2')* | |
| q304_M104a *(required)* | q304_M104a. What percent of patients require this item? *Question relevant when: selected( ${q104_M104_full_list} , '2')* *Response constrained to: .>0 and .<=100 or .=999* | |

| Field | Question | Answer |
|---|---|---|
| q304_M104b *(required)* | q304_M104b. How is the item administered, or given, to the patient?<br>*Question relevant when: selected( ${q104_M104_full_list} , '2')* | 1 Oral - solid (e.g. tablet)<br>2 Oral - liquid<br>3 Sublingual<br>4 Intravenous (IV)<br>5 Intramusculuar injection (IM)<br>6 Subcutaneous<br>7 Rectal<br>8 Inhaled (e.g. gas)<br>9 Inserted (e.g. vaginal pessaries)<br>10 Topical - cream<br>11 Topical - drops (e.g. eye drops)<br>12 Other<br>99 Don't know |
| q304_M104c *(required)* | q304_M104c. What is the item's smallest unit of measurement (mg, mcg, ml, g, etc)?<br>*Question relevant when: selected( ${q104_M104_full_list} , '2')* | microgram — microgram (mcg)<br>miligram — miligram (mg)<br>miligram_dissolved_in_liquid — miligram (mg) dissolved in liquid (e.g. 2 mg per 3 militers)<br>gram — gram (g)<br>militer — militer (ml)<br>centiliter — centiliter (cl)<br>litre — litre (l)<br>ml_of _solution — ml of solution (e.g. ml of 10% solution)<br>litre_of_solution — litre of solution (e.g. 1 litre of 5% solution)<br>cubic_meter — cubic meter (m^3)<br>other — other<br>dont_know — don't know |
| q304_M104c.1 | q304_M104c.1 If other unit of measurement or route of administration, specify here:<br>*Leave blank if not applicable*<br>*Question relevant when: selected( ${q104_M104_full_list} , '2')* | |
| q304_M104d *(required)* | q304_M104d. In total, how many units are used to treat a patient during all of her care?<br>*Remember to add all units! E.g. 5 days x 200 mg twice per day = 2,000mg*<br>*Question relevant when: selected( ${q104_M104_full_list} , '2')*<br>*Response constrained to: .>0* | |
| q304_M105 | ***q304_M105. Prostaglandin, uterotonic, etc. - Other 5: "[q104_M105_full_list_other]"***<br>*Question relevant when: selected( ${q104_M105_full_list} , '2')* | |
| q304_M105a *(required)* | q304_M105a. What percent of patients require this item?<br>*Question relevant when: selected( ${q104_M105_full_list} , '2')*<br>*Response constrained to: .>0 and .<=100 or .=999* | |

| Field | Question | Answer |
|---|---|---|
| q304_M105b *(required)* | q304_M105b. How is the item administered, or given, to the patient?<br>*Question relevant when: selected( ${q104_M105_full_list} , '2')* | 1 Oral - solid (e.g. tablet)<br>2 Oral - liquid<br>3 Sublingual<br>4 Intravenous (IV)<br>5 Intramusculuar injection (IM)<br>6 Subcutaneous<br>7 Rectal<br>8 Inhaled (e.g. gas)<br>9 Inserted (e.g. vaginal pessaries)<br>10 Topical - cream<br>11 Topical - drops (e.g. eye drops)<br>12 Other<br>99 Don't know |
| q304_M105c *(required)* | q304_M105c. What is the item's smallest unit of measurement (mg, mcg, ml, g, etc)?<br>*Question relevant when: selected( ${q104_M105_full_list} , '2')* | microgram — microgram (mcg)<br>miligram — miligram (mg)<br>miligram_dissolved_in_liquid — miligram (mg) dissolved in liquid (e.g. 2 mg per 3 militers)<br>gram — gram (g)<br>militer — militer (ml)<br>centiliter — centiliter (cl)<br>litre — litre (l)<br>ml_of _solution — ml of solution (e.g. ml of 10% solution)<br>litre_of_solution — litre of solution (e.g. 1 litre of 5% solution)<br>cubic_meter — cubic meter (m^3)<br>other — other<br>dont_know — don't know |
| q304_M105c.1 | q304_M105c.1 If other unit of measurement or route of administration, specify here:<br>*Leave blank if not applicable*<br>*Question relevant when: selected( ${q104_M105_full_list} , '2')* | |
| q304_M105d *(required)* | q304_M105d. In total, how many units are used to treat a patient during all of her care?<br>*Remember to add all units! E.g. 5 days x 200 mg twice per day = 2,000mg*<br>*Question relevant when: selected( ${q104_M105_full_list} , '2')*<br>*Response constrained to: .>0* | |
| <p><strong><em>D. Medications - Sepsis (11)</em></strong></p><br>*Group relevant when: selected( ${section_three_skip_med} , '1')* | | |
| q304_med_notE10 | 304 CONTINUED For each of the following medications and other medical products that are used for sepsis, can you tell me …? | |
| note_304_vitamin | ***Vitamin, immunological, etc.*** | |

| Field | Question | Answer |
|---|---|---|
| q304_M106 | ***q304_M106. Ferrous sulphate (tablets)***<br>*Question relevant when: selected( ${q104_M106_full_list} , '2')* | |
| q304_M106a *(required)* | q304_M106a. What percent of patients require this item?<br>*Question relevant when: selected( ${q104_M106_full_list} , '2')*<br>*Response constrained to: .>0 and .<=100 or .=999* | |

| Field | Question | Answer |
|---|---|---|
| q304_M106b *(required)* | q304_M106b. How is the item administered, or given, to the patient?<br>*Question relevant when: selected( ${q104_M106_full_list} , '2')* | 1 Oral - solid (e.g. tablet)<br>2 Oral - liquid<br>3 Sublingual<br>4 Intravenous (IV)<br>5 Intramusculuar injection (IM)<br>6 Subcutaneous<br>7 Rectal<br>8 Inhaled (e.g. gas)<br>9 Inserted (e.g. vaginal pessaries)<br>10 Topical - cream<br>11 Topical - drops (e.g. eye drops)<br>12 Other<br>99 Don't know |
| q304_M106c *(required)* | q304_M106c. What is the item's smallest unit of measurement (mg, mcg, ml, g, etc)?<br>*Question relevant when: selected( ${q104_M106_full_list} , '2')* | microgram — microgram (mcg)<br>miligram — miligram (mg)<br>miligram_dissolved_in_liquid — miligram (mg) dissolved in liquid (e.g. 2 mg per 3 militers)<br>gram — gram (g)<br>militer — militer (ml)<br>centiliter — centiliter (cl)<br>litre — litre (l)<br>ml_of _solution — ml of solution (e.g. ml of 10% solution)<br>litre_of_solution — litre of solution (e.g. 1 litre of 5% solution)<br>cubic_meter — cubic meter (m^3)<br>other — other<br>dont_know — don't know |
| q304_M106c.1 | q304_M106c.1 If other unit of measurement or route of administration, specify here:<br>*Leave blank if not applicable*<br>*Question relevant when: selected( ${q104_M106_full_list} , '2')* | |
| q304_M106d *(required)* | q304_M106d. In total, how many units are used to treat a patient during all of her care?<br>*Remember to add all units! E.g. 5 days x 200 mg twice per day = 2,000mg*<br>*Question relevant when: selected( ${q104_M106_full_list} , '2')*<br>*Response constrained to: .>0* | |
| q304_M107 | ***q304_M107. Ferrous sulphate + folic acid (Ferrotone capsules)***<br>*Question relevant when: selected( ${q104_M107_full_list} , '2')* | |
| q304_M107a *(required)* | q304_M107a. What percent of patients require this item?<br>*Question relevant when: selected( ${q104_M107_full_list} , '2')*<br>*Response constrained to: .>0 and .<=100 or .=999* | |

| Field | Question | Answer |
|---|---|---|
| q304_M107b *(required)* | q304_M107b. How is the item administered, or given, to the patient?<br>*Question relevant when: selected( ${q104_M107_full_list} , '2')* | 1 Oral - solid (e.g. tablet)<br>2 Oral - liquid<br>3 Sublingual<br>4 Intravenous (IV)<br>5 Intramusculuar injection (IM)<br>6 Subcutaneous<br>7 Rectal<br>8 Inhaled (e.g. gas)<br>9 Inserted (e.g. vaginal pessaries)<br>10 Topical - cream<br>11 Topical - drops (e.g. eye drops)<br>12 Other<br>99 Don't know |
| q304_M107c *(required)* | q304_M107c. What is the item's smallest unit of measurement (mg, mcg, ml, g, etc)?<br>*Question relevant when: selected( ${q104_M107_full_list} , '2')* | microgram: microgram (mcg)<br>miligram: miligram (mg)<br>miligram_dissolved_in_liquid: miligram (mg) dissolved in liquid (e.g. 2 mg per 3 militers)<br>gram: gram (g)<br>militer: militer (ml)<br>centiliter: centiliter (cl)<br>litre: litre (l)<br>ml_of _solution: ml of solution (e.g. ml of 10% solution)<br>litre_of_solution: litre of solution (e.g. 1 litre of 5% solution)<br>cubic_meter: cubic meter (m^3)<br>other: other<br>dont_know: don't know |
| q304_M107c.1 | q304_M107c.1 If other unit of measurement or route of administration, specify here:<br>*Leave blank if not applicable*<br>*Question relevant when: selected( ${q104_M107_full_list} , '2')* | |
| q304_M107d *(required)* | q304_M107d. In total, how many units are used to treat a patient during all of her care?<br>*Remember to add all units! E.g. 5 days x 200 mg twice per day = 2,000mg*<br>*Question relevant when: selected( ${q104_M107_full_list} , '2')*<br>*Response constrained to: .>0* | |
| q304_M108 | ***q304_M108. Folic acid (Tablets)***<br>*Question relevant when: selected( ${q104_M108_full_list} , '2')* | |
| q304_M108a *(required)* | q304_M108a. What percent of patients require this item?<br>*Question relevant when: selected( ${q104_M108_full_list} , '2')*<br>*Response constrained to: .>0 and .<=100 or .=999* | |

| Field | Question | Answer |
|---|---|---|
| q304_M108b *(required)* | q304_M108b. How is the item administered, or given, to the patient?<br>*Question relevant when: selected( ${q104_M108_full_list} , '2')* | 1 Oral - solid (e.g. tablet)<br>2 Oral - liquid<br>3 Sublingual<br>4 Intravenous (IV)<br>5 Intramusculuar injection (IM)<br>6 Subcutaneous<br>7 Rectal<br>8 Inhaled (e.g. gas)<br>9 Inserted (e.g. vaginal pessaries)<br>10 Topical - cream<br>11 Topical - drops (e.g. eye drops)<br>12 Other<br>99 Don't know |
| q304_M108c *(required)* | q304_M108c. What is the item's smallest unit of measurement (mg, mcg, ml, g, etc)?<br>*Question relevant when: selected( ${q104_M108_full_list} , '2')* | microgram — microgram (mcg)<br>miligram — miligram (mg)<br>miligram_dissolved_in_liquid — miligram (mg) dissolved in liquid (e.g. 2 mg per 3 militers)<br>gram — gram (g)<br>militer — militer (ml)<br>centiliter — centiliter (cl)<br>litre — litre (l)<br>ml_of _solution — ml of solution (e.g. ml of 10% solution)<br>litre_of_solution — litre of solution (e.g. 1 litre of 5% solution)<br>cubic_meter — cubic meter (m^3)<br>other — other<br>dont_know — don't know |
| q304_M108c.1 | q304_M108c.1 If other unit of measurement or route of administration, specify here:<br>*Leave blank if not applicable*<br>*Question relevant when: selected( ${q104_M108_full_list} , '2')* | |
| q304_M108d *(required)* | q304_M108d. In total, how many units are used to treat a patient during all of her care?<br>*Remember to add all units! E.g. 5 days x 200 mg twice per day = 2,000mg*<br>*Question relevant when: selected( ${q104_M108_full_list} , '2')*<br>*Response constrained to: .>0* | |
| q304_M109 | ***q304_M109. Serum anti-tetanus***<br>*Question relevant when: selected( ${q104_M109_full_list} , '2')* | |
| q304_M109a *(required)* | q304_M109a. What percent of patients require this item?<br>*Question relevant when: selected( ${q104_M109_full_list} , '2')*<br>*Response constrained to: .>0 and .<=100 or .=999* | |

| Field | Question | Answer |
|---|---|---|
| q304_M109b *(required)* | q304_M109b. How is the item administered, or given, to the patient?<br>*Question relevant when: selected( ${q104_M109_full_list} , '2')* | 1 Oral - solid (e.g. tablet)<br>2 Oral - liquid<br>3 Sublingual<br>4 Intravenous (IV)<br>5 Intramusculuar injection (IM)<br>6 Subcutaneous<br>7 Rectal<br>8 Inhaled (e.g. gas)<br>9 Inserted (e.g. vaginal pessaries)<br>10 Topical - cream<br>11 Topical - drops (e.g. eye drops)<br>12 Other<br>99 Don't know |
| q304_M109c *(required)* | q304_M109c. What is the item's smallest unit of measurement (mg, mcg, ml, g, etc)?<br>*Question relevant when: selected( ${q104_M109_full_list} , '2')* | microgram: microgram (mcg)<br>miligram: miligram (mg)<br>miligram_dissolved_in_liquid: miligram (mg) dissolved in liquid (e.g. 2 mg per 3 militers)<br>gram: gram (g)<br>militer: militer (ml)<br>centiliter: centiliter (cl)<br>litre: litre (l)<br>ml_of _solution: ml of solution (e.g. ml of 10% solution)<br>litre_of_solution: litre of solution (e.g. 1 litre of 5% solution)<br>cubic_meter: cubic meter (m^3)<br>other: other<br>dont_know: don't know |
| q304_M109c.1 | q304_M109c.1 If other unit of measurement or route of administration, specify here:<br>*Leave blank if not applicable*<br>*Question relevant when: selected( ${q104_M109_full_list} , '2')* | |
| q304_M109d *(required)* | q304_M109d. In total, how many units are used to treat a patient during all of her care?<br>*Remember to add all units! E.g. 5 days x 200 mg twice per day = 2,000mg*<br>*Question relevant when: selected( ${q104_M109_full_list} , '2')*<br>*Response constrained to: .>0* | |
| q304_M110 | ***q304_M110. Anti D (Rh)-Immunoglobulin***<br>*Question relevant when: selected( ${q104_M110_full_list} , '2')* | |
| q304_M110a *(required)* | q304_M110a. What percent of patients require this item?<br>*Question relevant when: selected( ${q104_M110_full_list} , '2')*<br>*Response constrained to: .>0 and .<=100 or .=999* | |

| Field | Question | Answer |
|---|---|---|
| q304_M110b *(required)* | q304_M110b. How is the item administered, or given, to the patient?<br>*Question relevant when: selected( ${q104_M110_full_list} , '2')* | 1 Oral - solid (e.g. tablet)<br>2 Oral - liquid<br>3 Sublingual<br>4 Intravenous (IV)<br>5 Intramusculuar injection (IM)<br>6 Subcutaneous<br>7 Rectal<br>8 Inhaled (e.g. gas)<br>9 Inserted (e.g. vaginal pessaries)<br>10 Topical - cream<br>11 Topical - drops (e.g. eye drops)<br>12 Other<br>99 Don't know |
| q304_M110c *(required)* | q304_M110c. What is the item's smallest unit of measurement (mg, mcg, ml, g, etc)?<br>*Question relevant when: selected( ${q104_M110_full_list} , '2')* | microgram — microgram (mcg)<br>miligram — miligram (mg)<br>miligram_dissolved_in_liquid — miligram (mg) dissolved in liquid (e.g. 2 mg per 3 militers)<br>gram — gram (g)<br>militer — militer (ml)<br>centiliter — centiliter (cl)<br>litre — litre (l)<br>ml_of _solution — ml of solution (e.g. ml of 10% solution)<br>litre_of_solution — litre of solution (e.g. 1 litre of 5% solution)<br>cubic_meter — cubic meter (m^3)<br>other — other<br>dont_know — don't know |
| q304_M110c.1 | q304_M110c.1 If other unit of measurement or route of administration, specify here:<br>*Leave blank if not applicable*<br>*Question relevant when: selected( ${q104_M110_full_list} , '2')* | |
| q304_M110d *(required)* | q304_M110d. In total, how many units are used to treat a patient during all of her care?<br>*Remember to add all units! E.g. 5 days x 200 mg twice per day = 2,000mg*<br>*Question relevant when: selected( ${q104_M110_full_list} , '2')*<br>*Response constrained to: .>0* | |
| q304_M111 | ***q304_M111. Vitamin, immunological, etc. - Other 1: "[q104_M111_full_list_other]"***<br>*Question relevant when: selected( ${q104_M111_full_list} , '2')* | |
| q304_M111a *(required)* | q304_M111a. What percent of patients require this item?<br>*Question relevant when: selected( ${q104_M111_full_list} , '2')*<br>*Response constrained to: .>0 and .<=100 or .=999* | |

| Field | Question | Answer |
|---|---|---|
| q304_M111b *(required)* | q304_M111b. How is the item administered, or given, to the patient?<br>*Question relevant when: selected( ${q104_M111_full_list} , '2')* | 1 Oral - solid (e.g. tablet)<br>2 Oral - liquid<br>3 Sublingual<br>4 Intravenous (IV)<br>5 Intramusculuar injection (IM)<br>6 Subcutaneous<br>7 Rectal<br>8 Inhaled (e.g. gas)<br>9 Inserted (e.g. vaginal pessaries)<br>10 Topical - cream<br>11 Topical - drops (e.g. eye drops)<br>12 Other<br>99 Don't know |
| q304_M111c *(required)* | q304_M111c. What is the item's smallest unit of measurement (mg, mcg, ml, g, etc)?<br>*Question relevant when: selected( ${q104_M111_full_list} , '2')* | microgram — microgram (mcg)<br>miligram — miligram (mg)<br>miligram_dissolved_in_liquid — miligram (mg) dissolved in liquid (e.g. 2 mg per 3 militers)<br>gram — gram (g)<br>militer — militer (ml)<br>centiliter — centiliter (cl)<br>litre — litre (l)<br>ml_of _solution — ml of solution (e.g. ml of 10% solution)<br>litre_of_solution — litre of solution (e.g. 1 litre of 5% solution)<br>cubic_meter — cubic meter (m^3)<br>other — other<br>dont_know — don't know |
| q304_M111c.1 | q304_M111c.1 If other unit of measurement or route of administration, specify here:<br>*Leave blank if not applicable*<br>*Question relevant when: selected( ${q104_M111_full_list} , '2')* | |
| q304_M111d *(required)* | q304_M111d. In total, how many units are used to treat a patient during all of her care?<br>*Remember to add all units! E.g. 5 days x 200 mg twice per day = 2,000mg*<br>*Question relevant when: selected( ${q104_M111_full_list} , '2')*<br>*Response constrained to: .>0* | |
| q304_M112 | ***q304_M112. Vitamin, immunological, etc. - Other 2: "[q104_M112_full_list_other]"***<br>*Question relevant when: selected( ${q104_M112_full_list} , '2')* | |
| q304_M112a *(required)* | q304_M112a. What percent of patients require this item?<br>*Question relevant when: selected( ${q104_M112_full_list} , '2')*<br>*Response constrained to: .>0 and .<=100 or .=999* | |

| Field | Question | Answer |
|---|---|---|
| q304_M112b *(required)* | q304_M112b. How is the item administered, or given, to the patient?<br>*Question relevant when: selected( ${q104_M112_full_list} , '2')* | 1 Oral - solid (e.g. tablet)<br>2 Oral - liquid<br>3 Sublingual<br>4 Intravenous (IV)<br>5 Intramusculuar injection (IM)<br>6 Subcutaneous<br>7 Rectal<br>8 Inhaled (e.g. gas)<br>9 Inserted (e.g. vaginal pessaries)<br>10 Topical - cream<br>11 Topical - drops (e.g. eye drops)<br>12 Other<br>99 Don't know |
| q304_M112c *(required)* | q304_M112c. What is the item's smallest unit of measurement (mg, mcg, ml, g, etc)?<br>*Question relevant when: selected( ${q104_M112_full_list} , '2')* | microgram — microgram (mcg)<br>miligram — miligram (mg)<br>miligram_dissolved_in_liquid — miligram (mg) dissolved in liquid (e.g. 2 mg per 3 militers)<br>gram — gram (g)<br>militer — militer (ml)<br>centiliter — centiliter (cl)<br>litre — litre (l)<br>ml_of _solution — ml of solution (e.g. ml of 10% solution)<br>litre_of_solution — litre of solution (e.g. 1 litre of 5% solution)<br>cubic_meter — cubic meter (m^3)<br>other — other<br>dont_know — don't know |
| q304_M112c.1 | q304_M112c.1 If other unit of measurement or route of administration, specify here:<br>*Leave blank if not applicable*<br>*Question relevant when: selected( ${q104_M112_full_list} , '2')* | |
| q304_M112d *(required)* | q304_M112d. In total, how many units are used to treat a patient during all of her care?<br>*Remember to add all units! E.g. 5 days x 200 mg twice per day = 2,000mg*<br>*Question relevant when: selected( ${q104_M112_full_list} , '2')*<br>*Response constrained to: .>0* | |
| q304_M113 | ***q304_M113. Vitamin, immunological, etc. - Other 3: "[q104_M113_full_list_other]"***<br>*Question relevant when: selected( ${q104_M113_full_list} , '2')* | |
| q304_M113a *(required)* | q304_M113a. What percent of patients require this item?<br>*Question relevant when: selected( ${q104_M113_full_list} , '2')*<br>*Response constrained to: .>0 and .<=100 or .=999* | |

| Field | Question | Answer |
|---|---|---|
| q304_M113b *(required)* | q304_M113b. How is the item administered, or given, to the patient?<br>*Question relevant when: selected( ${q104_M113_full_list} , '2')* | 1 Oral - solid (e.g. tablet)<br>2 Oral - liquid<br>3 Sublingual<br>4 Intravenous (IV)<br>5 Intramusculuar injection (IM)<br>6 Subcutaneous<br>7 Rectal<br>8 Inhaled (e.g. gas)<br>9 Inserted (e.g. vaginal pessaries)<br>10 Topical - cream<br>11 Topical - drops (e.g. eye drops)<br>12 Other<br>99 Don't know |
| q304_M113c *(required)* | q304_M113c. What is the item's smallest unit of measurement (mg, mcg, ml, g, etc)?<br>*Question relevant when: selected( ${q104_M113_full_list} , '2')* | microgram — microgram (mcg)<br>miligram — miligram (mg)<br>miligram_dissolved_in_liquid — miligram (mg) dissolved in liquid (e.g. 2 mg per 3 militers)<br>gram — gram (g)<br>militer — militer (ml)<br>centiliter — centiliter (cl)<br>litre — litre (l)<br>ml_of _solution — ml of solution (e.g. ml of 10% solution)<br>litre_of_solution — litre of solution (e.g. 1 litre of 5% solution)<br>cubic_meter — cubic meter (m^3)<br>other — other<br>dont_know — don't know |
| q304_M113c.1 | q304_M113c.1 If other unit of measurement or route of administration, specify here:<br>*Leave blank if not applicable*<br>*Question relevant when: selected( ${q104_M113_full_list} , '2')* | |
| q304_M113d *(required)* | q304_M113d. In total, how many units are used to treat a patient during all of her care?<br>*Remember to add all units! E.g. 5 days x 200 mg twice per day = 2,000mg*<br>*Question relevant when: selected( ${q104_M113_full_list} , '2')*<br>*Response constrained to: .>0* | |
| q304_M114 | ***q304_M114. Vitamin, immunological, etc. - Other 4: "[q104_M114_full_list_other]"***<br>*Question relevant when: selected( ${q104_M114_full_list} , '2')* | |
| q304_M114a *(required)* | q304_M114a. What percent of patients require this item?<br>*Question relevant when: selected( ${q104_M114_full_list} , '2')*<br>*Response constrained to: .>0 and .<=100 or .=999* | |

| Field | Question | Answer |
|---|---|---|
| q304_M114b *(required)* | q304_M114b. How is the item administered, or given, to the patient?<br>*Question relevant when: selected( ${q104_M114_full_list} , '2')* | 1 Oral - solid (e.g. tablet)<br>2 Oral - liquid<br>3 Sublingual<br>4 Intravenous (IV)<br>5 Intramusculuar injection (IM)<br>6 Subcutaneous<br>7 Rectal<br>8 Inhaled (e.g. gas)<br>9 Inserted (e.g. vaginal pessaries)<br>10 Topical - cream<br>11 Topical - drops (e.g. eye drops)<br>12 Other<br>99 Don't know |
| q304_M114c *(required)* | q304_M114c. What is the item's smallest unit of measurement (mg, mcg, ml, g, etc)?<br>*Question relevant when: selected( ${q104_M114_full_list} , '2')* | microgram — microgram (mcg)<br>miligram — miligram (mg)<br>miligram_dissolved_in_liquid — miligram (mg) dissolved in liquid (e.g. 2 mg per 3 militers)<br>gram — gram (g)<br>militer — militer (ml)<br>centiliter — centiliter (cl)<br>litre — litre (l)<br>ml_of _solution — ml of solution (e.g. ml of 10% solution)<br>litre_of_solution — litre of solution (e.g. 1 litre of 5% solution)<br>cubic_meter — cubic meter (m^3)<br>other — other<br>dont_know — don't know |
| q304_M114c.1 | q304_M114c.1 If other unit of measurement or route of administration, specify here:<br>*Leave blank if not applicable*<br>*Question relevant when: selected( ${q104_M114_full_list} , '2')* | |
| q304_M114d *(required)* | q304_M114d. In total, how many units are used to treat a patient during all of her care?<br>*Remember to add all units! E.g. 5 days x 200 mg twice per day = 2,000mg*<br>*Question relevant when: selected( ${q104_M114_full_list} , '2')*<br>*Response constrained to: .>0* | |
| q304_M115 | ***q304_M115. Vitamin, immunological, etc. - Other 5: "[q104_M115_full_list_other]"***<br>*Question relevant when: selected( ${q104_M115_full_list} , '2')* | |
| q304_M115a *(required)* | q304_M115a. What percent of patients require this item?<br>*Question relevant when: selected( ${q104_M115_full_list} , '2')*<br>*Response constrained to: .>0 and .<=100 or .=999* | |

| Field | Question | Answer |
|---|---|---|
| q304_M115b *(required)* | q304_M115b. How is the item administered, or given, to the patient?<br>*Question relevant when: selected( ${q104_M115_full_list} , '2')* | 1 Oral - solid (e.g. tablet)<br>2 Oral - liquid<br>3 Sublingual<br>4 Intravenous (IV)<br>5 Intramusculuar injection (IM)<br>6 Subcutaneous<br>7 Rectal<br>8 Inhaled (e.g. gas)<br>9 Inserted (e.g. vaginal pessaries)<br>10 Topical - cream<br>11 Topical - drops (e.g. eye drops)<br>12 Other<br>99 Don't know |
| q304_M115c *(required)* | q304_M115c. What is the item's smallest unit of measurement (mg, mcg, ml, g, etc)?<br>*Question relevant when: selected( ${q104_M115_full_list} , '2')* | microgram: microgram (mcg)<br>miligram: miligram (mg)<br>miligram_dissolved_in_liquid: miligram (mg) dissolved in liquid (e.g. 2 mg per 3 militers)<br>gram: gram (g)<br>militer: militer (ml)<br>centiliter: centiliter (cl)<br>litre: litre (l)<br>ml_of _solution: ml of solution (e.g. ml of 10% solution)<br>litre_of_solution: litre of solution (e.g. 1 litre of 5% solution)<br>cubic_meter: cubic meter (m^3)<br>other: other<br>dont_know: don't know |
| q304_M115c.1 | q304_M115c.1 If other unit of measurement or route of administration, specify here:<br>*Leave blank if not applicable*<br>*Question relevant when: selected( ${q104_M115_full_list} , '2')* | |
| q304_M115d *(required)* | q304_M115d. In total, how many units are used to treat a patient during all of her care?<br>*Remember to add all units! E.g. 5 days x 200 mg twice per day = 2,000mg*<br>*Question relevant when: selected( ${q104_M115_full_list} , '2')*<br>*Response constrained to: .>0* | |
| <p><strong><em>D. Medications - Sepsis (12)</em></strong></p><br>*Group relevant when: selected( ${section_three_skip_med} , '1')* | | |
| q304_med_notE11 | 304 CONTINUED For each of the following medications and other medical products that are used for sepsis, can you tell me …? | |
| note_304_Other | ***Other medication, any class*** | |

| Field | Question | Answer |
|---|---|---|
| q304_M116 | ***q304_M116. Medication - any class - Other 1: "[q104_M116_full_list_other]"***<br>*Question relevant when: selected( ${q104_M116_full_list} , '2')* | |
| q304_M116a *(required)* | q304_M116a. What percent of patients require this item?<br>*Question relevant when: selected( ${q104_M116_full_list} , '2')*<br>*Response constrained to: .>0 and .<=100 or .=999* | |

| | | |
|---|---|---|
| q304_M116b *(required)* | q304_M116b. How is the item administered, or given, to the patient?<br>*Question relevant when: selected( ${q104_M116_full_list} , '2')* | 1 Oral - solid (e.g. tablet)<br>2 Oral - liquid<br>3 Sublingual<br>4 Intravenous (IV)<br>5 Intramusculuar injection (IM)<br>6 Subcutaneous<br>7 Rectal<br>8 Inhaled (e.g. gas)<br>9 Inserted (e.g. vaginal pessaries)<br>10 Topical - cream<br>11 Topical - drops (e.g. eye drops)<br>12 Other<br>99 Don't know |
| q304_M116c *(required)* | q304_M116c. What is the item's smallest unit of measurement (mg, mcg, ml, g, etc)?<br>*Question relevant when: selected( ${q104_M116_full_list} , '2')* | microgram — microgram (mcg)<br>miligram — miligram (mg)<br>miligram_dissolved_in_liquid — miligram (mg) dissolved in liquid (e.g. 2 mg per 3 militers)<br>gram — gram (g)<br>militer — militer (ml)<br>centiliter — centiliter (cl)<br>litre — litre (l)<br>ml_of _solution — ml of solution (e.g. ml of 10% solution)<br>litre_of_solution — litre of solution (e.g. 1 litre of 5% solution)<br>cubic_meter — cubic meter (m^3)<br>other — other<br>dont_know — don't know |
| q304_M116c.1 | q304_M116c.1 If other unit of measurement or route of administration, specify here:<br>*Leave blank if not applicable*<br>*Question relevant when: selected( ${q104_M116_full_list} , '2')* | |
| q304_M116d *(required)* | q304_M116d. In total, how many units are used to treat a patient during all of her care?<br>*Remember to add all units! E.g. 5 days x 200 mg twice per day = 2,000mg*<br>*Question relevant when: selected( ${q104_M116_full_list} , '2')*<br>*Response constrained to: .>0* | |
| q304_M117 | ***q304_M117. Medication - any class - Other 2: "[q104_M117_full_list_other]"***<br>*Question relevant when: selected( ${q104_M117_full_list} , '2')* | |
| q304_M117a *(required)* | q304_M117a. What percent of patients require this item?<br>[illegible] | |

| Field | Question | Answer |
|---|---|---|
| | *Question relevant when: selected( ${q104_M117_full_list} , '2')*<br>*Response constrained to: .>0 and .<=100 or .=999* | |
| q304_M117b *(required)* | q304_M117b. How is the item administered, or given, to the patient?<br>*Question relevant when: selected( ${q104_M117_full_list} , '2')* | 1 Oral - solid (e.g. tablet)<br>2 Oral - liquid<br>3 Sublingual<br>4 Intravenous (IV)<br>5 Intramusculuar injection (IM)<br>6 Subcutaneous<br>7 Rectal<br>8 Inhaled (e.g. gas)<br>9 Inserted (e.g. vaginal pessaries)<br>10 Topical - cream<br>11 Topical - drops (e.g. eye drops)<br>12 Other<br>99 Don't know |
| q304_M117c *(required)* | q304_M117c. What is the item's smallest unit of measurement (mg, mcg, ml, g, etc)?<br>*Question relevant when: selected( ${q104_M117_full_list} , '2')* | microgram — microgram (mcg)<br>miligram — miligram (mg)<br>miligram_dissolved_in_liquid — miligram (mg) dissolved in liquid (e.g. 2 mg per 3 militers)<br>gram — gram (g)<br>militer — militer (ml)<br>centiliter — centiliter (cl)<br>litre — litre (l)<br>ml_of _solution — ml of solution (e.g. ml of 10% solution)<br>litre_of_solution — litre of solution (e.g. 1 litre of 5% solution)<br>cubic_meter — cubic meter (m^3)<br>other — other<br>dont_know — don't know |
| q304_M117c.1 | q304_M117c.1 If other unit of measurement or route of administration, specify here:<br>*Leave blank if not applicable*<br>*Question relevant when: selected( ${q104_M117_full_list} , '2')* | |
| q304_M117d *(required)* | q304_M117d. In total, how many units are used to treat a patient during all of her care?<br>*Remember to add all units! E.g. 5 days x 200 mg twice per day = 2,000mg*<br>*Question relevant when: selected( ${q104_M117_full_list} , '2')*<br>*Response constrained to: .>0* | |
| q304_M118 | ***q304_M118. Medication - any class - Other 3: "[q104_M118_full_list_other]"***<br>*Question relevant when: selected( ${q104_M118_full_list} , '2')* | |
| q304_M118a *(required)* | q304_M118a. What percent of patients require this item?<br>*Question relevant when: selected( ${q104_M118_full_list} , '2')* | |

| Field | Question | Answer |
|---|---|---|
| | *Question relevant when: selected( ${q104_M118_full_list} , '2')*<br>*Response constrained to: .>0 and .<=100 or .=999* | |
| q304_M118b *(required)* | q304_M118b. How is the item administered, or given, to the patient?<br>*Question relevant when: selected( ${q104_M118_full_list} , '2')* | 1 Oral - solid (e.g. tablet)<br>2 Oral - liquid<br>3 Sublingual<br>4 Intravenous (IV)<br>5 Intramusculuar injection (IM)<br>6 Subcutaneous<br>7 Rectal<br>8 Inhaled (e.g. gas)<br>9 Inserted (e.g. vaginal pessaries)<br>10 Topical - cream<br>11 Topical - drops (e.g. eye drops)<br>12 Other<br>99 Don't know |
| q304_M118c *(required)* | q304_M118c. What is the item's smallest unit of measurement (mg, mcg, ml, g, etc)?<br>*Question relevant when: selected( ${q104_M118_full_list} , '2')* | microgram — microgram (mcg)<br>miligram — miligram (mg)<br>miligram_dissolved_in_liquid — miligram (mg) dissolved in liquid (e.g. 2 mg per 3 militers)<br>gram — gram (g)<br>militer — militer (ml)<br>centiliter — centiliter (cl)<br>litre — litre (l)<br>ml_of _solution — ml of solution (e.g. ml of 10% solution)<br>litre_of_solution — litre of solution (e.g. 1 litre of 5% solution)<br>cubic_meter — cubic meter (m^3)<br>other — other<br>dont_know — don't know |
| q304_M118c.1 | q304_M118c.1 If other unit of measurement or route of administration, specify here:<br>*Leave blank if not applicable*<br>*Question relevant when: selected( ${q104_M118_full_list} , '2')* | |
| q304_M118d *(required)* | q304_M118d. In total, how many units are used to treat a patient during all of her care?<br>*Remember to add all units! E.g. 5 days x 200 mg twice per day = 2,000mg*<br>*Question relevant when: selected( ${q104_M118_full_list} , '2')*<br>*Response constrained to: .>0* | |
| q304_M119 | ***q304_M119. Medication - any class - Other 4: "[q104_M119_full_list_other]"***<br>*Question relevant when: selected( ${q104_M119_full_list} , '2')* | |
| q304_M119a *(required)* | q304_M119a. What percent of patients require this item?<br>*Question relevant when: selected( ${q104_M119_full_list} , '2')* | |

| Field | Question | Answer |
|---|---|---|
| | *Question relevant when: selected( ${q104_M119_full_list} , '2')*<br>*Response constrained to: .>0 and .<=100 or .=999* | |
| q304_M119b *(required)* | q304_M119b. How is the item administered, or given, to the patient?<br>*Question relevant when: selected( ${q104_M119_full_list} , '2')* | 1 Oral - solid (e.g. tablet)<br>2 Oral - liquid<br>3 Sublingual<br>4 Intravenous (IV)<br>5 Intramuscular injection (IM)<br>6 Subcutaneous<br>7 Rectal<br>8 Inhaled (e.g. gas)<br>9 Inserted (e.g. vaginal pessaries)<br>10 Topical - cream<br>11 Topical - drops (e.g. eye drops)<br>12 Other<br>99 Don't know |
| q304_M119c *(required)* | q304_M119c. What is the item's smallest unit of measurement (mg, mcg, ml, g, etc)?<br>*Question relevant when: selected( ${q104_M119_full_list} , '2')* | microgram — microgram (mcg)<br>miligram — miligram (mg)<br>miligram_dissolved_in_liquid — miligram (mg) dissolved in liquid (e.g. 2 mg per 3 militers)<br>gram — gram (g)<br>militer — militer (ml)<br>centiliter — centiliter (cl)<br>litre — litre (l)<br>ml_of _solution — ml of solution (e.g. ml of 10% solution)<br>litre_of_solution — litre of solution (e.g. 1 litre of 5% solution)<br>cubic_meter — cubic meter (m^3)<br>other — other<br>dont_know — don't know |
| q304_M119c.1 | q304_M119c.1 If other unit of measurement or route of administration, specify here:<br>*Leave blank if not applicable*<br>*Question relevant when: selected( ${q104_M119_full_list} , '2')* | |
| q304_M119d *(required)* | q304_M119d. In total, how many units are used to treat a patient during all of her care?<br>*Remember to add all units! E.g. 5 days x 200 mg twice per day = 2,000mg*<br>*Question relevant when: selected( ${q104_M119_full_list} , '2')*<br>*Response constrained to: .>0* | |
| q304_M120 | ***q304_M120. Medication - any class - Other 5: "[q104_M120_full_list_other]"***<br>*Question relevant when: selected( ${q104_M120_full_list} , '2')* | |
| q304_M120a *(required)* | q304_M120a. What percent of patients require this item?<br>*Question relevant when: selected( ${q104_M120_full_list} , '2')* | |

Question relevant when: selected( ${q104_M120_full_list} , '2')

| Field | Question | Answer |
|---|---|---|
| | Response constrained to: .>0 and .<=100 or .=999 | |
| q304_M120b *(required)* | q304_M120b. How is the item administered, or given, to the patient?<br>*Question relevant when: selected( ${q104_M120_full_list} , '2')* | 1 Oral - solid (e.g. tablet)<br>2 Oral - liquid<br>3 Sublingual<br>4 Intravenous (IV)<br>5 Intramusculuar injection (IM)<br>6 Subcutaneous<br>7 Rectal<br>8 Inhaled (e.g. gas)<br>9 Inserted (e.g. vaginal pessaries)<br>10 Topical - cream<br>11 Topical - drops (e.g. eye drops)<br>12 Other<br>99 Don't know |
| q304_M120c *(required)* | q304_M120c. What is the item's smallest unit of measurement (mg, mcg, ml, g, etc)?<br>*Question relevant when: selected( ${q104_M120_full_list} , '2')* | microgram — microgram (mcg)<br>miligram — miligram (mg)<br>miligram_dissolved_in_liquid — miligram (mg) dissolved in liquid (e.g. 2 mg per 3 militers)<br>gram — gram (g)<br>militer — militer (ml)<br>centiliter — centiliter (cl)<br>litre — litre (l)<br>ml_of _solution — ml of solution (e.g. ml of 10% solution)<br>litre_of_solution — litre of solution (e.g. 1 litre of 5% solution)<br>cubic_meter — cubic meter (m^3)<br>other — other<br>dont_know — don't know |
| q304_M120c.1 | q304_M120c.1 If other unit of measurement or route of administration, specify here:<br>*Leave blank if not applicable*<br>*Question relevant when: selected( ${q104_M120_full_list} , '2')* | |
| q304_M120d *(required)* | q304_M120d. In total, how many units are used to treat a patient during all of her care?<br>*Remember to add all units! E.g. 5 days x 200 mg twice per day = 2,000mg*<br>*Question relevant when: selected( ${q104_M120_full_list} , '2')*<br>*Response constrained to: .>0* | |
| group_section_four_intro | | |
| section4_start | **SECTION IV. SHOCK - USAGE OF ALL ITEMS** | |
| section_four_skip_med | INTERVIEWER: WOULD YOU LIKE TO COMPLETE THIS SECTION NOW OR SKIP THIS SECTION AND RETURN TO IT LATER? | 1 Do not skip, complete this section |

| Field | Question | Answer |
|---|---|---|
| | RETURN TO IT LATER?<br>*You may need to skip if the participant has indicated that s/he cannot answer the questions in this section.* | now.<br>2 Skip and come back to this section later. |
| group_section_four_introB<br>*Group relevant when: selected( ${section_four_skip_med} , '1')* | | |
| section4_start2 | In this section of the interview, we will review all of the items that you said are used for management of shock. For each item that is used, I'm going to ask questions on how many women need it and how much of it is used. | |

| Field | Question | Answer |
|---|---|---|
| section4_start3 | INTERVIEWER: ENTER WHOLE NUMBERS OR DECIMALS. DO NOT TYPE PERCENT SIGNS. ENTER 999 FOR ANY THAT ARE UNKNOWN. | |
| <p><strong><em>D. Medications - Shock (1)</em></strong></p><br>*Group relevant when: selected( ${section_four_skip_med} , '1')* | | |
| q404_med_note1 | 404 For each of the following medications and other medical products that are used for shock, can you tell me …? | |
| note_404_analgesic | ***Analgesic, anti-inflammatory, narcotic, etc.*** | |
| q404_M1 | ***q404_M1. Acetylsalicylic acid (aspirin)***<br>*Question relevant when: selected( ${q104_M1_full_list} , '3')* | |
| q404_M1a *(required)* | q404_M1a. What percent of patients require this item?<br>*Question relevant when: selected( ${q104_M1_full_list} , '3')*<br>*Response constrained to: .>0 and .<=100 or .=999* | |
| q404_M1b *(required)* | q404_M1b. How is the item administered, or given, to the patient?<br>*Question relevant when: selected( ${q104_M1_full_list} , '3')* | 1 Oral - solid (e.g. tablet)<br>2 Oral - liquid<br>3 Sublingual<br>4 Intravenous (IV)<br>5 Intramusculuar injection (IM)<br>6 Subcutaneous<br>7 Rectal<br>8 Inhaled (e.g. gas)<br>9 Inserted (e.g. vaginal pessaries)<br>10 Topical - cream<br>11 Topical - drops (e.g. eye drops)<br>12 Other<br>99 Don't know |
| q404_M1c *(required)* | q404_M1c. What is the item's smallest unit of measurement (mg, mcg, ml, g, etc)?<br>*Question relevant when: selected( ${q104_M1_full_list} , '3')* | microgram — microgram (mcg)<br>miligram — miligram (mg)<br>miligram_dissolved_in_liquid — miligram (mg) dissolved in liquid (e.g. 2 mg per 3 militers)<br>gram — gram (g)<br>militer — militer (ml)<br>centiliter — centiliter (cl)<br>litre — litre (l)<br>ml_of _solution — ml of solution (e.g. ml of 10% solution)<br>litre_of_solution — litre of solution (e.g. 1 litre of 5% solution)<br>cubic_meter — cubic meter (m^3)<br>other — other<br>dont_know — don't |

| Field | Question | Answer | |
|---|---|---|---|
| | | | know |
| q404_M1c.1 | q404_M1c.1 If other unit of measurement or route of administration, specify here:<br>*Leave blank if not applicable*<br>*Question relevant when: selected( ${q104_M1_full_list} , '3')* | | |
| q404_M1d *(required)* | q404_M1d. In total, how many units are used to treat a patient during all of her care?<br>*Remember to add all units! E.g. 5 days x 200 mg twice per day = 2,000mg*<br>*Question relevant when: selected( ${q104_M1_full_list} , '3')*<br>*Response constrained to: .>0* | | |

| | | | |
|---|---|---|---|
| q404_M2 | ***q404_M2. Paracetamol***<br>*Question relevant when: selected( ${q104_M2_full_list} , '3')* | | |
| q404_M2a *(required)* | q404_M2a. What percent of patients require this item?<br>*Question relevant when: selected( ${q104_M2_full_list} , '3')*<br>*Response constrained to: .>0 and .<=100 or .=999* | | |
| q404_M2b *(required)* | q404_M2b. How is the item administered, or given, to the patient?<br>*Question relevant when: selected( ${q104_M2_full_list} , '3')* | 1 | Oral - solid (e.g. tablet) |
| | | 2 | Oral - liquid |
| | | 3 | Sublingual |
| | | 4 | Intravenous (IV) |
| | | 5 | Intramusculuar injection (IM) |
| | | 6 | Subcutaneous |
| | | 7 | Rectal |
| | | 8 | Inhaled (e.g. gas) |
| | | 9 | Inserted (e.g. vaginal pessaries) |
| | | 10 | Topical - cream |
| | | 11 | Topical - drops (e.g. eye drops) |
| | | 12 | Other |
| | | 99 | Don't know |
| q404_M2c *(required)* | q404_M2c. What is the item's smallest unit of measurement (mg, mcg, ml, g, etc)?<br>*Question relevant when: selected( ${q104_M2_full_list} , '3')* | microgram | microgram (mcg) |
| | | miligram | miligram (mg) |
| | | miligram_dissolved_in_liquid | miligram (mg) dissolved in liquid (e.g. 2 mg per 3 militers) |
| | | gram | gram (g) |
| | | militer | militer (ml) |
| | | centiliter | centiliter (cl) |
| | | litre | litre (l) |
| | | ml_of _solution | ml of solution (e.g. ml of 10% solution) |
| | | litre_of_solution | litre of solution (e.g. 1 litre of 5% solution) |
| | | cubic_meter | cubic meter (m^3) |
| | | other | other |
| | | dont_know | don't know |
| q404_M2c.1 | q404_M2c.1 If other unit of measurement or route of administration, specify here:<br>*Leave blank if not applicable*<br>*Question relevant when: selected( ${q104_M2_full_list} , '3')* | | |
| q404_M2d *(required)* | q404_M2d. In total, how many units are used to treat a patient during all of her care?<br>*Remember to add all units! E.g. 5 days x 200 mg twice per day = 2,000mg*<br>*Question relevant when: selected( ${q104_M2_full_list} , '3')* | | |

Question relevant when: selected( ${q104_M2_full_list} , '3')
Response constrained to: .>0

| Field | Question | Answer |
|---|---|---|
| q404_M3 | ***q404_M3. Pethidine hydrochloride***<br>*Question relevant when: selected( ${q104_M3_full_list} , '3')* | |
| q404_M3a *(required)* | q404_M3a. What percent of patients require this item?<br>*Question relevant when: selected( ${q104_M3_full_list} , '3')*<br>*Response constrained to: .>0 and .<=100 or .=999* | |

| Field | Question | Answer |
|---|---|---|
| q404_M3b *(required)* | q404_M3b. How is the item administered, or given, to the patient?<br>*Question relevant when: selected( ${q104_M3_full_list} , '3')* | 1 Oral - solid (e.g. tablet)<br>2 Oral - liquid<br>3 Sublingual<br>4 Intravenous (IV)<br>5 Intramusculuar injection (IM)<br>6 Subcutaneous<br>7 Rectal<br>8 Inhaled (e.g. gas)<br>9 Inserted (e.g. vaginal pessaries)<br>10 Topical - cream<br>11 Topical - drops (e.g. eye drops)<br>12 Other<br>99 Don't know |
| q404_M3c *(required)* | q404_M3c. What is the item's smallest unit of measurement (mg, mcg, ml, g, etc)?<br>*Question relevant when: selected( ${q104_M3_full_list} , '3')* | microgram — microgram (mcg)<br>miligram — miligram (mg)<br>miligram_dissolved_in_liquid — miligram (mg) dissolved in liquid (e.g. 2 mg per 3 militers)<br>gram — gram (g)<br>militer — militer (ml)<br>centiliter — centiliter (cl)<br>litre — litre (l)<br>ml_of _solution — ml of solution (e.g. ml of 10% solution)<br>litre_of_solution — litre of solution (e.g. 1 litre of 5% solution)<br>cubic_meter — cubic meter (m^3)<br>other — other<br>dont_know — don't know |
| q404_M3c.1 | q404_M3c.1 If other unit of measurement or route of administration, specify here:<br>*Leave blank if not applicable*<br>*Question relevant when: selected( ${q104_M3_full_list} , '3')* | |
| q404_M3d *(required)* | q404_M3d. In total, how many units are used to treat a patient during all of her care?<br>*Remember to add all units! E.g. 5 days x 200 mg twice per day = 2,000mg*<br>*Question relevant when: selected( ${q104_M3_full_list} , '3')*<br>*Response constrained to: .>0* | |
| q404_M4 | ***q404_M4. Tramadol Hydrochloride***<br>*Question relevant when: selected( ${q104_M4_full_list} , '3')* | |
| q404_M4a *(required)* | q404_M4a. What percent of patients require this item?<br>*Question relevant when: selected( ${q104_M4_full_list} , '3')* | |

*Question relevant when: selected( ${q104_M4_full_list} , '3')*

*Response constrained to: .>0 and .<=100 or .=999*

| Field | Question | Answer |
|---|---|---|
| q404_M4b *(required)* | q404_M4b. How is the item administered, or given, to the patient?<br>*Question relevant when: selected( ${q104_M4_full_list} , '3')* | 1 Oral - solid (e.g. tablet)<br>2 Oral - liquid<br>3 Sublingual<br>4 Intravenous (IV)<br>5 Intramusculuar injection (IM)<br>6 Subcutaneous<br>7 Rectal<br>8 Inhaled (e.g. gas)<br>9 Inserted (e.g. vaginal pessaries)<br>10 Topical - cream<br>11 Topical - drops (e.g. eye drops)<br>12 Other<br>99 Don't know |
| q404_M4c *(required)* | q404_M4c. What is the item's smallest unit of measurement (mg, mcg, ml, g, etc)?<br>*Question relevant when: selected( ${q104_M4_full_list} , '3')* | microgram — microgram (mcg)<br>miligram — miligram (mg)<br>miligram_dissolved_in_liquid — miligram (mg) dissolved in liquid (e.g. 2 mg per 3 militers)<br>gram — gram (g)<br>militer — militer (ml)<br>centiliter — centiliter (cl)<br>litre — litre (l)<br>ml_of _solution — ml of solution (e.g. ml of 10% solution)<br>litre_of_solution — litre of solution (e.g. 1 litre of 5% solution)<br>cubic_meter — cubic meter (m^3)<br>other — other<br>dont_know — don't know |
| q404_M4c.1 | q404_M4c.1 If other unit of measurement or route of administration, specify here:<br>*Leave blank if not applicable*<br>*Question relevant when: selected( ${q104_M4_full_list} , '3')* | |
| q404_M4d *(required)* | q404_M4d. In total, how many units are used to treat a patient during all of her care?<br>*Remember to add all units! E.g. 5 days x 200 mg twice per day = 2,000mg*<br>*Question relevant when: selected( ${q104_M4_full_list} , '3')*<br>*Response constrained to: .>0* | |
| q404_M5 | ***q404_M5. Ibuprofen***<br>*Question relevant when: selected( ${q104_M5_full_list} , '3')* | |
| q404_M5a *(required)* | q404_M5a. What percent of patients require this item?<br>*Question relevant when: selected( ${q104_M5_full_list} , '3')* | |

| Field | Question | Answer |
|---|---|---|
| | *Question relevant when: selected( ${q104_M5_full_list} , '3')* *Response constrained to: .>0 and .<=100 or .=999* | |
| q404_M5b *(required)* | q404_M5b. How is the item administered, or given, to the patient? *Question relevant when: selected( ${q104_M5_full_list} , '3')* | 1 Oral - solid (e.g. tablet)<br>2 Oral - liquid<br>3 Sublingual<br>4 Intravenous (IV)<br>5 Intramusculuar injection (IM)<br>6 Subcutaneous<br>7 Rectal<br>8 Inhaled (e.g. gas)<br>9 Inserted (e.g. vaginal pessaries)<br>10 Topical - cream<br>11 Topical - drops (e.g. eye drops)<br>12 Other<br>99 Don't know |
| q404_M5c *(required)* | q404_M5c. What is the item's smallest unit of measurement (mg, mcg, ml, g, etc)? *Question relevant when: selected( ${q104_M5_full_list} , '3')* | microgram — microgram (mcg)<br>miligram — miligram (mg)<br>miligram_dissolved_in_liquid — miligram (mg) dissolved in liquid (e.g. 2 mg per 3 militers)<br>gram — gram (g)<br>militer — militer (ml)<br>centiliter — centiliter (cl)<br>litre — litre (l)<br>ml_of _solution — ml of solution (e.g. ml of 10% solution)<br>litre_of_solution — litre of solution (e.g. 1 litre of 5% solution)<br>cubic_meter — cubic meter (m^3)<br>other — other<br>dont_know — don't know |
| q404_M5c.1 | q404_M5c.1 If other unit of measurement or route of administration, specify here: *Leave blank if not applicable* *Question relevant when: selected( ${q104_M5_full_list} , '3')* | |
| q404_M5d *(required)* | q404_M5d. In total, how many units are used to treat a patient during all of her care? *Remember to add all units! E.g. 5 days x 200 mg twice per day = 2,000mg* *Question relevant when: selected( ${q104_M5_full_list} , '3')* *Response constrained to: .>0* | |
| q404_M6 | ***q404_M6. Diclofenac*** *Question relevant when: selected( ${q104_M6_full_list} , '3')* | |
| q404_M6a *(required)* | q404_M6a. What percent of patients require this item? *Question relevant when: selected( ${q104_M6_full_list} , '3')* | |

| Field | Question | Answer |
|---|---|---|
| | Question relevant when: selected( ${q104_M6_full_list} , '3')<br>Response constrained to: .>0 and .<=100 or .=999 | |

| Field | Question | Answer |
|---|---|---|
| q404_M6b *(required)* | q404_M6b. How is the item administered, or given, to the patient?<br>*Question relevant when: selected( ${q104_M6_full_list} , '3')* | 1 Oral - solid (e.g. tablet)<br>2 Oral - liquid<br>3 Sublingual<br>4 Intravenous (IV)<br>5 Intramusculuar injection (IM)<br>6 Subcutaneous<br>7 Rectal<br>8 Inhaled (e.g. gas)<br>9 Inserted (e.g. vaginal pessaries)<br>10 Topical - cream<br>11 Topical - drops (e.g. eye drops)<br>12 Other<br>99 Don't know |
| q404_M6c *(required)* | q404_M6c. What is the item's smallest unit of measurement (mg, mcg, ml, g, etc)?<br>*Question relevant when: selected( ${q104_M6_full_list} , '3')* | microgram — microgram (mcg)<br>miligram — miligram (mg)<br>miligram_dissolved_in_liquid — miligram (mg) dissolved in liquid (e.g. 2 mg per 3 militers)<br>gram — gram (g)<br>militer — militer (ml)<br>centiliter — centiliter (cl)<br>litre — litre (l)<br>ml_of _solution — ml of solution (e.g. ml of 10% solution)<br>litre_of_solution — litre of solution (e.g. 1 litre of 5% solution)<br>cubic_meter — cubic meter (m^3)<br>other — other<br>dont_know — don't know |
| q404_M6c.1 | q404_M6c.1 If other unit of measurement or route of administration, specify here:<br>*Leave blank if not applicable*<br>*Question relevant when: selected( ${q104_M6_full_list} , '3')* | |
| q404_M6d *(required)* | q404_M6d. In total, how many units are used to treat a patient during all of her care?<br>*Remember to add all units! E.g. 5 days x 200 mg twice per day = 2,000mg*<br>*Question relevant when: selected( ${q104_M6_full_list} , '3')*<br>*Response constrained to: .>0* | |
| q404_M7 | ***q404_M7. Diclofenac sodium***<br>*Question relevant when: selected( ${q104_M7_full_list} , '3')* | |
| q404_M7a *(required)* | q404_M7a. What percent of patients require this item?<br>*Question relevant when: selected( ${q104_M7_full_list} , '3')* | |

*Question relevant when: selected( ${q104_M7_full_list} , '3')*

*Response constrained to: .>0 and .<=100 or .=999*

| Field | Question | Answer |
|---|---|---|

| Field | Question | Answer |
|---|---|---|
| q404_M7b *(required)* | q404_M7b. How is the item administered, or given, to the patient?<br>*Question relevant when: selected( ${q104_M7_full_list} , '3')* | 1 Oral - solid (e.g. tablet)<br>2 Oral - liquid<br>3 Sublingual<br>4 Intravenous (IV)<br>5 Intramusculuar injection (IM)<br>6 Subcutaneous<br>7 Rectal<br>8 Inhaled (e.g. gas)<br>9 Inserted (e.g. vaginal pessaries)<br>10 Topical - cream<br>11 Topical - drops (e.g. eye drops)<br>12 Other<br>99 Don't know |
| q404_M7c *(required)* | q404_M7c. What is the item's smallest unit of measurement (mg, mcg, ml, g, etc)?<br>*Question relevant when: selected( ${q104_M7_full_list} , '3')* | microgram – microgram (mcg)<br>miligram – miligram (mg)<br>miligram_dissolved_in_liquid – miligram (mg) dissolved in liquid (e.g. 2 mg per 3 militers)<br>gram – gram (g)<br>militer – militer (ml)<br>centiliter – centiliter (cl)<br>litre – litre (l)<br>ml_of _solution – ml of solution (e.g. ml of 10% solution)<br>litre_of_solution – litre of solution (e.g. 1 litre of 5% solution)<br>cubic_meter – cubic meter (m^3)<br>other – other<br>dont_know – don't know |
| q404_M7c.1 | q404_M7c.1 If other unit of measurement or route of administration, specify here:<br>*Leave blank if not applicable*<br>*Question relevant when: selected( ${q104_M7_full_list} , '3')* | |
| q404_M7d *(required)* | q404_M7d. In total, how many units are used to treat a patient during all of her care?<br>*Remember to add all units! E.g. 5 days x 200 mg twice per day = 2,000mg*<br>*Question relevant when: selected( ${q104_M7_full_list} , '3')*<br>*Response constrained to: .>0* | |
| q404_M8 | ***q404_M8. Dypron (dipyrone), 250mg/ml (Metamizole)***<br>*Question relevant when: selected( ${q104_M8_full_list} , '3')* | |
| q404_M8a *(required)* | q404_M8a. What percent of patients require this item?<br>*Question relevant when: selected( ${q104_M8_full_list} , '3')* | |

| Field | Question | Answer |
|---|---|---|
| | *Question relevant when: selected( ${q104_M8_full_list} , '3')*<br>*Response constrained to: .>0 and .<=100 or .=999* | |
| q404_M8b *(required)* | q404_M8b. How is the item administered, or given, to the patient?<br>*Question relevant when: selected( ${q104_M8_full_list} , '3')* | 1 Oral - solid (e.g. tablet)<br>2 Oral - liquid<br>3 Sublingual<br>4 Intravenous (IV)<br>5 Intramusculuar injection (IM)<br>6 Subcutaneous<br>7 Rectal<br>8 Inhaled (e.g. gas)<br>9 Inserted (e.g. vaginal pessaries)<br>10 Topical - cream<br>11 Topical - drops (e.g. eye drops)<br>12 Other<br>99 Don't know |
| q404_M8c *(required)* | q404_M8c. What is the item's smallest unit of measurement (mg, mcg, ml, g, etc)?<br>*Question relevant when: selected( ${q104_M8_full_list} , '3')* | microgram – microgram (mcg)<br>miligram – miligram (mg)<br>miligram_dissolved_in_liquid – miligram (mg) dissolved in liquid (e.g. 2 mg per 3 militers)<br>gram – gram (g)<br>militer – militer (ml)<br>centiliter – centiliter (cl)<br>litre – litre (l)<br>ml_of _solution – ml of solution (e.g. ml of 10% solution)<br>litre_of_solution – litre of solution (e.g. 1 litre of 5% solution)<br>cubic_meter – cubic meter (m^3)<br>other – other<br>dont_know – don't know |
| q404_M8c.1 | q404_M8c.1 If other unit of measurement or route of administration, specify here:<br>*Leave blank if not applicable*<br>*Question relevant when: selected( ${q104_M8_full_list} , '3')* | |
| q404_M8d *(required)* | q404_M8d. In total, how many units are used to treat a patient during all of her care?<br>*Remember to add all units! E.g. 5 days x 200 mg twice per day = 2,000mg*<br>*Question relevant when: selected( ${q104_M8_full_list} , '3')*<br>*Response constrained to: .>0* | |
| q404_M9 | ***q404_M9. Fentanyl citrate, 50 mcg/ml***<br>*Question relevant when: selected( ${q104_M9_full_list} , '3')* | |
| q404_M9a *(required)* | q404_M9a. What percent of patients require this item?<br>*Question relevant when: selected( ${q104_M9_full_list} , '3')* | |

| Field | Question | Answer |
|---|---|---|
| | *Question relevant when: selected( ${q104_M9_full_list} , '3')*<br>*Response constrained to: .>0 and .<=100 or .=999* | |
| q404_M9b *(required)* | q404_M9b. How is the item administered, or given, to the patient?<br>*Question relevant when: selected( ${q104_M9_full_list} , '3')* | 1 Oral - solid (e.g. tablet)<br>2 Oral - liquid<br>3 Sublingual<br>4 Intravenous (IV)<br>5 Intramusculuar injection (IM)<br>6 Subcutaneous<br>7 Rectal<br>8 Inhaled (e.g. gas)<br>9 Inserted (e.g. vaginal pessaries)<br>10 Topical - cream<br>11 Topical - drops (e.g. eye drops)<br>12 Other<br>99 Don't know |
| q404_M9c *(required)* | q404_M9c. What is the item's smallest unit of measurement (mg, mcg, ml, g, etc)?<br>*Question relevant when: selected( ${q104_M9_full_list} , '3')* | microgram — microgram (mcg)<br>miligram — miligram (mg)<br>miligram_dissolved_in_liquid — miligram (mg) dissolved in liquid (e.g. 2 mg per 3 militers)<br>gram — gram (g)<br>militer — militer (ml)<br>centiliter — centiliter (cl)<br>litre — litre (l)<br>ml_of _solution — ml of solution (e.g. ml of 10% solution)<br>litre_of_solution — litre of solution (e.g. 1 litre of 5% solution)<br>cubic_meter — cubic meter (m^3)<br>other — other<br>dont_know — don't know |
| q404_M9c.1 | q404_M9c.1 If other unit of measurement or route of administration, specify here:<br>*Leave blank if not applicable*<br>*Question relevant when: selected( ${q104_M9_full_list} , '3')* | |
| q404_M9d *(required)* | q404_M9d. In total, how many units are used to treat a patient during all of her care?<br>*Remember to add all units! E.g. 5 days x 200 mg twice per day = 2,000mg*<br>*Question relevant when: selected( ${q104_M9_full_list} , '3')*<br>*Response constrained to: .>0* | |
| q404_M10 | ***q404_M10. Analgesic - other 1: "[q104_M10_full_list_other]"***<br>*Question relevant when: selected( ${q104_M10_full_list} , '3')* | |
| q404_M10a *(required)* | q404_M10a. What percent of patients require this item?<br>*Question relevant when: selected( ${q104_M10_full_list} , '3')* | |

*Question relevant when: selected( ${q104_M10_full_list} , '3')*

*Response constrained to: .>0 and .<=100 or .=999*

<table>
<tr><th>Field</th><th>Question</th><th>Answer</th></tr>
<tr><td></td><td></td><td></td></tr>
<tr><td>q404_M10b (required)</td><td>q404_M10b. How is the item administered, or given, to the patient?<br>Question relevant when: selected( ${q104_M10_full_list} , '3')</td><td><table>
<tr><td>1</td><td>Oral - solid (e.g. tablet)</td></tr>
<tr><td>2</td><td>Oral - liquid</td></tr>
<tr><td>3</td><td>Sublingual</td></tr>
<tr><td>4</td><td>Intravenous (IV)</td></tr>
<tr><td>5</td><td>Intramusculuar injection (IM)</td></tr>
<tr><td>6</td><td>Subcutaneous</td></tr>
<tr><td>7</td><td>Rectal</td></tr>
<tr><td>8</td><td>Inhaled (e.g. gas)</td></tr>
<tr><td>9</td><td>Inserted (e.g. vaginal pessaries)</td></tr>
<tr><td>10</td><td>Topical - cream</td></tr>
<tr><td>11</td><td>Topical - drops (e.g. eye drops)</td></tr>
<tr><td>12</td><td>Other</td></tr>
<tr><td>99</td><td>Don't know</td></tr>
</table></td></tr>
<tr><td>q404_M10c (required)</td><td>q404_M10c. What is the item's smallest unit of measurement (mg, mcg, ml, g, etc)?<br>Question relevant when: selected( ${q104_M10_full_list} , '3')</td><td><table>
<tr><td>microgram</td><td>microgram (mcg)</td></tr>
<tr><td>miligram</td><td>miligram (mg)</td></tr>
<tr><td>miligram_dissolved_in_liquid</td><td>miligram (mg) dissolved in liquid (e.g. 2 mg per 3 militers)</td></tr>
<tr><td>gram</td><td>gram (g)</td></tr>
<tr><td>militer</td><td>militer (ml)</td></tr>
<tr><td>centiliter</td><td>centiliter (cl)</td></tr>
<tr><td>litre</td><td>litre (l)</td></tr>
<tr><td>ml_of _solution</td><td>ml of solution (e.g. ml of 10% solution)</td></tr>
<tr><td>litre_of_solution</td><td>litre of solution (e.g. 1 litre of 5% solution)</td></tr>
<tr><td>cubic_meter</td><td>cubic meter (m^3)</td></tr>
<tr><td>other</td><td>other</td></tr>
<tr><td>dont_know</td><td>don't know</td></tr>
</table></td></tr>
<tr><td>q404_M10c.1</td><td>q404_M10c.1 If other unit of measurement or route of administration, specify here:<br>Leave blank if not applicable<br>Question relevant when: selected( ${q104_M10_full_list} , '3')</td><td></td></tr>
<tr><td>q404_M10d (required)</td><td>q404_M10d. In total, how many units are used to treat a patient during all of her care?<br>Remember to add all units! E.g. 5 days x 200 mg twice per day = 2,000mg<br>Question relevant when: selected( ${q104_M10_full_list} , '3')<br>Response constrained to: .>0</td><td></td></tr>
<tr><td>q404_M11</td><td>q404_M11. Analgesic - other 2: "[q104_M11_full_list_other]"<br>Question relevant when: selected( ${q104_M11_full_list} , '3')</td><td></td></tr>
<tr><td>q404_M11a (required)</td><td>q404_M11a. What percent of patients require this item?<br>Question relevant when: selected( ${q104_M11_full_list} , '3')</td><td></td></tr>
</table>

Question relevant when: selected( ${q104_M11_full_list} , '3')

| Field | Question | Answer |
|---|---|---|
| | Response constrained to: .>0 and .<=100 or .=999 | |

| Field | Question | Answer |
|---|---|---|
| q404_M11b *(required)* | q404_M11b. How is the item administered, or given, to the patient?<br>*Question relevant when: selected( ${q104_M11_full_list} , '3')* | 1 Oral - solid (e.g. tablet)<br>2 Oral - liquid<br>3 Sublingual<br>4 Intravenous (IV)<br>5 Intramusculuar injection (IM)<br>6 Subcutaneous<br>7 Rectal<br>8 Inhaled (e.g. gas)<br>9 Inserted (e.g. vaginal pessaries)<br>10 Topical - cream<br>11 Topical - drops (e.g. eye drops)<br>12 Other<br>99 Don't know |
| q404_M11c *(required)* | q404_M11c. What is the item's smallest unit of measurement (mg, mcg, ml, g, etc)?<br>*Question relevant when: selected( ${q104_M11_full_list} , '3')* | microgram: microgram (mcg)<br>miligram: miligram (mg)<br>miligram_dissolved_in_liquid: miligram (mg) dissolved in liquid (e.g. 2 mg per 3 militers)<br>gram: gram (g)<br>militer: militer (ml)<br>centiliter: centiliter (cl)<br>litre: litre (l)<br>ml_of _solution: ml of solution (e.g. ml of 10% solution)<br>litre_of_solution: litre of solution (e.g. 1 litre of 5% solution)<br>cubic_meter: cubic meter (m^3)<br>other: other<br>dont_know: don't know |
| q404_M11c.1 | q404_M11c.1 If other unit of measurement or route of administration, specify here:<br>*Leave blank if not applicable*<br>*Question relevant when: selected( ${q104_M11_full_list} , '3')* | |
| q404_M11d *(required)* | q404_M11d. In total, how many units are used to treat a patient during all of her care?<br>*Remember to add all units! E.g. 5 days x 200 mg twice per day = 2,000mg*<br>*Question relevant when: selected( ${q104_M11_full_list} , '3')*<br>*Response constrained to: .>0* | |
| q404_M12 | ***q404_M12. Analgesic - other 3: "[q104_M12_full_list_other]"***<br>*Question relevant when: selected( ${q104_M12_full_list} , '3')* | |
| q404_M12a *(required)* | q404_M12a. What percent of patients require this item?<br>*Question relevant when: selected( ${q104_M12_full_list} , '3')* | |

| Field | Question | Answer |
|---|---|---|
| | Question relevant when: selected( ${q104_M12_full_list} , '3')<br>Response constrained to: .>0 and .<=100 or .=999 | |
| q404_M12b *(required)* | q404_M12b. How is the item administered, or given, to the patient?<br>*Question relevant when: selected( ${q104_M12_full_list} , '3')* | 1 Oral - solid (e.g. tablet)<br>2 Oral - liquid<br>3 Sublingual<br>4 Intravenous (IV)<br>5 Intramuscuular injection (IM)<br>6 Subcutaneous<br>7 Rectal<br>8 Inhaled (e.g. gas)<br>9 Inserted (e.g. vaginal pessaries)<br>10 Topical - cream<br>11 Topical - drops (e.g. eye drops)<br>12 Other<br>99 Don't know |
| q404_M12c *(required)* | q404_M12c. What is the item's smallest unit of measurement (mg, mcg, ml, g, etc)?<br>*Question relevant when: selected( ${q104_M12_full_list} , '3')* | microgram — microgram (mcg)<br>miligram — miligram (mg)<br>miligram_dissolved_in_liquid — miligram (mg) dissolved in liquid (e.g. 2 mg per 3 militers)<br>gram — gram (g)<br>militer — militer (ml)<br>centiliter — centiliter (cl)<br>litre — litre (l)<br>ml_of _solution — ml of solution (e.g. ml of 10% solution)<br>litre_of_solution — litre of solution (e.g. 1 litre of 5% solution)<br>cubic_meter — cubic meter (m^3)<br>other — other<br>dont_know — don't know |
| q404_M12c.1 | q404_M12c.1 If other unit of measurement or route of administration, specify here:<br>*Leave blank if not applicable*<br>*Question relevant when: selected( ${q104_M12_full_list} , '3')* | |
| q404_M12d *(required)* | q404_M12d. In total, how many units are used to treat a patient during all of her care?<br>*Remember to add all units! E.g. 5 days x 200 mg twice per day = 2,000mg*<br>*Question relevant when: selected( ${q104_M12_full_list} , '3')*<br>*Response constrained to: .>0* | |
| q404_M13 | ***q404_M13. Analgesic - other 4: "[q104_M13_full_list_other]"***<br>*Question relevant when: selected( ${q104_M13_full_list} , '3')* | |
| q404_M13a *(required)* | q404_M13a. What percent of patients require this item?<br>*Question relevant when: selected( ${q104_M13_full_list} , '3')* | |

Question relevant when: selected( ${q104_M13_full_list} , '3')

Response constrained to: .>0 and .<=100 or .=999

| Field | Question | Answer |
|---|---|---|
| q404_M13b *(required)* | q404_M13b. How is the item administered, or given, to the patient?<br>*Question relevant when: selected( ${q104_M13_full_list} , '3')* | 1 Oral - solid (e.g. tablet)<br>2 Oral - liquid<br>3 Sublingual<br>4 Intravenous (IV)<br>5 Intramusculuar injection (IM)<br>6 Subcutaneous<br>7 Rectal<br>8 Inhaled (e.g. gas)<br>9 Inserted (e.g. vaginal pessaries)<br>10 Topical - cream<br>11 Topical - drops (e.g. eye drops)<br>12 Other<br>99 Don't know |
| q404_M13c *(required)* | q404_M13c. What is the item's smallest unit of measurement (mg, mcg, ml, g, etc)?<br>*Question relevant when: selected( ${q104_M13_full_list} , '3')* | microgram — microgram (mcg)<br>miligram — miligram (mg)<br>miligram_dissolved_in_liquid — miligram (mg) dissolved in liquid (e.g. 2 mg per 3 militers)<br>gram — gram (g)<br>militer — militer (ml)<br>centiliter — centiliter (cl)<br>litre — litre (l)<br>ml_of _solution — ml of solution (e.g. ml of 10% solution)<br>litre_of_solution — litre of solution (e.g. 1 litre of 5% solution)<br>cubic_meter — cubic meter (m^3)<br>other — other<br>dont_know — don't know |
| q404_M13c.1 | q404_M13c.1 If other unit of measurement or route of administration, specify here:<br>*Leave blank if not applicable*<br>*Question relevant when: selected( ${q104_M13_full_list} , '3')* | |
| q404_M13d *(required)* | q404_M13d. In total, how many units are used to treat a patient during all of her care?<br>*Remember to add all units! E.g. 5 days x 200 mg twice per day = 2,000mg*<br>*Question relevant when: selected( ${q104_M13_full_list} , '3')*<br>*Response constrained to: .>0* | |
| q404_M14 | ***q404_M14. Analgesic - other 5: "[q104_M14_full_list_other]"***<br>*Question relevant when: selected( ${q104_M14_full_list} , '3')* | |
| q404_M14a *(required)* | q404_M14a. What percent of patients require this item?<br>*Question relevant when: selected( ${q104_M14_full_list} , '3')* | |

| Field | Question | Answer |
|---|---|---|
| | *Question relevant when: selected( ${q104_M14_full_list} , '3')*<br>*Response constrained to: .>0 and .<=100 or .=999* | |
| q404_M14b *(required)* | q404_M14b. How is the item administered, or given, to the patient?<br>*Question relevant when: selected( ${q104_M14_full_list} , '3')* | 1 Oral - solid (e.g. tablet)<br>2 Oral - liquid<br>3 Sublingual<br>4 Intravenous (IV)<br>5 Intramusculuar injection (IM)<br>6 Subcutaneous<br>7 Rectal<br>8 Inhaled (e.g. gas)<br>9 Inserted (e.g. vaginal pessaries)<br>10 Topical - cream<br>11 Topical - drops (e.g. eye drops)<br>12 Other<br>99 Don't know |
| q404_M14c *(required)* | q404_M14c. What is the item's smallest unit of measurement (mg, mcg, ml, g, etc)?<br>*Question relevant when: selected( ${q104_M14_full_list} , '3')* | microgram — microgram (mcg)<br>miligram — miligram (mg)<br>miligram_dissolved_in_liquid — miligram (mg) dissolved in liquid (e.g. 2 mg per 3 militers)<br>gram — gram (g)<br>militer — militer (ml)<br>centiliter — centiliter (cl)<br>litre — litre (l)<br>ml_of _solution — ml of solution (e.g. ml of 10% solution)<br>litre_of_solution — litre of solution (e.g. 1 litre of 5% solution)<br>cubic_meter — cubic meter (m^3)<br>other — other<br>dont_know — don't know |
| q404_M14c.1 | q404_M14c.1 If other unit of measurement or route of administration, specify here:<br>*Leave blank if not applicable*<br>*Question relevant when: selected( ${q104_M14_full_list} , '3')* | |
| q404_M14d *(required)* | q404_M14d. In total, how many units are used to treat a patient during all of her care?<br>*Remember to add all units! E.g. 5 days x 200 mg twice per day = 2,000mg*<br>*Question relevant when: selected( ${q104_M14_full_list} , '3')*<br>*Response constrained to: .>0* | |
| <p><strong><em>D. Medications - Shock (2)</em></strong></p><br>*Group relevant when: selected( ${section_four_skip_med} , '1')* | | |
| q404_med_note2 | 404 For each of the following medications and other medical products that are used for shock, can you tell me …? | |
| note_404_anesthetic | ***Anesthetic - local, general, etc. (and oxygen)*** | |

| Field | Question | Answer |
|---|---|---|
| note_404_anesthetic | ***Anesthetic - local, general, etc. (and oxygen)*** | |
| q404_M15 | ***q404_M15. Halothane***<br>*Question relevant when: selected( ${q104_M15_full_list} , '3')* | |
| q404_M15a *(required)* | q404_M15a. What percent of patients require this item?<br>*Question relevant when: selected( ${q104_M15_full_list} , '3')*<br>*Response constrained to: .>0 and .<=100 or .=999* | |

| Field | Question | Answer |
|---|---|---|
| q404_M15b *(required)* | q404_M15b. How is the item administered, or given, to the patient?<br>*Question relevant when: selected( ${q104_M15_full_list} , '3')* | 1 Oral - solid (e.g. tablet)<br>2 Oral - liquid<br>3 Sublingual<br>4 Intravenous (IV)<br>5 Intramusculuar injection (IM)<br>6 Subcutaneous<br>7 Rectal<br>8 Inhaled (e.g. gas)<br>9 Inserted (e.g. vaginal pessaries)<br>10 Topical - cream<br>11 Topical - drops (e.g. eye drops)<br>12 Other<br>99 Don't know |
| q404_M15c *(required)* | q404_M15c. What is the item's smallest unit of measurement (mg, mcg, ml, g, etc)?<br>*Question relevant when: selected( ${q104_M15_full_list} , '3')* | microgram — microgram (mcg)<br>miligram — miligram (mg)<br>miligram_dissolved_in_liquid — miligram (mg) dissolved in liquid (e.g. 2 mg per 3 militers)<br>gram — gram (g)<br>militer — militer (ml)<br>centiliter — centiliter (cl)<br>litre — litre (l)<br>ml_of _solution — ml of solution (e.g. ml of 10% solution)<br>litre_of_solution — litre of solution (e.g. 1 litre of 5% solution)<br>cubic_meter — cubic meter (m^3)<br>other — other<br>dont_know — don't know |
| q404_M15c.1 | q404_M15c.1 If other unit of measurement or route of administration, specify here:<br>*Leave blank if not applicable*<br>*Question relevant when: selected( ${q104_M15_full_list} , '3')* | |
| q404_M15d *(required)* | q404_M15d. In total, how many units are used to treat a patient during all of her care?<br>*Remember to add all units! E.g. 5 days x 200 mg twice per day = 2,000mg*<br>*Question relevant when: selected( ${q104_M15_full_list} , '3')*<br>*Response constrained to: .>0* | |
| q404_M16 | ***q404_M16. Ketamine (Ketalar), 50mg/ml***<br>*Question relevant when: selected( ${q104_M16_full_list} , '3')* | |
| q404_M16a *(required)* | q404_M16a. What percent of patients require this item?<br>*Question relevant when: selected( ${q104_M16_full_list} , '3')* | |

Question relevant when: selected( ${q104_M16_full_list} , '3')

| Field | Question | Answer |
|---|---|---|
| | *Response constrained to: .>0 and .<=100 or .=999* | |

| Field | Question | Answer |
|---|---|---|
| q404_M16b *(required)* | q404_M16b. How is the item administered, or given, to the patient? *Question relevant when: selected( ${q104_M16_full_list} , '3')* | 1 Oral - solid (e.g. tablet)<br>2 Oral - liquid<br>3 Sublingual<br>4 Intravenous (IV)<br>5 Intramusculuar injection (IM)<br>6 Subcutaneous<br>7 Rectal<br>8 Inhaled (e.g. gas)<br>9 Inserted (e.g. vaginal pessaries)<br>10 Topical - cream<br>11 Topical - drops (e.g. eye drops)<br>12 Other<br>99 Don't know |
| q404_M16c *(required)* | q404_M16c. What is the item's smallest unit of measurement (mg, mcg, ml, g, etc)? *Question relevant when: selected( ${q104_M16_full_list} , '3')* | microgram — microgram (mcg)<br>miligram — miligram (mg)<br>miligram_dissolved_in_liquid — miligram (mg) dissolved in liquid (e.g. 2 mg per 3 militers)<br>gram — gram (g)<br>militer — militer (ml)<br>centiliter — centiliter (cl)<br>litre — litre (l)<br>ml_of _solution — ml of solution (e.g. ml of 10% solution)<br>litre_of_solution — litre of solution (e.g. 1 litre of 5% solution)<br>cubic_meter — cubic meter (m^3)<br>other — other<br>dont_know — don't know |
| q404_M16c.1 | q404_M16c.1 If other unit of measurement or route of administration, specify here: *Leave blank if not applicable* *Question relevant when: selected( ${q104_M16_full_list} , '3')* | |
| q404_M16d *(required)* | q404_M16d. In total, how many units are used to treat a patient during all of her care? *Remember to add all units! E.g. 5 days x 200 mg twice per day = 2,000mg* *Question relevant when: selected( ${q104_M16_full_list} , '3')* *Response constrained to: .>0* | |
| q404_M17 | ***q404_M17. Propofol (Diprivan), 10mg/ml*** *Question relevant when: selected( ${q104_M17_full_list} , '3')* | |
| q404_M17a *(required)* | q404_M17a. What percent of patients require this item? *Question relevant when: selected( ${q104_M17_full_list} , '3')* | |

Question relevant when: selected( ${q104_M17_full_list} , '3')

| Field | Question | Answer |
|---|---|---|
| | Response constrained to: .>0 and .<=100 or .=999 | |
| q404_M17b *(required)* | q404_M17b. How is the item administered, or given, to the patient?<br>*Question relevant when: selected( ${q104_M17_full_list} , '3')* | 1 Oral - solid (e.g. tablet)<br>2 Oral - liquid<br>3 Sublingual<br>4 Intravenous (IV)<br>5 Intramusculuar injection (IM)<br>6 Subcutaneous<br>7 Rectal<br>8 Inhaled (e.g. gas)<br>9 Inserted (e.g. vaginal pessaries)<br>10 Topical - cream<br>11 Topical - drops (e.g. eye drops)<br>12 Other<br>99 Don't know |
| q404_M17c *(required)* | q404_M17c. What is the item's smallest unit of measurement (mg, mcg, ml, g, etc)?<br>*Question relevant when: selected( ${q104_M17_full_list} , '3')* | microgram: microgram (mcg)<br>miligram: miligram (mg)<br>miligram_dissolved_in_liquid: miligram (mg) dissolved in liquid (e.g. 2 mg per 3 militers)<br>gram: gram (g)<br>militer: militer (ml)<br>centiliter: centiliter (cl)<br>litre: litre (l)<br>ml_of _solution: ml of solution (e.g. ml of 10% solution)<br>litre_of_solution: litre of solution (e.g. 1 litre of 5% solution)<br>cubic_meter: cubic meter (m^3)<br>other: other<br>dont_know: don't know |
| q404_M17c.1 | q404_M17c.1 If other unit of measurement or route of administration, specify here:<br>*Leave blank if not applicable*<br>*Question relevant when: selected( ${q104_M17_full_list} , '3')* | |
| q404_M17d *(required)* | q404_M17d. In total, how many units are used to treat a patient during all of her care?<br>*Remember to add all units! E.g. 5 days x 200 mg twice per day = 2,000mg*<br>*Question relevant when: selected( ${q104_M17_full_list} , '3')*<br>*Response constrained to: .>0* | |
| q404_M18 | ***q404_M18. Suxamethonium chloride***<br>*Question relevant when: selected( ${q104_M18_full_list} , '3')* | |
| q404_M18a *(required)* | q404_M18a. What percent of patients require this item?<br>*Question relevant when: selected( ${q104_M18_full_list} , '3')* | |

| Field | Question | Answer |
|---|---|---|
| | Question relevant when: selected( ${q104_M18_full_list} , '3') Response constrained to: .>0 and .<=100 or .=999 | |

| Field | Question | Answer |
|---|---|---|
| q404_M18b *(required)* | q404_M18b. How is the item administered, or given, to the patient?<br>*Question relevant when: selected( ${q104_M18_full_list} , '3')* | 1 Oral - solid (e.g. tablet)<br>2 Oral - liquid<br>3 Sublingual<br>4 Intravenous (IV)<br>5 Intramusculuar injection (IM)<br>6 Subcutaneous<br>7 Rectal<br>8 Inhaled (e.g. gas)<br>9 Inserted (e.g. vaginal pessaries)<br>10 Topical - cream<br>11 Topical - drops (e.g. eye drops)<br>12 Other<br>99 Don't know |
| q404_M18c *(required)* | q404_M18c. What is the item's smallest unit of measurement (mg, mcg, ml, g, etc)?<br>*Question relevant when: selected( ${q104_M18_full_list} , '3')* | microgram: microgram (mcg)<br>miligram: miligram (mg)<br>miligram_dissolved_in_liquid: miligram (mg) dissolved in liquid (e.g. 2 mg per 3 militers)<br>gram: gram (g)<br>militer: militer (ml)<br>centiliter: centiliter (cl)<br>litre: litre (l)<br>ml_of _solution: ml of solution (e.g. ml of 10% solution)<br>litre_of_solution: litre of solution (e.g. 1 litre of 5% solution)<br>cubic_meter: cubic meter (m^3)<br>other: other<br>dont_know: don't know |
| q404_M18c.1 | q404_M18c.1 If other unit of measurement or route of administration, specify here:<br>*Leave blank if not applicable*<br>*Question relevant when: selected( ${q104_M18_full_list} , '3')* | |
| q404_M18d *(required)* | q404_M18d. In total, how many units are used to treat a patient during all of her care?<br>*Remember to add all units! E.g. 5 days x 200 mg twice per day = 2,000mg*<br>*Question relevant when: selected( ${q104_M18_full_list} , '3')*<br>*Response constrained to: .>0* | |
| q404_M19 | ***q404_M19. Thiopental sodium (Intraval)***<br>*Question relevant when: selected( ${q104_M19_full_list} , '3')* | |
| q404_M19a *(required)* | q404_M19a. What percent of patients require this item?<br>*Question relevant when: selected( ${q104_M19_full_list} , '3')* | |

*Question relevant when: selected( ${q104_M19_full_list} , '3')*
*Response constrained to: .>0 and .<=100 or .=999*

| Field | Question | Answer |
|---|---|---|
| q404_M19b *(required)* | q404_M19b. How is the item administered, or given, to the patient?<br>*Question relevant when: selected( ${q104_M19_full_list} , '3')* | 1 Oral - solid (e.g. tablet)<br>2 Oral - liquid<br>3 Sublingual<br>4 Intravenous (IV)<br>5 Intramusculuar injection (IM)<br>6 Subcutaneous<br>7 Rectal<br>8 Inhaled (e.g. gas)<br>9 Inserted (e.g. vaginal pessaries)<br>10 Topical - cream<br>11 Topical - drops (e.g. eye drops)<br>12 Other<br>99 Don't know |
| q404_M19c *(required)* | q404_M19c. What is the item's smallest unit of measurement (mg, mcg, ml, g, etc)?<br>*Question relevant when: selected( ${q104_M19_full_list} , '3')* | microgram — microgram (mcg)<br>miligram — miligram (mg)<br>miligram_dissolved_in_liquid — miligram (mg) dissolved in liquid (e.g. 2 mg per 3 militers)<br>gram — gram (g)<br>militer — militer (ml)<br>centiliter — centiliter (cl)<br>litre — litre (l)<br>ml_of _solution — ml of solution (e.g. ml of 10% solution)<br>litre_of_solution — litre of solution (e.g. 1 litre of 5% solution)<br>cubic_meter — cubic meter (m^3)<br>other — other<br>dont_know — don't know |
| q404_M19c.1 | q404_M19c.1 If other unit of measurement or route of administration, specify here:<br>*Leave blank if not applicable*<br>*Question relevant when: selected( ${q104_M19_full_list} , '3')* | |
| q404_M19d *(required)* | q404_M19d. In total, how many units are used to treat a patient during all of her care?<br>*Remember to add all units! E.g. 5 days x 200 mg twice per day = 2,000mg*<br>*Question relevant when: selected( ${q104_M19_full_list} , '3')*<br>*Response constrained to: .>0* | |
| q404_M20 | ***q404_M20. Oxygen***<br>*Question relevant when: selected( ${q104_M20_full_list} , '3')* | |
| q404_M20a *(required)* | q404_M20a. What percent of patients require this item?<br>*Question relevant when: selected( ${q104_M20_full_list} , '3')* | |

| Field | Question | Answer |
|---|---|---|
| | Question relevant when: selected( ${q104_M20_full_list} , '3') Response constrained to: .>0 and .<=100 or .=999 | |

| Field | Question | Answer |
|---|---|---|
| q404_M20b *(required)* | q404_M20b. How is the item administered, or given, to the patient? *Question relevant when: selected( ${q104_M20_full_list} , '3')* | 1 Oral - solid (e.g. tablet)<br>2 Oral - liquid<br>3 Sublingual<br>4 Intravenous (IV)<br>5 Intramuscular injection (IM)<br>6 Subcutaneous<br>7 Rectal<br>8 Inhaled (e.g. gas)<br>9 Inserted (e.g. vaginal pessaries)<br>10 Topical - cream<br>11 Topical - drops (e.g. eye drops)<br>12 Other<br>99 Don't know |
| q404_M20c *(required)* | q404_M20c. What is the item's smallest unit of measurement (mg, mcg, ml, g, etc)? *Question relevant when: selected( ${q104_M20_full_list} , '3')* | microgram — microgram (mcg)<br>miligram — miligram (mg)<br>miligram_dissolved_in_liquid — miligram (mg) dissolved in liquid (e.g. 2 mg per 3 militers)<br>gram — gram (g)<br>militer — militer (ml)<br>centiliter — centiliter (cl)<br>litre — litre (l)<br>ml_of _solution — ml of solution (e.g. ml of 10% solution)<br>litre_of_solution — litre of solution (e.g. 1 litre of 5% solution)<br>cubic_meter — cubic meter (m^3)<br>other — other<br>dont_know — don't know |
| q404_M20c.1 | q404_M20c.1 If other unit of measurement or route of administration, specify here: *Leave blank if not applicable* *Question relevant when: selected( ${q104_M20_full_list} , '3')* | |
| q404_M20d *(required)* | q404_M20d. In total, how many units are used to treat a patient during all of her care? *Remember to add all units! E.g. 5 days x 200 mg twice per day = 2,000mg* *Question relevant when: selected( ${q104_M20_full_list} , '3')* *Response constrained to: .>0* | |
| q404_M21 | ***q404_M21. Bupivacaine HCL 0.5% (Marcaine)*** *Question relevant when: selected( ${q104_M21_full_list} , '3')* | |
| q404_M21a *(required)* | q404_M21a. What percent of patients require this item? *Question relevant when: selected( ${q104_M21_full_list} , '3')* | |

| Field | Question | Answer |
|---|---|---|
| | *Question relevant when: selected( ${q104_M21_full_list} , '3')* *Response constrained to: .>0 and .<=100 or .=999* | |
| q404_M21b *(required)* | q404_M21b. How is the item administered, or given, to the patient? *Question relevant when: selected( ${q104_M21_full_list} , '3')* | 1 Oral - solid (e.g. tablet)<br>2 Oral - liquid<br>3 Sublingual<br>4 Intravenous (IV)<br>5 Intramusculuar injection (IM)<br>6 Subcutaneous<br>7 Rectal<br>8 Inhaled (e.g. gas)<br>9 Inserted (e.g. vaginal pessaries)<br>10 Topical - cream<br>11 Topical - drops (e.g. eye drops)<br>12 Other<br>99 Don't know |
| q404_M21c *(required)* | q404_M21c. What is the item's smallest unit of measurement (mg, mcg, ml, g, etc)? *Question relevant when: selected( ${q104_M21_full_list} , '3')* | microgram — microgram (mcg)<br>miligram — miligram (mg)<br>miligram_dissolved_in_liquid — miligram (mg) dissolved in liquid (e.g. 2 mg per 3 militers)<br>gram — gram (g)<br>militer — militer (ml)<br>centiliter — centiliter (cl)<br>litre — litre (l)<br>ml_of _solution — ml of solution (e.g. ml of 10% solution)<br>litre_of_solution — litre of solution (e.g. 1 litre of 5% solution)<br>cubic_meter — cubic meter (m^3)<br>other — other<br>dont_know — don't know |
| q404_M21c.1 | q404_M21c.1 If other unit of measurement or route of administration, specify here: *Leave blank if not applicable* *Question relevant when: selected( ${q104_M21_full_list} , '3')* | |
| q404_M21d *(required)* | q404_M21d. In total, how many units are used to treat a patient during all of her care? *Remember to add all units! E.g. 5 days x 200 mg twice per day = 2,000mg* *Question relevant when: selected( ${q104_M21_full_list} , '3')* *Response constrained to: .>0* | |
| q404_M22 | ***q404_M22. Lidocaine HCl 2% + epinephrin 1:100,000*** *Question relevant when: selected( ${q104_M22_full_list} , '3')* | |

| Field | Question | Answer |
|---|---|---|
| q404_M22a *(required)* | q404_M22a. What percent of patients require this item?<br>*Question relevant when: selected( ${q104_M22_full_list} , '3')*<br>*Response constrained to: .>0 and .<=100 or .=999* | |
| q404_M22b *(required)* | q404_M22b. How is the item administered, or given, to the patient?<br>*Question relevant when: selected( ${q104_M22_full_list} , '3')* | 1 Oral - solid (e.g. tablet)<br>2 Oral - liquid<br>3 Sublingual<br>4 Intravenous (IV)<br>5 Intramusculuar injection (IM)<br>6 Subcutaneous<br>7 Rectal<br>8 Inhaled (e.g. gas)<br>9 Inserted (e.g. vaginal pessaries)<br>10 Topical - cream<br>11 Topical - drops (e.g. eye drops)<br>12 Other<br>99 Don't know |
| q404_M22c *(required)* | q404_M22c. What is the item's smallest unit of measurement (mg, mcg, ml, g, etc)?<br>*Question relevant when: selected( ${q104_M22_full_list} , '3')* | microgram — microgram (mcg)<br>miligram — miligram (mg)<br>miligram_dissolved_in_liquid — miligram (mg) dissolved in liquid (e.g. 2 mg per 3 militers)<br>gram — gram (g)<br>militer — militer (ml)<br>centiliter — centiliter (cl)<br>litre — litre (l)<br>ml_of _solution — ml of solution (e.g. ml of 10% solution)<br>litre_of_solution — litre of solution (e.g. 1 litre of 5% solution)<br>cubic_meter — cubic meter (m^3)<br>other — other<br>dont_know — don't know |
| q404_M22c.1 | q404_M22c.1 If other unit of measurement or route of administration, specify here:<br>*Leave blank if not applicable*<br>*Question relevant when: selected( ${q104_M22_full_list} , '3')* | |
| q404_M22d *(required)* | q404_M22d. In total, how many units are used to treat a patient during all of her care?<br>*Remember to add all units! E.g. 5 days x 200 mg twice per day = 2,000mg*<br>*Question relevant when: selected( ${q104_M22_full_list} , '3')*<br>*Response constrained to: .>0* | |
| q404_M23 | ***q404_M23. Lidocaine HCl 5% + dextrose 7.5%***<br>*Question relevant when: selected( ${q104_M23_full_list} , '3')* | |

| Field | Question | Answer |
|---|---|---|
| q404_M23a *(required)* | q404_M23a. What percent of patients require this item?<br>*Question relevant when: selected( ${q104_M23_full_list} , '3')*<br>*Response constrained to: .>0 and .<=100 or .=999* | |
| q404_M23b *(required)* | q404_M23b. How is the item administered, or given, to the patient?<br>*Question relevant when: selected( ${q104_M23_full_list} , '3')* | 1 Oral - solid (e.g. tablet)<br>2 Oral - liquid<br>3 Sublingual<br>4 Intravenous (IV)<br>5 Intramuscular injection (IM)<br>6 Subcutaneous<br>7 Rectal<br>8 Inhaled (e.g. gas)<br>9 Inserted (e.g. vaginal pessaries)<br>10 Topical - cream<br>11 Topical - drops (e.g. eye drops)<br>12 Other<br>99 Don't know |
| q404_M23c *(required)* | q404_M23c. What is the item's smallest unit of measurement (mg, mcg, ml, g, etc)?<br>*Question relevant when: selected( ${q104_M23_full_list} , '3')* | microgram — microgram (mcg)<br>miligram — miligram (mg)<br>miligram_dissolved_in_liquid — miligram (mg) dissolved in liquid (e.g. 2 mg per 3 militers)<br>gram — gram (g)<br>militer — militer (ml)<br>centiliter — centiliter (cl)<br>litre — litre (l)<br>ml_of _solution — ml of solution (e.g. ml of 10% solution)<br>litre_of_solution — litre of solution (e.g. 1 litre of 5% solution)<br>cubic_meter — cubic meter (m^3)<br>other — other<br>dont_know — don't know |
| q404_M23c.1 | q404_M23c.1 If other unit of measurement or route of administration, specify here:<br>*Leave blank if not applicable*<br>*Question relevant when: selected( ${q104_M23_full_list} , '3')* | |
| q404_M23d *(required)* | q404_M23d. In total, how many units are used to treat a patient during all of her care?<br>*Remember to add all units! E.g. 5 days x 200 mg twice per day = 2,000mg*<br>*Question relevant when: selected( ${q104_M23_full_list} , '3')*<br>*Response constrained to: .>0* | |
| q404_M24 | ***q404_M24. Lidocaine HCl, 1%***<br>*Question relevant when: selected( ${q104_M24_full_list} , '3')* | |

| Field | Question | Answer |
|---|---|---|
| q404_M24a *(required)* | q404_M24a. What percent of patients require this item?<br>*Question relevant when: selected( ${q104_M24_full_list} , '3')*<br>*Response constrained to: .>0 and .<=100 or .=999* | |
| q404_M24b *(required)* | q404_M24b. How is the item administered, or given, to the patient?<br>*Question relevant when: selected( ${q104_M24_full_list} , '3')* | 1 Oral - solid (e.g. tablet)<br>2 Oral - liquid<br>3 Sublingual<br>4 Intravenous (IV)<br>5 Intramusculuar injection (IM)<br>6 Subcutaneous<br>7 Rectal<br>8 Inhaled (e.g. gas)<br>9 Inserted (e.g. vaginal pessaries)<br>10 Topical - cream<br>11 Topical - drops (e.g. eye drops)<br>12 Other<br>99 Don't know |
| q404_M24c *(required)* | q404_M24c. What is the item's smallest unit of measurement (mg, mcg, ml, g, etc)?<br>*Question relevant when: selected( ${q104_M24_full_list} , '3')* | microgram: microgram (mcg)<br>miligram: miligram (mg)<br>miligram_dissolved_in_liquid: miligram (mg) dissolved in liquid (e.g. 2 mg per 3 militers)<br>gram: gram (g)<br>militer: militer (ml)<br>centiliter: centiliter (cl)<br>litre: litre (l)<br>ml_of _solution: ml of solution (e.g. ml of 10% solution)<br>litre_of_solution: litre of solution (e.g. 1 litre of 5% solution)<br>cubic_meter: cubic meter (m^3)<br>other: other<br>dont_know: don't know |
| q404_M24c.1 | q404_M24c.1 If other unit of measurement or route of administration, specify here:<br>*Leave blank if not applicable*<br>*Question relevant when: selected( ${q104_M24_full_list} , '3')* | |
| q404_M24d *(required)* | q404_M24d. In total, how many units are used to treat a patient during all of her care?<br>*Remember to add all units! E.g. 5 days x 200 mg twice per day = 2,000mg*<br>*Question relevant when: selected( ${q104_M24_full_list} , '3')*<br>*Response constrained to: .>0* | |
| q404_M25 | ***q404_M25. Anesthetic - other 1: "[q104_M25_full_list_other]"***<br>*Question relevant when: selected( ${q104_M25_full_list} , '3')* | |

| Field | Question | Answer |
|---|---|---|
| q404_M25a *(required)* | q404_M25a. What percent of patients require this item?<br>*Question relevant when: selected( ${q104_M25_full_list} , '3')*<br>*Response constrained to: .>0 and .<=100 or .=999* | |
| q404_M25b *(required)* | q404_M25b. How is the item administered, or given, to the patient?<br>*Question relevant when: selected( ${q104_M25_full_list} , '3')* | 1 Oral - solid (e.g. tablet)<br>2 Oral - liquid<br>3 Sublingual<br>4 Intravenous (IV)<br>5 Intramusculuar injection (IM)<br>6 Subcutaneous<br>7 Rectal<br>8 Inhaled (e.g. gas)<br>9 Inserted (e.g. vaginal pessaries)<br>10 Topical - cream<br>11 Topical - drops (e.g. eye drops)<br>12 Other<br>99 Don't know |
| q404_M25c *(required)* | q404_M25c. What is the item's smallest unit of measurement (mg, mcg, ml, g, etc)?<br>*Question relevant when: selected( ${q104_M25_full_list} , '3')* | microgram — microgram (mcg)<br>miligram — miligram (mg)<br>miligram_dissolved_in_liquid — miligram (mg) dissolved in liquid (e.g. 2 mg per 3 militers)<br>gram — gram (g)<br>militer — militer (ml)<br>centiliter — centiliter (cl)<br>litre — litre (l)<br>ml_of _solution — ml of solution (e.g. ml of 10% solution)<br>litre_of_solution — litre of solution (e.g. 1 litre of 5% solution)<br>cubic_meter — cubic meter (m^3)<br>other — other<br>dont_know — don't know |
| q404_M25c.1 | q404_M25c.1 If other unit of measurement or route of administration, specify here:<br>*Leave blank if not applicable*<br>*Question relevant when: selected( ${q104_M25_full_list} , '3')* | |
| q404_M25d *(required)* | q404_M25d. In total, how many units are used to treat a patient during all of her care?<br>*Remember to add all units! E.g. 5 days x 200 mg twice per day = 2,000mg*<br>*Question relevant when: selected( ${q104_M25_full_list} , '3')*<br>*Response constrained to: .>0* | |
| q404_M26 | ***q404_M26. Anesthetic - other 2: "[q104_M26_full_list_other]"***<br>*Question relevant when: selected( ${q104_M26_full_list} , '3')* | |

| Field | Question | Answer |
|---|---|---|
| q404_M26a *(required)* | q404_M26a. What percent of patients require this item?<br>*Question relevant when: selected( ${q104_M26_full_list} , '3')*<br>*Response constrained to: .>0 and .<=100 or .=999* | |

| Field | Question | Answer |
|---|---|---|
| q404_M26b *(required)* | q404_M26b. How is the item administered, or given, to the patient?<br>*Question relevant when: selected( ${q104_M26_full_list} , '3')* | 1 Oral - solid (e.g. tablet)<br>2 Oral - liquid<br>3 Sublingual<br>4 Intravenous (IV)<br>5 Intramusculuar injection (IM)<br>6 Subcutaneous<br>7 Rectal<br>8 Inhaled (e.g. gas)<br>9 Inserted (e.g. vaginal pessaries)<br>10 Topical - cream<br>11 Topical - drops (e.g. eye drops)<br>12 Other<br>99 Don't know |
| q404_M26c *(required)* | q404_M26c. What is the item's smallest unit of measurement (mg, mcg, ml, g, etc)?<br>*Question relevant when: selected( ${q104_M26_full_list} , '3')* | microgram — microgram (mcg)<br>miligram — miligram (mg)<br>miligram_dissolved_in_liquid — miligram (mg) dissolved in liquid (e.g. 2 mg per 3 militers)<br>gram — gram (g)<br>militer — militer (ml)<br>centiliter — centiliter (cl)<br>litre — litre (l)<br>ml_of _solution — ml of solution (e.g. ml of 10% solution)<br>litre_of_solution — litre of solution (e.g. 1 litre of 5% solution)<br>cubic_meter — cubic meter (m^3)<br>other — other<br>dont_know — don't know |
| q404_M26c.1 | q404_M26c.1 If other unit of measurement or route of administration, specify here:<br>*Leave blank if not applicable*<br>*Question relevant when: selected( ${q104_M26_full_list} , '3')* | |
| q404_M26d *(required)* | q404_M26d. In total, how many units are used to treat a patient during all of her care?<br>*Remember to add all units! E.g. 5 days x 200 mg twice per day = 2,000mg*<br>*Question relevant when: selected( ${q104_M26_full_list} , '3')*<br>*Response constrained to: .>0* | |
| q404_M27 | ***q404_M27. Anesthetic - other 3: "[q104_M27_full_list_other]"***<br>*Question relevant when: selected( ${q104_M27_full_list} , '3')* | |

| Field | Question | Answer |
|---|---|---|
| q404_M27a *(required)* | q404_M27a. What percent of patients require this item?<br>*Question relevant when: selected( ${q104_M27_full_list} , '3')*<br>*Response constrained to: .>0 and .<=100 or .=999* | |
| q404_M27b *(required)* | q404_M27b. How is the item administered, or given, to the patient?<br>*Question relevant when: selected( ${q104_M27_full_list} , '3')* | 1 Oral - solid (e.g. tablet)<br>2 Oral - liquid<br>3 Sublingual<br>4 Intravenous (IV)<br>5 Intramusculuar injection (IM)<br>6 Subcutaneous<br>7 Rectal<br>8 Inhaled (e.g. gas)<br>9 Inserted (e.g. vaginal pessaries)<br>10 Topical - cream<br>11 Topical - drops (e.g. eye drops)<br>12 Other<br>99 Don't know |
| q404_M27c *(required)* | q404_M27c. What is the item's smallest unit of measurement (mg, mcg, ml, g, etc)?<br>*Question relevant when: selected( ${q104_M27_full_list} , '3')* | microgram — microgram (mcg)<br>miligram — miligram (mg)<br>miligram_dissolved_in_liquid — miligram (mg) dissolved in liquid (e.g. 2 mg per 3 militers)<br>gram — gram (g)<br>militer — militer (ml)<br>centiliter — centiliter (cl)<br>litre — litre (l)<br>ml_of _solution — ml of solution (e.g. ml of 10% solution)<br>litre_of_solution — litre of solution (e.g. 1 litre of 5% solution)<br>cubic_meter — cubic meter (m^3)<br>other — other<br>dont_know — don't know |
| q404_M27c.1 | q404_M27c.1 If other unit of measurement or route of administration, specify here:<br>*Leave blank if not applicable*<br>*Question relevant when: selected( ${q104_M27_full_list} , '3')* | |
| q404_M27d *(required)* | q404_M27d. In total, how many units are used to treat a patient during all of her care?<br>*Remember to add all units! E.g. 5 days x 200 mg twice per day = 2,000mg*<br>*Question relevant when: selected( ${q104_M27_full_list} , '3')*<br>*Response constrained to: .>0* | |
| q404_M28 | ***q404_M28. Anesthetic - other 4: "[q104_M28_full_list_other]"***<br>*Question relevant when: selected( ${q104_M28_full_list} , '3')* | |

| Field | Question | Answer |
|---|---|---|
| q404_M28a *(required)* | q404_M28a. What percent of patients require this item?<br>*Question relevant when: selected( ${q104_M28_full_list} , '3')*<br>*Response constrained to: .>0 and .<=100 or .=999* | |
| q404_M28b *(required)* | q404_M28b. How is the item administered, or given, to the patient?<br>*Question relevant when: selected( ${q104_M28_full_list} , '3')* | 1 Oral - solid (e.g. tablet)<br>2 Oral - liquid<br>3 Sublingual<br>4 Intravenous (IV)<br>5 Intramusculuar injection (IM)<br>6 Subcutaneous<br>7 Rectal<br>8 Inhaled (e.g. gas)<br>9 Inserted (e.g. vaginal pessaries)<br>10 Topical - cream<br>11 Topical - drops (e.g. eye drops)<br>12 Other<br>99 Don't know |
| q404_M28c *(required)* | q404_M28c. What is the item's smallest unit of measurement (mg, mcg, ml, g, etc)?<br>*Question relevant when: selected( ${q104_M28_full_list} , '3')* | microgram — microgram (mcg)<br>miligram — miligram (mg)<br>miligram_dissolved_in_liquid — miligram (mg) dissolved in liquid (e.g. 2 mg per 3 militers)<br>gram — gram (g)<br>militer — militer (ml)<br>centiliter — centiliter (cl)<br>litre — litre (l)<br>ml_of _solution — ml of solution (e.g. ml of 10% solution)<br>litre_of_solution — litre of solution (e.g. 1 litre of 5% solution)<br>cubic_meter — cubic meter (m^3)<br>other — other<br>dont_know — don't know |
| q404_M28c.1 | q404_M28c.1 If other unit of measurement or route of administration, specify here:<br>*Leave blank if not applicable*<br>*Question relevant when: selected( ${q104_M28_full_list} , '3')* | |
| q404_M28d *(required)* | q404_M28d. In total, how many units are used to treat a patient during all of her care?<br>*Remember to add all units! E.g. 5 days x 200 mg twice per day = 2,000mg*<br>*Question relevant when: selected( ${q104_M28_full_list} , '3')*<br>*Response constrained to: .>0* | |
| q404_M29 | ***q404_M29. Anesthetic - other 5: "[q104_M29_full_list_other]"***<br>*Question relevant when: selected( ${q104_M29_full_list} , '3')* | |

| Field | Question | Answer |
|---|---|---|
| q404_M29a *(required)* | q404_M29a. What percent of patients require this item?<br>*Question relevant when: selected( ${q104_M29_full_list} , '3')*<br>*Response constrained to: .>0 and .<=100 or .=999* | |
| q404_M29b *(required)* | q404_M29b. How is the item administered, or given, to the patient?<br>*Question relevant when: selected( ${q104_M29_full_list} , '3')* | 1 Oral - solid (e.g. tablet)<br>2 Oral - liquid<br>3 Sublingual<br>4 Intravenous (IV)<br>5 Intramusculuar injection (IM)<br>6 Subcutaneous<br>7 Rectal<br>8 Inhaled (e.g. gas)<br>9 Inserted (e.g. vaginal pessaries)<br>10 Topical - cream<br>11 Topical - drops (e.g. eye drops)<br>12 Other<br>99 Don't know |
| q404_M29c *(required)* | q404_M29c. What is the item's smallest unit of measurement (mg, mcg, ml, g, etc)?<br>*Question relevant when: selected( ${q104_M29_full_list} , '3')* | microgram — microgram (mcg)<br>miligram — miligram (mg)<br>miligram_dissolved_in_liquid — miligram (mg) dissolved in liquid (e.g. 2 mg per 3 militers)<br>gram — gram (g)<br>militer — militer (ml)<br>centiliter — centiliter (cl)<br>litre — litre (l)<br>ml_of _solution — ml of solution (e.g. ml of 10% solution)<br>litre_of_solution — litre of solution (e.g. 1 litre of 5% solution)<br>cubic_meter — cubic meter (m^3)<br>other — other<br>dont_know — don't know |
| q404_M29c.1 | q404_M29c.1 If other unit of measurement or route of administration, specify here:<br>*Leave blank if not applicable*<br>*Question relevant when: selected( ${q104_M29_full_list} , '3')* | |
| q404_M29d *(required)* | q404_M29d. In total, how many units are used to treat a patient during all of her care?<br>*Remember to add all units! E.g. 5 days x 200 mg twice per day = 2,000mg*<br>*Question relevant when: selected( ${q104_M29_full_list} , '3')*<br>*Response constrained to: .>0* | |

<p><strong><em>D. Medications - Shock (3)</em></strong></p>

*Group relevant when: selected( ${section_four_skip_med} , '1')*

| Field | Question | Answer |
|---|---|---|
| q404_med_note3 | 404 CONTINUED For each of the following medications and other medical products that are used for shock, can you tell me …? | |
| note_404_antibiotics | ***Antibiotics*** | |
| q404_M30 | ***q404_M30. Amoxicillin***<br>*Question relevant when: selected( ${q104_M30_full_list} , '3')* | |
| q404_M30a *(required)* | q404_M30a. What percent of patients require this item?<br>*Question relevant when: selected( ${q104_M30_full_list} , '3')*<br>*Response constrained to: .>0 and .<=100 or .=999* | |
| q404_M30b *(required)* | q404_M30b. How is the item administered, or given, to the patient?<br>*Question relevant when: selected( ${q104_M30_full_list} , '3')* | 1 Oral - solid (e.g. tablet)<br>2 Oral - liquid<br>3 Sublingual<br>4 Intravenous (IV)<br>5 Intramusculuar injection (IM)<br>6 Subcutaneous<br>7 Rectal<br>8 Inhaled (e.g. gas)<br>9 Inserted (e.g. vaginal pessaries)<br>10 Topical - cream<br>11 Topical - drops (e.g. eye drops)<br>12 Other<br>99 Don't know |
| q404_M30c *(required)* | q404_M30c. What is the item's smallest unit of measurement (mg, mcg, ml, g, etc)?<br>*Question relevant when: selected( ${q104_M30_full_list} , '3')* | microgram — microgram (mcg)<br>miligram — miligram (mg)<br>miligram_dissolved_in_liquid — miligram (mg) dissolved in liquid (e.g. 2 mg per 3 militers)<br>gram — gram (g)<br>militer — militer (ml)<br>centiliter — centiliter (cl)<br>litre — litre (l)<br>ml_of _solution — ml of solution (e.g. ml of 10% solution)<br>litre_of_solution — litre of solution (e.g. 1 litre of 5% solution)<br>cubic_meter — cubic meter (m^3)<br>other — other<br>dont_know — don't know |
| q404_M30c.1 | q404_M30c.1 If other unit of measurement or route of administration, specify here:<br>*Leave blank if not applicable*<br>*Question relevant when: selected( ${q104_M30_full_list} , '3')* | |
| q404_M30d *(required)* | q404_M30d. In total, how many units are used to treat a patient during all of her care?<br>*Remember to add all units! E.g. 5 days x 200 mg twice per day = 2,000mg*<br>*Question relevant when: selected( ${q104_M30_full_list} , '3')*<br>*Response constrained to: .>0* | |
| q404_M31 | ***q404_M31. Amoxicillin + Clauvulenic Acid 635mg***<br>*Question relevant when: selected( ${q104_M31_full_list} , '3')* | |

| Field | Question | Answer |
|---|---|---|
| q404_M31a *(required)* | q404_M31a. What percent of patients require this item?<br>*Question relevant when: selected( ${q104_M31_full_list} , '3')*<br>*Response constrained to: .>0 and .<=100 or .=999* | |
| q404_M31b *(required)* | q404_M31b. How is the item administered, or given, to the patient?<br>*Question relevant when: selected( ${q104_M31_full_list} , '3')* | 1 Oral - solid (e.g. tablet)<br>2 Oral - liquid<br>3 Sublingual<br>4 Intravenous (IV)<br>5 Intramusculuar injection (IM)<br>6 Subcutaneous<br>7 Rectal<br>8 Inhaled (e.g. gas)<br>9 Inserted (e.g. vaginal pessaries)<br>10 Topical - cream<br>11 Topical - drops (e.g. eye drops)<br>12 Other<br>99 Don't know |
| q404_M31c *(required)* | q404_M31c. What is the item's smallest unit of measurement (mg, mcg, ml, g, etc)?<br>*Question relevant when: selected( ${q104_M31_full_list} , '3')* | microgram — microgram (mcg)<br>miligram — miligram (mg)<br>miligram_dissolved_in_liquid — miligram (mg) dissolved in liquid (e.g. 2 mg per 3 militers)<br>gram — gram (g)<br>militer — militer (ml)<br>centiliter — centiliter (cl)<br>litre — litre (l)<br>ml_of _solution — ml of solution (e.g. ml of 10% solution)<br>litre_of_solution — litre of solution (e.g. 1 litre of 5% solution)<br>cubic_meter — cubic meter (m^3)<br>other — other<br>dont_know — don't know |
| q404_M31c.1 | q404_M31c.1 If other unit of measurement or route of administration, specify here:<br>*Leave blank if not applicable*<br>*Question relevant when: selected( ${q104_M31_full_list} , '3')* | |
| q404_M31d *(required)* | q404_M31d. In total, how many units are used to treat a patient during all of her care?<br>*Remember to add all units! E.g. 5 days x 200 mg twice per day = 2,000mg*<br>*Question relevant when: selected( ${q104_M31_full_list} , '3')*<br>*Response constrained to: .>0* | |
| q404_M32 | ***q404_M32. Ampicillin***<br>*Question relevant when: selected( ${q104_M32_full_list} , '3')* | |

| Field | Question | Answer |
|---|---|---|
| q404_M32a *(required)* | q404_M32a. What percent of patients require this item?<br>*Question relevant when: selected( ${q104_M32_full_list} , '3')*<br>*Response constrained to: .>0 and .<=100 or .=999* | |

| Field | Question | Answer |
|---|---|---|
| q404_M32b *(required)* | q404_M32b. How is the item administered, or given, to the patient?<br>*Question relevant when: selected( ${q104_M32_full_list} , '3')* | 1 Oral - solid (e.g. tablet)<br>2 Oral - liquid<br>3 Sublingual<br>4 Intravenous (IV)<br>5 Intramuscluar injection (IM)<br>6 Subcutaneous<br>7 Rectal<br>8 Inhaled (e.g. gas)<br>9 Inserted (e.g. vaginal pessaries)<br>10 Topical - cream<br>11 Topical - drops (e.g. eye drops)<br>12 Other<br>99 Don't know |
| q404_M32c *(required)* | q404_M32c. What is the item's smallest unit of measurement (mg, mcg, ml, g, etc)?<br>*Question relevant when: selected( ${q104_M32_full_list} , '3')* | microgram — microgram (mcg)<br>miligram — miligram (mg)<br>miligram_dissolved_in_liquid — miligram (mg) dissolved in liquid (e.g. 2 mg per 3 militers)<br>gram — gram (g)<br>militer — militer (ml)<br>centiliter — centiliter (cl)<br>litre — litre (l)<br>ml_of _solution — ml of solution (e.g. ml of 10% solution)<br>litre_of_solution — litre of solution (e.g. 1 litre of 5% solution)<br>cubic_meter — cubic meter (m^3)<br>other — other<br>dont_know — don't know |
| q404_M32c.1 | q404_M32c.1 If other unit of measurement or route of administration, specify here:<br>*Leave blank if not applicable*<br>*Question relevant when: selected( ${q104_M32_full_list} , '3')* | |
| q404_M32d *(required)* | q404_M32d. In total, how many units are used to treat a patient during all of her care?<br>*Remember to add all units! E.g. 5 days x 200 mg twice per day = 2,000mg*<br>*Question relevant when: selected( ${q104_M32_full_list} , '3')*<br>*Response constrained to: .>0* | |
| q404_M33 | ***q404_M33. Ampicillin (injection)***<br>*Question relevant when: selected( ${q104_M33_full_list} , '3')* | |

| Field | Question | Answer |
|---|---|---|
| q404_M33a *(required)* | q404_M33a. What percent of patients require this item?<br>*Question relevant when: selected( ${q104_M33_full_list} , '3')*<br>*Response constrained to: .>0 and .<=100 or .=999* | |
| q404_M33b *(required)* | q404_M33b. How is the item administered, or given, to the patient?<br>*Question relevant when: selected( ${q104_M33_full_list} , '3')* | 1 Oral - solid (e.g. tablet)<br>2 Oral - liquid<br>3 Sublingual<br>4 Intravenous (IV)<br>5 Intramusculuar injection (IM)<br>6 Subcutaneous<br>7 Rectal<br>8 Inhaled (e.g. gas)<br>9 Inserted (e.g. vaginal pessaries)<br>10 Topical - cream<br>11 Topical - drops (e.g. eye drops)<br>12 Other<br>99 Don't know |
| q404_M33c *(required)* | q404_M33c. What is the item's smallest unit of measurement (mg, mcg, ml, g, etc)?<br>*Question relevant when: selected( ${q104_M33_full_list} , '3')* | microgram — microgram (mcg)<br>miligram — miligram (mg)<br>miligram_dissolved_in_liquid — miligram (mg) dissolved in liquid (e.g. 2 mg per 3 militers)<br>gram — gram (g)<br>militer — militer (ml)<br>centiliter — centiliter (cl)<br>litre — litre (l)<br>ml_of _solution — ml of solution (e.g. ml of 10% solution)<br>litre_of_solution — litre of solution (e.g. 1 litre of 5% solution)<br>cubic_meter — cubic meter (m^3)<br>other — other<br>dont_know — don't know |
| q404_M33c.1 | q404_M33c.1 If other unit of measurement or route of administration, specify here:<br>*Leave blank if not applicable*<br>*Question relevant when: selected( ${q104_M33_full_list} , '3')* | |
| q404_M33d *(required)* | q404_M33d. In total, how many units are used to treat a patient during all of her care?<br>*Remember to add all units! E.g. 5 days x 200 mg twice per day = 2,000mg*<br>*Question relevant when: selected( ${q104_M33_full_list} , '3')*<br>*Response constrained to: .>0* | |
| q404_M34 | ***q404_M34. Ampicillin/sulbactam (Unasyn)***<br>*Question relevant when: selected( ${q104_M34_full_list} , '3')* | |

| Field | Question | Answer |
|---|---|---|
| q404_M34a *(required)* | q404_M34a. What percent of patients require this item?<br>*Question relevant when: selected( ${q104_M34_full_list} , '3')*<br>*Response constrained to: .>0 and .<=100 or .=999* | |
| q404_M34b *(required)* | q404_M34b. How is the item administered, or given, to the patient?<br>*Question relevant when: selected( ${q104_M34_full_list} , '3')* | 1 Oral - solid (e.g. tablet)<br>2 Oral - liquid<br>3 Sublingual<br>4 Intravenous (IV)<br>5 Intramusculuar injection (IM)<br>6 Subcutaneous<br>7 Rectal<br>8 Inhaled (e.g. gas)<br>9 Inserted (e.g. vaginal pessaries)<br>10 Topical - cream<br>11 Topical - drops (e.g. eye drops)<br>12 Other<br>99 Don't know |
| q404_M34c *(required)* | q404_M34c. What is the item's smallest unit of measurement (mg, mcg, ml, g, etc)?<br>*Question relevant when: selected( ${q104_M34_full_list} , '3')* | microgram — microgram (mcg)<br>miligram — miligram (mg)<br>miligram_dissolved_in_liquid — miligram (mg) dissolved in liquid (e.g. 2 mg per 3 militers)<br>gram — gram (g)<br>militer — militer (ml)<br>centiliter — centiliter (cl)<br>litre — litre (l)<br>ml_of _solution — ml of solution (e.g. ml of 10% solution)<br>litre_of_solution — litre of solution (e.g. 1 litre of 5% solution)<br>cubic_meter — cubic meter (m^3)<br>other — other<br>dont_know — don't know |
| q404_M34c.1 | q404_M34c.1 If other unit of measurement or route of administration, specify here:<br>*Leave blank if not applicable*<br>*Question relevant when: selected( ${q104_M34_full_list} , '3')* | |
| q404_M34d *(required)* | q404_M34d. In total, how many units are used to treat a patient during all of her care?<br>*Remember to add all units! E.g. 5 days x 200 mg twice per day = 2,000mg*<br>*Question relevant when: selected( ${q104_M34_full_list} , '3')*<br>*Response constrained to: .>0* | |
| q404_M35 | ***q404_M35. Ceftriaxone***<br>*Question relevant when: selected( ${q104_M35_full_list} , '3')* | |

| Field | Question | Answer |
|---|---|---|
| q404_M35a *(required)* | q404_M35a. What percent of patients require this item?<br>*Question relevant when: selected( ${q104_M35_full_list} , '3')*<br>*Response constrained to: .>0 and .<=100 or .=999* | |
| q404_M35b *(required)* | q404_M35b. How is the item administered, or given, to the patient?<br>*Question relevant when: selected( ${q104_M35_full_list} , '3')* | 1 Oral - solid (e.g. tablet)<br>2 Oral - liquid<br>3 Sublingual<br>4 Intravenous (IV)<br>5 Intramusculuar injection (IM)<br>6 Subcutaneous<br>7 Rectal<br>8 Inhaled (e.g. gas)<br>9 Inserted (e.g. vaginal pessaries)<br>10 Topical - cream<br>11 Topical - drops (e.g. eye drops)<br>12 Other<br>99 Don't know |
| q404_M35c *(required)* | q404_M35c. What is the item's smallest unit of measurement (mg, mcg, ml, g, etc)?<br>*Question relevant when: selected( ${q104_M35_full_list} , '3')* | microgram — microgram (mcg)<br>miligram — miligram (mg)<br>miligram_dissolved_in_liquid — miligram (mg) dissolved in liquid (e.g. 2 mg per 3 militers)<br>gram — gram (g)<br>militer — militer (ml)<br>centiliter — centiliter (cl)<br>litre — litre (l)<br>ml_of _solution — ml of solution (e.g. ml of 10% solution)<br>litre_of_solution — litre of solution (e.g. 1 litre of 5% solution)<br>cubic_meter — cubic meter (m^3)<br>other — other<br>dont_know — don't know |
| q404_M35c.1 | q404_M35c.1 If other unit of measurement or route of administration, specify here:<br>*Leave blank if not applicable*<br>*Question relevant when: selected( ${q104_M35_full_list} , '3')* | |
| q404_M35d *(required)* | q404_M35d. In total, how many units are used to treat a patient during all of her care?<br>*Remember to add all units! E.g. 5 days x 200 mg twice per day = 2,000mg*<br>*Question relevant when: selected( ${q104_M35_full_list} , '3')*<br>*Response constrained to: .>0* | |
| q404_M36 | ***q404_M36. Chloramphenicol Sodium Succinate (injection)***<br>*Question relevant when: selected( ${q104_M36_full_list} , '3')* | |

| Field | Question | Answer |
|---|---|---|
| q404_M36a *(required)* | q404_M36a. What percent of patients require this item?<br>*Question relevant when: selected( ${q104_M36_full_list} , '3')*<br>*Response constrained to: .>0 and .<=100 or .=999* | |
| q404_M36b *(required)* | q404_M36b. How is the item administered, or given, to the patient?<br>*Question relevant when: selected( ${q104_M36_full_list} , '3')* | 1 Oral - solid (e.g. tablet)<br>2 Oral - liquid<br>3 Sublingual<br>4 Intravenous (IV)<br>5 Intramusculuar injection (IM)<br>6 Subcutaneous<br>7 Rectal<br>8 Inhaled (e.g. gas)<br>9 Inserted (e.g. vaginal pessaries)<br>10 Topical - cream<br>11 Topical - drops (e.g. eye drops)<br>12 Other<br>99 Don't know |
| q404_M36c *(required)* | q404_M36c. What is the item's smallest unit of measurement (mg, mcg, ml, g, etc)?<br>*Question relevant when: selected( ${q104_M36_full_list} , '3')* | microgram — microgram (mcg)<br>miligram — miligram (mg)<br>miligram_dissolved_in_liquid — miligram (mg) dissolved in liquid (e.g. 2 mg per 3 militers)<br>gram — gram (g)<br>militer — militer (ml)<br>centiliter — centiliter (cl)<br>litre — litre (l)<br>ml_of _solution — ml of solution (e.g. ml of 10% solution)<br>litre_of_solution — litre of solution (e.g. 1 litre of 5% solution)<br>cubic_meter — cubic meter (m^3)<br>other — other<br>dont_know — don't know |
| q404_M36c.1 | q404_M36c.1 If other unit of measurement or route of administration, specify here:<br>*Leave blank if not applicable*<br>*Question relevant when: selected( ${q104_M36_full_list} , '3')* | |
| q404_M36d *(required)* | q404_M36d. In total, how many units are used to treat a patient during all of her care?<br>*Remember to add all units! E.g. 5 days x 200 mg twice per day = 2,000mg*<br>*Question relevant when: selected( ${q104_M36_full_list} , '3')*<br>*Response constrained to: .>0* | |
| q404_M37 | ***q404_M37. Ciprofloxacin***<br>*Question relevant when: selected( ${q104_M37_full_list} , '3')* | |

| Field | Question | Answer |
|---|---|---|
| q404_M37a *(required)* | q404_M37a. What percent of patients require this item?<br>*Question relevant when: selected( ${q104_M37_full_list} , '3')*<br>*Response constrained to: .>0 and .<=100 or .=999* | |
| q404_M37b *(required)* | q404_M37b. How is the item administered, or given, to the patient?<br>*Question relevant when: selected( ${q104_M37_full_list} , '3')* | 1 Oral - solid (e.g. tablet)<br>2 Oral - liquid<br>3 Sublingual<br>4 Intravenous (IV)<br>5 Intramusculuar injection (IM)<br>6 Subcutaneous<br>7 Rectal<br>8 Inhaled (e.g. gas)<br>9 Inserted (e.g. vaginal pessaries)<br>10 Topical - cream<br>11 Topical - drops (e.g. eye drops)<br>12 Other<br>99 Don't know |
| q404_M37c *(required)* | q404_M37c. What is the item's smallest unit of measurement (mg, mcg, ml, g, etc)?<br>*Question relevant when: selected( ${q104_M37_full_list} , '3')* | microgram: microgram (mcg)<br>miligram: miligram (mg)<br>miligram_dissolved_in_liquid: miligram (mg) dissolved in liquid (e.g. 2 mg per 3 militers)<br>gram: gram (g)<br>militer: militer (ml)<br>centiliter: centiliter (cl)<br>litre: litre (l)<br>ml_of _solution: ml of solution (e.g. ml of 10% solution)<br>litre_of_solution: litre of solution (e.g. 1 litre of 5% solution)<br>cubic_meter: cubic meter (m^3)<br>other: other<br>dont_know: don't know |
| q404_M37c.1 | q404_M37c.1 If other unit of measurement or route of administration, specify here:<br>*Leave blank if not applicable*<br>*Question relevant when: selected( ${q104_M37_full_list} , '3')* | |
| q404_M37d *(required)* | q404_M37d. In total, how many units are used to treat a patient during all of her care?<br>*Remember to add all units! E.g. 5 days x 200 mg twice per day = 2,000mg*<br>*Question relevant when: selected( ${q104_M37_full_list} , '3')*<br>*Response constrained to: .>0* | |
| q404_M38 | ***q404_M38. Doxycycline***<br>*Question relevant when: selected( ${q104_M38_full_list} , '3')* | |

| Field | Question | Answer |
|---|---|---|
| q404_M38a *(required)* | q404_M38a. What percent of patients require this item?<br>*Question relevant when: selected( ${q104_M38_full_list} , '3')*<br>*Response constrained to: .>0 and .<=100 or .=999* | |
| q404_M38b *(required)* | q404_M38b. How is the item administered, or given, to the patient?<br>*Question relevant when: selected( ${q104_M38_full_list} , '3')* | 1 Oral - solid (e.g. tablet)<br>2 Oral - liquid<br>3 Sublingual<br>4 Intravenous (IV)<br>5 Intramusculuar injection (IM)<br>6 Subcutaneous<br>7 Rectal<br>8 Inhaled (e.g. gas)<br>9 Inserted (e.g. vaginal pessaries)<br>10 Topical - cream<br>11 Topical - drops (e.g. eye drops)<br>12 Other<br>99 Don't know |
| q404_M38c *(required)* | q404_M38c. What is the item's smallest unit of measurement (mg, mcg, ml, g, etc)?<br>*Question relevant when: selected( ${q104_M38_full_list} , '3')* | microgram — microgram (mcg)<br>miligram — miligram (mg)<br>miligram_dissolved_in_liquid — miligram (mg) dissolved in liquid (e.g. 2 mg per 3 militers)<br>gram — gram (g)<br>militer — militer (ml)<br>centiliter — centiliter (cl)<br>litre — litre (l)<br>ml_of _solution — ml of solution (e.g. ml of 10% solution)<br>litre_of_solution — litre of solution (e.g. 1 litre of 5% solution)<br>cubic_meter — cubic meter (m^3)<br>other — other<br>dont_know — don't know |
| q404_M38c.1 | q404_M38c.1 If other unit of measurement or route of administration, specify here:<br>*Leave blank if not applicable*<br>*Question relevant when: selected( ${q104_M38_full_list} , '3')* | |
| q404_M38d *(required)* | q404_M38d. In total, how many units are used to treat a patient during all of her care?<br>*Remember to add all units! E.g. 5 days x 200 mg twice per day = 2,000mg*<br>*Question relevant when: selected( ${q104_M38_full_list} , '3')*<br>*Response constrained to: .>0* | |
| q404_M39 | ***q404_M39. Erythromycin***<br>*Question relevant when: selected( ${q104_M39_full_list} , '3')* | |

| Field | Question | Answer |
|---|---|---|
| q404_M39a *(required)* | q404_M39a. What percent of patients require this item?<br>*Question relevant when: selected( ${q104_M39_full_list} , '3')*<br>*Response constrained to: .>0 and .<=100 or .=999* | |
| q404_M39b *(required)* | q404_M39b. How is the item administered, or given, to the patient?<br>*Question relevant when: selected( ${q104_M39_full_list} , '3')* | 1 Oral - solid (e.g. tablet)<br>2 Oral - liquid<br>3 Sublingual<br>4 Intravenous (IV)<br>5 Intramusculuar injection (IM)<br>6 Subcutaneous<br>7 Rectal<br>8 Inhaled (e.g. gas)<br>9 Inserted (e.g. vaginal pessaries)<br>10 Topical - cream<br>11 Topical - drops (e.g. eye drops)<br>12 Other<br>99 Don't know |
| q404_M39c *(required)* | q404_M39c. What is the item's smallest unit of measurement (mg, mcg, ml, g, etc)?<br>*Question relevant when: selected( ${q104_M39_full_list} , '3')* | microgram — microgram (mcg)<br>miligram — miligram (mg)<br>miligram_dissolved_in_liquid — miligram (mg) dissolved in liquid (e.g. 2 mg per 3 militers)<br>gram — gram (g)<br>militer — militer (ml)<br>centiliter — centiliter (cl)<br>litre — litre (l)<br>ml_of _solution — ml of solution (e.g. ml of 10% solution)<br>litre_of_solution — litre of solution (e.g. 1 litre of 5% solution)<br>cubic_meter — cubic meter (m^3)<br>other — other<br>dont_know — don't know |
| q404_M39c.1 | q404_M39c.1 If other unit of measurement or route of administration, specify here:<br>*Leave blank if not applicable*<br>*Question relevant when: selected( ${q104_M39_full_list} , '3')* | |
| q404_M39d *(required)* | q404_M39d. In total, how many units are used to treat a patient during all of her care?<br>*Remember to add all units! E.g. 5 days x 200 mg twice per day = 2,000mg*<br>*Question relevant when: selected( ${q104_M39_full_list} , '3')*<br>*Response constrained to: .>0* | |
| q404_M40 | ***q404_M40. Gentamycin***<br>*Question relevant when: selected( ${q104_M40_full_list} , '3')* | |

| Field | Question | Answer |
|---|---|---|
| q404_M40a *(required)* | q404_M40a. What percent of patients require this item?<br>*Question relevant when: selected( ${q104_M40_full_list} , '3')*<br>*Response constrained to: .>0 and .<=100 or .=999* | |
| q404_M40b *(required)* | q404_M40b. How is the item administered, or given, to the patient?<br>*Question relevant when: selected( ${q104_M40_full_list} , '3')* | 1 Oral - solid (e.g. tablet)<br>2 Oral - liquid<br>3 Sublingual<br>4 Intravenous (IV)<br>5 Intramuscular injection (IM)<br>6 Subcutaneous<br>7 Rectal<br>8 Inhaled (e.g. gas)<br>9 Inserted (e.g. vaginal pessaries)<br>10 Topical - cream<br>11 Topical - drops (e.g. eye drops)<br>12 Other<br>99 Don't know |
| q404_M40c *(required)* | q404_M40c. What is the item's smallest unit of measurement (mg, mcg, ml, g, etc)?<br>*Question relevant when: selected( ${q104_M40_full_list} , '3')* | microgram — microgram (mcg)<br>miligram — miligram (mg)<br>miligram_dissolved_in_liquid — miligram (mg) dissolved in liquid (e.g. 2 mg per 3 militers)<br>gram — gram (g)<br>militer — militer (ml)<br>centiliter — centiliter (cl)<br>litre — litre (l)<br>ml_of _solution — ml of solution (e.g. ml of 10% solution)<br>litre_of_solution — litre of solution (e.g. 1 litre of 5% solution)<br>cubic_meter — cubic meter (m^3)<br>other — other<br>dont_know — don't know |
| q404_M40c.1 | q404_M40c.1 If other unit of measurement or route of administration, specify here:<br>*Leave blank if not applicable*<br>*Question relevant when: selected( ${q104_M40_full_list} , '3')* | |
| q404_M40d *(required)* | q404_M40d. In total, how many units are used to treat a patient during all of her care?<br>*Remember to add all units! E.g. 5 days x 200 mg twice per day = 2,000mg*<br>*Question relevant when: selected( ${q104_M40_full_list} , '3')*<br>*Response constrained to: .>0* | |
| q404_M41 | ***q404_M41. Metronidazole (for infusion)***<br>*Question relevant when: selected( ${q104_M41_full_list} , '3')* | |

| Field | Question | Answer |
|---|---|---|
| q404_M41a *(required)* | q404_M41a. What percent of patients require this item?<br>*Question relevant when: selected( ${q104_M41_full_list} , '3')*<br>*Response constrained to: .>0 and .<=100 or .=999* | |
| q404_M41b *(required)* | q404_M41b. How is the item administered, or given, to the patient?<br>*Question relevant when: selected( ${q104_M41_full_list} , '3')* | 1 Oral - solid (e.g. tablet)<br>2 Oral - liquid<br>3 Sublingual<br>4 Intravenous (IV)<br>5 Intramusculuar injection (IM)<br>6 Subcutaneous<br>7 Rectal<br>8 Inhaled (e.g. gas)<br>9 Inserted (e.g. vaginal pessaries)<br>10 Topical - cream<br>11 Topical - drops (e.g. eye drops)<br>12 Other<br>99 Don't know |
| q404_M41c *(required)* | q404_M41c. What is the item's smallest unit of measurement (mg, mcg, ml, g, etc)?<br>*Question relevant when: selected( ${q104_M41_full_list} , '3')* | microgram — microgram (mcg)<br>miligram — miligram (mg)<br>miligram_dissolved_in_liquid — miligram (mg) dissolved in liquid (e.g. 2 mg per 3 militers)<br>gram — gram (g)<br>militer — militer (ml)<br>centiliter — centiliter (cl)<br>litre — litre (l)<br>ml_of _solution — ml of solution (e.g. ml of 10% solution)<br>litre_of_solution — litre of solution (e.g. 1 litre of 5% solution)<br>cubic_meter — cubic meter (m^3)<br>other — other<br>dont_know — don't know |
| q404_M41c.1 | q404_M41c.1 If other unit of measurement or route of administration, specify here:<br>*Leave blank if not applicable*<br>*Question relevant when: selected( ${q104_M41_full_list} , '3')* | |
| q404_M41d *(required)* | q404_M41d. In total, how many units are used to treat a patient during all of her care?<br>*Remember to add all units! E.g. 5 days x 200 mg twice per day = 2,000mg*<br>*Question relevant when: selected( ${q104_M41_full_list} , '3')*<br>*Response constrained to: .>0* | |
| q404_M42 | ***q404_M42. Metronidazole (injection)***<br>*Question relevant when: selected( ${q104_M42_full_list} , '3')* | |

| Field | Question | Answer |
|---|---|---|
| q404_M42a *(required)* | q404_M42a. What percent of patients require this item?<br>*Question relevant when: selected( ${q104_M42_full_list} , '3')*<br>*Response constrained to: .>0 and .<=100 or .=999* | |
| q404_M42b *(required)* | q404_M42b. How is the item administered, or given, to the patient?<br>*Question relevant when: selected( ${q104_M42_full_list} , '3')* | 1 Oral - solid (e.g. tablet)<br>2 Oral - liquid<br>3 Sublingual<br>4 Intravenous (IV)<br>5 Intramusculuar injection (IM)<br>6 Subcutaneous<br>7 Rectal<br>8 Inhaled (e.g. gas)<br>9 Inserted (e.g. vaginal pessaries)<br>10 Topical - cream<br>11 Topical - drops (e.g. eye drops)<br>12 Other<br>99 Don't know |
| q404_M42c *(required)* | q404_M42c. What is the item's smallest unit of measurement (mg, mcg, ml, g, etc)?<br>*Question relevant when: selected( ${q104_M42_full_list} , '3')* | microgram — microgram (mcg)<br>miligram — miligram (mg)<br>miligram_dissolved_in_liquid — miligram (mg) dissolved in liquid (e.g. 2 mg per 3 militers)<br>gram — gram (g)<br>militer — militer (ml)<br>centiliter — centiliter (cl)<br>litre — litre (l)<br>ml_of _solution — ml of solution (e.g. ml of 10% solution)<br>litre_of_solution — litre of solution (e.g. 1 litre of 5% solution)<br>cubic_meter — cubic meter (m^3)<br>other — other<br>dont_know — don't know |
| q404_M42c.1 | q404_M42c.1 If other unit of measurement or route of administration, specify here:<br>*Leave blank if not applicable*<br>*Question relevant when: selected( ${q104_M42_full_list} , '3')* | |
| q404_M42d *(required)* | q404_M42d. In total, how many units are used to treat a patient during all of her care?<br>*Remember to add all units! E.g. 5 days x 200 mg twice per day = 2,000mg*<br>*Question relevant when: selected( ${q104_M42_full_list} , '3')*<br>*Response constrained to: .>0* | |
| q404_M43 | ***q404_M43. Metronidazole (oral)***<br>*Question relevant when: selected( ${q104_M43_full_list} , '3')* | |

| Field | Question | Answer |
|---|---|---|
| q404_M43a *(required)* | q404_M43a. What percent of patients require this item?<br>*Question relevant when: selected( ${q104_M43_full_list} , '3')*<br>*Response constrained to: .>0 and .<=100 or .=999* | |
| q404_M43b *(required)* | q404_M43b. How is the item administered, or given, to the patient?<br>*Question relevant when: selected( ${q104_M43_full_list} , '3')* | 1 Oral - solid (e.g. tablet)<br>2 Oral - liquid<br>3 Sublingual<br>4 Intravenous (IV)<br>5 Intramusculuar injection (IM)<br>6 Subcutaneous<br>7 Rectal<br>8 Inhaled (e.g. gas)<br>9 Inserted (e.g. vaginal pessaries)<br>10 Topical - cream<br>11 Topical - drops (e.g. eye drops)<br>12 Other<br>99 Don't know |
| q404_M43c *(required)* | q404_M43c. What is the item's smallest unit of measurement (mg, mcg, ml, g, etc)?<br>*Question relevant when: selected( ${q104_M43_full_list} , '3')* | microgram — microgram (mcg)<br>miligram — miligram (mg)<br>miligram_dissolved_in_liquid — miligram (mg) dissolved in liquid (e.g. 2 mg per 3 militers)<br>gram — gram (g)<br>militer — militer (ml)<br>centiliter — centiliter (cl)<br>litre — litre (l)<br>ml_of _solution — ml of solution (e.g. ml of 10% solution)<br>litre_of_solution — litre of solution (e.g. 1 litre of 5% solution)<br>cubic_meter — cubic meter (m^3)<br>other — other<br>dont_know — don't know |
| q404_M43c.1 | q404_M43c.1 If other unit of measurement or route of administration, specify here:<br>*Leave blank if not applicable*<br>*Question relevant when: selected( ${q104_M43_full_list} , '3')* | |
| q404_M43d *(required)* | q404_M43d. In total, how many units are used to treat a patient during all of her care?<br>*Remember to add all units! E.g. 5 days x 200 mg twice per day = 2,000mg*<br>*Question relevant when: selected( ${q104_M43_full_list} , '3')*<br>*Response constrained to: .>0* | |
| q404_M44 | ***q404_M44. Penicillin***<br>*Question relevant when: selected( ${q104_M44_full_list} , '3')* | |

| Field | Question | Answer |
| --- | --- | --- |
| q404_M44a *(required)* | q404_M44a. What percent of patients require this item?<br>*Question relevant when: selected( ${q104_M44_full_list} , '3')*<br>*Response constrained to: .>0 and .<=100 or .=999* | |
| q404_M44b *(required)* | q404_M44b. How is the item administered, or given, to the patient?<br>*Question relevant when: selected( ${q104_M44_full_list} , '3')* | 1 Oral - solid (e.g. tablet)<br>2 Oral - liquid<br>3 Sublingual<br>4 Intravenous (IV)<br>5 Intramusculuar injection (IM)<br>6 Subcutaneous<br>7 Rectal<br>8 Inhaled (e.g. gas)<br>9 Inserted (e.g. vaginal pessaries)<br>10 Topical - cream<br>11 Topical - drops (e.g. eye drops)<br>12 Other<br>99 Don't know |
| q404_M44c *(required)* | q404_M44c. What is the item's smallest unit of measurement (mg, mcg, ml, g, etc)?<br>*Question relevant when: selected( ${q104_M44_full_list} , '3')* | microgram — microgram (mcg)<br>miligram — miligram (mg)<br>miligram_dissolved_in_liquid — miligram (mg) dissolved in liquid (e.g. 2 mg per 3 militers)<br>gram — gram (g)<br>militer — militer (ml)<br>centiliter — centiliter (cl)<br>litre — litre (l)<br>ml_of _solution — ml of solution (e.g. ml of 10% solution)<br>litre_of_solution — litre of solution (e.g. 1 litre of 5% solution)<br>cubic_meter — cubic meter (m^3)<br>other — other<br>dont_know — don't know |
| q404_M44c.1 | q404_M44c.1 If other unit of measurement or route of administration, specify here:<br>*Leave blank if not applicable*<br>*Question relevant when: selected( ${q104_M44_full_list} , '3')* | |
| q404_M44d *(required)* | q404_M44d. In total, how many units are used to treat a patient during all of her care?<br>*Remember to add all units! E.g. 5 days x 200 mg twice per day = 2,000mg*<br>*Question relevant when: selected( ${q104_M44_full_list} , '3')*<br>*Response constrained to: .>0* | |
| q404_M45 | ***q404_M45. Antibiotic - other 1: "[q104_M45_full_list_other]"***<br>*Question relevant when: selected( ${q104_M45_full_list} , '3')* | |

| Field | Question | Answer |
|---|---|---|
| q404_M45a *(required)* | q404_M45a. What percent of patients require this item?<br>*Question relevant when: selected( ${q104_M45_full_list} , '3')*<br>*Response constrained to: .>0 and .<=100 or .=999* | |
| q404_M45b *(required)* | q404_M45b. How is the item administered, or given, to the patient?<br>*Question relevant when: selected( ${q104_M45_full_list} , '3')* | 1 Oral - solid (e.g. tablet)<br>2 Oral - liquid<br>3 Sublingual<br>4 Intravenous (IV)<br>5 Intramuscluar injection (IM)<br>6 Subcutaneous<br>7 Rectal<br>8 Inhaled (e.g. gas)<br>9 Inserted (e.g. vaginal pessaries)<br>10 Topical - cream<br>11 Topical - drops (e.g. eye drops)<br>12 Other<br>99 Don't know |
| q404_M45c *(required)* | q404_M45c. What is the item's smallest unit of measurement (mg, mcg, ml, g, etc)?<br>*Question relevant when: selected( ${q104_M45_full_list} , '3')* | microgram — microgram (mcg)<br>miligram — miligram (mg)<br>miligram_dissolved_in_liquid — miligram (mg) dissolved in liquid (e.g. 2 mg per 3 militers)<br>gram — gram (g)<br>militer — militer (ml)<br>centiliter — centiliter (cl)<br>litre — litre (l)<br>ml_of _solution — ml of solution (e.g. ml of 10% solution)<br>litre_of_solution — litre of solution (e.g. 1 litre of 5% solution)<br>cubic_meter — cubic meter (m^3)<br>other — other<br>dont_know — don't know |
| q404_M45c.1 | q404_M45c.1 If other unit of measurement or route of administration, specify here:<br>*Leave blank if not applicable*<br>*Question relevant when: selected( ${q104_M45_full_list} , '3')* | |
| q404_M45d *(required)* | q404_M45d. In total, how many units are used to treat a patient during all of her care?<br>*Remember to add all units! E.g. 5 days x 200 mg twice per day = 2,000mg*<br>*Question relevant when: selected( ${q104_M45_full_list} , '3')*<br>*Response constrained to: .>0* | |
| q404_M46 | ***q404_M46. Antibiotic - other 2: "[q104_M46_full_list_other]"***<br>*Question relevant when: selected( ${q104_M46_full_list} , '3')* | |

| Field | Question | Answer |
|---|---|---|
| q404_M46a *(required)* | q404_M46a. What percent of patients require this item?<br>*Question relevant when: selected( ${q104_M46_full_list} , '3')*<br>*Response constrained to: .>0 and .<=100 or .=999* | |
| q404_M46b *(required)* | q404_M46b. How is the item administered, or given, to the patient?<br>*Question relevant when: selected( ${q104_M46_full_list} , '3')* | 1 Oral - solid (e.g. tablet)<br>2 Oral - liquid<br>3 Sublingual<br>4 Intravenous (IV)<br>5 Intramusculuar injection (IM)<br>6 Subcutaneous<br>7 Rectal<br>8 Inhaled (e.g. gas)<br>9 Inserted (e.g. vaginal pessaries)<br>10 Topical - cream<br>11 Topical - drops (e.g. eye drops)<br>12 Other<br>99 Don't know |
| q404_M46c *(required)* | q404_M46c. What is the item's smallest unit of measurement (mg, mcg, ml, g, etc)?<br>*Question relevant when: selected( ${q104_M46_full_list} , '3')* | microgram: microgram (mcg)<br>miligram: miligram (mg)<br>miligram_dissolved_in_liquid: miligram (mg) dissolved in liquid (e.g. 2 mg per 3 militers)<br>gram: gram (g)<br>militer: militer (ml)<br>centiliter: centiliter (cl)<br>litre: litre (l)<br>ml_of _solution: ml of solution (e.g. ml of 10% solution)<br>litre_of_solution: litre of solution (e.g. 1 litre of 5% solution)<br>cubic_meter: cubic meter (m^3)<br>other: other<br>dont_know: don't know |
| q404_M46c.1 | q404_M46c.1 If other unit of measurement or route of administration, specify here:<br>*Leave blank if not applicable*<br>*Question relevant when: selected( ${q104_M46_full_list} , '3')* | |
| q404_M46d *(required)* | q404_M46d. In total, how many units are used to treat a patient during all of her care?<br>*Remember to add all units! E.g. 5 days x 200 mg twice per day = 2,000mg*<br>*Question relevant when: selected( ${q104_M46_full_list} , '3')*<br>*Response constrained to: .>0* | |
| q404_M47 | ***q404_M47. Antibiotic - other 3: "[q104_M47_full_list_other]"***<br>*Question relevant when: selected( ${q104_M47_full_list} , '3')* | |

| Field | Question | Answer |
|---|---|---|
| q404_M47a *(required)* | q404_M47a. What percent of patients require this item?<br>*Question relevant when: selected( ${q104_M47_full_list} , '3')* | |
| | *Response constrained to: .>0 and .<=100 or .=999* | |

| Field | Question | Answer | | |
|---|---|---|---|---|
| q404_M47b *(required)* | q404_M47b. How is the item administered, or given, to the patient?<br>*Question relevant when: selected( ${q104_M47_full_list} , '3')* | | 1 | Oral - solid (e.g. tablet) |
| | | | 2 | Oral - liquid |
| | | | 3 | Sublingual |
| | | | 4 | Intravenous (IV) |
| | | | 5 | Intramusculuar injection (IM) |
| | | | 6 | Subcutaneous |
| | | | 7 | Rectal |
| | | | 8 | Inhaled (e.g. gas) |
| | | | 9 | Inserted (e.g. vaginal pessaries) |
| | | | 10 | Topical - cream |
| | | | 11 | Topical - drops (e.g. eye drops) |
| | | | 12 | Other |
| | | | 99 | Don't know |
| q404_M47c *(required)* | q404_M47c. What is the item's smallest unit of measurement (mg, mcg, ml, g, etc)?<br>*Question relevant when: selected( ${q104_M47_full_list} , '3')* | | microgram | microgram (mcg) |
| | | | miligram | miligram (mg) |
| | | | miligram_dissolved_in_liquid | miligram (mg) dissolved in liquid (e.g. 2 mg per 3 militers) |
| | | | gram | gram (g) |
| | | | militer | militer (ml) |
| | | | centiliter | centiliter (cl) |
| | | | litre | litre (l) |
| | | | ml_of _solution | ml of solution (e.g. ml of 10% solution) |
| | | | litre_of_solution | litre of solution (e.g. 1 litre of 5% solution) |
| | | | cubic_meter | cubic meter (m^3) |
| | | | other | other |
| | | | dont_know | don't know |
| q404_M47c.1 | q404_M47c.1 If other unit of measurement or route of administration, specify here:<br>*Leave blank if not applicable*<br>*Question relevant when: selected( ${q104_M47_full_list} , '3')* | | | |
| q404_M47d *(required)* | q404_M47d. In total, how many units are used to treat a patient during all of her care?<br>*Remember to add all units! E.g. 5 days x 200 mg twice per day = 2,000mg*<br>*Question relevant when: selected( ${q104_M47_full_list} , '3')*<br>*Response constrained to: .>0* | | | |
| q404_M48 | ***q404_M48. Antibiotic - other 4: "[q104_M48_full_list_other]"***<br>*Question relevant when: selected( ${q104_M48_full_list} , '3')* | | | |

| Field | Question | Answer |
|---|---|---|
| q404_M48a *(required)* | q404_M48a. What percent of patients require this item?<br>*Question relevant when: selected( ${q104_M48_full_list} , '3')* | |
| | *Response constrained to: .>0 and .<=100 or .=999* | |

| Field | Question | Answer |
|---|---|---|
| q404_M48b *(required)* | q404_M48b. How is the item administered, or given, to the patient?<br>*Question relevant when: selected( ${q104_M48_full_list} , '3')* | 1 Oral - solid (e.g. tablet)<br>2 Oral - liquid<br>3 Sublingual<br>4 Intravenous (IV)<br>5 Intramusculuar injection (IM)<br>6 Subcutaneous<br>7 Rectal<br>8 Inhaled (e.g. gas)<br>9 Inserted (e.g. vaginal pessaries)<br>10 Topical - cream<br>11 Topical - drops (e.g. eye drops)<br>12 Other<br>99 Don't know |
| q404_M48c *(required)* | q404_M48c. What is the item's smallest unit of measurement (mg, mcg, ml, g, etc)?<br>*Question relevant when: selected( ${q104_M48_full_list} , '3')* | microgram — microgram (mcg)<br>miligram — miligram (mg)<br>miligram_dissolved_in_liquid — miligram (mg) dissolved in liquid (e.g. 2 mg per 3 militers)<br>gram — gram (g)<br>militer — militer (ml)<br>centiliter — centiliter (cl)<br>litre — litre (l)<br>ml_of _solution — ml of solution (e.g. ml of 10% solution)<br>litre_of_solution — litre of solution (e.g. 1 litre of 5% solution)<br>cubic_meter — cubic meter (m^3)<br>other — other<br>dont_know — don't know |
| q404_M48c.1 | q404_M48c.1 If other unit of measurement or route of administration, specify here:<br>*Leave blank if not applicable*<br>*Question relevant when: selected( ${q104_M48_full_list} , '3')* | |
| q404_M48d *(required)* | q404_M48d. In total, how many units are used to treat a patient during all of her care?<br>*Remember to add all units! E.g. 5 days x 200 mg twice per day = 2,000mg*<br>*Question relevant when: selected( ${q104_M48_full_list} , '3')*<br>*Response constrained to: .>0* | |
| q404_M49 | ***q404_M49. Antibiotic - other 5: "[q104_M49_full_list_other]"***<br>*Question relevant when: selected( ${q104_M49_full_list} , '3')* | |

| Field | Question | Answer |
|---|---|---|
| q404_M49a *(required)* | q404_M49a. What percent of patients require this item?<br>*Question relevant when: selected( ${q104_M49_full_list} , '3')*<br>*Response constrained to: .>0 and .<=100 or .=999* | |
| q404_M49b *(required)* | q404_M49b. How is the item administered, or given, to the patient?<br>*Question relevant when: selected( ${q104_M49_full_list} , '3')* | 1 Oral - solid (e.g. tablet)<br>2 Oral - liquid<br>3 Sublingual<br>4 Intravenous (IV)<br>5 Intramusculuar injection (IM)<br>6 Subcutaneous<br>7 Rectal<br>8 Inhaled (e.g. gas)<br>9 Inserted (e.g. vaginal pessaries)<br>10 Topical - cream<br>11 Topical - drops (e.g. eye drops)<br>12 Other<br>99 Don't know |
| q404_M49c *(required)* | q404_M49c. What is the item's smallest unit of measurement (mg, mcg, ml, g, etc)?<br>*Question relevant when: selected( ${q104_M49_full_list} , '3')* | microgram — microgram (mcg)<br>miligram — miligram (mg)<br>miligram_dissolved_in_liquid — miligram (mg) dissolved in liquid (e.g. 2 mg per 3 militers)<br>gram — gram (g)<br>militer — militer (ml)<br>centiliter — centiliter (cl)<br>litre — litre (l)<br>ml_of _solution — ml of solution (e.g. ml of 10% solution)<br>litre_of_solution — litre of solution (e.g. 1 litre of 5% solution)<br>cubic_meter — cubic meter (m^3)<br>other — other<br>dont_know — don't know |
| q404_M49c.1 | q404_M49c.1 If other unit of measurement or route of administration, specify here:<br>*Leave blank if not applicable*<br>*Question relevant when: selected( ${q104_M49_full_list} , '3')* | |
| q404_M49d *(required)* | q404_M49d. In total, how many units are used to treat a patient during all of her care?<br>*Remember to add all units! E.g. 5 days x 200 mg twice per day = 2,000mg*<br>*Question relevant when: selected( ${q104_M49_full_list} , '3')*<br>*Response constrained to: .>0* | |

<p><strong><em>D. Medications - Shock (4)</em></strong></p>

*Group relevant when: selected( ${section_four_skip_med} , '1')*

| Field | Question | Answer |
|---|---|---|
| q404_med_note4 | 404 CONTINUED For each of the following medications and other medical products that are used for shock, can you tell me …? | |
| note_404_antiseptic | ***Antiseptic*** | |
| q404_M50 | ***q404_M50. Cetrimide 15% ("Savlon")***<br>*Question relevant when: selected( ${q104_M50_full_list} , '3')* | |
| q404_M50a *(required)* | q404_M50a. What percent of patients require this item?<br>*Question relevant when: selected( ${q104_M50_full_list} , '3')*<br>*Response constrained to: .>0 and .<=100 or .=999* | |

| Field | Question | Answer | | |
|---|---|---|---|---|
| q404_M50b *(required)* | q404_M50b. How is the item administered, or given, to the patient?<br>*Question relevant when: selected( ${q104_M50_full_list} , '3')* | | 1 | Oral - solid (e.g. tablet) |
| | | | 2 | Oral - liquid |
| | | | 3 | Sublingual |
| | | | 4 | Intravenous (IV) |
| | | | 5 | Intramusculuar injection (IM) |
| | | | 6 | Subcutaneous |
| | | | 7 | Rectal |
| | | | 8 | Inhaled (e.g. gas) |
| | | | 9 | Inserted (e.g. vaginal pessaries) |
| | | | 10 | Topical - cream |
| | | | 11 | Topical - drops (e.g. eye drops) |
| | | | 12 | Other |
| | | | 99 | Don't know |
| q404_M50c *(required)* | q404_M50c. What is the item's smallest unit of measurement (mg, mcg, ml, g, etc)?<br>*Question relevant when: selected( ${q104_M50_full_list} , '3')* | | microgram | microgram (mcg) |
| | | | miligram | miligram (mg) |
| | | | miligram_dissolved_in_liquid | miligram (mg) dissolved in liquid (e.g. 2 mg per 3 militers) |
| | | | gram | gram (g) |
| | | | militer | militer (ml) |
| | | | centiliter | centiliter (cl) |
| | | | litre | litre (l) |
| | | | ml_of _solution | ml of solution (e.g. ml of 10% solution) |
| | | | litre_of_solution | litre of solution (e.g. 1 litre of 5% solution) |
| | | | cubic_meter | cubic meter (m^3) |
| | | | other | other |
| | | | dont_know | don't know |
| q404_M50c.1 | q404_M50c.1 If other unit of measurement or route of administration, specify here:<br>*Leave blank if not applicable*<br>*Question relevant when: selected( ${q104_M50_full_list} , '3')* | | | |
| q404_M50d *(required)* | q404_M50d. In total, how many units are used to treat a patient during all of her care?<br>*Remember to add all units! E.g. 5 days x 200 mg twice per day = 2,000mg*<br>*Question relevant when: selected( ${q104_M50_full_list} , '3')*<br>*Response constrained to: .>0* | | | |
| q404_M51 | ***q404_M51. Povidone-iodine solution, 7.5%***<br>*Question relevant when: selected( ${q104_M51_full_list} , '3')* | | | |

| Field | Question | Answer |
|---|---|---|
| q404_M51a *(required)* | q404_M51a. What percent of patients require this item?<br>*Question relevant when: selected( ${q104_M51_full_list} , '3')*<br>*Response constrained to: .>0 and .<=100 or .=999* | |
| q404_M51b *(required)* | q404_M51b. How is the item administered, or given, to the patient?<br>*Question relevant when: selected( ${q104_M51_full_list} , '3')* | 1 Oral - solid (e.g. tablet)<br>2 Oral - liquid<br>3 Sublingual<br>4 Intravenous (IV)<br>5 Intramusculuar injection (IM)<br>6 Subcutaneous<br>7 Rectal<br>8 Inhaled (e.g. gas)<br>9 Inserted (e.g. vaginal pessaries)<br>10 Topical - cream<br>11 Topical - drops (e.g. eye drops)<br>12 Other<br>99 Don't know |
| q404_M51c *(required)* | q404_M51c. What is the item's smallest unit of measurement (mg, mcg, ml, g, etc)?<br>*Question relevant when: selected( ${q104_M51_full_list} , '3')* | microgram — microgram (mcg)<br>miligram — miligram (mg)<br>miligram_dissolved_in_liquid — miligram (mg) dissolved in liquid (e.g. 2 mg per 3 militers)<br>gram — gram (g)<br>militer — militer (ml)<br>centiliter — centiliter (cl)<br>litre — litre (l)<br>ml_of _solution — ml of solution (e.g. ml of 10% solution)<br>litre_of_solution — litre of solution (e.g. 1 litre of 5% solution)<br>cubic_meter — cubic meter (m^3)<br>other — other<br>dont_know — don't know |
| q404_M51c.1 | q404_M51c.1 If other unit of measurement or route of administration, specify here:<br>*Leave blank if not applicable*<br>*Question relevant when: selected( ${q104_M51_full_list} , '3')* | |
| q404_M51d *(required)* | q404_M51d. In total, how many units are used to treat a patient during all of her care?<br>*Remember to add all units! E.g. 5 days x 200 mg twice per day = 2,000mg*<br>*Question relevant when: selected( ${q104_M51_full_list} , '3')*<br>*Response constrained to: .>0* | |
| q404_M52 | ***q404_M52. Antiseptic - other 1: "[q104_M52_full_list_other]"***<br>*Question relevant when: selected( ${q104_M52_full_list} , '3')* | |

| Field | Question | Answer |
|---|---|---|
| q404_M52a *(required)* | q404_M52a. What percent of patients require this item?<br>*Question relevant when: selected( ${q104_M52_full_list} , '3')*<br>*Response constrained to: .>0 and .<=100 or .=999* | |
| q404_M52b *(required)* | q404_M52b. How is the item administered, or given, to the patient?<br>*Question relevant when: selected( ${q104_M52_full_list} , '3')* | 1 Oral - solid (e.g. tablet)<br>2 Oral - liquid<br>3 Sublingual<br>4 Intravenous (IV)<br>5 Intramusculuar injection (IM)<br>6 Subcutaneous<br>7 Rectal<br>8 Inhaled (e.g. gas)<br>9 Inserted (e.g. vaginal pessaries)<br>10 Topical - cream<br>11 Topical - drops (e.g. eye drops)<br>12 Other<br>99 Don't know |
| q404_M52c *(required)* | q404_M52c. What is the item's smallest unit of measurement (mg, mcg, ml, g, etc)?<br>*Question relevant when: selected( ${q104_M52_full_list} , '3')* | microgram — microgram (mcg)<br>miligram — miligram (mg)<br>miligram_dissolved_in_liquid — miligram (mg) dissolved in liquid (e.g. 2 mg per 3 militers)<br>gram — gram (g)<br>militer — militer (ml)<br>centiliter — centiliter (cl)<br>litre — litre (l)<br>ml_of _solution — ml of solution (e.g. ml of 10% solution)<br>litre_of_solution — litre of solution (e.g. 1 litre of 5% solution)<br>cubic_meter — cubic meter (m^3)<br>other — other<br>dont_know — don't know |
| q404_M52c.1 | q404_M52c.1 If other unit of measurement or route of administration, specify here:<br>*Leave blank if not applicable*<br>*Question relevant when: selected( ${q104_M52_full_list} , '3')* | |
| q404_M52d *(required)* | q404_M52d. In total, how many units are used to treat a patient during all of her care?<br>*Remember to add all units! E.g. 5 days x 200 mg twice per day = 2,000mg*<br>*Question relevant when: selected( ${q104_M52_full_list} , '3')*<br>*Response constrained to: .>0* | |
| q404_M53 | ***q404_M53. Antiseptic - other 2: "[q104_M53_full_list_other]"***<br>*Question relevant when: selected( ${q104_M53_full_list} , '3')* | |

| Field | Question | Answer |
|---|---|---|
| q404_M53a *(required)* | q404_M53a. What percent of patients require this item?<br>*Question relevant when: selected( ${q104_M53_full_list} , '3')*<br>*Response constrained to: .>0 and .<=100 or .=999* | |
| q404_M53b *(required)* | q404_M53b. How is the item administered, or given, to the patient?<br>*Question relevant when: selected( ${q104_M53_full_list} , '3')* | 1 Oral - solid (e.g. tablet)<br>2 Oral - liquid<br>3 Sublingual<br>4 Intravenous (IV)<br>5 Intramusculuiar injection (IM)<br>6 Subcutaneous<br>7 Rectal<br>8 Inhaled (e.g. gas)<br>9 Inserted (e.g. vaginal pessaries)<br>10 Topical - cream<br>11 Topical - drops (e.g. eye drops)<br>12 Other<br>99 Don't know |
| q404_M53c *(required)* | q404_M53c. What is the item's smallest unit of measurement (mg, mcg, ml, g, etc)?<br>*Question relevant when: selected( ${q104_M53_full_list} , '3')* | microgram — microgram (mcg)<br>miligram — miligram (mg)<br>miligram_dissolved_in_liquid — miligram (mg) dissolved in liquid (e.g. 2 mg per 3 militers)<br>gram — gram (g)<br>militer — militer (ml)<br>centiliter — centiliter (cl)<br>litre — litre (l)<br>ml_of _solution — ml of solution (e.g. ml of 10% solution)<br>litre_of_solution — litre of solution (e.g. 1 litre of 5% solution)<br>cubic_meter — cubic meter (m^3)<br>other — other<br>dont_know — don't know |
| q404_M53c.1 | q404_M53c.1 If other unit of measurement or route of administration, specify here:<br>*Leave blank if not applicable*<br>*Question relevant when: selected( ${q104_M53_full_list} , '3')* | |
| q404_M53d *(required)* | q404_M53d. In total, how many units are used to treat a patient during all of her care?<br>*Remember to add all units! E.g. 5 days x 200 mg twice per day = 2,000mg*<br>*Question relevant when: selected( ${q104_M53_full_list} , '3')*<br>*Response constrained to: .>0* | |
| q404_M54 | ***q404_M54. Antiseptic - other 3: "[q104_M54_full_list_other]"***<br>*Question relevant when: selected( ${q104_M54_full_list} , '3')* | |

| Field | Question | Answer |
|---|---|---|
| q404_M54a *(required)* | q404_M54a. What percent of patients require this item?<br>*Question relevant when: selected( ${q104_M54_full_list} , '3')*<br>*Response constrained to: .>0 and .<=100 or .=999* | |
| q404_M54b *(required)* | q404_M54b. How is the item administered, or given, to the patient?<br>*Question relevant when: selected( ${q104_M54_full_list} , '3')* | 1 Oral - solid (e.g. tablet)<br>2 Oral - liquid<br>3 Sublingual<br>4 Intravenous (IV)<br>5 Intramusculuar injection (IM)<br>6 Subcutaneous<br>7 Rectal<br>8 Inhaled (e.g. gas)<br>9 Inserted (e.g. vaginal pessaries)<br>10 Topical - cream<br>11 Topical - drops (e.g. eye drops)<br>12 Other<br>99 Don't know |
| q404_M54c *(required)* | q404_M54c. What is the item's smallest unit of measurement (mg, mcg, ml, g, etc)?<br>*Question relevant when: selected( ${q104_M54_full_list} , '3')* | microgram — microgram (mcg)<br>miligram — miligram (mg)<br>miligram_dissolved_in_liquid — miligram (mg) dissolved in liquid (e.g. 2 mg per 3 militers)<br>gram — gram (g)<br>militer — militer (ml)<br>centiliter — centiliter (cl)<br>litre — litre (l)<br>ml_of _solution — ml of solution (e.g. ml of 10% solution)<br>litre_of_solution — litre of solution (e.g. 1 litre of 5% solution)<br>cubic_meter — cubic meter (m^3)<br>other — other<br>dont_know — don't know |
| q404_M54c.1 | q404_M54c.1 If other unit of measurement or route of administration, specify here:<br>*Leave blank if not applicable*<br>*Question relevant when: selected( ${q104_M54_full_list} , '3')* | |
| q404_M54d *(required)* | q404_M54d. In total, how many units are used to treat a patient during all of her care?<br>*Remember to add all units! E.g. 5 days x 200 mg twice per day = 2,000mg*<br>*Question relevant when: selected( ${q104_M54_full_list} , '3')*<br>*Response constrained to: .>0* | |
| q404_M54.1 | ***q404_M54.1. Antiseptic - other 4: "[q104_M54.1_full_list_other]"***<br>*Question relevant when: selected( ${q104_M54.1_full_list} , '2')* | |

| Field | Question | Answer |
|---|---|---|
| q404_M54.1a *(required)* | q404_M54.1a. What percent of patients require this item?<br>*Question relevant when: selected( ${q104_M54.1_full_list} , '2')*<br>*Response constrained to: .>0 and .<=100 or .=999* | |
| q404_M54.1b *(required)* | q404_M54.1b. How is the item administered, or given, to the patient?<br>*Question relevant when: selected( ${q104_M54.1_full_list} , '2')* | 1 Oral - solid (e.g. tablet)<br>2 Oral - liquid<br>3 Sublingual<br>4 Intravenous (IV)<br>5 Intramusculuar injection (IM)<br>6 Subcutaneous<br>7 Rectal<br>8 Inhaled (e.g. gas)<br>9 Inserted (e.g. vaginal pessaries)<br>10 Topical - cream<br>11 Topical - drops (e.g. eye drops)<br>12 Other<br>99 Don't know |
| q404_M54.1c *(required)* | q404_M54.1c. What is the item's smallest unit of measurement (mg, mcg, ml, g, etc)?<br>*Question relevant when: selected( ${q104_M54.1_full_list} , '2')* | microgram — microgram (mcg)<br>miligram — miligram (mg)<br>miligram_dissolved_in_liquid — miligram (mg) dissolved in liquid (e.g. 2 mg per 3 militers)<br>gram — gram (g)<br>militer — militer (ml)<br>centiliter — centiliter (cl)<br>litre — litre (l)<br>ml_of _solution — ml of solution (e.g. ml of 10% solution)<br>litre_of_solution — litre of solution (e.g. 1 litre of 5% solution)<br>cubic_meter — cubic meter (m^3)<br>other — other<br>dont_know — don't know |
| q404_M54.1c.1 | q404_M54.1c.1 If other unit of measurement or route of administration, specify here:<br>*Leave blank if not applicable*<br>*Question relevant when: selected( ${q104_M54.1_full_list} , '2')* | |
| q404_M54.1d *(required)* | q404_M54.1d. In total, how many units are used to treat a patient during all of her care?<br>*Remember to add all units! E.g. 5 days x 200 mg twice per day = 2,000mg*<br>*Question relevant when: selected( ${q104_M54.1_full_list} , '2')*<br>*Response constrained to: .>0* | |
| q404_M54.2 | ***q404_M54.2. Antiseptic - other 5: "[q104_M54.2_full_list_other]"***<br>*Question relevant when: selected( ${q104_M54.2_full_list} , '2')* | |

| Field | Question | Answer |
|---|---|---|
| q404_M54.2a *(required)* | q404_M54.2a. What percent of patients require this item?<br>*Question relevant when: selected( ${q104_M54.2_full_list} , '2')*<br>*Response constrained to: .>0 and .<=100 or .=999* | |
| q404_M54.2b *(required)* | q404_M54.2b. How is the item administered, or given, to the patient?<br>*Question relevant when: selected( ${q104_M54.2_full_list} , '2')* | 1 Oral - solid (e.g. tablet)<br>2 Oral - liquid<br>3 Sublingual<br>4 Intravenous (IV)<br>5 Intramusculuar injection (IM)<br>6 Subcutaneous<br>7 Rectal<br>8 Inhaled (e.g. gas)<br>9 Inserted (e.g. vaginal pessaries)<br>10 Topical - cream<br>11 Topical - drops (e.g. eye drops)<br>12 Other<br>99 Don't know |
| q404_M54.2c *(required)* | q404_M54.2c. What is the item's smallest unit of measurement (mg, mcg, ml, g, etc)?<br>*Question relevant when: selected( ${q104_M54.2_full_list} , '2')* | microgram — microgram (mcg)<br>miligram — miligram (mg)<br>miligram_dissolved_in_liquid — miligram (mg) dissolved in liquid (e.g. 2 mg per 3 militers)<br>gram — gram (g)<br>militer — militer (ml)<br>centiliter — centiliter (cl)<br>litre — litre (l)<br>ml_of _solution — ml of solution (e.g. ml of 10% solution)<br>litre_of_solution — litre of solution (e.g. 1 litre of 5% solution)<br>cubic_meter — cubic meter (m^3)<br>other — other<br>dont_know — don't know |
| q404_M54.2c.1 | q404_M54.2c.1 If other unit of measurement or route of administration, specify here:<br>*Leave blank if not applicable*<br>*Question relevant when: selected( ${q104_M54.2_full_list} , '2')* | |
| q404_M54.2d *(required)* | q404_M54.2d. In total, how many units are used to treat a patient during all of her care?<br>*Remember to add all units! E.g. 5 days x 200 mg twice per day = 2,000mg*<br>*Question relevant when: selected( ${q104_M54.2_full_list} , '2')*<br>*Response constrained to: .>0* | |
| <p><strong><em>D. Medications - Shock (5)</em></strong></p><br>*Group relevant when: selected( ${section_four_skip_med} , '1')* | | |

| Field | Question | Answer |
|---|---|---|
| q404_med_notE4 | 404 CONTINUED For each of the following medications and other medical products that are used for shock, can you tell me …? | |
| note_404_antispas | ***Antispasmodic, anxiolytic, sedative, etc*** | |
| q404_M55 | ***q404_M55. Hyoscine butylbromide / scopolamine butilhisocin (Buscopan )***<br>*Question relevant when: selected( ${q104_M55_full_list} , '3')* | |
| q404_M55a *(required)* | q404_M55a. What percent of patients require this item?<br>*Question relevant when: selected( ${q104_M55_full_list} , '3')*<br>*Response constrained to: .>0 and .<=100 or .=999* | |

| Field | Question | Answer |
|---|---|---|
| q404_M55b *(required)* | q404_M55b. How is the item administered, or given, to the patient?<br>*Question relevant when: selected( ${q104_M55_full_list} , '3')* | 1 Oral - solid (e.g. tablet)<br>2 Oral - liquid<br>3 Sublingual<br>4 Intravenous (IV)<br>5 Intramuscuklar injection (IM)<br>6 Subcutaneous<br>7 Rectal<br>8 Inhaled (e.g. gas)<br>9 Inserted (e.g. vaginal pessaries)<br>10 Topical - cream<br>11 Topical - drops (e.g. eye drops)<br>12 Other<br>99 Don't know |
| q404_M55c *(required)* | q404_M55c. What is the item's smallest unit of measurement (mg, mcg, ml, g, etc)?<br>*Question relevant when: selected( ${q104_M55_full_list} , '3')* | microgram — microgram (mcg)<br>miligram — miligram (mg)<br>miligram_dissolved_in_liquid — miligram (mg) dissolved in liquid (e.g. 2 mg per 3 militers)<br>gram — gram (g)<br>militer — militer (ml)<br>centiliter — centiliter (cl)<br>litre — litre (l)<br>ml_of _solution — ml of solution (e.g. ml of 10% solution)<br>litre_of_solution — litre of solution (e.g. 1 litre of 5% solution)<br>cubic_meter — cubic meter (m^3)<br>other — other<br>dont_know — don't know |
| q404_M55c.1 | q404_M55c.1 If other unit of measurement or route of administration, specify here:<br>*Leave blank if not applicable*<br>*Question relevant when: selected( ${q104_M55_full_list} , '3')* | |
| q404_M55d *(required)* | q404_M55d. In total, how many units are used to treat a patient during all of her care?<br>*Remember to add all units! E.g. 5 days x 200 mg twice per day = 2,000mg*<br>*Question relevant when: selected( ${q104_M55_full_list} , '3')*<br>*Response constrained to: .>0* | |
| q404_M56 | ***q404_M56. Atropine sulfate (or sulphate)***<br>[illegible] | |

Question relevant when: selected( ${q104_M56_full_list} , '3')

| Field | Question | Answer |
|---|---|---|
| q404_M56a *(required)* | q404_M56a. What percent of patients require this item?<br>*Question relevant when: selected( ${q104_M56_full_list} , '3')*<br>*Response constrained to: .>0 and .<=100 or .=999* | |
| q404_M56b *(required)* | q404_M56b. How is the item administered, or given, to the patient?<br>*Question relevant when: selected( ${q104_M56_full_list} , '3')* | 1 Oral - solid (e.g. tablet)<br>2 Oral - liquid<br>3 Sublingual<br>4 Intravenous (IV)<br>5 Intramusculuar injection (IM)<br>6 Subcutaneous<br>7 Rectal<br>8 Inhaled (e.g. gas)<br>9 Inserted (e.g. vaginal pessaries)<br>10 Topical - cream<br>11 Topical - drops (e.g. eye drops)<br>12 Other<br>99 Don't know |
| q404_M56c *(required)* | q404_M56c. What is the item's smallest unit of measurement (mg, mcg, ml, g, etc)?<br>*Question relevant when: selected( ${q104_M56_full_list} , '3')* | microgram: microgram (mcg)<br>miligram: miligram (mg)<br>miligram_dissolved_in_liquid: miligram (mg) dissolved in liquid (e.g. 2 mg per 3 militers)<br>gram: gram (g)<br>militer: militer (ml)<br>centiliter: centiliter (cl)<br>litre: litre (l)<br>ml_of _solution: ml of solution (e.g. ml of 10% solution)<br>litre_of_solution: litre of solution (e.g. 1 litre of 5% solution)<br>cubic_meter: cubic meter (m^3)<br>other: other<br>dont_know: don't know |
| q404_M56c.1 | q404_M56c.1 If other unit of measurement or route of administration, specify here:<br>*Leave blank if not applicable*<br>*Question relevant when: selected( ${q104_M56_full_list} , '3')* | |
| q404_M56d *(required)* | q404_M56d. In total, how many units are used to treat a patient during all of her care?<br>*Remember to add all units! E.g. 5 days x 200 mg twice per day = 2,000mg*<br>*Question relevant when: selected( ${q104_M56_full_list} , '3')*<br>*Response constrained to: .>0* | |
| q404_M57 | ***q404_M57. Papaverine HCl*** | |

*Question relevant when: selected( ${q104_M57_full_list} , '3')*

| Field | Question | Answer |
|---|---|---|
| q404_M57a *(required)* | q404_M57a. What percent of patients require this item?<br>*Question relevant when: selected( ${q104_M57_full_list} , '3')*<br>*Response constrained to: .>0 and .<=100 or .=999* | |
| q404_M57b *(required)* | q404_M57b. How is the item administered, or given, to the patient?<br>*Question relevant when: selected( ${q104_M57_full_list} , '3')* | 1 Oral - solid (e.g. tablet)<br>2 Oral - liquid<br>3 Sublingual<br>4 Intravenous (IV)<br>5 Intramusculuar injection (IM)<br>6 Subcutaneous<br>7 Rectal<br>8 Inhaled (e.g. gas)<br>9 Inserted (e.g. vaginal pessaries)<br>10 Topical - cream<br>11 Topical - drops (e.g. eye drops)<br>12 Other<br>99 Don't know |
| q404_M57c *(required)* | q404_M57c. What is the item's smallest unit of measurement (mg, mcg, ml, g, etc)?<br>*Question relevant when: selected( ${q104_M57_full_list} , '3')* | microgram — microgram (mcg)<br>miligram — miligram (mg)<br>miligram_dissolved_in_liquid — miligram (mg) dissolved in liquid (e.g. 2 mg per 3 militers)<br>gram — gram (g)<br>militer — militer (ml)<br>centiliter — centiliter (cl)<br>litre — litre (l)<br>ml_of _solution — ml of solution (e.g. ml of 10% solution)<br>litre_of_solution — litre of solution (e.g. 1 litre of 5% solution)<br>cubic_meter — cubic meter (m^3)<br>other — other<br>dont_know — don't know |
| q404_M57c.1 | q404_M57c.1 If other unit of measurement or route of administration, specify here:<br>*Leave blank if not applicable*<br>*Question relevant when: selected( ${q104_M57_full_list} , '3')* | |
| q404_M57d *(required)* | q404_M57d. In total, how many units are used to treat a patient during all of her care?<br>*Remember to add all units! E.g. 5 days x 200 mg twice per day = 2,000mg*<br>*Question relevant when: selected( ${q104_M57_full_list} , '3')*<br>*Response constrained to: .>0* | |
| q404_M58 | ***q404_M58. Diazepam (injection)*** | |

Question relevant when: selected( ${q104_M58_full_list} , '3')

| Field | Question | Answer |
|---|---|---|
| q404_M58a *(required)* | q404_M58a. What percent of patients require this item?<br>*Question relevant when: selected( ${q104_M58_full_list} , '3')*<br>*Response constrained to: .>0 and .<=100 or .=999* | |
| q404_M58b *(required)* | q404_M58b. How is the item administered, or given, to the patient?<br>*Question relevant when: selected( ${q104_M58_full_list} , '3')* | 1 Oral - solid (e.g. tablet)<br>2 Oral - liquid<br>3 Sublingual<br>4 Intravenous (IV)<br>5 Intramusculuar injection (IM)<br>6 Subcutaneous<br>7 Rectal<br>8 Inhaled (e.g. gas)<br>9 Inserted (e.g. vaginal pessaries)<br>10 Topical - cream<br>11 Topical - drops (e.g. eye drops)<br>12 Other<br>99 Don't know |
| q404_M58c *(required)* | q404_M58c. What is the item's smallest unit of measurement (mg, mcg, ml, g, etc)?<br>*Question relevant when: selected( ${q104_M58_full_list} , '3')* | microgram: microgram (mcg)<br>miligram: miligram (mg)<br>miligram_dissolved_in_liquid: miligram (mg) dissolved in liquid (e.g. 2 mg per 3 militers)<br>gram: gram (g)<br>militer: militer (ml)<br>centiliter: centiliter (cl)<br>litre: litre (l)<br>ml_of _solution: ml of solution (e.g. ml of 10% solution)<br>litre_of_solution: litre of solution (e.g. 1 litre of 5% solution)<br>cubic_meter: cubic meter (m^3)<br>other: other<br>dont_know: don't know |
| q404_M58c.1 | q404_M58c.1 If other unit of measurement or route of administration, specify here:<br>*Leave blank if not applicable*<br>*Question relevant when: selected( ${q104_M58_full_list} , '3')* | |
| q404_M58d *(required)* | q404_M58d. In total, how many units are used to treat a patient during all of her care?<br>*Remember to add all units! E.g. 5 days x 200 mg twice per day = 2,000mg*<br>*Question relevant when: selected( ${q104_M58_full_list} , '3')*<br>*Response constrained to: .>0* | |
| q404_M59 | ***q404_M59. Diazepam (oral)*** | |

Question relevant when: selected( ${q104_M59_full_list} , '3')

| Field | Question | Answer |
|---|---|---|
| q404_M59a *(required)* | q404_M59a. What percent of patients require this item?<br>*Question relevant when: selected( ${q104_M59_full_list} , '3')*<br>*Response constrained to: .>0 and .<=100 or .=999* | |
| q404_M59b *(required)* | q404_M59b. How is the item administered, or given, to the patient?<br>*Question relevant when: selected( ${q104_M59_full_list} , '3')* | 1 Oral - solid (e.g. tablet)<br>2 Oral - liquid<br>3 Sublingual<br>4 Intravenous (IV)<br>5 Intramusculuar injection (IM)<br>6 Subcutaneous<br>7 Rectal<br>8 Inhaled (e.g. gas)<br>9 Inserted (e.g. vaginal pessaries)<br>10 Topical - cream<br>11 Topical - drops (e.g. eye drops)<br>12 Other<br>99 Don't know |
| q404_M59c *(required)* | q404_M59c. What is the item's smallest unit of measurement (mg, mcg, ml, g, etc)?<br>*Question relevant when: selected( ${q104_M59_full_list} , '3')* | microgram — microgram (mcg)<br>miligram — miligram (mg)<br>miligram_dissolved_in_liquid — miligram (mg) dissolved in liquid (e.g. 2 mg per 3 militers)<br>gram — gram (g)<br>militer — militer (ml)<br>centiliter — centiliter (cl)<br>litre — litre (l)<br>ml_of _solution — ml of solution (e.g. ml of 10% solution)<br>litre_of_solution — litre of solution (e.g. 1 litre of 5% solution)<br>cubic_meter — cubic meter (m^3)<br>other — other<br>dont_know — don't know |
| q404_M59c.1 | q404_M59c.1 If other unit of measurement or route of administration, specify here:<br>*Leave blank if not applicable*<br>*Question relevant when: selected( ${q104_M59_full_list} , '3')* | |
| q404_M59d *(required)* | q404_M59d. In total, how many units are used to treat a patient during all of her care?<br>*Remember to add all units! E.g. 5 days x 200 mg twice per day = 2,000mg*<br>*Question relevant when: selected( ${q104_M59_full_list} , '3')*<br>*Response constrained to: .>0* | |
| q404_M60 | ***q404_M60. Antispasmodic, anxiolytic, sedative, etc - Other 1: "[q104_M60_full_list_other]"*** | |

*Question relevant when: selected( ${q104_M60_full_list} , '3')*

| Field | Question | Answer |
|---|---|---|
| q404_M60a *(required)* | q404_M60a. What percent of patients require this item?<br>*Question relevant when: selected( ${q104_M60_full_list} , '3')*<br>*Response constrained to: .>0 and .<=100 or .=999* | |
| q404_M60b *(required)* | q404_M60b. How is the item administered, or given, to the patient?<br>*Question relevant when: selected( ${q104_M60_full_list} , '3')* | 1 Oral - solid (e.g. tablet)<br>2 Oral - liquid<br>3 Sublingual<br>4 Intravenous (IV)<br>5 Intramusculuar injection (IM)<br>6 Subcutaneous<br>7 Rectal<br>8 Inhaled (e.g. gas)<br>9 Inserted (e.g. vaginal pessaries)<br>10 Topical - cream<br>11 Topical - drops (e.g. eye drops)<br>12 Other<br>99 Don't know |
| q404_M60c *(required)* | q404_M60c. What is the item's smallest unit of measurement (mg, mcg, ml, g, etc)?<br>*Question relevant when: selected( ${q104_M60_full_list} , '3')* | microgram — microgram (mcg)<br>miligram — miligram (mg)<br>miligram_dissolved_in_liquid — miligram (mg) dissolved in liquid (e.g. 2 mg per 3 militers)<br>gram — gram (g)<br>militer — militer (ml)<br>centiliter — centiliter (cl)<br>litre — litre (l)<br>ml_of _solution — ml of solution (e.g. ml of 10% solution)<br>litre_of_solution — litre of solution (e.g. 1 litre of 5% solution)<br>cubic_meter — cubic meter (m^3)<br>other — other<br>dont_know — don't know |
| q404_M60c.1 | q404_M60c.1 If other unit of measurement or route of administration, specify here:<br>*Leave blank if not applicable*<br>*Question relevant when: selected( ${q104_M60_full_list} , '3')* | |
| q404_M60d *(required)* | q404_M60d. In total, how many units are used to treat a patient during all of her care?<br>*Remember to add all units! E.g. 5 days x 200 mg twice per day = 2,000mg*<br>*Question relevant when: selected( ${q104_M60_full_list} , '3')*<br>*Response constrained to: .>0* | |
| q404_M61 | ***q404_M61. Antispasmodic, anxiolytic, sedative, etc - Other 2: "[q104_M61_full_list_other]"***<br>[illegible] | |

Question relevant when: selected( ${q104_M61_full_list} , '3')

| Field | Question | Answer |
|---|---|---|
| q404_M61a *(required)* | q404_M61a. What percent of patients require this item?<br>*Question relevant when: selected( ${q104_M61_full_list} , '3')*<br>*Response constrained to: .>0 and .<=100 or .=999* | |

| Field | Question | Answer |
|---|---|---|
| q404_M61b *(required)* | q404_M61b. How is the item administered, or given, to the patient?<br>*Question relevant when: selected( ${q104_M61_full_list} , '3')* | 1 Oral - solid (e.g. tablet)<br>2 Oral - liquid<br>3 Sublingual<br>4 Intravenous (IV)<br>5 Intramusculuar injection (IM)<br>6 Subcutaneous<br>7 Rectal<br>8 Inhaled (e.g. gas)<br>9 Inserted (e.g. vaginal pessaries)<br>10 Topical - cream<br>11 Topical - drops (e.g. eye drops)<br>12 Other<br>99 Don't know |
| q404_M61c *(required)* | q404_M61c. What is the item's smallest unit of measurement (mg, mcg, ml, g, etc)?<br>*Question relevant when: selected( ${q104_M61_full_list} , '3')* | microgram — microgram (mcg)<br>miligram — miligram (mg)<br>miligram_dissolved_in_liquid — miligram (mg) dissolved in liquid (e.g. 2 mg per 3 militers)<br>gram — gram (g)<br>militer — militer (ml)<br>centiliter — centiliter (cl)<br>litre — litre (l)<br>ml_of _solution — ml of solution (e.g. ml of 10% solution)<br>litre_of_solution — litre of solution (e.g. 1 litre of 5% solution)<br>cubic_meter — cubic meter (m^3)<br>other — other<br>dont_know — don't know |
| q404_M61c.1 | q404_M61c.1 If other unit of measurement or route of administration, specify here:<br>*Leave blank if not applicable*<br>*Question relevant when: selected( ${q104_M61_full_list} , '3')* | |
| q404_M61d *(required)* | q404_M61d. In total, how many units are used to treat a patient during all of her care?<br>*Remember to add all units! E.g. 5 days x 200 mg twice per day = 2,000mg*<br>*Question relevant when: selected( ${q104_M61_full_list} , '3')*<br>*Response constrained to: .>0* | |
| q404_M62 | ***q404_M62. Antispasmodic, anxiolytic, sedative, etc - Other 3: "[q104_M62_full_list_other]"*** | |

*Question relevant when: selected( ${q104_M62_full_list} , '3')*

| Field | Question | Answer |
|---|---|---|
| q404_M62a *(required)* | q404_M62a. What percent of patients require this item?<br>*Question relevant when: selected( ${q104_M62_full_list} , '3')*<br>*Response constrained to: .>0 and .<=100 or .=999* | |

| Field | Question | Answer |
|---|---|---|
| q404_M62b *(required)* | q404_M62b. How is the item administered, or given, to the patient?<br>*Question relevant when: selected( ${q104_M62_full_list} , '3')* | 1 Oral - solid (e.g. tablet)<br>2 Oral - liquid<br>3 Sublingual<br>4 Intravenous (IV)<br>5 Intramusculuar injection (IM)<br>6 Subcutaneous<br>7 Rectal<br>8 Inhaled (e.g. gas)<br>9 Inserted (e.g. vaginal pessaries)<br>10 Topical - cream<br>11 Topical - drops (e.g. eye drops)<br>12 Other<br>99 Don't know |
| q404_M62c *(required)* | q404_M62c. What is the item's smallest unit of measurement (mg, mcg, ml, g, etc)?<br>*Question relevant when: selected( ${q104_M62_full_list} , '3')* | microgram — microgram (mcg)<br>miligram — miligram (mg)<br>miligram_dissolved_in_liquid — miligram (mg) dissolved in liquid (e.g. 2 mg per 3 militers)<br>gram — gram (g)<br>militer — militer (ml)<br>centiliter — centiliter (cl)<br>litre — litre (l)<br>ml_of _solution — ml of solution (e.g. ml of 10% solution)<br>litre_of_solution — litre of solution (e.g. 1 litre of 5% solution)<br>cubic_meter — cubic meter (m^3)<br>other — other<br>dont_know — don't know |
| q404_M62c.1 | q404_M62c.1 If other unit of measurement or route of administration, specify here:<br>*Leave blank if not applicable*<br>*Question relevant when: selected( ${q104_M62_full_list} , '3')* | |
| q404_M62d *(required)* | q404_M62d. In total, how many units are used to treat a patient during all of her care?<br>*Remember to add all units! E.g. 5 days x 200 mg twice per day = 2,000mg*<br>*Question relevant when: selected( ${q104_M62_full_list} , '3')*<br>*Response constrained to: .>0* | |
| q404_M63 | ***q404_M63. Antispasmodic, anxiolytic, sedative, etc - Other 4: "[q104_M63_full_list_other]"*** | |

*Question relevant when: selected( ${q104_M63_full_list} , '3')*

| Field | Question | Answer |
|---|---|---|
| q404_M63a *(required)* | q404_M63a. What percent of patients require this item?<br>*Question relevant when: selected( ${q104_M63_full_list} , '3')*<br>*Response constrained to: .>0 and .<=100 or .=999* | |
| q404_M63b *(required)* | q404_M63b. How is the item administered, or given, to the patient?<br>*Question relevant when: selected( ${q104_M63_full_list} , '3')* | 1 Oral - solid (e.g. tablet)<br>2 Oral - liquid<br>3 Sublingual<br>4 Intravenous (IV)<br>5 Intramusculuar injection (IM)<br>6 Subcutaneous<br>7 Rectal<br>8 Inhaled (e.g. gas)<br>9 Inserted (e.g. vaginal pessaries)<br>10 Topical - cream<br>11 Topical - drops (e.g. eye drops)<br>12 Other<br>99 Don't know |
| q404_M63c *(required)* | q404_M63c. What is the item's smallest unit of measurement (mg, mcg, ml, g, etc)?<br>*Question relevant when: selected( ${q104_M63_full_list} , '3')* | microgram — microgram (mcg)<br>miligram — miligram (mg)<br>miligram_dissolved_in_liquid — miligram (mg) dissolved in liquid (e.g. 2 mg per 3 militers)<br>gram — gram (g)<br>militer — militer (ml)<br>centiliter — centiliter (cl)<br>litre — litre (l)<br>ml_of _solution — ml of solution (e.g. ml of 10% solution)<br>litre_of_solution — litre of solution (e.g. 1 litre of 5% solution)<br>cubic_meter — cubic meter (m^3)<br>other — other<br>dont_know — don't know |
| q404_M63c.1 | q404_M63c.1 If other unit of measurement or route of administration, specify here:<br>*Leave blank if not applicable*<br>*Question relevant when: selected( ${q104_M63_full_list} , '3')* | |
| q404_M63d *(required)* | q404_M63d. In total, how many units are used to treat a patient during all of her care?<br>*Remember to add all units! E.g. 5 days x 200 mg twice per day = 2,000mg*<br>*Question relevant when: selected( ${q104_M63_full_list} , '3')*<br>*Response constrained to: .>0* | |
| q404_M64 | ***q404_M64. Antispasmodic, anxiolytic, sedative, etc - Other 5: "[q104_M64_full_list_other]"*** | |

*Question relevant when: selected( ${q104_M64_full_list} , '3')*

| Field | Question | Answer |
|---|---|---|
| q404_M64a *(required)* | q404_M64a. What percent of patients require this item?<br>*Question relevant when: selected( ${q104_M64_full_list} , '3')*<br>*Response constrained to: .>0 and .<=100 or .=999* | |
| q404_M64b *(required)* | q404_M64b. How is the item administered, or given, to the patient?<br>*Question relevant when: selected( ${q104_M64_full_list} , '3')* | 1 Oral - solid (e.g. tablet)<br>2 Oral - liquid<br>3 Sublingual<br>4 Intravenous (IV)<br>5 Intramusculuar injection (IM)<br>6 Subcutaneous<br>7 Rectal<br>8 Inhaled (e.g. gas)<br>9 Inserted (e.g. vaginal pessaries)<br>10 Topical - cream<br>11 Topical - drops (e.g. eye drops)<br>12 Other<br>99 Don't know |
| q404_M64c *(required)* | q404_M64c. What is the item's smallest unit of measurement (mg, mcg, ml, g, etc)?<br>*Question relevant when: selected( ${q104_M64_full_list} , '3')* | microgram — microgram (mcg)<br>miligram — miligram (mg)<br>miligram_dissolved_in_liquid — miligram (mg) dissolved in liquid (e.g. 2 mg per 3 militers)<br>gram — gram (g)<br>militer — militer (ml)<br>centiliter — centiliter (cl)<br>litre — litre (l)<br>ml_of _solution — ml of solution (e.g. ml of 10% solution)<br>litre_of_solution — litre of solution (e.g. 1 litre of 5% solution)<br>cubic_meter — cubic meter (m^3)<br>other — other<br>dont_know — don't know |
| q404_M64c.1 | q404_M64c.1 If other unit of measurement or route of administration, specify here:<br>*Leave blank if not applicable*<br>*Question relevant when: selected( ${q104_M64_full_list} , '3')* | |
| q404_M64d *(required)* | q404_M64d. In total, how many units are used to treat a patient during all of her care?<br>*Remember to add all units! E.g. 5 days x 200 mg twice per day = 2,000mg*<br>*Question relevant when: selected( ${q104_M64_full_list} , '3')*<br>*Response constrained to: .>0* | |

<p><strong><em>D. Medications - Shock (6)</em></strong></p>

[illegible]

Group relevant when: selected( ${section_four_skip_med} , '1')

| Field | Question | Answer |
|---|---|---|
| q404_med_notE5 | 404 CONTINUED For each of the following medications and other medical products that are used for shock, can you tell me …? | |
| note_404_adrenergic | ***Adrenergic*** | |
| q404_M65 | ***q404_M65. Ephedrine HCl***<br>*Question relevant when: selected( ${q104_M65_full_list} , '3')* | |
| q404_M65a *(required)* | q404_M65a. What percent of patients require this item?<br>*Question relevant when: selected( ${q104_M65_full_list} , '3')*<br>*Response constrained to: .>0 and .<=100 or .=999* | |

| Field | Question | Answer |
|---|---|---|
| q404_M65b *(required)* | q404_M65b. How is the item administered, or given, to the patient?<br>*Question relevant when: selected( ${q104_M65_full_list} , '3')* | 1 Oral - solid (e.g. tablet)<br>2 Oral - liquid<br>3 Sublingual<br>4 Intravenous (IV)<br>5 Intramusculuar injection (IM)<br>6 Subcutaneous<br>7 Rectal<br>8 Inhaled (e.g. gas)<br>9 Inserted (e.g. vaginal pessaries)<br>10 Topical - cream<br>11 Topical - drops (e.g. eye drops)<br>12 Other<br>99 Don't know |
| q404_M65c *(required)* | q404_M65c. What is the item's smallest unit of measurement (mg, mcg, ml, g, etc)?<br>*Question relevant when: selected( ${q104_M65_full_list} , '3')* | microgram — microgram (mcg)<br>miligram — miligram (mg)<br>miligram_dissolved_in_liquid — miligram (mg) dissolved in liquid (e.g. 2 mg per 3 militers)<br>gram — gram (g)<br>militer — militer (ml)<br>centiliter — centiliter (cl)<br>litre — litre (l)<br>ml_of _solution — ml of solution (e.g. ml of 10% solution)<br>litre_of_solution — litre of solution (e.g. 1 litre of 5% solution)<br>cubic_meter — cubic meter (m^3)<br>other — other<br>dont_know — don't know |
| q404_M65c.1 | q404_M65c.1 If other unit of measurement or route of administration, specify here:<br>*Leave blank if not applicable*<br>*Question relevant when: selected( ${q104_M65_full_list} , '3')* | |
| q404_M65d *(required)* | q404_M65d. In total, how many units are used to treat a patient during all of her care?<br>*Remember to add all units! E.g. 5 days x 200 mg twice per day = 2,000mg*<br>*Question relevant when: selected( ${q104_M65_full_list} , '3')*<br>*Response constrained to: .>0* | |
| q404_M66 | ***q404_M66. Epinephrine***<br>[illegible] | |

*Question relevant when: selected( ${q104_M66_full_list} , '3')*

| Field | Question | Answer |
|---|---|---|
| q404_M66a *(required)* | q404_M66a. What percent of patients require this item?<br>*Question relevant when: selected( ${q104_M66_full_list} , '3')*<br>*Response constrained to: .>0 and .<=100 or .=999* | |

| | | |
|---|---|---|
| q404_M66b *(required)* | q404_M66b. How is the item administered, or given, to the patient?<br>*Question relevant when: selected( ${q104_M66_full_list} , '3')* | 1 Oral - solid (e.g. tablet)<br>2 Oral - liquid<br>3 Sublingual<br>4 Intravenous (IV)<br>5 Intramusculuar injection (IM)<br>6 Subcutaneous<br>7 Rectal<br>8 Inhaled (e.g. gas)<br>9 Inserted (e.g. vaginal pessaries)<br>10 Topical - cream<br>11 Topical - drops (e.g. eye drops)<br>12 Other<br>99 Don't know |
| q404_M66c *(required)* | q404_M66c. What is the item's smallest unit of measurement (mg, mcg, ml, g, etc)?<br>*Question relevant when: selected( ${q104_M66_full_list} , '3')* | microgram — microgram (mcg)<br>miligram — miligram (mg)<br>miligram_dissolved_in_liquid — miligram (mg) dissolved in liquid (e.g. 2 mg per 3 militers)<br>gram — gram (g)<br>militer — militer (ml)<br>centiliter — centiliter (cl)<br>litre — litre (l)<br>ml_of _solution — ml of solution (e.g. ml of 10% solution)<br>litre_of_solution — litre of solution (e.g. 1 litre of 5% solution)<br>cubic_meter — cubic meter (m^3)<br>other — other<br>dont_know — don't know |
| q404_M66c.1 | q404_M66c.1 If other unit of measurement or route of administration, specify here:<br>*Leave blank if not applicable*<br>*Question relevant when: selected( ${q104_M66_full_list} , '3')* | |
| q404_M66d *(required)* | q404_M66d. In total, how many units are used to treat a patient during all of her care?<br>*Remember to add all units! E.g. 5 days x 200 mg twice per day = 2,000mg*<br>*Question relevant when: selected( ${q104_M66_full_list} , '3')*<br>*Response constrained to: .>0* | |
| q404_M67 | ***q404_M67. Adrenergic - other 1: "[q104_M67_full_list_other]"*** | |

*Question relevant when: selected( ${q104_M67_full_list} , '3')*

| Field | Question | Answer |
|---|---|---|
| q404_M67a *(required)* | q404_M67a. What percent of patients require this item?<br>*Question relevant when: selected( ${q104_M67_full_list} , '3')*<br>*Response constrained to: .>0 and .<=100 or .=999* | |
| q404_M67b *(required)* | q404_M67b. How is the item administered, or given, to the patient?<br>*Question relevant when: selected( ${q104_M67_full_list} , '3')* | 1 Oral - solid (e.g. tablet)<br>2 Oral - liquid<br>3 Sublingual<br>4 Intravenous (IV)<br>5 Intramusculuar injection (IM)<br>6 Subcutaneous<br>7 Rectal<br>8 Inhaled (e.g. gas)<br>9 Inserted (e.g. vaginal pessaries)<br>10 Topical - cream<br>11 Topical - drops (e.g. eye drops)<br>12 Other<br>99 Don't know |
| q404_M67c *(required)* | q404_M67c. What is the item's smallest unit of measurement (mg, mcg, ml, g, etc)?<br>*Question relevant when: selected( ${q104_M67_full_list} , '3')* | microgram — microgram (mcg)<br>miligram — miligram (mg)<br>miligram_dissolved_in_liquid — miligram (mg) dissolved in liquid (e.g. 2 mg per 3 militers)<br>gram — gram (g)<br>militer — militer (ml)<br>centiliter — centiliter (cl)<br>litre — litre (l)<br>ml_of _solution — ml of solution (e.g. ml of 10% solution)<br>litre_of_solution — litre of solution (e.g. 1 litre of 5% solution)<br>cubic_meter — cubic meter (m^3)<br>other — other<br>dont_know — don't know |
| q404_M67c.1 | q404_M67c.1 If other unit of measurement or route of administration, specify here:<br>*Leave blank if not applicable*<br>*Question relevant when: selected( ${q104_M67_full_list} , '3')* | |
| q404_M67d *(required)* | q404_M67d. In total, how many units are used to treat a patient during all of her care?<br>*Remember to add all units! E.g. 5 days x 200 mg twice per day = 2,000mg*<br>*Question relevant when: selected( ${q104_M67_full_list} , '3')*<br>*Response constrained to: .>0* | |
| q404_M68 | ***q404_M68. Adrenergic - other 2: "[q104_M68_full_list_other]"*** | |

Question relevant when: selected( ${q104_M68_full_list} , '3')

| Field | Question | Answer |
|---|---|---|
| q404_M68a *(required)* | q404_M68a. What percent of patients require this item?<br>*Question relevant when: selected( ${q104_M68_full_list} , '3')*<br>*Response constrained to: .>0 and .<=100 or .=999* | |
| q404_M68b *(required)* | q404_M68b. How is the item administered, or given, to the patient?<br>*Question relevant when: selected( ${q104_M68_full_list} , '3')* | 1 Oral - solid (e.g. tablet)<br>2 Oral - liquid<br>3 Sublingual<br>4 Intravenous (IV)<br>5 Intramusculuar injection (IM)<br>6 Subcutaneous<br>7 Rectal<br>8 Inhaled (e.g. gas)<br>9 Inserted (e.g. vaginal pessaries)<br>10 Topical - cream<br>11 Topical - drops (e.g. eye drops)<br>12 Other<br>99 Don't know |
| q404_M68c *(required)* | q404_M68c. What is the item's smallest unit of measurement (mg, mcg, ml, g, etc)?<br>*Question relevant when: selected( ${q104_M68_full_list} , '3')* | microgram: microgram (mcg)<br>miligram: miligram (mg)<br>miligram_dissolved_in_liquid: miligram (mg) dissolved in liquid (e.g. 2 mg per 3 militers)<br>gram: gram (g)<br>militer: militer (ml)<br>centiliter: centiliter (cl)<br>litre: litre (l)<br>ml_of _solution: ml of solution (e.g. ml of 10% solution)<br>litre_of_solution: litre of solution (e.g. 1 litre of 5% solution)<br>cubic_meter: cubic meter (m^3)<br>other: other<br>dont_know: don't know |
| q404_M68c.1 | q404_M68c.1 If other unit of measurement or route of administration, specify here:<br>*Leave blank if not applicable*<br>*Question relevant when: selected( ${q104_M68_full_list} , '3')* | |
| q404_M68d *(required)* | q404_M68d. In total, how many units are used to treat a patient during all of her care?<br>*Remember to add all units! E.g. 5 days x 200 mg twice per day = 2,000mg*<br>*Question relevant when: selected( ${q104_M68_full_list} , '3')*<br>*Response constrained to: .>0* | |
| q404_M69 | ***q404_M69. Adrenergic - other 3: "[q104_M69_full_list_other]"*** | |

Question relevant when: selected( ${q104_M69_full_list} , '3')

| Field | Question | Answer |
|---|---|---|
| q404_M69a *(required)* | q404_M69a. What percent of patients require this item?<br>*Question relevant when: selected( ${q104_M69_full_list} , '3')*<br>*Response constrained to: .>0 and .<=100 or .=999* | |

| Field | Question | Answer |
|---|---|---|
| q404_M69b *(required)* | q404_M69b. How is the item administered, or given, to the patient?<br>*Question relevant when: selected( ${q104_M69_full_list} , '3')* | 1 Oral - solid (e.g. tablet)<br>2 Oral - liquid<br>3 Sublingual<br>4 Intravenous (IV)<br>5 Intramusculuar injection (IM)<br>6 Subcutaneous<br>7 Rectal<br>8 Inhaled (e.g. gas)<br>9 Inserted (e.g. vaginal pessaries)<br>10 Topical - cream<br>11 Topical - drops (e.g. eye drops)<br>12 Other<br>99 Don't know |
| q404_M69c *(required)* | q404_M69c. What is the item's smallest unit of measurement (mg, mcg, ml, g, etc)?<br>*Question relevant when: selected( ${q104_M69_full_list} , '3')* | microgram — microgram (mcg)<br>miligram — miligram (mg)<br>miligram_dissolved_in_liquid — miligram (mg) dissolved in liquid (e.g. 2 mg per 3 militers)<br>gram — gram (g)<br>militer — militer (ml)<br>centiliter — centiliter (cl)<br>litre — litre (l)<br>ml_of _solution — ml of solution (e.g. ml of 10% solution)<br>litre_of_solution — litre of solution (e.g. 1 litre of 5% solution)<br>cubic_meter — cubic meter (m^3)<br>other — other<br>dont_know — don't know |
| q404_M69c.1 | q404_M69c.1 If other unit of measurement or route of administration, specify here:<br>*Leave blank if not applicable*<br>*Question relevant when: selected( ${q104_M69_full_list} , '3')* | |
| q404_M69d *(required)* | q404_M69d. In total, how many units are used to treat a patient during all of her care?<br>*Remember to add all units! E.g. 5 days x 200 mg twice per day = 2,000mg*<br>*Question relevant when: selected( ${q104_M69_full_list} , '3')*<br>*Response constrained to: .>0* | |
| q404_M69.1 | ***q404_M69.1. Adrenergic - other 4: "[q104_M69.1_full_list_other]"*** | |

Question relevant when: selected( ${q104_M69.1_full_list} , '2')

| Field | Question | Answer |
|---|---|---|
| q404_M69.1a *(required)* | q404_M69.1a. What percent of patients require this item?<br>*Question relevant when: selected( ${q104_M69.1_full_list} , '2')*<br>*Response constrained to: .>0 and .<=100 or .=999* | |

| Field | Question | Answer | |
|---|---|---|---|
| q404_M69.1b *(required)* | q404_M69.1b. How is the item administered, or given, to the patient?<br>*Question relevant when: selected( ${q104_M69.1_full_list} , '2')* | 1 | Oral - solid (e.g. tablet) |
| | | 2 | Oral - liquid |
| | | 3 | Sublingual |
| | | 4 | Intravenous (IV) |
| | | 5 | Intramusculuar injection (IM) |
| | | 6 | Subcutaneous |
| | | 7 | Rectal |
| | | 8 | Inhaled (e.g. gas) |
| | | 9 | Inserted (e.g. vaginal pessaries) |
| | | 10 | Topical - cream |
| | | 11 | Topical - drops (e.g. eye drops) |
| | | 12 | Other |
| | | 99 | Don't know |
| q404_M69.1c *(required)* | q404_M69.1c. What is the item's smallest unit of measurement (mg, mcg, ml, g, etc)?<br>*Question relevant when: selected( ${q104_M69.1_full_list} , '2')* | microgram | microgram (mcg) |
| | | miligram | miligram (mg) |
| | | miligram_dissolved_in_liquid | miligram (mg) dissolved in liquid (e.g. 2 mg per 3 militers) |
| | | gram | gram (g) |
| | | militer | militer (ml) |
| | | centiliter | centiliter (cl) |
| | | litre | litre (l) |
| | | ml_of _solution | ml of solution (e.g. ml of 10% solution) |
| | | litre_of_solution | litre of solution (e.g. 1 litre of 5% solution) |
| | | cubic_meter | cubic meter (m^3) |
| | | other | other |
| | | dont_know | don't know |
| q404_M69.1c.1 | q404_M69.1c.1 If other unit of measurement or route of administration, specify here:<br>*Leave blank if not applicable*<br>*Question relevant when: selected( ${q104_M69.1_full_list} , '2')* | | |
| q404_M69.1d *(required)* | q404_M69.1d. In total, how many units are used to treat a patient during all of her care?<br>*Remember to add all units! E.g. 5 days x 200 mg twice per day = 2,000mg*<br>*Question relevant when: selected( ${q104_M69.1_full_list} , '2')*<br>*Response constrained to: .>0* | | |
| q404_M69.2 | ***q404_M69.2. Adrenergic - other 5: "[q104_M69.2_full_list_other]"*** | | |

Question relevant when: selected( ${q104_M69.2_full_list} , '2')

| Field | Question | Answer |
|---|---|---|
| q404_M69.2a *(required)* | q404_M69.2a. What percent of patients require this item?<br>*Question relevant when: selected( ${q104_M69.2_full_list} , '2')*<br>*Response constrained to: .>0 and .<=100 or .=999* | |

| | | |
|---|---|---|
| q404_M69.2b *(required)* | q404_M69.2b. How is the item administered, or given, to the patient?<br>*Question relevant when: selected( ${q104_M69.2_full_list} , '2')* | 1 Oral - solid (e.g. tablet)<br>2 Oral - liquid<br>3 Sublingual<br>4 Intravenous (IV)<br>5 Intramusculuar injection (IM)<br>6 Subcutaneous<br>7 Rectal<br>8 Inhaled (e.g. gas)<br>9 Inserted (e.g. vaginal pessaries)<br>10 Topical - cream<br>11 Topical - drops (e.g. eye drops)<br>12 Other<br>99 Don't know |
| q404_M69.2c *(required)* | q404_M69.2c. What is the item's smallest unit of measurement (mg, mcg, ml, g, etc)?<br>*Question relevant when: selected( ${q104_M69.2_full_list} , '2')* | microgram — microgram (mcg)<br>miligram — miligram (mg)<br>miligram_dissolved_in_liquid — miligram (mg) dissolved in liquid (e.g. 2 mg per 3 militers)<br>gram — gram (g)<br>militer — militer (ml)<br>centiliter — centiliter (cl)<br>litre — litre (l)<br>ml_of _solution — ml of solution (e.g. ml of 10% solution)<br>litre_of_solution — litre of solution (e.g. 1 litre of 5% solution)<br>cubic_meter — cubic meter (m^3)<br>other — other<br>dont_know — don't know |
| q404_M69.2c.1 | q404_M69.2c.1 If other unit of measurement or route of administration, specify here:<br>*Leave blank if not applicable*<br>*Question relevant when: selected( ${q104_M69.2_full_list} , '2')* | |
| q404_M69.2d *(required)* | q404_M69.2d. In total, how many units are used to treat a patient during all of her care?<br>*Remember to add all units! E.g. 5 days x 200 mg twice per day = 2,000mg*<br>*Question relevant when: selected( ${q104_M69.2_full_list} , '2')*<br>*Response constrained to: .>0* | |

<p><strong><em>D. Medications - Shock (7)</em></strong></p>

*Group relevant when: selected( ${section_four_skip_med} , '1')*

| Field | Question | Answer |
|---|---|---|
| q404_med_notE6 | 404 CONTINUED For each of the following medications and other medical products that are used for shock, can you tell me …? | |
| note_404_blood | ***Blood products*** | |
| q404_M70 | ***q404_M70. Unit of packed red blood cells***<br>*Question relevant when: selected( ${q104_M70_full_list} , '3')* | |
| q404_M70a *(required)* | q404_M70a. What percent of patients require this item?<br>*Question relevant when: selected( ${q104_M70_full_list} , '3')*<br>*Response constrained to: .>0 and .<=100 or .=999* | |

| Field | Question | Answer |
|---|---|---|
| q404_M70b *(required)* | q404_M70b. How is the item administered, or given, to the patient?<br>*Question relevant when: selected( ${q104_M70_full_list} , '3')* | 1 Oral - solid (e.g. tablet)<br>2 Oral - liquid<br>3 Sublingual<br>4 Intravenous (IV)<br>5 Intramusculuar injection (IM)<br>6 Subcutaneous<br>7 Rectal<br>8 Inhaled (e.g. gas)<br>9 Inserted (e.g. vaginal pessaries)<br>10 Topical - cream<br>11 Topical - drops (e.g. eye drops)<br>12 Other<br>99 Don't know |
| q404_M70c *(required)* | q404_M70c. What is the item's smallest unit of measurement (mg, mcg, ml, g, etc)?<br>*Question relevant when: selected( ${q104_M70_full_list} , '3')* | microgram — microgram (mcg)<br>miligram — miligram (mg)<br>miligram_dissolved_in_liquid — miligram (mg) dissolved in liquid (e.g. 2 mg per 3 militers)<br>gram — gram (g)<br>militer — militer (ml)<br>centiliter — centiliter (cl)<br>litre — litre (l)<br>ml_of _solution — ml of solution (e.g. ml of 10% solution)<br>litre_of_solution — litre of solution (e.g. 1 litre of 5% solution)<br>cubic_meter — cubic meter (m^3)<br>other — other<br>dont_know — don't know |
| q404_M70c.1 | q404_M70c.1 If other unit of measurement or route of administration, specify here:<br>*Leave blank if not applicable*<br>*Question relevant when: selected( ${q104_M70_full_list} , '3')* | |
| q404_M70d *(required)* | q404_M70d. In total, how many units are used to treat a patient during all of her care?<br>*Remember to add all units! E.g. 5 days x 200 mg twice per day = 2,000mg*<br>*Question relevant when: selected( ${q104_M70_full_list} , '3')*<br>*Response constrained to: .>0* | |
| q404_M71 | ***q404_M71. Unit of platelets***<br>[illegible] | |

| Field | Question | Answer |
|---|---|---|
| | *Question relevant when: selected( ${q104_M71_full_list} , '3')* | |
| q404_M71a *(required)* | q404_M71a. What percent of patients require this item?<br>*Question relevant when: selected( ${q104_M71_full_list} , '3')*<br>*Response constrained to: .>0 and .<=100 or .=999* | |
| q404_M71b *(required)* | q404_M71b. How is the item administered, or given, to the patient?<br>*Question relevant when: selected( ${q104_M71_full_list} , '3')* | 1 Oral - solid (e.g. tablet)<br>2 Oral - liquid<br>3 Sublingual<br>4 Intravenous (IV)<br>5 Intramusculuar injection (IM)<br>6 Subcutaneous<br>7 Rectal<br>8 Inhaled (e.g. gas)<br>9 Inserted (e.g. vaginal pessaries)<br>10 Topical - cream<br>11 Topical - drops (e.g. eye drops)<br>12 Other<br>99 Don't know |
| q404_M71c *(required)* | q404_M71c. What is the item's smallest unit of measurement (mg, mcg, ml, g, etc)?<br>*Question relevant when: selected( ${q104_M71_full_list} , '3')* | microgram — microgram (mcg)<br>miligram — miligram (mg)<br>miligram_dissolved_in_liquid — miligram (mg) dissolved in liquid (e.g. 2 mg per 3 militers)<br>gram — gram (g)<br>militer — militer (ml)<br>centiliter — centiliter (cl)<br>litre — litre (l)<br>ml_of _solution — ml of solution (e.g. ml of 10% solution)<br>litre_of_solution — litre of solution (e.g. 1 litre of 5% solution)<br>cubic_meter — cubic meter (m^3)<br>other — other<br>dont_know — don't know |
| q404_M71c.1 | q404_M71c.1 If other unit of measurement or route of administration, specify here:<br>*Leave blank if not applicable*<br>*Question relevant when: selected( ${q104_M71_full_list} , '3')* | |
| q404_M71d *(required)* | q404_M71d. In total, how many units are used to treat a patient during all of her care?<br>*Remember to add all units! E.g. 5 days x 200 mg twice per day = 2,000mg*<br>*Question relevant when: selected( ${q104_M71_full_list} , '3')*<br>*Response constrained to: .>0* | |
| q404_M72 | ***q404_M72. Unit of whole blood***<br>[illegible] | |

*Question relevant when: selected( ${q104_M72_full_list} , '3')*

| Field | Question | Answer |
|---|---|---|
| q404_M72a *(required)* | q404_M72a. What percent of patients require this item?<br>*Question relevant when: selected( ${q104_M72_full_list} , '3')*<br>*Response constrained to: .>0 and .<=100 or .=999* | |
| q404_M72b *(required)* | q404_M72b. How is the item administered, or given, to the patient?<br>*Question relevant when: selected( ${q104_M72_full_list} , '3')* | 1 Oral - solid (e.g. tablet)<br>2 Oral - liquid<br>3 Sublingual<br>4 Intravenous (IV)<br>5 Intramusculuar injection (IM)<br>6 Subcutaneous<br>7 Rectal<br>8 Inhaled (e.g. gas)<br>9 Inserted (e.g. vaginal pessaries)<br>10 Topical - cream<br>11 Topical - drops (e.g. eye drops)<br>12 Other<br>99 Don't know |
| q404_M72c *(required)* | q404_M72c. What is the item's smallest unit of measurement (mg, mcg, ml, g, etc)?<br>*Question relevant when: selected( ${q104_M72_full_list} , '3')* | microgram — microgram (mcg)<br>miligram — miligram (mg)<br>miligram_dissolved_in_liquid — miligram (mg) dissolved in liquid (e.g. 2 mg per 3 militers)<br>gram — gram (g)<br>militer — militer (ml)<br>centiliter — centiliter (cl)<br>litre — litre (l)<br>ml_of _solution — ml of solution (e.g. ml of 10% solution)<br>litre_of_solution — litre of solution (e.g. 1 litre of 5% solution)<br>cubic_meter — cubic meter (m^3)<br>other — other<br>dont_know — don't know |
| q404_M72c.1 | q404_M72c.1 If other unit of measurement or route of administration, specify here:<br>*Leave blank if not applicable*<br>*Question relevant when: selected( ${q104_M72_full_list} , '3')* | |
| q404_M72d *(required)* | q404_M72d. In total, how many units are used to treat a patient during all of her care?<br>*Remember to add all units! E.g. 5 days x 200 mg twice per day = 2,000mg*<br>*Question relevant when: selected( ${q104_M72_full_list} , '3')*<br>*Response constrained to: .>0* | |
| q404_M73 | ***q404_M73. Blood product - other 1: "[q104_M73_full_list_other]"*** | |

*Question relevant when: selected( ${q104_M73_full_list} , '3')*

| Field | Question | Answer |
|---|---|---|
| q404_M73a *(required)* | q404_M73a. What percent of patients require this item?<br>*Question relevant when: selected( ${q104_M73_full_list} , '3')*<br>*Response constrained to: .>0 and .<=100 or .=999* | |
| q404_M73b *(required)* | q404_M73b. How is the item administered, or given, to the patient?<br>*Question relevant when: selected( ${q104_M73_full_list} , '3')* | 1 Oral - solid (e.g. tablet)<br>2 Oral - liquid<br>3 Sublingual<br>4 Intravenous (IV)<br>5 Intramusculuar injection (IM)<br>6 Subcutaneous<br>7 Rectal<br>8 Inhaled (e.g. gas)<br>9 Inserted (e.g. vaginal pessaries)<br>10 Topical - cream<br>11 Topical - drops (e.g. eye drops)<br>12 Other<br>99 Don't know |
| q404_M73c *(required)* | q404_M73c. What is the item's smallest unit of measurement (mg, mcg, ml, g, etc)?<br>*Question relevant when: selected( ${q104_M73_full_list} , '3')* | microgram — microgram (mcg)<br>miligram — miligram (mg)<br>miligram_dissolved_in_liquid — miligram (mg) dissolved in liquid (e.g. 2 mg per 3 militers)<br>gram — gram (g)<br>militer — militer (ml)<br>centiliter — centiliter (cl)<br>litre — litre (l)<br>ml_of _solution — ml of solution (e.g. ml of 10% solution)<br>litre_of_solution — litre of solution (e.g. 1 litre of 5% solution)<br>cubic_meter — cubic meter (m^3)<br>other — other<br>dont_know — don't know |
| q404_M73c.1 | q404_M73c.1 If other unit of measurement or route of administration, specify here:<br>*Leave blank if not applicable*<br>*Question relevant when: selected( ${q104_M73_full_list} , '3')* | |
| q404_M73d *(required)* | q404_M73d. In total, how many units are used to treat a patient during all of her care?<br>*Remember to add all units! E.g. 5 days x 200 mg twice per day = 2,000mg*<br>*Question relevant when: selected( ${q104_M73_full_list} , '3')*<br>*Response constrained to: .>0* | |
| q404_M74 | ***q404_M74. Blood product - other 2: "[q104_M74_full_list_other]"*** | |

| Field | Question | Answer |
|---|---|---|
| | *Question relevant when: selected( ${q104_M74_full_list} , '3')* | |
| q404_M74a *(required)* | q404_M74a. What percent of patients require this item?<br>*Question relevant when: selected( ${q104_M74_full_list} , '3')*<br>*Response constrained to: .>0 and .<=100 or .=999* | |
| q404_M74b *(required)* | q404_M74b. How is the item administered, or given, to the patient?<br>*Question relevant when: selected( ${q104_M74_full_list} , '3')* | 1 Oral - solid (e.g. tablet)<br>2 Oral - liquid<br>3 Sublingual<br>4 Intravenous (IV)<br>5 Intramusculuar injection (IM)<br>6 Subcutaneous<br>7 Rectal<br>8 Inhaled (e.g. gas)<br>9 Inserted (e.g. vaginal pessaries)<br>10 Topical - cream<br>11 Topical - drops (e.g. eye drops)<br>12 Other<br>99 Don't know |
| q404_M74c *(required)* | q404_M74c. What is the item's smallest unit of measurement (mg, mcg, ml, g, etc)?<br>*Question relevant when: selected( ${q104_M74_full_list} , '3')* | microgram — microgram (mcg)<br>miligram — miligram (mg)<br>miligram_dissolved_in_liquid — miligram (mg) dissolved in liquid (e.g. 2 mg per 3 militers)<br>gram — gram (g)<br>militer — militer (ml)<br>centiliter — centiliter (cl)<br>litre — litre (l)<br>ml_of _solution — ml of solution (e.g. ml of 10% solution)<br>litre_of_solution — litre of solution (e.g. 1 litre of 5% solution)<br>cubic_meter — cubic meter (m^3)<br>other — other<br>dont_know — don't know |
| q404_M74c.1 | q404_M74c.1 If other unit of measurement or route of administration, specify here:<br>*Leave blank if not applicable*<br>*Question relevant when: selected( ${q104_M74_full_list} , '3')* | |
| q404_M74d *(required)* | q404_M74d. In total, how many units are used to treat a patient during all of her care?<br>*Remember to add all units! E.g. 5 days x 200 mg twice per day = 2,000mg*<br>*Question relevant when: selected( ${q104_M74_full_list} , '3')*<br>*Response constrained to: .>0* | |
| q404_M75 | ***q404_M75. Blood product - other 3: "[q104_M75_full_list_other]"***<br>[illegible] | |

*Question relevant when: selected( ${q104_M75_full_list} , '3')*

| Field | Question | Answer |
|---|---|---|
| q404_M75a *(required)* | q404_M75a. What percent of patients require this item?<br>*Question relevant when: selected( ${q104_M75_full_list} , '3')*<br>*Response constrained to: .>0 and .<=100 or .=999* | |
| q404_M75b *(required)* | q404_M75b. How is the item administered, or given, to the patient?<br>*Question relevant when: selected( ${q104_M75_full_list} , '3')* | 1 Oral - solid (e.g. tablet)<br>2 Oral - liquid<br>3 Sublingual<br>4 Intravenous (IV)<br>5 Intramusculuar injection (IM)<br>6 Subcutaneous<br>7 Rectal<br>8 Inhaled (e.g. gas)<br>9 Inserted (e.g. vaginal pessaries)<br>10 Topical - cream<br>11 Topical - drops (e.g. eye drops)<br>12 Other<br>99 Don't know |
| q404_M75c *(required)* | q404_M75c. What is the item's smallest unit of measurement (mg, mcg, ml, g, etc)?<br>*Question relevant when: selected( ${q104_M75_full_list} , '3')* | microgram — microgram (mcg)<br>miligram — miligram (mg)<br>miligram_dissolved_in_liquid — miligram (mg) dissolved in liquid (e.g. 2 mg per 3 militers)<br>gram — gram (g)<br>militer — militer (ml)<br>centiliter — centiliter (cl)<br>litre — litre (l)<br>ml_of _solution — ml of solution (e.g. ml of 10% solution)<br>litre_of_solution — litre of solution (e.g. 1 litre of 5% solution)<br>cubic_meter — cubic meter (m^3)<br>other — other<br>dont_know — don't know |
| q404_M75c.1 | q404_M75c.1 If other unit of measurement or route of administration, specify here:<br>*Leave blank if not applicable*<br>*Question relevant when: selected( ${q104_M75_full_list} , '3')* | |
| q404_M75d *(required)* | q404_M75d. In total, how many units are used to treat a patient during all of her care?<br>*Remember to add all units! E.g. 5 days x 200 mg twice per day = 2,000mg*<br>*Question relevant when: selected( ${q104_M75_full_list} , '3')*<br>*Response constrained to: .>0* | |
| q404_M75.1 | ***q404_M75.1. Blood product - other 4: "[q104_M75.1_full_list_other]"*** | |

Question relevant when: selected( ${q104_M75.1_full_list} , '2')

| Field | Question | Answer |
|---|---|---|
| q404_M75.1a *(required)* | q404_M75.1a. What percent of patients require this item?<br>*Question relevant when: selected( ${q104_M75.1_full_list} , '2')*<br>*Response constrained to: .>0 and .<=100 or .=999* | |

| | | |
|---|---|---|
| q404_M75.1b *(required)* | q404_M75.1b. How is the item administered, or given, to the patient?<br>*Question relevant when: selected( ${q104_M75.1_full_list} , '2')* | 1 Oral - solid (e.g. tablet)<br>2 Oral - liquid<br>3 Sublingual<br>4 Intravenous (IV)<br>5 Intramusculuar injection (IM)<br>6 Subcutaneous<br>7 Rectal<br>8 Inhaled (e.g. gas)<br>9 Inserted (e.g. vaginal pessaries)<br>10 Topical - cream<br>11 Topical - drops (e.g. eye drops)<br>12 Other<br>99 Don't know |
| q404_M75.1c *(required)* | q404_M75.1c. What is the item's smallest unit of measurement (mg, mcg, ml, g, etc)?<br>*Question relevant when: selected( ${q104_M75.1_full_list} , '2')* | microgram — microgram (mcg)<br>miligram — miligram (mg)<br>miligram_dissolved_in_liquid — miligram (mg) dissolved in liquid (e.g. 2 mg per 3 militers)<br>gram — gram (g)<br>militer — militer (ml)<br>centiliter — centiliter (cl)<br>litre — litre (l)<br>ml_of _solution — ml of solution (e.g. ml of 10% solution)<br>litre_of_solution — litre of solution (e.g. 1 litre of 5% solution)<br>cubic_meter — cubic meter (m^3)<br>other — other<br>dont_know — don't know |
| q404_M75.1c.1 | q404_M75.1c.1 If other unit of measurement or route of administration, specify here:<br>*Leave blank if not applicable*<br>*Question relevant when: selected( ${q104_M75.1_full_list} , '2')* | |
| q404_M75.1d *(required)* | q404_M75.1d. In total, how many units are used to treat a patient during all of her care?<br>*Remember to add all units! E.g. 5 days x 200 mg twice per day = 2,000mg*<br>*Question relevant when: selected( ${q104_M75.1_full_list} , '2')*<br>*Response constrained to: .>0* | |
| q404_M75.2 | ***q404_M75.2. Blood product - other 5: "[q104_M75.2_full_list_other]"*** | |

Question relevant when: selected( ${q104_M75.2_full_list} , '2')

| Field | Question | Answer |
|---|---|---|
| q404_M75.2a *(required)* | q404_M75.2a. What percent of patients require this item?<br>*Question relevant when: selected( ${q104_M75.2_full_list} , '2')*<br>*Response constrained to: .>0 and .<=100 or .=999* | |
| q404_M75.2b *(required)* | q404_M75.2b. How is the item administered, or given, to the patient?<br>*Question relevant when: selected( ${q104_M75.2_full_list} , '2')* | 1 Oral - solid (e.g. tablet)<br>2 Oral - liquid<br>3 Sublingual<br>4 Intravenous (IV)<br>5 Intramusculuar injection (IM)<br>6 Subcutaneous<br>7 Rectal<br>8 Inhaled (e.g. gas)<br>9 Inserted (e.g. vaginal pessaries)<br>10 Topical - cream<br>11 Topical - drops (e.g. eye drops)<br>12 Other<br>99 Don't know |
| q404_M75.2c *(required)* | q404_M75.2c. What is the item's smallest unit of measurement (mg, mcg, ml, g, etc)?<br>*Question relevant when: selected( ${q104_M75.2_full_list} , '2')* | microgram — microgram (mcg)<br>miligram — miligram (mg)<br>miligram_dissolved_in_liquid — miligram (mg) dissolved in liquid (e.g. 2 mg per 3 militers)<br>gram — gram (g)<br>militer — militer (ml)<br>centiliter — centiliter (cl)<br>litre — litre (l)<br>ml_of _solution — ml of solution (e.g. ml of 10% solution)<br>litre_of_solution — litre of solution (e.g. 1 litre of 5% solution)<br>cubic_meter — cubic meter (m^3)<br>other — other<br>dont_know — don't know |
| q404_M75.2c.1 | q404_M75.2c.1 If other unit of measurement or route of administration, specify here:<br>*Leave blank if not applicable*<br>*Question relevant when: selected( ${q104_M75.2_full_list} , '2')* | |
| q404_M75.2d *(required)* | q404_M75.2d. In total, how many units are used to treat a patient during all of her care?<br>*Remember to add all units! E.g. 5 days x 200 mg twice per day = 2,000mg*<br>*Question relevant when: selected( ${q104_M75.2_full_list} , '2')*<br>*Response constrained to: .>0* | |

<p><strong><em>D. Medications - Shock (8)</em></strong></p>

*Group relevant when: selected( ${section_four_skip_med} , '1')*

| Field | Question | Answer |
|---|---|---|
| q404_med_note8 | 404 CONTINUED For each of the following medications and other medical products that are used for shock, can you tell me …? | |
| note_404_hemostatic | ***Hemostatics*** | |
| q404_M76 | ***q404_M76. Dicynone / Dicynene (Etamsylate)***<br>*Question relevant when: selected( ${q104_M76_full_list} , '3')* | |
| q404_M76a *(required)* | q404_M76a. What percent of patients require this item?<br>*Question relevant when: selected( ${q104_M76_full_list} , '3')*<br>*Response constrained to: .>0 and .<=100 or .=999* | |
| q404_M76b *(required)* | q404_M76b. How is the item administered, or given, to the patient?<br>*Question relevant when: selected( ${q104_M76_full_list} , '3')* | 1 Oral - solid (e.g. tablet)<br>2 Oral - liquid<br>3 Sublingual<br>4 Intravenous (IV)<br>5 Intramusculuar injection (IM)<br>6 Subcutaneous<br>7 Rectal<br>8 Inhaled (e.g. gas)<br>9 Inserted (e.g. vaginal pessaries)<br>10 Topical - cream<br>11 Topical - drops (e.g. eye drops)<br>12 Other<br>99 Don't know |
| q404_M76c *(required)* | q404_M76c. What is the item's smallest unit of measurement (mg, mcg, ml, g, etc)?<br>*Question relevant when: selected( ${q104_M76_full_list} , '3')* | microgram — microgram (mcg)<br>miligram — miligram (mg)<br>miligram_dissolved_in_liquid — miligram (mg) dissolved in liquid (e.g. 2 mg per 3 militers)<br>gram — gram (g)<br>militer — militer (ml)<br>centiliter — centiliter (cl)<br>litre — litre (l)<br>ml_of _solution — ml of solution (e.g. ml of 10% solution)<br>litre_of_solution — litre of solution (e.g. 1 litre of 5% solution)<br>cubic_meter — cubic meter (m^3)<br>other — other<br>dont_know — don't know |
| q404_M76c.1 | q404_M76c.1 If other unit of measurement or route of administration, specify here:<br>*Leave blank if not applicable*<br>*Question relevant when: selected( ${q104_M76_full_list} , '3')* | |
| q404_M76d *(required)* | q404_M76d. In total, how many units are used to treat a patient during all of her care?<br>*Remember to add all units! E.g. 5 days x 200 mg twice per day = 2,000mg*<br>*Question relevant when: selected( ${q104_M76_full_list} , '3')*<br>*Response constrained to: .>0* | |
| q404_M77 | ***q404_M77. Tranexamic acid (Kapron, Cyklokapron)*** | |

| Field | Question | Answer |
|---|---|---|
| | *Question relevant when: selected( ${q104_M77_full_list} , '3')* | |
| q404_M77a *(required)* | q404_M77a. What percent of patients require this item?<br>*Question relevant when: selected( ${q104_M77_full_list} , '3')*<br>*Response constrained to: .>0 and .<=100 or .=999* | |
| q404_M77b *(required)* | q404_M77b. How is the item administered, or given, to the patient?<br>*Question relevant when: selected( ${q104_M77_full_list} , '3')* | 1 Oral - solid (e.g. tablet)<br>2 Oral - liquid<br>3 Sublingual<br>4 Intravenous (IV)<br>5 Intramusculuar injection (IM)<br>6 Subcutaneous<br>7 Rectal<br>8 Inhaled (e.g. gas)<br>9 Inserted (e.g. vaginal pessaries)<br>10 Topical - cream<br>11 Topical - drops (e.g. eye drops)<br>12 Other<br>99 Don't know |
| q404_M77c *(required)* | q404_M77c. What is the item's smallest unit of measurement (mg, mcg, ml, g, etc)?<br>*Question relevant when: selected( ${q104_M77_full_list} , '3')* | microgram: microgram (mcg)<br>miligram: miligram (mg)<br>miligram_dissolved_in_liquid: miligram (mg) dissolved in liquid (e.g. 2 mg per 3 militers)<br>gram: gram (g)<br>militer: militer (ml)<br>centiliter: centiliter (cl)<br>litre: litre (l)<br>ml_of _solution: ml of solution (e.g. ml of 10% solution)<br>litre_of_solution: litre of solution (e.g. 1 litre of 5% solution)<br>cubic_meter: cubic meter (m^3)<br>other: other<br>dont_know: don't know |
| q404_M77c.1 | q404_M77c.1 If other unit of measurement or route of administration, specify here:<br>*Leave blank if not applicable*<br>*Question relevant when: selected( ${q104_M77_full_list} , '3')* | |
| q404_M77d *(required)* | q404_M77d. In total, how many units are used to treat a patient during all of her care?<br>*Remember to add all units! E.g. 5 days x 200 mg twice per day = 2,000mg*<br>*Question relevant when: selected( ${q104_M77_full_list} , '3')*<br>*Response constrained to: .>0* | |
| q404_M78 | ***q404_M78. Polygeline 4%*** | |

| Field | Question | Answer |
|---|---|---|
| | *Question relevant when: selected( ${q104_M78_full_list} , '3')* | |
| q404_M78a *(required)* | q404_M78a. What percent of patients require this item? | |
| | *Question relevant when: selected( ${q104_M78_full_list} , '3')*<br>*Response constrained to: .>0 and .<=100 or .=999* | |
| q404_M78b *(required)* | q404_M78b. How is the item administered, or given, to the patient?<br>*Question relevant when: selected( ${q104_M78_full_list} , '3')* | 1 Oral - solid (e.g. tablet)<br>2 Oral - liquid<br>3 Sublingual<br>4 Intravenous (IV)<br>5 Intramusculuar injection (IM)<br>6 Subcutaneous<br>7 Rectal<br>8 Inhaled (e.g. gas)<br>9 Inserted (e.g. vaginal pessaries)<br>10 Topical - cream<br>11 Topical - drops (e.g. eye drops)<br>12 Other<br>99 Don't know |
| q404_M78c *(required)* | q404_M78c. What is the item's smallest unit of measurement (mg, mcg, ml, g, etc)?<br>*Question relevant when: selected( ${q104_M78_full_list} , '3')* | microgram — microgram (mcg)<br>miligram — miligram (mg)<br>miligram_dissolved_in_liquid — miligram (mg) dissolved in liquid (e.g. 2 mg per 3 militers)<br>gram — gram (g)<br>militer — militer (ml)<br>centiliter — centiliter (cl)<br>litre — litre (l)<br>ml_of _solution — ml of solution (e.g. ml of 10% solution)<br>litre_of_solution — litre of solution (e.g. 1 litre of 5% solution)<br>cubic_meter — cubic meter (m^3)<br>other — other<br>dont_know — don't know |
| q404_M78c.1 | q404_M78c.1 If other unit of measurement or route of administration, specify here:<br>*Leave blank if not applicable*<br>*Question relevant when: selected( ${q104_M78_full_list} , '3')* | |
| q404_M78d *(required)* | q404_M78d. In total, how many units are used to treat a patient during all of her care?<br>*Remember to add all units! E.g. 5 days x 200 mg twice per day = 2,000mg*<br>*Question relevant when: selected( ${q104_M78_full_list} , '3')*<br>*Response constrained to: .>0* | |
| q404_M79 | ***q404_M79. Hemostatic - other 1: "[q104_M79_full_list_other]"*** | |

| Field | Question | Answer |
|---|---|---|
| | *Question relevant when: selected( ${q104_M79_full_list} , '3')* | |
| q404_M79a *(required)* | q404_M79a. What percent of patients require this item?<br>*Question relevant when: selected( ${q104_M79_full_list} , '3')*<br>*Response constrained to: .>0 and .<=100 or .=999* | |
| q404_M79b *(required)* | q404_M79b. How is the item administered, or given, to the patient?<br>*Question relevant when: selected( ${q104_M79_full_list} , '3')* | 1 Oral - solid (e.g. tablet)<br>2 Oral - liquid<br>3 Sublingual<br>4 Intravenous (IV)<br>5 Intramusculuar injection (IM)<br>6 Subcutaneous<br>7 Rectal<br>8 Inhaled (e.g. gas)<br>9 Inserted (e.g. vaginal pessaries)<br>10 Topical - cream<br>11 Topical - drops (e.g. eye drops)<br>12 Other<br>99 Don't know |
| q404_M79c *(required)* | q404_M79c. What is the item's smallest unit of measurement (mg, mcg, ml, g, etc)?<br>*Question relevant when: selected( ${q104_M79_full_list} , '3')* | microgram — microgram (mcg)<br>miligram — miligram (mg)<br>miligram_dissolved_in_liquid — miligram (mg) dissolved in liquid (e.g. 2 mg per 3 militers)<br>gram — gram (g)<br>militer — militer (ml)<br>centiliter — centiliter (cl)<br>litre — litre (l)<br>ml_of _solution — ml of solution (e.g. ml of 10% solution)<br>litre_of_solution — litre of solution (e.g. 1 litre of 5% solution)<br>cubic_meter — cubic meter (m^3)<br>other — other<br>dont_know — don't know |
| q404_M79c.1 | q404_M79c.1 If other unit of measurement or route of administration, specify here:<br>*Leave blank if not applicable*<br>*Question relevant when: selected( ${q104_M79_full_list} , '3')* | |
| q404_M79d *(required)* | q404_M79d. In total, how many units are used to treat a patient during all of her care?<br>*Remember to add all units! E.g. 5 days x 200 mg twice per day = 2,000mg*<br>*Question relevant when: selected( ${q104_M79_full_list} , '3')*<br>*Response constrained to: .>0* | |
| q404_M80 | ***q404_M80. Hemostatic - other 2: "[q104_M80_full_list_other]"*** | |

| Field | Question | Answer |
|---|---|---|
| | *Question relevant when: selected( ${q104_M80_full_list} , '3')* | |
| q404_M80a *(required)* | q404_M80a. What percent of patients require this item?<br>*Question relevant when: selected( ${q104_M80_full_list} , '3')*<br>*Response constrained to: .>0 and .<=100 or .=999* | |
| q404_M80b *(required)* | q404_M80b. How is the item administered, or given, to the patient?<br>*Question relevant when: selected( ${q104_M80_full_list} , '3')* | 1 Oral - solid (e.g. tablet)<br>2 Oral - liquid<br>3 Sublingual<br>4 Intravenous (IV)<br>5 Intramusculuar injection (IM)<br>6 Subcutaneous<br>7 Rectal<br>8 Inhaled (e.g. gas)<br>9 Inserted (e.g. vaginal pessaries)<br>10 Topical - cream<br>11 Topical - drops (e.g. eye drops)<br>12 Other<br>99 Don't know |
| q404_M80c *(required)* | q404_M80c. What is the item's smallest unit of measurement (mg, mcg, ml, g, etc)?<br>*Question relevant when: selected( ${q104_M80_full_list} , '3')* | microgram — microgram (mcg)<br>miligram — miligram (mg)<br>miligram_dissolved_in_liquid — miligram (mg) dissolved in liquid (e.g. 2 mg per 3 militers)<br>gram — gram (g)<br>militer — militer (ml)<br>centiliter — centiliter (cl)<br>litre — litre (l)<br>ml_of _solution — ml of solution (e.g. ml of 10% solution)<br>litre_of_solution — litre of solution (e.g. 1 litre of 5% solution)<br>cubic_meter — cubic meter (m^3)<br>other — other<br>dont_know — don't know |
| q404_M80c.1 | q404_M80c.1 If other unit of measurement or route of administration, specify here:<br>*Leave blank if not applicable*<br>*Question relevant when: selected( ${q104_M80_full_list} , '3')* | |
| q404_M80d *(required)* | q404_M80d. In total, how many units are used to treat a patient during all of her care?<br>*Remember to add all units! E.g. 5 days x 200 mg twice per day = 2,000mg*<br>*Question relevant when: selected( ${q104_M80_full_list} , '3')*<br>*Response constrained to: .>0* | |
| q404_M81 | ***q404_M81. Hemostatic - other 3: "[q104_M81_full_list_other]"*** | |

| Field | Question | Answer |
|---|---|---|
| | *Question relevant when: selected( ${q104_M81_full_list} , '3')* | |
| q404_M81a *(required)* | q404_M81a. What percent of patients require this item? | |
| | *Question relevant when: selected( ${q104_M81_full_list} , '3')*<br>*Response constrained to: .>0 and .<=100 or .=999* | |

| | | |
|---|---|---|
| q404_M81b *(required)* | q404_M81b. How is the item administered, or given, to the patient?<br>*Question relevant when: selected( ${q104_M81_full_list} , '3')* | 1 Oral - solid (e.g. tablet)<br>2 Oral - liquid<br>3 Sublingual<br>4 Intravenous (IV)<br>5 Intramusculuar injection (IM)<br>6 Subcutaneous<br>7 Rectal<br>8 Inhaled (e.g. gas)<br>9 Inserted (e.g. vaginal pessaries)<br>10 Topical - cream<br>11 Topical - drops (e.g. eye drops)<br>12 Other<br>99 Don't know |
| q404_M81c *(required)* | q404_M81c. What is the item's smallest unit of measurement (mg, mcg, ml, g, etc)?<br>*Question relevant when: selected( ${q104_M81_full_list} , '3')* | microgram — microgram (mcg)<br>miligram — miligram (mg)<br>miligram_dissolved_in_liquid — miligram (mg) dissolved in liquid (e.g. 2 mg per 3 militers)<br>gram — gram (g)<br>militer — militer (ml)<br>centiliter — centiliter (cl)<br>litre — litre (l)<br>ml_of _solution — ml of solution (e.g. ml of 10% solution)<br>litre_of_solution — litre of solution (e.g. 1 litre of 5% solution)<br>cubic_meter — cubic meter (m^3)<br>other — other<br>dont_know — don't know |
| q404_M81c.1 | q404_M81c.1 If other unit of measurement or route of administration, specify here:<br>*Leave blank if not applicable*<br>*Question relevant when: selected( ${q104_M81_full_list} , '3')* | |
| q404_M81d *(required)* | q404_M81d. In total, how many units are used to treat a patient during all of her care?<br>*Remember to add all units! E.g. 5 days x 200 mg twice per day = 2,000mg*<br>*Question relevant when: selected( ${q104_M81_full_list} , '3')*<br>*Response constrained to: .>0* | |
| q404_M81.1 | ***q404_M81.1. Hemostatic - other 4: "[q104_M81.1_full_list_other]"*** | |

| Field | Question | Answer |
|---|---|---|
| | *Question relevant when: selected( ${q104_M81.1_full_list} , '2')* | |
| q404_M81.1a *(required)* | q404_M81.1a. What percent of patients require this item?<br>*Question relevant when: selected( ${q104_M81.1_full_list} , '2')*<br>*Response constrained to: .>0 and .<=100 or .=999* | |

| | | |
|---|---|---|
| q404_M81.1b *(required)* | q404_M81.1b. How is the item administered, or given, to the patient?<br>*Question relevant when: selected( ${q104_M81.1_full_list} , '2')* | 1 Oral - solid (e.g. tablet)<br>2 Oral - liquid<br>3 Sublingual<br>4 Intravenous (IV)<br>5 Intramusculuar injection (IM)<br>6 Subcutaneous<br>7 Rectal<br>8 Inhaled (e.g. gas)<br>9 Inserted (e.g. vaginal pessaries)<br>10 Topical - cream<br>11 Topical - drops (e.g. eye drops)<br>12 Other<br>99 Don't know |
| q404_M81.1c *(required)* | q404_M81.1c. What is the item's smallest unit of measurement (mg, mcg, ml, g, etc)?<br>*Question relevant when: selected( ${q104_M81.1_full_list} , '2')* | microgram — microgram (mcg)<br>miligram — miligram (mg)<br>miligram_dissolved_in_liquid — miligram (mg) dissolved in liquid (e.g. 2 mg per 3 militers)<br>gram — gram (g)<br>militer — militer (ml)<br>centiliter — centiliter (cl)<br>litre — litre (l)<br>ml_of _solution — ml of solution (e.g. ml of 10% solution)<br>litre_of_solution — litre of solution (e.g. 1 litre of 5% solution)<br>cubic_meter — cubic meter (m^3)<br>other — other<br>dont_know — don't know |
| q404_M81.1c.1 | q404_M81.1c.1 If other unit of measurement or route of administration, specify here:<br>*Leave blank if not applicable*<br>*Question relevant when: selected( ${q104_M81.1_full_list} , '2')* | |
| q404_M81.1d *(required)* | q404_M81.1d. In total, how many units are used to treat a patient during all of her care?<br>*Remember to add all units! E.g. 5 days x 200 mg twice per day = 2,000mg*<br>*Question relevant when: selected( ${q104_M81.1_full_list} , '2')*<br>*Response constrained to: .>0* | |
| q404_M81.2 | ***q404_M81.2. Hemostatic - other 5: "[q104_M81.2_full_list_other]"*** | |

| Field | Question | Answer |
|---|---|---|
| | *Question relevant when: selected( ${q104_M81.2_full_list} , '2')* | |
| q404_M81.2a *(required)* | q404_M81.2a. What percent of patients require this item? | |
| | *Question relevant when: selected( ${q104_M81.2_full_list} , '2')*<br>*Response constrained to: .>0 and .<=100 or .=999* | |

| Field | Question | Answer | |
|---|---|---|---|
| q404_M81.2b *(required)* | q404_M81.2b. How is the item administered, or given, to the patient?<br>*Question relevant when: selected( ${q104_M81.2_full_list} , '2')* | 1 | Oral - solid (e.g. tablet) |
| | | 2 | Oral - liquid |
| | | 3 | Sublingual |
| | | 4 | Intravenous (IV) |
| | | 5 | Intramusculuar injection (IM) |
| | | 6 | Subcutaneous |
| | | 7 | Rectal |
| | | 8 | Inhaled (e.g. gas) |
| | | 9 | Inserted (e.g. vaginal pessaries) |
| | | 10 | Topical - cream |
| | | 11 | Topical - drops (e.g. eye drops) |
| | | 12 | Other |
| | | 99 | Don't know |
| q404_M81.2c *(required)* | q404_M81.2c. What is the item's smallest unit of measurement (mg, mcg, ml, g, etc)?<br>*Question relevant when: selected( ${q104_M81.2_full_list} , '2')* | microgram | microgram (mcg) |
| | | miligram | miligram (mg) |
| | | miligram_dissolved_in_liquid | miligram (mg) dissolved in liquid (e.g. 2 mg per 3 militers) |
| | | gram | gram (g) |
| | | militer | militer (ml) |
| | | centiliter | centiliter (cl) |
| | | litre | litre (l) |
| | | ml_of _solution | ml of solution (e.g. ml of 10% solution) |
| | | litre_of_solution | litre of solution (e.g. 1 litre of 5% solution) |
| | | cubic_meter | cubic meter (m^3) |
| | | other | other |
| | | dont_know | don't know |
| q404_M81.2c.1 | q404_M81.2c.1 If other unit of measurement or route of administration, specify here:<br>*Leave blank if not applicable*<br>*Question relevant when: selected( ${q104_M81.2_full_list} , '2')* | | |
| q404_M81.2d *(required)* | q404_M81.2d. In total, how many units are used to treat a patient during all of her care?<br>*Remember to add all units! E.g. 5 days x 200 mg twice per day = 2,000mg*<br>*Question relevant when: selected( ${q104_M81.2_full_list} , '2')*<br>*Response constrained to: .>0* | | |

<p><strong><em>D. Medications - Shock (9)</em></strong></p>

*Group relevant when: selected( ${section_four_skip_med} , '1')*

| Field | Question | Answer |
|---|---|---|
| q404_med_notE8 | 404 CONTINUED For each of the following medications and other medical products that are used for shock, can you tell me …? | |
| note_404_saline | ***Saline, glucose, water, etc*** | |
| q404_M82 | ***q404_M82. IV solution, dextrose 2.5% in NaCl 0.45%***<br>*Question relevant when: selected( ${q104_M82_full_list} , '3')* | |
| q404_M82a *(required)* | q404_M82a. What percent of patients require this item?<br>*Question relevant when: selected( ${q104_M82_full_list} , '3')*<br>*Response constrained to: .>0 and .<=100 or .=999* | |

| Field | Question | Answer |
|---|---|---|
| q404_M82b *(required)* | q404_M82b. How is the item administered, or given, to the patient?<br>*Question relevant when: selected( ${q104_M82_full_list} , '3')* | 1 Oral - solid (e.g. tablet)<br>2 Oral - liquid<br>3 Sublingual<br>4 Intravenous (IV)<br>5 Intramusculuar injection (IM)<br>6 Subcutaneous<br>7 Rectal<br>8 Inhaled (e.g. gas)<br>9 Inserted (e.g. vaginal pessaries)<br>10 Topical - cream<br>11 Topical - drops (e.g. eye drops)<br>12 Other<br>99 Don't know |
| q404_M82c *(required)* | q404_M82c. What is the item's smallest unit of measurement (mg, mcg, ml, g, etc)?<br>*Question relevant when: selected( ${q104_M82_full_list} , '3')* | microgram: microgram (mcg)<br>miligram: miligram (mg)<br>miligram_dissolved_in_liquid: miligram (mg) dissolved in liquid (e.g. 2 mg per 3 militers)<br>gram: gram (g)<br>militer: militer (ml)<br>centiliter: centiliter (cl)<br>litre: litre (l)<br>ml_of _solution: ml of solution (e.g. ml of 10% solution)<br>litre_of_solution: litre of solution (e.g. 1 litre of 5% solution)<br>cubic_meter: cubic meter (m^3)<br>other: other<br>dont_know: don't know |
| q404_M82c.1 | q404_M82c.1 If other unit of measurement or route of administration, specify here:<br>*Leave blank if not applicable*<br>*Question relevant when: selected( ${q104_M82_full_list} , '3')* | |
| q404_M82d *(required)* | q404_M82d. In total, how many units are used to treat a patient during all of her care?<br>*Remember to add all units! E.g. 5 days x 200 mg twice per day = 2,000mg*<br>*Question relevant when: selected( ${q104_M82_full_list} , '3')*<br>*Response constrained to: .>0* | |
| q404_M83 | ***q404_M83. IV solution, glucose (5%), isotonic*** | |

| Field | Question | Answer |
|---|---|---|
| | *Question relevant when: selected( ${q104_M83_full_list} , '3')*<br>q404_M83a. What percent of patients require this item? | |
| q404_M83a *(required)* | *Question relevant when: selected( ${q104_M83_full_list} , '3')*<br>*Response constrained to: .>0 and .<=100 or .=999* | |

| | | |
|---|---|---|
| q404_M83b *(required)* | q404_M83b. How is the item administered, or given, to the patient?<br>*Question relevant when: selected( ${q104_M83_full_list} , '3')* | 1 Oral - solid (e.g. tablet)<br>2 Oral - liquid<br>3 Sublingual<br>4 Intravenous (IV)<br>5 Intramusculuar injection (IM)<br>6 Subcutaneous<br>7 Rectal<br>8 Inhaled (e.g. gas)<br>9 Inserted (e.g. vaginal pessaries)<br>10 Topical - cream<br>11 Topical - drops (e.g. eye drops)<br>12 Other<br>99 Don't know |
| q404_M83c *(required)* | q404_M83c. What is the item's smallest unit of measurement (mg, mcg, ml, g, etc)?<br>*Question relevant when: selected( ${q104_M83_full_list} , '3')* | microgram — microgram (mcg)<br>miligram — miligram (mg)<br>miligram_dissolved_in_liquid — miligram (mg) dissolved in liquid (e.g. 2 mg per 3 militers)<br>gram — gram (g)<br>militer — militer (ml)<br>centiliter — centiliter (cl)<br>litre — litre (l)<br>ml_of _solution — ml of solution (e.g. ml of 10% solution)<br>litre_of_solution — litre of solution (e.g. 1 litre of 5% solution)<br>cubic_meter — cubic meter (m^3)<br>other — other<br>dont_know — don't know |
| q404_M83c.1 | q404_M83c.1 If other unit of measurement or route of administration, specify here:<br>*Leave blank if not applicable*<br>*Question relevant when: selected( ${q104_M83_full_list} , '3')* | |
| q404_M83d *(required)* | q404_M83d. In total, how many units are used to treat a patient during all of her care?<br>*Remember to add all units! E.g. 5 days x 200 mg twice per day = 2,000mg*<br>*Question relevant when: selected( ${q104_M83_full_list} , '3')*<br>*Response constrained to: .>0* | |

| Field | Question | Answer |
|---|---|---|
| q404_M84 | ***q404_M84. Ringers lactate (prepared onsite)***<br>*Question relevant when: selected( ${q104_M84_full_list} , '3')* | |
| q404_M84a *(required)* | q404_M84a. What percent of patients require this item?<br>*Question relevant when: selected( ${q104_M84_full_list} , '3')*<br>*Response constrained to: .>0 and .<=100 or .=999* | |
| q404_M84b *(required)* | q404_M84b. How is the item administered, or given, to the patient?<br>*Question relevant when: selected( ${q104_M84_full_list} , '3')* | 1 Oral - solid (e.g. tablet)<br>2 Oral - liquid<br>3 Sublingual<br>4 Intravenous (IV)<br>5 Intramusculuar injection (IM)<br>6 Subcutaneous<br>7 Rectal<br>8 Inhaled (e.g. gas)<br>9 Inserted (e.g. vaginal pessaries)<br>10 Topical - cream<br>11 Topical - drops (e.g. eye drops)<br>12 Other<br>99 Don't know |
| q404_M84c *(required)* | q404_M84c. What is the item's smallest unit of measurement (mg, mcg, ml, g, etc)?<br>*Question relevant when: selected( ${q104_M84_full_list} , '3')* | microgram: microgram (mcg)<br>miligram: miligram (mg)<br>miligram_dissolved_in_liquid: miligram (mg) dissolved in liquid (e.g. 2 mg per 3 militers)<br>gram: gram (g)<br>militer: militer (ml)<br>centiliter: centiliter (cl)<br>litre: litre (l)<br>ml_of _solution: ml of solution (e.g. ml of 10% solution)<br>litre_of_solution: litre of solution (e.g. 1 litre of 5% solution)<br>cubic_meter: cubic meter (m^3)<br>other: other<br>dont_know: don't know |
| q404_M84c.1 | q404_M84c.1 If other unit of measurement or route of administration, specify here:<br>*Leave blank if not applicable*<br>*Question relevant when: selected( ${q104_M84_full_list} , '3')* | |
| q404_M84d *(required)* | q404_M84d. In total, how many units are used to treat a patient during all of her care?<br>*Remember to add all units! E.g. 5 days x 200 mg twice per day = 2,000mg*<br>*Question relevant when: selected( ${q104_M84_full_list} , '3')*<br>*Response constrained to: .>0* | |

| Field | Question | Answer |
|---|---|---|
| q404_M85 | ***q404_M85. Ringers lactate (bought from pharmacy/MSD)***<br>*Question relevant when: selected( ${q104_M85_full_list} , '3')* | |
| q404_M85a *(required)* | q404_M85a. What percent of patients require this item?<br>*Question relevant when: selected( ${q104_M85_full_list} , '3')*<br>*Response constrained to: .>0 and .<=100 or .=999* | |
| q404_M85b *(required)* | q404_M85b. How is the item administered, or given, to the patient?<br>*Question relevant when: selected( ${q104_M85_full_list} , '3')* | 1 Oral - solid (e.g. tablet)<br>2 Oral - liquid<br>3 Sublingual<br>4 Intravenous (IV)<br>5 Intramusculuar injection (IM)<br>6 Subcutaneous<br>7 Rectal<br>8 Inhaled (e.g. gas)<br>9 Inserted (e.g. vaginal pessaries)<br>10 Topical - cream<br>11 Topical - drops (e.g. eye drops)<br>12 Other<br>99 Don't know |
| q404_M85c *(required)* | q404_M85c. What is the item's smallest unit of measurement (mg, mcg, ml, g, etc)?<br>*Question relevant when: selected( ${q104_M85_full_list} , '3')* | microgram — microgram (mcg)<br>miligram — miligram (mg)<br>miligram_dissolved_in_liquid — miligram (mg) dissolved in liquid (e.g. 2 mg per 3 militers)<br>gram — gram (g)<br>militer — militer (ml)<br>centiliter — centiliter (cl)<br>litre — litre (l)<br>ml_of _solution — ml of solution (e.g. ml of 10% solution)<br>litre_of_solution — litre of solution (e.g. 1 litre of 5% solution)<br>cubic_meter — cubic meter (m^3)<br>other — other<br>dont_know — don't know |
| q404_M85c.1 | q404_M85c.1 If other unit of measurement or route of administration, specify here:<br>*Leave blank if not applicable*<br>*Question relevant when: selected( ${q104_M85_full_list} , '3')* | |
| q404_M85d *(required)* | q404_M85d. In total, how many units are used to treat a patient during all of her care?<br>*Remember to add all units! E.g. 5 days x 200 mg twice per day = 2,000mg*<br>*Question relevant when: selected( ${q104_M85_full_list} , '3')*<br>*Response constrained to: .>0* | |

| Field | Question | Answer |
|---|---|---|
| q404_M86 | ***q404_M86. Saline, 0.9% (Normal Saline) (prepared onsite)***<br>*Question relevant when: selected( ${q104_M86_full_list} , '3')* | |
| q404_M86a *(required)* | q404_M86a. What percent of patients require this item?<br>*Question relevant when: selected( ${q104_M86_full_list} , '3')*<br>*Response constrained to: .>0 and .<=100 or .=999* | |
| q404_M86b *(required)* | q404_M86b. How is the item administered, or given, to the patient?<br>*Question relevant when: selected( ${q104_M86_full_list} , '3')* | 1 Oral - solid (e.g. tablet)<br>2 Oral - liquid<br>3 Sublingual<br>4 Intravenous (IV)<br>5 Intramusculuar injection (IM)<br>6 Subcutaneous<br>7 Rectal<br>8 Inhaled (e.g. gas)<br>9 Inserted (e.g. vaginal pessaries)<br>10 Topical - cream<br>11 Topical - drops (e.g. eye drops)<br>12 Other<br>99 Don't know |
| q404_M86c *(required)* | q404_M86c. What is the item's smallest unit of measurement (mg, mcg, ml, g, etc)?<br>*Question relevant when: selected( ${q104_M86_full_list} , '3')* | microgram — microgram (mcg)<br>miligram — miligram (mg)<br>miligram_dissolved_in_liquid — miligram (mg) dissolved in liquid (e.g. 2 mg per 3 militers)<br>gram — gram (g)<br>militer — militer (ml)<br>centiliter — centiliter (cl)<br>litre — litre (l)<br>ml_of _solution — ml of solution (e.g. ml of 10% solution)<br>litre_of_solution — litre of solution (e.g. 1 litre of 5% solution)<br>cubic_meter — cubic meter (m^3)<br>other — other<br>dont_know — don't know |
| q404_M86c.1 | q404_M86c.1 If other unit of measurement or route of administration, specify here:<br>*Leave blank if not applicable*<br>*Question relevant when: selected( ${q104_M86_full_list} , '3')* | |
| q404_M86d *(required)* | q404_M86d. In total, how many units are used to treat a patient during all of her care?<br>*Remember to add all units! E.g. 5 days x 200 mg twice per day = 2,000mg*<br>*Question relevant when: selected( ${q104_M86_full_list} , '3')*<br>*Response constrained to: .>0* | |

| Field | Question | Answer |
|---|---|---|
| q404_M87 | ***q404_M87. Saline, 0.9% (Normal Saline) (bought from pharmacy/MSD)***<br>*Question relevant when: selected( ${q104_M87_full_list} , '3')* | |
| q404_M87a *(required)* | q404_M87a. What percent of patients require this item?<br>*Question relevant when: selected( ${q104_M87_full_list} , '3')*<br>*Response constrained to: .>0 and .<=100 or .=999* | |
| q404_M87b *(required)* | q404_M87b. How is the item administered, or given, to the patient?<br>*Question relevant when: selected( ${q104_M87_full_list} , '3')* | 1 Oral - solid (e.g. tablet)<br>2 Oral - liquid<br>3 Sublingual<br>4 Intravenous (IV)<br>5 Intramusculuar injection (IM)<br>6 Subcutaneous<br>7 Rectal<br>8 Inhaled (e.g. gas)<br>9 Inserted (e.g. vaginal pessaries)<br>10 Topical - cream<br>11 Topical - drops (e.g. eye drops)<br>12 Other<br>99 Don't know |
| q404_M87c *(required)* | q404_M87c. What is the item's smallest unit of measurement (mg, mcg, ml, g, etc)?<br>*Question relevant when: selected( ${q104_M87_full_list} , '3')* | microgram — microgram (mcg)<br>miligram — miligram (mg)<br>miligram_dissolved_in_liquid — miligram (mg) dissolved in liquid (e.g. 2 mg per 3 militers)<br>gram — gram (g)<br>militer — militer (ml)<br>centiliter — centiliter (cl)<br>litre — litre (l)<br>ml_of _solution — ml of solution (e.g. ml of 10% solution)<br>litre_of_solution — litre of solution (e.g. 1 litre of 5% solution)<br>cubic_meter — cubic meter (m^3)<br>other — other<br>dont_know — don't know |
| q404_M87c.1 | q404_M87c.1 If other unit of measurement or route of administration, specify here:<br>*Leave blank if not applicable*<br>*Question relevant when: selected( ${q104_M87_full_list} , '3')* | |
| q404_M87d *(required)* | q404_M87d. In total, how many units are used to treat a patient during all of her care?<br>*Remember to add all units! E.g. 5 days x 200 mg twice per day = 2,000mg*<br>*Question relevant when: selected( ${q104_M87_full_list} , '3')*<br>*Response constrained to: .>0* | |

| Field | Question | Answer |
|---|---|---|
| q404_M88 | ***q404_M88. Solution for Infusion, glucose 5% + NaCl 0.9% (prepared onsite)***<br>*Question relevant when: selected( ${q104_M88_full_list} , '3')* | |
| q404_M88a *(required)* | q404_M88a. What percent of patients require this item?<br>*Question relevant when: selected( ${q104_M88_full_list} , '3')*<br>*Response constrained to: .>0 and .<=100 or .=999* | |

| Field | Question | Answer |
|---|---|---|
| q404_M88b *(required)* | q404_M88b. How is the item administered, or given, to the patient?<br>*Question relevant when: selected( ${q104_M88_full_list} , '3')* | 1 Oral - solid (e.g. tablet)<br>2 Oral - liquid<br>3 Sublingual<br>4 Intravenous (IV)<br>5 Intramusculuar injection (IM)<br>6 Subcutaneous<br>7 Rectal<br>8 Inhaled (e.g. gas)<br>9 Inserted (e.g. vaginal pessaries)<br>10 Topical - cream<br>11 Topical - drops (e.g. eye drops)<br>12 Other<br>99 Don't know |
| q404_M88c *(required)* | q404_M88c. What is the item's smallest unit of measurement (mg, mcg, ml, g, etc)?<br>*Question relevant when: selected( ${q104_M88_full_list} , '3')* | microgram — microgram (mcg)<br>miligram — miligram (mg)<br>miligram_dissolved_in_liquid — miligram (mg) dissolved in liquid (e.g. 2 mg per 3 militers)<br>gram — gram (g)<br>militer — militer (ml)<br>centiliter — centiliter (cl)<br>litre — litre (l)<br>ml_of _solution — ml of solution (e.g. ml of 10% solution)<br>litre_of_solution — litre of solution (e.g. 1 litre of 5% solution)<br>cubic_meter — cubic meter (m^3)<br>other — other<br>dont_know — don't know |
| q404_M88c.1 | q404_M88c.1 If other unit of measurement or route of administration, specify here:<br>*Leave blank if not applicable*<br>*Question relevant when: selected( ${q104_M88_full_list} , '3')* | |
| q404_M88d *(required)* | q404_M88d. In total, how many units are used to treat a patient during all of her care?<br>*Remember to add all units! E.g. 5 days x 200 mg twice per day = 2,000mg*<br>*Question relevant when: selected( ${q104_M88_full_list} , '3')*<br>*Response constrained to: .>0* | |

| Field | Question | Answer |
|---|---|---|
| q404_M89 | ***q404_M89. Solution for Infusion, glucose 5% + NaCl 0.9% (bought from pharmacy or MSD)***<br>*Question relevant when: selected( ${q104_M89_full_list} , '3')* | |
| q404_M89a *(required)* | q404_M89a. What percent of patients require this item?<br>*Question relevant when: selected( ${q104_M89_full_list} , '3')*<br>*Response constrained to: .>0 and .<=100 or .=999* | |

| Field | Question | Answer |
|---|---|---|
| q404_M89b *(required)* | q404_M89b. How is the item administered, or given, to the patient?<br>*Question relevant when: selected( ${q104_M89_full_list} , '3')* | 1 Oral - solid (e.g. tablet)<br>2 Oral - liquid<br>3 Sublingual<br>4 Intravenous (IV)<br>5 Intramusculuar injection (IM)<br>6 Subcutaneous<br>7 Rectal<br>8 Inhaled (e.g. gas)<br>9 Inserted (e.g. vaginal pessaries)<br>10 Topical - cream<br>11 Topical - drops (e.g. eye drops)<br>12 Other<br>99 Don't know |
| q404_M89c *(required)* | q404_M89c. What is the item's smallest unit of measurement (mg, mcg, ml, g, etc)?<br>*Question relevant when: selected( ${q104_M89_full_list} , '3')* | microgram: microgram (mcg)<br>miligram: miligram (mg)<br>miligram_dissolved_in_liquid: miligram (mg) dissolved in liquid (e.g. 2 mg per 3 militers)<br>gram: gram (g)<br>militer: militer (ml)<br>centiliter: centiliter (cl)<br>litre: litre (l)<br>ml_of _solution: ml of solution (e.g. ml of 10% solution)<br>litre_of_solution: litre of solution (e.g. 1 litre of 5% solution)<br>cubic_meter: cubic meter (m^3)<br>other: other<br>dont_know: don't know |
| q404_M89c.1 | q404_M89c.1 If other unit of measurement or route of administration, specify here:<br>*Leave blank if not applicable*<br>*Question relevant when: selected( ${q104_M89_full_list} , '3')* | |
| q404_M89d *(required)* | q404_M89d. In total, how many units are used to treat a patient during all of her care?<br>*Remember to add all units! E.g. 5 days x 200 mg twice per day = 2,000mg*<br>*Question relevant when: selected( ${q104_M89_full_list} , '3')*<br>*Response constrained to: .>0* | |

| Field | Question | Answer |
|---|---|---|
| q404_M90 | ***q404_M90. Water for injection***<br>*Question relevant when: selected( ${q104_M90_full_list} , '3')* | |
| q404_M90a *(required)* | q404_M90a. What percent of patients require this item?<br>*Question relevant when: selected( ${q104_M90_full_list} , '3')*<br>*Response constrained to: .>0 and .<=100 or .=999* | |
| q404_M90b *(required)* | q404_M90b. How is the item administered, or given, to the patient?<br>*Question relevant when: selected( ${q104_M90_full_list} , '3')* | 1 Oral - solid (e.g. tablet)<br>2 Oral - liquid<br>3 Sublingual<br>4 Intravenous (IV)<br>5 Intramusculuar injection (IM)<br>6 Subcutaneous<br>7 Rectal<br>8 Inhaled (e.g. gas)<br>9 Inserted (e.g. vaginal pessaries)<br>10 Topical - cream<br>11 Topical - drops (e.g. eye drops)<br>12 Other<br>99 Don't know |
| q404_M90c *(required)* | q404_M90c. What is the item's smallest unit of measurement (mg, mcg, ml, g, etc)?<br>*Question relevant when: selected( ${q104_M90_full_list} , '3')* | microgram — microgram (mcg)<br>miligram — miligram (mg)<br>miligram_dissolved_in_liquid — miligram (mg) dissolved in liquid (e.g. 2 mg per 3 militers)<br>gram — gram (g)<br>militer — militer (ml)<br>centiliter — centiliter (cl)<br>litre — litre (l)<br>ml_of _solution — ml of solution (e.g. ml of 10% solution)<br>litre_of_solution — litre of solution (e.g. 1 litre of 5% solution)<br>cubic_meter — cubic meter (m^3)<br>other — other<br>dont_know — don't know |
| q404_M90c.1 | q404_M90c.1 If other unit of measurement or route of administration, specify here:<br>*Leave blank if not applicable*<br>*Question relevant when: selected( ${q104_M90_full_list} , '3')* | |
| q404_M90d *(required)* | q404_M90d. In total, how many units are used to treat a patient during all of her care?<br>*Remember to add all units! E.g. 5 days x 200 mg twice per day = 2,000mg*<br>*Question relevant when: selected( ${q104_M90_full_list} , '3')*<br>*Response constrained to: .>0* | |

| Field | Question | Answer |
|---|---|---|
| q404_M91 | ***q404_M91. Saline, glucose, water, etc. - Other 1: "[q104_M91_full_list_other]"***<br>*Question relevant when: selected( ${q104_M91_full_list} , '3')* | |
| q404_M91a *(required)* | q404_M91a. What percent of patients require this item?<br>*Question relevant when: selected( ${q104_M91_full_list} , '3')*<br>*Response constrained to: .>0 and .<=100 or .=999* | |
| q404_M91b *(required)* | q404_M91b. How is the item administered, or given, to the patient?<br>*Question relevant when: selected( ${q104_M91_full_list} , '3')* | 1 Oral - solid (e.g. tablet)<br>2 Oral - liquid<br>3 Sublingual<br>4 Intravenous (IV)<br>5 Intramusculuar injection (IM)<br>6 Subcutaneous<br>7 Rectal<br>8 Inhaled (e.g. gas)<br>9 Inserted (e.g. vaginal pessaries)<br>10 Topical - cream<br>11 Topical - drops (e.g. eye drops)<br>12 Other<br>99 Don't know |
| q404_M91c *(required)* | q404_M91c. What is the item's smallest unit of measurement (mg, mcg, ml, g, etc)?<br>*Question relevant when: selected( ${q104_M91_full_list} , '3')* | microgram — microgram (mcg)<br>miligram — miligram (mg)<br>miligram_dissolved_in_liquid — miligram (mg) dissolved in liquid (e.g. 2 mg per 3 militers)<br>gram — gram (g)<br>militer — militer (ml)<br>centiliter — centiliter (cl)<br>litre — litre (l)<br>ml_of _solution — ml of solution (e.g. ml of 10% solution)<br>litre_of_solution — litre of solution (e.g. 1 litre of 5% solution)<br>cubic_meter — cubic meter (m^3)<br>other — other<br>dont_know — don't know |
| q404_M91c.1 | q404_M91c.1 If other unit of measurement or route of administration, specify here:<br>*Leave blank if not applicable*<br>*Question relevant when: selected( ${q104_M91_full_list} , '3')* | |
| q404_M91d *(required)* | q404_M91d. In total, how many units are used to treat a patient during all of her care?<br>*Remember to add all units! E.g. 5 days x 200 mg twice per day = 2,000mg*<br>*Question relevant when: selected( ${q104_M91_full_list} , '3')*<br>*Response constrained to: .>0* | |

| Field | Question | Answer |
|---|---|---|
| q404_M92 | ***q404_M92. Saline, glucose, water, etc. - Other 2: "[q104_M92_full_list_other]"***<br>*Question relevant when: selected( ${q104_M92_full_list} , '3')* | |
| q404_M92a *(required)* | q404_M92a. What percent of patients require this item?<br>*Question relevant when: selected( ${q104_M92_full_list} , '3')*<br>*Response constrained to: .>0 and .<=100 or .=999* | |
| q404_M92b *(required)* | q404_M92b. How is the item administered, or given, to the patient?<br>*Question relevant when: selected( ${q104_M92_full_list} , '3')* | 1 Oral - solid (e.g. tablet)<br>2 Oral - liquid<br>3 Sublingual<br>4 Intravenous (IV)<br>5 Intramusculuar injection (IM)<br>6 Subcutaneous<br>7 Rectal<br>8 Inhaled (e.g. gas)<br>9 Inserted (e.g. vaginal pessaries)<br>10 Topical - cream<br>11 Topical - drops (e.g. eye drops)<br>12 Other<br>99 Don't know |
| q404_M92c *(required)* | q404_M92c. What is the item's smallest unit of measurement (mg, mcg, ml, g, etc)?<br>*Question relevant when: selected( ${q104_M92_full_list} , '3')* | microgram — microgram (mcg)<br>miligram — miligram (mg)<br>miligram_dissolved_in_liquid — miligram (mg) dissolved in liquid (e.g. 2 mg per 3 militers)<br>gram — gram (g)<br>militer — militer (ml)<br>centiliter — centiliter (cl)<br>litre — litre (l)<br>ml_of _solution — ml of solution (e.g. ml of 10% solution)<br>litre_of_solution — litre of solution (e.g. 1 litre of 5% solution)<br>cubic_meter — cubic meter (m^3)<br>other — other<br>dont_know — don't know |
| q404_M92c.1 | q404_M92c.1 If other unit of measurement or route of administration, specify here:<br>*Leave blank if not applicable*<br>*Question relevant when: selected( ${q104_M92_full_list} , '3')* | |
| q404_M92d *(required)* | q404_M92d. In total, how many units are used to treat a patient during all of her care?<br>*Remember to add all units! E.g. 5 days x 200 mg twice per day = 2,000mg*<br>*Question relevant when: selected( ${q104_M92_full_list} , '3')*<br>*Response constrained to: .>0* | |

| Field | Question | Answer |
|---|---|---|
| q404_M93 | ***q404_M93. Saline, glucose, water, etc. - Other 3: "[q104_M93_full_list_other]"*** *Question relevant when: selected( ${q104_M93_full_list} , '3')* | |
| q404_M93a *(required)* | q404_M93a. What percent of patients require this item? *Question relevant when: selected( ${q104_M93_full_list} , '3')* *Response constrained to: .>0 and .<=100 or .=999* | |

| Field | Question | Answer | |
|---|---|---|---|
| q404_M93b *(required)* | q404_M93b. How is the item administered, or given, to the patient? *Question relevant when: selected( ${q104_M93_full_list} , '3')* | 1 | Oral - solid (e.g. tablet) |
| | | 2 | Oral - liquid |
| | | 3 | Sublingual |
| | | 4 | Intravenous (IV) |
| | | 5 | Intramusculuar injection (IM) |
| | | 6 | Subcutaneous |
| | | 7 | Rectal |
| | | 8 | Inhaled (e.g. gas) |
| | | 9 | Inserted (e.g. vaginal pessaries) |
| | | 10 | Topical - cream |
| | | 11 | Topical - drops (e.g. eye drops) |
| | | 12 | Other |
| | | 99 | Don't know |
| q404_M93c *(required)* | q404_M93c. What is the item's smallest unit of measurement (mg, mcg, ml, g, etc)? *Question relevant when: selected( ${q104_M93_full_list} , '3')* | microgram | microgram (mcg) |
| | | miligram | miligram (mg) |
| | | miligram_dissolved_in_liquid | miligram (mg) dissolved in liquid (e.g. 2 mg per 3 militers) |
| | | gram | gram (g) |
| | | militer | militer (ml) |
| | | centiliter | centiliter (cl) |
| | | litre | litre (l) |
| | | ml_of _solution | ml of solution (e.g. ml of 10% solution) |
| | | litre_of_solution | litre of solution (e.g. 1 litre of 5% solution) |
| | | cubic_meter | cubic meter (m^3) |
| | | other | other |
| | | dont_know | don't know |
| q404_M93c.1 | q404_M93c.1 If other unit of measurement or route of administration, specify here: *Leave blank if not applicable* *Question relevant when: selected( ${q104_M93_full_list} , '3')* | | |
| q404_M93d *(required)* | q404_M93d. In total, how many units are used to treat a patient during all of her care? *Remember to add all units! E.g. 5 days x 200 mg twice per day = 2,000mg* *Question relevant when: selected( ${q104_M93_full_list} , '3')* *Response constrained to: .>0* | | |

| Field | Question | Answer |
|---|---|---|
| q404_M94 | ***q404_M94. Saline, glucose, water, etc. - Other 4: "[q104_M94_full_list_other]"*** *Question relevant when: selected( ${q104_M94_full_list} , '3')* | |
| q404_M94a *(required)* | q404_M94a. What percent of patients require this item? *Question relevant when: selected( ${q104_M94_full_list} , '3')* *Response constrained to: .>0 and .<=100 or .=999* | |
| q404_M94b *(required)* | q404_M94b. How is the item administered, or given, to the patient? *Question relevant when: selected( ${q104_M94_full_list} , '3')* | 1 Oral - solid (e.g. tablet)<br>2 Oral - liquid<br>3 Sublingual<br>4 Intravenous (IV)<br>5 Intramusculuar injection (IM)<br>6 Subcutaneous<br>7 Rectal<br>8 Inhaled (e.g. gas)<br>9 Inserted (e.g. vaginal pessaries)<br>10 Topical - cream<br>11 Topical - drops (e.g. eye drops)<br>12 Other<br>99 Don't know |
| q404_M94c *(required)* | q404_M94c. What is the item's smallest unit of measurement (mg, mcg, ml, g, etc)? *Question relevant when: selected( ${q104_M94_full_list} , '3')* | microgram: microgram (mcg)<br>miligram: miligram (mg)<br>miligram_dissolved_in_liquid: miligram (mg) dissolved in liquid (e.g. 2 mg per 3 militers)<br>gram: gram (g)<br>militer: militer (ml)<br>centiliter: centiliter (cl)<br>litre: litre (l)<br>ml_of _solution: ml of solution (e.g. ml of 10% solution)<br>litre_of_solution: litre of solution (e.g. 1 litre of 5% solution)<br>cubic_meter: cubic meter (m^3)<br>other: other<br>dont_know: don't know |
| q404_M94c.1 | q404_M94c.1 If other unit of measurement or route of administration, specify here: *Leave blank if not applicable* *Question relevant when: selected( ${q104_M94_full_list} , '3')* | |
| q404_M94d *(required)* | q404_M94d. In total, how many units are used to treat a patient during all of her care? *Remember to add all units! E.g. 5 days x 200 mg twice per day = 2,000mg* *Question relevant when: selected( ${q104_M94_full_list} , '3')* *Response constrained to: .>0* | |

| Field | Question | Answer |
|---|---|---|
| q404_M95 | ***q404_M95. Saline, glucose, water, etc. - Other 5: "[q104_M95_full_list_other]"***<br>*Question relevant when: selected( ${q104_M95_full_list} , '3')* | |
| q404_M95a *(required)* | q404_M95a. What percent of patients require this item?<br>*Question relevant when: selected( ${q104_M95_full_list} , '3')*<br>*Response constrained to: .>0 and .<=100 or .=999* | |
| q404_M95b *(required)* | q404_M95b. How is the item administered, or given, to the patient?<br>*Question relevant when: selected( ${q104_M95_full_list} , '3')* | 1 Oral - solid (e.g. tablet)<br>2 Oral - liquid<br>3 Sublingual<br>4 Intravenous (IV)<br>5 Intramusculuar injection (IM)<br>6 Subcutaneous<br>7 Rectal<br>8 Inhaled (e.g. gas)<br>9 Inserted (e.g. vaginal pessaries)<br>10 Topical - cream<br>11 Topical - drops (e.g. eye drops)<br>12 Other<br>99 Don't know |
| q404_M95c *(required)* | q404_M95c. What is the item's smallest unit of measurement (mg, mcg, ml, g, etc)?<br>*Question relevant when: selected( ${q104_M95_full_list} , '3')* | microgram — microgram (mcg)<br>miligram — miligram (mg)<br>miligram_dissolved_in_liquid — miligram (mg) dissolved in liquid (e.g. 2 mg per 3 militers)<br>gram — gram (g)<br>militer — militer (ml)<br>centiliter — centiliter (cl)<br>litre — litre (l)<br>ml_of _solution — ml of solution (e.g. ml of 10% solution)<br>litre_of_solution — litre of solution (e.g. 1 litre of 5% solution)<br>cubic_meter — cubic meter (m^3)<br>other — other<br>dont_know — don't know |
| q404_M95c.1 | q404_M95c.1 If other unit of measurement or route of administration, specify here:<br>*Leave blank if not applicable*<br>*Question relevant when: selected( ${q104_M95_full_list} , '3')* | |
| q404_M95d *(required)* | q404_M95d. In total, how many units are used to treat a patient during all of her care?<br>*Remember to add all units! E.g. 5 days x 200 mg twice per day = 2,000mg*<br>*Question relevant when: selected( ${q104_M95_full_list} , '3')*<br>*Response constrained to: .>0* | |

<p><strong><em>D. Medications - Shock (10)</em></strong></p>

*Group relevant when: selected( ${section_four_skip_med} , '1')*

| Field | Question | Answer |
|---|---|---|
| q404_med_notE9 | 404 CONTINUED For each of the following medications and other medical products that are used for shock, can you tell me …? | |
| note_404_prost_utero | ***Prostaglandin, uterotonic, etc.*** | |
| q404_M96 | ***q404_M96. Misoprostol***<br>*Question relevant when: selected( ${q104_M96_full_list} , '3')* | |
| q404_M96a *(required)* | q404_M96a. What percent of patients require this item?<br>*Question relevant when: selected( ${q104_M96_full_list} , '3')*<br>*Response constrained to: .>0 and .<=100 or .=999* | |

| Field | Question | Answer | | |
|---|---|---|---|---|
| q404_M96b *(required)* | q404_M96b. How is the item administered, or given, to the patient?<br>*Question relevant when: selected( ${q104_M96_full_list} , '3')* | | 1 | Oral - solid (e.g. tablet) |
| | | | 2 | Oral - liquid |
| | | | 3 | Sublingual |
| | | | 4 | Intravenous (IV) |
| | | | 5 | Intramusculuar injection (IM) |
| | | | 6 | Subcutaneous |
| | | | 7 | Rectal |
| | | | 8 | Inhaled (e.g. gas) |
| | | | 9 | Inserted (e.g. vaginal pessaries) |
| | | | 10 | Topical - cream |
| | | | 11 | Topical - drops (e.g. eye drops) |
| | | | 12 | Other |
| | | | 99 | Don't know |
| q404_M96c *(required)* | q404_M96c. What is the item's smallest unit of measurement (mg, mcg, ml, g, etc)?<br>*Question relevant when: selected( ${q104_M96_full_list} , '3')* | | microgram | microgram (mcg) |
| | | | miligram | miligram (mg) |
| | | | miligram_dissolved_in_liquid | miligram (mg) dissolved in liquid (e.g. 2 mg per 3 militers) |
| | | | gram | gram (g) |
| | | | militer | militer (ml) |
| | | | centiliter | centiliter (cl) |
| | | | litre | litre (l) |
| | | | ml_of _solution | ml of solution (e.g. ml of 10% solution) |
| | | | litre_of_solution | litre of solution (e.g. 1 litre of 5% solution) |
| | | | cubic_meter | cubic meter (m^3) |
| | | | other | other |
| | | | dont_know | don't know |
| q404_M96c.1 | q404_M96c.1 If other unit of measurement or route of administration, specify here:<br>*Leave blank if not applicable*<br>*Question relevant when: selected( ${q104_M96_full_list} , '3')* | | | |
| q404_M96d *(required)* | q404_M96d. In total, how many units are used to treat a patient during all of her care?<br>*Remember to add all units! E.g. 5 days x 200 mg twice per day = 2,000mg*<br>*Question relevant when: selected( ${q104_M96_full_list} , '3')*<br>*Response constrained to: .>0* | | | |

| Field | Question | Answer |
|---|---|---|
| q404_M97 | ***q404_M97. Ergometrine***<br>*Question relevant when: selected( ${q104_M97_full_list} , '3')* | |
| q404_M97a *(required)* | q404_M97a. What percent of patients require this item?<br>*Question relevant when: selected( ${q104_M97_full_list} , '3')*<br>*Response constrained to: .>0 and .<=100 or .=999* | |
| q404_M97b *(required)* | q404_M97b. How is the item administered, or given, to the patient?<br>*Question relevant when: selected( ${q104_M97_full_list} , '3')* | 1 Oral - solid (e.g. tablet)<br>2 Oral - liquid<br>3 Sublingual<br>4 Intravenous (IV)<br>5 Intramusculuar injection (IM)<br>6 Subcutaneous<br>7 Rectal<br>8 Inhaled (e.g. gas)<br>9 Inserted (e.g. vaginal pessaries)<br>10 Topical - cream<br>11 Topical - drops (e.g. eye drops)<br>12 Other<br>99 Don't know |
| q404_M97c *(required)* | q404_M97c. What is the item's smallest unit of measurement (mg, mcg, ml, g, etc)?<br>*Question relevant when: selected( ${q104_M97_full_list} , '3')* | microgram — microgram (mcg)<br>miligram — miligram (mg)<br>miligram_dissolved_in_liquid — miligram (mg) dissolved in liquid (e.g. 2 mg per 3 militers)<br>gram — gram (g)<br>militer — militer (ml)<br>centiliter — centiliter (cl)<br>litre — litre (l)<br>ml_of _solution — ml of solution (e.g. ml of 10% solution)<br>litre_of_solution — litre of solution (e.g. 1 litre of 5% solution)<br>cubic_meter — cubic meter (m^3)<br>other — other<br>dont_know — don't know |
| q404_M97c.1 | q404_M97c.1 If other unit of measurement or route of administration, specify here:<br>*Leave blank if not applicable*<br>*Question relevant when: selected( ${q104_M97_full_list} , '3')* | |
| q404_M97d *(required)* | q404_M97d. In total, how many units are used to treat a patient during all of her care?<br>*Remember to add all units! E.g. 5 days x 200 mg twice per day = 2,000mg*<br>*Question relevant when: selected( ${q104_M97_full_list} , '3')*<br>*Response constrained to: .>0* | |

| Field | Question | Answer |
|---|---|---|
| q404_M98 | ***q404_M98. Methylergonovine Maleate (Methergine)***<br>*Question relevant when: selected( ${q104_M98_full_list} , '3')* | |
| q404_M98a *(required)* | q404_M98a. What percent of patients require this item?<br>*Question relevant when: selected( ${q104_M98_full_list} , '3')*<br>*Response constrained to: .>0 and .<=100 or .=999* | |
| q404_M98b *(required)* | q404_M98b. How is the item administered, or given, to the patient?<br>*Question relevant when: selected( ${q104_M98_full_list} , '3')* | 1 Oral - solid (e.g. tablet)<br>2 Oral - liquid<br>3 Sublingual<br>4 Intravenous (IV)<br>5 Intramusculuar injection (IM)<br>6 Subcutaneous<br>7 Rectal<br>8 Inhaled (e.g. gas)<br>9 Inserted (e.g. vaginal pessaries)<br>10 Topical - cream<br>11 Topical - drops (e.g. eye drops)<br>12 Other<br>99 Don't know |
| q404_M98c *(required)* | q404_M98c. What is the item's smallest unit of measurement (mg, mcg, ml, g, etc)?<br>*Question relevant when: selected( ${q104_M98_full_list} , '3')* | microgram — microgram (mcg)<br>miligram — miligram (mg)<br>miligram_dissolved_in_liquid — miligram (mg) dissolved in liquid (e.g. 2 mg per 3 militers)<br>gram — gram (g)<br>militer — militer (ml)<br>centiliter — centiliter (cl)<br>litre — litre (l)<br>ml_of _solution — ml of solution (e.g. ml of 10% solution)<br>litre_of_solution — litre of solution (e.g. 1 litre of 5% solution)<br>cubic_meter — cubic meter (m^3)<br>other — other<br>dont_know — don't know |
| q404_M98c.1 | q404_M98c.1 If other unit of measurement or route of administration, specify here:<br>*Leave blank if not applicable*<br>*Question relevant when: selected( ${q104_M98_full_list} , '3')* | |
| q404_M98d *(required)* | q404_M98d. In total, how many units are used to treat a patient during all of her care?<br>*Remember to add all units! E.g. 5 days x 200 mg twice per day = 2,000mg*<br>*Question relevant when: selected( ${q104_M98_full_list} , '3')*<br>*Response constrained to: .>0* | |

| Field | Question | Answer |
|---|---|---|
| q404_M99 | ***q404_M99. Mifepristone***<br>*Question relevant when: selected( ${q104_M99_full_list} , '3')* | |
| q404_M99a *(required)* | q404_M99a. What percent of patients require this item?<br>*Question relevant when: selected( ${q104_M99_full_list} , '3')*<br>*Response constrained to: .>0 and .<=100 or .=999* | |
| q404_M99b *(required)* | q404_M99b. How is the item administered, or given, to the patient?<br>*Question relevant when: selected( ${q104_M99_full_list} , '3')* | 1 Oral - solid (e.g. tablet)<br>2 Oral - liquid<br>3 Sublingual<br>4 Intravenous (IV)<br>5 Intramusculuar injection (IM)<br>6 Subcutaneous<br>7 Rectal<br>8 Inhaled (e.g. gas)<br>9 Inserted (e.g. vaginal pessaries)<br>10 Topical - cream<br>11 Topical - drops (e.g. eye drops)<br>12 Other<br>99 Don't know |
| q404_M99c *(required)* | q404_M99c. What is the item's smallest unit of measurement (mg, mcg, ml, g, etc)?<br>*Question relevant when: selected( ${q104_M99_full_list} , '3')* | microgram — microgram (mcg)<br>miligram — miligram (mg)<br>miligram_dissolved_in_liquid — miligram (mg) dissolved in liquid (e.g. 2 mg per 3 militers)<br>gram — gram (g)<br>militer — militer (ml)<br>centiliter — centiliter (cl)<br>litre — litre (l)<br>ml_of _solution — ml of solution (e.g. ml of 10% solution)<br>litre_of_solution — litre of solution (e.g. 1 litre of 5% solution)<br>cubic_meter — cubic meter (m^3)<br>other — other<br>dont_know — don't know |
| q404_M99c.1 | q404_M99c.1 If other unit of measurement or route of administration, specify here:<br>*Leave blank if not applicable*<br>*Question relevant when: selected( ${q104_M99_full_list} , '3')* | |
| q404_M99d *(required)* | q404_M99d. In total, how many units are used to treat a patient during all of her care?<br>*Remember to add all units! E.g. 5 days x 200 mg twice per day = 2,000mg*<br>*Question relevant when: selected( ${q104_M99_full_list} , '3')*<br>*Response constrained to: .>0* | |

| Field | Question | Answer |
|---|---|---|
| q404_M100 | ***q404_M100. Oxytocin***<br>*Question relevant when: selected( ${q104_M100_full_list} , '3')* | |
| q404_M100a *(required)* | q404_M100a. What percent of patients require this item?<br>*Question relevant when: selected( ${q104_M100_full_list} , '3')*<br>*Response constrained to: .>0 and .<=100 or .=999* | |
| q404_M100b *(required)* | q404_M100b. How is the item administered, or given, to the patient?<br>*Question relevant when: selected( ${q104_M100_full_list} , '3')* | 1 Oral - solid (e.g. tablet)<br>2 Oral - liquid<br>3 Sublingual<br>4 Intravenous (IV)<br>5 Intramusculuar injection (IM)<br>6 Subcutaneous<br>7 Rectal<br>8 Inhaled (e.g. gas)<br>9 Inserted (e.g. vaginal pessaries)<br>10 Topical - cream<br>11 Topical - drops (e.g. eye drops)<br>12 Other<br>99 Don't know |
| q404_M100c *(required)* | q404_M100c. What is the item's smallest unit of measurement (mg, mcg, ml, g, etc)?<br>*Question relevant when: selected( ${q104_M100_full_list} , '3')* | microgram — microgram (mcg)<br>miligram — miligram (mg)<br>miligram_dissolved_in_liquid — miligram (mg) dissolved in liquid (e.g. 2 mg per 3 militers)<br>gram — gram (g)<br>militer — militer (ml)<br>centiliter — centiliter (cl)<br>litre — litre (l)<br>ml_of _solution — ml of solution (e.g. ml of 10% solution)<br>litre_of_solution — litre of solution (e.g. 1 litre of 5% solution)<br>cubic_meter — cubic meter (m^3)<br>other — other<br>dont_know — don't know |
| q404_M100c.1 | q404_M100c.1 If other unit of measurement or route of administration, specify here:<br>*Leave blank if not applicable*<br>*Question relevant when: selected( ${q104_M100_full_list} , '3')* | |
| q404_M100d *(required)* | q404_M100d. In total, how many units are used to treat a patient during all of her care?<br>*Remember to add all units! E.g. 5 days x 200 mg twice per day = 2,000mg*<br>*Question relevant when: selected( ${q104_M100_full_list} , '3')*<br>*Response constrained to: .>0* | |

| Field | Question | Answer |
|---|---|---|
| q404_M101 | ***q404_M101. Prostaglandin, uterotonic, etc. - Other 1: "[q104_M101_full_list_other]"***<br>*Question relevant when: selected( ${q104_M101_full_list} , '3')* | |
| q404_M101a *(required)* | q404_M101a. What percent of patients require this item?<br>*Question relevant when: selected( ${q104_M101_full_list} , '3')*<br>*Response constrained to: .>0 and .<=100 or .=999* | |
| q404_M101b *(required)* | q404_M101b. How is the item administered, or given, to the patient?<br>*Question relevant when: selected( ${q104_M101_full_list} , '3')* | 1 Oral - solid (e.g. tablet)<br>2 Oral - liquid<br>3 Sublingual<br>4 Intravenous (IV)<br>5 Intramusculuar injection (IM)<br>6 Subcutaneous<br>7 Rectal<br>8 Inhaled (e.g. gas)<br>9 Inserted (e.g. vaginal pessaries)<br>10 Topical - cream<br>11 Topical - drops (e.g. eye drops)<br>12 Other<br>99 Don't know |
| q404_M101c *(required)* | q404_M101c. What is the item's smallest unit of measurement (mg, mcg, ml, g, etc)?<br>*Question relevant when: selected( ${q104_M101_full_list} , '3')* | microgram — microgram (mcg)<br>miligram — miligram (mg)<br>miligram_dissolved_in_liquid — miligram (mg) dissolved in liquid (e.g. 2 mg per 3 militers)<br>gram — gram (g)<br>militer — militer (ml)<br>centiliter — centiliter (cl)<br>litre — litre (l)<br>ml_of _solution — ml of solution (e.g. ml of 10% solution)<br>litre_of_solution — litre of solution (e.g. 1 litre of 5% solution)<br>cubic_meter — cubic meter (m^3)<br>other — other<br>dont_know — don't know |
| q404_M101c.1 | q404_M101c.1 If other unit of measurement or route of administration, specify here:<br>*Leave blank if not applicable*<br>*Question relevant when: selected( ${q104_M101_full_list} , '3')* | |
| q404_M101d *(required)* | q404_M101d. In total, how many units are used to treat a patient during all of her care?<br>*Remember to add all units! E.g. 5 days x 200 mg twice per day = 2,000mg*<br>*Question relevant when: selected( ${q104_M101_full_list} , '3')*<br>*Response constrained to: .>0* | |

| Field | Question | Answer |
|---|---|---|
| q404_M102 | ***q404_M102. Prostaglandin, uterotonic, etc. - Other 2: "[q104_M102_full_list_other]"***<br>*Question relevant when: selected( ${q104_M102_full_list} , '3')* | |
| q404_M102a *(required)* | q404_M102a. What percent of patients require this item?<br>*Question relevant when: selected( ${q104_M102_full_list} , '3')*<br>*Response constrained to: .>0 and .<=100 or .=999* | |
| q404_M102b *(required)* | q404_M102b. How is the item administered, or given, to the patient?<br>*Question relevant when: selected( ${q104_M102_full_list} , '3')* | 1 Oral - solid (e.g. tablet)<br>2 Oral - liquid<br>3 Sublingual<br>4 Intravenous (IV)<br>5 Intramusculuar injection (IM)<br>6 Subcutaneous<br>7 Rectal<br>8 Inhaled (e.g. gas)<br>9 Inserted (e.g. vaginal pessaries)<br>10 Topical - cream<br>11 Topical - drops (e.g. eye drops)<br>12 Other<br>99 Don't know |
| q404_M102c *(required)* | q404_M102c. What is the item's smallest unit of measurement (mg, mcg, ml, g, etc)?<br>*Question relevant when: selected( ${q104_M102_full_list} , '3')* | microgram — microgram (mcg)<br>miligram — miligram (mg)<br>miligram_dissolved_in_liquid — miligram (mg) dissolved in liquid (e.g. 2 mg per 3 militers)<br>gram — gram (g)<br>militer — militer (ml)<br>centiliter — centiliter (cl)<br>litre — litre (l)<br>ml_of _solution — ml of solution (e.g. ml of 10% solution)<br>litre_of_solution — litre of solution (e.g. 1 litre of 5% solution)<br>cubic_meter — cubic meter (m^3)<br>other — other<br>dont_know — don't know |
| q404_M102c.1 | q404_M102c.1 If other unit of measurement or route of administration, specify here:<br>*Leave blank if not applicable*<br>*Question relevant when: selected( ${q104_M102_full_list} , '3')* | |
| q404_M102d *(required)* | q404_M102d. In total, how many units are used to treat a patient during all of her care?<br>*Remember to add all units! E.g. 5 days x 200 mg twice per day = 2,000mg*<br>*Question relevant when: selected( ${q104_M102_full_list} , '3')*<br>*Response constrained to: .>0* | |

| Field | Question | Answer |
|---|---|---|
| q404_M103 | ***q404_M103. Prostaglandin, uterotonic, etc. - Other 3: "[q104_M103_full_list_other]"***<br>*Question relevant when: selected( ${q104_M104_full_list} , '3')* | |
| q404_M103a *(required)* | q404_M103a. What percent of patients require this item?<br>*Question relevant when: selected( ${q104_M104_full_list} , '3')*<br>*Response constrained to: .>0 and .<=100 or .=999* | |
| q404_M103b *(required)* | q404_M103b. How is the item administered, or given, to the patient?<br>*Question relevant when: selected( ${q104_M104_full_list} , '3')* | 1 Oral - solid (e.g. tablet)<br>2 Oral - liquid<br>3 Sublingual<br>4 Intravenous (IV)<br>5 Intramusculuar injection (IM)<br>6 Subcutaneous<br>7 Rectal<br>8 Inhaled (e.g. gas)<br>9 Inserted (e.g. vaginal pessaries)<br>10 Topical - cream<br>11 Topical - drops (e.g. eye drops)<br>12 Other<br>99 Don't know |
| q404_M103c *(required)* | q404_M103c. What is the item's smallest unit of measurement (mg, mcg, ml, g, etc)?<br>*Question relevant when: selected( ${q104_M104_full_list} , '3')* | microgram — microgram (mcg)<br>miligram — miligram (mg)<br>miligram_dissolved_in_liquid — miligram (mg) dissolved in liquid (e.g. 2 mg per 3 militers)<br>gram — gram (g)<br>militer — militer (ml)<br>centiliter — centiliter (cl)<br>litre — litre (l)<br>ml_of _solution — ml of solution (e.g. ml of 10% solution)<br>litre_of_solution — litre of solution (e.g. 1 litre of 5% solution)<br>cubic_meter — cubic meter (m^3)<br>other — other<br>dont_know — don't know |
| q404_M103c.1 | q404_M103c.1 If other unit of measurement or route of administration, specify here:<br>*Leave blank if not applicable*<br>*Question relevant when: selected( ${q104_M104_full_list} , '3')* | |
| q404_M103d *(required)* | q404_M103d. In total, how many units are used to treat a patient during all of her care?<br>*Remember to add all units! E.g. 5 days x 200 mg twice per day = 2,000mg*<br>*Question relevant when: selected( ${q104_M104_full_list} , '3')*<br>*Response constrained to: .>0* | |

| Field | Question | Answer |
|---|---|---|
| q404_M104 | ***q404_M104. Prostaglandin, uterotonic, etc. - Other 4: "[q104_M104_full_list_other]"***<br>*Question relevant when: selected( ${q104_M104_full_list} , '3')* | |
| q404_M104a *(required)* | q404_M104a. What percent of patients require this item?<br>*Question relevant when: selected( ${q104_M104_full_list} , '3')*<br>*Response constrained to: .>0 and .<=100 or .=999* | |
| q404_M104b *(required)* | q404_M104b. How is the item administered, or given, to the patient?<br>*Question relevant when: selected( ${q104_M104_full_list} , '3')* | 1 Oral - solid (e.g. tablet)<br>2 Oral - liquid<br>3 Sublingual<br>4 Intravenous (IV)<br>5 Intramusculuar injection (IM)<br>6 Subcutaneous<br>7 Rectal<br>8 Inhaled (e.g. gas)<br>9 Inserted (e.g. vaginal pessaries)<br>10 Topical - cream<br>11 Topical - drops (e.g. eye drops)<br>12 Other<br>99 Don't know |
| q404_M104c *(required)* | q404_M104c. What is the item's smallest unit of measurement (mg, mcg, ml, g, etc)?<br>*Question relevant when: selected( ${q104_M104_full_list} , '3')* | microgram — microgram (mcg)<br>miligram — miligram (mg)<br>miligram_dissolved_in_liquid — miligram (mg) dissolved in liquid (e.g. 2 mg per 3 militers)<br>gram — gram (g)<br>militer — militer (ml)<br>centiliter — centiliter (cl)<br>litre — litre (l)<br>ml_of _solution — ml of solution (e.g. ml of 10% solution)<br>litre_of_solution — litre of solution (e.g. 1 litre of 5% solution)<br>cubic_meter — cubic meter (m^3)<br>other — other<br>dont_know — don't know |
| q404_M104c.1 | q404_M104c.1 If other unit of measurement or route of administration, specify here:<br>*Leave blank if not applicable*<br>*Question relevant when: selected( ${q104_M104_full_list} , '3')* | |
| q404_M104d *(required)* | q404_M104d. In total, how many units are used to treat a patient during all of her care?<br>*Remember to add all units! E.g. 5 days x 200 mg twice per day = 2,000mg*<br>*Question relevant when: selected( ${q104_M104_full_list} , '3')*<br>*Response constrained to: .>0* | |

| Field | Question | Answer |
|---|---|---|
| q404_M105 | ***q404_M105. Prostaglandin, uterotonic, etc. - Other 5: "[q104_M105_full_list_other]"*** <br> *Question relevant when: selected( ${q104_M105_full_list} , '3')* | |
| q404_M105a *(required)* | q404_M105a. What percent of patients require this item? <br> *Question relevant when: selected( ${q104_M105_full_list} , '3')* <br> *Response constrained to: .>0 and .<=100 or .=999* | |
| q404_M105b *(required)* | q404_M105b. How is the item administered, or given, to the patient? <br> *Question relevant when: selected( ${q104_M105_full_list} , '3')* | 1 Oral - solid (e.g. tablet) <br> 2 Oral - liquid <br> 3 Sublingual <br> 4 Intravenous (IV) <br> 5 Intramusculuar injection (IM) <br> 6 Subcutaneous <br> 7 Rectal <br> 8 Inhaled (e.g. gas) <br> 9 Inserted (e.g. vaginal pessaries) <br> 10 Topical - cream <br> 11 Topical - drops (e.g. eye drops) <br> 12 Other <br> 99 Don't know |
| q404_M105c *(required)* | q404_M105c. What is the item's smallest unit of measurement (mg, mcg, ml, g, etc)? <br> *Question relevant when: selected( ${q104_M105_full_list} , '3')* | microgram — microgram (mcg) <br> miligram — miligram (mg) <br> miligram_dissolved_in_liquid — miligram (mg) dissolved in liquid (e.g. 2 mg per 3 militers) <br> gram — gram (g) <br> militer — militer (ml) <br> centiliter — centiliter (cl) <br> litre — litre (l) <br> ml_of _solution — ml of solution (e.g. ml of 10% solution) <br> litre_of_solution — litre of solution (e.g. 1 litre of 5% solution) <br> cubic_meter — cubic meter (m^3) <br> other — other <br> dont_know — don't know |
| q404_M105c.1 | q404_M105c.1 If other unit of measurement or route of administration, specify here: <br> *Leave blank if not applicable* <br> *Question relevant when: selected( ${q104_M105_full_list} , '3')* | |
| q404_M105d *(required)* | q404_M105d. In total, how many units are used to treat a patient during all of her care? <br> *Remember to add all units! E.g. 5 days x 200 mg twice per day = 2,000mg* <br> *Question relevant when: selected( ${q104_M105_full_list} , '3')* <br> *Response constrained to: .>0* | |

<p><strong><em>D. Medications - Shock (11)</em></strong></p>

*Group relevant when: selected( ${section_four_skip_med} , '1')*

| Field | Question | Answer |
|---|---|---|
| q404_med_notE10 | 404 CONTINUED For each of the following medications and other medical products that are used for shock, can you tell me …? | |
| note_404_vitamin | ***Vitamin, immunological, etc.*** | |
| q404_M106 | ***q404_M106. Ferrous sulphate (tablets)***<br>*Question relevant when: selected( ${q104_M106_full_list} , '3')* | |
| q404_M106a *(required)* | q404_M106a. What percent of patients require this item?<br>*Question relevant when: selected( ${q104_M106_full_list} , '3')*<br>*Response constrained to: .>0 and .<=100 or .=999* | |

| Field | Question | Answer | | |
|---|---|---|---|---|
| q404_M106b *(required)* | q404_M106b. How is the item administered, or given, to the patient?<br>*Question relevant when: selected( ${q104_M106_full_list} , '3')* | | 1 | Oral - solid (e.g. tablet) |
| | | | 2 | Oral - liquid |
| | | | 3 | Sublingual |
| | | | 4 | Intravenous (IV) |
| | | | 5 | Intramusculuar injection (IM) |
| | | | 6 | Subcutaneous |
| | | | 7 | Rectal |
| | | | 8 | Inhaled (e.g. gas) |
| | | | 9 | Inserted (e.g. vaginal pessaries) |
| | | | 10 | Topical - cream |
| | | | 11 | Topical - drops (e.g. eye drops) |
| | | | 12 | Other |
| | | | 99 | Don't know |
| q404_M106c *(required)* | q404_M106c. What is the item's smallest unit of measurement (mg, mcg, ml, g, etc)?<br>*Question relevant when: selected( ${q104_M106_full_list} , '3')* | | microgram | microgram (mcg) |
| | | | miligram | miligram (mg) |
| | | | miligram_dissolved_in_liquid | miligram (mg) dissolved in liquid (e.g. 2 mg per 3 militers) |
| | | | gram | gram (g) |
| | | | militer | militer (ml) |
| | | | centiliter | centiliter (cl) |
| | | | litre | litre (l) |
| | | | ml_of _solution | ml of solution (e.g. ml of 10% solution) |
| | | | litre_of_solution | litre of solution (e.g. 1 litre of 5% solution) |
| | | | cubic_meter | cubic meter (m^3) |
| | | | other | other |
| | | | dont_know | don't know |
| q404_M106c.1 | q404_M106c.1 If other unit of measurement or route of administration, specify here:<br>*Leave blank if not applicable*<br>*Question relevant when: selected( ${q104_M106_full_list} , '3')* | | | |
| q404_M106d *(required)* | q404_M106d. In total, how many units are used to treat a patient during all of her care?<br>*Remember to add all units! E.g. 5 days x 200 mg twice per day = 2,000mg*<br>*Question relevant when: selected( ${q104_M106_full_list} , '3')*<br>*Response constrained to: .>0* | | | |

| Field | Question | Answer |
|---|---|---|
| q404_M107 | ***q404_M107. Ferrous sulphate + folic acid (Ferrotone capsules)***<br>*Question relevant when: selected( ${q104_M107_full_list} , '3')* | |
| q404_M107a *(required)* | q404_M107a. What percent of patients require this item?<br>*Question relevant when: selected( ${q104_M107_full_list} , '3')*<br>*Response constrained to: .>0 and .<=100 or .=999* | |
| q404_M107b *(required)* | q404_M107b. How is the item administered, or given, to the patient?<br>*Question relevant when: selected( ${q104_M107_full_list} , '3')* | 1 Oral - solid (e.g. tablet)<br>2 Oral - liquid<br>3 Sublingual<br>4 Intravenous (IV)<br>5 Intramusculuar injection (IM)<br>6 Subcutaneous<br>7 Rectal<br>8 Inhaled (e.g. gas)<br>9 Inserted (e.g. vaginal pessaries)<br>10 Topical - cream<br>11 Topical - drops (e.g. eye drops)<br>12 Other<br>99 Don't know |
| q404_M107c *(required)* | q404_M107c. What is the item's smallest unit of measurement (mg, mcg, ml, g, etc)?<br>*Question relevant when: selected( ${q104_M107_full_list} , '3')* | microgram — microgram (mcg)<br>miligram — miligram (mg)<br>miligram_dissolved_in_liquid — miligram (mg) dissolved in liquid (e.g. 2 mg per 3 militers)<br>gram — gram (g)<br>militer — militer (ml)<br>centiliter — centiliter (cl)<br>litre — litre (l)<br>ml_of _solution — ml of solution (e.g. ml of 10% solution)<br>litre_of_solution — litre of solution (e.g. 1 litre of 5% solution)<br>cubic_meter — cubic meter (m^3)<br>other — other<br>dont_know — don't know |
| q404_M107c.1 | q404_M107c.1 If other unit of measurement or route of administration, specify here:<br>*Leave blank if not applicable*<br>*Question relevant when: selected( ${q104_M107_full_list} , '3')* | |
| q404_M107d *(required)* | q404_M107d. In total, how many units are used to treat a patient during all of her care?<br>*Remember to add all units! E.g. 5 days x 200 mg twice per day = 2,000mg*<br>*Question relevant when: selected( ${q104_M107_full_list} , '3')*<br>*Response constrained to: .>0* | |

| Field | Question | Answer |
|---|---|---|
| q404_M108 | ***q404_M108. Folic acid (Tablets)***<br>*Question relevant when: selected( ${q104_M108_full_list} , '3')* | |
| q404_M108a *(required)* | q404_M108a. What percent of patients require this item?<br>*Question relevant when: selected( ${q104_M108_full_list} , '3')*<br>*Response constrained to: .>0 and .<=100 or .=999* | |
| q404_M108b *(required)* | q404_M108b. How is the item administered, or given, to the patient?<br>*Question relevant when: selected( ${q104_M108_full_list} , '3')* | 1 Oral - solid (e.g. tablet)<br>2 Oral - liquid<br>3 Sublingual<br>4 Intravenous (IV)<br>5 Intramusculuar injection (IM)<br>6 Subcutaneous<br>7 Rectal<br>8 Inhaled (e.g. gas)<br>9 Inserted (e.g. vaginal pessaries)<br>10 Topical - cream<br>11 Topical - drops (e.g. eye drops)<br>12 Other<br>99 Don't know |
| q404_M108c *(required)* | q404_M108c. What is the item's smallest unit of measurement (mg, mcg, ml, g, etc)?<br>*Question relevant when: selected( ${q104_M108_full_list} , '3')* | microgram — microgram (mcg)<br>miligram — miligram (mg)<br>miligram_dissolved_in_liquid — miligram (mg) dissolved in liquid (e.g. 2 mg per 3 militers)<br>gram — gram (g)<br>militer — militer (ml)<br>centiliter — centiliter (cl)<br>litre — litre (l)<br>ml_of _solution — ml of solution (e.g. ml of 10% solution)<br>litre_of_solution — litre of solution (e.g. 1 litre of 5% solution)<br>cubic_meter — cubic meter (m^3)<br>other — other<br>dont_know — don't know |
| q404_M108c.1 | q404_M108c.1 If other unit of measurement or route of administration, specify here:<br>*Leave blank if not applicable*<br>*Question relevant when: selected( ${q104_M108_full_list} , '3')* | |
| q404_M108d *(required)* | q404_M108d. In total, how many units are used to treat a patient during all of her care?<br>*Remember to add all units! E.g. 5 days x 200 mg twice per day = 2,000mg*<br>*Question relevant when: selected( ${q104_M108_full_list} , '3')*<br>*Response constrained to: .>0* | |

| Field | Question | Answer |
|---|---|---|
| q404_M109 | ***q404_M109. Serum anti-tetanus***<br>*Question relevant when: selected( ${q104_M109_full_list} , '3')* | |
| q404_M109a *(required)* | q404_M109a. What percent of patients require this item?<br>*Question relevant when: selected( ${q104_M109_full_list} , '3')*<br>*Response constrained to: .>0 and .<=100 or .=999* | |
| q404_M109b *(required)* | q404_M109b. How is the item administered, or given, to the patient?<br>*Question relevant when: selected( ${q104_M109_full_list} , '3')* | 1 Oral - solid (e.g. tablet)<br>2 Oral - liquid<br>3 Sublingual<br>4 Intravenous (IV)<br>5 Intramusculuar injection (IM)<br>6 Subcutaneous<br>7 Rectal<br>8 Inhaled (e.g. gas)<br>9 Inserted (e.g. vaginal pessaries)<br>10 Topical - cream<br>11 Topical - drops (e.g. eye drops)<br>12 Other<br>99 Don't know |
| q404_M109c *(required)* | q404_M109c. What is the item's smallest unit of measurement (mg, mcg, ml, g, etc)?<br>*Question relevant when: selected( ${q104_M109_full_list} , '3')* | microgram — microgram (mcg)<br>miligram — miligram (mg)<br>miligram_dissolved_in_liquid — miligram (mg) dissolved in liquid (e.g. 2 mg per 3 militers)<br>gram — gram (g)<br>militer — militer (ml)<br>centiliter — centiliter (cl)<br>litre — litre (l)<br>ml_of _solution — ml of solution (e.g. ml of 10% solution)<br>litre_of_solution — litre of solution (e.g. 1 litre of 5% solution)<br>cubic_meter — cubic meter (m^3)<br>other — other<br>dont_know — don't know |
| q404_M109c.1 | q404_M109c.1 If other unit of measurement or route of administration, specify here:<br>*Leave blank if not applicable*<br>*Question relevant when: selected( ${q104_M109_full_list} , '3')* | |
| q404_M109d *(required)* | q404_M109d. In total, how many units are used to treat a patient during all of her care?<br>*Remember to add all units! E.g. 5 days x 200 mg twice per day = 2,000mg*<br>*Question relevant when: selected( ${q104_M109_full_list} , '3')*<br>*Response constrained to: .>0* | |

| Field | Question | Answer |
|---|---|---|
| q404_M110 | ***q404_M110. Anti D (Rh)-Immunoglobulin***<br>*Question relevant when: selected( ${q104_M110_full_list} , '3')* | |
| q404_M110a *(required)* | q404_M110a. What percent of patients require this item?<br>*Question relevant when: selected( ${q104_M110_full_list} , '3')*<br>*Response constrained to: .>0 and .<=100 or .=999* | |
| q404_M110b *(required)* | q404_M110b. How is the item administered, or given, to the patient?<br>*Question relevant when: selected( ${q104_M110_full_list} , '3')* | 1 Oral - solid (e.g. tablet)<br>2 Oral - liquid<br>3 Sublingual<br>4 Intravenous (IV)<br>5 Intramusculuar injection (IM)<br>6 Subcutaneous<br>7 Rectal<br>8 Inhaled (e.g. gas)<br>9 Inserted (e.g. vaginal pessaries)<br>10 Topical - cream<br>11 Topical - drops (e.g. eye drops)<br>12 Other<br>99 Don't know |
| q404_M110c *(required)* | q404_M110c. What is the item's smallest unit of measurement (mg, mcg, ml, g, etc)?<br>*Question relevant when: selected( ${q104_M110_full_list} , '3')* | microgram — microgram (mcg)<br>miligram — miligram (mg)<br>miligram_dissolved_in_liquid — miligram (mg) dissolved in liquid (e.g. 2 mg per 3 militers)<br>gram — gram (g)<br>militer — militer (ml)<br>centiliter — centiliter (cl)<br>litre — litre (l)<br>ml_of _solution — ml of solution (e.g. ml of 10% solution)<br>litre_of_solution — litre of solution (e.g. 1 litre of 5% solution)<br>cubic_meter — cubic meter (m^3)<br>other — other<br>dont_know — don't know |
| q404_M110c.1 | q404_M110c.1 If other unit of measurement or route of administration, specify here:<br>*Leave blank if not applicable*<br>*Question relevant when: selected( ${q104_M110_full_list} , '3')* | |
| q404_M110d *(required)* | q404_M110d. In total, how many units are used to treat a patient during all of her care?<br>*Remember to add all units! E.g. 5 days x 200 mg twice per day = 2,000mg*<br>*Question relevant when: selected( ${q104_M110_full_list} , '3')*<br>*Response constrained to: .>0* | |

| Field | Question | Answer |
|---|---|---|
| q404_M111 | ***q404_M111. Vitamin, immunological, etc. - Other 1: "[q104_M111_full_list_other]"***<br>*Question relevant when: selected( ${q104_M111_full_list} , '3')* | |
| q404_M111a *(required)* | q404_M111a. What percent of patients require this item?<br>*Question relevant when: selected( ${q104_M111_full_list} , '3')*<br>*Response constrained to: .>0 and .<=100 or .=999* | |
| q404_M111b *(required)* | q404_M111b. How is the item administered, or given, to the patient?<br>*Question relevant when: selected( ${q104_M111_full_list} , '3')* | 1 Oral - solid (e.g. tablet)<br>2 Oral - liquid<br>3 Sublingual<br>4 Intravenous (IV)<br>5 Intramusculuar injection (IM)<br>6 Subcutaneous<br>7 Rectal<br>8 Inhaled (e.g. gas)<br>9 Inserted (e.g. vaginal pessaries)<br>10 Topical - cream<br>11 Topical - drops (e.g. eye drops)<br>12 Other<br>99 Don't know |
| q404_M111c *(required)* | q404_M111c. What is the item's smallest unit of measurement (mg, mcg, ml, g, etc)?<br>*Question relevant when: selected( ${q104_M111_full_list} , '3')* | microgram — microgram (mcg)<br>miligram — miligram (mg)<br>miligram_dissolved_in_liquid — miligram (mg) dissolved in liquid (e.g. 2 mg per 3 militers)<br>gram — gram (g)<br>militer — militer (ml)<br>centiliter — centiliter (cl)<br>litre — litre (l)<br>ml_of _solution — ml of solution (e.g. ml of 10% solution)<br>litre_of_solution — litre of solution (e.g. 1 litre of 5% solution)<br>cubic_meter — cubic meter (m^3)<br>other — other<br>dont_know — don't know |
| q404_M111c.1 | q404_M111c.1 If other unit of measurement or route of administration, specify here:<br>*Leave blank if not applicable*<br>*Question relevant when: selected( ${q104_M111_full_list} , '3')* | |
| q404_M111d *(required)* | q404_M111d. In total, how many units are used to treat a patient during all of her care?<br>*Remember to add all units! E.g. 5 days x 200 mg twice per day = 2,000mg*<br>*Question relevant when: selected( ${q104_M111_full_list} , '3')*<br>*Response constrained to: .>0* | |

| Field | Question | Answer |
|---|---|---|
| q404_M112 | ***q404_M112. Vitamin, immunological, etc. - Other 2: "[q104_M112_full_list_other]"***<br>*Question relevant when: selected( ${q104_M112_full_list} , '3')* | |
| q404_M112a *(required)* | q404_M112a. What percent of patients require this item?<br>*Question relevant when: selected( ${q104_M112_full_list} , '3')*<br>*Response constrained to: .>0 and .<=100 or .=999* | |
| q404_M112b *(required)* | q404_M112b. How is the item administered, or given, to the patient?<br>*Question relevant when: selected( ${q104_M112_full_list} , '3')* | 1 Oral - solid (e.g. tablet)<br>2 Oral - liquid<br>3 Sublingual<br>4 Intravenous (IV)<br>5 Intramusculuar injection (IM)<br>6 Subcutaneous<br>7 Rectal<br>8 Inhaled (e.g. gas)<br>9 Inserted (e.g. vaginal pessaries)<br>10 Topical - cream<br>11 Topical - drops (e.g. eye drops)<br>12 Other<br>99 Don't know |
| q404_M112c *(required)* | q404_M112c. What is the item's smallest unit of measurement (mg, mcg, ml, g, etc)?<br>*Question relevant when: selected( ${q104_M112_full_list} , '3')* | microgram — microgram (mcg)<br>miligram — miligram (mg)<br>miligram_dissolved_in_liquid — miligram (mg) dissolved in liquid (e.g. 2 mg per 3 militers)<br>gram — gram (g)<br>militer — militer (ml)<br>centiliter — centiliter (cl)<br>litre — litre (l)<br>ml_of _solution — ml of solution (e.g. ml of 10% solution)<br>litre_of_solution — litre of solution (e.g. 1 litre of 5% solution)<br>cubic_meter — cubic meter (m^3)<br>other — other<br>dont_know — don't know |
| q404_M112c.1 | q404_M112c.1 If other unit of measurement or route of administration, specify here:<br>*Leave blank if not applicable*<br>*Question relevant when: selected( ${q104_M112_full_list} , '3')* | |
| q404_M112d *(required)* | q404_M112d. In total, how many units are used to treat a patient during all of her care?<br>*Remember to add all units! E.g. 5 days x 200 mg twice per day = 2,000mg*<br>*Question relevant when: selected( ${q104_M112_full_list} , '3')*<br>*Response constrained to: .>0* | |

| Field | Question | Answer |
|---|---|---|
| q404_M113 | ***q404_M113. Vitamin, immunological, etc. - Other 3: "[q104_M113_full_list_other]"***<br>*Question relevant when: selected( ${q104_M113_full_list} , '3')* | |
| q404_M113a *(required)* | q404_M113a. What percent of patients require this item?<br>*Question relevant when: selected( ${q104_M113_full_list} , '3')*<br>*Response constrained to: .>0 and .<=100 or .=999* | |
| q404_M113b *(required)* | q404_M113b. How is the item administered, or given, to the patient?<br>*Question relevant when: selected( ${q104_M113_full_list} , '3')* | 1 Oral - solid (e.g. tablet)<br>2 Oral - liquid<br>3 Sublingual<br>4 Intravenous (IV)<br>5 Intramusculuar injection (IM)<br>6 Subcutaneous<br>7 Rectal<br>8 Inhaled (e.g. gas)<br>9 Inserted (e.g. vaginal pessaries)<br>10 Topical - cream<br>11 Topical - drops (e.g. eye drops)<br>12 Other<br>99 Don't know |
| q404_M113c *(required)* | q404_M113c. What is the item's smallest unit of measurement (mg, mcg, ml, g, etc)?<br>*Question relevant when: selected( ${q104_M113_full_list} , '3')* | microgram — microgram (mcg)<br>miligram — miligram (mg)<br>miligram_dissolved_in_liquid — miligram (mg) dissolved in liquid (e.g. 2 mg per 3 militers)<br>gram — gram (g)<br>militer — militer (ml)<br>centiliter — centiliter (cl)<br>litre — litre (l)<br>ml_of _solution — ml of solution (e.g. ml of 10% solution)<br>litre_of_solution — litre of solution (e.g. 1 litre of 5% solution)<br>cubic_meter — cubic meter (m^3)<br>other — other<br>dont_know — don't know |
| q404_M113c.1 | q404_M113c.1 If other unit of measurement or route of administration, specify here:<br>*Leave blank if not applicable*<br>*Question relevant when: selected( ${q104_M113_full_list} , '3')* | |
| q404_M113d *(required)* | q404_M113d. In total, how many units are used to treat a patient during all of her care?<br>*Remember to add all units! E.g. 5 days x 200 mg twice per day = 2,000mg*<br>*Question relevant when: selected( ${q104_M113_full_list} , '3')*<br>*Response constrained to: .>0* | |

| Field | Question | Answer |
|---|---|---|
| q404_M114 | ***q404_M114. Vitamin, immunological, etc. - Other 4: "[q104_M114_full_list_other]"***<br>*Question relevant when: selected( ${q104_M114_full_list} , '3')* | |
| q404_M114a *(required)* | q404_M114a. What percent of patients require this item?<br>*Question relevant when: selected( ${q104_M114_full_list} , '3')*<br>*Response constrained to: .>0 and .<=100 or .=999* | |

| Field | Question | Answer |
|---|---|---|
| q404_M114b *(required)* | q404_M114b. How is the item administered, or given, to the patient?<br>*Question relevant when: selected( ${q104_M114_full_list} , '3')* | 1 Oral - solid (e.g. tablet)<br>2 Oral - liquid<br>3 Sublingual<br>4 Intravenous (IV)<br>5 Intramusculuar injection (IM)<br>6 Subcutaneous<br>7 Rectal<br>8 Inhaled (e.g. gas)<br>9 Inserted (e.g. vaginal pessaries)<br>10 Topical - cream<br>11 Topical - drops (e.g. eye drops)<br>12 Other<br>99 Don't know |
| q404_M114c *(required)* | q404_M114c. What is the item's smallest unit of measurement (mg, mcg, ml, g, etc)?<br>*Question relevant when: selected( ${q104_M114_full_list} , '3')* | microgram — microgram (mcg)<br>miligram — miligram (mg)<br>miligram_dissolved_in_liquid — miligram (mg) dissolved in liquid (e.g. 2 mg per 3 militers)<br>gram — gram (g)<br>militer — militer (ml)<br>centiliter — centiliter (cl)<br>litre — litre (l)<br>ml_of _solution — ml of solution (e.g. ml of 10% solution)<br>litre_of_solution — litre of solution (e.g. 1 litre of 5% solution)<br>cubic_meter — cubic meter (m^3)<br>other — other<br>dont_know — don't know |
| q404_M114c.1 | q404_M114c.1 If other unit of measurement or route of administration, specify here:<br>*Leave blank if not applicable*<br>*Question relevant when: selected( ${q104_M114_full_list} , '3')* | |
| q404_M114d *(required)* | q404_M114d. In total, how many units are used to treat a patient during all of her care?<br>*Remember to add all units! E.g. 5 days x 200 mg twice per day = 2,000mg*<br>*Question relevant when: selected( ${q104_M114_full_list} , '3')*<br>*Response constrained to: .>0* | |

| Field | Question | Answer |
|---|---|---|
| q404_M115 | ***q404_M115. Vitamin, immunological, etc. - Other 5: "[q104_M115_full_list_other]"***<br>*Question relevant when: selected( ${q104_M115_full_list} , '3')* | |
| q404_M115a *(required)* | q404_M115a. What percent of patients require this item?<br>*Question relevant when: selected( ${q104_M115_full_list} , '3')*<br>*Response constrained to: .>0 and .<=100 or .=999* | |
| q404_M115b *(required)* | q404_M115b. How is the item administered, or given, to the patient?<br>*Question relevant when: selected( ${q104_M115_full_list} , '3')* | 1 Oral - solid (e.g. tablet)<br>2 Oral - liquid<br>3 Sublingual<br>4 Intravenous (IV)<br>5 Intramusculuar injection (IM)<br>6 Subcutaneous<br>7 Rectal<br>8 Inhaled (e.g. gas)<br>9 Inserted (e.g. vaginal pessaries)<br>10 Topical - cream<br>11 Topical - drops (e.g. eye drops)<br>12 Other<br>99 Don't know |
| q404_M115c *(required)* | q404_M115c. What is the item's smallest unit of measurement (mg, mcg, ml, g, etc)?<br>*Question relevant when: selected( ${q104_M115_full_list} , '3')* | microgram — microgram (mcg)<br>miligram — miligram (mg)<br>miligram_dissolved_in_liquid — miligram (mg) dissolved in liquid (e.g. 2 mg per 3 militers)<br>gram — gram (g)<br>militer — militer (ml)<br>centiliter — centiliter (cl)<br>litre — litre (l)<br>ml_of _solution — ml of solution (e.g. ml of 10% solution)<br>litre_of_solution — litre of solution (e.g. 1 litre of 5% solution)<br>cubic_meter — cubic meter (m^3)<br>other — other<br>dont_know — don't know |
| q404_M115c.1 | q404_M115c.1 If other unit of measurement or route of administration, specify here:<br>*Leave blank if not applicable*<br>*Question relevant when: selected( ${q104_M115_full_list} , '3')* | |
| q404_M115d *(required)* | q404_M115d. In total, how many units are used to treat a patient during all of her care?<br>*Remember to add all units! E.g. 5 days x 200 mg twice per day = 2,000mg*<br>*Question relevant when: selected( ${q104_M115_full_list} , '3')*<br>*Response constrained to: .>0* | |

<p><strong><em>D. Medications - Shock (12)</em></strong></p>

*Group relevant when: selected( ${section_four_skip_med} , '1')*

| Field | Question | Answer |
|---|---|---|
| q404_med_notE11 | 404 CONTINUED For each of the following medications and other medical products that are used for shock, can you tell me …? | |
| note_404_Other | ***Other medication, any class*** | |
| q404_M116 | ***q404_M116. Medication - any class - Other 1: "[q104_M116_full_list_other]"***<br>*Question relevant when: selected( ${q104_M116_full_list} , '3')* | |
| q404_M116a *(required)* | q404_M116a. What percent of patients require this item?<br>*Question relevant when: selected( ${q104_M116_full_list} , '3')*<br>*Response constrained to: .>0 and .<=100 or .=999* | |

| Field | Question | Answer |
|---|---|---|
| q404_M116b *(required)* | q404_M116b. How is the item administered, or given, to the patient?<br>*Question relevant when: selected( ${q104_M116_full_list} , '3')* | 1 Oral - solid (e.g. tablet)<br>2 Oral - liquid<br>3 Sublingual<br>4 Intravenous (IV)<br>5 Intramusculuar injection (IM)<br>6 Subcutaneous<br>7 Rectal<br>8 Inhaled (e.g. gas)<br>9 Inserted (e.g. vaginal pessaries)<br>10 Topical - cream<br>11 Topical - drops (e.g. eye drops)<br>12 Other<br>99 Don't know |
| q404_M116c *(required)* | q404_M116c. What is the item's smallest unit of measurement (mg, mcg, ml, g, etc)?<br>*Question relevant when: selected( ${q104_M116_full_list} , '3')* | microgram — microgram (mcg)<br>miligram — miligram (mg)<br>miligram_dissolved_in_liquid — miligram (mg) dissolved in liquid (e.g. 2 mg per 3 militers)<br>gram — gram (g)<br>militer — militer (ml)<br>centiliter — centiliter (cl)<br>litre — litre (l)<br>ml_of _solution — ml of solution (e.g. ml of 10% solution)<br>litre_of_solution — litre of solution (e.g. 1 litre of 5% solution)<br>cubic_meter — cubic meter (m^3)<br>other — other<br>dont_know — don't know |
| q404_M116c.1 | q404_M116c.1 If other unit of measurement or route of administration, specify here:<br>*Leave blank if not applicable*<br>*Question relevant when: selected( ${q104_M116_full_list} , '3')* | |
| q404_M116d *(required)* | q404_M116d. In total, how many units are used to treat a patient during all of her care?<br>*Remember to add all units! E.g. 5 days x 200 mg twice per day = 2,000mg*<br>*Question relevant when: selected( ${q104_M116_full_list} , '3')*<br>*Response constrained to: .>0* | |

| Field | Question | Answer |
|---|---|---|
| q404_M117 | ***q404_M117. Medication - any class - Other 2: "[q104_M117_full_list_other]"***<br>*Question relevant when: selected( ${q104_M117_full_list} , '3')* | |
| q404_M117a *(required)* | q404_M117a. What percent of patients require this item?<br>*Question relevant when: selected( ${q104_M117_full_list} , '3')*<br>*Response constrained to: .>0 and .<=100 or .=999* | |
| q404_M117b *(required)* | q404_M117b. How is the item administered, or given, to the patient?<br>*Question relevant when: selected( ${q104_M117_full_list} , '3')* | 1 Oral - solid (e.g. tablet)<br>2 Oral - liquid<br>3 Sublingual<br>4 Intravenous (IV)<br>5 Intramusculuar injection (IM)<br>6 Subcutaneous<br>7 Rectal<br>8 Inhaled (e.g. gas)<br>9 Inserted (e.g. vaginal pessaries)<br>10 Topical - cream<br>11 Topical - drops (e.g. eye drops)<br>12 Other<br>99 Don't know |
| q404_M117c *(required)* | q404_M117c. What is the item's smallest unit of measurement (mg, mcg, ml, g, etc)?<br>*Question relevant when: selected( ${q104_M117_full_list} , '3')* | microgram — microgram (mcg)<br>miligram — miligram (mg)<br>miligram_dissolved_in_liquid — miligram (mg) dissolved in liquid (e.g. 2 mg per 3 militers)<br>gram — gram (g)<br>militer — militer (ml)<br>centiliter — centiliter (cl)<br>litre — litre (l)<br>ml_of _solution — ml of solution (e.g. ml of 10% solution)<br>litre_of_solution — litre of solution (e.g. 1 litre of 5% solution)<br>cubic_meter — cubic meter (m^3)<br>other — other<br>dont_know — don't know |
| q404_M117c.1 | q404_M117c.1 If other unit of measurement or route of administration, specify here:<br>*Leave blank if not applicable*<br>*Question relevant when: selected( ${q104_M117_full_list} , '3')* | |
| q404_M117d *(required)* | q404_M117d. In total, how many units are used to treat a patient during all of her care?<br>*Remember to add all units! E.g. 5 days x 200 mg twice per day = 2,000mg*<br>*Question relevant when: selected( ${q104_M117_full_list} , '3')*<br>*Response constrained to: .>0* | |

| Field | Question | Answer |
|---|---|---|
| q404_M118 | ***q404_M118. Medication - any class - Other 3: "[q104_M118_full_list_other]"***<br>*Question relevant when: selected( ${q104_M118_full_list} , '3')* | |
| q404_M118a *(required)* | q404_M118a. What percent of patients require this item?<br>*Question relevant when: selected( ${q104_M118_full_list} , '3')*<br>*Response constrained to: .>0 and .<=100 or .=999* | |
| q404_M118b *(required)* | q404_M118b. How is the item administered, or given, to the patient?<br>*Question relevant when: selected( ${q104_M118_full_list} , '3')* | 1 Oral - solid (e.g. tablet)<br>2 Oral - liquid<br>3 Sublingual<br>4 Intravenous (IV)<br>5 Intramusculuar injection (IM)<br>6 Subcutaneous<br>7 Rectal<br>8 Inhaled (e.g. gas)<br>9 Inserted (e.g. vaginal pessaries)<br>10 Topical - cream<br>11 Topical - drops (e.g. eye drops)<br>12 Other<br>99 Don't know |
| q404_M118c *(required)* | q404_M118c. What is the item's smallest unit of measurement (mg, mcg, ml, g, etc)?<br>*Question relevant when: selected( ${q104_M118_full_list} , '3')* | microgram — microgram (mcg)<br>miligram — miligram (mg)<br>miligram_dissolved_in_liquid — miligram (mg) dissolved in liquid (e.g. 2 mg per 3 militers)<br>gram — gram (g)<br>militer — militer (ml)<br>centiliter — centiliter (cl)<br>litre — litre (l)<br>ml_of _solution — ml of solution (e.g. ml of 10% solution)<br>litre_of_solution — litre of solution (e.g. 1 litre of 5% solution)<br>cubic_meter — cubic meter (m^3)<br>other — other<br>dont_know — don't know |
| q404_M118c.1 | q404_M118c.1 If other unit of measurement or route of administration, specify here:<br>*Leave blank if not applicable*<br>*Question relevant when: selected( ${q104_M118_full_list} , '3')* | |
| q404_M118d *(required)* | q404_M118d. In total, how many units are used to treat a patient during all of her care?<br>*Remember to add all units! E.g. 5 days x 200 mg twice per day = 2,000mg*<br>*Question relevant when: selected( ${q104_M118_full_list} , '3')*<br>*Response constrained to: .>0* | |

| Field | Question | Answer |
|---|---|---|
| q404_M119 | ***q404_M119. Medication - any class - Other 4: "[q104_M119_full_list_other]"***<br>*Question relevant when: selected( ${q104_M119_full_list} , '3')* | |
| q404_M119a *(required)* | q404_M119a. What percent of patients require this item?<br>*Question relevant when: selected( ${q104_M119_full_list} , '3')*<br>*Response constrained to: .>0 and .<=100 or .=999* | |
| q404_M119b *(required)* | q404_M119b. How is the item administered, or given, to the patient?<br>*Question relevant when: selected( ${q104_M119_full_list} , '3')* | 1 Oral - solid (e.g. tablet)<br>2 Oral - liquid<br>3 Sublingual<br>4 Intravenous (IV)<br>5 Intramusculuar injection (IM)<br>6 Subcutaneous<br>7 Rectal<br>8 Inhaled (e.g. gas)<br>9 Inserted (e.g. vaginal pessaries)<br>10 Topical - cream<br>11 Topical - drops (e.g. eye drops)<br>12 Other<br>99 Don't know |
| q404_M119c *(required)* | q404_M119c. What is the item's smallest unit of measurement (mg, mcg, ml, g, etc)?<br>*Question relevant when: selected( ${q104_M119_full_list} , '3')* | microgram — microgram (mcg)<br>miligram — miligram (mg)<br>miligram_dissolved_in_liquid — miligram (mg) dissolved in liquid (e.g. 2 mg per 3 militers)<br>gram — gram (g)<br>militer — militer (ml)<br>centiliter — centiliter (cl)<br>litre — litre (l)<br>ml_of _solution — ml of solution (e.g. ml of 10% solution)<br>litre_of_solution — litre of solution (e.g. 1 litre of 5% solution)<br>cubic_meter — cubic meter (m^3)<br>other — other<br>dont_know — don't know |
| q404_M119c.1 | q404_M119c.1 If other unit of measurement or route of administration, specify here:<br>*Leave blank if not applicable*<br>*Question relevant when: selected( ${q104_M119_full_list} , '3')* | |
| q404_M119d *(required)* | q404_M119d. In total, how many units are used to treat a patient during all of her care?<br>*Remember to add all units! E.g. 5 days x 200 mg twice per day = 2,000mg*<br>*Question relevant when: selected( ${q104_M119_full_list} , '3')*<br>*Response constrained to: .>0* | |

| Field | Question | Answer |
|---|---|---|
| q404_M120 | ***q404_M120. Medication - any class - Other 5: "[q104_M120_full_list_other]"***<br>*Question relevant when: selected( ${q104_M120_full_list} , '3')* | |
| q404_M120a *(required)* | q404_M120a. What percent of patients require this item?<br>*Question relevant when: selected( ${q104_M120_full_list} , '3')*<br>*Response constrained to: .>0 and .<=100 or .=999* | |
| q404_M120b *(required)* | q404_M120b. How is the item administered, or given, to the patient?<br>*Question relevant when: selected( ${q104_M120_full_list} , '3')* | 1 Oral - solid (e.g. tablet)<br>2 Oral - liquid<br>3 Sublingual<br>4 Intravenous (IV)<br>5 Intramusculuar injection (IM)<br>6 Subcutaneous<br>7 Rectal<br>8 Inhaled (e.g. gas)<br>9 Inserted (e.g. vaginal pessaries)<br>10 Topical - cream<br>11 Topical - drops (e.g. eye drops)<br>12 Other<br>99 Don't know |
| q404_M120c *(required)* | q404_M120c. What is the item's smallest unit of measurement (mg, mcg, ml, g, etc)?<br>*Question relevant when: selected( ${q104_M120_full_list} , '3')* | microgram — microgram (mcg)<br>miligram — miligram (mg)<br>miligram_dissolved_in_liquid — miligram (mg) dissolved in liquid (e.g. 2 mg per 3 militers)<br>gram — gram (g)<br>militer — militer (ml)<br>centiliter — centiliter (cl)<br>litre — litre (l)<br>ml_of _solution — ml of solution (e.g. ml of 10% solution)<br>litre_of_solution — litre of solution (e.g. 1 litre of 5% solution)<br>cubic_meter — cubic meter (m^3)<br>other — other<br>dont_know — don't know |
| q404_M120c.1 | q404_M120c.1 If other unit of measurement or route of administration, specify here:<br>*Leave blank if not applicable*<br>*Question relevant when: selected( ${q104_M120_full_list} , '3')* | |
| q404_M120d *(required)* | q404_M120d. In total, how many units are used to treat a patient during all of her care?<br>*Remember to add all units! E.g. 5 days x 200 mg twice per day = 2,000mg*<br>*Question relevant when: selected( ${q104_M120_full_list} , '3')*<br>*Response constrained to: .>0* | |

| Field | Question | Answer |
|---|---|---|
| group_section_five_intro | | |
| section5_start | **SECTION V. LACERATIONS - USAGE OF ALL ITEMS** | |
| section_five_skip_med | INTERVIEWER: WOULD YOU LIKE TO COMPLETE THIS SECTION NOW OR SKIP THIS SECTION AND RETURN TO IT LATER?<br>*You may need to skip if the participant has indicated that s/he cannot answer the questions in this section.* | 1 Do not skip, complete this section now.<br>2 Skip and come back to this section later. |
| group_section_five_introB<br>*Group relevant when: selected( ${section_five_skip_med} , '1')* | | |
| section5_start2 | In this section of the interview, we will review all of the items that you said are used for management of cervical or vaginal lacerations. For each item that is used, I'm going to ask questions on how many women need it and how much of it is used. | |

| | | |
|---|---|---|
| section5_start3 | INTERVIEWER: ENTER WHOLE NUMBERS OR DECIMALS. DO NOT TYPE PERCENT SIGNS. ENTER 999 FOR ANY THAT ARE UNKNOWN. | |
| <p><strong><em>D. Medications - Lacerations (1)</em></strong></p><br>*Group relevant when: selected( ${section_five_skip_med} , '1')* | | |
| q504_med_note1 | 504 For each of the following medications and other medical products that are used for lacerations, can you tell me …? | |
| note_504_analgesic | ***Analgesic, anti-inflammatory, narcotic, etc.*** | |
| q504_M1 | ***q504_M1. Acetylsalicylic acid (aspirin)***<br>*Question relevant when: selected( ${q104_M1_full_list} , '4')* | |
| q504_M1a *(required)* | q504_M1a. What percent of patients require this item?<br>*Question relevant when: selected( ${q104_M1_full_list} , '4')*<br>*Response constrained to: .>0 and .<=100 or .=999* | |
| q504_M1b *(required)* | q504_M1b. How is the item administered, or given, to the patient?<br>*Question relevant when: selected( ${q104_M1_full_list} , '4')* | 1 Oral - solid (e.g. tablet)<br>2 Oral - liquid<br>3 Sublingual<br>4 Intravenous (IV)<br>5 Intramusculuar injection (IM)<br>6 Subcutaneous<br>7 Rectal<br>8 Inhaled (e.g. gas)<br>9 Inserted (e.g. vaginal pessaries)<br>10 Topical - cream<br>11 Topical - drops (e.g. eye drops)<br>12 Other<br>99 Don't know |
| q504_M1c *(required)* | q504_M1c. What is the item's smallest unit of measurement (mg, mcg, ml, g, etc)?<br>*Question relevant when: selected( ${q104_M1_full_list} , '4')* | microgram — microgram (mcg)<br>miligram — miligram (mg)<br>miligram_dissolved_in_liquid — miligram (mg) dissolved in liquid (e.g. 2 mg per 3 militers)<br>gram — gram (g)<br>militer — militer (ml)<br>centiliter — centiliter (cl)<br>litre — litre (l)<br>ml_of _solution — ml of solution (e.g. ml of 10% solution)<br>litre_of_solution — litre of solution (e.g. 1 litre of 5% solution)<br>cubic_meter — cubic |

| Field | Question | Answer | | |
|---|---|---|---|---|
| | | | cubic_meter | cubic meter (m^3) |
| | | | other | other |
| | | | dont_know | don't know |
| q504_M1c.1 | q504_M1c.1 If other unit of measurement or route of administration, specify here:<br>*Leave blank if not applicable*<br>*Question relevant when: selected( ${q104_M1_full_list} , '4')* | | | |

| | | | | |
|---|---|---|---|---|
| q504_M1d *(required)* | q504_M1d. In total, how many units are used to treat a patient during all of her care?<br>*Remember to add all units! E.g. 5 days x 200 mg twice per day = 2,000mg*<br>*Question relevant when: selected( ${q104_M1_full_list} , '4')*<br>*Response constrained to: .>0* | | | |
| q504_M2 | ***q504_M2. Paracetamol***<br>*Question relevant when: selected( ${q104_M2_full_list} , '4')* | | | |
| q504_M2a *(required)* | q504_M2a. What percent of patients require this item?<br>*Question relevant when: selected( ${q104_M2_full_list} , '4')*<br>*Response constrained to: .>0 and .<=100 or .=999* | | | |
| q504_M2b *(required)* | q504_M2b. How is the item administered, or given, to the patient?<br>*Question relevant when: selected( ${q104_M2_full_list} , '4')* | | 1 | Oral - solid (e.g. tablet) |
| | | | 2 | Oral - liquid |
| | | | 3 | Sublingual |
| | | | 4 | Intravenous (IV) |
| | | | 5 | Intramuscular injection (IM) |
| | | | 6 | Subcutaneous |
| | | | 7 | Rectal |
| | | | 8 | Inhaled (e.g. gas) |
| | | | 9 | Inserted (e.g. vaginal pessaries) |
| | | | 10 | Topical - cream |
| | | | 11 | Topical - drops (e.g. eye drops) |
| | | | 12 | Other |
| | | | 99 | Don't know |
| q504_M2c *(required)* | q504_M2c. What is the item's smallest unit of measurement (mg, mcg, ml, g, etc)?<br>*Question relevant when: selected( ${q104_M2_full_list} , '4')* | | microgram | microgram (mcg) |
| | | | miligram | miligram (mg) |
| | | | miligram_dissolved_in_liquid | miligram (mg) dissolved in liquid (e.g. 2 mg per 3 militers) |
| | | | gram | gram (g) |
| | | | militer | militer (ml) |
| | | | centiliter | centiliter (cl) |
| | | | litre | litre (l) |
| | | | ml_of _solution | ml of solution (e.g. ml of 10% solution) |
| | | | litre_of_solution | litre of solution (e.g. 1 litre of 5% solution) |
| | | | cubic_meter | cubic meter (m^3) |
| | | | other | other |

| Field | Question | Answer | | |
|---|---|---|---|---|
| | | | dont_know | don't know |
| q504_M2c.1 | q504_M2c.1 If other unit of measurement or route of administration, specify here:<br>*Leave blank if not applicable*<br>*Question relevant when: selected( ${q104_M2_full_list} , '4')* | | | |
| q504_M2d *(required)* | q504_M2d. In total, how many units are used to treat a patient during all of her care?<br>*Remember to add all units! E.g. 5 days x 200 mg twice per day = 2,000mg*<br>*Question relevant when: selected( ${q104_M2_full_list} , '4')*<br>*Response constrained to: .>0* | | | |
| q504_M3 | ***q504_M3. Pethidine hydrochloride***<br>*Question relevant when: selected( ${q104_M3_full_list} , '4')* | | | |

| | | | | |
|---|---|---|---|---|
| q504_M3a *(required)* | q504_M3a. What percent of patients require this item?<br>*Question relevant when: selected( ${q104_M3_full_list} , '4')*<br>*Response constrained to: .>0 and .<=100 or .=999* | | | |
| q504_M3b *(required)* | q504_M3b. How is the item administered, or given, to the patient?<br>*Question relevant when: selected( ${q104_M3_full_list} , '4')* | | 1 | Oral - solid (e.g. tablet) |
| | | | 2 | Oral - liquid |
| | | | 3 | Sublingual |
| | | | 4 | Intravenous (IV) |
| | | | 5 | Intramusculuar injection (IM) |
| | | | 6 | Subcutaneous |
| | | | 7 | Rectal |
| | | | 8 | Inhaled (e.g. gas) |
| | | | 9 | Inserted (e.g. vaginal pessaries) |
| | | | 10 | Topical - cream |
| | | | 11 | Topical - drops (e.g. eye drops) |
| | | | 12 | Other |
| | | | 99 | Don't know |
| q504_M3c *(required)* | q504_M3c. What is the item's smallest unit of measurement (mg, mcg, ml, g, etc)?<br>*Question relevant when: selected( ${q104_M3_full_list} , '4')* | | microgram | microgram (mcg) |
| | | | miligram | miligram (mg) |
| | | | miligram_dissolved_in_liquid | miligram (mg) dissolved in liquid (e.g. 2 mg per 3 militers) |
| | | | gram | gram (g) |
| | | | militer | militer (ml) |
| | | | centiliter | centiliter (cl) |
| | | | litre | litre (l) |
| | | | ml_of _solution | ml of solution (e.g. ml of 10% solution) |
| | | | litre_of_solution | litre of solution (e.g. 1 litre of 5% solution) |
| | | | cubic_meter | cubic meter (m^3) |
| | | | other | other |
| | | | dont_know | don't know |
| q504_M3c.1 | q504_M3c.1 If other unit of measurement or route of administration, specify here:<br>*Leave blank if not applicable*<br>*Question relevant when: selected( ${q104_M3_full_list} , '4')* | | | |
| q504_M3d *(required)* | q504_M3d. In total, how many units are used to treat a patient during all of her care? | | | |

| Field | Question | Answer |
|---|---|---|
| | *Remember to add all units! E.g. 5 days x 200 mg twice per day = 2,000mg*<br>*Question relevant when: selected( ${q104_M3_full_list} , '4')* | |
| | *Response constrained to: .>0* | |
| q504_M4 | ***q504_M4. Tramadol Hydrochloride***<br>*Question relevant when: selected( ${q104_M4_full_list} , '4')* | |
| q504_M4a *(required)* | q504_M4a. What percent of patients require this item?<br>*Question relevant when: selected( ${q104_M4_full_list} , '4')*<br>*Response constrained to: .>0 and .<=100 or .=999* | |

| Field | Question | Answer | | |
|---|---|---|---|---|
| q504_M4b *(required)* | q504_M4b. How is the item administered, or given, to the patient?<br>*Question relevant when: selected( ${q104_M4_full_list} , '4')* | | 1 | Oral - solid (e.g. tablet) |
| | | | 2 | Oral - liquid |
| | | | 3 | Sublingual |
| | | | 4 | Intravenous (IV) |
| | | | 5 | Intramusculuar injection (IM) |
| | | | 6 | Subcutaneous |
| | | | 7 | Rectal |
| | | | 8 | Inhaled (e.g. gas) |
| | | | 9 | Inserted (e.g. vaginal pessaries) |
| | | | 10 | Topical - cream |
| | | | 11 | Topical - drops (e.g. eye drops) |
| | | | 12 | Other |
| | | | 99 | Don't know |
| q504_M4c *(required)* | q504_M4c. What is the item's smallest unit of measurement (mg, mcg, ml, g, etc)?<br>*Question relevant when: selected( ${q104_M4_full_list} , '4')* | | microgram | microgram (mcg) |
| | | | miligram | miligram (mg) |
| | | | miligram_dissolved_in_liquid | miligram (mg) dissolved in liquid (e.g. 2 mg per 3 militers) |
| | | | gram | gram (g) |
| | | | militer | militer (ml) |
| | | | centiliter | centiliter (cl) |
| | | | litre | litre (l) |
| | | | ml_of _solution | ml of solution (e.g. ml of 10% solution) |
| | | | litre_of_solution | litre of solution (e.g. 1 litre of 5% solution) |
| | | | cubic_meter | cubic meter (m^3) |
| | | | other | other |
| | | | dont_know | don't know |
| q504_M4c.1 | q504_M4c.1 If other unit of measurement or route of administration, specify here:<br>*Leave blank if not applicable*<br>*Question relevant when: selected( ${q104_M4_full_list} , '4')* | | | |
| q504_M4d *(required)* | q504_M4d. In total, how many units are used to treat a patient during all of her care?<br>*Remember to add all units! E.g. 5 days x 200 mg twice per day = 2,000mg*<br>*Question relevant when: selected( ${q104_M4_full_list} , '4')*<br>*Response constrained to: .>0* | | | |

| Field | Question | Answer |
|---|---|---|
| q504_M5 | ***q504_M5. Ibuprofen***<br>*Question relevant when: selected( ${q104_M5_full_list} , '4')* | |
| q504_M5a *(required)* | q504_M5a. What percent of patients require this item?<br>*Question relevant when: selected( ${q104_M5_full_list} , '4')*<br>*Response constrained to: .>0 and .<=100 or .=999* | |
| q504_M5b *(required)* | q504_M5b. How is the item administered, or given, to the patient?<br>*Question relevant when: selected( ${q104_M5_full_list} , '4')* | 1 Oral - solid (e.g. tablet)<br>2 Oral - liquid<br>3 Sublingual<br>4 Intravenous (IV)<br>5 Intramusculuar injection (IM)<br>6 Subcutaneous<br>7 Rectal<br>8 Inhaled (e.g. gas)<br>9 Inserted (e.g. vaginal pessaries)<br>10 Topical - cream<br>11 Topical - drops (e.g. eye drops)<br>12 Other<br>99 Don't know |
| q504_M5c *(required)* | q504_M5c. What is the item's smallest unit of measurement (mg, mcg, ml, g, etc)?<br>*Question relevant when: selected( ${q104_M5_full_list} , '4')* | microgram — microgram (mcg)<br>miligram — miligram (mg)<br>miligram_dissolved_in_liquid — miligram (mg) dissolved in liquid (e.g. 2 mg per 3 militers)<br>gram — gram (g)<br>militer — militer (ml)<br>centiliter — centiliter (cl)<br>litre — litre (l)<br>ml_of _solution — ml of solution (e.g. ml of 10% solution)<br>litre_of_solution — litre of solution (e.g. 1 litre of 5% solution)<br>cubic_meter — cubic meter (m^3)<br>other — other<br>dont_know — don't know |
| q504_M5c.1 | q504_M5c.1 If other unit of measurement or route of administration, specify here:<br>*Leave blank if not applicable*<br>*Question relevant when: selected( ${q104_M5_full_list} , '4')* | |
| q504_M5d *(required)* | q504_M5d. In total, how many units are used to treat a patient during all of her care?<br>*Remember to add all units! E.g. 5 days x 200 mg twice per day = 2,000mg*<br>*Question relevant when: selected( ${q104_M5_full_list} , '4')*<br>*Response constrained to: .>0* | |

| Field | Question | Answer |
|---|---|---|
| q504_M6 | ***q504_M6. Diclofenac*** *Question relevant when: selected( ${q104_M6_full_list} , '4')* | |
| q504_M6a *(required)* | q504_M6a. What percent of patients require this item? *Question relevant when: selected( ${q104_M6_full_list} , '4')* *Response constrained to: .>0 and .<=100 or .=999* | |
| q504_M6b *(required)* | q504_M6b. How is the item administered, or given, to the patient? *Question relevant when: selected( ${q104_M6_full_list} , '4')* | 1 Oral - solid (e.g. tablet)<br>2 Oral - liquid<br>3 Sublingual<br>4 Intravenous (IV)<br>5 Intramusculuar injection (IM)<br>6 Subcutaneous<br>7 Rectal<br>8 Inhaled (e.g. gas)<br>9 Inserted (e.g. vaginal pessaries)<br>10 Topical - cream<br>11 Topical - drops (e.g. eye drops)<br>12 Other<br>99 Don't know |
| q504_M6c *(required)* | q504_M6c. What is the item's smallest unit of measurement (mg, mcg, ml, g, etc)? *Question relevant when: selected( ${q104_M6_full_list} , '4')* | microgram — microgram (mcg)<br>miligram — miligram (mg)<br>miligram_dissolved_in_liquid — miligram (mg) dissolved in liquid (e.g. 2 mg per 3 militers)<br>gram — gram (g)<br>militer — militer (ml)<br>centiliter — centiliter (cl)<br>litre — litre (l)<br>ml_of _solution — ml of solution (e.g. ml of 10% solution)<br>litre_of_solution — litre of solution (e.g. 1 litre of 5% solution)<br>cubic_meter — cubic meter (m^3)<br>other — other<br>dont_know — don't know |
| q504_M6c.1 | q504_M6c.1 If other unit of measurement or route of administration, specify here: *Leave blank if not applicable* *Question relevant when: selected( ${q104_M6_full_list} , '4')* | |
| q504_M6d *(required)* | q504_M6d. In total, how many units are used to treat a patient during all of her care? *Remember to add all units! E.g. 5 days x 200 mg twice per day = 2,000mg* *Question relevant when: selected( ${q104_M6_full_list} , '4')* *Response constrained to: .>0* | |

| Field | Question | Answer |
|---|---|---|
| q504_M7 | ***q504_M7. Diclofenac sodium***<br>*Question relevant when: selected( ${q104_M7_full_list} , '4')* | |
| q504_M7a *(required)* | q504_M7a. What percent of patients require this item?<br>*Question relevant when: selected( ${q104_M7_full_list} , '4')*<br>*Response constrained to: .>0 and .<=100 or .=999* | |
| q504_M7b *(required)* | q504_M7b. How is the item administered, or given, to the patient?<br>*Question relevant when: selected( ${q104_M7_full_list} , '4')* | 1 Oral - solid (e.g. tablet)<br>2 Oral - liquid<br>3 Sublingual<br>4 Intravenous (IV)<br>5 Intramusculuar injection (IM)<br>6 Subcutaneous<br>7 Rectal<br>8 Inhaled (e.g. gas)<br>9 Inserted (e.g. vaginal pessaries)<br>10 Topical - cream<br>11 Topical - drops (e.g. eye drops)<br>12 Other<br>99 Don't know |
| q504_M7c *(required)* | q504_M7c. What is the item's smallest unit of measurement (mg, mcg, ml, g, etc)?<br>*Question relevant when: selected( ${q104_M7_full_list} , '4')* | microgram — microgram (mcg)<br>miligram — miligram (mg)<br>miligram_dissolved_in_liquid — miligram (mg) dissolved in liquid (e.g. 2 mg per 3 militers)<br>gram — gram (g)<br>militer — militer (ml)<br>centiliter — centiliter (cl)<br>litre — litre (l)<br>ml_of _solution — ml of solution (e.g. ml of 10% solution)<br>litre_of_solution — litre of solution (e.g. 1 litre of 5% solution)<br>cubic_meter — cubic meter (m^3)<br>other — other<br>dont_know — don't know |
| q504_M7c.1 | q504_M7c.1 If other unit of measurement or route of administration, specify here:<br>*Leave blank if not applicable*<br>*Question relevant when: selected( ${q104_M7_full_list} , '4')* | |
| q504_M7d *(required)* | q504_M7d. In total, how many units are used to treat a patient during all of her care?<br>*Remember to add all units! E.g. 5 days x 200 mg twice per day = 2,000mg*<br>*Question relevant when: selected( ${q104_M7_full_list} , '4')*<br>*Response constrained to: .>0* | |

| Field | Question | Answer |
|---|---|---|
| q504_M8 | ***q504_M8. Dypron (dipyrone), 250mg/ml (Metamizole)***<br>*Question relevant when: selected( ${q104_M8_full_list} , '4')* | |
| q504_M8a *(required)* | q504_M8a. What percent of patients require this item?<br>*Question relevant when: selected( ${q104_M8_full_list} , '4')*<br>*Response constrained to: .>0 and .<=100 or .=999* | |
| q504_M8b *(required)* | q504_M8b. How is the item administered, or given, to the patient?<br>*Question relevant when: selected( ${q104_M8_full_list} , '4')* | 1 Oral - solid (e.g. tablet)<br>2 Oral - liquid<br>3 Sublingual<br>4 Intravenous (IV)<br>5 Intramusculuar injection (IM)<br>6 Subcutaneous<br>7 Rectal<br>8 Inhaled (e.g. gas)<br>9 Inserted (e.g. vaginal pessaries)<br>10 Topical - cream<br>11 Topical - drops (e.g. eye drops)<br>12 Other<br>99 Don't know |
| q504_M8c *(required)* | q504_M8c. What is the item's smallest unit of measurement (mg, mcg, ml, g, etc)?<br>*Question relevant when: selected( ${q104_M8_full_list} , '4')* | microgram — microgram (mcg)<br>miligram — miligram (mg)<br>miligram_dissolved_in_liquid — miligram (mg) dissolved in liquid (e.g. 2 mg per 3 militers)<br>gram — gram (g)<br>militer — militer (ml)<br>centiliter — centiliter (cl)<br>litre — litre (l)<br>ml_of _solution — ml of solution (e.g. ml of 10% solution)<br>litre_of_solution — litre of solution (e.g. 1 litre of 5% solution)<br>cubic_meter — cubic meter (m^3)<br>other — other<br>dont_know — don't know |
| q504_M8c.1 | q504_M8c.1 If other unit of measurement or route of administration, specify here:<br>*Leave blank if not applicable*<br>*Question relevant when: selected( ${q104_M8_full_list} , '4')* | |
| q504_M8d *(required)* | q504_M8d. In total, how many units are used to treat a patient during all of her care?<br>*Remember to add all units! E.g. 5 days x 200 mg twice per day = 2,000mg*<br>*Question relevant when: selected( ${q104_M8_full_list} , '4')*<br>*Response constrained to: .>0* | |

| Field | Question | Answer |
|---|---|---|
| q504_M9 | ***q504_M9. Fentanyl citrate, 50 mcg/ml***<br>*Question relevant when: selected( ${q104_M9_full_list} , '4')* | |
| q504_M9a *(required)* | q504_M9a. What percent of patients require this item?<br>*Question relevant when: selected( ${q104_M9_full_list} , '4')*<br>*Response constrained to: .>0 and .<=100 or .=999* | |

| Field | Question | Answer | | |
|---|---|---|---|---|
| q504_M9b *(required)* | q504_M9b. How is the item administered, or given, to the patient?<br>*Question relevant when: selected( ${q104_M9_full_list} , '4')* | | 1 | Oral - solid (e.g. tablet) |
| | | | 2 | Oral - liquid |
| | | | 3 | Sublingual |
| | | | 4 | Intravenous (IV) |
| | | | 5 | Intramusculuar injection (IM) |
| | | | 6 | Subcutaneous |
| | | | 7 | Rectal |
| | | | 8 | Inhaled (e.g. gas) |
| | | | 9 | Inserted (e.g. vaginal pessaries) |
| | | | 10 | Topical - cream |
| | | | 11 | Topical - drops (e.g. eye drops) |
| | | | 12 | Other |
| | | | 99 | Don't know |
| q504_M9c *(required)* | q504_M9c. What is the item's smallest unit of measurement (mg, mcg, ml, g, etc)?<br>*Question relevant when: selected( ${q104_M9_full_list} , '4')* | | microgram | microgram (mcg) |
| | | | miligram | miligram (mg) |
| | | | miligram_dissolved_in_liquid | miligram (mg) dissolved in liquid (e.g. 2 mg per 3 militers) |
| | | | gram | gram (g) |
| | | | militer | militer (ml) |
| | | | centiliter | centiliter (cl) |
| | | | litre | litre (l) |
| | | | ml_of _solution | ml of solution (e.g. ml of 10% solution) |
| | | | litre_of_solution | litre of solution (e.g. 1 litre of 5% solution) |
| | | | cubic_meter | cubic meter (m^3) |
| | | | other | other |
| | | | dont_know | don't know |
| q504_M9c.1 | q504_M9c.1 If other unit of measurement or route of administration, specify here:<br>*Leave blank if not applicable*<br>*Question relevant when: selected( ${q104_M9_full_list} , '4')* | | | |
| q504_M9d *(required)* | q504_M9d. In total, how many units are used to treat a patient during all of her care?<br>*Remember to add all units! E.g. 5 days x 200 mg twice per day = 2,000mg*<br>*Question relevant when: selected( ${q104_M9_full_list} , '4')*<br>*Response constrained to: .>0* | | | |

| Field | Question | Answer |
|---|---|---|
| q504_M10 | ***q504_M10. Analgesic - other 1: "[q104_M10_full_list_other]"*** *Question relevant when: selected( ${q104_M10_full_list} , '4')* | |
| q504_M10a *(required)* | q504_M10a. What percent of patients require this item? *Question relevant when: selected( ${q104_M10_full_list} , '4')* *Response constrained to: .>0 and .<=100 or .=999* | |

| Field | Question | Answer | | |
|---|---|---|---|---|
| q504_M10b *(required)* | q504_M10b. How is the item administered, or given, to the patient? *Question relevant when: selected( ${q104_M10_full_list} , '4')* | | 1 | Oral - solid (e.g. tablet) |
| | | | 2 | Oral - liquid |
| | | | 3 | Sublingual |
| | | | 4 | Intravenous (IV) |
| | | | 5 | Intramusculuar injection (IM) |
| | | | 6 | Subcutaneous |
| | | | 7 | Rectal |
| | | | 8 | Inhaled (e.g. gas) |
| | | | 9 | Inserted (e.g. vaginal pessaries) |
| | | | 10 | Topical - cream |
| | | | 11 | Topical - drops (e.g. eye drops) |
| | | | 12 | Other |
| | | | 99 | Don't know |
| q504_M10c *(required)* | q504_M10c. What is the item's smallest unit of measurement (mg, mcg, ml, g, etc)? *Question relevant when: selected( ${q104_M10_full_list} , '4')* | | microgram | microgram (mcg) |
| | | | miligram | miligram (mg) |
| | | | miligram_dissolved_in_liquid | miligram (mg) dissolved in liquid (e.g. 2 mg per 3 militers) |
| | | | gram | gram (g) |
| | | | militer | militer (ml) |
| | | | centiliter | centiliter (cl) |
| | | | litre | litre (l) |
| | | | ml_of _solution | ml of solution (e.g. ml of 10% solution) |
| | | | litre_of_solution | litre of solution (e.g. 1 litre of 5% solution) |
| | | | cubic_meter | cubic meter (m^3) |
| | | | other | other |
| | | | dont_know | don't know |
| q504_M10c.1 | q504_M10c.1 If other unit of measurement or route of administration, specify here: *Leave blank if not applicable* *Question relevant when: selected( ${q104_M10_full_list} , '4')* | | | |
| q504_M10d *(required)* | q504_M10d. In total, how many units are used to treat a patient during all of her care? *Remember to add all units! E.g. 5 days x 200 mg twice per day = 2,000mg* *Question relevant when: selected( ${q104_M10_full_list} , '4')* *Response constrained to: .>0* | | | |

| Field | Question | Answer |
|---|---|---|
| q504_M11 | ***q504_M11. Analgesic - other 2: "[q104_M11_full_list_other]"***<br>*Question relevant when: selected( ${q104_M11_full_list} , '4')* | |
| q504_M11a *(required)* | q504_M11a. What percent of patients require this item?<br>*Question relevant when: selected( ${q104_M11_full_list} , '4')*<br>*Response constrained to: .>0 and .<=100 or .=999* | |

| Field | Question | Answer | |
|---|---|---|---|
| q504_M11b *(required)* | q504_M11b. How is the item administered, or given, to the patient?<br>*Question relevant when: selected( ${q104_M11_full_list} , '4')* | 1 | Oral - solid (e.g. tablet) |
| | | 2 | Oral - liquid |
| | | 3 | Sublingual |
| | | 4 | Intravenous (IV) |
| | | 5 | Intramusculuar injection (IM) |
| | | 6 | Subcutaneous |
| | | 7 | Rectal |
| | | 8 | Inhaled (e.g. gas) |
| | | 9 | Inserted (e.g. vaginal pessaries) |
| | | 10 | Topical - cream |
| | | 11 | Topical - drops (e.g. eye drops) |
| | | 12 | Other |
| | | 99 | Don't know |
| q504_M11c *(required)* | q504_M11c. What is the item's smallest unit of measurement (mg, mcg, ml, g, etc)?<br>*Question relevant when: selected( ${q104_M11_full_list} , '4')* | microgram | microgram (mcg) |
| | | miligram | miligram (mg) |
| | | miligram_dissolved_in_liquid | miligram (mg) dissolved in liquid (e.g. 2 mg per 3 militers) |
| | | gram | gram (g) |
| | | militer | militer (ml) |
| | | centiliter | centiliter (cl) |
| | | litre | litre (l) |
| | | ml_of _solution | ml of solution (e.g. ml of 10% solution) |
| | | litre_of_solution | litre of solution (e.g. 1 litre of 5% solution) |
| | | cubic_meter | cubic meter (m^3) |
| | | other | other |
| | | dont_know | don't know |
| q504_M11c.1 | q504_M11c.1 If other unit of measurement or route of administration, specify here:<br>*Leave blank if not applicable*<br>*Question relevant when: selected( ${q104_M11_full_list} , '4')* | | |
| q504_M11d *(required)* | q504_M11d. In total, how many units are used to treat a patient during all of her care?<br>*Remember to add all units! E.g. 5 days x 200 mg twice per day = 2,000mg*<br>*Question relevant when: selected( ${q104_M11_full_list} , '4')*<br>*Response constrained to: .>0* | | |

| Field | Question | Answer |
|---|---|---|
| q504_M12 | ***q504_M12. Analgesic - other 3: "[q104_M12_full_list_other]"*** *Question relevant when: selected( ${q104_M12_full_list} , '4')* | |
| q504_M12a *(required)* | q504_M12a. What percent of patients require this item? *Question relevant when: selected( ${q104_M12_full_list} , '4')* *Response constrained to: .>0 and .<=100 or .=999* | |

| | | |
|---|---|---|
| q504_M12b *(required)* | q504_M12b. How is the item administered, or given, to the patient? *Question relevant when: selected( ${q104_M12_full_list} , '4')* | 1 Oral - solid (e.g. tablet)<br>2 Oral - liquid<br>3 Sublingual<br>4 Intravenous (IV)<br>5 Intramusculuar injection (IM)<br>6 Subcutaneous<br>7 Rectal<br>8 Inhaled (e.g. gas)<br>9 Inserted (e.g. vaginal pessaries)<br>10 Topical - cream<br>11 Topical - drops (e.g. eye drops)<br>12 Other<br>99 Don't know |
| q504_M12c *(required)* | q504_M12c. What is the item's smallest unit of measurement (mg, mcg, ml, g, etc)? *Question relevant when: selected( ${q104_M12_full_list} , '4')* | microgram: microgram (mcg)<br>miligram: miligram (mg)<br>miligram_dissolved_in_liquid: miligram (mg) dissolved in liquid (e.g. 2 mg per 3 militers)<br>gram: gram (g)<br>militer: militer (ml)<br>centiliter: centiliter (cl)<br>litre: litre (l)<br>ml_of _solution: ml of solution (e.g. ml of 10% solution)<br>litre_of_solution: litre of solution (e.g. 1 litre of 5% solution)<br>cubic_meter: cubic meter (m^3)<br>other: other<br>dont_know: don't know |
| q504_M12c.1 | q504_M12c.1 If other unit of measurement or route of administration, specify here: *Leave blank if not applicable* *Question relevant when: selected( ${q104_M12_full_list} , '4')* | |
| q504_M12d *(required)* | q504_M12d. In total, how many units are used to treat a patient during all of her care? *Remember to add all units! E.g. 5 days x 200 mg twice per day = 2,000mg* *Question relevant when: selected( ${q104_M12_full_list} , '4')* *Response constrained to: .>0* | |

| Field | Question | Answer |
|---|---|---|
| q504_M13 | ***q504_M13. Analgesic - other 4: "[q104_M13_full_list_other]"***<br>*Question relevant when: selected( ${q104_M13_full_list} , '4')* | |
| q504_M13a *(required)* | q504_M13a. What percent of patients require this item?<br>*Question relevant when: selected( ${q104_M13_full_list} , '4')*<br>*Response constrained to: .>0 and .<=100 or .=999* | |
| q504_M13b *(required)* | q504_M13b. How is the item administered, or given, to the patient?<br>*Question relevant when: selected( ${q104_M13_full_list} , '4')* | 1 Oral - solid (e.g. tablet)<br>2 Oral - liquid<br>3 Sublingual<br>4 Intravenous (IV)<br>5 Intramusculuar injection (IM)<br>6 Subcutaneous<br>7 Rectal<br>8 Inhaled (e.g. gas)<br>9 Inserted (e.g. vaginal pessaries)<br>10 Topical - cream<br>11 Topical - drops (e.g. eye drops)<br>12 Other<br>99 Don't know |
| q504_M13c *(required)* | q504_M13c. What is the item's smallest unit of measurement (mg, mcg, ml, g, etc)?<br>*Question relevant when: selected( ${q104_M13_full_list} , '4')* | microgram — microgram (mcg)<br>miligram — miligram (mg)<br>miligram_dissolved_in_liquid — miligram (mg) dissolved in liquid (e.g. 2 mg per 3 militers)<br>gram — gram (g)<br>militer — militer (ml)<br>centiliter — centiliter (cl)<br>litre — litre (l)<br>ml_of _solution — ml of solution (e.g. ml of 10% solution)<br>litre_of_solution — litre of solution (e.g. 1 litre of 5% solution)<br>cubic_meter — cubic meter (m^3)<br>other — other<br>dont_know — don't know |
| q504_M13c.1 | q504_M13c.1 If other unit of measurement or route of administration, specify here:<br>*Leave blank if not applicable*<br>*Question relevant when: selected( ${q104_M13_full_list} , '4')* | |
| q504_M13d *(required)* | q504_M13d. In total, how many units are used to treat a patient during all of her care?<br>*Remember to add all units! E.g. 5 days x 200 mg twice per day = 2,000mg*<br>*Question relevant when: selected( ${q104_M13_full_list} , '4')*<br>*Response constrained to: .>0* | |

| Field | Question | Answer |
|---|---|---|
| q504_M14 | ***q504_M14. Analgesic - other 5: "[q104_M14_full_list_other]"*** *Question relevant when: selected( ${q104_M14_full_list} , '4')* | |
| q504_M14a *(required)* | q504_M14a. What percent of patients require this item? *Question relevant when: selected( ${q104_M14_full_list} , '4')* *Response constrained to: .>0 and .<=100 or .=999* | |
| q504_M14b *(required)* | q504_M14b. How is the item administered, or given, to the patient? *Question relevant when: selected( ${q104_M14_full_list} , '4')* | 1 Oral - solid (e.g. tablet); 2 Oral - liquid; 3 Sublingual; 4 Intravenous (IV); 5 Intramusculuar injection (IM); 6 Subcutaneous; 7 Rectal; 8 Inhaled (e.g. gas); 9 Inserted (e.g. vaginal pessaries); 10 Topical - cream; 11 Topical - drops (e.g. eye drops); 12 Other; 99 Don't know |
| q504_M14c *(required)* | q504_M14c. What is the item's smallest unit of measurement (mg, mcg, ml, g, etc)? *Question relevant when: selected( ${q104_M14_full_list} , '4')* | microgram: microgram (mcg); miligram: miligram (mg); miligram_dissolved_in_liquid: miligram (mg) dissolved in liquid (e.g. 2 mg per 3 militers); gram: gram (g); militer: militer (ml); centiliter: centiliter (cl); litre: litre (l); ml_of _solution: ml of solution (e.g. ml of 10% solution); litre_of_solution: litre of solution (e.g. 1 litre of 5% solution); cubic_meter: cubic meter (m^3); other: other; dont_know: don't know |
| q504_M14c.1 | q504_M14c.1 If other unit of measurement or route of administration, specify here: *Leave blank if not applicable* *Question relevant when: selected( ${q104_M14_full_list} , '4')* | |
| q504_M14d *(required)* | q504_M14d. In total, how many units are used to treat a patient during all of her care? *Remember to add all units! E.g. 5 days x 200 mg twice per day = 2,000mg* *Question relevant when: selected( ${q104_M14_full_list} , '4')* *Response constrained to: .>0* | |

<p><strong><em>D. Medications - Lacerations (2)</em></strong></p>
*Group relevant when: selected( ${section_five_skip_med} , '1')*

| Field | Question | Answer |
|---|---|---|
| q504_med_note2 | 504 For each of the following medications and other medical products that are used for lacerations, can you tell me …? | |
| note_504_anesthetic | ***Anesthetic - local, general, etc. (and oxygen)*** | |
| q504_M15 | ***q504_M15. Halothane***<br>*Question relevant when: selected( ${q104_M15_full_list} , '4')* | |
| q504_M15a *(required)* | q504_M15a. What percent of patients require this item?<br>*Question relevant when: selected( ${q104_M15_full_list} , '4')*<br>*Response constrained to: .>0 and .<=100 or .=999* | |

| Field | Question | Answer | | |
|---|---|---|---|---|
| q504_M15b *(required)* | q504_M15b. How is the item administered, or given, to the patient?<br>*Question relevant when: selected( ${q104_M15_full_list} , '4')* | | 1 | Oral - solid (e.g. tablet) |
| | | | 2 | Oral - liquid |
| | | | 3 | Sublingual |
| | | | 4 | Intravenous (IV) |
| | | | 5 | Intramusculuar injection (IM) |
| | | | 6 | Subcutaneous |
| | | | 7 | Rectal |
| | | | 8 | Inhaled (e.g. gas) |
| | | | 9 | Inserted (e.g. vaginal pessaries) |
| | | | 10 | Topical - cream |
| | | | 11 | Topical - drops (e.g. eye drops) |
| | | | 12 | Other |
| | | | 99 | Don't know |
| q504_M15c *(required)* | q504_M15c. What is the item's smallest unit of measurement (mg, mcg, ml, g, etc)?<br>*Question relevant when: selected( ${q104_M15_full_list} , '4')* | | microgram | microgram (mcg) |
| | | | miligram | miligram (mg) |
| | | | miligram_dissolved_in_liquid | miligram (mg) dissolved in liquid (e.g. 2 mg per 3 militers) |
| | | | gram | gram (g) |
| | | | militer | militer (ml) |
| | | | centiliter | centiliter (cl) |
| | | | litre | litre (l) |
| | | | ml_of _solution | ml of solution (e.g. ml of 10% solution) |
| | | | litre_of_solution | litre of solution (e.g. 1 litre of 5% solution) |
| | | | cubic_meter | cubic meter (m^3) |
| | | | other | other |
| | | | dont_know | don't know |
| q504_M15c.1 | q504_M15c.1 If other unit of measurement or route of administration, specify here:<br>*Leave blank if not applicable*<br>*Question relevant when: selected( ${q104_M15_full_list} , '4')* | | | |
| q504_M15d *(required)* | q504_M15d. In total, how many units are used to treat a patient during all of her care?<br>*Remember to add all units! E.g. 5 days x 200 mg twice per day = 2,000mg*<br>*Question relevant when: selected( ${q104_M15_full_list} , '4')*<br>*Response constrained to: .>0* | | | |

| Field | Question | Answer |
|---|---|---|
| q504_M16 | ***q504_M16. Ketamine (Ketalar), 50mg/ml***<br>*Question relevant when: selected( ${q104_M16_full_list} , '4')* | |
| q504_M16a *(required)* | q504_M16a. What percent of patients require this item?<br>*Question relevant when: selected( ${q104_M16_full_list} , '4')*<br>*Response constrained to: .>0 and .<=100 or .=999* | |

| Field | Question | Answer | |
|---|---|---|---|
| q504_M16b *(required)* | q504_M16b. How is the item administered, or given, to the patient?<br>*Question relevant when: selected( ${q104_M16_full_list} , '4')* | 1 | Oral - solid (e.g. tablet) |
| | | 2 | Oral - liquid |
| | | 3 | Sublingual |
| | | 4 | Intravenous (IV) |
| | | 5 | Intramusculuar injection (IM) |
| | | 6 | Subcutaneous |
| | | 7 | Rectal |
| | | 8 | Inhaled (e.g. gas) |
| | | 9 | Inserted (e.g. vaginal pessaries) |
| | | 10 | Topical - cream |
| | | 11 | Topical - drops (e.g. eye drops) |
| | | 12 | Other |
| | | 99 | Don't know |
| q504_M16c *(required)* | q504_M16c. What is the item's smallest unit of measurement (mg, mcg, ml, g, etc)?<br>*Question relevant when: selected( ${q104_M16_full_list} , '4')* | microgram | microgram (mcg) |
| | | miligram | miligram (mg) |
| | | miligram_dissolved_in_liquid | miligram (mg) dissolved in liquid (e.g. 2 mg per 3 militers) |
| | | gram | gram (g) |
| | | militer | militer (ml) |
| | | centiliter | centiliter (cl) |
| | | litre | litre (l) |
| | | ml_of _solution | ml of solution (e.g. ml of 10% solution) |
| | | litre_of_solution | litre of solution (e.g. 1 litre of 5% solution) |
| | | cubic_meter | cubic meter (m^3) |
| | | other | other |
| | | dont_know | don't know |
| q504_M16c.1 | q504_M16c.1 If other unit of measurement or route of administration, specify here:<br>*Leave blank if not applicable*<br>*Question relevant when: selected( ${q104_M16_full_list} , '4')* | | |
| q504_M16d *(required)* | q504_M16d. In total, how many units are used to treat a patient during all of her care?<br>*Remember to add all units! E.g. 5 days x 200 mg twice per day = 2,000mg*<br>*Question relevant when: selected( ${q104_M16_full_list} , '4')*<br>*Response constrained to: .>0* | | |

| Field | Question | Answer |
|---|---|---|
| q504_M17 | ***q504_M17. Propofol (DiprVan), 10mg/ml***<br>*Question relevant when: selected( ${q104_M17_full_list} , '4')* | |
| q504_M17a *(required)* | q504_M17a. What percent of patients require this item?<br>*Question relevant when: selected( ${q104_M17_full_list} , '4')*<br>*Response constrained to: .>0 and .<=100 or .=999* | |
| q504_M17b *(required)* | q504_M17b. How is the item administered, or given, to the patient?<br>*Question relevant when: selected( ${q104_M17_full_list} , '4')* | 1 Oral - solid (e.g. tablet)<br>2 Oral - liquid<br>3 Sublingual<br>4 Intravenous (IV)<br>5 Intramusculuar injection (IM)<br>6 Subcutaneous<br>7 Rectal<br>8 Inhaled (e.g. gas)<br>9 Inserted (e.g. vaginal pessaries)<br>10 Topical - cream<br>11 Topical - drops (e.g. eye drops)<br>12 Other<br>99 Don't know |
| q504_M17c *(required)* | q504_M17c. What is the item's smallest unit of measurement (mg, mcg, ml, g, etc)?<br>*Question relevant when: selected( ${q104_M17_full_list} , '4')* | microgram — microgram (mcg)<br>miligram — miligram (mg)<br>miligram_dissolved_in_liquid — miligram (mg) dissolved in liquid (e.g. 2 mg per 3 militers)<br>gram — gram (g)<br>militer — militer (ml)<br>centiliter — centiliter (cl)<br>litre — litre (l)<br>ml_of _solution — ml of solution (e.g. ml of 10% solution)<br>litre_of_solution — litre of solution (e.g. 1 litre of 5% solution)<br>cubic_meter — cubic meter (m^3)<br>other — other<br>dont_know — don't know |
| q504_M17c.1 | q504_M17c.1 If other unit of measurement or route of administration, specify here:<br>*Leave blank if not applicable*<br>*Question relevant when: selected( ${q104_M17_full_list} , '4')* | |
| q504_M17d *(required)* | q504_M17d. In total, how many units are used to treat a patient during all of her care?<br>*Remember to add all units! E.g. 5 days x 200 mg twice per day = 2,000mg*<br>*Question relevant when: selected( ${q104_M17_full_list} , '4')*<br>*Response constrained to: .>0* | |

| Field | Question | Answer |
|---|---|---|
| q504_M18 | ***q504_M18. Suxamethonium chloride*** *Question relevant when: selected( ${q104_M18_full_list} , '4')* | |
| q504_M18a *(required)* | q504_M18a. What percent of patients require this item? *Question relevant when: selected( ${q104_M18_full_list} , '4')* *Response constrained to: .>0 and .<=100 or .=999* | |
| q504_M18b *(required)* | q504_M18b. How is the item administered, or given, to the patient? *Question relevant when: selected( ${q104_M18_full_list} , '4')* | 1 Oral - solid (e.g. tablet)<br>2 Oral - liquid<br>3 Sublingual<br>4 Intravenous (IV)<br>5 Intramusculuar injection (IM)<br>6 Subcutaneous<br>7 Rectal<br>8 Inhaled (e.g. gas)<br>9 Inserted (e.g. vaginal pessaries)<br>10 Topical - cream<br>11 Topical - drops (e.g. eye drops)<br>12 Other<br>99 Don't know |
| q504_M18c *(required)* | q504_M18c. What is the item's smallest unit of measurement (mg, mcg, ml, g, etc)? *Question relevant when: selected( ${q104_M18_full_list} , '4')* | microgram: microgram (mcg)<br>miligram: miligram (mg)<br>miligram_dissolved_in_liquid: miligram (mg) dissolved in liquid (e.g. 2 mg per 3 militers)<br>gram: gram (g)<br>militer: militer (ml)<br>centiliter: centiliter (cl)<br>litre: litre (l)<br>ml_of _solution: ml of solution (e.g. ml of 10% solution)<br>litre_of_solution: litre of solution (e.g. 1 litre of 5% solution)<br>cubic_meter: cubic meter (m^3)<br>other: other<br>dont_know: don't know |
| q504_M18c.1 | q504_M18c.1 If other unit of measurement or route of administration, specify here: *Leave blank if not applicable* *Question relevant when: selected( ${q104_M18_full_list} , '4')* | |
| q504_M18d *(required)* | q504_M18d. In total, how many units are used to treat a patient during all of her care? *Remember to add all units! E.g. 5 days x 200 mg twice per day = 2,000mg* *Question relevant when: selected( ${q104_M18_full_list} , '4')* *Response constrained to: .>0* | |

| Field | Question | Answer |
|---|---|---|
| q504_M19 | ***q504_M19. Thiopental sodium (Intraval)***<br>*Question relevant when: selected( ${q104_M19_full_list} , '4')* | |
| q504_M19a *(required)* | q504_M19a. What percent of patients require this item?<br>*Question relevant when: selected( ${q104_M19_full_list} , '4')*<br>*Response constrained to: .>0 and .<=100 or .=999* | |
| q504_M19b *(required)* | q504_M19b. How is the item administered, or given, to the patient?<br>*Question relevant when: selected( ${q104_M19_full_list} , '4')* | 1 Oral - solid (e.g. tablet)<br>2 Oral - liquid<br>3 Sublingual<br>4 Intravenous (IV)<br>5 Intramusculuar injection (IM)<br>6 Subcutaneous<br>7 Rectal<br>8 Inhaled (e.g. gas)<br>9 Inserted (e.g. vaginal pessaries)<br>10 Topical - cream<br>11 Topical - drops (e.g. eye drops)<br>12 Other<br>99 Don't know |
| q504_M19c *(required)* | q504_M19c. What is the item's smallest unit of measurement (mg, mcg, ml, g, etc)?<br>*Question relevant when: selected( ${q104_M19_full_list} , '4')* | microgram — microgram (mcg)<br>miligram — miligram (mg)<br>miligram_dissolved_in_liquid — miligram (mg) dissolved in liquid (e.g. 2 mg per 3 militers)<br>gram — gram (g)<br>militer — militer (ml)<br>centiliter — centiliter (cl)<br>litre — litre (l)<br>ml_of _solution — ml of solution (e.g. ml of 10% solution)<br>litre_of_solution — litre of solution (e.g. 1 litre of 5% solution)<br>cubic_meter — cubic meter (m^3)<br>other — other<br>dont_know — don't know |
| q504_M19c.1 | q504_M19c.1 If other unit of measurement or route of administration, specify here:<br>*Leave blank if not applicable*<br>*Question relevant when: selected( ${q104_M19_full_list} , '4')* | |
| q504_M19d *(required)* | q504_M19d. In total, how many units are used to treat a patient during all of her care?<br>*Remember to add all units! E.g. 5 days x 200 mg twice per day = 2,000mg*<br>*Question relevant when: selected( ${q104_M19_full_list} , '4')*<br>*Response constrained to: .>0* | |

| Field | ***q504_M20. Oxygen*** Question *Question relevant when: selected( ${q104_M20_full_list} , '4')* | Answer |
|---|---|---|
| q504_M20a *(required)* q504_M20 | q504_M20a. What percent of patients require this item?<br>*Question relevant when: selected( ${q104_M20_full_list} , '4')*<br>*Response constrained to: .>0 and .<=100 or .=999* | |

| Field | Question | Answer |
|---|---|---|
| q504_M20b *(required)* | q504_M20b. How is the item administered, or given, to the patient?<br>*Question relevant when: selected( ${q104_M20_full_list} , '4')* | 1 Oral - solid (e.g. tablet)<br>2 Oral - liquid<br>3 Sublingual<br>4 Intravenous (IV)<br>5 Intramusculuar injection (IM)<br>6 Subcutaneous<br>7 Rectal<br>8 Inhaled (e.g. gas)<br>9 Inserted (e.g. vaginal pessaries)<br>10 Topical - cream<br>11 Topical - drops (e.g. eye drops)<br>12 Other<br>99 Don't know |
| q504_M20c *(required)* | q504_M20c. What is the item's smallest unit of measurement (mg, mcg, ml, g, etc)?<br>*Question relevant when: selected( ${q104_M20_full_list} , '4')* | microgram — microgram (mcg)<br>miligram — miligram (mg)<br>miligram_dissolved_in_liquid — miligram (mg) dissolved in liquid (e.g. 2 mg per 3 militers)<br>gram — gram (g)<br>militer — militer (ml)<br>centiliter — centiliter (cl)<br>litre — litre (l)<br>ml_of _solution — ml of solution (e.g. ml of 10% solution)<br>litre_of_solution — litre of solution (e.g. 1 litre of 5% solution)<br>cubic_meter — cubic meter (m^3)<br>other — other<br>dont_know — don't know |
| q504_M20c.1 | q504_M20c.1 If other unit of measurement or route of administration, specify here:<br>*Leave blank if not applicable*<br>*Question relevant when: selected( ${q104_M20_full_list} , '4')* | |
| q504_M20d *(required)* | q504_M20d. In total, how many units are used to treat a patient during all of her care?<br>*Remember to add all units! E.g. 5 days x 200 mg twice per day = 2,000mg*<br>*Question relevant when: selected( ${q104_M20_full_list} , '4')*<br>*Response constrained to: .>0* | |

| Field | Question | Answer |
| --- | --- | --- |
| q504_M21 | ***q504_M21. Bupivacaine HCL 0.5% (Marcaine)***<br>*Question relevant when: selected( ${q104_M21_full_list} , '4')* | |
| q504_M21a *(required)* | q504_M21a. What percent of patients require this item?<br>*Question relevant when: selected( ${q104_M21_full_list} , '4')*<br>*Response constrained to: .>0 and .<=100 or .=999* | |
| q504_M21b *(required)* | q504_M21b. How is the item administered, or given, to the patient?<br>*Question relevant when: selected( ${q104_M21_full_list} , '4')* | 1 Oral - solid (e.g. tablet)<br>2 Oral - liquid<br>3 Sublingual<br>4 Intravenous (IV)<br>5 Intramusculuar injection (IM)<br>6 Subcutaneous<br>7 Rectal<br>8 Inhaled (e.g. gas)<br>9 Inserted (e.g. vaginal pessaries)<br>10 Topical - cream<br>11 Topical - drops (e.g. eye drops)<br>12 Other<br>99 Don't know |
| q504_M21c *(required)* | q504_M21c. What is the item's smallest unit of measurement (mg, mcg, ml, g, etc)?<br>*Question relevant when: selected( ${q104_M21_full_list} , '4')* | microgram — microgram (mcg)<br>miligram — miligram (mg)<br>miligram_dissolved_in_liquid — miligram (mg) dissolved in liquid (e.g. 2 mg per 3 militers)<br>gram — gram (g)<br>militer — militer (ml)<br>centiliter — centiliter (cl)<br>litre — litre (l)<br>ml_of _solution — ml of solution (e.g. ml of 10% solution)<br>litre_of_solution — litre of solution (e.g. 1 litre of 5% solution)<br>cubic_meter — cubic meter (m^3)<br>other — other<br>dont_know — don't know |
| q504_M21c.1 | q504_M21c.1 If other unit of measurement or route of administration, specify here:<br>*Leave blank if not applicable*<br>*Question relevant when: selected( ${q104_M21_full_list} , '4')* | |
| q504_M21d *(required)* | q504_M21d. In total, how many units are used to treat a patient during all of her care?<br>*Remember to add all units! E.g. 5 days x 200 mg twice per day = 2,000mg*<br>*Question relevant when: selected( ${q104_M21_full_list} , '4')*<br>*Response constrained to: .>0* | |

| Field | Question | Answer |
|---|---|---|
| q504_M22 | ***q504_M22. Lidocaine HCl 2% + epinephrin 1:100,000***<br>*Question relevant when: selected( ${q104_M22_full_list} , '4')* | |
| q504_M22a *(required)* | q504_M22a. What percent of patients require this item?<br>*Question relevant when: selected( ${q104_M22_full_list} , '4')*<br>*Response constrained to: .>0 and .<=100 or .=999* | |
| q504_M22b *(required)* | q504_M22b. How is the item administered, or given, to the patient?<br>*Question relevant when: selected( ${q104_M22_full_list} , '4')* | 1 Oral - solid (e.g. tablet)<br>2 Oral - liquid<br>3 Sublingual<br>4 Intravenous (IV)<br>5 Intramusculuar injection (IM)<br>6 Subcutaneous<br>7 Rectal<br>8 Inhaled (e.g. gas)<br>9 Inserted (e.g. vaginal pessaries)<br>10 Topical - cream<br>11 Topical - drops (e.g. eye drops)<br>12 Other<br>99 Don't know |
| q504_M22c *(required)* | q504_M22c. What is the item's smallest unit of measurement (mg, mcg, ml, g, etc)?<br>*Question relevant when: selected( ${q104_M22_full_list} , '4')* | microgram — microgram (mcg)<br>miligram — miligram (mg)<br>miligram_dissolved_in_liquid — miligram (mg) dissolved in liquid (e.g. 2 mg per 3 militers)<br>gram — gram (g)<br>militer — militer (ml)<br>centiliter — centiliter (cl)<br>litre — litre (l)<br>ml_of _solution — ml of solution (e.g. ml of 10% solution)<br>litre_of_solution — litre of solution (e.g. 1 litre of 5% solution)<br>cubic_meter — cubic meter (m^3)<br>other — other<br>dont_know — don't know |
| q504_M22c.1 | q504_M22c.1 If other unit of measurement or route of administration, specify here:<br>*Leave blank if not applicable*<br>*Question relevant when: selected( ${q104_M22_full_list} , '4')* | |
| q504_M22d *(required)* | q504_M22d. In total, how many units are used to treat a patient during all of her care?<br>*Remember to add all units! E.g. 5 days x 200 mg twice per day = 2,000mg*<br>*Question relevant when: selected( ${q104_M22_full_list} , '4')*<br>*Response constrained to: .>0* | |

| Field | Question | Answer |
|---|---|---|
| q504_M23 | ***q504_M23. Lidocaine HCl 5% + dextrose 7.5%***<br>*Question relevant when: selected( ${q104_M23_full_list} , '4')* | |
| q504_M23a *(required)* | q504_M23a. What percent of patients require this item?<br>*Question relevant when: selected( ${q104_M23_full_list} , '4')*<br>*Response constrained to: .>0 and .<=100 or .=999* | |
| q504_M23b *(required)* | q504_M23b. How is the item administered, or given, to the patient?<br>*Question relevant when: selected( ${q104_M23_full_list} , '4')* | 1 Oral - solid (e.g. tablet)<br>2 Oral - liquid<br>3 Sublingual<br>4 Intravenous (IV)<br>5 Intramusculuar injection (IM)<br>6 Subcutaneous<br>7 Rectal<br>8 Inhaled (e.g. gas)<br>9 Inserted (e.g. vaginal pessaries)<br>10 Topical - cream<br>11 Topical - drops (e.g. eye drops)<br>12 Other<br>99 Don't know |
| q504_M23c *(required)* | q504_M23c. What is the item's smallest unit of measurement (mg, mcg, ml, g, etc)?<br>*Question relevant when: selected( ${q104_M23_full_list} , '4')* | microgram — microgram (mcg)<br>miligram — miligram (mg)<br>miligram_dissolved_in_liquid — miligram (mg) dissolved in liquid (e.g. 2 mg per 3 militers)<br>gram — gram (g)<br>militer — militer (ml)<br>centiliter — centiliter (cl)<br>litre — litre (l)<br>ml_of _solution — ml of solution (e.g. ml of 10% solution)<br>litre_of_solution — litre of solution (e.g. 1 litre of 5% solution)<br>cubic_meter — cubic meter (m^3)<br>other — other<br>dont_know — don't know |
| q504_M23c.1 | q504_M23c.1 If other unit of measurement or route of administration, specify here:<br>*Leave blank if not applicable*<br>*Question relevant when: selected( ${q104_M23_full_list} , '4')* | |
| q504_M23d *(required)* | q504_M23d. In total, how many units are used to treat a patient during all of her care?<br>*Remember to add all units! E.g. 5 days x 200 mg twice per day = 2,000mg*<br>*Question relevant when: selected( ${q104_M23_full_list} , '4')*<br>*Response constrained to: .>0* | |

| Field | Question | Answer |
|---|---|---|
| q504_M24 | ***q504_M24. Lidocaine HCl, 1%*** <br> *Question relevant when: selected( ${q104_M24_full_list} , '4')* | |
| q504_M24a *(required)* | q504_M24a. What percent of patients require this item? <br> *Question relevant when: selected( ${q104_M24_full_list} , '4')* <br> *Response constrained to: .>0 and .<=100 or .=999* | |
| q504_M24b *(required)* | q504_M24b. How is the item administered, or given, to the patient? <br> *Question relevant when: selected( ${q104_M24_full_list} , '4')* | 1 Oral - solid (e.g. tablet) <br> 2 Oral - liquid <br> 3 Sublingual <br> 4 Intravenous (IV) <br> 5 Intramusculuar injection (IM) <br> 6 Subcutaneous <br> 7 Rectal <br> 8 Inhaled (e.g. gas) <br> 9 Inserted (e.g. vaginal pessaries) <br> 10 Topical - cream <br> 11 Topical - drops (e.g. eye drops) <br> 12 Other <br> 99 Don't know |
| q504_M24c *(required)* | q504_M24c. What is the item's smallest unit of measurement (mg, mcg, ml, g, etc)? <br> *Question relevant when: selected( ${q104_M24_full_list} , '4')* | microgram — microgram (mcg) <br> miligram — miligram (mg) <br> miligram_dissolved_in_liquid — miligram (mg) dissolved in liquid (e.g. 2 mg per 3 militers) <br> gram — gram (g) <br> militer — militer (ml) <br> centiliter — centiliter (cl) <br> litre — litre (l) <br> ml_of _solution — ml of solution (e.g. ml of 10% solution) <br> litre_of_solution — litre of solution (e.g. 1 litre of 5% solution) <br> cubic_meter — cubic meter (m^3) <br> other — other <br> dont_know — don't know |
| q504_M24c.1 | q504_M24c.1 If other unit of measurement or route of administration, specify here: <br> *Leave blank if not applicable* <br> *Question relevant when: selected( ${q104_M24_full_list} , '4')* | |
| q504_M24d *(required)* | q504_M24d. In total, how many units are used to treat a patient during all of her care? <br> *Remember to add all units! E.g. 5 days x 200 mg twice per day = 2,000mg* <br> *Question relevant when: selected( ${q104_M24_full_list} , '4')* <br> *Response constrained to: .>0* | |

| Field | Question | Answer |
|---|---|---|
| q504_M25 | ***q504_M25. Anesthetic - other 1: "[q104_M25_full_list_other]"*** *Question relevant when: selected( ${q104_M25_full_list} , '4')* | |
| q504_M25a *(required)* | q504_M25a. What percent of patients require this item? *Question relevant when: selected( ${q104_M25_full_list} , '4')* *Response constrained to: .>0 and .<=100 or .=999* | |

| Field | Question | | | Answer |
|---|---|---|---|---|
| q504_M25b *(required)* | q504_M25b. How is the item administered, or given, to the patient? *Question relevant when: selected( ${q104_M25_full_list} , '4')* | | 1 | Oral - solid (e.g. tablet) |
| | | | 2 | Oral - liquid |
| | | | 3 | Sublingual |
| | | | 4 | Intravenous (IV) |
| | | | 5 | Intramusculuar injection (IM) |
| | | | 6 | Subcutaneous |
| | | | 7 | Rectal |
| | | | 8 | Inhaled (e.g. gas) |
| | | | 9 | Inserted (e.g. vaginal pessaries) |
| | | | 10 | Topical - cream |
| | | | 11 | Topical - drops (e.g. eye drops) |
| | | | 12 | Other |
| | | | 99 | Don't know |
| q504_M25c *(required)* | q504_M25c. What is the item's smallest unit of measurement (mg, mcg, ml, g, etc)? *Question relevant when: selected( ${q104_M25_full_list} , '4')* | | microgram | microgram (mcg) |
| | | | miligram | miligram (mg) |
| | | | miligram_dissolved_in_liquid | miligram (mg) dissolved in liquid (e.g. 2 mg per 3 militers) |
| | | | gram | gram (g) |
| | | | militer | militer (ml) |
| | | | centiliter | centiliter (cl) |
| | | | litre | litre (l) |
| | | | ml_of _solution | ml of solution (e.g. ml of 10% solution) |
| | | | litre_of_solution | litre of solution (e.g. 1 litre of 5% solution) |
| | | | cubic_meter | cubic meter (m^3) |
| | | | other | other |
| | | | dont_know | don't know |
| q504_M25c.1 | q504_M25c.1 If other unit of measurement or route of administration, specify here: *Leave blank if not applicable* *Question relevant when: selected( ${q104_M25_full_list} , '4')* | | | |
| q504_M25d *(required)* | q504_M25d. In total, how many units are used to treat a patient during all of her care? *Remember to add all units! E.g. 5 days x 200 mg twice per day = 2,000mg* *Question relevant when: selected( ${q104_M25_full_list} , '4')* *Response constrained to: .>0* | | | |

| Field | Question | Answer |
|---|---|---|
| q504_M26 | ***q504_M26. Anesthetic - other 2: "[q104_M26_full_list_other]"*** *Question relevant when: selected( ${q104_M26_full_list} , '4')* | |
| q504_M26a *(required)* | q504_M26a. What percent of patients require this item? *Question relevant when: selected( ${q104_M26_full_list} , '4')* *Response constrained to: .>0 and .<=100 or .=999* | |

| Field | Question | Answer |
|---|---|---|
| q504_M26b *(required)* | q504_M26b. How is the item administered, or given, to the patient? *Question relevant when: selected( ${q104_M26_full_list} , '4')* | 1 Oral - solid (e.g. tablet)<br>2 Oral - liquid<br>3 Sublingual<br>4 Intravenous (IV)<br>5 Intramusculuar injection (IM)<br>6 Subcutaneous<br>7 Rectal<br>8 Inhaled (e.g. gas)<br>9 Inserted (e.g. vaginal pessaries)<br>10 Topical - cream<br>11 Topical - drops (e.g. eye drops)<br>12 Other<br>99 Don't know |
| q504_M26c *(required)* | q504_M26c. What is the item's smallest unit of measurement (mg, mcg, ml, g, etc)? *Question relevant when: selected( ${q104_M26_full_list} , '4')* | microgram — microgram (mcg)<br>miligram — miligram (mg)<br>miligram_dissolved_in_liquid — miligram (mg) dissolved in liquid (e.g. 2 mg per 3 militers)<br>gram — gram (g)<br>militer — militer (ml)<br>centiliter — centiliter (cl)<br>litre — litre (l)<br>ml_of _solution — ml of solution (e.g. ml of 10% solution)<br>litre_of_solution — litre of solution (e.g. 1 litre of 5% solution)<br>cubic_meter — cubic meter (m^3)<br>other — other<br>dont_know — don't know |
| q504_M26c.1 | q504_M26c.1 If other unit of measurement or route of administration, specify here: *Leave blank if not applicable* *Question relevant when: selected( ${q104_M26_full_list} , '4')* | |
| q504_M26d *(required)* | q504_M26d. In total, how many units are used to treat a patient during all of her care? *Remember to add all units! E.g. 5 days x 200 mg twice per day = 2,000mg* *Question relevant when: selected( ${q104_M26_full_list} , '4')* *Response constrained to: .>0* | |

| Field | Question | Answer |
|---|---|---|
| q504_M27 | ***q504_M27. Anesthetic - other 3: "[q104_M27_full_list_other]"***<br>*Question relevant when: selected( ${q104_M27_full_list} , '4')* | |
| q504_M27a *(required)* | q504_M27a. What percent of patients require this item?<br>*Question relevant when: selected( ${q104_M27_full_list} , '4')*<br>*Response constrained to: .>0 and .<=100 or .=999* | |
| q504_M27b *(required)* | q504_M27b. How is the item administered, or given, to the patient?<br>*Question relevant when: selected( ${q104_M27_full_list} , '4')* | 1 Oral - solid (e.g. tablet)<br>2 Oral - liquid<br>3 Sublingual<br>4 Intravenous (IV)<br>5 Intramusculuar injection (IM)<br>6 Subcutaneous<br>7 Rectal<br>8 Inhaled (e.g. gas)<br>9 Inserted (e.g. vaginal pessaries)<br>10 Topical - cream<br>11 Topical - drops (e.g. eye drops)<br>12 Other<br>99 Don't know |
| q504_M27c *(required)* | q504_M27c. What is the item's smallest unit of measurement (mg, mcg, ml, g, etc)?<br>*Question relevant when: selected( ${q104_M27_full_list} , '4')* | microgram — microgram (mcg)<br>miligram — miligram (mg)<br>miligram_dissolved_in_liquid — miligram (mg) dissolved in liquid (e.g. 2 mg per 3 militers)<br>gram — gram (g)<br>militer — militer (ml)<br>centiliter — centiliter (cl)<br>litre — litre (l)<br>ml_of _solution — ml of solution (e.g. ml of 10% solution)<br>litre_of_solution — litre of solution (e.g. 1 litre of 5% solution)<br>cubic_meter — cubic meter (m^3)<br>other — other<br>dont_know — don't know |
| q504_M27c.1 | q504_M27c.1 If other unit of measurement or route of administration, specify here:<br>*Leave blank if not applicable*<br>*Question relevant when: selected( ${q104_M27_full_list} , '4')* | |
| q504_M27d *(required)* | q504_M27d. In total, how many units are used to treat a patient during all of her care?<br>*Remember to add all units! E.g. 5 days x 200 mg twice per day = 2,000mg*<br>*Question relevant when: selected( ${q104_M27_full_list} , '4')*<br>*Response constrained to: .>0* | |

| Field | Question | Answer |
|---|---|---|
| q504_M28 | ***q504_M28. Anesthetic - other 4: "[q104_M28_full_list_other]"*** <br> *Question relevant when: selected( ${q104_M28_full_list} , '4')* | |
| q504_M28a *(required)* | q504_M28a. What percent of patients require this item? <br> *Question relevant when: selected( ${q104_M28_full_list} , '4')* <br> *Response constrained to: .>0 and .<=100 or .=999* | |
| q504_M28b *(required)* | q504_M28b. How is the item administered, or given, to the patient? <br> *Question relevant when: selected( ${q104_M28_full_list} , '4')* | 1 Oral - solid (e.g. tablet)<br>2 Oral - liquid<br>3 Sublingual<br>4 Intravenous (IV)<br>5 Intramusculuar injection (IM)<br>6 Subcutaneous<br>7 Rectal<br>8 Inhaled (e.g. gas)<br>9 Inserted (e.g. vaginal pessaries)<br>10 Topical - cream<br>11 Topical - drops (e.g. eye drops)<br>12 Other<br>99 Don't know |
| q504_M28c *(required)* | q504_M28c. What is the item's smallest unit of measurement (mg, mcg, ml, g, etc)? <br> *Question relevant when: selected( ${q104_M28_full_list} , '4')* | microgram — microgram (mcg)<br>miligram — miligram (mg)<br>miligram_dissolved_in_liquid — miligram (mg) dissolved in liquid (e.g. 2 mg per 3 militers)<br>gram — gram (g)<br>militer — militer (ml)<br>centiliter — centiliter (cl)<br>litre — litre (l)<br>ml_of _solution — ml of solution (e.g. ml of 10% solution)<br>litre_of_solution — litre of solution (e.g. 1 litre of 5% solution)<br>cubic_meter — cubic meter (m^3)<br>other — other<br>dont_know — don't know |
| q504_M28c.1 | q504_M28c.1 If other unit of measurement or route of administration, specify here: <br> *Leave blank if not applicable* <br> *Question relevant when: selected( ${q104_M28_full_list} , '4')* | |
| q504_M28d *(required)* | q504_M28d. In total, how many units are used to treat a patient during all of her care? <br> *Remember to add all units! E.g. 5 days x 200 mg twice per day = 2,000mg* <br> *Question relevant when: selected( ${q104_M28_full_list} , '4')* <br> *Response constrained to: .>0* | |

| Field | Question | Answer |
|---|---|---|
| q504_M29 | ***q504_M29. Anesthetic - other 5: "[q104_M29_full_list_other]"*** *Question relevant when: selected( ${q104_M29_full_list} , '4')* | |
| q504_M29a *(required)* | q504_M29a. What percent of patients require this item? *Question relevant when: selected( ${q104_M29_full_list} , '4')* *Response constrained to: .>0 and .<=100 or .=999* | |
| q504_M29b *(required)* | q504_M29b. How is the item administered, or given, to the patient? *Question relevant when: selected( ${q104_M29_full_list} , '4')* | 1 Oral - solid (e.g. tablet)<br>2 Oral - liquid<br>3 Sublingual<br>4 Intravenous (IV)<br>5 Intramusculuar injection (IM)<br>6 Subcutaneous<br>7 Rectal<br>8 Inhaled (e.g. gas)<br>9 Inserted (e.g. vaginal pessaries)<br>10 Topical - cream<br>11 Topical - drops (e.g. eye drops)<br>12 Other<br>99 Don't know |
| q504_M29c *(required)* | q504_M29c. What is the item's smallest unit of measurement (mg, mcg, ml, g, etc)? *Question relevant when: selected( ${q104_M29_full_list} , '4')* | microgram — microgram (mcg)<br>miligram — miligram (mg)<br>miligram_dissolved_in_liquid — miligram (mg) dissolved in liquid (e.g. 2 mg per 3 militers)<br>gram — gram (g)<br>militer — militer (ml)<br>centiliter — centiliter (cl)<br>litre — litre (l)<br>ml_of _solution — ml of solution (e.g. ml of 10% solution)<br>litre_of_solution — litre of solution (e.g. 1 litre of 5% solution)<br>cubic_meter — cubic meter (m^3)<br>other — other<br>dont_know — don't know |
| q504_M29c.1 | q504_M29c.1 If other unit of measurement or route of administration, specify here: *Leave blank if not applicable* *Question relevant when: selected( ${q104_M29_full_list} , '4')* | |
| q504_M29d *(required)* | q504_M29d. In total, how many units are used to treat a patient during all of her care? *Remember to add all units! E.g. 5 days x 200 mg twice per day = 2,000mg* *Question relevant when: selected( ${q104_M29_full_list} , '4')* *Response constrained to: .>0* | |

<p><strong><em>D. Medications - Lacerations (3)</em></strong></p>
*Group relevant when: selected( ${section_five_skip_med} , '1')*

| Field | Question | Answer |
|---|---|---|
| q504_med_note3 | 504 CONTINUED For each of the following medications and other medical products that are used for lacerations, can you tell me …? | |
| note_504_antibiotics | ***Antibiotics*** | |
| q504_M30 | ***q504_M30. Amoxicillin***<br>*Question relevant when: selected( ${q104_M30_full_list} , '4')* | |
| q504_M30a *(required)* | q504_M30a. What percent of patients require this item?<br>*Question relevant when: selected( ${q104_M30_full_list} , '4')*<br>*Response constrained to: .>0 and .<=100 or .=999* | |

| Field | Question | Answer | |
|---|---|---|---|
| q504_M30b *(required)* | q504_M30b. How is the item administered, or given, to the patient?<br>*Question relevant when: selected( ${q104_M30_full_list} , '4')* | 1 | Oral - solid (e.g. tablet) |
| | | 2 | Oral - liquid |
| | | 3 | Sublingual |
| | | 4 | Intravenous (IV) |
| | | 5 | Intramusculuar injection (IM) |
| | | 6 | Subcutaneous |
| | | 7 | Rectal |
| | | 8 | Inhaled (e.g. gas) |
| | | 9 | Inserted (e.g. vaginal pessaries) |
| | | 10 | Topical - cream |
| | | 11 | Topical - drops (e.g. eye drops) |
| | | 12 | Other |
| | | 99 | Don't know |
| q504_M30c *(required)* | q504_M30c. What is the item's smallest unit of measurement (mg, mcg, ml, g, etc)?<br>*Question relevant when: selected( ${q104_M30_full_list} , '4')* | microgram | microgram (mcg) |
| | | miligram | miligram (mg) |
| | | miligram_dissolved_in_liquid | miligram (mg) dissolved in liquid (e.g. 2 mg per 3 militers) |
| | | gram | gram (g) |
| | | militer | militer (ml) |
| | | centiliter | centiliter (cl) |
| | | litre | litre (l) |
| | | ml_of _solution | ml of solution (e.g. ml of 10% solution) |
| | | litre_of_solution | litre of solution (e.g. 1 litre of 5% solution) |
| | | cubic_meter | cubic meter (m^3) |
| | | other | other |
| | | dont_know | don't know |
| q504_M30c.1 | q504_M30c.1 If other unit of measurement or route of administration, specify here:<br>*Leave blank if not applicable*<br>*Question relevant when: selected( ${q104_M30_full_list} , '4')* | | |
| q504_M30d *(required)* | q504_M30d. In total, how many units are used to treat a patient during all of her care?<br>*Remember to add all units! E.g. 5 days x 200 mg twice per day = 2,000mg*<br>*Question relevant when: selected( ${q104_M30_full_list} , '4')*<br>*Response constrained to: .>0* | | |

| Field | Question | Answer |
|---|---|---|
| q504_M31 | ***q504_M31. Amoxicillin + Clauvulenic Acid 635mg***<br>*Question relevant when: selected( ${q104_M31_full_list} , '4')* | |
| q504_M31a *(required)* | q504_M31a. What percent of patients require this item?<br>*Question relevant when: selected( ${q104_M31_full_list} , '4')*<br>*Response constrained to: .>0 and .<=100 or .=999* | |
| q504_M31b *(required)* | q504_M31b. How is the item administered, or given, to the patient?<br>*Question relevant when: selected( ${q104_M31_full_list} , '4')* | 1 Oral - solid (e.g. tablet)<br>2 Oral - liquid<br>3 Sublingual<br>4 Intravenous (IV)<br>5 Intramusculuar injection (IM)<br>6 Subcutaneous<br>7 Rectal<br>8 Inhaled (e.g. gas)<br>9 Inserted (e.g. vaginal pessaries)<br>10 Topical - cream<br>11 Topical - drops (e.g. eye drops)<br>12 Other<br>99 Don't know |
| q504_M31c *(required)* | q504_M31c. What is the item's smallest unit of measurement (mg, mcg, ml, g, etc)?<br>*Question relevant when: selected( ${q104_M31_full_list} , '4')* | microgram — microgram (mcg)<br>miligram — miligram (mg)<br>miligram_dissolved_in_liquid — miligram (mg) dissolved in liquid (e.g. 2 mg per 3 militers)<br>gram — gram (g)<br>militer — militer (ml)<br>centiliter — centiliter (cl)<br>litre — litre (l)<br>ml_of _solution — ml of solution (e.g. ml of 10% solution)<br>litre_of_solution — litre of solution (e.g. 1 litre of 5% solution)<br>cubic_meter — cubic meter (m^3)<br>other — other<br>dont_know — don't know |
| q504_M31c.1 | q504_M31c.1 If other unit of measurement or route of administration, specify here:<br>*Leave blank if not applicable*<br>*Question relevant when: selected( ${q104_M31_full_list} , '4')* | |
| q504_M31d *(required)* | q504_M31d. In total, how many units are used to treat a patient during all of her care?<br>*Remember to add all units! E.g. 5 days x 200 mg twice per day = 2,000mg*<br>*Question relevant when: selected( ${q104_M31_full_list} , '4')*<br>*Response constrained to: .>0* | |

| Field | Question | Answer |
|---|---|---|
| q504_M32 | ***q504_M32. Ampicillin***<br>*Question relevant when: selected( ${q104_M32_full_list} , '4')* | |
| q504_M32a *(required)* | q504_M32a. What percent of patients require this item?<br>*Question relevant when: selected( ${q104_M32_full_list} , '4')*<br>*Response constrained to: .>0 and .<=100 or .=999* | |
| q504_M32b *(required)* | q504_M32b. How is the item administered, or given, to the patient?<br>*Question relevant when: selected( ${q104_M32_full_list} , '4')* | 1 Oral - solid (e.g. tablet)<br>2 Oral - liquid<br>3 Sublingual<br>4 Intravenous (IV)<br>5 Intramusculuar injection (IM)<br>6 Subcutaneous<br>7 Rectal<br>8 Inhaled (e.g. gas)<br>9 Inserted (e.g. vaginal pessaries)<br>10 Topical - cream<br>11 Topical - drops (e.g. eye drops)<br>12 Other<br>99 Don't know |
| q504_M32c *(required)* | q504_M32c. What is the item's smallest unit of measurement (mg, mcg, ml, g, etc)?<br>*Question relevant when: selected( ${q104_M32_full_list} , '4')* | microgram — microgram (mcg)<br>miligram — miligram (mg)<br>miligram_dissolved_in_liquid — miligram (mg) dissolved in liquid (e.g. 2 mg per 3 militers)<br>gram — gram (g)<br>militer — militer (ml)<br>centiliter — centiliter (cl)<br>litre — litre (l)<br>ml_of _solution — ml of solution (e.g. ml of 10% solution)<br>litre_of_solution — litre of solution (e.g. 1 litre of 5% solution)<br>cubic_meter — cubic meter (m^3)<br>other — other<br>dont_know — don't know |
| q504_M32c.1 | q504_M32c.1 If other unit of measurement or route of administration, specify here:<br>*Leave blank if not applicable*<br>*Question relevant when: selected( ${q104_M32_full_list} , '4')* | |
| q504_M32d *(required)* | q504_M32d. In total, how many units are used to treat a patient during all of her care?<br>*Remember to add all units! E.g. 5 days x 200 mg twice per day = 2,000mg*<br>*Question relevant when: selected( ${q104_M32_full_list} , '4')*<br>*Response constrained to: .>0* | |

| Field | Question | Answer |
|---|---|---|
| q504_M33 | ***q504_M33. Ampicillin (injection)***<br>*Question relevant when: selected( ${q104_M33_full_list} , '4')* | |
| q504_M33a *(required)* | q504_M33a. What percent of patients require this item?<br>*Question relevant when: selected( ${q104_M33_full_list} , '4')*<br>*Response constrained to: .>0 and .<=100 or .=999* | |
| q504_M33b *(required)* | q504_M33b. How is the item administered, or given, to the patient?<br>*Question relevant when: selected( ${q104_M33_full_list} , '4')* | 1 Oral - solid (e.g. tablet)<br>2 Oral - liquid<br>3 Sublingual<br>4 Intravenous (IV)<br>5 Intramusculuar injection (IM)<br>6 Subcutaneous<br>7 Rectal<br>8 Inhaled (e.g. gas)<br>9 Inserted (e.g. vaginal pessaries)<br>10 Topical - cream<br>11 Topical - drops (e.g. eye drops)<br>12 Other<br>99 Don't know |
| q504_M33c *(required)* | q504_M33c. What is the item's smallest unit of measurement (mg, mcg, ml, g, etc)?<br>*Question relevant when: selected( ${q104_M33_full_list} , '4')* | microgram — microgram (mcg)<br>miligram — miligram (mg)<br>miligram_dissolved_in_liquid — miligram (mg) dissolved in liquid (e.g. 2 mg per 3 militers)<br>gram — gram (g)<br>militer — militer (ml)<br>centiliter — centiliter (cl)<br>litre — litre (l)<br>ml_of _solution — ml of solution (e.g. ml of 10% solution)<br>litre_of_solution — litre of solution (e.g. 1 litre of 5% solution)<br>cubic_meter — cubic meter (m^3)<br>other — other<br>dont_know — don't know |
| q504_M33c.1 | q504_M33c.1 If other unit of measurement or route of administration, specify here:<br>*Leave blank if not applicable*<br>*Question relevant when: selected( ${q104_M33_full_list} , '4')* | |
| q504_M33d *(required)* | q504_M33d. In total, how many units are used to treat a patient during all of her care?<br>*Remember to add all units! E.g. 5 days x 200 mg twice per day = 2,000mg*<br>*Question relevant when: selected( ${q104_M33_full_list} , '4')*<br>*Response constrained to: .>0* | |

| Field | Question | Answer |
|---|---|---|
| q504_M34 | ***q504_M34. Ampicillin/sulbactam (Unasyn)***<br>*Question relevant when: selected( ${q104_M34_full_list} , '4')* | |
| q504_M34a *(required)* | q504_M34a. What percent of patients require this item?<br>*Question relevant when: selected( ${q104_M34_full_list} , '4')*<br>*Response constrained to: .>0 and .<=100 or .=999* | |
| q504_M34b *(required)* | q504_M34b. How is the item administered, or given, to the patient?<br>*Question relevant when: selected( ${q104_M34_full_list} , '4')* | 1 Oral - solid (e.g. tablet)<br>2 Oral - liquid<br>3 Sublingual<br>4 Intravenous (IV)<br>5 Intramusculuar injection (IM)<br>6 Subcutaneous<br>7 Rectal<br>8 Inhaled (e.g. gas)<br>9 Inserted (e.g. vaginal pessaries)<br>10 Topical - cream<br>11 Topical - drops (e.g. eye drops)<br>12 Other<br>99 Don't know |
| q504_M34c *(required)* | q504_M34c. What is the item's smallest unit of measurement (mg, mcg, ml, g, etc)?<br>*Question relevant when: selected( ${q104_M34_full_list} , '4')* | microgram — microgram (mcg)<br>miligram — miligram (mg)<br>miligram_dissolved_in_liquid — miligram (mg) dissolved in liquid (e.g. 2 mg per 3 militers)<br>gram — gram (g)<br>militer — militer (ml)<br>centiliter — centiliter (cl)<br>litre — litre (l)<br>ml_of _solution — ml of solution (e.g. ml of 10% solution)<br>litre_of_solution — litre of solution (e.g. 1 litre of 5% solution)<br>cubic_meter — cubic meter (m^3)<br>other — other<br>dont_know — don't know |
| q504_M34c.1 | q504_M34c.1 If other unit of measurement or route of administration, specify here:<br>*Leave blank if not applicable*<br>*Question relevant when: selected( ${q104_M34_full_list} , '4')* | |
| q504_M34d *(required)* | q504_M34d. In total, how many units are used to treat a patient during all of her care?<br>*Remember to add all units! E.g. 5 days x 200 mg twice per day = 2,000mg*<br>*Question relevant when: selected( ${q104_M34_full_list} , '4')*<br>*Response constrained to: .>0* | |

| Field | Question | Answer |
|---|---|---|
| q504_M35 | ***q504_M35. Ceftriaxone***<br>*Question relevant when: selected( ${q104_M35_full_list} , '4')* | |
| q504_M35a *(required)* | q504_M35a. What percent of patients require this item?<br>*Question relevant when: selected( ${q104_M35_full_list} , '4')*<br>*Response constrained to: .>0 and .<=100 or .=999* | |
| q504_M35b *(required)* | q504_M35b. How is the item administered, or given, to the patient?<br>*Question relevant when: selected( ${q104_M35_full_list} , '4')* | 1 Oral - solid (e.g. tablet)<br>2 Oral - liquid<br>3 Sublingual<br>4 Intravenous (IV)<br>5 Intramusculuar injection (IM)<br>6 Subcutaneous<br>7 Rectal<br>8 Inhaled (e.g. gas)<br>9 Inserted (e.g. vaginal pessaries)<br>10 Topical - cream<br>11 Topical - drops (e.g. eye drops)<br>12 Other<br>99 Don't know |
| q504_M35c *(required)* | q504_M35c. What is the item's smallest unit of measurement (mg, mcg, ml, g, etc)?<br>*Question relevant when: selected( ${q104_M35_full_list} , '4')* | microgram — microgram (mcg)<br>miligram — miligram (mg)<br>miligram_dissolved_in_liquid — miligram (mg) dissolved in liquid (e.g. 2 mg per 3 militers)<br>gram — gram (g)<br>militer — militer (ml)<br>centiliter — centiliter (cl)<br>litre — litre (l)<br>ml_of _solution — ml of solution (e.g. ml of 10% solution)<br>litre_of_solution — litre of solution (e.g. 1 litre of 5% solution)<br>cubic_meter — cubic meter (m^3)<br>other — other<br>dont_know — don't know |
| q504_M35c.1 | q504_M35c.1 If other unit of measurement or route of administration, specify here:<br>*Leave blank if not applicable*<br>*Question relevant when: selected( ${q104_M35_full_list} , '4')* | |
| q504_M35d *(required)* | q504_M35d. In total, how many units are used to treat a patient during all of her care?<br>*Remember to add all units! E.g. 5 days x 200 mg twice per day = 2,000mg*<br>*Question relevant when: selected( ${q104_M35_full_list} , '4')*<br>*Response constrained to: .>0* | |

| Field | Question | Answer |
|---|---|---|
| q504_M36 | ***q504_M36. Chloramphenicol Sodium Succinate (injection)***<br>*Question relevant when: selected( ${q104_M36_full_list} , '4')* | |
| q504_M36a *(required)* | q504_M36a. What percent of patients require this item?<br>*Question relevant when: selected( ${q104_M36_full_list} , '4')*<br>*Response constrained to: .>0 and .<=100 or .=999* | |

| Field | Question | Answer |
|---|---|---|
| q504_M36b *(required)* | q504_M36b. How is the item administered, or given, to the patient?<br>*Question relevant when: selected( ${q104_M36_full_list} , '4')* | 1 Oral - solid (e.g. tablet)<br>2 Oral - liquid<br>3 Sublingual<br>4 Intravenous (IV)<br>5 Intramusculuar injection (IM)<br>6 Subcutaneous<br>7 Rectal<br>8 Inhaled (e.g. gas)<br>9 Inserted (e.g. vaginal pessaries)<br>10 Topical - cream<br>11 Topical - drops (e.g. eye drops)<br>12 Other<br>99 Don't know |
| q504_M36c *(required)* | q504_M36c. What is the item's smallest unit of measurement (mg, mcg, ml, g, etc)?<br>*Question relevant when: selected( ${q104_M36_full_list} , '4')* | microgram: microgram (mcg)<br>miligram: miligram (mg)<br>miligram_dissolved_in_liquid: miligram (mg) dissolved in liquid (e.g. 2 mg per 3 militers)<br>gram: gram (g)<br>militer: militer (ml)<br>centiliter: centiliter (cl)<br>litre: litre (l)<br>ml_of _solution: ml of solution (e.g. ml of 10% solution)<br>litre_of_solution: litre of solution (e.g. 1 litre of 5% solution)<br>cubic_meter: cubic meter (m^3)<br>other: other<br>dont_know: don't know |
| q504_M36c.1 | q504_M36c.1 If other unit of measurement or route of administration, specify here:<br>*Leave blank if not applicable*<br>*Question relevant when: selected( ${q104_M36_full_list} , '4')* | |
| q504_M36d *(required)* | q504_M36d. In total, how many units are used to treat a patient during all of her care?<br>*Remember to add all units! E.g. 5 days x 200 mg twice per day = 2,000mg*<br>*Question relevant when: selected( ${q104_M36_full_list} , '4')*<br>*Response constrained to: .>0* | |

| Field | Question | Answer |
|---|---|---|
| q504_M37 | ***q504_M37. Ciprofloxacin***<br>*Question relevant when: selected( ${q104_M37_full_list} , '4')* | |
| q504_M37a *(required)* | q504_M37a. What percent of patients require this item?<br>*Question relevant when: selected( ${q104_M37_full_list} , '4')*<br>*Response constrained to: .>0 and .<=100 or .=999* | |
| q504_M37b *(required)* | q504_M37b. How is the item administered, or given, to the patient?<br>*Question relevant when: selected( ${q104_M37_full_list} , '4')* | 1 Oral - solid (e.g. tablet)<br>2 Oral - liquid<br>3 Sublingual<br>4 Intravenous (IV)<br>5 Intramusculuar injection (IM)<br>6 Subcutaneous<br>7 Rectal<br>8 Inhaled (e.g. gas)<br>9 Inserted (e.g. vaginal pessaries)<br>10 Topical - cream<br>11 Topical - drops (e.g. eye drops)<br>12 Other<br>99 Don't know |
| q504_M37c *(required)* | q504_M37c. What is the item's smallest unit of measurement (mg, mcg, ml, g, etc)?<br>*Question relevant when: selected( ${q104_M37_full_list} , '4')* | microgram — microgram (mcg)<br>miligram — miligram (mg)<br>miligram_dissolved_in_liquid — miligram (mg) dissolved in liquid (e.g. 2 mg per 3 militers)<br>gram — gram (g)<br>militer — militer (ml)<br>centiliter — centiliter (cl)<br>litre — litre (l)<br>ml_of _solution — ml of solution (e.g. ml of 10% solution)<br>litre_of_solution — litre of solution (e.g. 1 litre of 5% solution)<br>cubic_meter — cubic meter (m^3)<br>other — other<br>dont_know — don't know |
| q504_M37c.1 | q504_M37c.1 If other unit of measurement or route of administration, specify here:<br>*Leave blank if not applicable*<br>*Question relevant when: selected( ${q104_M37_full_list} , '4')* | |
| q504_M37d *(required)* | q504_M37d. In total, how many units are used to treat a patient during all of her care?<br>*Remember to add all units! E.g. 5 days x 200 mg twice per day = 2,000mg*<br>*Question relevant when: selected( ${q104_M37_full_list} , '4')*<br>*Response constrained to: .>0* | |

| Field | Question | Answer |
|---|---|---|
| q504_M38 | ***q504_M38. Doxycycline*** <br> *Question relevant when: selected( ${q104_M38_full_list} , '4')* | |
| q504_M38a *(required)* | q504_M38a. What percent of patients require this item? <br> *Question relevant when: selected( ${q104_M38_full_list} , '4')* <br> *Response constrained to: .>0 and .<=100 or .=999* | |
| q504_M38b *(required)* | q504_M38b. How is the item administered, or given, to the patient? <br> *Question relevant when: selected( ${q104_M38_full_list} , '4')* | 1 Oral - solid (e.g. tablet) <br> 2 Oral - liquid <br> 3 Sublingual <br> 4 Intravenous (IV) <br> 5 Intramusculuar injection (IM) <br> 6 Subcutaneous <br> 7 Rectal <br> 8 Inhaled (e.g. gas) <br> 9 Inserted (e.g. vaginal pessaries) <br> 10 Topical - cream <br> 11 Topical - drops (e.g. eye drops) <br> 12 Other <br> 99 Don't know |
| q504_M38c *(required)* | q504_M38c. What is the item's smallest unit of measurement (mg, mcg, ml, g, etc)? <br> *Question relevant when: selected( ${q104_M38_full_list} , '4')* | microgram: microgram (mcg) <br> miligram: miligram (mg) <br> miligram_dissolved_in_liquid: miligram (mg) dissolved in liquid (e.g. 2 mg per 3 militers) <br> gram: gram (g) <br> militer: militer (ml) <br> centiliter: centiliter (cl) <br> litre: litre (l) <br> ml_of _solution: ml of solution (e.g. ml of 10% solution) <br> litre_of_solution: litre of solution (e.g. 1 litre of 5% solution) <br> cubic_meter: cubic meter (m^3) <br> other: other <br> dont_know: don't know |
| q504_M38c.1 | q504_M38c.1 If other unit of measurement or route of administration, specify here: <br> *Leave blank if not applicable* <br> *Question relevant when: selected( ${q104_M38_full_list} , '4')* | |
| q504_M38d *(required)* | q504_M38d. In total, how many units are used to treat a patient during all of her care? <br> *Remember to add all units! E.g. 5 days x 200 mg twice per day = 2,000mg* <br> *Question relevant when: selected( ${q104_M38_full_list} , '4')* <br> *Response constrained to: .>0* | |

| Field | Question | Answer |
|---|---|---|
| q504_M39 | ***q504_M39. Erythromycin***<br>*Question relevant when: selected( ${q104_M39_full_list} , '4')* | |
| q504_M39a *(required)* | q504_M39a. What percent of patients require this item?<br>*Question relevant when: selected( ${q104_M39_full_list} , '4')*<br>*Response constrained to: .>0 and .<=100 or .=999* | |

| Field | Question | Answer | | |
|---|---|---|---|---|
| q504_M39b *(required)* | q504_M39b. How is the item administered, or given, to the patient?<br>*Question relevant when: selected( ${q104_M39_full_list} , '4')* | | 1 | Oral - solid (e.g. tablet) |
| | | | 2 | Oral - liquid |
| | | | 3 | Sublingual |
| | | | 4 | Intravenous (IV) |
| | | | 5 | Intramusculuar injection (IM) |
| | | | 6 | Subcutaneous |
| | | | 7 | Rectal |
| | | | 8 | Inhaled (e.g. gas) |
| | | | 9 | Inserted (e.g. vaginal pessaries) |
| | | | 10 | Topical - cream |
| | | | 11 | Topical - drops (e.g. eye drops) |
| | | | 12 | Other |
| | | | 99 | Don't know |
| q504_M39c *(required)* | q504_M39c. What is the item's smallest unit of measurement (mg, mcg, ml, g, etc)?<br>*Question relevant when: selected( ${q104_M39_full_list} , '4')* | | microgram | microgram (mcg) |
| | | | miligram | miligram (mg) |
| | | | miligram_dissolved_in_liquid | miligram (mg) dissolved in liquid (e.g. 2 mg per 3 militers) |
| | | | gram | gram (g) |
| | | | militer | militer (ml) |
| | | | centiliter | centiliter (cl) |
| | | | litre | litre (l) |
| | | | ml_of _solution | ml of solution (e.g. ml of 10% solution) |
| | | | litre_of_solution | litre of solution (e.g. 1 litre of 5% solution) |
| | | | cubic_meter | cubic meter (m^3) |
| | | | other | other |
| | | | dont_know | don't know |
| q504_M39c.1 | q504_M39c.1 If other unit of measurement or route of administration, specify here:<br>*Leave blank if not applicable*<br>*Question relevant when: selected( ${q104_M39_full_list} , '4')* | | | |
| q504_M39d *(required)* | q504_M39d. In total, how many units are used to treat a patient during all of her care?<br>*Remember to add all units! E.g. 5 days x 200 mg twice per day = 2,000mg*<br>*Question relevant when: selected( ${q104_M39_full_list} , '4')*<br>*Response constrained to: .>0* | | | |

| Field | Question | Answer |
|---|---|---|
| q504_M40 | ***q504_M40. Gentamycin***<br>*Question relevant when: selected( ${q104_M40_full_list} , '4')* | |
| q504_M40a *(required)* | q504_M40a. What percent of patients require this item?<br>*Question relevant when: selected( ${q104_M40_full_list} , '4')*<br>*Response constrained to: .>0 and .<=100 or .=999* | |
| q504_M40b *(required)* | q504_M40b. How is the item administered, or given, to the patient?<br>*Question relevant when: selected( ${q104_M40_full_list} , '4')* | 1 Oral - solid (e.g. tablet)<br>2 Oral - liquid<br>3 Sublingual<br>4 Intravenous (IV)<br>5 Intramusculuar injection (IM)<br>6 Subcutaneous<br>7 Rectal<br>8 Inhaled (e.g. gas)<br>9 Inserted (e.g. vaginal pessaries)<br>10 Topical - cream<br>11 Topical - drops (e.g. eye drops)<br>12 Other<br>99 Don't know |
| q504_M40c *(required)* | q504_M40c. What is the item's smallest unit of measurement (mg, mcg, ml, g, etc)?<br>*Question relevant when: selected( ${q104_M40_full_list} , '4')* | microgram — microgram (mcg)<br>miligram — miligram (mg)<br>miligram_dissolved_in_liquid — miligram (mg) dissolved in liquid (e.g. 2 mg per 3 militers)<br>gram — gram (g)<br>militer — militer (ml)<br>centiliter — centiliter (cl)<br>litre — litre (l)<br>ml_of _solution — ml of solution (e.g. ml of 10% solution)<br>litre_of_solution — litre of solution (e.g. 1 litre of 5% solution)<br>cubic_meter — cubic meter (m^3)<br>other — other<br>dont_know — don't know |
| q504_M40c.1 | q504_M40c.1 If other unit of measurement or route of administration, specify here:<br>*Leave blank if not applicable*<br>*Question relevant when: selected( ${q104_M40_full_list} , '4')* | |
| q504_M40d *(required)* | q504_M40d. In total, how many units are used to treat a patient during all of her care?<br>*Remember to add all units! E.g. 5 days x 200 mg twice per day = 2,000mg*<br>*Question relevant when: selected( ${q104_M40_full_list} , '4')*<br>*Response constrained to: .>0* | |

| Field | Question | Answer |
|---|---|---|
| q504_M41 | ***q504_M41. Metronidazole (for infusion)***<br>*Question relevant when: selected( ${q104_M41_full_list} , '4')* | |
| q504_M41a *(required)* | q504_M41a. What percent of patients require this item?<br>*Question relevant when: selected( ${q104_M41_full_list} , '4')*<br>*Response constrained to: .>0 and .<=100 or .=999* | |
| q504_M41b *(required)* | q504_M41b. How is the item administered, or given, to the patient?<br>*Question relevant when: selected( ${q104_M41_full_list} , '4')* | 1 Oral - solid (e.g. tablet)<br>2 Oral - liquid<br>3 Sublingual<br>4 Intravenous (IV)<br>5 Intramusculuar injection (IM)<br>6 Subcutaneous<br>7 Rectal<br>8 Inhaled (e.g. gas)<br>9 Inserted (e.g. vaginal pessaries)<br>10 Topical - cream<br>11 Topical - drops (e.g. eye drops)<br>12 Other<br>99 Don't know |
| q504_M41c *(required)* | q504_M41c. What is the item's smallest unit of measurement (mg, mcg, ml, g, etc)?<br>*Question relevant when: selected( ${q104_M41_full_list} , '4')* | microgram — microgram (mcg)<br>miligram — miligram (mg)<br>miligram_dissolved_in_liquid — miligram (mg) dissolved in liquid (e.g. 2 mg per 3 militers)<br>gram — gram (g)<br>militer — militer (ml)<br>centiliter — centiliter (cl)<br>litre — litre (l)<br>ml_of _solution — ml of solution (e.g. ml of 10% solution)<br>litre_of_solution — litre of solution (e.g. 1 litre of 5% solution)<br>cubic_meter — cubic meter (m^3)<br>other — other<br>dont_know — don't know |
| q504_M41c.1 | q504_M41c.1 If other unit of measurement or route of administration, specify here:<br>*Leave blank if not applicable*<br>*Question relevant when: selected( ${q104_M41_full_list} , '4')* | |
| q504_M41d *(required)* | q504_M41d. In total, how many units are used to treat a patient during all of her care?<br>*Remember to add all units! E.g. 5 days x 200 mg twice per day = 2,000mg*<br>*Question relevant when: selected( ${q104_M41_full_list} , '4')*<br>*Response constrained to: .>0* | |

| Field | Question | Answer |
|---|---|---|
| q504_M42 | ***q504_M42. Metronidazole (injection)***<br>*Question relevant when: selected( ${q104_M42_full_list} , '4')* | |
| q504_M42a *(required)* | q504_M42a. What percent of patients require this item?<br>*Question relevant when: selected( ${q104_M42_full_list} , '4')*<br>*Response constrained to: .>0 and .<=100 or .=999* | |
| q504_M42b *(required)* | q504_M42b. How is the item administered, or given, to the patient?<br>*Question relevant when: selected( ${q104_M42_full_list} , '4')* | 1 Oral - solid (e.g. tablet)<br>2 Oral - liquid<br>3 Sublingual<br>4 Intravenous (IV)<br>5 Intramusculuar injection (IM)<br>6 Subcutaneous<br>7 Rectal<br>8 Inhaled (e.g. gas)<br>9 Inserted (e.g. vaginal pessaries)<br>10 Topical - cream<br>11 Topical - drops (e.g. eye drops)<br>12 Other<br>99 Don't know |
| q504_M42c *(required)* | q504_M42c. What is the item's smallest unit of measurement (mg, mcg, ml, g, etc)?<br>*Question relevant when: selected( ${q104_M42_full_list} , '4')* | microgram — microgram (mcg)<br>miligram — miligram (mg)<br>miligram_dissolved_in_liquid — miligram (mg) dissolved in liquid (e.g. 2 mg per 3 militers)<br>gram — gram (g)<br>militer — militer (ml)<br>centiliter — centiliter (cl)<br>litre — litre (l)<br>ml_of _solution — ml of solution (e.g. ml of 10% solution)<br>litre_of_solution — litre of solution (e.g. 1 litre of 5% solution)<br>cubic_meter — cubic meter (m^3)<br>other — other<br>dont_know — don't know |
| q504_M42c.1 | q504_M42c.1 If other unit of measurement or route of administration, specify here:<br>*Leave blank if not applicable*<br>*Question relevant when: selected( ${q104_M42_full_list} , '4')* | |
| q504_M42d *(required)* | q504_M42d. In total, how many units are used to treat a patient during all of her care?<br>*Remember to add all units! E.g. 5 days x 200 mg twice per day = 2,000mg*<br>*Question relevant when: selected( ${q104_M42_full_list} , '4')*<br>*Response constrained to: .>0* | |

| Field | Question | Answer |
|---|---|---|
| q504_M43 | ***q504_M43. Metronidazole (oral)***<br>*Question relevant when: selected( ${q104_M43_full_list} , '4')* | |
| q504_M43a *(required)* | q504_M43a. What percent of patients require this item?<br>*Question relevant when: selected( ${q104_M43_full_list} , '4')*<br>*Response constrained to: .>0 and .<=100 or .=999* | |

| Field | Question | Answer | |
|---|---|---|---|
| q504_M43b *(required)* | q504_M43b. How is the item administered, or given, to the patient?<br>*Question relevant when: selected( ${q104_M43_full_list} , '4')* | 1 | Oral - solid (e.g. tablet) |
| | | 2 | Oral - liquid |
| | | 3 | Sublingual |
| | | 4 | Intravenous (IV) |
| | | 5 | Intramusculuar injection (IM) |
| | | 6 | Subcutaneous |
| | | 7 | Rectal |
| | | 8 | Inhaled (e.g. gas) |
| | | 9 | Inserted (e.g. vaginal pessaries) |
| | | 10 | Topical - cream |
| | | 11 | Topical - drops (e.g. eye drops) |
| | | 12 | Other |
| | | 99 | Don't know |
| q504_M43c *(required)* | q504_M43c. What is the item's smallest unit of measurement (mg, mcg, ml, g, etc)?<br>*Question relevant when: selected( ${q104_M43_full_list} , '4')* | microgram | microgram (mcg) |
| | | miligram | miligram (mg) |
| | | miligram_dissolved_in_liquid | miligram (mg) dissolved in liquid (e.g. 2 mg per 3 militers) |
| | | gram | gram (g) |
| | | militer | militer (ml) |
| | | centiliter | centiliter (cl) |
| | | litre | litre (l) |
| | | ml_of _solution | ml of solution (e.g. ml of 10% solution) |
| | | litre_of_solution | litre of solution (e.g. 1 litre of 5% solution) |
| | | cubic_meter | cubic meter (m^3) |
| | | other | other |
| | | dont_know | don't know |
| q504_M43c.1 | q504_M43c.1 If other unit of measurement or route of administration, specify here:<br>*Leave blank if not applicable*<br>*Question relevant when: selected( ${q104_M43_full_list} , '4')* | | |
| q504_M43d *(required)* | q504_M43d. In total, how many units are used to treat a patient during all of her care?<br>*Remember to add all units! E.g. 5 days x 200 mg twice per day = 2,000mg*<br>*Question relevant when: selected( ${q104_M43_full_list} , '4')*<br>*Response constrained to: .>0* | | |

| Field | Question | Answer |
|---|---|---|
| q504_M44 | ***q504_M44. Penicillin***<br>*Question relevant when: selected( ${q104_M44_full_list} , '4')* | |
| q504_M44a *(required)* | q504_M44a. What percent of patients require this item?<br>*Question relevant when: selected( ${q104_M44_full_list} , '4')*<br>*Response constrained to: .>0 and .<=100 or .=999* | |
| q504_M44b *(required)* | q504_M44b. How is the item administered, or given, to the patient?<br>*Question relevant when: selected( ${q104_M44_full_list} , '4')* | 1 Oral - solid (e.g. tablet)<br>2 Oral - liquid<br>3 Sublingual<br>4 Intravenous (IV)<br>5 Intramusculuar injection (IM)<br>6 Subcutaneous<br>7 Rectal<br>8 Inhaled (e.g. gas)<br>9 Inserted (e.g. vaginal pessaries)<br>10 Topical - cream<br>11 Topical - drops (e.g. eye drops)<br>12 Other<br>99 Don't know |
| q504_M44c *(required)* | q504_M44c. What is the item's smallest unit of measurement (mg, mcg, ml, g, etc)?<br>*Question relevant when: selected( ${q104_M44_full_list} , '4')* | microgram — microgram (mcg)<br>miligram — miligram (mg)<br>miligram_dissolved_in_liquid — miligram (mg) dissolved in liquid (e.g. 2 mg per 3 militers)<br>gram — gram (g)<br>militer — militer (ml)<br>centiliter — centiliter (cl)<br>litre — litre (l)<br>ml_of _solution — ml of solution (e.g. ml of 10% solution)<br>litre_of_solution — litre of solution (e.g. 1 litre of 5% solution)<br>cubic_meter — cubic meter (m^3)<br>other — other<br>dont_know — don't know |
| q504_M44c.1 | q504_M44c.1 If other unit of measurement or route of administration, specify here:<br>*Leave blank if not applicable*<br>*Question relevant when: selected( ${q104_M44_full_list} , '4')* | |
| q504_M44d *(required)* | q504_M44d. In total, how many units are used to treat a patient during all of her care?<br>*Remember to add all units! E.g. 5 days x 200 mg twice per day = 2,000mg*<br>*Question relevant when: selected( ${q104_M44_full_list} , '4')*<br>*Response constrained to: .>0* | |

| Field | Question | Answer |
|---|---|---|
| q504_M45 | ***q504_M45. Antibiotic - other 1: "[q104_M45_full_list_other]"***<br>*Question relevant when: selected( ${q104_M45_full_list} , '4')* | |
| q504_M45a *(required)* | q504_M45a. What percent of patients require this item?<br>*Question relevant when: selected( ${q104_M45_full_list} , '4')*<br>*Response constrained to: .>0 and .<=100 or .=999* | |
| q504_M45b *(required)* | q504_M45b. How is the item administered, or given, to the patient?<br>*Question relevant when: selected( ${q104_M45_full_list} , '4')* | 1 Oral - solid (e.g. tablet)<br>2 Oral - liquid<br>3 Sublingual<br>4 Intravenous (IV)<br>5 Intramusculuar injection (IM)<br>6 Subcutaneous<br>7 Rectal<br>8 Inhaled (e.g. gas)<br>9 Inserted (e.g. vaginal pessaries)<br>10 Topical - cream<br>11 Topical - drops (e.g. eye drops)<br>12 Other<br>99 Don't know |
| q504_M45c *(required)* | q504_M45c. What is the item's smallest unit of measurement (mg, mcg, ml, g, etc)?<br>*Question relevant when: selected( ${q104_M45_full_list} , '4')* | microgram — microgram (mcg)<br>miligram — miligram (mg)<br>miligram_dissolved_in_liquid — miligram (mg) dissolved in liquid (e.g. 2 mg per 3 militers)<br>gram — gram (g)<br>militer — militer (ml)<br>centiliter — centiliter (cl)<br>litre — litre (l)<br>ml_of _solution — ml of solution (e.g. ml of 10% solution)<br>litre_of_solution — litre of solution (e.g. 1 litre of 5% solution)<br>cubic_meter — cubic meter (m^3)<br>other — other<br>dont_know — don't know |
| q504_M45c.1 | q504_M45c.1 If other unit of measurement or route of administration, specify here:<br>*Leave blank if not applicable*<br>*Question relevant when: selected( ${q104_M45_full_list} , '4')* | |
| q504_M45d *(required)* | q504_M45d. In total, how many units are used to treat a patient during all of her care?<br>*Remember to add all units! E.g. 5 days x 200 mg twice per day = 2,000mg*<br>*Question relevant when: selected( ${q104_M45_full_list} , '4')*<br>*Response constrained to: .>0* | |

| Field | Question | Answer |
|---|---|---|
| q504_M46 | ***q504_M46. Antibiotic - other 2: "[q104_M46_full_list_other]"***<br>*Question relevant when: selected( ${q104_M46_full_list} , '4')* | |
| q504_M46a *(required)* | q504_M46a. What percent of patients require this item?<br>*Question relevant when: selected( ${q104_M46_full_list} , '4')*<br>*Response constrained to: .>0 and .<=100 or .=999* | |
| q504_M46b *(required)* | q504_M46b. How is the item administered, or given, to the patient?<br>*Question relevant when: selected( ${q104_M46_full_list} , '4')* | 1 Oral - solid (e.g. tablet)<br>2 Oral - liquid<br>3 Sublingual<br>4 Intravenous (IV)<br>5 Intramusculuar injection (IM)<br>6 Subcutaneous<br>7 Rectal<br>8 Inhaled (e.g. gas)<br>9 Inserted (e.g. vaginal pessaries)<br>10 Topical - cream<br>11 Topical - drops (e.g. eye drops)<br>12 Other<br>99 Don't know |
| q504_M46c *(required)* | q504_M46c. What is the item's smallest unit of measurement (mg, mcg, ml, g, etc)?<br>*Question relevant when: selected( ${q104_M46_full_list} , '4')* | microgram — microgram (mcg)<br>miligram — miligram (mg)<br>miligram_dissolved_in_liquid — miligram (mg) dissolved in liquid (e.g. 2 mg per 3 militers)<br>gram — gram (g)<br>militer — militer (ml)<br>centiliter — centiliter (cl)<br>litre — litre (l)<br>ml_of _solution — ml of solution (e.g. ml of 10% solution)<br>litre_of_solution — litre of solution (e.g. 1 litre of 5% solution)<br>cubic_meter — cubic meter (m^3)<br>other — other<br>dont_know — don't know |
| q504_M46c.1 | q504_M46c.1 If other unit of measurement or route of administration, specify here:<br>*Leave blank if not applicable*<br>*Question relevant when: selected( ${q104_M46_full_list} , '4')* | |
| q504_M46d *(required)* | q504_M46d. In total, how many units are used to treat a patient during all of her care?<br>*Remember to add all units! E.g. 5 days x 200 mg twice per day = 2,000mg*<br>*Question relevant when: selected( ${q104_M46_full_list} , '4')*<br>*Response constrained to: .>0* | |

| Field | Question | Answer |
|---|---|---|
| q504_M47 | ***q504_M47. Antibiotic - other 3: "[q104_M47_full_list_other]"***<br>*Question relevant when: selected( ${q104_M47_full_list} , '4')* | |
| q504_M47a *(required)* | q504_M47a. What percent of patients require this item?<br>*Question relevant when: selected( ${q104_M47_full_list} , '4')*<br>*Response constrained to: .>0 and .<=100 or .=999* | |
| q504_M47b *(required)* | q504_M47b. How is the item administered, or given, to the patient?<br>*Question relevant when: selected( ${q104_M47_full_list} , '4')* | 1 Oral - solid (e.g. tablet)<br>2 Oral - liquid<br>3 Sublingual<br>4 Intravenous (IV)<br>5 Intramusculuar injection (IM)<br>6 Subcutaneous<br>7 Rectal<br>8 Inhaled (e.g. gas)<br>9 Inserted (e.g. vaginal pessaries)<br>10 Topical - cream<br>11 Topical - drops (e.g. eye drops)<br>12 Other<br>99 Don't know |
| q504_M47c *(required)* | q504_M47c. What is the item's smallest unit of measurement (mg, mcg, ml, g, etc)?<br>*Question relevant when: selected( ${q104_M47_full_list} , '4')* | microgram — microgram (mcg)<br>miligram — miligram (mg)<br>miligram_dissolved_in_liquid — miligram (mg) dissolved in liquid (e.g. 2 mg per 3 militers)<br>gram — gram (g)<br>militer — militer (ml)<br>centiliter — centiliter (cl)<br>litre — litre (l)<br>ml_of _solution — ml of solution (e.g. ml of 10% solution)<br>litre_of_solution — litre of solution (e.g. 1 litre of 5% solution)<br>cubic_meter — cubic meter (m^3)<br>other — other<br>dont_know — don't know |
| q504_M47c.1 | q504_M47c.1 If other unit of measurement or route of administration, specify here:<br>*Leave blank if not applicable*<br>*Question relevant when: selected( ${q104_M47_full_list} , '4')* | |
| q504_M47d *(required)* | q504_M47d. In total, how many units are used to treat a patient during all of her care?<br>*Remember to add all units! E.g. 5 days x 200 mg twice per day = 2,000mg*<br>*Question relevant when: selected( ${q104_M47_full_list} , '4')*<br>*Response constrained to: .>0* | |

| Field | Question | Answer |
|---|---|---|
| q504_M48 | ***q504_M48. Antibiotic - other 4: "[q104_M48_full_list_other]"***<br>*Question relevant when: selected( ${q104_M48_full_list} , '4')* | |
| q504_M48a *(required)* | q504_M48a. What percent of patients require this item?<br>*Question relevant when: selected( ${q104_M48_full_list} , '4')*<br>*Response constrained to: .>0 and .<=100 or .=999* | |

| Field | Question | | Answer |
|---|---|---|---|
| q504_M48b *(required)* | q504_M48b. How is the item administered, or given, to the patient?<br>*Question relevant when: selected( ${q104_M48_full_list} , '4')* | 1 | Oral - solid (e.g. tablet) |
| | | 2 | Oral - liquid |
| | | 3 | Sublingual |
| | | 4 | Intravenous (IV) |
| | | 5 | Intramusculuar injection (IM) |
| | | 6 | Subcutaneous |
| | | 7 | Rectal |
| | | 8 | Inhaled (e.g. gas) |
| | | 9 | Inserted (e.g. vaginal pessaries) |
| | | 10 | Topical - cream |
| | | 11 | Topical - drops (e.g. eye drops) |
| | | 12 | Other |
| | | 99 | Don't know |
| q504_M48c *(required)* | q504_M48c. What is the item's smallest unit of measurement (mg, mcg, ml, g, etc)?<br>*Question relevant when: selected( ${q104_M48_full_list} , '4')* | microgram | microgram (mcg) |
| | | miligram | miligram (mg) |
| | | miligram_dissolved_in_liquid | miligram (mg) dissolved in liquid (e.g. 2 mg per 3 militers) |
| | | gram | gram (g) |
| | | militer | militer (ml) |
| | | centiliter | centiliter (cl) |
| | | litre | litre (l) |
| | | ml_of _solution | ml of solution (e.g. ml of 10% solution) |
| | | litre_of_solution | litre of solution (e.g. 1 litre of 5% solution) |
| | | cubic_meter | cubic meter (m^3) |
| | | other | other |
| | | dont_know | don't know |
| q504_M48c.1 | q504_M48c.1 If other unit of measurement or route of administration, specify here:<br>*Leave blank if not applicable*<br>*Question relevant when: selected( ${q104_M48_full_list} , '4')* | | |
| q504_M48d *(required)* | q504_M48d. In total, how many units are used to treat a patient during all of her care?<br>*Remember to add all units! E.g. 5 days x 200 mg twice per day = 2,000mg*<br>*Question relevant when: selected( ${q104_M48_full_list} , '4')*<br>*Response constrained to: .>0* | | |

| Field | Question | Answer |
|---|---|---|
| q504_M49 | ***q504_M49. Antibiotic - other 5: "[q104_M49_full_list_other]"***<br>*Question relevant when: selected( ${q104_M49_full_list} , '4')* | |
| q504_M49a *(required)* | q504_M49a. What percent of patients require this item?<br>*Question relevant when: selected( ${q104_M49_full_list} , '4')*<br>*Response constrained to: .>0 and .<=100 or .=999* | |
| q504_M49b *(required)* | q504_M49b. How is the item administered, or given, to the patient?<br>*Question relevant when: selected( ${q104_M49_full_list} , '4')* | 1 Oral - solid (e.g. tablet)<br>2 Oral - liquid<br>3 Sublingual<br>4 Intravenous (IV)<br>5 Intramusculuar injection (IM)<br>6 Subcutaneous<br>7 Rectal<br>8 Inhaled (e.g. gas)<br>9 Inserted (e.g. vaginal pessaries)<br>10 Topical - cream<br>11 Topical - drops (e.g. eye drops)<br>12 Other<br>99 Don't know |
| q504_M49c *(required)* | q504_M49c. What is the item's smallest unit of measurement (mg, mcg, ml, g, etc)?<br>*Question relevant when: selected( ${q104_M49_full_list} , '4')* | microgram — microgram (mcg)<br>miligram — miligram (mg)<br>miligram_dissolved_in_liquid — miligram (mg) dissolved in liquid (e.g. 2 mg per 3 militers)<br>gram — gram (g)<br>militer — militer (ml)<br>centiliter — centiliter (cl)<br>litre — litre (l)<br>ml_of _solution — ml of solution (e.g. ml of 10% solution)<br>litre_of_solution — litre of solution (e.g. 1 litre of 5% solution)<br>cubic_meter — cubic meter (m^3)<br>other — other<br>dont_know — don't know |
| q504_M49c.1 | q504_M49c.1 If other unit of measurement or route of administration, specify here:<br>*Leave blank if not applicable*<br>*Question relevant when: selected( ${q104_M49_full_list} , '4')* | |
| q504_M49d *(required)* | q504_M49d. In total, how many units are used to treat a patient during all of her care?<br>*Remember to add all units! E.g. 5 days x 200 mg twice per day = 2,000mg*<br>*Question relevant when: selected( ${q104_M49_full_list} , '4')*<br>*Response constrained to: .>0* | |

<p><strong><em>D. Medications - Lacerations (4)</em></strong></p>
*Group relevant when: selected( ${section_five_skip_med} , '1')*

| Field | Question | Answer |
|---|---|---|
| q504_med_note4 | 504 CONTINUED For each of the following medications and other medical products that are used for lacerations, can you tell me …? | |
| note_504_antiseptic | ***Antiseptic*** | |
| q504_M50 | ***q504_M50. Cetrimide 15% ("Savlon")***<br>*Question relevant when: selected( ${q104_M50_full_list} , '4')* | |
| q504_M50a *(required)* | q504_M50a. What percent of patients require this item?<br>*Question relevant when: selected( ${q104_M50_full_list} , '4')*<br>*Response constrained to: .>0 and .<=100 or .=999* | |

| Field | Question | Answer | | |
|---|---|---|---|---|
| q504_M50b *(required)* | q504_M50b. How is the item administered, or given, to the patient?<br>*Question relevant when: selected( ${q104_M50_full_list} , '4')* | | 1 | Oral - solid (e.g. tablet) |
| | | | 2 | Oral - liquid |
| | | | 3 | Sublingual |
| | | | 4 | Intravenous (IV) |
| | | | 5 | Intramusculuar injection (IM) |
| | | | 6 | Subcutaneous |
| | | | 7 | Rectal |
| | | | 8 | Inhaled (e.g. gas) |
| | | | 9 | Inserted (e.g. vaginal pessaries) |
| | | | 10 | Topical - cream |
| | | | 11 | Topical - drops (e.g. eye drops) |
| | | | 12 | Other |
| | | | 99 | Don't know |
| q504_M50c *(required)* | q504_M50c. What is the item's smallest unit of measurement (mg, mcg, ml, g, etc)?<br>*Question relevant when: selected( ${q104_M50_full_list} , '4')* | | microgram | microgram (mcg) |
| | | | miligram | miligram (mg) |
| | | | miligram_dissolved_in_liquid | miligram (mg) dissolved in liquid (e.g. 2 mg per 3 militers) |
| | | | gram | gram (g) |
| | | | militer | militer (ml) |
| | | | centiliter | centiliter (cl) |
| | | | litre | litre (l) |
| | | | ml_of _solution | ml of solution (e.g. ml of 10% solution) |
| | | | litre_of_solution | litre of solution (e.g. 1 litre of 5% solution) |
| | | | cubic_meter | cubic meter (m^3) |
| | | | other | other |
| | | | dont_know | don't know |
| q504_M50c.1 | q504_M50c.1 If other unit of measurement or route of administration, specify here:<br>*Leave blank if not applicable*<br>*Question relevant when: selected( ${q104_M50_full_list} , '4')* | | | |
| q504_M50d *(required)* | q504_M50d. In total, how many units are used to treat a patient during all of her care?<br>*Remember to add all units! E.g. 5 days x 200 mg twice per day = 2,000mg*<br>*Question relevant when: selected( ${q104_M50_full_list} , '4')*<br>*Response constrained to: .>0* | | | |

| Field | Question | Answer |
|---|---|---|
| q504_M51 | ***q504_M51. Povidone-iodine solution, 7.5%***<br>*Question relevant when: selected( ${q104_M51_full_list} , '4')* | |
| q504_M51a *(required)* | q504_M51a. What percent of patients require this item?<br>*Question relevant when: selected( ${q104_M51_full_list} , '4')*<br>*Response constrained to: .>0 and .<=100 or .=999* | |
| q504_M51b *(required)* | q504_M51b. How is the item administered, or given, to the patient?<br>*Question relevant when: selected( ${q104_M51_full_list} , '4')* | 1 Oral - solid (e.g. tablet)<br>2 Oral - liquid<br>3 Sublingual<br>4 Intravenous (IV)<br>5 Intramusculuar injection (IM)<br>6 Subcutaneous<br>7 Rectal<br>8 Inhaled (e.g. gas)<br>9 Inserted (e.g. vaginal pessaries)<br>10 Topical - cream<br>11 Topical - drops (e.g. eye drops)<br>12 Other<br>99 Don't know |
| q504_M51c *(required)* | q504_M51c. What is the item's smallest unit of measurement (mg, mcg, ml, g, etc)?<br>*Question relevant when: selected( ${q104_M51_full_list} , '4')* | microgram — microgram (mcg)<br>miligram — miligram (mg)<br>miligram_dissolved_in_liquid — miligram (mg) dissolved in liquid (e.g. 2 mg per 3 militers)<br>gram — gram (g)<br>militer — militer (ml)<br>centiliter — centiliter (cl)<br>litre — litre (l)<br>ml_of _solution — ml of solution (e.g. ml of 10% solution)<br>litre_of_solution — litre of solution (e.g. 1 litre of 5% solution)<br>cubic_meter — cubic meter (m^3)<br>other — other<br>dont_know — don't know |
| q504_M51c.1 | q504_M51c.1 If other unit of measurement or route of administration, specify here:<br>*Leave blank if not applicable*<br>*Question relevant when: selected( ${q104_M51_full_list} , '4')* | |
| q504_M51d *(required)* | q504_M51d. In total, how many units are used to treat a patient during all of her care?<br>*Remember to add all units! E.g. 5 days x 200 mg twice per day = 2,000mg*<br>*Question relevant when: selected( ${q104_M51_full_list} , '4')*<br>*Response constrained to: .>0* | |

| Field | Question | Answer |
|---|---|---|
| q504_M52 | ***q504_M52. Antiseptic - other 1: "[q104_M52_full_list_other]"***<br>*Question relevant when: selected( ${q104_M52_full_list} , '4')* | |
| q504_M52a *(required)* | q504_M52a. What percent of patients require this item?<br>*Question relevant when: selected( ${q104_M52_full_list} , '4')*<br>*Response constrained to: .>0 and .<=100 or .=999* | |
| q504_M52b *(required)* | q504_M52b. How is the item administered, or given, to the patient?<br>*Question relevant when: selected( ${q104_M52_full_list} , '4')* | 1 Oral - solid (e.g. tablet)<br>2 Oral - liquid<br>3 Sublingual<br>4 Intravenous (IV)<br>5 Intramusculuar injection (IM)<br>6 Subcutaneous<br>7 Rectal<br>8 Inhaled (e.g. gas)<br>9 Inserted (e.g. vaginal pessaries)<br>10 Topical - cream<br>11 Topical - drops (e.g. eye drops)<br>12 Other<br>99 Don't know |
| q504_M52c *(required)* | q504_M52c. What is the item's smallest unit of measurement (mg, mcg, ml, g, etc)?<br>*Question relevant when: selected( ${q104_M52_full_list} , '4')* | microgram — microgram (mcg)<br>miligram — miligram (mg)<br>miligram_dissolved_in_liquid — miligram (mg) dissolved in liquid (e.g. 2 mg per 3 militers)<br>gram — gram (g)<br>militer — militer (ml)<br>centiliter — centiliter (cl)<br>litre — litre (l)<br>ml_of _solution — ml of solution (e.g. ml of 10% solution)<br>litre_of_solution — litre of solution (e.g. 1 litre of 5% solution)<br>cubic_meter — cubic meter (m^3)<br>other — other<br>dont_know — don't know |
| q504_M52c.1 | q504_M52c.1 If other unit of measurement or route of administration, specify here:<br>*Leave blank if not applicable*<br>*Question relevant when: selected( ${q104_M52_full_list} , '4')* | |
| q504_M52d *(required)* | q504_M52d. In total, how many units are used to treat a patient during all of her care?<br>*Remember to add all units! E.g. 5 days x 200 mg twice per day = 2,000mg*<br>*Question relevant when: selected( ${q104_M52_full_list} , '4')*<br>*Response constrained to: .>0* | |

| Field | Question | Answer |
|---|---|---|
| q504_M53 | ***q504_M53. Antiseptic - other 2: "[q104_M53_full_list_other]"***<br>*Question relevant when: selected( ${q104_M53_full_list} , '4')* | |
| q504_M53a *(required)* | q504_M53a. What percent of patients require this item?<br>*Question relevant when: selected( ${q104_M53_full_list} , '4')*<br>*Response constrained to: .>0 and .<=100 or .=999* | |
| q504_M53b *(required)* | q504_M53b. How is the item administered, or given, to the patient?<br>*Question relevant when: selected( ${q104_M53_full_list} , '4')* | 1 Oral - solid (e.g. tablet)<br>2 Oral - liquid<br>3 Sublingual<br>4 Intravenous (IV)<br>5 Intramusculuar injection (IM)<br>6 Subcutaneous<br>7 Rectal<br>8 Inhaled (e.g. gas)<br>9 Inserted (e.g. vaginal pessaries)<br>10 Topical - cream<br>11 Topical - drops (e.g. eye drops)<br>12 Other<br>99 Don't know |
| q504_M53c *(required)* | q504_M53c. What is the item's smallest unit of measurement (mg, mcg, ml, g, etc)?<br>*Question relevant when: selected( ${q104_M53_full_list} , '4')* | microgram — microgram (mcg)<br>miligram — miligram (mg)<br>miligram_dissolved_in_liquid — miligram (mg) dissolved in liquid (e.g. 2 mg per 3 militers)<br>gram — gram (g)<br>militer — militer (ml)<br>centiliter — centiliter (cl)<br>litre — litre (l)<br>ml_of _solution — ml of solution (e.g. ml of 10% solution)<br>litre_of_solution — litre of solution (e.g. 1 litre of 5% solution)<br>cubic_meter — cubic meter (m^3)<br>other — other<br>dont_know — don't know |
| q504_M53c.1 | q504_M53c.1 If other unit of measurement or route of administration, specify here:<br>*Leave blank if not applicable*<br>*Question relevant when: selected( ${q104_M53_full_list} , '4')* | |
| q504_M53d *(required)* | q504_M53d. In total, how many units are used to treat a patient during all of her care?<br>*Remember to add all units! E.g. 5 days x 200 mg twice per day = 2,000mg*<br>*Question relevant when: selected( ${q104_M53_full_list} , '4')*<br>*Response constrained to: .>0* | |

| Field | Question | Answer |
|---|---|---|
| q504_M54 | ***q504_M54. Antiseptic - other 3: "[q104_M54_full_list_other]"*** *Question relevant when: selected( ${q104_M54_full_list} , '4')* | |
| q504_M54a *(required)* | q504_M54a. What percent of patients require this item? *Question relevant when: selected( ${q104_M54_full_list} , '4')* *Response constrained to: .>0 and .<=100 or .=999* | |
| q504_M54b *(required)* | q504_M54b. How is the item administered, or given, to the patient? *Question relevant when: selected( ${q104_M54_full_list} , '4')* | 1 Oral - solid (e.g. tablet); 2 Oral - liquid; 3 Sublingual; 4 Intravenous (IV); 5 Intramusculuar injection (IM); 6 Subcutaneous; 7 Rectal; 8 Inhaled (e.g. gas); 9 Inserted (e.g. vaginal pessaries); 10 Topical - cream; 11 Topical - drops (e.g. eye drops); 12 Other; 99 Don't know |
| q504_M54c *(required)* | q504_M54c. What is the item's smallest unit of measurement (mg, mcg, ml, g, etc)? *Question relevant when: selected( ${q104_M54_full_list} , '4')* | microgram: microgram (mcg); miligram: miligram (mg); miligram_dissolved_in_liquid: miligram (mg) dissolved in liquid (e.g. 2 mg per 3 militers); gram: gram (g); militer: militer (ml); centiliter: centiliter (cl); litre: litre (l); ml_of _solution: ml of solution (e.g. ml of 10% solution); litre_of_solution: litre of solution (e.g. 1 litre of 5% solution); cubic_meter: cubic meter (m^3); other: other; dont_know: don't know |
| q504_M54c.1 | q504_M54c.1 If other unit of measurement or route of administration, specify here: *Leave blank if not applicable* *Question relevant when: selected( ${q104_M54_full_list} , '4')* | |
| q504_M54d *(required)* | q504_M54d. In total, how many units are used to treat a patient during all of her care? *Remember to add all units! E.g. 5 days x 200 mg twice per day = 2,000mg* *Question relevant when: selected( ${q104_M54_full_list} , '4')* *Response constrained to: .>0* | |

| Field | Question | Answer |
|---|---|---|
| q504_M54.1 | ***q504_M54.1. Antiseptic - other 4: "[q104_M54.1_full_list_other]"***<br>*Question relevant when: selected( ${q104_M54.1_full_list} , '2')* | |
| q504_M54.1a *(required)* | q504_M54.1a. What percent of patients require this item?<br>*Question relevant when: selected( ${q104_M54.1_full_list} , '2')*<br>*Response constrained to: .>0 and .<=100 or .=999* | |
| q504_M54.1b *(required)* | q504_M54.1b. How is the item administered, or given, to the patient?<br>*Question relevant when: selected( ${q104_M54.1_full_list} , '2')* | 1 Oral - solid (e.g. tablet)<br>2 Oral - liquid<br>3 Sublingual<br>4 Intravenous (IV)<br>5 Intramusculuar injection (IM)<br>6 Subcutaneous<br>7 Rectal<br>8 Inhaled (e.g. gas)<br>9 Inserted (e.g. vaginal pessaries)<br>10 Topical - cream<br>11 Topical - drops (e.g. eye drops)<br>12 Other<br>99 Don't know |
| q504_M54.1c *(required)* | q504_M54.1c. What is the item's smallest unit of measurement (mg, mcg, ml, g, etc)?<br>*Question relevant when: selected( ${q104_M54.1_full_list} , '2')* | microgram: microgram (mcg)<br>miligram: miligram (mg)<br>miligram_dissolved_in_liquid: miligram (mg) dissolved in liquid (e.g. 2 mg per 3 militers)<br>gram: gram (g)<br>militer: militer (ml)<br>centiliter: centiliter (cl)<br>litre: litre (l)<br>ml_of _solution: ml of solution (e.g. ml of 10% solution)<br>litre_of_solution: litre of solution (e.g. 1 litre of 5% solution)<br>cubic_meter: cubic meter (m^3)<br>other: other<br>dont_know: don't know |
| q504_M54.1c.1 | q504_M54.1c.1 If other unit of measurement or route of administration, specify here:<br>*Leave blank if not applicable*<br>*Question relevant when: selected( ${q104_M54.1_full_list} , '2')* | |
| q504_M54.1d *(required)* | q504_M54.1d. In total, how many units are used to treat a patient during all of her care?<br>*Remember to add all units! E.g. 5 days x 200 mg twice per day = 2,000mg*<br>*Question relevant when: selected( ${q104_M54.1_full_list} , '2')*<br>*Response constrained to: .>0* | |

| Field | Question | Answer |
|---|---|---|
| q504_M54.2 | ***q504_M54.2. Antiseptic - other 5: "[q104_M54.2_full_list_other]"*** *Question relevant when: selected( ${q104_M54.2_full_list} , '2')* | |
| q504_M54.2a *(required)* | q504_M54.2a. What percent of patients require this item? *Question relevant when: selected( ${q104_M54.2_full_list} , '2')* *Response constrained to: .>0 and .<=100 or .=999* | |

| Field | Question | Answer | |
|---|---|---|---|
| q504_M54.2b *(required)* | q504_M54.2b. How is the item administered, or given, to the patient? *Question relevant when: selected( ${q104_M54.2_full_list} , '2')* | 1 | Oral - solid (e.g. tablet) |
| | | 2 | Oral - liquid |
| | | 3 | Sublingual |
| | | 4 | Intravenous (IV) |
| | | 5 | Intramusculuar injection (IM) |
| | | 6 | Subcutaneous |
| | | 7 | Rectal |
| | | 8 | Inhaled (e.g. gas) |
| | | 9 | Inserted (e.g. vaginal pessaries) |
| | | 10 | Topical - cream |
| | | 11 | Topical - drops (e.g. eye drops) |
| | | 12 | Other |
| | | 99 | Don't know |
| q504_M54.2c *(required)* | q504_M54.2c. What is the item's smallest unit of measurement (mg, mcg, ml, g, etc)? *Question relevant when: selected( ${q104_M54.2_full_list} , '2')* | microgram | microgram (mcg) |
| | | miligram | miligram (mg) |
| | | miligram_dissolved_in_liquid | miligram (mg) dissolved in liquid (e.g. 2 mg per 3 militers) |
| | | gram | gram (g) |
| | | militer | militer (ml) |
| | | centiliter | centiliter (cl) |
| | | litre | litre (l) |
| | | ml_of _solution | ml of solution (e.g. ml of 10% solution) |
| | | litre_of_solution | litre of solution (e.g. 1 litre of 5% solution) |
| | | cubic_meter | cubic meter (m^3) |
| | | other | other |
| | | dont_know | don't know |
| q504_M54.2c.1 | q504_M54.2c.1 If other unit of measurement or route of administration, specify here: *Leave blank if not applicable* *Question relevant when: selected( ${q104_M54.2_full_list} , '2')* | | |
| q504_M54.2d *(required)* | q504_M54.2d. In total, how many units are used to treat a patient during all of her care? *Remember to add all units! E.g. 5 days x 200 mg twice per day = 2,000mg* *Question relevant when: selected( ${q104_M54.2_full_list} , '2')* *Response constrained to: .>0* | | |

<p><strong><em>D. Medications - Lacerations (5)</em></strong></p>
*Group relevant when: selected( ${section_five_skip_med} , '1')*

| Field | Question | Answer |
|---|---|---|
| q504_med_notE4 | 504 CONTINUED For each of the following medications and other medical products that are used for lacerations, can you tell me …? | |
| note_504_antispas | ***Antispasmodic, anxiolytic, sedative, etc*** | |
| q504_M55 | ***q504_M55. Hyoscine butylbromide / scopolamine butilhisocin (Buscopan )***<br>*Question relevant when: selected( ${q104_M55_full_list} , '4')* | |
| q504_M55a *(required)* | q504_M55a. What percent of patients require this item?<br>*Question relevant when: selected( ${q104_M55_full_list} , '4')*<br>*Response constrained to: .>0 and .<=100 or .=999* | |

| Field | Question | Answer | |
|---|---|---|---|
| q504_M55b *(required)* | q504_M55b. How is the item administered, or given, to the patient?<br>*Question relevant when: selected( ${q104_M55_full_list} , '4')* | 1 | Oral - solid (e.g. tablet) |
| | | 2 | Oral - liquid |
| | | 3 | Sublingual |
| | | 4 | Intravenous (IV) |
| | | 5 | Intramusculuar injection (IM) |
| | | 6 | Subcutaneous |
| | | 7 | Rectal |
| | | 8 | Inhaled (e.g. gas) |
| | | 9 | Inserted (e.g. vaginal pessaries) |
| | | 10 | Topical - cream |
| | | 11 | Topical - drops (e.g. eye drops) |
| | | 12 | Other |
| | | 99 | Don't know |
| q504_M55c *(required)* | q504_M55c. What is the item's smallest unit of measurement (mg, mcg, ml, g, etc)?<br>*Question relevant when: selected( ${q104_M55_full_list} , '4')* | microgram | microgram (mcg) |
| | | miligram | miligram (mg) |
| | | miligram_dissolved_in_liquid | miligram (mg) dissolved in liquid (e.g. 2 mg per 3 militers) |
| | | gram | gram (g) |
| | | militer | militer (ml) |
| | | centiliter | centiliter (cl) |
| | | litre | litre (l) |
| | | ml_of _solution | ml of solution (e.g. ml of 10% solution) |
| | | litre_of_solution | litre of solution (e.g. 1 litre of 5% solution) |
| | | cubic_meter | cubic meter (m^3) |
| | | other | other |
| | | dont_know | don't know |
| q504_M55c.1 | q504_M55c.1 If other unit of measurement or route of administration, specify here:<br>*Leave blank if not applicable*<br>*Question relevant when: selected( ${q104_M55_full_list} , '4')* | | |
| q504_M55d *(required)* | q504_M55d. In total, how many units are used to treat a patient during all of her care?<br>*Remember to add all units! E.g. 5 days x 200 mg twice per day = 2,000mg*<br>*Question relevant when: selected( ${q104_M55_full_list} , '4')*<br>*Response constrained to: .>0* | | |

| Field | Question | Answer |
|---|---|---|
| q504_M56 | ***q504_M56. Atropine sulfate (or sulphate)***<br>*Question relevant when: selected( ${q104_M56_full_list} , '4')* | |
| q504_M56a *(required)* | q504_M56a. What percent of patients require this item?<br>*Question relevant when: selected( ${q104_M56_full_list} , '4')*<br>*Response constrained to: .>0 and .<=100 or .=999* | |
| q504_M56b *(required)* | q504_M56b. How is the item administered, or given, to the patient?<br>*Question relevant when: selected( ${q104_M56_full_list} , '4')* | 1 Oral - solid (e.g. tablet)<br>2 Oral - liquid<br>3 Sublingual<br>4 Intravenous (IV)<br>5 Intramuscular injection (IM)<br>6 Subcutaneous<br>7 Rectal<br>8 Inhaled (e.g. gas)<br>9 Inserted (e.g. vaginal pessaries)<br>10 Topical - cream<br>11 Topical - drops (e.g. eye drops)<br>12 Other<br>99 Don't know |
| q504_M56c *(required)* | q504_M56c. What is the item's smallest unit of measurement (mg, mcg, ml, g, etc)?<br>*Question relevant when: selected( ${q104_M56_full_list} , '4')* | microgram — microgram (mcg)<br>miligram — miligram (mg)<br>miligram_dissolved_in_liquid — miligram (mg) dissolved in liquid (e.g. 2 mg per 3 militers)<br>gram — gram (g)<br>militer — militer (ml)<br>centiliter — centiliter (cl)<br>litre — litre (l)<br>ml_of _solution — ml of solution (e.g. ml of 10% solution)<br>litre_of_solution — litre of solution (e.g. 1 litre of 5% solution)<br>cubic_meter — cubic meter (m^3)<br>other — other<br>dont_know — don't know |
| q504_M56c.1 | q504_M56c.1 If other unit of measurement or route of administration, specify here:<br>*Leave blank if not applicable*<br>*Question relevant when: selected( ${q104_M56_full_list} , '4')* | |
| q504_M56d *(required)* | q504_M56d. In total, how many units are used to treat a patient during all of her care?<br>*Remember to add all units! E.g. 5 days x 200 mg twice per day = 2,000mg*<br>*Question relevant when: selected( ${q104_M56_full_list} , '4')*<br>*Response constrained to: .>0* | |

| Field | Question | Answer |
|---|---|---|
| q504_M57 | ***q504_M57. Papaverine HCl***<br>*Question relevant when: selected( ${q104_M57_full_list} , '4')* | |
| q504_M57a *(required)* | q504_M57a. What percent of patients require this item?<br>*Question relevant when: selected( ${q104_M57_full_list} , '4')*<br>*Response constrained to: .>0 and .<=100 or .=999* | |
| q504_M57b *(required)* | q504_M57b. How is the item administered, or given, to the patient?<br>*Question relevant when: selected( ${q104_M57_full_list} , '4')* | 1 Oral - solid (e.g. tablet)<br>2 Oral - liquid<br>3 Sublingual<br>4 Intravenous (IV)<br>5 Intramusculuar injection (IM)<br>6 Subcutaneous<br>7 Rectal<br>8 Inhaled (e.g. gas)<br>9 Inserted (e.g. vaginal pessaries)<br>10 Topical - cream<br>11 Topical - drops (e.g. eye drops)<br>12 Other<br>99 Don't know |
| q504_M57c *(required)* | q504_M57c. What is the item's smallest unit of measurement (mg, mcg, ml, g, etc)?<br>*Question relevant when: selected( ${q104_M57_full_list} , '4')* | microgram — microgram (mcg)<br>miligram — miligram (mg)<br>miligram_dissolved_in_liquid — miligram (mg) dissolved in liquid (e.g. 2 mg per 3 militers)<br>gram — gram (g)<br>militer — militer (ml)<br>centiliter — centiliter (cl)<br>litre — litre (l)<br>ml_of _solution — ml of solution (e.g. ml of 10% solution)<br>litre_of_solution — litre of solution (e.g. 1 litre of 5% solution)<br>cubic_meter — cubic meter (m^3)<br>other — other<br>dont_know — don't know |
| q504_M57c.1 | q504_M57c.1 If other unit of measurement or route of administration, specify here:<br>*Leave blank if not applicable*<br>*Question relevant when: selected( ${q104_M57_full_list} , '4')* | |
| q504_M57d *(required)* | q504_M57d. In total, how many units are used to treat a patient during all of her care?<br>*Remember to add all units! E.g. 5 days x 200 mg twice per day = 2,000mg*<br>*Question relevant when: selected( ${q104_M57_full_list} , '4')*<br>*Response constrained to: .>0* | |

| Field | Question | Answer |
|---|---|---|
| q504_M58 | ***q504_M58. Diazepam (injection)***<br>*Question relevant when: selected( ${q104_M58_full_list} , '4')* | |
| q504_M58a *(required)* | q504_M58a. What percent of patients require this item?<br>*Question relevant when: selected( ${q104_M58_full_list} , '4')*<br>*Response constrained to: .>0 and .<=100 or .=999* | |
| q504_M58b *(required)* | q504_M58b. How is the item administered, or given, to the patient?<br>*Question relevant when: selected( ${q104_M58_full_list} , '4')* | 1 Oral - solid (e.g. tablet)<br>2 Oral - liquid<br>3 Sublingual<br>4 Intravenous (IV)<br>5 Intramusculuar injection (IM)<br>6 Subcutaneous<br>7 Rectal<br>8 Inhaled (e.g. gas)<br>9 Inserted (e.g. vaginal pessaries)<br>10 Topical - cream<br>11 Topical - drops (e.g. eye drops)<br>12 Other<br>99 Don't know |
| q504_M58c *(required)* | q504_M58c. What is the item's smallest unit of measurement (mg, mcg, ml, g, etc)?<br>*Question relevant when: selected( ${q104_M58_full_list} , '4')* | microgram — microgram (mcg)<br>miligram — miligram (mg)<br>miligram_dissolved_in_liquid — miligram (mg) dissolved in liquid (e.g. 2 mg per 3 militers)<br>gram — gram (g)<br>militer — militer (ml)<br>centiliter — centiliter (cl)<br>litre — litre (l)<br>ml_of _solution — ml of solution (e.g. ml of 10% solution)<br>litre_of_solution — litre of solution (e.g. 1 litre of 5% solution)<br>cubic_meter — cubic meter (m^3)<br>other — other<br>dont_know — don't know |
| q504_M58c.1 | q504_M58c.1 If other unit of measurement or route of administration, specify here:<br>*Leave blank if not applicable*<br>*Question relevant when: selected( ${q104_M58_full_list} , '4')* | |
| q504_M58d *(required)* | q504_M58d. In total, how many units are used to treat a patient during all of her care?<br>*Remember to add all units! E.g. 5 days x 200 mg twice per day = 2,000mg*<br>*Question relevant when: selected( ${q104_M58_full_list} , '4')*<br>*Response constrained to: .>0* | |

| Field | Question | Answer |
|---|---|---|
| q504_M59 | ***q504_M59. Diazepam (oral)***<br>*Question relevant when: selected( ${q104_M59_full_list} , '4')* | |
| q504_M59a *(required)* | q504_M59a. What percent of patients require this item?<br>*Question relevant when: selected( ${q104_M59_full_list} , '4')*<br>*Response constrained to: .>0 and .<=100 or .=999* | |
| q504_M59b *(required)* | q504_M59b. How is the item administered, or given, to the patient?<br>*Question relevant when: selected( ${q104_M59_full_list} , '4')* | 1 Oral - solid (e.g. tablet)<br>2 Oral - liquid<br>3 Sublingual<br>4 Intravenous (IV)<br>5 Intramuscular injection (IM)<br>6 Subcutaneous<br>7 Rectal<br>8 Inhaled (e.g. gas)<br>9 Inserted (e.g. vaginal pessaries)<br>10 Topical - cream<br>11 Topical - drops (e.g. eye drops)<br>12 Other<br>99 Don't know |
| q504_M59c *(required)* | q504_M59c. What is the item's smallest unit of measurement (mg, mcg, ml, g, etc)?<br>*Question relevant when: selected( ${q104_M59_full_list} , '4')* | microgram — microgram (mcg)<br>miligram — miligram (mg)<br>miligram_dissolved_in_liquid — miligram (mg) dissolved in liquid (e.g. 2 mg per 3 militers)<br>gram — gram (g)<br>militer — militer (ml)<br>centiliter — centiliter (cl)<br>litre — litre (l)<br>ml_of _solution — ml of solution (e.g. ml of 10% solution)<br>litre_of_solution — litre of solution (e.g. 1 litre of 5% solution)<br>cubic_meter — cubic meter (m^3)<br>other — other<br>dont_know — don't know |
| q504_M59c.1 | q504_M59c.1 If other unit of measurement or route of administration, specify here:<br>*Leave blank if not applicable*<br>*Question relevant when: selected( ${q104_M59_full_list} , '4')* | |
| q504_M59d *(required)* | q504_M59d. In total, how many units are used to treat a patient during all of her care?<br>*Remember to add all units! E.g. 5 days x 200 mg twice per day = 2,000mg*<br>*Question relevant when: selected( ${q104_M59_full_list} , '4')*<br>*Response constrained to: .>0* | |

| Field | Question | Answer |
|---|---|---|
| q504_M60 | ***q504_M60. Antispasmodic, anxiolytic, sedative, etc - Other 1: "[q104_M60_full_list_other]"***<br>*Question relevant when: selected( ${q104_M60_full_list} , '4')* | |
| q504_M60a *(required)* | q504_M60a. What percent of patients require this item?<br>*Question relevant when: selected( ${q104_M60_full_list} , '4')*<br>*Response constrained to: .>0 and .<=100 or .=999* | |
| q504_M60b *(required)* | q504_M60b. How is the item administered, or given, to the patient?<br>*Question relevant when: selected( ${q104_M60_full_list} , '4')* | 1 Oral - solid (e.g. tablet)<br>2 Oral - liquid<br>3 Sublingual<br>4 Intravenous (IV)<br>5 Intramusculuar injection (IM)<br>6 Subcutaneous<br>7 Rectal<br>8 Inhaled (e.g. gas)<br>9 Inserted (e.g. vaginal pessaries)<br>10 Topical - cream<br>11 Topical - drops (e.g. eye drops)<br>12 Other<br>99 Don't know |
| q504_M60c *(required)* | q504_M60c. What is the item's smallest unit of measurement (mg, mcg, ml, g, etc)?<br>*Question relevant when: selected( ${q104_M60_full_list} , '4')* | microgram — microgram (mcg)<br>miligram — miligram (mg)<br>miligram_dissolved_in_liquid — miligram (mg) dissolved in liquid (e.g. 2 mg per 3 militers)<br>gram — gram (g)<br>militer — militer (ml)<br>centiliter — centiliter (cl)<br>litre — litre (l)<br>ml_of _solution — ml of solution (e.g. ml of 10% solution)<br>litre_of_solution — litre of solution (e.g. 1 litre of 5% solution)<br>cubic_meter — cubic meter (m^3)<br>other — other<br>dont_know — don't know |
| q504_M60c.1 | q504_M60c.1 If other unit of measurement or route of administration, specify here:<br>*Leave blank if not applicable*<br>*Question relevant when: selected( ${q104_M60_full_list} , '4')* | |
| q504_M60d *(required)* | q504_M60d. In total, how many units are used to treat a patient during all of her care?<br>*Remember to add all units! E.g. 5 days x 200 mg twice per day = 2,000mg*<br>*Question relevant when: selected( ${q104_M60_full_list} , '4')*<br>*Response constrained to: .>0* | |

| Field | Question | Answer |
|---|---|---|
| q504_M61 | ***q504_M61. Antispasmodic, anxiolytic, sedative, etc - Other 2: "[q104_M61_full_list_other]"***<br>*Question relevant when: selected( ${q104_M61_full_list} , '4')* | |
| q504_M61a *(required)* | q504_M61a. What percent of patients require this item?<br>*Question relevant when: selected( ${q104_M61_full_list} , '4')*<br>*Response constrained to: .>0 and .<=100 or .=999* | |

| Field | Question | Answer | | |
|---|---|---|---|---|
| q504_M61b *(required)* | q504_M61b. How is the item administered, or given, to the patient?<br>*Question relevant when: selected( ${q104_M61_full_list} , '4')* | | 1 | Oral - solid (e.g. tablet) |
| | | | 2 | Oral - liquid |
| | | | 3 | Sublingual |
| | | | 4 | Intravenous (IV) |
| | | | 5 | Intramuscular injection (IM) |
| | | | 6 | Subcutaneous |
| | | | 7 | Rectal |
| | | | 8 | Inhaled (e.g. gas) |
| | | | 9 | Inserted (e.g. vaginal pessaries) |
| | | | 10 | Topical - cream |
| | | | 11 | Topical - drops (e.g. eye drops) |
| | | | 12 | Other |
| | | | 99 | Don't know |
| q504_M61c *(required)* | q504_M61c. What is the item's smallest unit of measurement (mg, mcg, ml, g, etc)?<br>*Question relevant when: selected( ${q104_M61_full_list} , '4')* | | microgram | microgram (mcg) |
| | | | miligram | miligram (mg) |
| | | | miligram_dissolved_in_liquid | miligram (mg) dissolved in liquid (e.g. 2 mg per 3 militers) |
| | | | gram | gram (g) |
| | | | militer | militer (ml) |
| | | | centiliter | centiliter (cl) |
| | | | litre | litre (l) |
| | | | ml_of _solution | ml of solution (e.g. ml of 10% solution) |
| | | | litre_of_solution | litre of solution (e.g. 1 litre of 5% solution) |
| | | | cubic_meter | cubic meter (m^3) |
| | | | other | other |
| | | | dont_know | don't know |
| q504_M61c.1 | q504_M61c.1 If other unit of measurement or route of administration, specify here:<br>*Leave blank if not applicable*<br>*Question relevant when: selected( ${q104_M61_full_list} , '4')* | | | |
| q504_M61d *(required)* | q504_M61d. In total, how many units are used to treat a patient during all of her care?<br>*Remember to add all units! E.g. 5 days x 200 mg twice per day = 2,000mg*<br>*Question relevant when: selected( ${q104_M61_full_list} , '4')*<br>*Response constrained to: .>0* | | | |

| Field | Question | Answer |
|---|---|---|
| q504_M62 | ***q504_M62. Antispasmodic, anxiolytic, sedative, etc - Other 3: "[q104_M62_full_list_other]"***<br>*Question relevant when: selected( ${q104_M62_full_list} , '4')* | |
| q504_M62a *(required)* | q504_M62a. What percent of patients require this item?<br>*Question relevant when: selected( ${q104_M62_full_list} , '4')*<br>*Response constrained to: .>0 and .<=100 or .=999* | |
| q504_M62b *(required)* | q504_M62b. How is the item administered, or given, to the patient?<br>*Question relevant when: selected( ${q104_M62_full_list} , '4')* | 1 Oral - solid (e.g. tablet)<br>2 Oral - liquid<br>3 Sublingual<br>4 Intravenous (IV)<br>5 Intramuscular injection (IM)<br>6 Subcutaneous<br>7 Rectal<br>8 Inhaled (e.g. gas)<br>9 Inserted (e.g. vaginal pessaries)<br>10 Topical - cream<br>11 Topical - drops (e.g. eye drops)<br>12 Other<br>99 Don't know |
| q504_M62c *(required)* | q504_M62c. What is the item's smallest unit of measurement (mg, mcg, ml, g, etc)?<br>*Question relevant when: selected( ${q104_M62_full_list} , '4')* | microgram — microgram (mcg)<br>miligram — miligram (mg)<br>miligram_dissolved_in_liquid — miligram (mg) dissolved in liquid (e.g. 2 mg per 3 militers)<br>gram — gram (g)<br>militer — militer (ml)<br>centiliter — centiliter (cl)<br>litre — litre (l)<br>ml_of _solution — ml of solution (e.g. ml of 10% solution)<br>litre_of_solution — litre of solution (e.g. 1 litre of 5% solution)<br>cubic_meter — cubic meter (m^3)<br>other — other<br>dont_know — don't know |
| q504_M62c.1 | q504_M62c.1 If other unit of measurement or route of administration, specify here:<br>*Leave blank if not applicable*<br>*Question relevant when: selected( ${q104_M62_full_list} , '4')* | |
| q504_M62d *(required)* | q504_M62d. In total, how many units are used to treat a patient during all of her care?<br>*Remember to add all units! E.g. 5 days x 200 mg twice per day = 2,000mg*<br>*Question relevant when: selected( ${q104_M62_full_list} , '4')*<br>*Response constrained to: .>0* | |

| Field | Question | Answer |
|---|---|---|
| q504_M63 | ***q504_M63. Antispasmodic, anxiolytic, sedative, etc - Other 4: "[q104_M63_full_list_other]"***<br>*Question relevant when: selected( ${q104_M63_full_list} , '4')* | |
| q504_M63a *(required)* | q504_M63a. What percent of patients require this item?<br>*Question relevant when: selected( ${q104_M63_full_list} , '4')*<br>*Response constrained to: .>0 and .<=100 or .=999* | |
| q504_M63b *(required)* | q504_M63b. How is the item administered, or given, to the patient?<br>*Question relevant when: selected( ${q104_M63_full_list} , '4')* | 1 Oral - solid (e.g. tablet)<br>2 Oral - liquid<br>3 Sublingual<br>4 Intravenous (IV)<br>5 Intramuscular injection (IM)<br>6 Subcutaneous<br>7 Rectal<br>8 Inhaled (e.g. gas)<br>9 Inserted (e.g. vaginal pessaries)<br>10 Topical - cream<br>11 Topical - drops (e.g. eye drops)<br>12 Other<br>99 Don't know |
| q504_M63c *(required)* | q504_M63c. What is the item's smallest unit of measurement (mg, mcg, ml, g, etc)?<br>*Question relevant when: selected( ${q104_M63_full_list} , '4')* | microgram — microgram (mcg)<br>miligram — miligram (mg)<br>miligram_dissolved_in_liquid — miligram (mg) dissolved in liquid (e.g. 2 mg per 3 militers)<br>gram — gram (g)<br>militer — militer (ml)<br>centiliter — centiliter (cl)<br>litre — litre (l)<br>ml_of _solution — ml of solution (e.g. ml of 10% solution)<br>litre_of_solution — litre of solution (e.g. 1 litre of 5% solution)<br>cubic_meter — cubic meter (m^3)<br>other — other<br>dont_know — don't know |
| q504_M63c.1 | q504_M63c.1 If other unit of measurement or route of administration, specify here:<br>*Leave blank if not applicable*<br>*Question relevant when: selected( ${q104_M63_full_list} , '4')* | |
| q504_M63d *(required)* | q504_M63d. In total, how many units are used to treat a patient during all of her care?<br>*Remember to add all units! E.g. 5 days x 200 mg twice per day = 2,000mg*<br>*Question relevant when: selected( ${q104_M63_full_list} , '4')*<br>*Response constrained to: .>0* | |

| Field | Question | Answer |
|---|---|---|
| q504_M64 | ***q504_M64. Antispasmodic, anxiolytic, sedative, etc - Other 5: "[q104_M64_full_list_other]"***<br>*Question relevant when: selected( ${q104_M64_full_list} , '4')* | |
| q504_M64a *(required)* | q504_M64a. What percent of patients require this item?<br>*Question relevant when: selected( ${q104_M64_full_list} , '4')*<br>*Response constrained to: .>0 and .<=100 or .=999* | |
| q504_M64b *(required)* | q504_M64b. How is the item administered, or given, to the patient?<br>*Question relevant when: selected( ${q104_M64_full_list} , '4')* | 1 Oral - solid (e.g. tablet)<br>2 Oral - liquid<br>3 Sublingual<br>4 Intravenous (IV)<br>5 Intramusculuar injection (IM)<br>6 Subcutaneous<br>7 Rectal<br>8 Inhaled (e.g. gas)<br>9 Inserted (e.g. vaginal pessaries)<br>10 Topical - cream<br>11 Topical - drops (e.g. eye drops)<br>12 Other<br>99 Don't know |
| q504_M64c *(required)* | q504_M64c. What is the item's smallest unit of measurement (mg, mcg, ml, g, etc)?<br>*Question relevant when: selected( ${q104_M64_full_list} , '4')* | microgram — microgram (mcg)<br>miligram — miligram (mg)<br>miligram_dissolved_in_liquid — miligram (mg) dissolved in liquid (e.g. 2 mg per 3 militers)<br>gram — gram (g)<br>militer — militer (ml)<br>centiliter — centiliter (cl)<br>litre — litre (l)<br>ml_of _solution — ml of solution (e.g. ml of 10% solution)<br>litre_of_solution — litre of solution (e.g. 1 litre of 5% solution)<br>cubic_meter — cubic meter (m^3)<br>other — other<br>dont_know — don't know |
| q504_M64c.1 | q504_M64c.1 If other unit of measurement or route of administration, specify here:<br>*Leave blank if not applicable*<br>*Question relevant when: selected( ${q104_M64_full_list} , '4')* | |
| q504_M64d *(required)* | q504_M64d. In total, how many units are used to treat a patient during all of her care?<br>*Remember to add all units! E.g. 5 days x 200 mg twice per day = 2,000mg*<br>*Question relevant when: selected( ${q104_M64_full_list} , '4')*<br>*Response constrained to: .>0* | |

<p><strong><em>D. Medications - Lacerations (6)</em></strong></p>

*Group relevant when: selected( ${section_five_skip_med} , '1')*

| Field | Question | Answer |
|---|---|---|
| q504_med_notE5 | 504 CONTINUED For each of the following medications and other medical products that are used for lacerations, can you tell me …? | |
| note_504_adrenergic | ***Adrenergic*** | |
| q504_M65 | ***q504_M65. Ephedrine HCl***<br>*Question relevant when: selected( ${q104_M65_full_list} , '4')* | |
| q504_M65a *(required)* | q504_M65a. What percent of patients require this item?<br>*Question relevant when: selected( ${q104_M65_full_list} , '4')*<br>*Response constrained to: .>0 and .<=100 or .=999* | |

| Field | Question | Answer |
|---|---|---|
| q504_M65b *(required)* | q504_M65b. How is the item administered, or given, to the patient?<br>*Question relevant when: selected( ${q104_M65_full_list} , '4')* | 1 Oral - solid (e.g. tablet)<br>2 Oral - liquid<br>3 Sublingual<br>4 Intravenous (IV)<br>5 Intramusculuar injection (IM)<br>6 Subcutaneous<br>7 Rectal<br>8 Inhaled (e.g. gas)<br>9 Inserted (e.g. vaginal pessaries)<br>10 Topical - cream<br>11 Topical - drops (e.g. eye drops)<br>12 Other<br>99 Don't know |
| q504_M65c *(required)* | q504_M65c. What is the item's smallest unit of measurement (mg, mcg, ml, g, etc)?<br>*Question relevant when: selected( ${q104_M65_full_list} , '4')* | microgram — microgram (mcg)<br>miligram — miligram (mg)<br>miligram_dissolved_in_liquid — miligram (mg) dissolved in liquid (e.g. 2 mg per 3 militers)<br>gram — gram (g)<br>militer — militer (ml)<br>centiliter — centiliter (cl)<br>litre — litre (l)<br>ml_of _solution — ml of solution (e.g. ml of 10% solution)<br>litre_of_solution — litre of solution (e.g. 1 litre of 5% solution)<br>cubic_meter — cubic meter (m^3)<br>other — other<br>dont_know — don't know |
| q504_M65c.1 | q504_M65c.1 If other unit of measurement or route of administration, specify here:<br>*Leave blank if not applicable*<br>*Question relevant when: selected( ${q104_M65_full_list} , '4')* | |
| q504_M65d *(required)* | q504_M65d. In total, how many units are used to treat a patient during all of her care?<br>*Remember to add all units! E.g. 5 days x 200 mg twice per day = 2,000mg*<br>*Question relevant when: selected( ${q104_M65_full_list} , '4')*<br>*Response constrained to: .>0* | |

| Field | Question | Answer |
|---|---|---|
| q504_M66 | ***q504_M66. Epinephrine***<br>*Question relevant when: selected( ${q104_M66_full_list} , '4')* | |
| q504_M66a *(required)* | q504_M66a. What percent of patients require this item?<br>*Question relevant when: selected( ${q104_M66_full_list} , '4')*<br>*Response constrained to: .>0 and .<=100 or .=999* | |

| Field | Question | Answer | | |
|---|---|---|---|---|
| q504_M66b *(required)* | q504_M66b. How is the item administered, or given, to the patient?<br>*Question relevant when: selected( ${q104_M66_full_list} , '4')* | | 1 | Oral - solid (e.g. tablet) |
| | | | 2 | Oral - liquid |
| | | | 3 | Sublingual |
| | | | 4 | Intravenous (IV) |
| | | | 5 | Intramuscular injection (IM) |
| | | | 6 | Subcutaneous |
| | | | 7 | Rectal |
| | | | 8 | Inhaled (e.g. gas) |
| | | | 9 | Inserted (e.g. vaginal pessaries) |
| | | | 10 | Topical - cream |
| | | | 11 | Topical - drops (e.g. eye drops) |
| | | | 12 | Other |
| | | | 99 | Don't know |
| q504_M66c *(required)* | q504_M66c. What is the item's smallest unit of measurement (mg, mcg, ml, g, etc)?<br>*Question relevant when: selected( ${q104_M66_full_list} , '4')* | | microgram | microgram (mcg) |
| | | | miligram | miligram (mg) |
| | | | miligram_dissolved_in_liquid | miligram (mg) dissolved in liquid (e.g. 2 mg per 3 militers) |
| | | | gram | gram (g) |
| | | | militer | militer (ml) |
| | | | centiliter | centiliter (cl) |
| | | | litre | litre (l) |
| | | | ml_of _solution | ml of solution (e.g. ml of 10% solution) |
| | | | litre_of_solution | litre of solution (e.g. 1 litre of 5% solution) |
| | | | cubic_meter | cubic meter (m^3) |
| | | | other | other |
| | | | dont_know | don't know |
| q504_M66c.1 | q504_M66c.1 If other unit of measurement or route of administration, specify here:<br>*Leave blank if not applicable*<br>*Question relevant when: selected( ${q104_M66_full_list} , '4')* | | | |
| q504_M66d *(required)* | q504_M66d. In total, how many units are used to treat a patient during all of her care?<br>*Remember to add all units! E.g. 5 days x 200 mg twice per day = 2,000mg*<br>*Question relevant when: selected( ${q104_M66_full_list} , '4')*<br>*Response constrained to: .>0* | | | |

| Field | Question | Answer |
|---|---|---|
| q504_M67 | ***q504_M67. Adrenergic - other 1: "[q104_M67_full_list_other]"***<br>*Question relevant when: selected( ${q104_M67_full_list} , '4')* | |
| q504_M67a *(required)* | q504_M67a. What percent of patients require this item?<br>*Question relevant when: selected( ${q104_M67_full_list} , '4')*<br>*Response constrained to: .>0 and .<=100 or .=999* | |
| q504_M67b *(required)* | q504_M67b. How is the item administered, or given, to the patient?<br>*Question relevant when: selected( ${q104_M67_full_list} , '4')* | 1 Oral - solid (e.g. tablet)<br>2 Oral - liquid<br>3 Sublingual<br>4 Intravenous (IV)<br>5 Intramusculuar injection (IM)<br>6 Subcutaneous<br>7 Rectal<br>8 Inhaled (e.g. gas)<br>9 Inserted (e.g. vaginal pessaries)<br>10 Topical - cream<br>11 Topical - drops (e.g. eye drops)<br>12 Other<br>99 Don't know |
| q504_M67c *(required)* | q504_M67c. What is the item's smallest unit of measurement (mg, mcg, ml, g, etc)?<br>*Question relevant when: selected( ${q104_M67_full_list} , '4')* | microgram — microgram (mcg)<br>miligram — miligram (mg)<br>miligram_dissolved_in_liquid — miligram (mg) dissolved in liquid (e.g. 2 mg per 3 militers)<br>gram — gram (g)<br>militer — militer (ml)<br>centiliter — centiliter (cl)<br>litre — litre (l)<br>ml_of _solution — ml of solution (e.g. ml of 10% solution)<br>litre_of_solution — litre of solution (e.g. 1 litre of 5% solution)<br>cubic_meter — cubic meter (m^3)<br>other — other<br>dont_know — don't know |
| q504_M67c.1 | q504_M67c.1 If other unit of measurement or route of administration, specify here:<br>*Leave blank if not applicable*<br>*Question relevant when: selected( ${q104_M67_full_list} , '4')* | |
| q504_M67d *(required)* | q504_M67d. In total, how many units are used to treat a patient during all of her care?<br>*Remember to add all units! E.g. 5 days x 200 mg twice per day = 2,000mg*<br>*Question relevant when: selected( ${q104_M67_full_list} , '4')*<br>*Response constrained to: .>0* | |

| Field | Question | Answer |
|---|---|---|
| q504_M68 | ***q504_M68. Adrenergic - other 2: "[q104_M68_full_list_other]"***<br>*Question relevant when: selected( ${q104_M68_full_list} , '4')* | |
| q504_M68a *(required)* | q504_M68a. What percent of patients require this item?<br>*Question relevant when: selected( ${q104_M68_full_list} , '4')*<br>*Response constrained to: .>0 and .<=100 or .=999* | |
| q504_M68b *(required)* | q504_M68b. How is the item administered, or given, to the patient?<br>*Question relevant when: selected( ${q104_M68_full_list} , '4')* | 1 Oral - solid (e.g. tablet)<br>2 Oral - liquid<br>3 Sublingual<br>4 Intravenous (IV)<br>5 Intramuscular injection (IM)<br>6 Subcutaneous<br>7 Rectal<br>8 Inhaled (e.g. gas)<br>9 Inserted (e.g. vaginal pessaries)<br>10 Topical - cream<br>11 Topical - drops (e.g. eye drops)<br>12 Other<br>99 Don't know |
| q504_M68c *(required)* | q504_M68c. What is the item's smallest unit of measurement (mg, mcg, ml, g, etc)?<br>*Question relevant when: selected( ${q104_M68_full_list} , '4')* | microgram — microgram (mcg)<br>miligram — miligram (mg)<br>miligram_dissolved_in_liquid — miligram (mg) dissolved in liquid (e.g. 2 mg per 3 militers)<br>gram — gram (g)<br>militer — militer (ml)<br>centiliter — centiliter (cl)<br>litre — litre (l)<br>ml_of _solution — ml of solution (e.g. ml of 10% solution)<br>litre_of_solution — litre of solution (e.g. 1 litre of 5% solution)<br>cubic_meter — cubic meter (m^3)<br>other — other<br>dont_know — don't know |
| q504_M68c.1 | q504_M68c.1 If other unit of measurement or route of administration, specify here:<br>*Leave blank if not applicable*<br>*Question relevant when: selected( ${q104_M68_full_list} , '4')* | |
| q504_M68d *(required)* | q504_M68d. In total, how many units are used to treat a patient during all of her care?<br>*Remember to add all units! E.g. 5 days x 200 mg twice per day = 2,000mg*<br>*Question relevant when: selected( ${q104_M68_full_list} , '4')*<br>*Response constrained to: .>0* | |

| Field | Question | Answer |
|---|---|---|
| q504_M69 | ***q504_M69. Adrenergic - other 3: "[q104_M69_full_list_other]"***<br>*Question relevant when: selected( ${q104_M69_full_list} , '4')* | |
| q504_M69a *(required)* | q504_M69a. What percent of patients require this item?<br>*Question relevant when: selected( ${q104_M69_full_list} , '4')*<br>*Response constrained to: .>0 and .<=100 or .=999* | |
| q504_M69b *(required)* | q504_M69b. How is the item administered, or given, to the patient?<br>*Question relevant when: selected( ${q104_M69_full_list} , '4')* | 1 Oral - solid (e.g. tablet)<br>2 Oral - liquid<br>3 Sublingual<br>4 Intravenous (IV)<br>5 Intramusculuar injection (IM)<br>6 Subcutaneous<br>7 Rectal<br>8 Inhaled (e.g. gas)<br>9 Inserted (e.g. vaginal pessaries)<br>10 Topical - cream<br>11 Topical - drops (e.g. eye drops)<br>12 Other<br>99 Don't know |
| q504_M69c *(required)* | q504_M69c. What is the item's smallest unit of measurement (mg, mcg, ml, g, etc)?<br>*Question relevant when: selected( ${q104_M69_full_list} , '4')* | microgram — microgram (mcg)<br>miligram — miligram (mg)<br>miligram_dissolved_in_liquid — miligram (mg) dissolved in liquid (e.g. 2 mg per 3 militers)<br>gram — gram (g)<br>militer — militer (ml)<br>centiliter — centiliter (cl)<br>litre — litre (l)<br>ml_of _solution — ml of solution (e.g. ml of 10% solution)<br>litre_of_solution — litre of solution (e.g. 1 litre of 5% solution)<br>cubic_meter — cubic meter (m^3)<br>other — other<br>dont_know — don't know |
| q504_M69c.1 | q504_M69c.1 If other unit of measurement or route of administration, specify here:<br>*Leave blank if not applicable*<br>*Question relevant when: selected( ${q104_M69_full_list} , '4')* | |
| q504_M69d *(required)* | q504_M69d. In total, how many units are used to treat a patient during all of her care?<br>*Remember to add all units! E.g. 5 days x 200 mg twice per day = 2,000mg*<br>*Question relevant when: selected( ${q104_M69_full_list} , '4')*<br>*Response constrained to: .>0* | |

| Field | Question | Answer |
|---|---|---|
| q504_M69.1 | ***q504_M69.1. Adrenergic - other 4: "[q104_M69.1_full_list_other]"***<br>*Question relevant when: selected( ${q104_M69.1_full_list} , '2')* | |
| q504_M69.1a *(required)* | q504_M69.1a. What percent of patients require this item?<br>*Question relevant when: selected( ${q104_M69.1_full_list} , '2')*<br>*Response constrained to: .>0 and .<=100 or .=999* | |
| q504_M69.1b *(required)* | q504_M69.1b. How is the item administered, or given, to the patient?<br>*Question relevant when: selected( ${q104_M69.1_full_list} , '2')* | 1 Oral - solid (e.g. tablet)<br>2 Oral - liquid<br>3 Sublingual<br>4 Intravenous (IV)<br>5 Intramusculuar injection (IM)<br>6 Subcutaneous<br>7 Rectal<br>8 Inhaled (e.g. gas)<br>9 Inserted (e.g. vaginal pessaries)<br>10 Topical - cream<br>11 Topical - drops (e.g. eye drops)<br>12 Other<br>99 Don't know |
| q504_M69.1c *(required)* | q504_M69.1c. What is the item's smallest unit of measurement (mg, mcg, ml, g, etc)?<br>*Question relevant when: selected( ${q104_M69.1_full_list} , '2')* | microgram — microgram (mcg)<br>miligram — miligram (mg)<br>miligram_dissolved_in_liquid — miligram (mg) dissolved in liquid (e.g. 2 mg per 3 militers)<br>gram — gram (g)<br>militer — militer (ml)<br>centiliter — centiliter (cl)<br>litre — litre (l)<br>ml_of _solution — ml of solution (e.g. ml of 10% solution)<br>litre_of_solution — litre of solution (e.g. 1 litre of 5% solution)<br>cubic_meter — cubic meter (m^3)<br>other — other<br>dont_know — don't know |
| q504_M69.1c.1 | q504_M69.1c.1 If other unit of measurement or route of administration, specify here:<br>*Leave blank if not applicable*<br>*Question relevant when: selected( ${q104_M69.1_full_list} , '2')* | |
| q504_M69.1d *(required)* | q504_M69.1d. In total, how many units are used to treat a patient during all of her care?<br>*Remember to add all units! E.g. 5 days x 200 mg twice per day = 2,000mg*<br>*Question relevant when: selected( ${q104_M69.1_full_list} , '2')*<br>*Response constrained to: .>0* | |

| Field | Question | Answer |
|---|---|---|
| q504_M69.2 | ***q504_M69.2. Adrenergic - other 5: "[q104_M69.2_full_list_other]"***<br>*Question relevant when: selected( ${q104_M69.2_full_list} , '2')* | |
| q504_M69.2a *(required)* | q504_M69.2a. What percent of patients require this item?<br>*Question relevant when: selected( ${q104_M69.2_full_list} , '2')*<br>*Response constrained to: .>0 and .<=100 or .=999* | |
| q504_M69.2b *(required)* | q504_M69.2b. How is the item administered, or given, to the patient?<br>*Question relevant when: selected( ${q104_M69.2_full_list} , '2')* | 1 Oral - solid (e.g. tablet)<br>2 Oral - liquid<br>3 Sublingual<br>4 Intravenous (IV)<br>5 Intramusculuar injection (IM)<br>6 Subcutaneous<br>7 Rectal<br>8 Inhaled (e.g. gas)<br>9 Inserted (e.g. vaginal pessaries)<br>10 Topical - cream<br>11 Topical - drops (e.g. eye drops)<br>12 Other<br>99 Don't know |
| q504_M69.2c *(required)* | q504_M69.2c. What is the item's smallest unit of measurement (mg, mcg, ml, g, etc)?<br>*Question relevant when: selected( ${q104_M69.2_full_list} , '2')* | microgram — microgram (mcg)<br>miligram — miligram (mg)<br>miligram_dissolved_in_liquid — miligram (mg) dissolved in liquid (e.g. 2 mg per 3 militers)<br>gram — gram (g)<br>militer — militer (ml)<br>centiliter — centiliter (cl)<br>litre — litre (l)<br>ml_of _solution — ml of solution (e.g. ml of 10% solution)<br>litre_of_solution — litre of solution (e.g. 1 litre of 5% solution)<br>cubic_meter — cubic meter (m^3)<br>other — other<br>dont_know — don't know |
| q504_M69.2c.1 | q504_M69.2c.1 If other unit of measurement or route of administration, specify here:<br>*Leave blank if not applicable*<br>*Question relevant when: selected( ${q104_M69.2_full_list} , '2')* | |
| q504_M69.2d *(required)* | q504_M69.2d. In total, how many units are used to treat a patient during all of her care?<br>*Remember to add all units! E.g. 5 days x 200 mg twice per day = 2,000mg*<br>*Question relevant when: selected( ${q104_M69.2_full_list} , '2')*<br>*Response constrained to: .>0* | |

<p><strong><em>D. Medications - Lacerations (7)</em></strong></p>

*Group relevant when: selected( ${section_five_skip_med} , '1')*

| Field | Question | Answer |
|---|---|---|
| q504_med_notE6 | 504 CONTINUED For each of the following medications and other medical products that are used for lacerations, can you tell me …? | |
| note_504_blood | ***Blood products*** | |
| q504_M70 | ***q504_M70. Unit of packed red blood cells***<br>*Question relevant when: selected( ${q104_M70_full_list} , '4')* | |
| q504_M70a *(required)* | q504_M70a. What percent of patients require this item?<br>*Question relevant when: selected( ${q104_M70_full_list} , '4')*<br>*Response constrained to: .>0 and .<=100 or .=999* | |

| Field | Question | Answer |
|---|---|---|
| q504_M70b *(required)* | q504_M70b. How is the item administered, or given, to the patient?<br>*Question relevant when: selected( ${q104_M70_full_list} , '4')* | 1 Oral - solid (e.g. tablet)<br>2 Oral - liquid<br>3 Sublingual<br>4 Intravenous (IV)<br>5 Intramuscular injection (IM)<br>6 Subcutaneous<br>7 Rectal<br>8 Inhaled (e.g. gas)<br>9 Inserted (e.g. vaginal pessaries)<br>10 Topical - cream<br>11 Topical - drops (e.g. eye drops)<br>12 Other<br>99 Don't know |
| q504_M70c *(required)* | q504_M70c. What is the item's smallest unit of measurement (mg, mcg, ml, g, etc)?<br>*Question relevant when: selected( ${q104_M70_full_list} , '4')* | microgram — microgram (mcg)<br>miligram — miligram (mg)<br>miligram_dissolved_in_liquid — miligram (mg) dissolved in liquid (e.g. 2 mg per 3 militers)<br>gram — gram (g)<br>militer — militer (ml)<br>centiliter — centiliter (cl)<br>litre — litre (l)<br>ml_of _solution — ml of solution (e.g. ml of 10% solution)<br>litre_of_solution — litre of solution (e.g. 1 litre of 5% solution)<br>cubic_meter — cubic meter (m^3)<br>other — other<br>dont_know — don't know |
| q504_M70c.1 | q504_M70c.1 If other unit of measurement or route of administration, specify here:<br>*Leave blank if not applicable*<br>*Question relevant when: selected( ${q104_M70_full_list} , '4')* | |
| q504_M70d *(required)* | q504_M70d. In total, how many units are used to treat a patient during all of her care?<br>*Remember to add all units! E.g. 5 days x 200 mg twice per day = 2,000mg*<br>*Question relevant when: selected( ${q104_M70_full_list} , '4')*<br>*Response constrained to: .>0* | |

| Field | Question | Answer |
|---|---|---|
| q504_M71 | ***q504_M71. Unit of platelets***<br>*Question relevant when: selected( ${q104_M71_full_list} , '4')* | |
| q504_M71a *(required)* | q504_M71a. What percent of patients require this item?<br>*Question relevant when: selected( ${q104_M71_full_list} , '4')*<br>*Response constrained to: .>0 and .<=100 or .=999* | |
| q504_M71b *(required)* | q504_M71b. How is the item administered, or given, to the patient?<br>*Question relevant when: selected( ${q104_M71_full_list} , '4')* | 1 Oral - solid (e.g. tablet)<br>2 Oral - liquid<br>3 Sublingual<br>4 Intravenous (IV)<br>5 Intramusculuar injection (IM)<br>6 Subcutaneous<br>7 Rectal<br>8 Inhaled (e.g. gas)<br>9 Inserted (e.g. vaginal pessaries)<br>10 Topical - cream<br>11 Topical - drops (e.g. eye drops)<br>12 Other<br>99 Don't know |
| q504_M71c *(required)* | q504_M71c. What is the item's smallest unit of measurement (mg, mcg, ml, g, etc)?<br>*Question relevant when: selected( ${q104_M71_full_list} , '4')* | microgram: microgram (mcg)<br>miligram: miligram (mg)<br>miligram_dissolved_in_liquid: miligram (mg) dissolved in liquid (e.g. 2 mg per 3 militers)<br>gram: gram (g)<br>militer: militer (ml)<br>centiliter: centiliter (cl)<br>litre: litre (l)<br>ml_of _solution: ml of solution (e.g. ml of 10% solution)<br>litre_of_solution: litre of solution (e.g. 1 litre of 5% solution)<br>cubic_meter: cubic meter (m^3)<br>other: other<br>dont_know: don't know |
| q504_M71c.1 | q504_M71c.1 If other unit of measurement or route of administration, specify here:<br>*Leave blank if not applicable*<br>*Question relevant when: selected( ${q104_M71_full_list} , '4')* | |
| q504_M71d *(required)* | q504_M71d. In total, how many units are used to treat a patient during all of her care?<br>*Remember to add all units! E.g. 5 days x 200 mg twice per day = 2,000mg*<br>*Question relevant when: selected( ${q104_M71_full_list} , '4')*<br>*Response constrained to: .>0* | |

| Field | Question | Answer |
|---|---|---|
| q504_M72 | ***q504_M72. Unit of whole blood***<br>*Question relevant when: selected( ${q104_M72_full_list} , '4')* | |
| q504_M72a *(required)* | q504_M72a. What percent of patients require this item?<br>*Question relevant when: selected( ${q104_M72_full_list} , '4')*<br>*Response constrained to: .>0 and .<=100 or .=999* | |
| q504_M72b *(required)* | q504_M72b. How is the item administered, or given, to the patient?<br>*Question relevant when: selected( ${q104_M72_full_list} , '4')* | 1 Oral - solid (e.g. tablet)<br>2 Oral - liquid<br>3 Sublingual<br>4 Intravenous (IV)<br>5 Intramuscular injection (IM)<br>6 Subcutaneous<br>7 Rectal<br>8 Inhaled (e.g. gas)<br>9 Inserted (e.g. vaginal pessaries)<br>10 Topical - cream<br>11 Topical - drops (e.g. eye drops)<br>12 Other<br>99 Don't know |
| q504_M72c *(required)* | q504_M72c. What is the item's smallest unit of measurement (mg, mcg, ml, g, etc)?<br>*Question relevant when: selected( ${q104_M72_full_list} , '4')* | microgram — microgram (mcg)<br>miligram — miligram (mg)<br>miligram_dissolved_in_liquid — miligram (mg) dissolved in liquid (e.g. 2 mg per 3 militers)<br>gram — gram (g)<br>militer — militer (ml)<br>centiliter — centiliter (cl)<br>litre — litre (l)<br>ml_of _solution — ml of solution (e.g. ml of 10% solution)<br>litre_of_solution — litre of solution (e.g. 1 litre of 5% solution)<br>cubic_meter — cubic meter (m^3)<br>other — other<br>dont_know — don't know |
| q504_M72c.1 | q504_M72c.1 If other unit of measurement or route of administration, specify here:<br>*Leave blank if not applicable*<br>*Question relevant when: selected( ${q104_M72_full_list} , '4')* | |
| q504_M72d *(required)* | q504_M72d. In total, how many units are used to treat a patient during all of her care?<br>*Remember to add all units! E.g. 5 days x 200 mg twice per day = 2,000mg*<br>*Question relevant when: selected( ${q104_M72_full_list} , '4')*<br>*Response constrained to: .>0* | |

| Field | Question | Answer |
|---|---|---|
| q504_M73 | ***q504_M73. Blood product - other 1: "[q104_M73_full_list_other]"***<br>*Question relevant when: selected( ${q104_M73_full_list} , '4')* | |
| q504_M73a *(required)* | q504_M73a. What percent of patients require this item?<br>*Question relevant when: selected( ${q104_M73_full_list} , '4')*<br>*Response constrained to: .>0 and .<=100 or .=999* | |
| q504_M73b *(required)* | q504_M73b. How is the item administered, or given, to the patient?<br>*Question relevant when: selected( ${q104_M73_full_list} , '4')* | 1 Oral - solid (e.g. tablet)<br>2 Oral - liquid<br>3 Sublingual<br>4 Intravenous (IV)<br>5 Intramuscular injection (IM)<br>6 Subcutaneous<br>7 Rectal<br>8 Inhaled (e.g. gas)<br>9 Inserted (e.g. vaginal pessaries)<br>10 Topical - cream<br>11 Topical - drops (e.g. eye drops)<br>12 Other<br>99 Don't know |
| q504_M73c *(required)* | q504_M73c. What is the item's smallest unit of measurement (mg, mcg, ml, g, etc)?<br>*Question relevant when: selected( ${q104_M73_full_list} , '4')* | microgram — microgram (mcg)<br>miligram — miligram (mg)<br>miligram_dissolved_in_liquid — miligram (mg) dissolved in liquid (e.g. 2 mg per 3 militers)<br>gram — gram (g)<br>militer — militer (ml)<br>centiliter — centiliter (cl)<br>litre — litre (l)<br>ml_of _solution — ml of solution (e.g. ml of 10% solution)<br>litre_of_solution — litre of solution (e.g. 1 litre of 5% solution)<br>cubic_meter — cubic meter (m^3)<br>other — other<br>dont_know — don't know |
| q504_M73c.1 | q504_M73c.1 If other unit of measurement or route of administration, specify here:<br>*Leave blank if not applicable*<br>*Question relevant when: selected( ${q104_M73_full_list} , '4')* | |
| q504_M73d *(required)* | q504_M73d. In total, how many units are used to treat a patient during all of her care?<br>*Remember to add all units! E.g. 5 days x 200 mg twice per day = 2,000mg*<br>*Question relevant when: selected( ${q104_M73_full_list} , '4')*<br>*Response constrained to: .>0* | |

| Field | Question | Answer |
|---|---|---|
| q504_M74 | ***q504_M74. Blood product - other 2: "[q104_M74_full_list_other]"***<br>*Question relevant when: selected( ${q104_M74_full_list} , '4')* | |
| q504_M74a *(required)* | q504_M74a. What percent of patients require this item?<br>*Question relevant when: selected( ${q104_M74_full_list} , '4')*<br>*Response constrained to: .>0 and .<=100 or .=999* | |
| q504_M74b *(required)* | q504_M74b. How is the item administered, or given, to the patient?<br>*Question relevant when: selected( ${q104_M74_full_list} , '4')* | 1 Oral - solid (e.g. tablet)<br>2 Oral - liquid<br>3 Sublingual<br>4 Intravenous (IV)<br>5 Intramuscular injection (IM)<br>6 Subcutaneous<br>7 Rectal<br>8 Inhaled (e.g. gas)<br>9 Inserted (e.g. vaginal pessaries)<br>10 Topical - cream<br>11 Topical - drops (e.g. eye drops)<br>12 Other<br>99 Don't know |
| q504_M74c *(required)* | q504_M74c. What is the item's smallest unit of measurement (mg, mcg, ml, g, etc)?<br>*Question relevant when: selected( ${q104_M74_full_list} , '4')* | microgram — microgram (mcg)<br>miligram — miligram (mg)<br>miligram_dissolved_in_liquid — miligram (mg) dissolved in liquid (e.g. 2 mg per 3 militers)<br>gram — gram (g)<br>militer — militer (ml)<br>centiliter — centiliter (cl)<br>litre — litre (l)<br>ml_of _solution — ml of solution (e.g. ml of 10% solution)<br>litre_of_solution — litre of solution (e.g. 1 litre of 5% solution)<br>cubic_meter — cubic meter (m^3)<br>other — other<br>dont_know — don't know |
| q504_M74c.1 | q504_M74c.1 If other unit of measurement or route of administration, specify here:<br>*Leave blank if not applicable*<br>*Question relevant when: selected( ${q104_M74_full_list} , '4')* | |
| q504_M74d *(required)* | q504_M74d. In total, how many units are used to treat a patient during all of her care?<br>*Remember to add all units! E.g. 5 days x 200 mg twice per day = 2,000mg*<br>*Question relevant when: selected( ${q104_M74_full_list} , '4')*<br>*Response constrained to: .>0* | |

| Field | Question | Answer |
|---|---|---|
| q504_M75 | ***q504_M75. Blood product - other 3: "[q104_M75_full_list_other]"***<br>*Question relevant when: selected( ${q104_M75_full_list} , '4')* | |
| q504_M75a *(required)* | q504_M75a. What percent of patients require this item?<br>*Question relevant when: selected( ${q104_M75_full_list} , '4')*<br>*Response constrained to: .>0 and .<=100 or .=999* | |

| Field | Question | Answer | | |
|---|---|---|---|---|
| q504_M75b *(required)* | q504_M75b. How is the item administered, or given, to the patient?<br>*Question relevant when: selected( ${q104_M75_full_list} , '4')* | | 1 | Oral - solid (e.g. tablet) |
| | | | 2 | Oral - liquid |
| | | | 3 | Sublingual |
| | | | 4 | Intravenous (IV) |
| | | | 5 | Intramusculuar injection (IM) |
| | | | 6 | Subcutaneous |
| | | | 7 | Rectal |
| | | | 8 | Inhaled (e.g. gas) |
| | | | 9 | Inserted (e.g. vaginal pessaries) |
| | | | 10 | Topical - cream |
| | | | 11 | Topical - drops (e.g. eye drops) |
| | | | 12 | Other |
| | | | 99 | Don't know |
| q504_M75c *(required)* | q504_M75c. What is the item's smallest unit of measurement (mg, mcg, ml, g, etc)?<br>*Question relevant when: selected( ${q104_M75_full_list} , '4')* | | microgram | microgram (mcg) |
| | | | miligram | miligram (mg) |
| | | | miligram_dissolved_in_liquid | miligram (mg) dissolved in liquid (e.g. 2 mg per 3 militers) |
| | | | gram | gram (g) |
| | | | militer | militer (ml) |
| | | | centiliter | centiliter (cl) |
| | | | litre | litre (l) |
| | | | ml_of _solution | ml of solution (e.g. ml of 10% solution) |
| | | | litre_of_solution | litre of solution (e.g. 1 litre of 5% solution) |
| | | | cubic_meter | cubic meter (m^3) |
| | | | other | other |
| | | | dont_know | don't know |
| q504_M75c.1 | q504_M75c.1 If other unit of measurement or route of administration, specify here:<br>*Leave blank if not applicable*<br>*Question relevant when: selected( ${q104_M75_full_list} , '4')* | | | |
| q504_M75d *(required)* | q504_M75d. In total, how many units are used to treat a patient during all of her care?<br>*Remember to add all units! E.g. 5 days x 200 mg twice per day = 2,000mg*<br>*Question relevant when: selected( ${q104_M75_full_list} , '4')*<br>[illegible] | | | |

Response constrained to: .>0

| Field | Question | Answer |
|---|---|---|
| q504_M75.1 | ***q504_M75.1. Blood product - other 4: "[q104_M75.1_full_list_other]"***<br>*Question relevant when: selected( ${q104_M75.1_full_list} , '2')* | |
| q504_M75.1a *(required)* | q504_M75.1a. What percent of patients require this item?<br>*Question relevant when: selected( ${q104_M75.1_full_list} , '2')*<br>*Response constrained to: .>0 and .<=100 or .=999* | |
| q504_M75.1b *(required)* | q504_M75.1b. How is the item administered, or given, to the patient?<br>*Question relevant when: selected( ${q104_M75.1_full_list} , '2')* | 1 Oral - solid (e.g. tablet)<br>2 Oral - liquid<br>3 Sublingual<br>4 Intravenous (IV)<br>5 Intramusculuar injection (IM)<br>6 Subcutaneous<br>7 Rectal<br>8 Inhaled (e.g. gas)<br>9 Inserted (e.g. vaginal pessaries)<br>10 Topical - cream<br>11 Topical - drops (e.g. eye drops)<br>12 Other<br>99 Don't know |
| q504_M75.1c *(required)* | q504_M75.1c. What is the item's smallest unit of measurement (mg, mcg, ml, g, etc)?<br>*Question relevant when: selected( ${q104_M75.1_full_list} , '2')* | microgram — microgram (mcg)<br>miligram — miligram (mg)<br>miligram_dissolved_in_liquid — miligram (mg) dissolved in liquid (e.g. 2 mg per 3 militers)<br>gram — gram (g)<br>militer — militer (ml)<br>centiliter — centiliter (cl)<br>litre — litre (l)<br>ml_of _solution — ml of solution (e.g. ml of 10% solution)<br>litre_of_solution — litre of solution (e.g. 1 litre of 5% solution)<br>cubic_meter — cubic meter (m^3)<br>other — other<br>dont_know — don't know |
| q504_M75.1c.1 | q504_M75.1c.1 If other unit of measurement or route of administration, specify here:<br>*Leave blank if not applicable*<br>*Question relevant when: selected( ${q104_M75.1_full_list} , '2')* | |
| q504_M75.1d *(required)* | q504_M75.1d. In total, how many units are used to treat a patient during all of her care?<br>*Remember to add all units! E.g. 5 days x 200 mg twice per day = 2,000mg*<br>*Question relevant when: selected( ${q104_M75.1_full_list} , '2')*<br>*Response constrained to: .>0* | |

*Response constrained to: .>0*

| Field | Question | Answer |
|---|---|---|
| q504_M75.2 | ***q504_M75.2. Blood product - other 5: "[q104_M75.2_full_list_other]"***<br>*Question relevant when: selected( ${q104_M75.2_full_list} , '2')* | |
| q504_M75.2a *(required)* | q504_M75.2a. What percent of patients require this item?<br>*Question relevant when: selected( ${q104_M75.2_full_list} , '2')*<br>*Response constrained to: .>0 and .<=100 or .=999* | |
| q504_M75.2b *(required)* | q504_M75.2b. How is the item administered, or given, to the patient?<br>*Question relevant when: selected( ${q104_M75.2_full_list} , '2')* | 1 Oral - solid (e.g. tablet)<br>2 Oral - liquid<br>3 Sublingual<br>4 Intravenous (IV)<br>5 Intramusculuar injection (IM)<br>6 Subcutaneous<br>7 Rectal<br>8 Inhaled (e.g. gas)<br>9 Inserted (e.g. vaginal pessaries)<br>10 Topical - cream<br>11 Topical - drops (e.g. eye drops)<br>12 Other<br>99 Don't know |
| q504_M75.2c *(required)* | q504_M75.2c. What is the item's smallest unit of measurement (mg, mcg, ml, g, etc)?<br>*Question relevant when: selected( ${q104_M75.2_full_list} , '2')* | microgram — microgram (mcg)<br>miligram — miligram (mg)<br>miligram_dissolved_in_liquid — miligram (mg) dissolved in liquid (e.g. 2 mg per 3 militers)<br>gram — gram (g)<br>militer — militer (ml)<br>centiliter — centiliter (cl)<br>litre — litre (l)<br>ml_of _solution — ml of solution (e.g. ml of 10% solution)<br>litre_of_solution — litre of solution (e.g. 1 litre of 5% solution)<br>cubic_meter — cubic meter (m^3)<br>other — other<br>dont_know — don't know |
| q504_M75.2c.1 | q504_M75.2c.1 If other unit of measurement or route of administration, specify here:<br>*Leave blank if not applicable*<br>*Question relevant when: selected( ${q104_M75.2_full_list} , '2')* | |
| q504_M75.2d *(required)* | q504_M75.2d. In total, how many units are used to treat a patient during all of her care?<br>*Remember to add all units! E.g. 5 days x 200 mg twice per day = 2,000mg*<br>*Question relevant when: selected( ${q104_M75.2_full_list} , '2')*<br>*Response constrained to: .>0* | |

*Response constrained to: .>0*

<p><strong><em>D. Medications - Lacerations (4)</em></strong></p>

*Group relevant when: selected( ${section_five_skip_med} , '1')*

| Field | Question | Answer |
|---|---|---|
| q504_med_note8 | 504 CONTINUED For each of the following medications and other medical products that are used for lacerations, can you tell me …? | |
| note_504_hemostatic | ***Hemostatics*** | |
| q504_M76 | ***q504_M76. Dicynone / Dicynene (Etamsylate)***<br>*Question relevant when: selected( ${q104_M76_full_list} , '4')* | |
| q504_M76a *(required)* | q504_M76a. What percent of patients require this item?<br>*Question relevant when: selected( ${q104_M76_full_list} , '4')*<br>*Response constrained to: .>0 and .<=100 or .=999* | |
| q504_M76b *(required)* | q504_M76b. How is the item administered, or given, to the patient?<br>*Question relevant when: selected( ${q104_M76_full_list} , '4')* | 1 Oral - solid (e.g. tablet)<br>2 Oral - liquid<br>3 Sublingual<br>4 Intravenous (IV)<br>5 Intramusculuar injection (IM)<br>6 Subcutaneous<br>7 Rectal<br>8 Inhaled (e.g. gas)<br>9 Inserted (e.g. vaginal pessaries)<br>10 Topical - cream<br>11 Topical - drops (e.g. eye drops)<br>12 Other<br>99 Don't know |
| q504_M76c *(required)* | q504_M76c. What is the item's smallest unit of measurement (mg, mcg, ml, g, etc)?<br>*Question relevant when: selected( ${q104_M76_full_list} , '4')* | microgram — microgram (mcg)<br>miligram — miligram (mg)<br>miligram_dissolved_in_liquid — miligram (mg) dissolved in liquid (e.g. 2 mg per 3 militers)<br>gram — gram (g)<br>militer — militer (ml)<br>centiliter — centiliter (cl)<br>litre — litre (l)<br>ml_of _solution — ml of solution (e.g. ml of 10% solution)<br>litre_of_solution — litre of solution (e.g. 1 litre of 5% solution)<br>cubic_meter — cubic meter (m^3)<br>other — other<br>dont_know — don't know |
| q504_M76c.1 | q504_M76c.1 If other unit of measurement or route of administration, specify here:<br>*Leave blank if not applicable*<br>*Question relevant when: selected( ${q104_M76_full_list} , '4')* | |
| q504_M76d *(required)* | q504_M76d. In total, how many units are used to treat a patient during all of her care?<br>*Remember to add all units! E.g. 5 days x 200 mg twice per day = 2,000mg*<br>*Question relevant when: selected( ${q104_M76_full_list} , '4')*<br>*Response constrained to: .>0* | |

Response constrained to: .>0

| Field | Question | Answer |
|---|---|---|
| q504_M77 | ***q504_M77. Tranexamic acid (Kapron, Cyklokapron)***<br>*Question relevant when: selected( ${q104_M77_full_list} , '4')* | |
| q504_M77a *(required)* | q504_M77a. What percent of patients require this item?<br>*Question relevant when: selected( ${q104_M77_full_list} , '4')*<br>*Response constrained to: .>0 and .<=100 or .=999* | |

| | | |
|---|---|---|
| q504_M77b *(required)* | q504_M77b. How is the item administered, or given, to the patient?<br>*Question relevant when: selected( ${q104_M77_full_list} , '4')* | 1 Oral - solid (e.g. tablet)<br>2 Oral - liquid<br>3 Sublingual<br>4 Intravenous (IV)<br>5 Intramusculuar injection (IM)<br>6 Subcutaneous<br>7 Rectal<br>8 Inhaled (e.g. gas)<br>9 Inserted (e.g. vaginal pessaries)<br>10 Topical - cream<br>11 Topical - drops (e.g. eye drops)<br>12 Other<br>99 Don't know |
| q504_M77c *(required)* | q504_M77c. What is the item's smallest unit of measurement (mg, mcg, ml, g, etc)?<br>*Question relevant when: selected( ${q104_M77_full_list} , '4')* | microgram — microgram (mcg)<br>miligram — miligram (mg)<br>miligram_dissolved_in_liquid — miligram (mg) dissolved in liquid (e.g. 2 mg per 3 militers)<br>gram — gram (g)<br>militer — militer (ml)<br>centiliter — centiliter (cl)<br>litre — litre (l)<br>ml_of _solution — ml of solution (e.g. ml of 10% solution)<br>litre_of_solution — litre of solution (e.g. 1 litre of 5% solution)<br>cubic_meter — cubic meter (m^3)<br>other — other<br>dont_know — don't know |
| q504_M77c.1 | q504_M77c.1 If other unit of measurement or route of administration, specify here:<br>*Leave blank if not applicable*<br>*Question relevant when: selected( ${q104_M77_full_list} , '4')* | |
| q504_M77d *(required)* | q504_M77d. In total, how many units are used to treat a patient during all of her care?<br>*Remember to add all units! E.g. 5 days x 200 mg twice per day = 2,000mg*<br>*Question relevant when: selected( ${q104_M77_full_list} , '4')*<br>*Response constrained to: .>0* | |

*Response constrained to: .>0*

| Field | Question | Answer |
|---|---|---|
| q504_M78 | ***q504_M78. Polygeline 4%***<br>*Question relevant when: selected( ${q104_M78_full_list} , '4')* | |
| q504_M78a *(required)* | q504_M78a. What percent of patients require this item?<br>*Question relevant when: selected( ${q104_M78_full_list} , '4')*<br>*Response constrained to: .>0 and .<=100 or .=999* | |
| q504_M78b *(required)* | q504_M78b. How is the item administered, or given, to the patient?<br>*Question relevant when: selected( ${q104_M78_full_list} , '4')* | 1 Oral - solid (e.g. tablet)<br>2 Oral - liquid<br>3 Sublingual<br>4 Intravenous (IV)<br>5 Intramusculuar injection (IM)<br>6 Subcutaneous<br>7 Rectal<br>8 Inhaled (e.g. gas)<br>9 Inserted (e.g. vaginal pessaries)<br>10 Topical - cream<br>11 Topical - drops (e.g. eye drops)<br>12 Other<br>99 Don't know |
| q504_M78c *(required)* | q504_M78c. What is the item's smallest unit of measurement (mg, mcg, ml, g, etc)?<br>*Question relevant when: selected( ${q104_M78_full_list} , '4')* | microgram: microgram (mcg)<br>miligram: miligram (mg)<br>miligram_dissolved_in_liquid: miligram (mg) dissolved in liquid (e.g. 2 mg per 3 militers)<br>gram: gram (g)<br>militer: militer (ml)<br>centiliter: centiliter (cl)<br>litre: litre (l)<br>ml_of _solution: ml of solution (e.g. ml of 10% solution)<br>litre_of_solution: litre of solution (e.g. 1 litre of 5% solution)<br>cubic_meter: cubic meter (m^3)<br>other: other<br>dont_know: don't know |
| q504_M78c.1 | q504_M78c.1 If other unit of measurement or route of administration, specify here:<br>*Leave blank if not applicable*<br>*Question relevant when: selected( ${q104_M78_full_list} , '4')* | |
| q504_M78d *(required)* | q504_M78d. In total, how many units are used to treat a patient during all of her care?<br>*Remember to add all units! E.g. 5 days x 200 mg twice per day = 2,000mg*<br>*Question relevant when: selected( ${q104_M78_full_list} , '4')*<br>*Response constrained to: .>0* | |

*Response constrained to: .>0*

| Field | Question | Answer |
|---|---|---|
| q504_M79 | ***q504_M79. Hemostatic - other 1: "[q104_M79_full_list_other]"***<br>*Question relevant when: selected( ${q104_M79_full_list} , '4')* | |
| q504_M79a *(required)* | q504_M79a. What percent of patients require this item?<br>*Question relevant when: selected( ${q104_M79_full_list} , '4')*<br>*Response constrained to: .>0 and .<=100 or .=999* | |
| q504_M79b *(required)* | q504_M79b. How is the item administered, or given, to the patient?<br>*Question relevant when: selected( ${q104_M79_full_list} , '4')* | 1 Oral - solid (e.g. tablet)<br>2 Oral - liquid<br>3 Sublingual<br>4 Intravenous (IV)<br>5 Intramusculuar injection (IM)<br>6 Subcutaneous<br>7 Rectal<br>8 Inhaled (e.g. gas)<br>9 Inserted (e.g. vaginal pessaries)<br>10 Topical - cream<br>11 Topical - drops (e.g. eye drops)<br>12 Other<br>99 Don't know |
| q504_M79c *(required)* | q504_M79c. What is the item's smallest unit of measurement (mg, mcg, ml, g, etc)?<br>*Question relevant when: selected( ${q104_M79_full_list} , '4')* | microgram — microgram (mcg)<br>miligram — miligram (mg)<br>miligram_dissolved_in_liquid — miligram (mg) dissolved in liquid (e.g. 2 mg per 3 militers)<br>gram — gram (g)<br>militer — militer (ml)<br>centiliter — centiliter (cl)<br>litre — litre (l)<br>ml_of _solution — ml of solution (e.g. ml of 10% solution)<br>litre_of_solution — litre of solution (e.g. 1 litre of 5% solution)<br>cubic_meter — cubic meter (m^3)<br>other — other<br>dont_know — don't know |
| q504_M79c.1 | q504_M79c.1 If other unit of measurement or route of administration, specify here:<br>*Leave blank if not applicable*<br>*Question relevant when: selected( ${q104_M79_full_list} , '4')* | |
| q504_M79d *(required)* | q504_M79d. In total, how many units are used to treat a patient during all of her care?<br>*Remember to add all units! E.g. 5 days x 200 mg twice per day = 2,000mg*<br>*Question relevant when: selected( ${q104_M79_full_list} , '4')*<br>*Response constrained to: .>0* | |

Response constrained to: .>0

| Field | Question | Answer |
|---|---|---|
| q504_M80 | ***q504_M80. Hemostatic - other 2: "[q104_M80_full_list_other]"***<br>*Question relevant when: selected( ${q104_M80_full_list} , '4')* | |
| q504_M80a *(required)* | q504_M80a. What percent of patients require this item?<br>*Question relevant when: selected( ${q104_M80_full_list} , '4')*<br>*Response constrained to: .>0 and .<=100 or .=999* | |
| q504_M80b *(required)* | q504_M80b. How is the item administered, or given, to the patient?<br>*Question relevant when: selected( ${q104_M80_full_list} , '4')* | 1 Oral - solid (e.g. tablet)<br>2 Oral - liquid<br>3 Sublingual<br>4 Intravenous (IV)<br>5 Intramusculuar injection (IM)<br>6 Subcutaneous<br>7 Rectal<br>8 Inhaled (e.g. gas)<br>9 Inserted (e.g. vaginal pessaries)<br>10 Topical - cream<br>11 Topical - drops (e.g. eye drops)<br>12 Other<br>99 Don't know |
| q504_M80c *(required)* | q504_M80c. What is the item's smallest unit of measurement (mg, mcg, ml, g, etc)?<br>*Question relevant when: selected( ${q104_M80_full_list} , '4')* | microgram — microgram (mcg)<br>miligram — miligram (mg)<br>miligram_dissolved_in_liquid — miligram (mg) dissolved in liquid (e.g. 2 mg per 3 militers)<br>gram — gram (g)<br>militer — militer (ml)<br>centiliter — centiliter (cl)<br>litre — litre (l)<br>ml_of _solution — ml of solution (e.g. ml of 10% solution)<br>litre_of_solution — litre of solution (e.g. 1 litre of 5% solution)<br>cubic_meter — cubic meter (m^3)<br>other — other<br>dont_know — don't know |
| q504_M80c.1 | q504_M80c.1 If other unit of measurement or route of administration, specify here:<br>*Leave blank if not applicable*<br>*Question relevant when: selected( ${q104_M80_full_list} , '4')* | |
| q504_M80d *(required)* | q504_M80d. In total, how many units are used to treat a patient during all of her care?<br>*Remember to add all units! E.g. 5 days x 200 mg twice per day = 2,000mg*<br>*Question relevant when: selected( ${q104_M80_full_list} , '4')*<br>*Response constrained to: .>0* | |

*Response constrained to: .>0*

| Field | Question | Answer |
|---|---|---|
| q504_M81 | ***q504_M81. Hemostatic - other 3: "[q104_M81_full_list_other]"*** <br> *Question relevant when: selected( ${q104_M81_full_list} , '4')* | |
| q504_M81a *(required)* | q504_M81a. What percent of patients require this item? <br> *Question relevant when: selected( ${q104_M81_full_list} , '4')* <br> *Response constrained to: .>0 and .<=100 or .=999* | |
| q504_M81b *(required)* | q504_M81b. How is the item administered, or given, to the patient? <br> *Question relevant when: selected( ${q104_M81_full_list} , '4')* | 1 Oral - solid (e.g. tablet) <br> 2 Oral - liquid <br> 3 Sublingual <br> 4 Intravenous (IV) <br> 5 Intramusculuar injection (IM) <br> 6 Subcutaneous <br> 7 Rectal <br> 8 Inhaled (e.g. gas) <br> 9 Inserted (e.g. vaginal pessaries) <br> 10 Topical - cream <br> 11 Topical - drops (e.g. eye drops) <br> 12 Other <br> 99 Don't know |
| q504_M81c *(required)* | q504_M81c. What is the item's smallest unit of measurement (mg, mcg, ml, g, etc)? <br> *Question relevant when: selected( ${q104_M81_full_list} , '4')* | microgram: microgram (mcg) <br> miligram: miligram (mg) <br> miligram_dissolved_in_liquid: miligram (mg) dissolved in liquid (e.g. 2 mg per 3 militers) <br> gram: gram (g) <br> militer: militer (ml) <br> centiliter: centiliter (cl) <br> litre: litre (l) <br> ml_of _solution: ml of solution (e.g. ml of 10% solution) <br> litre_of_solution: litre of solution (e.g. 1 litre of 5% solution) <br> cubic_meter: cubic meter (m^3) <br> other: other <br> dont_know: don't know |
| q504_M81c.1 | q504_M81c.1 If other unit of measurement or route of administration, specify here: <br> *Leave blank if not applicable* <br> *Question relevant when: selected( ${q104_M81_full_list} , '4')* | |
| q504_M81d *(required)* | q504_M81d. In total, how many units are used to treat a patient during all of her care? <br> *Remember to add all units! E.g. 5 days x 200 mg twice per day = 2,000mg* <br> *Question relevant when: selected( ${q104_M81_full_list} , '4')* <br> *Response constrained to: .>0* | |

*Response constrained to: .>0*

| Field | Question | Answer |
|---|---|---|
| q504_M81.1 | ***q504_M81.1. Hemostatic - other 4: "[q104_M81.1_full_list_other]"***<br>*Question relevant when: selected( ${q104_M81.1_full_list} , '2')* | |
| q504_M81.1a *(required)* | q504_M81.1a. What percent of patients require this item?<br>*Question relevant when: selected( ${q104_M81.1_full_list} , '2')*<br>*Response constrained to: .>0 and .<=100 or .=999* | |
| q504_M81.1b *(required)* | q504_M81.1b. How is the item administered, or given, to the patient?<br>*Question relevant when: selected( ${q104_M81.1_full_list} , '2')* | 1 Oral - solid (e.g. tablet)<br>2 Oral - liquid<br>3 Sublingual<br>4 Intravenous (IV)<br>5 Intramusculuar injection (IM)<br>6 Subcutaneous<br>7 Rectal<br>8 Inhaled (e.g. gas)<br>9 Inserted (e.g. vaginal pessaries)<br>10 Topical - cream<br>11 Topical - drops (e.g. eye drops)<br>12 Other<br>99 Don't know |
| q504_M81.1c *(required)* | q504_M81.1c. What is the item's smallest unit of measurement (mg, mcg, ml, g, etc)?<br>*Question relevant when: selected( ${q104_M81.1_full_list} , '2')* | microgram — microgram (mcg)<br>miligram — miligram (mg)<br>miligram_dissolved_in_liquid — miligram (mg) dissolved in liquid (e.g. 2 mg per 3 militers)<br>gram — gram (g)<br>militer — militer (ml)<br>centiliter — centiliter (cl)<br>litre — litre (l)<br>ml_of _solution — ml of solution (e.g. ml of 10% solution)<br>litre_of_solution — litre of solution (e.g. 1 litre of 5% solution)<br>cubic_meter — cubic meter (m^3)<br>other — other<br>dont_know — don't know |
| q504_M81.1c.1 | q504_M81.1c.1 If other unit of measurement or route of administration, specify here:<br>*Leave blank if not applicable*<br>*Question relevant when: selected( ${q104_M81.1_full_list} , '2')* | |
| q504_M81.1d *(required)* | q504_M81.1d. In total, how many units are used to treat a patient during all of her care?<br>*Remember to add all units! E.g. 5 days x 200 mg twice per day = 2,000mg*<br>*Question relevant when: selected( ${q104_M81.1_full_list} , '2')*<br>*Response constrained to: .>0* | |

*Response constrained to: .>0*

| Field | Question | Answer |
|---|---|---|
| q504_M81.2 | ***q504_M81.2. Hemostatic - other 5: "[q104_M81.2_full_list_other]"***<br>*Question relevant when: selected( ${q104_M81.2_full_list} , '2')* | |
| q504_M81.2a *(required)* | q504_M81.2a. What percent of patients require this item?<br>*Question relevant when: selected( ${q104_M81.2_full_list} , '2')*<br>*Response constrained to: .>0 and .<=100 or .=999* | |
| q504_M81.2b *(required)* | q504_M81.2b. How is the item administered, or given, to the patient?<br>*Question relevant when: selected( ${q104_M81.2_full_list} , '2')* | 1 Oral - solid (e.g. tablet)<br>2 Oral - liquid<br>3 Sublingual<br>4 Intravenous (IV)<br>5 Intramusculuar injection (IM)<br>6 Subcutaneous<br>7 Rectal<br>8 Inhaled (e.g. gas)<br>9 Inserted (e.g. vaginal pessaries)<br>10 Topical - cream<br>11 Topical - drops (e.g. eye drops)<br>12 Other<br>99 Don't know |
| q504_M81.2c *(required)* | q504_M81.2c. What is the item's smallest unit of measurement (mg, mcg, ml, g, etc)?<br>*Question relevant when: selected( ${q104_M81.2_full_list} , '2')* | microgram — microgram (mcg)<br>miligram — miligram (mg)<br>miligram_dissolved_in_liquid — miligram (mg) dissolved in liquid (e.g. 2 mg per 3 militers)<br>gram — gram (g)<br>militer — militer (ml)<br>centiliter — centiliter (cl)<br>litre — litre (l)<br>ml_of _solution — ml of solution (e.g. ml of 10% solution)<br>litre_of_solution — litre of solution (e.g. 1 litre of 5% solution)<br>cubic_meter — cubic meter (m^3)<br>other — other<br>dont_know — don't know |
| q504_M81.2c.1 | q504_M81.2c.1 If other unit of measurement or route of administration, specify here:<br>*Leave blank if not applicable*<br>*Question relevant when: selected( ${q104_M81.2_full_list} , '2')* | |
| q504_M81.2d *(required)* | q504_M81.2d. In total, how many units are used to treat a patient during all of her care?<br>*Remember to add all units! E.g. 5 days x 200 mg twice per day = 2,000mg*<br>*Question relevant when: selected( ${q104_M81.2_full_list} , '2')* | |

| Field | Question | Answer |
|---|---|---|
| | *Question relevant when: selected( [illegible]*<br>*Response constrained to: .>0* | |
| <p><strong><em>D. Medications - Lacerations (9)</em></strong></p><br>*Group relevant when: selected( ${section_five_skip_med} , '1')* | | |
| q504_med_notE8 | 504 CONTINUED For each of the following medications and other medical products that are used for lacerations, can you tell me …? | |
| note_504_saline | ***Saline, glucose, water, etc*** | |
| q504_M82 | ***q504_M82.IV solution, dextrose 2.5% in NaCl 0.45%***<br>*Question relevant when: selected( ${q104_M82_full_list} , '4')* | |
| q504_M82a *(required)* | q504_M82a. What percent of patients require this item?<br>*Question relevant when: selected( ${q104_M82_full_list} , '4')*<br>*Response constrained to: .>0 and .<=100 or .=999* | |
| q504_M82b *(required)* | q504_M82b. How is the item administered, or given, to the patient?<br>*Question relevant when: selected( ${q104_M82_full_list} , '4')* | 1 Oral - solid (e.g. tablet)<br>2 Oral - liquid<br>3 Sublingual<br>4 Intravenous (IV)<br>5 Intramusculuar injection (IM)<br>6 Subcutaneous<br>7 Rectal<br>8 Inhaled (e.g. gas)<br>9 Inserted (e.g. vaginal pessaries)<br>10 Topical - cream<br>11 Topical - drops (e.g. eye drops)<br>12 Other<br>99 Don't know |
| q504_M82c *(required)* | q504_M82c. What is the item's smallest unit of measurement (mg, mcg, ml, g, etc)?<br>*Question relevant when: selected( ${q104_M82_full_list} , '4')* | microgram — microgram (mcg)<br>miligram — miligram (mg)<br>miligram_dissolved_in_liquid — miligram (mg) dissolved in liquid (e.g. 2 mg per 3 militers)<br>gram — gram (g)<br>militer — militer (ml)<br>centiliter — centiliter (cl)<br>litre — litre (l)<br>ml_of _solution — ml of solution (e.g. ml of 10% solution)<br>litre_of_solution — litre of solution (e.g. 1 litre of 5% solution)<br>cubic_meter — cubic meter (m^3)<br>other — other<br>dont_know — don't know |
| q504_M82c.1 | q504_M82c.1 If other unit of measurement or route of administration, specify here:<br>*Leave blank if not applicable*<br>*Question relevant when: selected( ${q104_M82_full_list} , '4')* | |
| q504_M82d *(required)* | q504_M82d. In total, how many units are used to treat a patient during all of her care?<br>*Remember to add all units! E.g. 5 days x 200 mg twice per day = 2,000mg*<br>*Question relevant when: selected( ${q104_M82_full_list} , '4')* | |

Question relevant when: selected( ${q104_M82_full_list} , '4')

Response constrained to: .>0

| Field | Question | Answer |
|---|---|---|
| q504_M83 | ***q504_M83.IV solution, glucose (5%), isotonic***<br>*Question relevant when: selected( ${q104_M83_full_list} , '4')* | |
| q504_M83a *(required)* | q504_M83a. What percent of patients require this item?<br>*Question relevant when: selected( ${q104_M83_full_list} , '4')*<br>*Response constrained to: .>0 and .<=100 or .=999* | |
| q504_M83b *(required)* | q504_M83b. How is the item administered, or given, to the patient?<br>*Question relevant when: selected( ${q104_M83_full_list} , '4')* | 1 Oral - solid (e.g. tablet)<br>2 Oral - liquid<br>3 Sublingual<br>4 Intravenous (IV)<br>5 Intramusculuar injection (IM)<br>6 Subcutaneous<br>7 Rectal<br>8 Inhaled (e.g. gas)<br>9 Inserted (e.g. vaginal pessaries)<br>10 Topical - cream<br>11 Topical - drops (e.g. eye drops)<br>12 Other<br>99 Don't know |
| q504_M83c *(required)* | q504_M83c. What is the item's smallest unit of measurement (mg, mcg, ml, g, etc)?<br>*Question relevant when: selected( ${q104_M83_full_list} , '4')* | microgram — microgram (mcg)<br>miligram — miligram (mg)<br>miligram_dissolved_in_liquid — miligram (mg) dissolved in liquid (e.g. 2 mg per 3 militers)<br>gram — gram (g)<br>militer — militer (ml)<br>centiliter — centiliter (cl)<br>litre — litre (l)<br>ml_of _solution — ml of solution (e.g. ml of 10% solution)<br>litre_of_solution — litre of solution (e.g. 1 litre of 5% solution)<br>cubic_meter — cubic meter (m^3)<br>other — other<br>dont_know — don't know |
| q504_M83c.1 | q504_M83c.1 If other unit of measurement or route of administration, specify here:<br>*Leave blank if not applicable*<br>*Question relevant when: selected( ${q104_M83_full_list} , '4')* | |
| q504_M83d *(required)* | q504_M83d. In total, how many units are used to treat a patient during all of her care?<br>*Remember to add all units! E.g. 5 days x 200 mg twice per day = 2,000mg*<br>*Question relevant when: selected( ${q104_M83_full_list} , '4')* | |

Question relevant when: selected( ${q104_[illegible]_full_list} , '4')

Response constrained to: .>0

| Field | Question | Answer |
|---|---|---|
| q504_M84 | ***q504_M84. Ringers lactate (prepared onsite)***<br>*Question relevant when: selected( ${q104_M84_full_list} , '4')* | |
| q504_M84a *(required)* | q504_M84a. What percent of patients require this item?<br>*Question relevant when: selected( ${q104_M84_full_list} , '4')*<br>*Response constrained to: .>0 and .<=100 or .=999* | |
| q504_M84b *(required)* | q504_M84b. How is the item administered, or given, to the patient?<br>*Question relevant when: selected( ${q104_M84_full_list} , '4')* | 1 Oral - solid (e.g. tablet)<br>2 Oral - liquid<br>3 Sublingual<br>4 Intravenous (IV)<br>5 Intramusculuar injection (IM)<br>6 Subcutaneous<br>7 Rectal<br>8 Inhaled (e.g. gas)<br>9 Inserted (e.g. vaginal pessaries)<br>10 Topical - cream<br>11 Topical - drops (e.g. eye drops)<br>12 Other<br>99 Don't know |
| q504_M84c *(required)* | q504_M84c. What is the item's smallest unit of measurement (mg, mcg, ml, g, etc)?<br>*Question relevant when: selected( ${q104_M84_full_list} , '4')* | microgram — microgram (mcg)<br>miligram — miligram (mg)<br>miligram_dissolved_in_liquid — miligram (mg) dissolved in liquid (e.g. 2 mg per 3 militers)<br>gram — gram (g)<br>militer — militer (ml)<br>centiliter — centiliter (cl)<br>litre — litre (l)<br>ml_of _solution — ml of solution (e.g. ml of 10% solution)<br>litre_of_solution — litre of solution (e.g. 1 litre of 5% solution)<br>cubic_meter — cubic meter (m^3)<br>other — other<br>dont_know — don't know |
| q504_M84c.1 | q504_M84c.1 If other unit of measurement or route of administration, specify here:<br>*Leave blank if not applicable*<br>*Question relevant when: selected( ${q104_M84_full_list} , '4')* | |
| q504_M84d *(required)* | q504_M84d. In total, how many units are used to treat a patient during all of her care?<br>*Remember to add all units! E.g. 5 days x 200 mg twice per day = 2,000mg*<br>*Question relevant when: selected( ${q104_M84_full_list} , '4')* | |

Question relevant when: selected( ${q104_M84_full_list} , '4')

Response constrained to: .>0

| Field | Question | Answer |
|---|---|---|
| q504_M85 | ***q504_M85. Ringers lactate (bought from pharmacy/MSD)***<br>*Question relevant when: selected( ${q104_M85_full_list} , '4')* | |
| q504_M85a *(required)* | q504_M85a. What percent of patients require this item?<br>*Question relevant when: selected( ${q104_M85_full_list} , '4')*<br>*Response constrained to: .>0 and .<=100 or .=999* | |
| q504_M85b *(required)* | q504_M85b. How is the item administered, or given, to the patient?<br>*Question relevant when: selected( ${q104_M85_full_list} , '4')* | 1 Oral - solid (e.g. tablet)<br>2 Oral - liquid<br>3 Sublingual<br>4 Intravenous (IV)<br>5 Intramusculuar injection (IM)<br>6 Subcutaneous<br>7 Rectal<br>8 Inhaled (e.g. gas)<br>9 Inserted (e.g. vaginal pessaries)<br>10 Topical - cream<br>11 Topical - drops (e.g. eye drops)<br>12 Other<br>99 Don't know |
| q504_M85c *(required)* | q504_M85c. What is the item's smallest unit of measurement (mg, mcg, ml, g, etc)?<br>*Question relevant when: selected( ${q104_M85_full_list} , '4')* | microgram — microgram (mcg)<br>miligram — miligram (mg)<br>miligram_dissolved_in_liquid — miligram (mg) dissolved in liquid (e.g. 2 mg per 3 militers)<br>gram — gram (g)<br>militer — militer (ml)<br>centiliter — centiliter (cl)<br>litre — litre (l)<br>ml_of _solution — ml of solution (e.g. ml of 10% solution)<br>litre_of_solution — litre of solution (e.g. 1 litre of 5% solution)<br>cubic_meter — cubic meter (m^3)<br>other — other<br>dont_know — don't know |
| q504_M85c.1 | q504_M85c.1 If other unit of measurement or route of administration, specify here:<br>*Leave blank if not applicable*<br>*Question relevant when: selected( ${q104_M85_full_list} , '4')* | |
| q504_M85d *(required)* | q504_M85d. In total, how many units are used to treat a patient during all of her care?<br>*Remember to add all units! E.g. 5 days x 200 mg twice per day = 2,000mg*<br>*Question relevant when: selected( ${q104_M85_full_list} , '4')* | |

Question relevant when: selected( ${q104_M[illegible]_full_list} , '4')

*Response constrained to: .>0*

| Field | Question | Answer |
|---|---|---|
| q504_M86 | ***q504_M86. Saline, 0.9% (Normal Saline) (prepared onsite)***<br>*Question relevant when: selected( ${q104_M86_full_list} , '4')* | |
| q504_M86a *(required)* | q504_M86a. What percent of patients require this item?<br>*Question relevant when: selected( ${q104_M86_full_list} , '4')*<br>*Response constrained to: .>0 and .<=100 or .=999* | |
| q504_M86b *(required)* | q504_M86b. How is the item administered, or given, to the patient?<br>*Question relevant when: selected( ${q104_M86_full_list} , '4')* | 1 Oral - solid (e.g. tablet)<br>2 Oral - liquid<br>3 Sublingual<br>4 Intravenous (IV)<br>5 Intramusculuar injection (IM)<br>6 Subcutaneous<br>7 Rectal<br>8 Inhaled (e.g. gas)<br>9 Inserted (e.g. vaginal pessaries)<br>10 Topical - cream<br>11 Topical - drops (e.g. eye drops)<br>12 Other<br>99 Don't know |
| q504_M86c *(required)* | q504_M86c. What is the item's smallest unit of measurement (mg, mcg, ml, g, etc)?<br>*Question relevant when: selected( ${q104_M86_full_list} , '4')* | microgram — microgram (mcg)<br>miligram — miligram (mg)<br>miligram_dissolved_in_liquid — miligram (mg) dissolved in liquid (e.g. 2 mg per 3 militers)<br>gram — gram (g)<br>militer — militer (ml)<br>centiliter — centiliter (cl)<br>litre — litre (l)<br>ml_of _solution — ml of solution (e.g. ml of 10% solution)<br>litre_of_solution — litre of solution (e.g. 1 litre of 5% solution)<br>cubic_meter — cubic meter (m^3)<br>other — other<br>dont_know — don't know |
| q504_M86c.1 | q504_M86c.1 If other unit of measurement or route of administration, specify here:<br>*Leave blank if not applicable*<br>*Question relevant when: selected( ${q104_M86_full_list} , '4')* | |
| q504_M86d *(required)* | q504_M86d. In total, how many units are used to treat a patient during all of her care?<br>*Remember to add all units! E.g. 5 days x 200 mg twice per day = 2,000mg*<br>*Question relevant when: selected( ${q104_M86_full_list} , '4')* | |

[illegible]

*Response constrained to: .>0*

| Field | Question | Answer |
|---|---|---|
| q504_M87 | ***q504_M87. Saline, 0.9% (Normal Saline) (bought from pharmacy/MSD)***<br>*Question relevant when: selected( ${q104_M87_full_list} , '4')* | |
| q504_M87a *(required)* | q504_M87a. What percent of patients require this item?<br>*Question relevant when: selected( ${q104_M87_full_list} , '4')*<br>*Response constrained to: .>0 and .<=100 or .=999* | |
| q504_M87b *(required)* | q504_M87b. How is the item administered, or given, to the patient?<br>*Question relevant when: selected( ${q104_M87_full_list} , '4')* | 1 Oral - solid (e.g. tablet)<br>2 Oral - liquid<br>3 Sublingual<br>4 Intravenous (IV)<br>5 Intramusculuar injection (IM)<br>6 Subcutaneous<br>7 Rectal<br>8 Inhaled (e.g. gas)<br>9 Inserted (e.g. vaginal pessaries)<br>10 Topical - cream<br>11 Topical - drops (e.g. eye drops)<br>12 Other<br>99 Don't know |
| q504_M87c *(required)* | q504_M87c. What is the item's smallest unit of measurement (mg, mcg, ml, g, etc)?<br>*Question relevant when: selected( ${q104_M87_full_list} , '4')* | microgram — microgram (mcg)<br>miligram — miligram (mg)<br>miligram_dissolved_in_liquid — miligram (mg) dissolved in liquid (e.g. 2 mg per 3 militers)<br>gram — gram (g)<br>militer — militer (ml)<br>centiliter — centiliter (cl)<br>litre — litre (l)<br>ml_of _solution — ml of solution (e.g. ml of 10% solution)<br>litre_of_solution — litre of solution (e.g. 1 litre of 5% solution)<br>cubic_meter — cubic meter (m^3)<br>other — other<br>dont_know — don't know |
| q504_M87c.1 | q504_M87c.1 If other unit of measurement or route of administration, specify here:<br>*Leave blank if not applicable*<br>*Question relevant when: selected( ${q104_M87_full_list} , '4')* | |
| q504_M87d *(required)* | q504_M87d. In total, how many units are used to treat a patient during all of her care?<br>*Remember to add all units! E.g. 5 days x 200 mg twice per day = 2,000mg*<br>*Question relevant when: selected( ${q104_M87_full_list} , '4')* | |

| Field | Question | Answer |
|---|---|---|
| | *Question relevant when: selected( ${q104_M87_full_list} , '4')*<br>*Response constrained to: .>0* | |
| q504_M88 | ***q504_M88. Solution for Infusion, glucose 5% + NaCl 0.9% (prepared onsite)***<br>*Question relevant when: selected( ${q104_M88_full_list} , '4')* | |
| q504_M88a *(required)* | q504_M88a. What percent of patients require this item?<br>*Question relevant when: selected( ${q104_M88_full_list} , '4')*<br>*Response constrained to: .>0 and .<=100 or .=999* | |
| q504_M88b *(required)* | q504_M88b. How is the item administered, or given, to the patient?<br>*Question relevant when: selected( ${q104_M88_full_list} , '4')* | 1 Oral - solid (e.g. tablet)<br>2 Oral - liquid<br>3 Sublingual<br>4 Intravenous (IV)<br>5 Intramusculuar injection (IM)<br>6 Subcutaneous<br>7 Rectal<br>8 Inhaled (e.g. gas)<br>9 Inserted (e.g. vaginal pessaries)<br>10 Topical - cream<br>11 Topical - drops (e.g. eye drops)<br>12 Other<br>99 Don't know |
| q504_M88c *(required)* | q504_M88c. What is the item's smallest unit of measurement (mg, mcg, ml, g, etc)?<br>*Question relevant when: selected( ${q104_M88_full_list} , '4')* | microgram — microgram (mcg)<br>miligram — miligram (mg)<br>miligram_dissolved_in_liquid — miligram (mg) dissolved in liquid (e.g. 2 mg per 3 militers)<br>gram — gram (g)<br>militer — militer (ml)<br>centiliter — centiliter (cl)<br>litre — litre (l)<br>ml_of _solution — ml of solution (e.g. ml of 10% solution)<br>litre_of_solution — litre of solution (e.g. 1 litre of 5% solution)<br>cubic_meter — cubic meter (m^3)<br>other — other<br>dont_know — don't know |
| q504_M88c.1 | q504_M88c.1 If other unit of measurement or route of administration, specify here:<br>*Leave blank if not applicable*<br>*Question relevant when: selected( ${q104_M88_full_list} , '4')* | |
| q504_M88d *(required)* | q504_M88d. In total, how many units are used to treat a patient during all of her care?<br>*Remember to add all units! E.g. 5 days x 200 mg twice per day = 2,000mg*<br>*Question relevant when: selected( ${q104_M88_full_list} , '4')* | |

Question relevant when: selected( ${q104_[illegible]_full_list} , '4')

*Response constrained to: .>0*

| Field | Question | Answer |
|---|---|---|
| q504_M89 | ***q504_M89. Solution for Infusion, glucose 5% + NaCl 0.9% (bought from pharmacy or MSD)***<br>*Question relevant when: selected( ${q104_M89_full_list} , '4')* | |
| q504_M89a *(required)* | q504_M89a. What percent of patients require this item?<br>*Question relevant when: selected( ${q104_M89_full_list} , '4')*<br>*Response constrained to: .>0 and .<=100 or .=999* | |
| q504_M89b *(required)* | q504_M89b. How is the item administered, or given, to the patient?<br>*Question relevant when: selected( ${q104_M89_full_list} , '4')* | 1 Oral - solid (e.g. tablet)<br>2 Oral - liquid<br>3 Sublingual<br>4 Intravenous (IV)<br>5 Intramusculuar injection (IM)<br>6 Subcutaneous<br>7 Rectal<br>8 Inhaled (e.g. gas)<br>9 Inserted (e.g. vaginal pessaries)<br>10 Topical - cream<br>11 Topical - drops (e.g. eye drops)<br>12 Other<br>99 Don't know |
| q504_M89c *(required)* | q504_M89c. What is the item's smallest unit of measurement (mg, mcg, ml, g, etc)?<br>*Question relevant when: selected( ${q104_M89_full_list} , '4')* | microgram — microgram (mcg)<br>miligram — miligram (mg)<br>miligram_dissolved_in_liquid — miligram (mg) dissolved in liquid (e.g. 2 mg per 3 militers)<br>gram — gram (g)<br>militer — militer (ml)<br>centiliter — centiliter (cl)<br>litre — litre (l)<br>ml_of _solution — ml of solution (e.g. ml of 10% solution)<br>litre_of_solution — litre of solution (e.g. 1 litre of 5% solution)<br>cubic_meter — cubic meter (m^3)<br>other — other<br>dont_know — don't know |
| q504_M89c.1 | q504_M89c.1 If other unit of measurement or route of administration, specify here:<br>*Leave blank if not applicable*<br>*Question relevant when: selected( ${q104_M89_full_list} , '4')* | |
| q504_M89d *(required)* | q504_M89d. In total, how many units are used to treat a patient during all of her care?<br>*Remember to add all units! E.g. 5 days x 200 mg twice per day = 2,000mg*<br>*Question relevant when: selected( ${q104_M89_full_list} , '4')* | |

*Response constrained to: .>0*

| Field | Question | Answer |
|---|---|---|
| q504_M90 | ***q504_M90. Water for injection***<br>*Question relevant when: selected( ${q104_M90_full_list} , '4')* | |
| q504_M90a *(required)* | q504_M90a. What percent of patients require this item?<br>*Question relevant when: selected( ${q104_M90_full_list} , '4')*<br>*Response constrained to: .>0 and .<=100 or .=999* | |
| q504_M90b *(required)* | q504_M90b. How is the item administered, or given, to the patient?<br>*Question relevant when: selected( ${q104_M90_full_list} , '4')* | 1 Oral - solid (e.g. tablet)<br>2 Oral - liquid<br>3 Sublingual<br>4 Intravenous (IV)<br>5 Intramusculuar injection (IM)<br>6 Subcutaneous<br>7 Rectal<br>8 Inhaled (e.g. gas)<br>9 Inserted (e.g. vaginal pessaries)<br>10 Topical - cream<br>11 Topical - drops (e.g. eye drops)<br>12 Other<br>99 Don't know |
| q504_M90c *(required)* | q504_M90c. What is the item's smallest unit of measurement (mg, mcg, ml, g, etc)?<br>*Question relevant when: selected( ${q104_M90_full_list} , '4')* | microgram — microgram (mcg)<br>miligram — miligram (mg)<br>miligram_dissolved_in_liquid — miligram (mg) dissolved in liquid (e.g. 2 mg per 3 militers)<br>gram — gram (g)<br>militer — militer (ml)<br>centiliter — centiliter (cl)<br>litre — litre (l)<br>ml_of _solution — ml of solution (e.g. ml of 10% solution)<br>litre_of_solution — litre of solution (e.g. 1 litre of 5% solution)<br>cubic_meter — cubic meter (m^3)<br>other — other<br>dont_know — don't know |
| q504_M90c.1 | q504_M90c.1 If other unit of measurement or route of administration, specify here:<br>*Leave blank if not applicable*<br>*Question relevant when: selected( ${q104_M90_full_list} , '4')* | |
| q504_M90d *(required)* | q504_M90d. In total, how many units are used to treat a patient during all of her care?<br>*Remember to add all units! E.g. 5 days x 200 mg twice per day = 2,000mg*<br>*Question relevant when: selected( ${q104_M90_full_list} , '4')* | |

*Question relevant when: selected( ${q104_M90_full_list} , '4')*

*Response constrained to: .>0*

| Field | Question | Answer |
|---|---|---|
| q504_M91 | ***q504_M91. Saline, glucose, water, etc. - Other 1: "[q104_M91_full_list_other]"***<br>*Question relevant when: selected( ${q104_M91_full_list} , '4')* | |
| q504_M91a *(required)* | q504_M91a. What percent of patients require this item?<br>*Question relevant when: selected( ${q104_M91_full_list} , '4')*<br>*Response constrained to: .>0 and .<=100 or .=999* | |
| q504_M91b *(required)* | q504_M91b. How is the item administered, or given, to the patient?<br>*Question relevant when: selected( ${q104_M91_full_list} , '4')* | 1 Oral - solid (e.g. tablet)<br>2 Oral - liquid<br>3 Sublingual<br>4 Intravenous (IV)<br>5 Intramusculuar injection (IM)<br>6 Subcutaneous<br>7 Rectal<br>8 Inhaled (e.g. gas)<br>9 Inserted (e.g. vaginal pessaries)<br>10 Topical - cream<br>11 Topical - drops (e.g. eye drops)<br>12 Other<br>99 Don't know |
| q504_M91c *(required)* | q504_M91c. What is the item's smallest unit of measurement (mg, mcg, ml, g, etc)?<br>*Question relevant when: selected( ${q104_M91_full_list} , '4')* | microgram: microgram (mcg)<br>miligram: miligram (mg)<br>miligram_dissolved_in_liquid: miligram (mg) dissolved in liquid (e.g. 2 mg per 3 militers)<br>gram: gram (g)<br>militer: militer (ml)<br>centiliter: centiliter (cl)<br>litre: litre (l)<br>ml_of _solution: ml of solution (e.g. ml of 10% solution)<br>litre_of_solution: litre of solution (e.g. 1 litre of 5% solution)<br>cubic_meter: cubic meter (m^3)<br>other: other<br>dont_know: don't know |
| q504_M91c.1 | q504_M91c.1 If other unit of measurement or route of administration, specify here:<br>*Leave blank if not applicable*<br>*Question relevant when: selected( ${q104_M91_full_list} , '4')* | |
| q504_M91d *(required)* | q504_M91d. In total, how many units are used to treat a patient during all of her care?<br>*Remember to add all units! E.g. 5 days x 200 mg twice per day = 2,000mg*<br>*Question relevant when: selected( ${q104_M91_full_list} , '4')* | |

| Field | Question | Answer |
| --- | --- | --- |
| | *Question relevant when: selected( ${q104_M[illegible]_full_list} , '4')*<br>*Response constrained to: .>0* | |
| q504_M92 | ***q504_M92. Saline, glucose, water, etc. - Other 2: "[q104_M92_full_list_other]"***<br>*Question relevant when: selected( ${q104_M92_full_list} , '4')* | |
| q504_M92a *(required)* | q504_M92a. What percent of patients require this item?<br>*Question relevant when: selected( ${q104_M92_full_list} , '4')*<br>*Response constrained to: .>0 and .<=100 or .=999* | |
| q504_M92b *(required)* | q504_M92b. How is the item administered, or given, to the patient?<br>*Question relevant when: selected( ${q104_M92_full_list} , '4')* | 1 Oral - solid (e.g. tablet)<br>2 Oral - liquid<br>3 Sublingual<br>4 Intravenous (IV)<br>5 Intramusculuar injection (IM)<br>6 Subcutaneous<br>7 Rectal<br>8 Inhaled (e.g. gas)<br>9 Inserted (e.g. vaginal pessaries)<br>10 Topical - cream<br>11 Topical - drops (e.g. eye drops)<br>12 Other<br>99 Don't know |
| q504_M92c *(required)* | q504_M92c. What is the item's smallest unit of measurement (mg, mcg, ml, g, etc)?<br>*Question relevant when: selected( ${q104_M92_full_list} , '4')* | microgram — microgram (mcg)<br>miligram — miligram (mg)<br>miligram_dissolved_in_liquid — miligram (mg) dissolved in liquid (e.g. 2 mg per 3 militers)<br>gram — gram (g)<br>militer — militer (ml)<br>centiliter — centiliter (cl)<br>litre — litre (l)<br>ml_of _solution — ml of solution (e.g. ml of 10% solution)<br>litre_of_solution — litre of solution (e.g. 1 litre of 5% solution)<br>cubic_meter — cubic meter (m^3)<br>other — other<br>dont_know — don't know |
| q504_M92c.1 | q504_M92c.1 If other unit of measurement or route of administration, specify here:<br>*Leave blank if not applicable*<br>*Question relevant when: selected( ${q104_M92_full_list} , '4')* | |
| q504_M92d *(required)* | q504_M92d. In total, how many units are used to treat a patient during all of her care?<br>*Remember to add all units! E.g. 5 days x 200 mg twice per day = 2,000mg*<br>*Question relevant when: selected( ${q104_M92_full_list} , '4')* | |

Question relevant when: selected( ${q104_M92_full_list} , '4')

Response constrained to: .>0

| Field | Question | Answer |
|---|---|---|
| q504_M93 | ***q504_M93. Saline, glucose, water, etc. - Other 3: "[q104_M93_full_list_other]"***<br>*Question relevant when: selected( ${q104_M93_full_list} , '4')* | |
| q504_M93a *(required)* | q504_M93a. What percent of patients require this item?<br>*Question relevant when: selected( ${q104_M93_full_list} , '4')*<br>*Response constrained to: .>0 and .<=100 or .=999* | |
| q504_M93b *(required)* | q504_M93b. How is the item administered, or given, to the patient?<br>*Question relevant when: selected( ${q104_M93_full_list} , '4')* | 1 Oral - solid (e.g. tablet)<br>2 Oral - liquid<br>3 Sublingual<br>4 Intravenous (IV)<br>5 Intramusculuar injection (IM)<br>6 Subcutaneous<br>7 Rectal<br>8 Inhaled (e.g. gas)<br>9 Inserted (e.g. vaginal pessaries)<br>10 Topical - cream<br>11 Topical - drops (e.g. eye drops)<br>12 Other<br>99 Don't know |
| q504_M93c *(required)* | q504_M93c. What is the item's smallest unit of measurement (mg, mcg, ml, g, etc)?<br>*Question relevant when: selected( ${q104_M93_full_list} , '4')* | microgram — microgram (mcg)<br>miligram — miligram (mg)<br>miligram_dissolved_in_liquid — miligram (mg) dissolved in liquid (e.g. 2 mg per 3 militers)<br>gram — gram (g)<br>militer — militer (ml)<br>centiliter — centiliter (cl)<br>litre — litre (l)<br>ml_of _solution — ml of solution (e.g. ml of 10% solution)<br>litre_of_solution — litre of solution (e.g. 1 litre of 5% solution)<br>cubic_meter — cubic meter (m^3)<br>other — other<br>dont_know — don't know |
| q504_M93c.1 | q504_M93c.1 If other unit of measurement or route of administration, specify here:<br>*Leave blank if not applicable*<br>*Question relevant when: selected( ${q104_M93_full_list} , '4')* | |
| q504_M93d *(required)* | q504_M93d. In total, how many units are used to treat a patient during all of her care?<br>*Remember to add all units! E.g. 5 days x 200 mg twice per day = 2,000mg*<br>*Question relevant when: selected( ${q104_M93_full_list} , '4')* | |

Question relevant when: selected( ${q104_M93_full_list} , '4')

Response constrained to: .>0

| Field | Question | Answer |
|---|---|---|
| q504_M94 | ***q504_M94. Saline, glucose, water, etc. - Other 4: "[q104_M94_full_list_other]"*** <br> *Question relevant when: selected( ${q104_M94_full_list} , '4')* | |
| q504_M94a *(required)* | q504_M94a. What percent of patients require this item? <br> *Question relevant when: selected( ${q104_M94_full_list} , '4')* <br> *Response constrained to: .>0 and .<=100 or .=999* | |

| Field | Question | Answer | | |
|---|---|---|---|---|
| q504_M94b *(required)* | q504_M94b. How is the item administered, or given, to the patient? <br> *Question relevant when: selected( ${q104_M94_full_list} , '4')* | | 1 | Oral - solid (e.g. tablet) |
| | | | 2 | Oral - liquid |
| | | | 3 | Sublingual |
| | | | 4 | Intravenous (IV) |
| | | | 5 | Intramusculuar injection (IM) |
| | | | 6 | Subcutaneous |
| | | | 7 | Rectal |
| | | | 8 | Inhaled (e.g. gas) |
| | | | 9 | Inserted (e.g. vaginal pessaries) |
| | | | 10 | Topical - cream |
| | | | 11 | Topical - drops (e.g. eye drops) |
| | | | 12 | Other |
| | | | 99 | Don't know |
| q504_M94c *(required)* | q504_M94c. What is the item's smallest unit of measurement (mg, mcg, ml, g, etc)? <br> *Question relevant when: selected( ${q104_M94_full_list} , '4')* | | microgram | microgram (mcg) |
| | | | miligram | miligram (mg) |
| | | | miligram_dissolved_in_liquid | miligram (mg) dissolved in liquid (e.g. 2 mg per 3 militers) |
| | | | gram | gram (g) |
| | | | militer | militer (ml) |
| | | | centiliter | centiliter (cl) |
| | | | litre | litre (l) |
| | | | ml_of _solution | ml of solution (e.g. ml of 10% solution) |
| | | | litre_of_solution | litre of solution (e.g. 1 litre of 5% solution) |
| | | | cubic_meter | cubic meter (m^3) |
| | | | other | other |
| | | | dont_know | don't know |
| q504_M94c.1 | q504_M94c.1 If other unit of measurement or route of administration, specify here: <br> *Leave blank if not applicable* <br> *Question relevant when: selected( ${q104_M94_full_list} , '4')* | | | |
| q504_M94d *(required)* | q504_M94d. In total, how many units are used to treat a patient during all of her care? <br> *Remember to add all units! E.g. 5 days x 200 mg twice per day = 2,000mg* <br> *Question relevant when: selected( ${q104_M94_full_list} , '4')* | | | |

[illegible]

*Response constrained to: .>0*

| Field | Question | Answer |
|---|---|---|
| q504_M95 | ***q504_M95. Saline, glucose, water, etc. - Other 5: "[q104_M95_full_list_other]"***<br>*Question relevant when: selected( ${q104_M95_full_list} , '4')* | |
| q504_M95a *(required)* | q504_M95a. What percent of patients require this item?<br>*Question relevant when: selected( ${q104_M95_full_list} , '4')*<br>*Response constrained to: .>0 and .<=100 or .=999* | |
| q504_M95b *(required)* | q504_M95b. How is the item administered, or given, to the patient?<br>*Question relevant when: selected( ${q104_M95_full_list} , '4')* | 1 Oral - solid (e.g. tablet)<br>2 Oral - liquid<br>3 Sublingual<br>4 Intravenous (IV)<br>5 Intramusculuar injection (IM)<br>6 Subcutaneous<br>7 Rectal<br>8 Inhaled (e.g. gas)<br>9 Inserted (e.g. vaginal pessaries)<br>10 Topical - cream<br>11 Topical - drops (e.g. eye drops)<br>12 Other<br>99 Don't know |
| q504_M95c *(required)* | q504_M95c. What is the item's smallest unit of measurement (mg, mcg, ml, g, etc)?<br>*Question relevant when: selected( ${q104_M95_full_list} , '4')* | microgram — microgram (mcg)<br>miligram — miligram (mg)<br>miligram_dissolved_in_liquid — miligram (mg) dissolved in liquid (e.g. 2 mg per 3 militers)<br>gram — gram (g)<br>militer — militer (ml)<br>centiliter — centiliter (cl)<br>litre — litre (l)<br>ml_of _solution — ml of solution (e.g. ml of 10% solution)<br>litre_of_solution — litre of solution (e.g. 1 litre of 5% solution)<br>cubic_meter — cubic meter (m^3)<br>other — other<br>dont_know — don't know |
| q504_M95c.1 | q504_M95c.1 If other unit of measurement or route of administration, specify here:<br>*Leave blank if not applicable*<br>*Question relevant when: selected( ${q104_M95_full_list} , '4')* | |
| q504_M95d *(required)* | q504_M95d. In total, how many units are used to treat a patient during all of her care?<br>*Remember to add all units! E.g. 5 days x 200 mg twice per day = 2,000mg*<br>*Question relevant when: selected( ${q104_M95_full_list} , '4')* | |

Question relevant when: selected( ${q104_[illegible]_full_list} , '4')
Response constrained to: .>0

| Field | Question | Answer |
|---|---|---|
| <p><strong><em>D. Medications - Lacerations (10)</em></strong></p> | | |
| *Group relevant when: selected( ${section_five_skip_med} , '1')* | | |
| q504_med_notE9 | 504 CONTINUED For each of the following medications and other medical products that are used for lacerations, can you tell me …? | |
| note_504_prost_utero | ***Prostaglandin, uterotonic, etc.*** | |
| q504_M96 | ***q504_M96. Misoprostol***<br>*Question relevant when: selected( ${q104_M96_full_list} , '4')* | |
| q504_M96a *(required)* | q504_M96a. What percent of patients require this item?<br>*Question relevant when: selected( ${q104_M96_full_list} , '4')*<br>*Response constrained to: .>0 and .<=100 or .=999* | |

| Field | Question | Answer |
|---|---|---|
| q504_M96b *(required)* | q504_M96b. How is the item administered, or given, to the patient?<br>*Question relevant when: selected( ${q104_M96_full_list} , '4')* | 1 Oral - solid (e.g. tablet)<br>2 Oral - liquid<br>3 Sublingual<br>4 Intravenous (IV)<br>5 Intramusculuar injection (IM)<br>6 Subcutaneous<br>7 Rectal<br>8 Inhaled (e.g. gas)<br>9 Inserted (e.g. vaginal pessaries)<br>10 Topical - cream<br>11 Topical - drops (e.g. eye drops)<br>12 Other<br>99 Don't know |
| q504_M96c *(required)* | q504_M96c. What is the item's smallest unit of measurement (mg, mcg, ml, g, etc)?<br>*Question relevant when: selected( ${q104_M96_full_list} , '4')* | microgram — microgram (mcg)<br>miligram — miligram (mg)<br>miligram_dissolved_in_liquid — miligram (mg) dissolved in liquid (e.g. 2 mg per 3 militers)<br>gram — gram (g)<br>militer — militer (ml)<br>centiliter — centiliter (cl)<br>litre — litre (l)<br>ml_of _solution — ml of solution (e.g. ml of 10% solution)<br>litre_of_solution — litre of solution (e.g. 1 litre of 5% solution)<br>cubic_meter — cubic meter (m^3)<br>other — other<br>dont_know — don't know |
| q504_M96c.1 | q504_M96c.1 If other unit of measurement or route of administration, specify here:<br>*Leave blank if not applicable*<br>*Question relevant when: selected( ${q104_M96_full_list} , '4')* | |
| q504_M96d *(required)* | q504_M96d. In total, how many units are used to treat a patient during all of her care?<br>*Remember to add all units! E.g. 5 days x 200 mg twice per day = 2,000mg*<br>*Question relevant when: selected( ${q104_M96_full_list} , '4')* | |

Question relevant when: selected( ${q104_M96_full_list} , '4')
Response constrained to: .>0

| Field | Question | Answer |
|---|---|---|
| q504_M97 | ***q504_M97. Ergometrine***<br>*Question relevant when: selected( ${q104_M97_full_list} , '4')* | |
| q504_M97a *(required)* | q504_M97a. What percent of patients require this item?<br>*Question relevant when: selected( ${q104_M97_full_list} , '4')*<br>*Response constrained to: .>0 and .<=100 or .=999* | |
| q504_M97b *(required)* | q504_M97b. How is the item administered, or given, to the patient?<br>*Question relevant when: selected( ${q104_M97_full_list} , '4')* | 1 Oral - solid (e.g. tablet)<br>2 Oral - liquid<br>3 Sublingual<br>4 Intravenous (IV)<br>5 Intramusculuar injection (IM)<br>6 Subcutaneous<br>7 Rectal<br>8 Inhaled (e.g. gas)<br>9 Inserted (e.g. vaginal pessaries)<br>10 Topical - cream<br>11 Topical - drops (e.g. eye drops)<br>12 Other<br>99 Don't know |
| q504_M97c *(required)* | q504_M97c. What is the item's smallest unit of measurement (mg, mcg, ml, g, etc)?<br>*Question relevant when: selected( ${q104_M97_full_list} , '4')* | microgram: microgram (mcg)<br>miligram: miligram (mg)<br>miligram_dissolved_in_liquid: miligram (mg) dissolved in liquid (e.g. 2 mg per 3 militers)<br>gram: gram (g)<br>militer: militer (ml)<br>centiliter: centiliter (cl)<br>litre: litre (l)<br>ml_of _solution: ml of solution (e.g. ml of 10% solution)<br>litre_of_solution: litre of solution (e.g. 1 litre of 5% solution)<br>cubic_meter: cubic meter (m^3)<br>other: other<br>dont_know: don't know |
| q504_M97c.1 | q504_M97c.1 If other unit of measurement or route of administration, specify here:<br>*Leave blank if not applicable*<br>*Question relevant when: selected( ${q104_M97_full_list} , '4')* | |
| q504_M97d *(required)* | q504_M97d. In total, how many units are used to treat a patient during all of her care?<br>*Remember to add all units! E.g. 5 days x 200 mg twice per day = 2,000mg*<br>*Question relevant when: selected( ${q104_M97_full_list} , '4')* | |

*Response constrained to: .>0*

| Field | Question | Answer |
|---|---|---|
| q504_M98 | ***q504_M98. Methylergonovine Maleate (Methergine)***<br>*Question relevant when: selected( ${q104_M98_full_list} , '4')* | |
| q504_M98a *(required)* | q504_M98a. What percent of patients require this item?<br>*Question relevant when: selected( ${q104_M98_full_list} , '4')*<br>*Response constrained to: .>0 and .<=100 or .=999* | |
| q504_M98b *(required)* | q504_M98b. How is the item administered, or given, to the patient?<br>*Question relevant when: selected( ${q104_M98_full_list} , '4')* | 1 Oral - solid (e.g. tablet)<br>2 Oral - liquid<br>3 Sublingual<br>4 Intravenous (IV)<br>5 Intramusculuar injection (IM)<br>6 Subcutaneous<br>7 Rectal<br>8 Inhaled (e.g. gas)<br>9 Inserted (e.g. vaginal pessaries)<br>10 Topical - cream<br>11 Topical - drops (e.g. eye drops)<br>12 Other<br>99 Don't know |
| q504_M98c *(required)* | q504_M98c. What is the item's smallest unit of measurement (mg, mcg, ml, g, etc)?<br>*Question relevant when: selected( ${q104_M98_full_list} , '4')* | microgram — microgram (mcg)<br>miligram — miligram (mg)<br>miligram_dissolved_in_liquid — miligram (mg) dissolved in liquid (e.g. 2 mg per 3 militers)<br>gram — gram (g)<br>militer — militer (ml)<br>centiliter — centiliter (cl)<br>litre — litre (l)<br>ml_of _solution — ml of solution (e.g. ml of 10% solution)<br>litre_of_solution — litre of solution (e.g. 1 litre of 5% solution)<br>cubic_meter — cubic meter (m^3)<br>other — other<br>dont_know — don't know |
| q504_M98c.1 | q504_M98c.1 If other unit of measurement or route of administration, specify here:<br>*Leave blank if not applicable*<br>*Question relevant when: selected( ${q104_M98_full_list} , '4')* | |
| q504_M98d *(required)* | q504_M98d. In total, how many units are used to treat a patient during all of her care?<br>*Remember to add all units! E.g. 5 days x 200 mg twice per day = 2,000mg*<br>*Question relevant when: selected( ${q104_M98_full_list} , '4')* | |

Question relevant when: selected( ${q104_M98_full_list} , '4')

Response constrained to: .>0

| Field | Question | Answer |
|---|---|---|
| q504_M99 | ***q504_M99. Mifepristone***<br>*Question relevant when: selected( ${q104_M99_full_list} , '4')* | |
| q504_M99a *(required)* | q504_M99a. What percent of patients require this item?<br>*Question relevant when: selected( ${q104_M99_full_list} , '4')*<br>*Response constrained to: .>0 and .<=100 or .=999* | |
| q504_M99b *(required)* | q504_M99b. How is the item administered, or given, to the patient?<br>*Question relevant when: selected( ${q104_M99_full_list} , '4')* | 1 Oral - solid (e.g. tablet)<br>2 Oral - liquid<br>3 Sublingual<br>4 Intravenous (IV)<br>5 Intramusculuar injection (IM)<br>6 Subcutaneous<br>7 Rectal<br>8 Inhaled (e.g. gas)<br>9 Inserted (e.g. vaginal pessaries)<br>10 Topical - cream<br>11 Topical - drops (e.g. eye drops)<br>12 Other<br>99 Don't know |
| q504_M99c *(required)* | q504_M99c. What is the item's smallest unit of measurement (mg, mcg, ml, g, etc)?<br>*Question relevant when: selected( ${q104_M99_full_list} , '4')* | microgram — microgram (mcg)<br>miligram — miligram (mg)<br>miligram_dissolved_in_liquid — miligram (mg) dissolved in liquid (e.g. 2 mg per 3 militers)<br>gram — gram (g)<br>militer — militer (ml)<br>centiliter — centiliter (cl)<br>litre — litre (l)<br>ml_of _solution — ml of solution (e.g. ml of 10% solution)<br>litre_of_solution — litre of solution (e.g. 1 litre of 5% solution)<br>cubic_meter — cubic meter (m^3)<br>other — other<br>dont_know — don't know |
| q504_M99c.1 | q504_M99c.1 If other unit of measurement or route of administration, specify here:<br>*Leave blank if not applicable*<br>*Question relevant when: selected( ${q104_M99_full_list} , '4')* | |
| q504_M99d *(required)* | q504_M99d. In total, how many units are used to treat a patient during all of her care?<br>*Remember to add all units! E.g. 5 days x 200 mg twice per day = 2,000mg*<br>*Question relevant when: selected( ${q104_M99_full_list} , '4')* | |

Question relevant when: selected( ${q104_[illegible]_full_list} , '4')

Response constrained to: .>0

| Field | Question | Answer |
|---|---|---|
| q504_M100 | ***q504_M100. Oxytocin***<br>*Question relevant when: selected( ${q104_M100_full_list} , '4')* | |
| q504_M100a *(required)* | q504_M100a. What percent of patients require this item?<br>*Question relevant when: selected( ${q104_M100_full_list} , '4')*<br>*Response constrained to: .>0 and .<=100 or .=999* | |
| q504_M100b *(required)* | q504_M100b. How is the item administered, or given, to the patient?<br>*Question relevant when: selected( ${q104_M100_full_list} , '4')* | 1 Oral - solid (e.g. tablet)<br>2 Oral - liquid<br>3 Sublingual<br>4 Intravenous (IV)<br>5 Intramusculuar injection (IM)<br>6 Subcutaneous<br>7 Rectal<br>8 Inhaled (e.g. gas)<br>9 Inserted (e.g. vaginal pessaries)<br>10 Topical - cream<br>11 Topical - drops (e.g. eye drops)<br>12 Other<br>99 Don't know |
| q504_M100c *(required)* | q504_M100c. What is the item's smallest unit of measurement (mg, mcg, ml, g, etc)?<br>*Question relevant when: selected( ${q104_M100_full_list} , '4')* | microgram — microgram (mcg)<br>miligram — miligram (mg)<br>miligram_dissolved_in_liquid — miligram (mg) dissolved in liquid (e.g. 2 mg per 3 militers)<br>gram — gram (g)<br>militer — militer (ml)<br>centiliter — centiliter (cl)<br>litre — litre (l)<br>ml_of _solution — ml of solution (e.g. ml of 10% solution)<br>litre_of_solution — litre of solution (e.g. 1 litre of 5% solution)<br>cubic_meter — cubic meter (m^3)<br>other — other<br>dont_know — don't know |
| q504_M100c.1 | q504_M100c.1 If other unit of measurement or route of administration, specify here:<br>*Leave blank if not applicable*<br>*Question relevant when: selected( ${q104_M100_full_list} , '4')* | |
| q504_M100d *(required)* | q504_M100d. In total, how many units are used to treat a patient during all of her care?<br>*Remember to add all units! E.g. 5 days x 200 mg twice per day = 2,000mg*<br>*Question relevant when: selected( ${q104_M100_full_list} , '4')* | |

*Question relevant when: selected( ${q104_M100_full_list} , '4')*

*Response constrained to: .>0*

| Field | Question | Answer |
|---|---|---|
| q504_M101 | ***q504_M101. Prostaglandin, uterotonic, etc. - Other 1: "[q104_M101_full_list_other]"***<br>*Question relevant when: selected( ${q104_M101_full_list} , '4')* | |
| q504_M101a *(required)* | q504_M101a. What percent of patients require this item?<br>*Question relevant when: selected( ${q104_M101_full_list} , '4')*<br>*Response constrained to: .>0 and .<=100 or .=999* | |
| q504_M101b *(required)* | q504_M101b. How is the item administered, or given, to the patient?<br>*Question relevant when: selected( ${q104_M101_full_list} , '4')* | 1 Oral - solid (e.g. tablet)<br>2 Oral - liquid<br>3 Sublingual<br>4 Intravenous (IV)<br>5 Intramusculuar injection (IM)<br>6 Subcutaneous<br>7 Rectal<br>8 Inhaled (e.g. gas)<br>9 Inserted (e.g. vaginal pessaries)<br>10 Topical - cream<br>11 Topical - drops (e.g. eye drops)<br>12 Other<br>99 Don't know |
| q504_M101c *(required)* | q504_M101c. What is the item's smallest unit of measurement (mg, mcg, ml, g, etc)?<br>*Question relevant when: selected( ${q104_M101_full_list} , '4')* | microgram — microgram (mcg)<br>miligram — miligram (mg)<br>miligram_dissolved_in_liquid — miligram (mg) dissolved in liquid (e.g. 2 mg per 3 militers)<br>gram — gram (g)<br>militer — militer (ml)<br>centiliter — centiliter (cl)<br>litre — litre (l)<br>ml_of _solution — ml of solution (e.g. ml of 10% solution)<br>litre_of_solution — litre of solution (e.g. 1 litre of 5% solution)<br>cubic_meter — cubic meter (m^3)<br>other — other<br>dont_know — don't know |
| q504_M101c.1 | q504_M101c.1 If other unit of measurement or route of administration, specify here:<br>*Leave blank if not applicable*<br>*Question relevant when: selected( ${q104_M101_full_list} , '4')* | |
| q504_M101d *(required)* | q504_M101d. In total, how many units are used to treat a patient during all of her care?<br>*Remember to add all units! E.g. 5 days x 200 mg twice per day = 2,000mg*<br>*Question relevant when: selected( ${q104_M101_full_list} , '4')* | |

*Question relevant when: selected( ${q104_M101_full_list} , '4')*

*Response constrained to: .>0*

| Field | Question | Answer |
|---|---|---|
| q504_M102 | ***q504_M102. Prostaglandin, uterotonic, etc. - Other 2: "[q104_M102_full_list_other]"***<br>*Question relevant when: selected( ${q104_M102_full_list} , '4')* | |
| q504_M102a *(required)* | q504_M102a. What percent of patients require this item?<br>*Question relevant when: selected( ${q104_M102_full_list} , '4')*<br>*Response constrained to: .>0 and .<=100 or .=999* | |
| q504_M102b *(required)* | q504_M102b. How is the item administered, or given, to the patient?<br>*Question relevant when: selected( ${q104_M102_full_list} , '4')* | 1 Oral - solid (e.g. tablet)<br>2 Oral - liquid<br>3 Sublingual<br>4 Intravenous (IV)<br>5 Intramusculuar injection (IM)<br>6 Subcutaneous<br>7 Rectal<br>8 Inhaled (e.g. gas)<br>9 Inserted (e.g. vaginal pessaries)<br>10 Topical - cream<br>11 Topical - drops (e.g. eye drops)<br>12 Other<br>99 Don't know |
| q504_M102c *(required)* | q504_M102c. What is the item's smallest unit of measurement (mg, mcg, ml, g, etc)?<br>*Question relevant when: selected( ${q104_M102_full_list} , '4')* | microgram — microgram (mcg)<br>miligram — miligram (mg)<br>miligram_dissolved_in_liquid — miligram (mg) dissolved in liquid (e.g. 2 mg per 3 militers)<br>gram — gram (g)<br>militer — militer (ml)<br>centiliter — centiliter (cl)<br>litre — litre (l)<br>ml_of _solution — ml of solution (e.g. ml of 10% solution)<br>litre_of_solution — litre of solution (e.g. 1 litre of 5% solution)<br>cubic_meter — cubic meter (m^3)<br>other — other<br>dont_know — don't know |
| q504_M102c.1 | q504_M102c.1 If other unit of measurement or route of administration, specify here:<br>*Leave blank if not applicable*<br>*Question relevant when: selected( ${q104_M102_full_list} , '4')* | |
| q504_M102d *(required)* | q504_M102d. In total, how many units are used to treat a patient during all of her care?<br>*Remember to add all units! E.g. 5 days x 200 mg twice per day = 2,000mg*<br>*Question relevant when: selected( ${q104_M102_full_list} , '4')* | |

| Field | Question | Answer |
|---|---|---|
| | *Question relevant when: selected( ${q104_M[illegible]_full_list} , '4')*<br>*Response constrained to: .>0* | |
| q504_M103 | ***q504_M103. Prostaglandin, uterotonic, etc. - Other 3: "[q104_M103_full_list_other]"***<br>*Question relevant when: selected( ${q104_M104_full_list} , '4')* | |
| q504_M103a *(required)* | q504_M103a. What percent of patients require this item?<br>*Question relevant when: selected( ${q104_M104_full_list} , '4')*<br>*Response constrained to: .>0 and .<=100 or .=999* | |
| q504_M103b *(required)* | q504_M103b. How is the item administered, or given, to the patient?<br>*Question relevant when: selected( ${q104_M104_full_list} , '4')* | 1 Oral - solid (e.g. tablet)<br>2 Oral - liquid<br>3 Sublingual<br>4 Intravenous (IV)<br>5 Intramusculuar injection (IM)<br>6 Subcutaneous<br>7 Rectal<br>8 Inhaled (e.g. gas)<br>9 Inserted (e.g. vaginal pessaries)<br>10 Topical - cream<br>11 Topical - drops (e.g. eye drops)<br>12 Other<br>99 Don't know |
| q504_M103c *(required)* | q504_M103c. What is the item's smallest unit of measurement (mg, mcg, ml, g, etc)?<br>*Question relevant when: selected( ${q104_M104_full_list} , '4')* | microgram — microgram (mcg)<br>miligram — miligram (mg)<br>miligram_dissolved_in_liquid — miligram (mg) dissolved in liquid (e.g. 2 mg per 3 militers)<br>gram — gram (g)<br>militer — militer (ml)<br>centiliter — centiliter (cl)<br>litre — litre (l)<br>ml_of _solution — ml of solution (e.g. ml of 10% solution)<br>litre_of_solution — litre of solution (e.g. 1 litre of 5% solution)<br>cubic_meter — cubic meter (m^3)<br>other — other<br>dont_know — don't know |
| q504_M103c.1 | q504_M103c.1 If other unit of measurement or route of administration, specify here:<br>*Leave blank if not applicable*<br>*Question relevant when: selected( ${q104_M104_full_list} , '4')* | |
| q504_M103d *(required)* | q504_M103d. In total, how many units are used to treat a patient during all of her care?<br>*Remember to add all units! E.g. 5 days x 200 mg twice per day = 2,000mg*<br>*Question relevant when: selected( ${q104_M104_full_list} , '4')* | |

Question relevant when: selected( ${q104_M104_full_list} , '4')

Response constrained to: .>0

| Field | Question | Answer |
|---|---|---|
| q504_M104 | ***q504_M104. Prostaglandin, uterotonic, etc. - Other 4: "[q104_M104_full_list_other]"***<br>*Question relevant when: selected( ${q104_M104_full_list} , '4')* | |
| q504_M104a *(required)* | q504_M104a. What percent of patients require this item?<br>*Question relevant when: selected( ${q104_M104_full_list} , '4')*<br>*Response constrained to: .>0 and .<=100 or .=999* | |
| q504_M104b *(required)* | q504_M104b. How is the item administered, or given, to the patient?<br>*Question relevant when: selected( ${q104_M104_full_list} , '4')* | 1 Oral - solid (e.g. tablet)<br>2 Oral - liquid<br>3 Sublingual<br>4 Intravenous (IV)<br>5 Intramusculuar injection (IM)<br>6 Subcutaneous<br>7 Rectal<br>8 Inhaled (e.g. gas)<br>9 Inserted (e.g. vaginal pessaries)<br>10 Topical - cream<br>11 Topical - drops (e.g. eye drops)<br>12 Other<br>99 Don't know |
| q504_M104c *(required)* | q504_M104c. What is the item's smallest unit of measurement (mg, mcg, ml, g, etc)?<br>*Question relevant when: selected( ${q104_M104_full_list} , '4')* | microgram: microgram (mcg)<br>miligram: miligram (mg)<br>miligram_dissolved_in_liquid: miligram (mg) dissolved in liquid (e.g. 2 mg per 3 militers)<br>gram: gram (g)<br>militer: militer (ml)<br>centiliter: centiliter (cl)<br>litre: litre (l)<br>ml_of _solution: ml of solution (e.g. ml of 10% solution)<br>litre_of_solution: litre of solution (e.g. 1 litre of 5% solution)<br>cubic_meter: cubic meter (m^3)<br>other: other<br>dont_know: don't know |
| q504_M104c.1 | q504_M104c.1 If other unit of measurement or route of administration, specify here:<br>*Leave blank if not applicable*<br>*Question relevant when: selected( ${q104_M104_full_list} , '4')* | |
| q504_M104d *(required)* | q504_M104d. In total, how many units are used to treat a patient during all of her care?<br>*Remember to add all units! E.g. 5 days x 200 mg twice per day = 2,000mg*<br>*Question relevant when: selected( ${q104_M104_full_list} , '4')* | |

Question relevant when: selected( ${q104_M104_full_list} , '4')

Response constrained to: .>0

| Field | Question | Answer |
|---|---|---|
| q504_M105 | ***q504_M105. Prostaglandin, uterotonic, etc. - Other 5: "[q104_M105_full_list_other]"*** <br> *Question relevant when: selected( ${q104_M105_full_list} , '4')* | |
| q504_M105a *(required)* | q504_M105a. What percent of patients require this item? <br> *Question relevant when: selected( ${q104_M105_full_list} , '4')* <br> *Response constrained to: .>0 and .<=100 or .=999* | |
| q504_M105b *(required)* | q504_M105b. How is the item administered, or given, to the patient? <br> *Question relevant when: selected( ${q104_M105_full_list} , '4')* | 1 Oral - solid (e.g. tablet) <br> 2 Oral - liquid <br> 3 Sublingual <br> 4 Intravenous (IV) <br> 5 Intramusculuar injection (IM) <br> 6 Subcutaneous <br> 7 Rectal <br> 8 Inhaled (e.g. gas) <br> 9 Inserted (e.g. vaginal pessaries) <br> 10 Topical - cream <br> 11 Topical - drops (e.g. eye drops) <br> 12 Other <br> 99 Don't know |
| q504_M105c *(required)* | q504_M105c. What is the item's smallest unit of measurement (mg, mcg, ml, g, etc)? <br> *Question relevant when: selected( ${q104_M105_full_list} , '4')* | microgram — microgram (mcg) <br> miligram — miligram (mg) <br> miligram_dissolved_in_liquid — miligram (mg) dissolved in liquid (e.g. 2 mg per 3 militers) <br> gram — gram (g) <br> militer — militer (ml) <br> centiliter — centiliter (cl) <br> litre — litre (l) <br> ml_of _solution — ml of solution (e.g. ml of 10% solution) <br> litre_of_solution — litre of solution (e.g. 1 litre of 5% solution) <br> cubic_meter — cubic meter (m^3) <br> other — other <br> dont_know — don't know |
| q504_M105c.1 | q504_M105c.1 If other unit of measurement or route of administration, specify here: <br> *Leave blank if not applicable* <br> *Question relevant when: selected( ${q104_M105_full_list} , '4')* | |
| q504_M105d *(required)* | q504_M105d. In total, how many units are used to treat a patient during all of her care? <br> *Remember to add all units! E.g. 5 days x 200 mg twice per day = 2,000mg* <br> *Question relevant when: selected( ${q104_M105_full_list} , '4')* | |

[illegible]

*Response constrained to: .>0*

| Field | Question | Answer |
|---|---|---|
| <p><strong><em>D. Medications - Lacerations (11)</em></strong></p> | | |
| *Group relevant when: selected( ${section_five_skip_med} , '1')* | | |
| q504_med_notE10 | 504 CONTINUED For each of the following medications and other medical products that are used for lacerations, can you tell me …? | |
| note_504_vitamin | ***Vitamin, immunological, etc.*** | |
| q504_M106 | ***q504_M106. Ferrous sulphate (tablets)***<br>*Question relevant when: selected( ${q104_M106_full_list} , '4')* | |
| q504_M106a *(required)* | q504_M106a. What percent of patients require this item?<br>*Question relevant when: selected( ${q104_M106_full_list} , '4')*<br>*Response constrained to: .>0 and .<=100 or .=999* | |
| q504_M106b *(required)* | q504_M106b. How is the item administered, or given, to the patient?<br>*Question relevant when: selected( ${q104_M106_full_list} , '4')* | 1 Oral - solid (e.g. tablet)<br>2 Oral - liquid<br>3 Sublingual<br>4 Intravenous (IV)<br>5 Intramusculuar injection (IM)<br>6 Subcutaneous<br>7 Rectal<br>8 Inhaled (e.g. gas)<br>9 Inserted (e.g. vaginal pessaries)<br>10 Topical - cream<br>11 Topical - drops (e.g. eye drops)<br>12 Other<br>99 Don't know |
| q504_M106c *(required)* | q504_M106c. What is the item's smallest unit of measurement (mg, mcg, ml, g, etc)?<br>*Question relevant when: selected( ${q104_M106_full_list} , '4')* | microgram — microgram (mcg)<br>miligram — miligram (mg)<br>miligram_dissolved_in_liquid — miligram (mg) dissolved in liquid (e.g. 2 mg per 3 militers)<br>gram — gram (g)<br>militer — militer (ml)<br>centiliter — centiliter (cl)<br>litre — litre (l)<br>ml_of _solution — ml of solution (e.g. ml of 10% solution)<br>litre_of_solution — litre of solution (e.g. 1 litre of 5% solution)<br>cubic_meter — cubic meter (m^3)<br>other — other<br>dont_know — don't know |
| q504_M106c.1 | q504_M106c.1 If other unit of measurement or route of administration, specify here:<br>*Leave blank if not applicable*<br>*Question relevant when: selected( ${q104_M106_full_list} , '4')* | |
| q504_M106d *(required)* | q504_M106d. In total, how many units are used to treat a patient during all of her care?<br>*Remember to add all units! E.g. 5 days x 200 mg twice per day = 2,000mg*<br>*Question relevant when: selected( ${q104_M106_full_list} , '4')* | |

Question relevant when: selected( ${q104_M106_full_list} , '4')

Response constrained to: .>0

| Field | Question | Answer |
|---|---|---|
| q504_M107 | ***q504_M107. Ferrous sulphate + folic acid (Ferrotone capsules)***<br>*Question relevant when: selected( ${q104_M107_full_list} , '4')* | |
| q504_M107a *(required)* | q504_M107a. What percent of patients require this item?<br>*Question relevant when: selected( ${q104_M107_full_list} , '4')*<br>*Response constrained to: .>0 and .<=100 or .=999* | |
| q504_M107b *(required)* | q504_M107b. How is the item administered, or given, to the patient?<br>*Question relevant when: selected( ${q104_M107_full_list} , '4')* | 1 Oral - solid (e.g. tablet)<br>2 Oral - liquid<br>3 Sublingual<br>4 Intravenous (IV)<br>5 Intramusculuar injection (IM)<br>6 Subcutaneous<br>7 Rectal<br>8 Inhaled (e.g. gas)<br>9 Inserted (e.g. vaginal pessaries)<br>10 Topical - cream<br>11 Topical - drops (e.g. eye drops)<br>12 Other<br>99 Don't know |
| q504_M107c *(required)* | q504_M107c. What is the item's smallest unit of measurement (mg, mcg, ml, g, etc)?<br>*Question relevant when: selected( ${q104_M107_full_list} , '4')* | microgram — microgram (mcg)<br>miligram — miligram (mg)<br>miligram_dissolved_in_liquid — miligram (mg) dissolved in liquid (e.g. 2 mg per 3 militers)<br>gram — gram (g)<br>militer — militer (ml)<br>centiliter — centiliter (cl)<br>litre — litre (l)<br>ml_of _solution — ml of solution (e.g. ml of 10% solution)<br>litre_of_solution — litre of solution (e.g. 1 litre of 5% solution)<br>cubic_meter — cubic meter (m^3)<br>other — other<br>dont_know — don't know |
| q504_M107c.1 | q504_M107c.1 If other unit of measurement or route of administration, specify here:<br>*Leave blank if not applicable*<br>*Question relevant when: selected( ${q104_M107_full_list} , '4')* | |
| q504_M107d *(required)* | q504_M107d. In total, how many units are used to treat a patient during all of her care?<br>*Remember to add all units! E.g. 5 days x 200 mg twice per day = 2,000mg*<br>*Question relevant when: selected( ${q104_M107_full_list} , '4')* | |

Question relevant when: selected( ${q104_M107_full_list} , '4')

| Field | Question | Answer |
|---|---|---|
| | *Response constrained to: .>0* | |
| q504_M108 | ***q504_M108. Folic acid (Tablets)***<br>*Question relevant when: selected( ${q104_M108_full_list} , '4')* | |
| q504_M108a *(required)* | q504_M108a. What percent of patients require this item?<br>*Question relevant when: selected( ${q104_M108_full_list} , '4')*<br>*Response constrained to: .>0 and .<=100 or .=999* | |
| q504_M108b *(required)* | q504_M108b. How is the item administered, or given, to the patient?<br>*Question relevant when: selected( ${q104_M108_full_list} , '4')* | 1 Oral - solid (e.g. tablet)<br>2 Oral - liquid<br>3 Sublingual<br>4 Intravenous (IV)<br>5 Intramusculuar injection (IM)<br>6 Subcutaneous<br>7 Rectal<br>8 Inhaled (e.g. gas)<br>9 Inserted (e.g. vaginal pessaries)<br>10 Topical - cream<br>11 Topical - drops (e.g. eye drops)<br>12 Other<br>99 Don't know |
| q504_M108c *(required)* | q504_M108c. What is the item's smallest unit of measurement (mg, mcg, ml, g, etc)?<br>*Question relevant when: selected( ${q104_M108_full_list} , '4')* | microgram — microgram (mcg)<br>miligram — miligram (mg)<br>miligram_dissolved_in_liquid — miligram (mg) dissolved in liquid (e.g. 2 mg per 3 militers)<br>gram — gram (g)<br>militer — militer (ml)<br>centiliter — centiliter (cl)<br>litre — litre (l)<br>ml_of _solution — ml of solution (e.g. ml of 10% solution)<br>litre_of_solution — litre of solution (e.g. 1 litre of 5% solution)<br>cubic_meter — cubic meter (m^3)<br>other — other<br>dont_know — don't know |
| q504_M108c.1 | q504_M108c.1 If other unit of measurement or route of administration, specify here:<br>*Leave blank if not applicable*<br>*Question relevant when: selected( ${q104_M108_full_list} , '4')* | |
| q504_M108d *(required)* | q504_M108d. In total, how many units are used to treat a patient during all of her care?<br>*Remember to add all units! E.g. 5 days x 200 mg twice per day = 2,000mg*<br>*Question relevant when: selected( ${q104_M108_full_list} , '4')* | |

[illegible]

*Response constrained to: .>0*

| Field | Question | Answer |
|---|---|---|
| q504_M109 | ***q504_M109. Serum anti-tetanus***<br>*Question relevant when: selected( ${q104_M109_full_list} , '4')* | |
| q504_M109a *(required)* | q504_M109a. What percent of patients require this item?<br>*Question relevant when: selected( ${q104_M109_full_list} , '4')*<br>*Response constrained to: .>0 and .<=100 or .=999* | |
| q504_M109b *(required)* | q504_M109b. How is the item administered, or given, to the patient?<br>*Question relevant when: selected( ${q104_M109_full_list} , '4')* | 1 Oral - solid (e.g. tablet)<br>2 Oral - liquid<br>3 Sublingual<br>4 Intravenous (IV)<br>5 Intramusculuar injection (IM)<br>6 Subcutaneous<br>7 Rectal<br>8 Inhaled (e.g. gas)<br>9 Inserted (e.g. vaginal pessaries)<br>10 Topical - cream<br>11 Topical - drops (e.g. eye drops)<br>12 Other<br>99 Don't know |
| q504_M109c *(required)* | q504_M109c. What is the item's smallest unit of measurement (mg, mcg, ml, g, etc)?<br>*Question relevant when: selected( ${q104_M109_full_list} , '4')* | microgram — microgram (mcg)<br>miligram — miligram (mg)<br>miligram_dissolved_in_liquid — miligram (mg) dissolved in liquid (e.g. 2 mg per 3 militers)<br>gram — gram (g)<br>militer — militer (ml)<br>centiliter — centiliter (cl)<br>litre — litre (l)<br>ml_of _solution — ml of solution (e.g. ml of 10% solution)<br>litre_of_solution — litre of solution (e.g. 1 litre of 5% solution)<br>cubic_meter — cubic meter (m^3)<br>other — other<br>dont_know — don't know |
| q504_M109c.1 | q504_M109c.1 If other unit of measurement or route of administration, specify here:<br>*Leave blank if not applicable*<br>*Question relevant when: selected( ${q104_M109_full_list} , '4')* | |
| q504_M109d *(required)* | q504_M109d. In total, how many units are used to treat a patient during all of her care?<br>*Remember to add all units! E.g. 5 days x 200 mg twice per day = 2,000mg*<br>*Question relevant when: selected( ${q104_M109_full_list} , '4')* | |

| Field | Question | Answer |
|---|---|---|
| | Question relevant when: selected( ${q104_M109_full_list} , '4')<br>Response constrained to: .>0 | |
| q504_M110 | ***q504_M110. Anti D (Rh)-Immunoglobulin***<br>*Question relevant when: selected( ${q104_M110_full_list} , '4')* | |
| q504_M110a *(required)* | q504_M110a. What percent of patients require this item?<br>*Question relevant when: selected( ${q104_M110_full_list} , '4')*<br>*Response constrained to: .>0 and .<=100 or .=999* | |

| Field | Question | Answer |
|---|---|---|
| q504_M110b *(required)* | q504_M110b. How is the item administered, or given, to the patient?<br>*Question relevant when: selected( ${q104_M110_full_list} , '4')* | 1 Oral - solid (e.g. tablet)<br>2 Oral - liquid<br>3 Sublingual<br>4 Intravenous (IV)<br>5 Intramusculuar injection (IM)<br>6 Subcutaneous<br>7 Rectal<br>8 Inhaled (e.g. gas)<br>9 Inserted (e.g. vaginal pessaries)<br>10 Topical - cream<br>11 Topical - drops (e.g. eye drops)<br>12 Other<br>99 Don't know |
| q504_M110c *(required)* | q504_M110c. What is the item's smallest unit of measurement (mg, mcg, ml, g, etc)?<br>*Question relevant when: selected( ${q104_M110_full_list} , '4')* | microgram — microgram (mcg)<br>miligram — miligram (mg)<br>miligram_dissolved_in_liquid — miligram (mg) dissolved in liquid (e.g. 2 mg per 3 militers)<br>gram — gram (g)<br>militer — militer (ml)<br>centiliter — centiliter (cl)<br>litre — litre (l)<br>ml_of _solution — ml of solution (e.g. ml of 10% solution)<br>litre_of_solution — litre of solution (e.g. 1 litre of 5% solution)<br>cubic_meter — cubic meter (m^3)<br>other — other<br>dont_know — don't know |
| q504_M110c.1 | q504_M110c.1 If other unit of measurement or route of administration, specify here:<br>*Leave blank if not applicable*<br>*Question relevant when: selected( ${q104_M110_full_list} , '4')* | |
| q504_M110d *(required)* | q504_M110d. In total, how many units are used to treat a patient during all of her care?<br>*Remember to add all units! E.g. 5 days x 200 mg twice per day = 2,000mg*<br>*Question relevant when: selected( ${q104_M110_full_list} , '4')* | |

Question relevant when: selected( ${q104_M110_full_list} , '4')

Response constrained to: .>0

| Field | Question | Answer |
|---|---|---|
| q504_M111 | ***q504_M111. Vitamin, immunological, etc. - Other 1: "[q104_M111_full_list_other]"*** *Question relevant when: selected( ${q104_M111_full_list} , '4')* | |
| q504_M111a *(required)* | q504_M111a. What percent of patients require this item? *Question relevant when: selected( ${q104_M111_full_list} , '4')* *Response constrained to: .>0 and .<=100 or .=999* | |
| q504_M111b *(required)* | q504_M111b. How is the item administered, or given, to the patient? *Question relevant when: selected( ${q104_M111_full_list} , '4')* | 1 Oral - solid (e.g. tablet)<br>2 Oral - liquid<br>3 Sublingual<br>4 Intravenous (IV)<br>5 Intramusculuar injection (IM)<br>6 Subcutaneous<br>7 Rectal<br>8 Inhaled (e.g. gas)<br>9 Inserted (e.g. vaginal pessaries)<br>10 Topical - cream<br>11 Topical - drops (e.g. eye drops)<br>12 Other<br>99 Don't know |
| q504_M111c *(required)* | q504_M111c. What is the item's smallest unit of measurement (mg, mcg, ml, g, etc)? *Question relevant when: selected( ${q104_M111_full_list} , '4')* | microgram — microgram (mcg)<br>miligram — miligram (mg)<br>miligram_dissolved_in_liquid — miligram (mg) dissolved in liquid (e.g. 2 mg per 3 militers)<br>gram — gram (g)<br>militer — militer (ml)<br>centiliter — centiliter (cl)<br>litre — litre (l)<br>ml_of _solution — ml of solution (e.g. ml of 10% solution)<br>litre_of_solution — litre of solution (e.g. 1 litre of 5% solution)<br>cubic_meter — cubic meter (m^3)<br>other — other<br>dont_know — don't know |
| q504_M111c.1 | q504_M111c.1 If other unit of measurement or route of administration, specify here: *Leave blank if not applicable* *Question relevant when: selected( ${q104_M111_full_list} , '4')* | |
| q504_M111d *(required)* | q504_M111d. In total, how many units are used to treat a patient during all of her care? *Remember to add all units! E.g. 5 days x 200 mg twice per day = 2,000mg* *Question relevant when: selected( ${q104_M111_full_list} , '4')* | |

Question relevant when: selected( ${q104_M111_full_list} , '4')

Response constrained to: .>0

| Field | Question | Answer |
|---|---|---|
| q504_M112 | ***q504_M112. Vitamin, immunological, etc. - Other 2: "[q104_M112_full_list_other]"*** <br> *Question relevant when: selected( ${q104_M112_full_list} , '4')* | |
| q504_M112a *(required)* | q504_M112a. What percent of patients require this item? <br> *Question relevant when: selected( ${q104_M112_full_list} , '4')* <br> *Response constrained to: .>0 and .<=100 or .=999* | |
| q504_M112b *(required)* | q504_M112b. How is the item administered, or given, to the patient? <br> *Question relevant when: selected( ${q104_M112_full_list} , '4')* | 1 Oral - solid (e.g. tablet) <br> 2 Oral - liquid <br> 3 Sublingual <br> 4 Intravenous (IV) <br> 5 Intramusculuar injection (IM) <br> 6 Subcutaneous <br> 7 Rectal <br> 8 Inhaled (e.g. gas) <br> 9 Inserted (e.g. vaginal pessaries) <br> 10 Topical - cream <br> 11 Topical - drops (e.g. eye drops) <br> 12 Other <br> 99 Don't know |
| q504_M112c *(required)* | q504_M112c. What is the item's smallest unit of measurement (mg, mcg, ml, g, etc)? <br> *Question relevant when: selected( ${q104_M112_full_list} , '4')* | microgram — microgram (mcg) <br> miligram — miligram (mg) <br> miligram_dissolved_in_liquid — miligram (mg) dissolved in liquid (e.g. 2 mg per 3 militers) <br> gram — gram (g) <br> militer — militer (ml) <br> centiliter — centiliter (cl) <br> litre — litre (l) <br> ml_of _solution — ml of solution (e.g. ml of 10% solution) <br> litre_of_solution — litre of solution (e.g. 1 litre of 5% solution) <br> cubic_meter — cubic meter (m^3) <br> other — other <br> dont_know — don't know |
| q504_M112c.1 | q504_M112c.1 If other unit of measurement or route of administration, specify here: <br> *Leave blank if not applicable* <br> *Question relevant when: selected( ${q104_M112_full_list} , '4')* | |
| q504_M112d *(required)* | q504_M112d. In total, how many units are used to treat a patient during all of her care? <br> *Remember to add all units! E.g. 5 days x 200 mg twice per day = 2,000mg* <br> *Question relevant when: selected( ${q104_M112_full_list} , '4')* | |

*Question relevant when: selected( ${q104_M112_full_list} , '4')*

*Response constrained to: .>0*

| Field | Question | Answer |
|---|---|---|
| q504_M113 | ***q504_M113. Vitamin, immunological, etc. - Other 3: "[q104_M113_full_list_other]"***<br>*Question relevant when: selected( ${q104_M113_full_list} , '4')* | |
| q504_M113a *(required)* | q504_M113a. What percent of patients require this item?<br>*Question relevant when: selected( ${q104_M113_full_list} , '4')*<br>*Response constrained to: .>0 and .<=100 or .=999* | |
| q504_M113b *(required)* | q504_M113b. How is the item administered, or given, to the patient?<br>*Question relevant when: selected( ${q104_M113_full_list} , '4')* | 1 Oral - solid (e.g. tablet)<br>2 Oral - liquid<br>3 Sublingual<br>4 Intravenous (IV)<br>5 Intramusculuar injection (IM)<br>6 Subcutaneous<br>7 Rectal<br>8 Inhaled (e.g. gas)<br>9 Inserted (e.g. vaginal pessaries)<br>10 Topical - cream<br>11 Topical - drops (e.g. eye drops)<br>12 Other<br>99 Don't know |
| q504_M113c *(required)* | q504_M113c. What is the item's smallest unit of measurement (mg, mcg, ml, g, etc)?<br>*Question relevant when: selected( ${q104_M113_full_list} , '4')* | microgram — microgram (mcg)<br>miligram — miligram (mg)<br>miligram_dissolved_in_liquid — miligram (mg) dissolved in liquid (e.g. 2 mg per 3 militers)<br>gram — gram (g)<br>militer — militer (ml)<br>centiliter — centiliter (cl)<br>litre — litre (l)<br>ml_of _solution — ml of solution (e.g. ml of 10% solution)<br>litre_of_solution — litre of solution (e.g. 1 litre of 5% solution)<br>cubic_meter — cubic meter (m^3)<br>other — other<br>dont_know — don't know |
| q504_M113c.1 | q504_M113c.1 If other unit of measurement or route of administration, specify here:<br>*Leave blank if not applicable*<br>*Question relevant when: selected( ${q104_M113_full_list} , '4')* | |
| q504_M113d *(required)* | q504_M113d. In total, how many units are used to treat a patient during all of her care?<br>*Remember to add all units! E.g. 5 days x 200 mg twice per day = 2,000mg*<br>*Question relevant when: selected( ${q104_M113_full_list} , '4')* | |

[illegible]

*Response constrained to: .>0*

| Field | Question | Answer |
|---|---|---|
| q504_M114 | ***q504_M114. Vitamin, immunological, etc. - Other 4: "[q104_M114_full_list_other]"***<br>*Question relevant when: selected( ${q104_M114_full_list} , '4')* | |
| q504_M114a *(required)* | q504_M114a. What percent of patients require this item?<br>*Question relevant when: selected( ${q104_M114_full_list} , '4')*<br>*Response constrained to: .>0 and .<=100 or .=999* | |
| q504_M114b *(required)* | q504_M114b. How is the item administered, or given, to the patient?<br>*Question relevant when: selected( ${q104_M114_full_list} , '4')* | 1 Oral - solid (e.g. tablet)<br>2 Oral - liquid<br>3 Sublingual<br>4 Intravenous (IV)<br>5 Intramusculuar injection (IM)<br>6 Subcutaneous<br>7 Rectal<br>8 Inhaled (e.g. gas)<br>9 Inserted (e.g. vaginal pessaries)<br>10 Topical - cream<br>11 Topical - drops (e.g. eye drops)<br>12 Other<br>99 Don't know |
| q504_M114c *(required)* | q504_M114c. What is the item's smallest unit of measurement (mg, mcg, ml, g, etc)?<br>*Question relevant when: selected( ${q104_M114_full_list} , '4')* | microgram — microgram (mcg)<br>miligram — miligram (mg)<br>miligram_dissolved_in_liquid — miligram (mg) dissolved in liquid (e.g. 2 mg per 3 militers)<br>gram — gram (g)<br>militer — militer (ml)<br>centiliter — centiliter (cl)<br>litre — litre (l)<br>ml_of _solution — ml of solution (e.g. ml of 10% solution)<br>litre_of_solution — litre of solution (e.g. 1 litre of 5% solution)<br>cubic_meter — cubic meter (m^3)<br>other — other<br>dont_know — don't know |
| q504_M114c.1 | q504_M114c.1 If other unit of measurement or route of administration, specify here:<br>*Leave blank if not applicable*<br>*Question relevant when: selected( ${q104_M114_full_list} , '4')* | |
| q504_M114d *(required)* | q504_M114d. In total, how many units are used to treat a patient during all of her care?<br>*Remember to add all units! E.g. 5 days x 200 mg twice per day = 2,000mg*<br>*Question relevant when: selected( ${q104_M114_full_list} , '4')* | |

*Response constrained to: .>0*

| Field | Question | Answer |
|---|---|---|
| q504_M115 | ***q504_M115. Vitamin, immunological, etc. - Other 5: "[q104_M115_full_list_other]"***<br>*Question relevant when: selected( ${q104_M115_full_list} , '4')* | |
| q504_M115a *(required)* | q504_M115a. What percent of patients require this item?<br>*Question relevant when: selected( ${q104_M115_full_list} , '4')*<br>*Response constrained to: .>0 and .<=100 or .=999* | |
| q504_M115b *(required)* | q504_M115b. How is the item administered, or given, to the patient?<br>*Question relevant when: selected( ${q104_M115_full_list} , '4')* | 1 Oral - solid (e.g. tablet)<br>2 Oral - liquid<br>3 Sublingual<br>4 Intravenous (IV)<br>5 Intramusculuar injection (IM)<br>6 Subcutaneous<br>7 Rectal<br>8 Inhaled (e.g. gas)<br>9 Inserted (e.g. vaginal pessaries)<br>10 Topical - cream<br>11 Topical - drops (e.g. eye drops)<br>12 Other<br>99 Don't know |
| q504_M115c *(required)* | q504_M115c. What is the item's smallest unit of measurement (mg, mcg, ml, g, etc)?<br>*Question relevant when: selected( ${q104_M115_full_list} , '4')* | microgram — microgram (mcg)<br>miligram — miligram (mg)<br>miligram_dissolved_in_liquid — miligram (mg) dissolved in liquid (e.g. 2 mg per 3 militers)<br>gram — gram (g)<br>militer — militer (ml)<br>centiliter — centiliter (cl)<br>litre — litre (l)<br>ml_of _solution — ml of solution (e.g. ml of 10% solution)<br>litre_of_solution — litre of solution (e.g. 1 litre of 5% solution)<br>cubic_meter — cubic meter (m^3)<br>other — other<br>dont_know — don't know |
| q504_M115c.1 | q504_M115c.1 If other unit of measurement or route of administration, specify here:<br>*Leave blank if not applicable*<br>*Question relevant when: selected( ${q104_M115_full_list} , '4')* | |
| q504_M115d *(required)* | q504_M115d. In total, how many units are used to treat a patient during all of her care?<br>*Remember to add all units! E.g. 5 days x 200 mg twice per day = 2,000mg*<br>*Question relevant when: selected( ${q104_M115_full_list} , '4')* | |

Question relevant when: selected( ${q104_M116_full_list} , '4')

Response constrained to: .>0

| Field | Question | Answer |
|---|---|---|
| <p><strong><em>D. Medications - Lacerations (12)</em></strong></p> | | |
| *Group relevant when: selected( ${section_five_skip_med} , '1')* | | |
| q504_med_notE11 | 504 CONTINUED For each of the following medications and other medical products that are used for lacerations, can you tell me …? | |
| note_504_Other | ***Other medication, any class*** | |
| q504_M116 | ***q504_M116. Medication - any class - Other 1: "[q104_M116_full_list_other]"***<br>*Question relevant when: selected( ${q104_M116_full_list} , '4')* | |
| q504_M116a *(required)* | q504_M116a. What percent of patients require this item?<br>*Question relevant when: selected( ${q104_M116_full_list} , '4')*<br>*Response constrained to: .>0 and .<=100 or .=999* | |
| q504_M116b *(required)* | q504_M116b. How is the item administered, or given, to the patient?<br>*Question relevant when: selected( ${q104_M116_full_list} , '4')* | 1 Oral - solid (e.g. tablet)<br>2 Oral - liquid<br>3 Sublingual<br>4 Intravenous (IV)<br>5 Intramusculuar injection (IM)<br>6 Subcutaneous<br>7 Rectal<br>8 Inhaled (e.g. gas)<br>9 Inserted (e.g. vaginal pessaries)<br>10 Topical - cream<br>11 Topical - drops (e.g. eye drops)<br>12 Other<br>99 Don't know |
| q504_M116c *(required)* | q504_M116c. What is the item's smallest unit of measurement (mg, mcg, ml, g, etc)?<br>*Question relevant when: selected( ${q104_M116_full_list} , '4')* | microgram — microgram (mcg)<br>miligram — miligram (mg)<br>miligram_dissolved_in_liquid — miligram (mg) dissolved in liquid (e.g. 2 mg per 3 militers)<br>gram — gram (g)<br>militer — militer (ml)<br>centiliter — centiliter (cl)<br>litre — litre (l)<br>ml_of _solution — ml of solution (e.g. ml of 10% solution)<br>litre_of_solution — litre of solution (e.g. 1 litre of 5% solution)<br>cubic_meter — cubic meter (m^3)<br>other — other<br>dont_know — don't know |
| q504_M116c.1 | q504_M116c.1 If other unit of measurement or route of administration, specify here:<br>*Leave blank if not applicable*<br>*Question relevant when: selected( ${q104_M116_full_list} , '4')* | |
| q504_M116d *(required)* | q504_M116d. In total, how many units are used to treat a patient during all of her care?<br>*Remember to add all units! E.g. 5 days x 200 mg twice per day = 2,000mg*<br>*Question relevant when: selected( ${q104_M116_full_list} , '4')* | |

*Question relevant when: selected( ${q104_M11[illegible]_full_list} , '4')*

*Response constrained to: .>0*

| Field | Question | Answer |
|---|---|---|
| q504_M117 | ***q504_M117. Medication - any class - Other 2: "[q104_M117_full_list_other]"***<br>*Question relevant when: selected( ${q104_M117_full_list} , '4')* | |
| q504_M117a *(required)* | q504_M117a. What percent of patients require this item?<br>*Question relevant when: selected( ${q104_M117_full_list} , '4')*<br>*Response constrained to: .>0 and .<=100 or .=999* | |
| q504_M117b *(required)* | q504_M117b. How is the item administered, or given, to the patient?<br>*Question relevant when: selected( ${q104_M117_full_list} , '4')* | 1 Oral - solid (e.g. tablet)<br>2 Oral - liquid<br>3 Sublingual<br>4 Intravenous (IV)<br>5 Intramusculuar injection (IM)<br>6 Subcutaneous<br>7 Rectal<br>8 Inhaled (e.g. gas)<br>9 Inserted (e.g. vaginal pessaries)<br>10 Topical - cream<br>11 Topical - drops (e.g. eye drops)<br>12 Other<br>99 Don't know |
| q504_M117c *(required)* | q504_M117c. What is the item's smallest unit of measurement (mg, mcg, ml, g, etc)?<br>*Question relevant when: selected( ${q104_M117_full_list} , '4')* | microgram — microgram (mcg)<br>miligram — miligram (mg)<br>miligram_dissolved_in_liquid — miligram (mg) dissolved in liquid (e.g. 2 mg per 3 militers)<br>gram — gram (g)<br>militer — militer (ml)<br>centiliter — centiliter (cl)<br>litre — litre (l)<br>ml_of _solution — ml of solution (e.g. ml of 10% solution)<br>litre_of_solution — litre of solution (e.g. 1 litre of 5% solution)<br>cubic_meter — cubic meter (m^3)<br>other — other<br>dont_know — don't know |
| q504_M117c.1 | q504_M117c.1 If other unit of measurement or route of administration, specify here:<br>*Leave blank if not applicable*<br>*Question relevant when: selected( ${q104_M117_full_list} , '4')* | |
| q504_M117d *(required)* | q504_M117d. In total, how many units are used to treat a patient during all of her care?<br>*Remember to add all units! E.g. 5 days x 200 mg twice per day = 2,000mg*<br>*Question relevant when: selected( ${q104_M117_full_list} , '4')* | |

Question relevant when: selected( ${q104_M117_full_list} , '4')

| Field | Question | Answer |
|---|---|---|
| | Response constrained to: .>0 | |
| q504_M118 | ***q504_M118. Medication - any class - Other 3: "[q104_M118_full_list_other]"*** <br> *Question relevant when: selected( ${q104_M118_full_list} , '4')* | |
| q504_M118a *(required)* | q504_M118a. What percent of patients require this item? <br> *Question relevant when: selected( ${q104_M118_full_list} , '4')* <br> *Response constrained to: .>0 and .<=100 or .=999* | |
| q504_M118b *(required)* | q504_M118b. How is the item administered, or given, to the patient? <br> *Question relevant when: selected( ${q104_M118_full_list} , '4')* | 1 Oral - solid (e.g. tablet) <br> 2 Oral - liquid <br> 3 Sublingual <br> 4 Intravenous (IV) <br> 5 Intramusculuar injection (IM) <br> 6 Subcutaneous <br> 7 Rectal <br> 8 Inhaled (e.g. gas) <br> 9 Inserted (e.g. vaginal pessaries) <br> 10 Topical - cream <br> 11 Topical - drops (e.g. eye drops) <br> 12 Other <br> 99 Don't know |
| q504_M118c *(required)* | q504_M118c. What is the item's smallest unit of measurement (mg, mcg, ml, g, etc)? <br> *Question relevant when: selected( ${q104_M118_full_list} , '4')* | microgram — microgram (mcg) <br> miligram — miligram (mg) <br> miligram_dissolved_in_liquid — miligram (mg) dissolved in liquid (e.g. 2 mg per 3 militers) <br> gram — gram (g) <br> militer — militer (ml) <br> centiliter — centiliter (cl) <br> litre — litre (l) <br> ml_of _solution — ml of solution (e.g. ml of 10% solution) <br> litre_of_solution — litre of solution (e.g. 1 litre of 5% solution) <br> cubic_meter — cubic meter (m^3) <br> other — other <br> dont_know — don't know |
| q504_M118c.1 | q504_M118c.1 If other unit of measurement or route of administration, specify here: <br> *Leave blank if not applicable* <br> *Question relevant when: selected( ${q104_M118_full_list} , '4')* | |
| q504_M118d *(required)* | q504_M118d. In total, how many units are used to treat a patient during all of her care? <br> *Remember to add all units! E.g. 5 days x 200 mg twice per day = 2,000mg* <br> *Question relevant when: selected( ${q104_M118_full_list} , '4')* | |

*Question relevant when: selected( ${q104_M11[illegible]_full_list} , '4')*

*Response constrained to: .>0*

| Field | Question | Answer |
|---|---|---|
| q504_M119 | ***q504_M119. Medication - any class - Other 4: "[q104_M119_full_list_other]"***<br>*Question relevant when: selected( ${q104_M119_full_list} , '4')* | |
| q504_M119a *(required)* | q504_M119a. What percent of patients require this item?<br>*Question relevant when: selected( ${q104_M119_full_list} , '4')*<br>*Response constrained to: .>0 and .<=100 or .=999* | |
| q504_M119b *(required)* | q504_M119b. How is the item administered, or given, to the patient?<br>*Question relevant when: selected( ${q104_M119_full_list} , '4')* | 1 Oral - solid (e.g. tablet)<br>2 Oral - liquid<br>3 Sublingual<br>4 Intravenous (IV)<br>5 Intramusculuar injection (IM)<br>6 Subcutaneous<br>7 Rectal<br>8 Inhaled (e.g. gas)<br>9 Inserted (e.g. vaginal pessaries)<br>10 Topical - cream<br>11 Topical - drops (e.g. eye drops)<br>12 Other<br>99 Don't know |
| q504_M119c *(required)* | q504_M119c. What is the item's smallest unit of measurement (mg, mcg, ml, g, etc)?<br>*Question relevant when: selected( ${q104_M119_full_list} , '4')* | microgram — microgram (mcg)<br>miligram — miligram (mg)<br>miligram_dissolved_in_liquid — miligram (mg) dissolved in liquid (e.g. 2 mg per 3 militers)<br>gram — gram (g)<br>militer — militer (ml)<br>centiliter — centiliter (cl)<br>litre — litre (l)<br>ml_of _solution — ml of solution (e.g. ml of 10% solution)<br>litre_of_solution — litre of solution (e.g. 1 litre of 5% solution)<br>cubic_meter — cubic meter (m^3)<br>other — other<br>dont_know — don't know |
| q504_M119c.1 | q504_M119c.1 If other unit of measurement or route of administration, specify here:<br>*Leave blank if not applicable*<br>*Question relevant when: selected( ${q104_M119_full_list} , '4')* | |
| q504_M119d *(required)* | q504_M119d. In total, how many units are used to treat a patient during all of her care?<br>*Remember to add all units! E.g. 5 days x 200 mg twice per day = 2,000mg*<br>*Question relevant when: selected( ${q104_M119_full_list} , '4')* | |

Question relevant when: selected( ${q104_M119_full_list} , '4')

Response constrained to: .>0

| Field | Question | Answer |
|---|---|---|
| q504_M120 | ***q504_M120. Medication - any class - Other 5: "[q104_M120_full_list_other]"***<br>*Question relevant when: selected( ${q104_M120_full_list} , '4')* | |
| q504_M120a *(required)* | q504_M120a. What percent of patients require this item?<br>*Question relevant when: selected( ${q104_M120_full_list} , '4')*<br>*Response constrained to: .>0 and .<=100 or .=999* | |

| Field | Question | Answer | | |
|---|---|---|---|---|
| q504_M120b *(required)* | q504_M120b. How is the item administered, or given, to the patient?<br>*Question relevant when: selected( ${q104_M120_full_list} , '4')* | | 1 | Oral - solid (e.g. tablet) |
| | | | 2 | Oral - liquid |
| | | | 3 | Sublingual |
| | | | 4 | Intravenous (IV) |
| | | | 5 | Intramusculuar injection (IM) |
| | | | 6 | Subcutaneous |
| | | | 7 | Rectal |
| | | | 8 | Inhaled (e.g. gas) |
| | | | 9 | Inserted (e.g. vaginal pessaries) |
| | | | 10 | Topical - cream |
| | | | 11 | Topical - drops (e.g. eye drops) |
| | | | 12 | Other |
| | | | 99 | Don't know |
| q504_M120c *(required)* | q504_M120c. What is the item's smallest unit of measurement (mg, mcg, ml, g, etc)?<br>*Question relevant when: selected( ${q104_M120_full_list} , '4')* | | microgram | microgram (mcg) |
| | | | miligram | miligram (mg) |
| | | | miligram_dissolved_in_liquid | miligram (mg) dissolved in liquid (e.g. 2 mg per 3 militers) |
| | | | gram | gram (g) |
| | | | militer | militer (ml) |
| | | | centiliter | centiliter (cl) |
| | | | litre | litre (l) |
| | | | ml_of _solution | ml of solution (e.g. ml of 10% solution) |
| | | | litre_of_solution | litre of solution (e.g. 1 litre of 5% solution) |
| | | | cubic_meter | cubic meter (m^3) |
| | | | other | other |
| | | | dont_know | don't know |
| q504_M120c.1 | q504_M120c.1 If other unit of measurement or route of administration, specify here:<br>*Leave blank if not applicable*<br>*Question relevant when: selected( ${q104_M120_full_list} , '4')* | | | |
| q504_M120d *(required)* | q504_M120d. In total, how many units are used to treat a patient during all of her care?<br>*Remember to add all units! E.g. 5 days x 200 mg twice per day = 2,000mg*<br>*Question relevant when: selected( ${q104_M120_full_list} , '4')* | | | |

[illegible]
*Response constrained to: .>0*

| Field | Question | Answer |
| --- | --- | --- |
| group_section_six_intro | | |
| section6_start | **SECTION VI. PERFORATIONS - USAGE OF ALL ITEMS** | |
| section_six_skip_med | INTERVIEWER: WOULD YOU LIKE TO COMPLETE THIS SECTION NOW OR SKIP THIS SECTION AND RETURN TO IT LATER?<br>*You may need to skip if the participant has indicated that s/he cannot answer the questions in this section.* | 1 Do not skip, complete this section now.<br>2 Skip and come back to this section later. |
| group_section_six_introB<br>*Group relevant when: selected( ${section_six_skip_med} , '1')* | | |
| section6_start2 | In this section of the interview, we will review all of the items that you said are used for management of vaginal or uterine perforations. For each item that is used, I'm going to ask questions on how many women need it and how much of it is used. | |

| Field | Question | Answer |
| --- | --- | --- |
| section6_start3 | INTERVIEWER: ENTER WHOLE NUMBERS OR DECIMALS. DO NOT TYPE PERCENT SIGNS. ENTER 999 FOR ANY THAT ARE UNKNOWN. | |
| <p><strong><em>D. Medications - Perforations (1)</em></strong></p><br>*Group relevant when: selected( ${section_six_skip_med} , '1')* | | |
| q604_med_note1 | 604 For each of the following medications and other medical products that are used for perforations, can you tell me …? | |
| note_604_analgesic | ***Analgesic, anti-inflammatory, narcotic, etc.*** | |
| q604_M1 | ***q604_M1. Acetylsalicylic acid (aspirin)***<br>*Question relevant when: selected( ${q104_M1_full_list} , '5')* | |
| q604_M1a *(required)* | q604_M1a. What percent of patients require this item?<br>*Question relevant when: selected( ${q104_M1_full_list} , '5')*<br>*Response constrained to: .>0 and .<=100 or .=999* | |
| q604_M1b *(required)* | q604_M1b. How is the item administered, or given, to the patient?<br>*Question relevant when: selected( ${q104_M1_full_list} , '5')* | 1 Oral - solid (e.g. tablet)<br>2 Oral - liquid<br>3 Sublingual<br>4 Intravenous (IV)<br>5 Intramusculuar injection (IM)<br>6 Subcutaneous<br>7 Rectal<br>8 Inhaled (e.g. gas)<br>9 Inserted (e.g. vaginal pessaries)<br>10 Topical - cream<br>11 Topical - drops (e.g. eye drops)<br>12 Other<br>99 Don't know |
| q604_M1c *(required)* | q604_M1c. What is the item's smallest unit of measurement (mg, mcg, ml, g, etc)?<br>*Question relevant when: selected( ${q104_M1_full_list} , '5')* | microgram — microgram (mcg)<br>miligram — miligram (mg)<br>miligram_dissolved_in_liquid — miligram (mg) dissolved in liquid (e.g. 2 mg per 3 militers)<br>gram — gram (g)<br>militer — militer (ml)<br>centiliter — centiliter (cl)<br>litre — litre (l)<br>ml_of _solution — ml of solution (e.g. ml of 10% solution)<br>litre_of_solution — litre of solution (e.g. 1 litre of 5% |

| Field | Question | Answer |
|---|---|---|
| | | solution) |
| | | cubic_meter \| cubic meter (m^3) |
| | | other \| other |
| | | dont_know \| don't know |
| q604_M1c.1 | q604_M1c.1 If other unit of measurement or route of administration, specify here:<br>*Leave blank if not applicable*<br>*Question relevant when: selected( ${q104_M1_full_list} , '5')* | |

| | | |
|---|---|---|
| q604_M1d *(required)* | q604_M1d. In total, how many units are used to treat a patient during all of her care?<br>*Remember to add all units! E.g. 5 days x 200 mg twice per day = 2,000mg*<br>*Question relevant when: selected( ${q104_M1_full_list} , '5')*<br>*Response constrained to: .>0* | |
| q604_M2 | ***q604_M2. Paracetamol***<br>*Question relevant when: selected( ${q104_M2_full_list} , '5')* | |
| q604_M2a *(required)* | q604_M2a. What percent of patients require this item?<br>*Question relevant when: selected( ${q104_M2_full_list} , '5')*<br>*Response constrained to: .>0 and .<=100 or .=999* | |
| q604_M2b *(required)* | q604_M2b. How is the item administered, or given, to the patient?<br>*Question relevant when: selected( ${q104_M2_full_list} , '5')* | 1 Oral - solid (e.g. tablet)<br>2 Oral - liquid<br>3 Sublingual<br>4 Intravenous (IV)<br>5 Intramusculuar injection (IM)<br>6 Subcutaneous<br>7 Rectal<br>8 Inhaled (e.g. gas)<br>9 Inserted (e.g. vaginal pessaries)<br>10 Topical - cream<br>11 Topical - drops (e.g. eye drops)<br>12 Other<br>99 Don't know |
| q604_M2c *(required)* | q604_M2c. What is the item's smallest unit of measurement (mg, mcg, ml, g, etc)?<br>*Question relevant when: selected( ${q104_M2_full_list} , '5')* | microgram \| microgram (mcg)<br>miligram \| miligram (mg)<br>miligram_dissolved_in_liquid \| miligram (mg) dissolved in liquid (e.g. 2 mg per 3 militers)<br>gram \| gram (g)<br>militer \| militer (ml)<br>centiliter \| centiliter (cl)<br>litre \| litre (l)<br>ml_of _solution \| ml of solution (e.g. ml of 10% solution)<br>litre_of_solution \| litre of solution (e.g. 1 litre of 5% solution)<br>cubic_meter \| cubic meter [illegible] |

| Field | Question | Answer |
|---|---|---|
| | | (m^3)<br>other \| other<br>dont_know \| don't know |
| q604_M2c.1 | q604_M2c.1 If other unit of measurement or route of administration, specify here:<br>*Leave blank if not applicable*<br>*Question relevant when: selected( ${q104_M2_full_list} , '5')* | |
| q604_M2d *(required)* | q604_M2d. In total, how many units are used to treat a patient during all of her care?<br>*Remember to add all units! E.g. 5 days x 200 mg twice per day = 2,000mg*<br>*Question relevant when: selected( ${q104_M2_full_list} , '5')*<br>*Response constrained to: .>0* | |
| q604_M3 | ***q604_M3. Pethidine hydrochloride***<br>*Question relevant when: selected( ${q104_M3_full_list} , '5')* | |

| Field | Question | Answer |
|---|---|---|
| q604_M3a *(required)* | q604_M3a. What percent of patients require this item?<br>*Question relevant when: selected( ${q104_M3_full_list} , '5')*<br>*Response constrained to: .>0 and .<=100 or .=999* | |
| q604_M3b *(required)* | q604_M3b. How is the item administered, or given, to the patient?<br>*Question relevant when: selected( ${q104_M3_full_list} , '5')* | 1 \| Oral - solid (e.g. tablet)<br>2 \| Oral - liquid<br>3 \| Sublingual<br>4 \| Intravenous (IV)<br>5 \| Intramusculuar injection (IM)<br>6 \| Subcutaneous<br>7 \| Rectal<br>8 \| Inhaled (e.g. gas)<br>9 \| Inserted (e.g. vaginal pessaries)<br>10 \| Topical - cream<br>11 \| Topical - drops (e.g. eye drops)<br>12 \| Other<br>99 \| Don't know |
| q604_M3c *(required)* | q604_M3c. What is the item's smallest unit of measurement (mg, mcg, ml, g, etc)?<br>*Question relevant when: selected( ${q104_M3_full_list} , '5')* | microgram \| microgram (mcg)<br>miligram \| miligram (mg)<br>miligram_dissolved_in_liquid \| miligram (mg) dissolved in liquid (e.g. 2 mg per 3 militers)<br>gram \| gram (g)<br>militer \| militer (ml)<br>centiliter \| centiliter (cl)<br>litre \| litre (l)<br>ml_of _solution \| ml of solution (e.g. ml of 10% solution)<br>litre_of_solution \| litre of solution (e.g. 1 litre of 5% solution)<br>cubic_meter \| cubic meter (m^3)<br>other \| other<br>dont_know \| don't know |
| q604_M3c.1 | q604_M3c.1 If other unit of measurement or route of administration, specify here:<br>*Leave blank if not applicable*<br>[illegible] | |

| Field | Question | Answer |
|---|---|---|
| | *Question relevant when: selected( ${q104_M3_full_list} , '5')* | |
| q604_M3d *(required)* | q604_M3d. In total, how many units are used to treat a patient during all of her care?<br>*Remember to add all units! E.g. 5 days x 200 mg twice per day = 2,000mg*<br>*Question relevant when: selected( ${q104_M3_full_list} , '5')*<br>*Response constrained to: .>0* | |
| q604_M4 | ***q604_M4. Tramadol Hydrochloride***<br>*Question relevant when: selected( ${q104_M4_full_list} , '5')* | |
| q604_M4a *(required)* | q604_M4a. What percent of patients require this item?<br>*Question relevant when: selected( ${q104_M4_full_list} , '5')*<br>*Response constrained to: .>0 and .<=100 or .=999* | |
| q604_M4b *(required)* | q604_M4b. How is the item administered, or given, to the patient?<br>*Question relevant when: selected( ${q104_M4_full_list} , '5')* | 1 Oral - solid (e.g. tablet)<br>2 Oral - liquid<br>3 Sublingual<br>4 Intravenous (IV)<br>5 Intramusculuar injection (IM)<br>6 Subcutaneous<br>7 Rectal<br>8 Inhaled (e.g. gas)<br>9 Inserted (e.g. vaginal pessaries)<br>10 Topical - cream<br>11 Topical - drops (e.g. eye drops)<br>12 Other<br>99 Don't know |
| q604_M4c *(required)* | q604_M4c. What is the item's smallest unit of measurement (mg, mcg, ml, g, etc)?<br>*Question relevant when: selected( ${q104_M4_full_list} , '5')* | microgram — microgram (mcg)<br>miligram — miligram (mg)<br>miligram_dissolved_in_liquid — miligram (mg) dissolved in liquid (e.g. 2 mg per 3 militers)<br>gram — gram (g)<br>militer — militer (ml)<br>centiliter — centiliter (cl)<br>litre — litre (l)<br>ml_of _solution — ml of solution (e.g. ml of 10% solution)<br>litre_of_solution — litre of solution (e.g. 1 litre of 5% solution)<br>cubic_meter — cubic meter (m^3)<br>other — other<br>dont_know — don't know |
| q604_M4c.1 | q604_M4c.1 If other unit of measurement or route of administration, specify here:<br>*Leave blank if not applicable*<br>*Question relevant when: selected( ${q104_M4_full_list} , '5')* | |
| q604_M4d *(required)* | q604_M4d. In total, how many units are used to treat a patient during all of her care?<br>*Remember to add all units! E.g. 5 days x 200 mg twice per day = 2,000mg*<br>*Question relevant when: selected( ${q104_M4_full_list} , '5')* | |

*Question relevant when: selected( ${q104_M[illegible]_full_list} , '[illegible]')*

*Response constrained to: .>0*

| Field | Question | Answer |
|---|---|---|
| q604_M5 | ***q604_M5. Ibuprofen***<br>*Question relevant when: selected( ${q104_M5_full_list} , '5')* | |
| q604_M5a *(required)* | q604_M5a. What percent of patients require this item?<br>*Question relevant when: selected( ${q104_M5_full_list} , '5')*<br>*Response constrained to: .>0 and .<=100 or .=999* | |
| q604_M5b *(required)* | q604_M5b. How is the item administered, or given, to the patient?<br>*Question relevant when: selected( ${q104_M5_full_list} , '5')* | 1 Oral - solid (e.g. tablet)<br>2 Oral - liquid<br>3 Sublingual<br>4 Intravenous (IV)<br>5 Intramusculuar injection (IM)<br>6 Subcutaneous<br>7 Rectal<br>8 Inhaled (e.g. gas)<br>9 Inserted (e.g. vaginal pessaries)<br>10 Topical - cream<br>11 Topical - drops (e.g. eye drops)<br>12 Other<br>99 Don't know |
| q604_M5c *(required)* | q604_M5c. What is the item's smallest unit of measurement (mg, mcg, ml, g, etc)?<br>*Question relevant when: selected( ${q104_M5_full_list} , '5')* | microgram — microgram (mcg)<br>miligram — miligram (mg)<br>miligram_dissolved_in_liquid — miligram (mg) dissolved in liquid (e.g. 2 mg per 3 militers)<br>gram — gram (g)<br>militer — militer (ml)<br>centiliter — centiliter (cl)<br>litre — litre (l)<br>ml_of _solution — ml of solution (e.g. ml of 10% solution)<br>litre_of_solution — litre of solution (e.g. 1 litre of 5% solution)<br>cubic_meter — cubic meter (m^3)<br>other — other<br>dont_know — don't know |
| q604_M5c.1 | q604_M5c.1 If other unit of measurement or route of administration, specify here:<br>*Leave blank if not applicable*<br>*Question relevant when: selected( ${q104_M5_full_list} , '5')* | |
| q604_M5d *(required)* | q604_M5d. In total, how many units are used to treat a patient during all of her care?<br>*Remember to add all units! E.g. 5 days x 200 mg twice per day = 2,000mg*<br>*Question relevant when: selected( ${q104_M5_full_list} , '5')* | |

[illegible]

*Response constrained to: .>0*

| Field | Question | Answer |
|---|---|---|
| q604_M6 | ***q604_M6. Diclofenac***<br>*Question relevant when: selected( ${q104_M6_full_list} , '5')* | |
| q604_M6a *(required)* | q604_M6a. What percent of patients require this item?<br>*Question relevant when: selected( ${q104_M6_full_list} , '5')*<br>*Response constrained to: .>0 and .<=100 or .=999* | |
| q604_M6b *(required)* | q604_M6b. How is the item administered, or given, to the patient?<br>*Question relevant when: selected( ${q104_M6_full_list} , '5')* | 1 Oral - solid (e.g. tablet)<br>2 Oral - liquid<br>3 Sublingual<br>4 Intravenous (IV)<br>5 Intramusculuar injection (IM)<br>6 Subcutaneous<br>7 Rectal<br>8 Inhaled (e.g. gas)<br>9 Inserted (e.g. vaginal pessaries)<br>10 Topical - cream<br>11 Topical - drops (e.g. eye drops)<br>12 Other<br>99 Don't know |
| q604_M6c *(required)* | q604_M6c. What is the item's smallest unit of measurement (mg, mcg, ml, g, etc)?<br>*Question relevant when: selected( ${q104_M6_full_list} , '5')* | microgram — microgram (mcg)<br>miligram — miligram (mg)<br>miligram_dissolved_in_liquid — miligram (mg) dissolved in liquid (e.g. 2 mg per 3 militers)<br>gram — gram (g)<br>militer — militer (ml)<br>centiliter — centiliter (cl)<br>litre — litre (l)<br>ml_of _solution — ml of solution (e.g. ml of 10% solution)<br>litre_of_solution — litre of solution (e.g. 1 litre of 5% solution)<br>cubic_meter — cubic meter (m^3)<br>other — other<br>dont_know — don't know |
| q604_M6c.1 | q604_M6c.1 If other unit of measurement or route of administration, specify here:<br>*Leave blank if not applicable*<br>*Question relevant when: selected( ${q104_M6_full_list} , '5')* | |
| q604_M6d *(required)* | q604_M6d. In total, how many units are used to treat a patient during all of her care?<br>*Remember to add all units! E.g. 5 days x 200 mg twice per day = 2,000mg*<br>*Question relevant when: selected( ${q104_M6_full_list} , '5')* | |

*Question relevant when: selected( ${q10[illegible]_full_list} , '5')*

*Response constrained to: .>0*

| Field | Question | Answer |
|---|---|---|
| q604_M7 | ***q604_M7. Diclofenac sodium***<br>*Question relevant when: selected( ${q104_M7_full_list} , '5')* | |
| q604_M7a *(required)* | q604_M7a. What percent of patients require this item?<br>*Question relevant when: selected( ${q104_M7_full_list} , '5')*<br>*Response constrained to: .>0 and .<=100 or .=999* | |
| q604_M7b *(required)* | q604_M7b. How is the item administered, or given, to the patient?<br>*Question relevant when: selected( ${q104_M7_full_list} , '5')* | 1 Oral - solid (e.g. tablet)<br>2 Oral - liquid<br>3 Sublingual<br>4 Intravenous (IV)<br>5 Intramusculuar injection (IM)<br>6 Subcutaneous<br>7 Rectal<br>8 Inhaled (e.g. gas)<br>9 Inserted (e.g. vaginal pessaries)<br>10 Topical - cream<br>11 Topical - drops (e.g. eye drops)<br>12 Other<br>99 Don't know |
| q604_M7c *(required)* | q604_M7c. What is the item's smallest unit of measurement (mg, mcg, ml, g, etc)?<br>*Question relevant when: selected( ${q104_M7_full_list} , '5')* | microgram — microgram (mcg)<br>miligram — miligram (mg)<br>miligram_dissolved_in_liquid — miligram (mg) dissolved in liquid (e.g. 2 mg per 3 militers)<br>gram — gram (g)<br>militer — militer (ml)<br>centiliter — centiliter (cl)<br>litre — litre (l)<br>ml_of _solution — ml of solution (e.g. ml of 10% solution)<br>litre_of_solution — litre of solution (e.g. 1 litre of 5% solution)<br>cubic_meter — cubic meter (m^3)<br>other — other<br>dont_know — don't know |
| q604_M7c.1 | q604_M7c.1 If other unit of measurement or route of administration, specify here:<br>*Leave blank if not applicable*<br>*Question relevant when: selected( ${q104_M7_full_list} , '5')* | |
| q604_M7d *(required)* | q604_M7d. In total, how many units are used to treat a patient during all of her care?<br>*Remember to add all units! E.g. 5 days x 200 mg twice per day = 2,000mg*<br>*Question relevant when: selected( ${q104_M7_full_list} , '5')* | |

*Question relevant when: selected( ${q104_M[illegible]_full_list} , '5')*

*Response constrained to: .>0*

| Field | Question | Answer |
|---|---|---|
| q604_M8 | ***q604_M8. Dypron (dipyrone), 250mg/ml (Metamizole)***<br>*Question relevant when: selected( ${q104_M8_full_list} , '5')* | |
| q604_M8a *(required)* | q604_M8a. What percent of patients require this item?<br>*Question relevant when: selected( ${q104_M8_full_list} , '5')*<br>*Response constrained to: .>0 and .<=100 or .=999* | |
| q604_M8b *(required)* | q604_M8b. How is the item administered, or given, to the patient?<br>*Question relevant when: selected( ${q104_M8_full_list} , '5')* | 1 Oral - solid (e.g. tablet)<br>2 Oral - liquid<br>3 Sublingual<br>4 Intravenous (IV)<br>5 Intramusculuar injection (IM)<br>6 Subcutaneous<br>7 Rectal<br>8 Inhaled (e.g. gas)<br>9 Inserted (e.g. vaginal pessaries)<br>10 Topical - cream<br>11 Topical - drops (e.g. eye drops)<br>12 Other<br>99 Don't know |
| q604_M8c *(required)* | q604_M8c. What is the item's smallest unit of measurement (mg, mcg, ml, g, etc)?<br>*Question relevant when: selected( ${q104_M8_full_list} , '5')* | microgram — microgram (mcg)<br>miligram — miligram (mg)<br>miligram_dissolved_in_liquid — miligram (mg) dissolved in liquid (e.g. 2 mg per 3 militers)<br>gram — gram (g)<br>militer — militer (ml)<br>centiliter — centiliter (cl)<br>litre — litre (l)<br>ml_of _solution — ml of solution (e.g. ml of 10% solution)<br>litre_of_solution — litre of solution (e.g. 1 litre of 5% solution)<br>cubic_meter — cubic meter (m^3)<br>other — other<br>dont_know — don't know |
| q604_M8c.1 | q604_M8c.1 If other unit of measurement or route of administration, specify here:<br>*Leave blank if not applicable*<br>*Question relevant when: selected( ${q104_M8_full_list} , '5')* | |
| q604_M8d *(required)* | q604_M8d. In total, how many units are used to treat a patient during all of her care?<br>*Remember to add all units! E.g. 5 days x 200 mg twice per day = 2,000mg*<br>*Question relevant when: selected( ${q104_M8_full_list} , '5')* | |

Question relevant when: selected( ${q104_M[illegible]_full_list} , '5')

Response constrained to: .>0

| Field | Question | Answer |
|---|---|---|
| q604_M9 | ***q604_M9. Fentanyl citrate, 50 mcg/ml***<br>*Question relevant when: selected( ${q104_M9_full_list} , '5')* | |
| q604_M9a *(required)* | q604_M9a. What percent of patients require this item?<br>*Question relevant when: selected( ${q104_M9_full_list} , '5')*<br>*Response constrained to: .>0 and .<=100 or .=999* | |
| q604_M9b *(required)* | q604_M9b. How is the item administered, or given, to the patient?<br>*Question relevant when: selected( ${q104_M9_full_list} , '5')* | 1 Oral - solid (e.g. tablet)<br>2 Oral - liquid<br>3 Sublingual<br>4 Intravenous (IV)<br>5 Intramusculuar injection (IM)<br>6 Subcutaneous<br>7 Rectal<br>8 Inhaled (e.g. gas)<br>9 Inserted (e.g. vaginal pessaries)<br>10 Topical - cream<br>11 Topical - drops (e.g. eye drops)<br>12 Other<br>99 Don't know |
| q604_M9c *(required)* | q604_M9c. What is the item's smallest unit of measurement (mg, mcg, ml, g, etc)?<br>*Question relevant when: selected( ${q104_M9_full_list} , '5')* | microgram — microgram (mcg)<br>miligram — miligram (mg)<br>miligram_dissolved_in_liquid — miligram (mg) dissolved in liquid (e.g. 2 mg per 3 militers)<br>gram — gram (g)<br>militer — militer (ml)<br>centiliter — centiliter (cl)<br>litre — litre (l)<br>ml_of _solution — ml of solution (e.g. ml of 10% solution)<br>litre_of_solution — litre of solution (e.g. 1 litre of 5% solution)<br>cubic_meter — cubic meter (m^3)<br>other — other<br>dont_know — don't know |
| q604_M9c.1 | q604_M9c.1 If other unit of measurement or route of administration, specify here:<br>*Leave blank if not applicable*<br>*Question relevant when: selected( ${q104_M9_full_list} , '5')* | |
| q604_M9d *(required)* | q604_M9d. In total, how many units are used to treat a patient during all of her care?<br>*Remember to add all units! E.g. 5 days x 200 mg twice per day = 2,000mg*<br>*Question relevant when: selected( ${q104_M9_full_list} , '5')* | |

| Field | Question | Answer |
|---|---|---|
| | *Question relevant when: selected( ${q104_[illegible]_full_list} , '5')*<br>*Response constrained to: .>0* | |
| q604_M10 | ***q604_M10. Analgesic - other 1: "[q104_M10_full_list_other]"***<br>*Question relevant when: selected( ${q104_M10_full_list} , '5')* | |
| q604_M10a *(required)* | q604_M10a. What percent of patients require this item?<br>*Question relevant when: selected( ${q104_M10_full_list} , '5')*<br>*Response constrained to: .>0 and .<=100 or .=999* | |
| q604_M10b *(required)* | q604_M10b. How is the item administered, or given, to the patient?<br>*Question relevant when: selected( ${q104_M10_full_list} , '5')* | 1 Oral - solid (e.g. tablet)<br>2 Oral - liquid<br>3 Sublingual<br>4 Intravenous (IV)<br>5 Intramusculuar injection (IM)<br>6 Subcutaneous<br>7 Rectal<br>8 Inhaled (e.g. gas)<br>9 Inserted (e.g. vaginal pessaries)<br>10 Topical - cream<br>11 Topical - drops (e.g. eye drops)<br>12 Other<br>99 Don't know |
| q604_M10c *(required)* | q604_M10c. What is the item's smallest unit of measurement (mg, mcg, ml, g, etc)?<br>*Question relevant when: selected( ${q104_M10_full_list} , '5')* | microgram — microgram (mcg)<br>miligram — miligram (mg)<br>miligram_dissolved_in_liquid — miligram (mg) dissolved in liquid (e.g. 2 mg per 3 militers)<br>gram — gram (g)<br>militer — militer (ml)<br>centiliter — centiliter (cl)<br>litre — litre (l)<br>ml_of _solution — ml of solution (e.g. ml of 10% solution)<br>litre_of_solution — litre of solution (e.g. 1 litre of 5% solution)<br>cubic_meter — cubic meter (m^3)<br>other — other<br>dont_know — don't know |
| q604_M10c.1 | q604_M10c.1 If other unit of measurement or route of administration, specify here:<br>*Leave blank if not applicable*<br>*Question relevant when: selected( ${q104_M10_full_list} , '5')* | |
| q604_M10d *(required)* | q604_M10d. In total, how many units are used to treat a patient during all of her care?<br>*Remember to add all units! E.g. 5 days x 200 mg twice per day = 2,000mg*<br>*Question relevant when: selected( ${q104_M10_full_list} , '5')* | |

*Question relevant when: selected( ${q104_M1[illegible]_full_list} , '5')*
*Response constrained to: .>0*

| Field | Question | Answer |
|---|---|---|
| q604_M11 | ***q604_M11. Analgesic - other 2: "[q104_M11_full_list_other]"***<br>*Question relevant when: selected( ${q104_M11_full_list} , '5')* | |
| q604_M11a *(required)* | q604_M11a. What percent of patients require this item?<br>*Question relevant when: selected( ${q104_M11_full_list} , '5')*<br>*Response constrained to: .>0 and .<=100 or .=999* | |
| q604_M11b *(required)* | q604_M11b. How is the item administered, or given, to the patient?<br>*Question relevant when: selected( ${q104_M11_full_list} , '5')* | 1 Oral - solid (e.g. tablet)<br>2 Oral - liquid<br>3 Sublingual<br>4 Intravenous (IV)<br>5 Intramusculuar injection (IM)<br>6 Subcutaneous<br>7 Rectal<br>8 Inhaled (e.g. gas)<br>9 Inserted (e.g. vaginal pessaries)<br>10 Topical - cream<br>11 Topical - drops (e.g. eye drops)<br>12 Other<br>99 Don't know |
| q604_M11c *(required)* | q604_M11c. What is the item's smallest unit of measurement (mg, mcg, ml, g, etc)?<br>*Question relevant when: selected( ${q104_M11_full_list} , '5')* | microgram – microgram (mcg)<br>miligram – miligram (mg)<br>miligram_dissolved_in_liquid – miligram (mg) dissolved in liquid (e.g. 2 mg per 3 militers)<br>gram – gram (g)<br>militer – militer (ml)<br>centiliter – centiliter (cl)<br>litre – litre (l)<br>ml_of _solution – ml of solution (e.g. ml of 10% solution)<br>litre_of_solution – litre of solution (e.g. 1 litre of 5% solution)<br>cubic_meter – cubic meter (m^3)<br>other – other<br>dont_know – don't know |
| q604_M11c.1 | q604_M11c.1 If other unit of measurement or route of administration, specify here:<br>*Leave blank if not applicable*<br>*Question relevant when: selected( ${q104_M11_full_list} , '5')* | |
| q604_M11d *(required)* | q604_M11d. In total, how many units are used to treat a patient during all of her care?<br>*Remember to add all units! E.g. 5 days x 200 mg twice per day = 2,000mg*<br>*Question relevant when: selected( ${q104_M11_full_list} , '5')* | |

*Question relevant when: selected( ${q104_M11_full_list} , '5')*

*Response constrained to: .>0*

| Field | Question | Answer |
|---|---|---|
| q604_M12 | ***q604_M12. Analgesic - other 3: "[q104_M12_full_list_other]"***<br>*Question relevant when: selected( ${q104_M12_full_list} , '5')* | |
| q604_M12a *(required)* | q604_M12a. What percent of patients require this item?<br>*Question relevant when: selected( ${q104_M12_full_list} , '5')*<br>*Response constrained to: .>0 and .<=100 or .=999* | |
| q604_M12b *(required)* | q604_M12b. How is the item administered, or given, to the patient?<br>*Question relevant when: selected( ${q104_M12_full_list} , '5')* | 1 Oral - solid (e.g. tablet)<br>2 Oral - liquid<br>3 Sublingual<br>4 Intravenous (IV)<br>5 Intramusculuar injection (IM)<br>6 Subcutaneous<br>7 Rectal<br>8 Inhaled (e.g. gas)<br>9 Inserted (e.g. vaginal pessaries)<br>10 Topical - cream<br>11 Topical - drops (e.g. eye drops)<br>12 Other<br>99 Don't know |
| q604_M12c *(required)* | q604_M12c. What is the item's smallest unit of measurement (mg, mcg, ml, g, etc)?<br>*Question relevant when: selected( ${q104_M12_full_list} , '5')* | microgram — microgram (mcg)<br>miligram — miligram (mg)<br>miligram_dissolved_in_liquid — miligram (mg) dissolved in liquid (e.g. 2 mg per 3 militers)<br>gram — gram (g)<br>militer — militer (ml)<br>centiliter — centiliter (cl)<br>litre — litre (l)<br>ml_of _solution — ml of solution (e.g. ml of 10% solution)<br>litre_of_solution — litre of solution (e.g. 1 litre of 5% solution)<br>cubic_meter — cubic meter (m^3)<br>other — other<br>dont_know — don't know |
| q604_M12c.1 | q604_M12c.1 If other unit of measurement or route of administration, specify here:<br>*Leave blank if not applicable*<br>*Question relevant when: selected( ${q104_M12_full_list} , '5')* | |
| q604_M12d *(required)* | q604_M12d. In total, how many units are used to treat a patient during all of her care?<br>*Remember to add all units! E.g. 5 days x 200 mg twice per day = 2,000mg*<br>*Question relevant when: selected( ${q104_M12_full_list} , '5')* | |

*Question relevant when: selected( ${q104_M12_full_list} , '5')*

*Response constrained to: .>0*

| Field | Question | Answer |
|---|---|---|
| q604_M13 | ***q604_M13. Analgesic - other 4: "[q104_M13_full_list_other]"***<br>*Question relevant when: selected( ${q104_M13_full_list} , '5')* | |
| q604_M13a *(required)* | q604_M13a. What percent of patients require this item?<br>*Question relevant when: selected( ${q104_M13_full_list} , '5')*<br>*Response constrained to: .>0 and .<=100 or .=999* | |
| q604_M13b *(required)* | q604_M13b. How is the item administered, or given, to the patient?<br>*Question relevant when: selected( ${q104_M13_full_list} , '5')* | 1 Oral - solid (e.g. tablet)<br>2 Oral - liquid<br>3 Sublingual<br>4 Intravenous (IV)<br>5 Intramusculuar injection (IM)<br>6 Subcutaneous<br>7 Rectal<br>8 Inhaled (e.g. gas)<br>9 Inserted (e.g. vaginal pessaries)<br>10 Topical - cream<br>11 Topical - drops (e.g. eye drops)<br>12 Other<br>99 Don't know |
| q604_M13c *(required)* | q604_M13c. What is the item's smallest unit of measurement (mg, mcg, ml, g, etc)?<br>*Question relevant when: selected( ${q104_M13_full_list} , '5')* | microgram — microgram (mcg)<br>miligram — miligram (mg)<br>miligram_dissolved_in_liquid — miligram (mg) dissolved in liquid (e.g. 2 mg per 3 militers)<br>gram — gram (g)<br>militer — militer (ml)<br>centiliter — centiliter (cl)<br>litre — litre (l)<br>ml_of _solution — ml of solution (e.g. ml of 10% solution)<br>litre_of_solution — litre of solution (e.g. 1 litre of 5% solution)<br>cubic_meter — cubic meter (m^3)<br>other — other<br>dont_know — don't know |
| q604_M13c.1 | q604_M13c.1 If other unit of measurement or route of administration, specify here:<br>*Leave blank if not applicable*<br>*Question relevant when: selected( ${q104_M13_full_list} , '5')* | |
| q604_M13d *(required)* | q604_M13d. In total, how many units are used to treat a patient during all of her care?<br>*Remember to add all units! E.g. 5 days x 200 mg twice per day = 2,000mg*<br>*Question relevant when: selected( ${q104_M13_full_list} , '5')* | |

*Question relevant when: selected( ${q104_M1[illegible]_full_list} , '5')*

*Response constrained to: .>0*

| Field | Question | Answer |
|---|---|---|
| q604_M14 | ***q604_M14. Analgesic - other 5: "[q104_M14_full_list_other]"***<br>*Question relevant when: selected( ${q104_M14_full_list} , '5')* | |
| q604_M14a *(required)* | q604_M14a. What percent of patients require this item?<br>*Question relevant when: selected( ${q104_M14_full_list} , '5')*<br>*Response constrained to: .>0 and .<=100 or .=999* | |
| q604_M14b *(required)* | q604_M14b. How is the item administered, or given, to the patient?<br>*Question relevant when: selected( ${q104_M14_full_list} , '5')* | 1 Oral - solid (e.g. tablet)<br>2 Oral - liquid<br>3 Sublingual<br>4 Intravenous (IV)<br>5 Intramusculuar injection (IM)<br>6 Subcutaneous<br>7 Rectal<br>8 Inhaled (e.g. gas)<br>9 Inserted (e.g. vaginal pessaries)<br>10 Topical - cream<br>11 Topical - drops (e.g. eye drops)<br>12 Other<br>99 Don't know |
| q604_M14c *(required)* | q604_M14c. What is the item's smallest unit of measurement (mg, mcg, ml, g, etc)?<br>*Question relevant when: selected( ${q104_M14_full_list} , '5')* | microgram — microgram (mcg)<br>miligram — miligram (mg)<br>miligram_dissolved_in_liquid — miligram (mg) dissolved in liquid (e.g. 2 mg per 3 militers)<br>gram — gram (g)<br>militer — militer (ml)<br>centiliter — centiliter (cl)<br>litre — litre (l)<br>ml_of _solution — ml of solution (e.g. ml of 10% solution)<br>litre_of_solution — litre of solution (e.g. 1 litre of 5% solution)<br>cubic_meter — cubic meter (m^3)<br>other — other<br>dont_know — don't know |
| q604_M14c.1 | q604_M14c.1 If other unit of measurement or route of administration, specify here:<br>*Leave blank if not applicable*<br>*Question relevant when: selected( ${q104_M14_full_list} , '5')* | |
| q604_M14d *(required)* | q604_M14d. In total, how many units are used to treat a patient during all of her care?<br>*Remember to add all units! E.g. 5 days x 200 mg twice per day = 2,000mg*<br>*Question relevant when: selected( ${q104_M14_full_list} , '5')* | |

*Question relevant when: selected( ${q104_M[illegible]_full_list} , '5')*
*Response constrained to: .>0*

| Field | Question | Answer |
|---|---|---|
| <p><strong><em>D. Medications - Perforations (2)</em></strong></p> | | |
| *Group relevant when: selected( ${section_six_skip_med} , '1')* | | |
| q604_med_note2 | 604 For each of the following medications and other medical products that are used for perforations, can you tell me …? | |
| note_604_anesthetic | ***Anesthetic - local, general, etc. (and oxygen)*** | |
| q604_M15 | ***q604_M15. Halothane***<br>*Question relevant when: selected( ${q104_M15_full_list} , '5')* | |
| q604_M15a *(required)* | q604_M15a. What percent of patients require this item?<br>*Question relevant when: selected( ${q104_M15_full_list} , '5')*<br>*Response constrained to: .>0 and .<=100 or .=999* | |
| q604_M15b *(required)* | q604_M15b. How is the item administered, or given, to the patient?<br>*Question relevant when: selected( ${q104_M15_full_list} , '5')* | 1 Oral - solid (e.g. tablet)<br>2 Oral - liquid<br>3 Sublingual<br>4 Intravenous (IV)<br>5 Intramusculuar injection (IM)<br>6 Subcutaneous<br>7 Rectal<br>8 Inhaled (e.g. gas)<br>9 Inserted (e.g. vaginal pessaries)<br>10 Topical - cream<br>11 Topical - drops (e.g. eye drops)<br>12 Other<br>99 Don't know |
| q604_M15c *(required)* | q604_M15c. What is the item's smallest unit of measurement (mg, mcg, ml, g, etc)?<br>*Question relevant when: selected( ${q104_M15_full_list} , '5')* | microgram — microgram (mcg)<br>miligram — miligram (mg)<br>miligram_dissolved_in_liquid — miligram (mg) dissolved in liquid (e.g. 2 mg per 3 militers)<br>gram — gram (g)<br>militer — militer (ml)<br>centiliter — centiliter (cl)<br>litre — litre (l)<br>ml_of _solution — ml of solution (e.g. ml of 10% solution)<br>litre_of_solution — litre of solution (e.g. 1 litre of 5% solution)<br>cubic_meter — cubic meter (m^3)<br>other — other<br>dont_know — don't know |
| q604_M15c.1 | q604_M15c.1 If other unit of measurement or route of administration, specify here:<br>*Leave blank if not applicable*<br>*Question relevant when: selected( ${q104_M15_full_list} , '5')* | |
| q604_M15d *(required)* | q604_M15d. In total, how many units are used to treat a patient during all of her care?<br>*Remember to add all units! E.g. 5 days x 200 mg twice per day = 2,000mg*<br>*Question relevant when: selected( ${q104_M15_full_list} , '5')* | |

*Question relevant when: selected( ${q10[illegible]_full_list} , '5')*

*Response constrained to: .>0*

| Field | Question | Answer |
|---|---|---|
| q604_M16 | ***q604_M16. Ketamine (Ketalar), 50mg/ml***<br>*Question relevant when: selected( ${q104_M16_full_list} , '5')* | |
| q604_M16a *(required)* | q604_M16a. What percent of patients require this item?<br>*Question relevant when: selected( ${q104_M16_full_list} , '5')*<br>*Response constrained to: .>0 and .<=100 or .=999* | |
| q604_M16b *(required)* | q604_M16b. How is the item administered, or given, to the patient?<br>*Question relevant when: selected( ${q104_M16_full_list} , '5')* | 1 Oral - solid (e.g. tablet)<br>2 Oral - liquid<br>3 Sublingual<br>4 Intravenous (IV)<br>5 Intramusculuar injection (IM)<br>6 Subcutaneous<br>7 Rectal<br>8 Inhaled (e.g. gas)<br>9 Inserted (e.g. vaginal pessaries)<br>10 Topical - cream<br>11 Topical - drops (e.g. eye drops)<br>12 Other<br>99 Don't know |
| q604_M16c *(required)* | q604_M16c. What is the item's smallest unit of measurement (mg, mcg, ml, g, etc)?<br>*Question relevant when: selected( ${q104_M16_full_list} , '5')* | microgram — microgram (mcg)<br>miligram — miligram (mg)<br>miligram_dissolved_in_liquid — miligram (mg) dissolved in liquid (e.g. 2 mg per 3 militers)<br>gram — gram (g)<br>militer — militer (ml)<br>centiliter — centiliter (cl)<br>litre — litre (l)<br>ml_of _solution — ml of solution (e.g. ml of 10% solution)<br>litre_of_solution — litre of solution (e.g. 1 litre of 5% solution)<br>cubic_meter — cubic meter (m^3)<br>other — other<br>dont_know — don't know |
| q604_M16c.1 | q604_M16c.1 If other unit of measurement or route of administration, specify here:<br>*Leave blank if not applicable*<br>*Question relevant when: selected( ${q104_M16_full_list} , '5')* | |
| q604_M16d *(required)* | q604_M16d. In total, how many units are used to treat a patient during all of her care?<br>*Remember to add all units! E.g. 5 days x 200 mg twice per day = 2,000mg*<br>*Question relevant when: selected( ${q104_M16_full_list} , '5')* | |

Question relevant when: selected( ${q104_M16_full_list} , '5')

Response constrained to: .>0

| Field | Question | Answer |
|---|---|---|
| q604_M17 | ***q604_M17. Propofol (DiprVan), 10mg/ml***<br>*Question relevant when: selected( ${q104_M17_full_list} , '5')* | |
| q604_M17a *(required)* | q604_M17a. What percent of patients require this item?<br>*Question relevant when: selected( ${q104_M17_full_list} , '5')*<br>*Response constrained to: .>0 and .<=100 or .=999* | |
| q604_M17b *(required)* | q604_M17b. How is the item administered, or given, to the patient?<br>*Question relevant when: selected( ${q104_M17_full_list} , '5')* | 1 Oral - solid (e.g. tablet)<br>2 Oral - liquid<br>3 Sublingual<br>4 Intravenous (IV)<br>5 Intramusculuar injection (IM)<br>6 Subcutaneous<br>7 Rectal<br>8 Inhaled (e.g. gas)<br>9 Inserted (e.g. vaginal pessaries)<br>10 Topical - cream<br>11 Topical - drops (e.g. eye drops)<br>12 Other<br>99 Don't know |
| q604_M17c *(required)* | q604_M17c. What is the item's smallest unit of measurement (mg, mcg, ml, g, etc)?<br>*Question relevant when: selected( ${q104_M17_full_list} , '5')* | microgram — microgram (mcg)<br>miligram — miligram (mg)<br>miligram_dissolved_in_liquid — miligram (mg) dissolved in liquid (e.g. 2 mg per 3 militers)<br>gram — gram (g)<br>militer — militer (ml)<br>centiliter — centiliter (cl)<br>litre — litre (l)<br>ml_of _solution — ml of solution (e.g. ml of 10% solution)<br>litre_of_solution — litre of solution (e.g. 1 litre of 5% solution)<br>cubic_meter — cubic meter (m^3)<br>other — other<br>dont_know — don't know |
| q604_M17c.1 | q604_M17c.1 If other unit of measurement or route of administration, specify here:<br>*Leave blank if not applicable*<br>*Question relevant when: selected( ${q104_M17_full_list} , '5')* | |
| q604_M17d *(required)* | q604_M17d. In total, how many units are used to treat a patient during all of her care?<br>*Remember to add all units! E.g. 5 days x 200 mg twice per day = 2,000mg*<br>*Question relevant when: selected( ${q104_M17_full_list} , '5')* | |

| Field | Question | Answer |
|---|---|---|
| | Question relevant when: selected( ${q104_M17_full_list} , '5')<br>*Response constrained to: .>0* | |
| q604_M18 | ***q604_M18. Suxamethonium chloride***<br>*Question relevant when: selected( ${q104_M18_full_list} , '5')* | |
| q604_M18a *(required)* | q604_M18a. What percent of patients require this item?<br>*Question relevant when: selected( ${q104_M18_full_list} , '5')*<br>*Response constrained to: .>0 and .<=100 or .=999* | |
| q604_M18b *(required)* | q604_M18b. How is the item administered, or given, to the patient?<br>*Question relevant when: selected( ${q104_M18_full_list} , '5')* | 1 Oral - solid (e.g. tablet)<br>2 Oral - liquid<br>3 Sublingual<br>4 Intravenous (IV)<br>5 Intramusculuar injection (IM)<br>6 Subcutaneous<br>7 Rectal<br>8 Inhaled (e.g. gas)<br>9 Inserted (e.g. vaginal pessaries)<br>10 Topical - cream<br>11 Topical - drops (e.g. eye drops)<br>12 Other<br>99 Don't know |
| q604_M18c *(required)* | q604_M18c. What is the item's smallest unit of measurement (mg, mcg, ml, g, etc)?<br>*Question relevant when: selected( ${q104_M18_full_list} , '5')* | microgram — microgram (mcg)<br>miligram — miligram (mg)<br>miligram_dissolved_in_liquid — miligram (mg) dissolved in liquid (e.g. 2 mg per 3 militers)<br>gram — gram (g)<br>militer — militer (ml)<br>centiliter — centiliter (cl)<br>litre — litre (l)<br>ml_of _solution — ml of solution (e.g. ml of 10% solution)<br>litre_of_solution — litre of solution (e.g. 1 litre of 5% solution)<br>cubic_meter — cubic meter (m^3)<br>other — other<br>dont_know — don't know |
| q604_M18c.1 | q604_M18c.1 If other unit of measurement or route of administration, specify here:<br>*Leave blank if not applicable*<br>*Question relevant when: selected( ${q104_M18_full_list} , '5')* | |
| q604_M18d *(required)* | q604_M18d. In total, how many units are used to treat a patient during all of her care? | |

| Field | Question | Answer |
|---|---|---|
| | *Remember to add all units! E.g. 5 days x 200 mg twice per day = 2,000mg*<br>*Question relevant when: selected( ${q104_M18_full_list} , '5')*<br>*Response constrained to: .>0* | |
| q604_M19 | ***q604_M19. Thiopental sodium (Intraval)***<br>*Question relevant when: selected( ${q104_M19_full_list} , '5')* | |
| q604_M19a *(required)* | q604_M19a. What percent of patients require this item?<br>*Question relevant when: selected( ${q104_M19_full_list} , '5')*<br>*Response constrained to: .>0 and .<=100 or .=999* | |
| q604_M19b *(required)* | q604_M19b. How is the item administered, or given, to the patient?<br>*Question relevant when: selected( ${q104_M19_full_list} , '5')* | 1 Oral - solid (e.g. tablet)<br>2 Oral - liquid<br>3 Sublingual<br>4 Intravenous (IV)<br>5 Intramusculuar injection (IM)<br>6 Subcutaneous<br>7 Rectal<br>8 Inhaled (e.g. gas)<br>9 Inserted (e.g. vaginal pessaries)<br>10 Topical - cream<br>11 Topical - drops (e.g. eye drops)<br>12 Other<br>99 Don't know |
| q604_M19c *(required)* | q604_M19c. What is the item's smallest unit of measurement (mg, mcg, ml, g, etc)?<br>*Question relevant when: selected( ${q104_M19_full_list} , '5')* | microgram — microgram (mcg)<br>miligram — miligram (mg)<br>miligram_dissolved_in_liquid — miligram (mg) dissolved in liquid (e.g. 2 mg per 3 militers)<br>gram — gram (g)<br>militer — militer (ml)<br>centiliter — centiliter (cl)<br>litre — litre (l)<br>ml_of _solution — ml of solution (e.g. ml of 10% solution)<br>litre_of_solution — litre of solution (e.g. 1 litre of 5% solution)<br>cubic_meter — cubic meter (m^3)<br>other — other<br>dont_know — don't know |
| q604_M19c.1 | q604_M19c.1 If other unit of measurement or route of administration, specify here:<br>*Leave blank if not applicable*<br>*Question relevant when: selected( ${q104_M19_full_list} , '5')* | |
| q604_M19d *(required)* | q604_M19d. In total, how many units are used to treat a patient during all of her care? | |

| Field | Question | Answer |
|---|---|---|
| | *Remember to add all units! E.g. 5 days x 200 mg twice per day = 2,000mg*<br>*Question relevant when: selected( ${q104_M19_full_list} , '5')*<br>*Response constrained to: .>0* | |
| q604_M20 | ***q604_M20. Oxygen***<br>*Question relevant when: selected( ${q104_M20_full_list} , '5')* | |
| q604_M20a *(required)* | q604_M20a. What percent of patients require this item?<br>*Question relevant when: selected( ${q104_M20_full_list} , '5')*<br>*Response constrained to: .>0 and .<=100 or .=999* | |
| q604_M20b *(required)* | q604_M20b. How is the item administered, or given, to the patient?<br>*Question relevant when: selected( ${q104_M20_full_list} , '5')* | 1 Oral - solid (e.g. tablet)<br>2 Oral - liquid<br>3 Sublingual<br>4 Intravenous (IV)<br>5 Intramusculuar injection (IM)<br>6 Subcutaneous<br>7 Rectal<br>8 Inhaled (e.g. gas)<br>9 Inserted (e.g. vaginal pessaries)<br>10 Topical - cream<br>11 Topical - drops (e.g. eye drops)<br>12 Other<br>99 Don't know |
| q604_M20c *(required)* | q604_M20c. What is the item's smallest unit of measurement (mg, mcg, ml, g, etc)?<br>*Question relevant when: selected( ${q104_M20_full_list} , '5')* | microgram — microgram (mcg)<br>miligram — miligram (mg)<br>miligram_dissolved_in_liquid — miligram (mg) dissolved in liquid (e.g. 2 mg per 3 militers)<br>gram — gram (g)<br>militer — militer (ml)<br>centiliter — centiliter (cl)<br>litre — litre (l)<br>ml_of _solution — ml of solution (e.g. ml of 10% solution)<br>litre_of_solution — litre of solution (e.g. 1 litre of 5% solution)<br>cubic_meter — cubic meter (m^3)<br>other — other<br>dont_know — don't know |
| q604_M20c.1 | q604_M20c.1 If other unit of measurement or route of administration, specify here:<br>*Leave blank if not applicable*<br>*Question relevant when: selected( ${q104_M20_full_list} , '5')* | |
| q604_M20d *(required)* | q604_M20d. In total, how many units are used to treat a patient during all of her care? | |

| Field | Question | Answer |
|---|---|---|
| | *Remember to add all units! E.g. 5 days x 200 mg twice per day = 2,000mg*<br>*Question relevant when: selected( ${q104_M20_full_list} , '5')*<br>*Response constrained to: .>0* | |
| q604_M21 | ***q604_M21. Bupivacaine HCL 0.5% (Marcaine)***<br>*Question relevant when: selected( ${q104_M21_full_list} , '5')* | |
| q604_M21a *(required)* | q604_M21a. What percent of patients require this item?<br>*Question relevant when: selected( ${q104_M21_full_list} , '5')*<br>*Response constrained to: .>0 and .<=100 or .=999* | |

| | | |
|---|---|---|
| q604_M21b *(required)* | q604_M21b. How is the item administered, or given, to the patient?<br>*Question relevant when: selected( ${q104_M21_full_list} , '5')* | 1 Oral - solid (e.g. tablet)<br>2 Oral - liquid<br>3 Sublingual<br>4 Intravenous (IV)<br>5 Intramusculuar injection (IM)<br>6 Subcutaneous<br>7 Rectal<br>8 Inhaled (e.g. gas)<br>9 Inserted (e.g. vaginal pessaries)<br>10 Topical - cream<br>11 Topical - drops (e.g. eye drops)<br>12 Other<br>99 Don't know |
| q604_M21c *(required)* | q604_M21c. What is the item's smallest unit of measurement (mg, mcg, ml, g, etc)?<br>*Question relevant when: selected( ${q104_M21_full_list} , '5')* | microgram — microgram (mcg)<br>miligram — miligram (mg)<br>miligram_dissolved_in_liquid — miligram (mg) dissolved in liquid (e.g. 2 mg per 3 militers)<br>gram — gram (g)<br>militer — militer (ml)<br>centiliter — centiliter (cl)<br>litre — litre (l)<br>ml_of _solution — ml of solution (e.g. ml of 10% solution)<br>litre_of_solution — litre of solution (e.g. 1 litre of 5% solution)<br>cubic_meter — cubic meter (m^3)<br>other — other<br>dont_know — don't know |
| q604_M21c.1 | q604_M21c.1 If other unit of measurement or route of administration, specify here:<br>*Leave blank if not applicable*<br>*Question relevant when: selected( ${q104_M21_full_list} , '5')* | |
| q604_M21d *(required)* | q604_M21d. In total, how many units are used to treat a patient during all of her care? | |

| Field | Question | Answer |
|---|---|---|
| | *Remember to add all units! E.g. 5 days x 200 mg twice per day = 2,000mg*<br>*Question relevant when: selected( ${q104_M21_full_list} , '5')*<br>*Response constrained to: .>0* | |
| q604_M22 | ***q604_M22. Lidocaine HCl 2% + epinephrin 1:100,000***<br>*Question relevant when: selected( ${q104_M22_full_list} , '5')* | |
| q604_M22a *(required)* | q604_M22a. What percent of patients require this item?<br>*Question relevant when: selected( ${q104_M22_full_list} , '5')*<br>*Response constrained to: .>0 and .<=100 or .=999* | |

| Field | Question | Answer | | |
|---|---|---|---|---|
| q604_M22b *(required)* | q604_M22b. How is the item administered, or given, to the patient?<br>*Question relevant when: selected( ${q104_M22_full_list} , '5')* | | 1 | Oral - solid (e.g. tablet) |
| | | | 2 | Oral - liquid |
| | | | 3 | Sublingual |
| | | | 4 | Intravenous (IV) |
| | | | 5 | Intramusculuar injection (IM) |
| | | | 6 | Subcutaneous |
| | | | 7 | Rectal |
| | | | 8 | Inhaled (e.g. gas) |
| | | | 9 | Inserted (e.g. vaginal pessaries) |
| | | | 10 | Topical - cream |
| | | | 11 | Topical - drops (e.g. eye drops) |
| | | | 12 | Other |
| | | | 99 | Don't know |
| q604_M22c *(required)* | q604_M22c. What is the item's smallest unit of measurement (mg, mcg, ml, g, etc)?<br>*Question relevant when: selected( ${q104_M22_full_list} , '5')* | | microgram | microgram (mcg) |
| | | | miligram | miligram (mg) |
| | | | miligram_dissolved_in_liquid | miligram (mg) dissolved in liquid (e.g. 2 mg per 3 militers) |
| | | | gram | gram (g) |
| | | | militer | militer (ml) |
| | | | centiliter | centiliter (cl) |
| | | | litre | litre (l) |
| | | | ml_of _solution | ml of solution (e.g. ml of 10% solution) |
| | | | litre_of_solution | litre of solution (e.g. 1 litre of 5% solution) |
| | | | cubic_meter | cubic meter (m^3) |
| | | | other | other |
| | | | dont_know | don't know |
| q604_M22c.1 | q604_M22c.1 If other unit of measurement or route of administration, specify here:<br>*Leave blank if not applicable*<br>*Question relevant when: selected( ${q104_M22_full_list} , '5')* | | | |
| q604_M22d *(required)* | q604_M22d. In total, how many units are used to treat a patient during all of her care? | | | |

| Field | Question | Answer |
|---|---|---|
| | *Remember to add all units! E.g. 5 days x 200 mg twice per day = 2,000mg*<br>*Question relevant when: selected( ${q104_M22_full_list} , '5')*<br>*Response constrained to: .>0* | |
| q604_M23 | ***q604_M23. Lidocaine HCl 5% + dextrose 7.5%***<br>*Question relevant when: selected( ${q104_M23_full_list} , '5')* | |
| q604_M23a *(required)* | q604_M23a. What percent of patients require this item?<br>*Question relevant when: selected( ${q104_M23_full_list} , '5')*<br>*Response constrained to: .>0 and .<=100 or .=999* | |
| q604_M23b *(required)* | q604_M23b. How is the item administered, or given, to the patient?<br>*Question relevant when: selected( ${q104_M23_full_list} , '5')* | 1 Oral - solid (e.g. tablet)<br>2 Oral - liquid<br>3 Sublingual<br>4 Intravenous (IV)<br>5 Intramusculuar injection (IM)<br>6 Subcutaneous<br>7 Rectal<br>8 Inhaled (e.g. gas)<br>9 Inserted (e.g. vaginal pessaries)<br>10 Topical - cream<br>11 Topical - drops (e.g. eye drops)<br>12 Other<br>99 Don't know |
| q604_M23c *(required)* | q604_M23c. What is the item's smallest unit of measurement (mg, mcg, ml, g, etc)?<br>*Question relevant when: selected( ${q104_M23_full_list} , '5')* | microgram — microgram (mcg)<br>miligram — miligram (mg)<br>miligram_dissolved_in_liquid — miligram (mg) dissolved in liquid (e.g. 2 mg per 3 militers)<br>gram — gram (g)<br>militer — militer (ml)<br>centiliter — centiliter (cl)<br>litre — litre (l)<br>ml_of _solution — ml of solution (e.g. ml of 10% solution)<br>litre_of_solution — litre of solution (e.g. 1 litre of 5% solution)<br>cubic_meter — cubic meter (m^3)<br>other — other<br>dont_know — don't know |
| q604_M23c.1 | q604_M23c.1 If other unit of measurement or route of administration, specify here:<br>*Leave blank if not applicable*<br>*Question relevant when: selected( ${q104_M23_full_list} , '5')* | |
| q604_M23d *(required)* | q604_M23d. In total, how many units are used to treat a patient during all of her care? | |

| Field | Question | Answer |
|---|---|---|
| | *Remember to add all units! E.g. 5 days x 200 mg twice per day = 2,000mg*<br>*Question relevant when: selected( ${q104_M23_full_list} , '5')*<br>*Response constrained to: .>0* | |
| q604_M24 | ***q604_M24. Lidocaine HCl, 1%***<br>*Question relevant when: selected( ${q104_M24_full_list} , '5')* | |
| q604_M24a *(required)* | q604_M24a. What percent of patients require this item?<br>*Question relevant when: selected( ${q104_M24_full_list} , '5')*<br>*Response constrained to: .>0 and .<=100 or .=999* | |

| Field | Question | Answer | |
|---|---|---|---|
| q604_M24b *(required)* | q604_M24b. How is the item administered, or given, to the patient?<br>*Question relevant when: selected( ${q104_M24_full_list} , '5')* | 1 | Oral - solid (e.g. tablet) |
| | | 2 | Oral - liquid |
| | | 3 | Sublingual |
| | | 4 | Intravenous (IV) |
| | | 5 | Intramusculuar injection (IM) |
| | | 6 | Subcutaneous |
| | | 7 | Rectal |
| | | 8 | Inhaled (e.g. gas) |
| | | 9 | Inserted (e.g. vaginal pessaries) |
| | | 10 | Topical - cream |
| | | 11 | Topical - drops (e.g. eye drops) |
| | | 12 | Other |
| | | 99 | Don't know |
| q604_M24c *(required)* | q604_M24c. What is the item's smallest unit of measurement (mg, mcg, ml, g, etc)?<br>*Question relevant when: selected( ${q104_M24_full_list} , '5')* | microgram | microgram (mcg) |
| | | miligram | miligram (mg) |
| | | miligram_dissolved_in_liquid | miligram (mg) dissolved in liquid (e.g. 2 mg per 3 militers) |
| | | gram | gram (g) |
| | | militer | militer (ml) |
| | | centiliter | centiliter (cl) |
| | | litre | litre (l) |
| | | ml_of _solution | ml of solution (e.g. ml of 10% solution) |
| | | litre_of_solution | litre of solution (e.g. 1 litre of 5% solution) |
| | | cubic_meter | cubic meter (m^3) |
| | | other | other |
| | | dont_know | don't know |
| q604_M24c.1 | q604_M24c.1 If other unit of measurement or route of administration, specify here:<br>*Leave blank if not applicable*<br>*Question relevant when: selected( ${q104_M24_full_list} , '5')* | | |
| q604_M24d *(required)* | q604_M24d. In total, how many units are used to treat a patient during all of her care? | | |

| Field | Question | Answer |
|---|---|---|
| | *Remember to add all units! E.g. 5 days x 200 mg twice per day = 2,000mg*<br>*Question relevant when: selected( ${q104_M24_full_list} , '5')*<br>*Response constrained to: .>0* | |
| q604_M25 | ***q604_M25. Anesthetic - other 1: "[q104_M25_full_list_other]"***<br>*Question relevant when: selected( ${q104_M25_full_list} , '5')* | |
| q604_M25a *(required)* | q604_M25a. What percent of patients require this item?<br>*Question relevant when: selected( ${q104_M25_full_list} , '5')*<br>*Response constrained to: .>0 and .<=100 or .=999* | |
| q604_M25b *(required)* | q604_M25b. How is the item administered, or given, to the patient?<br>*Question relevant when: selected( ${q104_M25_full_list} , '5')* | 1 Oral - solid (e.g. tablet)<br>2 Oral - liquid<br>3 Sublingual<br>4 Intravenous (IV)<br>5 Intramusculuar injection (IM)<br>6 Subcutaneous<br>7 Rectal<br>8 Inhaled (e.g. gas)<br>9 Inserted (e.g. vaginal pessaries)<br>10 Topical - cream<br>11 Topical - drops (e.g. eye drops)<br>12 Other<br>99 Don't know |
| q604_M25c *(required)* | q604_M25c. What is the item's smallest unit of measurement (mg, mcg, ml, g, etc)?<br>*Question relevant when: selected( ${q104_M25_full_list} , '5')* | microgram — microgram (mcg)<br>miligram — miligram (mg)<br>miligram_dissolved_in_liquid — miligram (mg) dissolved in liquid (e.g. 2 mg per 3 militers)<br>gram — gram (g)<br>militer — militer (ml)<br>centiliter — centiliter (cl)<br>litre — litre (l)<br>ml_of _solution — ml of solution (e.g. ml of 10% solution)<br>litre_of_solution — litre of solution (e.g. 1 litre of 5% solution)<br>cubic_meter — cubic meter (m^3)<br>other — other<br>dont_know — don't know |
| q604_M25c.1 | q604_M25c.1 If other unit of measurement or route of administration, specify here:<br>*Leave blank if not applicable*<br>*Question relevant when: selected( ${q104_M25_full_list} , '5')* | |
| q604_M25d *(required)* | q604_M25d. In total, how many units are used to treat a patient during all of her care? | |

| Field | Question | Answer |
|---|---|---|
| | *Remember to add all units! E.g. 5 days x 200 mg twice per day = 2,000mg*<br>*Question relevant when: selected( ${q104_M25_full_list} , '5')*<br>*Response constrained to: .>0* | |
| q604_M26 | ***q604_M26. Anesthetic - other 2: "[q104_M26_full_list_other]"***<br>*Question relevant when: selected( ${q104_M26_full_list} , '5')* | |
| q604_M26a *(required)* | q604_M26a. What percent of patients require this item?<br>*Question relevant when: selected( ${q104_M26_full_list} , '5')*<br>*Response constrained to: .>0 and .<=100 or .=999* | |
| q604_M26b *(required)* | q604_M26b. How is the item administered, or given, to the patient?<br>*Question relevant when: selected( ${q104_M26_full_list} , '5')* | 1 Oral - solid (e.g. tablet)<br>2 Oral - liquid<br>3 Sublingual<br>4 Intravenous (IV)<br>5 Intramusculuar injection (IM)<br>6 Subcutaneous<br>7 Rectal<br>8 Inhaled (e.g. gas)<br>9 Inserted (e.g. vaginal pessaries)<br>10 Topical - cream<br>11 Topical - drops (e.g. eye drops)<br>12 Other<br>99 Don't know |
| q604_M26c *(required)* | q604_M26c. What is the item's smallest unit of measurement (mg, mcg, ml, g, etc)?<br>*Question relevant when: selected( ${q104_M26_full_list} , '5')* | microgram — microgram (mcg)<br>miligram — miligram (mg)<br>miligram_dissolved_in_liquid — miligram (mg) dissolved in liquid (e.g. 2 mg per 3 militers)<br>gram — gram (g)<br>militer — militer (ml)<br>centiliter — centiliter (cl)<br>litre — litre (l)<br>ml_of _solution — ml of solution (e.g. ml of 10% solution)<br>litre_of_solution — litre of solution (e.g. 1 litre of 5% solution)<br>cubic_meter — cubic meter (m^3)<br>other — other<br>dont_know — don't know |
| q604_M26c.1 | q604_M26c.1 If other unit of measurement or route of administration, specify here:<br>*Leave blank if not applicable*<br>*Question relevant when: selected( ${q104_M26_full_list} , '5')* | |
| q604_M26d *(required)* | q604_M26d. In total, how many units are used to treat a patient during all of her care? | |

| Field | Question | Answer |
|---|---|---|
| | *Remember to add all units! E.g. 5 days x 200 mg twice per day = 2,000mg*<br>*Question relevant when: selected( ${q104_M26_full_list} , '5')*<br>*Response constrained to: .>0* | |
| q604_M27 | ***q604_M27. Anesthetic - other 3: "[q104_M27_full_list_other]"***<br>*Question relevant when: selected( ${q104_M27_full_list} , '5')* | |
| q604_M27a *(required)* | q604_M27a. What percent of patients require this item?<br>*Question relevant when: selected( ${q104_M27_full_list} , '5')*<br>*Response constrained to: .>0 and .<=100 or .=999* | |
| q604_M27b *(required)* | q604_M27b. How is the item administered, or given, to the patient?<br>*Question relevant when: selected( ${q104_M27_full_list} , '5')* | 1 Oral - solid (e.g. tablet)<br>2 Oral - liquid<br>3 Sublingual<br>4 Intravenous (IV)<br>5 Intramusculuar injection (IM)<br>6 Subcutaneous<br>7 Rectal<br>8 Inhaled (e.g. gas)<br>9 Inserted (e.g. vaginal pessaries)<br>10 Topical - cream<br>11 Topical - drops (e.g. eye drops)<br>12 Other<br>99 Don't know |
| q604_M27c *(required)* | q604_M27c. What is the item's smallest unit of measurement (mg, mcg, ml, g, etc)?<br>*Question relevant when: selected( ${q104_M27_full_list} , '5')* | microgram — microgram (mcg)<br>miligram — miligram (mg)<br>miligram_dissolved_in_liquid — miligram (mg) dissolved in liquid (e.g. 2 mg per 3 militers)<br>gram — gram (g)<br>militer — militer (ml)<br>centiliter — centiliter (cl)<br>litre — litre (l)<br>ml_of _solution — ml of solution (e.g. ml of 10% solution)<br>litre_of_solution — litre of solution (e.g. 1 litre of 5% solution)<br>cubic_meter — cubic meter (m^3)<br>other — other<br>dont_know — don't know |
| q604_M27c.1 | q604_M27c.1 If other unit of measurement or route of administration, specify here:<br>*Leave blank if not applicable*<br>*Question relevant when: selected( ${q104_M27_full_list} , '5')* | |
| q604_M27d *(required)* | q604_M27d. In total, how many units are used to treat a patient during all of her care? | |

| Field | Question | Answer |
|---|---|---|
| | *Remember to add all units! E.g. 5 days x 200 mg twice per day = 2,000mg*<br>*Question relevant when: selected( ${q104_M27_full_list} , '5')*<br>*Response constrained to: .>0* | |
| q604_M28 | ***q604_M28. Anesthetic - other 4: "[q104_M28_full_list_other]"***<br>*Question relevant when: selected( ${q104_M28_full_list} , '5')* | |
| q604_M28a *(required)* | q604_M28a. What percent of patients require this item?<br>*Question relevant when: selected( ${q104_M28_full_list} , '5')*<br>*Response constrained to: .>0 and .<=100 or .=999* | |
| q604_M28b *(required)* | q604_M28b. How is the item administered, or given, to the patient?<br>*Question relevant when: selected( ${q104_M28_full_list} , '5')* | 1 Oral - solid (e.g. tablet)<br>2 Oral - liquid<br>3 Sublingual<br>4 Intravenous (IV)<br>5 Intramusculuar injection (IM)<br>6 Subcutaneous<br>7 Rectal<br>8 Inhaled (e.g. gas)<br>9 Inserted (e.g. vaginal pessaries)<br>10 Topical - cream<br>11 Topical - drops (e.g. eye drops)<br>12 Other<br>99 Don't know |
| q604_M28c *(required)* | q604_M28c. What is the item's smallest unit of measurement (mg, mcg, ml, g, etc)?<br>*Question relevant when: selected( ${q104_M28_full_list} , '5')* | microgram — microgram (mcg)<br>miligram — miligram (mg)<br>miligram_dissolved_in_liquid — miligram (mg) dissolved in liquid (e.g. 2 mg per 3 militers)<br>gram — gram (g)<br>militer — militer (ml)<br>centiliter — centiliter (cl)<br>litre — litre (l)<br>ml_of _solution — ml of solution (e.g. ml of 10% solution)<br>litre_of_solution — litre of solution (e.g. 1 litre of 5% solution)<br>cubic_meter — cubic meter (m^3)<br>other — other<br>dont_know — don't know |
| q604_M28c.1 | q604_M28c.1 If other unit of measurement or route of administration, specify here:<br>*Leave blank if not applicable* | |

| Field | Question | Answer |
|---|---|---|
| | *Question relevant when: selected( ${q104_M28_full_list} , '5')* | |
| q604_M28d *(required)* | q604_M28d. In total, how many units are used to treat a patient during all of her care?<br>*Remember to add all units! E.g. 5 days x 200 mg twice per day = 2,000mg*<br>*Question relevant when: selected( ${q104_M28_full_list} , '5')*<br>*Response constrained to: .>0* | |
| q604_M29 | ***q604_M29. Anesthetic - other 5: "[q104_M29_full_list_other]"***<br>*Question relevant when: selected( ${q104_M29_full_list} , '5')* | |
| q604_M29a *(required)* | q604_M29a. What percent of patients require this item?<br>*Question relevant when: selected( ${q104_M29_full_list} , '5')*<br>*Response constrained to: .>0 and .<=100 or .=999* | |
| q604_M29b *(required)* | q604_M29b. How is the item administered, or given, to the patient?<br>*Question relevant when: selected( ${q104_M29_full_list} , '5')* | 1 Oral - solid (e.g. tablet)<br>2 Oral - liquid<br>3 Sublingual<br>4 Intravenous (IV)<br>5 Intramusculuar injection (IM)<br>6 Subcutaneous<br>7 Rectal<br>8 Inhaled (e.g. gas)<br>9 Inserted (e.g. vaginal pessaries)<br>10 Topical - cream<br>11 Topical - drops (e.g. eye drops)<br>12 Other<br>99 Don't know |
| q604_M29c *(required)* | q604_M29c. What is the item's smallest unit of measurement (mg, mcg, ml, g, etc)?<br>*Question relevant when: selected( ${q104_M29_full_list} , '5')* | microgram — microgram (mcg)<br>miligram — miligram (mg)<br>miligram_dissolved_in_liquid — miligram (mg) dissolved in liquid (e.g. 2 mg per 3 militers)<br>gram — gram (g)<br>militer — militer (ml)<br>centiliter — centiliter (cl)<br>litre — litre (l)<br>ml_of _solution — ml of solution (e.g. ml of 10% solution)<br>litre_of_solution — litre of solution (e.g. 1 litre of 5% solution)<br>cubic_meter — cubic meter (m^3)<br>other — other<br>dont_know — don't know |
| q604_M29c.1 | q604_M29c.1 If other unit of measurement or route of administration, specify here:<br>*Leave blank if not applicable* | |

| Field | Question | Answer |
|---|---|---|
| | *Question relevant when: selected( ${q104_M29_full_list} , '5')* | |
| q604_M29d *(required)* | q604_M29d. In total, how many units are used to treat a patient during all of her care?<br>*Remember to add all units! E.g. 5 days x 200 mg twice per day = 2,000mg*<br>*Question relevant when: selected( ${q104_M29_full_list} , '5')*<br>*Response constrained to: .>0* | |
| <p><strong><em>D. Medications - Perforations (3)</em></strong></p><br>*Group relevant when: selected( ${section_six_skip_med} , '1')* | | |
| q604_med_note3 | 604 CONTINUED For each of the following medications and other medical products that are used for perforations, can you tell me …? | |
| note_604_antibiotics | ***Antibiotics*** | |
| q604_M30 | ***q604_M30. Amoxicillin***<br>*Question relevant when: selected( ${q104_M30_full_list} , '5')* | |
| q604_M30a *(required)* | q604_M30a. What percent of patients require this item?<br>*Question relevant when: selected( ${q104_M30_full_list} , '5')*<br>*Response constrained to: .>0 and .<=100 or .=999* | |

| | | |
|---|---|---|
| q604_M30b *(required)* | q604_M30b. How is the item administered, or given, to the patient?<br>*Question relevant when: selected( ${q104_M30_full_list} , '5')* | 1 Oral - solid (e.g. tablet)<br>2 Oral - liquid<br>3 Sublingual<br>4 Intravenous (IV)<br>5 Intramusculuar injection (IM)<br>6 Subcutaneous<br>7 Rectal<br>8 Inhaled (e.g. gas)<br>9 Inserted (e.g. vaginal pessaries)<br>10 Topical - cream<br>11 Topical - drops (e.g. eye drops)<br>12 Other<br>99 Don't know |
| q604_M30c *(required)* | q604_M30c. What is the item's smallest unit of measurement (mg, mcg, ml, g, etc)?<br>*Question relevant when: selected( ${q104_M30_full_list} , '5')* | microgram — microgram (mcg)<br>miligram — miligram (mg)<br>miligram_dissolved_in_liquid — miligram (mg) dissolved in liquid (e.g. 2 mg per 3 militers)<br>gram — gram (g)<br>militer — militer (ml)<br>centiliter — centiliter (cl)<br>litre — litre (l)<br>ml_of _solution — ml of solution (e.g. ml of 10% solution)<br>litre_of_solution — litre of solution (e.g. 1 litre of 5% solution)<br>cubic_meter — cubic meter (m^3)<br>other — other<br>dont_know — don't know |
| q604_M30c.1 | q604_M30c.1 If other unit of measurement or route of administration, specify here:<br>*Leave blank if not applicable* | |

| Field | Question | Answer |
|---|---|---|
| | *Question relevant when: selected( ${q104_M30_full_list} , '5')* | |
| q604_M30d *(required)* | q604_M30d. In total, how many units are used to treat a patient during all of her care?<br>*Remember to add all units! E.g. 5 days x 200 mg twice per day = 2,000mg*<br>*Question relevant when: selected( ${q104_M30_full_list} , '5')*<br>*Response constrained to: .>0* | |
| q604_M31 | ***q604_M31. Amoxicillin + Clauvulenic Acid 635mg***<br>*Question relevant when: selected( ${q104_M31_full_list} , '5')* | |
| q604_M31a *(required)* | q604_M31a. What percent of patients require this item?<br>*Question relevant when: selected( ${q104_M31_full_list} , '5')*<br>*Response constrained to: .>0 and .<=100 or .=999* | |

| Field | Question | Answer |
|---|---|---|
| q604_M31b *(required)* | q604_M31b. How is the item administered, or given, to the patient?<br>*Question relevant when: selected( ${q104_M31_full_list} , '5')* | 1 Oral - solid (e.g. tablet)<br>2 Oral - liquid<br>3 Sublingual<br>4 Intravenous (IV)<br>5 Intramusculuar injection (IM)<br>6 Subcutaneous<br>7 Rectal<br>8 Inhaled (e.g. gas)<br>9 Inserted (e.g. vaginal pessaries)<br>10 Topical - cream<br>11 Topical - drops (e.g. eye drops)<br>12 Other<br>99 Don't know |
| q604_M31c *(required)* | q604_M31c. What is the item's smallest unit of measurement (mg, mcg, ml, g, etc)?<br>*Question relevant when: selected( ${q104_M31_full_list} , '5')* | microgram — microgram (mcg)<br>miligram — miligram (mg)<br>miligram_dissolved_in_liquid — miligram (mg) dissolved in liquid (e.g. 2 mg per 3 militers)<br>gram — gram (g)<br>militer — militer (ml)<br>centiliter — centiliter (cl)<br>litre — litre (l)<br>ml_of _solution — ml of solution (e.g. ml of 10% solution)<br>litre_of_solution — litre of solution (e.g. 1 litre of 5% solution)<br>cubic_meter — cubic meter (m^3)<br>other — other<br>dont_know — don't know |
| q604_M31c.1 | q604_M31c.1 If other unit of measurement or route of administration, specify here:<br>*Leave blank if not applicable* | |

| Field | Question | Answer |
|---|---|---|
| | *Question relevant when: selected( ${q104_M31_full_list} , '5')* | |
| q604_M31d *(required)* | q604_M31d. In total, how many units are used to treat a patient during all of her care?<br>*Remember to add all units! E.g. 5 days x 200 mg twice per day = 2,000mg*<br>*Question relevant when: selected( ${q104_M31_full_list} , '5')*<br>*Response constrained to: .>0* | |
| q604_M32 | ***q604_M32. Ampicillin***<br>*Question relevant when: selected( ${q104_M32_full_list} , '5')* | |
| q604_M32a *(required)* | q604_M32a. What percent of patients require this item?<br>*Question relevant when: selected( ${q104_M32_full_list} , '5')*<br>*Response constrained to: .>0 and .<=100 or .=999* | |

| Field | Question | Answer |
|---|---|---|
| q604_M32b *(required)* | q604_M32b. How is the item administered, or given, to the patient?<br>*Question relevant when: selected( ${q104_M32_full_list} , '5')* | 1 Oral - solid (e.g. tablet)<br>2 Oral - liquid<br>3 Sublingual<br>4 Intravenous (IV)<br>5 Intramusculuar injection (IM)<br>6 Subcutaneous<br>7 Rectal<br>8 Inhaled (e.g. gas)<br>9 Inserted (e.g. vaginal pessaries)<br>10 Topical - cream<br>11 Topical - drops (e.g. eye drops)<br>12 Other<br>99 Don't know |
| q604_M32c *(required)* | q604_M32c. What is the item's smallest unit of measurement (mg, mcg, ml, g, etc)?<br>*Question relevant when: selected( ${q104_M32_full_list} , '5')* | microgram — microgram (mcg)<br>miligram — miligram (mg)<br>miligram_dissolved_in_liquid — miligram (mg) dissolved in liquid (e.g. 2 mg per 3 militers)<br>gram — gram (g)<br>militer — militer (ml)<br>centiliter — centiliter (cl)<br>litre — litre (l)<br>ml_of _solution — ml of solution (e.g. ml of 10% solution)<br>litre_of_solution — litre of solution (e.g. 1 litre of 5% solution)<br>cubic_meter — cubic meter (m^3)<br>other — other<br>dont_know — don't know |
| q604_M32c.1 | q604_M32c.1 If other unit of measurement or route of administration, specify here:<br>*Leave blank if not applicable* | |

| Field | Question | Answer |
|---|---|---|
| | *Question relevant when: selected( ${q104_M32_full_list} , '5')* | |
| q604_M32d *(required)* | q604_M32d. In total, how many units are used to treat a patient during all of her care?<br>*Remember to add all units! E.g. 5 days x 200 mg twice per day = 2,000mg*<br>*Question relevant when: selected( ${q104_M32_full_list} , '5')*<br>*Response constrained to: .>0* | |
| q604_M33 | ***q604_M33. Ampicillin (injection)***<br>*Question relevant when: selected( ${q104_M33_full_list} , '5')* | |
| q604_M33a *(required)* | q604_M33a. What percent of patients require this item?<br>*Question relevant when: selected( ${q104_M33_full_list} , '5')*<br>*Response constrained to: .>0 and .<=100 or .=999* | |

| Field | Question | Answer |
|---|---|---|
| q604_M33b *(required)* | q604_M33b. How is the item administered, or given, to the patient?<br>*Question relevant when: selected( ${q104_M33_full_list} , '5')* | 1 Oral - solid (e.g. tablet)<br>2 Oral - liquid<br>3 Sublingual<br>4 Intravenous (IV)<br>5 Intramusculuar injection (IM)<br>6 Subcutaneous<br>7 Rectal<br>8 Inhaled (e.g. gas)<br>9 Inserted (e.g. vaginal pessaries)<br>10 Topical - cream<br>11 Topical - drops (e.g. eye drops)<br>12 Other<br>99 Don't know |
| q604_M33c *(required)* | q604_M33c. What is the item's smallest unit of measurement (mg, mcg, ml, g, etc)?<br>*Question relevant when: selected( ${q104_M33_full_list} , '5')* | microgram — microgram (mcg)<br>miligram — miligram (mg)<br>miligram_dissolved_in_liquid — miligram (mg) dissolved in liquid (e.g. 2 mg per 3 militers)<br>gram — gram (g)<br>militer — militer (ml)<br>centiliter — centiliter (cl)<br>litre — litre (l)<br>ml_of _solution — ml of solution (e.g. ml of 10% solution)<br>litre_of_solution — litre of solution (e.g. 1 litre of 5% solution)<br>cubic_meter — cubic meter (m^3)<br>other — other<br>dont_know — don't know |
| q604_M33c.1 | q604_M33c.1 If other unit of measurement or route of administration, specify here:<br>*Leave blank if not applicable* | |

| Field | Question | Answer |
|---|---|---|
| | *Question relevant when: selected( ${q104_M33_full_list} , '5')* | |
| q604_M33d *(required)* | q604_M33d. In total, how many units are used to treat a patient during all of her care?<br>*Remember to add all units! E.g. 5 days x 200 mg twice per day = 2,000mg*<br>*Question relevant when: selected( ${q104_M33_full_list} , '5')*<br>*Response constrained to: .>0* | |
| q604_M34 | ***q604_M34. Ampicillin/sulbactam (Unasyn)***<br>*Question relevant when: selected( ${q104_M34_full_list} , '5')* | |
| q604_M34a *(required)* | q604_M34a. What percent of patients require this item?<br>*Question relevant when: selected( ${q104_M34_full_list} , '5')*<br>*Response constrained to: .>0 and .<=100 or .=999* | |
| q604_M34b *(required)* | q604_M34b. How is the item administered, or given, to the patient?<br>*Question relevant when: selected( ${q104_M34_full_list} , '5')* | 1 Oral - solid (e.g. tablet)<br>2 Oral - liquid<br>3 Sublingual<br>4 Intravenous (IV)<br>5 Intramusculuar injection (IM)<br>6 Subcutaneous<br>7 Rectal<br>8 Inhaled (e.g. gas)<br>9 Inserted (e.g. vaginal pessaries)<br>10 Topical - cream<br>11 Topical - drops (e.g. eye drops)<br>12 Other<br>99 Don't know |
| q604_M34c *(required)* | q604_M34c. What is the item's smallest unit of measurement (mg, mcg, ml, g, etc)?<br>*Question relevant when: selected( ${q104_M34_full_list} , '5')* | microgram — microgram (mcg)<br>miligram — miligram (mg)<br>miligram_dissolved_in_liquid — miligram (mg) dissolved in liquid (e.g. 2 mg per 3 militers)<br>gram — gram (g)<br>militer — militer (ml)<br>centiliter — centiliter (cl)<br>litre — litre (l)<br>ml_of _solution — ml of solution (e.g. ml of 10% solution)<br>litre_of_solution — litre of solution (e.g. 1 litre of 5% solution)<br>cubic_meter — cubic meter (m^3)<br>other — other<br>dont_know — don't know |
| q604_M34c.1 | q604_M34c.1 If other unit of measurement or route of administration, specify here:<br>*Leave blank if not applicable* | |

| Field | Question | Answer |
|---|---|---|
| | *Question relevant when: selected( ${q104_M34_full_list} , '5')* | |
| q604_M34d *(required)* | q604_M34d. In total, how many units are used to treat a patient during all of her care?<br>*Remember to add all units! E.g. 5 days x 200 mg twice per day = 2,000mg*<br>*Question relevant when: selected( ${q104_M34_full_list} , '5')*<br>*Response constrained to: .>0* | |
| q604_M35 | ***q604_M35. Ceftriaxone***<br>*Question relevant when: selected( ${q104_M35_full_list} , '5')* | |
| q604_M35a *(required)* | q604_M35a. What percent of patients require this item?<br>*Question relevant when: selected( ${q104_M35_full_list} , '5')*<br>*Response constrained to: .>0 and .<=100 or .=999* | |

| Field | Question | Answer |
|---|---|---|
| q604_M35b *(required)* | q604_M35b. How is the item administered, or given, to the patient?<br>*Question relevant when: selected( ${q104_M35_full_list} , '5')* | 1 Oral - solid (e.g. tablet)<br>2 Oral - liquid<br>3 Sublingual<br>4 Intravenous (IV)<br>5 Intramusculuar injection (IM)<br>6 Subcutaneous<br>7 Rectal<br>8 Inhaled (e.g. gas)<br>9 Inserted (e.g. vaginal pessaries)<br>10 Topical - cream<br>11 Topical - drops (e.g. eye drops)<br>12 Other<br>99 Don't know |
| q604_M35c *(required)* | q604_M35c. What is the item's smallest unit of measurement (mg, mcg, ml, g, etc)?<br>*Question relevant when: selected( ${q104_M35_full_list} , '5')* | microgram — microgram (mcg)<br>miligram — miligram (mg)<br>miligram_dissolved_in_liquid — miligram (mg) dissolved in liquid (e.g. 2 mg per 3 militers)<br>gram — gram (g)<br>militer — militer (ml)<br>centiliter — centiliter (cl)<br>litre — litre (l)<br>ml_of _solution — ml of solution (e.g. ml of 10% solution)<br>litre_of_solution — litre of solution (e.g. 1 litre of 5% solution)<br>cubic_meter — cubic meter (m^3)<br>other — other<br>dont_know — don't know |
| q604_M35c.1 | q604_M35c.1 If other unit of measurement or route of administration, specify here:<br>*Leave blank if not applicable* | |

| Field | Question | Answer |
|---|---|---|
| | *Question relevant when: selected( ${q104_M35_full_list} , '5')* | |
| q604_M35d *(required)* | q604_M35d. In total, how many units are used to treat a patient during all of her care?<br>*Remember to add all units! E.g. 5 days x 200 mg twice per day = 2,000mg*<br>*Question relevant when: selected( ${q104_M35_full_list} , '5')*<br>*Response constrained to: .>0* | |
| q604_M36 | ***q604_M36. Chloramphenicol Sodium Succinate (injection)***<br>*Question relevant when: selected( ${q104_M36_full_list} , '5')* | |
| q604_M36a *(required)* | q604_M36a. What percent of patients require this item?<br>*Question relevant when: selected( ${q104_M36_full_list} , '5')*<br>*Response constrained to: .>0 and .<=100 or .=999* | |
| q604_M36b *(required)* | q604_M36b. How is the item administered, or given, to the patient?<br>*Question relevant when: selected( ${q104_M36_full_list} , '5')* | 1 Oral - solid (e.g. tablet)<br>2 Oral - liquid<br>3 Sublingual<br>4 Intravenous (IV)<br>5 Intramusculuar injection (IM)<br>6 Subcutaneous<br>7 Rectal<br>8 Inhaled (e.g. gas)<br>9 Inserted (e.g. vaginal pessaries)<br>10 Topical - cream<br>11 Topical - drops (e.g. eye drops)<br>12 Other<br>99 Don't know |
| q604_M36c *(required)* | q604_M36c. What is the item's smallest unit of measurement (mg, mcg, ml, g, etc)?<br>*Question relevant when: selected( ${q104_M36_full_list} , '5')* | microgram — microgram (mcg)<br>miligram — miligram (mg)<br>miligram_dissolved_in_liquid — miligram (mg) dissolved in liquid (e.g. 2 mg per 3 militers)<br>gram — gram (g)<br>militer — militer (ml)<br>centiliter — centiliter (cl)<br>litre — litre (l)<br>ml_of _solution — ml of solution (e.g. ml of 10% solution)<br>litre_of_solution — litre of solution (e.g. 1 litre of 5% solution)<br>cubic_meter — cubic meter (m^3)<br>other — other<br>dont_know — don't know |
| q604_M36c.1 | q604_M36c.1 If other unit of measurement or route of administration, specify here:<br>*Leave blank if not applicable* | |

| Field | Question | Answer |
|---|---|---|
| | *Question relevant when: selected( ${q104_M36_full_list} , '5')* | |
| q604_M36d *(required)* | q604_M36d. In total, how many units are used to treat a patient during all of her care?<br>*Remember to add all units! E.g. 5 days x 200 mg twice per day = 2,000mg*<br>*Question relevant when: selected( ${q104_M36_full_list} , '5')*<br>*Response constrained to: .>0* | |
| q604_M37 | ***q604_M37. Ciprofloxacin***<br>*Question relevant when: selected( ${q104_M37_full_list} , '5')* | |
| q604_M37a *(required)* | q604_M37a. What percent of patients require this item?<br>*Question relevant when: selected( ${q104_M37_full_list} , '5')*<br>*Response constrained to: .>0 and .<=100 or .=999* | |
| q604_M37b *(required)* | q604_M37b. How is the item administered, or given, to the patient?<br>*Question relevant when: selected( ${q104_M37_full_list} , '5')* | 1 Oral - solid (e.g. tablet)<br>2 Oral - liquid<br>3 Sublingual<br>4 Intravenous (IV)<br>5 Intramusculuar injection (IM)<br>6 Subcutaneous<br>7 Rectal<br>8 Inhaled (e.g. gas)<br>9 Inserted (e.g. vaginal pessaries)<br>10 Topical - cream<br>11 Topical - drops (e.g. eye drops)<br>12 Other<br>99 Don't know |
| q604_M37c *(required)* | q604_M37c. What is the item's smallest unit of measurement (mg, mcg, ml, g, etc)?<br>*Question relevant when: selected( ${q104_M37_full_list} , '5')* | microgram — microgram (mcg)<br>miligram — miligram (mg)<br>miligram_dissolved_in_liquid — miligram (mg) dissolved in liquid (e.g. 2 mg per 3 militers)<br>gram — gram (g)<br>militer — militer (ml)<br>centiliter — centiliter (cl)<br>litre — litre (l)<br>ml_of _solution — ml of solution (e.g. ml of 10% solution)<br>litre_of_solution — litre of solution (e.g. 1 litre of 5% solution)<br>cubic_meter — cubic meter (m^3)<br>other — other<br>dont_know — don't know |
| q604_M37c.1 | q604_M37c.1 If other unit of measurement or route of administration, specify here:<br>*Leave blank if not applicable* | |

| Field | Question | Answer |
|---|---|---|
| | *Question relevant when: selected( ${q104_M37_full_list} , '5')* | |
| q604_M37d *(required)* | q604_M37d. In total, how many units are used to treat a patient during all of her care?<br>*Remember to add all units! E.g. 5 days x 200 mg twice per day = 2,000mg*<br>*Question relevant when: selected( ${q104_M37_full_list} , '5')*<br>*Response constrained to: .>0* | |
| q604_M38 | ***q604_M38. Doxycycline***<br>*Question relevant when: selected( ${q104_M38_full_list} , '5')* | |
| q604_M38a *(required)* | q604_M38a. What percent of patients require this item?<br>*Question relevant when: selected( ${q104_M38_full_list} , '5')*<br>*Response constrained to: .>0 and .<=100 or .=999* | |

| | | |
|---|---|---|
| q604_M38b *(required)* | q604_M38b. How is the item administered, or given, to the patient?<br>*Question relevant when: selected( ${q104_M38_full_list} , '5')* | 1 Oral - solid (e.g. tablet)<br>2 Oral - liquid<br>3 Sublingual<br>4 Intravenous (IV)<br>5 Intramusculuar injection (IM)<br>6 Subcutaneous<br>7 Rectal<br>8 Inhaled (e.g. gas)<br>9 Inserted (e.g. vaginal pessaries)<br>10 Topical - cream<br>11 Topical - drops (e.g. eye drops)<br>12 Other<br>99 Don't know |
| q604_M38c *(required)* | q604_M38c. What is the item's smallest unit of measurement (mg, mcg, ml, g, etc)?<br>*Question relevant when: selected( ${q104_M38_full_list} , '5')* | microgram — microgram (mcg)<br>miligram — miligram (mg)<br>miligram_dissolved_in_liquid — miligram (mg) dissolved in liquid (e.g. 2 mg per 3 militers)<br>gram — gram (g)<br>militer — militer (ml)<br>centiliter — centiliter (cl)<br>litre — litre (l)<br>ml_of _solution — ml of solution (e.g. ml of 10% solution)<br>litre_of_solution — litre of solution (e.g. 1 litre of 5% solution)<br>cubic_meter — cubic meter (m^3)<br>other — other<br>dont_know — don't know |
| q604_M38c.1 | q604_M38c.1 If other unit of measurement or route of administration, specify here:<br>*Leave blank if not applicable* | |

| Field | Question | Answer |
|---|---|---|
| | *Question relevant when: selected( ${q104_M38_full_list} , '5')* | |
| q604_M38d *(required)* | q604_M38d. In total, how many units are used to treat a patient during all of her care?<br>*Remember to add all units! E.g. 5 days x 200 mg twice per day = 2,000mg*<br>*Question relevant when: selected( ${q104_M38_full_list} , '5')*<br>*Response constrained to: .>0* | |
| q604_M39 | ***q604_M39. Erythromycin***<br>*Question relevant when: selected( ${q104_M39_full_list} , '5')* | |
| q604_M39a *(required)* | q604_M39a. What percent of patients require this item?<br>*Question relevant when: selected( ${q104_M39_full_list} , '5')*<br>*Response constrained to: .>0 and .<=100 or .=999* | |

| | | | | |
|---|---|---|---|---|
| q604_M39b *(required)* | q604_M39b. How is the item administered, or given, to the patient?<br>*Question relevant when: selected( ${q104_M39_full_list} , '5')* | | 1 | Oral - solid (e.g. tablet) |
| | | | 2 | Oral - liquid |
| | | | 3 | Sublingual |
| | | | 4 | Intravenous (IV) |
| | | | 5 | Intramusculuar injection (IM) |
| | | | 6 | Subcutaneous |
| | | | 7 | Rectal |
| | | | 8 | Inhaled (e.g. gas) |
| | | | 9 | Inserted (e.g. vaginal pessaries) |
| | | | 10 | Topical - cream |
| | | | 11 | Topical - drops (e.g. eye drops) |
| | | | 12 | Other |
| | | | 99 | Don't know |
| q604_M39c *(required)* | q604_M39c. What is the item's smallest unit of measurement (mg, mcg, ml, g, etc)?<br>*Question relevant when: selected( ${q104_M39_full_list} , '5')* | | microgram | microgram (mcg) |
| | | | miligram | miligram (mg) |
| | | | miligram_dissolved_in_liquid | miligram (mg) dissolved in liquid (e.g. 2 mg per 3 militers) |
| | | | gram | gram (g) |
| | | | militer | militer (ml) |
| | | | centiliter | centiliter (cl) |
| | | | litre | litre (l) |
| | | | ml_of _solution | ml of solution (e.g. ml of 10% solution) |
| | | | litre_of_solution | litre of solution (e.g. 1 litre of 5% solution) |
| | | | cubic_meter | cubic meter (m^3) |
| | | | other | other |
| | | | dont_know | don't know |
| q604_M39c.1 | q604_M39c.1 If other unit of measurement or route of administration, specify here:<br>*Leave blank if not applicable* | | | |

| Field | Question | Answer |
|---|---|---|
| | *Question relevant when: selected( ${q104_M39_full_list} , '5')* | |
| q604_M39d *(required)* | q604_M39d. In total, how many units are used to treat a patient during all of her care?<br>*Remember to add all units! E.g. 5 days x 200 mg twice per day = 2,000mg*<br>*Question relevant when: selected( ${q104_M39_full_list} , '5')*<br>*Response constrained to: .>0* | |
| q604_M40 | ***q604_M40. Gentamycin***<br>*Question relevant when: selected( ${q104_M40_full_list} , '5')* | |
| q604_M40a *(required)* | q604_M40a. What percent of patients require this item?<br>*Question relevant when: selected( ${q104_M40_full_list} , '5')*<br>*Response constrained to: .>0 and .<=100 or .=999* | |

| Field | Question | Answer |
|---|---|---|
| q604_M40b *(required)* | q604_M40b. How is the item administered, or given, to the patient?<br>*Question relevant when: selected( ${q104_M40_full_list} , '5')* | 1 Oral - solid (e.g. tablet)<br>2 Oral - liquid<br>3 Sublingual<br>4 Intravenous (IV)<br>5 Intramusculuar injection (IM)<br>6 Subcutaneous<br>7 Rectal<br>8 Inhaled (e.g. gas)<br>9 Inserted (e.g. vaginal pessaries)<br>10 Topical - cream<br>11 Topical - drops (e.g. eye drops)<br>12 Other<br>99 Don't know |
| q604_M40c *(required)* | q604_M40c. What is the item's smallest unit of measurement (mg, mcg, ml, g, etc)?<br>*Question relevant when: selected( ${q104_M40_full_list} , '5')* | microgram — microgram (mcg)<br>miligram — miligram (mg)<br>miligram_dissolved_in_liquid — miligram (mg) dissolved in liquid (e.g. 2 mg per 3 militers)<br>gram — gram (g)<br>militer — militer (ml)<br>centiliter — centiliter (cl)<br>litre — litre (l)<br>ml_of _solution — ml of solution (e.g. ml of 10% solution)<br>litre_of_solution — litre of solution (e.g. 1 litre of 5% solution)<br>cubic_meter — cubic meter (m^3)<br>other — other<br>dont_know — don't know |
| q604_M40c.1 | q604_M40c.1 If other unit of measurement or route of administration, specify here:<br>*Leave blank if not applicable* | |

| Field | Question | Answer |
|---|---|---|
| | *Question relevant when: selected( ${q104_M40_full_list} , '5')* | |
| q604_M40d *(required)* | q604_M40d. In total, how many units are used to treat a patient during all of her care?<br>*Remember to add all units! E.g. 5 days x 200 mg twice per day = 2,000mg*<br>*Question relevant when: selected( ${q104_M40_full_list} , '5')*<br>*Response constrained to: .>0* | |
| q604_M41 | ***q604_M41. Metronidazole (for infusion)***<br>*Question relevant when: selected( ${q104_M41_full_list} , '5')* | |
| q604_M41a *(required)* | q604_M41a. What percent of patients require this item?<br>*Question relevant when: selected( ${q104_M41_full_list} , '5')*<br>*Response constrained to: .>0 and .<=100 or .=999* | |
| q604_M41b *(required)* | q604_M41b. How is the item administered, or given, to the patient?<br>*Question relevant when: selected( ${q104_M41_full_list} , '5')* | 1 Oral - solid (e.g. tablet)<br>2 Oral - liquid<br>3 Sublingual<br>4 Intravenous (IV)<br>5 Intramusculuar injection (IM)<br>6 Subcutaneous<br>7 Rectal<br>8 Inhaled (e.g. gas)<br>9 Inserted (e.g. vaginal pessaries)<br>10 Topical - cream<br>11 Topical - drops (e.g. eye drops)<br>12 Other<br>99 Don't know |
| q604_M41c *(required)* | q604_M41c. What is the item's smallest unit of measurement (mg, mcg, ml, g, etc)?<br>*Question relevant when: selected( ${q104_M41_full_list} , '5')* | microgram — microgram (mcg)<br>miligram — miligram (mg)<br>miligram_dissolved_in_liquid — miligram (mg) dissolved in liquid (e.g. 2 mg per 3 militers)<br>gram — gram (g)<br>militer — militer (ml)<br>centiliter — centiliter (cl)<br>litre — litre (l)<br>ml_of _solution — ml of solution (e.g. ml of 10% solution)<br>litre_of_solution — litre of solution (e.g. 1 litre of 5% solution)<br>cubic_meter — cubic meter (m^3)<br>other — other<br>dont_know — don't know |
| q604_M41c.1 | q604_M41c.1 If other unit of measurement or route of administration, specify here:<br>*Leave blank if not applicable* | |

| Field | Question | Answer |
|---|---|---|
| | *Question relevant when: selected( ${q104_M41_full_list} , '5')* | |
| q604_M41d *(required)* | q604_M41d. In total, how many units are used to treat a patient during all of her care?<br>*Remember to add all units! E.g. 5 days x 200 mg twice per day = 2,000mg*<br>*Question relevant when: selected( ${q104_M41_full_list} , '5')*<br>*Response constrained to: .>0* | |
| q604_M42 | ***q604_M42. Metronidazole (injection)***<br>*Question relevant when: selected( ${q104_M42_full_list} , '5')* | |
| q604_M42a *(required)* | q604_M42a. What percent of patients require this item?<br>*Question relevant when: selected( ${q104_M42_full_list} , '5')*<br>*Response constrained to: .>0 and .<=100 or .=999* | |

| Field | Question | Answer |
|---|---|---|
| q604_M42b *(required)* | q604_M42b. How is the item administered, or given, to the patient?<br>*Question relevant when: selected( ${q104_M42_full_list} , '5')* | 1 Oral - solid (e.g. tablet)<br>2 Oral - liquid<br>3 Sublingual<br>4 Intravenous (IV)<br>5 Intramusculuar injection (IM)<br>6 Subcutaneous<br>7 Rectal<br>8 Inhaled (e.g. gas)<br>9 Inserted (e.g. vaginal pessaries)<br>10 Topical - cream<br>11 Topical - drops (e.g. eye drops)<br>12 Other<br>99 Don't know |
| q604_M42c *(required)* | q604_M42c. What is the item's smallest unit of measurement (mg, mcg, ml, g, etc)?<br>*Question relevant when: selected( ${q104_M42_full_list} , '5')* | microgram — microgram (mcg)<br>miligram — miligram (mg)<br>miligram_dissolved_in_liquid — miligram (mg) dissolved in liquid (e.g. 2 mg per 3 militers)<br>gram — gram (g)<br>militer — militer (ml)<br>centiliter — centiliter (cl)<br>litre — litre (l)<br>ml_of _solution — ml of solution (e.g. ml of 10% solution)<br>litre_of_solution — litre of solution (e.g. 1 litre of 5% solution)<br>cubic_meter — cubic meter (m^3)<br>other — other<br>dont_know — don't know |
| q604_M42c.1 | q604_M42c.1 If other unit of measurement or route of administration, specify here:<br>*Leave blank if not applicable* | |

| Field | Question | Answer |
|---|---|---|
| | *Question relevant when: selected( ${q104_M42_full_list} , '5')* | |
| q604_M42d *(required)* | q604_M42d. In total, how many units are used to treat a patient during all of her care? | |
| | *Remember to add all units! E.g. 5 days x 200 mg twice per day = 2,000mg*<br>*Question relevant when: selected( ${q104_M42_full_list} , '5')*<br>*Response constrained to: .>0* | |
| q604_M43 | ***q604_M43. Metronidazole (oral)***<br>*Question relevant when: selected( ${q104_M43_full_list} , '5')* | |
| q604_M43a *(required)* | q604_M43a. What percent of patients require this item?<br>*Question relevant when: selected( ${q104_M43_full_list} , '5')*<br>*Response constrained to: .>0 and .<=100 or .=999* | |

| Field | Question | Answer |
|---|---|---|
| q604_M43b *(required)* | q604_M43b. How is the item administered, or given, to the patient?<br>*Question relevant when: selected( ${q104_M43_full_list} , '5')* | 1 Oral - solid (e.g. tablet)<br>2 Oral - liquid<br>3 Sublingual<br>4 Intravenous (IV)<br>5 Intramusculuar injection (IM)<br>6 Subcutaneous<br>7 Rectal<br>8 Inhaled (e.g. gas)<br>9 Inserted (e.g. vaginal pessaries)<br>10 Topical - cream<br>11 Topical - drops (e.g. eye drops)<br>12 Other<br>99 Don't know |
| q604_M43c *(required)* | q604_M43c. What is the item's smallest unit of measurement (mg, mcg, ml, g, etc)?<br>*Question relevant when: selected( ${q104_M43_full_list} , '5')* | microgram — microgram (mcg)<br>miligram — miligram (mg)<br>miligram_dissolved_in_liquid — miligram (mg) dissolved in liquid (e.g. 2 mg per 3 militers)<br>gram — gram (g)<br>militer — militer (ml)<br>centiliter — centiliter (cl)<br>litre — litre (l)<br>ml_of _solution — ml of solution (e.g. ml of 10% solution)<br>litre_of_solution — litre of solution (e.g. 1 litre of 5% solution)<br>cubic_meter — cubic meter (m^3)<br>other — other<br>dont_know — don't know |
| q604_M43c.1 | q604_M43c.1 If other unit of measurement or route of administration, specify here:<br>*Leave blank if not applicable* | |

| Field | Question | Answer |
|---|---|---|
| | *Question relevant when: selected( ${q104_M43_full_list} , '5')* | |
| q604_M43d *(required)* | q604_M43d. In total, how many units are used to treat a patient during all of her care?<br>*Remember to add all units! E.g. 5 days x 200 mg twice per day = 2,000mg*<br>*Question relevant when: selected( ${q104_M43_full_list} , '5')*<br>*Response constrained to: .>0* | |
| q604_M44 | ***q604_M44. Penicillin***<br>*Question relevant when: selected( ${q104_M44_full_list} , '5')* | |
| q604_M44a *(required)* | q604_M44a. What percent of patients require this item?<br>*Question relevant when: selected( ${q104_M44_full_list} , '5')*<br>*Response constrained to: .>0 and .<=100 or .=999* | |
| q604_M44b *(required)* | q604_M44b. How is the item administered, or given, to the patient?<br>*Question relevant when: selected( ${q104_M44_full_list} , '5')* | 1 Oral - solid (e.g. tablet)<br>2 Oral - liquid<br>3 Sublingual<br>4 Intravenous (IV)<br>5 Intramusculuar injection (IM)<br>6 Subcutaneous<br>7 Rectal<br>8 Inhaled (e.g. gas)<br>9 Inserted (e.g. vaginal pessaries)<br>10 Topical - cream<br>11 Topical - drops (e.g. eye drops)<br>12 Other<br>99 Don't know |
| q604_M44c *(required)* | q604_M44c. What is the item's smallest unit of measurement (mg, mcg, ml, g, etc)?<br>*Question relevant when: selected( ${q104_M44_full_list} , '5')* | microgram — microgram (mcg)<br>miligram — miligram (mg)<br>miligram_dissolved_in_liquid — miligram (mg) dissolved in liquid (e.g. 2 mg per 3 militers)<br>gram — gram (g)<br>militer — militer (ml)<br>centiliter — centiliter (cl)<br>litre — litre (l)<br>ml_of _solution — ml of solution (e.g. ml of 10% solution)<br>litre_of_solution — litre of solution (e.g. 1 litre of 5% solution)<br>cubic_meter — cubic meter (m^3)<br>other — other<br>dont_know — don't know |
| q604_M44c.1 | q604_M44c.1 If other unit of measurement or route of administration, specify here:<br>*Leave blank if not applicable* | |

| Field | Question | Answer |
|---|---|---|
| | *Question relevant when: selected( ${q104_M44_full_list} , '5')* | |
| q604_M44d *(required)* | q604_M44d. In total, how many units are used to treat a patient during all of her care?<br>*Remember to add all units! E.g. 5 days x 200 mg twice per day = 2,000mg*<br>*Question relevant when: selected( ${q104_M44_full_list} , '5')*<br>*Response constrained to: .>0* | |
| q604_M45 | ***q604_M45. Antibiotic - other 1: "[q104_M45_full_list_other]"***<br>*Question relevant when: selected( ${q104_M45_full_list} , '5')* | |
| q604_M45a *(required)* | q604_M45a. What percent of patients require this item?<br>*Question relevant when: selected( ${q104_M45_full_list} , '5')*<br>*Response constrained to: .>0 and .<=100 or .=999* | |

| Field | Question | Answer |
|---|---|---|
| q604_M45b *(required)* | q604_M45b. How is the item administered, or given, to the patient?<br>*Question relevant when: selected( ${q104_M45_full_list} , '5')* | 1 Oral - solid (e.g. tablet)<br>2 Oral - liquid<br>3 Sublingual<br>4 Intravenous (IV)<br>5 Intramusculuar injection (IM)<br>6 Subcutaneous<br>7 Rectal<br>8 Inhaled (e.g. gas)<br>9 Inserted (e.g. vaginal pessaries)<br>10 Topical - cream<br>11 Topical - drops (e.g. eye drops)<br>12 Other<br>99 Don't know |
| q604_M45c *(required)* | q604_M45c. What is the item's smallest unit of measurement (mg, mcg, ml, g, etc)?<br>*Question relevant when: selected( ${q104_M45_full_list} , '5')* | microgram — microgram (mcg)<br>miligram — miligram (mg)<br>miligram_dissolved_in_liquid — miligram (mg) dissolved in liquid (e.g. 2 mg per 3 militers)<br>gram — gram (g)<br>militer — militer (ml)<br>centiliter — centiliter (cl)<br>litre — litre (l)<br>ml_of _solution — ml of solution (e.g. ml of 10% solution)<br>litre_of_solution — litre of solution (e.g. 1 litre of 5% solution)<br>cubic_meter — cubic meter (m^3)<br>other — other<br>dont_know — don't know |
| q604_M45c.1 | q604_M45c.1 If other unit of measurement or route of administration, specify here:<br>*Leave blank if not applicable* | |

| Field | Question | Answer |
|---|---|---|
| | *Question relevant when: selected( ${q104_M45_full_list} , '5')* | |
| q604_M45d *(required)* | q604_M45d. In total, how many units are used to treat a patient during all of her care?<br>*Remember to add all units! E.g. 5 days x 200 mg twice per day = 2,000mg*<br>*Question relevant when: selected( ${q104_M45_full_list} , '5')*<br>*Response constrained to: .>0* | |
| q604_M46 | ***q604_M46. Antibiotic - other 2: "[q104_M46_full_list_other]"***<br>*Question relevant when: selected( ${q104_M46_full_list} , '5')* | |
| q604_M46a *(required)* | q604_M46a. What percent of patients require this item?<br>*Question relevant when: selected( ${q104_M46_full_list} , '5')*<br>*Response constrained to: .>0 and .<=100 or .=999* | |

| Field | Question | Answer |
|---|---|---|
| q604_M46b *(required)* | q604_M46b. How is the item administered, or given, to the patient?<br>*Question relevant when: selected( ${q104_M46_full_list} , '5')* | 1 Oral - solid (e.g. tablet)<br>2 Oral - liquid<br>3 Sublingual<br>4 Intravenous (IV)<br>5 Intramusculuar injection (IM)<br>6 Subcutaneous<br>7 Rectal<br>8 Inhaled (e.g. gas)<br>9 Inserted (e.g. vaginal pessaries)<br>10 Topical - cream<br>11 Topical - drops (e.g. eye drops)<br>12 Other<br>99 Don't know |
| q604_M46c *(required)* | q604_M46c. What is the item's smallest unit of measurement (mg, mcg, ml, g, etc)?<br>*Question relevant when: selected( ${q104_M46_full_list} , '5')* | microgram — microgram (mcg)<br>miligram — miligram (mg)<br>miligram_dissolved_in_liquid — miligram (mg) dissolved in liquid (e.g. 2 mg per 3 militers)<br>gram — gram (g)<br>militer — militer (ml)<br>centiliter — centiliter (cl)<br>litre — litre (l)<br>ml_of _solution — ml of solution (e.g. ml of 10% solution)<br>litre_of_solution — litre of solution (e.g. 1 litre of 5% solution)<br>cubic_meter — cubic meter (m^3)<br>other — other<br>dont_know — don't know |
| q604_M46c.1 | q604_M46c.1 If other unit of measurement or route of administration, specify here:<br>*Leave blank if not applicable* | |

| Field | Question | Answer |
|---|---|---|
| | *Question relevant when: selected( ${q104_M46_full_list} , '5')* | |
| q604_M46d *(required)* | q604_M46d. In total, how many units are used to treat a patient during all of her care?<br>*Remember to add all units! E.g. 5 days x 200 mg twice per day = 2,000mg*<br>*Question relevant when: selected( ${q104_M46_full_list} , '5')*<br>*Response constrained to: .>0* | |
| q604_M47 | ***q604_M47. Antibiotic - other 3: "[q104_M47_full_list_other]"***<br>*Question relevant when: selected( ${q104_M47_full_list} , '5')* | |
| q604_M47a *(required)* | q604_M47a. What percent of patients require this item?<br>*Question relevant when: selected( ${q104_M47_full_list} , '5')*<br>*Response constrained to: .>0 and .<=100 or .=999* | |

| Field | Question | Answer |
|---|---|---|
| q604_M47b *(required)* | q604_M47b. How is the item administered, or given, to the patient?<br>*Question relevant when: selected( ${q104_M47_full_list} , '5')* | 1 Oral - solid (e.g. tablet)<br>2 Oral - liquid<br>3 Sublingual<br>4 Intravenous (IV)<br>5 Intramusculuar injection (IM)<br>6 Subcutaneous<br>7 Rectal<br>8 Inhaled (e.g. gas)<br>9 Inserted (e.g. vaginal pessaries)<br>10 Topical - cream<br>11 Topical - drops (e.g. eye drops)<br>12 Other<br>99 Don't know |
| q604_M47c *(required)* | q604_M47c. What is the item's smallest unit of measurement (mg, mcg, ml, g, etc)?<br>*Question relevant when: selected( ${q104_M47_full_list} , '5')* | microgram: microgram (mcg)<br>miligram: miligram (mg)<br>miligram_dissolved_in_liquid: miligram (mg) dissolved in liquid (e.g. 2 mg per 3 militers)<br>gram: gram (g)<br>militer: militer (ml)<br>centiliter: centiliter (cl)<br>litre: litre (l)<br>ml_of _solution: ml of solution (e.g. ml of 10% solution)<br>litre_of_solution: litre of solution (e.g. 1 litre of 5% solution)<br>cubic_meter: cubic meter (m^3)<br>other: other<br>dont_know: don't know |
| q604_M47c.1 | q604_M47c.1 If other unit of measurement or route of administration, specify here:<br>*Leave blank if not applicable* | |

| Field | Question | Answer |
|---|---|---|
| | *Question relevant when: selected( ${q104_M47_full_list} , '5')* | |
| q604_M47d *(required)* | q604_M47d. In total, how many units are used to treat a patient during all of her care?<br>*Remember to add all units! E.g. 5 days x 200 mg twice per day = 2,000mg*<br>*Question relevant when: selected( ${q104_M47_full_list} , '5')*<br>*Response constrained to: .>0* | |
| q604_M48 | ***q604_M48. Antibiotic - other 4: "[q104_M48_full_list_other]"***<br>*Question relevant when: selected( ${q104_M48_full_list} , '5')* | |
| q604_M48a *(required)* | q604_M48a. What percent of patients require this item?<br>*Question relevant when: selected( ${q104_M48_full_list} , '5')*<br>*Response constrained to: .>0 and .<=100 or .=999* | |
| q604_M48b *(required)* | q604_M48b. How is the item administered, or given, to the patient?<br>*Question relevant when: selected( ${q104_M48_full_list} , '5')* | 1 Oral - solid (e.g. tablet)<br>2 Oral - liquid<br>3 Sublingual<br>4 Intravenous (IV)<br>5 Intramusculuar injection (IM)<br>6 Subcutaneous<br>7 Rectal<br>8 Inhaled (e.g. gas)<br>9 Inserted (e.g. vaginal pessaries)<br>10 Topical - cream<br>11 Topical - drops (e.g. eye drops)<br>12 Other<br>99 Don't know |
| q604_M48c *(required)* | q604_M48c. What is the item's smallest unit of measurement (mg, mcg, ml, g, etc)?<br>*Question relevant when: selected( ${q104_M48_full_list} , '5')* | microgram: microgram (mcg)<br>miligram: miligram (mg)<br>miligram_dissolved_in_liquid: miligram (mg) dissolved in liquid (e.g. 2 mg per 3 militers)<br>gram: gram (g)<br>militer: militer (ml)<br>centiliter: centiliter (cl)<br>litre: litre (l)<br>ml_of _solution: ml of solution (e.g. ml of 10% solution)<br>litre_of_solution: litre of solution (e.g. 1 litre of 5% solution)<br>cubic_meter: cubic meter (m^3)<br>other: other<br>dont_know: don't know |
| q604_M48c.1 | q604_M48c.1 If other unit of measurement or route of administration, specify here:<br>*Leave blank if not applicable* | |

| Field | Question | Answer |
|---|---|---|
| | *Question relevant when: selected( ${q104_M48_full_list} , '5')* | |
| q604_M48d *(required)* | q604_M48d. In total, how many units are used to treat a patient during all of her care?<br>*Remember to add all units! E.g. 5 days x 200 mg twice per day = 2,000mg*<br>*Question relevant when: selected( ${q104_M48_full_list} , '5')*<br>*Response constrained to: .>0* | |
| q604_M49 | ***q604_M49. Antibiotic - other 5: "[q104_M49_full_list_other]"***<br>*Question relevant when: selected( ${q104_M49_full_list} , '5')* | |
| q604_M49a *(required)* | q604_M49a. What percent of patients require this item?<br>*Question relevant when: selected( ${q104_M49_full_list} , '5')*<br>*Response constrained to: .>0 and .<=100 or .=999* | |
| q604_M49b *(required)* | q604_M49b. How is the item administered, or given, to the patient?<br>*Question relevant when: selected( ${q104_M49_full_list} , '5')* | 1 Oral - solid (e.g. tablet)<br>2 Oral - liquid<br>3 Sublingual<br>4 Intravenous (IV)<br>5 Intramusculuar injection (IM)<br>6 Subcutaneous<br>7 Rectal<br>8 Inhaled (e.g. gas)<br>9 Inserted (e.g. vaginal pessaries)<br>10 Topical - cream<br>11 Topical - drops (e.g. eye drops)<br>12 Other<br>99 Don't know |
| q604_M49c *(required)* | q604_M49c. What is the item's smallest unit of measurement (mg, mcg, ml, g, etc)?<br>*Question relevant when: selected( ${q104_M49_full_list} , '5')* | microgram — microgram (mcg)<br>miligram — miligram (mg)<br>miligram_dissolved_in_liquid — miligram (mg) dissolved in liquid (e.g. 2 mg per 3 militers)<br>gram — gram (g)<br>militer — militer (ml)<br>centiliter — centiliter (cl)<br>litre — litre (l)<br>ml_of _solution — ml of solution (e.g. ml of 10% solution)<br>litre_of_solution — litre of solution (e.g. 1 litre of 5% solution)<br>cubic_meter — cubic meter (m^3)<br>other — other<br>dont_know — don't know |
| q604_M49c.1 | q604_M49c.1 If other unit of measurement or route of administration, specify here:<br>*Leave blank if not applicable* | |

| Field | Question | Answer |
|---|---|---|
| | *Question relevant when: selected( ${q104_M49_full_list} , '5')* | |
| q604_M49d *(required)* | q604_M49d. In total, how many units are used to treat a patient during all of her care?<br>*Remember to add all units! E.g. 5 days x 200 mg twice per day = 2,000mg*<br>*Question relevant when: selected( ${q104_M49_full_list} , '5')*<br>*Response constrained to: .>0* | |
| <p><strong><em>D. Medications - Perforations (4)</em></strong></p><br>*Group relevant when: selected( ${section_six_skip_med} , '1')* | | |
| q604_med_note4 | 604 CONTINUED For each of the following medications and other medical products that are used for perforations, can you tell me …? | |
| note_604_antiseptic | ***Antiseptic*** | |
| q604_M50 | ***q604_M50. Cetrimide 15% ("Savlon")***<br>*Question relevant when: selected( ${q104_M50_full_list} , '5')* | |
| q604_M50a *(required)* | q604_M50a. What percent of patients require this item?<br>*Question relevant when: selected( ${q104_M50_full_list} , '5')*<br>*Response constrained to: .>0 and .<=100 or .=999* | |

| Field | Question | Answer |
|---|---|---|
| q604_M50b *(required)* | q604_M50b. How is the item administered, or given, to the patient?<br>*Question relevant when: selected( ${q104_M50_full_list} , '5')* | 1 Oral - solid (e.g. tablet)<br>2 Oral - liquid<br>3 Sublingual<br>4 Intravenous (IV)<br>5 Intramusculuar injection (IM)<br>6 Subcutaneous<br>7 Rectal<br>8 Inhaled (e.g. gas)<br>9 Inserted (e.g. vaginal pessaries)<br>10 Topical - cream<br>11 Topical - drops (e.g. eye drops)<br>12 Other<br>99 Don't know |
| q604_M50c *(required)* | q604_M50c. What is the item's smallest unit of measurement (mg, mcg, ml, g, etc)?<br>*Question relevant when: selected( ${q104_M50_full_list} , '5')* | microgram — microgram (mcg)<br>miligram — miligram (mg)<br>miligram_dissolved_in_liquid — miligram (mg) dissolved in liquid (e.g. 2 mg per 3 militers)<br>gram — gram (g)<br>militer — militer (ml)<br>centiliter — centiliter (cl)<br>litre — litre (l)<br>ml_of _solution — ml of solution (e.g. ml of 10% solution)<br>litre_of_solution — litre of solution (e.g. 1 litre of 5% solution)<br>cubic_meter — cubic meter (m^3)<br>other — other<br>dont_know — don't know |
| q604_M50c.1 | q604_M50c.1 If other unit of measurement or route of administration, specify here:<br>*Leave blank if not applicable* | |

| Field | Question | Answer |
|---|---|---|
| | *Question relevant when: selected( ${q104_M50_full_list} , '5')* | |
| q604_M50d *(required)* | q604_M50d. In total, how many units are used to treat a patient during all of her care?<br>*Remember to add all units! E.g. 5 days x 200 mg twice per day = 2,000mg*<br>*Question relevant when: selected( ${q104_M50_full_list} , '5')*<br>*Response constrained to: .>0* | |
| q604_M51 | ***q604_M51. Povidone-iodine solution, 7.5%***<br>*Question relevant when: selected( ${q104_M51_full_list} , '5')* | |
| q604_M51a *(required)* | q604_M51a. What percent of patients require this item?<br>*Question relevant when: selected( ${q104_M51_full_list} , '5')*<br>*Response constrained to: .>0 and .<=100 or .=999* | |
| q604_M51b *(required)* | q604_M51b. How is the item administered, or given, to the patient?<br>*Question relevant when: selected( ${q104_M51_full_list} , '5')* | 1 Oral - solid (e.g. tablet)<br>2 Oral - liquid<br>3 Sublingual<br>4 Intravenous (IV)<br>5 Intramusculuar injection (IM)<br>6 Subcutaneous<br>7 Rectal<br>8 Inhaled (e.g. gas)<br>9 Inserted (e.g. vaginal pessaries)<br>10 Topical - cream<br>11 Topical - drops (e.g. eye drops)<br>12 Other<br>99 Don't know |
| q604_M51c *(required)* | q604_M51c. What is the item's smallest unit of measurement (mg, mcg, ml, g, etc)?<br>*Question relevant when: selected( ${q104_M51_full_list} , '5')* | microgram — microgram (mcg)<br>miligram — miligram (mg)<br>miligram_dissolved_in_liquid — miligram (mg) dissolved in liquid (e.g. 2 mg per 3 militers)<br>gram — gram (g)<br>militer — militer (ml)<br>centiliter — centiliter (cl)<br>litre — litre (l)<br>ml_of _solution — ml of solution (e.g. ml of 10% solution)<br>litre_of_solution — litre of solution (e.g. 1 litre of 5% solution)<br>cubic_meter — cubic meter (m^3)<br>other — other<br>dont_know — don't know |
| q604_M51c.1 | q604_M51c.1 If other unit of measurement or route of administration, specify here:<br>*Leave blank if not applicable* | |

| Field | Question | Answer |
|---|---|---|
| | *Question relevant when: selected( ${q104_M51_full_list} , '5')* | |
| q604_M51d *(required)* | q604_M51d. In total, how many units are used to treat a patient during all of her care?<br>*Remember to add all units! E.g. 5 days x 200 mg twice per day = 2,000mg*<br>*Question relevant when: selected( ${q104_M51_full_list} , '5')*<br>*Response constrained to: .>0* | |
| q604_M52 | ***q604_M52. Antiseptic - other 1: "[q104_M52_full_list_other]"***<br>*Question relevant when: selected( ${q104_M52_full_list} , '5')* | |
| q604_M52a *(required)* | q604_M52a. What percent of patients require this item?<br>*Question relevant when: selected( ${q104_M52_full_list} , '5')*<br>*Response constrained to: .>0 and .<=100 or .=999* | |
| q604_M52b *(required)* | q604_M52b. How is the item administered, or given, to the patient?<br>*Question relevant when: selected( ${q104_M52_full_list} , '5')* | 1 Oral - solid (e.g. tablet)<br>2 Oral - liquid<br>3 Sublingual<br>4 Intravenous (IV)<br>5 Intramusculuar injection (IM)<br>6 Subcutaneous<br>7 Rectal<br>8 Inhaled (e.g. gas)<br>9 Inserted (e.g. vaginal pessaries)<br>10 Topical - cream<br>11 Topical - drops (e.g. eye drops)<br>12 Other<br>99 Don't know |
| q604_M52c *(required)* | q604_M52c. What is the item's smallest unit of measurement (mg, mcg, ml, g, etc)?<br>*Question relevant when: selected( ${q104_M52_full_list} , '5')* | microgram — microgram (mcg)<br>miligram — miligram (mg)<br>miligram_dissolved_in_liquid — miligram (mg) dissolved in liquid (e.g. 2 mg per 3 militers)<br>gram — gram (g)<br>militer — militer (ml)<br>centiliter — centiliter (cl)<br>litre — litre (l)<br>ml_of _solution — ml of solution (e.g. ml of 10% solution)<br>litre_of_solution — litre of solution (e.g. 1 litre of 5% solution)<br>cubic_meter — cubic meter (m^3)<br>other — other<br>dont_know — don't know |
| q604_M52c.1 | q604_M52c.1 If other unit of measurement or route of administration, specify here:<br>*Leave blank if not applicable* | |

| Field | Question | Answer |
|---|---|---|
| | *Question relevant when: selected( ${q104_M52_full_list} , '5')* | |
| q604_M52d *(required)* | q604_M52d. In total, how many units are used to treat a patient during all of her care? | |
| | *Remember to add all units! E.g. 5 days x 200 mg twice per day = 2,000mg*<br>*Question relevant when: selected( ${q104_M52_full_list} , '5')*<br>*Response constrained to: .>0* | |
| q604_M53 | ***q604_M53. Antiseptic - other 2: "[q104_M53_full_list_other]"***<br>*Question relevant when: selected( ${q104_M53_full_list} , '5')* | |
| q604_M53a *(required)* | q604_M53a. What percent of patients require this item?<br>*Question relevant when: selected( ${q104_M53_full_list} , '5')*<br>*Response constrained to: .>0 and .<=100 or .=999* | |
| q604_M53b *(required)* | q604_M53b. How is the item administered, or given, to the patient?<br>*Question relevant when: selected( ${q104_M53_full_list} , '5')* | 1 Oral - solid (e.g. tablet)<br>2 Oral - liquid<br>3 Sublingual<br>4 Intravenous (IV)<br>5 Intramusculuar injection (IM)<br>6 Subcutaneous<br>7 Rectal<br>8 Inhaled (e.g. gas)<br>9 Inserted (e.g. vaginal pessaries)<br>10 Topical - cream<br>11 Topical - drops (e.g. eye drops)<br>12 Other<br>99 Don't know |
| q604_M53c *(required)* | q604_M53c. What is the item's smallest unit of measurement (mg, mcg, ml, g, etc)?<br>*Question relevant when: selected( ${q104_M53_full_list} , '5')* | microgram — microgram (mcg)<br>miligram — miligram (mg)<br>miligram_dissolved_in_liquid — miligram (mg) dissolved in liquid (e.g. 2 mg per 3 militers)<br>gram — gram (g)<br>militer — militer (ml)<br>centiliter — centiliter (cl)<br>litre — litre (l)<br>ml_of _solution — ml of solution (e.g. ml of 10% solution)<br>litre_of_solution — litre of solution (e.g. 1 litre of 5% solution)<br>cubic_meter — cubic meter (m^3)<br>other — other<br>dont_know — don't know |
| q604_M53c.1 | q604_M53c.1 If other unit of measurement or route of administration, specify here:<br>*Leave blank if not applicable* | |

| Field | Question | Answer |
|---|---|---|
| | *Question relevant when: selected( ${q104_M53_full_list} , '5')* | |
| q604_M53d *(required)* | q604_M53d. In total, how many units are used to treat a patient during all of her care?<br>*Remember to add all units! E.g. 5 days x 200 mg twice per day = 2,000mg*<br>*Question relevant when: selected( ${q104_M53_full_list} , '5')*<br>*Response constrained to: .>0* | |
| q604_M54 | ***q604_M54. Antiseptic - other 3: "[q104_M54_full_list_other]"***<br>*Question relevant when: selected( ${q104_M54_full_list} , '5')* | |
| q604_M54a *(required)* | q604_M54a. What percent of patients require this item?<br>*Question relevant when: selected( ${q104_M54_full_list} , '5')*<br>*Response constrained to: .>0 and .<=100 or .=999* | |
| q604_M54b *(required)* | q604_M54b. How is the item administered, or given, to the patient?<br>*Question relevant when: selected( ${q104_M54_full_list} , '5')* | 1 Oral - solid (e.g. tablet)<br>2 Oral - liquid<br>3 Sublingual<br>4 Intravenous (IV)<br>5 Intramusculuar injection (IM)<br>6 Subcutaneous<br>7 Rectal<br>8 Inhaled (e.g. gas)<br>9 Inserted (e.g. vaginal pessaries)<br>10 Topical - cream<br>11 Topical - drops (e.g. eye drops)<br>12 Other<br>99 Don't know |
| q604_M54c *(required)* | q604_M54c. What is the item's smallest unit of measurement (mg, mcg, ml, g, etc)?<br>*Question relevant when: selected( ${q104_M54_full_list} , '5')* | microgram — microgram (mcg)<br>miligram — miligram (mg)<br>miligram_dissolved_in_liquid — miligram (mg) dissolved in liquid (e.g. 2 mg per 3 militers)<br>gram — gram (g)<br>militer — militer (ml)<br>centiliter — centiliter (cl)<br>litre — litre (l)<br>ml_of _solution — ml of solution (e.g. ml of 10% solution)<br>litre_of_solution — litre of solution (e.g. 1 litre of 5% solution)<br>cubic_meter — cubic meter (m^3)<br>other — other<br>dont_know — don't know |
| q604_M54c.1 | q604_M54c.1 If other unit of measurement or route of administration, specify here:<br>*Leave blank if not applicable* | |

| Field | Question | Answer |
|---|---|---|
| | *Question relevant when: selected( ${q104_M54_full_list} , '5')* | |
| q604_M54d *(required)* | q604_M54d. In total, how many units are used to treat a patient during all of her care?<br>*Remember to add all units! E.g. 5 days x 200 mg twice per day = 2,000mg*<br>*Question relevant when: selected( ${q104_M54_full_list} , '5')*<br>*Response constrained to: .>0* | |
| q604_M54.1 | ***q604_M54.1. Antiseptic - other 4: "[q104_M54.1_full_list_other]"***<br>*Question relevant when: selected( ${q104_M54.1_full_list} , '2')* | |
| q604_M54.1a *(required)* | q604_M54.1a. What percent of patients require this item?<br>*Question relevant when: selected( ${q104_M54.1_full_list} , '2')*<br>*Response constrained to: .>0 and .<=100 or .=999* | |

| Field | Question | Answer |
|---|---|---|
| q604_M54.1b *(required)* | q604_M54.1b. How is the item administered, or given, to the patient?<br>*Question relevant when: selected( ${q104_M54.1_full_list} , '2')* | 1 Oral - solid (e.g. tablet)<br>2 Oral - liquid<br>3 Sublingual<br>4 Intravenous (IV)<br>5 Intramusculuar injection (IM)<br>6 Subcutaneous<br>7 Rectal<br>8 Inhaled (e.g. gas)<br>9 Inserted (e.g. vaginal pessaries)<br>10 Topical - cream<br>11 Topical - drops (e.g. eye drops)<br>12 Other<br>99 Don't know |
| q604_M54.1c *(required)* | q604_M54.1c. What is the item's smallest unit of measurement (mg, mcg, ml, g, etc)?<br>*Question relevant when: selected( ${q104_M54.1_full_list} , '2')* | microgram: microgram (mcg)<br>miligram: miligram (mg)<br>miligram_dissolved_in_liquid: miligram (mg) dissolved in liquid (e.g. 2 mg per 3 militers)<br>gram: gram (g)<br>militer: militer (ml)<br>centiliter: centiliter (cl)<br>litre: litre (l)<br>ml_of _solution: ml of solution (e.g. ml of 10% solution)<br>litre_of_solution: litre of solution (e.g. 1 litre of 5% solution)<br>cubic_meter: cubic meter (m^3)<br>other: other<br>dont_know: don't know |
| q604_M54.1c.1 | q604_M54.1c.1 If other unit of measurement or route of administration, specify here:<br>*Leave blank if not applicable* | |

Leave blank if not applicable

| Field | Question | Answer |
|---|---|---|
| | *Question relevant when: selected( ${q104_M54.1_full_list} , '2')* | |
| q604_M54.1d *(required)* | q604_M54.1d. In total, how many units are used to treat a patient during all of her care?<br>*Remember to add all units! E.g. 5 days x 200 mg twice per day = 2,000mg*<br>*Question relevant when: selected( ${q104_M54.1_full_list} , '2')*<br>*Response constrained to: .>0* | |
| q604_M54.2 | ***q604_M54.2. Antiseptic - other 5: "[q104_M54.2_full_list_other]"***<br>*Question relevant when: selected( ${q104_M54.2_full_list} , '2')* | |
| q604_M54.2a *(required)* | q604_M54.2a. What percent of patients require this item?<br>*Question relevant when: selected( ${q104_M54.2_full_list} , '2')*<br>*Response constrained to: .>0 and .<=100 or .=999* | |

| Field | Question | Answer |
|---|---|---|
| q604_M54.2b *(required)* | q604_M54.2b. How is the item administered, or given, to the patient?<br>*Question relevant when: selected( ${q104_M54.2_full_list} , '2')* | 1 Oral - solid (e.g. tablet)<br>2 Oral - liquid<br>3 Sublingual<br>4 Intravenous (IV)<br>5 Intramusculuar injection (IM)<br>6 Subcutaneous<br>7 Rectal<br>8 Inhaled (e.g. gas)<br>9 Inserted (e.g. vaginal pessaries)<br>10 Topical - cream<br>11 Topical - drops (e.g. eye drops)<br>12 Other<br>99 Don't know |
| q604_M54.2c *(required)* | q604_M54.2c. What is the item's smallest unit of measurement (mg, mcg, ml, g, etc)?<br>*Question relevant when: selected( ${q104_M54.2_full_list} , '2')* | microgram — microgram (mcg)<br>miligram — miligram (mg)<br>miligram_dissolved_in_liquid — miligram (mg) dissolved in liquid (e.g. 2 mg per 3 militers)<br>gram — gram (g)<br>militer — militer (ml)<br>centiliter — centiliter (cl)<br>litre — litre (l)<br>ml_of _solution — ml of solution (e.g. ml of 10% solution)<br>litre_of_solution — litre of solution (e.g. 1 litre of 5% solution)<br>cubic_meter — cubic meter (m^3)<br>other — other<br>dont_know — don't know |
| q604_M54.2c.1 | q604_M54.2c.1 If other unit of measurement or route of administration, specify here:<br>*Leave blank if not applicable* | |

| Field | Question | Answer |
|---|---|---|
| | *Leave blank if not applicable*<br>*Question relevant when: selected( ${q104_M54.2_full_list} , '2')* | |
| q604_M54.2d *(required)* | q604_M54.2d. In total, how many units are used to treat a patient during all of her care?<br>*Remember to add all units! E.g. 5 days x 200 mg twice per day = 2,000mg*<br>*Question relevant when: selected( ${q104_M54.2_full_list} , '2')*<br>*Response constrained to: .>0* | |
| <p><strong><em>D. Medications - Perforations (5)</em></strong></p><br>*Group relevant when: selected( ${section_six_skip_med} , '1')* | | |
| q604_med_notE4 | 604 CONTINUED For each of the following medications and other medical products that are used for perforations, can you tell me …? | |
| note_604_antispas | ***Antispasmodic, anxiolytic, sedative, etc*** | |
| q604_M55 | ***q604_M55. Hyoscine butylbromide / scopolamine butilhisocin (Buscopan )***<br>*Question relevant when: selected( ${q104_M55_full_list} , '5')* | |
| q604_M55a *(required)* | q604_M55a. What percent of patients require this item?<br>*Question relevant when: selected( ${q104_M55_full_list} , '5')*<br>*Response constrained to: .>0 and .<=100 or .=999* | |
| q604_M55b *(required)* | q604_M55b. How is the item administered, or given, to the patient?<br>*Question relevant when: selected( ${q104_M55_full_list} , '5')* | 1 Oral - solid (e.g. tablet)<br>2 Oral - liquid<br>3 Sublingual<br>4 Intravenous (IV)<br>5 Intramusculuar injection (IM)<br>6 Subcutaneous<br>7 Rectal<br>8 Inhaled (e.g. gas)<br>9 Inserted (e.g. vaginal pessaries)<br>10 Topical - cream<br>11 Topical - drops (e.g. eye drops)<br>12 Other<br>99 Don't know |
| q604_M55c *(required)* | q604_M55c. What is the item's smallest unit of measurement (mg, mcg, ml, g, etc)?<br>*Question relevant when: selected( ${q104_M55_full_list} , '5')* | microgram — microgram (mcg)<br>miligram — miligram (mg)<br>miligram_dissolved_in_liquid — miligram (mg) dissolved in liquid (e.g. 2 mg per 3 militers)<br>gram — gram (g)<br>militer — militer (ml)<br>centiliter — centiliter (cl)<br>litre — litre (l)<br>ml_of _solution — ml of solution (e.g. ml of 10% solution)<br>litre_of_solution — litre of solution (e.g. 1 litre of 5% solution)<br>cubic_meter — cubic meter (m^3)<br>other — other<br>dont_know — don't know |
| q604_M55c.1 | q604_M55c.1 If other unit of measurement or route of administration, specify here:<br>*Leave blank if not applicable* | |

Leave blank if not applicable

| Field | Question | Answer |
|---|---|---|
| | *Question relevant when: selected( ${q104_M55_full_list} , '5')* | |
| q604_M55d *(required)* | q604_M55d. In total, how many units are used to treat a patient during all of her care?<br>*Remember to add all units! E.g. 5 days x 200 mg twice per day = 2,000mg*<br>*Question relevant when: selected( ${q104_M55_full_list} , '5')*<br>*Response constrained to: .>0* | |
| q604_M56 | ***q604_M56. Atropine sulfate (or sulphate)***<br>*Question relevant when: selected( ${q104_M56_full_list} , '5')* | |
| q604_M56a *(required)* | q604_M56a. What percent of patients require this item?<br>*Question relevant when: selected( ${q104_M56_full_list} , '5')*<br>*Response constrained to: .>0 and .<=100 or .=999* | |

| Field | Question | Answer |
|---|---|---|
| q604_M56b *(required)* | q604_M56b. How is the item administered, or given, to the patient?<br>*Question relevant when: selected( ${q104_M56_full_list} , '5')* | 1 Oral - solid (e.g. tablet)<br>2 Oral - liquid<br>3 Sublingual<br>4 Intravenous (IV)<br>5 Intramusculuar injection (IM)<br>6 Subcutaneous<br>7 Rectal<br>8 Inhaled (e.g. gas)<br>9 Inserted (e.g. vaginal pessaries)<br>10 Topical - cream<br>11 Topical - drops (e.g. eye drops)<br>12 Other<br>99 Don't know |
| q604_M56c *(required)* | q604_M56c. What is the item's smallest unit of measurement (mg, mcg, ml, g, etc)?<br>*Question relevant when: selected( ${q104_M56_full_list} , '5')* | microgram — microgram (mcg)<br>miligram — miligram (mg)<br>miligram_dissolved_in_liquid — miligram (mg) dissolved in liquid (e.g. 2 mg per 3 militers)<br>gram — gram (g)<br>militer — militer (ml)<br>centiliter — centiliter (cl)<br>litre — litre (l)<br>ml_of _solution — ml of solution (e.g. ml of 10% solution)<br>litre_of_solution — litre of solution (e.g. 1 litre of 5% solution)<br>cubic_meter — cubic meter (m^3)<br>other — other<br>dont_know — don't know |
| q604_M56c.1 | q604_M56c.1 If other unit of measurement or route of administration, specify here:<br>*Leave blank if not applicable* | |

Leave blank if not applicable

| Field | Question | Answer |
|---|---|---|
| | *Question relevant when: selected( ${q104_M56_full_list} , '5')* | |
| q604_M56d *(required)* | q604_M56d. In total, how many units are used to treat a patient during all of her care?<br>*Remember to add all units! E.g. 5 days x 200 mg twice per day = 2,000mg*<br>*Question relevant when: selected( ${q104_M56_full_list} , '5')*<br>*Response constrained to: .>0* | |
| q604_M57 | ***q604_M57. Papaverine HCl***<br>*Question relevant when: selected( ${q104_M57_full_list} , '5')* | |
| q604_M57a *(required)* | q604_M57a. What percent of patients require this item?<br>*Question relevant when: selected( ${q104_M57_full_list} , '5')*<br>*Response constrained to: .>0 and .<=100 or .=999* | |
| q604_M57b *(required)* | q604_M57b. How is the item administered, or given, to the patient?<br>*Question relevant when: selected( ${q104_M57_full_list} , '5')* | 1 Oral - solid (e.g. tablet)<br>2 Oral - liquid<br>3 Sublingual<br>4 Intravenous (IV)<br>5 Intramusculuar injection (IM)<br>6 Subcutaneous<br>7 Rectal<br>8 Inhaled (e.g. gas)<br>9 Inserted (e.g. vaginal pessaries)<br>10 Topical - cream<br>11 Topical - drops (e.g. eye drops)<br>12 Other<br>99 Don't know |
| q604_M57c *(required)* | q604_M57c. What is the item's smallest unit of measurement (mg, mcg, ml, g, etc)?<br>*Question relevant when: selected( ${q104_M57_full_list} , '5')* | microgram — microgram (mcg)<br>miligram — miligram (mg)<br>miligram_dissolved_in_liquid — miligram (mg) dissolved in liquid (e.g. 2 mg per 3 militers)<br>gram — gram (g)<br>militer — militer (ml)<br>centiliter — centiliter (cl)<br>litre — litre (l)<br>ml_of _solution — ml of solution (e.g. ml of 10% solution)<br>litre_of_solution — litre of solution (e.g. 1 litre of 5% solution)<br>cubic_meter — cubic meter (m^3)<br>other — other<br>dont_know — don't know |
| q604_M57c.1 | q604_M57c.1 If other unit of measurement or route of administration, specify here:<br>*Leave blank if not applicable* | |

Leave blank if not applicable

| Field | Question | Answer |
|---|---|---|
| | *Question relevant when: selected( ${q104_M57_full_list} , '5')* | |
| q604_M57d *(required)* | q604_M57d. In total, how many units are used to treat a patient during all of her care?<br>*Remember to add all units! E.g. 5 days x 200 mg twice per day = 2,000mg*<br>*Question relevant when: selected( ${q104_M57_full_list} , '5')*<br>*Response constrained to: .>0* | |
| q604_M58 | ***q604_M58. Diazepam (injection)***<br>*Question relevant when: selected( ${q104_M58_full_list} , '5')* | |
| q604_M58a *(required)* | q604_M58a. What percent of patients require this item?<br>*Question relevant when: selected( ${q104_M58_full_list} , '5')*<br>*Response constrained to: .>0 and .<=100 or .=999* | |
| q604_M58b *(required)* | q604_M58b. How is the item administered, or given, to the patient?<br>*Question relevant when: selected( ${q104_M58_full_list} , '5')* | 1 Oral - solid (e.g. tablet)<br>2 Oral - liquid<br>3 Sublingual<br>4 Intravenous (IV)<br>5 Intramusculuar injection (IM)<br>6 Subcutaneous<br>7 Rectal<br>8 Inhaled (e.g. gas)<br>9 Inserted (e.g. vaginal pessaries)<br>10 Topical - cream<br>11 Topical - drops (e.g. eye drops)<br>12 Other<br>99 Don't know |
| q604_M58c *(required)* | q604_M58c. What is the item's smallest unit of measurement (mg, mcg, ml, g, etc)?<br>*Question relevant when: selected( ${q104_M58_full_list} , '5')* | microgram — microgram (mcg)<br>miligram — miligram (mg)<br>miligram_dissolved_in_liquid — miligram (mg) dissolved in liquid (e.g. 2 mg per 3 militers)<br>gram — gram (g)<br>militer — militer (ml)<br>centiliter — centiliter (cl)<br>litre — litre (l)<br>ml_of _solution — ml of solution (e.g. ml of 10% solution)<br>litre_of_solution — litre of solution (e.g. 1 litre of 5% solution)<br>cubic_meter — cubic meter (m^3)<br>other — other<br>dont_know — don't know |
| q604_M58c.1 | q604_M58c.1 If other unit of measurement or route of administration, specify here:<br>*Leave blank if not applicable* | |

*Leave blank if not applicable*

| Field | Question | Answer |
|---|---|---|
| | *Question relevant when: selected( ${q104_M58_full_list} , '5')* | |
| q604_M58d *(required)* | q604_M58d. In total, how many units are used to treat a patient during all of her care?<br>*Remember to add all units! E.g. 5 days x 200 mg twice per day = 2,000mg*<br>*Question relevant when: selected( ${q104_M58_full_list} , '5')*<br>*Response constrained to: .>0* | |
| q604_M59 | ***q604_M59. Diazepam (oral)***<br>*Question relevant when: selected( ${q104_M59_full_list} , '5')* | |
| q604_M59a *(required)* | q604_M59a. What percent of patients require this item?<br>*Question relevant when: selected( ${q104_M59_full_list} , '5')*<br>*Response constrained to: .>0 and .<=100 or .=999* | |

| Field | Question | Answer |
|---|---|---|
| q604_M59b *(required)* | q604_M59b. How is the item administered, or given, to the patient?<br>*Question relevant when: selected( ${q104_M59_full_list} , '5')* | 1 Oral - solid (e.g. tablet)<br>2 Oral - liquid<br>3 Sublingual<br>4 Intravenous (IV)<br>5 Intramusculuar injection (IM)<br>6 Subcutaneous<br>7 Rectal<br>8 Inhaled (e.g. gas)<br>9 Inserted (e.g. vaginal pessaries)<br>10 Topical - cream<br>11 Topical - drops (e.g. eye drops)<br>12 Other<br>99 Don't know |
| q604_M59c *(required)* | q604_M59c. What is the item's smallest unit of measurement (mg, mcg, ml, g, etc)?<br>*Question relevant when: selected( ${q104_M59_full_list} , '5')* | microgram — microgram (mcg)<br>miligram — miligram (mg)<br>miligram_dissolved_in_liquid — miligram (mg) dissolved in liquid (e.g. 2 mg per 3 militers)<br>gram — gram (g)<br>militer — militer (ml)<br>centiliter — centiliter (cl)<br>litre — litre (l)<br>ml_of _solution — ml of solution (e.g. ml of 10% solution)<br>litre_of_solution — litre of solution (e.g. 1 litre of 5% solution)<br>cubic_meter — cubic meter (m^3)<br>other — other<br>dont_know — don't know |
| q604_M59c.1 | q604_M59c.1 If other unit of measurement or route of administration, specify here:<br>*Leave blank if not applicable* | |

Leave blank if not applicable

| Field | Question | Answer |
|---|---|---|
| | *Question relevant when: selected( ${q104_M59_full_list} , '5')* | |
| q604_M59d *(required)* | q604_M59d. In total, how many units are used to treat a patient during all of her care?<br>*Remember to add all units! E.g. 5 days x 200 mg twice per day = 2,000mg*<br>*Question relevant when: selected( ${q104_M59_full_list} , '5')*<br>*Response constrained to: .>0* | |
| q604_M60 | ***q604_M60. Antispasmodic, anxiolytic, sedative, etc - Other 1: "[q104_M60_full_list_other]"***<br>*Question relevant when: selected( ${q104_M60_full_list} , '5')* | |
| q604_M60a *(required)* | q604_M60a. What percent of patients require this item?<br>*Question relevant when: selected( ${q104_M60_full_list} , '5')*<br>*Response constrained to: .>0 and .<=100 or .=999* | |
| q604_M60b *(required)* | q604_M60b. How is the item administered, or given, to the patient?<br>*Question relevant when: selected( ${q104_M60_full_list} , '5')* | 1 Oral - solid (e.g. tablet)<br>2 Oral - liquid<br>3 Sublingual<br>4 Intravenous (IV)<br>5 Intramusculuar injection (IM)<br>6 Subcutaneous<br>7 Rectal<br>8 Inhaled (e.g. gas)<br>9 Inserted (e.g. vaginal pessaries)<br>10 Topical - cream<br>11 Topical - drops (e.g. eye drops)<br>12 Other<br>99 Don't know |
| q604_M60c *(required)* | q604_M60c. What is the item's smallest unit of measurement (mg, mcg, ml, g, etc)?<br>*Question relevant when: selected( ${q104_M60_full_list} , '5')* | microgram — microgram (mcg)<br>miligram — miligram (mg)<br>miligram_dissolved_in_liquid — miligram (mg) dissolved in liquid (e.g. 2 mg per 3 militers)<br>gram — gram (g)<br>militer — militer (ml)<br>centiliter — centiliter (cl)<br>litre — litre (l)<br>ml_of _solution — ml of solution (e.g. ml of 10% solution)<br>litre_of_solution — litre of solution (e.g. 1 litre of 5% solution)<br>cubic_meter — cubic meter (m^3)<br>other — other<br>dont_know — don't know |
| q604_M60c.1 | q604_M60c.1 If other unit of measurement or route of administration, specify here:<br>*Leave blank if not applicable* | |

Leave blank if not applicable

| Field | Question | Answer |
|---|---|---|
| | *Question relevant when: selected( ${q104_M60_full_list} , '5')* | |
| q604_M60d *(required)* | q604_M60d. In total, how many units are used to treat a patient during all of her care?<br>*Remember to add all units! E.g. 5 days x 200 mg twice per day = 2,000mg*<br>*Question relevant when: selected( ${q104_M60_full_list} , '5')*<br>*Response constrained to: .>0* | |
| q604_M61 | ***q604_M61. Antispasmodic, anxiolytic, sedative, etc - Other 2: "[q104_M61_full_list_other]"***<br>*Question relevant when: selected( ${q104_M61_full_list} , '5')* | |
| q604_M61a *(required)* | q604_M61a. What percent of patients require this item?<br>*Question relevant when: selected( ${q104_M61_full_list} , '5')*<br>*Response constrained to: .>0 and .<=100 or .=999* | |
| q604_M61b *(required)* | q604_M61b. How is the item administered, or given, to the patient?<br>*Question relevant when: selected( ${q104_M61_full_list} , '5')* | 1 Oral - solid (e.g. tablet)<br>2 Oral - liquid<br>3 Sublingual<br>4 Intravenous (IV)<br>5 Intramusculuar injection (IM)<br>6 Subcutaneous<br>7 Rectal<br>8 Inhaled (e.g. gas)<br>9 Inserted (e.g. vaginal pessaries)<br>10 Topical - cream<br>11 Topical - drops (e.g. eye drops)<br>12 Other<br>99 Don't know |
| q604_M61c *(required)* | q604_M61c. What is the item's smallest unit of measurement (mg, mcg, ml, g, etc)?<br>*Question relevant when: selected( ${q104_M61_full_list} , '5')* | microgram — microgram (mcg)<br>miligram — miligram (mg)<br>miligram_dissolved_in_liquid — miligram (mg) dissolved in liquid (e.g. 2 mg per 3 militers)<br>gram — gram (g)<br>militer — militer (ml)<br>centiliter — centiliter (cl)<br>litre — litre (l)<br>ml_of _solution — ml of solution (e.g. ml of 10% solution)<br>litre_of_solution — litre of solution (e.g. 1 litre of 5% solution)<br>cubic_meter — cubic meter (m^3)<br>other — other<br>dont_know — don't know |
| q604_M61c.1 | q604_M61c.1 If other unit of measurement or route of administration, specify here:<br>*Leave blank if not applicable* | |

*Leave blank if not applicable*
*Question relevant when: selected( ${q104_M61_full_list} , '5')*

| Field | Question | Answer |
|---|---|---|
| q604_M61d *(required)* | q604_M61d. In total, how many units are used to treat a patient during all of her care?<br>*Remember to add all units! E.g. 5 days x 200 mg twice per day = 2,000mg*<br>*Question relevant when: selected( ${q104_M61_full_list} , '5')*<br>*Response constrained to: .>0* | |
| q604_M62 | ***q604_M62. Antispasmodic, anxiolytic, sedative, etc - Other 3: "[q104_M62_full_list_other]"***<br>*Question relevant when: selected( ${q104_M62_full_list} , '5')* | |
| q604_M62a *(required)* | q604_M62a. What percent of patients require this item?<br>*Question relevant when: selected( ${q104_M62_full_list} , '5')*<br>*Response constrained to: .>0 and .<=100 or .=999* | |
| q604_M62b *(required)* | q604_M62b. How is the item administered, or given, to the patient?<br>*Question relevant when: selected( ${q104_M62_full_list} , '5')* | 1 Oral - solid (e.g. tablet)<br>2 Oral - liquid<br>3 Sublingual<br>4 Intravenous (IV)<br>5 Intramusculuar injection (IM)<br>6 Subcutaneous<br>7 Rectal<br>8 Inhaled (e.g. gas)<br>9 Inserted (e.g. vaginal pessaries)<br>10 Topical - cream<br>11 Topical - drops (e.g. eye drops)<br>12 Other<br>99 Don't know |
| q604_M62c *(required)* | q604_M62c. What is the item's smallest unit of measurement (mg, mcg, ml, g, etc)?<br>*Question relevant when: selected( ${q104_M62_full_list} , '5')* | microgram — microgram (mcg)<br>miligram — miligram (mg)<br>miligram_dissolved_in_liquid — miligram (mg) dissolved in liquid (e.g. 2 mg per 3 militers)<br>gram — gram (g)<br>militer — militer (ml)<br>centiliter — centiliter (cl)<br>litre — litre (l)<br>ml_of _solution — ml of solution (e.g. ml of 10% solution)<br>litre_of_solution — litre of solution (e.g. 1 litre of 5% solution)<br>cubic_meter — cubic meter (m^3)<br>other — other<br>dont_know — don't know |
| q604_M62c.1 | q604_M62c.1 If other unit of measurement or route of administration, specify here:<br>*Leave blank if not applicable* | |

*Leave blank if not applicable*

*Question relevant when: selected( ${q104_M62_full_list} , '5')*

| Field | Question | Answer |
|---|---|---|
| q604_M62d *(required)* | q604_M62d. In total, how many units are used to treat a patient during all of her care?<br>*Remember to add all units! E.g. 5 days x 200 mg twice per day = 2,000mg*<br>*Question relevant when: selected( ${q104_M62_full_list} , '5')*<br>*Response constrained to: .>0* | |
| q604_M63 | ***q604_M63. Antispasmodic, anxiolytic, sedative, etc - Other 4: "[q104_M63_full_list_other]"***<br>*Question relevant when: selected( ${q104_M63_full_list} , '5')* | |
| q604_M63a *(required)* | q604_M63a. What percent of patients require this item?<br>*Question relevant when: selected( ${q104_M63_full_list} , '5')*<br>*Response constrained to: .>0 and .<=100 or .=999* | |
| q604_M63b *(required)* | q604_M63b. How is the item administered, or given, to the patient?<br>*Question relevant when: selected( ${q104_M63_full_list} , '5')* | 1 Oral - solid (e.g. tablet)<br>2 Oral - liquid<br>3 Sublingual<br>4 Intravenous (IV)<br>5 Intramusculuar injection (IM)<br>6 Subcutaneous<br>7 Rectal<br>8 Inhaled (e.g. gas)<br>9 Inserted (e.g. vaginal pessaries)<br>10 Topical - cream<br>11 Topical - drops (e.g. eye drops)<br>12 Other<br>99 Don't know |
| q604_M63c *(required)* | q604_M63c. What is the item's smallest unit of measurement (mg, mcg, ml, g, etc)?<br>*Question relevant when: selected( ${q104_M63_full_list} , '5')* | microgram — microgram (mcg)<br>miligram — miligram (mg)<br>miligram_dissolved_in_liquid — miligram (mg) dissolved in liquid (e.g. 2 mg per 3 militers)<br>gram — gram (g)<br>militer — militer (ml)<br>centiliter — centiliter (cl)<br>litre — litre (l)<br>ml_of _solution — ml of solution (e.g. ml of 10% solution)<br>litre_of_solution — litre of solution (e.g. 1 litre of 5% solution)<br>cubic_meter — cubic meter (m^3)<br>other — other<br>dont_know — don't know |
| q604_M63c.1 | q604_M63c.1 If other unit of measurement or route of administration, specify here:<br>*Leave blank if not applicable* | |

Leave blank if not applicable

| Field | Question | Answer |
|---|---|---|
| | Question relevant when: selected( ${q104_M63_full_list} , '5') | |
| q604_M63d *(required)* | q604_M63d. In total, how many units are used to treat a patient during all of her care?<br>*Remember to add all units! E.g. 5 days x 200 mg twice per day = 2,000mg*<br>*Question relevant when: selected( ${q104_M63_full_list} , '5')*<br>*Response constrained to: .>0* | |
| q604_M64 | ***q604_M64. Antispasmodic, anxiolytic, sedative, etc - Other 5: "[q104_M64_full_list_other]"***<br>*Question relevant when: selected( ${q104_M64_full_list} , '5')* | |
| q604_M64a *(required)* | q604_M64a. What percent of patients require this item?<br>*Question relevant when: selected( ${q104_M64_full_list} , '5')*<br>*Response constrained to: .>0 and .<=100 or .=999* | |

| Field | Question | Answer | | |
|---|---|---|---|---|
| q604_M64b *(required)* | q604_M64b. How is the item administered, or given, to the patient?<br>*Question relevant when: selected( ${q104_M64_full_list} , '5')* | | 1 | Oral - solid (e.g. tablet) |
| | | | 2 | Oral - liquid |
| | | | 3 | Sublingual |
| | | | 4 | Intravenous (IV) |
| | | | 5 | Intramusculuar injection (IM) |
| | | | 6 | Subcutaneous |
| | | | 7 | Rectal |
| | | | 8 | Inhaled (e.g. gas) |
| | | | 9 | Inserted (e.g. vaginal pessaries) |
| | | | 10 | Topical - cream |
| | | | 11 | Topical - drops (e.g. eye drops) |
| | | | 12 | Other |
| | | | 99 | Don't know |
| q604_M64c *(required)* | q604_M64c. What is the item's smallest unit of measurement (mg, mcg, ml, g, etc)?<br>*Question relevant when: selected( ${q104_M64_full_list} , '5')* | | microgram | microgram (mcg) |
| | | | miligram | miligram (mg) |
| | | | miligram_dissolved_in_liquid | miligram (mg) dissolved in liquid (e.g. 2 mg per 3 militers) |
| | | | gram | gram (g) |
| | | | militer | militer (ml) |
| | | | centiliter | centiliter (cl) |
| | | | litre | litre (l) |
| | | | ml_of _solution | ml of solution (e.g. ml of 10% solution) |
| | | | litre_of_solution | litre of solution (e.g. 1 litre of 5% solution) |
| | | | cubic_meter | cubic meter (m^3) |
| | | | other | other |
| | | | dont_know | don't know |
| q604_M64c.1 | q604_M64c.1 If other unit of measurement or route of administration, specify here:<br>*Leave blank if not applicable* | | | |

| Field | Question | Answer |
|---|---|---|
| | *Leave blank if not applicable*<br>*Question relevant when: selected( ${q104_M64_full_list} , '5')* | |
| q604_M64d *(required)* | q604_M64d. In total, how many units are used to treat a patient during all of her care?<br>*Remember to add all units! E.g. 5 days x 200 mg twice per day = 2,000mg*<br>*Question relevant when: selected( ${q104_M64_full_list} , '5')*<br>*Response constrained to: .>0* | |
| <p><strong><em>D. Medications - Perforations (6)</em></strong></p><br>*Group relevant when: selected( ${section_six_skip_med} , '1')* | | |
| q604_med_notE5 | 604 CONTINUED For each of the following medications and other medical products that are used for perforations, can you tell me …? | |
| note_604_adrenergic | ***Adrenergic*** | |
| q604_M65 | ***q604_M65. Ephedrine HCl***<br>*Question relevant when: selected( ${q104_M65_full_list} , '5')* | |
| q604_M65a *(required)* | q604_M65a. What percent of patients require this item?<br>*Question relevant when: selected( ${q104_M65_full_list} , '5')*<br>*Response constrained to: .>0 and .<=100 or .=999* | |

| | | |
|---|---|---|
| q604_M65b *(required)* | q604_M65b. How is the item administered, or given, to the patient?<br>*Question relevant when: selected( ${q104_M65_full_list} , '5')* | 1 Oral - solid (e.g. tablet)<br>2 Oral - liquid<br>3 Sublingual<br>4 Intravenous (IV)<br>5 Intramusculuar injection (IM)<br>6 Subcutaneous<br>7 Rectal<br>8 Inhaled (e.g. gas)<br>9 Inserted (e.g. vaginal pessaries)<br>10 Topical - cream<br>11 Topical - drops (e.g. eye drops)<br>12 Other<br>99 Don't know |
| q604_M65c *(required)* | q604_M65c. What is the item's smallest unit of measurement (mg, mcg, ml, g, etc)?<br>*Question relevant when: selected( ${q104_M65_full_list} , '5')* | microgram — microgram (mcg)<br>miligram — miligram (mg)<br>miligram_dissolved_in_liquid — miligram (mg) dissolved in liquid (e.g. 2 mg per 3 militers)<br>gram — gram (g)<br>militer — militer (ml)<br>centiliter — centiliter (cl)<br>litre — litre (l)<br>ml_of _solution — ml of solution (e.g. ml of 10% solution)<br>litre_of_solution — litre of solution (e.g. 1 litre of 5% solution)<br>cubic_meter — cubic meter (m^3)<br>other — other<br>dont_know — don't know |
| q604_M65c.1 | q604_M65c.1 If other unit of measurement or route of administration, specify here:<br>*Leave blank if not applicable* | |

Leave blank if not applicable

| Field | Question | Answer |
|---|---|---|
| | *Question relevant when: selected( ${q104_M65_full_list} , '5')* | |
| q604_M65d *(required)* | q604_M65d. In total, how many units are used to treat a patient during all of her care?<br>*Remember to add all units! E.g. 5 days x 200 mg twice per day = 2,000mg*<br>*Question relevant when: selected( ${q104_M65_full_list} , '5')*<br>*Response constrained to: .>0* | |
| q604_M66 | ***q604_M66. Epinephrine***<br>*Question relevant when: selected( ${q104_M66_full_list} , '5')* | |
| q604_M66a *(required)* | q604_M66a. What percent of patients require this item?<br>*Question relevant when: selected( ${q104_M66_full_list} , '5')*<br>*Response constrained to: .>0 and .<=100 or .=999* | |

| Field | Question | Answer |
|---|---|---|
| q604_M66b *(required)* | q604_M66b. How is the item administered, or given, to the patient?<br>*Question relevant when: selected( ${q104_M66_full_list} , '5')* | 1 Oral - solid (e.g. tablet)<br>2 Oral - liquid<br>3 Sublingual<br>4 Intravenous (IV)<br>5 Intramusculuar injection (IM)<br>6 Subcutaneous<br>7 Rectal<br>8 Inhaled (e.g. gas)<br>9 Inserted (e.g. vaginal pessaries)<br>10 Topical - cream<br>11 Topical - drops (e.g. eye drops)<br>12 Other<br>99 Don't know |
| q604_M66c *(required)* | q604_M66c. What is the item's smallest unit of measurement (mg, mcg, ml, g, etc)?<br>*Question relevant when: selected( ${q104_M66_full_list} , '5')* | microgram — microgram (mcg)<br>miligram — miligram (mg)<br>miligram_dissolved_in_liquid — miligram (mg) dissolved in liquid (e.g. 2 mg per 3 militers)<br>gram — gram (g)<br>militer — militer (ml)<br>centiliter — centiliter (cl)<br>litre — litre (l)<br>ml_of _solution — ml of solution (e.g. ml of 10% solution)<br>litre_of_solution — litre of solution (e.g. 1 litre of 5% solution)<br>cubic_meter — cubic meter (m^3)<br>other — other<br>dont_know — don't know |
| q604_M66c.1 | q604_M66c.1 If other unit of measurement or route of administration, specify here:<br>*Leave blank if not applicable* | |

| Field | Question | Answer |
|---|---|---|
| | *Leave blank if not applicable*<br>*Question relevant when: selected( ${q104_M66_full_list} , '5')* | |
| q604_M66d *(required)* | q604_M66d. In total, how many units are used to treat a patient during all of her care?<br>*Remember to add all units! E.g. 5 days x 200 mg twice per day = 2,000mg*<br>*Question relevant when: selected( ${q104_M66_full_list} , '5')*<br>*Response constrained to: .>0* | |
| q604_M67 | ***q604_M67. Adrenergic - other 1: "[q104_M67_full_list_other]"***<br>*Question relevant when: selected( ${q104_M67_full_list} , '5')* | |
| q604_M67a *(required)* | q604_M67a. What percent of patients require this item?<br>*Question relevant when: selected( ${q104_M67_full_list} , '5')*<br>*Response constrained to: .>0 and .<=100 or .=999* | |
| q604_M67b *(required)* | q604_M67b. How is the item administered, or given, to the patient?<br>*Question relevant when: selected( ${q104_M67_full_list} , '5')* | 1 Oral - solid (e.g. tablet)<br>2 Oral - liquid<br>3 Sublingual<br>4 Intravenous (IV)<br>5 Intramusculuar injection (IM)<br>6 Subcutaneous<br>7 Rectal<br>8 Inhaled (e.g. gas)<br>9 Inserted (e.g. vaginal pessaries)<br>10 Topical - cream<br>11 Topical - drops (e.g. eye drops)<br>12 Other<br>99 Don't know |
| q604_M67c *(required)* | q604_M67c. What is the item's smallest unit of measurement (mg, mcg, ml, g, etc)?<br>*Question relevant when: selected( ${q104_M67_full_list} , '5')* | microgram — microgram (mcg)<br>miligram — miligram (mg)<br>miligram_dissolved_in_liquid — miligram (mg) dissolved in liquid (e.g. 2 mg per 3 militers)<br>gram — gram (g)<br>militer — militer (ml)<br>centiliter — centiliter (cl)<br>litre — litre (l)<br>ml_of _solution — ml of solution (e.g. ml of 10% solution)<br>litre_of_solution — litre of solution (e.g. 1 litre of 5% solution)<br>cubic_meter — cubic meter (m^3)<br>other — other<br>dont_know — don't know |
| q604_M67c.1 | q604_M67c.1 If other unit of measurement or route of administration, specify here:<br>*Leave blank if not applicable* | |

Leave blank if not applicable
Question relevant when: selected( ${q104_M67_full_list} , '5')

| Field | Question | Answer |
|---|---|---|
| q604_M67d *(required)* | q604_M67d. In total, how many units are used to treat a patient during all of her care?<br>*Remember to add all units! E.g. 5 days x 200 mg twice per day = 2,000mg*<br>*Question relevant when: selected( ${q104_M67_full_list} , '5')*<br>*Response constrained to: .>0* | |
| q604_M68 | ***q604_M68. Adrenergic - other 2: "[q104_M68_full_list_other]"***<br>*Question relevant when: selected( ${q104_M68_full_list} , '5')* | |
| q604_M68a *(required)* | q604_M68a. What percent of patients require this item?<br>*Question relevant when: selected( ${q104_M68_full_list} , '5')*<br>*Response constrained to: .>0 and .<=100 or .=999* | |

| Field | Question | Answer |
|---|---|---|
| q604_M68b *(required)* | q604_M68b. How is the item administered, or given, to the patient?<br>*Question relevant when: selected( ${q104_M68_full_list} , '5')* | 1 Oral - solid (e.g. tablet)<br>2 Oral - liquid<br>3 Sublingual<br>4 Intravenous (IV)<br>5 Intramusculuar injection (IM)<br>6 Subcutaneous<br>7 Rectal<br>8 Inhaled (e.g. gas)<br>9 Inserted (e.g. vaginal pessaries)<br>10 Topical - cream<br>11 Topical - drops (e.g. eye drops)<br>12 Other<br>99 Don't know |
| q604_M68c *(required)* | q604_M68c. What is the item's smallest unit of measurement (mg, mcg, ml, g, etc)?<br>*Question relevant when: selected( ${q104_M68_full_list} , '5')* | microgram — microgram (mcg)<br>miligram — miligram (mg)<br>miligram_dissolved_in_liquid — miligram (mg) dissolved in liquid (e.g. 2 mg per 3 militers)<br>gram — gram (g)<br>militer — militer (ml)<br>centiliter — centiliter (cl)<br>litre — litre (l)<br>ml_of _solution — ml of solution (e.g. ml of 10% solution)<br>litre_of_solution — litre of solution (e.g. 1 litre of 5% solution)<br>cubic_meter — cubic meter (m^3)<br>other — other<br>dont_know — don't know |
| q604_M68c.1 | q604_M68c.1 If other unit of measurement or route of administration, specify here:<br>*Leave blank if not applicable* | |

| Field | Question | Answer |
|---|---|---|
| | *Leave blank if not applicable*<br>*Question relevant when: selected( ${q104_M68_full_list} , '5')* | |
| q604_M68d *(required)* | q604_M68d. In total, how many units are used to treat a patient during all of her care?<br>*Remember to add all units! E.g. 5 days x 200 mg twice per day = 2,000mg*<br>*Question relevant when: selected( ${q104_M68_full_list} , '5')*<br>*Response constrained to: .>0* | |
| q604_M69 | ***q604_M69. Adrenergic - other 3: "[q104_M69_full_list_other]"***<br>*Question relevant when: selected( ${q104_M69_full_list} , '5')* | |
| q604_M69a *(required)* | q604_M69a. What percent of patients require this item?<br>*Question relevant when: selected( ${q104_M69_full_list} , '5')*<br>*Response constrained to: .>0 and .<=100 or .=999* | |

| Field | Question | Answer |
|---|---|---|
| q604_M69b *(required)* | q604_M69b. How is the item administered, or given, to the patient?<br>*Question relevant when: selected( ${q104_M69_full_list} , '5')* | 1 Oral - solid (e.g. tablet)<br>2 Oral - liquid<br>3 Sublingual<br>4 Intravenous (IV)<br>5 Intramusculuar injection (IM)<br>6 Subcutaneous<br>7 Rectal<br>8 Inhaled (e.g. gas)<br>9 Inserted (e.g. vaginal pessaries)<br>10 Topical - cream<br>11 Topical - drops (e.g. eye drops)<br>12 Other<br>99 Don't know |
| q604_M69c *(required)* | q604_M69c. What is the item's smallest unit of measurement (mg, mcg, ml, g, etc)?<br>*Question relevant when: selected( ${q104_M69_full_list} , '5')* | microgram — microgram (mcg)<br>miligram — miligram (mg)<br>miligram_dissolved_in_liquid — miligram (mg) dissolved in liquid (e.g. 2 mg per 3 militers)<br>gram — gram (g)<br>militer — militer (ml)<br>centiliter — centiliter (cl)<br>litre — litre (l)<br>ml_of _solution — ml of solution (e.g. ml of 10% solution)<br>litre_of_solution — litre of solution (e.g. 1 litre of 5% solution)<br>cubic_meter — cubic meter (m^3)<br>other — other<br>dont_know — don't know |
| q604_M69c.1 | q604_M69c.1 If other unit of measurement or route of administration, specify here:<br>*Leave blank if not applicable* | |

Leave blank if not applicable

| Field | Question | Answer |
|---|---|---|
| | *Question relevant when: selected( ${q104_M69_full_list} , '5')* | |
| q604_M69d *(required)* | q604_M69d. In total, how many units are used to treat a patient during all of her care?<br>*Remember to add all units! E.g. 5 days x 200 mg twice per day = 2,000mg*<br>*Question relevant when: selected( ${q104_M69_full_list} , '5')*<br>*Response constrained to: .>0* | |
| q604_M69.1 | ***q604_M69.1. Adrenergic - other 4: "[q104_M69.1_full_list_other]"***<br>*Question relevant when: selected( ${q104_M69.1_full_list} , '2')* | |
| q604_M69.1a *(required)* | q604_M69.1a. What percent of patients require this item?<br>*Question relevant when: selected( ${q104_M69.1_full_list} , '2')*<br>*Response constrained to: .>0 and .<=100 or .=999* | |

| | | |
|---|---|---|
| q604_M69.1b *(required)* | q604_M69.1b. How is the item administered, or given, to the patient?<br>*Question relevant when: selected( ${q104_M69.1_full_list} , '2')* | 1 Oral - solid (e.g. tablet)<br>2 Oral - liquid<br>3 Sublingual<br>4 Intravenous (IV)<br>5 Intramusculuar injection (IM)<br>6 Subcutaneous<br>7 Rectal<br>8 Inhaled (e.g. gas)<br>9 Inserted (e.g. vaginal pessaries)<br>10 Topical - cream<br>11 Topical - drops (e.g. eye drops)<br>12 Other<br>99 Don't know |
| q604_M69.1c *(required)* | q604_M69.1c. What is the item's smallest unit of measurement (mg, mcg, ml, g, etc)?<br>*Question relevant when: selected( ${q104_M69.1_full_list} , '2')* | microgram — microgram (mcg)<br>miligram — miligram (mg)<br>miligram_dissolved_in_liquid — miligram (mg) dissolved in liquid (e.g. 2 mg per 3 militers)<br>gram — gram (g)<br>militer — militer (ml)<br>centiliter — centiliter (cl)<br>litre — litre (l)<br>ml_of _solution — ml of solution (e.g. ml of 10% solution)<br>litre_of_solution — litre of solution (e.g. 1 litre of 5% solution)<br>cubic_meter — cubic meter (m^3)<br>other — other<br>dont_know — don't know |
| q604_M69.1c.1 | q604_M69.1c.1 If other unit of measurement or route of administration, specify here:<br>*Leave blank if not applicable* | |

| Field | Question | Answer |
|---|---|---|
| | *Leave blank if not applicable*<br>*Question relevant when: selected( ${q104_M69.1_full_list} , '2')* | |
| q604_M69.1d *(required)* | q604_M69.1d. In total, how many units are used to treat a patient during all of her care?<br>*Remember to add all units! E.g. 5 days x 200 mg twice per day = 2,000mg*<br>*Question relevant when: selected( ${q104_M69.1_full_list} , '2')*<br>*Response constrained to: .>0* | |
| q604_M69.2 | ***q604_M69.2. Adrenergic - other 5: "[q104_M69.2_full_list_other]"***<br>*Question relevant when: selected( ${q104_M69.2_full_list} , '2')* | |
| q604_M69.2a *(required)* | q604_M69.2a. What percent of patients require this item?<br>*Question relevant when: selected( ${q104_M69.2_full_list} , '2')*<br>*Response constrained to: .>0 and .<=100 or .=999* | |
| q604_M69.2b *(required)* | q604_M69.2b. How is the item administered, or given, to the patient?<br>*Question relevant when: selected( ${q104_M69.2_full_list} , '2')* | 1 Oral - solid (e.g. tablet)<br>2 Oral - liquid<br>3 Sublingual<br>4 Intravenous (IV)<br>5 Intramusculuar injection (IM)<br>6 Subcutaneous<br>7 Rectal<br>8 Inhaled (e.g. gas)<br>9 Inserted (e.g. vaginal pessaries)<br>10 Topical - cream<br>11 Topical - drops (e.g. eye drops)<br>12 Other<br>99 Don't know |
| q604_M69.2c *(required)* | q604_M69.2c. What is the item's smallest unit of measurement (mg, mcg, ml, g, etc)?<br>*Question relevant when: selected( ${q104_M69.2_full_list} , '2')* | microgram — microgram (mcg)<br>miligram — miligram (mg)<br>miligram_dissolved_in_liquid — miligram (mg) dissolved in liquid (e.g. 2 mg per 3 militers)<br>gram — gram (g)<br>militer — militer (ml)<br>centiliter — centiliter (cl)<br>litre — litre (l)<br>ml_of _solution — ml of solution (e.g. ml of 10% solution)<br>litre_of_solution — litre of solution (e.g. 1 litre of 5% solution)<br>cubic_meter — cubic meter (m^3)<br>other — other<br>dont_know — don't know |
| q604_M69.2c.1 | q604_M69.2c.1 If other unit of measurement or route of administration, specify here:<br>*Leave blank if not applicable* | |

| Field | Question | Answer |
|---|---|---|
| | *Leave blank if not applicable*<br>*Question relevant when: selected( ${q104_M69.2_full_list} , '2')* | |
| q604_M69.2d *(required)* | q604_M69.2d. In total, how many units are used to treat a patient during all of her care?<br>*Remember to add all units! E.g. 5 days x 200 mg twice per day = 2,000mg*<br>*Question relevant when: selected( ${q104_M69.2_full_list} , '2')*<br>*Response constrained to: .>0* | |
| <p><strong><em>D. Medications - Perforations (7)</em></strong></p><br>*Group relevant when: selected( ${section_six_skip_med} , '1')* | | |
| q604_med_notE6 | 604 CONTINUED For each of the following medications and other medical products that are used for perforations, can you tell me …? | |
| note_604_blood | ***Blood products*** | |
| q604_M70 | ***q604_M70. Unit of packed red blood cells***<br>*Question relevant when: selected( ${q104_M70_full_list} , '5')* | |
| q604_M70a *(required)* | q604_M70a. What percent of patients require this item?<br>*Question relevant when: selected( ${q104_M70_full_list} , '5')*<br>*Response constrained to: .>0 and .<=100 or .=999* | |
| q604_M70b *(required)* | q604_M70b. How is the item administered, or given, to the patient?<br>*Question relevant when: selected( ${q104_M70_full_list} , '5')* | 1 Oral - solid (e.g. tablet)<br>2 Oral - liquid<br>3 Sublingual<br>4 Intravenous (IV)<br>5 Intramuscular injection (IM)<br>6 Subcutaneous<br>7 Rectal<br>8 Inhaled (e.g. gas)<br>9 Inserted (e.g. vaginal pessaries)<br>10 Topical - cream<br>11 Topical - drops (e.g. eye drops)<br>12 Other<br>99 Don't know |
| q604_M70c *(required)* | q604_M70c. What is the item's smallest unit of measurement (mg, mcg, ml, g, etc)?<br>*Question relevant when: selected( ${q104_M70_full_list} , '5')* | microgram — microgram (mcg)<br>miligram — miligram (mg)<br>miligram_dissolved_in_liquid — miligram (mg) dissolved in liquid (e.g. 2 mg per 3 militers)<br>gram — gram (g)<br>militer — militer (ml)<br>centiliter — centiliter (cl)<br>litre — litre (l)<br>ml_of _solution — ml of solution (e.g. ml of 10% solution)<br>litre_of_solution — litre of solution (e.g. 1 litre of 5% solution)<br>cubic_meter — cubic meter (m^3)<br>other — other<br>dont_know — don't know |
| q604_M70c.1 | q604_M70c.1 If other unit of measurement or route of administration, specify here:<br>*Leave blank if not applicable* | |

*Leave blank if not applicable*

| Field | Question | Answer |
|---|---|---|
| | *Question relevant when: selected( ${q104_M70_full_list} , '5')* | |
| q604_M70d *(required)* | q604_M70d. In total, how many units are used to treat a patient during all of her care?<br>*Remember to add all units! E.g. 5 days x 200 mg twice per day = 2,000mg*<br>*Question relevant when: selected( ${q104_M70_full_list} , '5')*<br>*Response constrained to: .>0* | |
| q604_M71 | ***q604_M71. Unit of platelets***<br>*Question relevant when: selected( ${q104_M71_full_list} , '5')* | |
| q604_M71a *(required)* | q604_M71a. What percent of patients require this item?<br>*Question relevant when: selected( ${q104_M71_full_list} , '5')*<br>*Response constrained to: .>0 and .<=100 or .=999* | |
| q604_M71b *(required)* | q604_M71b. How is the item administered, or given, to the patient?<br>*Question relevant when: selected( ${q104_M71_full_list} , '5')* | 1 Oral - solid (e.g. tablet)<br>2 Oral - liquid<br>3 Sublingual<br>4 Intravenous (IV)<br>5 Intramusculuar injection (IM)<br>6 Subcutaneous<br>7 Rectal<br>8 Inhaled (e.g. gas)<br>9 Inserted (e.g. vaginal pessaries)<br>10 Topical - cream<br>11 Topical - drops (e.g. eye drops)<br>12 Other<br>99 Don't know |
| q604_M71c *(required)* | q604_M71c. What is the item's smallest unit of measurement (mg, mcg, ml, g, etc)?<br>*Question relevant when: selected( ${q104_M71_full_list} , '5')* | microgram — microgram (mcg)<br>miligram — miligram (mg)<br>miligram_dissolved_in_liquid — miligram (mg) dissolved in liquid (e.g. 2 mg per 3 militers)<br>gram — gram (g)<br>militer — militer (ml)<br>centiliter — centiliter (cl)<br>litre — litre (l)<br>ml_of _solution — ml of solution (e.g. ml of 10% solution)<br>litre_of_solution — litre of solution (e.g. 1 litre of 5% solution)<br>cubic_meter — cubic meter (m^3)<br>other — other<br>dont_know — don't know |
| q604_M71c.1 | q604_M71c.1 If other unit of measurement or route of administration, specify here:<br>*Leave blank if not applicable* | |

| Field | Question | Answer |
|---|---|---|
| | *Leave blank if not applicable*<br>*Question relevant when: selected( ${q104_M71_full_list} , '5')* | |
| q604_M71d *(required)* | q604_M71d. In total, how many units are used to treat a patient during all of her care?<br>*Remember to add all units! E.g. 5 days x 200 mg twice per day = 2,000mg*<br>*Question relevant when: selected( ${q104_M71_full_list} , '5')*<br>*Response constrained to: .>0* | |
| q604_M72 | ***q604_M72. Unit of whole blood***<br>*Question relevant when: selected( ${q104_M72_full_list} , '5')* | |
| q604_M72a *(required)* | q604_M72a. What percent of patients require this item?<br>*Question relevant when: selected( ${q104_M72_full_list} , '5')*<br>*Response constrained to: .>0 and .<=100 or .=999* | |
| q604_M72b *(required)* | q604_M72b. How is the item administered, or given, to the patient?<br>*Question relevant when: selected( ${q104_M72_full_list} , '5')* | 1 Oral - solid (e.g. tablet)<br>2 Oral - liquid<br>3 Sublingual<br>4 Intravenous (IV)<br>5 Intramusculuar injection (IM)<br>6 Subcutaneous<br>7 Rectal<br>8 Inhaled (e.g. gas)<br>9 Inserted (e.g. vaginal pessaries)<br>10 Topical - cream<br>11 Topical - drops (e.g. eye drops)<br>12 Other<br>99 Don't know |
| q604_M72c *(required)* | q604_M72c. What is the item's smallest unit of measurement (mg, mcg, ml, g, etc)?<br>*Question relevant when: selected( ${q104_M72_full_list} , '5')* | microgram — microgram (mcg)<br>miligram — miligram (mg)<br>miligram_dissolved_in_liquid — miligram (mg) dissolved in liquid (e.g. 2 mg per 3 militers)<br>gram — gram (g)<br>militer — militer (ml)<br>centiliter — centiliter (cl)<br>litre — litre (l)<br>ml_of _solution — ml of solution (e.g. ml of 10% solution)<br>litre_of_solution — litre of solution (e.g. 1 litre of 5% solution)<br>cubic_meter — cubic meter (m^3)<br>other — other<br>dont_know — don't know |
| q604_M72c.1 | q604_M72c.1 If other unit of measurement or route of administration, specify here:<br>*Leave blank if not applicable* | |

Leave blank if not applicable

| Field | Question | Answer |
|---|---|---|
| | *Question relevant when: selected( ${q104_M72_full_list} , '5')* | |
| q604_M72d *(required)* | q604_M72d. In total, how many units are used to treat a patient during all of her care?<br>*Remember to add all units! E.g. 5 days x 200 mg twice per day = 2,000mg*<br>*Question relevant when: selected( ${q104_M72_full_list} , '5')*<br>*Response constrained to: .>0* | |
| q604_M73 | ***q604_M73. Blood product - other 1: "[q104_M73_full_list_other]"***<br>*Question relevant when: selected( ${q104_M73_full_list} , '5')* | |
| q604_M73a *(required)* | q604_M73a. What percent of patients require this item?<br>*Question relevant when: selected( ${q104_M73_full_list} , '5')*<br>*Response constrained to: .>0 and .<=100 or .=999* | |
| q604_M73b *(required)* | q604_M73b. How is the item administered, or given, to the patient?<br>*Question relevant when: selected( ${q104_M73_full_list} , '5')* | 1 Oral - solid (e.g. tablet)<br>2 Oral - liquid<br>3 Sublingual<br>4 Intravenous (IV)<br>5 Intramusculuar injection (IM)<br>6 Subcutaneous<br>7 Rectal<br>8 Inhaled (e.g. gas)<br>9 Inserted (e.g. vaginal pessaries)<br>10 Topical - cream<br>11 Topical - drops (e.g. eye drops)<br>12 Other<br>99 Don't know |
| q604_M73c *(required)* | q604_M73c. What is the item's smallest unit of measurement (mg, mcg, ml, g, etc)?<br>*Question relevant when: selected( ${q104_M73_full_list} , '5')* | microgram — microgram (mcg)<br>miligram — miligram (mg)<br>miligram_dissolved_in_liquid — miligram (mg) dissolved in liquid (e.g. 2 mg per 3 militers)<br>gram — gram (g)<br>militer — militer (ml)<br>centiliter — centiliter (cl)<br>litre — litre (l)<br>ml_of _solution — ml of solution (e.g. ml of 10% solution)<br>litre_of_solution — litre of solution (e.g. 1 litre of 5% solution)<br>cubic_meter — cubic meter (m^3)<br>other — other<br>dont_know — don't know |
| q604_M73c.1 | q604_M73c.1 If other unit of measurement or route of administration, specify here: | |

| Field | Question | Answer |
|---|---|---|
| | *Leave blank if not applicable*<br>*Question relevant when: selected( ${q104_M73_full_list} , '5')* | |
| q604_M73d *(required)* | q604_M73d. In total, how many units are used to treat a patient during all of her care?<br>*Remember to add all units! E.g. 5 days x 200 mg twice per day = 2,000mg*<br>*Question relevant when: selected( ${q104_M73_full_list} , '5')*<br>*Response constrained to: .>0* | |
| q604_M74 | ***q604_M74. Blood product - other 2: "[q104_M74_full_list_other]"***<br>*Question relevant when: selected( ${q104_M74_full_list} , '5')* | |
| q604_M74a *(required)* | q604_M74a. What percent of patients require this item?<br>*Question relevant when: selected( ${q104_M74_full_list} , '5')*<br>*Response constrained to: .>0 and .<=100 or .=999* | |
| q604_M74b *(required)* | q604_M74b. How is the item administered, or given, to the patient?<br>*Question relevant when: selected( ${q104_M74_full_list} , '5')* | 1 Oral - solid (e.g. tablet)<br>2 Oral - liquid<br>3 Sublingual<br>4 Intravenous (IV)<br>5 Intramuscluar injection (IM)<br>6 Subcutaneous<br>7 Rectal<br>8 Inhaled (e.g. gas)<br>9 Inserted (e.g. vaginal pessaries)<br>10 Topical - cream<br>11 Topical - drops (e.g. eye drops)<br>12 Other<br>99 Don't know |
| q604_M74c *(required)* | q604_M74c. What is the item's smallest unit of measurement (mg, mcg, ml, g, etc)?<br>*Question relevant when: selected( ${q104_M74_full_list} , '5')* | microgram — microgram (mcg)<br>miligram — miligram (mg)<br>miligram_dissolved_in_liquid — miligram (mg) dissolved in liquid (e.g. 2 mg per 3 militers)<br>gram — gram (g)<br>militer — militer (ml)<br>centiliter — centiliter (cl)<br>litre — litre (l)<br>ml_of _solution — ml of solution (e.g. ml of 10% solution)<br>litre_of_solution — litre of solution (e.g. 1 litre of 5% solution)<br>cubic_meter — cubic meter (m^3)<br>other — other<br>dont_know — don't know |
| q604_M74c.1 | q604_M74c.1 If other unit of measurement or route of administration, specify here: | |

| Field | Question | Answer |
|---|---|---|
| | *Leave blank if not applicable*<br>*Question relevant when: selected( ${q104_M74_full_list} , '5')* | |
| q604_M74d *(required)* | q604_M74d. In total, how many units are used to treat a patient during all of her care?<br>*Remember to add all units! E.g. 5 days x 200 mg twice per day = 2,000mg*<br>*Question relevant when: selected( ${q104_M74_full_list} , '5')*<br>*Response constrained to: .>0* | |
| q604_M75 | ***q604_M75. Blood product - other 3: "[q104_M75_full_list_other]"***<br>*Question relevant when: selected( ${q104_M75_full_list} , '5')* | |
| q604_M75a *(required)* | q604_M75a. What percent of patients require this item?<br>*Question relevant when: selected( ${q104_M75_full_list} , '5')*<br>*Response constrained to: .>0 and .<=100 or .=999* | |
| q604_M75b *(required)* | q604_M75b. How is the item administered, or given, to the patient?<br>*Question relevant when: selected( ${q104_M75_full_list} , '5')* | 1 Oral - solid (e.g. tablet)<br>2 Oral - liquid<br>3 Sublingual<br>4 Intravenous (IV)<br>5 Intramusculuar injection (IM)<br>6 Subcutaneous<br>7 Rectal<br>8 Inhaled (e.g. gas)<br>9 Inserted (e.g. vaginal pessaries)<br>10 Topical - cream<br>11 Topical - drops (e.g. eye drops)<br>12 Other<br>99 Don't know |
| q604_M75c *(required)* | q604_M75c. What is the item's smallest unit of measurement (mg, mcg, ml, g, etc)?<br>*Question relevant when: selected( ${q104_M75_full_list} , '5')* | microgram — microgram (mcg)<br>miligram — miligram (mg)<br>miligram_dissolved_in_liquid — miligram (mg) dissolved in liquid (e.g. 2 mg per 3 militers)<br>gram — gram (g)<br>militer — militer (ml)<br>centiliter — centiliter (cl)<br>litre — litre (l)<br>ml_of _solution — ml of solution (e.g. ml of 10% solution)<br>litre_of_solution — litre of solution (e.g. 1 litre of 5% solution)<br>cubic_meter — cubic meter (m^3)<br>other — other<br>dont_know — don't know |
| q604_M75c.1 | q604_M75c.1 If other unit of measurement or route of administration, specify here: | |

| Field | Question | Answer |
|---|---|---|
| | *Leave blank if not applicable*<br>*Question relevant when: selected( ${q104_M75_full_list} , '5')* | |
| q604_M75d *(required)* | q604_M75d. In total, how many units are used to treat a patient during all of her care?<br>*Remember to add all units! E.g. 5 days x 200 mg twice per day = 2,000mg*<br>*Question relevant when: selected( ${q104_M75_full_list} , '5')*<br>*Response constrained to: .>0* | |
| q604_M75.1 | ***q604_M75.1. Blood product - other 4: "[q104_M75.1_full_list_other]"***<br>*Question relevant when: selected( ${q104_M75.1_full_list} , '2')* | |
| q604_M75.1a *(required)* | q604_M75.1a. What percent of patients require this item?<br>*Question relevant when: selected( ${q104_M75.1_full_list} , '2')*<br>*Response constrained to: .>0 and .<=100 or .=999* | |

| Field | Question | Answer | | |
|---|---|---|---|---|
| q604_M75.1b *(required)* | q604_M75.1b. How is the item administered, or given, to the patient?<br>*Question relevant when: selected( ${q104_M75.1_full_list} , '2')* | | 1 | Oral - solid (e.g. tablet) |
| | | | 2 | Oral - liquid |
| | | | 3 | Sublingual |
| | | | 4 | Intravenous (IV) |
| | | | 5 | Intramusculuar injection (IM) |
| | | | 6 | Subcutaneous |
| | | | 7 | Rectal |
| | | | 8 | Inhaled (e.g. gas) |
| | | | 9 | Inserted (e.g. vaginal pessaries) |
| | | | 10 | Topical - cream |
| | | | 11 | Topical - drops (e.g. eye drops) |
| | | | 12 | Other |
| | | | 99 | Don't know |
| q604_M75.1c *(required)* | q604_M75.1c. What is the item's smallest unit of measurement (mg, mcg, ml, g, etc)?<br>*Question relevant when: selected( ${q104_M75.1_full_list} , '2')* | | microgram | microgram (mcg) |
| | | | miligram | miligram (mg) |
| | | | miligram_dissolved_in_liquid | miligram (mg) dissolved in liquid (e.g. 2 mg per 3 militers) |
| | | | gram | gram (g) |
| | | | militer | militer (ml) |
| | | | centiliter | centiliter (cl) |
| | | | litre | litre (l) |
| | | | ml_of _solution | ml of solution (e.g. ml of 10% solution) |
| | | | litre_of_solution | litre of solution (e.g. 1 litre of 5% solution) |
| | | | cubic_meter | cubic meter (m^3) |
| | | | other | other |
| | | | dont_know | don't know |
| q604_M75.1c.1 | q604_M75.1c.1 If other unit of measurement or route of administration, specify here: | | | |

| Field | Question | Answer |
|---|---|---|
| | *Leave blank if not applicable*<br>*Question relevant when: selected( ${q104_M75.1_full_list} , '2')* | |
| q604_M75.1d *(required)* | q604_M75.1d. In total, how many units are used to treat a patient during all of her care?<br>*Remember to add all units! E.g. 5 days x 200 mg twice per day = 2,000mg*<br>*Question relevant when: selected( ${q104_M75.1_full_list} , '2')*<br>*Response constrained to: .>0* | |
| q604_M75.2 | ***q604_M75.2. Blood product - other 5: "[q104_M75.2_full_list_other]"***<br>*Question relevant when: selected( ${q104_M75.2_full_list} , '2')* | |
| q604_M75.2a *(required)* | q604_M75.2a. What percent of patients require this item?<br>*Question relevant when: selected( ${q104_M75.2_full_list} , '2')*<br>*Response constrained to: .>0 and .<=100 or .=999* | |

| Field | Question | Answer | | |
|---|---|---|---|---|
| q604_M75.2b *(required)* | q604_M75.2b. How is the item administered, or given, to the patient?<br>*Question relevant when: selected( ${q104_M75.2_full_list} , '2')* | | 1 | Oral - solid (e.g. tablet) |
| | | | 2 | Oral - liquid |
| | | | 3 | Sublingual |
| | | | 4 | Intravenous (IV) |
| | | | 5 | Intramusculuar injection (IM) |
| | | | 6 | Subcutaneous |
| | | | 7 | Rectal |
| | | | 8 | Inhaled (e.g. gas) |
| | | | 9 | Inserted (e.g. vaginal pessaries) |
| | | | 10 | Topical - cream |
| | | | 11 | Topical - drops (e.g. eye drops) |
| | | | 12 | Other |
| | | | 99 | Don't know |
| q604_M75.2c *(required)* | q604_M75.2c. What is the item's smallest unit of measurement (mg, mcg, ml, g, etc)?<br>*Question relevant when: selected( ${q104_M75.2_full_list} , '2')* | | microgram | microgram (mcg) |
| | | | miligram | miligram (mg) |
| | | | miligram_dissolved_in_liquid | miligram (mg) dissolved in liquid (e.g. 2 mg per 3 militers) |
| | | | gram | gram (g) |
| | | | militer | militer (ml) |
| | | | centiliter | centiliter (cl) |
| | | | litre | litre (l) |
| | | | ml_of _solution | ml of solution (e.g. ml of 10% solution) |
| | | | litre_of_solution | litre of solution (e.g. 1 litre of 5% solution) |
| | | | cubic_meter | cubic meter (m^3) |
| | | | other | other |
| | | | dont_know | don't know |
| q604_M75.2c.1 | q604_M75.2c.1 If other unit of measurement or route of administration, specify here: | | | |

| Field | Question | Answer |
|---|---|---|
| | *Leave blank if not applicable*<br>*Question relevant when: selected( ${q104_M75.2_full_list} , '2')* | |
| q604_M75.2d *(required)* | q604_M75.2d. In total, how many units are used to treat a patient during all of her care?<br>*Remember to add all units! E.g. 5 days x 200 mg twice per day = 2,000mg*<br>*Question relevant when: selected( ${q104_M75.2_full_list} , '2')*<br>*Response constrained to: .>0* | |
| <p><strong><em>D. Medications - Perforations (8)</em></strong></p><br>*Group relevant when: selected( ${section_six_skip_med} , '1')* | | |
| q604_med_note8 | 604 CONTINUED For each of the following medications and other medical products that are used for perforations, can you tell me …? | |
| note_604_hemostatic | ***Hemostatics*** | |
| q604_M76 | ***q604_M76. Dicynone / Dicynene (Etamsylate)***<br>*Question relevant when: selected( ${q104_M76_full_list} , '5')* | |
| q604_M76a *(required)* | q604_M76a. What percent of patients require this item?<br>*Question relevant when: selected( ${q104_M76_full_list} , '5')*<br>*Response constrained to: .>0 and .<=100 or .=999* | |

| Field | Question | Answer |
|---|---|---|
| q604_M76b *(required)* | q604_M76b. How is the item administered, or given, to the patient?<br>*Question relevant when: selected( ${q104_M76_full_list} , '5')* | 1 Oral - solid (e.g. tablet)<br>2 Oral - liquid<br>3 Sublingual<br>4 Intravenous (IV)<br>5 Intramusculuar injection (IM)<br>6 Subcutaneous<br>7 Rectal<br>8 Inhaled (e.g. gas)<br>9 Inserted (e.g. vaginal pessaries)<br>10 Topical - cream<br>11 Topical - drops (e.g. eye drops)<br>12 Other<br>99 Don't know |
| q604_M76c *(required)* | q604_M76c. What is the item's smallest unit of measurement (mg, mcg, ml, g, etc)?<br>*Question relevant when: selected( ${q104_M76_full_list} , '5')* | microgram — microgram (mcg)<br>miligram — miligram (mg)<br>miligram_dissolved_in_liquid — miligram (mg) dissolved in liquid (e.g. 2 mg per 3 militers)<br>gram — gram (g)<br>militer — militer (ml)<br>centiliter — centiliter (cl)<br>litre — litre (l)<br>ml_of _solution — ml of solution (e.g. ml of 10% solution)<br>litre_of_solution — litre of solution (e.g. 1 litre of 5% solution)<br>cubic_meter — cubic meter (m^3)<br>other — other<br>dont_know — don't know |
| q604_M76c.1 | q604_M76c.1 If other unit of measurement or route of administration, specify here: | |

| Field | Question | Answer |
|---|---|---|
| | *Leave blank if not applicable*<br>*Question relevant when: selected( ${q104_M76_full_list} , '5')* | |
| q604_M76d *(required)* | q604_M76d. In total, how many units are used to treat a patient during all of her care?<br>*Remember to add all units! E.g. 5 days x 200 mg twice per day = 2,000mg*<br>*Question relevant when: selected( ${q104_M76_full_list} , '5')*<br>*Response constrained to: .>0* | |
| q604_M77 | ***q604_M77. Tranexamic acid (Kapron, Cyklokapron)***<br>*Question relevant when: selected( ${q104_M77_full_list} , '5')* | |
| q604_M77a *(required)* | q604_M77a. What percent of patients require this item?<br>*Question relevant when: selected( ${q104_M77_full_list} , '5')*<br>*Response constrained to: .>0 and .<=100 or .=999* | |

| Field | Question | Answer | | |
|---|---|---|---|---|
| q604_M77b *(required)* | q604_M77b. How is the item administered, or given, to the patient?<br>*Question relevant when: selected( ${q104_M77_full_list} , '5')* | | 1 | Oral - solid (e.g. tablet) |
| | | | 2 | Oral - liquid |
| | | | 3 | Sublingual |
| | | | 4 | Intravenous (IV) |
| | | | 5 | Intramusculuar injection (IM) |
| | | | 6 | Subcutaneous |
| | | | 7 | Rectal |
| | | | 8 | Inhaled (e.g. gas) |
| | | | 9 | Inserted (e.g. vaginal pessaries) |
| | | | 10 | Topical - cream |
| | | | 11 | Topical - drops (e.g. eye drops) |
| | | | 12 | Other |
| | | | 99 | Don't know |
| q604_M77c *(required)* | q604_M77c. What is the item's smallest unit of measurement (mg, mcg, ml, g, etc)?<br>*Question relevant when: selected( ${q104_M77_full_list} , '5')* | | microgram | microgram (mcg) |
| | | | miligram | miligram (mg) |
| | | | miligram_dissolved_in_liquid | miligram (mg) dissolved in liquid (e.g. 2 mg per 3 militers) |
| | | | gram | gram (g) |
| | | | militer | militer (ml) |
| | | | centiliter | centiliter (cl) |
| | | | litre | litre (l) |
| | | | ml_of _solution | ml of solution (e.g. ml of 10% solution) |
| | | | litre_of_solution | litre of solution (e.g. 1 litre of 5% solution) |
| | | | cubic_meter | cubic meter (m^3) |
| | | | other | other |
| | | | dont_know | don't know |
| q604_M77c.1 | q604_M77c.1 If other unit of measurement or route of administration, specify here: | | | |

| Field | Question | Answer |
|---|---|---|
| | *Leave blank if not applicable*<br>*Question relevant when: selected( ${q104_M77_full_list} , '5')* | |
| q604_M77d *(required)* | q604_M77d. In total, how many units are used to treat a patient during all of her care?<br>*Remember to add all units! E.g. 5 days x 200 mg twice per day = 2,000mg*<br>*Question relevant when: selected( ${q104_M77_full_list} , '5')*<br>*Response constrained to: .>0* | |
| q604_M78 | ***q604_M78. Polygeline 4%***<br>*Question relevant when: selected( ${q104_M78_full_list} , '5')* | |
| q604_M78a *(required)* | q604_M78a. What percent of patients require this item?<br>*Question relevant when: selected( ${q104_M78_full_list} , '5')*<br>*Response constrained to: .>0 and .<=100 or .=999* | |

| Field | Question | Answer |
|---|---|---|
| q604_M78b *(required)* | q604_M78b. How is the item administered, or given, to the patient?<br>*Question relevant when: selected( ${q104_M78_full_list} , '5')* | 1 Oral - solid (e.g. tablet)<br>2 Oral - liquid<br>3 Sublingual<br>4 Intravenous (IV)<br>5 Intramusculuar injection (IM)<br>6 Subcutaneous<br>7 Rectal<br>8 Inhaled (e.g. gas)<br>9 Inserted (e.g. vaginal pessaries)<br>10 Topical - cream<br>11 Topical - drops (e.g. eye drops)<br>12 Other<br>99 Don't know |
| q604_M78c *(required)* | q604_M78c. What is the item's smallest unit of measurement (mg, mcg, ml, g, etc)?<br>*Question relevant when: selected( ${q104_M78_full_list} , '5')* | microgram — microgram (mcg)<br>miligram — miligram (mg)<br>miligram_dissolved_in_liquid — miligram (mg) dissolved in liquid (e.g. 2 mg per 3 militers)<br>gram — gram (g)<br>militer — militer (ml)<br>centiliter — centiliter (cl)<br>litre — litre (l)<br>ml_of _solution — ml of solution (e.g. ml of 10% solution)<br>litre_of_solution — litre of solution (e.g. 1 litre of 5% solution)<br>cubic_meter — cubic meter (m^3)<br>other — other<br>dont_know — don't know |
| q604_M78c.1 | q604_M78c.1 If other unit of measurement or route of administration, specify here: | |

| Field | Question | Answer |
|---|---|---|
| | *Leave blank if not applicable*<br>*Question relevant when: selected( ${q104_M78_full_list} , '5')* | |
| q604_M78d *(required)* | q604_M78d. In total, how many units are used to treat a patient during all of her care?<br>*Remember to add all units! E.g. 5 days x 200 mg twice per day = 2,000mg*<br>*Question relevant when: selected( ${q104_M78_full_list} , '5')*<br>*Response constrained to: .>0* | |
| q604_M79 | ***q604_M79. Hemostatic - other 1: "[q104_M79_full_list_other]"***<br>*Question relevant when: selected( ${q104_M79_full_list} , '5')* | |
| q604_M79a *(required)* | q604_M79a. What percent of patients require this item?<br>*Question relevant when: selected( ${q104_M79_full_list} , '5')*<br>*Response constrained to: .>0 and .<=100 or .=999* | |
| q604_M79b *(required)* | q604_M79b. How is the item administered, or given, to the patient?<br>*Question relevant when: selected( ${q104_M79_full_list} , '5')* | 1 Oral - solid (e.g. tablet)<br>2 Oral - liquid<br>3 Sublingual<br>4 Intravenous (IV)<br>5 Intramusculuar injection (IM)<br>6 Subcutaneous<br>7 Rectal<br>8 Inhaled (e.g. gas)<br>9 Inserted (e.g. vaginal pessaries)<br>10 Topical - cream<br>11 Topical - drops (e.g. eye drops)<br>12 Other<br>99 Don't know |
| q604_M79c *(required)* | q604_M79c. What is the item's smallest unit of measurement (mg, mcg, ml, g, etc)?<br>*Question relevant when: selected( ${q104_M79_full_list} , '5')* | microgram — microgram (mcg)<br>miligram — miligram (mg)<br>miligram_dissolved_in_liquid — miligram (mg) dissolved in liquid (e.g. 2 mg per 3 militers)<br>gram — gram (g)<br>militer — militer (ml)<br>centiliter — centiliter (cl)<br>litre — litre (l)<br>ml_of _solution — ml of solution (e.g. ml of 10% solution)<br>litre_of_solution — litre of solution (e.g. 1 litre of 5% solution)<br>cubic_meter — cubic meter (m^3)<br>other — other<br>dont_know — don't know |
| q604_M79c.1 | q604_M79c.1 If other unit of measurement or route of administration, specify here: | |

| Field | Question | Answer |
|---|---|---|
| | *Leave blank if not applicable*<br>*Question relevant when: selected( ${q104_M79_full_list} , '5')* | |
| q604_M79d *(required)* | q604_M79d. In total, how many units are used to treat a patient during all of her care?<br>*Remember to add all units! E.g. 5 days x 200 mg twice per day = 2,000mg*<br>*Question relevant when: selected( ${q104_M79_full_list} , '5')*<br>*Response constrained to: .>0* | |
| q604_M80 | ***q604_M80. Hemostatic - other 2: "[q104_M80_full_list_other]"***<br>*Question relevant when: selected( ${q104_M80_full_list} , '5')* | |
| q604_M80a *(required)* | q604_M80a. What percent of patients require this item?<br>*Question relevant when: selected( ${q104_M80_full_list} , '5')*<br>*Response constrained to: .>0 and .<=100 or .=999* | |
| q604_M80b *(required)* | q604_M80b. How is the item administered, or given, to the patient?<br>*Question relevant when: selected( ${q104_M80_full_list} , '5')* | 1 Oral - solid (e.g. tablet)<br>2 Oral - liquid<br>3 Sublingual<br>4 Intravenous (IV)<br>5 Intramusculuar injection (IM)<br>6 Subcutaneous<br>7 Rectal<br>8 Inhaled (e.g. gas)<br>9 Inserted (e.g. vaginal pessaries)<br>10 Topical - cream<br>11 Topical - drops (e.g. eye drops)<br>12 Other<br>99 Don't know |
| q604_M80c *(required)* | q604_M80c. What is the item's smallest unit of measurement (mg, mcg, ml, g, etc)?<br>*Question relevant when: selected( ${q104_M80_full_list} , '5')* | microgram: microgram (mcg)<br>miligram: miligram (mg)<br>miligram_dissolved_in_liquid: miligram (mg) dissolved in liquid (e.g. 2 mg per 3 militers)<br>gram: gram (g)<br>militer: militer (ml)<br>centiliter: centiliter (cl)<br>litre: litre (l)<br>ml_of _solution: ml of solution (e.g. ml of 10% solution)<br>litre_of_solution: litre of solution (e.g. 1 litre of 5% solution)<br>cubic_meter: cubic meter (m^3)<br>other: other<br>dont_know: don't know |
| q604_M80c.1 | q604_M80c.1 If other unit of measurement or route of administration, specify here: | |

| Field | Question | Answer |
|---|---|---|
| | *Leave blank if not applicable*<br>*Question relevant when: selected( ${q104_M80_full_list} , '5')* | |
| q604_M80d *(required)* | q604_M80d. In total, how many units are used to treat a patient during all of her care?<br>*Remember to add all units! E.g. 5 days x 200 mg twice per day = 2,000mg*<br>*Question relevant when: selected( ${q104_M80_full_list} , '5')*<br>*Response constrained to: .>0* | |
| q604_M81 | ***q604_M81. Hemostatic - other 3: "[q104_M81_full_list_other]"***<br>*Question relevant when: selected( ${q104_M81_full_list} , '5')* | |
| q604_M81a *(required)* | q604_M81a. What percent of patients require this item?<br>*Question relevant when: selected( ${q104_M81_full_list} , '5')*<br>*Response constrained to: .>0 and .<=100 or .=999* | |

| Field | Question | Answer |
|---|---|---|
| q604_M81b *(required)* | q604_M81b. How is the item administered, or given, to the patient?<br>*Question relevant when: selected( ${q104_M81_full_list} , '5')* | 1 Oral - solid (e.g. tablet)<br>2 Oral - liquid<br>3 Sublingual<br>4 Intravenous (IV)<br>5 Intramusculuar injection (IM)<br>6 Subcutaneous<br>7 Rectal<br>8 Inhaled (e.g. gas)<br>9 Inserted (e.g. vaginal pessaries)<br>10 Topical - cream<br>11 Topical - drops (e.g. eye drops)<br>12 Other<br>99 Don't know |
| q604_M81c *(required)* | q604_M81c. What is the item's smallest unit of measurement (mg, mcg, ml, g, etc)?<br>*Question relevant when: selected( ${q104_M81_full_list} , '5')* | microgram — microgram (mcg)<br>miligram — miligram (mg)<br>miligram_dissolved_in_liquid — miligram (mg) dissolved in liquid (e.g. 2 mg per 3 militers)<br>gram — gram (g)<br>militer — militer (ml)<br>centiliter — centiliter (cl)<br>litre — litre (l)<br>ml_of _solution — ml of solution (e.g. ml of 10% solution)<br>litre_of_solution — litre of solution (e.g. 1 litre of 5% solution)<br>cubic_meter — cubic meter (m^3)<br>other — other<br>dont_know — don't know |

| Field | Question | Answer |
|---|---|---|
| q604_M81c.1 | q604_M81c.1 If other unit of measurement or route of administration, specify here:<br>*Leave blank if not applicable*<br>*Question relevant when: selected( ${q104_M81_full_list} , '5')* | |
| q604_M81d *(required)* | q604_M81d. In total, how many units are used to treat a patient during all of her care?<br>*Remember to add all units! E.g. 5 days x 200 mg twice per day = 2,000mg*<br>*Question relevant when: selected( ${q104_M81_full_list} , '5')*<br>*Response constrained to: .>0* | |
| q604_M81.1 | ***q604_M81.1. Hemostatic - other 4: "[q104_M81.1_full_list_other]"***<br>*Question relevant when: selected( ${q104_M81.1_full_list} , '2')* | |
| q604_M81.1a *(required)* | q604_M81.1a. What percent of patients require this item?<br>*Question relevant when: selected( ${q104_M81.1_full_list} , '2')*<br>*Response constrained to: .>0 and .<=100 or .=999* | |
| q604_M81.1b *(required)* | q604_M81.1b. How is the item administered, or given, to the patient?<br>*Question relevant when: selected( ${q104_M81.1_full_list} , '2')* | 1 Oral - solid (e.g. tablet)<br>2 Oral - liquid<br>3 Sublingual<br>4 Intravenous (IV)<br>5 Intramusculuar injection (IM)<br>6 Subcutaneous<br>7 Rectal<br>8 Inhaled (e.g. gas)<br>9 Inserted (e.g. vaginal pessaries)<br>10 Topical - cream<br>11 Topical - drops (e.g. eye drops)<br>12 Other<br>99 Don't know |
| q604_M81.1c *(required)* | q604_M81.1c. What is the item's smallest unit of measurement (mg, mcg, ml, g, etc)?<br>*Question relevant when: selected( ${q104_M81.1_full_list} , '2')* | microgram — microgram (mcg)<br>miligram — miligram (mg)<br>miligram_dissolved_in_liquid — miligram (mg) dissolved in liquid (e.g. 2 mg per 3 militers)<br>gram — gram (g)<br>militer — militer (ml)<br>centiliter — centiliter (cl)<br>litre — litre (l)<br>ml_of _solution — ml of solution (e.g. ml of 10% solution)<br>litre_of_solution — litre of solution (e.g. 1 litre of 5% solution)<br>cubic_meter — cubic meter (m^3)<br>other — other<br>dont_know — don't know |

| Field | Question | Answer |
|---|---|---|
| q604_M81.1c.1 | q604_M81.1c.1 If other unit of measurement or route of administration, specify here:<br>*Leave blank if not applicable*<br>*Question relevant when: selected( ${q104_M81.1_full_list} , '2')* | |
| q604_M81.1d *(required)* | q604_M81.1d. In total, how many units are used to treat a patient during all of her care?<br>*Remember to add all units! E.g. 5 days x 200 mg twice per day = 2,000mg*<br>*Question relevant when: selected( ${q104_M81.1_full_list} , '2')*<br>*Response constrained to: .>0* | |
| q604_M81.2 | ***q604_M81.2. Hemostatic - other 5: "[q104_M81.2_full_list_other]"***<br>*Question relevant when: selected( ${q104_M81.2_full_list} , '2')* | |
| q604_M81.2a *(required)* | q604_M81.2a. What percent of patients require this item?<br>*Question relevant when: selected( ${q104_M81.2_full_list} , '2')*<br>*Response constrained to: .>0 and .<=100 or .=999* | |
| q604_M81.2b *(required)* | q604_M81.2b. How is the item administered, or given, to the patient?<br>*Question relevant when: selected( ${q104_M81.2_full_list} , '2')* | 1 Oral - solid (e.g. tablet)<br>2 Oral - liquid<br>3 Sublingual<br>4 Intravenous (IV)<br>5 Intramusculuar injection (IM)<br>6 Subcutaneous<br>7 Rectal<br>8 Inhaled (e.g. gas)<br>9 Inserted (e.g. vaginal pessaries)<br>10 Topical - cream<br>11 Topical - drops (e.g. eye drops)<br>12 Other<br>99 Don't know |
| q604_M81.2c *(required)* | q604_M81.2c. What is the item's smallest unit of measurement (mg, mcg, ml, g, etc)?<br>*Question relevant when: selected( ${q104_M81.2_full_list} , '2')* | microgram — microgram (mcg)<br>miligram — miligram (mg)<br>miligram_dissolved_in_liquid — miligram (mg) dissolved in liquid (e.g. 2 mg per 3 militers)<br>gram — gram (g)<br>militer — militer (ml)<br>centiliter — centiliter (cl)<br>litre — litre (l)<br>ml_of _solution — ml of solution (e.g. ml of 10% solution)<br>litre_of_solution — litre of solution (e.g. 1 litre of 5% solution)<br>cubic_meter — cubic meter (m^3)<br>other — other<br>dont_know — don't know |

| Field | Question | Answer |
|---|---|---|
| q604_M81.2c.1 | q604_M81.2c.1 If other unit of measurement or route of administration, specify here:<br>*Leave blank if not applicable*<br>*Question relevant when: selected( ${q104_M81.2_full_list} , '2')* | |
| q604_M81.2d *(required)* | q604_M81.2d. In total, how many units are used to treat a patient during all of her care?<br>*Remember to add all units! E.g. 5 days x 200 mg twice per day = 2,000mg*<br>*Question relevant when: selected( ${q104_M81.2_full_list} , '2')*<br>*Response constrained to: .>0* | |
| <p><strong><em>D. Medications - Perforations (9)</em></strong></p><br>*Group relevant when: selected( ${section_six_skip_med} , '1')* | | |
| q604_med_notE8 | 604 CONTINUED For each of the following medications and other medical products that are used for perforations, can you tell me …? | |
| note_604_saline | ***Saline, glucose, water, etc*** | |
| q604_M82 | ***q604_M82.IV solution, dextrose 2.5% in NaCl 0.45%***<br>*Question relevant when: selected( ${q104_M82_full_list} , '5')* | |
| q604_M82a *(required)* | q604_M82a. What percent of patients require this item?<br>*Question relevant when: selected( ${q104_M82_full_list} , '5')*<br>*Response constrained to: .>0 and .<=100 or .=999* | |
| q604_M82b *(required)* | q604_M82b. How is the item administered, or given, to the patient?<br>*Question relevant when: selected( ${q104_M82_full_list} , '5')* | 1 Oral - solid (e.g. tablet)<br>2 Oral - liquid<br>3 Sublingual<br>4 Intravenous (IV)<br>5 Intramusculuar injection (IM)<br>6 Subcutaneous<br>7 Rectal<br>8 Inhaled (e.g. gas)<br>9 Inserted (e.g. vaginal pessaries)<br>10 Topical - cream<br>11 Topical - drops (e.g. eye drops)<br>12 Other<br>99 Don't know |
| q604_M82c *(required)* | q604_M82c. What is the item's smallest unit of measurement (mg, mcg, ml, g, etc)?<br>*Question relevant when: selected( ${q104_M82_full_list} , '5')* | microgram — microgram (mcg)<br>miligram — miligram (mg)<br>miligram_dissolved_in_liquid — miligram (mg) dissolved in liquid (e.g. 2 mg per 3 militers)<br>gram — gram (g)<br>militer — militer (ml)<br>centiliter — centiliter (cl)<br>litre — litre (l)<br>ml_of _solution — ml of solution (e.g. ml of 10% solution)<br>litre_of_solution — litre of solution (e.g. 1 litre of 5% solution)<br>cubic_meter — cubic meter (m^3)<br>other — other<br>dont_know — don't know |

| Field | Question | Answer |
|---|---|---|
| q604_M82c.1 | q604_M82c.1 If other unit of measurement or route of administration, specify here:<br>*Leave blank if not applicable*<br>*Question relevant when: selected( ${q104_M82_full_list} , '5')* | |
| q604_M82d *(required)* | q604_M82d. In total, how many units are used to treat a patient during all of her care?<br>*Remember to add all units! E.g. 5 days x 200 mg twice per day = 2,000mg*<br>*Question relevant when: selected( ${q104_M82_full_list} , '5')*<br>*Response constrained to: .>0* | |
| q604_M83 | ***q604_M83.IV solution, glucose (5%), isotonic***<br>*Question relevant when: selected( ${q104_M83_full_list} , '5')* | |
| q604_M83a *(required)* | q604_M83a. What percent of patients require this item?<br>*Question relevant when: selected( ${q104_M83_full_list} , '5')*<br>*Response constrained to: .>0 and .<=100 or .=999* | |
| q604_M83b *(required)* | q604_M83b. How is the item administered, or given, to the patient?<br>*Question relevant when: selected( ${q104_M83_full_list} , '5')* | 1 Oral - solid (e.g. tablet)<br>2 Oral - liquid<br>3 Sublingual<br>4 Intravenous (IV)<br>5 Intramusculuar injection (IM)<br>6 Subcutaneous<br>7 Rectal<br>8 Inhaled (e.g. gas)<br>9 Inserted (e.g. vaginal pessaries)<br>10 Topical - cream<br>11 Topical - drops (e.g. eye drops)<br>12 Other<br>99 Don't know |
| q604_M83c *(required)* | q604_M83c. What is the item's smallest unit of measurement (mg, mcg, ml, g, etc)?<br>*Question relevant when: selected( ${q104_M83_full_list} , '5')* | microgram — microgram (mcg)<br>miligram — miligram (mg)<br>miligram_dissolved_in_liquid — miligram (mg) dissolved in liquid (e.g. 2 mg per 3 militers)<br>gram — gram (g)<br>militer — militer (ml)<br>centiliter — centiliter (cl)<br>litre — litre (l)<br>ml_of _solution — ml of solution (e.g. ml of 10% solution)<br>litre_of_solution — litre of solution (e.g. 1 litre of 5% solution)<br>cubic_meter — cubic meter (m^3)<br>other — other<br>dont_know — don't know |

| Field | Question | Answer |
|---|---|---|
| q604_M83c.1 | q604_M83c.1 If other unit of measurement or route of administration, specify here:<br>*Leave blank if not applicable*<br>*Question relevant when: selected( ${q104_M83_full_list} , '5')* | |
| q604_M83d *(required)* | q604_M83d. In total, how many units are used to treat a patient during all of her care?<br>*Remember to add all units! E.g. 5 days x 200 mg twice per day = 2,000mg*<br>*Question relevant when: selected( ${q104_M83_full_list} , '5')*<br>*Response constrained to: .>0* | |
| q604_M84 | ***q604_M84. Ringers lactate (prepared onsite)***<br>*Question relevant when: selected( ${q104_M84_full_list} , '5')* | |
| q604_M84a *(required)* | q604_M84a. What percent of patients require this item?<br>*Question relevant when: selected( ${q104_M84_full_list} , '5')*<br>*Response constrained to: .>0 and .<=100 or .=999* | |
| q604_M84b *(required)* | q604_M84b. How is the item administered, or given, to the patient?<br>*Question relevant when: selected( ${q104_M84_full_list} , '5')* | 1 Oral - solid (e.g. tablet)<br>2 Oral - liquid<br>3 Sublingual<br>4 Intravenous (IV)<br>5 Intramusculuar injection (IM)<br>6 Subcutaneous<br>7 Rectal<br>8 Inhaled (e.g. gas)<br>9 Inserted (e.g. vaginal pessaries)<br>10 Topical - cream<br>11 Topical - drops (e.g. eye drops)<br>12 Other<br>99 Don't know |
| q604_M84c *(required)* | q604_M84c. What is the item's smallest unit of measurement (mg, mcg, ml, g, etc)?<br>*Question relevant when: selected( ${q104_M84_full_list} , '5')* | microgram — microgram (mcg)<br>miligram — miligram (mg)<br>miligram_dissolved_in_liquid — miligram (mg) dissolved in liquid (e.g. 2 mg per 3 militers)<br>gram — gram (g)<br>militer — militer (ml)<br>centiliter — centiliter (cl)<br>litre — litre (l)<br>ml_of _solution — ml of solution (e.g. ml of 10% solution)<br>litre_of_solution — litre of solution (e.g. 1 litre of 5% solution)<br>cubic_meter — cubic meter (m^3)<br>other — other<br>dont_know — don't know |

| Field | Question | Answer |
|---|---|---|
| q604_M84c.1 | q604_M84c.1 If other unit of measurement or route of administration, specify here:<br>*Leave blank if not applicable*<br>*Question relevant when: selected( ${q104_M84_full_list} , '5')* | |
| q604_M84d *(required)* | q604_M84d. In total, how many units are used to treat a patient during all of her care?<br>*Remember to add all units! E.g. 5 days x 200 mg twice per day = 2,000mg*<br>*Question relevant when: selected( ${q104_M84_full_list} , '5')*<br>*Response constrained to: .>0* | |
| q604_M85 | ***q604_M85. Ringers lactate (bought from pharmacy/MSD)***<br>*Question relevant when: selected( ${q104_M85_full_list} , '5')* | |
| q604_M85a *(required)* | q604_M85a. What percent of patients require this item?<br>*Question relevant when: selected( ${q104_M85_full_list} , '5')*<br>*Response constrained to: .>0 and .<=100 or .=999* | |
| q604_M85b *(required)* | q604_M85b. How is the item administered, or given, to the patient?<br>*Question relevant when: selected( ${q104_M85_full_list} , '5')* | 1 Oral - solid (e.g. tablet)<br>2 Oral - liquid<br>3 Sublingual<br>4 Intravenous (IV)<br>5 Intramusculuar injection (IM)<br>6 Subcutaneous<br>7 Rectal<br>8 Inhaled (e.g. gas)<br>9 Inserted (e.g. vaginal pessaries)<br>10 Topical - cream<br>11 Topical - drops (e.g. eye drops)<br>12 Other<br>99 Don't know |
| q604_M85c *(required)* | q604_M85c. What is the item's smallest unit of measurement (mg, mcg, ml, g, etc)?<br>*Question relevant when: selected( ${q104_M85_full_list} , '5')* | microgram — microgram (mcg)<br>miligram — miligram (mg)<br>miligram_dissolved_in_liquid — miligram (mg) dissolved in liquid (e.g. 2 mg per 3 militers)<br>gram — gram (g)<br>militer — militer (ml)<br>centiliter — centiliter (cl)<br>litre — litre (l)<br>ml_of _solution — ml of solution (e.g. ml of 10% solution)<br>litre_of_solution — litre of solution (e.g. 1 litre of 5% solution)<br>cubic_meter — cubic meter (m^3)<br>other — other<br>dont_know — don't know |

| Field | Question | Answer |
|---|---|---|
| q604_M85c.1 | q604_M85c.1 If other unit of measurement or route of administration, specify here:<br>*Leave blank if not applicable*<br>*Question relevant when: selected( ${q104_M85_full_list} , '5')* | |
| q604_M85d *(required)* | q604_M85d. In total, how many units are used to treat a patient during all of her care?<br>*Remember to add all units! E.g. 5 days x 200 mg twice per day = 2,000mg*<br>*Question relevant when: selected( ${q104_M85_full_list} , '5')*<br>*Response constrained to: .>0* | |
| q604_M86 | ***q604_M86. Saline, 0.9% (Normal Saline) (prepared onsite)***<br>*Question relevant when: selected( ${q104_M86_full_list} , '5')* | |
| q604_M86a *(required)* | q604_M86a. What percent of patients require this item?<br>*Question relevant when: selected( ${q104_M86_full_list} , '5')*<br>*Response constrained to: .>0 and .<=100 or .=999* | |
| q604_M86b *(required)* | q604_M86b. How is the item administered, or given, to the patient?<br>*Question relevant when: selected( ${q104_M86_full_list} , '5')* | 1 Oral - solid (e.g. tablet)<br>2 Oral - liquid<br>3 Sublingual<br>4 Intravenous (IV)<br>5 Intramusculuar injection (IM)<br>6 Subcutaneous<br>7 Rectal<br>8 Inhaled (e.g. gas)<br>9 Inserted (e.g. vaginal pessaries)<br>10 Topical - cream<br>11 Topical - drops (e.g. eye drops)<br>12 Other<br>99 Don't know |
| q604_M86c *(required)* | q604_M86c. What is the item's smallest unit of measurement (mg, mcg, ml, g, etc)?<br>*Question relevant when: selected( ${q104_M86_full_list} , '5')* | microgram — microgram (mcg)<br>miligram — miligram (mg)<br>miligram_dissolved_in_liquid — miligram (mg) dissolved in liquid (e.g. 2 mg per 3 militers)<br>gram — gram (g)<br>militer — militer (ml)<br>centiliter — centiliter (cl)<br>litre — litre (l)<br>ml_of _solution — ml of solution (e.g. ml of 10% solution)<br>litre_of_solution — litre of solution (e.g. 1 litre of 5% solution)<br>cubic_meter — cubic meter (m^3)<br>other — other<br>dont_know — don't know |

| Field | Question | Answer |
|---|---|---|
| q604_M86c.1 | q604_M86c.1 If other unit of measurement or route of administration, specify here:<br>*Leave blank if not applicable*<br>*Question relevant when: selected( ${q104_M86_full_list} , '5')* | |
| q604_M86d *(required)* | q604_M86d. In total, how many units are used to treat a patient during all of her care?<br>*Remember to add all units! E.g. 5 days x 200 mg twice per day = 2,000mg*<br>*Question relevant when: selected( ${q104_M86_full_list} , '5')*<br>*Response constrained to: .>0* | |
| q604_M87 | ***q604_M87. Saline, 0.9% (Normal Saline) (bought from pharmacy/MSD)***<br>*Question relevant when: selected( ${q104_M87_full_list} , '5')* | |
| q604_M87a *(required)* | q604_M87a. What percent of patients require this item?<br>*Question relevant when: selected( ${q104_M87_full_list} , '5')*<br>*Response constrained to: .>0 and .<=100 or .=999* | |

| Field | Question | Answer | | |
|---|---|---|---|---|
| q604_M87b *(required)* | q604_M87b. How is the item administered, or given, to the patient?<br>*Question relevant when: selected( ${q104_M87_full_list} , '5')* | | 1 | Oral - solid (e.g. tablet) |
| | | | 2 | Oral - liquid |
| | | | 3 | Sublingual |
| | | | 4 | Intravenous (IV) |
| | | | 5 | Intramusculuar injection (IM) |
| | | | 6 | Subcutaneous |
| | | | 7 | Rectal |
| | | | 8 | Inhaled (e.g. gas) |
| | | | 9 | Inserted (e.g. vaginal pessaries) |
| | | | 10 | Topical - cream |
| | | | 11 | Topical - drops (e.g. eye drops) |
| | | | 12 | Other |
| | | | 99 | Don't know |
| q604_M87c *(required)* | q604_M87c. What is the item's smallest unit of measurement (mg, mcg, ml, g, etc)?<br>*Question relevant when: selected( ${q104_M87_full_list} , '5')* | | microgram | microgram (mcg) |
| | | | miligram | miligram (mg) |
| | | | miligram_dissolved_in_liquid | miligram (mg) dissolved in liquid (e.g. 2 mg per 3 militers) |
| | | | gram | gram (g) |
| | | | militer | militer (ml) |
| | | | centiliter | centiliter (cl) |
| | | | litre | litre (l) |
| | | | ml_of _solution | ml of solution (e.g. ml of 10% solution) |
| | | | litre_of_solution | litre of solution (e.g. 1 litre of 5% solution) |
| | | | cubic_meter | cubic meter (m^3) |
| | | | other | other |
| | | | dont_know | don't know |

| Field | Question | Answer |
|---|---|---|
| q604_M87c.1 | q604_M87c.1 If other unit of measurement or route of administration, specify here:<br>*Leave blank if not applicable*<br>*Question relevant when: selected( ${q104_M87_full_list} , '5')* | |
| q604_M87d *(required)* | q604_M87d. In total, how many units are used to treat a patient during all of her care?<br>*Remember to add all units! E.g. 5 days x 200 mg twice per day = 2,000mg*<br>*Question relevant when: selected( ${q104_M87_full_list} , '5')*<br>*Response constrained to: .>0* | |
| q604_M88 | ***q604_M88. Solution for Infusion, glucose 5% + NaCl 0.9% (prepared onsite)***<br>*Question relevant when: selected( ${q104_M88_full_list} , '5')* | |
| q604_M88a *(required)* | q604_M88a. What percent of patients require this item?<br>*Question relevant when: selected( ${q104_M88_full_list} , '5')*<br>*Response constrained to: .>0 and .<=100 or .=999* | |
| q604_M88b *(required)* | q604_M88b. How is the item administered, or given, to the patient?<br>*Question relevant when: selected( ${q104_M88_full_list} , '5')* | 1 Oral - solid (e.g. tablet)<br>2 Oral - liquid<br>3 Sublingual<br>4 Intravenous (IV)<br>5 Intramusculuar injection (IM)<br>6 Subcutaneous<br>7 Rectal<br>8 Inhaled (e.g. gas)<br>9 Inserted (e.g. vaginal pessaries)<br>10 Topical - cream<br>11 Topical - drops (e.g. eye drops)<br>12 Other<br>99 Don't know |
| q604_M88c *(required)* | q604_M88c. What is the item's smallest unit of measurement (mg, mcg, ml, g, etc)?<br>*Question relevant when: selected( ${q104_M88_full_list} , '5')* | microgram — microgram (mcg)<br>miligram — miligram (mg)<br>miligram_dissolved_in_liquid — miligram (mg) dissolved in liquid (e.g. 2 mg per 3 militers)<br>gram — gram (g)<br>militer — militer (ml)<br>centiliter — centiliter (cl)<br>litre — litre (l)<br>ml_of _solution — ml of solution (e.g. ml of 10% solution)<br>litre_of_solution — litre of solution (e.g. 1 litre of 5% solution)<br>cubic_meter — cubic meter (m^3)<br>other — other<br>dont_know — don't know |

| Field | Question | Answer |
|---|---|---|
| q604_M88c.1 | q604_M88c.1 If other unit of measurement or route of administration, specify here:<br>*Leave blank if not applicable*<br>*Question relevant when: selected( ${q104_M88_full_list} , '5')* | |
| q604_M88d *(required)* | q604_M88d. In total, how many units are used to treat a patient during all of her care?<br>*Remember to add all units! E.g. 5 days x 200 mg twice per day = 2,000mg*<br>*Question relevant when: selected( ${q104_M88_full_list} , '5')*<br>*Response constrained to: .>0* | |
| q604_M89 | ***q604_M89. Solution for Infusion, glucose 5% + NaCl 0.9% (bought from pharmacy or MSD)***<br>*Question relevant when: selected( ${q104_M89_full_list} , '5')* | |
| q604_M89a *(required)* | q604_M89a. What percent of patients require this item?<br>*Question relevant when: selected( ${q104_M89_full_list} , '5')*<br>*Response constrained to: .>0 and .<=100 or .=999* | |
| q604_M89b *(required)* | q604_M89b. How is the item administered, or given, to the patient?<br>*Question relevant when: selected( ${q104_M89_full_list} , '5')* | 1 Oral - solid (e.g. tablet)<br>2 Oral - liquid<br>3 Sublingual<br>4 Intravenous (IV)<br>5 Intramusculuar injection (IM)<br>6 Subcutaneous<br>7 Rectal<br>8 Inhaled (e.g. gas)<br>9 Inserted (e.g. vaginal pessaries)<br>10 Topical - cream<br>11 Topical - drops (e.g. eye drops)<br>12 Other<br>99 Don't know |
| q604_M89c *(required)* | q604_M89c. What is the item's smallest unit of measurement (mg, mcg, ml, g, etc)?<br>*Question relevant when: selected( ${q104_M89_full_list} , '5')* | microgram — microgram (mcg)<br>miligram — miligram (mg)<br>miligram_dissolved_in_liquid — miligram (mg) dissolved in liquid (e.g. 2 mg per 3 militers)<br>gram — gram (g)<br>militer — militer (ml)<br>centiliter — centiliter (cl)<br>litre — litre (l)<br>ml_of _solution — ml of solution (e.g. ml of 10% solution)<br>litre_of_solution — litre of solution (e.g. 1 litre of 5% solution)<br>cubic_meter — cubic meter (m^3)<br>other — other<br>dont_know — don't know |

| Field | Question | Answer |
|---|---|---|
| q604_M89c.1 | q604_M89c.1 If other unit of measurement or route of administration, specify here:<br>*Leave blank if not applicable*<br>*Question relevant when: selected( ${q104_M89_full_list} , '5')* | |
| q604_M89d *(required)* | q604_M89d. In total, how many units are used to treat a patient during all of her care?<br>*Remember to add all units! E.g. 5 days x 200 mg twice per day = 2,000mg*<br>*Question relevant when: selected( ${q104_M89_full_list} , '5')*<br>*Response constrained to: .>0* | |
| q604_M90 | ***q604_M90. Water for injection***<br>*Question relevant when: selected( ${q104_M90_full_list} , '5')* | |
| q604_M90a *(required)* | q604_M90a. What percent of patients require this item?<br>*Question relevant when: selected( ${q104_M90_full_list} , '5')*<br>*Response constrained to: .>0 and .<=100 or .=999* | |
| q604_M90b *(required)* | q604_M90b. How is the item administered, or given, to the patient?<br>*Question relevant when: selected( ${q104_M90_full_list} , '5')* | 1 Oral - solid (e.g. tablet)<br>2 Oral - liquid<br>3 Sublingual<br>4 Intravenous (IV)<br>5 Intramusculuar injection (IM)<br>6 Subcutaneous<br>7 Rectal<br>8 Inhaled (e.g. gas)<br>9 Inserted (e.g. vaginal pessaries)<br>10 Topical - cream<br>11 Topical - drops (e.g. eye drops)<br>12 Other<br>99 Don't know |
| q604_M90c *(required)* | q604_M90c. What is the item's smallest unit of measurement (mg, mcg, ml, g, etc)?<br>*Question relevant when: selected( ${q104_M90_full_list} , '5')* | microgram — microgram (mcg)<br>miligram — miligram (mg)<br>miligram_dissolved_in_liquid — miligram (mg) dissolved in liquid (e.g. 2 mg per 3 militers)<br>gram — gram (g)<br>militer — militer (ml)<br>centiliter — centiliter (cl)<br>litre — litre (l)<br>ml_of _solution — ml of solution (e.g. ml of 10% solution)<br>litre_of_solution — litre of solution (e.g. 1 litre of 5% solution)<br>cubic_meter — cubic meter (m^3)<br>other — other<br>dont_know — don't know |

| Field | Question | Answer |
|---|---|---|
| q604_M90c.1 | q604_M90c.1 If other unit of measurement or route of administration, specify here:<br>*Leave blank if not applicable*<br>*Question relevant when: selected( ${q104_M90_full_list} , '5')* | |
| q604_M90d *(required)* | q604_M90d. In total, how many units are used to treat a patient during all of her care?<br>*Remember to add all units! E.g. 5 days x 200 mg twice per day = 2,000mg*<br>*Question relevant when: selected( ${q104_M90_full_list} , '5')*<br>*Response constrained to: .>0* | |
| q604_M91 | ***q604_M91. Saline, glucose, water, etc. - Other 1: "[q104_M91_full_list_other]"***<br>*Question relevant when: selected( ${q104_M91_full_list} , '5')* | |
| q604_M91a *(required)* | q604_M91a. What percent of patients require this item?<br>*Question relevant when: selected( ${q104_M91_full_list} , '5')*<br>*Response constrained to: .>0 and .<=100 or .=999* | |

| Field | Question | Answer |
|---|---|---|
| q604_M91b *(required)* | q604_M91b. How is the item administered, or given, to the patient?<br>*Question relevant when: selected( ${q104_M91_full_list} , '5')* | 1 Oral - solid (e.g. tablet)<br>2 Oral - liquid<br>3 Sublingual<br>4 Intravenous (IV)<br>5 Intramusculuar injection (IM)<br>6 Subcutaneous<br>7 Rectal<br>8 Inhaled (e.g. gas)<br>9 Inserted (e.g. vaginal pessaries)<br>10 Topical - cream<br>11 Topical - drops (e.g. eye drops)<br>12 Other<br>99 Don't know |
| q604_M91c *(required)* | q604_M91c. What is the item's smallest unit of measurement (mg, mcg, ml, g, etc)?<br>*Question relevant when: selected( ${q104_M91_full_list} , '5')* | microgram — microgram (mcg)<br>miligram — miligram (mg)<br>miligram_dissolved_in_liquid — miligram (mg) dissolved in liquid (e.g. 2 mg per 3 militers)<br>gram — gram (g)<br>militer — militer (ml)<br>centiliter — centiliter (cl)<br>litre — litre (l)<br>ml_of _solution — ml of solution (e.g. ml of 10% solution)<br>litre_of_solution — litre of solution (e.g. 1 litre of 5% solution)<br>cubic_meter — cubic meter (m^3)<br>other — other<br>dont_know — don't know |

| Field | Question | Answer |
|---|---|---|
| q604_M91c.1 | q604_M91c.1 If other unit of measurement or route of administration, specify here:<br>*Leave blank if not applicable*<br>*Question relevant when: selected( ${q104_M91_full_list} , '5')* | |
| q604_M91d *(required)* | q604_M91d. In total, how many units are used to treat a patient during all of her care?<br>*Remember to add all units! E.g. 5 days x 200 mg twice per day = 2,000mg*<br>*Question relevant when: selected( ${q104_M91_full_list} , '5')*<br>*Response constrained to: .>0* | |
| q604_M92 | ***q604_M92. Saline, glucose, water, etc. - Other 2: "[q104_M92_full_list_other]"***<br>*Question relevant when: selected( ${q104_M92_full_list} , '5')* | |
| q604_M92a *(required)* | q604_M92a. What percent of patients require this item?<br>*Question relevant when: selected( ${q104_M92_full_list} , '5')*<br>*Response constrained to: .>0 and .<=100 or .=999* | |
| q604_M92b *(required)* | q604_M92b. How is the item administered, or given, to the patient?<br>*Question relevant when: selected( ${q104_M92_full_list} , '5')* | 1 Oral - solid (e.g. tablet)<br>2 Oral - liquid<br>3 Sublingual<br>4 Intravenous (IV)<br>5 Intramusculuar injection (IM)<br>6 Subcutaneous<br>7 Rectal<br>8 Inhaled (e.g. gas)<br>9 Inserted (e.g. vaginal pessaries)<br>10 Topical - cream<br>11 Topical - drops (e.g. eye drops)<br>12 Other<br>99 Don't know |
| q604_M92c *(required)* | q604_M92c. What is the item's smallest unit of measurement (mg, mcg, ml, g, etc)?<br>*Question relevant when: selected( ${q104_M92_full_list} , '5')* | microgram — microgram (mcg)<br>miligram — miligram (mg)<br>miligram_dissolved_in_liquid — miligram (mg) dissolved in liquid (e.g. 2 mg per 3 militers)<br>gram — gram (g)<br>militer — militer (ml)<br>centiliter — centiliter (cl)<br>litre — litre (l)<br>ml_of _solution — ml of solution (e.g. ml of 10% solution)<br>litre_of_solution — litre of solution (e.g. 1 litre of 5% solution)<br>cubic_meter — cubic meter (m^3)<br>other — other<br>dont_know — don't know |

| Field | Question | Answer |
|---|---|---|
| q604_M92c.1 | q604_M92c.1 If other unit of measurement or route of administration, specify here: *Leave blank if not applicable* *Question relevant when: selected( ${q104_M92_full_list} , '5')* | |
| q604_M92d *(required)* | q604_M92d. In total, how many units are used to treat a patient during all of her care? *Remember to add all units! E.g. 5 days x 200 mg twice per day = 2,000mg* *Question relevant when: selected( ${q104_M92_full_list} , '5')* *Response constrained to: .>0* | |
| q604_M93 | ***q604_M93. Saline, glucose, water, etc. - Other 3: "[q104_M93_full_list_other]"*** *Question relevant when: selected( ${q104_M93_full_list} , '5')* | |
| q604_M93a *(required)* | q604_M93a. What percent of patients require this item? *Question relevant when: selected( ${q104_M93_full_list} , '5')* *Response constrained to: .>0 and .<=100 or .=999* | |
| q604_M93b *(required)* | q604_M93b. How is the item administered, or given, to the patient? *Question relevant when: selected( ${q104_M93_full_list} , '5')* | 1 Oral - solid (e.g. tablet)<br>2 Oral - liquid<br>3 Sublingual<br>4 Intravenous (IV)<br>5 Intramusculuar injection (IM)<br>6 Subcutaneous<br>7 Rectal<br>8 Inhaled (e.g. gas)<br>9 Inserted (e.g. vaginal pessaries)<br>10 Topical - cream<br>11 Topical - drops (e.g. eye drops)<br>12 Other<br>99 Don't know |
| q604_M93c *(required)* | q604_M93c. What is the item's smallest unit of measurement (mg, mcg, ml, g, etc)? *Question relevant when: selected( ${q104_M93_full_list} , '5')* | microgram: microgram (mcg)<br>miligram: miligram (mg)<br>miligram_dissolved_in_liquid: miligram (mg) dissolved in liquid (e.g. 2 mg per 3 militers)<br>gram: gram (g)<br>militer: militer (ml)<br>centiliter: centiliter (cl)<br>litre: litre (l)<br>ml_of _solution: ml of solution (e.g. ml of 10% solution)<br>litre_of_solution: litre of solution (e.g. 1 litre of 5% solution)<br>cubic_meter: cubic meter (m^3)<br>other: other<br>dont_know: don't know |

| Field | Question | Answer |
|---|---|---|
| q604_M93c.1 | q604_M93c.1 If other unit of measurement or route of administration, specify here:<br>*Leave blank if not applicable*<br>*Question relevant when: selected( ${q104_M93_full_list} , '5')* | |
| q604_M93d *(required)* | q604_M93d. In total, how many units are used to treat a patient during all of her care?<br>*Remember to add all units! E.g. 5 days x 200 mg twice per day = 2,000mg*<br>*Question relevant when: selected( ${q104_M93_full_list} , '5')*<br>*Response constrained to: .>0* | |
| q604_M94 | ***q604_M94. Saline, glucose, water, etc. - Other 4: "[q104_M94_full_list_other]"***<br>*Question relevant when: selected( ${q104_M94_full_list} , '5')* | |
| q604_M94a *(required)* | q604_M94a. What percent of patients require this item?<br>*Question relevant when: selected( ${q104_M94_full_list} , '5')*<br>*Response constrained to: .>0 and .<=100 or .=999* | |
| q604_M94b *(required)* | q604_M94b. How is the item administered, or given, to the patient?<br>*Question relevant when: selected( ${q104_M94_full_list} , '5')* | 1 Oral - solid (e.g. tablet)<br>2 Oral - liquid<br>3 Sublingual<br>4 Intravenous (IV)<br>5 Intramusculuar injection (IM)<br>6 Subcutaneous<br>7 Rectal<br>8 Inhaled (e.g. gas)<br>9 Inserted (e.g. vaginal pessaries)<br>10 Topical - cream<br>11 Topical - drops (e.g. eye drops)<br>12 Other<br>99 Don't know |
| q604_M94c *(required)* | q604_M94c. What is the item's smallest unit of measurement (mg, mcg, ml, g, etc)?<br>*Question relevant when: selected( ${q104_M94_full_list} , '5')* | microgram — microgram (mcg)<br>miligram — miligram (mg)<br>miligram_dissolved_in_liquid — miligram (mg) dissolved in liquid (e.g. 2 mg per 3 militers)<br>gram — gram (g)<br>militer — militer (ml)<br>centiliter — centiliter (cl)<br>litre — litre (l)<br>ml_of _solution — ml of solution (e.g. ml of 10% solution)<br>litre_of_solution — litre of solution (e.g. 1 litre of 5% solution)<br>cubic_meter — cubic meter (m^3)<br>other — other<br>dont_know — don't know |

| Field | Question | Answer |
|---|---|---|
| q604_M94c.1 | q604_M94c.1 If other unit of measurement or route of administration, specify here:<br>*Leave blank if not applicable*<br>*Question relevant when: selected( ${q104_M94_full_list} , '5')* | |
| q604_M94d *(required)* | q604_M94d. In total, how many units are used to treat a patient during all of her care?<br>*Remember to add all units! E.g. 5 days x 200 mg twice per day = 2,000mg*<br>*Question relevant when: selected( ${q104_M94_full_list} , '5')*<br>*Response constrained to: .>0* | |
| q604_M95 | ***q604_M95. Saline, glucose, water, etc. - Other 5: "[q104_M95_full_list_other]"***<br>*Question relevant when: selected( ${q104_M95_full_list} , '5')* | |
| q604_M95a *(required)* | q604_M95a. What percent of patients require this item?<br>*Question relevant when: selected( ${q104_M95_full_list} , '5')*<br>*Response constrained to: .>0 and .<=100 or .=999* | |
| q604_M95b *(required)* | q604_M95b. How is the item administered, or given, to the patient?<br>*Question relevant when: selected( ${q104_M95_full_list} , '5')* | 1 Oral - solid (e.g. tablet)<br>2 Oral - liquid<br>3 Sublingual<br>4 Intravenous (IV)<br>5 Intramusculuar injection (IM)<br>6 Subcutaneous<br>7 Rectal<br>8 Inhaled (e.g. gas)<br>9 Inserted (e.g. vaginal pessaries)<br>10 Topical - cream<br>11 Topical - drops (e.g. eye drops)<br>12 Other<br>99 Don't know |
| q604_M95c *(required)* | q604_M95c. What is the item's smallest unit of measurement (mg, mcg, ml, g, etc)?<br>*Question relevant when: selected( ${q104_M95_full_list} , '5')* | microgram — microgram (mcg)<br>miligram — miligram (mg)<br>miligram_dissolved_in_liquid — miligram (mg) dissolved in liquid (e.g. 2 mg per 3 militers)<br>gram — gram (g)<br>militer — militer (ml)<br>centiliter — centiliter (cl)<br>litre — litre (l)<br>ml_of _solution — ml of solution (e.g. ml of 10% solution)<br>litre_of_solution — litre of solution (e.g. 1 litre of 5% solution)<br>cubic_meter — cubic meter (m^3)<br>other — other<br>dont_know — don't know |

| Field | Question | Answer |
|---|---|---|
| q604_M95c.1 | q604_M95c.1 If other unit of measurement or route of administration, specify here:<br>*Leave blank if not applicable*<br>*Question relevant when: selected( ${q104_M95_full_list} , '5')* | |
| q604_M95d *(required)* | q604_M95d. In total, how many units are used to treat a patient during all of her care?<br>*Remember to add all units! E.g. 5 days x 200 mg twice per day = 2,000mg*<br>*Question relevant when: selected( ${q104_M95_full_list} , '5')*<br>*Response constrained to: .>0* | |
| <p><strong><em>D. Medications - Perforations (10)</em></strong></p><br>*Group relevant when: selected( ${section_six_skip_med} , '1')* | | |
| q604_med_notE9 | 604 CONTINUED For each of the following medications and other medical products that are used for perforations, can you tell me …? | |
| note_604_prost_utero | ***Prostaglandin, uterotonic, etc.*** | |
| q604_M96 | ***q604_M96. Misoprostol***<br>*Question relevant when: selected( ${q104_M96_full_list} , '5')* | |
| q604_M96a *(required)* | q604_M96a. What percent of patients require this item?<br>*Question relevant when: selected( ${q104_M96_full_list} , '5')*<br>*Response constrained to: .>0 and .<=100 or .=999* | |

| | | |
|---|---|---|
| q604_M96b *(required)* | q604_M96b. How is the item administered, or given, to the patient?<br>*Question relevant when: selected( ${q104_M96_full_list} , '5')* | 1 Oral - solid (e.g. tablet)<br>2 Oral - liquid<br>3 Sublingual<br>4 Intravenous (IV)<br>5 Intramusculuar injection (IM)<br>6 Subcutaneous<br>7 Rectal<br>8 Inhaled (e.g. gas)<br>9 Inserted (e.g. vaginal pessaries)<br>10 Topical - cream<br>11 Topical - drops (e.g. eye drops)<br>12 Other<br>99 Don't know |
| q604_M96c *(required)* | q604_M96c. What is the item's smallest unit of measurement (mg, mcg, ml, g, etc)?<br>*Question relevant when: selected( ${q104_M96_full_list} , '5')* | microgram — microgram (mcg)<br>miligram — miligram (mg)<br>miligram_dissolved_in_liquid — miligram (mg) dissolved in liquid (e.g. 2 mg per 3 militers)<br>gram — gram (g)<br>militer — militer (ml)<br>centiliter — centiliter (cl)<br>litre — litre (l)<br>ml_of _solution — ml of solution (e.g. ml of 10% solution)<br>litre_of_solution — litre of solution (e.g. 1 litre of 5% solution)<br>cubic_meter — cubic meter (m^3)<br>other — other<br>dont_know — don't know |

| Field | Question | Answer |
|---|---|---|
| q604_M96c.1 | q604_M96c.1 If other unit of measurement or route of administration, specify here:<br>*Leave blank if not applicable*<br>*Question relevant when: selected( ${q104_M96_full_list} , '5')* | |
| q604_M96d *(required)* | q604_M96d. In total, how many units are used to treat a patient during all of her care?<br>*Remember to add all units! E.g. 5 days x 200 mg twice per day = 2,000mg*<br>*Question relevant when: selected( ${q104_M96_full_list} , '5')*<br>*Response constrained to: .>0* | |
| q604_M97 | ***q604_M97. Ergometrine***<br>*Question relevant when: selected( ${q104_M97_full_list} , '5')* | |
| q604_M97a *(required)* | q604_M97a. What percent of patients require this item?<br>*Question relevant when: selected( ${q104_M97_full_list} , '5')*<br>*Response constrained to: .>0 and .<=100 or .=999* | |
| q604_M97b *(required)* | q604_M97b. How is the item administered, or given, to the patient?<br>*Question relevant when: selected( ${q104_M97_full_list} , '5')* | 1 Oral - solid (e.g. tablet)<br>2 Oral - liquid<br>3 Sublingual<br>4 Intravenous (IV)<br>5 Intramusculuar injection (IM)<br>6 Subcutaneous<br>7 Rectal<br>8 Inhaled (e.g. gas)<br>9 Inserted (e.g. vaginal pessaries)<br>10 Topical - cream<br>11 Topical - drops (e.g. eye drops)<br>12 Other<br>99 Don't know |
| q604_M97c *(required)* | q604_M97c. What is the item's smallest unit of measurement (mg, mcg, ml, g, etc)?<br>*Question relevant when: selected( ${q104_M97_full_list} , '5')* | microgram — microgram (mcg)<br>miligram — miligram (mg)<br>miligram_dissolved_in_liquid — miligram (mg) dissolved in liquid (e.g. 2 mg per 3 militers)<br>gram — gram (g)<br>militer — militer (ml)<br>centiliter — centiliter (cl)<br>litre — litre (l)<br>ml_of _solution — ml of solution (e.g. ml of 10% solution)<br>litre_of_solution — litre of solution (e.g. 1 litre of 5% solution)<br>cubic_meter — cubic meter (m^3)<br>other — other<br>dont_know — don't know |

| Field | Question | Answer |
|---|---|---|
| q604_M97c.1 | q604_M97c.1 If other unit of measurement or route of administration, specify here:<br>*Leave blank if not applicable*<br>*Question relevant when: selected( ${q104_M97_full_list} , '5')* | |
| q604_M97d *(required)* | q604_M97d. In total, how many units are used to treat a patient during all of her care?<br>*Remember to add all units! E.g. 5 days x 200 mg twice per day = 2,000mg*<br>*Question relevant when: selected( ${q104_M97_full_list} , '5')*<br>*Response constrained to: .>0* | |
| q604_M98 | ***q604_M98. Methylergonovine Maleate (Methergine)***<br>*Question relevant when: selected( ${q104_M98_full_list} , '5')* | |
| q604_M98a *(required)* | q604_M98a. What percent of patients require this item?<br>*Question relevant when: selected( ${q104_M98_full_list} , '5')*<br>*Response constrained to: .>0 and .<=100 or .=999* | |
| q604_M98b *(required)* | q604_M98b. How is the item administered, or given, to the patient?<br>*Question relevant when: selected( ${q104_M98_full_list} , '5')* | 1 Oral - solid (e.g. tablet)<br>2 Oral - liquid<br>3 Sublingual<br>4 Intravenous (IV)<br>5 Intramusculuar injection (IM)<br>6 Subcutaneous<br>7 Rectal<br>8 Inhaled (e.g. gas)<br>9 Inserted (e.g. vaginal pessaries)<br>10 Topical - cream<br>11 Topical - drops (e.g. eye drops)<br>12 Other<br>99 Don't know |
| q604_M98c *(required)* | q604_M98c. What is the item's smallest unit of measurement (mg, mcg, ml, g, etc)?<br>*Question relevant when: selected( ${q104_M98_full_list} , '5')* | microgram — microgram (mcg)<br>miligram — miligram (mg)<br>miligram_dissolved_in_liquid — miligram (mg) dissolved in liquid (e.g. 2 mg per 3 militers)<br>gram — gram (g)<br>militer — militer (ml)<br>centiliter — centiliter (cl)<br>litre — litre (l)<br>ml_of _solution — ml of solution (e.g. ml of 10% solution)<br>litre_of_solution — litre of solution (e.g. 1 litre of 5% solution)<br>cubic_meter — cubic meter (m^3)<br>other — other<br>dont_know — don't know |

| Field | Question | Answer |
|---|---|---|
| q604_M98c.1 | q604_M98c.1 If other unit of measurement or route of administration, specify here:<br>*Leave blank if not applicable*<br>*Question relevant when: selected( ${q104_M98_full_list} , '5')* | |
| q604_M98d *(required)* | q604_M98d. In total, how many units are used to treat a patient during all of her care?<br>*Remember to add all units! E.g. 5 days x 200 mg twice per day = 2,000mg*<br>*Question relevant when: selected( ${q104_M98_full_list} , '5')*<br>*Response constrained to: .>0* | |
| q604_M99 | ***q604_M99. Mifepristone***<br>*Question relevant when: selected( ${q104_M99_full_list} , '5')* | |
| q604_M99a *(required)* | q604_M99a. What percent of patients require this item?<br>*Question relevant when: selected( ${q104_M99_full_list} , '5')*<br>*Response constrained to: .>0 and .<=100 or .=999* | |
| q604_M99b *(required)* | q604_M99b. How is the item administered, or given, to the patient?<br>*Question relevant when: selected( ${q104_M99_full_list} , '5')* | 1 Oral - solid (e.g. tablet)<br>2 Oral - liquid<br>3 Sublingual<br>4 Intravenous (IV)<br>5 Intramusculuar injection (IM)<br>6 Subcutaneous<br>7 Rectal<br>8 Inhaled (e.g. gas)<br>9 Inserted (e.g. vaginal pessaries)<br>10 Topical - cream<br>11 Topical - drops (e.g. eye drops)<br>12 Other<br>99 Don't know |
| q604_M99c *(required)* | q604_M99c. What is the item's smallest unit of measurement (mg, mcg, ml, g, etc)?<br>*Question relevant when: selected( ${q104_M99_full_list} , '5')* | microgram — microgram (mcg)<br>miligram — miligram (mg)<br>miligram_dissolved_in_liquid — miligram (mg) dissolved in liquid (e.g. 2 mg per 3 militers)<br>gram — gram (g)<br>militer — militer (ml)<br>centiliter — centiliter (cl)<br>litre — litre (l)<br>ml_of _solution — ml of solution (e.g. ml of 10% solution)<br>litre_of_solution — litre of solution (e.g. 1 litre of 5% solution)<br>cubic_meter — cubic meter (m^3)<br>other — other<br>dont_know — don't know |

| Field | Question | Answer |
|---|---|---|
| q604_M99c.1 | q604_M99c.1 If other unit of measurement or route of administration, specify here:<br>*Leave blank if not applicable*<br>*Question relevant when: selected( ${q104_M99_full_list} , '5')* | |
| q604_M99d *(required)* | q604_M99d. In total, how many units are used to treat a patient during all of her care?<br>*Remember to add all units! E.g. 5 days x 200 mg twice per day = 2,000mg*<br>*Question relevant when: selected( ${q104_M99_full_list} , '5')*<br>*Response constrained to: .>0* | |
| q604_M100 | ***q604_M100. Oxytocin***<br>*Question relevant when: selected( ${q104_M100_full_list} , '5')* | |
| q604_M100a *(required)* | q604_M100a. What percent of patients require this item?<br>*Question relevant when: selected( ${q104_M100_full_list} , '5')*<br>*Response constrained to: .>0 and .<=100 or .=999* | |
| q604_M100b *(required)* | q604_M100b. How is the item administered, or given, to the patient?<br>*Question relevant when: selected( ${q104_M100_full_list} , '5')* | 1 Oral - solid (e.g. tablet)<br>2 Oral - liquid<br>3 Sublingual<br>4 Intravenous (IV)<br>5 Intramusculuar injection (IM)<br>6 Subcutaneous<br>7 Rectal<br>8 Inhaled (e.g. gas)<br>9 Inserted (e.g. vaginal pessaries)<br>10 Topical - cream<br>11 Topical - drops (e.g. eye drops)<br>12 Other<br>99 Don't know |
| q604_M100c *(required)* | q604_M100c. What is the item's smallest unit of measurement (mg, mcg, ml, g, etc)?<br>*Question relevant when: selected( ${q104_M100_full_list} , '5')* | microgram — microgram (mcg)<br>miligram — miligram (mg)<br>miligram_dissolved_in_liquid — miligram (mg) dissolved in liquid (e.g. 2 mg per 3 militers)<br>gram — gram (g)<br>militer — militer (ml)<br>centiliter — centiliter (cl)<br>litre — litre (l)<br>ml_of _solution — ml of solution (e.g. ml of 10% solution)<br>litre_of_solution — litre of solution (e.g. 1 litre of 5% solution)<br>cubic_meter — cubic meter (m^3)<br>other — other<br>dont_know — don't know |

| Field | Question | Answer |
|---|---|---|
| q604_M100c.1 | q604_M100c.1 If other unit of measurement or route of administration, specify here:<br>*Leave blank if not applicable*<br>*Question relevant when: selected( ${q104_M100_full_list} , '5')* | |
| q604_M100d *(required)* | q604_M100d. In total, how many units are used to treat a patient during all of her care?<br>*Remember to add all units! E.g. 5 days x 200 mg twice per day = 2,000mg*<br>*Question relevant when: selected( ${q104_M100_full_list} , '5')*<br>*Response constrained to: .>0* | |
| q604_M101 | ***q604_M101. Prostaglandin, uterotonic, etc. - Other 1: "[q104_M101_full_list_other]"***<br>*Question relevant when: selected( ${q104_M101_full_list} , '5')* | |
| q604_M101a *(required)* | q604_M101a. What percent of patients require this item?<br>*Question relevant when: selected( ${q104_M101_full_list} , '5')*<br>*Response constrained to: .>0 and .<=100 or .=999* | |
| q604_M101b *(required)* | q604_M101b. How is the item administered, or given, to the patient?<br>*Question relevant when: selected( ${q104_M101_full_list} , '5')* | 1 Oral - solid (e.g. tablet)<br>2 Oral - liquid<br>3 Sublingual<br>4 Intravenous (IV)<br>5 Intramusculuar injection (IM)<br>6 Subcutaneous<br>7 Rectal<br>8 Inhaled (e.g. gas)<br>9 Inserted (e.g. vaginal pessaries)<br>10 Topical - cream<br>11 Topical - drops (e.g. eye drops)<br>12 Other<br>99 Don't know |
| q604_M101c *(required)* | q604_M101c. What is the item's smallest unit of measurement (mg, mcg, ml, g, etc)?<br>*Question relevant when: selected( ${q104_M101_full_list} , '5')* | microgram — microgram (mcg)<br>miligram — miligram (mg)<br>miligram_dissolved_in_liquid — miligram (mg) dissolved in liquid (e.g. 2 mg per 3 militers)<br>gram — gram (g)<br>militer — militer (ml)<br>centiliter — centiliter (cl)<br>litre — litre (l)<br>ml_of _solution — ml of solution (e.g. ml of 10% solution)<br>litre_of_solution — litre of solution (e.g. 1 litre of 5% solution)<br>cubic_meter — cubic meter (m^3)<br>other — other<br>dont_know — don't know |

| Field | Question | Answer |
|---|---|---|
| q604_M101c.1 | q604_M101c.1 If other unit of measurement or route of administration, specify here:<br>*Leave blank if not applicable*<br>*Question relevant when: selected( ${q104_M101_full_list} , '5')* | |
| q604_M101d *(required)* | q604_M101d. In total, how many units are used to treat a patient during all of her care?<br>*Remember to add all units! E.g. 5 days x 200 mg twice per day = 2,000mg*<br>*Question relevant when: selected( ${q104_M101_full_list} , '5')*<br>*Response constrained to: .>0* | |
| q604_M102 | ***q604_M102. Prostaglandin, uterotonic, etc. - Other 2: "[q104_M102_full_list_other]"***<br>*Question relevant when: selected( ${q104_M102_full_list} , '5')* | |
| q604_M102a *(required)* | q604_M102a. What percent of patients require this item?<br>*Question relevant when: selected( ${q104_M102_full_list} , '5')*<br>*Response constrained to: .>0 and .<=100 or .=999* | |
| q604_M102b *(required)* | q604_M102b. How is the item administered, or given, to the patient?<br>*Question relevant when: selected( ${q104_M102_full_list} , '5')* | 1 Oral - solid (e.g. tablet)<br>2 Oral - liquid<br>3 Sublingual<br>4 Intravenous (IV)<br>5 Intramusculuar injection (IM)<br>6 Subcutaneous<br>7 Rectal<br>8 Inhaled (e.g. gas)<br>9 Inserted (e.g. vaginal pessaries)<br>10 Topical - cream<br>11 Topical - drops (e.g. eye drops)<br>12 Other<br>99 Don't know |
| q604_M102c *(required)* | q604_M102c. What is the item's smallest unit of measurement (mg, mcg, ml, g, etc)?<br>*Question relevant when: selected( ${q104_M102_full_list} , '5')* | microgram — microgram (mcg)<br>miligram — miligram (mg)<br>miligram_dissolved_in_liquid — miligram (mg) dissolved in liquid (e.g. 2 mg per 3 militers)<br>gram — gram (g)<br>militer — militer (ml)<br>centiliter — centiliter (cl)<br>litre — litre (l)<br>ml_of _solution — ml of solution (e.g. ml of 10% solution)<br>litre_of_solution — litre of solution (e.g. 1 litre of 5% solution)<br>cubic_meter — cubic meter (m^3)<br>other — other<br>dont_know — don't know |

| Field | Question | Answer |
|---|---|---|
| q604_M102c.1 | q604_M102c.1 If other unit of measurement or route of administration, specify here:<br>*Leave blank if not applicable*<br>*Question relevant when: selected( ${q104_M102_full_list} , '5')* | |
| q604_M102d *(required)* | q604_M102d. In total, how many units are used to treat a patient during all of her care?<br>*Remember to add all units! E.g. 5 days x 200 mg twice per day = 2,000mg*<br>*Question relevant when: selected( ${q104_M102_full_list} , '5')*<br>*Response constrained to: .>0* | |
| q604_M103 | ***q604_M103. Prostaglandin, uterotonic, etc. - Other 3: "[q104_M103_full_list_other]"***<br>*Question relevant when: selected( ${q104_M104_full_list} , '5')* | |
| q604_M103a *(required)* | q604_M103a. What percent of patients require this item?<br>*Question relevant when: selected( ${q104_M104_full_list} , '5')*<br>*Response constrained to: .>0 and .<=100 or .=999* | |

| Field | Question | Answer |
|---|---|---|
| q604_M103b *(required)* | q604_M103b. How is the item administered, or given, to the patient?<br>*Question relevant when: selected( ${q104_M104_full_list} , '5')* | 1 Oral - solid (e.g. tablet)<br>2 Oral - liquid<br>3 Sublingual<br>4 Intravenous (IV)<br>5 Intramusculuar injection (IM)<br>6 Subcutaneous<br>7 Rectal<br>8 Inhaled (e.g. gas)<br>9 Inserted (e.g. vaginal pessaries)<br>10 Topical - cream<br>11 Topical - drops (e.g. eye drops)<br>12 Other<br>99 Don't know |
| q604_M103c *(required)* | q604_M103c. What is the item's smallest unit of measurement (mg, mcg, ml, g, etc)?<br>*Question relevant when: selected( ${q104_M104_full_list} , '5')* | microgram — microgram (mcg)<br>miligram — miligram (mg)<br>miligram_dissolved_in_liquid — miligram (mg) dissolved in liquid (e.g. 2 mg per 3 militers)<br>gram — gram (g)<br>militer — militer (ml)<br>centiliter — centiliter (cl)<br>litre — litre (l)<br>ml_of _solution — ml of solution (e.g. ml of 10% solution)<br>litre_of_solution — litre of solution (e.g. 1 litre of 5% solution)<br>cubic_meter — cubic meter (m^3)<br>other — other<br>dont_know — don't know |

| Field | Question | Answer |
|---|---|---|
| q604_M103c.1 | q604_M103c.1 If other unit of measurement or route of administration, specify here:<br>*Leave blank if not applicable*<br>*Question relevant when: selected( ${q104_M104_full_list} , '5')* | |
| q604_M103d *(required)* | q604_M103d. In total, how many units are used to treat a patient during all of her care?<br>*Remember to add all units! E.g. 5 days x 200 mg twice per day = 2,000mg*<br>*Question relevant when: selected( ${q104_M104_full_list} , '5')*<br>*Response constrained to: .>0* | |
| q604_M104 | ***q604_M104. Prostaglandin, uterotonic, etc. - Other 4: "[q104_M104_full_list_other]"***<br>*Question relevant when: selected( ${q104_M104_full_list} , '5')* | |
| q604_M104a *(required)* | q604_M104a. What percent of patients require this item?<br>*Question relevant when: selected( ${q104_M104_full_list} , '5')*<br>*Response constrained to: .>0 and .<=100 or .=999* | |
| q604_M104b *(required)* | q604_M104b. How is the item administered, or given, to the patient?<br>*Question relevant when: selected( ${q104_M104_full_list} , '5')* | 1 Oral - solid (e.g. tablet)<br>2 Oral - liquid<br>3 Sublingual<br>4 Intravenous (IV)<br>5 Intramusculuar injection (IM)<br>6 Subcutaneous<br>7 Rectal<br>8 Inhaled (e.g. gas)<br>9 Inserted (e.g. vaginal pessaries)<br>10 Topical - cream<br>11 Topical - drops (e.g. eye drops)<br>12 Other<br>99 Don't know |
| q604_M104c *(required)* | q604_M104c. What is the item's smallest unit of measurement (mg, mcg, ml, g, etc)?<br>*Question relevant when: selected( ${q104_M104_full_list} , '5')* | microgram — microgram (mcg)<br>miligram — miligram (mg)<br>miligram_dissolved_in_liquid — miligram (mg) dissolved in liquid (e.g. 2 mg per 3 militers)<br>gram — gram (g)<br>militer — militer (ml)<br>centiliter — centiliter (cl)<br>litre — litre (l)<br>ml_of _solution — ml of solution (e.g. ml of 10% solution)<br>litre_of_solution — litre of solution (e.g. 1 litre of 5% solution)<br>cubic_meter — cubic meter (m^3)<br>other — other<br>dont_know — don't know |

| Field | Question | Answer |
|---|---|---|
| q604_M104c.1 | q604_M104c.1 If other unit of measurement or route of administration, specify here:<br>*Leave blank if not applicable*<br>*Question relevant when: selected( ${q104_M104_full_list} , '5')* | |
| q604_M104d *(required)* | q604_M104d. In total, how many units are used to treat a patient during all of her care?<br>*Remember to add all units! E.g. 5 days x 200 mg twice per day = 2,000mg*<br>*Question relevant when: selected( ${q104_M104_full_list} , '5')*<br>*Response constrained to: .>0* | |
| q604_M105 | ***q604_M105. Prostaglandin, uterotonic, etc. - Other 5: "[q104_M105_full_list_other]"***<br>*Question relevant when: selected( ${q104_M105_full_list} , '5')* | |
| q604_M105a *(required)* | q604_M105a. What percent of patients require this item?<br>*Question relevant when: selected( ${q104_M105_full_list} , '5')*<br>*Response constrained to: .>0 and .<=100 or .=999* | |
| q604_M105b *(required)* | q604_M105b. How is the item administered, or given, to the patient?<br>*Question relevant when: selected( ${q104_M105_full_list} , '5')* | 1 Oral - solid (e.g. tablet)<br>2 Oral - liquid<br>3 Sublingual<br>4 Intravenous (IV)<br>5 Intramusculuar injection (IM)<br>6 Subcutaneous<br>7 Rectal<br>8 Inhaled (e.g. gas)<br>9 Inserted (e.g. vaginal pessaries)<br>10 Topical - cream<br>11 Topical - drops (e.g. eye drops)<br>12 Other<br>99 Don't know |
| q604_M105c *(required)* | q604_M105c. What is the item's smallest unit of measurement (mg, mcg, ml, g, etc)?<br>*Question relevant when: selected( ${q104_M105_full_list} , '5')* | microgram — microgram (mcg)<br>miligram — miligram (mg)<br>miligram_dissolved_in_liquid — miligram (mg) dissolved in liquid (e.g. 2 mg per 3 militers)<br>gram — gram (g)<br>militer — militer (ml)<br>centiliter — centiliter (cl)<br>litre — litre (l)<br>ml_of _solution — ml of solution (e.g. ml of 10% solution)<br>litre_of_solution — litre of solution (e.g. 1 litre of 5% solution)<br>cubic_meter — cubic meter (m^3)<br>other — other<br>dont_know — don't know |

| Field | Question | Answer |
|---|---|---|
| q604_M105c.1 | q604_M105c.1 If other unit of measurement or route of administration, specify here:<br>*Leave blank if not applicable*<br>*Question relevant when: selected( ${q104_M105_full_list} , '5')* | |
| q604_M105d *(required)* | q604_M105d. In total, how many units are used to treat a patient during all of her care?<br>*Remember to add all units! E.g. 5 days x 200 mg twice per day = 2,000mg*<br>*Question relevant when: selected( ${q104_M105_full_list} , '5')*<br>*Response constrained to: .>0* | |
| <p><strong><em>D. Medications - Perforations (11)</em></strong></p><br>*Group relevant when: selected( ${section_six_skip_med} , '1')* | | |
| q604_med_notE10 | 604 CONTINUED For each of the following medications and other medical products that are used for perforations, can you tell me …? | |
| note_604_vitamin | ***Vitamin, immunological, etc.*** | |
| q604_M106 | ***q604_M106. Ferrous sulphate (tablets)***<br>*Question relevant when: selected( ${q104_M106_full_list} , '5')* | |
| q604_M106a *(required)* | q604_M106a. What percent of patients require this item?<br>*Question relevant when: selected( ${q104_M106_full_list} , '5')*<br>*Response constrained to: .>0 and .<=100 or .=999* | |

| Field | Question | Answer |
|---|---|---|
| q604_M106b *(required)* | q604_M106b. How is the item administered, or given, to the patient?<br>*Question relevant when: selected( ${q104_M106_full_list} , '5')* | 1 Oral - solid (e.g. tablet)<br>2 Oral - liquid<br>3 Sublingual<br>4 Intravenous (IV)<br>5 Intramusculuar injection (IM)<br>6 Subcutaneous<br>7 Rectal<br>8 Inhaled (e.g. gas)<br>9 Inserted (e.g. vaginal pessaries)<br>10 Topical - cream<br>11 Topical - drops (e.g. eye drops)<br>12 Other<br>99 Don't know |
| q604_M106c *(required)* | q604_M106c. What is the item's smallest unit of measurement (mg, mcg, ml, g, etc)?<br>*Question relevant when: selected( ${q104_M106_full_list} , '5')* | microgram — microgram (mcg)<br>miligram — miligram (mg)<br>miligram_dissolved_in_liquid — miligram (mg) dissolved in liquid (e.g. 2 mg per 3 militers)<br>gram — gram (g)<br>militer — militer (ml)<br>centiliter — centiliter (cl)<br>litre — litre (l)<br>ml_of _solution — ml of solution (e.g. ml of 10% solution)<br>litre_of_solution — litre of solution (e.g. 1 litre of 5% solution)<br>cubic_meter — cubic meter (m^3)<br>other — other<br>dont_know — don't know |

| Field | Question | Answer |
|---|---|---|
| q604_M106c.1 | q604_M106c.1 If other unit of measurement or route of administration, specify here:<br>*Leave blank if not applicable*<br>*Question relevant when: selected( ${q104_M106_full_list} , '5')* | |
| q604_M106d *(required)* | q604_M106d. In total, how many units are used to treat a patient during all of her care?<br>*Remember to add all units! E.g. 5 days x 200 mg twice per day = 2,000mg*<br>*Question relevant when: selected( ${q104_M106_full_list} , '5')*<br>*Response constrained to: .>0* | |
| q604_M107 | ***q604_M107. Ferrous sulphate + folic acid (Ferrotone capsules)***<br>*Question relevant when: selected( ${q104_M107_full_list} , '5')* | |
| q604_M107a *(required)* | q604_M107a. What percent of patients require this item?<br>*Question relevant when: selected( ${q104_M107_full_list} , '5')*<br>*Response constrained to: .>0 and .<=100 or .=999* | |

| Field | Question | Answer |
|---|---|---|
| q604_M107b *(required)* | q604_M107b. How is the item administered, or given, to the patient?<br>*Question relevant when: selected( ${q104_M107_full_list} , '5')* | 1 Oral - solid (e.g. tablet)<br>2 Oral - liquid<br>3 Sublingual<br>4 Intravenous (IV)<br>5 Intramusculuar injection (IM)<br>6 Subcutaneous<br>7 Rectal<br>8 Inhaled (e.g. gas)<br>9 Inserted (e.g. vaginal pessaries)<br>10 Topical - cream<br>11 Topical - drops (e.g. eye drops)<br>12 Other<br>99 Don't know |
| q604_M107c *(required)* | q604_M107c. What is the item's smallest unit of measurement (mg, mcg, ml, g, etc)?<br>*Question relevant when: selected( ${q104_M107_full_list} , '5')* | microgram — microgram (mcg)<br>miligram — miligram (mg)<br>miligram_dissolved_in_liquid — miligram (mg) dissolved in liquid (e.g. 2 mg per 3 militers)<br>gram — gram (g)<br>militer — militer (ml)<br>centiliter — centiliter (cl)<br>litre — litre (l)<br>ml_of _solution — ml of solution (e.g. ml of 10% solution)<br>litre_of_solution — litre of solution (e.g. 1 litre of 5% solution)<br>cubic_meter — cubic meter (m^3)<br>other — other<br>dont_know — don't know |

| Field | Question | Answer |
|---|---|---|
| q604_M107c.1 | q604_M107c.1 If other unit of measurement or route of administration, specify here:<br>*Leave blank if not applicable*<br>*Question relevant when: selected( ${q104_M107_full_list} , '5')* | |
| q604_M107d *(required)* | q604_M107d. In total, how many units are used to treat a patient during all of her care?<br>*Remember to add all units! E.g. 5 days x 200 mg twice per day = 2,000mg*<br>*Question relevant when: selected( ${q104_M107_full_list} , '5')*<br>*Response constrained to: .>0* | |
| q604_M108 | ***q604_M108. Folic acid (Tablets)***<br>*Question relevant when: selected( ${q104_M108_full_list} , '5')* | |
| q604_M108a *(required)* | q604_M108a. What percent of patients require this item?<br>*Question relevant when: selected( ${q104_M108_full_list} , '5')*<br>*Response constrained to: .>0 and .<=100 or .=999* | |

| Field | Question | Answer |
|---|---|---|
| q604_M108b *(required)* | q604_M108b. How is the item administered, or given, to the patient?<br>*Question relevant when: selected( ${q104_M108_full_list} , '5')* | 1 Oral - solid (e.g. tablet)<br>2 Oral - liquid<br>3 Sublingual<br>4 Intravenous (IV)<br>5 Intramusculuar injection (IM)<br>6 Subcutaneous<br>7 Rectal<br>8 Inhaled (e.g. gas)<br>9 Inserted (e.g. vaginal pessaries)<br>10 Topical - cream<br>11 Topical - drops (e.g. eye drops)<br>12 Other<br>99 Don't know |
| q604_M108c *(required)* | q604_M108c. What is the item's smallest unit of measurement (mg, mcg, ml, g, etc)?<br>*Question relevant when: selected( ${q104_M108_full_list} , '5')* | microgram — microgram (mcg)<br>miligram — miligram (mg)<br>miligram_dissolved_in_liquid — miligram (mg) dissolved in liquid (e.g. 2 mg per 3 militers)<br>gram — gram (g)<br>militer — militer (ml)<br>centiliter — centiliter (cl)<br>litre — litre (l)<br>ml_of _solution — ml of solution (e.g. ml of 10% solution)<br>litre_of_solution — litre of solution (e.g. 1 litre of 5% solution)<br>cubic_meter — cubic meter (m^3)<br>other — other<br>dont_know — don't know |

| Field | Question | Answer |
|---|---|---|
| q604_M108c.1 | q604_M108c.1 If other unit of measurement or route of administration, specify here:<br>*Leave blank if not applicable*<br>*Question relevant when: selected( ${q104_M108_full_list} , '5')* | |
| q604_M108d *(required)* | q604_M108d. In total, how many units are used to treat a patient during all of her care?<br>*Remember to add all units! E.g. 5 days x 200 mg twice per day = 2,000mg*<br>*Question relevant when: selected( ${q104_M108_full_list} , '5')*<br>*Response constrained to: .>0* | |
| q604_M109 | ***q604_M109. Serum anti-tetanus***<br>*Question relevant when: selected( ${q104_M109_full_list} , '5')* | |
| q604_M109a *(required)* | q604_M109a. What percent of patients require this item?<br>*Question relevant when: selected( ${q104_M109_full_list} , '5')*<br>*Response constrained to: .>0 and .<=100 or .=999* | |
| q604_M109b *(required)* | q604_M109b. How is the item administered, or given, to the patient?<br>*Question relevant when: selected( ${q104_M109_full_list} , '5')* | 1 Oral - solid (e.g. tablet)<br>2 Oral - liquid<br>3 Sublingual<br>4 Intravenous (IV)<br>5 Intramusculuar injection (IM)<br>6 Subcutaneous<br>7 Rectal<br>8 Inhaled (e.g. gas)<br>9 Inserted (e.g. vaginal pessaries)<br>10 Topical - cream<br>11 Topical - drops (e.g. eye drops)<br>12 Other<br>99 Don't know |
| q604_M109c *(required)* | q604_M109c. What is the item's smallest unit of measurement (mg, mcg, ml, g, etc)?<br>*Question relevant when: selected( ${q104_M109_full_list} , '5')* | microgram — microgram (mcg)<br>miligram — miligram (mg)<br>miligram_dissolved_in_liquid — miligram (mg) dissolved in liquid (e.g. 2 mg per 3 militers)<br>gram — gram (g)<br>militer — militer (ml)<br>centiliter — centiliter (cl)<br>litre — litre (l)<br>ml_of _solution — ml of solution (e.g. ml of 10% solution)<br>litre_of_solution — litre of solution (e.g. 1 litre of 5% solution)<br>cubic_meter — cubic meter (m^3)<br>other — other<br>dont_know — don't know |

| Field | Question | Answer |
|---|---|---|
| q604_M109c.1 | q604_M109c.1 If other unit of measurement or route of administration, specify here:<br>*Leave blank if not applicable*<br>*Question relevant when: selected( ${q104_M109_full_list} , '5')* | |
| q604_M109d *(required)* | q604_M109d. In total, how many units are used to treat a patient during all of her care?<br>*Remember to add all units! E.g. 5 days x 200 mg twice per day = 2,000mg*<br>*Question relevant when: selected( ${q104_M109_full_list} , '5')*<br>*Response constrained to: .>0* | |
| q604_M110 | ***q604_M110. Anti D (Rh)-Immunoglobulin***<br>*Question relevant when: selected( ${q104_M110_full_list} , '5')* | |
| q604_M110a *(required)* | q604_M110a. What percent of patients require this item?<br>*Question relevant when: selected( ${q104_M110_full_list} , '5')*<br>*Response constrained to: .>0 and .<=100 or .=999* | |
| q604_M110b *(required)* | q604_M110b. How is the item administered, or given, to the patient?<br>*Question relevant when: selected( ${q104_M110_full_list} , '5')* | 1 Oral - solid (e.g. tablet)<br>2 Oral - liquid<br>3 Sublingual<br>4 Intravenous (IV)<br>5 Intramusculuar injection (IM)<br>6 Subcutaneous<br>7 Rectal<br>8 Inhaled (e.g. gas)<br>9 Inserted (e.g. vaginal pessaries)<br>10 Topical - cream<br>11 Topical - drops (e.g. eye drops)<br>12 Other<br>99 Don't know |
| q604_M110c *(required)* | q604_M110c. What is the item's smallest unit of measurement (mg, mcg, ml, g, etc)?<br>*Question relevant when: selected( ${q104_M110_full_list} , '5')* | microgram — microgram (mcg)<br>miligram — miligram (mg)<br>miligram_dissolved_in_liquid — miligram (mg) dissolved in liquid (e.g. 2 mg per 3 militers)<br>gram — gram (g)<br>militer — militer (ml)<br>centiliter — centiliter (cl)<br>litre — litre (l)<br>ml_of _solution — ml of solution (e.g. ml of 10% solution)<br>litre_of_solution — litre of solution (e.g. 1 litre of 5% solution)<br>cubic_meter — cubic meter (m^3)<br>other — other<br>dont_know — don't know |

| Field | Question | Answer |
|---|---|---|
| q604_M110c.1 | q604_M110c.1 If other unit of measurement or route of administration, specify here:<br>*Leave blank if not applicable*<br>*Question relevant when: selected( ${q104_M110_full_list} , '5')* | |
| q604_M110d *(required)* | q604_M110d. In total, how many units are used to treat a patient during all of her care?<br>*Remember to add all units! E.g. 5 days x 200 mg twice per day = 2,000mg*<br>*Question relevant when: selected( ${q104_M110_full_list} , '5')*<br>*Response constrained to: .>0* | |
| q604_M111 | ***q604_M111. Vitamin, immunological, etc. - Other 1: "[q104_M111_full_list_other]"***<br>*Question relevant when: selected( ${q104_M111_full_list} , '5')* | |
| q604_M111a *(required)* | q604_M111a. What percent of patients require this item?<br>*Question relevant when: selected( ${q104_M111_full_list} , '5')*<br>*Response constrained to: .>0 and .<=100 or .=999* | |
| q604_M111b *(required)* | q604_M111b. How is the item administered, or given, to the patient?<br>*Question relevant when: selected( ${q104_M111_full_list} , '5')* | 1 Oral - solid (e.g. tablet)<br>2 Oral - liquid<br>3 Sublingual<br>4 Intravenous (IV)<br>5 Intramusculuar injection (IM)<br>6 Subcutaneous<br>7 Rectal<br>8 Inhaled (e.g. gas)<br>9 Inserted (e.g. vaginal pessaries)<br>10 Topical - cream<br>11 Topical - drops (e.g. eye drops)<br>12 Other<br>99 Don't know |
| q604_M111c *(required)* | q604_M111c. What is the item's smallest unit of measurement (mg, mcg, ml, g, etc)?<br>*Question relevant when: selected( ${q104_M111_full_list} , '5')* | microgram — microgram (mcg)<br>miligram — miligram (mg)<br>miligram_dissolved_in_liquid — miligram (mg) dissolved in liquid (e.g. 2 mg per 3 militers)<br>gram — gram (g)<br>militer — militer (ml)<br>centiliter — centiliter (cl)<br>litre — litre (l)<br>ml_of _solution — ml of solution (e.g. ml of 10% solution)<br>litre_of_solution — litre of solution (e.g. 1 litre of 5% solution)<br>cubic_meter — cubic meter (m^3)<br>other — other<br>dont_know — don't know |

| Field | Question | Answer |
|---|---|---|
| q604_M111c.1 | q604_M111c.1 If other unit of measurement or route of administration, specify here:<br>*Leave blank if not applicable*<br>*Question relevant when: selected( ${q104_M111_full_list} , '5')* | |
| q604_M111d *(required)* | q604_M111d. In total, how many units are used to treat a patient during all of her care?<br>*Remember to add all units! E.g. 5 days x 200 mg twice per day = 2,000mg*<br>*Question relevant when: selected( ${q104_M111_full_list} , '5')*<br>*Response constrained to: .>0* | |
| q604_M112 | ***q604_M112. Vitamin, immunological, etc. - Other 2: "[q104_M112_full_list_other]"***<br>*Question relevant when: selected( ${q104_M112_full_list} , '5')* | |
| q604_M112a *(required)* | q604_M112a. What percent of patients require this item?<br>*Question relevant when: selected( ${q104_M112_full_list} , '5')*<br>*Response constrained to: .>0 and .<=100 or .=999* | |
| q604_M112b *(required)* | q604_M112b. How is the item administered, or given, to the patient?<br>*Question relevant when: selected( ${q104_M112_full_list} , '5')* | 1 Oral - solid (e.g. tablet)<br>2 Oral - liquid<br>3 Sublingual<br>4 Intravenous (IV)<br>5 Intramusculuar injection (IM)<br>6 Subcutaneous<br>7 Rectal<br>8 Inhaled (e.g. gas)<br>9 Inserted (e.g. vaginal pessaries)<br>10 Topical - cream<br>11 Topical - drops (e.g. eye drops)<br>12 Other<br>99 Don't know |
| q604_M112c *(required)* | q604_M112c. What is the item's smallest unit of measurement (mg, mcg, ml, g, etc)?<br>*Question relevant when: selected( ${q104_M112_full_list} , '5')* | microgram — microgram (mcg)<br>miligram — miligram (mg)<br>miligram_dissolved_in_liquid — miligram (mg) dissolved in liquid (e.g. 2 mg per 3 militers)<br>gram — gram (g)<br>militer — militer (ml)<br>centiliter — centiliter (cl)<br>litre — litre (l)<br>ml_of _solution — ml of solution (e.g. ml of 10% solution)<br>litre_of_solution — litre of solution (e.g. 1 litre of 5% solution)<br>cubic_meter — cubic meter (m^3)<br>other — other<br>dont_know — don't know |

| Field | Question | Answer |
|---|---|---|
| q604_M112c.1 | q604_M112c.1 If other unit of measurement or route of administration, specify here:<br>*Leave blank if not applicable*<br>*Question relevant when: selected( ${q104_M112_full_list} , '5')* | |
| q604_M112d *(required)* | q604_M112d. In total, how many units are used to treat a patient during all of her care?<br>*Remember to add all units! E.g. 5 days x 200 mg twice per day = 2,000mg*<br>*Question relevant when: selected( ${q104_M112_full_list} , '5')*<br>*Response constrained to: .>0* | |
| q604_M113 | ***q604_M113. Vitamin, immunological, etc. - Other 3: "[q104_M113_full_list_other]"***<br>*Question relevant when: selected( ${q104_M113_full_list} , '5')* | |
| q604_M113a *(required)* | q604_M113a. What percent of patients require this item?<br>*Question relevant when: selected( ${q104_M113_full_list} , '5')*<br>*Response constrained to: .>0 and .<=100 or .=999* | |

| Field | Question | Answer |
|---|---|---|
| q604_M113b *(required)* | q604_M113b. How is the item administered, or given, to the patient?<br>*Question relevant when: selected( ${q104_M113_full_list} , '5')* | 1 Oral - solid (e.g. tablet)<br>2 Oral - liquid<br>3 Sublingual<br>4 Intravenous (IV)<br>5 Intramusculuar injection (IM)<br>6 Subcutaneous<br>7 Rectal<br>8 Inhaled (e.g. gas)<br>9 Inserted (e.g. vaginal pessaries)<br>10 Topical - cream<br>11 Topical - drops (e.g. eye drops)<br>12 Other<br>99 Don't know |
| q604_M113c *(required)* | q604_M113c. What is the item's smallest unit of measurement (mg, mcg, ml, g, etc)?<br>*Question relevant when: selected( ${q104_M113_full_list} , '5')* | microgram — microgram (mcg)<br>miligram — miligram (mg)<br>miligram_dissolved_in_liquid — miligram (mg) dissolved in liquid (e.g. 2 mg per 3 militers)<br>gram — gram (g)<br>militer — militer (ml)<br>centiliter — centiliter (cl)<br>litre — litre (l)<br>ml_of _solution — ml of solution (e.g. ml of 10% solution)<br>litre_of_solution — litre of solution (e.g. 1 litre of 5% solution)<br>cubic_meter — cubic meter (m^3)<br>other — other<br>dont_know — don't know |

| Field | Question | Answer |
|---|---|---|
| q604_M113c.1 | q604_M113c.1 If other unit of measurement or route of administration, specify here:<br>*Leave blank if not applicable*<br>*Question relevant when: selected( ${q104_M113_full_list} , '5')* | |
| q604_M113d *(required)* | q604_M113d. In total, how many units are used to treat a patient during all of her care?<br>*Remember to add all units! E.g. 5 days x 200 mg twice per day = 2,000mg*<br>*Question relevant when: selected( ${q104_M113_full_list} , '5')*<br>*Response constrained to: .>0* | |
| q604_M114 | ***q604_M114. Vitamin, immunological, etc. - Other 4: "[q104_M114_full_list_other]"***<br>*Question relevant when: selected( ${q104_M114_full_list} , '5')* | |
| q604_M114a *(required)* | q604_M114a. What percent of patients require this item?<br>*Question relevant when: selected( ${q104_M114_full_list} , '5')*<br>*Response constrained to: .>0 and .<=100 or .=999* | |

| Field | Question | Answer |
|---|---|---|
| q604_M114b *(required)* | q604_M114b. How is the item administered, or given, to the patient?<br>*Question relevant when: selected( ${q104_M114_full_list} , '5')* | 1 Oral - solid (e.g. tablet)<br>2 Oral - liquid<br>3 Sublingual<br>4 Intravenous (IV)<br>5 Intramusculuar injection (IM)<br>6 Subcutaneous<br>7 Rectal<br>8 Inhaled (e.g. gas)<br>9 Inserted (e.g. vaginal pessaries)<br>10 Topical - cream<br>11 Topical - drops (e.g. eye drops)<br>12 Other<br>99 Don't know |
| q604_M114c *(required)* | q604_M114c. What is the item's smallest unit of measurement (mg, mcg, ml, g, etc)?<br>*Question relevant when: selected( ${q104_M114_full_list} , '5')* | microgram — microgram (mcg)<br>miligram — miligram (mg)<br>miligram_dissolved_in_liquid — miligram (mg) dissolved in liquid (e.g. 2 mg per 3 militers)<br>gram — gram (g)<br>militer — militer (ml)<br>centiliter — centiliter (cl)<br>litre — litre (l)<br>ml_of _solution — ml of solution (e.g. ml of 10% solution)<br>litre_of_solution — litre of solution (e.g. 1 litre of 5% solution)<br>cubic_meter — cubic meter (m^3)<br>other — other<br>dont_know — don't know |

| Field | Question | Answer |
|---|---|---|
| q604_M114c.1 | q604_M114c.1 If other unit of measurement or route of administration, specify here:<br>*Leave blank if not applicable*<br>*Question relevant when: selected( ${q104_M114_full_list} , '5')* | |
| q604_M114d *(required)* | q604_M114d. In total, how many units are used to treat a patient during all of her care?<br>*Remember to add all units! E.g. 5 days x 200 mg twice per day = 2,000mg*<br>*Question relevant when: selected( ${q104_M114_full_list} , '5')*<br>*Response constrained to: .>0* | |
| q604_M115 | ***q604_M115. Vitamin, immunological, etc. - Other 5: "[q104_M115_full_list_other]"***<br>*Question relevant when: selected( ${q104_M115_full_list} , '5')* | |
| q604_M115a *(required)* | q604_M115a. What percent of patients require this item?<br>*Question relevant when: selected( ${q104_M115_full_list} , '5')*<br>*Response constrained to: .>0 and .<=100 or .=999* | |

| Field | Question | Answer | |
|---|---|---|---|
| q604_M115b *(required)* | q604_M115b. How is the item administered, or given, to the patient?<br>*Question relevant when: selected( ${q104_M115_full_list} , '5')* | 1 | Oral - solid (e.g. tablet) |
| | | 2 | Oral - liquid |
| | | 3 | Sublingual |
| | | 4 | Intravenous (IV) |
| | | 5 | Intramusculuar injection (IM) |
| | | 6 | Subcutaneous |
| | | 7 | Rectal |
| | | 8 | Inhaled (e.g. gas) |
| | | 9 | Inserted (e.g. vaginal pessaries) |
| | | 10 | Topical - cream |
| | | 11 | Topical - drops (e.g. eye drops) |
| | | 12 | Other |
| | | 99 | Don't know |
| q604_M115c *(required)* | q604_M115c. What is the item's smallest unit of measurement (mg, mcg, ml, g, etc)?<br>*Question relevant when: selected( ${q104_M115_full_list} , '5')* | microgram | microgram (mcg) |
| | | miligram | miligram (mg) |
| | | miligram_dissolved_in_liquid | miligram (mg) dissolved in liquid (e.g. 2 mg per 3 militers) |
| | | gram | gram (g) |
| | | militer | militer (ml) |
| | | centiliter | centiliter (cl) |
| | | litre | litre (l) |
| | | ml_of _solution | ml of solution (e.g. ml of 10% solution) |
| | | litre_of_solution | litre of solution (e.g. 1 litre of 5% solution) |
| | | cubic_meter | cubic meter (m^3) |
| | | other | other |
| | | dont_know | don't know |

| Field | Question | Answer |
|---|---|---|
| q604_M115c.1 | q604_M115c.1 If other unit of measurement or route of administration, specify here:<br>*Leave blank if not applicable*<br>*Question relevant when: selected( ${q104_M115_full_list} , '5')* | |
| q604_M115d *(required)* | q604_M115d. In total, how many units are used to treat a patient during all of her care?<br>*Remember to add all units! E.g. 5 days x 200 mg twice per day = 2,000mg*<br>*Question relevant when: selected( ${q104_M115_full_list} , '5')*<br>*Response constrained to: .>0* | |
| <p><strong><em>D. Medications - Perforations (12)</em></strong></p><br>*Group relevant when: selected( ${section_six_skip_med} , '1')* | | |
| q604_med_notE11 | 604 CONTINUED For each of the following medications and other medical products that are used for perforations, can you tell me …? | |
| note_604_Other | ***Other medication, any class*** | |
| q604_M116 | ***q604_M116. Medication - any class - Other 1: "[q104_M116_full_list_other]"***<br>*Question relevant when: selected( ${q104_M116_full_list} , '5')* | |
| q604_M116a *(required)* | q604_M116a. What percent of patients require this item?<br>*Question relevant when: selected( ${q104_M116_full_list} , '5')*<br>*Response constrained to: .>0 and .<=100 or .=999* | |

| Field | Question | Answer |
|---|---|---|
| q604_M116b *(required)* | q604_M116b. How is the item administered, or given, to the patient?<br>*Question relevant when: selected( ${q104_M116_full_list} , '5')* | 1 Oral - solid (e.g. tablet)<br>2 Oral - liquid<br>3 Sublingual<br>4 Intravenous (IV)<br>5 Intramusculuar injection (IM)<br>6 Subcutaneous<br>7 Rectal<br>8 Inhaled (e.g. gas)<br>9 Inserted (e.g. vaginal pessaries)<br>10 Topical - cream<br>11 Topical - drops (e.g. eye drops)<br>12 Other<br>99 Don't know |
| q604_M116c *(required)* | q604_M116c. What is the item's smallest unit of measurement (mg, mcg, ml, g, etc)?<br>*Question relevant when: selected( ${q104_M116_full_list} , '5')* | microgram — microgram (mcg)<br>miligram — miligram (mg)<br>miligram_dissolved_in_liquid — miligram (mg) dissolved in liquid (e.g. 2 mg per 3 militers)<br>gram — gram (g)<br>militer — militer (ml)<br>centiliter — centiliter (cl)<br>litre — litre (l)<br>ml_of _solution — ml of solution (e.g. ml of 10% solution)<br>litre_of_solution — litre of solution (e.g. 1 litre of 5% solution)<br>cubic_meter — cubic meter (m^3)<br>other — other<br>dont_know — don't know |

| Field | Question | Answer |
|---|---|---|
| q604_M116c.1 | q604_M116c.1 If other unit of measurement or route of administration, specify here:<br>*Leave blank if not applicable*<br>*Question relevant when: selected( ${q104_M116_full_list} , '5')* | |
| q604_M116d *(required)* | q604_M116d. In total, how many units are used to treat a patient during all of her care?<br>*Remember to add all units! E.g. 5 days x 200 mg twice per day = 2,000mg*<br>*Question relevant when: selected( ${q104_M116_full_list} , '5')*<br>*Response constrained to: .>0* | |
| q604_M117 | ***q604_M117. Medication - any class - Other 2: "[q104_M117_full_list_other]"***<br>*Question relevant when: selected( ${q104_M117_full_list} , '5')* | |
| q604_M117a *(required)* | q604_M117a. What percent of patients require this item?<br>*Question relevant when: selected( ${q104_M117_full_list} , '5')*<br>*Response constrained to: .>0 and .<=100 or .=999* | |
| q604_M117b *(required)* | q604_M117b. How is the item administered, or given, to the patient?<br>*Question relevant when: selected( ${q104_M117_full_list} , '5')* | 1 Oral - solid (e.g. tablet)<br>2 Oral - liquid<br>3 Sublingual<br>4 Intravenous (IV)<br>5 Intramusculuar injection (IM)<br>6 Subcutaneous<br>7 Rectal<br>8 Inhaled (e.g. gas)<br>9 Inserted (e.g. vaginal pessaries)<br>10 Topical - cream<br>11 Topical - drops (e.g. eye drops)<br>12 Other<br>99 Don't know |
| q604_M117c *(required)* | q604_M117c. What is the item's smallest unit of measurement (mg, mcg, ml, g, etc)?<br>*Question relevant when: selected( ${q104_M117_full_list} , '5')* | microgram — microgram (mcg)<br>miligram — miligram (mg)<br>miligram_dissolved_in_liquid — miligram (mg) dissolved in liquid (e.g. 2 mg per 3 militers)<br>gram — gram (g)<br>militer — militer (ml)<br>centiliter — centiliter (cl)<br>litre — litre (l)<br>ml_of _solution — ml of solution (e.g. ml of 10% solution)<br>litre_of_solution — litre of solution (e.g. 1 litre of 5% solution)<br>cubic_meter — cubic meter (m^3)<br>other — other<br>dont_know — don't know |

| Field | Question | Answer |
|---|---|---|
| q604_M117c.1 | q604_M117c.1 If other unit of measurement or route of administration, specify here:<br>*Leave blank if not applicable*<br>*Question relevant when: selected( ${q104_M117_full_list} , '5')* | |
| q604_M117d *(required)* | q604_M117d. In total, how many units are used to treat a patient during all of her care?<br>*Remember to add all units! E.g. 5 days x 200 mg twice per day = 2,000mg*<br>*Question relevant when: selected( ${q104_M117_full_list} , '5')*<br>*Response constrained to: .>0* | |
| q604_M118 | ***q604_M118. Medication - any class - Other 3: "[q104_M118_full_list_other]"***<br>*Question relevant when: selected( ${q104_M118_full_list} , '5')* | |
| q604_M118a *(required)* | q604_M118a. What percent of patients require this item?<br>*Question relevant when: selected( ${q104_M118_full_list} , '5')*<br>*Response constrained to: .>0 and .<=100 or .=999* | |
| q604_M118b *(required)* | q604_M118b. How is the item administered, or given, to the patient?<br>*Question relevant when: selected( ${q104_M118_full_list} , '5')* | 1 Oral - solid (e.g. tablet)<br>2 Oral - liquid<br>3 Sublingual<br>4 Intravenous (IV)<br>5 Intramusculuar injection (IM)<br>6 Subcutaneous<br>7 Rectal<br>8 Inhaled (e.g. gas)<br>9 Inserted (e.g. vaginal pessaries)<br>10 Topical - cream<br>11 Topical - drops (e.g. eye drops)<br>12 Other<br>99 Don't know |
| q604_M118c *(required)* | q604_M118c. What is the item's smallest unit of measurement (mg, mcg, ml, g, etc)?<br>*Question relevant when: selected( ${q104_M118_full_list} , '5')* | microgram — microgram (mcg)<br>miligram — miligram (mg)<br>miligram_dissolved_in_liquid — miligram (mg) dissolved in liquid (e.g. 2 mg per 3 militers)<br>gram — gram (g)<br>militer — militer (ml)<br>centiliter — centiliter (cl)<br>litre — litre (l)<br>ml_of _solution — ml of solution (e.g. ml of 10% solution)<br>litre_of_solution — litre of solution (e.g. 1 litre of 5% solution)<br>cubic_meter — cubic meter (m^3)<br>other — other<br>dont_know — don't know |

| Field | Question | Answer |
|---|---|---|
| q604_M118c.1 | q604_M118c.1 If other unit of measurement or route of administration, specify here:<br>*Leave blank if not applicable*<br>*Question relevant when: selected( ${q104_M118_full_list} , '5')* | |
| q604_M118d *(required)* | q604_M118d. In total, how many units are used to treat a patient during all of her care?<br>*Remember to add all units! E.g. 5 days x 200 mg twice per day = 2,000mg*<br>*Question relevant when: selected( ${q104_M118_full_list} , '5')*<br>*Response constrained to: .>0* | |
| q604_M119 | ***q604_M119. Medication - any class - Other 4: "[q104_M119_full_list_other]"***<br>*Question relevant when: selected( ${q104_M119_full_list} , '5')* | |
| q604_M119a *(required)* | q604_M119a. What percent of patients require this item?<br>*Question relevant when: selected( ${q104_M119_full_list} , '5')*<br>*Response constrained to: .>0 and .<=100 or .=999* | |
| q604_M119b *(required)* | q604_M119b. How is the item administered, or given, to the patient?<br>*Question relevant when: selected( ${q104_M119_full_list} , '5')* | 1 Oral - solid (e.g. tablet)<br>2 Oral - liquid<br>3 Sublingual<br>4 Intravenous (IV)<br>5 Intramusculuar injection (IM)<br>6 Subcutaneous<br>7 Rectal<br>8 Inhaled (e.g. gas)<br>9 Inserted (e.g. vaginal pessaries)<br>10 Topical - cream<br>11 Topical - drops (e.g. eye drops)<br>12 Other<br>99 Don't know |
| q604_M119c *(required)* | q604_M119c. What is the item's smallest unit of measurement (mg, mcg, ml, g, etc)?<br>*Question relevant when: selected( ${q104_M119_full_list} , '5')* | microgram — microgram (mcg)<br>miligram — miligram (mg)<br>miligram_dissolved_in_liquid — miligram (mg) dissolved in liquid (e.g. 2 mg per 3 militers)<br>gram — gram (g)<br>militer — militer (ml)<br>centiliter — centiliter (cl)<br>litre — litre (l)<br>ml_of _solution — ml of solution (e.g. ml of 10% solution)<br>litre_of_solution — litre of solution (e.g. 1 litre of 5% solution)<br>cubic_meter — cubic meter (m^3)<br>other — other<br>dont_know — don't know |

| Field | Question | Answer |
|---|---|---|
| q604_M119c.1 | q604_M119c.1 If other unit of measurement or route of administration, specify here:<br>*Leave blank if not applicable*<br>*Question relevant when: selected( ${q104_M119_full_list} , '5')* | |
| q604_M119d *(required)* | q604_M119d. In total, how many units are used to treat a patient during all of her care?<br>*Remember to add all units! E.g. 5 days x 200 mg twice per day = 2,000mg*<br>*Question relevant when: selected( ${q104_M119_full_list} , '5')*<br>*Response constrained to: .>0* | |
| q604_M120 | ***q604_M120. Medication - any class - Other 5: "[q104_M120_full_list_other]"***<br>*Question relevant when: selected( ${q104_M120_full_list} , '5')* | |
| q604_M120a *(required)* | q604_M120a. What percent of patients require this item?<br>*Question relevant when: selected( ${q104_M120_full_list} , '5')*<br>*Response constrained to: .>0 and .<=100 or .=999* | |
| q604_M120b *(required)* | q604_M120b. How is the item administered, or given, to the patient?<br>*Question relevant when: selected( ${q104_M120_full_list} , '5')* | 1 Oral - solid (e.g. tablet)<br>2 Oral - liquid<br>3 Sublingual<br>4 Intravenous (IV)<br>5 Intramusculuar injection (IM)<br>6 Subcutaneous<br>7 Rectal<br>8 Inhaled (e.g. gas)<br>9 Inserted (e.g. vaginal pessaries)<br>10 Topical - cream<br>11 Topical - drops (e.g. eye drops)<br>12 Other<br>99 Don't know |
| q604_M120c *(required)* | q604_M120c. What is the item's smallest unit of measurement (mg, mcg, ml, g, etc)?<br>*Question relevant when: selected( ${q104_M120_full_list} , '5')* | microgram — microgram (mcg)<br>miligram — miligram (mg)<br>miligram_dissolved_in_liquid — miligram (mg) dissolved in liquid (e.g. 2 mg per 3 militers)<br>gram — gram (g)<br>militer — militer (ml)<br>centiliter — centiliter (cl)<br>litre — litre (l)<br>ml_of _solution — ml of solution (e.g. ml of 10% solution)<br>litre_of_solution — litre of solution (e.g. 1 litre of 5% solution)<br>cubic_meter — cubic meter (m^3)<br>other — other<br>dont_know — don't know |

| Field | Question | Answer |
|---|---|---|
| q604_M120c.1 | q604_M120c.1 If other unit of measurement or route of administration, specify here:<br>*Leave blank if not applicable*<br>*Question relevant when: selected( ${q104_M120_full_list} , '5')* | |
| q604_M120d *(required)* | q604_M120d. In total, how many units are used to treat a patient during all of her care?<br>*Remember to add all units! E.g. 5 days x 200 mg twice per day = 2,000mg*<br>*Question relevant when: selected( ${q104_M120_full_list} , '5')*<br>*Response constrained to: .>0* | |
| group_section_seven_intro | | |
| section7_start | **SECTION VII. COSTS OF ALL ITEMS** | |
| section_seven_skip_med | INTERVIEWER: WOULD YOU LIKE TO COMPLETE THIS SECTION NOW OR SKIP THIS SECTION AND RETURN TO IT LATER?<br>*You may need to skip if the participant has indicated that s/he cannot answer the questions in this section.* | 1 Do not skip, complete this section now.<br>2 Skip and come back to this section later. |
| group_section_seven_introB<br>*Group relevant when: selected( ${section_seven_skip_med} , '1')* | | |
| section7_start2 | In this section of the interview, we will review all of the items that you said are used for management of any complication type. For each item that is used, I'm going to ask how much the item costs and how it is procured. | |
| section7_start3 | INTERVIEWER: ENTER 999 FOR ANY THAT ARE UNKNOWN. | |

| | | |
|---|---|---|
| D. Medications - Costs (1) - Overview<br>*Group relevant when: selected( ${section_seven_skip_med} , '1')* | | |
| q704_med_note1 | INTERVIEWER: FOR EACH ITEM LISTED IN THIS SECTION, THE HINT CONTAINS ALL OF THE "SMALLEST UNITS" THAT WERE LISTED AS A HINT IN ITALICS. THESE ARE LISTED TO ALERT YOU TO ANY CASES WHERE THERE MAY BE DIFFERENT FORMS OF THE MEDICATION USED FOR DIFFERENT COMPLICATION TYPES.<br><br>PLEASE CHECK THE LIST IN THE HINT FOR EACH ITEM. IF THERE IS MORE THAN ONE UNIT TYPE LISTED (E.G. MILLIGRAM AND MILLITRES), PLEASE ASK THE QUESTION FOR THE FIRST TYPE LISTED. PLEASE ALSO MAKE A SEPARATE NOTE THAT A SECOND FORM WAS ALSO LISTED. INDICATE THAT IN THE INTERVIEWER COMMENTS AT THE END OF THE QUESTIONNAIRE. WE WILL LATER NEED TO GET PRICES FOR BOTH FORMS OF THE MEDICATION.<br><br>NB: IT MAY BE THE CASE THAT THE SAME UNIT TYPE IS LISTED MORE THAN ONCE (E.G. MILLIGRAM AND MILLIGRAM). THOSE CASES ARE NOT A PROBLEM. | |
| D. Medications - Costs (1)<br>*Group relevant when: selected( ${section_seven_skip_med} , '1')* | | |
| note_704_analgesic1 | ***Analgesic, anti-inflammatory, narcotic, etc.*** | |
| q704_M1 | ***q704_M1. Acetylsalicylic acid (aspirin)*** | |
| q704_M1a *(required)* | q704_M1a. When purchased, how many units of this item typically come in one pack, box, bottle, etc.? (e.g. 100 tablets, 1000 ml's, etc.) (Write 1 if purchased as a single item.)<br>*Smallest units listed were: "[q204_M1c]" , "[q304_M1c]" , "[q404_M1c]" , "[q504_M1c]" , "[q604_M1c]"*<br>*Question relevant when: not(selected( ${q104_M1_full_list} , '6')) and not(selected( ${q104_M1_full_list} , '99'))*<br>*Response constrained to: .>=0* | |
| q704_M1a.1 *(required)* | q704_M1a.1 What is the typical size of each item in the pack, box, etc. (e.g. 1 litre bottles, 250 mg tablets, 3 ml ampoules, etc.)?<br>*Please provide as much information as is available.*<br>*Question relevant when: not(selected( ${q104_M1_full_list} , '6')) and not(selected( ${q104_M1_full_list} , '99'))* | |
| q704_M1b *(required)* | q704_M1b. What is the typical purchase price for that quantity of units (i.e. the quantity noted in the question above)?<br>*Question relevant when: not(selected( ${q104_M1_full_list} , '6')) and not(selected( ${q104_M1_full_list} , '99'))*<br>*Response constrained to: .>=0* | |
| q704_M1c *(required)* | q704_M1c. Please specify the currency for the purchase price.<br>*Question relevant when: not(selected( ${q104_M1_full_list} , '6')) and not(selected( ${q104_M1_full_list} , '99'))* | 1 Tanzanian Shillings<br>2 US dollars<br>3 Euros<br>4 Other<br>99 Don't know |
| q704_M1c.1 | q704_M1c.1 If other currency, specify:<br>*Question relevant when: not(selected( ${q104_M1_full_list} , '6')) and not(selected( ${q104_M1_full_list} , '99'))* | |
| q704_M1d *(required)* | q704_M1d. Please specify the year of the purchase price.<br>*Question relevant when: not(selected( ${q104_M1_full_list} , '6')) and not(selected( ${q104_M1_full_list} , '99'))*<br>*Response constrained to: .>=2000 and .<=2019 or .=999* | |
| q704_M2 | ***q704_M2. Paracetamol***<br>*Question relevant when: not(selected( ${q104_M2_full_list} , '6')) and not(selected( ${q104_M2_full_list} , '99'))* | |
| q704_M2a *(required)* | q704_M2a. When purchased, how many units of this item typically come in one pack, box, bottle, etc.? (e.g. 100 tablets, 1000 ml's, etc.) (Write 1 if purchased as a single item.)<br>[illegible] | |

*Smallest units listed were: "[q204_M2c]" , "[q304_M2c]" , "[q404_M2c]" , "[q504_M2c]" , "[q604_M2c]"*
*Question relevant when: not(selected( ${q104_M2_full_list} , '6')) and not(selected( ${q104_M2_full_list} , '99'))*
*Response constrained to: .>=0*

| Field | Question | Answer |
|---|---|---|
| q704_M2a.1 *(required)* | q704_M2a.1 What is the typical size of each item in the pack, box, etc. (e.g. 1 litre bottles, 250 mg tablets, 3 ml ampoules, etc.)?<br>*Please provide as much information as is available.*<br>*Question relevant when: not(selected( ${q104_M2_full_list} , '6')) and not(selected( ${q104_M2_full_list} , '99'))* | |
| q704_M2b *(required)* | q704_M2b. What is the typical purchase price for that quantity of units (i.e. the quantity noted in the question above)?<br>*Question relevant when: not(selected( ${q104_M2_full_list} , '6')) and not(selected( ${q104_M2_full_list} , '99'))*<br>*Response constrained to: .>=0* | |
| q704_M2c *(required)* | q704_M2c. Please specify the currency for the purchase price.<br>*Question relevant when: not(selected( ${q104_M2_full_list} , '6')) and not(selected( ${q104_M2_full_list} , '99'))* | 1 Tanzanian Shillings<br>2 US dollars<br>3 Euros<br>4 Other<br>99 Don't know |
| q704_M2c.1 | q704_M2c.1 If other currency, specify:<br>*Question relevant when: not(selected( ${q104_M2_full_list} , '6')) and not(selected( ${q104_M2_full_list} , '99'))* | |

| | | |
|---|---|---|
| q704_M2d *(required)* | q704_M2d. Please specify the year of the purchase price.<br>*Question relevant when: not(selected( ${q104_M2_full_list} , '6')) and not(selected( ${q104_M2_full_list} , '99'))*<br>*Response constrained to: .>=2000 and .<=2019 or .=999* | |
| q704_M3 | ***q704_M3. Pethidine hydrochloride***<br>*Question relevant when: not(selected( ${q104_M3_full_list} , '6')) and not(selected( ${q104_M3_full_list} , '99'))* | |
| q704_M3a *(required)* | q704_M3a. When purchased, how many units of this item typically come in one pack, box, bottle, etc.? (e.g. 100 tablets, 1000 ml's, etc.) (Write 1 if purchased as a single item.)<br>*Smallest units listed were: "[q204_M3c]" , "[q304_M3c]" , "[q404_M3c]" , "[q504_M3c]" , "[q604_M3c]"*<br>*Question relevant when: not(selected( ${q104_M3_full_list} , '6')) and not(selected( ${q104_M3_full_list} , '99'))*<br>*Response constrained to: .>=0* | |
| q704_M3a.1 *(required)* | q704_M3a.1 What is the typical size of each item in the pack, box, etc. (e.g. 1 litre bottles, 250 mg tablets, 3 ml ampoules, etc.)?<br>*Please provide as much information as is available.*<br>*Question relevant when: not(selected( ${q104_M3_full_list} , '6')) and not(selected( ${q104_M3_full_list} , '99'))* | |
| q704_M3b *(required)* | q704_M3b. What is the typical purchase price for that quantity of units (i.e. the quantity noted in the question above)?<br>*Question relevant when: not(selected( ${q104_M3_full_list} , '6')) and not(selected( ${q104_M3_full_list} , '99'))*<br>*Response constrained to: .>=0* | |
| q704_M3c *(required)* | q704_M3c. Please specify the currency for the purchase price.<br>*Question relevant when: not(selected( ${q104_M3_full_list} , '6')) and not(selected( ${q104_M3_full_list} , '99'))* | 1 Tanzanian Shillings<br>2 US dollars<br>3 Euros<br>4 Other<br>99 Don't know |
| q704_M3c.1 | q704_M3c.1 If other currency, specify:<br>*Leave blank if not applicable*<br>*Question relevant when: not(selected( ${q104_M3_full_list} , '6')) and not(selected( ${q104_M3_full_list} , '99'))* | |
| q704_M3d *(required)* | q704_M3d. Please specify the year of the purchase price.<br>*Question relevant when: not(selected( ${q104_M3_full_list} , '6')) and not(selected( ${q104_M3_full_list} , '99'))*<br>*Response constrained to: .>=2000 and .<=2019 or .=999* | |
| q704_M4 | ***q704_M4. Tramadol Hydrochloride***<br>*Question relevant when: not(selected( ${q104_M4_full_list} , '6')) and not(selected( ${q104_M4_full_list} , '99'))* | |
| q704_M4a *(required)* | q704_M4a. When purchased, how many units of this item typically come in one pack, box, bottle, etc.? (e.g. 100 tablets, 1000 ml's, etc.) (Write 1 if purchased as a single item.)<br>*Smallest units listed were: "[q204_M4c]" , "[q304_M4c]" , "[q404_M4c]" , "[q504_M4c]" , "[q604_M4c]"*<br>*Question relevant when: not(selected( ${q104_M4_full_list} , '6')) and not(selected( ${q104_M4_full_list} , '99'))*<br>*Response constrained to: .>=0* | |
| q704_M4a.1 *(required)* | q704_M4a.1 What is the typical size of each item in the pack, box, etc. (e.g. 1 litre bottles, 250 mg tablets, 3 ml ampoules, etc.)?<br>*Please provide as much information as is available.*<br>*Question relevant when: not(selected( ${q104_M4_full_list} , '6')) and not(selected( ${q104_M4_full_list} , '99'))* | |
| q704_M4b *(required)* | q704_M4b. What is the typical purchase price for that quantity of units (i.e. the quantity noted in the question above)?<br>*Question relevant when: not(selected( ${q104_M4_full_list} , '6')) and not(selected( ${q104_M4_full_list} , '99'))*<br>*Response constrained to: .>=0* | |
| q704_M4c *(required)* | q704_M4c. Please specify the currency for the purchase price.<br>*Question relevant when: not(selected( ${q104_M4_full_list} , '6')) and not(selected( ${q104_M4_full_list} , '99'))* | 1 Tanzanian Shillings<br>2 US dollars<br>3 Euros |

| Field | Question | Answer |
|---|---|---|
| | | 4 Other<br>99 Don't know |
| q704_M4c.1 | q704_M4c.1 If other currency, specify:<br>*Leave blank if not applicable*<br>*Question relevant when: not(selected( ${q104_M4_full_list} , '6')) and not(selected( ${q104_M4_full_list} , '99'))* | |
| q704_M4d *(required)* | q704_M4d. Please specify the year of the purchase price.<br>*Question relevant when: not(selected( ${q104_M4_full_list} , '6')) and not(selected( ${q104_M4_full_list} , '99'))*<br>*Response constrained to: .>=2000 and .<=2019 or .=999* | |
| q704_M5 | ***q704_M5. Ibuprofen***<br>*Question relevant when: not(selected( ${q104_M5_full_list} , '6')) and not(selected( ${q104_M5_full_list} , '99'))* | |
| q704_M5a *(required)* | q704_M5a. When purchased, how many units of this item typically come in one pack, box, bottle, etc.? (e.g. 100 tablets, 1000 ml's, etc.) (Write 1 if purchased as a single item.)<br>*Smallest units listed were: "[q204_M5c]" , "[q304_M5c]" , "[q404_M5c]" , "[q504_M5c]" , "[q604_M5c]"*<br>*Question relevant when: not(selected( ${q104_M5_full_list} , '6')) and not(selected( ${q104_M5_full_list} , '99'))*<br>*Response constrained to: .>=0* | |
| q704_M5a.1 *(required)* | q704_M5a.1 What is the typical size of each item in the pack, box, etc. (e.g. 1 litre bottles, 250 mg tablets, 3 ml ampoules, etc.)?<br>*Please provide as much information as is available.*<br>*Question relevant when: not(selected( ${q104_M5_full_list} , '6')) and not(selected( ${q104_M5_full_list} , '99'))* | |
| q704_M5b *(required)* | q704_M5b. What is the typical purchase price for that quantity of units (i.e. the quantity noted in the question above)?<br>*Question relevant when: not(selected( ${q104_M5_full_list} , '6')) and not(selected( ${q104_M5_full_list} , '99'))*<br>*Response constrained to: .>=0* | |
| q704_M5c *(required)* | q704_M5c. Please specify the currency for the purchase price.<br>*Question relevant when: not(selected( ${q104_M5_full_list} , '6')) and not(selected( ${q104_M5_full_list} , '99'))* | 1 Tanzanian Shillings<br>2 US dollars<br>3 Euros<br>4 Other<br>99 Don't know |
| q704_M5c.1 | q704_M5c.1 If other currency, specify:<br>*Leave blank if not applicable*<br>*Question relevant when: not(selected( ${q104_M5_full_list} , '6')) and not(selected( ${q104_M5_full_list} , '99'))* | |
| q704_M5d *(required)* | q704_M5d. Please specify the year of the purchase price.<br>*Question relevant when: not(selected( ${q104_M5_full_list} , '6')) and not(selected( ${q104_M5_full_list} , '99'))*<br>*Response constrained to: .>=2000 and .<=2019 or .=999* | |
| q704_M6 | ***q704_M6. Diclofenac***<br>*Question relevant when: not(selected( ${q104_M6_full_list} , '6')) and not(selected( ${q104_M6_full_list} , '99'))* | |
| q704_M6a *(required)* | q704_M6a. When purchased, how many units of this item typically come in one pack, box, bottle, etc.? (e.g. 100 tablets, 1000 ml's, etc.) (Write 1 if purchased as a single item.)<br>*Smallest units listed were: "[q204_M6c]" , "[q304_M6c]" , "[q404_M6c]" , "[q504_M6c]" , "[q604_M6c]"*<br>*Question relevant when: not(selected( ${q104_M6_full_list} , '6')) and not(selected( ${q104_M6_full_list} , '99'))*<br>*Response constrained to: .>=0* | |
| q704_M6a.1 *(required)* | q704_M6a.1 What is the typical size of each item in the pack, box, etc. (e.g. 1 litre bottles, 250 mg tablets, 3 ml ampoules, etc.)?<br>*Please provide as much information as is available.*<br>*Question relevant when: not(selected( ${q104_M6_full_list} , '6')) and not(selected( ${q104_M6_full_list} , '99'))* | |
| q704_M6b *(required)* | q704_M6b. What is the typical purchase price for that quantity of units (i.e. the quantity noted in the question above)?<br>*Question relevant when: not(selected( ${q104_M6_full_list} , '6')) and not(selected( ${q104_M6_full_list} , '99'))*<br>*Response constrained to: .>=0* | |
| q704_M6c *(required)* | q704_M6c. Please specify the currency for the purchase price.<br>*Question relevant when: not(selected( ${q104_M6_full_list} , '6')) and not(selected( ${q104_M6_full_list} , '99'))* | 1 Tanzanian Shillings<br>2 US dollars<br>3 Euros<br>4 Other<br>99 Don't know |
| q704_M6c.1 | q704_M6c.1 If other currency, specify:<br>*Leave blank if not applicable*<br>*Question relevant when: not(selected( ${q104_M6_full_list} , '6')) and not(selected( ${q104_M6_full_list} , '99'))* | |
| q704_M6d *(required)* | q704_M6d. Please specify the year of the purchase price.<br>*Question relevant when: not(selected( ${q104_M6_full_list} , '6')) and not(selected( ${q104_M6_full_list} , '99'))*<br>*Response constrained to: .>=2000 and .<=2019 or .=999* | |
| q704_M7 | ***q704_M7. Diclofenac sodium***<br>*Question relevant when: not(selected( ${q104_M7_full_list} , '6')) and not(selected( ${q104_M7_full_list} , '99'))* | |

| Field | Question | Answer |
|---|---|---|
| q704_M7a *(required)* | q704_M7a. When purchased, how many units of this item typically come in one pack, box, bottle, etc.? (e.g. 100 tablets, 1000 ml's, etc.) (Write 1 if purchased as a single item.)<br>*Smallest units listed were: "[q204_M7c]" , "[q304_M7c]" , "[q404_M7c]" , "[q504_M7c]" , "[q604_M7c]"*<br>*Question relevant when: not(selected( ${q104_M7_full_list} , '6')) and not(selected( ${q104_M7_full_list} , '99'))*<br>*Response constrained to: .>=0* | |
| q704_M7a.1 *(required)* | q704_M7a.1 What is the typical size of each item in the pack, box, etc. (e.g. 1 litre bottles, 250 mg tablets, 3 ml ampoules, etc.)?<br>*Please provide as much information as is available.*<br>*Question relevant when: not(selected( ${q104_M7_full_list} , '6')) and not(selected( ${q104_M7_full_list} , '99'))* | |
| q704_M7b *(required)* | q704_M7b. What is the typical purchase price for that quantity of units (i.e. the quantity noted in the question above)?<br>*Question relevant when: not(selected( ${q104_M7_full_list} , '6')) and not(selected( ${q104_M7_full_list} , '99'))*<br>*Response constrained to: .>=0* | |
| q704_M7c *(required)* | q704_M7c. Please specify the currency for the purchase price.<br>*Question relevant when: not(selected( ${q104_M7_full_list} , '6')) and not(selected( ${q104_M7_full_list} , '99'))* | 1 Tanzanian Shillings<br>2 US dollars<br>3 Euros<br>4 Other<br>99 Don't know |

| | | |
|---|---|---|
| q704_M7c.1 | q704_M7c.1 If other currency, specify:<br>*Leave blank if not applicable*<br>*Question relevant when: not(selected( ${q104_M7_full_list} , '6')) and not(selected( ${q104_M7_full_list} , '99'))* | |
| q704_M7d *(required)* | q704_M7d. Please specify the year of the purchase price.<br>*Question relevant when: not(selected( ${q104_M7_full_list} , '6')) and not(selected( ${q104_M7_full_list} , '99'))*<br>*Response constrained to: .>=2000 and .<=2019 or .=999* | |
| q704_M8 | ***q704_M8. Dypron (dipyrone), 250mg/ml (Metamizole)***<br>*Question relevant when: not(selected( ${q104_M8_full_list} , '6')) and not(selected( ${q104_M8_full_list} , '99'))* | |
| q704_M8a *(required)* | q704_M8a. When purchased, how many units of this item typically come in one pack, box, bottle, etc.? (e.g. 100 tablets, 1000 ml's, etc.) (Write 1 if purchased as a single item.)<br>*Smallest units listed were: "[q204_M8c]" , "[q304_M8c]" , "[q404_M8c]" , "[q504_M8c]" , "[q604_M8c]"*<br>*Question relevant when: not(selected( ${q104_M8_full_list} , '6')) and not(selected( ${q104_M8_full_list} , '99'))*<br>*Response constrained to: .>=0* | |
| q704_M8a.1 *(required)* | q704_M8a.1 What is the typical size of each item in the pack, box, etc. (e.g. 1 litre bottles, 250 mg tablets, 3 ml ampoules, etc.)?<br>*Please provide as much information as is available.*<br>*Question relevant when: not(selected( ${q104_M8_full_list} , '6')) and not(selected( ${q104_M8_full_list} , '99'))* | |
| q704_M8b *(required)* | q704_M8b. What is the typical purchase price for that quantity of units (i.e. the quantity noted in the question above)?<br>*Question relevant when: not(selected( ${q104_M8_full_list} , '6')) and not(selected( ${q104_M8_full_list} , '99'))*<br>*Response constrained to: .>=0* | |
| q704_M8c *(required)* | q704_M8c. Please specify the currency for the purchase price.<br>*Question relevant when: not(selected( ${q104_M8_full_list} , '6')) and not(selected( ${q104_M8_full_list} , '99'))* | 1 Tanzanian Shillings<br>2 US dollars<br>3 Euros<br>4 Other<br>99 Don't know |
| q704_M8c.1 | q704_M8c.1 If other currency, specify:<br>*Leave blank if not applicable*<br>*Question relevant when: not(selected( ${q104_M8_full_list} , '6')) and not(selected( ${q104_M8_full_list} , '99'))* | |
| q704_M8d *(required)* | q704_M8d. Please specify the year of the purchase price.<br>*Question relevant when: not(selected( ${q104_M8_full_list} , '6')) and not(selected( ${q104_M8_full_list} , '99'))*<br>*Response constrained to: .>=2000 and .<=2019 or .=999* | |
| q704_M9 | ***q704_M9. Fentanyl citrate, 50 mcg/ml***<br>*Question relevant when: not(selected( ${q104_M9_full_list} , '6')) and not(selected( ${q104_M9_full_list} , '99'))* | |
| q704_M9a *(required)* | q704_M9a. When purchased, how many units of this item typically come in one pack, box, bottle, etc.? (e.g. 100 tablets, 1000 ml's, etc.) (Write 1 if purchased as a single item.)<br>*Smallest units listed were: "[q204_M9c]" , "[q304_M9c]" , "[q404_M9c]" , "[q504_M9c]" , "[q604_M9c]"*<br>*Question relevant when: not(selected( ${q104_M9_full_list} , '6')) and not(selected( ${q104_M9_full_list} , '99'))*<br>*Response constrained to: .>=0* | |
| q704_M9a.1 *(required)* | q704_M9a.1 What is the typical size of each item in the pack, box, etc. (e.g. 1 litre bottles, 250 mg tablets, 3 ml ampoules, etc.)?<br>*Please provide as much information as is available.*<br>*Question relevant when: not(selected( ${q104_M9_full_list} , '6')) and not(selected( ${q104_M9_full_list} , '99'))* | |
| q704_M9b *(required)* | q704_M9b. What is the typical purchase price for that quantity of units (i.e. the quantity noted in the question above)?<br>*Question relevant when: not(selected( ${q104_M9_full_list} , '6')) and not(selected( ${q104_M9_full_list} , '99'))* | |

| Field | Question | Answer |
|---|---|---|
| | *Response constrained to: .>=0* | |
| q704_M9c *(required)* | q704_M9c. Please specify the currency for the purchase price.<br>*Question relevant when: not(selected( ${q104_M9_full_list} , '6')) and not(selected( ${q104_M9_full_list} , '99'))* | 1 Tanzanian Shillings<br>2 US dollars<br>3 Euros<br>4 Other<br>99 Don't know |
| q704_M9c.1 | q704_M9c.1 If other currency, specify:<br>*Leave blank if not applicable*<br>*Question relevant when: not(selected( ${q104_M9_full_list} , '6')) and not(selected( ${q104_M9_full_list} , '99'))* | |
| q704_M9d *(required)* | q704_M9d. Please specify the year of the purchase price.<br>*Question relevant when: not(selected( ${q104_M9_full_list} , '6')) and not(selected( ${q104_M9_full_list} , '99'))*<br>*Response constrained to: .>=2000 and .<=2019 or .=999* | |
| q704_M10 | ***q704_M10. Analgesic - other 1: "[q104_M10_full_list_other]"***<br>*Question relevant when: not(selected( ${q104_M10_full_list} , '6')) and not(selected( ${q104_M10_full_list} , '99')) and string-length( ${q104_M10_full_list_other} ) >0* | |
| q704_M10a *(required)* | q704_M10a. When purchased, how many units of this item typically come in one pack, box, bottle, etc.? (e.g. 100 tablets, 1000 ml's, etc.) (Write 1 if purchased as a single item.)<br>*Smallest units listed were: "[q204_M10c]" , "[q304_M10c]" , "[q404_M10c]" , "[q504_M10c]" , "[q604_M10c]"*<br>*Question relevant when: not(selected( ${q104_M10_full_list} , '6')) and not(selected( ${q104_M10_full_list} , '99')) and string-length( ${q104_M10_full_list_other} ) >0*<br>*Response constrained to: .>=0* | |
| q704_M10a.1 *(required)* | q704_M10a.1 What is the typical size of each item in the pack, box, etc. (e.g. 1 litre bottles, 250 mg tablets, 3 ml ampoules, etc.)?<br>*Please provide as much information as is available.*<br>*Question relevant when: not(selected( ${q104_M10_full_list} , '6')) and not(selected( ${q104_M10_full_list} , '99')) and string-length( ${q104_M10_full_list_other} ) >0* | |
| q704_M10b *(required)* | q704_M10b. What is the typical purchase price for that quantity of units (i.e. the quantity noted in the question above)?<br>*Question relevant when: not(selected( ${q104_M10_full_list} , '6')) and not(selected( ${q104_M10_full_list} , '99')) and string-length( ${q104_M10_full_list_other} ) >0*<br>*Response constrained to: .>=0* | |
| q704_M10c *(required)* | q704_M10c. Please specify the currency for the purchase price.<br>*Question relevant when: not(selected( ${q104_M10_full_list} , '6')) and not(selected( ${q104_M10_full_list} , '99')) and string-length( ${q104_M10_full_list_other} ) >0* | 1 Tanzanian Shillings<br>2 US dollars<br>3 Euros<br>4 Other<br>99 Don't know |
| q704_M10c.1 | q704_M10c.1 If other currency, specify:<br>*Leave blank if not applicable*<br>*Question relevant when: not(selected( ${q104_M10_full_list} , '6')) and not(selected( ${q104_M10_full_list} , '99')) and string-length( ${q104_M10_full_list_other} ) >0* | |
| q704_M10d *(required)* | q704_M10d. Please specify the year of the purchase price.<br>*Question relevant when: not(selected( ${q104_M10_full_list} , '6')) and not(selected( ${q104_M10_full_list} , '99')) and string-length( ${q104_M10_full_list_other} ) >0*<br>*Response constrained to: .>=2000 and .<=2019 or .=999* | |
| q704_M11 | ***q704_M11. Analgesic - other 2: "[q104_M11_full_list_other]"***<br>*Question relevant when: not(selected( ${q104_M11_full_list} , '6')) and not(selected( ${q104_M11_full_list} , '99')) and string-length( ${q104_M11_full_list_other} ) >0* | |
| q704_M11a *(required)* | q704_M11a. When purchased, how many units of this item typically come in one pack, box, bottle, etc.? (e.g. 100 tablets, 1000 ml's, etc.) (Write 1 if purchased as a single item.)<br>*Smallest units listed were: "[q204_M11c]" , "[q304_M11c]" , "[q404_M11c]" , "[q504_M11c]" , "[q604_M11c]"*<br>*Question relevant when: not(selected( ${q104_M11_full_list} , '6')) and not(selected( ${q104_M11_full_list} , '99')) and string-length( ${q104_M11_full_list_other} ) >0*<br>*Response constrained to: .>=0* | |
| q704_M11a.1 *(required)* | q704_M11a.1 What is the typical size of each item in the pack, box, etc. (e.g. 1 litre bottles, 250 mg tablets, 3 ml ampoules, etc.)?<br>*Please provide as much information as is available.*<br>*Question relevant when: not(selected( ${q104_M11_full_list} , '6')) and not(selected( ${q104_M11_full_list} , '99')) and string-length( ${q104_M11_full_list_other} ) >0* | |
| q704_M11b *(required)* | q704_M11b. What is the typical purchase price for that quantity of units (i.e. the quantity noted in the question above)?<br>*Question relevant when: not(selected( ${q104_M11_full_list} , '6')) and not(selected( ${q104_M11_full_list} ,* | |

| Field | Question | Answer |
|---|---|---|
| | *'99')) and string-length( ${q104_M11_full_list_other} ) >0*<br>*Response constrained to: .>=0* | |
| q704_M11c *(required)* | q704_M11c. Please specify the currency for the purchase price.<br>*Question relevant when: not(selected( ${q104_M11_full_list} , '6')) and not(selected( ${q104_M11_full_list} , '99')) and string-length( ${q104_M11_full_list_other} ) >0* | 1 Tanzanian Shillings<br>2 US dollars<br>3 Euros<br>4 Other<br>99 Don't know |
| q704_M11c.1 | q704_M11c.1 If other currency, specify:<br>*Leave blank if not applicable*<br>*Question relevant when: not(selected( ${q104_M11_full_list} , '6')) and not(selected( ${q104_M11_full_list} , '99')) and string-length( ${q104_M11_full_list_other} ) >0* | |
| q704_M11d *(required)* | q704_M11d. Please specify the year of the purchase price.<br>*Question relevant when: not(selected( ${q104_M11_full_list} , '6')) and not(selected( ${q104_M11_full_list} , '99')) and string-length( ${q104_M11_full_list_other} ) >0*<br>*Response constrained to: .>=2000 and .<=2019 or .=999* | |
| q704_M12 | ***q704_M12. Analgesic - other 3: "[q104_M12_full_list_other]"***<br>*Question relevant when: not(selected( ${q104_M12_full_list} , '6')) and not(selected( ${q104_M12_full_list} , '99')) and string-length( ${q104_M12_full_list_other} ) >0* | |

| | | |
|---|---|---|
| q704_M12a *(required)* | q704_M12a. When purchased, how many units of this item typically come in one pack, box, bottle, etc.? (e.g. 100 tablets, 1000 ml's, etc.) (Write 1 if purchased as a single item.)<br>*Smallest units listed were: "[q204_M12c]" , "[q304_M12c]" , "[q404_M12c]" , "[q504_M12c]" , "[q604_M12c]"*<br>*Question relevant when: not(selected( ${q104_M12_full_list} , '6')) and not(selected( ${q104_M12_full_list} , '99')) and string-length( ${q104_M12_full_list_other} ) >0*<br>*Response constrained to: .>=0* | |
| q704_M12a.1 *(required)* | q704_M12a.1 What is the typical size of each item in the pack, box, etc. (e.g. 1 litre bottles, 250 mg tablets, 3 ml ampoules, etc.)?<br>*Please provide as much information as is available.*<br>*Question relevant when: not(selected( ${q104_M12_full_list} , '6')) and not(selected( ${q104_M12_full_list} , '99')) and string-length( ${q104_M12_full_list_other} ) >0* | |
| q704_M12b *(required)* | q704_M12b. What is the typical purchase price for that quantity of units (i.e. the quantity noted in the question above)?<br>*Question relevant when: not(selected( ${q104_M12_full_list} , '6')) and not(selected( ${q104_M12_full_list} , '99')) and string-length( ${q104_M12_full_list_other} ) >0*<br>*Response constrained to: .>=0* | |
| q704_M12c *(required)* | q704_M12c. Please specify the currency for the purchase price.<br>*Question relevant when: not(selected( ${q104_M12_full_list} , '6')) and not(selected( ${q104_M12_full_list} , '99')) and string-length( ${q104_M12_full_list_other} ) >0* | 1 Tanzanian Shillings<br>2 US dollars<br>3 Euros<br>4 Other<br>99 Don't know |
| q704_M12c.1 | q704_M12c.1 If other currency, specify:<br>*Leave blank if not applicable*<br>*Question relevant when: not(selected( ${q104_M12_full_list} , '6')) and not(selected( ${q104_M12_full_list} , '99')) and string-length( ${q104_M12_full_list_other} ) >0* | |
| q704_M12d *(required)* | q704_M12d. Please specify the year of the purchase price.<br>*Question relevant when: not(selected( ${q104_M12_full_list} , '6')) and not(selected( ${q104_M12_full_list} , '99')) and string-length( ${q104_M12_full_list_other} ) >0*<br>*Response constrained to: .>=2000 and .<=2019 or .=999* | |
| q704_M13 | ***q704_M13. Analgesic - other 4: "[q104_M13_full_list_other]"***<br>*Question relevant when: not(selected( ${q104_M13_full_list} , '6')) and not(selected( ${q104_M13_full_list} , '99')) and string-length( ${q104_M13_full_list_other} ) >0* | |
| q704_M13a *(required)* | q704_M13a. When purchased, how many units of this item typically come in one pack, box, bottle, etc.? (e.g. 100 tablets, 1000 ml's, etc.) (Write 1 if purchased as a single item.)<br>*Smallest units listed were: "[q204_M13c]" , "[q304_M13c]" , "[q404_M13c]" , "[q504_M13c]" , "[q604_M13c]"*<br>*Question relevant when: not(selected( ${q104_M13_full_list} , '6')) and not(selected( ${q104_M13_full_list} , '99')) and string-length( ${q104_M13_full_list_other} ) >0*<br>*Response constrained to: .>=0* | |
| q704_M13a.1 *(required)* | q704_M13a.1 What is the typical size of each item in the pack, box, etc. (e.g. 1 litre bottles, 250 mg tablets, 3 ml ampoules, etc.)?<br>*Please provide as much information as is available.*<br>*Question relevant when: not(selected( ${q104_M13_full_list} , '6')) and not(selected( ${q104_M13_full_list} , '99')) and string-length( ${q104_M13_full_list_other} ) >0* | |
| q704_M13b *(required)* | q704_M13b. What is the typical purchase price for that quantity of units (i.e. the quantity noted in the question above)?<br>*Question relevant when: not(selected( ${q104_M13_full_list} , '6')) and not(selected( ${q104_M13_full_list} ,* | |

| Field | Question | Answer |
|---|---|---|
| | *'99')) and string-length( ${q104_M13_full_list_other} ) >0*<br>*Response constrained to: .>=0* | |
| q704_M13c *(required)* | q704_M13c. Please specify the currency for the purchase price.<br>*Question relevant when: not(selected( ${q104_M13_full_list} , '6')) and not(selected( ${q104_M13_full_list} , '99')) and string-length( ${q104_M13_full_list_other} ) >0* | 1 Tanzanian Shillings<br>2 US dollars<br>3 Euros<br>4 Other<br>99 Don't know |
| q704_M13c.1 | q704_M13c.1 If other currency, specify:<br>*Leave blank if not applicable*<br>*Question relevant when: not(selected( ${q104_M13_full_list} , '6')) and not(selected( ${q104_M13_full_list} , '99')) and string-length( ${q104_M13_full_list_other} ) >0* | |
| q704_M13d *(required)* | q704_M13d. Please specify the year of the purchase price.<br>*Question relevant when: not(selected( ${q104_M13_full_list} , '6')) and not(selected( ${q104_M13_full_list} , '99')) and string-length( ${q104_M13_full_list_other} ) >0*<br>*Response constrained to: .>=2000 and .<=2019 or .=999* | |
| q704_M14 | ***q704_M14. Analgesic - other 5: "[q104_M14_full_list_other]"***<br>*Question relevant when: not(selected( ${q104_M14_full_list} , '6')) and not(selected( ${q104_M14_full_list} , '99')) and string-length( ${q104_M14_full_list_other} ) >0* | |
| q704_M14a *(required)* | q704_M14a. When purchased, how many units of this item typically come in one pack, box, bottle, etc.? (e.g. 100 tablets, 1000 ml's, etc.) (Write 1 if purchased as a single item.)<br>*Smallest units listed were: "[q204_M14c]" , "[q304_M14c]" , "[q404_M14c]" , "[q504_M14c]" , "[q604_M14c]"*<br>*Question relevant when: not(selected( ${q104_M14_full_list} , '6')) and not(selected( ${q104_M14_full_list} , '99')) and string-length( ${q104_M14_full_list_other} ) >0*<br>*Response constrained to: .>=0* | |
| q704_M14a.1 *(required)* | q704_M14a.1 What is the typical size of each item in the pack, box, etc. (e.g. 1 litre bottles, 250 mg tablets, 3 ml ampoules, etc.)?<br>*Please provide as much information as is available.*<br>*Question relevant when: not(selected( ${q104_M14_full_list} , '6')) and not(selected( ${q104_M14_full_list} , '99')) and string-length( ${q104_M14_full_list_other} ) >0* | |
| q704_M14b *(required)* | q704_M14b. What is the typical purchase price for that quantity of units (i.e. the quantity noted in the question above)?<br>*Question relevant when: not(selected( ${q104_M14_full_list} , '6')) and not(selected( ${q104_M14_full_list} , '99')) and string-length( ${q104_M14_full_list_other} ) >0*<br>*Response constrained to: .>=0* | |
| q704_M14c *(required)* | q704_M14c. Please specify the currency for the purchase price.<br>*Question relevant when: not(selected( ${q104_M14_full_list} , '6')) and not(selected( ${q104_M14_full_list} , '99')) and string-length( ${q104_M14_full_list_other} ) >0* | 1 Tanzanian Shillings<br>2 US dollars<br>3 Euros<br>4 Other<br>99 Don't know |
| q704_M14c.1 | q704_M14c.1 If other currency, specify:<br>*Leave blank if not applicable*<br>*Question relevant when: not(selected( ${q104_M14_full_list} , '6')) and not(selected( ${q104_M14_full_list} , '99')) and string-length( ${q104_M14_full_list_other} ) >0* | |
| q704_M14d *(required)* | q704_M14d. Please specify the year of the purchase price.<br>*Question relevant when: not(selected( ${q104_M14_full_list} , '6')) and not(selected( ${q104_M14_full_list} , '99')) and string-length( ${q104_M14_full_list_other} ) >0*<br>*Response constrained to: .>=2000 and .<=2019 or .=999* | |
| D. Medications - Cost (2)<br>*Group relevant when: selected( ${section_seven_skip_med} , '1')* | | |
| q704_med_note2 | 704 For each of the following medications and other medical products that are used for postabortion care, can you tell me …? | |
| note_704_anesthetic | ***Anesthetic - local, general, etc. (and oxygen)*** | |
| q704_M15 | ***q704_M15. Halothane***<br>*Question relevant when: not(selected( ${q104_M15_full_list} , '6')) and not(selected( ${q104_M15_full_list} , '99'))* | |
| q704_M15a *(required)* | q704_M15a. When purchased, how many units of this item typically come in one pack, box, bottle, etc.? (e.g. 100 tablets, 1000 ml's, etc.) (Write 1 if purchased as a single item.)<br>*Smallest units listed were: "[q204_M15c]" , "[q304_M15c]" , "[q404_M15c]" , "[q504_M15c]" , "[q604_M15c]"*<br>*Question relevant when: not(selected( ${q104_M15_full_list} , '6')) and not(selected( ${q104_M15_full_list} , '99'))*<br>*Response constrained to: .>=0* | |
| q704_M15a.1 *(required)* | q704_M15a.1 What is the typical size of each item in the pack, box, etc. (e.g. 1 litre bottles, 250 mg tablets, 3 ml ampoules, etc.)?<br>*Please provide as much information as is available.* | |

*Please provide as much information as is available.*

*Question relevant when: not(selected( ${q104_M15_full_list} , '6')) and not(selected( ${q104_M15_full_list} , '99'))*

| Field | Question | Answer |
|---|---|---|
| q704_M15b *(required)* | q704_M15b. What is the typical purchase price for that quantity of units (i.e. the quantity noted in the question above)?<br>*Question relevant when: not(selected( ${q104_M15_full_list} , '6')) and not(selected( ${q104_M15_full_list} , '99'))*<br>*Response constrained to: .>=0* | |
| q704_M15c *(required)* | q704_M15c. Please specify the currency for the purchase price.<br>*Question relevant when: not(selected( ${q104_M15_full_list} , '6')) and not(selected( ${q104_M15_full_list} , '99'))* | 1 Tanzanian Shillings<br>2 US dollars<br>3 Euros<br>4 Other<br>99 Don't know |
| q704_M15c.1 | q704_M15c.1 If other currency, specify:<br>*Leave blank if not applicable*<br>*Question relevant when: not(selected( ${q104_M15_full_list} , '6')) and not(selected( ${q104_M15_full_list} , '99'))* | |
| q704_M15d *(required)* | q704_M15d. Please specify the year of the purchase price.<br>*Question relevant when: not(selected( ${q104_M15_full_list} , '6')) and not(selected( ${q104_M15_full_list} , '99'))*<br>*Response constrained to: .>=2000 and .<=2019 or .=999* | |
| q704_M16 | ***q704_M16. Ketamine (Ketalar), 50mg/ml***<br>*Question relevant when: not(selected( ${q104_M16_full_list} , '6')) and not(selected( ${q104_M16_full_list} , '99'))* | |
| q704_M16a *(required)* | q704_M16a. When purchased, how many units of this item typically come in one pack, box, bottle, etc.? (e.g. 100 tablets, 1000 ml's, etc.) (Write 1 if purchased as a single item.)<br>*Smallest units listed were: "[q204_M16c]" , "[q304_M16c]" , "[q404_M16c]" , "[q504_M16c]" , "[q604_M16c]"*<br>*Question relevant when: not(selected( ${q104_M16_full_list} , '6')) and not(selected( ${q104_M16_full_list} , '99'))*<br>*Response constrained to: .>=0* | |
| q704_M16a.1 *(required)* | q704_M16a.1 What is the typical size of each item in the pack, box, etc. (e.g. 1 litre bottles, 250 mg tablets, 3 ml ampoules, etc.)?<br>*Please provide as much information as is available.*<br>*Question relevant when: not(selected( ${q104_M16_full_list} , '6')) and not(selected( ${q104_M16_full_list} , '99'))* | |
| q704_M16b *(required)* | q704_M16b. What is the typical purchase price for that quantity of units (i.e. the quantity noted in the question above)?<br>*Question relevant when: not(selected( ${q104_M16_full_list} , '6')) and not(selected( ${q104_M16_full_list} , '99'))*<br>*Response constrained to: .>=0* | |
| q704_M16c *(required)* | q704_M16c. Please specify the currency for the purchase price.<br>*Question relevant when: not(selected( ${q104_M16_full_list} , '6')) and not(selected( ${q104_M16_full_list} , '99'))* | 1 Tanzanian Shillings<br>2 US dollars<br>3 Euros<br>4 Other<br>99 Don't know |
| q704_M16c.1 | q704_M16c.1 If other currency, specify:<br>*Leave blank if not applicable*<br>*Question relevant when: not(selected( ${q104_M16_full_list} , '6')) and not(selected( ${q104_M16_full_list} , '99'))* | |
| q704_M16d *(required)* | q704_M16d. Please specify the year of the purchase price.<br>*Question relevant when: not(selected( ${q104_M16_full_list} , '6')) and not(selected( ${q104_M16_full_list} , '99'))*<br>*Response constrained to: .>=2000 and .<=2019 or .=999* | |
| q704_M17 | ***q704_M17. Propofol (DiprVan), 10mg/ml***<br>*Question relevant when: not(selected( ${q104_M17_full_list} , '6')) and not(selected( ${q104_M17_full_list} , '99'))* | |
| q704_M17a *(required)* | q704_M17a. When purchased, how many units of this item typically come in one pack, box, bottle, etc.? (e.g. 100 tablets, 1000 ml's, etc.) (Write 1 if purchased as a single item.)<br>*Smallest units listed were: "[q204_M17c]" , "[q304_M17c]" , "[q404_M17c]" , "[q504_M17c]" , "[q604_M17c]"*<br>*Question relevant when: not(selected( ${q104_M17_full_list} , '6')) and not(selected( ${q104_M17_full_list} , '99'))*<br>*Response constrained to: .>=0* | |
| q704_M17a.1 *(required)* | q704_M17a.1 What is the typical size of each item in the pack, box, etc. (e.g. 1 litre bottles, 250 mg tablets, 3 ml | |

| Field | Question | Answer |
|---|---|---|
| q704_M17a.1 *(required)* | q704_M17a.1 What is the typical size of each item in the pack, box, etc. (e.g. 1 litre bottles, 250 mg tablets, 3 ml [illegible] etc.)?<br>*Please provide as much information as is available.* | |
| | *Question relevant when: not(selected( ${q104_M17_full_list} , '6')) and not(selected( ${q104_M17_full_list} , '99'))* | |
| q704_M17b *(required)* | q704_M17b. What is the typical purchase price for that quantity of units (i.e. the quantity noted in the question above)?<br>*Question relevant when: not(selected( ${q104_M17_full_list} , '6')) and not(selected( ${q104_M17_full_list} , '99'))*<br>*Response constrained to: .>=0* | |
| q704_M17c *(required)* | q704_M17c. Please specify the currency for the purchase price.<br>*Question relevant when: not(selected( ${q104_M17_full_list} , '6')) and not(selected( ${q104_M17_full_list} , '99'))* | 1 Tanzanian Shillings<br>2 US dollars<br>3 Euros<br>4 Other<br>99 Don't know |
| q704_M17c.1 | q704_M17c.1 If other currency, specify:<br>*Leave blank if not applicable*<br>*Question relevant when: not(selected( ${q104_M17_full_list} , '6')) and not(selected( ${q104_M17_full_list} , '99'))* | |
| q704_M17d *(required)* | q704_M17d. Please specify the year of the purchase price.<br>*Question relevant when: not(selected( ${q104_M17_full_list} , '6')) and not(selected( ${q104_M17_full_list} , '99'))*<br>*Response constrained to: .>=2000 and .<=2019 or .=999* | |
| q704_M18 | ***q704_M18. Suxamethonium chloride***<br>*Question relevant when: not(selected( ${q104_M18_full_list} , '6')) and not(selected( ${q104_M18_full_list} , '99'))* | |
| q704_M18a *(required)* | q704_M18a. When purchased, how many units of this item typically come in one pack, box, bottle, etc.? (e.g. 100 tablets, 1000 ml's, etc.) (Write 1 if purchased as a single item.)<br>*Smallest units listed were: "[q204_M18c]" , "[q304_M18c]" , "[q404_M18c]" , "[q504_M18c]" , "[q604_M18c]"*<br>*Question relevant when: not(selected( ${q104_M18_full_list} , '6')) and not(selected( ${q104_M18_full_list} , '99'))*<br>*Response constrained to: .>=0* | |
| q704_M18a.1 *(required)* | q704_M18a.1 What is the typical size of each item in the pack, box, etc. (e.g. 1 litre bottles, 250 mg tablets, 3 ml ampoules, etc.)?<br>*Please provide as much information as is available.*<br>*Question relevant when: not(selected( ${q104_M18_full_list} , '6')) and not(selected( ${q104_M18_full_list} , '99'))* | |
| q704_M18b *(required)* | q704_M18b. What is the typical purchase price for that quantity of units (i.e. the quantity noted in the question above)?<br>*Question relevant when: not(selected( ${q104_M18_full_list} , '6')) and not(selected( ${q104_M18_full_list} , '99'))*<br>*Response constrained to: .>=0* | |
| q704_M18c *(required)* | q704_M18c. Please specify the currency for the purchase price.<br>*Question relevant when: not(selected( ${q104_M18_full_list} , '6')) and not(selected( ${q104_M18_full_list} , '99'))* | 1 Tanzanian Shillings<br>2 US dollars<br>3 Euros<br>4 Other<br>99 Don't know |
| q704_M18c.1 | q704_M18c.1 If other currency, specify:<br>*Leave blank if not applicable*<br>*Question relevant when: not(selected( ${q104_M18_full_list} , '6')) and not(selected( ${q104_M18_full_list} , '99'))* | |
| q704_M18d *(required)* | q704_M18d. Please specify the year of the purchase price.<br>*Question relevant when: not(selected( ${q104_M18_full_list} , '6')) and not(selected( ${q104_M18_full_list} , '99'))*<br>*Response constrained to: .>=2000 and .<=2019 or .=999* | |
| q704_M19 | ***q704_M19. Thiopental sodium (Intraval)***<br>*Question relevant when: not(selected( ${q104_M19_full_list} , '6')) and not(selected( ${q104_M19_full_list} , '99'))* | |
| q704_M19a *(required)* | q704_M19a. When purchased, how many units of this item typically come in one pack, box, bottle, etc.? (e.g. 100 tablets, 1000 ml's, etc.) (Write 1 if purchased as a single item.)<br>*Smallest units listed were: "[q204_M19c]" , "[q304_M19c]" , "[q404_M19c]" , "[q504_M19c]" , "[q604_M19c]"*<br>*Question relevant when: not(selected( ${q104_M19_full_list} , '6')) and not(selected( ${q104_M19_full_list} , '99'))*<br>*Response constrained to: .>=0* | |
| q704_M19a.1 *(required)* | q704_M19a.1 What is the typical size of each item in the pack, box, etc. (e.g. 1 litre bottles, 250 mg tablets, 3 ml | |

| Field | Question | Answer |
|---|---|---|
| q704_M19a.1 *(required)* | q704_M19a.1 [illegible] ampoules, etc.)?<br>*Please provide as much information as is available.*<br>*Question relevant when: not(selected( ${q104_M19_full_list} , '6')) and not(selected( ${q104_M19_full_list} , '99'))* | |
| q704_M19b *(required)* | q704_M19b. What is the typical purchase price for that quantity of units (i.e. the quantity noted in the question above)?<br>*Question relevant when: not(selected( ${q104_M19_full_list} , '6')) and not(selected( ${q104_M19_full_list} , '99'))*<br>*Response constrained to: .>=0* | |
| q704_M19c *(required)* | q704_M19c. Please specify the currency for the purchase price.<br>*Question relevant when: not(selected( ${q104_M19_full_list} , '6')) and not(selected( ${q104_M19_full_list} , '99'))* | 1 Tanzanian Shillings<br>2 US dollars<br>3 Euros<br>4 Other<br>99 Don't know |
| q704_M19c.1 | q704_M19c.1 If other currency, specify:<br>*Leave blank if not applicable*<br>*Question relevant when: not(selected( ${q104_M19_full_list} , '6')) and not(selected( ${q104_M19_full_list} , '99'))* | |
| q704_M19d *(required)* | q704_M19d. Please specify the year of the purchase price.<br>*Question relevant when: not(selected( ${q104_M19_full_list} , '6')) and not(selected( ${q104_M19_full_list} , '99'))*<br>*Response constrained to: .>=2000 and .<=2019 or .=999* | |
| q704_M20 | ***q704_M20. Oxygen***<br>*Question relevant when: not(selected( ${q104_M20_full_list} , '6')) and not(selected( ${q104_M20_full_list} , '99'))* | |
| q704_M20a *(required)* | q704_M20a. When purchased, how many units of this item typically come in one pack, box, bottle, etc.? (e.g. 100 tablets, 1000 ml's, etc.) (Write 1 if purchased as a single item.)<br>*Smallest units listed were: "[q204_M20c]" , "[q304_M20c]" , "[q404_M20c]" , "[q504_M20c]" , "[q604_M20c]"*<br>*Question relevant when: not(selected( ${q104_M20_full_list} , '6')) and not(selected( ${q104_M20_full_list} , '99'))*<br>*Response constrained to: .>=0* | |
| q704_M20a.1 *(required)* | q704_M20a.1 What is the typical size of each item in the pack, box, etc. (e.g. 1 litre bottles, 250 mg tablets, 3 ml ampoules, etc.)?<br>*Please provide as much information as is available.*<br>*Question relevant when: not(selected( ${q104_M20_full_list} , '6')) and not(selected( ${q104_M20_full_list} , '99'))* | |
| q704_M20b *(required)* | q704_M20b. What is the typical purchase price for that quantity of units (i.e. the quantity noted in the question above)?<br>*Question relevant when: not(selected( ${q104_M20_full_list} , '6')) and not(selected( ${q104_M20_full_list} , '99'))*<br>*Response constrained to: .>=0* | |
| q704_M20c *(required)* | q704_M20c. Please specify the currency for the purchase price.<br>*Question relevant when: not(selected( ${q104_M20_full_list} , '6')) and not(selected( ${q104_M20_full_list} , '99'))* | 1 Tanzanian Shillings<br>2 US dollars<br>3 Euros<br>4 Other<br>99 Don't know |
| q704_M20c.1 | q704_M20c.1 If other currency, specify:<br>*Leave blank if not applicable*<br>*Question relevant when: not(selected( ${q104_M20_full_list} , '6')) and not(selected( ${q104_M20_full_list} , '99'))* | |
| q704_M20d *(required)* | q704_M20d. Please specify the year of the purchase price.<br>*Question relevant when: not(selected( ${q104_M20_full_list} , '6')) and not(selected( ${q104_M20_full_list} , '99'))*<br>*Response constrained to: .>=2000 and .<=2019 or .=999* | |
| q704_M21 | ***q704_M21. Bupivacaine HCL 0.5% (Marcaine)***<br>*Question relevant when: not(selected( ${q104_M21_full_list} , '6')) and not(selected( ${q104_M21_full_list} , '99'))* | |
| q704_M21a *(required)* | q704_M21a. When purchased, how many units of this item typically come in one pack, box, bottle, etc.? (e.g. 100 tablets, 1000 ml's, etc.) (Write 1 if purchased as a single item.)<br>*Smallest units listed were: "[q204_M21c]" , "[q304_M21c]" , "[q404_M21c]" , "[q504_M21c]" , "[q604_M21c]"*<br>*Question relevant when: not(selected( ${q104_M21_full_list} , '6')) and not(selected( ${q104_M21_full_list} , '99'))*<br>*Response constrained to: .>=0* | |

| Field | Question | Answer |
|---|---|---|
| q704_M21a.1 *(required)* | q704_M21a.1 What is the typical size of each item in the pack, box, etc. (e.g. 1 litre bottles, 250 mg tablets, 3 ml ampoules, etc.)?<br>*Please provide as much information as is available.*<br>*Question relevant when: not(selected( ${q104_M21_full_list} , '6')) and not(selected( ${q104_M21_full_list} , '99'))* | |
| q704_M21b *(required)* | q704_M21b. What is the typical purchase price for that quantity of units (i.e. the quantity noted in the question above)?<br>*Question relevant when: not(selected( ${q104_M21_full_list} , '6')) and not(selected( ${q104_M21_full_list} , '99'))*<br>*Response constrained to: .>=0* | |
| q704_M21c *(required)* | q704_M21c. Please specify the currency for the purchase price.<br>*Question relevant when: not(selected( ${q104_M21_full_list} , '6')) and not(selected( ${q104_M21_full_list} , '99'))* | 1 Tanzanian Shillings<br>2 US dollars<br>3 Euros<br>4 Other<br>99 Don't know |
| q704_M21c.1 | q704_M21c.1 If other currency, specify:<br>*Leave blank if not applicable*<br>*Question relevant when: not(selected( ${q104_M21_full_list} , '6')) and not(selected( ${q104_M21_full_list} , '99'))* | |

| Field | Question | Answer |
|---|---|---|
| q704_M21d *(required)* | q704_M21d. Please specify the year of the purchase price.<br>*Question relevant when: not(selected( ${q104_M21_full_list} , '6')) and not(selected( ${q104_M21_full_list} , '99'))*<br>*Response constrained to: .>=2000 and .<=2019 or .=999* | |
| q704_M22 | ***q704_M22. Lidocaine HCl 2% + epinephrin 1:100,000***<br>*Question relevant when: not(selected( ${q104_M22_full_list} , '6')) and not(selected( ${q104_M22_full_list} , '99'))* | |
| q704_M22a *(required)* | q704_M22a. When purchased, how many units of this item typically come in one pack, box, bottle, etc.? (e.g. 100 tablets, 1000 ml's, etc.) (Write 1 if purchased as a single item.)<br>*Smallest units listed were: "[q204_M22c]" , "[q304_M22c]" , "[q404_M22c]" , "[q504_M22c]" , "[q604_M22c]"*<br>*Question relevant when: not(selected( ${q104_M22_full_list} , '6')) and not(selected( ${q104_M22_full_list} , '99'))*<br>*Response constrained to: .>=0* | |
| q704_M22a.1 *(required)* | q704_M22a.1 What is the typical size of each item in the pack, box, etc. (e.g. 1 litre bottles, 250 mg tablets, 3 ml ampoules, etc.)?<br>*Please provide as much information as is available.*<br>*Question relevant when: not(selected( ${q104_M22_full_list} , '6')) and not(selected( ${q104_M22_full_list} , '99'))* | |
| q704_M22b *(required)* | q704_M22b. What is the typical purchase price for that quantity of units (i.e. the quantity noted in the question above)?<br>*Question relevant when: not(selected( ${q104_M22_full_list} , '6')) and not(selected( ${q104_M22_full_list} , '99'))*<br>*Response constrained to: .>=0* | |
| q704_M22c *(required)* | q704_M22c. Please specify the currency for the purchase price.<br>*Question relevant when: not(selected( ${q104_M22_full_list} , '6')) and not(selected( ${q104_M22_full_list} , '99'))* | 1 Tanzanian Shillings<br>2 US dollars<br>3 Euros<br>4 Other<br>99 Don't know |
| q704_M22c.1 | q704_M22c.1 If other currency, specify:<br>*Leave blank if not applicable*<br>*Question relevant when: not(selected( ${q104_M22_full_list} , '6')) and not(selected( ${q104_M22_full_list} , '99'))* | |
| q704_M22d *(required)* | q704_M22d. Please specify the year of the purchase price.<br>*Question relevant when: not(selected( ${q104_M22_full_list} , '6')) and not(selected( ${q104_M22_full_list} , '99'))*<br>*Response constrained to: .>=2000 and .<=2019 or .=999* | |
| q704_M23 | ***q704_M23. Lidocaine HCl 5% + dextrose 7.5%***<br>*Question relevant when: not(selected( ${q104_M23_full_list} , '6')) and not(selected( ${q104_M23_full_list} , '99'))* | |
| q704_M23a *(required)* | q704_M23a. When purchased, how many units of this item typically come in one pack, box, bottle, etc.? (e.g. 100 tablets, 1000 ml's, etc.) (Write 1 if purchased as a single item.)<br>*Smallest units listed were: "[q204_M23c]" , "[q304_M23c]" , "[q404_M23c]" , "[q504_M23c]" , "[q604_M23c]"*<br>*Question relevant when: not(selected( ${q104_M23_full_list} , '6')) and not(selected( ${q104_M23_full_list} , '99'))*<br>*Response constrained to: .>=0* | |
| q704_M23a.1 *(required)* | q704_M23a.1 What is the typical size of each item in the pack, box, etc. (e.g. 1 litre bottles, 250 mg tablets, 3 ml | |

| Field | Question | Answer |
|---|---|---|
| q704_M23a.1 *(required)* | q704_M23a.1 What is the typical size of each item in the pack, box, etc. (e.g. 1 litre bottles, 250 mg tablets, 3 ml ampoules, etc.)?<br>*Please provide as much information as is available.*<br>*Question relevant when: not(selected( ${q104_M23_full_list} , '6')) and not(selected( ${q104_M23_full_list} , '99'))* | |
| q704_M23b *(required)* | q704_M23b. What is the typical purchase price for that quantity of units (i.e. the quantity noted in the question above)?<br>*Question relevant when: not(selected( ${q104_M23_full_list} , '6')) and not(selected( ${q104_M23_full_list} , '99'))*<br>*Response constrained to: .>=0* | |
| q704_M23c *(required)* | q704_M23c. Please specify the currency for the purchase price.<br>*Question relevant when: not(selected( ${q104_M23_full_list} , '6')) and not(selected( ${q104_M23_full_list} , '99'))* | 1 Tanzanian Shillings<br>2 US dollars<br>3 Euros<br>4 Other<br>99 Don't know |
| q704_M23c.1 | q704_M23c.1 If other currency, specify:<br>*Leave blank if not applicable*<br>*Question relevant when: not(selected( ${q104_M23_full_list} , '6')) and not(selected( ${q104_M23_full_list} , '99'))* | |
| q704_M23d *(required)* | q704_M23d. Please specify the year of the purchase price.<br>*Question relevant when: not(selected( ${q104_M23_full_list} , '6')) and not(selected( ${q104_M23_full_list} , '99'))*<br>*Response constrained to: .>=2000 and .<=2019 or .=999* | |
| q704_M24 | ***q704_M24. Lidocaine HCl, 1%***<br>*Question relevant when: not(selected( ${q104_M24_full_list} , '6')) and not(selected( ${q104_M24_full_list} , '99'))* | |
| q704_M24a *(required)* | q704_M24a. When purchased, how many units of this item typically come in one pack, box, bottle, etc.? (e.g. 100 tablets, 1000 ml's, etc.) (Write 1 if purchased as a single item.)<br>*Smallest units listed were: "[q204_M24c]" , "[q304_M24c]" , "[q404_M24c]" , "[q504_M24c]" , "[q604_M24c]"*<br>*Question relevant when: not(selected( ${q104_M24_full_list} , '6')) and not(selected( ${q104_M24_full_list} , '99'))*<br>*Response constrained to: .>=0* | |
| q704_M24a.1 *(required)* | q704_M24a.1 What is the typical size of each item in the pack, box, etc. (e.g. 1 litre bottles, 250 mg tablets, 3 ml ampoules, etc.)?<br>*Please provide as much information as is available.*<br>*Question relevant when: not(selected( ${q104_M24_full_list} , '6')) and not(selected( ${q104_M24_full_list} , '99'))* | |
| q704_M24b *(required)* | q704_M24b. What is the typical purchase price for that quantity of units (i.e. the quantity noted in the question above)?<br>*Question relevant when: not(selected( ${q104_M24_full_list} , '6')) and not(selected( ${q104_M24_full_list} , '99'))*<br>*Response constrained to: .>=0* | |
| q704_M24c *(required)* | q704_M24c. Please specify the currency for the purchase price.<br>*Question relevant when: not(selected( ${q104_M24_full_list} , '6')) and not(selected( ${q104_M24_full_list} , '99'))* | 1 Tanzanian Shillings<br>2 US dollars<br>3 Euros<br>4 Other<br>99 Don't know |
| q704_M24c.1 | q704_M24c.1 If other currency, specify:<br>*Leave blank if not applicable*<br>*Question relevant when: not(selected( ${q104_M24_full_list} , '6')) and not(selected( ${q104_M24_full_list} , '99'))* | |
| q704_M24d *(required)* | q704_M24d. Please specify the year of the purchase price.<br>*Question relevant when: not(selected( ${q104_M24_full_list} , '6')) and not(selected( ${q104_M24_full_list} , '99'))*<br>*Response constrained to: .>=2000 and .<=2019 or .=999* | |
| q704_M25 | ***q704_M25. Anesthetic - other 1: "[q104_M25_full_list_other]"***<br>*Question relevant when: not(selected( ${q104_M25_full_list} , '6')) and not(selected( ${q104_M25_full_list} , '99')) and string-length( ${q104_M25_full_list_other} ) >0* | |
| q704_M25a *(required)* | q704_M25a. When purchased, how many units of this item typically come in one pack, box, bottle, etc.? (e.g. 100 tablets, 1000 ml's, etc.) (Write 1 if purchased as a single item.)<br>*Smallest units listed were: "[q204_M25c]" , "[q304_M25c]" , "[q404_M25c]" , "[q504_M25c]" , "[q604_M25c]"*<br>*Question relevant when: not(selected( ${q104_M25_full_list} , '6')) and not(selected( ${q104_M25_full_list} , '99')) and string-length( ${q104_M25_full_list_other} ) >0*<br>*Response constrained to: .>=0* | |
| q704_M25a.1 *(required)* | q704_M25a.1 What is the typical size of each item in the pack, box, etc. (e.g. 1 litre bottles, 250 mg tablets, 3 ml | |

| Field | Question | Answer |
|---|---|---|
| q704_M25a.1 *(required)* | q704_M25a.1 What is the typical size of each item in the pack, box, etc. (e.g. 1 litre bottles, 250 mg tablets, 3 ml ampoules, etc.)?<br>*Please provide as much information as is available.*<br>*Question relevant when: not(selected( ${q104_M25_full_list} , '6')) and not(selected( ${q104_M25_full_list} , '99')) and string-length( ${q104_M25_full_list_other} ) >0* | |
| q704_M25b *(required)* | q704_M25b. What is the typical purchase price for that quantity of units (i.e. the quantity noted in the question above)?<br>*Question relevant when: not(selected( ${q104_M25_full_list} , '6')) and not(selected( ${q104_M25_full_list} , '99')) and string-length( ${q104_M25_full_list_other} ) >0*<br>*Response constrained to: .>=0* | |
| q704_M25c *(required)* | q704_M25c. Please specify the currency for the purchase price.<br>*Question relevant when: not(selected( ${q104_M25_full_list} , '6')) and not(selected( ${q104_M25_full_list} , '99')) and string-length( ${q104_M25_full_list_other} ) >0* | 1 Tanzanian Shillings<br>2 US dollars<br>3 Euros<br>4 Other<br>99 Don't know |
| q704_M25c.1 | q704_M25c.1 If other currency, specify:<br>*Leave blank if not applicable*<br>*Question relevant when: not(selected( ${q104_M25_full_list} , '6')) and not(selected( ${q104_M25_full_list} , '99')) and string-length( ${q104_M25_full_list_other} ) >0* | |

| | | |
|---|---|---|
| q704_M25d *(required)* | q704_M25d. Please specify the year of the purchase price.<br>*Question relevant when: not(selected( ${q104_M25_full_list} , '6')) and not(selected( ${q104_M25_full_list} , '99')) and string-length( ${q104_M25_full_list_other} ) >0*<br>*Response constrained to: .>=2000 and .<=2019 or .=999* | |
| q704_M26 | ***q704_M26. Anesthetic - other 2: "[q104_M26_full_list_other]"***<br>*Question relevant when: not(selected( ${q104_M26_full_list} , '6')) and not(selected( ${q104_M26_full_list} , '99')) and string-length( ${q104_M26_full_list_other} ) >0* | |
| q704_M26a *(required)* | q704_M26a. When purchased, how many units of this item typically come in one pack, box, bottle, etc.? (e.g. 100 tablets, 1000 ml's, etc.) (Write 1 if purchased as a single item.)<br>*Smallest units listed were: "[q204_M26c]" , "[q304_M26c]" , "[q404_M26c]" , "[q504_M26c]" , "[q604_M26c]"*<br>*Question relevant when: not(selected( ${q104_M26_full_list} , '6')) and not(selected( ${q104_M26_full_list} , '99')) and string-length( ${q104_M26_full_list_other} ) >0*<br>*Response constrained to: .>=0* | |
| q704_M26a.1 *(required)* | q704_M26a.1 What is the typical size of each item in the pack, box, etc. (e.g. 1 litre bottles, 250 mg tablets, 3 ml ampoules, etc.)?<br>*Please provide as much information as is available.*<br>*Question relevant when: not(selected( ${q104_M26_full_list} , '6')) and not(selected( ${q104_M26_full_list} , '99')) and string-length( ${q104_M26_full_list_other} ) >0* | |
| q704_M26b *(required)* | q704_M26b. What is the typical purchase price for that quantity of units (i.e. the quantity noted in the question above)?<br>*Question relevant when: not(selected( ${q104_M26_full_list} , '6')) and not(selected( ${q104_M26_full_list} , '99')) and string-length( ${q104_M26_full_list_other} ) >0*<br>*Response constrained to: .>=0* | |
| q704_M26c *(required)* | q704_M26c. Please specify the currency for the purchase price.<br>*Question relevant when: not(selected( ${q104_M26_full_list} , '6')) and not(selected( ${q104_M26_full_list} , '99')) and string-length( ${q104_M26_full_list_other} ) >0* | 1 Tanzanian Shillings<br>2 US dollars<br>3 Euros<br>4 Other<br>99 Don't know |
| q704_M26c.1 | q704_M26c.1 If other currency, specify:<br>*Leave blank if not applicable*<br>*Question relevant when: not(selected( ${q104_M26_full_list} , '6')) and not(selected( ${q104_M26_full_list} , '99')) and string-length( ${q104_M26_full_list_other} ) >0* | |
| q704_M26d *(required)* | q704_M26d. Please specify the year of the purchase price.<br>*Question relevant when: not(selected( ${q104_M26_full_list} , '6')) and not(selected( ${q104_M26_full_list} , '99')) and string-length( ${q104_M26_full_list_other} ) >0*<br>*Response constrained to: .>=2000 and .<=2019 or .=999* | |
| q704_M27 | ***q704_M27. Anesthetic - other 3: "[q104_M27_full_list_other]"***<br>*Question relevant when: not(selected( ${q104_M27_full_list} , '6')) and not(selected( ${q104_M27_full_list} , '99')) and string-length( ${q104_M27_full_list_other} ) >0* | |
| q704_M27a *(required)* | q704_M27a. When purchased, how many units of this item typically come in one pack, box, bottle, etc.? (e.g. 100 tablets, 1000 ml's, etc.) (Write 1 if purchased as a single item.)<br>*Smallest units listed were: "[q204_M27c]" , "[q304_M27c]" , "[q404_M27c]" , "[q504_M27c]" , "[q604_M27c]"*<br>*Question relevant when: not(selected( ${q104_M27_full_list} , '6')) and not(selected( ${q104_M27_full_list} , '99')) and string-length( ${q104_M27_full_list_other} ) >0*<br>*Response constrained to: .>=0* | |
| q704_M27a.1 *(required)* | q704_M27a.1 What is the typical size of each item in the pack, box, etc. (e.g. 1 litre bottles, 250 mg tablets, 3 ml | |

| Field | Question | Answer |
|---|---|---|
| q704_M27a.1 *(required)* | q704_M27a.1 What is the typical size of each item in the pack, box, etc. (e.g. 1 litre bottles, 250 mg tablets, 3 ml ampoules, etc.)?<br>*Please provide as much information as is available.* | |
| | *Question relevant when: not(selected( ${q104_M27_full_list} , '6')) and not(selected( ${q104_M27_full_list} , '99')) and string-length( ${q104_M27_full_list_other} ) >0* | |
| q704_M27b *(required)* | q704_M27b. What is the typical purchase price for that quantity of units (i.e. the quantity noted in the question above)?<br>*Question relevant when: not(selected( ${q104_M27_full_list} , '6')) and not(selected( ${q104_M27_full_list} , '99')) and string-length( ${q104_M27_full_list_other} ) >0*<br>*Response constrained to: .>=0* | |
| q704_M27c *(required)* | q704_M27c. Please specify the currency for the purchase price.<br>*Question relevant when: not(selected( ${q104_M27_full_list} , '6')) and not(selected( ${q104_M27_full_list} , '99')) and string-length( ${q104_M27_full_list_other} ) >0* | 1 Tanzanian Shillings<br>2 US dollars<br>3 Euros<br>4 Other<br>99 Don't know |
| q704_M27c.1 | q704_M27c.1 If other currency, specify:<br>*Leave blank if not applicable*<br>*Question relevant when: not(selected( ${q104_M27_full_list} , '6')) and not(selected( ${q104_M27_full_list} , '99')) and string-length( ${q104_M27_full_list_other} ) >0* | |

| | | |
|---|---|---|
| q704_M27d *(required)* | q704_M27d. Please specify the year of the purchase price.<br>*Question relevant when: not(selected( ${q104_M27_full_list} , '6')) and not(selected( ${q104_M27_full_list} , '99')) and string-length( ${q104_M27_full_list_other} ) >0*<br>*Response constrained to: .>=2000 and .<=2019 or .=999* | |
| q704_M28 | ***q704_M28. Anesthetic - other 4: "[q104_M28_full_list_other]"***<br>*Question relevant when: not(selected( ${q104_M28_full_list} , '6')) and not(selected( ${q104_M28_full_list} , '99')) and string-length( ${q104_M28_full_list_other} ) >0* | |
| q704_M28a *(required)* | q704_M28a. When purchased, how many units of this item typically come in one pack, box, bottle, etc.? (e.g. 100 tablets, 1000 ml's, etc.) (Write 1 if purchased as a single item.)<br>*Smallest units listed were: "[q204_M28c]" , "[q304_M28c]" , "[q404_M28c]" , "[q504_M28c]" , "[q604_M28c]"*<br>*Question relevant when: not(selected( ${q104_M28_full_list} , '6')) and not(selected( ${q104_M28_full_list} , '99')) and string-length( ${q104_M28_full_list_other} ) >0*<br>*Response constrained to: .>=0* | |
| q704_M28a.1 *(required)* | q704_M28a.1 What is the typical size of each item in the pack, box, etc. (e.g. 1 litre bottles, 250 mg tablets, 3 ml ampoules, etc.)?<br>*Please provide as much information as is available.*<br>*Question relevant when: not(selected( ${q104_M28_full_list} , '6')) and not(selected( ${q104_M28_full_list} , '99')) and string-length( ${q104_M28_full_list_other} ) >0* | |
| q704_M28b *(required)* | q704_M28b. What is the typical purchase price for that quantity of units (i.e. the quantity noted in the question above)?<br>*Question relevant when: not(selected( ${q104_M28_full_list} , '6')) and not(selected( ${q104_M28_full_list} , '99')) and string-length( ${q104_M28_full_list_other} ) >0*<br>*Response constrained to: .>=0* | |
| q704_M28c *(required)* | q704_M28c. Please specify the currency for the purchase price.<br>*Question relevant when: not(selected( ${q104_M28_full_list} , '6')) and not(selected( ${q104_M28_full_list} , '99')) and string-length( ${q104_M28_full_list_other} ) >0* | 1 Tanzanian Shillings<br>2 US dollars<br>3 Euros<br>4 Other<br>99 Don't know |
| q704_M28c.1 | q704_M28c.1 If other currency, specify:<br>*Leave blank if not applicable*<br>*Question relevant when: not(selected( ${q104_M28_full_list} , '6')) and not(selected( ${q104_M28_full_list} , '99')) and string-length( ${q104_M28_full_list_other} ) >0* | |
| q704_M28d *(required)* | q704_M28d. Please specify the year of the purchase price.<br>*Question relevant when: not(selected( ${q104_M28_full_list} , '6')) and not(selected( ${q104_M28_full_list} , '99')) and string-length( ${q104_M28_full_list_other} ) >0*<br>*Response constrained to: .>=2000 and .<=2019 or .=999* | |
| q704_M29 | ***q704_M29. Anesthetic - other 5: "[q104_M29_full_list_other]"***<br>*Question relevant when: not(selected( ${q104_M29_full_list} , '6')) and not(selected( ${q104_M29_full_list} , '99')) and string-length( ${q104_M29_full_list_other} ) >0* | |
| q704_M29a *(required)* | q704_M29a. When purchased, how many units of this item typically come in one pack, box, bottle, etc.? (e.g. 100 tablets, 1000 ml's, etc.) (Write 1 if purchased as a single item.)<br>*Smallest units listed were: "[q204_M29c]" , "[q304_M29c]" , "[q404_M29c]" , "[q504_M29c]" , "[q604_M29c]"*<br>*Question relevant when: not(selected( ${q104_M29_full_list} , '6')) and not(selected( ${q104_M29_full_list} , '99')) and string-length( ${q104_M29_full_list_other} ) >0*<br>*Response constrained to: .>=0* | |
| q704_M29a.1 *(required)* | q704_M29a.1 What is the typical size of each item in the pack, box, etc. (e.g. 1 litre bottles, 250 mg tablets, 3 ml | |

| Field | Question | Answer |
|---|---|---|
| q704_M29a.1 *(required)* | q704_M29a.1 What is the typical size of each item in the pack, box, etc. (e.g. 1 litre bottles, 250 mg tablets, 3 ml ampoules, etc.)?<br>*Please provide as much information as is available.* | |
| | *Question relevant when: not(selected( ${q104_M29_full_list} , '6')) and not(selected( ${q104_M29_full_list} , '99')) and string-length( ${q104_M29_full_list_other} ) >0* | |
| q704_M29b *(required)* | q704_M29b. What is the typical purchase price for that quantity of units (i.e. the quantity noted in the question above)?<br>*Question relevant when: not(selected( ${q104_M29_full_list} , '6')) and not(selected( ${q104_M29_full_list} , '99')) and string-length( ${q104_M29_full_list_other} ) >0*<br>*Response constrained to: .>=0* | |
| q704_M29c *(required)* | q704_M29c. Please specify the currency for the purchase price.<br>*Question relevant when: not(selected( ${q104_M29_full_list} , '6')) and not(selected( ${q104_M29_full_list} , '99')) and string-length( ${q104_M29_full_list_other} ) >0* | 1 Tanzanian Shillings<br>2 US dollars<br>3 Euros<br>4 Other<br>99 Don't know |
| q704_M29c.1 | q704_M29c.1 If other currency, specify:<br>*Leave blank if not applicable*<br>*Question relevant when: not(selected( ${q104_M29_full_list} , '6')) and not(selected( ${q104_M29_full_list} , '99')) and string-length( ${q104_M29_full_list_other} ) >0* | |

| | | |
|---|---|---|
| q704_M29d *(required)* | q704_M29d. Please specify the year of the purchase price.<br>*Question relevant when: not(selected( ${q104_M29_full_list} , '6')) and not(selected( ${q104_M29_full_list} , '99')) and string-length( ${q104_M29_full_list_other} ) >0*<br>*Response constrained to: .>=2000 and .<=2019 or .=999* | |
| D. Medications - Cost (3)<br>*Group relevant when: selected( ${section_seven_skip_med} , '1')* | | |
| q704_med_note3 | 704 CONTINUED For each of the following medications and other medical products that are used for postabortion care, can you tell me …? | |
| note_704_antibiotics | ***Antibiotics*** | |
| q704_M30 | ***q704_M30. Amoxicillin***<br>*Question relevant when: not(selected( ${q104_M30_full_list} , '6')) and not(selected( ${q104_M30_full_list} , '99'))* | |
| q704_M30a *(required)* | q704_M30a. When purchased, how many units of this item typically come in one pack, box, bottle, etc.? (e.g. 100 tablets, 1000 ml's, etc.) (Write 1 if purchased as a single item.)<br>*Smallest units listed were: "[q204_M30c]" , "[q304_M30c]" , "[q404_M30c]" , "[q504_M30c]" , "[q604_M30c]"*<br>*Question relevant when: not(selected( ${q104_M30_full_list} , '6')) and not(selected( ${q104_M30_full_list} , '99'))*<br>*Response constrained to: .>=0* | |
| q704_M30a.1 *(required)* | q704_M30a.1 What is the typical size of each item in the pack, box, etc. (e.g. 1 litre bottles, 250 mg tablets, 3 ml ampoules, etc.)?<br>*Please provide as much information as is available.*<br>*Question relevant when: not(selected( ${q104_M30_full_list} , '6')) and not(selected( ${q104_M30_full_list} , '99'))* | |
| q704_M30b *(required)* | q704_M30b. What is the typical purchase price for that quantity of units (i.e. the quantity noted in the question above)?<br>*Question relevant when: not(selected( ${q104_M30_full_list} , '6')) and not(selected( ${q104_M30_full_list} , '99'))*<br>*Response constrained to: .>=0* | |
| q704_M30c *(required)* | q704_M30c. Please specify the currency for the purchase price.<br>*Question relevant when: not(selected( ${q104_M30_full_list} , '6')) and not(selected( ${q104_M30_full_list} , '99'))* | 1 Tanzanian Shillings<br>2 US dollars<br>3 Euros<br>4 Other<br>99 Don't know |
| q704_M30c.1 | q704_M30c.1 If other currency, specify:<br>*Leave blank if not applicable*<br>*Question relevant when: not(selected( ${q104_M30_full_list} , '6')) and not(selected( ${q104_M30_full_list} , '99'))* | |
| q704_M30d *(required)* | q704_M30d. Please specify the year of the purchase price.<br>*Question relevant when: not(selected( ${q104_M30_full_list} , '6')) and not(selected( ${q104_M30_full_list} , '99'))*<br>*Response constrained to: .>=2000 and .<=2019 or .=999* | |
| q704_M31 | ***q704_M31. Amoxicillin + Clauvulenic Acid 635mg***<br>*Question relevant when: not(selected( ${q104_M31_full_list} , '6')) and not(selected( ${q104_M31_full_list} , '99'))* | |
| q704_M31a *(required)* | q704_M31a. When purchased, how many units of this item typically come in one pack, box, bottle, etc.? (e.g. 100 | |

| Field | Question | Answer |
|---|---|---|
| | tablets, 1000 ml's, etc.) (Write 1 if purchased as a single item.)<br>*Smallest units listed were: "[q204_M31c]" , "[q304_M31c]" , "[q404_M31c]" , "[q504_M31c]" , "[q604_M31c]"*<br>*Question relevant when: not(selected( ${q104_M31_full_list} , '6')) and not(selected( ${q104_M31_full_list} , '99'))*<br>*Response constrained to: .>=0* | |
| q704_M31a.1 *(required)* | q704_M31a.1 What is the typical size of each item in the pack, box, etc. (e.g. 1 litre bottles, 250 mg tablets, 3 ml ampoules, etc.)?<br>*Please provide as much information as is available.*<br>*Question relevant when: not(selected( ${q104_M31_full_list} , '6')) and not(selected( ${q104_M31_full_list} , '99'))* | |
| q704_M31b *(required)* | q704_M31b. What is the typical purchase price for that quantity of units (i.e. the quantity noted in the question above)?<br>*Question relevant when: not(selected( ${q104_M31_full_list} , '6')) and not(selected( ${q104_M31_full_list} , '99'))*<br>*Response constrained to: .>=0* | |
| q704_M31c *(required)* | q704_M31c. Please specify the currency for the purchase price.<br>*Question relevant when: not(selected( ${q104_M31_full_list} , '6')) and not(selected( ${q104_M31_full_list} , '99'))* | 1 Tanzanian Shillings<br>2 US dollars<br>3 Euros<br>4 Other<br>99 Don't know |
| q704_M31c.1 | q704_M31c.1 If other currency, specify:<br>*Leave blank if not applicable*<br>*Question relevant when: not(selected( ${q104_M31_full_list} , '6')) and not(selected( ${q104_M31_full_list} , '99'))* | |
| q704_M31d *(required)* | q704_M31d. Please specify the year of the purchase price.<br>*Question relevant when: not(selected( ${q104_M31_full_list} , '6')) and not(selected( ${q104_M31_full_list} , '99'))*<br>*Response constrained to: .>=2000 and .<=2019 or .=999* | |
| q704_M32 | ***q704_M32. Ampicillin***<br>*Question relevant when: not(selected( ${q104_M32_full_list} , '6')) and not(selected( ${q104_M32_full_list} , '99'))* | |
| q704_M32a *(required)* | q704_M32a. When purchased, how many units of this item typically come in one pack, box, bottle, etc.? (e.g. 100 tablets, 1000 ml's, etc.) (Write 1 if purchased as a single item.)<br>*Smallest units listed were: "[q204_M32c]" , "[q304_M32c]" , "[q404_M32c]" , "[q504_M32c]" , "[q604_M32c]"*<br>*Question relevant when: not(selected( ${q104_M32_full_list} , '6')) and not(selected( ${q104_M32_full_list} , '99'))*<br>*Response constrained to: .>=0* | |
| q704_M32a.1 *(required)* | q704_M32a.1 What is the typical size of each item in the pack, box, etc. (e.g. 1 litre bottles, 250 mg tablets, 3 ml ampoules, etc.)?<br>*Please provide as much information as is available.*<br>*Question relevant when: not(selected( ${q104_M32_full_list} , '6')) and not(selected( ${q104_M32_full_list} , '99'))* | |
| q704_M32b *(required)* | q704_M32b. What is the typical purchase price for that quantity of units (i.e. the quantity noted in the question above)?<br>*Question relevant when: not(selected( ${q104_M32_full_list} , '6')) and not(selected( ${q104_M32_full_list} , '99'))*<br>*Response constrained to: .>=0* | |
| q704_M32c *(required)* | q704_M32c. Please specify the currency for the purchase price.<br>*Question relevant when: not(selected( ${q104_M32_full_list} , '6')) and not(selected( ${q104_M32_full_list} , '99'))* | 1 Tanzanian Shillings<br>2 US dollars<br>3 Euros<br>4 Other<br>99 Don't know |
| q704_M32c.1 | q704_M32c.1 If other currency, specify:<br>*Leave blank if not applicable*<br>*Question relevant when: not(selected( ${q104_M32_full_list} , '6')) and not(selected( ${q104_M32_full_list} , '99'))* | |
| q704_M32d *(required)* | q704_M32d. Please specify the year of the purchase price.<br>*Question relevant when: not(selected( ${q104_M32_full_list} , '6')) and not(selected( ${q104_M32_full_list} , '99'))*<br>*Response constrained to: .>=2000 and .<=2019 or .=999* | |

| Field | Question | Answer |
|---|---|---|
| q704_M33 | ***q704_M33. Ampicillin (injection)***<br>*Question relevant when: not(selected( ${q104_M33_full_list} , '6')) and not(selected( ${q104_M33_full_list} , '99'))* | |
| q704_M33a *(required)* | q704_M33a. When purchased, how many units of this item typically come in one pack, box, bottle, etc.? (e.g. 100 tablets, 1000 ml's, etc.) (Write 1 if purchased as a single item.)<br>*Smallest units listed were: "[q204_M33c]" , "[q304_M33c]" , "[q404_M33c]" , "[q504_M33c]" , "[q604_M33c]"*<br>*Question relevant when: not(selected( ${q104_M33_full_list} , '6')) and not(selected( ${q104_M33_full_list} , '99'))*<br>*Response constrained to: .>=0* | |
| q704_M33a.1 *(required)* | q704_M33a.1 What is the typical size of each item in the pack, box, etc. (e.g. 1 litre bottles, 250 mg tablets, 3 ml ampoules, etc.)?<br>*Please provide as much information as is available.*<br>*Question relevant when: not(selected( ${q104_M33_full_list} , '6')) and not(selected( ${q104_M33_full_list} , '99'))* | |
| q704_M33b *(required)* | q704_M33b. What is the typical purchase price for that quantity of units (i.e. the quantity noted in the question above)?<br>*Question relevant when: not(selected( ${q104_M33_full_list} , '6')) and not(selected( ${q104_M33_full_list} , '99'))*<br>*Response constrained to: .>=0* | |

| | | |
|---|---|---|
| q704_M33c *(required)* | q704_M33c. Please specify the currency for the purchase price.<br>*Question relevant when: not(selected( ${q104_M33_full_list} , '6')) and not(selected( ${q104_M33_full_list} , '99'))* | 1 Tanzanian Shillings<br>2 US dollars<br>3 Euros<br>4 Other<br>99 Don't know |
| q704_M33c.1 | q704_M33c.1 If other currency, specify:<br>*Leave blank if not applicable*<br>*Question relevant when: not(selected( ${q104_M33_full_list} , '6')) and not(selected( ${q104_M33_full_list} , '99'))* | |
| q704_M33d *(required)* | q704_M33d. Please specify the year of the purchase price.<br>*Question relevant when: not(selected( ${q104_M33_full_list} , '6')) and not(selected( ${q104_M33_full_list} , '99'))*<br>*Response constrained to: .>=2000 and .<=2019 or .=999* | |
| q704_M34 | ***q704_M34. Ampicillin/sulbactam (Unasyn)***<br>*Question relevant when: not(selected( ${q104_M34_full_list} , '6')) and not(selected( ${q104_M34_full_list} , '99'))* | |
| q704_M34a *(required)* | q704_M34a. When purchased, how many units of this item typically come in one pack, box, bottle, etc.? (e.g. 100 tablets, 1000 ml's, etc.) (Write 1 if purchased as a single item.)<br>*Smallest units listed were: "[q204_M34c]" , "[q304_M34c]" , "[q404_M34c]" , "[q504_M34c]" , "[q604_M34c]"*<br>*Question relevant when: not(selected( ${q104_M34_full_list} , '6')) and not(selected( ${q104_M34_full_list} , '99'))*<br>*Response constrained to: .>=0* | |
| q704_M34a.1 *(required)* | q704_M34a.1 What is the typical size of each item in the pack, box, etc. (e.g. 1 litre bottles, 250 mg tablets, 3 ml ampoules, etc.)?<br>*Please provide as much information as is available.*<br>*Question relevant when: not(selected( ${q104_M34_full_list} , '6')) and not(selected( ${q104_M34_full_list} , '99'))* | |
| q704_M34b *(required)* | q704_M34b. What is the typical purchase price for that quantity of units (i.e. the quantity noted in the question above)?<br>*Question relevant when: not(selected( ${q104_M34_full_list} , '6')) and not(selected( ${q104_M34_full_list} , '99'))*<br>*Response constrained to: .>=0* | |
| q704_M34c *(required)* | q704_M34c. Please specify the currency for the purchase price.<br>*Question relevant when: not(selected( ${q104_M34_full_list} , '6')) and not(selected( ${q104_M34_full_list} , '99'))* | 1 Tanzanian Shillings<br>2 US dollars<br>3 Euros<br>4 Other<br>99 Don't know |
| q704_M34c.1 | q704_M34c.1 If other currency, specify:<br>*Leave blank if not applicable*<br>*Question relevant when: not(selected( ${q104_M34_full_list} , '6')) and not(selected( ${q104_M34_full_list} , '99'))* | |
| q704_M34d *(required)* | q704_M34d. Please specify the year of the purchase price.<br>*Question relevant when: not(selected( ${q104_M34_full_list} , '6')) and not(selected( ${q104_M34_full_list} , '99'))*<br>*Response constrained to: .>=2000 and .<=2019 or .=999* | |

| Field | Question | Answer |
|---|---|---|
| q704_M35 | ***q704_M35. Ceftriaxone***<br>*Question relevant when: not(selected( ${q104_M35_full_list} , '6')) and not(selected( ${q104_M35_full_list} , '99'))* | |
| q704_M35a *(required)* | q704_M35a. When purchased, how many units of this item typically come in one pack, box, bottle, etc.? (e.g. 100 tablets, 1000 ml's, etc.) (Write 1 if purchased as a single item.)<br>*Smallest units listed were: "[q204_M35c]" , "[q304_M35c]" , "[q404_M35c]" , "[q504_M35c]" , "[q604_M35c]"*<br>*Question relevant when: not(selected( ${q104_M35_full_list} , '6')) and not(selected( ${q104_M35_full_list} , '99'))*<br>*Response constrained to: .>=0* | |
| q704_M35a.1 *(required)* | q704_M35a.1 What is the typical size of each item in the pack, box, etc. (e.g. 1 litre bottles, 250 mg tablets, 3 ml ampoules, etc.)?<br>*Please provide as much information as is available.*<br>*Question relevant when: not(selected( ${q104_M35_full_list} , '6')) and not(selected( ${q104_M35_full_list} , '99'))* | |
| q704_M35b *(required)* | q704_M35b. What is the typical purchase price for that quantity of units (i.e. the quantity noted in the question above)?<br>*Question relevant when: not(selected( ${q104_M35_full_list} , '6')) and not(selected( ${q104_M35_full_list} , '99'))*<br>*Response constrained to: .>=0* | |

| | | |
|---|---|---|
| q704_M35c *(required)* | q704_M35c. Please specify the currency for the purchase price.<br>*Question relevant when: not(selected( ${q104_M35_full_list} , '6')) and not(selected( ${q104_M35_full_list} , '99'))* | 1 Tanzanian Shillings<br>2 US dollars<br>3 Euros<br>4 Other<br>99 Don't know |
| q704_M35c.1 | q704_M35c.1 If other currency, specify:<br>*Leave blank if not applicable*<br>*Question relevant when: not(selected( ${q104_M35_full_list} , '6')) and not(selected( ${q104_M35_full_list} , '99'))* | |
| q704_M35d *(required)* | q704_M35d. Please specify the year of the purchase price.<br>*Question relevant when: not(selected( ${q104_M35_full_list} , '6')) and not(selected( ${q104_M35_full_list} , '99'))*<br>*Response constrained to: .>=2000 and .<=2019 or .=999* | |
| q704_M36 | ***q704_M36. Chloramphenicol Sodium Succinate (injection)***<br>*Question relevant when: not(selected( ${q104_M36_full_list} , '6')) and not(selected( ${q104_M36_full_list} , '99'))* | |
| q704_M36a *(required)* | q704_M36a. When purchased, how many units of this item typically come in one pack, box, bottle, etc.? (e.g. 100 tablets, 1000 ml's, etc.) (Write 1 if purchased as a single item.)<br>*Smallest units listed were: "[q204_M36c]" , "[q304_M36c]" , "[q404_M36c]" , "[q504_M36c]" , "[q604_M36c]"*<br>*Question relevant when: not(selected( ${q104_M36_full_list} , '6')) and not(selected( ${q104_M36_full_list} , '99'))*<br>*Response constrained to: .>=0* | |
| q704_M36a.1 *(required)* | q704_M36a.1 What is the typical size of each item in the pack, box, etc. (e.g. 1 litre bottles, 250 mg tablets, 3 ml ampoules, etc.)?<br>*Please provide as much information as is available.*<br>*Question relevant when: not(selected( ${q104_M36_full_list} , '6')) and not(selected( ${q104_M36_full_list} , '99'))* | |
| q704_M36b *(required)* | q704_M36b. What is the typical purchase price for that quantity of units (i.e. the quantity noted in the question above)?<br>*Question relevant when: not(selected( ${q104_M36_full_list} , '6')) and not(selected( ${q104_M36_full_list} , '99'))*<br>*Response constrained to: .>=0* | |
| q704_M36c *(required)* | q704_M36c. Please specify the currency for the purchase price.<br>*Question relevant when: not(selected( ${q104_M36_full_list} , '6')) and not(selected( ${q104_M36_full_list} , '99'))* | 1 Tanzanian Shillings<br>2 US dollars<br>3 Euros<br>4 Other<br>99 Don't know |
| q704_M36c.1 | q704_M36c.1 If other currency, specify:<br>*Leave blank if not applicable*<br>*Question relevant when: not(selected( ${q104_M36_full_list} , '6')) and not(selected( ${q104_M36_full_list} , '99'))* | |
| q704_M36d *(required)* | q704_M36d. Please specify the year of the purchase price.<br>*Question relevant when: not(selected( ${q104_M36_full_list} , '6')) and not(selected( ${q104_M36_full_list} , '99'))*<br>*Response constrained to: .>=2000 and .<=2019 or .=999* | |

Response constrained to: .>=2000 and .<=2019 or .=999

| Field | Question | Answer |
|---|---|---|
| q704_M37 | ***q704_M37. Ciprofloxacin***<br>*Question relevant when: not(selected( ${q104_M37_full_list} , '6')) and not(selected( ${q104_M37_full_list} , '99'))* | |
| q704_M37a *(required)* | q704_M37a. When purchased, how many units of this item typically come in one pack, box, bottle, etc.? (e.g. 100 tablets, 1000 ml's, etc.) (Write 1 if purchased as a single item.)<br>*Smallest units listed were: "[q204_M37c]" , "[q304_M37c]" , "[q404_M37c]" , "[q504_M37c]" , "[q604_M37c]"*<br>*Question relevant when: not(selected( ${q104_M37_full_list} , '6')) and not(selected( ${q104_M37_full_list} , '99'))*<br>*Response constrained to: .>=0* | |
| q704_M37a.1 *(required)* | q704_M37a.1 What is the typical size of each item in the pack, box, etc. (e.g. 1 litre bottles, 250 mg tablets, 3 ml ampoules, etc.)?<br>*Please provide as much information as is available.*<br>*Question relevant when: not(selected( ${q104_M37_full_list} , '6')) and not(selected( ${q104_M37_full_list} , '99'))* | |
| q704_M37b *(required)* | q704_M37b. What is the typical purchase price for that quantity of units (i.e. the quantity noted in the question above)?<br>*Question relevant when: not(selected( ${q104_M37_full_list} , '6')) and not(selected( ${q104_M37_full_list} , '99'))*<br>*Response constrained to: .>=0* | |
| q704_M37c *(required)* | q704_M37c. Please specify the currency for the purchase price.<br>*Question relevant when: not(selected( ${q104_M37_full_list} , '6')) and not(selected( ${q104_M37_full_list} , '99'))* | 1 Tanzanian Shillings<br>2 US dollars<br>3 Euros<br>4 Other<br>99 Don't know |
| q704_M37c.1 | q704_M37c.1 If other currency, specify:<br>*Leave blank if not applicable*<br>*Question relevant when: not(selected( ${q104_M37_full_list} , '6')) and not(selected( ${q104_M37_full_list} , '99'))* | |
| q704_M37d *(required)* | q704_M37d. Please specify the year of the purchase price.<br>*Question relevant when: not(selected( ${q104_M37_full_list} , '6')) and not(selected( ${q104_M37_full_list} , '99'))*<br>*Response constrained to: .>=2000 and .<=2019 or .=999* | |
| q704_M38 | ***q704_M38. Doxycycline***<br>*Question relevant when: not(selected( ${q104_M38_full_list} , '6')) and not(selected( ${q104_M38_full_list} , '99'))* | |
| q704_M38a *(required)* | q704_M38a. When purchased, how many units of this item typically come in one pack, box, bottle, etc.? (e.g. 100 tablets, 1000 ml's, etc.) (Write 1 if purchased as a single item.)<br>*Smallest units listed were: "[q204_M38c]" , "[q304_M38c]" , "[q404_M38c]" , "[q504_M38c]" , "[q604_M38c]"*<br>*Question relevant when: not(selected( ${q104_M38_full_list} , '6')) and not(selected( ${q104_M38_full_list} , '99'))*<br>*Response constrained to: .>=0* | |
| q704_M38a.1 *(required)* | q704_M38a.1 What is the typical size of each item in the pack, box, etc. (e.g. 1 litre bottles, 250 mg tablets, 3 ml ampoules, etc.)?<br>*Please provide as much information as is available.*<br>*Question relevant when: not(selected( ${q104_M38_full_list} , '6')) and not(selected( ${q104_M38_full_list} , '99'))* | |
| q704_M38b *(required)* | q704_M38b. What is the typical purchase price for that quantity of units (i.e. the quantity noted in the question above)?<br>*Question relevant when: not(selected( ${q104_M38_full_list} , '6')) and not(selected( ${q104_M38_full_list} , '99'))*<br>*Response constrained to: .>=0* | |
| q704_M38c *(required)* | q704_M38c. Please specify the currency for the purchase price.<br>*Question relevant when: not(selected( ${q104_M38_full_list} , '6')) and not(selected( ${q104_M38_full_list} , '99'))* | 1 Tanzanian Shillings<br>2 US dollars<br>3 Euros<br>4 Other<br>99 Don't know |
| q704_M38c.1 | q704_M38c.1 If other currency, specify:<br>*Leave blank if not applicable*<br>*Question relevant when: not(selected( ${q104_M38_full_list} , '6')) and not(selected( ${q104_M38_full_list} , '99'))* | |
| q704_M38d *(required)* | q704_M38d. Please specify the year of the purchase price.<br>*Question relevant when: not(selected( ${q104_M38_full_list} , '6')) and not(selected( ${q104_M38_full_list} , '99'))*<br>*Response constrained to: .>=2000 and .<=2019 or .=999* | |

*Response constrained to: .>=2000 and .<=2019 or .=999*

| Field | Question | Answer |
|---|---|---|
| q704_M39 | ***q704_M39. Erythromycin***<br>*Question relevant when: not(selected( ${q104_M39_full_list} , '6')) and not(selected( ${q104_M39_full_list} , '99'))* | |
| q704_M39a *(required)* | q704_M39a. When purchased, how many units of this item typically come in one pack, box, bottle, etc.? (e.g. 100 tablets, 1000 ml's, etc.) (Write 1 if purchased as a single item.)<br>*Smallest units listed were: "[q204_M39c]" , "[q304_M39c]" , "[q404_M39c]" , "[q504_M39c]" , "[q604_M39c]"*<br>*Question relevant when: not(selected( ${q104_M39_full_list} , '6')) and not(selected( ${q104_M39_full_list} , '99'))*<br>*Response constrained to: .>=0* | |
| q704_M39a.1 *(required)* | q704_M39a.1 What is the typical size of each item in the pack, box, etc. (e.g. 1 litre bottles, 250 mg tablets, 3 ml ampoules, etc.)?<br>*Please provide as much information as is available.*<br>*Question relevant when: not(selected( ${q104_M39_full_list} , '6')) and not(selected( ${q104_M39_full_list} , '99'))* | |
| q704_M39b *(required)* | q704_M39b. What is the typical purchase price for that quantity of units (i.e. the quantity noted in the question above)?<br>*Question relevant when: not(selected( ${q104_M39_full_list} , '6')) and not(selected( ${q104_M39_full_list} , '99'))*<br>*Response constrained to: .>=0* | |
| q704_M39c *(required)* | q704_M39c. Please specify the currency for the purchase price.<br>*Question relevant when: not(selected( ${q104_M39_full_list} , '6')) and not(selected( ${q104_M39_full_list} , '99'))* | 1 Tanzanian Shillings<br>2 US dollars<br>3 Euros<br>4 Other<br>99 Don't know |
| q704_M39c.1 | q704_M39c.1 If other currency, specify:<br>*Leave blank if not applicable*<br>*Question relevant when: not(selected( ${q104_M39_full_list} , '6')) and not(selected( ${q104_M39_full_list} , '99'))* | |
| q704_M39d *(required)* | q704_M39d. Please specify the year of the purchase price.<br>*Question relevant when: not(selected( ${q104_M39_full_list} , '6')) and not(selected( ${q104_M39_full_list} , '99'))*<br>*Response constrained to: .>=2000 and .<=2019 or .=999* | |
| q704_M40 | ***q704_M40. Gentamycin***<br>*Question relevant when: not(selected( ${q104_M40_full_list} , '6')) and not(selected( ${q104_M40_full_list} , '99'))* | |
| q704_M40a *(required)* | q704_M40a. When purchased, how many units of this item typically come in one pack, box, bottle, etc.? (e.g. 100 tablets, 1000 ml's, etc.) (Write 1 if purchased as a single item.)<br>*Smallest units listed were: "[q204_M40c]" , "[q304_M40c]" , "[q404_M40c]" , "[q504_M40c]" , "[q604_M40c]"*<br>*Question relevant when: not(selected( ${q104_M40_full_list} , '6')) and not(selected( ${q104_M40_full_list} , '99'))*<br>*Response constrained to: .>=0* | |
| q704_M40a.1 *(required)* | q704_M40a.1 What is the typical size of each item in the pack, box, etc. (e.g. 1 litre bottles, 250 mg tablets, 3 ml ampoules, etc.)?<br>*Please provide as much information as is available.*<br>*Question relevant when: not(selected( ${q104_M40_full_list} , '6')) and not(selected( ${q104_M40_full_list} , '99'))* | |
| q704_M40b *(required)* | q704_M40b. What is the typical purchase price for that quantity of units (i.e. the quantity noted in the question above)?<br>*Question relevant when: not(selected( ${q104_M40_full_list} , '6')) and not(selected( ${q104_M40_full_list} , '99'))*<br>*Response constrained to: .>=0* | |
| q704_M40c *(required)* | q704_M40c. Please specify the currency for the purchase price.<br>*Question relevant when: not(selected( ${q104_M40_full_list} , '6')) and not(selected( ${q104_M40_full_list} , '99'))* | 1 Tanzanian Shillings<br>2 US dollars<br>3 Euros<br>4 Other<br>99 Don't know |
| q704_M40c.1 | q704_M40c.1 If other currency, specify:<br>*Leave blank if not applicable*<br>*Question relevant when: not(selected( ${q104_M40_full_list} , '6')) and not(selected( ${q104_M40_full_list} , '99'))* | |
| q704_M40d *(required)* | q704_M40d. Please specify the year of the purchase price.<br>*Question relevant when: not(selected( ${q104_M40_full_list} , '6')) and not(selected( ${q104_M40_full_list} , '99'))*<br>*Response constrained to: .>=2000 and .<=2019 or .=999* | |

Response constrained to: .>=2000 and .<=2019 or .=999

| Field | Question | Answer |
|---|---|---|
| q704_M41 | ***q704_M41. Metronidazole (for infusion)***<br>*Question relevant when: not(selected( ${q104_M41_full_list} , '6')) and not(selected( ${q104_M41_full_list} , '99'))* | |
| q704_M41a *(required)* | q704_M41a. When purchased, how many units of this item typically come in one pack, box, bottle, etc.? (e.g. 100 tablets, 1000 ml's, etc.) (Write 1 if purchased as a single item.)<br>*Smallest units listed were: "[q204_M41c]" , "[q304_M41c]" , "[q404_M41c]" , "[q504_M41c]" , "[q604_M41c]"*<br>*Question relevant when: not(selected( ${q104_M41_full_list} , '6')) and not(selected( ${q104_M41_full_list} , '99'))*<br>*Response constrained to: .>=0* | |
| q704_M41a.1 *(required)* | q704_M41a.1 What is the typical size of each item in the pack, box, etc. (e.g. 1 litre bottles, 250 mg tablets, 3 ml ampoules, etc.)?<br>*Please provide as much information as is available.*<br>*Question relevant when: not(selected( ${q104_M41_full_list} , '6')) and not(selected( ${q104_M41_full_list} , '99'))* | |
| q704_M41b *(required)* | q704_M41b. What is the typical purchase price for that quantity of units (i.e. the quantity noted in the question above)?<br>*Question relevant when: not(selected( ${q104_M41_full_list} , '6')) and not(selected( ${q104_M41_full_list} , '99'))*<br>*Response constrained to: .>=0* | |
| q704_M41c *(required)* | q704_M41c. Please specify the currency for the purchase price.<br>*Question relevant when: not(selected( ${q104_M41_full_list} , '6')) and not(selected( ${q104_M41_full_list} , '99'))* | 1 Tanzanian Shillings<br>2 US dollars<br>3 Euros<br>4 Other<br>99 Don't know |
| q704_M41c.1 | q704_M41c.1 If other currency, specify:<br>*Leave blank if not applicable*<br>*Question relevant when: not(selected( ${q104_M41_full_list} , '6')) and not(selected( ${q104_M41_full_list} , '99'))* | |
| q704_M41d *(required)* | q704_M41d. Please specify the year of the purchase price.<br>*Question relevant when: not(selected( ${q104_M41_full_list} , '6')) and not(selected( ${q104_M41_full_list} , '99'))*<br>*Response constrained to: .>=2000 and .<=2019 or .=999* | |
| q704_M42 | ***q704_M42. Metronidazole (injection)***<br>*Question relevant when: not(selected( ${q104_M42_full_list} , '6')) and not(selected( ${q104_M42_full_list} , '99'))* | |
| q704_M42a *(required)* | q704_M42a. When purchased, how many units of this item typically come in one pack, box, bottle, etc.? (e.g. 100 tablets, 1000 ml's, etc.) (Write 1 if purchased as a single item.)<br>*Smallest units listed were: "[q204_M42c]" , "[q304_M42c]" , "[q404_M42c]" , "[q504_M42c]" , "[q604_M42c]"*<br>*Question relevant when: not(selected( ${q104_M42_full_list} , '6')) and not(selected( ${q104_M42_full_list} , '99'))*<br>*Response constrained to: .>=0* | |
| q704_M42a.1 *(required)* | q704_M42a.1 What is the typical size of each item in the pack, box, etc. (e.g. 1 litre bottles, 250 mg tablets, 3 ml ampoules, etc.)?<br>*Please provide as much information as is available.*<br>*Question relevant when: not(selected( ${q104_M42_full_list} , '6')) and not(selected( ${q104_M42_full_list} , '99'))* | |
| q704_M42b *(required)* | q704_M42b. What is the typical purchase price for that quantity of units (i.e. the quantity noted in the question above)?<br>*Question relevant when: not(selected( ${q104_M42_full_list} , '6')) and not(selected( ${q104_M42_full_list} , '99'))*<br>*Response constrained to: .>=0* | |
| q704_M42c *(required)* | q704_M42c. Please specify the currency for the purchase price.<br>*Question relevant when: not(selected( ${q104_M42_full_list} , '6')) and not(selected( ${q104_M42_full_list} , '99'))* | 1 Tanzanian Shillings<br>2 US dollars<br>3 Euros<br>4 Other<br>99 Don't know |
| q704_M42c.1 | q704_M42c.1 If other currency, specify:<br>*Leave blank if not applicable*<br>*Question relevant when: not(selected( ${q104_M42_full_list} , '6')) and not(selected( ${q104_M42_full_list} , '99'))* | |
| q704_M42d *(required)* | q704_M42d. Please specify the year of the purchase price.<br>*Question relevant when: not(selected( ${q104_M42_full_list} , '6')) and not(selected( ${q104_M42_full_list} , '99'))*<br>*Response constrained to: .>=2000 and .<=2019 or .=999* | |

*Response constrained to: .>=2000 and .<=2019 or .=999*

| Field | Question | Answer |
|---|---|---|
| q704_M43 | ***q704_M43. Metronidazole (oral)***<br>*Question relevant when: not(selected( ${q104_M43_full_list} , '6')) and not(selected( ${q104_M43_full_list} , '99'))* | |
| q704_M43a *(required)* | q704_M43a. When purchased, how many units of this item typically come in one pack, box, bottle, etc.? (e.g. 100 tablets, 1000 ml's, etc.) (Write 1 if purchased as a single item.)<br>*Smallest units listed were: "[q204_M43c]" , "[q304_M43c]" , "[q404_M43c]" , "[q504_M43c]" , "[q604_M43c]"*<br>*Question relevant when: not(selected( ${q104_M43_full_list} , '6')) and not(selected( ${q104_M43_full_list} , '99'))*<br>*Response constrained to: .>=0* | |
| q704_M43a.1 *(required)* | q704_M43a.1 What is the typical size of each item in the pack, box, etc. (e.g. 1 litre bottles, 250 mg tablets, 3 ml ampoules, etc.)?<br>*Please provide as much information as is available.*<br>*Question relevant when: not(selected( ${q104_M43_full_list} , '6')) and not(selected( ${q104_M43_full_list} , '99'))* | |
| q704_M43b *(required)* | q704_M43b. What is the typical purchase price for that quantity of units (i.e. the quantity noted in the question above)?<br>*Question relevant when: not(selected( ${q104_M43_full_list} , '6')) and not(selected( ${q104_M43_full_list} , '99'))*<br>*Response constrained to: .>=0* | |
| q704_M43c *(required)* | q704_M43c. Please specify the currency for the purchase price.<br>*Question relevant when: not(selected( ${q104_M43_full_list} , '6')) and not(selected( ${q104_M43_full_list} , '99'))* | 1 Tanzanian Shillings<br>2 US dollars<br>3 Euros<br>4 Other<br>99 Don't know |
| q704_M43c.1 | q704_M43c.1 If other currency, specify:<br>*Leave blank if not applicable*<br>*Question relevant when: not(selected( ${q104_M43_full_list} , '6')) and not(selected( ${q104_M43_full_list} , '99'))* | |
| q704_M43d *(required)* | q704_M43d. Please specify the year of the purchase price.<br>*Question relevant when: not(selected( ${q104_M43_full_list} , '6')) and not(selected( ${q104_M43_full_list} , '99'))*<br>*Response constrained to: .>=2000 and .<=2019 or .=999* | |
| q704_M44 | ***q704_M44. Penicillin***<br>*Question relevant when: not(selected( ${q104_M44_full_list} , '6')) and not(selected( ${q104_M44_full_list} , '99'))* | |
| q704_M44a *(required)* | q704_M44a. When purchased, how many units of this item typically come in one pack, box, bottle, etc.? (e.g. 100 tablets, 1000 ml's, etc.) (Write 1 if purchased as a single item.)<br>*Smallest units listed were: "[q204_M44c]" , "[q304_M44c]" , "[q404_M44c]" , "[q504_M44c]" , "[q604_M44c]"*<br>*Question relevant when: not(selected( ${q104_M44_full_list} , '6')) and not(selected( ${q104_M44_full_list} , '99'))*<br>*Response constrained to: .>=0* | |
| q704_M44a.1 *(required)* | q704_M44a.1 What is the typical size of each item in the pack, box, etc. (e.g. 1 litre bottles, 250 mg tablets, 3 ml ampoules, etc.)?<br>*Please provide as much information as is available.*<br>*Question relevant when: not(selected( ${q104_M44_full_list} , '6')) and not(selected( ${q104_M44_full_list} , '99'))* | |
| q704_M44b *(required)* | q704_M44b. What is the typical purchase price for that quantity of units (i.e. the quantity noted in the question above)?<br>*Question relevant when: not(selected( ${q104_M44_full_list} , '6')) and not(selected( ${q104_M44_full_list} , '99'))*<br>*Response constrained to: .>=0* | |
| q704_M44c *(required)* | q704_M44c. Please specify the currency for the purchase price.<br>*Question relevant when: not(selected( ${q104_M44_full_list} , '6')) and not(selected( ${q104_M44_full_list} , '99'))* | 1 Tanzanian Shillings<br>2 US dollars<br>3 Euros<br>4 Other<br>99 Don't know |
| q704_M44c.1 | q704_M44c.1 If other currency, specify:<br>*Leave blank if not applicable*<br>*Question relevant when: not(selected( ${q104_M44_full_list} , '6')) and not(selected( ${q104_M44_full_list} , '99'))* | |
| q704_M44d *(required)* | q704_M44d. Please specify the year of the purchase price.<br>*Question relevant when: not(selected( ${q104_M44_full_list} , '6')) and not(selected( ${q104_M44_full_list} , '99'))*<br>*Response constrained to: .>=2000 and .<=2019 or .=999* | |

Response constrained to: .>=2000 and .<=2019 or .=999

| Field | Question | Answer |
|---|---|---|
| q704_M45 | ***q704_M45. Antibiotic - other 1: "[q104_M45_full_list_other]"***<br>*Question relevant when: not(selected( ${q104_M45_full_list} , '6')) and not(selected( ${q104_M45_full_list} , '99')) and string-length( ${q104_M45_full_list_other} ) >0* | |
| q704_M45a *(required)* | q704_M45a. When purchased, how many units of this item typically come in one pack, box, bottle, etc.? (e.g. 100 tablets, 1000 ml's, etc.) (Write 1 if purchased as a single item.)<br>*Smallest units listed were: "[q204_M45c]" , "[q304_M45c]" , "[q404_M45c]" , "[q504_M45c]" , "[q604_M45c]"*<br>*Question relevant when: not(selected( ${q104_M45_full_list} , '6')) and not(selected( ${q104_M45_full_list} , '99')) and string-length( ${q104_M45_full_list_other} ) >0*<br>*Response constrained to: .>=0* | |
| q704_M45a.1 *(required)* | q704_M45a.1 What is the typical size of each item in the pack, box, etc. (e.g. 1 litre bottles, 250 mg tablets, 3 ml ampoules, etc.)?<br>*Please provide as much information as is available.*<br>*Question relevant when: not(selected( ${q104_M45_full_list} , '6')) and not(selected( ${q104_M45_full_list} , '99')) and string-length( ${q104_M45_full_list_other} ) >0* | |
| q704_M45b *(required)* | q704_M45b. What is the typical purchase price for that quantity of units (i.e. the quantity noted in the question above)?<br>*Question relevant when: not(selected( ${q104_M45_full_list} , '6')) and not(selected( ${q104_M45_full_list} , '99')) and string-length( ${q104_M45_full_list_other} ) >0*<br>*Response constrained to: .>=0* | |

| | | |
|---|---|---|
| q704_M45c *(required)* | q704_M45c. Please specify the currency for the purchase price.<br>*Question relevant when: not(selected( ${q104_M45_full_list} , '6')) and not(selected( ${q104_M45_full_list} , '99')) and string-length( ${q104_M45_full_list_other} ) >0* | 1 Tanzanian Shillings<br>2 US dollars<br>3 Euros<br>4 Other<br>99 Don't know |
| q704_M45c.1 | q704_M45c.1 If other currency, specify:<br>*Leave blank if not applicable*<br>*Question relevant when: not(selected( ${q104_M45_full_list} , '6')) and not(selected( ${q104_M45_full_list} , '99')) and string-length( ${q104_M45_full_list_other} ) >0* | |
| q704_M45d *(required)* | q704_M45d. Please specify the year of the purchase price.<br>*Question relevant when: not(selected( ${q104_M45_full_list} , '6')) and not(selected( ${q104_M45_full_list} , '99')) and string-length( ${q104_M45_full_list_other} ) >0*<br>*Response constrained to: .>=2000 and .<=2019 or .=999* | |
| q704_M46 | ***q704_M46. Antibiotic - other 2: "[q104_M46_full_list_other]"***<br>*Question relevant when: not(selected( ${q104_M46_full_list} , '6')) and not(selected( ${q104_M46_full_list} , '99')) and string-length( ${q104_M46_full_list_other} ) >0* | |
| q704_M46a *(required)* | q704_M46a. When purchased, how many units of this item typically come in one pack, box, bottle, etc.? (e.g. 100 tablets, 1000 ml's, etc.) (Write 1 if purchased as a single item.)<br>*Smallest units listed were: "[q204_M46c]" , "[q304_M46c]" , "[q404_M46c]" , "[q504_M46c]" , "[q604_M46c]"*<br>*Question relevant when: not(selected( ${q104_M46_full_list} , '6')) and not(selected( ${q104_M46_full_list} , '99')) and string-length( ${q104_M46_full_list_other} ) >0*<br>*Response constrained to: .>=0* | |
| q704_M46a.1 *(required)* | q704_M46a.1 What is the typical size of each item in the pack, box, etc. (e.g. 1 litre bottles, 250 mg tablets, 3 ml ampoules, etc.)?<br>*Please provide as much information as is available.*<br>*Question relevant when: not(selected( ${q104_M46_full_list} , '6')) and not(selected( ${q104_M46_full_list} , '99')) and string-length( ${q104_M46_full_list_other} ) >0* | |
| q704_M46b *(required)* | q704_M46b. What is the typical purchase price for that quantity of units (i.e. the quantity noted in the question above)?<br>*Question relevant when: not(selected( ${q104_M46_full_list} , '6')) and not(selected( ${q104_M46_full_list} , '99')) and string-length( ${q104_M46_full_list_other} ) >0*<br>*Response constrained to: .>=0* | |
| q704_M46c *(required)* | q704_M46c. Please specify the currency for the purchase price.<br>*Question relevant when: not(selected( ${q104_M46_full_list} , '6')) and not(selected( ${q104_M46_full_list} , '99')) and string-length( ${q104_M46_full_list_other} ) >0* | 1 Tanzanian Shillings<br>2 US dollars<br>3 Euros<br>4 Other<br>99 Don't know |
| q704_M46c.1 | q704_M46c.1 If other currency, specify:<br>*Leave blank if not applicable*<br>*Question relevant when: not(selected( ${q104_M46_full_list} , '6')) and not(selected( ${q104_M46_full_list} , '99')) and string-length( ${q104_M46_full_list_other} ) >0* | |
| q704_M46d *(required)* | q704_M46d. Please specify the year of the purchase price.<br>*Question relevant when: not(selected( ${q104_M46_full_list} , '6')) and not(selected( ${q104_M46_full_list} , '99')) and string-length( ${q104_M46_full_list_other} ) >0*<br>*Response constrained to: .>=2000 and .<=2019 or .=999* | |

Response constrained to: .>=2000 and .<=2019 or .=999

| Field | Question | Answer |
|---|---|---|
| q704_M47 | ***q704_M47. Antibiotic - other 3: "[q104_M47_full_list_other]"***<br>*Question relevant when: not(selected( ${q104_M47_full_list} , '6')) and not(selected( ${q104_M47_full_list} , '99')) and string-length( ${q104_M47_full_list_other} ) >0* | |
| q704_M47a *(required)* | q704_M47a. When purchased, how many units of this item typically come in one pack, box, bottle, etc.? (e.g. 100 tablets, 1000 ml's, etc.) (Write 1 if purchased as a single item.)<br>*Smallest units listed were: "[q204_M47c]" , "[q304_M47c]" , "[q404_M47c]" , "[q504_M47c]" , "[q604_M47c]"*<br>*Question relevant when: not(selected( ${q104_M47_full_list} , '6')) and not(selected( ${q104_M47_full_list} , '99')) and string-length( ${q104_M47_full_list_other} ) >0*<br>*Response constrained to: .>=0* | |
| q704_M47a.1 *(required)* | q704_M47a.1 What is the typical size of each item in the pack, box, etc. (e.g. 1 litre bottles, 250 mg tablets, 3 ml ampoules, etc.)?<br>*Please provide as much information as is available.*<br>*Question relevant when: not(selected( ${q104_M47_full_list} , '6')) and not(selected( ${q104_M47_full_list} , '99')) and string-length( ${q104_M47_full_list_other} ) >0* | |
| q704_M47b *(required)* | q704_M47b. What is the typical purchase price for that quantity of units (i.e. the quantity noted in the question above)?<br>*Question relevant when: not(selected( ${q104_M47_full_list} , '6')) and not(selected( ${q104_M47_full_list} , '99')) and string-length( ${q104_M47_full_list_other} ) >0*<br>*Response constrained to: .>=0* | |
| q704_M47c *(required)* | q704_M47c. Please specify the currency for the purchase price.<br>*Question relevant when: not(selected( ${q104_M47_full_list} , '6')) and not(selected( ${q104_M47_full_list} , '99')) and string-length( ${q104_M47_full_list_other} ) >0* | 1 Tanzanian Shillings<br>2 US dollars<br>3 Euros<br>4 Other<br>99 Don't know |
| q704_M47c.1 | q704_M47c.1 If other currency, specify:<br>*Leave blank if not applicable*<br>*Question relevant when: not(selected( ${q104_M47_full_list} , '6')) and not(selected( ${q104_M47_full_list} , '99')) and string-length( ${q104_M47_full_list_other} ) >0* | |
| q704_M47d *(required)* | q704_M47d. Please specify the year of the purchase price.<br>*Question relevant when: not(selected( ${q104_M47_full_list} , '6')) and not(selected( ${q104_M47_full_list} , '99')) and string-length( ${q104_M47_full_list_other} ) >0*<br>*Response constrained to: .>=2000 and .<=2019 or .=999* | |
| q704_M48 | ***q704_M48. Antibiotic - other 4: "[q104_M48_full_list_other]"***<br>*Question relevant when: not(selected( ${q104_M48_full_list} , '6')) and not(selected( ${q104_M48_full_list} , '99')) and string-length( ${q104_M48_full_list_other} ) >0* | |
| q704_M48a *(required)* | q704_M48a. When purchased, how many units of this item typically come in one pack, box, bottle, etc.? (e.g. 100 tablets, 1000 ml's, etc.) (Write 1 if purchased as a single item.)<br>*Smallest units listed were: "[q204_M48c]" , "[q304_M48c]" , "[q404_M48c]" , "[q504_M48c]" , "[q604_M48c]"*<br>*Question relevant when: not(selected( ${q104_M48_full_list} , '6')) and not(selected( ${q104_M48_full_list} , '99')) and string-length( ${q104_M48_full_list_other} ) >0*<br>*Response constrained to: .>=0* | |
| q704_M48a.1 *(required)* | q704_M48a.1 What is the typical size of each item in the pack, box, etc. (e.g. 1 litre bottles, 250 mg tablets, 3 ml ampoules, etc.)?<br>*Please provide as much information as is available.*<br>*Question relevant when: not(selected( ${q104_M48_full_list} , '6')) and not(selected( ${q104_M48_full_list} , '99')) and string-length( ${q104_M48_full_list_other} ) >0* | |
| q704_M48b *(required)* | q704_M48b. What is the typical purchase price for that quantity of units (i.e. the quantity noted in the question above)?<br>*Question relevant when: not(selected( ${q104_M48_full_list} , '6')) and not(selected( ${q104_M48_full_list} , '99')) and string-length( ${q104_M48_full_list_other} ) >0*<br>*Response constrained to: .>=0* | |
| q704_M48c *(required)* | q704_M48c. Please specify the currency for the purchase price.<br>*Question relevant when: not(selected( ${q104_M48_full_list} , '6')) and not(selected( ${q104_M48_full_list} , '99')) and string-length( ${q104_M48_full_list_other} ) >0* | 1 Tanzanian Shillings<br>2 US dollars<br>3 Euros<br>4 Other<br>99 Don't know |
| q704_M48c.1 | q704_M48c.1 If other currency, specify:<br>*Leave blank if not applicable*<br>*Question relevant when: not(selected( ${q104_M48_full_list} , '6')) and not(selected( ${q104_M48_full_list} , '99')) and string-length( ${q104_M48_full_list_other} ) >0* | |
| q704_M48d *(required)* | q704_M48d. Please specify the year of the purchase price.<br>*Question relevant when: not(selected( ${q104_M48_full_list} , '6')) and not(selected( ${q104_M48_full_list} , '99')) and string-length( ${q104_M48_full_list_other} ) >0*<br>*Response constrained to: .>=2000 and .<=2019 or .=999* | |

| Field | Question | Answer |
|---|---|---|
| q704_M49 | ***q704_M49. Antibiotic - other 5: "[q104_M49_full_list_other]"*** <br> *Question relevant when: not(selected( ${q104_M49_full_list} , '6')) and not(selected( ${q104_M49_full_list} , '99')) and string-length( ${q104_M49_full_list_other} ) >0* | |
| q704_M49a *(required)* | q704_M49a. When purchased, how many units of this item typically come in one pack, box, bottle, etc.? (e.g. 100 tablets, 1000 ml's, etc.) (Write 1 if purchased as a single item.) <br> *Smallest units listed were: "[q204_M49c]" , "[q304_M49c]" , "[q404_M49c]" , "[q504_M49c]" , "[q604_M49c]"* <br> *Question relevant when: not(selected( ${q104_M49_full_list} , '6')) and not(selected( ${q104_M49_full_list} , '99')) and string-length( ${q104_M49_full_list_other} ) >0* <br> *Response constrained to: .>=0* | |
| q704_M49a.1 *(required)* | q704_M49a.1 What is the typical size of each item in the pack, box, etc. (e.g. 1 litre bottles, 250 mg tablets, 3 ml ampoules, etc.)? <br> *Please provide as much information as is available.* <br> *Question relevant when: not(selected( ${q104_M49_full_list} , '6')) and not(selected( ${q104_M49_full_list} , '99')) and string-length( ${q104_M49_full_list_other} ) >0* | |
| q704_M49b *(required)* | q704_M49b. What is the typical purchase price for that quantity of units (i.e. the quantity noted in the question above)? <br> *Question relevant when: not(selected( ${q104_M49_full_list} , '6')) and not(selected( ${q104_M49_full_list} , '99')) and string-length( ${q104_M49_full_list_other} ) >0* <br> *Response constrained to: .>=0* | |
| q704_M49c *(required)* | q704_M49c. Please specify the currency for the purchase price. <br> *Question relevant when: not(selected( ${q104_M49_full_list} , '6')) and not(selected( ${q104_M49_full_list} , '99')) and string-length( ${q104_M49_full_list_other} ) >0* | 1 Tanzanian Shillings <br> 2 US dollars <br> 3 Euros <br> 4 Other <br> 99 Don't know |
| q704_M49c.1 | q704_M49c.1 If other currency, specify: <br> *Leave blank if not applicable* <br> *Question relevant when: not(selected( ${q104_M49_full_list} , '6')) and not(selected( ${q104_M49_full_list} , '99')) and string-length( ${q104_M49_full_list_other} ) >0* | |
| q704_M49d *(required)* | q704_M49d. Please specify the year of the purchase price. <br> *Question relevant when: not(selected( ${q104_M49_full_list} , '6')) and not(selected( ${q104_M49_full_list} , '99')) and string-length( ${q104_M49_full_list_other} ) >0* <br> *Response constrained to: .>=2000 and .<=2019 or .=999* | |
| D. Medications - Cost (4) <br> *Group relevant when: selected( ${section_seven_skip_med} , '1')* | | |
| q704_med_note4 | 704 CONTINUED For each of the following medications and other medical products that are used for postabortion care, can you tell me …? | |
| note_704_antiseptic | ***Antiseptic*** | |
| q704_M50 | ***q704_M50. Cetrimide 15% ("Savlon")*** <br> *Question relevant when: not(selected( ${q104_M50_full_list} , '6')) and not(selected( ${q104_M50_full_list} , '99'))* | |
| q704_M50a *(required)* | q704_M50a. When purchased, how many units of this item typically come in one pack, box, bottle, etc.? (e.g. 100 tablets, 1000 ml's, etc.) (Write 1 if purchased as a single item.) <br> *Smallest units listed were: "[q204_M50c]" , "[q304_M50c]" , "[q404_M50c]" , "[q504_M50c]" , "[q604_M50c]"* <br> *Question relevant when: not(selected( ${q104_M50_full_list} , '6')) and not(selected( ${q104_M50_full_list} , '99'))* <br> *Response constrained to: .>=0* | |
| q704_M50a.1 *(required)* | q704_M50a.1 What is the typical size of each item in the pack, box, etc. (e.g. 1 litre bottles, 250 mg tablets, 3 ml ampoules, etc.)? <br> *Please provide as much information as is available.* <br> *Question relevant when: not(selected( ${q104_M50_full_list} , '6')) and not(selected( ${q104_M50_full_list} , '99'))* | |
| q704_M50b *(required)* | q704_M50b. What is the typical purchase price for that quantity of units (i.e. the quantity noted in the question above)? <br> *Question relevant when: not(selected( ${q104_M50_full_list} , '6')) and not(selected( ${q104_M50_full_list} , '99'))* <br> *Response constrained to: .>=0* | |
| q704_M50c *(required)* | q704_M50c. Please specify the currency for the purchase price. <br> *Question relevant when: not(selected( ${q104_M50_full_list} , '6')) and not(selected( ${q104_M50_full_list} , '99'))* | 1 Tanzanian Shillings <br> 2 US dollars <br> 3 Euros <br> 4 Other <br> 99 Don't know |
| q704_M50c.1 | q704_M50c.1 If other currency, specify: <br> *Leave blank if not applicable* | |

*Question relevant when: not(selected( ${q104_M50_full_list} , '6')) and not(selected( ${q104_M50_full_list} , '99'))*

| Field | Question | Answer |
|---|---|---|
| q704_M50d *(required)* | q704_M50d. Please specify the year of the purchase price.<br>*Question relevant when: not(selected( ${q104_M50_full_list} , '6')) and not(selected( ${q104_M50_full_list} , '99'))*<br>*Response constrained to: .>=2000 and .<=2019 or .=999* | |
| q704_M51 | ***q704_M51. Povidone-iodine solution, 7.5%***<br>*Question relevant when: not(selected( ${q104_M51_full_list} , '6')) and not(selected( ${q104_M51_full_list} , '99'))* | |
| q704_M51a *(required)* | q704_M51a. When purchased, how many units of this item typically come in one pack, box, bottle, etc.? (e.g. 100 tablets, 1000 ml's, etc.) (Write 1 if purchased as a single item.)<br>*Smallest units listed were: "[q204_M51c]" , "[q304_M51c]" , "[q404_M51c]" , "[q504_M51c]" , "[q604_M51c]"*<br>*Question relevant when: not(selected( ${q104_M51_full_list} , '6')) and not(selected( ${q104_M51_full_list} , '99'))*<br>*Response constrained to: .>=0* | |
| q704_M51a.1 *(required)* | q704_M51a.1 What is the typical size of each item in the pack, box, etc. (e.g. 1 litre bottles, 250 mg tablets, 3 ml ampoules, etc.)?<br>*Please provide as much information as is available.*<br>*Question relevant when: not(selected( ${q104_M51_full_list} , '6')) and not(selected( ${q104_M51_full_list} , '99'))* | |
| q704_M51b *(required)* | q704_M51b. What is the typical purchase price for that quantity of units (i.e. the quantity noted in the question above)?<br>*Question relevant when: not(selected( ${q104_M51_full_list} , '6')) and not(selected( ${q104_M51_full_list} , '99'))*<br>*Response constrained to: .>=0* | |
| q704_M51c *(required)* | q704_M51c. Please specify the currency for the purchase price.<br>*Question relevant when: not(selected( ${q104_M51_full_list} , '6')) and not(selected( ${q104_M51_full_list} , '99'))* | 1 Tanzanian Shillings<br>2 US dollars<br>3 Euros<br>4 Other<br>99 Don't know |
| q704_M51c.1 | q704_M51c.1 If other currency, specify:<br>*Leave blank if not applicable*<br>*Question relevant when: not(selected( ${q104_M51_full_list} , '6')) and not(selected( ${q104_M51_full_list} , '99'))* | |
| q704_M51d *(required)* | q704_M51d. Please specify the year of the purchase price.<br>*Question relevant when: not(selected( ${q104_M51_full_list} , '6')) and not(selected( ${q104_M51_full_list} , '99'))*<br>*Response constrained to: .>=2000 and .<=2019 or .=999* | |
| q704_M52 | ***q704_M52. Antiseptic - other 1: "[q104_M52_full_list_other]"***<br>*Question relevant when: not(selected( ${q104_M52_full_list} , '6')) and not(selected( ${q104_M52_full_list} , '99')) and string-length( ${q104_M52_full_list_other} ) >0* | |
| q704_M52a *(required)* | q704_M52a. When purchased, how many units of this item typically come in one pack, box, bottle, etc.? (e.g. 100 tablets, 1000 ml's, etc.) (Write 1 if purchased as a single item.)<br>*Smallest units listed were: "[q204_M52c]" , "[q304_M52c]" , "[q404_M52c]" , "[q504_M52c]" , "[q604_M52c]"*<br>*Question relevant when: not(selected( ${q104_M52_full_list} , '6')) and not(selected( ${q104_M52_full_list} , '99')) and string-length( ${q104_M52_full_list_other} ) >0*<br>*Response constrained to: .>=0* | |
| q704_M52a.1 *(required)* | q704_M52a.1 What is the typical size of each item in the pack, box, etc. (e.g. 1 litre bottles, 250 mg tablets, 3 ml ampoules, etc.)?<br>*Please provide as much information as is available.*<br>*Question relevant when: not(selected( ${q104_M52_full_list} , '6')) and not(selected( ${q104_M52_full_list} , '99')) and string-length( ${q104_M52_full_list_other} ) >0* | |
| q704_M52b *(required)* | q704_M52b. What is the typical purchase price for that quantity of units (i.e. the quantity noted in the question above)?<br>*Question relevant when: not(selected( ${q104_M52_full_list} , '6')) and not(selected( ${q104_M52_full_list} , '99')) and string-length( ${q104_M52_full_list_other} ) >0*<br>*Response constrained to: .>=0* | |
| q704_M52c *(required)* | q704_M52c. Please specify the currency for the purchase price.<br>*Question relevant when: not(selected( ${q104_M52_full_list} , '6')) and not(selected( ${q104_M52_full_list} , '99')) and string-length( ${q104_M52_full_list_other} ) >0* | 1 Tanzanian Shillings<br>2 US dollars<br>3 Euros<br>4 Other<br>99 Don't know |
| q704_M52c.1 | q704_M52c.1 If other currency, specify:<br>*Leave blank if not applicable*<br>*Question relevant when: not(selected( ${q104_M52_full_list} , '6')) and not(selected( ${q104_M52_full_list}* | |

| Field | Question | Answer |
|---|---|---|
| | *Question relevant when: not(selected( ${q104_M52_full_list} , '6')) and not(selected( ${q104_M52_full_list} ,*<br>*'99')) and string-length( ${q104_M52_full_list_other} ) >0* | |
| q704_M52d *(required)* | q704_M52d. Please specify the year of the purchase price.<br>*Question relevant when: not(selected( ${q104_M52_full_list} , '6')) and not(selected( ${q104_M52_full_list} , '99')) and string-length( ${q104_M52_full_list_other} ) >0*<br>*Response constrained to: .>=2000 and .<=2019 or .=999* | |
| q704_M53 | ***q704_M53. Antiseptic - other 2: "[q104_M53_full_list_other]"***<br>*Question relevant when: not(selected( ${q104_M53_full_list} , '6')) and not(selected( ${q104_M53_full_list} , '99')) and string-length( ${q104_M53_full_list_other} ) >0* | |
| q704_M53a *(required)* | q704_M53a. When purchased, how many units of this item typically come in one pack, box, bottle, etc.? (e.g. 100 tablets, 1000 ml's, etc.) (Write 1 if purchased as a single item.)<br>*Smallest units listed were: "[q204_M53c]" , "[q304_M53c]" , "[q404_M53c]" , "[q504_M53c]" , "[q604_M53c]"*<br>*Question relevant when: not(selected( ${q104_M53_full_list} , '6')) and not(selected( ${q104_M53_full_list} , '99')) and string-length( ${q104_M53_full_list_other} ) >0*<br>*Response constrained to: .>=0* | |
| q704_M53a.1 *(required)* | q704_M53a.1 What is the typical size of each item in the pack, box, etc. (e.g. 1 litre bottles, 250 mg tablets, 3 ml ampoules, etc.)?<br>*Please provide as much information as is available.*<br>*Question relevant when: not(selected( ${q104_M53_full_list} , '6')) and not(selected( ${q104_M53_full_list} , '99')) and string-length( ${q104_M53_full_list_other} ) >0* | |

| | | |
|---|---|---|
| q704_M53b *(required)* | q704_M53b. What is the typical purchase price for that quantity of units (i.e. the quantity noted in the question above)?<br>*Question relevant when: not(selected( ${q104_M53_full_list} , '6')) and not(selected( ${q104_M53_full_list} , '99')) and string-length( ${q104_M53_full_list_other} ) >0*<br>*Response constrained to: .>=0* | |
| q704_M53c *(required)* | q704_M53c. Please specify the currency for the purchase price.<br>*Question relevant when: not(selected( ${q104_M53_full_list} , '6')) and not(selected( ${q104_M53_full_list} , '99')) and string-length( ${q104_M53_full_list_other} ) >0* | 1 Tanzanian Shillings<br>2 US dollars<br>3 Euros<br>4 Other<br>99 Don't know |
| q704_M53c.1 | q704_M53c.1 If other currency, specify:<br>*Leave blank if not applicable*<br>*Question relevant when: not(selected( ${q104_M53_full_list} , '6')) and not(selected( ${q104_M53_full_list} , '99')) and string-length( ${q104_M53_full_list_other} ) >0* | |
| q704_M53d *(required)* | q704_M53d. Please specify the year of the purchase price.<br>*Question relevant when: not(selected( ${q104_M53_full_list} , '6')) and not(selected( ${q104_M53_full_list} , '99')) and string-length( ${q104_M53_full_list_other} ) >0*<br>*Response constrained to: .>=2000 and .<=2019 or .=999* | |
| q704_M54 | ***q704_M54. Antiseptic - other 3: "[q104_M54_full_list_other]"***<br>*Question relevant when: not(selected( ${q104_M54_full_list} , '6')) and not(selected( ${q104_M54_full_list} , '99')) and string-length( ${q104_M54_full_list_other} ) >0* | |
| q704_M54a *(required)* | q704_M54a. When purchased, how many units of this item typically come in one pack, box, bottle, etc.? (e.g. 100 tablets, 1000 ml's, etc.) (Write 1 if purchased as a single item.)<br>*Smallest units listed were: "[q204_M54c]" , "[q304_M54c]" , "[q404_M54c]" , "[q504_M54c]" , "[q604_M54c]"*<br>*Question relevant when: not(selected( ${q104_M54_full_list} , '6')) and not(selected( ${q104_M54_full_list} , '99')) and string-length( ${q104_M54_full_list_other} ) >0*<br>*Response constrained to: .>=0* | |
| q704_M54a.1 *(required)* | q704_M54a.1 What is the typical size of each item in the pack, box, etc. (e.g. 1 litre bottles, 250 mg tablets, 3 ml ampoules, etc.)?<br>*Please provide as much information as is available.*<br>*Question relevant when: not(selected( ${q104_M54_full_list} , '6')) and not(selected( ${q104_M54_full_list} , '99')) and string-length( ${q104_M54_full_list_other} ) >0* | |
| q704_M54b *(required)* | q704_M54b. What is the typical purchase price for that quantity of units (i.e. the quantity noted in the question above)?<br>*Question relevant when: not(selected( ${q104_M54_full_list} , '6')) and not(selected( ${q104_M54_full_list} , '99')) and string-length( ${q104_M54_full_list_other} ) >0*<br>*Response constrained to: .>=0* | |
| q704_M54c *(required)* | q704_M54c. Please specify the currency for the purchase price.<br>*Question relevant when: not(selected( ${q104_M54_full_list} , '6')) and not(selected( ${q104_M54_full_list} , '99')) and string-length( ${q104_M54_full_list_other} ) >0* | 1 Tanzanian Shillings<br>2 US dollars<br>3 Euros<br>4 Other<br>99 Don't know |
| q704_M54c.1 | q704_M54c.1 If other currency, specify:<br>*Leave blank if not applicable*<br>*Question relevant when: not(selected( ${q104_M54_full_list} , '6')) and not(selected( ${q104_M54_full_list}* | |

| Field | Question | Answer |
|---|---|---|
| | *Question relevant when: not(selected( ${q104_M54_full_list} , '6')) and not(selected( ${q104_M54_full_list} , '99')) and string-length( ${q104_M54_full_list_other} ) >0* | |
| q704_M54d *(required)* | q704_M54d. Please specify the year of the purchase price.<br>*Question relevant when: not(selected( ${q104_M54_full_list} , '6')) and not(selected( ${q104_M54_full_list} , '99')) and string-length( ${q104_M54_full_list_other} ) >0*<br>*Response constrained to: .>=2000 and .<=2019 or .=999* | |
| q704_M54.1 | ***q704_M54.1. Antiseptic - other 3: "[q104_M54.1_full_list_other]"***<br>*Question relevant when: not(selected( ${q104_M54.1_full_list} , '6')) and not(selected( ${q104_M54.1_full_list} , '99')) and string-length( ${q104_M54.1_full_list_other} ) >0* | |
| q704_M54.1a *(required)* | q704_M54.1a. When purchased, how many units of this item typically come in one pack, box, bottle, etc.? (e.g. 100 tablets, 1000 ml's, etc.) (Write 1 if purchased as a single item.)<br>*Smallest units listed were: "[q204_M54.1c]" , "[q304_M54.1c]" , "[q404_M54.1c]" , "[q504_M54.1c]" , "[q604_M54.1c]"*<br>*Question relevant when: not(selected( ${q104_M54.1_full_list} , '6')) and not(selected( ${q104_M54.1_full_list} , '99')) and string-length( ${q104_M54.1_full_list_other} ) >0*<br>*Response constrained to: .>=0* | |
| q704_M54.1a.1 *(required)* | q704_M54.1a.1 What is the typical size of each item in the pack, box, etc. (e.g. 1 litre bottles, 250 mg tablets, 3 ml ampoules, etc.)?<br>*Please provide as much information as is available.*<br>*Question relevant when: not(selected( ${q104_M54.1_full_list} , '6')) and not(selected( ${q104_M54.1_full_list} , '99')) and string-length( ${q104_M54.1_full_list_other} ) >0* | |

| Field | Question | Answer |
|---|---|---|
| q704_M54.1b *(required)* | q704_M54.1b. What is the typical purchase price for that quantity of units (i.e. the quantity noted in the question above)?<br>*Question relevant when: not(selected( ${q104_M54.1_full_list} , '6')) and not(selected( ${q104_M54.1_full_list} , '99')) and string-length( ${q104_M54.1_full_list_other} ) >0*<br>*Response constrained to: .>=0* | |
| q704_M54.1c *(required)* | q704_M54.1c. Please specify the currency for the purchase price.<br>*Question relevant when: not(selected( ${q104_M54.1_full_list} , '6')) and not(selected( ${q104_M54.1_full_list} , '99')) and string-length( ${q104_M54.1_full_list_other} ) >0* | 1 Tanzanian Shillings<br>2 US dollars<br>3 Euros<br>4 Other<br>99 Don't know |
| q704_M54.1c.1 | q704_M54.1c.1 If other currency, specify:<br>*Leave blank if not applicable*<br>*Question relevant when: not(selected( ${q104_M54.1_full_list} , '6')) and not(selected( ${q104_M54.1_full_list} , '99')) and string-length( ${q104_M54.1_full_list_other} ) >0* | |
| q704_M54.1d *(required)* | q704_M54.1d. Please specify the year of the purchase price.<br>*Question relevant when: not(selected( ${q104_M54.1_full_list} , '6')) and not(selected( ${q104_M54.1_full_list} , '99')) and string-length( ${q104_M54.1_full_list_other} ) >0*<br>*Response constrained to: .>=2000 and .<=2019 or .=999* | |
| q704_M54.2 | ***q704_M54.2. Antiseptic - other 3: "[q104_M54.2_full_list_other]"***<br>*Question relevant when: not(selected( ${q104_M54.2_full_list} , '6')) and not(selected( ${q104_M54.2_full_list} , '99')) and string-length( ${q104_M54.2_full_list_other} ) >0* | |
| q704_M54.2a *(required)* | q704_M54.2a. When purchased, how many units of this item typically come in one pack, box, bottle, etc.? (e.g. 100 tablets, 1000 ml's, etc.) (Write 1 if purchased as a single item.)<br>*Smallest units listed were: "[q204_M54.2c]" , "[q304_M54.2c]" , "[q404_M54.2c]" , "[q504_M54.2c]" , "[q604_M54.2c]"*<br>*Question relevant when: not(selected( ${q104_M54.2_full_list} , '6')) and not(selected( ${q104_M54.2_full_list} , '99')) and string-length( ${q104_M54.2_full_list_other} ) >0*<br>*Response constrained to: .>=0* | |
| q704_M54.2a.1 *(required)* | q704_M54.2a.1 What is the typical size of each item in the pack, box, etc. (e.g. 1 litre bottles, 250 mg tablets, 3 ml ampoules, etc.)?<br>*Please provide as much information as is available.*<br>*Question relevant when: not(selected( ${q104_M54.2_full_list} , '6')) and not(selected( ${q104_M54.2_full_list} , '99')) and string-length( ${q104_M54.2_full_list_other} ) >0* | |
| q704_M54.2b *(required)* | q704_M54.2b. What is the typical purchase price for that quantity of units (i.e. the quantity noted in the question above)?<br>*Question relevant when: not(selected( ${q104_M54.2_full_list} , '6')) and not(selected( ${q104_M54.2_full_list} , '99')) and string-length( ${q104_M54.2_full_list_other} ) >0*<br>*Response constrained to: .>=0* | |
| q704_M54.2c *(required)* | q704_M54.2c. Please specify the currency for the purchase price.<br>*Question relevant when: not(selected( ${q104_M54.2_full_list} , '6')) and not(selected( ${q104_M54.2_full_list} , '99')) and string-length( ${q104_M54.2_full_list_other} ) >0* | 1 Tanzanian Shillings<br>2 US dollars<br>3 Euros<br>4 Other<br>99 Don't know |
| q704_M54.2c.1 | q704_M54.2c.1 If other currency, specify:<br>*Leave blank if not applicable*<br>*Question relevant when: not(selected( ${q104_M54.2_full_list} , '6')) and not(selected( ${q104_M54.2_full_list}* | |

'99')) and string-length( ${q104_M54.2_full_list_other} ) >0

| Field | Question | Answer |
|---|---|---|
| q704_M54.2d *(required)* | q704_M54.2d. Please specify the year of the purchase price.<br>*Question relevant when: not(selected( ${q104_M54.2_full_list} , '6')) and not(selected( ${q104_M54.2_full_list} , '99')) and string-length( ${q104_M54.2_full_list_other} ) >0*<br>*Response constrained to: .>=2000 and .<=2019 or .=999* | |
| D. Medications - Cost (5)<br>*Group relevant when: selected( ${section_seven_skip_med} , '1')* | | |
| q704_med_notE4 | 704 CONTINUED For each of the following medications and other medical products that are used for postabortion care, can you tell me …? | |
| note_704_antispas | ***Antispasmodic, anxiolytic, sedative, etc*** | |
| q704_M55 | ***q704_M55. Hyoscine butylbromide / scopolamine butilhisocin (Buscopan )***<br>*Question relevant when: not(selected( ${q104_M55_full_list} , '6')) and not(selected( ${q104_M55_full_list} , '99'))* | |
| q704_M55a *(required)* | q704_M55a. When purchased, how many units of this item typically come in one pack, box, bottle, etc.? (e.g. 100 tablets, 1000 ml's, etc.) (Write 1 if purchased as a single item.)<br>*Smallest units listed were: "[q204_M55c]" , "[q304_M55c]" , "[q404_M55c]" , "[q504_M55c]" , "[q604_M55c]"*<br>*Question relevant when: not(selected( ${q104_M55_full_list} , '6')) and not(selected( ${q104_M55_full_list} , '99'))*<br>*Response constrained to: .>=0* | |
| q704_M55a.1 *(required)* | q704_M55a.1 What is the typical size of each item in the pack, box, etc. (e.g. 1 litre bottles, 250 mg tablets, 3 ml ampoules, etc.)?<br>*Please provide as much information as is available.*<br>*Question relevant when: not(selected( ${q104_M55_full_list} , '6')) and not(selected( ${q104_M55_full_list} , '99'))* | |
| q704_M55b *(required)* | q704_M55b. What is the typical purchase price for that quantity of units (i.e. the quantity noted in the question above)?<br>*Question relevant when: not(selected( ${q104_M55_full_list} , '6')) and not(selected( ${q104_M55_full_list} , '99'))*<br>*Response constrained to: .>=0* | |
| q704_M55c *(required)* | q704_M55c. Please specify the currency for the purchase price.<br>*Question relevant when: not(selected( ${q104_M55_full_list} , '6')) and not(selected( ${q104_M55_full_list} , '99'))* | 1 Tanzanian Shillings<br>2 US dollars<br>3 Euros<br>4 Other<br>99 Don't know |
| q704_M55c.1 | q704_M55c.1 If other currency, specify:<br>*Leave blank if not applicable*<br>*Question relevant when: not(selected( ${q104_M55_full_list} , '6')) and not(selected( ${q104_M55_full_list} , '99'))* | |
| q704_M55d *(required)* | q704_M55d. Please specify the year of the purchase price.<br>*Question relevant when: not(selected( ${q104_M55_full_list} , '6')) and not(selected( ${q104_M55_full_list} , '99'))*<br>*Response constrained to: .>=2000 and .<=2019 or .=999* | |
| q704_M56 | ***q704_M56. Atropine sulfate (or sulphate)***<br>*Question relevant when: not(selected( ${q104_M56_full_list} , '6')) and not(selected( ${q104_M56_full_list} , '99'))* | |
| q704_M56a *(required)* | q704_M56a. When purchased, how many units of this item typically come in one pack, box, bottle, etc.? (e.g. 100 tablets, 1000 ml's, etc.) (Write 1 if purchased as a single item.)<br>*Smallest units listed were: "[q204_M56c]" , "[q304_M56c]" , "[q404_M56c]" , "[q504_M56c]" , "[q604_M56c]"*<br>*Question relevant when: not(selected( ${q104_M56_full_list} , '6')) and not(selected( ${q104_M56_full_list} , '99'))*<br>*Response constrained to: .>=0* | |
| q704_M56a.1 *(required)* | q704_M56a.1 What is the typical size of each item in the pack, box, etc. (e.g. 1 litre bottles, 250 mg tablets, 3 ml ampoules, etc.)?<br>*Please provide as much information as is available.*<br>*Question relevant when: not(selected( ${q104_M56_full_list} , '6')) and not(selected( ${q104_M56_full_list} , '99'))* | |
| q704_M56b *(required)* | q704_M56b. What is the typical purchase price for that quantity of units (i.e. the quantity noted in the question above)?<br>*Question relevant when: not(selected( ${q104_M56_full_list} , '6')) and not(selected( ${q104_M56_full_list} , '99'))*<br>*Response constrained to: .>=0* | |
| q704_M56c *(required)* | q704_M56c. Please specify the currency for the purchase price.<br>*Question relevant when: not(selected( ${q104_M56_full_list} , '6')) and not(selected( ${q104_M56_full_list} , '99'))* | 1 Tanzanian Shillings<br>2 US dollars<br>3 Euros |

| Field | Question | Answer |
|---|---|---|
| | | 4 Other<br>99 Don't know |
| q704_M56c.1 | q704_M56c.1 If other currency, specify:<br>*Leave blank if not applicable*<br>*Question relevant when: not(selected( ${q104_M56_full_list} , '6')) and not(selected( ${q104_M56_full_list} , '99'))* | |
| q704_M56d *(required)* | q704_M56d. Please specify the year of the purchase price.<br>*Question relevant when: not(selected( ${q104_M56_full_list} , '6')) and not(selected( ${q104_M56_full_list} , '99'))*<br>*Response constrained to: .>=2000 and .<=2019 or .=999* | |
| q704_M57 | ***q704_M57. Papaverine HCl***<br>*Question relevant when: not(selected( ${q104_M57_full_list} , '6')) and not(selected( ${q104_M57_full_list} , '99'))* | |
| q704_M57a *(required)* | q704_M57a. When purchased, how many units of this item typically come in one pack, box, bottle, etc.? (e.g. 100 tablets, 1000 ml's, etc.) (Write 1 if purchased as a single item.)<br>*Smallest units listed were: "[q204_M57c]" , "[q304_M57c]" , "[q404_M57c]" , "[q504_M57c]" , "[q604_M57c]"*<br>*Question relevant when: not(selected( ${q104_M57_full_list} , '6')) and not(selected( ${q104_M57_full_list} , '99'))*<br>*Response constrained to: .>=0* | |
| q704_M57a.1 *(required)* | q704_M57a.1 What is the typical size of each item in the pack, box, etc. (e.g. 1 litre bottles, 250 mg tablets, 3 ml ampoules, etc.)?<br>*Please provide as much information as is available.*<br>*Question relevant when: not(selected( ${q104_M57_full_list} , '6')) and not(selected( ${q104_M57_full_list} , '99'))* | |
| q704_M57b *(required)* | q704_M57b. What is the typical purchase price for that quantity of units (i.e. the quantity noted in the question above)?<br>*Question relevant when: not(selected( ${q104_M57_full_list} , '6')) and not(selected( ${q104_M57_full_list} , '99'))*<br>*Response constrained to: .>=0* | |
| q704_M57c *(required)* | q704_M57c. Please specify the currency for the purchase price.<br>*Question relevant when: not(selected( ${q104_M57_full_list} , '6')) and not(selected( ${q104_M57_full_list} , '99'))* | 1 Tanzanian Shillings<br>2 US dollars<br>3 Euros<br>4 Other<br>99 Don't know |
| q704_M57c.1 | q704_M57c.1 If other currency, specify:<br>*Leave blank if not applicable*<br>*Question relevant when: not(selected( ${q104_M57_full_list} , '6')) and not(selected( ${q104_M57_full_list} , '99'))* | |
| q704_M57d *(required)* | q704_M57d. Please specify the year of the purchase price.<br>*Question relevant when: not(selected( ${q104_M57_full_list} , '6')) and not(selected( ${q104_M57_full_list} , '99'))*<br>*Response constrained to: .>=2000 and .<=2019 or .=999* | |
| q704_M58 | ***q704_M58. Diazepam (injection)***<br>*Question relevant when: not(selected( ${q104_M58_full_list} , '6')) and not(selected( ${q104_M58_full_list} , '99'))* | |
| q704_M58a *(required)* | q704_M58a. When purchased, how many units of this item typically come in one pack, box, bottle, etc.? (e.g. 100 tablets, 1000 ml's, etc.) (Write 1 if purchased as a single item.)<br>*Smallest units listed were: "[q204_M58c]" , "[q304_M58c]" , "[q404_M58c]" , "[q504_M58c]" , "[q604_M58c]"*<br>*Question relevant when: not(selected( ${q104_M58_full_list} , '6')) and not(selected( ${q104_M58_full_list} , '99'))*<br>*Response constrained to: .>=0* | |
| q704_M58a.1 *(required)* | q704_M58a.1 What is the typical size of each item in the pack, box, etc. (e.g. 1 litre bottles, 250 mg tablets, 3 ml ampoules, etc.)?<br>*Please provide as much information as is available.*<br>*Question relevant when: not(selected( ${q104_M58_full_list} , '6')) and not(selected( ${q104_M58_full_list} , '99'))* | |
| q704_M58b *(required)* | q704_M58b. What is the typical purchase price for that quantity of units (i.e. the quantity noted in the question above)?<br>*Question relevant when: not(selected( ${q104_M58_full_list} , '6')) and not(selected( ${q104_M58_full_list} , '99'))*<br>*Response constrained to: .>=0* | |
| q704_M58c *(required)* | q704_M58c. Please specify the currency for the purchase price.<br>*Question relevant when: not(selected( ${q104_M58_full_list} , '6')) and not(selected( ${q104_M58_full_list} , '99'))* | 1 Tanzanian Shillings<br>2 US dollars<br>3 Euros |

| Field | Question | Answer |
|---|---|---|
| | | 4 Other<br>99 Don't know |
| q704_M58c.1 | q704_M58c.1 If other currency, specify:<br>*Leave blank if not applicable*<br>*Question relevant when: not(selected( ${q104_M58_full_list} , '6')) and not(selected( ${q104_M58_full_list} , '99'))* | |
| q704_M58d *(required)* | q704_M58d. Please specify the year of the purchase price.<br>*Question relevant when: not(selected( ${q104_M58_full_list} , '6')) and not(selected( ${q104_M58_full_list} , '99'))*<br>*Response constrained to: .>=2000 and .<=2019 or .=999* | |
| q704_M59 | ***q704_M59. Diazepam (oral)***<br>*Question relevant when: not(selected( ${q104_M59_full_list} , '6')) and not(selected( ${q104_M59_full_list} , '99'))* | |
| q704_M59a *(required)* | q704_M59a. When purchased, how many units of this item typically come in one pack, box, bottle, etc.? (e.g. 100 tablets, 1000 ml's, etc.) (Write 1 if purchased as a single item.)<br>*Smallest units listed were: "[q204_M59c]" , "[q304_M59c]" , "[q404_M59c]" , "[q504_M59c]" , "[q604_M59c]"*<br>*Question relevant when: not(selected( ${q104_M59_full_list} , '6')) and not(selected( ${q104_M59_full_list} , '99'))*<br>*Response constrained to: .>=0* | |

| | | |
|---|---|---|
| q704_M59a.1 *(required)* | q704_M59a.1 What is the typical size of each item in the pack, box, etc. (e.g. 1 litre bottles, 250 mg tablets, 3 ml ampoules, etc.)?<br>*Please provide as much information as is available.*<br>*Question relevant when: not(selected( ${q104_M59_full_list} , '6')) and not(selected( ${q104_M59_full_list} , '99'))* | |
| q704_M59b *(required)* | q704_M59b. What is the typical purchase price for that quantity of units (i.e. the quantity noted in the question above)?<br>*Question relevant when: not(selected( ${q104_M59_full_list} , '6')) and not(selected( ${q104_M59_full_list} , '99'))*<br>*Response constrained to: .>=0* | |
| q704_M59c *(required)* | q704_M59c. Please specify the currency for the purchase price.<br>*Question relevant when: not(selected( ${q104_M59_full_list} , '6')) and not(selected( ${q104_M59_full_list} , '99'))* | 1 Tanzanian Shillings<br>2 US dollars<br>3 Euros<br>4 Other<br>99 Don't know |
| q704_M59c.1 | q704_M59c.1 If other currency, specify:<br>*Leave blank if not applicable*<br>*Question relevant when: not(selected( ${q104_M59_full_list} , '6')) and not(selected( ${q104_M59_full_list} , '99'))* | |
| q704_M59d *(required)* | q704_M59d. Please specify the year of the purchase price.<br>*Question relevant when: not(selected( ${q104_M59_full_list} , '6')) and not(selected( ${q104_M59_full_list} , '99'))*<br>*Response constrained to: .>=2000 and .<=2019 or .=999* | |
| q704_M60 | ***q704_M60. Antispasmodic, anxiolytic, sedative, etc - Other 1: "[q104_M60_full_list_other]"***<br>*Question relevant when: not(selected( ${q104_M60_full_list} , '6')) and not(selected( ${q104_M60_full_list} , '99')) and string-length( ${q104_M60_full_list_other} ) >0* | |
| q704_M60a *(required)* | q704_M60a. When purchased, how many units of this item typically come in one pack, box, bottle, etc.? (e.g. 100 tablets, 1000 ml's, etc.) (Write 1 if purchased as a single item.)<br>*Smallest units listed were: "[q204_M60c]" , "[q304_M60c]" , "[q404_M60c]" , "[q504_M60c]" , "[q604_M60c]"*<br>*Question relevant when: not(selected( ${q104_M60_full_list} , '6')) and not(selected( ${q104_M60_full_list} , '99')) and string-length( ${q104_M60_full_list_other} ) >0*<br>*Response constrained to: .>=0* | |
| q704_M60a.1 *(required)* | q704_M60a.1 What is the typical size of each item in the pack, box, etc. (e.g. 1 litre bottles, 250 mg tablets, 3 ml ampoules, etc.)?<br>*Please provide as much information as is available.*<br>*Question relevant when: not(selected( ${q104_M60_full_list} , '6')) and not(selected( ${q104_M60_full_list} , '99')) and string-length( ${q104_M60_full_list_other} ) >0* | |
| q704_M60b *(required)* | q704_M60b. What is the typical purchase price for that quantity of units (i.e. the quantity noted in the question above)?<br>*Question relevant when: not(selected( ${q104_M60_full_list} , '6')) and not(selected( ${q104_M60_full_list} , '99')) and string-length( ${q104_M60_full_list_other} ) >0*<br>*Response constrained to: .>=0* | |
| q704_M60c *(required)* | q704_M60c. Please specify the currency for the purchase price.<br>*Question relevant when: not(selected( ${q104_M60_full_list} , '6')) and not(selected( ${q104_M60_full_list} , '99')) and string-length( ${q104_M60_full_list_other} ) >0* | 1 Tanzanian Shillings<br>2 US dollars<br>3 Euros |

| Field | Question | Answer |
|---|---|---|
| | | 4 Other<br>99 Don't know |
| q704_M60c.1 | q704_M60c.1 If other currency, specify:<br>*Leave blank if not applicable*<br>*Question relevant when: not(selected( ${q104_M60_full_list} , '6')) and not(selected( ${q104_M60_full_list} , '99')) and string-length( ${q104_M60_full_list_other} ) >0* | |
| q704_M60d *(required)* | q704_M60d. Please specify the year of the purchase price.<br>*Question relevant when: not(selected( ${q104_M60_full_list} , '6')) and not(selected( ${q104_M60_full_list} , '99')) and string-length( ${q104_M60_full_list_other} ) >0*<br>*Response constrained to: .>=2000 and .<=2019 or .=999* | |
| q704_M61 | ***q704_M61. Antispasmodic, anxiolytic, sedative, etc - Other 2: "[q104_M61_full_list_other]"***<br>*Question relevant when: not(selected( ${q104_M61_full_list} , '6')) and not(selected( ${q104_M61_full_list} , '99')) and string-length( ${q104_M61_full_list_other} ) >0* | |
| q704_M61a *(required)* | q704_M61a. When purchased, how many units of this item typically come in one pack, box, bottle, etc.? (e.g. 100 tablets, 1000 ml's, etc.) (Write 1 if purchased as a single item.)<br>*Smallest units listed were: "[q204_M61c]" , "[q304_M61c]" , "[q404_M61c]" , "[q504_M61c]" , "[q604_M61c]"*<br>*Question relevant when: not(selected( ${q104_M61_full_list} , '6')) and not(selected( ${q104_M61_full_list} , '99')) and string-length( ${q104_M61_full_list_other} ) >0*<br>*Response constrained to: .>=0* | |

| | | |
|---|---|---|
| q704_M61a.1 *(required)* | q704_M61a.1 What is the typical size of each item in the pack, box, etc. (e.g. 1 litre bottles, 250 mg tablets, 3 ml ampoules, etc.)?<br>*Please provide as much information as is available.*<br>*Question relevant when: not(selected( ${q104_M61_full_list} , '6')) and not(selected( ${q104_M61_full_list} , '99')) and string-length( ${q104_M61_full_list_other} ) >0* | |
| q704_M61b *(required)* | q704_M61b. What is the typical purchase price for that quantity of units (i.e. the quantity noted in the question above)?<br>*Question relevant when: not(selected( ${q104_M61_full_list} , '6')) and not(selected( ${q104_M61_full_list} , '99')) and string-length( ${q104_M61_full_list_other} ) >0*<br>*Response constrained to: .>=0* | |
| q704_M61c *(required)* | q704_M61c. Please specify the currency for the purchase price.<br>*Question relevant when: not(selected( ${q104_M61_full_list} , '6')) and not(selected( ${q104_M61_full_list} , '99')) and string-length( ${q104_M61_full_list_other} ) >0* | 1 Tanzanian Shillings<br>2 US dollars<br>3 Euros<br>4 Other<br>99 Don't know |
| q704_M61c.1 | q704_M61c.1 If other currency, specify:<br>*Leave blank if not applicable*<br>*Question relevant when: not(selected( ${q104_M61_full_list} , '6')) and not(selected( ${q104_M61_full_list} , '99')) and string-length( ${q104_M61_full_list_other} ) >0* | |
| q704_M61d *(required)* | q704_M61d. Please specify the year of the purchase price.<br>*Question relevant when: not(selected( ${q104_M61_full_list} , '6')) and not(selected( ${q104_M61_full_list} , '99')) and string-length( ${q104_M61_full_list_other} ) >0*<br>*Response constrained to: .>=2000 and .<=2019 or .=999* | |
| q704_M62 | ***q704_M62. Antispasmodic, anxiolytic, sedative, etc - Other 3: "[q104_M62_full_list_other]"***<br>*Question relevant when: not(selected( ${q104_M62_full_list} , '6')) and not(selected( ${q104_M62_full_list} , '99')) and string-length( ${q104_M62_full_list_other} ) >0* | |
| q704_M62a *(required)* | q704_M62a. When purchased, how many units of this item typically come in one pack, box, bottle, etc.? (e.g. 100 tablets, 1000 ml's, etc.) (Write 1 if purchased as a single item.)<br>*Smallest units listed were: "[q204_M62c]" , "[q304_M62c]" , "[q404_M62c]" , "[q504_M62c]" , "[q604_M62c]"*<br>*Question relevant when: not(selected( ${q104_M62_full_list} , '6')) and not(selected( ${q104_M62_full_list} , '99')) and string-length( ${q104_M62_full_list_other} ) >0*<br>*Response constrained to: .>=0* | |
| q704_M62a.1 *(required)* | q704_M62a.1 What is the typical size of each item in the pack, box, etc. (e.g. 1 litre bottles, 250 mg tablets, 3 ml ampoules, etc.)?<br>*Please provide as much information as is available.*<br>*Question relevant when: not(selected( ${q104_M62_full_list} , '6')) and not(selected( ${q104_M62_full_list} , '99')) and string-length( ${q104_M62_full_list_other} ) >0* | |
| q704_M62b *(required)* | q704_M62b. What is the typical purchase price for that quantity of units (i.e. the quantity noted in the question above)?<br>*Question relevant when: not(selected( ${q104_M62_full_list} , '6')) and not(selected( ${q104_M62_full_list} , '99')) and string-length( ${q104_M62_full_list_other} ) >0*<br>*Response constrained to: .>=0* | |
| q704_M62c *(required)* | q704_M62c. Please specify the currency for the purchase price.<br>*Question relevant when: not(selected( ${q104_M62_full_list} , '6')) and not(selected( ${q104_M62_full_list} , '99')) and string-length( ${q104_M62_full_list_other} ) >0* | 1 Tanzanian Shillings<br>2 US dollars<br>3 Euros |

| Field | Question | Answer |
|---|---|---|
| | | 4 Other<br>99 Don't know |
| q704_M62c.1 | q704_M62c.1 If other currency, specify:<br>*Leave blank if not applicable*<br>*Question relevant when: not(selected( ${q104_M62_full_list} , '6')) and not(selected( ${q104_M62_full_list} , '99')) and string-length( ${q104_M62_full_list_other} ) >0* | |
| q704_M62d *(required)* | q704_M62d. Please specify the year of the purchase price.<br>*Question relevant when: not(selected( ${q104_M62_full_list} , '6')) and not(selected( ${q104_M62_full_list} , '99')) and string-length( ${q104_M62_full_list_other} ) >0*<br>*Response constrained to: .>=2000 and .<=2019 or .=999* | |
| q704_M63 | ***q704_M63. Antispasmodic, anxiolytic, sedative, etc - Other 4: "[q104_M63_full_list_other]"***<br>*Question relevant when: not(selected( ${q104_M63_full_list} , '6')) and not(selected( ${q104_M63_full_list} , '99')) and string-length( ${q104_M63_full_list_other} ) >0* | |
| q704_M63a *(required)* | q704_M63a. When purchased, how many units of this item typically come in one pack, box, bottle, etc.? (e.g. 100 tablets, 1000 ml's, etc.) (Write 1 if purchased as a single item.)<br>*Smallest units listed were: "[q204_M63c]" , "[q304_M63c]" , "[q404_M63c]" , "[q504_M63c]" , "[q604_M63c]"*<br>*Question relevant when: not(selected( ${q104_M63_full_list} , '6')) and not(selected( ${q104_M63_full_list} , '99')) and string-length( ${q104_M63_full_list_other} ) >0*<br>*Response constrained to: .>=0* | |
| q704_M63a.1 *(required)* | q704_M63a.1 What is the typical size of each item in the pack, box, etc. (e.g. 1 litre bottles, 250 mg tablets, 3 ml ampoules, etc.)?<br>*Please provide as much information as is available.*<br>*Question relevant when: not(selected( ${q104_M63_full_list} , '6')) and not(selected( ${q104_M63_full_list} , '99')) and string-length( ${q104_M63_full_list_other} ) >0* | |
| q704_M63b *(required)* | q704_M63b. What is the typical purchase price for that quantity of units (i.e. the quantity noted in the question above)?<br>*Question relevant when: not(selected( ${q104_M63_full_list} , '6')) and not(selected( ${q104_M63_full_list} , '99')) and string-length( ${q104_M63_full_list_other} ) >0*<br>*Response constrained to: .>=0* | |
| q704_M63c *(required)* | q704_M63c. Please specify the currency for the purchase price.<br>*Question relevant when: not(selected( ${q104_M63_full_list} , '6')) and not(selected( ${q104_M63_full_list} , '99')) and string-length( ${q104_M63_full_list_other} ) >0* | 1 Tanzanian Shillings<br>2 US dollars<br>3 Euros<br>4 Other<br>99 Don't know |
| q704_M63c.1 | q704_M63c.1 If other currency, specify:<br>*Leave blank if not applicable*<br>*Question relevant when: not(selected( ${q104_M63_full_list} , '6')) and not(selected( ${q104_M63_full_list} , '99')) and string-length( ${q104_M63_full_list_other} ) >0* | |
| q704_M63d *(required)* | q704_M63d. Please specify the year of the purchase price.<br>*Question relevant when: not(selected( ${q104_M63_full_list} , '6')) and not(selected( ${q104_M63_full_list} , '99')) and string-length( ${q104_M63_full_list_other} ) >0*<br>*Response constrained to: .>=2000 and .<=2019 or .=999* | |
| q704_M64 | ***q704_M64. Antispasmodic, anxiolytic, sedative, etc - Other 5: "[q104_M64_full_list_other]"***<br>*Question relevant when: not(selected( ${q104_M64_full_list} , '6')) and not(selected( ${q104_M64_full_list} , '99')) and string-length( ${q104_M64_full_list_other} ) >0* | |
| q704_M64a *(required)* | q704_M64a. When purchased, how many units of this item typically come in one pack, box, bottle, etc.? (e.g. 100 tablets, 1000 ml's, etc.) (Write 1 if purchased as a single item.)<br>*Smallest units listed were: "[q204_M64c]" , "[q304_M64c]" , "[q404_M64c]" , "[q504_M64c]" , "[q604_M64c]"*<br>*Question relevant when: not(selected( ${q104_M64_full_list} , '6')) and not(selected( ${q104_M64_full_list} , '99')) and string-length( ${q104_M64_full_list_other} ) >0*<br>*Response constrained to: .>=0* | |
| q704_M64a.1 *(required)* | q704_M64a.1 What is the typical size of each item in the pack, box, etc. (e.g. 1 litre bottles, 250 mg tablets, 3 ml ampoules, etc.)?<br>*Please provide as much information as is available.*<br>*Question relevant when: not(selected( ${q104_M64_full_list} , '6')) and not(selected( ${q104_M64_full_list} , '99')) and string-length( ${q104_M64_full_list_other} ) >0* | |
| q704_M64b *(required)* | q704_M64b. What is the typical purchase price for that quantity of units (i.e. the quantity noted in the question above)?<br>*Question relevant when: not(selected( ${q104_M64_full_list} , '6')) and not(selected( ${q104_M64_full_list} , '99')) and string-length( ${q104_M64_full_list_other} ) >0*<br>*Response constrained to: .>=0* | |
| q704_M64c *(required)* | q704_M64c. Please specify the currency for the purchase price.<br>*Question relevant when: not(selected( ${q104_M64_full_list} , '6')) and not(selected( ${q104_M64_full_list} , '99')) and string-length( ${q104_M64_full_list_other} ) >0* | 1 Tanzanian Shillings<br>2 US dollars<br>3 Euros |

| Field | Question | Answer |
|---|---|---|
| | | 4 Other<br>99 Don't know |
| q704_M64c.1 | q704_M64c.1 If other currency, specify:<br>*Leave blank if not applicable*<br>*Question relevant when: not(selected( ${q104_M64_full_list} , '6')) and not(selected( ${q104_M64_full_list} , '99')) and string-length( ${q104_M64_full_list_other} ) >0* | |
| q704_M64d (required) | q704_M64d. Please specify the year of the purchase price.<br>*Question relevant when: not(selected( ${q104_M64_full_list} , '6')) and not(selected( ${q104_M64_full_list} , '99')) and string-length( ${q104_M64_full_list_other} ) >0*<br>*Response constrained to: .>=2000 and .<=2019 or .=999* | |
| D. Medications - Cost (6)<br>*Group relevant when: selected( ${section_seven_skip_med} , '1')* | | |
| q704_med_notE5 | 704 CONTINUED For each of the following medications and other medical products that are used for postabortion care, can you tell me …? | |
| note_704_adrenergic | ***Adrenergic*** | |
| q704_M65 | ***q704_M65. Ephedrine HCl***<br>*Question relevant when: not(selected( ${q104_M65_full_list} , '6')) and not(selected( ${q104_M65_full_list} , '99'))* | |

| | | |
|---|---|---|
| q704_M65a (required) | q704_M65a. When purchased, how many units of this item typically come in one pack, box, bottle, etc.? (e.g. 100 tablets, 1000 ml's, etc.) (Write 1 if purchased as a single item.)<br>*Smallest units listed were: "[q204_M65c]" , "[q304_M65c]" , "[q404_M65c]" , "[q504_M65c]" , "[q604_M65c]"*<br>*Question relevant when: not(selected( ${q104_M65_full_list} , '6')) and not(selected( ${q104_M65_full_list} , '99'))*<br>*Response constrained to: .>=0* | |
| q704_M65a.1 (required) | q704_M65a.1 What is the typical size of each item in the pack, box, etc. (e.g. 1 litre bottles, 250 mg tablets, 3 ml ampoules, etc.)?<br>*Please provide as much information as is available.*<br>*Question relevant when: not(selected( ${q104_M65_full_list} , '6')) and not(selected( ${q104_M65_full_list} , '99'))* | |
| q704_M65b (required) | q704_M65b. What is the typical purchase price for that quantity of units (i.e. the quantity noted in the question above)?<br>*Question relevant when: not(selected( ${q104_M65_full_list} , '6')) and not(selected( ${q104_M65_full_list} , '99'))*<br>*Response constrained to: .>=0* | |
| q704_M65c (required) | q704_M65c. Please specify the currency for the purchase price.<br>*Question relevant when: not(selected( ${q104_M65_full_list} , '6')) and not(selected( ${q104_M65_full_list} , '99'))* | 1 Tanzanian Shillings<br>2 US dollars<br>3 Euros<br>4 Other<br>99 Don't know |
| q704_M65c.1 | q704_M65c.1 If other currency, specify:<br>*Leave blank if not applicable*<br>*Question relevant when: not(selected( ${q104_M65_full_list} , '6')) and not(selected( ${q104_M65_full_list} , '99'))* | |
| q704_M65d (required) | q704_M65d. Please specify the year of the purchase price.<br>*Question relevant when: not(selected( ${q104_M65_full_list} , '6')) and not(selected( ${q104_M65_full_list} , '99'))*<br>*Response constrained to: .>=2000 and .<=2019 or .=999* | |
| q704_M66 | ***q704_M66. Epinephrine***<br>*Question relevant when: not(selected( ${q104_M66_full_list} , '6')) and not(selected( ${q104_M66_full_list} , '99'))* | |
| q704_M66a (required) | q704_M66a. When purchased, how many units of this item typically come in one pack, box, bottle, etc.? (e.g. 100 tablets, 1000 ml's, etc.) (Write 1 if purchased as a single item.)<br>*Smallest units listed were: "[q204_M66c]" , "[q304_M66c]" , "[q404_M66c]" , "[q504_M66c]" , "[q604_M66c]"*<br>*Question relevant when: not(selected( ${q104_M66_full_list} , '6')) and not(selected( ${q104_M66_full_list} , '99'))*<br>*Response constrained to: .>=0* | |
| q704_M66a.1 (required) | q704_M66a.1 What is the typical size of each item in the pack, box, etc. (e.g. 1 litre bottles, 250 mg tablets, 3 ml ampoules, etc.)?<br>*Please provide as much information as is available.*<br>*Question relevant when: not(selected( ${q104_M66_full_list} , '6')) and not(selected( ${q104_M66_full_list} , '99'))* | |
| q704_M66b (required) | q704_M66b. What is the typical purchase price for that quantity of units (i.e. the quantity noted in the question above)? | |

| Field | Question | Answer |
|---|---|---|
| | *Question relevant when: not(selected( ${q104_M66_full_list} , '6')) and not(selected( ${q104_M66_full_list} , '99'))* <br> *Response constrained to: .>=0* | |
| q704_M66c *(required)* | q704_M66c. Please specify the currency for the purchase price. <br> *Question relevant when: not(selected( ${q104_M66_full_list} , '6')) and not(selected( ${q104_M66_full_list} , '99'))* | 1 Tanzanian Shillings <br> 2 US dollars <br> 3 Euros <br> 4 Other <br> 99 Don't know |
| q704_M66c.1 | q704_M66c.1 If other currency, specify: <br> *Leave blank if not applicable* <br> *Question relevant when: not(selected( ${q104_M66_full_list} , '6')) and not(selected( ${q104_M66_full_list} , '99'))* | |
| q704_M66d *(required)* | q704_M66d. Please specify the year of the purchase price. <br> *Question relevant when: not(selected( ${q104_M66_full_list} , '6')) and not(selected( ${q104_M66_full_list} , '99'))* <br> *Response constrained to: .>=2000 and .<=2019 or .=999* | |
| q704_M67 | ***q704_M67. Adrenergic - other 1: "[q104_M67_full_list_other]"*** <br> *Question relevant when: not(selected( ${q104_M67_full_list} , '6')) and not(selected( ${q104_M67_full_list} , '99')) and string-length( ${q104_M67_full_list_other} ) >0* | |

| | | |
|---|---|---|
| q704_M67a *(required)* | q704_M67a. When purchased, how many units of this item typically come in one pack, box, bottle, etc.? (e.g. 100 tablets, 1000 ml's, etc.) (Write 1 if purchased as a single item.) <br> *Smallest units listed were: "[q204_M67c]" , "[q304_M67c]" , "[q404_M67c]" , "[q504_M67c]" , "[q604_M67c]"* <br> *Question relevant when: not(selected( ${q104_M67_full_list} , '6')) and not(selected( ${q104_M67_full_list} , '99')) and string-length( ${q104_M67_full_list_other} ) >0* <br> *Response constrained to: .>=0* | |
| q704_M67a.1 *(required)* | q704_M67a.1 What is the typical size of each item in the pack, box, etc. (e.g. 1 litre bottles, 250 mg tablets, 3 ml ampoules, etc.)? <br> *Please provide as much information as is available.* <br> *Question relevant when: not(selected( ${q104_M67_full_list} , '6')) and not(selected( ${q104_M67_full_list} , '99')) and string-length( ${q104_M67_full_list_other} ) >0* | |
| q704_M67b *(required)* | q704_M67b. What is the typical purchase price for that quantity of units (i.e. the quantity noted in the question above)? <br> *Question relevant when: not(selected( ${q104_M67_full_list} , '6')) and not(selected( ${q104_M67_full_list} , '99')) and string-length( ${q104_M67_full_list_other} ) >0* <br> *Response constrained to: .>=0* | |
| q704_M67c *(required)* | q704_M67c. Please specify the currency for the purchase price. <br> *Question relevant when: not(selected( ${q104_M67_full_list} , '6')) and not(selected( ${q104_M67_full_list} , '99')) and string-length( ${q104_M67_full_list_other} ) >0* | 1 Tanzanian Shillings <br> 2 US dollars <br> 3 Euros <br> 4 Other <br> 99 Don't know |
| q704_M67c.1 | q704_M67c.1 If other currency, specify: <br> *Leave blank if not applicable* <br> *Question relevant when: not(selected( ${q104_M67_full_list} , '6')) and not(selected( ${q104_M67_full_list} , '99')) and string-length( ${q104_M67_full_list_other} ) >0* | |
| q704_M67d *(required)* | q704_M67d. Please specify the year of the purchase price. <br> *Question relevant when: not(selected( ${q104_M67_full_list} , '6')) and not(selected( ${q104_M67_full_list} , '99')) and string-length( ${q104_M67_full_list_other} ) >0* <br> *Response constrained to: .>=2000 and .<=2019 or .=999* | |
| q704_M68 | ***q704_M68. Adrenergic - other 2: "[q104_M68_full_list_other]"*** <br> *Question relevant when: not(selected( ${q104_M68_full_list} , '6')) and not(selected( ${q104_M68_full_list} , '99')) and string-length( ${q104_M68_full_list_other} ) >0* | |
| q704_M68a *(required)* | q704_M68a. When purchased, how many units of this item typically come in one pack, box, bottle, etc.? (e.g. 100 tablets, 1000 ml's, etc.) (Write 1 if purchased as a single item.) <br> *Smallest units listed were: "[q204_M68c]" , "[q304_M68c]" , "[q404_M68c]" , "[q504_M68c]" , "[q604_M68c]"* <br> *Question relevant when: not(selected( ${q104_M68_full_list} , '6')) and not(selected( ${q104_M68_full_list} , '99')) and string-length( ${q104_M68_full_list_other} ) >0* <br> *Response constrained to: .>=0* | |
| q704_M68a.1 *(required)* | q704_M68a.1 What is the typical size of each item in the pack, box, etc. (e.g. 1 litre bottles, 250 mg tablets, 3 ml ampoules, etc.)? <br> *Please provide as much information as is available.* <br> *Question relevant when: not(selected( ${q104_M68_full_list} , '6')) and not(selected( ${q104_M68_full_list} , '99')) and string-length( ${q104_M68_full_list_other} ) >0* | |
| q704_M68b *(required)* | q704_M68b. What is the typical purchase price for that quantity of units (i.e. the quantity noted in the question above)? | |

| Field | Question | Answer |
|---|---|---|
| | *Question relevant when: not(selected( ${q104_M68_full_list} , '6')) and not(selected( ${q104_M68_full_list} , '99')) and string-length( ${q104_M68_full_list_other} ) >0*<br>*Response constrained to: .>=0* | |
| q704_M68c *(required)* | q704_M68c. Please specify the currency for the purchase price.<br>*Question relevant when: not(selected( ${q104_M68_full_list} , '6')) and not(selected( ${q104_M68_full_list} , '99')) and string-length( ${q104_M68_full_list_other} ) >0* | 1 Tanzanian Shillings<br>2 US dollars<br>3 Euros<br>4 Other<br>99 Don't know |
| q704_M68c.1 | q704_M68c.1 If other currency, specify:<br>*Leave blank if not applicable*<br>*Question relevant when: not(selected( ${q104_M68_full_list} , '6')) and not(selected( ${q104_M68_full_list} , '99')) and string-length( ${q104_M68_full_list_other} ) >0* | |
| q704_M68d *(required)* | q704_M68d. Please specify the year of the purchase price.<br>*Question relevant when: not(selected( ${q104_M68_full_list} , '6')) and not(selected( ${q104_M68_full_list} , '99')) and string-length( ${q104_M68_full_list_other} ) >0*<br>*Response constrained to: .>=2000 and .<=2019 or .=999* | |
| q704_M69 | ***q704_M69. Adrenergic - other 3: "[q104_M69_full_list_other]"***<br>*Question relevant when: not(selected( ${q104_M69_full_list} , '6')) and not(selected( ${q104_M69_full_list} , '99')) and string-length( ${q104_M69_full_list_other} ) >0* | |

| | | |
|---|---|---|
| q704_M69a *(required)* | q704_M69a. When purchased, how many units of this item typically come in one pack, box, bottle, etc.? (e.g. 100 tablets, 1000 ml's, etc.) (Write 1 if purchased as a single item.)<br>*Smallest units listed were: "[q204_M69c]" , "[q304_M69c]" , "[q404_M69c]" , "[q504_M69c]" , "[q604_M69c]"*<br>*Question relevant when: not(selected( ${q104_M69_full_list} , '6')) and not(selected( ${q104_M69_full_list} , '99')) and string-length( ${q104_M69_full_list_other} ) >0*<br>*Response constrained to: .>=0* | |
| q704_M69a.1 *(required)* | q704_M69a.1 What is the typical size of each item in the pack, box, etc. (e.g. 1 litre bottles, 250 mg tablets, 3 ml ampoules, etc.)?<br>*Please provide as much information as is available.*<br>*Question relevant when: not(selected( ${q104_M69_full_list} , '6')) and not(selected( ${q104_M69_full_list} , '99')) and string-length( ${q104_M69_full_list_other} ) >0* | |
| q704_M69b *(required)* | q704_M69b. What is the typical purchase price for that quantity of units (i.e. the quantity noted in the question above)?<br>*Question relevant when: not(selected( ${q104_M69_full_list} , '6')) and not(selected( ${q104_M69_full_list} , '99')) and string-length( ${q104_M69_full_list_other} ) >0*<br>*Response constrained to: .>=0* | |
| q704_M69c *(required)* | q704_M69c. Please specify the currency for the purchase price.<br>*Question relevant when: not(selected( ${q104_M69_full_list} , '6')) and not(selected( ${q104_M69_full_list} , '99')) and string-length( ${q104_M69_full_list_other} ) >0* | 1 Tanzanian Shillings<br>2 US dollars<br>3 Euros<br>4 Other<br>99 Don't know |
| q704_M69c.1 | q704_M69c.1 If other currency, specify:<br>*Leave blank if not applicable*<br>*Question relevant when: not(selected( ${q104_M69_full_list} , '6')) and not(selected( ${q104_M69_full_list} , '99')) and string-length( ${q104_M69_full_list_other} ) >0* | |
| q704_M69d *(required)* | q704_M69d. Please specify the year of the purchase price.<br>*Question relevant when: not(selected( ${q104_M69_full_list} , '6')) and not(selected( ${q104_M69_full_list} , '99')) and string-length( ${q104_M69_full_list_other} ) >0*<br>*Response constrained to: .>=2000 and .<=2019 or .=999* | |
| q704_M69.1 | ***q704_M69.1. Adrenergic - other 4: "[q104_M69.1_full_list_other]"***<br>*Question relevant when: not(selected( ${q104_M69.1_full_list} , '6')) and not(selected( ${q104_M69.1_full_list} , '99')) and string-length( ${q104_M69.1_full_list_other} ) >0* | |
| q704_M69.1a *(required)* | q704_M69.1a. When purchased, how many units of this item typically come in one pack, box, bottle, etc.? (e.g. 100 tablets, 1000 ml's, etc.) (Write 1 if purchased as a single item.)<br>*Smallest units listed were: "[q204_M69.1c]" , "[q304_M69.1c]" , "[q404_M69.1c]" , "[q504_M69.1c]" , "[q604_M69.1c]"*<br>*Question relevant when: not(selected( ${q104_M69.1_full_list} , '6')) and not(selected( ${q104_M69.1_full_list} , '99')) and string-length( ${q104_M69.1_full_list_other} ) >0*<br>*Response constrained to: .>=0* | |
| q704_M69.1a.1 *(required)* | q704_M69.1a.1 What is the typical size of each item in the pack, box, etc. (e.g. 1 litre bottles, 250 mg tablets, 3 ml ampoules, etc.)?<br>*Please provide as much information as is available.*<br>*Question relevant when: not(selected( ${q104_M69.1_full_list} , '6')) and not(selected( ${q104_M69.1_full_list} , '99')) and string-length( ${q104_M69.1_full_list_other} ) >0* | |
| q704_M69.1b *(required)* | q704_M69.1b. What is the typical purchase price for that quantity of units (i.e. the quantity noted in the question above)? | |

above)?

| Field | Question | Answer |
|---|---|---|
| | *Question relevant when: not(selected( ${q104_M69.1_full_list} , '6')) and not(selected( ${q104_M69.1_full_list} , '99')) and string-length( ${q104_M69.1_full_list_other} ) >0*<br>*Response constrained to: .>=0* | |
| q704_M69.1c *(required)* | q704_M69.1c. Please specify the currency for the purchase price.<br>*Question relevant when: not(selected( ${q104_M69.1_full_list} , '6')) and not(selected( ${q104_M69.1_full_list} , '99')) and string-length( ${q104_M69.1_full_list_other} ) >0* | 1 Tanzanian Shillings<br>2 US dollars<br>3 Euros<br>4 Other<br>99 Don't know |
| q704_M69..1 | q704_M69..1 If other currency, specify:<br>*Leave blank if not applicable*<br>*Question relevant when: not(selected( ${q104_M69.1_full_list} , '6')) and not(selected( ${q104_M69.1_full_list} , '99')) and string-length( ${q104_M69.1_full_list_other} ) >0* | |
| q704_M69.1d *(required)* | q704_M69.1d. Please specify the year of the purchase price.<br>*Question relevant when: not(selected( ${q104_M69.1_full_list} , '6')) and not(selected( ${q104_M69.1_full_list} , '99')) and string-length( ${q104_M69.1_full_list_other} ) >0*<br>*Response constrained to: .>=2000 and .<=2019 or .=999* | |
| q704_M69.2 | ***q704_M69.2. Adrenergic - other 5: "[q104_M69.2_full_list_other]"***<br>*Question relevant when: not(selected( ${q104_M69.2_full_list} , '6')) and not(selected( ${q104_M69.2_full_list} , '99')) and string-length( ${q104_M69.2_full_list_other} ) >0* | |
| q704_M69.2a *(required)* | q704_M69.2a. When purchased, how many units of this item typically come in one pack, box, bottle, etc.? (e.g. 100 tablets, 1000 ml's, etc.) (Write 1 if purchased as a single item.)<br>*Smallest units listed were: "[q204_M69.2c]" , "[q304_M69.2c]" , "[q404_M69.2c]" , "[q504_M69.2c]" , "[q604_M69.2c]"*<br>*Question relevant when: not(selected( ${q104_M69.2_full_list} , '6')) and not(selected( ${q104_M69.2_full_list} , '99')) and string-length( ${q104_M69.2_full_list_other} ) >0*<br>*Response constrained to: .>=0* | |
| q704_M69.2a.1 *(required)* | q704_M69.2a.1 What is the typical size of each item in the pack, box, etc. (e.g. 1 litre bottles, 250 mg tablets, 3 ml ampoules, etc.)?<br>*Please provide as much information as is available.*<br>*Question relevant when: not(selected( ${q104_M69.2_full_list} , '6')) and not(selected( ${q104_M69.2_full_list} , '99')) and string-length( ${q104_M69.2_full_list_other} ) >0* | |
| q704_M69.2b *(required)* | q704_M69.2b. What is the typical purchase price for that quantity of units (i.e. the quantity noted in the question above)?<br>*Question relevant when: not(selected( ${q104_M69.2_full_list} , '6')) and not(selected( ${q104_M69.2_full_list} , '99')) and string-length( ${q104_M69.2_full_list_other} ) >0*<br>*Response constrained to: .>=0* | |
| q704_M69.2c *(required)* | q704_M69.2c. Please specify the currency for the purchase price.<br>*Question relevant when: not(selected( ${q104_M69.2_full_list} , '6')) and not(selected( ${q104_M69.2_full_list} , '99')) and string-length( ${q104_M69.2_full_list_other} ) >0* | 1 Tanzanian Shillings<br>2 US dollars<br>3 Euros<br>4 Other<br>99 Don't know |
| q704_M69.2c.1 | q704_M69.2c.1 If other currency, specify:<br>*Leave blank if not applicable*<br>*Question relevant when: not(selected( ${q104_M69.2_full_list} , '6')) and not(selected( ${q104_M69.2_full_list} , '99')) and string-length( ${q104_M69.2_full_list_other} ) >0* | |
| q704_M69.2d *(required)* | q704_M69.2d. Please specify the year of the purchase price.<br>*Question relevant when: not(selected( ${q104_M69.2_full_list} , '6')) and not(selected( ${q104_M69.2_full_list} , '99')) and string-length( ${q104_M69.2_full_list_other} ) >0*<br>*Response constrained to: .>=2000 and .<=2019 or .=999* | |
| D. Medications - Cost (7)<br>*Group relevant when: selected( ${section_seven_skip_med} , '1')* | | |
| q704_med_notE6 | 704 CONTINUED For each of the following medications and other medical products that are used for postabortion care, can you tell me …? | |
| note_704_blood | ***Blood products*** | |
| q704_M70 | ***q704_M70. Unit of packed red blood cells***<br>*Question relevant when: not(selected( ${q104_M70_full_list} , '6')) and not(selected( ${q104_M70_full_list} , '99'))* | |
| q704_M70a *(required)* | q704_M70a. When purchased, how many units of this item typically come in one pack, box, bottle, etc.? (e.g. 100 tablets, 1000 ml's, etc.) (Write 1 if purchased as a single item.)<br>*Smallest units listed were: "[q204_M70c]" , "[q304_M70c]" , "[q404_M70c]" , "[q504_M70c]" , "[q604_M70c]"*<br>*Question relevant when: not(selected( ${q104_M70_full_list} , '6')) and not(selected( ${q104_M70_full_list} , '99'))*<br>*Response constrained to: .>=0* | |
| q704_M70a.1 *(required)* | q704_M70a.1 What is the typical size of each item in the pack, box, etc. (e.g. 1 litre bottles, 250 mg tablets, 3 ml | |

| Field | Question | Answer |
|---|---|---|
| | ampoules, etc.)?<br>*Please provide as much information as is available.*<br>*Question relevant when: not(selected( ${q104_M70_full_list} , '6')) and not(selected( ${q104_M70_full_list} , '99'))* | |
| q704_M70b *(required)* | q704_M70b. What is the typical purchase price for that quantity of units (i.e. the quantity noted in the question above)?<br>*Question relevant when: not(selected( ${q104_M70_full_list} , '6')) and not(selected( ${q104_M70_full_list} , '99'))*<br>*Response constrained to: .>=0* | |
| q704_M70c *(required)* | q704_M70c. Please specify the currency for the purchase price.<br>*Question relevant when: not(selected( ${q104_M70_full_list} , '6')) and not(selected( ${q104_M70_full_list} , '99'))* | 1 Tanzanian Shillings<br>2 US dollars<br>3 Euros<br>4 Other<br>99 Don't know |
| q704_M70c.1 | q704_M70c.1 If other currency, specify:<br>*Leave blank if not applicable*<br>*Question relevant when: not(selected( ${q104_M70_full_list} , '6')) and not(selected( ${q104_M70_full_list} , '99'))* | |

| Field | Question | Answer |
|---|---|---|
| q704_M70d *(required)* | q704_M70d. Please specify the year of the purchase price.<br>*Question relevant when: not(selected( ${q104_M70_full_list} , '6')) and not(selected( ${q104_M70_full_list} , '99'))*<br>*Response constrained to: .>=2000 and .<=2019 or .=999* | |
| q704_M71 | ***q704_M71. Unit of platelets***<br>*Question relevant when: not(selected( ${q104_M71_full_list} , '6')) and not(selected( ${q104_M71_full_list} , '99'))* | |
| q704_M71a *(required)* | q704_M71a. When purchased, how many units of this item typically come in one pack, box, bottle, etc.? (e.g. 100 tablets, 1000 ml's, etc.) (Write 1 if purchased as a single item.)<br>*Smallest units listed were: "[q204_M71c]" , "[q304_M71c]" , "[q404_M71c]" , "[q504_M71c]" , "[q604_M71c]"*<br>*Question relevant when: not(selected( ${q104_M71_full_list} , '6')) and not(selected( ${q104_M71_full_list} , '99'))*<br>*Response constrained to: .>=0* | |
| q704_M71a.1 *(required)* | q704_M71a.1 What is the typical size of each item in the pack, box, etc. (e.g. 1 litre bottles, 250 mg tablets, 3 ml ampoules, etc.)?<br>*Please provide as much information as is available.*<br>*Question relevant when: not(selected( ${q104_M71_full_list} , '6')) and not(selected( ${q104_M71_full_list} , '99'))* | |
| q704_M71b *(required)* | q704_M71b. What is the typical purchase price for that quantity of units (i.e. the quantity noted in the question above)?<br>*Question relevant when: not(selected( ${q104_M71_full_list} , '6')) and not(selected( ${q104_M71_full_list} , '99'))*<br>*Response constrained to: .>=0* | |
| q704_M71c *(required)* | q704_M71c. Please specify the currency for the purchase price.<br>*Question relevant when: not(selected( ${q104_M71_full_list} , '6')) and not(selected( ${q104_M71_full_list} , '99'))* | 1 Tanzanian Shillings<br>2 US dollars<br>3 Euros<br>4 Other<br>99 Don't know |
| q704_M71c.1 | q704_M71c.1 If other currency, specify:<br>*Leave blank if not applicable*<br>*Question relevant when: not(selected( ${q104_M71_full_list} , '6')) and not(selected( ${q104_M71_full_list} , '99'))* | |
| q704_M71d *(required)* | q704_M71d. Please specify the year of the purchase price.<br>*Question relevant when: not(selected( ${q104_M71_full_list} , '6')) and not(selected( ${q104_M71_full_list} , '99'))*<br>*Response constrained to: .>=2000 and .<=2019 or .=999* | |
| q704_M72 | ***q704_M72. Unit of whole blood***<br>*Question relevant when: not(selected( ${q104_M72_full_list} , '6')) and not(selected( ${q104_M72_full_list} , '99'))* | |
| q704_M72a *(required)* | q704_M72a. When purchased, how many units of this item typically come in one pack, box, bottle, etc.? (e.g. 100 tablets, 1000 ml's, etc.) (Write 1 if purchased as a single item.)<br>*Smallest units listed were: "[q204_M72c]" , "[q304_M72c]" , "[q404_M72c]" , "[q504_M72c]" , "[q604_M72c]"*<br>*Question relevant when: not(selected( ${q104_M72_full_list} , '6')) and not(selected( ${q104_M72_full_list} , '99'))* | |

| Field | Question | Answer |
|---|---|---|
| | *Response constrained to: .>=0* | |
| q704_M72a.1 (required) | q704_M72a.1 What is the typical size of each item in the pack, box, etc. (e.g. 1 litre bottles, 250 mg tablets, 3 ml ampoules, etc.)?<br>*Please provide as much information as is available.*<br>*Question relevant when: not(selected( ${q104_M72_full_list} , '6')) and not(selected( ${q104_M72_full_list} , '99'))* | |
| q704_M72b (required) | q704_M72b. What is the typical purchase price for that quantity of units (i.e. the quantity noted in the question above)?<br>*Question relevant when: not(selected( ${q104_M72_full_list} , '6')) and not(selected( ${q104_M72_full_list} , '99'))*<br>*Response constrained to: .>=0* | |
| q704_M72c (required) | q704_M72c. Please specify the currency for the purchase price.<br>*Question relevant when: not(selected( ${q104_M72_full_list} , '6')) and not(selected( ${q104_M72_full_list} , '99'))* | 1 Tanzanian Shillings<br>2 US dollars<br>3 Euros<br>4 Other<br>99 Don't know |
| q704_M72c.1 | q704_M72c.1 If other currency, specify:<br>*Leave blank if not applicable*<br>*Question relevant when: not(selected( ${q104_M72_full_list} , '6')) and not(selected( ${q104_M72_full_list} , '99'))* | |
| q704_M72d (required) | q704_M72d. Please specify the year of the purchase price.<br>*Question relevant when: not(selected( ${q104_M72_full_list} , '6')) and not(selected( ${q104_M72_full_list} , '99'))*<br>*Response constrained to: .>=2000 and .<=2019 or .=999* | |
| q704_M73 | ***q704_M73. Blood product - other 1: "[q104_M73_full_list_other]"***<br>*Question relevant when: not(selected( ${q104_M73_full_list} , '6')) and not(selected( ${q104_M73_full_list} , '99')) and string-length( ${q104_M73_full_list_other} ) >0* | |
| q704_M73a (required) | q704_M73a. When purchased, how many units of this item typically come in one pack, box, bottle, etc.? (e.g. 100 tablets, 1000 ml's, etc.) (Write 1 if purchased as a single item.)<br>*Smallest units listed were: "[q204_M73c]" , "[q304_M73c]" , "[q404_M73c]" , "[q504_M73c]" , "[q604_M73c]"*<br>*Question relevant when: not(selected( ${q104_M73_full_list} , '6')) and not(selected( ${q104_M73_full_list} , '99')) and string-length( ${q104_M73_full_list_other} ) >0*<br>*Response constrained to: .>=0* | |
| q704_M73a.1 (required) | q704_M73a.1 What is the typical size of each item in the pack, box, etc. (e.g. 1 litre bottles, 250 mg tablets, 3 ml ampoules, etc.)?<br>*Please provide as much information as is available.*<br>*Question relevant when: not(selected( ${q104_M73_full_list} , '6')) and not(selected( ${q104_M73_full_list} , '99')) and string-length( ${q104_M73_full_list_other} ) >0* | |
| q704_M73b (required) | q704_M73b. What is the typical purchase price for that quantity of units (i.e. the quantity noted in the question above)?<br>*Question relevant when: not(selected( ${q104_M73_full_list} , '6')) and not(selected( ${q104_M73_full_list} , '99')) and string-length( ${q104_M73_full_list_other} ) >0*<br>*Response constrained to: .>=0* | |
| q704_M73c (required) | q704_M73c. Please specify the currency for the purchase price.<br>*Question relevant when: not(selected( ${q104_M73_full_list} , '6')) and not(selected( ${q104_M73_full_list} , '99')) and string-length( ${q104_M73_full_list_other} ) >0* | 1 Tanzanian Shillings<br>2 US dollars<br>3 Euros<br>4 Other<br>99 Don't know |
| q704_M73c.1 | q704_M73c.1 If other currency, specify:<br>*Leave blank if not applicable*<br>*Question relevant when: not(selected( ${q104_M73_full_list} , '6')) and not(selected( ${q104_M73_full_list} , '99')) and string-length( ${q104_M73_full_list_other} ) >0* | |
| q704_M73d (required) | q704_M73d. Please specify the year of the purchase price.<br>*Question relevant when: not(selected( ${q104_M73_full_list} , '6')) and not(selected( ${q104_M73_full_list} , '99')) and string-length( ${q104_M73_full_list_other} ) >0*<br>*Response constrained to: .>=2000 and .<=2019 or .=999* | |
| q704_M74 | ***q704_M74. Blood product - other 2: "[q104_M74_full_list_other]"***<br>*Question relevant when: not(selected( ${q104_M74_full_list} , '6')) and not(selected( ${q104_M74_full_list} , '99')) and string-length( ${q104_M74_full_list_other} ) >0* | |
| q704_M74a (required) | q704_M74a. When purchased, how many units of this item typically come in one pack, box, bottle, etc.? (e.g. 100 tablets, 1000 ml's, etc.) (Write 1 if purchased as a single item.)<br>*Smallest units listed were: "[q204_M74c]" , "[q304_M74c]" , "[q404_M74c]" , "[q504_M74c]" , "[q604_M74c]"*<br>*Question relevant when: not(selected( ${q104_M74_full_list} , '6')) and not(selected( ${q104_M74_full_list} , '99')) and string-length( ${q104_M74_full_list_other} ) >0*<br>[illegible] | |

*Response constrained to: .>=0*

| Field | Question | Answer |
|---|---|---|
| q704_M74a.1 *(required)* | q704_M74a.1 What is the typical size of each item in the pack, box, etc. (e.g. 1 litre bottles, 250 mg tablets, 3 ml ampoules, etc.)?<br>*Please provide as much information as is available.*<br>*Question relevant when: not(selected( ${q104_M74_full_list} , '6')) and not(selected( ${q104_M74_full_list} , '99')) and string-length( ${q104_M74_full_list_other} ) >0* | |
| q704_M74b *(required)* | q704_M74b. What is the typical purchase price for that quantity of units (i.e. the quantity noted in the question above)?<br>*Question relevant when: not(selected( ${q104_M74_full_list} , '6')) and not(selected( ${q104_M74_full_list} , '99')) and string-length( ${q104_M74_full_list_other} ) >0*<br>*Response constrained to: .>=0* | |
| q704_M74c *(required)* | q704_M74c. Please specify the currency for the purchase price.<br>*Question relevant when: not(selected( ${q104_M74_full_list} , '6')) and not(selected( ${q104_M74_full_list} , '99')) and string-length( ${q104_M74_full_list_other} ) >0* | 1 Tanzanian Shillings<br>2 US dollars<br>3 Euros<br>4 Other<br>99 Don't know |
| q704_M74c.1 | q704_M74c.1 If other currency, specify:<br>*Leave blank if not applicable*<br>*Question relevant when: not(selected( ${q104_M74_full_list} , '6')) and not(selected( ${q104_M74_full_list} , '99')) and string-length( ${q104_M74_full_list_other} ) >0* | |

| | | |
|---|---|---|
| q704_M74d *(required)* | q704_M74d. Please specify the year of the purchase price.<br>*Question relevant when: not(selected( ${q104_M74_full_list} , '6')) and not(selected( ${q104_M74_full_list} , '99')) and string-length( ${q104_M74_full_list_other} ) >0*<br>*Response constrained to: .>=2000 and .<=2019 or .=999* | |
| q704_M75 | ***q704_M75. Blood product - other 3: "[q104_M75_full_list_other]"***<br>*Question relevant when: not(selected( ${q104_M75_full_list} , '6')) and not(selected( ${q104_M75_full_list} , '99')) and string-length( ${q104_M75_full_list_other} ) >0* | |
| q704_M75a *(required)* | q704_M75a. When purchased, how many units of this item typically come in one pack, box, bottle, etc.? (e.g. 100 tablets, 1000 ml's, etc.) (Write 1 if purchased as a single item.)<br>*Smallest units listed were: "[q204_M75c]" , "[q304_M75c]" , "[q404_M75c]" , "[q504_M75c]" , "[q604_M75c]"*<br>*Question relevant when: not(selected( ${q104_M75_full_list} , '6')) and not(selected( ${q104_M75_full_list} , '99')) and string-length( ${q104_M75_full_list_other} ) >0*<br>*Response constrained to: .>=0* | |
| q704_M75a.1 *(required)* | q704_M75a.1 What is the typical size of each item in the pack, box, etc. (e.g. 1 litre bottles, 250 mg tablets, 3 ml ampoules, etc.)?<br>*Please provide as much information as is available.*<br>*Question relevant when: not(selected( ${q104_M75_full_list} , '6')) and not(selected( ${q104_M75_full_list} , '99')) and string-length( ${q104_M75_full_list_other} ) >0* | |
| q704_M75b *(required)* | q704_M75b. What is the typical purchase price for that quantity of units (i.e. the quantity noted in the question above)?<br>*Question relevant when: not(selected( ${q104_M75_full_list} , '6')) and not(selected( ${q104_M75_full_list} , '99')) and string-length( ${q104_M75_full_list_other} ) >0*<br>*Response constrained to: .>=0* | |
| q704_M75c *(required)* | q704_M75c. Please specify the currency for the purchase price.<br>*Question relevant when: not(selected( ${q104_M75_full_list} , '6')) and not(selected( ${q104_M75_full_list} , '99')) and string-length( ${q104_M75_full_list_other} ) >0* | 1 Tanzanian Shillings<br>2 US dollars<br>3 Euros<br>4 Other<br>99 Don't know |
| q704_M75c.1 | q704_M75c.1 If other currency, specify:<br>*Leave blank if not applicable*<br>*Question relevant when: not(selected( ${q104_M75_full_list} , '6')) and not(selected( ${q104_M75_full_list} , '99')) and string-length( ${q104_M75_full_list_other} ) >0* | |
| q704_M75d *(required)* | q704_M75d. Please specify the year of the purchase price.<br>*Question relevant when: not(selected( ${q104_M75_full_list} , '6')) and not(selected( ${q104_M75_full_list} , '99')) and string-length( ${q104_M75_full_list_other} ) >0*<br>*Response constrained to: .>=2000 and .<=2019 or .=999* | |
| q704_M75.1 | ***q704_M75.1. Blood product - other 3: "[q104_M75.1_full_list_other]"***<br>*Question relevant when: not(selected( ${q104_M75.1_full_list} , '6')) and not(selected( ${q104_M75.1_full_list} , '99')) and string-length( ${q104_M75.1_full_list_other} ) >0* | |
| q704_M75.1a *(required)* | q704_M75.1a. When purchased, how many units of this item typically come in one pack, box, bottle, etc.? (e.g. 100 tablets, 1000 ml's, etc.) (Write 1 if purchased as a single item.)<br>*Smallest units listed were: "[q204_M75.1c]" , "[q304_M75.1c]" , "[q404_M75.1c]" , "[q504_M75.1c]" , "[q604_M75.1c]"*<br>*Question relevant when: not(selected( ${q104_M75.1_full_list} , '6')) and not(selected( ${q104_M75.1_full_list} , '99')) and string-length( ${q104_M75.1_full_list_other} ) >0*<br>[illegible] | |

| Field | Question | Answer |
|---|---|---|
| | *Response constrained to: .>=0* | |
| q704_M75.1a.1 *(required)* | q704_M75.1a.1 What is the typical size of each item in the pack, box, etc. (e.g. 1 litre bottles, 250 mg tablets, 3 ml ampoules, etc.)?<br>*Please provide as much information as is available.*<br>*Question relevant when: not(selected( ${q104_M75.1_full_list} , '6')) and not(selected( ${q104_M75.1_full_list} , '99')) and string-length( ${q104_M75.1_full_list_other} ) >0* | |
| q704_M75.1b *(required)* | q704_M75.1b. What is the typical purchase price for that quantity of units (i.e. the quantity noted in the question above)?<br>*Question relevant when: not(selected( ${q104_M75.1_full_list} , '6')) and not(selected( ${q104_M75.1_full_list} , '99')) and string-length( ${q104_M75.1_full_list_other} ) >0*<br>*Response constrained to: .>=0* | |
| q704_M75.1c *(required)* | q704_M75.1c. Please specify the currency for the purchase price.<br>*Question relevant when: not(selected( ${q104_M75.1_full_list} , '6')) and not(selected( ${q104_M75.1_full_list} , '99')) and string-length( ${q104_M75.1_full_list_other} ) >0* | 1 Tanzanian Shillings<br>2 US dollars<br>3 Euros<br>4 Other<br>99 Don't know |
| q704_M75.1c.1 | q704_M75.1c.1 If other currency, specify:<br>*Leave blank if not applicable*<br>*Question relevant when: not(selected( ${q104_M75.1_full_list} , '6')) and not(selected( ${q104_M75.1_full_list} , '99')) and string-length( ${q104_M75.1_full_list_other} ) >0* | |
| q704_M75.1d *(required)* | q704_M75.1d. Please specify the year of the purchase price.<br>*Question relevant when: not(selected( ${q104_M75.1_full_list} , '6')) and not(selected( ${q104_M75.1_full_list} , '99')) and string-length( ${q104_M75.1_full_list_other} ) >0*<br>*Response constrained to: .>=2000 and .<=2019 or .=999* | |
| q704_M75.2 | ***q704_M75.2. Blood product - other 3: "[q104_M75.2_full_list_other]"***<br>*Question relevant when: not(selected( ${q104_M75.2_full_list} , '6')) and not(selected( ${q104_M75.2_full_list} , '99')) and string-length( ${q104_M75.2_full_list_other} ) >0* | |
| q704_M75.2a *(required)* | q704_M75.2a. When purchased, how many units of this item typically come in one pack, box, bottle, etc.? (e.g. 100 tablets, 1000 ml's, etc.) (Write 1 if purchased as a single item.)<br>*Smallest units listed were: "[q204_M75.2c]" , "[q304_M75.2c]" , "[q404_M75.2c]" , "[q504_M75.2c]" , "[q604_M75.2c]"*<br>*Question relevant when: not(selected( ${q104_M75.2_full_list} , '6')) and not(selected( ${q104_M75.2_full_list} , '99')) and string-length( ${q104_M75.2_full_list_other} ) >0*<br>*Response constrained to: .>=0* | |
| q704_M75.2a.1 *(required)* | q704_M75.2a.1 What is the typical size of each item in the pack, box, etc. (e.g. 1 litre bottles, 250 mg tablets, 3 ml ampoules, etc.)?<br>*Please provide as much information as is available.*<br>*Question relevant when: not(selected( ${q104_M75.2_full_list} , '6')) and not(selected( ${q104_M75.2_full_list} , '99')) and string-length( ${q104_M75.2_full_list_other} ) >0* | |
| q704_M75.2b *(required)* | q704_M75.2b. What is the typical purchase price for that quantity of units (i.e. the quantity noted in the question above)?<br>*Question relevant when: not(selected( ${q104_M75.2_full_list} , '6')) and not(selected( ${q104_M75.2_full_list} , '99')) and string-length( ${q104_M75.2_full_list_other} ) >0*<br>*Response constrained to: .>=0* | |
| q704_M75.2c *(required)* | q704_M75.2c. Please specify the currency for the purchase price.<br>*Question relevant when: not(selected( ${q104_M75.2_full_list} , '6')) and not(selected( ${q104_M75.2_full_list} , '99')) and string-length( ${q104_M75.2_full_list_other} ) >0* | 1 Tanzanian Shillings<br>2 US dollars<br>3 Euros<br>4 Other<br>99 Don't know |
| q704_M75.2c.1 | q704_M75.2c.1 If other currency, specify:<br>*Leave blank if not applicable*<br>*Question relevant when: not(selected( ${q104_M75.2_full_list} , '6')) and not(selected( ${q104_M75.2_full_list} , '99')) and string-length( ${q104_M75.2_full_list_other} ) >0* | |
| q704_M75.2d *(required)* | q704_M75.2d. Please specify the year of the purchase price.<br>*Question relevant when: not(selected( ${q104_M75.2_full_list} , '6')) and not(selected( ${q104_M75.2_full_list} , '99')) and string-length( ${q104_M75.2_full_list_other} ) >0*<br>*Response constrained to: .>=2000 and .<=2019 or .=999* | |
| D. Medications - Cost (8)<br>*Group relevant when: selected( ${section_seven_skip_med} , '1')* | | |
| q704_med_note8 | 704 CONTINUED For each of the following medications and other medical products that are used for postabortion care, can you tell me …? | |
| note_704_hemostatic | ***Hemostatics*** | |
| q704_M76 | ***q704_M76. Dicynone / Dicynene (Etamsylate)***<br>*Question relevant when: not(selected( ${q104_M76_full_list} , '6')) and not(selected( ${q104_M76_full_list} , '99'))* | |

| Field | Question | Answer |
|---|---|---|
| q704_M76a *(required)* | q704_M76a. When purchased, how many units of this item typically come in one pack, box, bottle, etc.? (e.g. 100 tablets, 1000 ml's, etc.) (Write 1 if purchased as a single item.)<br>*Smallest units listed were: "[q204_M76c]" , "[q304_M76c]" , "[q404_M76c]" , "[q504_M76c]" , "[q604_M76c]"*<br>*Question relevant when: not(selected( ${q104_M76_full_list} , '6')) and not(selected( ${q104_M76_full_list} , '99'))*<br>*Response constrained to: .>=0* | |
| q704_M76a.1 *(required)* | q704_M76a.1 What is the typical size of each item in the pack, box, etc. (e.g. 1 litre bottles, 250 mg tablets, 3 ml ampoules, etc.)?<br>*Please provide as much information as is available.*<br>*Question relevant when: not(selected( ${q104_M76_full_list} , '6')) and not(selected( ${q104_M76_full_list} , '99'))* | |
| q704_M76b *(required)* | q704_M76b. What is the typical purchase price for that quantity of units (i.e. the quantity noted in the question above)?<br>*Question relevant when: not(selected( ${q104_M76_full_list} , '6')) and not(selected( ${q104_M76_full_list} , '99'))*<br>*Response constrained to: .>=0* | |

| | | |
|---|---|---|
| q704_M76c *(required)* | q704_M76c. Please specify the currency for the purchase price.<br>*Question relevant when: not(selected( ${q104_M76_full_list} , '6')) and not(selected( ${q104_M76_full_list} , '99'))* | 1 Tanzanian Shillings<br>2 US dollars<br>3 Euros<br>4 Other<br>99 Don't know |
| q704_M76c.1 | q704_M76c.1 If other currency, specify:<br>*Leave blank if not applicable*<br>*Question relevant when: not(selected( ${q104_M76_full_list} , '6')) and not(selected( ${q104_M76_full_list} , '99'))* | |
| q704_M76d *(required)* | q704_M76d. Please specify the year of the purchase price.<br>*Question relevant when: not(selected( ${q104_M76_full_list} , '6')) and not(selected( ${q104_M76_full_list} , '99'))*<br>*Response constrained to: .>=2000 and .<=2019 or .=999* | |
| q704_M77 | ***q704_M77. Tranexamic acid (Kapron, Cyklokapron)***<br>*Question relevant when: not(selected( ${q104_M77_full_list} , '6')) and not(selected( ${q104_M77_full_list} , '99'))* | |
| q704_M77a *(required)* | q704_M77a. When purchased, how many units of this item typically come in one pack, box, bottle, etc.? (e.g. 100 tablets, 1000 ml's, etc.) (Write 1 if purchased as a single item.)<br>*Smallest units listed were: "[q204_M77c]" , "[q304_M77c]" , "[q404_M77c]" , "[q504_M77c]" , "[q604_M77c]"*<br>*Question relevant when: not(selected( ${q104_M77_full_list} , '6')) and not(selected( ${q104_M77_full_list} , '99'))*<br>*Response constrained to: .>=0* | |
| q704_M77a.1 *(required)* | q704_M77a.1 What is the typical size of each item in the pack, box, etc. (e.g. 1 litre bottles, 250 mg tablets, 3 ml ampoules, etc.)?<br>*Please provide as much information as is available.*<br>*Question relevant when: not(selected( ${q104_M77_full_list} , '6')) and not(selected( ${q104_M77_full_list} , '99'))* | |
| q704_M77b *(required)* | q704_M77b. What is the typical purchase price for that quantity of units (i.e. the quantity noted in the question above)?<br>*Question relevant when: not(selected( ${q104_M77_full_list} , '6')) and not(selected( ${q104_M77_full_list} , '99'))*<br>*Response constrained to: .>=0* | |
| q704_M77c *(required)* | q704_M77c. Please specify the currency for the purchase price.<br>*Question relevant when: not(selected( ${q104_M77_full_list} , '6')) and not(selected( ${q104_M77_full_list} , '99'))* | 1 Tanzanian Shillings<br>2 US dollars<br>3 Euros<br>4 Other<br>99 Don't know |
| q704_M77c.1 | q704_M77c.1 If other currency, specify:<br>*Leave blank if not applicable*<br>*Question relevant when: not(selected( ${q104_M77_full_list} , '6')) and not(selected( ${q104_M77_full_list} , '99'))* | |
| q704_M77d *(required)* | q704_M77d. Please specify the year of the purchase price.<br>*Question relevant when: not(selected( ${q104_M77_full_list} , '6')) and not(selected( ${q104_M77_full_list} , '99'))* | |

| Field | Question | Answer |
| --- | --- | --- |
| | *Response constrained to: .>=2000 and .<=2019 or .=999* | |
| q704_M78 | ***q704_M78. Polygeline 4%*** | |
| | *Question relevant when: not(selected( ${q104_M78_full_list} , '6')) and not(selected( ${q104_M78_full_list} , '99'))* | |
| q704_M78a *(required)* | q704_M78a. When purchased, how many units of this item typically come in one pack, box, bottle, etc.? (e.g. 100 tablets, 1000 ml's, etc.) (Write 1 if purchased as a single item.)<br>*Smallest units listed were: "[q204_M78c]" , "[q304_M78c]" , "[q404_M78c]" , "[q504_M78c]" , "[q604_M78c]"*<br>*Question relevant when: not(selected( ${q104_M78_full_list} , '6')) and not(selected( ${q104_M78_full_list} , '99'))*<br>*Response constrained to: .>=0* | |
| q704_M78a.1 *(required)* | q704_M78a.1 What is the typical size of each item in the pack, box, etc. (e.g. 1 litre bottles, 250 mg tablets, 3 ml ampoules, etc.)?<br>*Please provide as much information as is available.*<br>*Question relevant when: not(selected( ${q104_M78_full_list} , '6')) and not(selected( ${q104_M78_full_list} , '99'))* | |
| q704_M78b *(required)* | q704_M78b. What is the typical purchase price for that quantity of units (i.e. the quantity noted in the question above)?<br>*Question relevant when: not(selected( ${q104_M78_full_list} , '6')) and not(selected( ${q104_M78_full_list} , '99'))*<br>*Response constrained to: .>=0* | |
| q704_M78c *(required)* | q704_M78c. Please specify the currency for the purchase price.<br>*Question relevant when: not(selected( ${q104_M78_full_list} , '6')) and not(selected( ${q104_M78_full_list} , '99'))* | 1 Tanzanian Shillings<br>2 US dollars<br>3 Euros<br>4 Other<br>99 Don't know |
| q704_M78c.1 | q704_M78c.1 If other currency, specify:<br>*Leave blank if not applicable*<br>*Question relevant when: not(selected( ${q104_M78_full_list} , '6')) and not(selected( ${q104_M78_full_list} , '99'))* | |
| q704_M78d *(required)* | q704_M78d. Please specify the year of the purchase price.<br>*Question relevant when: not(selected( ${q104_M78_full_list} , '6')) and not(selected( ${q104_M78_full_list} , '99'))*<br>*Response constrained to: .>=2000 and .<=2019 or .=999* | |
| q704_M79 | ***q704_M79. Hemostatic - other 1: "[q104_M79_full_list_other]"***<br>*Question relevant when: not(selected( ${q104_M79_full_list} , '6')) and not(selected( ${q104_M79_full_list} , '99')) and string-length( ${q104_M79_full_list_other} ) >0* | |
| q704_M79a *(required)* | q704_M79a. When purchased, how many units of this item typically come in one pack, box, bottle, etc.? (e.g. 100 tablets, 1000 ml's, etc.) (Write 1 if purchased as a single item.)<br>*Smallest units listed were: "[q204_M79c]" , "[q304_M79c]" , "[q404_M79c]" , "[q504_M79c]" , "[q604_M79c]"*<br>*Question relevant when: not(selected( ${q104_M79_full_list} , '6')) and not(selected( ${q104_M79_full_list} , '99')) and string-length( ${q104_M79_full_list_other} ) >0*<br>*Response constrained to: .>=0* | |
| q704_M79a.1 *(required)* | q704_M79a.1 What is the typical size of each item in the pack, box, etc. (e.g. 1 litre bottles, 250 mg tablets, 3 ml ampoules, etc.)?<br>*Please provide as much information as is available.*<br>*Question relevant when: not(selected( ${q104_M79_full_list} , '6')) and not(selected( ${q104_M79_full_list} , '99')) and string-length( ${q104_M79_full_list_other} ) >0* | |
| q704_M79b *(required)* | q704_M79b. What is the typical purchase price for that quantity of units (i.e. the quantity noted in the question above)?<br>*Question relevant when: not(selected( ${q104_M79_full_list} , '6')) and not(selected( ${q104_M79_full_list} , '99')) and string-length( ${q104_M79_full_list_other} ) >0*<br>*Response constrained to: .>=0* | |
| q704_M79c *(required)* | q704_M79c. Please specify the currency for the purchase price.<br>*Question relevant when: not(selected( ${q104_M79_full_list} , '6')) and not(selected( ${q104_M79_full_list} , '99')) and string-length( ${q104_M79_full_list_other} ) >0* | 1 Tanzanian Shillings<br>2 US dollars<br>3 Euros<br>4 Other<br>99 Don't know |
| q704_M79c.1 | q704_M79c.1 If other currency, specify:<br>*Leave blank if not applicable*<br>*Question relevant when: not(selected( ${q104_M79_full_list} , '6')) and not(selected( ${q104_M79_full_list} , '99')) and string-length( ${q104_M79_full_list_other} ) >0* | |
| q704_M79d *(required)* | q704_M79d. Please specify the year of the purchase price.<br>*Question relevant when: not(selected( ${q104_M79_full_list} , '6')) and not(selected( ${q104_M79_full_list} , '99')) and string-length( ${q104_M79_full_list_other} ) >0* | |

| Field | Question | Answer |
|---|---|---|
| | *Response constrained to: .>=2000 and .<=2019 or .=999* | |
| q704_M80 | ***q704_M80. Hemostatic - other 2: "[q104_M80_full_list_other]"*** | |
| | *Question relevant when: not(selected( ${q104_M80_full_list} , '6')) and not(selected( ${q104_M80_full_list} , '99')) and string-length( ${q104_M80_full_list_other} ) >0* | |
| q704_M80a *(required)* | q704_M80a. When purchased, how many units of this item typically come in one pack, box, bottle, etc.? (e.g. 100 tablets, 1000 ml's, etc.) (Write 1 if purchased as a single item.)<br>*Smallest units listed were: "[q204_M80c]" , "[q304_M80c]" , "[q404_M80c]" , "[q504_M80c]" , "[q604_M80c]"*<br>*Question relevant when: not(selected( ${q104_M80_full_list} , '6')) and not(selected( ${q104_M80_full_list} , '99')) and string-length( ${q104_M80_full_list_other} ) >0*<br>*Response constrained to: .>=0* | |
| q704_M80a.1 *(required)* | q704_M80a.1 What is the typical size of each item in the pack, box, etc. (e.g. 1 litre bottles, 250 mg tablets, 3 ml ampoules, etc.)?<br>*Please provide as much information as is available.*<br>*Question relevant when: not(selected( ${q104_M80_full_list} , '6')) and not(selected( ${q104_M80_full_list} , '99')) and string-length( ${q104_M80_full_list_other} ) >0* | |
| q704_M80b *(required)* | q704_M80b. What is the typical purchase price for that quantity of units (i.e. the quantity noted in the question above)?<br>*Question relevant when: not(selected( ${q104_M80_full_list} , '6')) and not(selected( ${q104_M80_full_list} , '99')) and string-length( ${q104_M80_full_list_other} ) >0*<br>*Response constrained to: .>=0* | |

| Field | Question | Answer |
|---|---|---|
| q704_M80c *(required)* | q704_M80c. Please specify the currency for the purchase price.<br>*Question relevant when: not(selected( ${q104_M80_full_list} , '6')) and not(selected( ${q104_M80_full_list} , '99')) and string-length( ${q104_M80_full_list_other} ) >0* | 1 Tanzanian Shillings<br>2 US dollars<br>3 Euros<br>4 Other<br>99 Don't know |
| q704_M80c.1 | q704_M80c.1 If other currency, specify:<br>*Leave blank if not applicable*<br>*Question relevant when: not(selected( ${q104_M80_full_list} , '6')) and not(selected( ${q104_M80_full_list} , '99')) and string-length( ${q104_M80_full_list_other} ) >0* | |
| q704_M80d *(required)* | q704_M80d. Please specify the year of the purchase price.<br>*Question relevant when: not(selected( ${q104_M80_full_list} , '6')) and not(selected( ${q104_M80_full_list} , '99')) and string-length( ${q104_M80_full_list_other} ) >0*<br>*Response constrained to: .>=2000 and .<=2019 or .=999* | |
| q704_M81 | ***q704_M81. Hemostatic - other 3: "[q104_M81_full_list_other]"***<br>*Question relevant when: not(selected( ${q104_M81_full_list} , '6')) and not(selected( ${q104_M81_full_list} , '99')) and string-length( ${q104_M81_full_list_other} ) >0* | |
| q704_M81a *(required)* | q704_M81a. When purchased, how many units of this item typically come in one pack, box, bottle, etc.? (e.g. 100 tablets, 1000 ml's, etc.) (Write 1 if purchased as a single item.)<br>*Smallest units listed were: "[q204_M81c]" , "[q304_M81c]" , "[q404_M81c]" , "[q504_M81c]" , "[q604_M81c]"*<br>*Question relevant when: not(selected( ${q104_M81_full_list} , '6')) and not(selected( ${q104_M81_full_list} , '99')) and string-length( ${q104_M81_full_list_other} ) >0*<br>*Response constrained to: .>=0* | |
| q704_M81a.1 *(required)* | q704_M81a.1 What is the typical size of each item in the pack, box, etc. (e.g. 1 litre bottles, 250 mg tablets, 3 ml ampoules, etc.)?<br>*Please provide as much information as is available.*<br>*Question relevant when: not(selected( ${q104_M81_full_list} , '6')) and not(selected( ${q104_M81_full_list} , '99')) and string-length( ${q104_M81_full_list_other} ) >0* | |
| q704_M81b *(required)* | q704_M81b. What is the typical purchase price for that quantity of units (i.e. the quantity noted in the question above)?<br>*Question relevant when: not(selected( ${q104_M81_full_list} , '6')) and not(selected( ${q104_M81_full_list} , '99')) and string-length( ${q104_M81_full_list_other} ) >0*<br>*Response constrained to: .>=0* | |
| q704_M81c *(required)* | q704_M81c. Please specify the currency for the purchase price.<br>*Question relevant when: not(selected( ${q104_M81_full_list} , '6')) and not(selected( ${q104_M81_full_list} , '99')) and string-length( ${q104_M81_full_list_other} ) >0* | 1 Tanzanian Shillings<br>2 US dollars<br>3 Euros<br>4 Other<br>99 Don't know |
| q704_M81c.1 | q704_M81c.1 If other currency, specify:<br>*Leave blank if not applicable*<br>*Question relevant when: not(selected( ${q104_M81_full_list} , '6')) and not(selected( ${q104_M81_full_list} , '99')) and string-length( ${q104_M81_full_list_other} ) >0* | |
| q704_M81d *(required)* | q704_M81d. Please specify the year of the purchase price.<br>*Question relevant when: not(selected( ${q104_M81_full_list} , '6')) and not(selected( ${q104_M81_full_list} , '99')) and string-length( ${q104_M81_full_list_other} ) >0* | |

| Field | Question | Answer |
|---|---|---|
| | *Response constrained to: .>=2000 and .<=2019 or .=999* | |
| q704_M81.1 | ***q704_M81.1. Hemostatic - other 4: "[q104_M81.1_full_list_other]"***<br>*Question relevant when: not(selected( ${q104_M81.1_full_list} , '6')) and not(selected( ${q104_M81.1_full_list} , '99')) and string-length( ${q104_M81.1_full_list_other} ) >0* | |
| q704_M81.1a *(required)* | q704_M81.1a. When purchased, how many units of this item typically come in one pack, box, bottle, etc.? (e.g. 100 tablets, 1000 ml's, etc.) (Write 1 if purchased as a single item.)<br>*Smallest units listed were: "[q204_M81.1c]" , "[q304_M81.1c]" , "[q404_M81.1c]" , "[q504_M81.1c]" , "[q604_M81.1c]"*<br>*Question relevant when: not(selected( ${q104_M81.1_full_list} , '6')) and not(selected( ${q104_M81.1_full_list} , '99')) and string-length( ${q104_M81.1_full_list_other} ) >0*<br>*Response constrained to: .>=0* | |
| q704_M81.1a.1 *(required)* | q704_M81.1a.1 What is the typical size of each item in the pack, box, etc. (e.g. 1 litre bottles, 250 mg tablets, 3 ml ampoules, etc.)?<br>*Please provide as much information as is available.*<br>*Question relevant when: not(selected( ${q104_M81.1_full_list} , '6')) and not(selected( ${q104_M81.1_full_list} , '99')) and string-length( ${q104_M81.1_full_list_other} ) >0* | |
| q704_M81.1b *(required)* | q704_M81.1b. What is the typical purchase price for that quantity of units (i.e. the quantity noted in the question above)?<br>*Question relevant when: not(selected( ${q104_M81.1_full_list} , '6')) and not(selected( ${q104_M81.1_full_list} , '99')) and string-length( ${q104_M81.1_full_list_other} ) >0*<br>*Response constrained to: .>=0* | |

| Field | Question | Answer |
|---|---|---|
| q704_M81.1c *(required)* | q704_M81.1c. Please specify the currency for the purchase price.<br>*Question relevant when: not(selected( ${q104_M81.1_full_list} , '6')) and not(selected( ${q104_M81.1_full_list} , '99')) and string-length( ${q104_M81.1_full_list_other} ) >0* | 1 Tanzanian Shillings<br>2 US dollars<br>3 Euros<br>4 Other<br>99 Don't know |
| q704_M81.1c.1 | q704_M81.1c.1 If other currency, specify:<br>*Leave blank if not applicable*<br>*Question relevant when: not(selected( ${q104_M81.1_full_list} , '6')) and not(selected( ${q104_M81.1_full_list} , '99')) and string-length( ${q104_M81.1_full_list_other} ) >0* | |
| q704_M81.1d *(required)* | q704_M81.1d. Please specify the year of the purchase price.<br>*Question relevant when: not(selected( ${q104_M81.1_full_list} , '6')) and not(selected( ${q104_M81.1_full_list} , '99')) and string-length( ${q104_M81.1_full_list_other} ) >0*<br>*Response constrained to: .>=2000 and .<=2019 or .=999* | |
| q704_M81.2 | ***q704_M81.2. Hemostatic - other 5: "[q104_M81.2_full_list_other]"***<br>*Question relevant when: not(selected( ${q104_M81.2_full_list} , '6')) and not(selected( ${q104_M81.2_full_list} , '99')) and string-length( ${q104_M81.2_full_list_other} ) >0* | |
| q704_M81.2a *(required)* | q704_M81.2a. When purchased, how many units of this item typically come in one pack, box, bottle, etc.? (e.g. 100 tablets, 1000 ml's, etc.) (Write 1 if purchased as a single item.)<br>*Smallest units listed were: "[q204_M81.2c]" , "[q304_M81.2c]" , "[q404_M81.2c]" , "[q504_M81.2c]" , "[q604_M81.2c]"*<br>*Question relevant when: not(selected( ${q104_M81.2_full_list} , '6')) and not(selected( ${q104_M81.2_full_list} , '99')) and string-length( ${q104_M81.2_full_list_other} ) >0*<br>*Response constrained to: .>=0* | |
| q704_M81.2a.1 *(required)* | q704_M81.2a.1 What is the typical size of each item in the pack, box, etc. (e.g. 1 litre bottles, 250 mg tablets, 3 ml ampoules, etc.)?<br>*Please provide as much information as is available.*<br>*Question relevant when: not(selected( ${q104_M81.2_full_list} , '6')) and not(selected( ${q104_M81.2_full_list} , '99')) and string-length( ${q104_M81.2_full_list_other} ) >0* | |
| q704_M81.2b *(required)* | q704_M81.2b. What is the typical purchase price for that quantity of units (i.e. the quantity noted in the question above)?<br>*Question relevant when: not(selected( ${q104_M81.2_full_list} , '6')) and not(selected( ${q104_M81.2_full_list} , '99')) and string-length( ${q104_M81.2_full_list_other} ) >0*<br>*Response constrained to: .>=0* | |
| q704_M81.2c *(required)* | q704_M81.2c. Please specify the currency for the purchase price.<br>*Question relevant when: not(selected( ${q104_M81.2_full_list} , '6')) and not(selected( ${q104_M81.2_full_list} , '99')) and string-length( ${q104_M81.2_full_list_other} ) >0* | 1 Tanzanian Shillings<br>2 US dollars<br>3 Euros<br>4 Other<br>99 Don't know |
| q704_M81.2c.1 | q704_M81.2c.1 If other currency, specify:<br>*Leave blank if not applicable*<br>*Question relevant when: not(selected( ${q104_M81.2_full_list} , '6')) and not(selected( ${q104_M81.2_full_list} , '99')) and string-length( ${q104_M81.2_full_list_other} ) >0* | |
| q704_M81.2d *(required)* | q704_M81.2d. Please specify the year of the purchase price.<br>*Question relevant when: not(selected( ${q104_M81.2_full_list} , '6')) and not(selected( ${q104_M81.2_full_list} , '99')) and string-length( ${q104_M81.2_full_list_other} ) >0* | |

| Field | Question | Answer |
|---|---|---|
| D. Medications - Cost (9) | *Response constrained to: .>=2000 and .<=2019 or .=999* | |
| *Group relevant when: selected( ${section_seven_skip_med} , '1')* | | |
| q704_med_notE8 | 704 CONTINUED For each of the following medications and other medical products that are used for postabortion care, can you tell me …? | |
| note_704_saline | ***Saline, glucose, water, etc*** | |
| q704_M82 | ***q704_M82.IV solution, dextrose 2.5% in NaCl 0.45%***<br>*Question relevant when: not(selected( ${q104_M82_full_list} , '6')) and not(selected( ${q104_M82_full_list} , '99'))* | |
| q704_M82a *(required)* | q704_M82a. When purchased, how many units of this item typically come in one pack, box, bottle, etc.? (e.g. 100 tablets, 1000 ml's, etc.) (Write 1 if purchased as a single item.)<br>*Smallest units listed were: "[q204_M82c]" , "[q304_M82c]" , "[q404_M82c]" , "[q504_M82c]" , "[q604_M82c]"*<br>*Question relevant when: not(selected( ${q104_M82_full_list} , '6')) and not(selected( ${q104_M82_full_list} , '99'))*<br>*Response constrained to: .>=0* | |
| q704_M82a.1 *(required)* | q704_M82a.1 What is the typical size of each item in the pack, box, etc. (e.g. 1 litre bottles, 250 mg tablets, 3 ml ampoules, etc.)?<br>*Please provide as much information as is available.*<br>*Question relevant when: not(selected( ${q104_M82_full_list} , '6')) and not(selected( ${q104_M82_full_list} , '99'))* | |
| q704_M82b *(required)* | q704_M82b. What is the typical purchase price for that quantity of units (i.e. the quantity noted in the question above)?<br>*Question relevant when: not(selected( ${q104_M82_full_list} , '6')) and not(selected( ${q104_M82_full_list} , '99'))*<br>*Response constrained to: .>=0* | |
| q704_M82c *(required)* | q704_M82c. Please specify the currency for the purchase price.<br>*Question relevant when: not(selected( ${q104_M82_full_list} , '6')) and not(selected( ${q104_M82_full_list} , '99'))* | 1 Tanzanian Shillings<br>2 US dollars<br>3 Euros<br>4 Other<br>99 Don't know |
| q704_M82c.1 | q704_M82c.1 If other currency, specify:<br>*Leave blank if not applicable*<br>*Question relevant when: not(selected( ${q104_M82_full_list} , '6')) and not(selected( ${q104_M82_full_list} , '99'))* | |
| q704_M82d *(required)* | q704_M82d. Please specify the year of the purchase price.<br>*Question relevant when: not(selected( ${q104_M82_full_list} , '6')) and not(selected( ${q104_M82_full_list} , '99'))*<br>*Response constrained to: .>=2000 and .<=2019 or .=999* | |
| q704_M83 | ***q704_M83.IV solution, glucose (5%), isotonic***<br>*Question relevant when: not(selected( ${q104_M83_full_list} , '6')) and not(selected( ${q104_M83_full_list} , '99'))* | |
| q704_M83a *(required)* | q704_M83a. When purchased, how many units of this item typically come in one pack, box, bottle, etc.? (e.g. 100 tablets, 1000 ml's, etc.) (Write 1 if purchased as a single item.)<br>*Smallest units listed were: "[q204_M83c]" , "[q304_M83c]" , "[q404_M83c]" , "[q504_M83c]" , "[q604_M83c]"*<br>*Question relevant when: not(selected( ${q104_M83_full_list} , '6')) and not(selected( ${q104_M83_full_list} , '99'))*<br>*Response constrained to: .>=0* | |
| q704_M83a.1 *(required)* | q704_M83a.1 What is the typical size of each item in the pack, box, etc. (e.g. 1 litre bottles, 250 mg tablets, 3 ml ampoules, etc.)?<br>*Please provide as much information as is available.*<br>*Question relevant when: not(selected( ${q104_M83_full_list} , '6')) and not(selected( ${q104_M83_full_list} , '99'))* | |
| q704_M83b *(required)* | q704_M83b. What is the typical purchase price for that quantity of units (i.e. the quantity noted in the question above)?<br>*Question relevant when: not(selected( ${q104_M83_full_list} , '6')) and not(selected( ${q104_M83_full_list} , '99'))*<br>*Response constrained to: .>=0* | |
| q704_M83c *(required)* | q704_M83c. Please specify the currency for the purchase price.<br>*Question relevant when: not(selected( ${q104_M83_full_list} , '6')) and not(selected( ${q104_M83_full_list} , '99'))* | 1 Tanzanian Shillings<br>2 US dollars<br>3 Euros<br>4 Other<br>99 Don't know |
| q704_M83c.1 | q704_M83c.1 If other currency, specify:<br>*Leave blank if not applicable* | |

| Field | Question | Answer |
|---|---|---|
| | *Question relevant when: not(selected( ${q104_M83_full_list} , '6')) and not(selected( ${q104_M83_full_list} , '99'))* | |
| q704_M83d *(required)* | q704_M83d. Please specify the year of the purchase price.<br>*Question relevant when: not(selected( ${q104_M83_full_list} , '6')) and not(selected( ${q104_M83_full_list} , '99'))*<br>*Response constrained to: .>=2000 and .<=2019 or .=999* | |
| q704_M84 | ***q704_M84. Ringers lactate (prepared onsite)***<br>*Question relevant when: not(selected( ${q104_M84_full_list} , '6')) and not(selected( ${q104_M84_full_list} , '99'))* | |
| q704_M84a *(required)* | q704_M84a. When purchased, how many units of this item typically come in one pack, box, bottle, etc.? (e.g. 100 tablets, 1000 ml's, etc.) (Write 1 if purchased as a single item.)<br>*Smallest units listed were: "[q204_M84c]" , "[q304_M84c]" , "[q404_M84c]" , "[q504_M84c]" , "[q604_M84c]"*<br>*Question relevant when: not(selected( ${q104_M84_full_list} , '6')) and not(selected( ${q104_M84_full_list} , '99'))*<br>*Response constrained to: .>=0* | |
| q704_M84a.1 *(required)* | q704_M84a.1 What is the typical size of each item in the pack, box, etc. (e.g. 1 litre bottles, 250 mg tablets, 3 ml ampoules, etc.)?<br>*Please provide as much information as is available.*<br>*Question relevant when: not(selected( ${q104_M84_full_list} , '6')) and not(selected( ${q104_M84_full_list} , '99'))* | |
| q704_M84b *(required)* | q704_M84b. What is the typical purchase price for that quantity of units (i.e. the quantity noted in the question above)?<br>*Question relevant when: not(selected( ${q104_M84_full_list} , '6')) and not(selected( ${q104_M84_full_list} , '99'))*<br>*Response constrained to: .>=0* | |
| q704_M84c *(required)* | q704_M84c. Please specify the currency for the purchase price.<br>*Question relevant when: not(selected( ${q104_M84_full_list} , '6')) and not(selected( ${q104_M84_full_list} , '99'))* | 1 Tanzanian Shillings<br>2 US dollars<br>3 Euros<br>4 Other<br>99 Don't know |
| q704_M84c.1 | q704_M84c.1 If other currency, specify:<br>*Leave blank if not applicable*<br>*Question relevant when: not(selected( ${q104_M84_full_list} , '6')) and not(selected( ${q104_M84_full_list} , '99'))* | |
| q704_M84d *(required)* | q704_M84d. Please specify the year of the purchase price.<br>*Question relevant when: not(selected( ${q104_M84_full_list} , '6')) and not(selected( ${q104_M84_full_list} , '99'))*<br>*Response constrained to: .>=2000 and .<=2019 or .=999* | |
| q704_M85 | ***q704_M85. Ringers lactate (bought from pharmacy/MSD)***<br>*Question relevant when: not(selected( ${q104_M85_full_list} , '6')) and not(selected( ${q104_M85_full_list} , '99'))* | |
| q704_M85a *(required)* | q704_M85a. When purchased, how many units of this item typically come in one pack, box, bottle, etc.? (e.g. 100 tablets, 1000 ml's, etc.) (Write 1 if purchased as a single item.)<br>*Smallest units listed were: "[q204_M85c]" , "[q304_M85c]" , "[q404_M85c]" , "[q504_M85c]" , "[q604_M85c]"*<br>*Question relevant when: not(selected( ${q104_M85_full_list} , '6')) and not(selected( ${q104_M85_full_list} , '99'))*<br>*Response constrained to: .>=0* | |
| q704_M85a.1 *(required)* | q704_M85a.1 What is the typical size of each item in the pack, box, etc. (e.g. 1 litre bottles, 250 mg tablets, 3 ml ampoules, etc.)?<br>*Please provide as much information as is available.*<br>*Question relevant when: not(selected( ${q104_M85_full_list} , '6')) and not(selected( ${q104_M85_full_list} , '99'))* | |
| q704_M85b *(required)* | q704_M85b. What is the typical purchase price for that quantity of units (i.e. the quantity noted in the question above)?<br>*Question relevant when: not(selected( ${q104_M85_full_list} , '6')) and not(selected( ${q104_M85_full_list} , '99'))*<br>*Response constrained to: .>=0* | |
| q704_M85c *(required)* | q704_M85c. Please specify the currency for the purchase price.<br>*Question relevant when: not(selected( ${q104_M85_full_list} , '6')) and not(selected( ${q104_M85_full_list} , '99'))* | 1 Tanzanian Shillings<br>2 US dollars<br>3 Euros<br>4 Other<br>99 Don't know |
| q704_M85c.1 | q704_M85c.1 If other currency, specify: | |

| Field | Question | Answer |
|---|---|---|
| | *Leave blank if not applicable*<br>*Question relevant when: not(selected( ${q104_M85_full_list} , '6')) and not(selected( ${q104_M85_full_list} , '99'))* | |
| q704_M85d *(required)* | q704_M85d. Please specify the year of the purchase price.<br>*Question relevant when: not(selected( ${q104_M85_full_list} , '6')) and not(selected( ${q104_M85_full_list} , '99'))*<br>*Response constrained to: .>=2000 and .<=2019 or .=999* | |
| q704_M86 | ***q704_M86. Saline, 0.9% (Normal Saline) (prepared onsite)***<br>*Question relevant when: not(selected( ${q104_M86_full_list} , '6')) and not(selected( ${q104_M86_full_list} , '99'))* | |
| q704_M86a *(required)* | q704_M86a. When purchased, how many units of this item typically come in one pack, box, bottle, etc.? (e.g. 100 tablets, 1000 ml's, etc.) (Write 1 if purchased as a single item.)<br>*Smallest units listed were: "[q204_M86c]" , "[q304_M86c]" , "[q404_M86c]" , "[q504_M86c]" , "[q604_M86c]"*<br>*Question relevant when: not(selected( ${q104_M86_full_list} , '6')) and not(selected( ${q104_M86_full_list} , '99'))*<br>*Response constrained to: .>=0* | |
| q704_M86a.1 *(required)* | q704_M86a.1 What is the typical size of each item in the pack, box, etc. (e.g. 1 litre bottles, 250 mg tablets, 3 ml ampoules, etc.)?<br>*Please provide as much information as is available.*<br>*Question relevant when: not(selected( ${q104_M86_full_list} , '6')) and not(selected( ${q104_M86_full_list} , '99'))* | |
| q704_M86b *(required)* | q704_M86b. What is the typical purchase price for that quantity of units (i.e. the quantity noted in the question above)?<br>*Question relevant when: not(selected( ${q104_M86_full_list} , '6')) and not(selected( ${q104_M86_full_list} , '99'))*<br>*Response constrained to: .>=0* | |
| q704_M86c *(required)* | q704_M86c. Please specify the currency for the purchase price.<br>*Question relevant when: not(selected( ${q104_M86_full_list} , '6')) and not(selected( ${q104_M86_full_list} , '99'))* | 1 Tanzanian Shillings<br>2 US dollars<br>3 Euros<br>4 Other<br>99 Don't know |
| q704_M86c.1 | q704_M86c.1 If other currency, specify:<br>*Leave blank if not applicable*<br>*Question relevant when: not(selected( ${q104_M86_full_list} , '6')) and not(selected( ${q104_M86_full_list} , '99'))* | |
| q704_M86d *(required)* | q704_M86d. Please specify the year of the purchase price.<br>*Question relevant when: not(selected( ${q104_M86_full_list} , '6')) and not(selected( ${q104_M86_full_list} , '99'))*<br>*Response constrained to: .>=2000 and .<=2019 or .=999* | |
| q704_M87 | ***q704_M87. Saline, 0.9% (Normal Saline) (bought from pharmacy/MSD)***<br>*Question relevant when: not(selected( ${q104_M87_full_list} , '6')) and not(selected( ${q104_M87_full_list} , '99'))* | |
| q704_M87a *(required)* | q704_M87a. When purchased, how many units of this item typically come in one pack, box, bottle, etc.? (e.g. 100 tablets, 1000 ml's, etc.) (Write 1 if purchased as a single item.)<br>*Smallest units listed were: "[q204_M87c]" , "[q304_M87c]" , "[q404_M87c]" , "[q504_M87c]" , "[q604_M87c]"*<br>*Question relevant when: not(selected( ${q104_M87_full_list} , '6')) and not(selected( ${q104_M87_full_list} , '99'))*<br>*Response constrained to: .>=0* | |
| q704_M87a.1 *(required)* | q704_M87a.1 What is the typical size of each item in the pack, box, etc. (e.g. 1 litre bottles, 250 mg tablets, 3 ml ampoules, etc.)?<br>*Please provide as much information as is available.*<br>*Question relevant when: not(selected( ${q104_M87_full_list} , '6')) and not(selected( ${q104_M87_full_list} , '99'))* | |
| q704_M87b *(required)* | q704_M87b. What is the typical purchase price for that quantity of units (i.e. the quantity noted in the question above)?<br>*Question relevant when: not(selected( ${q104_M87_full_list} , '6')) and not(selected( ${q104_M87_full_list} , '99'))*<br>*Response constrained to: .>=0* | |
| q704_M87c *(required)* | q704_M87c. Please specify the currency for the purchase price.<br>*Question relevant when: not(selected( ${q104_M87_full_list} , '6')) and not(selected( ${q104_M87_full_list} , '99'))* | 1 Tanzanian Shillings<br>2 US dollars<br>3 Euros<br>4 Other<br>99 Don't know |
| q704_M87c.1 | q704_M87c.1 If other currency, specify: | |

| Field | Question | Answer |
|---|---|---|
| | *Leave blank if not applicable*<br>*Question relevant when: not(selected( ${q104_M87_full_list} , '6')) and not(selected( ${q104_M87_full_list} , '99'))* | |
| q704_M87d *(required)* | q704_M87d. Please specify the year of the purchase price.<br>*Question relevant when: not(selected( ${q104_M87_full_list} , '6')) and not(selected( ${q104_M87_full_list} , '99'))*<br>*Response constrained to: .>=2000 and .<=2019 or .=999* | |
| q704_M88 | ***q704_M88. Solution for Infusion, glucose 5% + NaCl 0.9% (prepared onsite)***<br>*Question relevant when: not(selected( ${q104_M88_full_list} , '6')) and not(selected( ${q104_M88_full_list} , '99'))* | |
| q704_M88a *(required)* | q704_M88a. When purchased, how many units of this item typically come in one pack, box, bottle, etc.? (e.g. 100 tablets, 1000 ml's, etc.) (Write 1 if purchased as a single item.)<br>*Smallest units listed were: "[q204_M88c]" , "[q304_M88c]" , "[q404_M88c]" , "[q504_M88c]" , "[q604_M88c]"*<br>*Question relevant when: not(selected( ${q104_M88_full_list} , '6')) and not(selected( ${q104_M88_full_list} , '99'))*<br>*Response constrained to: .>=0* | |
| q704_M88a.1 *(required)* | q704_M88a.1 What is the typical size of each item in the pack, box, etc. (e.g. 1 litre bottles, 250 mg tablets, 3 ml ampoules, etc.)?<br>*Please provide as much information as is available.*<br>*Question relevant when: not(selected( ${q104_M88_full_list} , '6')) and not(selected( ${q104_M88_full_list} , '99'))* | |

| Field | Question | Answer |
|---|---|---|
| q704_M88b *(required)* | q704_M88b. What is the typical purchase price for that quantity of units (i.e. the quantity noted in the question above)?<br>*Question relevant when: not(selected( ${q104_M88_full_list} , '6')) and not(selected( ${q104_M88_full_list} , '99'))*<br>*Response constrained to: .>=0* | |
| q704_M88c *(required)* | q704_M88c. Please specify the currency for the purchase price.<br>*Question relevant when: not(selected( ${q104_M88_full_list} , '6')) and not(selected( ${q104_M88_full_list} , '99'))* | 1 Tanzanian Shillings<br>2 US dollars<br>3 Euros<br>4 Other<br>99 Don't know |
| q704_M88c.1 | q704_M88c.1 If other currency, specify:<br>*Leave blank if not applicable*<br>*Question relevant when: not(selected( ${q104_M88_full_list} , '6')) and not(selected( ${q104_M88_full_list} , '99'))* | |
| q704_M88d *(required)* | q704_M88d. Please specify the year of the purchase price.<br>*Question relevant when: not(selected( ${q104_M88_full_list} , '6')) and not(selected( ${q104_M88_full_list} , '99'))*<br>*Response constrained to: .>=2000 and .<=2019 or .=999* | |
| q704_M89 | ***q704_M89. Solution for Infusion, glucose 5% + NaCl 0.9% (bought from pharmacy or MSD)***<br>*Question relevant when: not(selected( ${q104_M89_full_list} , '6')) and not(selected( ${q104_M89_full_list} , '99'))* | |
| q704_M89a *(required)* | q704_M89a. When purchased, how many units of this item typically come in one pack, box, bottle, etc.? (e.g. 100 tablets, 1000 ml's, etc.) (Write 1 if purchased as a single item.)<br>*Smallest units listed were: "[q204_M89c]" , "[q304_M89c]" , "[q404_M89c]" , "[q504_M89c]" , "[q604_M89c]"*<br>*Question relevant when: not(selected( ${q104_M89_full_list} , '6')) and not(selected( ${q104_M89_full_list} , '99'))*<br>*Response constrained to: .>=0* | |
| q704_M89a.1 *(required)* | q704_M89a.1 What is the typical size of each item in the pack, box, etc. (e.g. 1 litre bottles, 250 mg tablets, 3 ml ampoules, etc.)?<br>*Please provide as much information as is available.*<br>*Question relevant when: not(selected( ${q104_M89_full_list} , '6')) and not(selected( ${q104_M89_full_list} , '99'))* | |
| q704_M89b *(required)* | q704_M89b. What is the typical purchase price for that quantity of units (i.e. the quantity noted in the question above)?<br>*Question relevant when: not(selected( ${q104_M89_full_list} , '6')) and not(selected( ${q104_M89_full_list} , '99'))*<br>*Response constrained to: .>=0* | |
| q704_M89c *(required)* | q704_M89c. Please specify the currency for the purchase price.<br>*Question relevant when: not(selected( ${q104_M89_full_list} , '6')) and not(selected( ${q104_M89_full_list} , '99'))* | 1 Tanzanian Shillings<br>2 US dollars<br>3 Euros<br>4 Other<br>99 Don't know |
| q704_M89c.1 | q704_M89c.1 If other currency, specify: | |

| Field | Question | Answer |
|---|---|---|
| | *Leave blank if not applicable*<br>*Question relevant when: not(selected( ${q104_M89_full_list} , '6')) and not(selected( ${q104_M89_full_list} , '99'))* | |
| q704_M89d *(required)* | q704_M89d. Please specify the year of the purchase price.<br>*Question relevant when: not(selected( ${q104_M89_full_list} , '6')) and not(selected( ${q104_M89_full_list} , '99'))*<br>*Response constrained to: .>=2000 and .<=2019 or .=999* | |
| q704_M90 | ***q704_M90. Water for injection***<br>*Question relevant when: not(selected( ${q104_M90_full_list} , '6')) and not(selected( ${q104_M90_full_list} , '99'))* | |
| q704_M90a *(required)* | q704_M90a. When purchased, how many units of this item typically come in one pack, box, bottle, etc.? (e.g. 100 tablets, 1000 ml's, etc.) (Write 1 if purchased as a single item.)<br>*Smallest units listed were: "[q204_M90c]" , "[q304_M90c]" , "[q404_M90c]" , "[q504_M90c]" , "[q604_M90c]"*<br>*Question relevant when: not(selected( ${q104_M90_full_list} , '6')) and not(selected( ${q104_M90_full_list} , '99'))*<br>*Response constrained to: .>=0* | |
| q704_M90a.1 *(required)* | q704_M90a.1 What is the typical size of each item in the pack, box, etc. (e.g. 1 litre bottles, 250 mg tablets, 3 ml ampoules, etc.)?<br>*Please provide as much information as is available.*<br>*Question relevant when: not(selected( ${q104_M90_full_list} , '6')) and not(selected( ${q104_M90_full_list} , '99'))* | |
| q704_M90b *(required)* | q704_M90b. What is the typical purchase price for that quantity of units (i.e. the quantity noted in the question above)?<br>*Question relevant when: not(selected( ${q104_M90_full_list} , '6')) and not(selected( ${q104_M90_full_list} , '99'))*<br>*Response constrained to: .>=0* | |
| q704_M90c *(required)* | q704_M90c. Please specify the currency for the purchase price.<br>*Question relevant when: not(selected( ${q104_M90_full_list} , '6')) and not(selected( ${q104_M90_full_list} , '99'))* | 1 Tanzanian Shillings<br>2 US dollars<br>3 Euros<br>4 Other<br>99 Don't know |
| q704_M90c.1 | q704_M90c.1 If other currency, specify:<br>*Leave blank if not applicable*<br>*Question relevant when: not(selected( ${q104_M90_full_list} , '6')) and not(selected( ${q104_M90_full_list} , '99'))* | |
| q704_M90d *(required)* | q704_M90d. Please specify the year of the purchase price.<br>*Question relevant when: not(selected( ${q104_M90_full_list} , '6')) and not(selected( ${q104_M90_full_list} , '99'))*<br>*Response constrained to: .>=2000 and .<=2019 or .=999* | |
| q704_M91 | ***q704_M91. Saline, glucose, water, etc. - Other 1: "[q104_M91_full_list_other]"***<br>*Question relevant when: not(selected( ${q104_M91_full_list} , '6')) and not(selected( ${q104_M91_full_list} , '99')) and string-length( ${q104_M91_full_list_other} ) >0* | |
| q704_M91a *(required)* | q704_M91a. When purchased, how many units of this item typically come in one pack, box, bottle, etc.? (e.g. 100 tablets, 1000 ml's, etc.) (Write 1 if purchased as a single item.)<br>*Smallest units listed were: "[q204_M91c]" , "[q304_M91c]" , "[q404_M91c]" , "[q504_M91c]" , "[q604_M91c]"*<br>*Question relevant when: not(selected( ${q104_M91_full_list} , '6')) and not(selected( ${q104_M91_full_list} , '99')) and string-length( ${q104_M91_full_list_other} ) >0*<br>*Response constrained to: .>=0* | |
| q704_M91a.1 *(required)* | q704_M91a.1 What is the typical size of each item in the pack, box, etc. (e.g. 1 litre bottles, 250 mg tablets, 3 ml ampoules, etc.)?<br>*Please provide as much information as is available.*<br>*Question relevant when: not(selected( ${q104_M91_full_list} , '6')) and not(selected( ${q104_M91_full_list} , '99')) and string-length( ${q104_M91_full_list_other} ) >0* | |
| q704_M91b *(required)* | q704_M91b. What is the typical purchase price for that quantity of units (i.e. the quantity noted in the question above)?<br>*Question relevant when: not(selected( ${q104_M91_full_list} , '6')) and not(selected( ${q104_M91_full_list} , '99')) and string-length( ${q104_M91_full_list_other} ) >0*<br>*Response constrained to: .>=0* | |
| q704_M91c *(required)* | q704_M91c. Please specify the currency for the purchase price.<br>*Question relevant when: not(selected( ${q104_M91_full_list} , '6')) and not(selected( ${q104_M91_full_list} , '99')) and string-length( ${q104_M91_full_list_other} ) >0* | 1 Tanzanian Shillings<br>2 US dollars<br>3 Euros<br>4 Other<br>99 Don't know |
| q704_M91c.1 | q704_M91c.1 If other currency, specify: | |

| Field | Question | Answer |
|---|---|---|
| | *Leave blank if not applicable*<br>*Question relevant when: not(selected( ${q104_M91_full_list} , '6')) and not(selected( ${q104_M91_full_list} , '99')) and string-length( ${q104_M91_full_list_other} ) >0* | |
| q704_M91d *(required)* | q704_M91d. Please specify the year of the purchase price.<br>*Question relevant when: not(selected( ${q104_M91_full_list} , '6')) and not(selected( ${q104_M91_full_list} , '99')) and string-length( ${q104_M91_full_list_other} ) >0*<br>*Response constrained to: .>=2000 and .<=2019 or .=999* | |
| q704_M92 | ***q704_M92. Saline, glucose, water, etc. - Other 2: "[q104_M92_full_list_other]"***<br>*Question relevant when: not(selected( ${q104_M92_full_list} , '6')) and not(selected( ${q104_M92_full_list} , '99')) and string-length( ${q104_M92_full_list_other} ) >0* | |
| q704_M92a *(required)* | q704_M92a. When purchased, how many units of this item typically come in one pack, box, bottle, etc.? (e.g. 100 tablets, 1000 ml's, etc.) (Write 1 if purchased as a single item.)<br>*Smallest units listed were: "[q204_M92c]" , "[q304_M92c]" , "[q404_M92c]" , "[q504_M92c]" , "[q604_M92c]"*<br>*Question relevant when: not(selected( ${q104_M92_full_list} , '6')) and not(selected( ${q104_M92_full_list} , '99')) and string-length( ${q104_M92_full_list_other} ) >0*<br>*Response constrained to: .>=0* | |
| q704_M92a.1 *(required)* | q704_M92a.1 What is the typical size of each item in the pack, box, etc. (e.g. 1 litre bottles, 250 mg tablets, 3 ml ampoules, etc.)?<br>*Please provide as much information as is available.*<br>*Question relevant when: not(selected( ${q104_M92_full_list} , '6')) and not(selected( ${q104_M92_full_list} , '99')) and string-length( ${q104_M92_full_list_other} ) >0* | |
| q704_M92b *(required)* | q704_M92b. What is the typical purchase price for that quantity of units (i.e. the quantity noted in the question above)?<br>*Question relevant when: not(selected( ${q104_M92_full_list} , '6')) and not(selected( ${q104_M92_full_list} , '99')) and string-length( ${q104_M92_full_list_other} ) >0*<br>*Response constrained to: .>=0* | |
| q704_M92c *(required)* | q704_M92c. Please specify the currency for the purchase price.<br>*Question relevant when: not(selected( ${q104_M92_full_list} , '6')) and not(selected( ${q104_M92_full_list} , '99')) and string-length( ${q104_M92_full_list_other} ) >0* | 1 Tanzanian Shillings<br>2 US dollars<br>3 Euros<br>4 Other<br>99 Don't know |
| q704_M92c.1 | q704_M92c.1 If other currency, specify:<br>*Leave blank if not applicable*<br>*Question relevant when: not(selected( ${q104_M92_full_list} , '6')) and not(selected( ${q104_M92_full_list} , '99')) and string-length( ${q104_M92_full_list_other} ) >0* | |
| q704_M92d *(required)* | q704_M92d. Please specify the year of the purchase price.<br>*Question relevant when: not(selected( ${q104_M92_full_list} , '6')) and not(selected( ${q104_M92_full_list} , '99')) and string-length( ${q104_M92_full_list_other} ) >0*<br>*Response constrained to: .>=2000 and .<=2019 or .=999* | |
| q704_M93 | ***q704_M93. Saline, glucose, water, etc. - Other 3: "[q104_M93_full_list_other]"***<br>*Question relevant when: not(selected( ${q104_M93_full_list} , '6')) and not(selected( ${q104_M93_full_list} , '99')) and string-length( ${q104_M93_full_list_other} ) >0* | |
| q704_M93a *(required)* | q704_M93a. When purchased, how many units of this item typically come in one pack, box, bottle, etc.? (e.g. 100 tablets, 1000 ml's, etc.) (Write 1 if purchased as a single item.)<br>*Smallest units listed were: "[q204_M93c]" , "[q304_M93c]" , "[q404_M93c]" , "[q504_M93c]" , "[q604_M93c]"*<br>*Question relevant when: not(selected( ${q104_M93_full_list} , '6')) and not(selected( ${q104_M93_full_list} , '99')) and string-length( ${q104_M93_full_list_other} ) >0*<br>*Response constrained to: .>=0* | |
| q704_M93a.1 *(required)* | q704_M93a.1 What is the typical size of each item in the pack, box, etc. (e.g. 1 litre bottles, 250 mg tablets, 3 ml ampoules, etc.)?<br>*Please provide as much information as is available.*<br>*Question relevant when: not(selected( ${q104_M93_full_list} , '6')) and not(selected( ${q104_M93_full_list} , '99')) and string-length( ${q104_M93_full_list_other} ) >0* | |
| q704_M93b *(required)* | q704_M93b. What is the typical purchase price for that quantity of units (i.e. the quantity noted in the question above)?<br>*Question relevant when: not(selected( ${q104_M93_full_list} , '6')) and not(selected( ${q104_M93_full_list} , '99')) and string-length( ${q104_M93_full_list_other} ) >0*<br>*Response constrained to: .>=0* | |
| q704_M93c *(required)* | q704_M93c. Please specify the currency for the purchase price.<br>*Question relevant when: not(selected( ${q104_M93_full_list} , '6')) and not(selected( ${q104_M93_full_list} , '99')) and string-length( ${q104_M93_full_list_other} ) >0* | 1 Tanzanian Shillings<br>2 US dollars<br>3 Euros<br>4 Other<br>99 Don't know |
| q704_M93c.1 | q704_M93c.1 If other currency, specify: | |

| Field | Question | Answer |
|---|---|---|
| | *Leave blank if not applicable*<br>*Question relevant when: not(selected( ${q104_M93_full_list} , '6')) and not(selected( ${q104_M93_full_list} , '99')) and string-length( ${q104_M93_full_list_other} ) >0* | |
| q704_M93d *(required)* | q704_M93d. Please specify the year of the purchase price.<br>*Question relevant when: not(selected( ${q104_M93_full_list} , '6')) and not(selected( ${q104_M93_full_list} , '99')) and string-length( ${q104_M93_full_list_other} ) >0*<br>*Response constrained to: .>=2000 and .<=2019 or .=999* | |
| q704_M94 | ***q704_M94. Saline, glucose, water, etc. - Other 4: "[q104_M94_full_list_other]"***<br>*Question relevant when: not(selected( ${q104_M94_full_list} , '6')) and not(selected( ${q104_M94_full_list} , '99')) and string-length( ${q104_M94_full_list_other} ) >0* | |
| q704_M94a *(required)* | q704_M94a. When purchased, how many units of this item typically come in one pack, box, bottle, etc.? (e.g. 100 tablets, 1000 ml's, etc.) (Write 1 if purchased as a single item.)<br>*Smallest units listed were: "[q204_M94c]" , "[q304_M94c]" , "[q404_M94c]" , "[q504_M94c]" , "[q604_M94c]"*<br>*Question relevant when: not(selected( ${q104_M94_full_list} , '6')) and not(selected( ${q104_M94_full_list} , '99')) and string-length( ${q104_M94_full_list_other} ) >0*<br>*Response constrained to: .>=0* | |
| q704_M94a.1 *(required)* | q704_M94a.1 What is the typical size of each item in the pack, box, etc. (e.g. 1 litre bottles, 250 mg tablets, 3 ml ampoules, etc.)?<br>*Please provide as much information as is available.*<br>*Question relevant when: not(selected( ${q104_M94_full_list} , '6')) and not(selected( ${q104_M94_full_list} , '99')) and string-length( ${q104_M94_full_list_other} ) >0* | |
| q704_M94b *(required)* | q704_M94b. What is the typical purchase price for that quantity of units (i.e. the quantity noted in the question above)?<br>*Question relevant when: not(selected( ${q104_M94_full_list} , '6')) and not(selected( ${q104_M94_full_list} , '99')) and string-length( ${q104_M94_full_list_other} ) >0*<br>*Response constrained to: .>=0* | |
| q704_M94c *(required)* | q704_M94c. Please specify the currency for the purchase price.<br>*Question relevant when: not(selected( ${q104_M94_full_list} , '6')) and not(selected( ${q104_M94_full_list} , '99')) and string-length( ${q104_M94_full_list_other} ) >0* | 1 Tanzanian Shillings<br>2 US dollars<br>3 Euros<br>4 Other<br>99 Don't know |
| q704_M94c.1 | q704_M94c.1 If other currency, specify:<br>*Leave blank if not applicable*<br>*Question relevant when: not(selected( ${q104_M94_full_list} , '6')) and not(selected( ${q104_M94_full_list} , '99')) and string-length( ${q104_M94_full_list_other} ) >0* | |
| q704_M94d *(required)* | q704_M94d. Please specify the year of the purchase price.<br>*Question relevant when: not(selected( ${q104_M94_full_list} , '6')) and not(selected( ${q104_M94_full_list} , '99')) and string-length( ${q104_M94_full_list_other} ) >0*<br>*Response constrained to: .>=2000 and .<=2019 or .=999* | |
| q704_M95 | ***q704_M95. Saline, glucose, water, etc. - Other 5: "[q104_M95_full_list_other]"***<br>*Question relevant when: not(selected( ${q104_M95_full_list} , '6')) and not(selected( ${q104_M95_full_list} , '99')) and string-length( ${q104_M95_full_list_other} ) >0* | |
| q704_M95a *(required)* | q704_M95a. When purchased, how many units of this item typically come in one pack, box, bottle, etc.? (e.g. 100 tablets, 1000 ml's, etc.) (Write 1 if purchased as a single item.)<br>*Smallest units listed were: "[q204_M95c]" , "[q304_M95c]" , "[q404_M95c]" , "[q504_M95c]" , "[q604_M95c]"*<br>*Question relevant when: not(selected( ${q104_M95_full_list} , '6')) and not(selected( ${q104_M95_full_list} , '99')) and string-length( ${q104_M95_full_list_other} ) >0*<br>*Response constrained to: .>=0* | |
| q704_M95a.1 *(required)* | q704_M95a.1 What is the typical size of each item in the pack, box, etc. (e.g. 1 litre bottles, 250 mg tablets, 3 ml ampoules, etc.)?<br>*Please provide as much information as is available.*<br>*Question relevant when: not(selected( ${q104_M95_full_list} , '6')) and not(selected( ${q104_M95_full_list} , '99')) and string-length( ${q104_M95_full_list_other} ) >0* | |
| q704_M95b *(required)* | q704_M95b. What is the typical purchase price for that quantity of units (i.e. the quantity noted in the question above)?<br>*Question relevant when: not(selected( ${q104_M95_full_list} , '6')) and not(selected( ${q104_M95_full_list} , '99')) and string-length( ${q104_M95_full_list_other} ) >0*<br>*Response constrained to: .>=0* | |
| q704_M95c *(required)* | q704_M95c. Please specify the currency for the purchase price.<br>*Question relevant when: not(selected( ${q104_M95_full_list} , '6')) and not(selected( ${q104_M95_full_list} , '99')) and string-length( ${q104_M95_full_list_other} ) >0* | 1 Tanzanian Shillings<br>2 US dollars<br>3 Euros<br>4 Other<br>99 Don't know |
| q704_M95c.1 | q704_M95c.1 If other currency, specify: | |

| Field | Question | Answer |
|---|---|---|
| | Leave blank if not applicable<br>*Question relevant when: not(selected( ${q104_M95_full_list} , '6')) and not(selected( ${q104_M95_full_list} , '99')) and string-length( ${q104_M95_full_list_other} ) >0* | |
| q704_M95d *(required)* | q704_M95d. Please specify the year of the purchase price.<br>*Question relevant when: not(selected( ${q104_M95_full_list} , '6')) and not(selected( ${q104_M95_full_list} , '99')) and string-length( ${q104_M95_full_list_other} ) >0*<br>*Response constrained to: .>=2000 and .<=2019 or .=999* | |
| D. Medications - Cost (10)<br>*Group relevant when: selected( ${section_seven_skip_med} , '1')* | | |
| q704_med_notE9 | 704 CONTINUED For each of the following medications and other medical products that are used for postabortion care, can you tell me …? | |
| note_704_prost_utero | ***Prostaglandin, uterotonic, etc.*** | |
| q704_M96 | ***q704_M96. Misoprostol***<br>*Question relevant when: not(selected( ${q104_M96_full_list} , '6')) and not(selected( ${q104_M96_full_list} , '99'))* | |
| q704_M96a *(required)* | q704_M96a. When purchased, how many units of this item typically come in one pack, box, bottle, etc.? (e.g. 100 tablets, 1000 ml's, etc.) (Write 1 if purchased as a single item.)<br>*Smallest units listed were: "[q204_M96c]" , "[q304_M96c]" , "[q404_M96c]" , "[q504_M96c]" , "[q604_M96c]"*<br>*Question relevant when: not(selected( ${q104_M96_full_list} , '6')) and not(selected( ${q104_M96_full_list} , '99'))*<br>*Response constrained to: .>=0* | |

| Field | Question | Answer |
|---|---|---|
| q704_M96a.1 *(required)* | q704_M96a.1 What is the typical size of each item in the pack, box, etc. (e.g. 1 litre bottles, 250 mg tablets, 3 ml ampoules, etc.)?<br>*Please provide as much information as is available.*<br>*Question relevant when: not(selected( ${q104_M96_full_list} , '6')) and not(selected( ${q104_M96_full_list} , '99'))* | |
| q704_M96b *(required)* | q704_M96b. What is the typical purchase price for that quantity of units (i.e. the quantity noted in the question above)?<br>*Question relevant when: not(selected( ${q104_M96_full_list} , '6')) and not(selected( ${q104_M96_full_list} , '99'))*<br>*Response constrained to: .>=0* | |
| q704_M96c *(required)* | q704_M96c. Please specify the currency for the purchase price.<br>*Question relevant when: not(selected( ${q104_M96_full_list} , '6')) and not(selected( ${q104_M96_full_list} , '99'))* | 1 Tanzanian Shillings<br>2 US dollars<br>3 Euros<br>4 Other<br>99 Don't know |
| q704_M96c.1 | q704_M96c.1 If other currency, specify:<br>*Leave blank if not applicable*<br>*Question relevant when: not(selected( ${q104_M96_full_list} , '6')) and not(selected( ${q104_M96_full_list} , '99'))* | |
| q704_M96d *(required)* | q704_M96d. Please specify the year of the purchase price.<br>*Question relevant when: not(selected( ${q104_M96_full_list} , '6')) and not(selected( ${q104_M96_full_list} , '99'))*<br>*Response constrained to: .>=2000 and .<=2019 or .=999* | |
| q704_M97 | ***q704_M97. Ergometrine***<br>*Question relevant when: not(selected( ${q104_M97_full_list} , '6')) and not(selected( ${q104_M97_full_list} , '99'))* | |
| q704_M97a *(required)* | q704_M97a. When purchased, how many units of this item typically come in one pack, box, bottle, etc.? (e.g. 100 tablets, 1000 ml's, etc.) (Write 1 if purchased as a single item.)<br>*Smallest units listed were: "[q204_M97c]" , "[q304_M97c]" , "[q404_M97c]" , "[q504_M97c]" , "[q604_M97c]"*<br>*Question relevant when: not(selected( ${q104_M97_full_list} , '6')) and not(selected( ${q104_M97_full_list} , '99'))*<br>*Response constrained to: .>=0* | |
| q704_M97a.1 *(required)* | q704_M97a.1 What is the typical size of each item in the pack, box, etc. (e.g. 1 litre bottles, 250 mg tablets, 3 ml ampoules, etc.)?<br>*Please provide as much information as is available.*<br>*Question relevant when: not(selected( ${q104_M97_full_list} , '6')) and not(selected( ${q104_M97_full_list} , '99'))* | |
| q704_M97b *(required)* | q704_M97b. What is the typical purchase price for that quantity of units (i.e. the quantity noted in the question above)?<br>*Question relevant when: not(selected( ${q104_M97_full_list} , '6')) and not(selected( ${q104_M97_full_list} , '99'))*<br>*Response constrained to: .>=0* | |
| q704_M97c *(required)* | q704_M97c. Please specify the currency for the purchase price.<br>[illegible] | 1 Tanzanian Shillings |

| Field | Question | Answer |
|---|---|---|
| | Question relevant when: not(selected( ${q104_M97_full_list} , '6')) and not(selected( ${q104_M97_full_list} , '99')) | 2 US dollars<br>3 Euros<br>4 Other<br>99 Don't know |
| q704_M97c.1 | q704_M97c.1 If other currency, specify:<br>*Leave blank if not applicable*<br>*Question relevant when: not(selected( ${q104_M97_full_list} , '6')) and not(selected( ${q104_M97_full_list} , '99'))* | |
| q704_M97d *(required)* | q704_M97d. Please specify the year of the purchase price.<br>*Question relevant when: not(selected( ${q104_M97_full_list} , '6')) and not(selected( ${q104_M97_full_list} , '99'))*<br>*Response constrained to: .>=2000 and .<=2019 or .=999* | |
| q704_M98 | ***q704_M98. Methylergonovine Maleate (Methergine)***<br>*Question relevant when: not(selected( ${q104_M98_full_list} , '6')) and not(selected( ${q104_M98_full_list} , '99'))* | |
| q704_M98a *(required)* | q704_M98a. When purchased, how many units of this item typically come in one pack, box, bottle, etc.? (e.g. 100 tablets, 1000 ml's, etc.) (Write 1 if purchased as a single item.)<br>*Smallest units listed were: "[q204_M98c]" , "[q304_M98c]" , "[q404_M98c]" , "[q504_M98c]" , "[q604_M98c]"*<br>*Question relevant when: not(selected( ${q104_M98_full_list} , '6')) and not(selected( ${q104_M98_full_list} , '99'))*<br>*Response constrained to: .>=0* | |

| | | |
|---|---|---|
| q704_M98a.1 *(required)* | q704_M98a.1 What is the typical size of each item in the pack, box, etc. (e.g. 1 litre bottles, 250 mg tablets, 3 ml ampoules, etc.)?<br>*Please provide as much information as is available.*<br>*Question relevant when: not(selected( ${q104_M98_full_list} , '6')) and not(selected( ${q104_M98_full_list} , '99'))* | |
| q704_M98b *(required)* | q704_M98b. What is the typical purchase price for that quantity of units (i.e. the quantity noted in the question above)?<br>*Question relevant when: not(selected( ${q104_M98_full_list} , '6')) and not(selected( ${q104_M98_full_list} , '99'))*<br>*Response constrained to: .>=0* | |
| q704_M98c *(required)* | q704_M98c. Please specify the currency for the purchase price.<br>*Question relevant when: not(selected( ${q104_M98_full_list} , '6')) and not(selected( ${q104_M98_full_list} , '99'))* | 1 Tanzanian Shillings<br>2 US dollars<br>3 Euros<br>4 Other<br>99 Don't know |
| q704_M98c.1 | q704_M98c.1 If other currency, specify:<br>*Leave blank if not applicable*<br>*Question relevant when: not(selected( ${q104_M98_full_list} , '6')) and not(selected( ${q104_M98_full_list} , '99'))* | |
| q704_M98d *(required)* | q704_M98d. Please specify the year of the purchase price.<br>*Question relevant when: not(selected( ${q104_M98_full_list} , '6')) and not(selected( ${q104_M98_full_list} , '99'))*<br>*Response constrained to: .>=2000 and .<=2019 or .=999* | |
| q704_M99 | ***q704_M99. Mifepristone***<br>*Question relevant when: not(selected( ${q104_M99_full_list} , '6')) and not(selected( ${q104_M99_full_list} , '99'))* | |
| q704_M99a *(required)* | q704_M99a. When purchased, how many units of this item typically come in one pack, box, bottle, etc.? (e.g. 100 tablets, 1000 ml's, etc.) (Write 1 if purchased as a single item.)<br>*Smallest units listed were: "[q204_M99c]" , "[q304_M99c]" , "[q404_M99c]" , "[q504_M99c]" , "[q604_M99c]"*<br>*Question relevant when: not(selected( ${q104_M99_full_list} , '6')) and not(selected( ${q104_M99_full_list} , '99'))*<br>*Response constrained to: .>=0* | |
| q704_M99a.1 *(required)* | q704_M99a.1 What is the typical size of each item in the pack, box, etc. (e.g. 1 litre bottles, 250 mg tablets, 3 ml ampoules, etc.)?<br>*Please provide as much information as is available.*<br>*Question relevant when: not(selected( ${q104_M99_full_list} , '6')) and not(selected( ${q104_M99_full_list} , '99'))* | |
| q704_M99b *(required)* | q704_M99b. What is the typical purchase price for that quantity of units (i.e. the quantity noted in the question above)?<br>*Question relevant when: not(selected( ${q104_M99_full_list} , '6')) and not(selected( ${q104_M99_full_list} , '99'))*<br>*Response constrained to: .>=0* | |
| q704_M99c *(required)* | q704_M99c. Please specify the currency for the purchase price.<br>[illegible] | 1 Tanzanian Shillings<br>[illegible] |

| Field | Question | Answer |
|---|---|---|
| | *Question relevant when: not(selected( ${q104_M99_full_list} , '6')) and not(selected( ${q104_M99_full_list} , '99'))* | 2 US dollars<br>3 Euros<br>4 Other<br>99 Don't know |
| q704_M99c.1 | q704_M99c.1 If other currency, specify:<br>*Leave blank if not applicable*<br>*Question relevant when: not(selected( ${q104_M99_full_list} , '6')) and not(selected( ${q104_M99_full_list} , '99'))* | |
| q704_M99d *(required)* | q704_M99d. Please specify the year of the purchase price.<br>*Question relevant when: not(selected( ${q104_M99_full_list} , '6')) and not(selected( ${q104_M99_full_list} , '99'))*<br>*Response constrained to: .>=2000 and .<=2019 or .=999* | |
| q704_M100 | ***q704_M100. Oxytocin***<br>*Question relevant when: not(selected( ${q104_M100_full_list} , '6')) and not(selected( ${q104_M100_full_list} , '99'))* | |
| q704_M100a *(required)* | q704_M100a. When purchased, how many units of this item typically come in one pack, box, bottle, etc.? (e.g. 100 tablets, 1000 ml's, etc.) (Write 1 if purchased as a single item.)<br>*Smallest units listed were: "[q204_M100c]" , "[q304_M100c]" , "[q404_M100c]" , "[q504_M100c]" , "[q604_M100c]"*<br>*Question relevant when: not(selected( ${q104_M100_full_list} , '6')) and not(selected( ${q104_M100_full_list} , '99'))*<br>*Response constrained to: .>=0* | |
| q704_M100a.1 *(required)* | q704_M100a.1 What is the typical size of each item in the pack, box, etc. (e.g. 1 litre bottles, 250 mg tablets, 3 ml ampoules, etc.)?<br>*Please provide as much information as is available.*<br>*Question relevant when: not(selected( ${q104_M100_full_list} , '6')) and not(selected( ${q104_M100_full_list} , '99'))* | |
| q704_M100b *(required)* | q704_M100b. What is the typical purchase price for that quantity of units (i.e. the quantity noted in the question above)?<br>*Question relevant when: not(selected( ${q104_M100_full_list} , '6')) and not(selected( ${q104_M100_full_list} , '99'))*<br>*Response constrained to: .>=0* | |
| q704_M100c *(required)* | q704_M100c. Please specify the currency for the purchase price.<br>*Question relevant when: not(selected( ${q104_M100_full_list} , '6')) and not(selected( ${q104_M100_full_list} , '99'))* | 1 Tanzanian Shillings<br>2 US dollars<br>3 Euros<br>4 Other<br>99 Don't know |
| q704_M100c.1 | q704_M100c.1 If other currency, specify:<br>*Leave blank if not applicable*<br>*Question relevant when: not(selected( ${q104_M100_full_list} , '6')) and not(selected( ${q104_M100_full_list} , '99'))* | |
| q704_M100d *(required)* | q704_M100d. Please specify the year of the purchase price.<br>*Question relevant when: not(selected( ${q104_M100_full_list} , '6')) and not(selected( ${q104_M100_full_list} , '99'))*<br>*Response constrained to: .>=2000 and .<=2019 or .=999* | |
| q704_M101 | ***q704_M101. Prostaglandin, uterotonic, etc. - Other 1: "[q104_M101_full_list_other]"***<br>*Question relevant when: not(selected( ${q104_M101_full_list} , '6')) and not(selected( ${q104_M101_full_list} , '99')) and string-length( ${q104_M101_full_list_other} ) >0* | |
| q704_M101a *(required)* | q704_M101a. When purchased, how many units of this item typically come in one pack, box, bottle, etc.? (e.g. 100 tablets, 1000 ml's, etc.) (Write 1 if purchased as a single item.)<br>*Smallest units listed were: "[q204_M101c]" , "[q304_M101c]" , "[q404_M101c]" , "[q504_M101c]" , "[q604_M101c]"*<br>*Question relevant when: not(selected( ${q104_M101_full_list} , '6')) and not(selected( ${q104_M101_full_list} , '99')) and string-length( ${q104_M101_full_list_other} ) >0*<br>*Response constrained to: .>=0* | |
| q704_M101a.1 *(required)* | q704_M101a.1 What is the typical size of each item in the pack, box, etc. (e.g. 1 litre bottles, 250 mg tablets, 3 ml ampoules, etc.)?<br>*Please provide as much information as is available.*<br>*Question relevant when: not(selected( ${q104_M101_full_list} , '6')) and not(selected( ${q104_M101_full_list} , '99')) and string-length( ${q104_M101_full_list_other} ) >0* | |
| q704_M101b *(required)* | q704_M101b. What is the typical purchase price for that quantity of units (i.e. the quantity noted in the question above)?<br>*Question relevant when: not(selected( ${q104_M101_full_list} , '6')) and not(selected( ${q104_M101_full_list} , '99')) and string-length( ${q104_M101_full_list_other} ) >0*<br>*Response constrained to: .>=0* | |
| q704_M101c *(required)* | q704_M101c. Please specify the currency for the purchase price.<br>[illegible] | 1 Tanzanian Shillings<br>[illegible] |

| Field | Question | Answer |
|---|---|---|
| | *Question relevant when: not(selected( ${q104_M101_full_list} , '6')) and not(selected( ${q104_M101_full_list} ,*<br>*'99')) and string-length( ${q104_M101_full_list_other} ) >0* | 2 US dollars<br>3 Euros<br>4 Other<br>99 Don't know |
| q704_M101c.1 | q704_M101c.1 If other currency, specify:<br>*Leave blank if not applicable*<br>*Question relevant when: not(selected( ${q104_M101_full_list} , '6')) and not(selected( ${q104_M101_full_list} , '99')) and string-length( ${q104_M101_full_list_other} ) >0* | |
| q704_M101d *(required)* | q704_M101d. Please specify the year of the purchase price.<br>*Question relevant when: not(selected( ${q104_M101_full_list} , '6')) and not(selected( ${q104_M101_full_list} , '99')) and string-length( ${q104_M101_full_list_other} ) >0*<br>*Response constrained to: .>=2000 and .<=2019 or .=999* | |
| q704_M102 | ***q704_M102. Prostaglandin, uterotonic, etc. - Other 2: "[q104_M102_full_list_other]"***<br>*Question relevant when: not(selected( ${q104_M102_full_list} , '6')) and not(selected( ${q104_M102_full_list} , '99')) and string-length( ${q104_M102_full_list_other} ) >0* | |
| q704_M102a *(required)* | q704_M102a. When purchased, how many units of this item typically come in one pack, box, bottle, etc.? (e.g. 100 tablets, 1000 ml's, etc.) (Write 1 if purchased as a single item.)<br>*Smallest units listed were: "[q204_M102c]" , "[q304_M102c]" , "[q404_M102c]" , "[q504_M102c]" , "[q604_M102c]"*<br>*Question relevant when: not(selected( ${q104_M102_full_list} , '6')) and not(selected( ${q104_M102_full_list} , '99')) and string-length( ${q104_M102_full_list_other} ) >0*<br>*Response constrained to: .>=0* | |

| | | |
|---|---|---|
| q704_M102a.1 *(required)* | q704_M102a.1 What is the typical size of each item in the pack, box, etc. (e.g. 1 litre bottles, 250 mg tablets, 3 ml ampoules, etc.)?<br>*Please provide as much information as is available.*<br>*Question relevant when: not(selected( ${q104_M102_full_list} , '6')) and not(selected( ${q104_M102_full_list} , '99')) and string-length( ${q104_M102_full_list_other} ) >0* | |
| q704_M102b *(required)* | q704_M102b. What is the typical purchase price for that quantity of units (i.e. the quantity noted in the question above)?<br>*Question relevant when: not(selected( ${q104_M102_full_list} , '6')) and not(selected( ${q104_M102_full_list} , '99')) and string-length( ${q104_M102_full_list_other} ) >0*<br>*Response constrained to: .>=0* | |
| q704_M102c *(required)* | q704_M102c. Please specify the currency for the purchase price.<br>*Question relevant when: not(selected( ${q104_M102_full_list} , '6')) and not(selected( ${q104_M102_full_list} , '99')) and string-length( ${q104_M102_full_list_other} ) >0* | 1 Tanzanian Shillings<br>2 US dollars<br>3 Euros<br>4 Other<br>99 Don't know |
| q704_M102c.1 | q704_M102c.1 If other currency, specify:<br>*Leave blank if not applicable*<br>*Question relevant when: not(selected( ${q104_M102_full_list} , '6')) and not(selected( ${q104_M102_full_list} , '99')) and string-length( ${q104_M102_full_list_other} ) >0* | |
| q704_M102d *(required)* | q704_M102d. Please specify the year of the purchase price.<br>*Question relevant when: not(selected( ${q104_M102_full_list} , '6')) and not(selected( ${q104_M102_full_list} , '99')) and string-length( ${q104_M102_full_list_other} ) >0*<br>*Response constrained to: .>=2000 and .<=2019 or .=999* | |
| q704_M103 | ***q704_M103. Prostaglandin, uterotonic, etc. - Other 3: "[q104_M103_full_list_other]"***<br>*Question relevant when: not(selected( ${q104_M104_full_list} , '6')) and not(selected( ${q104_M104_full_list} , '99')) and string-length( ${q104_M103_full_list_other} ) >0* | |
| q704_M103a *(required)* | q704_M103a. When purchased, how many units of this item typically come in one pack, box, bottle, etc.? (e.g. 100 tablets, 1000 ml's, etc.) (Write 1 if purchased as a single item.)<br>*Smallest units listed were: "[q204_M103c]" , "[q304_M103c]" , "[q404_M103c]" , "[q504_M103c]" , "[q604_M103c]"*<br>*Question relevant when: not(selected( ${q104_M104_full_list} , '6')) and not(selected( ${q104_M104_full_list} , '99')) and string-length( ${q104_M103_full_list_other} ) >0*<br>*Response constrained to: .>=0* | |
| q704_M103a.1 *(required)* | q704_M103a.1 What is the typical size of each item in the pack, box, etc. (e.g. 1 litre bottles, 250 mg tablets, 3 ml ampoules, etc.)?<br>*Please provide as much information as is available.*<br>*Question relevant when: not(selected( ${q104_M104_full_list} , '6')) and not(selected( ${q104_M104_full_list} , '99')) and string-length( ${q104_M103_full_list_other} ) >0* | |
| q704_M103b *(required)* | q704_M103b. What is the typical purchase price for that quantity of units (i.e. the quantity noted in the question above)?<br>*Question relevant when: not(selected( ${q104_M104_full_list} , '6')) and not(selected( ${q104_M104_full_list} , '99')) and string-length( ${q104_M103_full_list_other} ) >0*<br>*Response constrained to: .>=0* | |
| q704_M103c *(required)* | q704_M103c. Please specify the currency for the purchase price. | 1 Tanzanian Shillings |

| Field | Question | Answer |
|---|---|---|
| | *Question relevant when: not(selected( ${q104_M104_full_list} , '6')) and not(selected( ${q104_M104_full_list} , '99')) and string-length( ${q104_M103_full_list_other} ) >0* | 2 US dollars<br>3 Euros<br>4 Other<br>99 Don't know |
| q704_M103c.1 | q704_M103c.1 If other currency, specify:<br>*Leave blank if not applicable*<br>*Question relevant when: not(selected( ${q104_M104_full_list} , '6')) and not(selected( ${q104_M104_full_list} , '99')) and string-length( ${q104_M103_full_list_other} ) >0* | |
| q704_M103d *(required)* | q704_M103d. Please specify the year of the purchase price.<br>*Question relevant when: not(selected( ${q104_M104_full_list} , '6')) and not(selected( ${q104_M104_full_list} , '99')) and string-length( ${q104_M103_full_list_other} ) >0*<br>*Response constrained to: .>=2000 and .<=2019 or .=999* | |
| q704_M104 | ***q704_M104. Prostaglandin, uterotonic, etc. - Other 4: "[q104_M104_full_list_other]"***<br>*Question relevant when: not(selected( ${q104_M104_full_list} , '6')) and not(selected( ${q104_M104_full_list} , '99')) and string-length( ${q104_M104_full_list_other} ) >0* | |
| q704_M104a *(required)* | q704_M104a. When purchased, how many units of this item typically come in one pack, box, bottle, etc.? (e.g. 100 tablets, 1000 ml's, etc.) (Write 1 if purchased as a single item.)<br>*Smallest units listed were: "[q204_M104c]" , "[q304_M104c]" , "[q404_M104c]" , "[q504_M104c]" , "[q604_M104c]"*<br>*Question relevant when: not(selected( ${q104_M104_full_list} , '6')) and not(selected( ${q104_M104_full_list} , '99')) and string-length( ${q104_M104_full_list_other} ) >0*<br>*Response constrained to: .>=0* | |
| q704_M104a.1 *(required)* | q704_M104a.1 What is the typical size of each item in the pack, box, etc. (e.g. 1 litre bottles, 250 mg tablets, 3 ml ampoules, etc.)?<br>*Please provide as much information as is available.*<br>*Question relevant when: not(selected( ${q104_M104_full_list} , '6')) and not(selected( ${q104_M104_full_list} , '99')) and string-length( ${q104_M104_full_list_other} ) >0* | |
| q704_M104b *(required)* | q704_M104b. What is the typical purchase price for that quantity of units (i.e. the quantity noted in the question above)?<br>*Question relevant when: not(selected( ${q104_M104_full_list} , '6')) and not(selected( ${q104_M104_full_list} , '99')) and string-length( ${q104_M104_full_list_other} ) >0*<br>*Response constrained to: .>=0* | |
| q704_M104c *(required)* | q704_M104c. Please specify the currency for the purchase price.<br>*Question relevant when: not(selected( ${q104_M104_full_list} , '6')) and not(selected( ${q104_M104_full_list} , '99')) and string-length( ${q104_M104_full_list_other} ) >0* | 1 Tanzanian Shillings<br>2 US dollars<br>3 Euros<br>4 Other<br>99 Don't know |
| q704_M104c.1 | q704_M104c.1 If other currency, specify:<br>*Leave blank if not applicable*<br>*Question relevant when: not(selected( ${q104_M104_full_list} , '6')) and not(selected( ${q104_M104_full_list} , '99')) and string-length( ${q104_M104_full_list_other} ) >0* | |
| q704_M104d *(required)* | q704_M104d. Please specify the year of the purchase price.<br>*Question relevant when: not(selected( ${q104_M104_full_list} , '6')) and not(selected( ${q104_M104_full_list} , '99')) and string-length( ${q104_M104_full_list_other} ) >0*<br>*Response constrained to: .>=2000 and .<=2019 or .=999* | |
| q704_M105 | ***q704_M105. Prostaglandin, uterotonic, etc. - Other 5: "[q104_M105_full_list_other]"***<br>*Question relevant when: not(selected( ${q104_M105_full_list} , '6')) and not(selected( ${q104_M105_full_list} , '99')) and string-length( ${q104_M105_full_list_other} ) >0* | |
| q704_M105a *(required)* | q704_M105a. When purchased, how many units of this item typically come in one pack, box, bottle, etc.? (e.g. 100 tablets, 1000 ml's, etc.) (Write 1 if purchased as a single item.)<br>*Smallest units listed were: "[q204_M105c]" , "[q304_M105c]" , "[q404_M105c]" , "[q504_M105c]" , "[q604_M105c]"*<br>*Question relevant when: not(selected( ${q104_M105_full_list} , '6')) and not(selected( ${q104_M105_full_list} , '99')) and string-length( ${q104_M105_full_list_other} ) >0*<br>*Response constrained to: .>=0* | |
| q704_M105a.1 *(required)* | q704_M105a.1 What is the typical size of each item in the pack, box, etc. (e.g. 1 litre bottles, 250 mg tablets, 3 ml ampoules, etc.)?<br>*Please provide as much information as is available.*<br>*Question relevant when: not(selected( ${q104_M105_full_list} , '6')) and not(selected( ${q104_M105_full_list} , '99')) and string-length( ${q104_M105_full_list_other} ) >0* | |
| q704_M105b *(required)* | q704_M105b. What is the typical purchase price for that quantity of units (i.e. the quantity noted in the question above)?<br>*Question relevant when: not(selected( ${q104_M105_full_list} , '6')) and not(selected( ${q104_M105_full_list} , '99')) and string-length( ${q104_M105_full_list_other} ) >0*<br>*Response constrained to: .>=0* | |
| q704_M105c *(required)* | q704_M105c. Please specify the currency for the purchase price. | 1 Tanzanian Shillings |

| Field | Question | Answer |
|---|---|---|
| | *Question relevant when: not(selected( ${q104_M105_full_list} , '6')) and not(selected( ${q104_M105_full_list} , '99')) and string-length( ${q104_M105_full_list_other} ) >0* | 2 US dollars<br>3 Euros<br>4 Other<br>99 Don't know |
| q704_M105c.1 | q704_M105c.1 If other currency, specify:<br>*Leave blank if not applicable*<br>*Question relevant when: not(selected( ${q104_M105_full_list} , '6')) and not(selected( ${q104_M105_full_list} , '99')) and string-length( ${q104_M105_full_list_other} ) >0* | |
| q704_M105d *(required)* | q704_M105d. Please specify the year of the purchase price.<br>*Question relevant when: not(selected( ${q104_M105_full_list} , '6')) and not(selected( ${q104_M105_full_list} , '99')) and string-length( ${q104_M105_full_list_other} ) >0*<br>*Response constrained to: .>=2000 and .<=2019 or .=999* | |
| D. Medications - Cost (11)<br>*Group relevant when: selected( ${section_seven_skip_med} , '1')* | | |
| q704_med_notE10 | 704 CONTINUED For each of the following medications and other medical products that are used for postabortion care, can you tell me …? | |
| note_704_vitamin | ***Vitamin, immunological, etc.*** | |
| q704_M106 | ***q704_M106. Ferrous sulphate (tablets)***<br>*Question relevant when: not(selected( ${q104_M106_full_list} , '6')) and not(selected( ${q104_M106_full_list} , '99'))* | |

| Field | Question | Answer |
|---|---|---|
| q704_M106a *(required)* | q704_M106a. When purchased, how many units of this item typically come in one pack, box, bottle, etc.? (e.g. 100 tablets, 1000 ml's, etc.) (Write 1 if purchased as a single item.)<br>*Smallest units listed were: "[q204_M106c]" , "[q304_M106c]" , "[q404_M106c]" , "[q504_M106c]" , "[q604_M106c]"*<br>*Question relevant when: not(selected( ${q104_M106_full_list} , '6')) and not(selected( ${q104_M106_full_list} , '99'))*<br>*Response constrained to: .>=0* | |
| q704_M106a.1 *(required)* | q704_M106a.1 What is the typical size of each item in the pack, box, etc. (e.g. 1 litre bottles, 250 mg tablets, 3 ml ampoules, etc.)?<br>*Please provide as much information as is available.*<br>*Question relevant when: not(selected( ${q104_M106_full_list} , '6')) and not(selected( ${q104_M106_full_list} , '99'))* | |
| q704_M106b *(required)* | q704_M106b. What is the typical purchase price for that quantity of units (i.e. the quantity noted in the question above)?<br>*Question relevant when: not(selected( ${q104_M106_full_list} , '6')) and not(selected( ${q104_M106_full_list} , '99'))*<br>*Response constrained to: .>=0* | |
| q704_M106c *(required)* | q704_M106c. Please specify the currency for the purchase price.<br>*Question relevant when: not(selected( ${q104_M106_full_list} , '6')) and not(selected( ${q104_M106_full_list} , '99'))* | 1 Tanzanian Shillings<br>2 US dollars<br>3 Euros<br>4 Other<br>99 Don't know |
| q704_M106c.1 | q704_M106c.1 If other currency, specify:<br>*Leave blank if not applicable*<br>*Question relevant when: not(selected( ${q104_M106_full_list} , '6')) and not(selected( ${q104_M106_full_list} , '99'))* | |
| q704_M106d *(required)* | q704_M106d. Please specify the year of the purchase price.<br>*Question relevant when: not(selected( ${q104_M106_full_list} , '6')) and not(selected( ${q104_M106_full_list} , '99'))*<br>*Response constrained to: .>=2000 and .<=2019 or .=999* | |
| q704_M107 | ***q704_M107. Ferrous sulphate + folic acid (Ferrotone capsules)***<br>*Question relevant when: not(selected( ${q104_M107_full_list} , '6')) and not(selected( ${q104_M107_full_list} , '99'))* | |
| q704_M107a *(required)* | q704_M107a. When purchased, how many units of this item typically come in one pack, box, bottle, etc.? (e.g. 100 tablets, 1000 ml's, etc.) (Write 1 if purchased as a single item.)<br>*Smallest units listed were: "[q204_M107c]" , "[q304_M107c]" , "[q404_M107c]" , "[q504_M107c]" , "[q604_M107c]"*<br>*Question relevant when: not(selected( ${q104_M107_full_list} , '6')) and not(selected( ${q104_M107_full_list} , '99'))*<br>*Response constrained to: .>=0* | |
| q704_M107a.1 *(required)* | q704_M107a.1 What is the typical size of each item in the pack, box, etc. (e.g. 1 litre bottles, 250 mg tablets, 3 ml ampoules, etc.)?<br>*Please provide as much information as is available.*<br>*Question relevant when: not(selected( ${q104_M107_full_list} , '6')) and not(selected( ${q104_M107_full_list} , '99'))* | |
| q704_M107b *(required)* | q704_M107b. What is the typical purchase price for that quantity of units (i.e. the quantity noted in the question | |

| Field | Question | Answer |
|---|---|---|
| | above)?<br>*Question relevant when: not(selected( ${q104_M107_full_list} , '6')) and not(selected( ${q104_M107_full_list} , '99'))*<br>*Response constrained to: .>=0* | |
| q704_M107c *(required)* | q704_M107c. Please specify the currency for the purchase price.<br>*Question relevant when: not(selected( ${q104_M107_full_list} , '6')) and not(selected( ${q104_M107_full_list} , '99'))* | 1 Tanzanian Shillings<br>2 US dollars<br>3 Euros<br>4 Other<br>99 Don't know |
| q704_M107c.1 | q704_M107c.1 If other currency, specify:<br>*Leave blank if not applicable*<br>*Question relevant when: not(selected( ${q104_M107_full_list} , '6')) and not(selected( ${q104_M107_full_list} , '99'))* | |
| q704_M107d *(required)* | q704_M107d. Please specify the year of the purchase price.<br>*Question relevant when: not(selected( ${q104_M107_full_list} , '6')) and not(selected( ${q104_M107_full_list} , '99'))*<br>*Response constrained to: .>=2000 and .<=2019 or .=999* | |
| q704_M108 | ***q704_M108. Folic acid (Tablets)***<br>*Question relevant when: not(selected( ${q104_M108_full_list} , '6')) and not(selected( ${q104_M108_full_list} , '99'))* | |

| | | |
|---|---|---|
| q704_M108a *(required)* | q704_M108a. When purchased, how many units of this item typically come in one pack, box, bottle, etc.? (e.g. 100 tablets, 1000 ml's, etc.) (Write 1 if purchased as a single item.)<br>*Smallest units listed were: "[q204_M108c]" , "[q304_M108c]" , "[q404_M108c]" , "[q504_M108c]" , "[q604_M108c]"*<br>*Question relevant when: not(selected( ${q104_M108_full_list} , '6')) and not(selected( ${q104_M108_full_list} , '99'))*<br>*Response constrained to: .>=0* | |
| q704_M108a.1 *(required)* | q704_M108a.1 What is the typical size of each item in the pack, box, etc. (e.g. 1 litre bottles, 250 mg tablets, 3 ml ampoules, etc.)?<br>*Please provide as much information as is available.*<br>*Question relevant when: not(selected( ${q104_M108_full_list} , '6')) and not(selected( ${q104_M108_full_list} , '99'))* | |
| q704_M108b *(required)* | q704_M108b. What is the typical purchase price for that quantity of units (i.e. the quantity noted in the question above)?<br>*Question relevant when: not(selected( ${q104_M108_full_list} , '6')) and not(selected( ${q104_M108_full_list} , '99'))*<br>*Response constrained to: .>=0* | |
| q704_M108c *(required)* | q704_M108c. Please specify the currency for the purchase price.<br>*Question relevant when: not(selected( ${q104_M108_full_list} , '6')) and not(selected( ${q104_M108_full_list} , '99'))* | 1 Tanzanian Shillings<br>2 US dollars<br>3 Euros<br>4 Other<br>99 Don't know |
| q704_M108c.1 | q704_M108c.1 If other currency, specify:<br>*Leave blank if not applicable*<br>*Question relevant when: not(selected( ${q104_M108_full_list} , '6')) and not(selected( ${q104_M108_full_list} , '99'))* | |
| q704_M108d *(required)* | q704_M108d. Please specify the year of the purchase price.<br>*Question relevant when: not(selected( ${q104_M108_full_list} , '6')) and not(selected( ${q104_M108_full_list} , '99'))*<br>*Response constrained to: .>=2000 and .<=2019 or .=999* | |
| q704_M109 | ***q704_M109. Serum anti-tetanus***<br>*Question relevant when: not(selected( ${q104_M109_full_list} , '6')) and not(selected( ${q104_M109_full_list} , '99'))* | |
| q704_M109a *(required)* | q704_M109a. When purchased, how many units of this item typically come in one pack, box, bottle, etc.? (e.g. 100 tablets, 1000 ml's, etc.) (Write 1 if purchased as a single item.)<br>*Smallest units listed were: "[q204_M109c]" , "[q304_M109c]" , "[q404_M109c]" , "[q504_M109c]" , "[q604_M109c]"*<br>*Question relevant when: not(selected( ${q104_M109_full_list} , '6')) and not(selected( ${q104_M109_full_list} , '99'))*<br>*Response constrained to: .>=0* | |
| q704_M109a.1 *(required)* | q704_M109a.1 What is the typical size of each item in the pack, box, etc. (e.g. 1 litre bottles, 250 mg tablets, 3 ml ampoules, etc.)?<br>*Please provide as much information as is available.*<br>*Question relevant when: not(selected( ${q104_M109_full_list} , '6')) and not(selected( ${q104_M109_full_list} , '99'))* | |
| q704_M109b *(required)* | q704_M109b. What is the typical purchase price for that quantity of units (i.e. the quantity noted in the question | |

| Field | Question | Answer |
|---|---|---|
| | above)?<br>*Question relevant when: not(selected( ${q104_M109_full_list} , '6')) and not(selected( ${q104_M109_full_list} , '99'))*<br>*Response constrained to: .>=0* | |
| q704_M109c *(required)* | q704_M109c. Please specify the currency for the purchase price.<br>*Question relevant when: not(selected( ${q104_M109_full_list} , '6')) and not(selected( ${q104_M109_full_list} , '99'))* | 1 Tanzanian Shillings<br>2 US dollars<br>3 Euros<br>4 Other<br>99 Don't know |
| q704_M109c.1 | q704_M109c.1 If other currency, specify:<br>*Leave blank if not applicable*<br>*Question relevant when: not(selected( ${q104_M109_full_list} , '6')) and not(selected( ${q104_M109_full_list} , '99'))* | |
| q704_M109d *(required)* | q704_M109d. Please specify the year of the purchase price.<br>*Question relevant when: not(selected( ${q104_M109_full_list} , '6')) and not(selected( ${q104_M109_full_list} , '99'))*<br>*Response constrained to: .>=2000 and .<=2019 or .=999* | |
| q704_M110 | ***q704_M110. Anti D (Rh)-Immunoglobulin***<br>*Question relevant when: not(selected( ${q104_M110_full_list} , '6')) and not(selected( ${q104_M110_full_list} , '99'))* | |

| | | |
|---|---|---|
| q704_M110a *(required)* | q704_M110a. When purchased, how many units of this item typically come in one pack, box, bottle, etc.? (e.g. 100 tablets, 1000 ml's, etc.) (Write 1 if purchased as a single item.)<br>*Smallest units listed were: "[q204_M110c]" , "[q304_M110c]" , "[q404_M110c]" , "[q504_M110c]" , "[q604_M110c]"*<br>*Question relevant when: not(selected( ${q104_M110_full_list} , '6')) and not(selected( ${q104_M110_full_list} , '99'))*<br>*Response constrained to: .>=0* | |
| q704_M110a.1 *(required)* | q704_M110a.1 What is the typical size of each item in the pack, box, etc. (e.g. 1 litre bottles, 250 mg tablets, 3 ml ampoules, etc.)?<br>*Please provide as much information as is available.*<br>*Question relevant when: not(selected( ${q104_M110_full_list} , '6')) and not(selected( ${q104_M110_full_list} , '99'))* | |
| q704_M110b *(required)* | q704_M110b. What is the typical purchase price for that quantity of units (i.e. the quantity noted in the question above)?<br>*Question relevant when: not(selected( ${q104_M110_full_list} , '6')) and not(selected( ${q104_M110_full_list} , '99'))*<br>*Response constrained to: .>=0* | |
| q704_M110c *(required)* | q704_M110c. Please specify the currency for the purchase price.<br>*Question relevant when: not(selected( ${q104_M110_full_list} , '6')) and not(selected( ${q104_M110_full_list} , '99'))* | 1 Tanzanian Shillings<br>2 US dollars<br>3 Euros<br>4 Other<br>99 Don't know |
| q704_M110c.1 | q704_M110c.1 If other currency, specify:<br>*Leave blank if not applicable*<br>*Question relevant when: not(selected( ${q104_M110_full_list} , '6')) and not(selected( ${q104_M110_full_list} , '99'))* | |
| q704_M110d *(required)* | q704_M110d. Please specify the year of the purchase price.<br>*Question relevant when: not(selected( ${q104_M110_full_list} , '6')) and not(selected( ${q104_M110_full_list} , '99'))*<br>*Response constrained to: .>=2000 and .<=2019 or .=999* | |
| q704_M111 | ***q704_M111. Vitamin, immunological, etc. - Other 1: "[q104_M111_full_list_other]"***<br>*Question relevant when: not(selected( ${q104_M111_full_list} , '6')) and not(selected( ${q104_M111_full_list} , '99')) and string-length( ${q104_M111_full_list_other} ) >0* | |
| q704_M111a *(required)* | q704_M111a. When purchased, how many units of this item typically come in one pack, box, bottle, etc.? (e.g. 100 tablets, 1000 ml's, etc.) (Write 1 if purchased as a single item.)<br>*Smallest units listed were: "[q204_M111c]" , "[q304_M111c]" , "[q404_M111c]" , "[q504_M111c]" , "[q604_M111c]"*<br>*Question relevant when: not(selected( ${q104_M111_full_list} , '6')) and not(selected( ${q104_M111_full_list} , '99')) and string-length( ${q104_M111_full_list_other} ) >0*<br>*Response constrained to: .>=0* | |
| q704_M111a.1 *(required)* | q704_M111a.1 What is the typical size of each item in the pack, box, etc. (e.g. 1 litre bottles, 250 mg tablets, 3 ml ampoules, etc.)?<br>*Please provide as much information as is available.*<br>*Question relevant when: not(selected( ${q104_M111_full_list} , '6')) and not(selected( ${q104_M111_full_list} , '99')) and string-length( ${q104_M111_full_list_other} ) >0* | |
| q704_M111b *(required)* | q704_M111b. What is the typical purchase price for that quantity of units (i.e. the quantity noted in the question | |

| Field | Question | Answer |
|---|---|---|
| | above)?<br>*Question relevant when: not(selected( ${q104_M111_full_list} , '6')) and not(selected( ${q104_M111_full_list} , '99')) and string-length( ${q104_M111_full_list_other} ) >0*<br>*Response constrained to: .>=0* | |
| q704_M111c *(required)* | q704_M111c. Please specify the currency for the purchase price.<br>*Question relevant when: not(selected( ${q104_M111_full_list} , '6')) and not(selected( ${q104_M111_full_list} , '99')) and string-length( ${q104_M111_full_list_other} ) >0* | 1 Tanzanian Shillings<br>2 US dollars<br>3 Euros<br>4 Other<br>99 Don't know |
| q704_M111c.1 | q704_M111c.1 If other currency, specify:<br>*Leave blank if not applicable*<br>*Question relevant when: not(selected( ${q104_M111_full_list} , '6')) and not(selected( ${q104_M111_full_list} , '99')) and string-length( ${q104_M111_full_list_other} ) >0* | |
| q704_M111d *(required)* | q704_M111d. Please specify the year of the purchase price.<br>*Question relevant when: not(selected( ${q104_M111_full_list} , '6')) and not(selected( ${q104_M111_full_list} , '99')) and string-length( ${q104_M111_full_list_other} ) >0*<br>*Response constrained to: .>=2000 and .<=2019 or .=999* | |
| q704_M112 | ***q704_M112. Vitamin, immunological, etc. - Other 2: "[q104_M112_full_list_other]"***<br>*Question relevant when: not(selected( ${q104_M112_full_list} , '6')) and not(selected( ${q104_M112_full_list} , '99')) and string-length( ${q104_M112_full_list_other} ) >0* | |
| q704_M112a *(required)* | q704_M112a. When purchased, how many units of this item typically come in one pack, box, bottle, etc.? (e.g. 100 tablets, 1000 ml's, etc.) (Write 1 if purchased as a single item.)<br>*Smallest units listed were: "[q204_M112c]" , "[q304_M112c]" , "[q404_M112c]" , "[q504_M112c]" , "[q604_M112c]"*<br>*Question relevant when: not(selected( ${q104_M112_full_list} , '6')) and not(selected( ${q104_M112_full_list} , '99')) and string-length( ${q104_M112_full_list_other} ) >0*<br>*Response constrained to: .>=0* | |
| q704_M112a.1 *(required)* | q704_M112a.1 What is the typical size of each item in the pack, box, etc. (e.g. 1 litre bottles, 250 mg tablets, 3 ml ampoules, etc.)?<br>*Please provide as much information as is available.*<br>*Question relevant when: not(selected( ${q104_M112_full_list} , '6')) and not(selected( ${q104_M112_full_list} , '99')) and string-length( ${q104_M112_full_list_other} ) >0* | |
| q704_M112b *(required)* | q704_M112b. What is the typical purchase price for that quantity of units (i.e. the quantity noted in the question above)?<br>*Question relevant when: not(selected( ${q104_M112_full_list} , '6')) and not(selected( ${q104_M112_full_list} , '99')) and string-length( ${q104_M112_full_list_other} ) >0*<br>*Response constrained to: .>=0* | |
| q704_M112c *(required)* | q704_M112c. Please specify the currency for the purchase price.<br>*Question relevant when: not(selected( ${q104_M112_full_list} , '6')) and not(selected( ${q104_M112_full_list} , '99')) and string-length( ${q104_M112_full_list_other} ) >0* | 1 Tanzanian Shillings<br>2 US dollars<br>3 Euros<br>4 Other<br>99 Don't know |
| q704_M112c.1 | q704_M112c.1 If other currency, specify:<br>*Leave blank if not applicable*<br>*Question relevant when: not(selected( ${q104_M112_full_list} , '6')) and not(selected( ${q104_M112_full_list} , '99')) and string-length( ${q104_M112_full_list_other} ) >0* | |
| q704_M112d *(required)* | q704_M112d. Please specify the year of the purchase price.<br>*Question relevant when: not(selected( ${q104_M112_full_list} , '6')) and not(selected( ${q104_M112_full_list} , '99')) and string-length( ${q104_M112_full_list_other} ) >0*<br>*Response constrained to: .>=2000 and .<=2019 or .=999* | |
| q704_M113 | ***q704_M113. Vitamin, immunological, etc. - Other 3: "[q104_M113_full_list_other]"***<br>*Question relevant when: not(selected( ${q104_M113_full_list} , '6')) and not(selected( ${q104_M113_full_list} , '99')) and string-length( ${q104_M113_full_list_other} ) >0* | |
| q704_M113a *(required)* | q704_M113a. When purchased, how many units of this item typically come in one pack, box, bottle, etc.? (e.g. 100 tablets, 1000 ml's, etc.) (Write 1 if purchased as a single item.)<br>*Smallest units listed were: "[q204_M113c]" , "[q304_M113c]" , "[q404_M113c]" , "[q504_M113c]" , "[q604_M113c]"*<br>*Question relevant when: not(selected( ${q104_M113_full_list} , '6')) and not(selected( ${q104_M113_full_list} , '99')) and string-length( ${q104_M113_full_list_other} ) >0*<br>*Response constrained to: .>=0* | |
| q704_M113a.1 *(required)* | q704_M113a.1 What is the typical size of each item in the pack, box, etc. (e.g. 1 litre bottles, 250 mg tablets, 3 ml ampoules, etc.)?<br>*Please provide as much information as is available.*<br>*Question relevant when: not(selected( ${q104_M113_full_list} , '6')) and not(selected( ${q104_M113_full_list} , '99')) and string-length( ${q104_M113_full_list_other} ) >0* | |
| q704_M113b *(required)* | q704_M113b. What is the typical purchase price for that quantity of units (i.e. the quantity noted in the question | |

| Field | Question | Answer |
|---|---|---|
| | above)?<br>*Question relevant when: not(selected( ${q104_M113_full_list} , '6')) and not(selected( ${q104_M113_full_list} , '99')) and string-length( ${q104_M113_full_list_other} ) >0*<br>*Response constrained to: .>=0* | |
| q704_M113c *(required)* | q704_M113c. Please specify the currency for the purchase price.<br>*Question relevant when: not(selected( ${q104_M113_full_list} , '6')) and not(selected( ${q104_M113_full_list} , '99')) and string-length( ${q104_M113_full_list_other} ) >0* | 1 Tanzanian Shillings<br>2 US dollars<br>3 Euros<br>4 Other<br>99 Don't know |
| q704_M113c.1 | q704_M113c.1 If other currency, specify:<br>*Leave blank if not applicable*<br>*Question relevant when: not(selected( ${q104_M113_full_list} , '6')) and not(selected( ${q104_M113_full_list} , '99')) and string-length( ${q104_M113_full_list_other} ) >0* | |
| q704_M113d *(required)* | q704_M113d. Please specify the year of the purchase price.<br>*Question relevant when: not(selected( ${q104_M113_full_list} , '6')) and not(selected( ${q104_M113_full_list} , '99')) and string-length( ${q104_M113_full_list_other} ) >0*<br>*Response constrained to: .>=2000 and .<=2019 or .=999* | |
| q704_M114 | ***q704_M114. Vitamin, immunological, etc. - Other 4: "[q104_M114_full_list_other]"***<br>*Question relevant when: not(selected( ${q104_M114_full_list} , '6')) and not(selected( ${q104_M114_full_list} , '99')) and string-length( ${q104_M114_full_list_other} ) >0* | |

| | | |
|---|---|---|
| q704_M114a *(required)* | q704_M114a. When purchased, how many units of this item typically come in one pack, box, bottle, etc.? (e.g. 100 tablets, 1000 ml's, etc.) (Write 1 if purchased as a single item.)<br>*Smallest units listed were: "[q204_M114c]" , "[q304_M114c]" , "[q404_M114c]" , "[q504_M114c]" , "[q604_M114c]"*<br>*Question relevant when: not(selected( ${q104_M114_full_list} , '6')) and not(selected( ${q104_M114_full_list} , '99')) and string-length( ${q104_M114_full_list_other} ) >0*<br>*Response constrained to: .>=0* | |
| q704_M114a.1 *(required)* | q704_M114a.1 What is the typical size of each item in the pack, box, etc. (e.g. 1 litre bottles, 250 mg tablets, 3 ml ampoules, etc.)?<br>*Please provide as much information as is available.*<br>*Question relevant when: not(selected( ${q104_M114_full_list} , '6')) and not(selected( ${q104_M114_full_list} , '99')) and string-length( ${q104_M114_full_list_other} ) >0* | |
| q704_M114b *(required)* | q704_M114b. What is the typical purchase price for that quantity of units (i.e. the quantity noted in the question above)?<br>*Question relevant when: not(selected( ${q104_M114_full_list} , '6')) and not(selected( ${q104_M114_full_list} , '99')) and string-length( ${q104_M114_full_list_other} ) >0*<br>*Response constrained to: .>=0* | |
| q704_M114c *(required)* | q704_M114c. Please specify the currency for the purchase price.<br>*Question relevant when: not(selected( ${q104_M114_full_list} , '6')) and not(selected( ${q104_M114_full_list} , '99')) and string-length( ${q104_M114_full_list_other} ) >0* | 1 Tanzanian Shillings<br>2 US dollars<br>3 Euros<br>4 Other<br>99 Don't know |
| q704_M114c.1 | q704_M114c.1 If other currency, specify:<br>*Leave blank if not applicable*<br>*Question relevant when: not(selected( ${q104_M114_full_list} , '6')) and not(selected( ${q104_M114_full_list} , '99')) and string-length( ${q104_M114_full_list_other} ) >0* | |
| q704_M114d *(required)* | q704_M114d. Please specify the year of the purchase price.<br>*Question relevant when: not(selected( ${q104_M114_full_list} , '6')) and not(selected( ${q104_M114_full_list} , '99')) and string-length( ${q104_M114_full_list_other} ) >0*<br>*Response constrained to: .>=2000 and .<=2019 or .=999* | |
| q704_M115 | ***q704_M115. Vitamin, immunological, etc. - Other 5: "[q104_M115_full_list_other]"***<br>*Question relevant when: not(selected( ${q104_M115_full_list} , '6')) and not(selected( ${q104_M115_full_list} , '99')) and string-length( ${q104_M115_full_list_other} ) >0* | |
| q704_M115a *(required)* | q704_M115a. When purchased, how many units of this item typically come in one pack, box, bottle, etc.? (e.g. 100 tablets, 1000 ml's, etc.) (Write 1 if purchased as a single item.)<br>*Smallest units listed were: "[q204_M115c]" , "[q304_M115c]" , "[q404_M115c]" , "[q504_M115c]" , "[q604_M115c]"*<br>*Question relevant when: not(selected( ${q104_M115_full_list} , '6')) and not(selected( ${q104_M115_full_list} , '99')) and string-length( ${q104_M115_full_list_other} ) >0*<br>*Response constrained to: .>=0* | |
| q704_M115a.1 *(required)* | q704_M115a.1 What is the typical size of each item in the pack, box, etc. (e.g. 1 litre bottles, 250 mg tablets, 3 ml ampoules, etc.)?<br>*Please provide as much information as is available.*<br>*Question relevant when: not(selected( ${q104_M115_full_list} , '6')) and not(selected( ${q104_M115_full_list} , '99')) and string-length( ${q104_M115_full_list_other} ) >0* | |
| q704_M115b *(required)* | q704_M115b. What is the typical purchase price for that quantity of units (i.e. the quantity noted in the question | |

| Field | Question | Answer |
|---|---|---|
| | above)?<br>*Question relevant when: not(selected( ${q104_M115_full_list} , '6')) and not(selected( ${q104_M115_full_list} , '99')) and string-length( ${q104_M115_full_list_other} ) >0*<br>*Response constrained to: .>=0* | |
| q704_M115c (required) | q704_M115c. Please specify the currency for the purchase price.<br>*Question relevant when: not(selected( ${q104_M115_full_list} , '6')) and not(selected( ${q104_M115_full_list} , '99')) and string-length( ${q104_M115_full_list_other} ) >0* | 1 Tanzanian Shillings<br>2 US dollars<br>3 Euros<br>4 Other<br>99 Don't know |
| q704_M115c.1 | q704_M115c.1 If other currency, specify:<br>*Leave blank if not applicable*<br>*Question relevant when: not(selected( ${q104_M115_full_list} , '6')) and not(selected( ${q104_M115_full_list} , '99')) and string-length( ${q104_M115_full_list_other} ) >0* | |
| q704_M115d (required) | q704_M115d. Please specify the year of the purchase price.<br>*Question relevant when: not(selected( ${q104_M115_full_list} , '6')) and not(selected( ${q104_M115_full_list} , '99')) and string-length( ${q104_M115_full_list_other} ) >0*<br>*Response constrained to: .>=2000 and .<=2019 or .=999* | |
| D. Medications - Cost (12)<br>*Group relevant when: selected( ${section_seven_skip_med} , '1')* | | |
| q704_med_notE11 | 704 CONTINUED For each of the following medications and other medical products that are used for postabortion care, can you tell me …? | |

| | | |
|---|---|---|
| note_704_Other | ***Other medication, any class*** | |
| q704_M116 | ***q704_M116. Medication - any class - Other 1: "[q104_M116_full_list_other]"***<br>*Question relevant when: not(selected( ${q104_M116_full_list} , '6')) and not(selected( ${q104_M116_full_list} , '99')) and string-length( ${q104_M116_full_list_other} ) >0* | |
| q704_M116a (required) | q704_M116a. When purchased, how many units of this item typically come in one pack, box, bottle, etc.? (e.g. 100 tablets, 1000 ml's, etc.) (Write 1 if purchased as a single item.)<br>*Smallest units listed were: "[q204_M116c]" , "[q304_M116c]" , "[q404_M116c]" , "[q504_M116c]" , "[q604_M116c]"*<br>*Question relevant when: not(selected( ${q104_M116_full_list} , '6')) and not(selected( ${q104_M116_full_list} , '99')) and string-length( ${q104_M116_full_list_other} ) >0*<br>*Response constrained to: .>=0* | |
| q704_M116a.1 (required) | q704_M116a.1 What is the typical size of each item in the pack, box, etc. (e.g. 1 litre bottles, 250 mg tablets, 3 ml ampoules, etc.)?<br>*Please provide as much information as is available.*<br>*Question relevant when: not(selected( ${q104_M116_full_list} , '6')) and not(selected( ${q104_M116_full_list} , '99')) and string-length( ${q104_M116_full_list_other} ) >0* | |
| q704_M116b (required) | q704_M116b. What is the typical purchase price for that quantity of units (i.e. the quantity noted in the question above)?<br>*Question relevant when: not(selected( ${q104_M116_full_list} , '6')) and not(selected( ${q104_M116_full_list} , '99')) and string-length( ${q104_M116_full_list_other} ) >0*<br>*Response constrained to: .>=0* | |
| q704_M116c (required) | q704_M116c. Please specify the currency for the purchase price.<br>*Question relevant when: not(selected( ${q104_M116_full_list} , '6')) and not(selected( ${q104_M116_full_list} , '99')) and string-length( ${q104_M116_full_list_other} ) >0* | 1 Tanzanian Shillings<br>2 US dollars<br>3 Euros<br>4 Other<br>99 Don't know |
| q704_M116c.1 | q704_M116c.1 If other currency, specify:<br>*Leave blank if not applicable*<br>*Question relevant when: not(selected( ${q104_M116_full_list} , '6')) and not(selected( ${q104_M116_full_list} , '99')) and string-length( ${q104_M116_full_list_other} ) >0* | |
| q704_M116d (required) | q704_M116d. Please specify the year of the purchase price.<br>*Question relevant when: not(selected( ${q104_M116_full_list} , '6')) and not(selected( ${q104_M116_full_list} , '99')) and string-length( ${q104_M116_full_list_other} ) >0*<br>*Response constrained to: .>=2000 and .<=2019 or .=999* | |
| q704_M117 | ***q704_M117. Medication - any class - Other 2: "[q104_M117_full_list_other]"***<br>*Question relevant when: not(selected( ${q104_M117_full_list} , '6')) and not(selected( ${q104_M117_full_list} , '99')) and string-length( ${q104_M117_full_list_other} ) >0* | |
| q704_M117a (required) | q704_M117a. When purchased, how many units of this item typically come in one pack, box, bottle, etc.? (e.g. 100 tablets, 1000 ml's, etc.) (Write 1 if purchased as a single item.)<br>*Smallest units listed were: "[q204_M117c]" , "[q304_M117c]" , "[q404_M117c]" , "[q504_M117c]" , "[q604_M117c]"*<br>*Question relevant when: not(selected( ${q104_M117_full_list} , '6')) and not(selected( ${q104_M117_full_list} , '99')) and string-length( ${q104_M117_full_list_other} ) >0*<br>*Response constrained to: .>=0* | |
| q704_M117a.1 (required) | q704_M117a.1 What is the typical size of each item in the pack, box, etc. (e.g. 1 litre bottles, 250 mg tablets, 3 ml | |

ampoules, etc.)?
*Please provide as much information as is available.*

| Field | Question | Answer |
|---|---|---|
| | *Question relevant when: not(selected( ${q104_M117_full_list} , '6')) and not(selected( ${q104_M117_full_list} , '99')) and string-length( ${q104_M117_full_list_other} ) >0* | |
| q704_M117b *(required)* | q704_M117b. What is the typical purchase price for that quantity of units (i.e. the quantity noted in the question above)?<br>*Question relevant when: not(selected( ${q104_M117_full_list} , '6')) and not(selected( ${q104_M117_full_list} , '99')) and string-length( ${q104_M117_full_list_other} ) >0*<br>*Response constrained to: .>=0* | |
| q704_M117c *(required)* | q704_M117c. Please specify the currency for the purchase price.<br>*Question relevant when: not(selected( ${q104_M117_full_list} , '6')) and not(selected( ${q104_M117_full_list} , '99')) and string-length( ${q104_M117_full_list_other} ) >0* | 1 Tanzanian Shillings<br>2 US dollars<br>3 Euros<br>4 Other<br>99 Don't know |
| q704_M117c.1 | q704_M117c.1 If other currency, specify:<br>*Leave blank if not applicable*<br>*Question relevant when: not(selected( ${q104_M117_full_list} , '6')) and not(selected( ${q104_M117_full_list} , '99')) and string-length( ${q104_M117_full_list_other} ) >0* | |
| q704_M117d *(required)* | q704_M117d. Please specify the year of the purchase price.<br>*Question relevant when: not(selected( ${q104_M117_full_list} , '6')) and not(selected( ${q104_M117_full_list} , '99')) and string-length( ${q104_M117_full_list_other} ) >0*<br>*Response constrained to: .>=2000 and .<=2019 or .=999* | |

| | | |
|---|---|---|
| q704_M118 | ***q704_M118. Medication - any class - Other 3: "[q104_M118_full_list_other]"***<br>*Question relevant when: not(selected( ${q104_M118_full_list} , '6')) and not(selected( ${q104_M118_full_list} , '99')) and string-length( ${q104_M118_full_list_other} ) >0* | |
| q704_M118a *(required)* | q704_M118a. When purchased, how many units of this item typically come in one pack, box, bottle, etc.? (e.g. 100 tablets, 1000 ml's, etc.) (Write 1 if purchased as a single item.)<br>*Smallest units listed were: "[q204_M118c]" , "[q304_M118c]" , "[q404_M118c]" , "[q504_M118c]" , "[q604_M118c]"*<br>*Question relevant when: not(selected( ${q104_M118_full_list} , '6')) and not(selected( ${q104_M118_full_list} , '99')) and string-length( ${q104_M118_full_list_other} ) >0*<br>*Response constrained to: .>=0* | |
| q704_M118a.1 *(required)* | q704_M118a.1 What is the typical size of each item in the pack, box, etc. (e.g. 1 litre bottles, 250 mg tablets, 3 ml ampoules, etc.)?<br>*Please provide as much information as is available.*<br>*Question relevant when: not(selected( ${q104_M118_full_list} , '6')) and not(selected( ${q104_M118_full_list} , '99')) and string-length( ${q104_M118_full_list_other} ) >0* | |
| q704_M118b *(required)* | q704_M118b. What is the typical purchase price for that quantity of units (i.e. the quantity noted in the question above)?<br>*Question relevant when: not(selected( ${q104_M118_full_list} , '6')) and not(selected( ${q104_M118_full_list} , '99')) and string-length( ${q104_M118_full_list_other} ) >0*<br>*Response constrained to: .>=0* | |
| q704_M118c *(required)* | q704_M118c. Please specify the currency for the purchase price.<br>*Question relevant when: not(selected( ${q104_M118_full_list} , '6')) and not(selected( ${q104_M118_full_list} , '99')) and string-length( ${q104_M118_full_list_other} ) >0* | 1 Tanzanian Shillings<br>2 US dollars<br>3 Euros<br>4 Other<br>99 Don't know |
| q704_M118c.1 | q704_M118c.1 If other currency, specify:<br>*Leave blank if not applicable*<br>*Question relevant when: not(selected( ${q104_M118_full_list} , '6')) and not(selected( ${q104_M118_full_list} , '99')) and string-length( ${q104_M118_full_list_other} ) >0* | |
| q704_M118d *(required)* | q704_M118d. Please specify the year of the purchase price.<br>*Question relevant when: not(selected( ${q104_M118_full_list} , '6')) and not(selected( ${q104_M118_full_list} , '99')) and string-length( ${q104_M118_full_list_other} ) >0*<br>*Response constrained to: .>=2000 and .<=2019 or .=999* | |
| q704_M119 | ***q704_M119. Medication - any class - Other 4: "[q104_M119_full_list_other]"***<br>*Question relevant when: not(selected( ${q104_M119_full_list} , '6')) and not(selected( ${q104_M119_full_list} , '99')) and string-length( ${q104_M119_full_list_other} ) >0* | |
| q704_M119a *(required)* | q704_M119a. When purchased, how many units of this item typically come in one pack, box, bottle, etc.? (e.g. 100 tablets, 1000 ml's, etc.) (Write 1 if purchased as a single item.)<br>*Smallest units listed were: "[q204_M119c]" , "[q304_M119c]" , "[q404_M119c]" , "[q504_M119c]" , "[q604_M119c]"*<br>*Question relevant when: not(selected( ${q104_M119_full_list} , '6')) and not(selected( ${q104_M119_full_list} , '99')) and string-length( ${q104_M119_full_list_other} ) >0*<br>*Response constrained to: .>=0* | |
| q704_M119a.1 *(required)* | q704_M119a.1 What is the typical size of each item in the pack, box, etc. (e.g. 1 litre bottles, 250 mg tablets, 3 ml ampoules, etc.)? | |

| Field | Question | Answer |
|---|---|---|
| | *Please provide as much information as is available.*<br>*Question relevant when: not(selected( ${q104_M119_full_list} , '6')) and not(selected( ${q104_M119_full_list} , '99')) and string-length( ${q104_M119_full_list_other} ) >0* | |
| q704_M119b *(required)* | q704_M119b. What is the typical purchase price for that quantity of units (i.e. the quantity noted in the question above)?<br>*Question relevant when: not(selected( ${q104_M119_full_list} , '6')) and not(selected( ${q104_M119_full_list} , '99')) and string-length( ${q104_M119_full_list_other} ) >0*<br>*Response constrained to: .>=0* | |
| q704_M119c *(required)* | q704_M119c. Please specify the currency for the purchase price.<br>*Question relevant when: not(selected( ${q104_M119_full_list} , '6')) and not(selected( ${q104_M119_full_list} , '99')) and string-length( ${q104_M119_full_list_other} ) >0* | 1 Tanzanian Shillings<br>2 US dollars<br>3 Euros<br>4 Other<br>99 Don't know |
| q704_M119c.1 | q704_M119c.1 If other currency, specify:<br>*Leave blank if not applicable*<br>*Question relevant when: not(selected( ${q104_M119_full_list} , '6')) and not(selected( ${q104_M119_full_list} , '99')) and string-length( ${q104_M119_full_list_other} ) >0* | |
| q704_M119d *(required)* | q704_M119d. Please specify the year of the purchase price.<br>*Question relevant when: not(selected( ${q104_M119_full_list} , '6')) and not(selected( ${q104_M119_full_list} , '99')) and string-length( ${q104_M119_full_list_other} ) >0*<br>*Response constrained to: .>=2000 and .<=2019 or .=999* | |

| | | |
|---|---|---|
| q704_M120 | ***q704_M120. Medication - any class - Other 5: "[q104_M120_full_list_other]"***<br>*Question relevant when: not(selected( ${q104_M120_full_list} , '6')) and not(selected( ${q104_M120_full_list} , '99')) and string-length( ${q104_M120_full_list_other} ) >0* | |
| q704_M120a *(required)* | q704_M120a. When purchased, how many units of this item typically come in one pack, box, bottle, etc.? (e.g. 100 tablets, 1000 ml's, etc.) (Write 1 if purchased as a single item.)<br>*Smallest units listed were: "[q204_M120c]" , "[q304_M120c]" , "[q404_M120c]" , "[q504_M120c]" , "[q604_M120c]"*<br>*Question relevant when: not(selected( ${q104_M120_full_list} , '6')) and not(selected( ${q104_M120_full_list} , '99')) and string-length( ${q104_M120_full_list_other} ) >0*<br>*Response constrained to: .>=0* | |
| q704_M120a.1 *(required)* | q704_M120a.1 What is the typical size of each item in the pack, box, etc. (e.g. 1 litre bottles, 250 mg tablets, 3 ml ampoules, etc.)?<br>*Please provide as much information as is available.*<br>*Question relevant when: not(selected( ${q104_M120_full_list} , '6')) and not(selected( ${q104_M120_full_list} , '99')) and string-length( ${q104_M120_full_list_other} ) >0* | |
| q704_M120b *(required)* | q704_M120b. What is the typical purchase price for that quantity of units (i.e. the quantity noted in the question above)?<br>*Question relevant when: not(selected( ${q104_M120_full_list} , '6')) and not(selected( ${q104_M120_full_list} , '99')) and string-length( ${q104_M120_full_list_other} ) >0*<br>*Response constrained to: .>=0* | |
| q704_M120c *(required)* | q704_M120c. Please specify the currency for the purchase price.<br>*Question relevant when: not(selected( ${q104_M120_full_list} , '6')) and not(selected( ${q104_M120_full_list} , '99')) and string-length( ${q104_M120_full_list_other} ) >0* | 1 Tanzanian Shillings<br>2 US dollars<br>3 Euros<br>4 Other<br>99 Don't know |
| q704_M120c.1 | q704_M120c.1 If other currency, specify:<br>*Leave blank if not applicable*<br>*Question relevant when: not(selected( ${q104_M120_full_list} , '6')) and not(selected( ${q104_M120_full_list} , '99')) and string-length( ${q104_M120_full_list_other} ) >0* | |
| q704_M120d *(required)* | q704_M120d. Please specify the year of the purchase price.<br>*Question relevant when: not(selected( ${q104_M120_full_list} , '6')) and not(selected( ${q104_M120_full_list} , '99')) and string-length( ${q104_M120_full_list_other} ) >0*<br>*Response constrained to: .>=2000 and .<=2019 or .=999* | |
| GPS_QB2_med *(required)* | GPS location capture<br>*Press the button to capture the GPS location at this point in the survey.* | |
| q705_time_end_qb_med *(required)* | ENTER THE END TIME OF THE INTERVIEW<br>*NB: The default is the current time.* | |
| thank_you | **END OF INTERVIEW QUESTIONS.**<br><br>**THANK THE PARTICIPANT FOR THEIR TIME.**<br><br>**THEN COMPLETE THE FOLLOWING TWO ITEMS.** | |
| q706_interviewer_comments_med | INTERVIEW COMMENTS - ENTER ANY RELEVANT NOTES AT THE END OF THE INTERVIEW. PLEASE ALSO EXPLAIN HOW COSTS WERE COLLECTED, I.E. VIA INTERVIEW, SITE REPRESENTATIVE FILLING OUT | |

| Field | Question | Answer |
|---|---|---|
| | FORM, OR EXTRACTION FROM RECORDS. | |
| End_note | **End of Questionnaire B part 4**<br><br>ON THE NEXT PAGE YOU'LL FIND THIS FACILITY'S ID NUMBER. TAKE NOTE OF THE NUMBER, AND THEN, ON THE FOLLOWING PAGE, NAME THIS FORM BY ADDING THE FACILITY ID BEFORE THE FORM NAME.<br><br>FOR EXAMPLE, "20 Tanzania PAC cost study – Quest. B part 4 medications".<br><br>IF THIS INTERVIEW IS COMPLETE - I.E. THE RESPONDENT HAS ANSWERED ALL OF THE QUESTIONS THAT THEY CAN ON THIS FORM, THEN LEAVE THE DEFAULT BOX CHECKED. IF THE FORM IS NOT FINAL FOR THIS FACILITY, UNTICK THE BOX.<br><br>THEN SAVE AND EXIT. | |
| facility_id_med2 | The ID for this facility is '[facility_id_med]'. | |
